# Transcriptomic Analysis of the Venom Gland and Enzymatic Characterization of the Venom of *Phoneutria depilata* (Ctenidae) from Colombia

**DOI:** 10.3390/toxins14050295

**Published:** 2022-04-21

**Authors:** Julieta Vásquez-Escobar, Teresa Romero-Gutiérrez, José Alejandro Morales, Herlinda C. Clement, Gerardo A. Corzo, Dora M. Benjumea, Ligia Luz Corrales-García

**Affiliations:** 1 Grupo de Toxinología y Alternativas Farmacéuticas y Alimentarias, Facultad de Ciencias Farmacéuticas y Alimentarias, Universidad de Antioquia, Medellin 1226, Colombia; dora.benjumea@udea.edu.co; 2 Traslational Bioengineering Department, Exact Sciences and Engineering University Center, Universidad de Guadalajara, Guadalajara 44430, Mexico; ro.teresa.gu@gmail.com (T.R.-G.); jalejandro.morales@academicos.udg.mx (J.A.M.); 3 Departamento de Medicina Molecular y Bioprocesos, Instituto de Biotecnología, Universidad Nacional Autónoma de México, Cuernavaca 62210, Mexico; herlinda.clement@ibt.unam.mx (H.C.C.); corzo@ibt.unam.mx (G.A.C.); 4 Departamento de Alimentos, Facultad de Ciencias Farmacéuticas y Alimentarias, Universidad de Antioquia, Medellin 1226, Colombia

**Keywords:** venom, transcriptome, *Phoneutria*, venom gland, RNA-seq

## Abstract

The transcriptome of the venom glands of the *Phoneutria depilata* spider was analyzed using RNA-seq with an Illumina protocol, which yielded 86,424 assembled transcripts. A total of 682 transcripts were identified as potentially coding for venom components. Most of the transcripts found were neurotoxins (156) that commonly act on sodium and calcium channels. Nevertheless, transcripts coding for some enzymes (239), growth factors (48), clotting factors (6), and a diuretic hormone (1) were found, which have not been described in this spider genus. Furthermore, an enzymatic characterization of the venom of *P. depilata* was performed, and the proteomic analysis showed a correlation between active protein bands and protein sequences found in the transcriptome. The transcriptomic analysis of *P. depilata* venom glands show a deeper description of its protein components, allowing the identification of novel molecules that could lead to the treatment of human diseases, or could be models for developing bioinsecticides.

## 1. Introduction

Animal venom glands are specialized organs that synthesize and secrete a complex mixture of toxin peptides, allowing the venomous animal to defend against predators or to trap prey. Spider venom encapsulates a highly toxic arsenal that has been predicted to contain more than 10 million bioactive peptides that target a diverse range of receptors, channels, and enzymes, making spider venoms valuable sources of potential pharmacological active compounds [1,2,3]. Therefore, spiders are considered to be one of the most successful venomous animals on Earth, with more than 49,100 accepted species described and an even more significant number remaining to be characterized [1,4,5]. Furthermore, spiders have achieved unprecedented perfection concerning their venom production and biological variability [2]. Peptides are the primary components of these venoms, and some of them contain more than 1000 unique, small, disulfide-rich peptides [1,5]. Despite this, the abundant peptide diversity of spider venom has been poorly characterized, both in its structure and pharmacological potential. Even so, most of the spider-venom peptides described to date are detailed in ArachnoServer, a manually curated database that provides detailed information about proteinaceous toxins from spiders [1], which, to date, accounts for the description of 1458 curated toxins.

Spiders from the *Phoneutria* genus are known as ‘‘banana or wandering spiders’’ because they often inhabit this crop, and they are also known as “armed” spiders for their aggressive behavior. Currently, the *Phoneutria* genus comprises nine large (17–48 mm) nocturnal wandering spider species, and they are widely distributed in Central America (Costa Rica) and South America (from the East of the Andes Mountains to North of Argentina) [6,7]. In Colombia, the reported species are *P. depilata*, *P. reidyi*, *P. fera* and *P. boliviensis* [8,9]. These spiders possess a potent neurotoxic venom that makes them among the most medically important spiders globally. Many researchers have analyzed the venom components and the epidemiology of the spider bites [7].

Around 400 peptides and proteins, ranging from 1.2 to 27 kDa, have been isolated from the genus *Phoneutria*. Out of these, about 100 peptides have determined a complete or partial amino acid sequence [3]. The purified reported peptides refer to venoms from *P. nigriventer*, *P. reidyi*, *P. keyserlingi*, and *P. salei*. There is only one available venom characterization from *P. depilata* (formerly synonymized as *P. boliviensis*) [3,9]. The peptides of these species represent a rich source of neurotoxins acting on different receptors and ion channels of the central nervous system. The mode of action of such peptides makes them a valuable source of possible pharmacological active compounds, helpful in treating different diseases, such as chronic pain and erectile dysfunction [2,3,5].

In this work, a transcriptomic analysis of the venom glands of an adult female of *P. depilata* was performed to identify potential peptides and proteins that compose such venom. We established the first complete structure of peptides and proteins from the venom of *P. depilata*, and new peptide structures were found besides the expected peptidic compounds, such as enzymes, growth factors, and hormones. This research stated that the function of novel putative toxins remains unrevealed, many of them with possible pharmacological and agro-industrial potential. Additionally, the value of this work also lies in the identification of several coding sequences for peptides that had not been previously reported in the venoms of other spiders of this genus, such as carboxylesterases, kinases, oxidoreductases, phosphodiesterases, endonucleases, phosphatases, transferases, carboxypeptidases, lipases, ligases, vascular endothelial growth factor, insulin type, platelet-derived, nerve-related, von Willebrand factor type C and the hormone HD-31.

## 2. Results

### 2.1. Transcriptomic Analysis

#### 2.1.1. Venom Gland, RNA Extraction, Transcriptome Sequencing, and Assembly

The RNA extraction from a pair of venom glands of the *P. depilata* spider provided 5.6 μg of total RNA, which showed a single ribosomal fraction with an 8.9 RIN value where a 28S subunit processing phenomenon occurred, similar to other arthropods, producing a fragment like the 18S subunit [10]. RNA sequencing yielded 33,538,542 read pairs, obtained with a minimum value of Q30 quality. Then, 86,424 transcripts were assembled with an N50 of 1334 bases. The assembly process produced 1006 transcripts that were systematically annotated using their identity compared with the Animal Toxin Annotation Project database. Data was submitted, in fastq format, to the European Nucleotide Archive registered with a study accession number ERS3605146. For the protein sequence analysis, the PFAM classification was used as the first function distribution parameter, due to the great diversity of annotations of the transcriptome sequences, allowing us to find common domains, functional regions, and identification to provide direction towards the elucidation of protein functions.

#### 2.1.2. Functional Transcripts Nomenclature

Currently, there is no standard nomenclature for naming transcripts identified by RNA-Seq and annotated as venom proteins. The identifier generated by the assembler is often preserved, making it challenging to classify sequences and can lead to confusion. Therefore, the nomenclature proposed by Romero-Gutiérrez et al. (2017) [11] was used in this work. Each reported transcript was named as follows: the first three characters define the species (Phd, from *P. depilata*), the following three characters describe the encoded peptide/protein family concerning its putative function, followed by another three characters related to the subtype, and, finally, the last two digits indicate the transcript number. Table 1 resumes this nomenclature for the transcripts reported for *P. depilata*. If a transcript was found with the same sequence as a previously reported one, the original name was kept, avoiding duplications in databases.

#### 2.1.3. Putative Venom Gland Components

**Figure 1:**
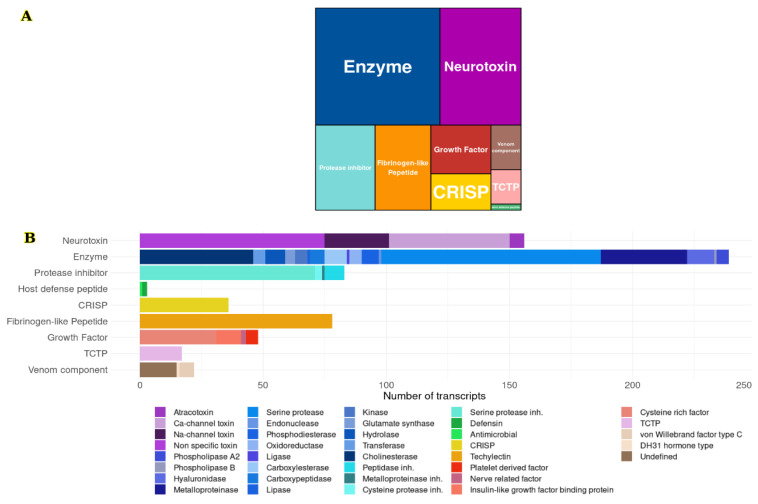
Relative abundances of the families identified as components of the venom. (**A**) Coding sequences annotated by family; (**B**) Expression levels of transcripts by family.

A search for transcripts with identity to venom components was done during the annotation process. The 682 coding transcripts for integral components of the venom were identified: 239 (35%) were related to enzymes, such as serine proteases, phospholipases, metalloproteases, hyaluronidases, among others; 156 (22.9%) had an identity with neurotoxins that affect voltage-gated sodium and calcium channels; 83 (12.2%) corresponded to inhibitors of different types of enzymes, mainly serine proteases; 78 (11.4%) were annotated as fibrinogen-like peptides; 48 (7%) were related to growth factors; 36 (5.3%) cysteine-rich secretory proteins (CRISPs, members of the CAP superfamily); 17 (2.5%) had an identity with translation-controlled tumor proteins (TCTP); 3 (0.4%) were related to host defense peptides. Finally, 22 (3.2%) were classified as other venom components, such as hormone-type peptides, von Willebrand factor, and some of them with unknown functions (Figure 1) (see Supplementary Material).

RNA-Seq quantification allows estimating of the relative abundance of transcripts to understand the venom gland transcriptomic profile. Since there is no reference genome to map, quantifying the reads can determine the expression levels of the transcripts. Those expression levels were represented in the function of transcripts per million (TPM) values (see Supplementary Table S1).

**Figure 2:**
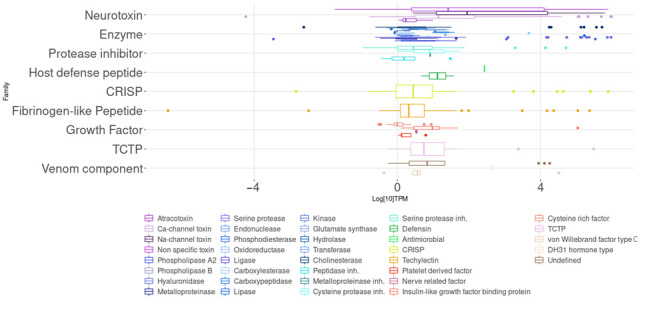
Distribution of the relative abundances of the transcripts identified as venom components.

According to the transcriptome quantification, the highest expression levels of the families were host defense peptides and neurotoxins. Neurotoxins, both with non-identified targets and those that alter sodium channels, had one of the highest transcript expression levels. We also found other families with relatively high expression, such as other venom components, like a diuretic hormone and TCTP. Meanwhile, the families with the lowest expression levels were the protease inhibitors, CRISP, enzymes, fibrinogen-like peptides, and growth factors (Figure 2).

Enzymes

239 enzyme-related transcripts (Enz) were found, constituting the amplest category in the transcriptome, including 18 types of enzymes. The most abundant were serine proteases, metalloproteases, and cholinesterases. Additionally, other sequences, habitually reported in poisonous animal venoms, such as hyaluronidases, phospholipases A2 and B, were found in smaller quantities (Table 2).

Serine proteases represented the highest number of enzyme coding sequences (89 NCS) found in the transcriptome of the venom glands of *P. depilata*. However, these transcripts did not have high expression levels according to the RNA-Seq quantification analysis of the total transcriptome (3.2 median TPM). Alternatively, in the transcriptome, coding sequences for serine proteases inhibitors (71 NCS) were found, but with lower expression levels than serine proteases. Furthermore, metalloproteinases presented the third largest number of enzyme coding sequences, but they also showed low expression levels. 

The coding sequences for hyaluronidases found in the transcriptome of the venom glands of *P. depilata* (11 NCS) agree with the activity evidenced in the hyaluronic acid zymogram. However, few coding sequences with low expression levels (2.8 median TPM) of this enzyme could explain why 2.0 µg of each fraction was not enough to show the degradation of hyaluronic acid in the gel. Alternatively, a few coding sequences were annotated as phospholipases, 5 for phospholipases type A2, and 1 for phospholipase type B. Nevertheless, phospholipase activity was not evident, either in the venom or in the fractions obtained by RP-HPLC. This result could be explained by the small number of coding sequences with low expression level; so, we believe that phospholipases do not play an essential role during envenoming. 

##### Neurotoxins

In the *P. depilata* transcriptome, 156 sequences had an identity to neurotoxins (Ntx). Among this peptide category, 49 coding sequences with a TPM of 13.5 for calcium channels toxins, 26 encoded transcripts with a TPM of 89.1 for voltage-gated sodium channel toxins, and 6 coding sequences for the MIT-type family of atracotoxins, with a TPM of 1.6 were found. Finally, 75 transcripts that belong to sequences annotated as neurotoxins with a TPM of 26.1 were obtained; however, their molecular target is unknown.

##### Fibrinogen-like Peptides

For fibrinogen-like peptides, 78 transcripts with a TPM of 2 were uncovered. They code for techylectins (FibTec), which are related to platelet aggregation and blood coagulation [12,13].

##### Proteases Inhibitors

For protease inhibitors (PIn), 80 coding sequences were detected. The vast majority were serine protease inhibitors, with 71 coding sequences but low expression levels (TPM of 2.6). Also, 8 sequences for peptidase inhibitors (TPM of 4.9), 3 for cysteine protease inhibitors (TPM of 20.6), and only one coding sequence for metalloprotease inhibitors (TPM of 8.2) were found.

##### Growth Factors

Forty-eight coding sequences related to growth factors (GrF) in the *P. depilata* transcriptome were obtained. Among these, 31 sequences were detected and classified as receptors rich in cysteine related to epidermal growth factors, but with low expression level (TPM of 3.4). Moreover, 10 coding sequences for an insulin-like growth factor binding protein (TPM of 9.6), 5 coding sequences related to platelet-derived growth factor (PDGF) (TPM of 1.3), and 2 for nerve-related growth factor (TPM of 3.4) were also found.

##### CRISP

In the Cysteine-Rich Secretory Proteins (CRISP) category, 36 coding transcripts were found, with a TPM of 2.3. The majority were venom allergens.

##### TCTP

In the *P. depilata* transcriptome, 17 annotated transcripts were identified as translationally controlled tumor proteins (TCTP), with low expression levels (TPM of 4.6). TCTP are proteins related to edema development after envenomation, and they can cause hypotension due to vasodilation. 

##### Host Defense Peptides

Among the group of host defense peptides, 2 of the coding transcripts were annotated as defensins (TPM of 21.1) and just one as antimicrobial (TPM of 271).

##### Other Venom Components

This protein category included transcripts annotated as venom peptides. However, the molecular target, or biological function, has not been demonstrated experimentally yet. In total, 22 coding transcripts were found; 6 of them had the von Willebrand factor type C domain with a TPM of 3.7 media. A coding sequence for a DH31 hormone-like peptide was also identified (TPM of 428.9). Also, 15 sequences with a TPM of 6.5 were attributed as venom components with unknown function. They are grouped into the “unknown” category (Und) because it was not possible to associate them with a particular protein domain, biological activity or molecular target.

### 2.2. Reverse Phase HPLC Separation of P. depilate Venom

**Figure 3:**
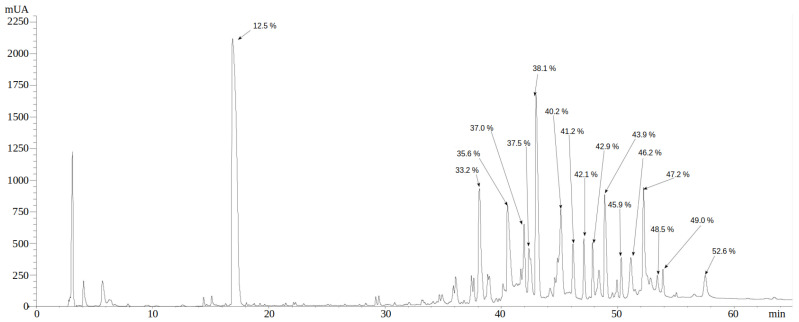
Chromatographic profile of *P. depilata’s* venom by RP-HPLC. The venom was separated using a C18 analytical column (250 × 4.6 mm), with an elution gradient from 0 to 60% ACN plus 0.1% TFA, for 60 min, with a flow of 1 mL/min. The absorbance was monitored at 230 nm. The percentages shown on each fraction correspond to the ACN gradient of elution.

The complete venom of *P. depilata* was separated by reverse phase HPLC (RP-HPLC). Figure 3 shows a chromatogram profile with at least 15 well-defined fractions with absorbances (230 nm) greater than 250 mAU. The same chromatogram also shows 27 fractions with lower absorbances. The 15 fractions greater than 250 mAU were tested as to whether they have any enzymatic activity.

### 2.3. Enzymatic Characterization of P. depilata Venom and Venom Fractions

#### 2.3.1. Phospholipase Activity

The evaluation of phospholipase type A2 (PLA2) activity of the *P. depilata* whole venom (5, 53, and 106 μg) was not evident in egg-yolk agarose plates. The positive control registered an 18 mm halo degradation. Different fractions of the venom (5 μg each) were taken to assess the activity. They were obtained by RP-HPLC between 25 and 40% of ACN, considered as the percentage range in which PLA2s eluted in invertebrates [14,15]. Nevertheless, enzymatic activity was not evident (See Supplementary Figure S1).

#### 2.3.2. Protease Activity

**Figure 4:**
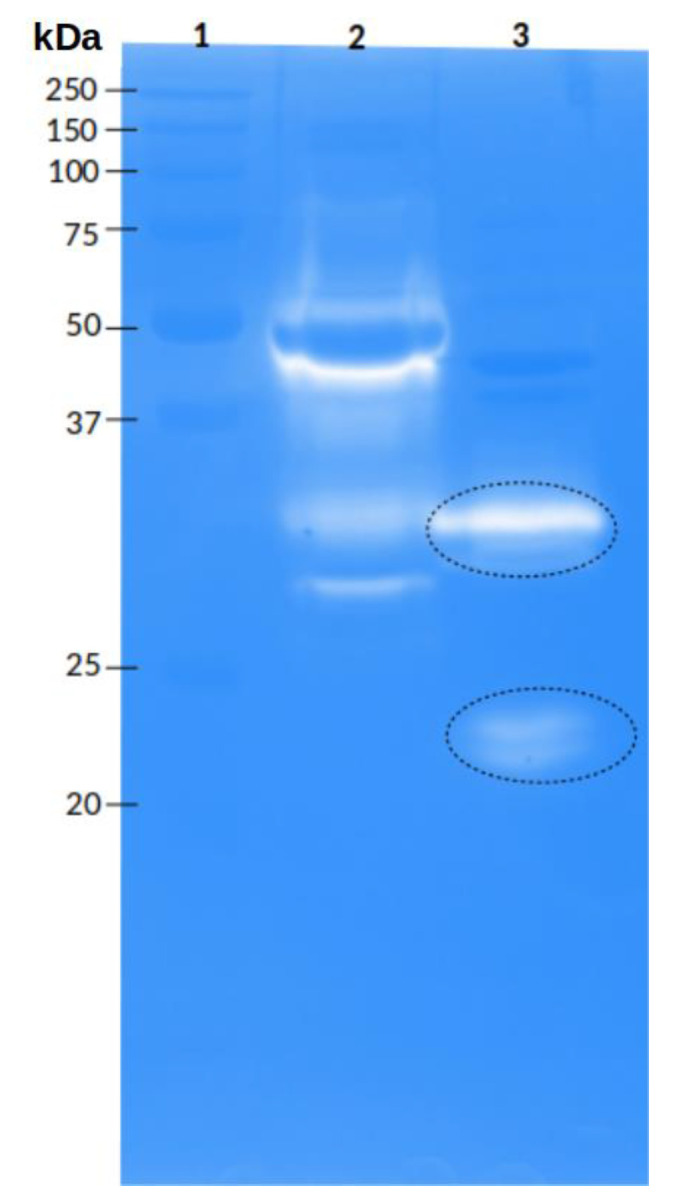
Protease activity of *P. depilata* venom. Enzymatic evaluation was performed by electrophoresis in a polyacrylamide gel (12.5%) copolymerized with gelatin (1.5 mg/mL). Lanes: 1. Molecular weight marker (kDa), 2. *Bothrops ammodytoides* snake venom (5 µg), 3. *P. depilata* venom (35 µg). The black dotted circles represent the bands that were sent for MS/MS analysis.

**Figure 5:**
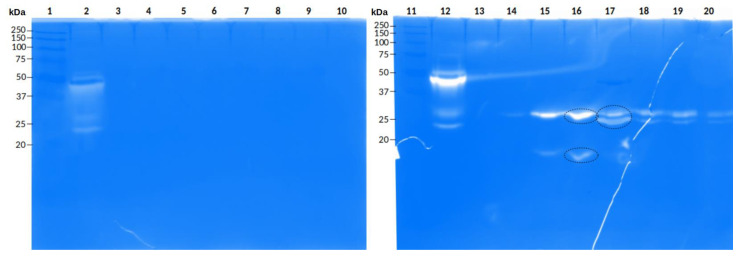
Protease activity of *P. depilata* venom fractions by electrophoresis in a polyacrylamide gel (12.5%) copolymerized with gelatin (1.5 mg/mL). Lanes: 1. Molecular weight marker (kDa), 2. *Bothrops ammodytoides* snake complete venom (5 µg), 3. Fraction 33.2% ACN (3 μg), 4. Fraction 35.6% ACN (3 μg), 5. Fraction 37.0% ACN (3 μg), 6. Fraction 37.5% ACN (3 μg), 7. Fraction 38.1% ACN (3 μg), 8. Fraction 40.2% ACN (3 μg), 9. Fraction 41.2% ACN (3 μg), 10. Fraction 42.1% ACN (3 µg). 11. Molecular weight marker (kDa), 12. *Bothrops ammodytoides* snake complete venom (5 μg), 13. Fraction 42.9% ACN (3 μg), 14. Fraction 43.9% ACN (3 μg), 15. Fraction 45.3% ACN (1 μg), 16. Fraction 46.2% ACN (3 μg), 17. Fraction 47.2% ACN (3 μg), 18. Fraction 48.5% ACN (1 μg), 19. Fraction 49.0% ACN (1 μg), 20. Fraction 52.6% ACN (1 µg). The black dotted circles represent the bands sent for MS/MS sequencing.

The whole venom of *P. depilata* showed protease activity in zymograms using gelatin as substrate. Degradation bands with apparent molecular weights of 24 and 32 kDa were observed (Figure 4, lane 3). Also, some fractions obtained from *P. depilata* venom by RP-HPLC that eluted at higher ACN percentages likewise presented protease activity (Figure 5). For example, the 46.2% ACN fraction (Figure 5, lane 16) showed two degradation bands with apparent molecular weights of 20 and 30 kDa, respectively. The 47.2% ACN fraction (Figure 5, lane 17) also exhibited two degradation halos. These halos were cut off and sent for MS/MS analysis.

#### 2.3.3. Hyaluronidase Activity

**Figure 6:**
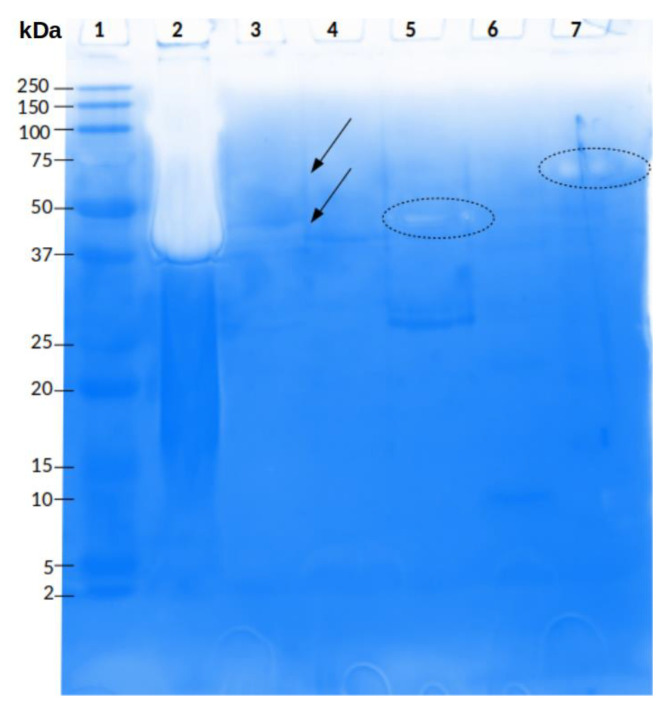
Hyaluronidase activity of *P. depilata* venom fractions. Enzymatic activity was evaluated using electrophoresis in a polyacrylamide gel (12.5%) copolymerized with hyaluronic acid (0.5 mg/mL). Lanes: 1. Molecular weight markers (kDa), 2. Venom of the spider *Brachipelma vagans* (5 µg), 3. *P. depilata* venom (35 µg), 4. Fraction 46.2% ACN (2 µg), 5. Fraction 47.2% ACN (2 µg), 6. Fraction 49% ACN (2 µg), 7. Fraction 52.6% ACN (2 µg). The black arrows indicate the bands of hyaluronidase activity of the whole venom. The black dotted circles represent the bands that were sent for MS/MS analysis.

The complete venom of *P. depilata* and its fractions showed degradation in bands with molecular masses between 45 and 70 kDa (Figure 6, lane 3). These bands cannot be seen in the complete venom, although they are in the 47.2 and 52.6% ACN fractions (Figure 6, lanes 5 and 7). These bands were cut off and sent for MS/MS analysis.

### 2.4. Proteomic Correlation between Transcripts and Enzymatic Activity

#### 2.4.1. Peptide Sequences Obtained from the Gelatin Zymogram

As mentioned, two bands that showed proteolytic activity with relative molecular weights of 23 and 30 kDa were cut. The 23 kDa blurred band was a keratin contaminant band with identity to a keratin fragment peptide from *Parasteatoda tepidariorum* (type II cytoskeletal 6A-like, NCBI: XP_015915717.1; UniParc: UPI00077F9947). The strongest degradation band of 30 kDa produced 8 clear sequenced fragments with identities with the putative PQM protease precursors of *Phoneutria fera* (UniProtKB: A0A2I5YNW5). The peptide fragments obtained from each band (23 o 30 kDa) were compared with the obtained transcripts. Coincidence peptide sequences with four peptide fragments (fragments 1, 5, 6, and 7) from the 30 kDa band were found. The PhdEnzSeP15 transcript identity with such four MS/MS peptide fragments was 100% and e-values ranged between 1.96 × 10^−13^ and 7.0596 × 10^−7^. The other four MS/MS peptide fragments had lower identities but higher than 92.3%. Supplementary Figure S2 shows the PhdEnzSeP15 protein sequence displaying the 8 MS/MS fragments found (identities from 92.3 to 100%). Finally, a protease-like sequence was obtained, and it was confirmed that the band with protease activity corresponded to a sequence previously annotated in the transcript and regarded as a sequence with a putative serine protease activity. The fragment obtained from the 23 kDa band had no identity with the annotated transcripts of *P. depilata*; so, it was confirmed that reflects a contamination.

When the *P. depilata* venom fractions, obtained using RP-HPLC, were used to perform the protease zymogram, three bands with activity were observed. Two bands were from fraction 46.2% ACN (~30 kDa and ~20 kDa), and one more band was from fraction 47.2% ACN (~25 kDa) (Figure 5). They were cut and analyzed by MS/MS. ands of 20 kDa and 30 kDa had peptide fragments with identity to keratin peptides from *P. tepidariorum* (type II cytoskeletal 6A-like, NCBI: XP_015915717. 1). The 25 kDa band delivered five MS/MS peptide fragments with identity to the putative peptide PQM protease precursor from *P. fera* (UniProtKB: A0A2I5YNW5). Similarly, the five MS/MS peptide fragments were compared to the annotated transcripts. One of them showed 100% identity with the transcript PhdEnzSeP16 (e-value 2.696 × 10^−6^). An alignment between all five MS/MS fragments and the PhdEnzSeP16 transcript is shown in Supplementary Figure S3. However, there are some small amino acid differences in fragments 1 and 5; that is the glutamic acid and the lysin in the same position. The fragments obtained from the 46.2% ACN fraction protease bands had no identity with the obtained transcripts; so, it was confirmed that reflects a keratin contamination.

#### 2.4.2. Peptide Sequences Obtained from the Hyaluronic Acid Zymogram

The hyaluronidase activity yielded two bands (Figure 6), which were MS/MS sequenced. The band obtained from fraction 47.2% ACN generated 3 fragments, 2 of them with identity for keratin, type II cytoskeletal 6A-like, of *Parasteatoda tepidariorum* (NCBI: XP_015915717.1; UniParc: UPI00077F9947), and the third had identity with Tricarboxylate transport protein-like protein of *Dinothrombium tinctorium* (GenBank: RWS13677.1; UniProtKB: A0A3S3PEL2). Fraction 52.6% ACN produced 4 sequenced fragments, 3 showed identity with Techylectin-like protein from *P. nigriventer* (UniProtKB/Swiss-Prot: P85031.1), and the remaining with U2-ctenitoin-Pk1a from *P. keiserlingi* (UniProtKB/Swiss-Prot: P83905.1). Fragments 1, 2, and 4 of hyaluronidase band of fraction 52.6% ACN showed homology with the obtained transcripts. Fragments 1 and 2 matched with the PhdFibTec40 transcript with 91.7% and 100% identity, and e-value of 3.896 × 10^−11^ and 7.696 × 10^−8^, respectively (Supplementary Figure S4). Fragment 4 had 100% identity with the PhdNtxNav24 transcript and an e-value of 9.896 × 10^−13^ (Supplementary Figure S5). Even though identities between the MS/MS peptide fragments and those transcripts were found, such transcripts did not encode for hyaluronidases.

## 3. Discussion

This work represents the first venom gland transcriptomic analysis conducted for *P. depilata*. A pair of venom glands and low venom amounts were required to provide new and valuable information. The transcriptome analysis showed a significant number of neurotoxins, which affect mainly sodium and calcium ion channels. Enzymes such as serine proteases, metalloproteases, cholinesterases, and hyaluronidases, that could explain some effects produced during envenomation, were also obtained. Additionally, transcriptome quantification showed that the transcripts with neurotoxin identity had one of the highest expression levels, consistent with effects on the vertebrate CNS that is observed during envenomation by the *P. depilata* spider.

Phospholipases (A2, B, D) present in the venoms of scorpions, spiders, and snakes are responsible for the degradation of cell membranes, causing damage to tissues surrounding the venom injection site [16]. Additionally, spider venom phospholipases induce local and systemic toxicity [17]. Phospholipases A2 (PLA2) in spider venoms have not been found abundantly; nevertheless, there are reports of PLA2 found in the spiders *Atrax versutus* (now *Hadronyche versuta*) and *Eresus niger* [18,19]. Furthermore, phospholipase D isoforms have been identified in the venom of spiders of the genus *Loxosceles*, which inhibit platelet aggregation in humans [18]. However, these types of enzymes were scarce in the *P. depilata* transcriptome, which was indirectly demonstrated by the absence of phospholipase inactivity in the venom and fractions. The absence of phospholipase activity agrees with those results found in other spider venoms from the same spider family, such as the tiger spider *Cupiennius salei* and *P. nigriventer* (see Supplementary Table S2) [19,20]. Furthermore, the transcriptomic analysis reported for *P. pertyi* does not indicate coding transcripts for phospholipases [21]. Additionally, they have not been reported to play a role in the envenoming produced by spiders of this genus [22].

Proteases are enzymes present in venoms of different animals that specifically hydrolyze the proteins’ peptide bonds [23]. Metalloproteolytic and serine proteolytic activities have been described for spider venoms [24,25]. The metalloproteinases generate several effects: hemorrhage, edema, dermonecrosis, myonecrosis, and hemostatic disorders [26,27]. These effects have been reported in spider venoms of various species of the genus *Loxosceles*, also known as brown spiders. Some have been purified from *L. intermedia* and *Hippasa partita* (wolf spider) venoms [26,27,28]. Serine proteases can also cause dermonecrosis, hemostasis alterations, platelet function, and Factor Xa-like activity [18]. Serine proteases are one of the prominent families found in the transcriptome of the venom glands of the genus *Phoneutria* [20,21]. Two low- and medium-molecular-weight serine proteases have been characterized from the venom gland of *Hippasa agelenoides* [29,30,31]. Specifically, for spiders of the genus *Phoneutria*, proteolytic activity has been reported for the venom of the species *P. nigriventer*, and two serine proteases have also been identified [32,33]. This work made it possible to verify the proteolytic activity of the complete venom of *P. depilata* and two of its proteic fractions. Although several putative protease transcripts were found in the venom gland of *P. depilata*, these enzymes could be regulated by protease inhibitors. We found a considerable number of transcripts coding for protease inhibitors, which may explain why the venom of *P. depilata* does not possess as high protease activity as others, such as the viperid *Bothrops ammodytoides*. Additionally, not all transcripts are suitably translated into proteins, since transcription and translation processes are subject to different regulations and dynamics [34].

Regarding the current work, we could infer that the defined parameters used for the read’s assembly was adequate, based on the identity between sequenced bands (~30 kDa from the whole venom and fraction 47.2% ACN), and two serine protease isoforms from the transcriptome of *P. depilata*. The amino acid difference (Lys vs. Glu) between the sequenced fragments and transcriptome (Supplementary material Figures S2 and S3) could be considered a genomic mutation, or a gene isoform; however, there is not enough evidence to estimate the difference as sequencing or assembly errors. Some amino acid mutations in proteins can occur in nature without implying a shift in function if they occur outside the protein’s active site and do not alter its three-dimensional structure. Nonetheless, most changes can often lead to reduced, or complete, loss of protein activity [35]. Sequenced fragments obtained from the ~23 kDa band (Figure 4) and fraction 46.2% ACN (Figure 5) could not be conclusive due to the lack of similarity with the transcriptome of *P. depilata*. Therefore, 11 amino acid residues were insufficient to assure identity considering the similar molecular weights. Another reason could be the time at which the venom gland was dissected to perform the transcriptomic analysis. The precursor of this enzyme was not recovered even though there are reports of asynchronous synthesis of venom components during the replenishment of the venom gland [36,37].

Hyaluronidases, which hydrolyze hyaluronic acid and chondroitin sulfate in venoms, are spreading factors, promoting the diffusion of toxins in the prey’s circulation [38,39,40,41]. Hyaluronidase activity has been reported in the venom of different spiders, such as *Lycosa raptorial*, *Ctenus nigriventer* (now *P. nigriventer*) [42], *Cupiennius salei* [43], in spiders of the genera *Lycosa*, *Lampona*, *Loxosceles*, and *Hippasa* [3,39,44,45,46,47], and the theraphosids *Brachypelma vagans* and *Dugesiella hentzi* [48]. Alternatively, other transcriptomics analyses from the Ctenidae family reported hyaluronidase presence in low proportions. For example, in *C. salei*, the enzymes represented 2.2% of the protein sequences [19], and in *P. nigriventer*, only four coding sequences have been described [20]. The presence of hyaluronidases in *P. depilata* venom could explain the rapid development of envenoming signs observed in the toxicity test, such as agitation, tremor, diarrhea, and walking difficulties.

The identity absence in the sequenced fragments of the hyaluronidase activity (band 47.2% ACN fraction) was probably because these sequences were insufficient to determine the activity since only three were found. Another explanation could be that the sequences were not long enough to generate a precise search for a transcript with high identity, with an e-value that ensures that the alignment was not by chance.

The fragments obtained from the 52.6% ACN fraction of the *P. depilata* venom did not match the related hyaluronidase transcripts. However, some additional unassociated bands with this activity could be removed, probably due to their small size and the dark staining gel. In the isolated band from 52.6% ACN fraction, we found 100% identity between the sequenced fragments and the transcriptome sequences related to techylectins and neurotoxins that affect sodium channels. The isolated sample could contain these toxins since they had been reported in this area of the electrophoretic profile in other *Phoneutria* venoms [49]. The high percentages of identity found in these fragments suggested that the *P. depilata* venom gland transcriptome assembly was performed correctly.

Cholinesterases lyse choline esters, many of which are neurotransmitters, such as acetylcholine. The catalytic activity of this enzyme is essential for cholinergic transmission and neuromuscular function [50]. The coding sequences for cholinesterases found in *P. depilata* transcriptome had identity with putative acetylcholinesterases from other spider venoms. The activity of these enzymes in the venom of *P. depilata* could be related to disturbance of the nervous functions causing motor alterations and hypersalivation evidenced in toxicity tests in mice [22,51].

Carboxylesterases, found with a lower relative abundance and expression, hydrolyze carboxylic acid esters into their corresponding acids and alcohols [52]. *Apis mellifera* venom carboxylesterase causes allergic reactions in humans [53]; therefore, we can explain some envenomation signs of the *P. depilata* spider. 

Kinases modify other proteins by chemically adding phosphate groups. They have been found in endoparasite venoms that help them inhibit their host’s immune system [54]. These substances could act as defense toxins for *P. depilata* since they could suppress the prey’s immune system during the bite. It should be noted that there are no reports about the presence of these enzymes in spider venoms; therefore, our results could be highlighted as the first report of a component not previously described in the venom of spiders of this genus. 

Oxidoreductases found in the transcriptomic analysis of *P. depilata* were related to the amino oxidoreductase domain, which comprises the L-amino acid oxidases (L-AAO), flavoenzymes responsible for specifically catalyzing the oxidative deamination of an L-amino acid. The coding sequences for glutamate synthase also have this amino oxidoreductase domain. These enzymes can induce apoptosis of various cell types, including vascular endothelial cells (VEC). Although the mechanism is not well established, it seems to involve H_2_O_2_ production, achieved by oxidation of some proteins of the plasma membrane of the VEC [55]. Other activities of L-AAOs include induction or inhibition of platelet aggregation, anticoagulant activity, stimulation of edema formation, hemorrhage, antibacterial, antiviral, and leishmanicidal functions [55]. 

The pharmacology of phosphodiesterase in spider venoms is unknown, although it has been postulated that it alters extracellular nucleotide levels, contributing to the prey’s death. Additionally, by regulating the concentration of cyclic AMP and cyclic GMP by degradation within cells, phosphodiesterase can generate vasoconstriction and increased blood pressure [16,56]. Phosphodiesterase activity was reported in the venoms of the theraphosids *Aphonopelma robustus* and *Aphonopelma cratus,* also in *Latrodectus mactans,* commonly known as the black widow [16]. Hypertension has been a symptom described during envenoming by spiders of the genus *Phoneutria* [22].

Endonucleases, found in the transcriptome of *P. depilata*, are especially abundant in snake venoms, and they work by releasing nucleotides and nucleosides that control various biological systems, including platelet function. Usually, these nucleosides are stored in large quantities in platelets to be secreted when activated [56]. Like phosphodiesterases, these enzymes could contribute to different effects of envenomation that alter homeostasis, such as blood pressure.

Other enzymes found in the *P. depilata* transcriptome can enhance toxins’ action by breaking down intercellular reinforcements and membrane molecules. This is the case of phosphatases, transferases, and carboxypeptidases, which belong to the hydrolase family [57].

Lipases hydrolyze triglycerides and cholesteryl esters. They have been previously reported in animal venoms, such as snake *Micrurus altirostris*, and their biological contributions to the venom are unknown [58]. Nevertheless, lipases had been found in the venoms of the velvet spider *Stegodyphus mimosarum* and the theraphosid *Acanthoscurria geniculata* [59]. The coding sequence cataloged as ligase is related to the domain associated with SPRY, whose function is unknown. The proteins obtained from animal venoms with this domain have been called serpins (vespryn-Venom PRY-SPRY domain-containing proteins). However, only a few proteins belonging to this family have been identified from the venoms of various snakes and lizards. A member of this family is ohanin, which decreases locomotion and produces hyperalgesia in mice [60]. These signs were evident in mice during the acute toxicity test of the *P. depilata* venom (data not shown). Alternatively, in the transcriptomic analysis of venom glands of the *Chilobrachys jingzhao* tarantula, ligases were associated with metabolism, exerting the same function in the venom of *P. depilata* [61].

Neurotoxins have been extensively studied in the hexathelids *Atrax robustus*, *Hadronyche versuta*, and the brown recluse *Loxosceles* [62,63,64,65]. Consequently, the transcriptomic analysis of venom glands of *P. depilata* revealed numerous transcripts with identity to neurotoxins with the highest expression levels, which was expected due to the venom of *Phoneutria* spiders being characterized by affecting the nervous system. These results agree with the findings in the venom glands of *P. nigriventer* and *P. pertyi* [20,21].

Voltage-gated calcium channels found in the transcriptome of *P. depilata* play a fundamental role in cardiac, muscular, and neuronal functions. The biological function of toxins that alter calcium channels in spider venom could be related to the production of muscle paralysis of the prey by blocking calcium entry and the presynaptic release of neurotransmitters, resulting in flaccid paralysis [66,67]. Alternatively, the action of calcium toxins on calcium channels in the heart may be responsible for arrhythmias generated during envenoming by spiders of this genus.

Toxins that alter voltage-gated sodium channels were also found in the transcriptome. These channels are present mainly in neurons and muscle cells. The toxins generate and propagate the action potential in excitable cells, increasing the sodium ions flux through the membrane. The cells are then intensely and durably depolarized, inducing presynaptic neuronal stimulation, and a massive neurotransmitter release that finally results in paralysis [66,67]. Acetylcholine is among these neurotransmitters, associated with hypersalivation seen in toxicity tests and priapism. Although paralysis was not a sign evidenced in the toxicity tests of *P. depilata* venom, motor alterations were observed, mainly walking difficulties, mediated by this type of neurotoxin.

Our transcriptomic analysis did not find neurotoxin sequences that could alter potassium channels; however, many sequences annotated as neurotoxins have not yet identified their possible target. 

Additionally, sequences with identity to atracotoxins belonging to the family of MIT-type atracotoxins were identified in *P. depilata*. Atracotoxins are related to the non-toxic peptide Atracotoxin-Hvf17 from *Hadronyche versuta*, which lacks insecticidal activity and does not affect smooth muscle contractility [68].

The presence of enzyme inhibitors is essential for regulating enzymes, which may cause damage at the cellular level [69]. Consequently, in the transcriptome of the venom glands of *P. depilata*, we found a directly proportional relationship between the expression levels of the enzymes and their inhibitors. The median expression levels observed for these inhibitors were slightly lower than the expression levels of the enzymes that they inhibit, except for metalloproteases, wherein the expression level of their inhibitor is much higher. Protease inhibitors have been reported in the venom of *L. laeta*, with anticoagulant activity on factor Xa and *L. intermedia* [70,71,72]. Furthermore, serine protease inhibitors have been identified in tarantula venoms with a substantial effect on trypsin and potassium channel blockage [73]. Our transcriptomic analysis of *P. depilata* venom glands agreed with the transcriptomic analysis of other *Phoneutria* species. For example, the enzyme inhibitors stood out in several studies for multiple coding sequences, mostly with action over serine proteases, including Kunitz-type and TIL-type trypsin inhibitors (Trypsin Inhibitor-like), Kazal-type serine protease, and squash family serine protease [20,21]. Additionally, the cystatins found had a thyroglobulin domain, a coding sequence for a metalloprotease inhibitor and other peptidases.

Different growth factors were found in the transcriptomic analysis of venom glands of *P. depilata*. Vascular endothelial growth factor (VEGF) could generate hypotension, angiogenic effects, and increased vascular permeability. VEGF has been reported in snake venoms, such as *Vipera ammodytes ammodytes*, *Vipera aspis*, *Daboia russelli russelli*, and *Bothrops insularis* [74,75]. The growth factor derived from platelets from *P. depilata* could play a protective role in the venom gland by being mitogenic for various connective tissue cells [76]. This factor has been reported in the horned viper *Vipera ammodytes ammodytes*’ venom to induce multiple biological effects, such as endothelial growth, vascular permeability, and hypotension [77].

Insulin-like growth factor-binding protein has been reported in our and other transcriptomic analyses. The mentioned factors are related to protecting the spider’s integrity since this protein could stimulate the growth of venom gland cells, preventing its breakdown by the cytolytic action of some venom components [78]. Furthermore, an insulin-like growth factor-binding protein has been found in the venom and venom gland transcriptome of the king cobra *Ophiophagus hanna*. Although its role has yet to be determined, it was suggested that it could alter insulin signaling pathways, glucose homeostasis and potentially exert toxic effect [19,20,79,80]. Nerve-related growth factor found in *P. depilata* transcriptomes is likely to make the venom injection site more susceptible to toxins and lead to the optimal distribution of substances that would otherwise hardly infiltrate into the body tissue. The nerve-related growth factor of snake *Naja naja atra* can influence the hemodynamic system, decreasing blood pressure in rats [81]. This finding could indicate that this growth factor and those previously mentioned generate hypotension and subsequent homeostasis alteration during *P. depilata* envenomation, but studies to prove this hypothesis are needed.

CRISP proteins are part of the CAP superfamily that includes families such as Antigen-5 and other proteins related to type 1 pathogenicity, which are antifungal proteins produced in plants when attacked by pathogens [82]. Members of this superfamily have been identified in many animals, including venomous animals, as one of the most common components in venoms. The contribution of CRISPs to venom toxicity, their exact molecular targets, and the mechanism of action remains unknown. The SCP domain (sperm-coating protein), typical of these molecules and present in the identified sequences, can function as endopeptidases. This domain can also have a Ca^2+^ chelating function, acting on signaling processes and damaging channels and receptors sensitive to this ion. The use of experimental approaches to reveal the possible actions of CRISP in envenomation, smooth muscle contraction, inflammation, expression induction of vascular endothelial cell adhesion molecules, and angiogenesis inhibition indicates that these molecules play a role in envenomation [19]. In *P. depilata* transcriptome, 36 coding sequences with low expression levels (TPM of 2.3) were identified as CRISP proteins. Even so, some transcripts had a high expression level, indicating that they could be one of the main components in the venom of this spider. Finding these CRISP sequences in our transcriptome analysis agreed with the identification of CRISP proteins in the *P. keyserlingii* venom, besides the abundant presence of putative venom components in the study of the venom gland of *P. nigriventer*. Additionally, CRISP proteins have been reported in the analysis of the venom glands of the Chilean rose tarantula *Grammostola rosea* and *C. salei* [19,83].

Although the physiological functions of TCTP have not yet been explored, it has been suggested that after envenoming, TCTP promotes histamine release from basophils and induces interleukin production from basophils and eosinophils [84,85]. Studies have indicated that histamine released by TCTP binds to their receptors by causing edema, increasing vascular permeability, and vasodilation [86,87]. TCTP or histamine-releasing factors were identified in the spider venoms of *G. rosea*, *L. intermedia*, *L. laeta*, *P. nigriventer*, and the wolf spider *Hogna aspersa* with the help of transcriptomic approaches [71]. These effects have been reported in the *Phoneutria* genus envenomation.

The sequences annotated as fibrinogen-like peptides found in the transcriptomic analysis of venom glands of *P. depilata* had identity with tequilectins. This type of protein with the fibrinogen-like domain has been previously reported in the venom of the dog-faced water snake *Cerberus rynchops*, where the ryncolin 1 and 2 proteins were identified. Tequilectins sequences are similar to ficolins and could be related to platelet aggregation or coagulation and initiate the activation of the lectin complement factor, which is an essential effector system of innate humoral immunity [12,13]. In *P. depilata*, they could exert an effect like that of snake venom, causing platelet aggregation in prey during envenomation, and participating in innate immunity. One of these proteins was reported in the venom of *P. nigriventer* as a lectin involved in innate immunity [50]. Furthermore, it has been reported in the venom glands of other Ctenids [19].

Antimicrobial peptides are present in all living beings and are part of the innate defense system against external agents [88]. Some of these peptides can also modulate the immune system [89,90]. The coding sequences for defensins were reported in the venom glands of *Cuppiennius getazi* and *C. salei* [19]. The transcriptome of venom glands of *P. depilata* threw a small number of sequences related to components with antimicrobial action with relatively low median expression levels, compared to others. This may explain the growth inhibition of *Pseudomonas aeruginosa* and *Staphylococcus aureus* at tested concentrations (data not shown). The coding sequences for von Willebrand factor type Care characteristic of the single domain of von Willebrand factor type C (SVWC) and respond to environmental challenges, such as bacterial infection and nutritional status. They are also involved in antiviral immunity [91].

Furthermore, a sequence of *P. depilata* transcriptome had identity with a diuretic peptide-like HD-31, a hormone that increases cAMP production by activating Na^+^, K^+^, and Cl^2−^ cotransporters. It also increases the secretion of fluids in the Malpighian tubules of several species of insects [92]. The expression level of the diuretic hormone type is considerable compared to other proteins, suggesting an important role during envenomation. Furthermore, activating ion cotransporters in cells may alter the amount of extracellular fluid and generate a homeostasis alteration.

It is common to identify transcripts with annotations as venom components with unknown functions. However, they are annotated even though they do not have an associated domain corresponding to some protein family or other information on activity or molecular target [93]. For *P. depilata* transcriptome, a few sequences with identity toward venom peptides, the functions of which are still unknown were identified, and nor were they related to a PFAM domain that could indicate their activity. Nevertheless, these transcripts have relatively high expression levels, compared to others, suggesting that they are sequences that code for peptides that possibly play an important role in the envenomation of *P. depilata*.

## 4. Conclusions

*P. depilata* venom has been poorly studied since it was confused with *P. boliviensis* until recently, and there are no available records for its envenomation. In this work, a computer algorithm was used, leading to the identification of 682 transcripts coding for putative venom components in the transcriptome of the venom glands of *P. depilata*. Consequently, it makes an enormous difference considering different strategies used for transcriptomic analysis for venom glands of other *Phoneutria*, with a lower number of coding sequences identified. The venomous arsenal is similar within spiders of this genus, mainly neurotoxic peptides with little enzymatic activity.

The value of this analysis lies in the identification of several coding sequences for peptides that had not been previously reported in the venoms of other spiders of this genus, such as carboxylesterases, kinases, oxidoreductases, phosphodiesterases, endonucleases, phosphatases, transferases, carboxypeptidases, lipases, ligases, vascular endothelial growth factor, insulin type, platelet-derived, nerve-related, von Willebrand factor type C and the hormone HD-31. 

This study shows that the venom of the *P. depilata* spider is a vast cocktail of interesting substances that have similarities with those of the other species of the same genus. Different compounds can be obtained with possible pharmacological and agro-industrial use. Therefore, future studies on this venom are promising; however, it is relevant to consider tools that prevent the capture and sacrifice of many specimens, such as those used in this research. 

## 5. Materials and Methods

### 5.1. Spiders’ Collection

Live spiders were captured in rural areigure s1as of Andes, Apartadó, Copacabana, Chigorodó, and Carepa, all of them in Antioquia, Colombia (Supplementary Figure S6). They were kept at the Serpentarium, in the arachnid collection of the University of Antioquia (COLVIOFAR-149), and adequately classified (see Acknowledgement). They were maintained in captivity at room temperature, with a natural light-dark cycle, with earth and wood hideouts, fed with cockroaches and crickets monthly, and having permanent access to water. 

### 5.2. Transcriptomic Analysis

#### 5.2.1. Venom Gland and RNA Extraction

An adult female *P. depilata* spider from Copacabana, Antioquia was euthanized with CO_2_ with permission granted by the Ethics Committee for Animal Experimentation of the Universidad de Antioquia, in act No. 104. The pair of venom glands were extracted and immersed in the TRIzol^®^ reagent (Thermo-Fisher Co.; San José, CA, USA). The gland tissue was homogenized with tungsten beads, and chloroform was used for phase separation. Total RNA was precipitated with isopropanol and finally washed with 75% ethanol. An analysis of RNA integrity was conducted.

#### 5.2.2. Sequencing, Transcriptome Assembly and Annotation

A cDNA library was prepared with an Illumina TruSeq mRNA kit, starting from a concentration of 295.8 nM, with an average fragment size of 305 bp. This library was sequenced on an Illumina Hiseq 2500 instrument with 100-base paired-end reads. The reads were cleaned with a minimum value of Q30 quality and assembled with the Trinity package v 2.5.1 (https://github.com/trinityrnaseq/trinityrnaseq/wiki (accessed on 14 May 2019)), with standard parameters.

The functional annotation of the transcripts was made using the BLAST 2.6.0 tool, seeking identity with the Animal Toxin Annotation Project database of UniProtKB/Swiss-Prot (https://www.uniprot.org/biocuration_project/Toxins/statistics (accessed on 23 May 2019)). A search for similar nucleotide regions with biological sequences already reported from the National Center for Biotechnology Information database was made using the Blastx tool (https://blast.ncbi.nlm.nih.gov/Blast.cgi?PROGRAM=blastx&PAGE_TYPE=BlastSearch&BLAST_SPEC=&LINK_LOC=blasttab&LAST_PAGE=blastp (accessed on 27 May to 28 June 2019)). The molecular mass was determined using ProtParam (http://web.expasy.org/protparam/ (accessed on 27 May to 28 June 2019)). Signal peptides and their cleavage site in proteins obtained from the transcriptome were predicted using the SignalP 5.0 server (http://www.cbs.dtu.dk/services/SignalP-5.0/ (accessed on 27 May to 28 June 2019)). The presence of transmembrane domains in the sequences was evaluated with the TMHMM Server v. 2.0 (http://www.cbs.dtu.dk/services/TMHMM/ (accessed on 27 May to 28 June 2019)).

The selection of sequences related to venom had the following criteria: (i) The transcript is similar to some of the sequences previously reported for the genus *Phoneutria*. (ii) The transcript has sequence identity with any sequences reported in the Animal Toxin Annotation Project. (iii) The e-value of the sequence identified in the transcriptome against the reported databases is less than 1 × 10^−4^. (iv) No transmembrane domains were found on the peptide. (v) The reference protein was secreted from the venom gland. All alignments were performed using the Clustal Omega software, using standard parameters (http://www.ebi.ac.uk/Tools/msa/clustalo/ (accessed on 27 May to 28 June 2019)). 

#### 5.2.3. RNA-Seq Quantification

In silico expression levels of the transcriptome were quantified using the Salmon algorithm v 0.7.2 (https://combine-lab.github.io/salmon/ (accessed on 27 May to 28 June 2019)). The previously obtained Trinity assembly for reads mapping was used as reference, and the standard parameters were used for the quantification per transcript.

### 5.3. Venom Extraction

*P. depilata* venom (males and females) was obtained by extracting the chelicerae using electrostimulation (45 volts). The extractions were performed monthly and one week before feeding. The venom obtained in each extraction was collected in the same vial, lyophilized, and stored at −70 °C until use.

### 5.4. RP-HPLC Profile

Two milligrams of *P. depilata* venom (males and females) were dissolved in 200 μL of solution A (0.1% TFA in water) and centrifuged at 2300× *g*. The supernatant was then applied to a reverse-phase Zorbax 300SB C_18_ column (250 × 4.6 mm; Agilent Technologies, Inc.; Santa Clara, CA, USA) and fractionated using a high-performance liquid chromatography system (HPLC), equipped with a binary pump and diode array detector systems (Agilent, series 1100). Fractions were eluted by a gradient toward solution B (0.1% TFA in acetonitrile –ACN–) as follows: 0% B for 5 min, 5–60% B over 60 min at a flow rate of 1.0 mL/min. The chromatographic run was monitored at 230 nm; fractions were collected, vacuum dried, and stored at −4 °C until used.

### 5.5. Enzymatic Characterization

#### 5.5.1. Phospholipase Activity

The PLA2 activity was determined by lysis of egg-yolk phospholipid in agarose plates, as Haberman & Hardt (1972) proposed with some modifications [94]. Briefly, 2% agarose was dissolved in 10 mL of 0.2 M Tris-HCl, pH 8, and boiled. Once the solution reached 50 °C, 1 mL of 20 mM CaCl_2_, 2 mL of 0.1% Rhodamine 6G, 100 μL of Triton X-100, and 2 mL of 10% egg-yolk solution was added. The solution was gently agitated to mix uniformly all components, then 20 mL of this mixture was poured into Petri dishes. Once the agarose was solidified, 3 mm diameter holes were drilled and 5 µL of sample was deposited. The positive control was 5 µg of a 1 µg/µL concentration of the viperid venom from *Bothrops ammodytoides*. MilliQ water was the negative control. The venom samples from *P. depilata* contain 5, 53 and 106 µg. The egg-yolk agarose plates were incubated at 37 °C for 1 h. The samples with phospholipase activity showed degradation halos visualized under ultraviolet light (UV).

#### 5.5.2. Protease Activity

The protease activity was determined by an electrophoretic analysis proposed by Heussen & Dowdle (1980), with some modifications [95]. Briefly, 12.5% acrylamide gels copolymerized with gelatin (1.5 mg/mL) were used. The viperid venom from *Bothrops ammodytoides* (5 μg) was also used as a positive control. Here, 35 μg of *P. depilata* venom was used. The venom samples were gently mixed with a loading buffer without 2-mercaptoethanol, but they were not heated. The samples were separated under electrophoresis After that, the gel was subjected to different wash processes; that is, the first wash was carried out for 1 h with 0.1 M Tris-HCl, pH 8, and 5% of a Triton X-100 solution; the second wash was performed for 1 h with a 0.1 M Tris-HCl solution, pH 8 and a 0.05% Triton X-100 solution; and the third wash was for 10 min with a 0.1 M Tris-HCl solution, pH 8. These steps were carried out at room temperature and under rotary shaking. Then, the gel was placed in a humid chamber overnight and later stained with Coomassie Brilliant Blue R 250. Samples with protease activity showed clearly visible substrate degradation bands against the blue background of the undigested gelatin.

#### 5.5.3. Hyaluronidase Activity

The hyaluronidase activity was determined by an electrophoretic analysis proposed by Cevallos et al. (1992), with some modifications [96]. Shortly, 12.5% SDS-PAGE gels were copolymerized with hyaluronic acid (0.5 mg/mL). *Brachypelma vagans* spider venom (5 µg) was used as a positive control. *P. depilata* venom (5 µg) and selected fractions (those eluted at 45% and higher of ACN [97,98,99]) were used to test the hyaluronidase activity. The samples for electrophoresis were mixed with a loading buffer without 2-mercaptoethanol and they also were unheated. After electrophoresis, the gel was subjected to different washes; that is, the first was performed for 1 h with 0.2 M acetic acid (HOAc), sodium acetate (NaOAc) 0.2 M, 0.15 M NaCl, pH 3.6, and 5% of a Triton X-100 solution; the second wash was performed for 10 min with a solution containing 0.2 M HOAc, 0.2 M NaOAc, 0.15 M NaCl, pH 3.6. These two steps were conducted at room temperature and under rotary stirring. After that, the gel was placed in a humid chamber overnight. After incubation, the gel was stained under gentle oscillation for at least 5 h and kept in the dark. The staining solution was prepared just before use by mixing 0.1% stains-all (1-ethyl-2-[3-(1-ethylnaphthol[1,2-d] thiazolin-2-ylidene)-2-methylpropenyl] naphtho [1,2-d] thiazolium bromide) with 5% formamide, 20% isopropanol, 0.015 M Tris-HCI, pH 8. Finally, the gel was placed into 5% formamide and 20% isopropanol, 0.015 M Tris-HCl, and pH 8 solution until the activity bands were visible against the deep blue background of undigested hyaluronic acid.

## Figures and Tables

**Table 1:** Nomenclature used for the transcripts identified in the *P. depilata* transcriptome.

Species Code	Meaning	Family Code	Meaning	Subtype Code	Meaning	Example
Phd	*Phoneutria depilata*	Enz	Enzyme	SeP	Serine protease	PhdEnzSeP01
				Cho	Cholinesterase	PhdEnzCho01
				MtP	Matelloprotease	PhdEnzMtP01
				Hya	Hyaluronidase	PhdEnzHya01
				Ces	Carboxylesterase	PhdEnzCes01
				Hyd	Hydrolase	PhdEnzHyd01
				Cxp	Carboxypeptidase	PhdEnzCxp01
				Kin	Kinase	PhdEnzKin01
				Pho	Phosphatase	PhdEnzPho01
				PA2	Phospholipase 2	PhdEnzPA201
				OxR	Oxidoreductase	PhdEnzOxR01
				Tra	Transferase	PhdEnzTra01
				GlS	Glutamate synthase	PhdEnzGlS01
				Phd	Phosphodiesterase	PhdEnzPhd01
				Lig	Ligase	PhdEnzLig01
				PLB	Phospholipase B	PhdEnzPLB01
				End	Endonuclease	PhdEnzEnd01
				Lip	Lipase	PhdEnzLip01
		Ntx	Neurotoxin	Nav	Na-channel toxin	PhdNtxNav01
				Cav	Ca-channel toxin	PhdNtxCav01
				Atx	Atracotoxin	PhdNtxAtx01
				NSp	Nonspecific toxin	PhdNtxNSp01
		PIn	Protease inhibitor	SeP	Serine protease inh.	PhdPInSeP01
				Pep	Peptidase inh.	PhdPInPeP01
				CyP	Cysteine protease inh.	PhdPInCyP01
				MtP	Metalloprotease inh.	PhdPInMtP01
		GrF	Growth factor	Cys	Cysteine rich factor	PhdGrFCyS01
				PDG	Platelet derived factor	PhdGrFPDG01
				Ins	Insulin-like growth factor binding protein	PhdGrFIns01
				NeR	Nerve related factor	PhdGrFNeR01
		Fib	Fibrinogen like	Tec	Techylectin	PhdFibTec01
		TCT	Translationally controlled tumor protein			PhdTCT01
		CRI	CRISP			PhdCRI01
		HDP	Host Defense Peptides	Def	Defensin	PhdHDPDef01
				AMB	Antimicrobial	PhdHDPAMB01
		Oth	Other venom components	vWC	von Willebrand factor type C	PhdOthvWC01
				HDH	DH31 hormone type	PhdOthHDH01
				Und	Undefined	PhdOthUnd01

**Table 2:** Sequences abundance in *P. depilata* venom from transcriptomic analysis of venom glands.

Family	Subtype	Number of Coding Sequences (NCS)	Abundance within the Family (%)	Abundance within the Transcriptome (%)	Median Transcripts per Million (TPM)
Enzyme	Serine proteases	89	37.2	13.1	3.2
Fibrinogen like	Techylectin	78	-	11.4	2.0
Neurotoxin	Nonspecific toxin	75	48.1	11.0	26.1
Protease inhibitor	Serine Proteases Inh.	71	85.5	10.4	2.6
Neurotoxin	Ca-channel toxin	49	31.4	7.2	13.5
Enzyme	Cholinesterases	46	19.3	6.7	1.2
CRISP		36	-	5.3	2.3
Enzyme	Metalloproteinases	35	14.6	5.1	1.7
Growth factor	Cysteine rich growth factor	31	64.6	4.5	3.4
Neurotoxin	Na-channel toxin	26	16.7	3.8	89.1
TCTP		17	-	2.5	4.6
Venom component	Undefined	15	68.2	2.2	6.5
Enzyme	Hyaluronidases	11	4.6	1.6	2.8
Growth factor	Insulin-like growth factor binding protein	10	20.8	1.5	9.6
Enzyme	Carboxylesterases	9	3.8	1.3	16.5
Enzyme	Hydrolases	8	3.4	1.2	2.5
Protease inhibitor	Peptidase inh.	8	9.6	1.2	4.9
Enzyme	Carboxypeptidases	6	2.5	0.9	2.7
Neurotoxin	Atracotoxin	6	3.8	0.9	1.6
Venom component	von Willebrand factor type C	6	27.3	0.9	3.7
Enzyme	Phospholipases A2	5	2.1	0.7	2.3
Enzyme	Kinases	5	2.1	0.7	1.7
Enzyme	Phosphatases	5	2.1	0.7	3.4
Enzyme	Oxidoreductases	5	2.1	0.7	1.1
Enzyme	Transferases	5	2.1	0.7	1.5
Growth factor	Platelet derived growth factor	5	10.4	0.7	1.3
Enzyme	Glutamate synthase	4	1.7	0.6	15.5
Protease inhibitor	Cysteine protease inh.	3	3.6	0.4	20.6
Enzyme	Phosphodiesterases	2	0.8	0.3	83,230
Growth factor	Nerve related growth factor	2	4.2	0.3	3.4
Host Defense Peptide	Defensin	2	66.7	0.3	21.1
Enzyme	Phospholipase B	1	0.4	0.2	13.8
Enzyme	Endonuclease	1	0.4	0.2	7.3
Enzyme	Ligases	1	0.4	0.2	0.8
Enzyme	Lipases	1	0.4	0.2	1
Protease inhibitor	Metalloproteinases Inh.	1	1.2	0.1	8.2
Host Defense Peptide	Antimicrobial	1	33.3	0.1	271
Venom component	DH31 hormone type	1	4.5	0.1	429

## Data Availability

The sequences were uploaded to the European Nucleotide Archive (ENA) under project PRJEB33730.

## Supplementary Materials

The following supporting information can be downloaded at: https://www.mdpi.com/article/10.3390/toxins14050295/s1, Supplementary Material: Distribution of transcripts that putatively code for venom components of *Phoneutria depilata*. Sequences were classified in accordance with Pfam. ID reference protein: UniProt accession numbers. In the amino acid sequences, the predicted signal peptides are underlined. The mature peptides are bolded and underlined. The molecular weight was calculated with ProtParam; the underlined MW represents the weight of the peptide with the signal peptide, the bolded and underlined MW represents the weight of the mature peptide. Stop codons are represented with *. Table S1: RNA-Seq quantification. Sequences were sorted by TPM. Table S2: Comparison of transcriptomic analysis of *Phoneutria* spiders. Figure S1. Phospholipase A2 activity. Figure S2. Alignment of sequenced fragments of the ~30 kDa protease bands (from zymogram) from the whole venom of *P. depilata* and the PhdEnzSeP15 transcript (from the transcriptome with the tblastn tool). Figure S3. Alignment of sequenced fragments of the protease band from fraction 47.2% and the PhdEnzSeP16 transcript (from the transcriptome with the tblastn tool). Figure S4. Alignment of sequenced fragments of hyaluronidase bands from fraction 52.6% ACN and the PhdFibTec40 transcript. Figure S5. Alignment of sequenfced fragments of hyaluronidase bands from fraction 52.6% ACN and the PhdNtxNav24 transcript. Figure S6. Map of Antioquia, highlighting in black circles the areas where the collections were made.
