# Supplementary material for: Transcriptomic Analysis of the Venom Gland and Enzymatic Characterization of the Venom of Phoneutria depilata (Ctenidae) from Colombia
Source: Toxins (Basel). 2022 Apr 21;14(5):295. doi: 10.3390/toxins14050295 (PMC9144723; doi:10.3390/toxins14050295)
Supplement: Supplementary file 1 [file toxins-14-00295-s001.zip › Suplementary_Figures and Tables.pdf]

# Supplementary Materials: Transcriptomic Analysis of the Venom Gland and Enzymatic Characterization of the Venom of *Phoneutria depilata* (Ctenidae) from Colombia

**Julieta Vásquez-Escobar, Teresa Romero-Gutiérrez, José Alejandro Morales, Herlinda C. Clement, Gerardo A. Corzo, Dora M. Benjumea and Ligia Luz Corrales-García**

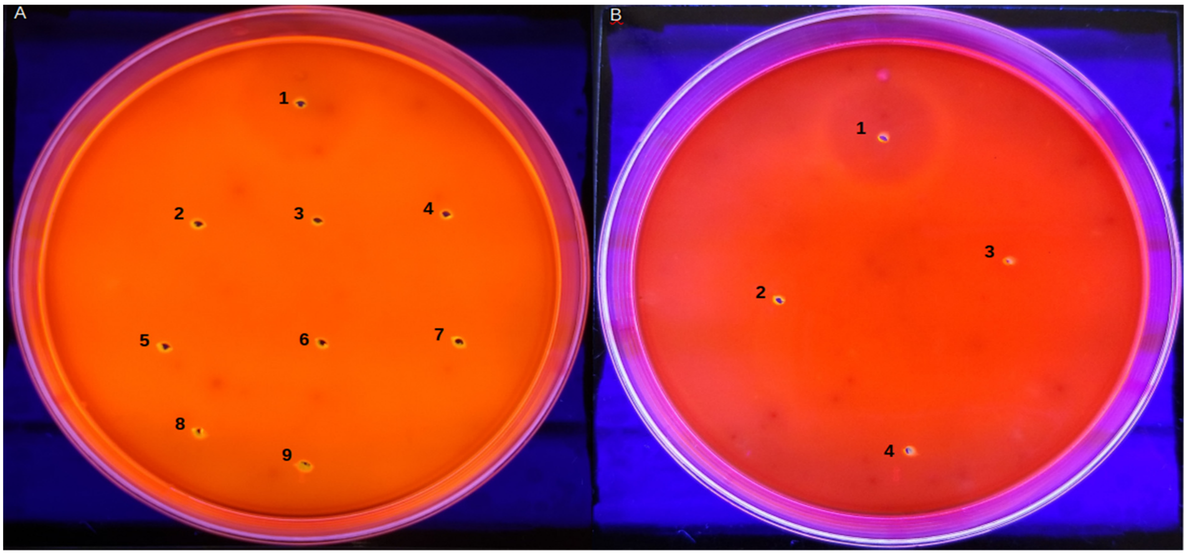


**Figure S1.** Phospholipase A2 activity. Agarose plate with egg yolk. A) Fractions of *P. depilata* venom (5 µg per fraction), 1. C+: *Bothrops ammodytoides* snake venom (5 µg), 2. Fraction 33.2 %, 3. Fraction 35.6, % 4. Fraction 37.0 %, 5. Fraction 37.5 %, 6. Fraction 38.1 %, 7. Fraction 40.2%, 8. Complete venom of *P. depilata* (5 µg), 9. C-: water. B) Complete venom, 1. C+: *Bothrops ammodytoides* snake venom (5 µg), 2. *P. depilata* venom (53.07 µg), 3. *P. depilata* venom (106.14 µg), 4. C -: Water.

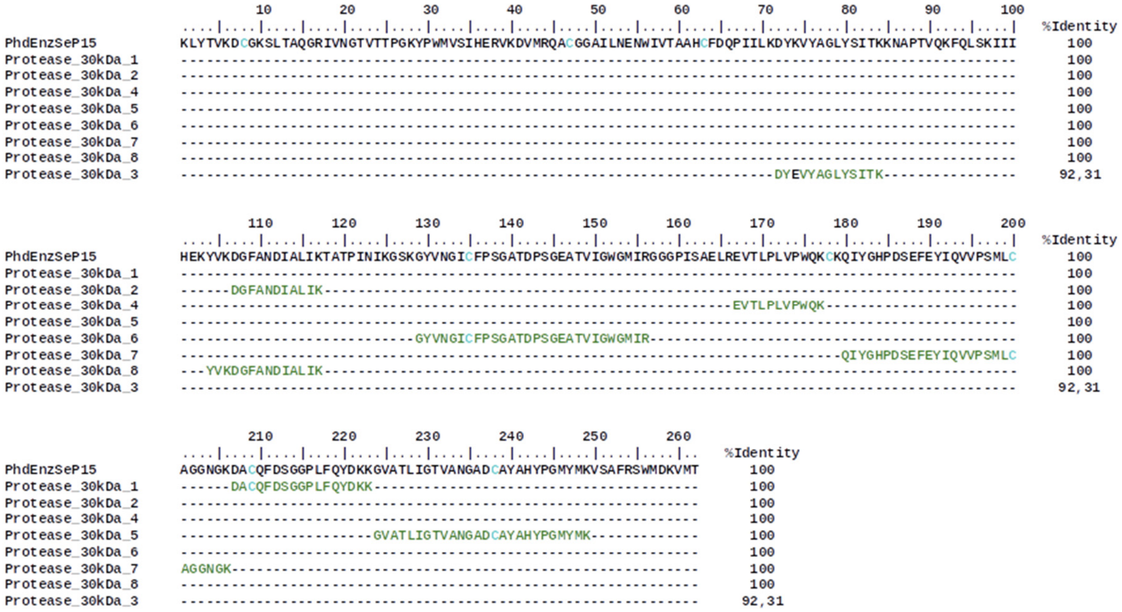


**Figure S2.** Alignment of sequenced fragments of the ~30 kDa protease bands (from zymogram) from the whole venom of *P. depilata* and the PhdEnzSeP15 transcript (from the transcriptome with the tblastn tool). The amino acid matches in the sequences are shown in green.

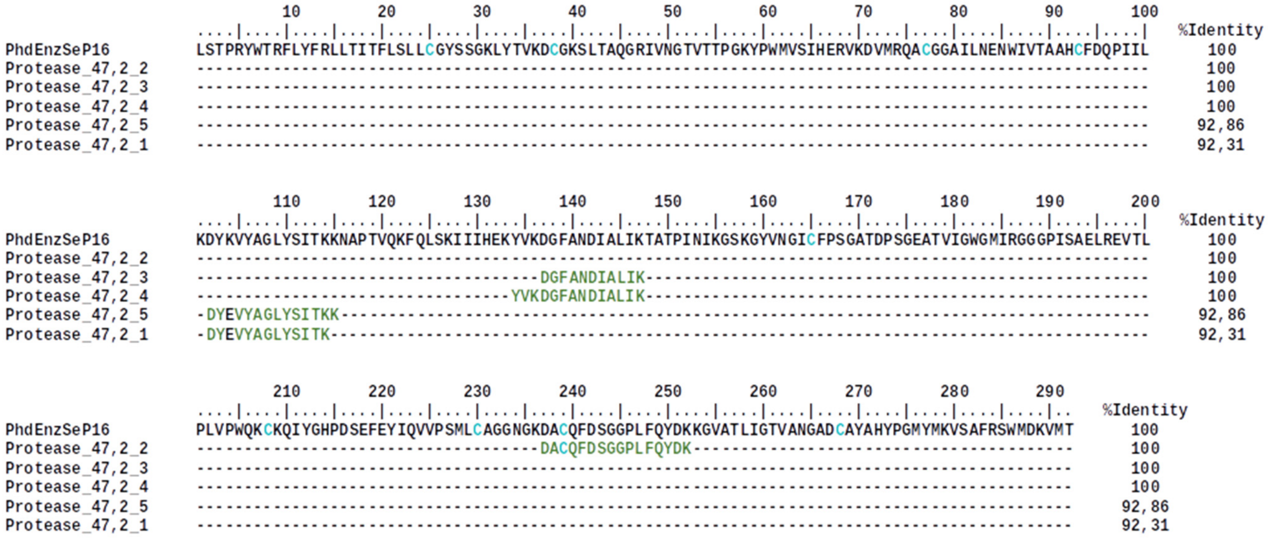


**Figure S3.** Alignment of sequenced fragments of the protease band from fraction 47.2% and the PhdEnzSeP16 transcript (from the transcriptome with the tblastn tool). The amino acid matches in the sequences are shown in green.

```
                  10        20        30        40        50        60        70        80        90       100
          ....|....|....|....|....|....|....|....|....|....|....|....|....|....|....|....|....|....|....|....|  %Identity
PhdFibTec44   ----VCPSPPLPIDCEEVLQRGQSKSGVYKVLPRSRAIPNPIEVYCDMITDGGGWTVIQRRGNFLRPKDYFYQDWQAYKKGFGSIEKDFWLGNDNIFGLS   100
Hasa_52,6_2   DNFPVCPSPPLPIDCEEVLQR-------------------------------------------------------------------------------   100
Hasa_52,6_1   ------------------------------------AIPNPIDVYCDMVTDGGGWTVIQR----------------------------------------   91,67
Hasa_52,6_4   --------------CG--------------------DINAPCQSDCD----CCGYS--------------------------------------------   29,63
Hasa_52,6_3   ----------------------------------------------------------------------------------------------------   18,75

                 110       120       130       140       150       160       170       180       190       200
          ....|....|....|....|....|....|....|....|....|....|....|....|....|....|....|....|....|....|....|....|  %Identity
PhdFibTec44   NQRMCSVRFDLKDVEGSRRHALYDTFWIDDEDHKYMLHINGYKGDAGDSMIGVHNEQKFSTKDQRNDNFPGATSCAQLYKGGWWYNRCHVSNLNGQYLKG   100
Hasa_52,6_2   ----------------------------------------------------------------------------------------------------   100
Hasa_52,6_1   ----------------------------------------------------------------------------------------------------   91,67
Hasa_52,6_4   --------------------VTCDCYWSK-----------------------------------------------------------------------   29,63
Hasa_52,6_3   --GICNLNLALQLITDVR----------------------------------------------------------------------------------   18,75

                 210       220       230
          ....|....|....|....|....|....|....|.  %Identity
PhdFibTec44   KHKSYADGVIWRSWKGYHESLGWTEIKIRSKDFRK*   100
Hasa_52,6_2   ------------------------------------   100
Hasa_52,6_1   ------------------------------------   91,67
Hasa_52,6_4   ------------------------------------   29,63
Hasa_52,6_3   ------------------------------------   18,75
```

**Figure S4.** Alignment of sequenced fragments of hyaluronidase bands from fraction 52.6% ACN and the PhdFibTec40 transcript. The amino acid matches in the sequences are shown in green.

```
                  10        20        30        40        50        60        70        80
          ....|....|....|....|....|....|....|....|....|....|....|....|....|....|....|....|...  %Identity
PhdNtxNav24   MKVAIFFILSLFVLAVASESIEEKREEFPVEESARGKCGDINAPCQSDCDCCGYSVTCDCYWSKDCKCRESNFAAGMALRKAFCKNKI   100
Hasa_52,6_4   -------------------------------------CGDINAPCQSDCDCCGYSVTCDCYWSK------------------------   100
Hasa_52,6_3   ---------GICNLNLALQLITDV.---------------------------------------------------------------   25,00
Hasa_52,6_2   -------------------------DNFPVCP.-----PPLPIDCEEVLQR-------------------------------------   23,81
Hasa_52,6_1   -----------------------------------AIPNPIDVYCDMVTDGGGWTVIQR-----------------------------   20,83
```

**Figure S5.** Alignment of sequenced fragments of hyaluronidase bands from fraction 52.6% ACN and the PhdNtxNav24 transcript. The amino acid matches in the sequences are shown in green.

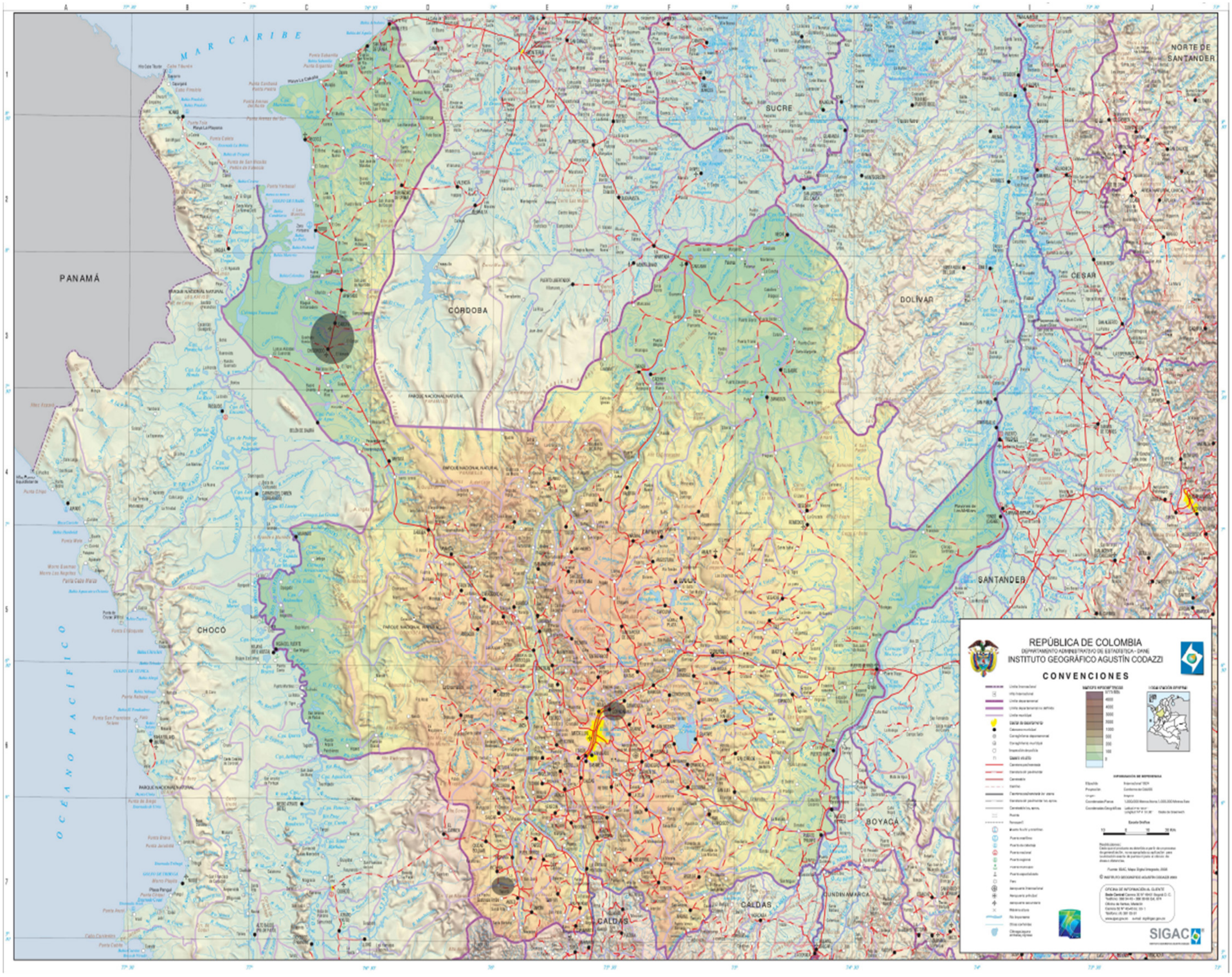


**Figure S6.** Map of Antioquia, highlighting in black circles the areas where the collections were made.

**Table S1.** RNA-Seq quantification

| Transcript ID | Length | Effective Length | TPM | Number of Reads |
|---|---|---|---|---|
| PhdNtxNav14 | 597 | 419443 | 62338.7 | 845088 |
| PhdNtxCav18 | 572 | 394481 | 38429.9 | 489966 |
| PhdNtxNav22 | 562 | 384496 | 32939.7 | 409339 |
| PhdNtxCav25 | 721 | 543373 | 31258.3 | 548952 |
| PhdCRI20 | 2702 | 2524.32 | 29072.8 | 2371930 |
| PhdFibTec39 | 671 | 493388 | 23227.8 | 370397 |

| | | | | |
|---|---|---|---|---|
| PhdNtxCav19 | 516 | 338614 | 20647 | 225961 |
| PhdNtxNav12 | 586 | 408454 | 20268.8 | 267574 |
| PhdEnzSeP15 | 1113 | 935318 | 16175.8 | 488984 |
| PhdNtxNav15 | 572 | 394481 | 15756.6 | 200891 |
| PhdNtxNSp68 | 553 | 375515 | 12658.3 | 153629 |
| PhdNtxNSp70 | 317 | 144875 | 12529.1 | 58666 |
| PhdNtxNSp35 | 569 | 391485 | 10066.4 | 127368 |
| PhdOthUnd07 | 547 | 369532 | 8836.03 | 105531 |
| PhdNtxNSp72 | 991 | 813347 | 6744.19 | 177287 |
| PhdCRI24 | 3579 | 3401.32 | 6298.7 | 692419 |
| PhdEnzCes08 | 983 | 805347 | 4748.99 | 123610 |
| PhdNtxCav16 | 288 | 119004 | 4538.69 | 17456.8 |
| PhdNtxNSp13 | 215 | 61.7821 | 4227.41 | 8441.28 |
| PhdNtxCav43 | 591 | 413449 | 3692.1 | 49336.3 |
| PhdNtxNav04 | 792 | 614358 | 3310 | 65723.6 |
| PhdFibTec38 | 822 | 644355 | 3067.62 | 63884.9 |
| PhdNtxNav06 | 446 | 269095 | 2702.34 | 23502.6 |
| PhdNtxNSp65 | 349 | 174668 | 2407.38 | 13590.4 |
| PhdTCT01 | 765 | 587365 | 2404.81 | 45652 |
| PhdPInSeP56 | 694 | 516379 | 1951.87 | 32575.4 |
| PhdNtxNSp23 | 642 | 464402 | 1837.35 | 27577.6 |
| PhdCRI14 | 1451 | 1273.32 | 1770.33 | 72855.5 |
| PhdNtxNSp39 | 436 | 259214 | 1588.01 | 13304 |
| PhdEnzSeP39 | 993 | 815347 | 1230.25 | 32419.6 |
| PhdNtxNSp58 | 647 | 469401 | 1218.1 | 18479.9 |
| PhdNtxNav08 | 400 | 223829 | 1129.45 | 8170.59 |
| PhdEnzHya08 | 1141 | 963318 | 1112.3 | 34630.9 |

| | | | | |
|---|---|---|---|---|
| PhdNtxCav35 | 545 | 367.54 | 957835 | 11378 |
| PhdEnzSeP10 | 1237 | 1059.32 | 928923 | 31803.7 |
| PhdNtxCav06 | 925 | 747349 | 913385 | 22062.2 |
| PhdNtxCav36 | 1599 | 1421.32 | 851.71 | 39125 |
| PhdCRI15 | 1510 | 1332.32 | 772141 | 33248.8 |
| PhdNtxNSp57 | 518 | 340608 | 770054 | 8477.11 |
| PhdEnzSeP37 | 741 | 563.37 | 734557 | 13374.9 |
| PhdNtxNav11 | 2783 | 2605.32 | 630262 | 53070.4 |
| PhdEnzCho36 | 5783 | 5605.32 | 519415 | 94099.1 |
| PhdNtxCav04 | 3782 | 3604.32 | 496818 | 57875.1 |
| PhdNtxNSp02 | 417 | 240498 | 450707 | 3503.29 |
| PhdNtxNSp69 | 553 | 375515 | 429507 | 5212.77 |
| PhdOthHDH01 | 664 | 486.39 | 428.94 | 6743 |
| PhdNtxNSp42 | 612 | 434426 | 418929 | 5882.03 |
| PhdNtxNSp44 | 612 | 434426 | 384072 | 5392.61 |
| PhdEnzCho24 | 5776 | 5598.32 | 362704 | 65626.8 |
| PhdNtxNSp05 | 426 | 249346 | 359659 | 2898.44 |
| PhdNtxNSp40 | 497 | 319695 | 348414 | 3600 |
| PhdEnzHya11 | 4002 | 3824.32 | 339005 | 41901.6 |
| PhdNtxNSp34 | 1031 | 853318 | 307603 | 8483.46 |
| PhdTCT10 | 878 | 700349 | 303693 | 6874.17 |
| PhdHDPAMB01 | 1760 | 1582.32 | 271 | 13859.1 |
| PhdNtxNSp64 | 283 | 114691 | 263839 | 978 |
| PhdCRI08 | 4893 | 4715.32 | 247962 | 37789.2 |
| PhdFibTec25 | 1246 | 1068.32 | 234129 | 8084 |
| PhdEnzSeP33 | 1394 | 1216.32 | 227418 | 8940.12 |
| PhdNtxNSp33 | 1093 | 915318 | 217386 | 6430.93 |

| | | | | |
|---|---|---|---|---|
| PhdNtxCav02 | 501 | 323673 | 209608 | 2192.73 |
| PhdNtxNSp38 | 620 | 442419 | 201972 | 2888 |
| PhdEnzSeP16 | 1248 | 1070.32 | 191652 | 6629.75 |
| PhdNtxNav03 | 341 | 167111 | 174421 | 942052 |
| PhdEnzPhd01 | 1603 | 1425.32 | 166456 | 7668 |
| PhdEnzCho34 | 5770 | 5592.32 | 163403 | 29534 |
| PhdNtxNSp32 | 614 | 436424 | 147754 | 2084.1 |
| PhdEnzSeP26 | 326 | 153.12 | 140927 | 697425 |
| PhdNtxNSp63 | 999 | 821318 | 139589 | 3705.39 |
| PhdEnzCho37 | 5763 | 5585.32 | 134208 | 24226.8 |
| PhdNtxNSp04 | 2634 | 2456.32 | 130853 | 10388.2 |
| PhdEnzSeP11 | 2641 | 2463.32 | 129072 | 10276 |
| PhdNtxCav07 | 2641 | 2463.32 | 118535 | 9437.08 |
| PhdNtxNSp59 | 2038 | 1860.32 | 117208 | 7047.19 |
| PhdNtxNav23 | 1413 | 1235.32 | 112951 | 4509.64 |
| PhdNtxNSp37 | 511 | 333628 | 112123 | 1209 |
| PhdNtxCav31 | 2174 | 1996.32 | 111688 | 7206.19 |
| PhdFibTec01 | 2428 | 2250.32 | 109611 | 7972 |
| PhdGrFIns01 | 1137 | 959318 | 109473 | 3394.24 |
| PhdNtxNSp03 | 346 | 171829 | 108.78 | 604113 |
| PhdNtxNSp46 | 1708 | 1530.32 | 104802 | 5183.48 |
| PhdNtxNSp12 | 978 | 800347 | 98.3892 | 2545.06 |
| PhdNtxNav24 | 571 | 393482 | 98.2268 | 1249.18 |
| PhdFibTec71 | 1953 | 1775.32 | 97.8087 | 5612.09 |
| PhdNtxNSp56 | 2592 | 2414.32 | 90.0823 | 7029.19 |
| PhdNtxNSp25 | 505 | 327655 | 89.4257 | 947 |
| PhdEnzSeP23 | 4061 | 3883.32 | 82.5635 | 10362.4 |

| | | | | |
|---|---|---|---|---|
| PhdNtxNSp19 | 831 | 653354 | 81.7127 | 1725.48 |
| PhdNtxNav07 | 440 | 263162 | 80.0092 | 680509 |
| PhdNtxCav34 | 251 | 88.2481 | 78.9832 | 225274 |
| PhdPInSeP06 | 544 | 366543 | 74.6201 | 884 |
| PhdNtxNSp60 | 1076 | 898318 | 65.6897 | 1907.21 |
| PhdNtxNSp16 | 756 | 578365 | 65.4406 | 1223.26 |
| PhdNtxCav01 | 238 | 78226 | 64.3697 | 162744 |
| PhdFibTec24 | 1624 | 1446.32 | 62.8945 | 2940 |
| PhdNtxNSp61 | 1922 | 1744.32 | 62.7516 | 3537.71 |
| PhdNtxCav41 | 637 | 459405 | 54.8151 | 813892 |
| PhdEnzSeP24 | 6185 | 6007.32 | 53868 | 10458.8 |
| PhdPInSeP41 | 800 | 622358 | 53.6968 | 1080.09 |
| PhdPInCyP01 | 1003 | 825318 | 52.8599 | 1410 |
| PhdEnzSeP13 | 2118 | 1940.32 | 52.4331 | 3288.13 |
| PhdEnzSeP43 | 2952 | 2774.32 | 51.7091 | 4636.55 |
| PhdPInSeP62 | 9626 | 9448.32 | 51096 | 15603.2 |
| PhdEnzSeP78 | 1162 | 984318 | 49.8536 | 1586 |
| PhdNtxNav09 | 459 | 281.96 | 48.9674 | 446237 |
| PhdGrFIns05 | 624 | 446412 | 47.8951 | 691032 |
| PhdTCT12 | 1036 | 858318 | 47.6986 | 1323.2 |
| PhdCRI17 | 1538 | 1360.32 | 46.9673 | 2064.94 |
| PhdFibTec08 | 1326 | 1148.32 | 46.1099 | 1711.31 |
| PhdNtxNav16 | 208 | 57.0718 | 46.1019 | 85.0377 |
| PhdEnzPA205 | 1268 | 1090.32 | 45.6028 | 1607 |
| PhdNtxNSp10 | 1776 | 1598.32 | 45.4524 | 2347.96 |
| PhdNtxCav20 | 423 | 246392 | 44.6375 | 355466 |
| PhdEnzMtP14 | 871 | 693.35 | 43.9554 | 985 |

| | | | | |
|---|---|---|---|---|
| PhdCRI32 | 275 | 107865 | 43027 | 150 |
| PhdPInSeP54 | 1232 | 1054.32 | 42.1123 | 1435 |
| PhdNtxNav18 | 764 | 586365 | 42.1017 | 797883 |
| PhdPInSeP42 | 800 | 622358 | 40.8057 | 820789 |
| PhdFibTec49 | 535 | 357567 | 39.8845 | 460928 |
| PhdEnzCxp05 | 1628 | 1450.32 | 39.8843 | 1869.55 |
| PhdNtxCav03 | 238 | 78226 | 38.3663 | 97 |
| PhdHDPDef02 | 336 | 162414 | 37.7198 | 198 |
| PhdFibTec22 | 422 | 245408 | 34.5454 | 274 |
| PhdNtxCav38 | 555 | 377.51 | 34.4468 | 420289 |
| PhdOthvWC01 | 573 | 395.48 | 32546 | 416 |
| PhdEnzCho25 | 428 | 251315 | 32.3127 | 262.46 |
| PhdEnzCxp01 | 2061 | 1883.32 | 31.4611 | 1915 |
| PhdPInSeP39 | 925 | 747349 | 30.5062 | 736857 |
| PhdPInPep08 | 709 | 531376 | 30.1505 | 517807 |
| PhdNtxNSp73 | 211 | 59.0704 | 29.7983 | 56.8895 |
| PhdPInSeP22 | 831 | 653354 | 29.6452 | 626 |
| PhdEnzSeP45 | 948 | 770347 | 29.0206 | 722545 |
| PhdFibTec02 | 799 | 621358 | 28.8029 | 578429 |
| PhdNtxCav26 | 1211 | 1033.32 | 27.3761 | 914273 |
| PhdEnzGlS04 | 4913 | 4735.32 | 27.2274 | 4167.03 |
| PhdNtxCav48 | 4762 | 4584.32 | 26.7013 | 3956.2 |
| PhdNtxNSp28 | 747 | 569369 | 26.1271 | 480.79 |
| PhdTCT11 | 696 | 518379 | 24.7906 | 415342 |
| PhdNtxNav05 | 695 | 517379 | 24.0668 | 402437 |
| PhdEnzSeP27 | 255 | 91.4268 | 24.0068 | 70938 |
| PhdCRI16 | 1451 | 1273.32 | 23.7693 | 978193 |

| | | | | |
|---|---|---|---|---|
| PhdEnzSeP34 | 4811 | 4633.32 | 23.4749 | 3515.33 |
| PhdNtxCav21 | 522 | 344596 | 22.0248 | 245297 |
| PhdEnzOxR01 | 1991 | 1813.32 | 21.9942 | 1289 |
| PhdOthUnd05 | 689 | 511379 | 21.4302 | 354193 |
| PhdNtxNSp22 | 784 | 606359 | 21.3042 | 417509 |
| PhdNtxCav39 | 321 | 148522 | 21.2982 | 102236 |
| PhdEnzGlS03 | 8176 | 7998.32 | 20.9744 | 5421.99 |
| PhdOthUnd03 | 384 | 208268 | 20.9471 | 141 |
| PhdEnzSeP44 | 998 | 820341 | 20.7027 | 548898 |
| PhdNtxNSp27 | 371 | 195699 | 20.4908 | 129604 |
| PhdPInSeP37 | 681 | 503385 | 19.8974 | 323719 |
| PhdNtxNSp31 | 802 | 624358 | 19.8684 | 400929 |
| PhdEnzCho11 | 804 | 626358 | 19716 | 399127 |
| PhdEnzSeP07 | 1460 | 1282.32 | 19.6166 | 813 |
| PhdNtxNSp62 | 2828 | 2650.32 | 19.2826 | 1651.72 |
| PhdFibTec26 | 1214 | 1036.32 | 19.1677 | 642 |
| PhdOthUnd01 | 1122 | 944318 | 17988 | 549 |
| PhdEnzSeP32 | 1428 | 1250.32 | 17.9845 | 726757 |
| PhdEnzSeP81 | 2298 | 2120.32 | 17.7444 | 1216 |
| PhdEnzCxp02 | 579 | 401467 | 17.5035 | 227116 |
| PhdEnzCho32 | 7619 | 7441.32 | 17441 | 4194.62 |
| PhdTCT13 | 714 | 536374 | 16.7326 | 290069 |
| PhdEnzSeP46 | 1163 | 985318 | 16.2013 | 515939 |
| PhdNtxNSp52 | 2351 | 2173.32 | 15.7907 | 1109.17 |
| PhdGrFIns02 | 459 | 281.96 | 15.6652 | 142756 |
| PhdEnzCxp03 | 1255 | 1077.32 | 15.5917 | 542884 |
| PhdEnzSeP28 | 2382 | 2204.32 | 15.5611 | 1108.63 |

| | | | | |
|---|---|---|---|---|
| PhdTCT04 | 739 | 561.37 | 15.2428 | 276558 |
| PhdFibTec36 | 1230 | 1052.32 | 15221 | 517681 |
| PhdNtxNav10 | 812 | 634357 | 15.1209 | 310015 |
| PhdNtxNav17 | 1490 | 1312.32 | 14983 | 635489 |
| PhdPInSeP26 | 331 | 157758 | 14.9057 | 76 |
| PhdNtxCav13 | 558 | 380503 | 14.8379 | 182474 |
| PhdEnzSeP42 | 3032 | 2854.32 | 14.7565 | 1361.31 |
| PhdEnzSeP14 | 1971 | 1793.32 | 14717 | 853 |
| PhdNtxCav11 | 1649 | 1471.32 | 14.6565 | 696961 |
| PhdNtxNSp71 | 210 | 58.4032 | 14304 | 27 |
| PhdTCT08 | 2017 | 1839.32 | 14.0013 | 832333 |
| PhdOthUnd04 | 509 | 331635 | 13.9013 | 149 |
| PhdPInSeP04 | 593 | 415448 | 13864 | 186156 |
| PhdEnzPLB01 | 1973 | 1795.32 | 13.8045 | 801 |
| PhdPInSeP45 | 695 | 517379 | 13635 | 228 |
| PhdNtxCav14 | 409 | 232641 | 13.5409 | 101813 |
| PhdEnzSeP35 | 919 | 741349 | 13.4939 | 323319 |
| PhdPInSeP57 | 596 | 418444 | 13.4708 | 182.18 |
| PhdEnzMtP08 | 1821 | 1643.32 | 12.8973 | 685 |
| PhdEnzCho30 | 7619 | 7441.32 | 12.8304 | 3085.76 |
| PhdOthUnd08 | 619 | 441.42 | 12808 | 182728 |
| PhdFibTec35 | 1045 | 867318 | 12.5818 | 352688 |
| PhdOthUnd12 | 4450 | 4272.32 | 12.1993 | 1684.49 |
| PhdPInSeP36 | 797 | 619358 | 11.9966 | 240143 |
| PhdGrFIns06 | 520 | 342602 | 11.9363 | 132.17 |
| PhdNtxNSp41 | 695 | 517379 | 11.9104 | 199162 |
| PhdPInSeP03 | 800 | 622358 | 11.7748 | 236844 |

| | | | | |
|---|---|---|---|---|
| PhdEnzTra05 | 4641 | 4463.32 | 11.6981 | 1687.5 |
| PhdNtxNav19 | 1929 | 1751.32 | 11.5571 | 654159 |
| PhdNtxNav02 | 2668 | 2490.32 | 11515 | 926.81 |
| PhdEnzPA203 | 1231 | 1053.32 | 11.5148 | 392 |
| PhdEnzKin01 | 6682 | 6504.32 | 11.2804 | 2371.36 |
| PhdEnzMtP30 | 503 | 325664 | 11.0467 | 116271 |
| PhdCRI07 | 695 | 517379 | 11.0037 | 184 |
| PhdNtxNSp66 | 709 | 531376 | 10.9274 | 187668 |
| PhdNtxNav26 | 1645 | 1467.32 | 10.7543 | 510.01 |
| PhdGrFIns07 | 477 | 299821 | 10.7325 | 104 |
| PhdNtxCav17 | 494 | 316.71 | 10.5041 | 107.52 |
| PhdPInSeP38 | 672 | 494388 | 10.1562 | 162281 |
| PhdNtxNSp08 | 448 | 271073 | 10.0707 | 88.2303 |
| PhdPInSeP43 | 636 | 458406 | 10.0569 | 149 |
| PhdEnzGlS02 | 8127 | 7949.32 | 9.98327 | 2564.92 |
| PhdNtxCav22 | 1088 | 910318 | 9.96047 | 293052 |
| PhdEnzSeP02 | 1194 | 1016.32 | 9.89424 | 325 |
| PhdNtxAtx03 | 821 | 643355 | 9.83207 | 204441 |
| PhdNtxCav49 | 787 | 609359 | 9.80014 | 193009 |
| PhdNtxCav05 | 601 | 423438 | 9.72034 | 133028 |
| PhdNtxCav23 | 594 | 416446 | 9.34152 | 125732 |
| PhdEnzCxp06 | 1629 | 1451.32 | 9.33023 | 437649 |
| PhdFibTec50 | 508 | 330.64 | 9.2475 | 98.8212 |
| PhdEnzSeP21 | 3959 | 3781.32 | 8.85506 | 1082.2 |
| PhdGrFCys12 | 4249 | 4071.32 | 8.81176 | 1159.49 |
| PhdNtxNav13 | 2915 | 2737.32 | 8.79589 | 778173 |
| PhdPInSeP28 | 480 | 302802 | 8.5832 | 84 |

| | | | | |
|---|---|---|---|---|
| PhdGrFIns04 | 1845 | 1667.32 | 8.49857 | 457968 |
| PhdPInSeP69 | 7107 | 6929.32 | 8.26057 | 1850 |
| PhdCRI33 | 710 | 532376 | 8.24111 | 141.8 |
| PhdPInMtP01 | 851 | 673353 | 8.22506 | 179 |
| PhdCRI09 | 4609 | 4431.32 | 8.07614 | 1156.67 |
| PhdNtxNSp09 | 5882 | 5704.32 | 8.05471 | 1484.99 |
| PhdNtxCav24 | 608 | 430431 | 8.01677 | 111526 |
| PhdEnzSeP52 | 2490 | 2312.32 | 7.93107 | 592721 |
| PhdPInCyP02 | 588 | 410453 | 7.91508 | 105 |
| PhdNtxNSp54 | 2159 | 1981.32 | 7.84485 | 502354 |
| PhdFibTec28 | 1202 | 1024.32 | 7.54418 | 249757 |
| PhdFibTec54 | 1595 | 1417.32 | 7.48783 | 343 |
| PhdPInSeP27 | 331 | 157758 | 7.45284 | 38 |
| PhdEnzCho15 | 7606 | 7428.32 | 7.38998 | 1774.21 |
| PhdFibTec03 | 797 | 619358 | 7.36802 | 147.49 |
| PhdEnzEnd01 | 217 | 63.1479 | 7.34956 | 15 |
| PhdPInSeP46 | 478 | 300815 | 7.30278 | 71 |
| PhdCRI05 | 1906 | 1728.32 | 7.30109 | 407833 |
| PhdFibTec43 | 916 | 738349 | 7.09097 | 169215 |
| PhdNtxNSp36 | 341 | 167111 | 7.03572 | 38 |
| PhdCRI18 | 629 | 451408 | 6.96477 | 101612 |
| PhdEnzHyd03 | 1767 | 1589.32 | 6.89162 | 354 |
| PhdOthUnd11 | 449 | 272062 | 6.78701 | 59.6784 |
| PhdEnzSeP47 | 1015 | 837318 | 6.76577 | 183096 |
| PhdEnzMtP19 | 742 | 564.37 | 6.75818 | 123272 |
| PhdPInSeP30 | 478 | 300815 | 6.68564 | 65 |
| PhdEnzSeP77 | 1540 | 1362.32 | 6.53921 | 287922 |

| | | | | |
|---|---|---|---|---|
| PhdEnzHyd06 | 2222 | 2044.32 | 6.41721 | 424 |
| PhdOthUnd13 | 589 | 411451 | 6.39189 | 85 |
| PhdNtxNSp20 | 853 | 675353 | 6.27045 | 136868 |
| PhdGrFPDG01 | 1105 | 927318 | 6.20602 | 186 |
| PhdEnzSeP25 | 3594 | 3416.32 | 6.20385 | 685 |
| PhdNtxNav25 | 1091 | 913318 | 6.1723 | 182197 |
| PhdEnzMtP09 | 2388 | 2210.32 | 6.13124 | 438 |
| PhdEnzMtP22 | 3191 | 3013.32 | 6.12012 | 596041 |
| PhdNtxCav47 | 702 | 524377 | 6.09911 | 103367 |
| PhdNtxNSp29 | 560 | 382.5 | 6.06671 | 74999 |
| PhdOthUnd06 | 1089 | 911318 | 6.02239 | 177382 |
| PhdPInSeP58 | 1210 | 1032.32 | 5.93748 | 198101 |
| PhdEnzTra03 | 1713 | 1535.32 | 5.92616 | 294065 |
| PhdEnzCho23 | 6452 | 6274.32 | 5.85163 | 1186.63 |
| PhdPInSeP70 | 7547 | 7369.32 | 5.84293 | 1391.65 |
| PhdCRI23 | 4080 | 3902.32 | 5.74147 | 724.13 |
| PhdFibTec07 | 1173 | 995318 | 5.69788 | 183293 |
| PhdFibTec13 | 576 | 398475 | 5.66827 | 73 |
| PhdEnzSeP38 | 2887 | 2709.32 | 5.60884 | 491139 |
| PhdGrFCys09 | 4378 | 4200.32 | 5.5909 | 758988 |
| PhdTCT07 | 1030 | 852318 | 5.5294 | 152318 |
| PhdEnzCho27 | 7606 | 7428.32 | 5.49367 | 1318.94 |
| PhdPInSeP49 | 1717 | 1539.32 | 5.38686 | 268 |
| PhdFibTec29 | 744 | 566.37 | 5.33566 | 97.6696 |
| PhdNtxCav44 | 703 | 525377 | 5.32897 | 90.4868 |
| PhdFibTec51 | 498 | 320.69 | 5.12045 | 53.0719 |
| PhdEnzCho28 | 1467 | 1289.32 | 5.11548 | 213166 |

| | | | | |
|---|---|---|---|---|
| PhdEnzSeP48 | 3197 | 3019.32 | 5.10191 | 497866 |
| PhdFibTec19 | 277 | 109556 | 5.08351 | 18 |
| PhdFibTec23 | 219 | 64.5114 | 5.06321 | 10.5568 |
| PhdEnzSeP36 | 415 | 238.53 | 5.04022 | 38.8565 |
| PhdNtxCav12 | 560 | 382.5 | 5.00729 | 61902 |
| PhdNtxNSp50 | 1066 | 888318 | 4.89584 | 140562 |
| PhdEnzMtP18 | 709 | 531376 | 4.87525 | 83.7279 |
| PhdEnzCho10 | 718 | 540373 | 4.65668 | 81.3283 |
| PhdTCT09 | 2251 | 2073.32 | 4.61901 | 309518 |
| PhdPInSeP17 | 3339 | 3161.32 | 4.60229 | 470233 |
| PhdHDPDef01 | 306 | 134.93 | 4.58616 | 20 |
| PhdPInSeP71 | 7544 | 7366.32 | 4.50418 | 1072.35 |
| PhdEnzSeP12 | 2271 | 2093.32 | 4.41942 | 299 |
| PhdEnzMtP33 | 1131 | 953318 | 4.41669 | 136084 |
| PhdOthvWC04 | 967 | 789347 | 4.4073 | 112438 |
| PhdEnzSeP09 | 1257 | 1079.32 | 4.39538 | 153326 |
| PhdCRI36 | 1194 | 1016.32 | 4.38391 | 144 |
| PhdNtxCav42 | 848 | 670353 | 4.35896 | 94.4403 |
| PhdEnzSeP63 | 3104 | 2926.32 | 4.35617 | 412 |
| PhdOthvWC02 | 824 | 646355 | 4.35611 | 91 |
| PhdEnzSeP29 | 2398 | 2220.32 | 4.33791 | 311291 |
| PhdFibTec45 | 593 | 415448 | 4.32843 | 58.1189 |
| PhdEnzMtP29 | 2746 | 2568.32 | 4.30076 | 356997 |
| PhdPInSeP40 | 1104 | 926318 | 4.2506 | 127257 |
| PhdCRI27 | 1054 | 876318 | 4.23542 | 119958 |
| PhdNtxNav01 | 311 | 139418 | 4.2166 | 19 |
| PhdEnzPhd02 | 2539 | 2361.32 | 4.17989 | 319 |

| | | | | |
|---|---|---|---|---|
| PhdEnzCho12 | 2648 | 2470.32 | 4.11124 | 328244 |
| PhdFibTec72 | 3145 | 2967.32 | 4.04162 | 387607 |
| PhdPInSeP07 | 5498 | 5320.32 | 3.97784 | 684 |
| PhdFibTec20 | 1018 | 840318 | 3.97657 | 108 |
| PhdGrFIns10 | 491 | 313.73 | 3.94488 | 40 |
| PhdEnzSeP17 | 1404 | 1226.32 | 3.91073 | 155 |
| PhdFibTec05 | 778 | 600.36 | 3.83998 | 74.5096 |
| PhdEnzSeP30 | 2627 | 2449.32 | 3.83974 | 303961 |
| PhdTCT02 | 1188 | 1010.32 | 3.75182 | 122.51 |
| PhdEnzSeP31 | 1516 | 1338.32 | 3.74758 | 162099 |
| PhdGrFNeR02 | 916 | 738349 | 3.73169 | 89051 |
| PhdEnzSeP68 | 3281 | 3103.32 | 3.61724 | 362807 |
| PhdCRI34 | 1594 | 1416.32 | 3.60456 | 165 |
| PhdPInSeP33 | 1925 | 1747.32 | 3.58554 | 202487 |
| PhdFibTec09 | 270 | 103675 | 3.58124 | 12 |
| PhdEnzHya10 | 3561 | 3383.32 | 3.54381 | 387511 |
| PhdEnzCho08 | 267 | 101178 | 3.50848 | 11473 |
| PhdEnzSeP51 | 3909 | 3731.32 | 3.4635 | 417684 |
| PhdEnzHya01 | 2445 | 2267.32 | 3.46287 | 253758 |
| PhdNtxCav37 | 2989 | 2811.32 | 3.41763 | 310532 |
| PhdFibTec40 | 847 | 669353 | 3.4157 | 73.8935 |
| PhdNtxNSp24 | 4539 | 4361.32 | 3.40475 | 479926 |
| PhdTCT15 | 1592 | 1414.32 | 3.37236 | 154153 |
| PhdPInSeP35 | 1748 | 1570.32 | 3.35939 | 170498 |
| PhdNtxAtx05 | 724 | 546373 | 3.34901 | 59.1393 |
| PhdPInSeP08 | 3592 | 3414.32 | 3.32562 | 366984 |
| PhdEnzSeP76 | 983 | 805347 | 3.30403 | 86 |

| | | | | |
|---|---|---|---|---|
| PhdEnzHya02 | 1106 | 928318 | 3.30012 | 99.0143 |
| PhdPInSeP23 | 725 | 547373 | 3.27849 | 58 |
| PhdPInPep04 | 640 | 462402 | 3.24383 | 48.4785 |
| PhdEnzHyd07 | 2446 | 2268.32 | 3.20548 | 235 |
| PhdEnzCho13 | 2635 | 2457.32 | 3.18551 | 252995 |
| PhdEnzSeP67 | 3327 | 3149.32 | 3.17893 | 323.57 |
| PhdEnzCes06 | 727 | 549373 | 3.12745 | 55.5301 |
| PhdGrFNeR01 | 919 | 741349 | 3.0863 | 73949 |
| PhdNtxNav21 | 334 | 160546 | 3.05303 | 15.8417 |
| PhdOthvWC06 | 968 | 790347 | 3.03943 | 77.6392 |
| PhdFibTec66 | 455 | 277999 | 3.00503 | 27 |
| PhdEnzCes05 | 721 | 543373 | 2.98764 | 52.4684 |
| PhdPInPep06 | 2443 | 2265.32 | 2.9229 | 214 |
| PhdEnzCes04 | 3180 | 3002.32 | 2.89692 | 281103 |
| PhdEnzSeP88 | 4494 | 4316.32 | 2.88165 | 402 |
| PhdEnzSeP69 | 3137 | 2959.32 | 2.80854 | 268623 |
| PhdEnzHya09 | 1313 | 1135.32 | 2.80704 | 103 |
| PhdEnzMtP31 | 388 | 212144 | 2.7981 | 19.1852 |
| PhdPInSeP47 | 364 | 188974 | 2.7834 | 17 |
| PhdCRI04 | 1970 | 1792.32 | 2.7822 | 161167 |
| PhdFibTec52 | 645 | 467401 | 2.73408 | 41.3021 |
| PhdEnzSeP49 | 767 | 589365 | 2.71883 | 51.7889 |
| PhdEnzCes07 | 1055 | 877318 | 2.71062 | 76.8594 |
| PhdPInSeP66 | 4667 | 4489.32 | 2.70858 | 393 |
| PhdFibTec33 | 1641 | 1463.32 | 2.70801 | 128074 |
| PhdNtxNav20 | 337 | 163352 | 2.68174 | 14.1583 |
| PhdNtxNSp11 | 1702 | 1524.32 | 2.63946 | 130035 |

| | | | | |
|---|---|---|---|---|
| PhdFibTec53 | 541 | 363552 | 2.6278 | 30.8767 |
| PhdPInSeP09 | 3417 | 3239.32 | 2.58343 | 270472 |
| PhdEnzHyd01 | 2103 | 1925.32 | 2.57126 | 160 |
| PhdNtxNSp48 | 276 | 108709 | 2.56156 | 9 |
| PhdOthvWC03 | 688 | 510.38 | 2.55372 | 42.1248 |
| PhdFibTec48 | 1195 | 1017.32 | 2.55094 | 83.8741 |
| PhdGrFIns08 | 370 | 194737 | 2.54214 | 16 |
| PhdNtxNSp07 | 1842 | 1664.32 | 2.53163 | 136178 |
| PhdGrFIns03 | 426 | 249346 | 2.51199 | 20.2438 |
| PhdFibTec04 | 813 | 635357 | 2.51143 | 51.5714 |
| PhdEnzHyd05 | 215 | 61.7821 | 2.50401 | 5 |
| PhdEnzSeP05 | 1023 | 845318 | 2.47355 | 67579 |
| PhdNtxNSp26 | 301 | 130457 | 2.46571 | 10.3963 |
| PhdGrFCys13 | 5018 | 4840.32 | 2.43934 | 381608 |
| PhdGrFPDG02 | 2214 | 2036.32 | 2.40552 | 158316 |
| PhdOthUnd02 | 643 | 465402 | 2.32685 | 35 |
| PhdEnzOxR05 | 1934 | 1756.32 | 2.29018 | 130 |
| PhdEnzHyd08 | 3219 | 3041.32 | 2.28389 | 224496 |
| PhdEnzPA202 | 884 | 706349 | 2.27779 | 52 |
| PhdFibTec67 | 1014 | 836318 | 2.25929 | 61.0681 |
| PhdEnzKin02 | 8321 | 8143.32 | 2.25105 | 592457 |
| PhdNtxCav08 | 2957 | 2779.32 | 2.24824 | 201954 |
| PhdEnzHya07 | 786 | 608359 | 2.24049 | 44.0529 |
| PhdPInSeP02 | 565 | 387492 | 2.23352 | 27972 |
| PhdEnzSeP66 | 1614 | 1436.32 | 2.21879 | 103 |
| PhdEnzCho39 | 6439 | 6261.32 | 2.19074 | 443331 |
| PhdEnzMtP25 | 1228 | 1050.32 | 2.18879 | 74.3014 |

| | | | | |
|---|---|---|---|---|
| PhdEnzCho09 | 267 | 101178 | 2.17293 | 7.10563 |
| PhdFibTec12 | 2493 | 2315.32 | 2.16488 | 162 |
| PhdEnzCho46 | 284 | 115553 | 2.14209 | 8 |
| PhdNtxCav09 | 496 | 318.7 | 2.13584 | 22 |
| PhdPInSeP64 | 9773 | 9595.32 | 2.13142 | 660998 |
| PhdEnzSeP79 | 4178 | 4000.32 | 2.11557 | 273523 |
| PhdNtxCav45 | 5100 | 4922.32 | 2.11404 | 336321 |
| PhdEnzSeP20 | 1093 | 915318 | 2.11403 | 62.5396 |
| PhdNtxNSp43 | 1102 | 924318 | 2.10731 | 62.9537 |
| PhdNtxCav28 | 269 | 102842 | 2.10613 | 7.00047 |
| PhdEnzSeP08 | 1471 | 1293.32 | 2.0832 | 87078 |
| PhdNtxNSp53 | 3256 | 3078.32 | 2.05526 | 204.48 |
| PhdNtxCav29 | 372 | 196661 | 2.04528 | 13 |
| PhdEnzSeP73 | 3116 | 2938.32 | 2.04476 | 194184 |
| PhdEnzSeP80 | 4137 | 3959.32 | 2.03553 | 260477 |
| PhdFibTec77 | 309 | 137624 | 2.02338 | 9 |
| PhdFibTec06 | 536 | 358565 | 2.01982 | 23.4073 |
| PhdFibTec44 | 1771 | 1593.32 | 2.01843 | 103941 |
| PhdEnzCho44 | 238 | 78226 | 1.97764 | 5 |
| PhdOthUnd14 | 217 | 63.1479 | 1.95988 | 4 |
| PhdFibTec60 | 728 | 550373 | 1.93806 | 34.4743 |
| PhdEnzHyd02 | 2062 | 1884.32 | 1.93757 | 118 |
| PhdCRI03 | 1441 | 1263.32 | 1.91034 | 78 |
| PhdEnzSeP03 | 1201 | 1023.32 | 1.90484 | 63 |
| PhdNtxCav40 | 288 | 119004 | 1.87886 | 7.22653 |
| PhdPInSeP65 | 4406 | 4228.32 | 1.85132 | 253 |
| PhdGrFCys10 | 5147 | 4969.32 | 1.85004 | 297132 |

| | | | | |
|---|---|---|---|---|
| PhdGrFCys04 | 266 | 100348 | 1.85001 | 6 |
| PhdNtxNSp17 | 628 | 450408 | 1.84205 | 26815 |
| PhdFibTec74 | 342 | 168052 | 1.84113 | 10 |
| PhdGrFCys05 | 4393 | 4215.32 | 1.82552 | 248707 |
| PhdPInSeP59 | 1906 | 1728.32 | 1.79718 | 100389 |
| PhdPInSeP01 | 679 | 501386 | 1.79132 | 29028 |
| PhdPInSeP55 | 201 | 52.5167 | 1.76747 | 3 |
| PhdEnzPho05 | 1765 | 1587.32 | 1.75431 | 90 |
| PhdCRI22 | 202 | 53.1591 | 1.74611 | 3 |
| PhdPInSeP11 | 3315 | 3137.32 | 1.74532 | 176972 |
| PhdEnzPho04 | 2186 | 2008.32 | 1.7409 | 113 |
| PhdFibTec14 | 390 | 214087 | 1.73428 | 12 |
| PhdEnzMtP17 | 499 | 321684 | 1.7313 | 18 |
| PhdNtxAtx02 | 537 | 359562 | 1.72102 | 20 |
| PhdEnzSeP01 | 1419 | 1241.32 | 1.71988 | 69.0003 |
| PhdCRI02 | 1758 | 1580.32 | 1.7151 | 87.6 |
| PhdFibTec76 | 430 | 253288 | 1.71018 | 14 |
| PhdFibTec55 | 496 | 318.7 | 1.70922 | 17.6056 |
| PhdTCT16 | 765 | 587365 | 1.68566 | 32 |
| PhdEnzKin05 | 233 | 74.5056 | 1.66112 | 4 |
| PhdFibTec27 | 1056 | 878318 | 1.59378 | 45.2429 |
| PhdGrFCys07 | 5635 | 5457.32 | 1.57518 | 277832 |
| PhdEnzKin04 | 211 | 59.0704 | 1.57138 | 3 |
| PhdEnzSeP64 | 1088 | 910318 | 1.56348 | 46 |
| PhdEnzSeP60 | 266 | 100348 | 1.54167 | 5 |
| PhdPInPep01 | 519 | 341606 | 1.53976 | 17 |
| PhdNtxCav33 | 2329 | 2151.32 | 1.53805 | 106941 |

| | | | | |
|---|---|---|---|---|
| PhdFibTec62 | 241 | 80.4898 | 1.53762 | 4 |
| PhdPInSeP61 | 10856 | 10678.3 | 1.51972 | 524489 |
| PhdFibTec63 | 639 | 461403 | 1.51569 | 22.6027 |
| PhdEnzTra01 | 861 | 683351 | 1.49417 | 33 |
| PhdFibTec75 | 271 | 104509 | 1.48029 | 5 |
| PhdNtxNSp14 | 808 | 630358 | 1.47972 | 30.1466 |
| PhdNtxCav10 | 1567 | 1389.32 | 1.47869 | 66397 |
| PhdNtxNSp30 | 890 | 712349 | 1.47673 | 33.9989 |
| PhdGrFCys02 | 272 | 105342 | 1.46858 | 5 |
| PhdFibTec37 | 1081 | 903318 | 1.45143 | 42.3748 |
| PhdNtxNSp75 | 322 | 149.44 | 1.44945 | 7.0007 |
| PhdFibTec61 | 987 | 809347 | 1.44132 | 37.7022 |
| PhdEnzSeP82 | 219 | 64.5114 | 1.43884 | 3 |
| PhdNtxAtx01 | 324 | 151275 | 1.43172 | 7 |
| PhdNtxNSp55 | 2395 | 2217.32 | 1.41564 | 101.45 |
| PhdGrFCys06 | 3808 | 3630.32 | 1.40627 | 165 |
| PhdEnzSeP40 | 636 | 458406 | 1.4049 | 20.8146 |
| PhdEnzMtP15 | 450 | 273051 | 1.39977 | 12.3529 |
| PhdPInSeP51 | 4664 | 4486.32 | 1.3974 | 202.62 |
| PhdEnzSeP74 | 1298 | 1120.32 | 1.39193 | 50.4 |
| PhdNtxNSp06 | 1050 | 872318 | 1.38886 | 39.1566 |
| PhdEnzSeP53 | 1055 | 877318 | 1.37542 | 39 |
| PhdTCT17 | 426 | 249346 | 1.36496 | 11 |
| PhdNtxCav30 | 332 | 158687 | 1.3649 | 7.00021 |
| PhdEnzCho05 | 2026 | 1848.32 | 1.35961 | 81.2198 |
| PhdEnzCho43 | 451 | 274041 | 1.35486 | 12 |
| PhdFibTec42 | 1092 | 914318 | 1.34548 | 39.7598 |

| | | | | |
|---|---|---|---|---|
| PhdFibTec68 | 1039 | 861318 | 1.33554 | 37.1785 |
| PhdNtxNSp49 | 285 | 116412 | 1.32921 | 5.00109 |
| PhdGrFPDG03 | 2235 | 2057.32 | 1.30366 | 86.6838 |
| PhdEnzSeP57 | 391 | 215.06 | 1.29483 | 9 |
| PhdEnzTra02 | 1015 | 837318 | 1.29332 | 35 |
| PhdFibTec73 | 440 | 263162 | 1.2933 | 11 |
| PhdGrFPDG05 | 1997 | 1819.32 | 1.29251 | 76 |
| PhdEnzPho02 | 489 | 311.74 | 1.29027 | 13 |
| PhdEnzPA201 | 468 | 290886 | 1.2764 | 12 |
| PhdGrFCys03 | 291 | 121618 | 1.27204 | 5 |
| PhdEnzCho42 | 231 | 73.0437 | 1.27077 | 3 |
| PhdEnzSeP55 | 231 | 73.0437 | 1.27077 | 3 |
| PhdEnzSeP06 | 871 | 693.35 | 1.26828 | 28421 |
| PhdEnzMtP12 | 422 | 245408 | 1.26078 | 10 |
| PhdEnzGlS01 | 8407 | 8229.32 | 1.2559 | 334033 |
| PhdEnzMtP03 | 264 | 98.6992 | 1.25394 | 4 |
| PhdEnzMtP24 | 4329 | 4151.32 | 1.25124 | 167879 |
| PhdFibTec18 | 796 | 618358 | 1.25092 | 25 |
| PhdGrFIns09 | 233 | 74.5056 | 1.24584 | 3 |
| PhdPInSeP48 | 322 | 149.44 | 1.24227 | 6 |
| PhdEnzMtP13 | 234 | 75.2428 | 1.23363 | 3 |
| PhdEnzCho01 | 266 | 100348 | 1.23334 | 4 |
| PhdEnzCho07 | 2150 | 1972.32 | 1.2248 | 78.0752 |
| PhdFibTec78 | 506 | 328.65 | 1.22388 | 13 |
| PhdNtxNSp18 | 999 | 821318 | 1.20257 | 31.9223 |
| PhdGrFCys15 | 299 | 128676 | 1.20226 | 5 |
| PhdPInSeP21 | 616 | 438423 | 1.19973 | 17 |

| | | | | |
|---|---|---|---|---|
| PhdEnzCes03 | 3192 | 3014.32 | 1.18882 | 115818 |
| PhdEnzSeP87 | 986 | 808347 | 1.18657 | 31 |
| PhdGrFCys28 | 358 | 183231 | 1.18203 | 7 |
| PhdEnzSeP65 | 1700 | 1522.32 | 1.17815 | 57.9665 |
| PhdFibTec16 | 333 | 159615 | 1.16307 | 6 |
| PhdEnzCes01 | 360 | 185144 | 1.14594 | 6.85714 |
| PhdCRI01 | 1758 | 1580.32 | 1.1434 | 58.4 |
| PhdGrFCys29 | 307 | 135828 | 1.13896 | 5 |
| PhdPInSeP68 | 205 | 55.0976 | 1.12312 | 2 |
| PhdFibTec56 | 1040 | 862318 | 1.11325 | 31.0265 |
| PhdEnzOxR03 | 457 | 279978 | 1.10511 | 10 |
| PhdNtxNSp51 | 1087 | 909318 | 1.10375 | 32.4384 |
| PhdEnzMtP05 | 246 | 84.3346 | 1.10064 | 3 |
| PhdNtxAtx06 | 807 | 629358 | 1.09806 | 22.3355 |
| PhdGrFCys27 | 283 | 114691 | 1.0791 | 4 |
| PhdEnzHya04 | 826 | 648354 | 1.06954 | 22412 |
| PhdEnzCho02 | 350 | 175614 | 1.05711 | 6 |
| PhdEnzCho03 | 350 | 175614 | 1.05711 | 6 |
| PhdGrFCys25 | 350 | 175614 | 1.05711 | 6 |
| PhdEnzMtP35 | 286 | 117273 | 1.05534 | 4 |
| PhdEnzSeP61 | 251 | 88.2481 | 1.05183 | 3 |
| PhdGrFPDG04 | 1443 | 1265.32 | 1.05147 | 43 |
| PhdEnzSeP71 | 650 | 472.4 | 1.04795 | 16 |
| PhdFibTec21 | 778 | 600.36 | 1.0449 | 20.2749 |
| PhdEnzMtP01 | 507 | 329644 | 1.03247 | 11 |
| PhdEnzPho01 | 289 | 119874 | 1.03243 | 4 |
| PhdEnzMtP27 | 2846 | 2668.32 | 1.03151 | 88.9573 |

| | | | | |
|---|---|---|---|---|
| PhdEnzMtP10 | 1793 | 1615.32 | 1.03019 | 53.7829 |
| PhdFibTec30 | 834 | 656354 | 1.02705 | 21.7871 |
| PhdNtxCav27 | 213 | 60.4173 | 1.02423 | 2 |
| PhdPInSeP50 | 4653 | 4475.32 | 1.01893 | 147.38 |
| PhdPInSeP16 | 3393 | 3215.32 | 1.01658 | 105642 |
| PhdPInCyP03 | 515 | 337617 | 1.00808 | 11 |
| PhdEnzPho03 | 329 | 155.9 | 0.992321 | 5 |
| PhdGrFCys24 | 295 | 125131 | 0.989067 | 4 |
| PhdEnzCes09 | 523 | 345593 | 0.984819 | 11 |
| PhdCRI31 | 396 | 219927 | 0.984801 | 7 |
| PhdGrFCys01 | 259 | 94.6377 | 0.980812 | 3 |
| PhdEnzLip01 | 259 | 94.6377 | 0.980812 | 3 |
| PhdGrFCys30 | 217 | 63.1479 | 0.979941 | 2 |
| PhdEnzMtP16 | 450 | 273051 | 0.979836 | 8.64706 |
| PhdCRI26 | 685 | 507381 | 0.975696 | 16 |
| PhdPInSeP67 | 432 | 255264 | 0.969683 | 8 |
| PhdPInSeP52 | 1011 | 833318 | 0.965365 | 26 |
| PhdEnzOxR04 | 262 | 97.0635 | 0.9563 | 3 |
| PhdCRI13 | 3441 | 3263.32 | 0.950676 | 100268 |
| PhdEnzCho40 | 220 | 65.1995 | 0.949105 | 2 |
| PhdEnzMtP32 | 1405 | 1227.32 | 0.94681 | 37557 |
| PhdEnzSeP85 | 606 | 428435 | 0.938832 | 13 |
| PhdPInPep07 | 1138 | 960318 | 0.934355 | 29 |
| PhdPInSeP29 | 476 | 298827 | 0.931861 | 9 |
| PhdPInSeP32 | 2222 | 2044.32 | 0.929484 | 61.4132 |
| PhdEnzMtP20 | 1942 | 1764.32 | 0.929454 | 53 |
| PhdGrFCys22 | 222 | 66.5942 | 0.929229 | 2 |

| | | | | |
|---|---|---|---|---|
| PhdGrFCys23 | 222 | 66.5942 | 0.929229 | 2 |
| PhdCRI35 | 679 | 501386 | 0.925653 | 15 |
| PhdEnzKin03 | 266 | 100348 | 0.925003 | 3 |
| PhdCRI25 | 306 | 134.93 | 0.917232 | 4 |
| PhdEnzSeP56 | 343 | 168993 | 0.915439 | 5 |
| PhdEnzMtP21 | 856 | 678352 | 0.912228 | 20 |
| PhdPInSeP05 | 225 | 68.7118 | 0.90059 | 2 |
| PhdGrFCys26 | 488 | 310746 | 0.896119 | 9 |
| PhdEnzMtP28 | 1310 | 1132.32 | 0.894036 | 32.7186 |
| PhdEnzHya05 | 2563 | 2385.32 | 0.893399 | 68.8752 |
| PhdOthUnd09 | 1228 | 1050.32 | 0.884134 | 30013 |
| PhdGrFCys18 | 386 | 210208 | 0.883144 | 6 |
| PhdEnzOxR02 | 350 | 175614 | 0.880926 | 5 |
| PhdPInSeP31 | 712 | 534375 | 0.878306 | 15.1692 |
| PhdNtxNSp67 | 441 | 264149 | 0.866859 | 7.40064 |
| PhdEnzSeP58 | 354 | 179414 | 0.862268 | 5 |
| PhdEnzCes02 | 360 | 185144 | 0.859458 | 5.14286 |
| PhdEnzCxp04 | 276 | 108709 | 0.853853 | 3 |
| PhdEnzMtP07 | 687 | 509.38 | 0.850384 | 14 |
| PhdOthUnd15 | 318 | 145783 | 0.848947 | 4 |
| PhdEnzPA204 | 849 | 671353 | 0.844099 | 18.3154 |
| PhdGrFCys17 | 320 | 147605 | 0.838469 | 4 |
| PhdEnzSeP04 | 995 | 817347 | 0.832809 | 22 |
| PhdFibTec57 | 1042 | 864318 | 0.831123 | 23.2172 |
| PhdNtxNSp45 | 2410 | 2232.32 | 0.83104 | 59.9583 |
| PhdEnzSeP70 | 705 | 527377 | 0.821365 | 14 |
| PhdTCT06 | 2147 | 1969.32 | 0.821251 | 52.2712 |

| | | | | |
|---|---|---|---|---|
| PhdCRI29 | 596 | 418444 | 0.813363 | 11 |
| PhdEnzLig01 | 325 | 152196 | 0.813176 | 4 |
| PhdEnzSeP54 | 327 | 154046 | 0.803414 | 4 |
| PhdEnzSeP18 | 1018 | 840318 | 0.800974 | 21.7537 |
| PhdEnzMtP06 | 369 | 193777 | 0.798355 | 5 |
| PhdPInSeP53 | 728 | 550373 | 0.787046 | 14 |
| PhdFibTec34 | 896 | 718349 | 0.786317 | 18.2559 |
| PhdGrFCys16 | 886 | 708349 | 0.786238 | 18 |
| PhdPInSeP63 | 577 | 399473 | 0.774536 | 10 |
| PhdEnzMtP34 | 417 | 240498 | 0.771914 | 6 |
| PhdEnzCho16 | 6870 | 6692.32 | 0.765936 | 165669 |
| PhdEnzSeP59 | 828 | 650354 | 0.7612 | 16 |
| PhdEnzSeP84 | 933 | 755347 | 0.753308 | 18.3904 |
| PhdEnzCho41 | 671 | 493388 | 0.752526 | 12 |
| PhdPInSeP24 | 388 | 212144 | 0.729236 | 5 |
| PhdFibTec17 | 390 | 214087 | 0.722616 | 5 |
| PhdEnzMtP23 | 4333 | 4155.32 | 0.718808 | 96.5358 |
| PhdCRI11 | 3435 | 3257.32 | 0.71144 | 74.8979 |
| PhdFibTec32 | 690 | 512379 | 0.710995 | 11.7741 |
| PhdEnzSeP86 | 395 | 218955 | 0.706553 | 5 |
| PhdCRI12 | 3437 | 3259.32 | 0.697594 | 73.4853 |
| PhdEnzSeP50 | 2951 | 2773.32 | 0.688376 | 61.7016 |
| PhdGrFCys19 | 766 | 588365 | 0.683637 | 13 |
| PhdEnzCho04 | 2020 | 1842.32 | 0.681008 | 40.5497 |
| PhdPInSeP25 | 357 | 182276 | 0.678984 | 4 |
| PhdEnzHyd04 | 863 | 685351 | 0.677185 | 15 |
| PhdFibTec15 | 309 | 137624 | 0.674459 | 3 |

| | | | | |
|---|---|---|---|---|
| PhdEnzSeP89 | 545 | 367.54 | 0.673465 | 8 |
| PhdEnzSeP62 | 456 | 278988 | 0.665418 | 6 |
| PhdFibTec31 | 661 | 483393 | 0.663432 | 10365 |
| PhdPInSeP44 | 412 | 235583 | 0.65668 | 5 |
| PhdEnzCho33 | 6472 | 6294.32 | 0.631972 | 128563 |
| PhdGrFCys21 | 422 | 245408 | 0.630391 | 5 |
| PhdNtxNSp21 | 924 | 746349 | 0.627837 | 15.1447 |
| PhdEnzMtP04 | 267 | 101178 | 0.611607 | 2 |
| PhdGrFCys31 | 378 | 202455 | 0.611307 | 4 |
| PhdEnzCho45 | 436 | 259214 | 0.596816 | 5 |
| PhdCRI06 | 778 | 600.36 | 0.577232 | 11.2004 |
| PhdPInSeP34 | 2030 | 1852.32 | 0.575151 | 34.4325 |
| PhdFibTec70 | 960 | 782347 | 0.570137 | 14.4162 |
| PhdGrFCys20 | 503 | 325664 | 0.570047 | 6 |
| PhdFibTec69 | 676 | 498386 | 0.549842 | 8.85677 |
| PhdOthUnd10 | 207 | 56.4103 | 0.548492 | 1 |
| PhdPInPep03 | 551 | 373.52 | 0.537777 | 6.49212 |
| PhdTCT05 | 2069 | 1891.32 | 0.532265 | 32.5359 |
| PhdEnzMtP02 | 212 | 59741 | 0.517912 | 1 |
| PhdEnzMtP26 | 1425 | 1247.32 | 0.51356 | 20.7033 |
| PhdEnzSeP83 | 1005 | 827318 | 0.49616 | 13.2668 |
| PhdEnzTra04 | 924 | 746349 | 0.494787 | 11.9352 |
| PhdGrFCys08 | 4191 | 4013.32 | 0.479544 | 62.2019 |
| PhdNtxNSp74 | 445 | 268105 | 0.461619 | 4 |
| PhdPInSeP12 | 3347 | 3169.32 | 0.459386 | 47056 |
| PhdCRI30 | 585 | 407455 | 0.455617 | 6 |
| PhdOthvWC05 | 968 | 790347 | 0.422732 | 10.7983 |

| | | | | |
|---|---|---|---|---|
| PhdCRI10 | 3445 | 3267.32 | 0.409882 | 43.2834 |
| PhdFibTec11 | 1322 | 1144.32 | 0.405283 | 14.9891 |
| PhdNtxNSp47 | 2379 | 2201.32 | 0.375275 | 26.6995 |
| PhdFibTec59 | 826 | 648354 | 0.35702 | 7.48128 |
| PhdFibTec47 | 249 | 86.6737 | 0.356978 | 1 |
| PhdPInPep05 | 621 | 443418 | 0.35094 | 5.02941 |
| PhdEnzSeP75 | 1298 | 1120.32 | 0.347983 | 12.6 |
| PhdGrFCys11 | 4831 | 4653.32 | 0.344056 | 51.7443 |
| PhdFibTec10 | 2514 | 2336.32 | 0.342798 | 25.8846 |
| PhdEnzCho19 | 6857 | 6679.32 | 0.341069 | 73.6284 |
| PhdEnzMtP11 | 1802 | 1624.32 | 0.327957 | 17.2171 |
| PhdNtxNSp15 | 777 | 599.36 | 0.315067 | 6.10326 |
| PhdFibTec58 | 963 | 785347 | 0.307849 | 7.81396 |
| PhdGrFCys14 | 4960 | 4782.32 | 0.304092 | 47.0019 |
| PhdPInSeP14 | 3425 | 3247.32 | 0.273541 | 28709 |
| PhdEnzCho18 | 6459 | 6281.32 | 0.243148 | 49.3621 |
| PhdEnzSeP72 | 2851 | 2673.32 | 0.240927 | 20.8165 |
| PhdPInSeP18 | 3323 | 3145.32 | 0.175019 | 17.7919 |
| PhdNtxCav32 | 2018 | 1840.32 | 0.165901 | 9.86764 |
| PhdCRI19 | 3430 | 3252.32 | 0.146684 | 15.4187 |
| PhdPInSeP60 | 11003 | 10825.3 | 0.1296 | 45.3436 |
| PhdPInSeP19 | 3401 | 3223.32 | 0.108003 | 11.2515 |
| PhdNtxNSp01 | 5799 | 5621.32 | 0.0179803 | 3.26668 |
| PhdFibTec41 | 1947 | 1769.32 | 0.0033343 | 0.19067 |
| PhdEnzCho06 | 2156 | 1978.32 | 0.00242857 | 0.155281 |
| PhdCRI28 | 1053 | 875318 | 0.00148633 | 0.0420487 |
| PhdEnzHya06 | 2444 | 2266.32 | 0.000345671 | 0.0253195 |

| | | | | |
|---|---|---|---|---|
| PhdNtxCav46 | 1601 | 1423.32 | 5.84E-05 | 0.00268661 |
| PhdFibTec65 | 1119 | 941318 | 3.92E-07 | 1.19E-05 |
| PhdPInPep02 | 447 | 270084 | 0 | 0 |
| PhdNtxAtx04 | 718 | 540373 | 0 | 0 |
| PhdTCT03 | 1965 | 1787.32 | 0 | 0 |
| PhdTCT14 | 1835 | 1657.32 | 0 | 0 |
| PhdEnzSeP19 | 8018 | 7840.32 | 0 | 0 |
| PhdEnzSeP22 | 5894 | 5716.32 | 0 | 0 |
| PhdEnzHya03 | 2564 | 2386.32 | 0 | 0 |
| PhdFibTec46 | 538 | 360.56 | 0 | 0 |
| PhdFibTec64 | 675 | 497387 | 0 | 0 |
| PhdPInSeP10 | 3612 | 3434.32 | 0 | 0 |
| PhdPInSeP13 | 3542 | 3364.32 | 0 | 0 |
| PhdPInSeP15 | 3620 | 3442.32 | 0 | 0 |
| PhdPInSeP20 | 3534 | 3356.32 | 0 | 0 |
| PhdNtxCav15 | 286 | 117273 | 0 | 0 |
| PhdCRI21 | 218 | 63.8284 | 0 | 0 |
| PhdEnzSeP41 | 1152 | 974318 | 0 | 0 |
| PhdEnzCho14 | 6864 | 6686.32 | 0 | 0 |
| PhdEnzCho17 | 6858 | 6680.32 | 0 | 0 |
| PhdEnzCho20 | 6473 | 6295.32 | 0 | 0 |
| PhdEnzCho21 | 7607 | 7429.32 | 0 | 0 |
| PhdEnzCho22 | 6871 | 6693.32 | 0 | 0 |
| PhdEnzCho26 | 6466 | 6288.32 | 0 | 0 |
| PhdEnzCho29 | 6453 | 6275.32 | 0 | 0 |
| PhdEnzCho31 | 7620 | 7442.32 | 0 | 0 |
| PhdEnzCho35 | 6851 | 6673.32 | 0 | 0 |

| | | | | |
|---|---|---|---|---|
| PhdEnzCho38 | 6460 | 6282.32 | 0 | 0 |

**Table S2.** Comparison of transcriptomic analysis of *Phoneutria* spiders.

| | *Phoneutria depilata* | *Phoneutria nigriventer [20]* | | *Phoneutria perty [21]* |
|---|---|---|---|---|
| **Time of gland extraction after milking** | 8 days | 48 hours | 48 hours | 48 hours |
| **Library** | cDNA<br>Illumina TruSeq mRNA | CDNA<br>TruSeq RNA Sample Prep Kit protocol | cDNA<br>Super Script Plasmid System with Gateway Technology for cDNA Synthesis and Cloning Kit | cDNA |
| **Sequencing** | Illumina<br>HiSeq 2500 System | Illumina<br>HiSeq 1500 System | EST sequencing<br>ABI 3130 sequencer | EST sequencing<br>ABI 3130 sequencer |
| **Assembler** | Trinity software | Trinity software | TGICL | TGICL v2.1 |
| **Sequence annotation** | Blastx against UniProt Animal Toxin Annotation Project and NCBI | Blastx against UniProt-Swissprot DB, NCBI Transcriptome Shotgun Assembly protein DB (TSA), UniProt Animal Toxin Annotation Project and Animal Toxin DB (ATDB) | Blastx against UniProt-Swissprot DB | Blastx against UniProt-Swissprot DB |
| **E-value cutoff** | 1e-4 | 1e-5 | 1e-5 | 1e-5 |
| | | *ENZYMES* | | |
| **Serine proteases** | 89 | 29 | 10 | 2 |
| **Cholinesterases** | 46 | 10* | 1 | 0 |
| **Metalloproteinases** | 35 | 33 | 6 | 0 |
| **Hyaluronidases** | 11 | 3 | 0 | 0 |
| **Carboxylesterases** | 9 | 0 | 0 | 0 |
| **Hydrolases** | 8 | 0 | 0 | 0 |
| **Carboxypeptidases** | 6 | 0 | 0 | 0 |
| **Phospholipases A2** | 5 | 7** | 0 | 0 |
| **Kinases** | 5 | 0 | 0 | 0 |
| **Phosphatases** | 5 | 0 | 0 | 0 |
| **Oxidoreductases** | 5 | 0 | 0 | 0 |
| **Transferases** | 5 | 0 | 0 | 0 |
| **Glutamate synthase** | 4 | 0 | 0 | 0 |
| **Phosphodiesterases** | 2 | 0 | 0 | 0 |
| **Phospholipase B** | 1 | 0 | 0 | 0 |
| **Endonuclease** | 1 | 0 | 0 | 0 |
| **Ligase** | 1 | 32 | 2 | 1 |
| **Lipase** | 1 | 34 | 2 | 0 |
| **Lectins** | 0 | 0 | 0 | 0 |
| **Cathepsins** | 0 | 19 | 1 | 0 |
| **Superoxide dismutases** | 0 | 5 | 2 | 1 |
| **Aminopeptidases** | 0 | 20 | 0 | 0 |
| **Dopamine beta-hydroxylases** | 0 | 2 | 1 | 0 |
| **5′ Nucleotidases** | 0 | 8 | 0 | 0 |
| **Sphingomyelinases** | 0 | 5 | 0 | 0 |

| | | | | |
|---|---|---|---|---|
| **Chitinases** | 0 | 8 | 1 | 0 |
| **Angiotensin-converting enzymes** | 0 | 2 | 1 | 0 |
| **Catalases** | 0 | 1 | 0 | 0 |
| **Gamma-glutamyl transpeptidases** | 0 | 2 | 0 | 0 |
| | | *NEUROTOXINS* | | |
| **Calcium-channel toxins** | 49 | 20 | | 26 |
| **Sodium-channel toxin** | 26 | 13 | | 11 |
| **Potassium-channel toxin** | 0 | 1 | | 0 |
| **Atracotoxin** | 6 | 0 | | 0 |
| **Non specific neurotoxins** | 75 | 64 | | 26 |
| | | *FIBRINOGEN LIKE PEPTIDES* | | |
| **Techyletines** | 78 | 0 | 0 | 0 |
| | | *PROTEASES INHIBITORS* | | |
| **Serine proteases inhibitors** | 71 | 11 | 0 | 3 |
| **Peptidase inhibitors** | 8 | 48 | 5 | 2 |
| **Cysteine protease inhibitors** | 3 | 4 | 1 | 0 |
| **Metalloproteinase inhibitors** | 1 | 10 | 3 | 1 |
| | | *CYSTEIN RICH SECRETORY PEPTIDES – CRISP* | | |
| **CRISP** | 36 | 22 | 4 | 3 |
| | | *GORWTH FACTORS* | | |
| **Cysteine rich growth factor** | 31 | 0 | 0 | 0 |
| **Insulin-like growth factor binding protein** | 10 | 12 | 0 | 0 |
| **Platelet derived growth factor** | 5 | 0 | 0 | 0 |
| **Nerve related growth factor** | 2 | 0 | 0 | 0 |
| | | *TRANSLATIONALLY CONTROLLED TUMOR PROTEINS – TCTP* | | |
| **TCTP** | 17 | 3 | 0 | 0 |
| | | *HOST DEFENSE PEPTIDES* | | |
| **Defensins** | 2 | 0 | 0 | 3 |
| **Antimicrobial peptides** | 1 | 1*** | 0 | 0 |
| | | *VENOM COMPONENT* | | |
| **von Willebrand factor type C** | 6 | 0 | 0 | 0 |
| **DH31 hormone type** | 1 | 0 | 0 | 0 |
| **Lectins** | 0 | 20 | 1 | 0 |
| **Glycine-rich proteins** | 0 | 0 | 0 | 8 |
| **Undefined** | 15 | 0 | 0 | 36 |

* Enzymes annotated as Acetylcholinesterases.

** Enzymes annotated as Phospholipases.

*** Antimicrobial peptides annotated as Warprins
