# Supplementary material for: Transcriptomic Analysis of the Venom Gland and Enzymatic Characterization of the Venom of Phoneutria depilata (Ctenidae) from Colombia
Source: Toxins (Basel). 2022 Apr 21;14(5):295. doi: 10.3390/toxins14050295 (PMC9144723; doi:10.3390/toxins14050295)
Supplement: Supplementary file 1 [file toxins-14-00295-s001.zip › Supplementary Materials.pdf]

Supplementary Materials: Transcriptomic Analysis of the Venom Gland and Enzymatic Characterization of the Venom of *Phoneutria depilata* (Ctenidae) from Colombia

Julieta Vásquez-Escobar, Teresa Romero-Gutiérrez, José Alejandro Morales, Herlinda C. Clement, Gerardo A. Corzo, Dora M. Benjumea and Ligia Luz Corrales-García

Distribution of transcripts that putatively code for venom components of *P. depilata*.

ENZYMES							
Serine proteases							
Transcript ID	Uniprot ID reference protein	e-value	Identity (%)	PFAM domain	MW (Da)	Amino acid sequence	Nucleotide sequence

PhdEnzSeP01	Q76B45	2,86E-16	37,879	PF00089	17800.43	MINGEFYMSHDLALAKLNAPVLMNPHTQVVCLPELNEELRSGEKCFATGWGMTRGSKSDHVLKQAEHPIQNGTKCNNIFQNFEKETMICAGRLEPLHGLCHGDSGGPLVCKKDGKWHVYGVASFVTDSNFVEGLCGIANQPTVFNKISPKIQWIKSIISGNS*	GAAAATTTCTAACATCTGAGTGCACAGGATAAAGTATAACAGAAACTAAACTACTGCTGAACTGAAAACATAATTTTTCAAAAGTTTAAGGAATATTTAATATTAAGATAATAAACTACTACAGACTAAGGAATACAGAGCAGTTGTAAGTTTTAACTGACACTCAGTTACATATGTAAACTATAAATTCTGAATACACAAAATAATTTTGATCTAGAATTTATCAAAACATGCAAAAGTAGAACTAACTATTTCCTGATATTATAGATTTGATCCATTGAATTTTGGGAGATATTTTATTGAAAACAGTTGGCTGATTTGCTATCCCACAGAGACCTTCAACAAAATTACTATCAGTTACAAAACTTGCTACACCATATACATGCCACTTTCCATCTTTCTTGCAGACAAGAGGGCCTCCACTATCTCCATGACATAAACCATGGAGAGGTTCTAAACGTCCAGCACAAATCATAGTTTCCTTTTCAAAGTTTTGAAAGATATTATTGCATTTAGTTCCATTTTGAATAGGATGTTCAGCTTGTTTTAAAACATGATCAGATTTAGACCCTCTTGTCATACCCCAACCAGTTGCAAAACATTTTTCACCACTTCTTAATTCTTCATTAAGTTCTGGCAAACAGACGACTTGTGTGTGTGGATTCATTAACACAGGTGCATTGAGTTTCGCAAGGGCTAAATCGTGAGACATATAGAATTCCCCATTTATCATAAATACGAGATAGCTGATCCATAACAGCAGGCTGATTACAGTCACTAATGAAACGCGAAACCACTTCTTTCAGAGCATTAAATTCTTCAGCAATAAGTTTTACTTTATCCTTATTCTTTTCAAAAATGCCACATTTTTTAGCAGAGTTGAAATAACAACCTTCACGTTTTGGGTTTTCTTCAATGTATTTGTTGCAATTCTCATCATCACAATCAAACAAATCATCGTTATCATGAGGTTTATCGTTCAGTAAGCACTCCAGTTTGAAACTGCTTAGCTTTTCTTTGAACGAATCTAACATGAATTTTATTTCTCGGGATCGCTGTATCAACACGGCTTGAGAATTAGAAATCTGCATTTCAACAACAGCCAAAATTCCTTGAATGATAGAGATAGGATACGAAAATTCAAAACTTTCCCAGTAACCATGGTTAAACCCACTCTGTAAAGTT

							GATTCTTCCCCTAAATATTTTCCATCTACATATCCCTCTTTTAA<br>TCGAGCTTCCTTCAATCTTTTCCAAGCGATTTCGTCAACTCCA<br>CACATATCACTTTCTTCATCAAACACATCTTCTACAAAATCTA<br>CTTCTCCAGTCATTGTAGCCATGTTTGTTGTCTATTTTCAGGA<br>AGAAGGAGAGGAGAGGAGGAAGAGGAATAAACAAAATTCT<br>TACTAAATTTATCGAATATTT

PhdEnzSeP<br>02	P84033	7,80E-63	43,265	PF00089	33600.12	MPTDARRSSFWTLC<br>TNVVSNSYLLLSILSA<br>LGCVANGRPRTVKK<br>CGLVDVPRARIIGGD<br>WVQHGDYPWMVT<br>VHEYYHGFDHVCG<br>GAILNDHWIITAAH<br>CIDYPKKPWKYEIYV<br>GLHRLSKKTRKQTK<br>KHKISRVIMHEDYDS<br>ETYLNDIALLRTKKP<br>IDFKASRGFVNGICL<br>PETDKDPAGWATVT<br>GWGHTLEAGDNSD<br>VLKAVKVPIVPRELC<br>NEAYDDDDFDDIVD<br>ENVFDTQICAGAAN<br>RDSCQNDSGGPLVQ<br>RNKTGVHTIIGIVSY<br>GAGCGNRDYPGIYT<br>KVAAYMDWLDEKM<br>KD*	GGAGTATACAGAGACCTTGAGTAGTGAGGCCGATAAAGGTG<br>CCCATTATCGTGCGTTATGCAAACGGCATTTCATCGTGAGTG<br>GAGCAGATGAAAGATTTTAAACAGAGTAGCGAATCGTTTCTT<br>CGCCATAAACTTGCTTAGCACACGCACACAAGGAATCGGAA<br>ACGATGCCGACGGACGCAAGGCGATCGTCATTTTGGACGTTA<br>TGCACAAACGTCGTCTCGAATTCTTATCTCCTTCTGTCGATTC<br>TATCTGCGTTAGGATGCGTGGCAAATGGACGGCCGAGGACT<br>GTGAAGAAATGTGGCCTCGTTGACGTTCCGCGGGCGAGGATT<br>ATCGGTGGTGATTGGGTTCAACATGGCGATTATCCATGGATG<br>GTCACTGTCCATGAGTATTATCATGGTTTTGATCACGTTTGCG<br>GAGGTGCCATCCTCAACGACCATTGGATCATAACAGCTGCTC<br>ACTGCATAGACTATCCTAAGAAACCATGGAAATACGAGATA<br>TACGTGGGACTTCATAGACTGTCGAAGAAAACTCGTAAGCA<br>AACTAAAAAACACAAGATATCTAGAGTGATTATGCACGAAG<br>ACTATGACTCGGAGACGTATCTCAACGATATCGCCTTGCTGC<br>GCACCAAAAAACCCATCGATTTCAAGGCTTCCAGGGGTTTCG<br>TAAACGGCATTTGTCTGCCAGAAACTGATAAAGACCCAGCT<br>GGATGGGCGACCGTTACTGGATGGGGTCACACTCTAGAAGC<br>TGGTGATAATTCCGATGTATTGAAAGCCGTGAAAGTTCCCAT<br>CGTTCCAAGAGAACTATGCAACGAAGCTTATGATGATGATG<br>ATTTCGATGATATTGTTGACGAGAATGTTTTTGATACACAAAT<br>ATGTGCTGGTGCAGCCAACAGAGACTCTTGTCAGAATGATTC<br>TGGGGGTCCCCTGGTTCAGAGGAATAAAACTGGCGTGCATA<br>CCATAATAGGAATTGTATCATATGGAGCAGGTTGCGGCAACC<br>GAGACTACCCTGGCATCTACACAAAAGTAGCTGCTTATATGG<br>ACTGGTTGGATGAGAAGATGAAAGACTGATGCATATTAGTG<br>GAGTATGAATGGACTCGGCGAATCTTATATCAGTTTGTAAAT

							TTGTGGCAGTATGTTCCTTTATCGACTCGGAAAATTTTAATAAAGGAATATAAATACTTCAAAAAAAA

PhdEnzSeP 03	Q9TXD8	4,00E-42	37,500	PF00089	28267.81	MEGFWSWNESTCG ASRGSEYQRNDDDD RKGYIVGGRDAIDG EFPWQISLQRHFEKD NGIYHICGGTIIAKD WVLTAAHCIRLSKIS SYRVVAGTSDLSEEN KIRLGSKSAYSIHKV VDAFIHEEFSAHNL QNDIALVQVEPPFEL GRFDGKITTACLPHP GHEPYGYATVSGWG ALKEGGRVIPTKLQ AVSVPLISDEKCREA YGEAIVPSMLCAGY EEGMKDSCQGDSGG PLVQKTSSGKSAIVG VVSWGVGCARPKKP	TGCAATTTCGGGAACTCCAGAGGCTATCAATCTTGCCCAGTA CCTCATCAATACCAGATTTGGCTGTTATTCTGCTCCGAATATA AGAATAACAGCAATGTTCATCCTGAAGCAAACTGCCAGAGA TGGTGTTCTCAATGGCCGACTGACTACGTGCAATTTTACAGA AGTAGAACTGATCTTTTAAGTAGACGCTAACAAGGTAATTAA ACAAAAAGAAGACAGCGAAAAGGAAAGAAATCTAATGAAA AATGAAACGAAAAATTGGAAGCGCTGGTAATCACCAGAGAC TGAACGCTATTTTCGGTCGATAGTAAAAGTTTAAAATTCTTGC ATTTCCTTGATTAGAATGATCAGTAAGCATCTCGTAAATGGG CTCTTAGTGGATGCTGATAGAATAATATCTTAAGAGGATCTTT CATGGAGGGATTTTGGTCCTGGAACGAATCAACTTGTGGTGC ATCAAGAGGATCTGAATATCAACGGAATGATGACGATGACA GAAAAGGTTACATTGTTGGGGGCAGAGATGCAATTGATGGT GAATTCCCTTGGCAGATTTCTCTTCAAAGGCATTTTGAAAAA GACAATGGAATTTATCACATATGTGGCGGAACCATTATTGCC AAAGACTGGGTTCTAACTGCAGCACATTGTATTCGTCTCTCA AAAATATCCAGTTACAGAGTGGTTGCGGGCACGAGTGATCTT TCTGAAGAAAATAAAATTAGATTAGGCAGTAAATCAGCGTA TTCGATACATAAGGTCGTGGATGCCTTTATTCATGAAGAATT CTCAGCTCATAACCTTCAAAATGATATCGCATTAGTTCAAGT AGAGCCTCCATTTGAACTTGGACGCTTTGATGGAAAGATAAC AACTGCCTGTTTACCTCATCCAGGTCACGAACCCTATGGGTA TGCAACTGTTTCTGGCTGGGGAGCTCTCAAAGAAGGAGGCC GAGTTATTCCAACTAAACTGCAAGCTGTGTCAGTCCCGCTCA TTAGTGACGAGAAATGCAGAGAAGCTTATGGTGAAGCCATT GTTCCATCAATGTTATGTGCCGGCTATGAAGAAGGCATGAAA GATTCTTGCCAAGGAGATTCCGGCGGTCCTCTCGTACAGAAA

							ACTAGTTCCGGAAAGAGTGCTATAGTTGGAGTCGTCTCCTGG GGTGTCGGCTGTGCAAGACCGAAAAAACCTG

PhdEnzSeP 04	Q9TXD8	6,71E-35	29,536	PF00089	36385,13	LFLTSMTWIVKCQLFSTTIDAIRSRSQCDCMEYWQCVGSGGKPYSYCSYSSKVCCFIDPNAVSVGLLPQPQKSGSCGTKGPNNDRDGVTEPGEWAWHAAVLEKPQDLYVCGASLLDEFWILTAAHCVDEYRSPTSLKVRLGEYDVSSTNEPHRYEEYDVSRIVVYPSFNNRTLLHDIALLRLERPAKRKAHVNIVCMPPEGTTNDDLLLSPRCYITGWGKTSENSDHSVVLKEVNVPLWRNDDCERSLKFHFGPNYKLPATTLCAGAEGRDACDGDGGGPLVCEKTGRWYQVGIVSFGIGCGQPNTPGIYTRVESYLNWVHRVVSTA*	TCTCCACTTTTTCATGCAGTTGATACAACCCTATGAACCCAGTTTAAATAGCTCTCAACCCTAGTATAGATTCCTGGAGTATTTGGCTGACCACAACCTATACCAAAAGAGACGATACCAACTTGATACCACCGACCGGTCTTCTCACACACGAGAGGGCCCCCTCCATCACCATCACATGCATCCCTTCCTTCTGCTCCTGCGCATAGTGTAGTTGCAGGAAGCTTGTAATTTGGTCCGAAATGAAATTTCAGAGATCGCTCACAGTCATCATTGCGCCACAGGGGGACGTTGACCTCTTTCAAGACAACAGAATGATCGCTGTTCTCTGATGTTTTACCCCATCCTGTTATGTAGCACCTAGGAGACAGCAAGAGATCATCGTTGGTGGTGCCCTCTGGTGGCATACAAACGATATTCACATGAGCTTTTCTCTTGGCCGGTCGTTCTAACCTTAGCAGAGCAATATCGTGCAGTAGAGTGCGGTTGTTGAAAGAAGGATACACCACAATGCGTGAGACATCGTATTCCTCATATCGATGTGGCTCATTGGTACTTGAAACATCATATTCCCCCAGACGAACCTTCAGTGATGTTGGAGAGCGATATTCATCGACGCAGTGTGCAGCGGTTAAAATCCAGAACTCGTCTAACAAAGATGCACCACATACATAAAGGTCCTGGGGTTTTTCAAGAACTGCAGCGTGCCAAGCCCATTCACCAGGCTCTGTGACGCCGTCCCTGTCGTTGTTGGGTCCCTTGGTTCCACAACTTCCAGACTTCTGCGGCTGCGGTAGAAGTCCTACCGAAACTGCATTTGGGTCAATGAAGCAGCATACTTTGCTGCTGTACGAACAATATGAATAAGGTTTTCCACCAGAACCTACACATTGCCAATATTCCATACAGTCGCACTGGCTGCGAGAACGTATGGCATCAATAGTCGTCGAAAATAATTGACATTTAACAATCCATGTCATAGAAGTCAAGAAAAG

PhdEnzSeP 05	Q9TXD9	4,24E-09	26,573	PF00089	17786,45	ILFQTSPLSKPILCGSSTRPICLPMFPKMADIGNWITVAGWGTMNIQNKSSQLKEGRLKIVNKTECERLDGFQEAISSAICITGEGQKSVCFSDSGGSIFMEKHGRYFILGVASAQSSETCSLQASIVFTDVYYHMSWIKKVISEVAPESDDLTVYHLDYK*	TGAAAACTTCGTTACTTTGCATTAAGCTTTGTAGTGAAACTTTGTTTTTATTATTTTGTAGCGTAAATGAAAATTAGATGATTAAAAGAAAAGCCCCTGATATTTATTTCCGGTCTGAACATAAACAATGGGAATTTATAAAGTGAATCAGTGGGTTTTCGTGACTTTTGTATTTTTCTTATTGGTATCCGACGGATTGACAAAGAAGAGAAAGAGTAAAAGTCTGACAACTAAGAAAGCAACATGTAGTAAGTATTAGGGATCGAAGCACAAACACCATAATCTGTGGAGGAACCATCATCTCCTCCAAAGTTATTCTTACAGCTGCTAAATGTTTACATAAAGCAGATAACAAAGACTCCAGTTACTGCAAAGAGCGAGGAGAACTTCCCGGAGAGTGCTGGAAAAAACCGGAAGACATCAACATTGGACTTTTGACTAAATATGAGACTGTCTCAATTGTCAGTAAGATAGCCAAGCTAATAAAACATCCCCACTTCAACTCATTAGATATTGTTTCAGACATCGCCCTTGTCAAAACCGATTTTGTGTGGCAGTTCTACGAGACCGATTTGTTTACCTATGTTCCCAAAAATGGCTGATATTGGGAACTGGATAACTGTTGCCGGATGGGGAACAATGAACATTCAGAACAAATCATCACAATTAAAAGAAGGAAGACTAAAAATAGTTAACAAAACAGAATGCGAACGTCTAGATGGATTTCAGGAAGCAATCAGTTCTGCCATATGTATTACGGGAGAAGGACAGAAGTCTGTGTGCTTTAGTGACTCTGGAGGTTCAATTTTTATGGAAAAACATGGCAGATACTTCATACTAGGAGTCGCATCAGCCCAGTCTTCAGAAACTTGTTCATTACAAGCATCCATAGTTTTCACTGACGTTTACTATCACATGTCCTGGATAAAGAAAGTTATATCTGAAGTTGCTCCGGAAAGTGATGACTTAACAGTTTATCACTTAGATTATAAGTGACACTAAGGTGATTCTGCCATTCTCCTG

PhdEnzSeP 06	Q9TXD10	7,14E-12	26,573	PF00089	17786,45	ILFQTSPLSKPILCGSS<br>TRPICLPMFPKMADI<br>GNWITVAGWGTMN<br>IQNKSSQLKEGRLKI<br>VNKTECERLDGFQE<br>AISSAICITGEGQKSV<br>CFSDSGGSIFMEKHG<br>RYFILGVASAQSSET<br>CSLQASIVFTDVYYH<br>MSWIKKVISEVAPES<br>DDLTVYHLDYK*	GAGAAAGACTGCGGAAAATCTAAGGAAGAAGGTTATCGTTC<br>ACTTGTTATTGATGACGACTCTGATGTTAAACCTCTAGACAA<br>ATATCCGTGGATAGTAAGTATTAGGGATCGAAGCACAAACA<br>CCATAATCTGTGGAGGAACCATCATCTCCTCCAAAGTTATTC<br>TTACAGCTGCTAAATGTTTACATAAAGCAGATAACAAAGACT<br>CCAGTTACTGCAAAGAGCGAGGAGAACTTCCCGGAGAGTGC<br>TGGAAAAAAACCGGAAGACATCAACATTGGACTTTTGACTAA<br>ATATGAGACTGTCTCAATTGTCAGTAAGATAGCCAAGCTAAT<br>AAAACATCCCCACTTCAACTCATTAGATATTGTTTCAGACAT<br>CGCCCTTGTCAAAACCGATTTTGTGTGGCAGTTCTACGAGAC<br>CGATTTGTTTACCTATGTTCCCAAAAATGGCTGATATTGGGA<br>ACTGGATAACTGTTGCCGGATGGGGAACAATGAACATTCAG<br>AACAAATCATCACAATTAAAAGAAGGAAGACTAAAAATAGT<br>TAACAAAACAGAATGCGAACGTCTAGATGGATTTCAGGAAG<br>CAATCAGTTCTGCCATATGTATTACGGGAGAAGGACAGAAG<br>TCTGTGTGCTTTAGTGACTCTGGAGGTTCAATTTTTATGGAAA<br>AACATGGCAGATACTTCATACTAGGAGTCGCATCAGCCCAGT<br>CTTCAGAAACTTGTTCATTACAAGCATCCATAGTTTTCACTGA<br>CGTTTACTATCACATGTCCTGGATAAAGAAAGTTATATCTGA<br>AGTTGCTCCGGAAAGTGATGACTTAACAGTTTATCACTTAGA<br>TTATAAGTGACACTAAGGTGATTCTGCCATTCTCCTG

PhdEnzSeP 07	Q7M4I3	1,12E-43	39,113	PF00089	48019.64<br><br>**45177.19**	MRLKTINTLLCFFIY<br>VTVDLARTQ**AVRYQ**<br>**ASNLDASCSEDNSR**<br>**CVVITSCDSVLQKI**<br>**KRKISPRVCGWAS**<br>**DVPLVCCPEDAKEV**<br>**SPKELKKINGLSSKS**<br>**CGLRKISAALKEEKI**<br>**AYPPSLPGGGILKS**<br>**GIEPRSRRDETPAAP**<br>**VEATLLSFDLLDYP**<br>**MFAVNGETAKDPW**<br>**PWMVAIFNGNGQ**<br>**QQLCGATLIDERHV**<br>**ITAAHCFAGRSLDP**<br>**NLYRIQIGENDLRN**<br>**ENTDHEVQEIKLHE**<br>**NYQARYYYDDIAIM**<br>**RLKNPLTSFTAACL**<br>**PAEDDVHHGDNVT**<br>**VLGWGDLSFGGPR**<br>**TANLQEVIGIPIVRN**<br>**AKCDEKYRTLPGAP**<br>**FPRGITNTFLCAGLE**<br>**EGGKDACQGDSG**<br>**GPLLQQNFDGSWT**<br>**LVGVVSFGYRCGEP**<br>**GYPGVYTKVSAYL**	ATTGCAATAAAACCGATCAGGAGTTTAACGTTTAGCTCTTTT<br>ATTGTGGTGACTGTTTACCGGCAACACGTGCACTGGAATATT<br>CACAATGCGGCTAAAGACAATCAACACGCTACTTTGTTTCTT<br>TATTTACGTAACAGTGGACCTCGCCAGGACACAGGCAGTAC<br>GTTATCAAGCCTCCAACCTCGACGCTTCTTGTTCTGAAGACA<br>ATTCTCGCTGCGTGGTCATCACCTCTTGCGACAGCGTCCTCCA<br>AAAAATCAAAAGGAAAATAAGTCCTCGTGTCTGCGGTTGGG<br>CCTCAGATGTGCCCCTCGTCTGCTGCCCAGAGGATGCCAAGG<br>AAGTATCACCAAAAGAACTGAAGAAAATAAATGGACTGAGT<br>TCGAAAAGCTGTGGCTTAAGGAAGATTTCAGCTGCTCTCAAA<br>GAAGAAAAAATAGCATATCCACCATCGCTTCCCGGCGGGGG<br>TATCCTTAAATCAGGAATTGAACCAAGAAGCCGGAGGGATG<br>AAACTCCAGCTGCGCCGGTTGAGGCGACGCTTCTCTCATTTG<br>ATCTGCTAGATTATCCTATGTTTGCTGTTAATGGAGAAACGG<br>CAAAGGACCCTTGGCCATGGATGGTAGCAATTTTCAACGGG<br>AATGGACAGCAACAACTGTGTGGTGCAACACTTATCGATGA<br>GCGGCATGTCATTACGGCAGCTCATTGTTTCGCTGGCAGGAG<br>TTTGGATCCGAATCTGTATAGGATTCAAATAGGAGAAAATGA<br>CCTCAGAAATGAGAACACAGATCATGAAGTGCAAGAAATCA<br>AACTCCACGAAAATTATCAGGCCAGGTACTATTACGATGAC<br>ATCGCTATAATGCGGTTGAAAAATCCCTTGACCAGCTTCACA<br>GCGGCTTGTCTTCCAGCTGAAGACGATGTTCATCATGGTGAC<br>AATGTGACTGTTTTAGGATGGGGTGACTTATCCTTTGGTGGAC<br>CGAGAACAGCGAATCTACAGGAAGTGATTGGCATACCAATA<br>GTTAGGAATGCAAAATGTGATGAAAAATACCGCACCTTACC<br>AGGTGCGCCTTTCCCGAGAGGCATAACAAATACATTTCTCTG<br>CGCTGGCCTAGAAGAAGGTGGAAAAGATGCATGTCAGGGTG<br>ACTCTGGTGGTCCTCTGCTTCAGCAGAATTTTGACGGATCTTG

						**QWIEKFIGNQNEVVRKKQARSQNFEYIDDRPIWRSVTERVLFPR***	GACTCTGGTGGGAGTCGTTTCTTTCGGATACAGGTGCGGAGAACCGGGATATCCTGGAGTTTACACGAAAGTTTCTGCCTACTTGCAGTGGATTGAAAAGTTTATCGGTAACCAGAATGAGGTAGTAAGGAAGAAACAAGCGAGATCCCAGAACTTTGAATACATCGATGACAGGCCGATTTGGCGATCGGTAACGGAGAGAGTTTTATTTCCAAGATAAAATTGAAGTAATCTGCACATTTCATTCATGTTTTTCATCTTCATCAAATAAAAAGTTTTTTTTTTTAAAAAAA

PhdEnzSeP 08	Q7M4I3	2,93E-48	37,109	PF00089	47169.66<br><br>**44621.42**	MKISCKQILFLSILCL<br>LGWIVSG**QDYDDP**<br>**DDMDPRFHERERS**<br>**CNYRRQQGFCRRR**<br>**SDCARPTRHICRFG**<br>**FNPVVCCLDQPEVK**<br>**TTTTTKQPVTTRRS**<br>**DVKPESVKSLDLTF**<br>**PGCGLRAPRIQGST**<br>**RPGSNNRNSNNRQ**<br>**RGFLSRGRRSILDD**<br>**FSYSNATRERRAIT**<br>**QPVIVGGVTAVAN**<br>**SWPWMVAIFKETN**<br>**ARSPKRFLCGASLIS**<br>**RKYVMSAAHCFDA**<br>**ERGNIDASKFFVVV**<br>**GAHSTKDGVEYPV**<br>**QSILIHPDYKQRQY**<br>**YNDISLLRIRNEVQL**<br>**TQKVYPVCVPSDG**<br>**LRDKIKPNNNNVT**<br>**VTGWGDTSFGGVS**<br>**SKVLQELTIPIVPLK**<br>**ECNEAFSKLLQSTFP**<br>**NGITNLFICAGVEE**<br>**GGKDACQGDSGG**<br>**PLVSILKDNSWVQL**	CACGAATTTAAGACAGTCAACATGTCAATGTGAGCAGATTTC<br>GTGAGGTGATAGGTTTCGGTATTGAGGCAGTTTCTACTTATAT<br>ATGTTAACTTATATAACTTAAAAGTTTTTAGTTTCCCACTAGT<br>TTTTTAAACGATGCTCAGCATATCTCAACTGCTGATTATTCCT<br>GCCATATTACTTCTTCGCTTTTTAGTGGGCCTCTCGGTATCAA<br>ATTTTTCTGTGAGAGGTGTCAGCTGGTCTCAAGAAGCAACTA<br>AATGGCATCATACACCTGCAAGATTAAGTGATGCAGAATTA<br>CATACAGTATCTACAGTGACATCTGACTATGATGATTTTAAC<br>AGAACTTTAGATGCCATATTAATAACAAGAGTTCCTGGAACA<br>GAAGGACATGAAAAAGTCAGAAATTTTATTCTGAAAGAAAT<br>GAGATCGTTAGACTGGGATGTTGAAGAAGATGAGTTTACTGA<br>TCGAACACCTCATGGACGTAAATCATTTTCTAATATCATCAC<br>CACATTGAATCCTAATGCATGTAGATTTCTGACTATTGCATGT<br>CATTATGATTCTTTACATAATAGAGAATATACTTTCTTGGGTG<br>CTACAGACTCTGCAGTTCCTTGTGCAATGATGATTCATGCAG<br>CCAAGATGTTGGATGCACCTCTCAAGGAACAAAAAGATAAG<br>AACAATGAACTGACACTTCAGTTTATATTTTTTGATGGTGAA<br>GAAGCATTTCAAAGATGGAGTCGTACTGATTCTCTTTATGGG<br>TCAAGACATTTAGCTGCTAAATGGAATCGTATGACTCCTTTTC<br>CTAGCGGATTATATGATGGCAAGCATTGTAAATCTAAAGAAT<br>ATGCTTCATATTTGGATCGAATGGATGTCATGGTGTTACTGGA<br>TTTAATTGGAGCAGCCAGCCCCAAATTTTATAGTTATTTTCCA<br>GATACATATGGCTTGTATTCTAATATTGTTGACATTGAGAATC<br>GTCTCAACAGCTTACATCTAGTCGAATCCCATCCCCCTGAAG<br>GAAAAACAAATTACTTTGATAGTAGATCAACTTTGGCATTTG<br>TGGAAGATGATCACATCCCTTTTATGAAAAGAGGTGTACCAA<br>TTATTCACGTTATTCCTTCCCCTTTTCCACAAGTTTGGCACCG<br>GGAAAGTGACAATCTTCAAAATGTCCATCATCCTACTGTCGA

						**GIVSFGYGCAQPGYPGVYTRVSQYTKWLYDNTDLGKA***	CAATCTGAACAAAATTTTACGTGTATTTATAGCTCAGTATTTCCATTTAAATATGGATGTGTCGAGGAACTTTAAAATAATGAAATGATATATCTATTTTATATACAAATACTTTGAAACGATGTGCATTTTAAAAAATTTGGTGCAGTTTATTTTTAAGAATCCATGGTTTATTGAAATTTTCGAGCTATACTTTATTTTTGAATATCCCGTCTACCATTCTGCATTTAATTGGTCTGTGTATACGCAACAAAATTTAAGTCGTGTTTTGTAATATAAAATCCACTGTTTAGGTATGTATAAATGTTTTAGCTACATGTCCTATTCTGGATGTGTGTATTGTTCATAGTGATATAAATTTTTCATTGGAAACTTGGTGAAACCTCTAAAATGGTCATCTAAGAACATCTCTGGTACTTAAACAAAATTTTTTTAAAACTTTAACTATTCCTATTTCTTTTGAATTTGAATCTCGATTATTCTCTTTACAAATAGGAAAAAGAAATATTGCTACTAACTTTGAATCACTG

PhdEnzSeP 09	Q7M4I3	2,22E-27	32,990	PF00089	30759.96<br>**29025.76**	MFVAICILSCLQLIQ<br>**GNPLERLSEIDERPL**<br>**CPCGSNAESMDTR**<br>**VVGGHAVGRHYFP**<br>**YASGLLFRSNFGER**<br>**EIPISPFCGGTLITDR**<br>**HIVTAAHCLRNRTP**<br>**DQVSVDVGDYSLK**<br>**DAEDRQILNTRNLT**<br>**KFPEYIPKSFHTDIG**<br>**IVELEHPVRFHQGL**<br>**RTAYLPNPDLDLKP**<br>**GTTVSVYGWGRLS**<br>**YYGGHPDKLQSVD**<br>**LPVVDNDKCQTQF**<br>**VSTIEPMMICAGGE**<br>**EGKDACISLYVYTR**<br>**VILEAVWSFVSTTN**<br>**TSSVVLFPSEGAAPS**<br>**LTFLESIPRSAAS***	GGCTACGCTACATTTTCCGAAGGGAATATCTCGATGTGTCCC<br>ATTATACCAAGTTTCAACAGTGCATGTCGGAAACGCAAGTGC<br>CTCGAATAACCCGTGTTACTCCAAAATTAAAGGCAAACGCA<br>GAGCAATACAACACGTTCTTTATTGAAACAACAAAATCACA<br>TCATCACTCTCCATAAATGCAAGGTTTACAGGAAGCCGACTG<br>CGTCTGTTCCTTGATCCAATTTACGAAGCTGCTGACCTTGGTA<br>TAGACTCCAGGAACGTGAGGGAGGGCGCAGCGCCTTCCGAA<br>GGAAACAACACCACAGAGGACGTATTCGTTGTCGAGACGAA<br>CGACCAAACCGCTTCCAGAATCACCCTAGTATAAACATAGA<br>GAGAGATGCAGGCGTCTTTGCCTTCTTCCCCGCCAGCGCAGA<br>TCATCATGGGTTCGATCGTAGACACGAACTGGGTCTGGCATT<br>TGTCGTTGTCCACTACAGGGAGGTCCACAGACTGCAACTTAT<br>CCGGATGACCACCGTAGTAACTCAATCTTCCCCATCCATAGA<br>CGGAAACCGTCGTCCCTGGTTTAAGATCGAGGTCAGGATTCG<br>GGAGGTATGCCGTCCTCAGCCCTTGATGAAACCGTACAGGAT<br>GCTCTAGCTCCACAATTCCAATATCCGTGTGGAAGGATTTCG<br>GTATGTACTCAGGAAATTTGGTAAGATTGCGAGTATTCAGAA<br>TCTGCCTGTCCTCTGCATCCTTCAGGGAGTAATCTCCAACGTC<br>CACAGATACCTGATCGGGGGTGCGATTTCTTAAGCAATGAGC<br>AGCCGTCACAATATGGCGATCCGTGATCAACGTACCACCAC<br>AAAATGGCGAAATAGGAATCTCGCGCTCGCCAAAATTCGAA<br>CGGAACAGCAAACCGCTTGCGTAAGGGAAATAATGTCTACC<br>AACAGCATGTCCACCGACCACTCGAGTGTCCATGGATTCGGC<br>ATTGCTACCGCATGGACACAGAGGCCTTTCGTCTATCTCAGA<br>CAACCTCTCCAGAGGATTTCCCTGAATCAGCTGAAGACAGG<br>AGAGAATGCAAATCGCCACGAACATATTACAATATGTATAC<br>CTGAGCGAAGGTCGGAACTGTTTCGAATCACAAATACTATGC<br>AACTCACAGGTGGCACACGTCAGGGTTTATAATCAAAATCG

							ACCTCAATACTTCGCAACTACATCGACGCACATGGCAATCGCTGAAAAGCATTTAATCGTGAATACCGAAGCTAGACTGACGTAAGGGAA

PhdEnzSeP 10	Q7M4I3	3,84E-36	35,983	PF00089	32215.67<br>**30481.47**	MFVAICILSCLQLIQ<br>**GNPLERLSEIDERPL<br>CPCGSNAESMDTR<br>VVGGHAVGRHYFP<br>YASGLLFRSNFGER<br>EIPISPFCGGTLITDR<br>HIVTAAHCLRNRTP<br>DQVSVDVGDYSLK<br>DAEDRQILNTRNLT<br>KFPEYIPKSFHTDIG<br>IVELEHPVRFHQGL<br>RTAYLPNPDLDLKP<br>GTTVSVYGWGRLS<br>YYGGHPDKLQSVD<br>LPVVDNDKCQTQF<br>VSTIEPMMICAGGE<br>EGKDACIGDSGSG<br>LVVRLDNEYVLCG<br>VVSFGRRCALPHVP<br>GVYTKVSSFVNWIK<br>EQTQSASCKPCIYG<br>E***	GGCTACGCTACATTTTCCGAAGGGAATATCTCGATGTGTCCC<br>ATTATACCAAGTTTCAACAGTGCATGTCGGAAACGCAAGTGC<br>CTCGAATAACCCGTGTTACTCCAAAATTAAAGGCAAACGCA<br>GAGCAATACAACACGTTCTTTATTGAAACAACAAAATCACA<br>TCATCACTCTCCATAAATGCAAGGTTTACAGGAAGCCGACTG<br>CGTCTGTTCCTTGATCCAATTTACGAAGCTGCTGACCTTGGTA<br>TAGACTCCAGGAACGTGAGGGAGGGCGCAGCGCCTTCCGAA<br>GGAAACAACACCACAGAGGACGTATTCGTTGTCGAGACGAA<br>CGACCAAACCGCTTCCAGAATCACCGATGCAGGCGTCTTTGC<br>CTTCTTCCCCGCCAGCGCAGATCATCATGGGTTCGATCGTAG<br>ACACGAACTGGGTCTGGCATTTGTCGTTGTCCACTACAGGGA<br>GGTCCACAGACTGCAACTTATCCGGATGACCACCGTAGTAAC<br>TCAATCTTCCCCATCCATAGACGGAAACCGTCGTCCCTGGTTT<br>AAGATCGAGGTCAGGATTCGGGAGGTATGCCGTCCTCAGCC<br>CTTGATGAAACCGTACAGGATGCTCTAGCTCCACAATTCCAA<br>TATCCGTGTGGAAGGATTTCGGTATGTACTCAGGAAATTTGG<br>TAAGATTGCGAGTATTCAGAATCTGCCTGTCCTCTGCATCCTT<br>CAGGGAGTAATCTCCAACGTCCACAGATACCTGATCGGGGG<br>TGCGATTTCTTAAGCAATGAGCAGCCGTCACAATATGGCGAT<br>CCGTGATCAACGTACCACCACAAAATGGCGAAATAGGAATC<br>TCGCGCTCGCCAAAATTCGAACGGAACAGCAAACCGCTTGC<br>GTAAGGGAAATAATGTCTACCAACAGCATGTCCACCGACCA<br>CTCGAGTGTCCATGGATTCGGCATTGCTACCGCATGGACACA<br>GAGGCCTTTCGTCTATCTCAGACAACCTCTCCAGAGGATTTC<br>CCTGAATCAGCTGAAGACAGGAGAGAATGCAAATCGCCACG<br>AACATATTACAATATGTATACCTGAGCGAAGGTCGGAACTGT<br>TTCGAATCACAAATACTATGCAACTCACAGGTGGCACACGTC<br>AGGGTTTATAATCAAAATCGACCTCAATACTTCGCAACTACA

							TCGACGCACATGGCAATCGCTGAAAAGCATTTAATCGTGAAT ACCGAAGCTAGACTGACGTAAGGGAA

PhdEnzSeP 11	Q7M4I3	4,37E-37	36,929	PF00089	69293.50<br><br>**66887.53**	MLTSKIGAIILLVFSF<br>LGDKSVA**RVRHLDE**<br>**FTGKSKSKECDCGI**<br>**PFFKSSRVIGNDTQ**<br>**VTYWHQYPWMVA**<br>**LWVGFERKIICGGS**<br>**LIDTQYVLTAAHCL**<br>**DRLILYDKMPAAIK**<br>**ALMLKPEVEIILSLH**<br>**RVGEAMVTMGIAA**<br>**YFIHPTYDENNSKPF**<br>**NDIAIIRLEKPIFYSK**<br>**RLKPICLAVDPKMT**<br>**RVGTKVRVTGFGYI**<br>**SNNKTMADVLQEI**<br>**EVPIQKNSVCKKNL**<br>**KTVVLETAICAGGR**<br>**KNEDACQGDSGGP**<br>**MFYTDSDDRSYQV**<br>**GIISWGEGCGRQG**<br>**KPAIYTRINSFLHWI**<br>**RNITADSPSCEVMF**<br>**PPLVVANIGDCGIP**<br>**EQRCTDGAPPCTSG**<br>**EFMYSWMAKIFYED**<br>**KYLGTGVLVNRSYI**<br>**LTSVKVLPPWRHFN**<br>**RTHLHVILGMRREV**	GCGGAAGTATCGGTGAGTGTTTACGTTTCCTTAGTAAATGGG<br>GAAAACCTTGATTTGCACGGAAATATGCATTCAGATGAGAA<br>ATTGCAGTGAAATGTGAAGGATGATGTTTTGTCCAATTGTATT<br>TTGTACTTTTTGCGTATCTCTGGATAGATAAATTTTTTCTATTG<br>TTCCTCTCTTTCATAAAGTTGTCAGAATAATCCTCGTACAAAC<br>AGTGACATAATATTCATGTTAACTTCGAAGATAGGAGCAATA<br>ATCCTGTTAGTATTTAGCTTTCTAGGGGATAAAAGCGTTGCTA<br>GAGTACGGCATCTGGATGAATTCACGGGGAAAAGCAAAAGT<br>AAAGAATGCGATTGTGGTATTCCTTTCTTCAAGTCGAGTCGA<br>GTCATTGGAAACGATACTCAAGTGACCTACTGGCATCAATAT<br>CCATGGATGGTCGCCCTTTGGGTAGGTTTTGAAAGAAAGATT<br>ATATGCGGGGGCTCTCTCATTGATACTCAGTACGTACTAACC<br>GCGGCGCATTGCCTTGACAGACTGATCTTGTACGACAAAATG<br>CCTGCTGCTATCAAAGCCCTGATGCTAAAACCCGAAGTTGAG<br>ATTATCTTGAGCCTGCATCGTGTGGGAGAGGCAATGGTGACC<br>ATGGGTATCGCTGCCTACTTCATACATCCCACTTACGACGAA<br>AACAACAGTAAGCCCTTCAACGATATAGCCATCATCCGGCT<br>GGAGAAACCCATATTCTACTCGAAGAGATTGAAACCCATCT<br>GCCTCGCAGTGGATCCCAAAATGACTCGCGTCGGAACTAAA<br>GTCAGGGTGACTGGCTTTGGATACATTTCTAACAATAAGACG<br>ATGGCCGACGTTCTACAAGAAATCGAGGTACCGATCCAGAA<br>AAACAGTGTATGTAAAAAGAATTTGAAAACTGTCGTTCTGGA<br>GACTGCTATTTGCGCAGGAGGAAGAAAGAACGAGGATGCCT<br>GCCAAGGTGATTCCGGTGGACCTATGTTCTACACGGATTCTG<br>ATGACCGGTCATATCAGGTGGGCATCATCTCTTGGGGAGAGG<br>GTTGTGGAAGACAAGGCAAACCTGCTATTTATACGCGCATCA<br>ATTCCTTTTTGCACTGGATAAGAAATATTACCGCAGATTCGC<br>CGTCATGTGAAGTGATGTTCCCACCTTTAGTGGTTGCCAATAT

						**KNDLDASQLKTRIA**<br>**SIIYHPLLQQVTAVI**<br>**AKLDIATKDITILHR**<br>**PLCVPERRTRRRVG**<br>**EKVSLMFWKEEKPI**<br>**PMRRKCKRKKCSR**<br>**RCRRKKKTGKKNR**<br>**MKFKQYRHSQEEPY**<br>**EVWNRWKLAHTV**<br>**VKIVEEKRCKVPKA**<br>**MKGRVMCGIMVN**<br>**KRQKCHENGAVLM**<br>**NYVAGRFYMLGIV**<br>**APETKKCSRTKLFQ**<br>**DISWNAEWIVKTM**<br>**TNLRAKVTEE***	TGGCGACTGCGGAATTCCAGAACAAAGATGCACTGATGGCG<br>CTCCCCCTTGCACATCTGGAGAGTTCATGTACAGTTGGATGG<br>CCAAAATTTTCTACGAAGATAAATACTTAGGTACTGGAGTAC<br>TTGTTAATCGATCGTATATCCTGACTTCTGTCAAGGTCTTACC<br>ACCGTGGCGACATTTCAACCGTACCCATCTCCACGTTATCCT<br>GGGCATGCGCCGCGAAGTAAAAAACGACCTAGATGCCAGCC<br>AACTCAAGACCAGAATTGCATCCATCATATACCATCCCCTCC<br>TGCAACAAGTAACCGCCGTCATCGCCAAACTGGATATTGCCA<br>CAAAAGACATAACAATCCTGCATCGTCCTCTCTGCGTGCCGG<br>AGCGTCGAACGAGGCGCAGGGTTGGCGAAAAAGTCAGCCTC<br>ATGTTCTGGAAGGAGGAAAAACCAATACCTATGCGGAGGAA<br>GTGCAAAAGAAAGAAGTGCTCTCGACGTTGCAGACGCAAAA<br>AGAAAACTGGCAAGAAAAACAGAATGAAATTTAAACAATA<br>CAGACATTCGCAGGAAGAGCCCTATGAAGTATGGAATCGCT<br>GGAAACTAGCACATACAGTTGTGAAAATTGTGGAGGAGAAA<br>AGGTGCAAAGTCCCTAAAGCTATGAAAGGCCGAGTGATGTG<br>TGGGATCATGGTAAACAAACGCCAGAAATGTCACGAAAATG<br>GAGCGGTCCTCATGAATTACGTAGCTGGGAGGTTTTACATGC<br>TGGGAATTGTCGCTCCGGAAACCAAAAAGTGTTCTAGAACG<br>AAACTGTTCCAAGACATTAGCTGGAATGCGGAGTGGATCGTT<br>AAGACGATGACTAACCTCAGAGCGAAGGTAACAGAAGAAT<br>GACCCTCGCATGGACTTCAAACCGTACTCCTTTACATCCTGC<br>CGGGGAAATGCAAAAATGTCAACTCGTTTGAAACCAAAACT<br>GAAGAGCTGAGTGGATAAACATGTTACGTAACAATTCAGTG<br>AATTTGAGAAAATCAGCTTTAAAAAATTTATAGGATGTTCTT<br>TTTTGTTACTTCACTCAACATTGTTTTTACGGCACTCTTGCGAA<br>GAAATAGCATTCAAAATGTTCGGGTTTTATTGACATGGCTAT<br>AGCTCTTCTGATCAGGTACCACCCGAAATCTCCATTTCTTCAA

							AAATGTCACTTAACATGTTCATCCACTCAGCTCTTCAACTCTCTGATTTCTCTCAGTATCTTTCGGATGTGGCACAAATCTTGCGTGCGAAATAATACGAGACATTTTATAGAGAAAATAAACACACATTTGTTGTTAAGTTTTAATATGTGTTGCTTAATCAAAGCAGTAAATGTATCTGACTTTGAAACGTCATATAAGGTCGAAATCCACATTTTTTCAAATTTAACATGTTATTAAGCACCAAAGCTTTAGCTCTTCAGAATGGTTTCAATTTTGTTTATAAGCTCATTAAAACTGGG

PhdEnzSeP 12	Q7M4I3	0	34,783	PF00089	54242.75<br><br>**51621.49**	MCFRIYLLTALLHLL<br>LHQTVHC**NPRRCK**<br>**TFAGLPGLCVALRF**<br>**CRVRAVKLSPCDA**<br>**RNELFCCPFPPNGT**<br>**LDWSMEDREPIIFP**<br>**NDVPKKPSVYEESE**<br>**NDHDPVDVVFPRD**<br>**DGAPRRRPTTPFTT**<br>**SATRVPIRTLQKMP**<br>**NTARERNPDESVYH**<br>**GGWVTEDEDRVLY**<br>**EVLPDPKGTKLTSIT**<br>**TTSTSIPPTVRSTTV**<br>**SVDERFEERTTQSSL**<br>**DKLTIGSFDSDDAK**<br>**KVPEPACGHVPFTP**<br>**FIAGGKESMPNQW**<br>**PWMAAIFQRFTTAR**<br>**PNKFLCGGSLISTRY**<br>**ILTAAHCCVSGVSS**<br>**IPLPASSFLVRMGSI**<br>**GLNDGDTFSVGKV**<br>**TVHSNFSYTDQFN**<br>**DIALMRLTAPLPSY**<br>**TDRIAPVCLPYPTLL**<br>**DTDLVDHRATVIG**<br>**WGANTLGGDPEQT**	ACCGTTGTTACAAGTTAAAAATAAAGTTACAATGGTTGGGAG<br>GTCAAAAATCACATACTGTAAACCAGAAAGTTTCTGGGTTCG<br>TAGATAGTATTTACTTTAAAATAATGATCACATATATGTATAT<br>TCTGTCTATTTATTTTTCCCTGAACCGTGGAATGAGCCAATGA<br>ATTTTACTCTCCGTTTCCACGCAGTTAGTTGAGTAAAGCATTT<br>CTGTGAAATTTCGGTGGTTTAGAGCAGAGTATCCTACACCTTT<br>TCAAAGAACCTTAAACAGACGTGGATGTTCGTTGAAATCGAT<br>AGGATCCGACTGAATTTTCATTCAGTCTAAATCGATCAGTCA<br>GTCTTGAGTGTTGCTATATATCCACTCCAGGTAGTGAGTGAC<br>CCTCGTATAAACTCCTGGAAATCCGGGTTCTGCGCATCTGTA<br>GCCAAAAGACACCAGACCCACGACGGTCCATCTATCGTTGTT<br>CAGCGGTAGCATCAGAGGGCCTCCTGAGTCACTCTTGCAAG<br>AATCCTTCCCGCCTTCTCTGAGACCGGCACAAAGAACGTGAG<br>TAGAACCCCTGGCGAGAAAGGCCGCACTCTTAATTCGAGAG<br>TAGGCCAGTGCGCAGTCGTCTATTGACACGATAGGGATGGTC<br>ACCTCATGAAGGGTCTGTTCCGGGTCGCCTCCGAGGGTGTTG<br>GCACCCCATCCAATGACCGTGGCCCTATGGTCCACGAGGTCT<br>GTATCGAGCAGAGTAGGGTATGGCAGACAGACGGGCGCGAT<br>GCGATCAGTGTAGGAAGGGAGCGGTGCGGTCAGCCTCATCA<br>GGGCGATGTCGTTGAATTGGTCAGTGTATGAGAAGTTAGAAT<br>GCACGGTCACCTTGCCCACGGAGAAGGTGTCCCCGTCGTTCA<br>ACCCGATGGAGCCCATGCGAACCAGGAACGACGATGCCGGG<br>AGTGGGATGGAGGAGACCCCGCTGACACAGCAGTGGGCAGC<br>GGTCAGTATGTACCTCGTGCTGATCAAGGAACCGCCGCAGA<br>GGAATTTGTTCGGACGAGCCGTGGTGAAACGCTGAAAGATG<br>GCGGCCATCCATGGCCACTGGTTAGGCATAGATTCTTTCCCT<br>CCGGCGATGAAAGGCGTGAACGGGACATGGCCGCAAGCTGG<br>TTCGGGCACTTTTTTGGCGTCATCGCTGTCAAAAGATCCGATC

						**<u>LHEVTIPIVSIDDCALAYSRIKSAAFLARGSTHVLCAGLREGGKDSCKSDSGGPLMLPLNNDRWTVVGLVSFGYRCAEPGFPGVYTRVTHYLEWIYSNTQD*</u>**	GTGAGTTTGTCCAGACTTGACTGGGTAGTCCTCTCTTCAAATCGTTCGTCGACCGATACAGTCGTAGACCGCACAGTCGGAGGTATCGATGTTGATGTCGTCGTTATCGAAGTCAATTTGGTTCCTTTAGGGTCGGGTAGAACTTCGTAAAGTACCCTGTCTTCGTCTTCGGTCACCCAGCCGCCGTGGTACACGCTTTCGTCCGGGTTCCTCTCTCTAGCGGTGTTGGGCATCTTCTGCAGGGTCCTAATCGGTACTCGGGTCGCGGAAGTGGTAAACGGAGTCGTCGGTCTCCTTCGGGGTGCCCCGTCGTCCCGGGGAAACACAACGTCAACGGGATCGTGGTCGTTTTCGGACTCTTCGTACACCGAAGGCTTCTTGGGCACATCGTTGGGGAAGATGATCGGTTCTCGGTCCTCCATGCTCCAGTCGAGGGTTCCGTTAGGGGGAAAGGGGCAGCAGAACAGTTCGTTTCTGGCATCGCAAGGGGACAGCTTCACGGCACGCACACGACAGAATCGTAAAGCAACACAAAGCCCAGGGAGTCCCGCGAACGTCTTGCAACGCCTCGGATTGCAGTGGACTGTCTGGTGCAGCAGTAGATGCAAAAGTGCAGTCAGTAGGTATATCCTGAAGCACATCGTGTTTATTTCTGTTTCAAGTATGGAAATGCATAATTTATTCTCTCTAGGCTTTCATGGATGGAATTCTCTCGCCCTGTTACCTCCTTTCTGAACTGCTGTGCGAAACCCAATACAAAATATTAGGACATCTTTCCAGTCAATGGACGTCGACTCGAATAAAACCAAACCACCACTCCCGTCATATTTCTTTACTCTTTATAACTTCAGGTTTTCGGACTCTAATTGCTTCTTACGACGTAAGTGCCTGCTTTCTCTCTGACCTTGAGAAATGCTAAACTGGTAACTTTCACGACACTGGGTTGAGTTGTTTGGACTTTCCTCGTACGAACGATACCTCTGCTTTCGTATTATACATACGGCATCTTGTATGGGGACATCCGGGTCACCAGTGTAGAGGTCATGAGTGTGGAGGTCACGACTGTCTCTACGGCGAATACATCTCCAGAATCTCCAAG

PhdEnzSeP13	Q7M4I3	5,12E-09	22,286	PF00089	23001,06	CTSVCFTSRDNLLDPDKWTYVGGLDHANNSSSGIQSHMVSKIIPYPDQSTPLFFYEHDVALVKIKDTIKMDEYSKYACVAKDASVGKDCFTVGWNKNSSDGDIQKQFYSIPLSIISPEECNGTMNNNGMLLDSLICGSNNNESIQLCQMDLGSPLFCLNRNKHWEIQGILTFPSPCDIAQPTVFNSIASVRKWIIDIITNDEKLLQ*	CTCCTTTGGACAAATATACAGTAAATGTCCAGTACAAATTGAAAATATTCTTCACAGTTCTTGAAATAAGTTATTTATCTGTATTTATATATCCCCATGCTTAACAAATTAATGTACTTCTGTATGTTTCACTTCCAGAGATAATCTACTGGATCCAGATAAGTGGACTTATGTAGGTGGTTTAGACCATGCCAACAATTCAAGCAGTGGAATTCAAAGTCATATGGTCTCCAAAATTATTCCATATCCTGATCAAAGTACACCACTTTTCTTTTATGAACATGATGTGGCTTTGGTGAAAATTAAAGATACAATAAAAATGGATGAGTACTCCAAATATGCATGCGTGGCAAAAGATGCTTCAGTAGGAAAAGACTGTTTCACAGTTGGCTGGAATAAAAATTCTTCTGATGGTGATATTCAGAAACAATTCTATTCCATTCCTTTATCAATAATTTCCCCTGAAGAATGCAATGGCACCATGAATAACAATGGAATGTTATTGGATTCCTTAATTTGTGGAAGTAACAATAATGAAAGCATTCAGTTATGTCAGATGGACTTGGGATCTCCTTTGTTTTGCCTTAACAGAAACAAGCACTGGGAAATTCAGGGAATTTTGACCTTCCCAAGCCCTTGTGATATAGCTCAACCAACTGTATTCAACAGCATTGCAAGTGTTAGAAAATGGATTATTGACATCATCACAAATGATGAAAAACTGCTACAGTGAATTGCTGCTCAAGACTAATACAATTATTTAAAATGTGATATTTTACATCTCAATATTTGGGAAAGTTCTGATGCTTCATTTACAAGACATTTTGCATACTTGTTGCGTTAAAAACTGTGATTAGTGTTTTAATAGCCTTCTCCAAAGGGGTGTATTTTGTTAATTTGACAAAATGTGTGTAAACTCTGAAAATATTTAAACATTCTATTCAAGACTAGTAACAGTGCAGTGAAGCTAGTCCTACATTTGCAGCATTAATGTGCCAAGAGCACATGTTAGATATCGTCCAAGTGTACGAATCATCTGTGATGTTGAACGTGCCTTAGAAGTGTGACTATACCTTCTGCAGATAGACTAACAACTTTTTCACATGGCAACACATGTACAGTCTTGTATAAAAATCTACCAAGAGTCAATGAACTACAATTTGACCAGTCATCTTCTCCTA

							TTGTGCCTAGCATGTTTTTTACATTTTATATGTGTGTGTCAGTGTGCACATTTAGAAGTAACTGTATGAATGTTGTGTTTTTAGGGTTTTTAAATTTCTGTTATTTAAAATGTGTTTTAACTGCAATATTTCTACTCTTATCAAAATGAGACACTTACCATTTTTCTTTTACAGCTGGTCAATTTACATGTTAGATTTTAAAAATTTTATCAGTTATCTTACGACATCTTGTGCTGATGTATAAGGTAAGAATATTTTATAATCGCTTTTACCTAGCCCACTGTACGTAATTCAATGATATTAACTGTGTTCAGCATTTTCAATTTTAATACAGTGTATCAGGAATATAAATTGTACTCAGTATGAGCATTTTTTATTCAGCCATTTTAAAATAATGAATTTTCTGCCCTTATTTTTTAAAATATTTTTTCACAGAGTAGGAAGTAATCTGGAATTCAAGCAAGTCTAGCAATTTCATATATCATTCCTGCTAAGCCTTTTGTTGTATGTACATGTGCCAGATGTGTTTTTCATGCTTTCATATTTTGTTTTTTCATCATCTTTTTATGTACAGGTCACTGCTTACTTTTAAAATAGCTTCTTTCAATTTCCTAATTCTTAAACAAACTACCAAAATAAATGTGTGGAGTAAATTATAAAATGATTTCATATTTAAGATAGATTTCAGTTCTTTGCGATACTTGAAGAGGTTGTAATATATATTTTGAAATGGATTTTAGACATTTTTGTTTTCAAGTTGTATCATATTGCTAGTACACATGAGTGATAATTCGTGATGTTTATGTGCCATTTATGAGTTTGAGTTTCTTTGGAATGATAATTTTCAATCATAGTGTCGATGGAAAAAAAATAGAAAAGAAAGAGTCCCATTCCTTTTTGAGTTGCTTGTTAATAAATATACTTATTCACCTAGTCG

PhdEnzSeP 14	Q7M4I3	9,71E-39	35,124	PF00089	41558.93<br>**39415.16**	MKKLSICLVLFLTLH<br>SVLC**APQSDEGLDY**<br>**SEPGAATEDCLCVP**<br>**FYQCVDGEIVDDG**<br>**SNIIDPRKKVQEEEL**<br>**GLDEKYVPPECGPY**<br>**HVCCRNPESSTAQP**<br>**YEHRCGTRNPGGI**<br>**NGRILTSQASGEAE**<br>**FGEWPWQVAVLKK**<br>**EGNEFIFKCGGTLID**<br>**ERHILTVAHCADKL**<br>**VEEMDKIVARLGE**<br>**WDTQNTDEFLPHE**<br>**DYGVSEIIIHPQYRS**<br>**NNLFNDIAILKLSED**<br>**VAFKPHIDTACLPR**<br>**DEDDFTGQECIVTG**<br>**WGTNAYKSGVFSM**<br>**IMKEVSVPVLGHNE**<br>**CQNMLRKTRLGRY**<br>**FKLHGSFLCAGGKE**<br>**GEDSCKGDGGGPL**<br>**VCFRNDNSYTVAG**<br>**LVAWGIDCGQPNV**<br>**PGVYVNVKKFIDWI**<br>**SSKTEKPLEDYWRQ**<br>*	TTTTTTTTTTTTTTTTACTGGAGGTTTTTTTTTTTTTTAATCAATAC<br>ATCTTATTGGTTTAAATTTTTTATCCTGAAACTGCTTTAATAA<br>ATGCGATTGAATGGGCAGATTATCGATAGTTACATCACTGTG<br>ATAGAATCTATAACTAGCATCTAAAAATATTTACTATTTTTAT<br>TTTTTGCTGTTGATTTAGAATTAGAAAATATAATTATAGGACT<br>TGTCTGGCATGTAAATCTAATACGGTAGAATTTTATAATTCTC<br>ACCCCCTGTAGATGAGTTACCCCCTTCGTGATGTACAATACTT<br>TCAATAAATATGCCATAATTTTCCCATGAGTATTGTGTAGTAT<br>TGCTAGAAATGTGCTAATTTTCCTCATGCAGTCAGTCATACAT<br>GCCAGCAAATGTGAAAAGTATACGAACCACAATGAAATATG<br>CCCCGAGTATGAACGAGATTTCGTTCAAAGTTGGCACTTCGT<br>TAAATTTCATGAACGGAGTGCCAACTTTTGTCCTCGAAGAAT<br>ATTTTATATATTCGCATGGATGCTTCAGTTTGAAGTGCATAAT<br>GGATTACTTTGGTTTAAGAGAAATGAAAACTTCATTCATTTTT<br>TAAAAAAATAAATAAATAAAGCTTTTAATAAACCAGTACAC<br>TTTTCTAAGCACCCGAACTGTGCCAAATGTTTCCCAAGAAAC<br>TTTACATTGAAAGTGGAACAGCTTAAGAATATTCTCTAATTTT<br>TGTAAGTCATATTTCGCTTCGAGATCACTGCCTCCAGTAATCT<br>TCTAAGGGTTTTTCTGTTTTTGATGAGATCCAGTCGATGAATT<br>TTTTTACGTTAACGTACACACCCGGTACGTTAGGTTGGCCGC<br>AATCAATACCCCAAGCGACCAAGCCAGCCACTGTGTAAGAA<br>TTATCATTTCTGAAGCATACAAGAGGACCACCACCATCACCT<br>TTGCATGAATCTTCACCTTCTTTGCCTCCAGCGCATAAGAAAC<br>TTCCATGCAACTTGAAATATCGTCCCAATCGGGTCTTACGTA<br>ACATGTTTTGGCATTCGTTGTGACCAAGAACTGGTACACTGA<br>CTTCTTTCATGATCATTGAAAACACTCCACTCTTATAGGCATT<br>GGTTCCCCATCCAGTTACTATGCATTCTTGTCCTGTAAAATCA<br>TCCTCGTCTCTGGGTAAACAGGCTGTGTCGATGTGGGGTTTA

							AAGGCAACATCTTCGGACAATTTCAAGATAGCGATGTCATTGAACAGATTGTTAGAACGATACTGTGGGTGAATGATTATTTCTGATACACCGTAGTCCTCGTGTGGAAGAAACTCATCGGTGTTTTGAGTATCCCACTCTCCAAGACGTGCTACTATCTTGTCCATCTCTTCTACCAATTTATCCGCGCAATGAGCCACTGTGAGGATATGTCTCTCATCAATGAGGGTTCCTCCACATTTAAAAATGAATTCATTGCCCTCTTTCTTCAAAACAGCAACTTGCCAGGGCCATTCCCCGAATTCTGCTTCTCCAGAGGCTTGCGAGGTTAGGATTCTTCCATTGATCCCACCTGGGTTTCTTGTTCCACATCTGTGCTCATAAGGTTGAGCGGTTGATGACTCGGGATTGCGACAACACACGTGATAGGGACCACATTCTGGAGGCACATACTTCTCATCTAATCCTAGTTCTTCTTCTTGAACTTTCTTCCTTGGATCTATGATGTTTGATCCGTCATCCACTATCTCTCCATCCACGCACTGGTAGAATGGTACACATAGACAATCCTCCGTAGCCGCTCCAGGTTCCGAATAGTCTAAACCTTCATCACTTTGGGGCGCACACAACACAGAGTGCAGAGTAAGAAATAGCACAAGACATATGGATAACTTCTTCATAACTAAAATTACATGTGGCCTGCAGCAAGACCGGTCTGACTCGTCGTATCCTCAAACACGGAATGGGATTGATGTAGCAAACAGGGAGAGCGTCCCTTTG

PhdEnzSeP<br>15	P84033	1,47E-<br>173	94,286	PF00089	30489.46<br><br>**28560.10**	MKLLTITFLSLLCGYS<br>SG**KLYTVKDCGKSL**<br>**TAQGRIVNGTVTTP**<br>**GKYPWMVSIHERV**<br>**KDVMRQACGGAIL**<br>**NENWIVTAAHCFD**<br>**QPIILKDYKVYAGL**<br>**YSITKKNAPTVQKF**<br>**QLSKIIIHEKYVKDG**<br>**FANDIALIKTATPIN**<br>**IKGSKGYVNGICFP**<br>**SGATDPSGEATVIG**<br>**WGMIRGGGPISAEL**<br>**REVTLPLVPWQKCK**<br>**QIYGHPDSEFEYIQV**<br>**VPSMLCAGGNGKD**<br>**ACQFDSGGPLFQY**<br>**DKKGVATLIGTVA**<br>**NGADCAYAHYPG**<br>**MYMKVSAFRSWM**<br>**DKVMT***	GCGGAACCCTTTTACCTGTTTTTAAGAACATCTGTGATCTCTA<br>CTTCTGCTCGTTTATAAAAGCTGATACCTCAGAAAAAATTGTT<br>GCATTTGAGTGGAGAGACCTCTGGCATTGAAGTTCAGATTTA<br>TTGCGTCACACGTTTTCGGCAGCGTCATGAAGTTGCTGACAA<br>TTACTTTTCTCTCTCTACTATGTGGATATTCTTCAGGTAAGCTC<br>TACACTGTCAAAGACTGCGGTAAAAGTCTCACTGCACAGGG<br>CCGGATCGTCAATGGAACTGTTACTACACCAGGAAAATACC<br>CTTGGATGGTCTCCATTCACGAAAGAGTTAAGGATGTCATGA<br>GGCAAGCCTGTGGAGGCGCCATCCTGAATGAAAATTGGATA<br>GTTACTGCAGCTCACTGTTTCGATCAGCCAATCATTCTGAAA<br>GACTATAAAGTATACGCTGGACTTTATTCCATAACAAAGAAG<br>AATGCACCAACTGTTCAAAAGTTTCAGTTATCAAAGATCATC<br>ATTCATGAGAAGTACGTTAAGGATGGATTCGCAAATGACATC<br>GCCCTGATCAAGACGGCCACTCCGATCAACATCAAGGGATC<br>CAAGGGATACGTCAACGGAATCTGTTTCCCTTCGGGTGCCAC<br>AGACCCATCTGGCGAAGCAACTGTCATCGGTTGGGGCATGAT<br>AAGAGGCGGGGGTCCCATCTCCGCTGAGCTGCGAGAAGTAA<br>CTCTTCCACTCGTGCCCTGGCAGAAATGCAAGCAGATCTACG<br>GACATCCCGACTCCGAATTTGAATATATACAGGTGGTGCCAT<br>CTATGTTGTGCGCGGGTGGTAATGGAAAAGACGCATGCCAGT<br>TTGATTCCGGAGGTCCTCTATTCCAGTACGACAAAAAAGGAG<br>TGGCGACTCTGATCGGAACCGTTGCAAATGGAGCAGACTGT<br>GCCTATGCCCATTACCCAGGAATGTACATGAAGGTATCCGCT<br>TTCAGGAGCTGGATGGACAAAGTTATGACGTGACCTCCTTTC<br>AAAAAATCTGTATTCGATAGAAACTAGACATCGTTACGCACT<br>AAAAGTATTGTTATTCTTTTAAGATTGTAACTGATTTTCAATA<br>AAAGTGGTTCGAGAAAAAAAAA

PhdEnzSeP 16	P84033	1,15E-172	94,286	PF00089	32118,3	LSTPRYWTRFLYFRL LTITFLSLLCGYSSGK LYTVKDCGKSLTAQ GRIVNGTVTTPGKYP WMVSIHERVKDVM RQACGGAILNENWI VTAAHCFDQPIILKD YKVYAGLYSITKKN APTVQKFQLSKIIIHE KYVKDGFANDIALI KTATPINIKGSKGYV NGICFPSGATDPSGE ATVIGWGMIRGGGP ISAELREVTLPLVPW QKCKQIYGHPDSEFE YIQVVPSMLCAGGN GKDACQFDSGGPLF QYDKKGVATLIGTV ANGADCAYAHYPG MYMKVSAFRSWMD KVMT*	GCGGAACCCTTTTACCTGTTTTTAAGAACATCTGTGATCTCTA CTTCTGCTCGTTTATAAAAGCTGATACCTCAGAAAAAATTGTT GCATTTGAGTGGAGAGACCTCTGGCATTGAAGTTCAGATTTA TTGCGTCACACGTTTTCGGCAGCGTCATGAAGTAGGTAGTCA AAATGACGAAGGTTTGGAAGAACCCATATTCCCAGTCGCATT GATAACCTAGTGTTAAAATCTTTCAAATGGATATTTTCTAATT ATCTACTCCTAGATATTGGACAAGATTCCTTTATTTCAGGTTG CTGACAATTACTTTTCTCTCTCTACTATGTGGATATTCTTCAGG TAAGCTCTACACTGTCAAAGACTGCGGTAAAAGTCTCACTGC ACAGGGCCGGATCGTCAATGGAACTGTTACTACACCAGGAA AATACCCTTGGATGGTCTCCATTCACGAAAGAGTTAAGGATG TCATGAGGCAAGCCTGTGGAGGCGCCATCCTGAATGAAAAT TGGATAGTTACTGCAGCTCACTGTTTCGATCAGCCAATCATTC TGAAAGACTATAAAGTATACGCTGGACTTTATTCCATAACAA AGAAGAATGCACCAACTGTTCAAAAGTTTCAGTTATCAAAG ATCATCATTCATGAGAAGTACGTTAAGGATGGATTCGCAAAT GACATCGCCCTGATCAAGACGGCCACTCCGATCAACATCAA GGGATCCAAGGGATACGTCAACGGAATCTGTTTCCCTTCGGG TGCCACAGACCCATCTGGCGAAGCAACTGTCATCGGTTGGGG CATGATAAGAGGCGGGGGTCCCATCTCCGCTGAGCTGCGAG AAGTAACTCTTCCACTCGTGCCCTGGCAGAAATGCAAGCAG ATCTACGGACATCCCGACTCCGAATTTGAATATATACAGGTG GTGCCATCTATGTTGTGCGCGGGTGGTAATGGAAAAGACGCA TGCCAGTTTGATTCCGGAGGTCCTCTATTCCAGTACGACAAA AAAGGAGTGGCGACTCTGATCGGAACCGTTGCAAATGGAGC AGACTGTGCCTATGCCCATTACCCAGGAATGTACATGAAGGT ATCCGCTTTCAGGAGCTGGATGGACAAAGTTATGACGTGACC TCCTTTCAAAAAATCTGTATTCGATAGAAACTAGACATCGTT

							ACGCACTAAAAGTATTGTTATTCTTTTAAGATTGTAACTGATT TTCAATAAAAGTGGTTCGAGAAAAAAAAA

PhdEnzSeP 17	Q7M4I3	2,68E-44	37,849	PF00089	43468.31<br>**40589.81**	MLLYKLLENVFTFTL<br>IFLATRVYS**QIIFPDD**<br>**NRPITYFPTCNTESS**<br>**CIPIRRCLSLRNRRD**<br>**WRTVGVTCGHGTE**<br>**GYRRVCCPRRVDV**<br>**VITRPPPPPPTTTTT**<br>**TPRPRRGGNAPSN**<br>**NQLPPSAGGRNNA**<br>**NCGTPSTPIGIRPHI**<br>**MGGRNATDGDWP**<br>**WMAAIRIRQSSGRT**<br>**TPWCTGFLIDRRHV**<br>**LSAAHCFERRDVDL**<br>**YSARIGRVNQDDA**<br>**DEYLVSEIRVPEGYR**<br>**GGFYYDDIAILKLSR**<br>**EVTDPNFNPVCLPD**<br>**ETITNMNLTGMGT**<br>**TVAGWGAEGPTNP**<br>**RMSRILKELSEMPV**<br>**MSNSQCLRTFRNEL**<br>**RSFRRQFPDGLTSG**<br>**FLCAGFPEGSKDAC**<br>**GGDSGAPLMFIDR**<br>**NRWYAVGIVSFGYS**<br>**CGVPGIPGGYTRVS**<br>**NYLDWIRENSRD**	CGACGAACTGCTTCTGCCTTTTTAACCCTTCAAGCTCCTCACC<br>ATTAATACAAGTAAATCATGTTACTATACAAGCTTTTAGAAA<br>ACGTGTTCACTTTTACACTCATATTTCTGGCCACAAGAGTTTA<br>TTCTCAAATAATCTTCCCAGACGATAATCGACCAATCACGTA<br>CTTCCCGACTTGTAATACTGAGAGTTCCTGCATTCCGATTCGG<br>CGATGCTTGTCTCTGCGAAATAGGAGAGACTGGAGAACAGT<br>CGGAGTGACCTGCGGCCATGGAACAGAAGGGTACCGAAGA<br>GTCTGTTGTCCGCGACGAGTAGACGTAGTAATAACAAGACC<br>ACCACCACCACCACCAACAACAACAACAACAACACCCAGA<br>CCGAGAAGAGGAGGAAATGCTCCTTCAAATAATCAGCTTCC<br>CCCATCTGCTGGTGGGCGTAACAACGCAAATTGTGGAACTCC<br>ATCCACGCCTATTGGAATTCGACCGCACATAATGGGTGGAAG<br>AAATGCGACAGATGGAGATTGGCCATGGATGGCGGCCATCA<br>GAATTCGACAGTCATCTGGGAGGACAACACCATGGTGTACA<br>GGATTTCTCATCGATAGAAGACACGTCCTGTCAGCGGCCCAT<br>TGCTTTGAGAGAAGAGACGTCGACCTGTATTCGGCACGTATC<br>GGCAGAGTCAATCAAGATGATGCTGATGAATATCTTGTAAGT<br>GAAATAAGAGTTCCAGAAGGTTACAGAGGAGGCTTTTACTA<br>TGATGATATAGCAATATTAAAATTGTCCAGAGAAGTGACCG<br>ATCCAAATTTTAATCCTGTGTGCTTGCCTGATGAAACAATTAC<br>CAATATGAACTTGACTGGTATGGGAACGACGGTGGCAGGAT<br>GGGGAGCTGAAGGACCAACCAATCCCCGGATGAGCCGTATT<br>CTGAAGGAACTGAGTGAAATGCCCGTTATGTCCAACTCACA<br>ATGCCTCCGAACATTTCGAAATGAACTAAGGAGCTTTCGACG<br>TCAGTTCCCTGATGGTTTGACAAGTGGATTTTTATGCGCAGGA<br>TTTCCAGAAGGGTCCAAAGATGCTTGTGGGGGTGACTCTGGT<br>GCGCCTCTTATGTTCATAGATAGAAATCGATGGTACGCTGTT<br>GGAATCGTCTCTTTCGGCTACTCCTGCGGGGTACCTGGAATA

							CCTGGTGGATACACGAGAGTGTCTAATTACTTGGACTGGATAAGAGAGAATAGTAGAGACTGAGTTCTGAACGGAAATTTATTTTTACAACACAGCACTTCTGCGTATTTACTTAAAATTCATTTTTGAGCATTTATGTGGACTTCAAATTTTGTTTGTTCACGAGCACACGTCCTCTTTTAAGCATATTCTTACTATTCATTGATGACAGTGTATTTATTTAGTCTCATTCTTGTG

PhdEnzSeP 18	Q9TXD8	2,76E-42	35,632	PF00089	36419,45	GIPKVCCGLPLVLGP TLSKKVEEEKARNR RCGTVTWQDDISET ERRFVEIHQSISPVDT SDPSKFDPPLDLDTE FGITVVGGTVAEQG TFPWMVSIRRLGNH WCGGSLIDRKHILSA AHCFVINGRKPDKT DFMVHVGNIHVDE GYPFSVAKIVIHPQY KPEHHYFDLAMLTL SEEILIPQFAHICLPSP RLSAMDLTGKNTSV LGWGDTSFGGKPTP VLNKVDGLPVVSNN ECKKSYKKFSVDQLP SGLTDDFICAGDQD GGRDACQRDSGGPL MYEDNHMNLPVYG SDIPWVLVGVVSFGF LCGEPGFPGVYTRVS SHMDWITKEMNE*	AGAACATACAGTTGTTCACCATAATTATTCATTCATTTCTTTT GTAATCCAATCCATGTGACTGGAGACTCTGGTATAAACTCCA GGGAAACCAGGTTCACCGCACAGGAATCCAAATGACACAAC ACCTACCAGCACCCAAGGTATATCAGAACCATAGACTGGCA AGTTCATGTGGTTGTCCTCATACATCAGAGGTCCTCCAGAAT CTCTCTGGCAAGCATCTCTTCCGCCATCTTGGTCACCGGCAC AGATGAAATCATCCGTCAATCCGGATGGCAACTGATCAACC GAAAATTTTTTGTAGCTCTTTTTGCATTCATTGTTGGAGACTA CTGGTAAGCCATCCACTTTGTTCAACACAGGTGTAGGTTTTCC ACCGAAAGAGGTGTCTCCCCATCCAAGCACTGATGTATTTTT CCCTGTCAAATCCATAGCGCTTAGTCGTGGTGAAGGCAAACA AATGTGTGCGAACTGTGGAATCAAAATTTCCTCGCTTAAAGT CAGCATGGCCAAGTCGAAGTAGTGGTGTTCAGGTTTATACTG CGGATGAATAACAATTTTTGCCACTGAGAAGGGGTAACCTTC GTCAACATGTATATTACCCACGTGTACCATAAAATCTGTTTTA TCAGGCTTCCGACCATTAATAACGAAACAGTGTGCTGCTGAC AGTATATGCTTTCTATCTATTAAACTGCCTCCGCACCAGTGAT TGCCTAGTCTTCTAATGGACACCATCCATGGAAAAGTTCCTT GTTCAGCTACTGTGCCTCCAACAACTGTTATTCCAAATTCGGT GTCGAGATCTAATGGTGGATCGAATTTAGACGGATCGCTTGT GTCAACTGGAGAAATGGACTGATGAATTTCAACGAATCTTCT TTCTGTCTCAGAAATATCGTCTTGCCATGTAACGGTTCCACAT CTTCTATTTCTAGCTTTTTCCTCTTCAACCTTCTTTGAAAGCGT AGGCCCGAGTACTAAAGGTAAGCCGCAGCATACCTTTGGAA TACCT

PhdEnzSeP 19	P84033	9,75E-55	42,387	PF00089	28066,24	GKKVTVRKCGRSSH<br>GRPRIVGGEPVKEGT<br>FPWIVTLHSKEDEDF<br>KHYCGGSILNERWIL<br>TAAHCLEFPVHPEL<br>YEIYVGLHRLSRKAA<br>KSVKRHGISKIILHD<br>DYSPFYKNDIALLKT<br>EDPIDFSGSEGFVNGI<br>CLPAMNEKTPRRRA<br>MIAGWGSTQSGGEY<br>SDVLLTVPVPMVSG<br>KICYKAYEAFLTEN<br>MLCAGNAGIDSCQG<br>DSGGPLVMWRNGK<br>AILIGIVSFGISCGTGT<br>HPGVYTKVSSYIAWI<br>EKTINSN*	CTCGGGTTTTCAGAGTGATGCGTTGAGGGCTTCTTGACAGATT<br>GATGAAGTTGTCTAGGGGCCTGGGTGTCCCACTGCTTTTGGG<br>TCTATAGTGTATGTGCATATTATACCTATTTTTTTCTGGAAAC<br>GCGCGAAATTGACGAGTTTTCATAAAACTGTGGAATCACCGA<br>TAATGATGCTGCAGATGGATCTCCCAAAAGCGGTTCGTCCGT<br>CACCCAGTTATTAAATTGATAGGTACCCTATTTTTCAGCAAA<br>GATACACATATGTAAAACTGTGAAAATGGTATCTTGTCAGTA<br>ATCTGGCCGCAGAAACGGGGATAAATTCTGGTTTAGTCTCCC<br>GCAATATGTTGGAACCCGTCAAATTGACGGGTATTCATAAAA<br>TTGTGGAATCCCCGATAATGATGCTGCAGATGGATTAGTCAA<br>AAGTGGTTCGTCCATCACGCAGTTATTAAAGAGATCGGTACC<br>CTATCATTCAATAAAGCTACACGTATGTAAAACTATGAAAAT<br>GGCATCTTGTCACAAATTGGTCTGCAGAAACAGCGATAAAC<br>CCTGGTTCAGTTTGCCGCGACATGTTCAGGAAGCTTCCGGAA<br>GCGAAGCTGTTGCCTCATTTCGTTCAGCCGTGGGTTCGTTTCG<br>TTAGCCACTTGCCCAGAGTCACGTAATGTACACAACGGTGAA<br>TCTGTCAAGTTAAAACGGTAGAAACAGGGCAGACAGACAGT<br>TATGCTCTGTCGGATGTCTAACACTGGCAAGAGCCTGAAGTC<br>TCGGAGAGTACGGGACGAGATTAACGCCGATGAAGGTTAAT<br>CTCGGCTGCTCCACCTCTTACCTTCCGCAACTCGATGAAATG<br>GTAATTTCCTTTAAGAGTCCGTCTTTTGGTCAAGAAGAATTTT<br>TATGCAACGAAAGAACAGCGATCTTCTGGGTCTACAGATAAT<br>AGCTGTACCCCAAATATGCAGTGCGCGAAGAGACAGATTCT<br>TGTAGATGGCCTCTTCTCTCCAGTTTTCAAACTAACATCTTGA<br>ACATAGCATCCATAAATGCGTAGTATTACATCACTAGAGCAA<br>TATAAAGAAATTATTTTTCGATAGGCTGACCAACTGGCCAGT<br>GATTTTAAGAACGCAAGACGAGTTTCGATCCCCGTTACCGGC<br>CATACGAAATAATTCTAAAATAAGCAAACCTTGTCGAATCG

							GATATTGTGAAAGGAAATATAGATCTAATAATATTTGTGCAA GGAGTGTGTTTGCGAAAAATGTACAGGCATGAGGGATAAGC TGCAAAATGACTTCTTAACCATCCACAATTATGTAGAAGAGC TGCTACTAGTTTCGGATTAACCGATGCAGTCCTTCGTCAAGG CGAGGTGCATTTGATGTGTACGTCAGTGACGACGAGGCGCA AATGCAGGGAGCATAATTGCTTTGATATGTATAAAGAGGATT AAAATAATTGCCAGAAAAAGGGAGGCGGGTGATACGTCAG GAAAGGGTGGGGAAAAGTGGGCAATACACCCCCTACTTTGA TACATATACAGAGACTTGAGGAGATTATAAGAAAAAGTGCT GATACGTCACAAATGGGGTGGGGCAACACGGGAAATAAACC TGCCAACGCATGGGAGATGCATATAGACAAAGCAGTAAGAT ATAGGAGGAATAAATAAAGTGAGAAATGAGTATAACGATTA AGAAAAAACGATTTTTCAGGCAGAAGGGAAAGATAAATTAG AAAGATCATTAACAAGCGGAATAGAGTTGAGATAAATCGAA AGGTTTTCTGGAAAATCCAATCCCCTTTCTCGCCCTTTTGGAA TGTATCCTCTTTTTCTCGTAACATCTTTAATTATCTTTATGGGT ATCAAAGTAGTTATGTTCTTTCGTTTCTGTTTCGTCTTCATTGA TGTACACATATAATGCGACTAAGGGCTACATTAGTCCGAAGC TAGGTGCAGCTCTTCTGCATAATTGTGGTTATTAATAGGTAAA TTTGAATCGTATCTGTTCTTCATAAGTTTCCTGCGAATATGAG GTACAATGCAATTTACGTGCAAGAAATGTACTATACAAATGT ATGTTTATAAATCTATCTTTGTAAATTAATGTTAAAATAAAAA TTGCTGGATAAAGTTTTTAATAACGAACCAATGATTTAAAAT GAGATGTACTATTATAAAAAAAAACTTCGACCGTTTAATGTA CCTGTCAATATGGCGGTTTTCGGGGAAAACAGGTATGCAGTC AATGTAAATCTAGAGTTAAGTTGGAGAGTGCCGGTATTAAAC CTATTCATGTCAAACATATGCGTCGGCGATCTCGAAATGTAG CTTATTTTTCAATTTTCGATCTCTCATTTGTCTTTAGGGATATG

							AAAAAATCGTGTAAAATCTTGATATTCATTTGACAGTCATTTT<br>ATACGAAAGATGACTGAAGTGCGTATCAAATAACCACATGT<br>CAGACAATACTTTCAGGACAGTGTAAAGCAGTATTACGCAA<br>TTACTTGCAGACTGACAGTCATTTCTAAGACAGACATACTGT<br>TACACACATCTGCAAAGGACTACATAGTGCTACATTTAGTAA<br>ATTTCATCGTCTGGCGACATTTATTGTTCTTGCCGGTAGGTCT<br>CCTTTCGGAACAGGTGTCTCGTATCTTCTAATAAATGCGAGA<br>GCAGACGCTGCTTTCAAACGGCAGTCACCGGAAACCAAAAG<br>CACCAAGAGACATGGTGGAAAATTTTAAGTGGTAAGAACAT<br>TTGCATGCTTAGGTCTCGAGATCCTCACCGTGATCTGAAAGT<br>CTTATTTGAAAGACTGTTAAATGCCTCACCTTATTTTGTACAT<br>TATTTAAAGATACGACTAATAAAACAGGTTATAGTAGCAGTA<br>CGCGCAGTGAAATTCTGAAGGAATGGTTCCATCGTATGTACT<br>GCTGCGATAGGGCGCGAAACAATATAAGAAATCGACAGTTA<br>TTTAGGTTTTAGATTTTTTTTCAATAATTTAAGGTATACCTTTA<br>AAAACACGATTTCCTGTCAAAATATTAATTTTTCAATTTTGTT<br>CTAATATTAAAGCTTGTTCTTTTATCTCTCCTAATCGGTTTAAA<br>TTTTGCTTCCGTCTTATTTCGAATATGCGATAACAGCTATTTTA<br>TTGTTGATTATTCGTAGCAACATTTTAGAACTGGAAGTGAGG<br>TAATTACATCGTAATGTTGTTAAAAGCGAACGTCAAATTTGG<br>ACGATTTAATGTAAAGGAAAAGCAGAAATTAGTATTTTCCCT<br>TGTTTAATATATTTAGAAACATCCGATATCTCAAATTGAGTA<br>ATGAGAACTTCTCTCGCGCAGCATTTACAGATAATACGAAAG<br>GAAGCTTTCGTTTGCCTTGAAAACCGTGAAAATAGTGCATAG<br>GATGTTGCAAAACTATTAAGATGTATGGGCTTGAAGACTGTT<br>TAAATGCATCGAAAATACTGCAATATTCATGTTTCAGGGCTG<br>TAAACATTTAGATATGAATCGAATACAAGGACTGACACAGC<br>ATACCGCCTCTAATACAAAAAAAATTAGAAAAATTGGACA

							GCCCTCAGGAAGGTGAAAAATTACCGTTTTAGACAGCAAAA<br>AGGGTATTTATCCTAAACTAGGGTCAATTTAAAGCTTAATTA<br>TTATTTTAAAACTAGGATTTGTGTTTAGATCTTGCTTTTTCTGC<br>TTTTTTCCCCCTCAAAATGAGATTTTTCATCTTCTGCCTTTACA<br>ACATCGTAACAAAAGAAAAAACTCATGCATGCAATACTTTA<br>AAACATTTTGGCAGTACAAAAGTTTTCCCAAGGGAATAGAC<br>CTTTCCCACGATTCTAGACGCATTAGTTTTTTTCCAATTTTTAT<br>TGAAAAAAAATTAATATATGAATTGTTGTAATTTTTATGAATT<br>TTCTCTTTCTCCGTACTTTAACCGTTATGGCTTATAAAGTAAC<br>AGCGCTATAATTACGGGAATGGTCTATTCCCCCAGAGGAAGT<br>TATTATTACAATAGAAAGTTTAAAATGAAAATATATAGATAT<br>GAATTTGAGTTATCGTGTTTTGCTCAGTAGTTAACTTTGGGCT<br>ATATTTTACTACATTTTTTGGCATGAAAATCTAAAAGAATAGT<br>GAAAACTATGCTCTTTGCTACATTTTTCCAATTTTTAGCCATA<br>TAGGATTATAAAATACAGCTAAGATAAATTTCAATGTTTAAA<br>AGCCATCTCTTAAGGTATACGTATATCTTCATAGAACCAAAC<br>AGGCCCGGCAAGGCCTTTCGGGGGGAAACCCCGCCGGTCAA<br>GAAACACACCGCAGTTGGCAGACGCTTAGATGGCACCCAGA<br>CGCCACATCCCAGGGCGGGCTGCACGAGGCTGGAGCCACAC<br>AAGCTCCGGTGCCGCAGACAGACAAGTTAATAGTTTCGAAC<br>AAATGGCGCGGGTCATGTACCAAAACGGAGAAACGACAGG<br>CAAGCAGGATTGTGAATAGAAATTCTAACACTGTGGCTTAAC<br>TATCTAACTTTCGTTCCAGGTTTTGCGCTATTCTATTGTTTGGT<br>CTCATAGTTGGAGATGCGTCAGGTAACATTTTACTTGTTTACA<br>GAATTATTGTTTTGTGTCCTTCATGATGCTAAGTTACTTACTTC<br>CTTTTATAAGGCAAAAAAGTGACCGTACGTAAGTGTGGCAG<br>GAGTTCGCACGGCCGGCCCAGGATAGTGGGTGGAGAACCGG<br>TCAAGGAAGGAACTTTTCCGTGGATAGTCACACTGCATTCAA

							AAGAGGACGAGGATTTTAAGCACTACTGCGGAGGCAGCATC<br>CTGAATGAACGGTGGATTCTGACAGCCGCGCATTGCTTAGAA<br>TTCCCAGTTCATCCCGAATTGTACGAGATCTACGTTGGATTGC<br>ATCGTTTATCGCGGAAAGCTGCAAAAAGTGTTAAGAGACAC<br>GGGATTTCTAAGATCATTCTACACGATGACTACTCTCCTTTCT<br>ATAAGAATGATATCGCCCTCCTAAAGACAGAGGATCCCATC<br>GACTTCAGTGGATCAGAAGGATTCGTGAACGGGATCTGCCTC<br>CCGGCGATGAACGAGAAAACACCGAGAAGACGTGCTATGAT<br>AGCGGGCTGGGGGTCAACCCAGTCTGGAGGAGAATACTCAG<br>ATGTGCTTCTTACCGTCCCTGTGCCTATGGTATCTGGAAAAAT<br>CTGCTACAAAGCGTATGAGGCATTTTTGACTGAAAATATGCT<br>ATGCGCTGGCAATGCGGGAATAGATTCATGTCAGGGTGATTC<br>CGGAGGACCTCTCGTCATGTGGAGGAATGGAAAAGCAATTT<br>TGATTGGAATCGTATCTTTTGGAATATCATGCGGCACAGGAA<br>CGCATCCCGGAGTCTATACCAAGGTATCTTCATACATAGCCT<br>GGATCGAAAAGACGATAAATTCTAATTAGTCCTAATCTGTGA<br>TCTCTGCTGTTCTTCATAATTGTGGATATTATCTTTTGCATTTT<br>AATGTTCATGTGCTATTAATAAAAATGATATTCAACCGGAAA<br>AAGCGTGTGTCATCTGTAAGATGTTCGAAATGTATTCTTGAA<br>GAAAACCGGTCACTTGCCTGACCGGTAGCGTTATGTAAACCC<br>GCCCACCCCGACAAACTTTTTATCGTTTCATATATACGATACT<br>ATAAACTTCCTATACCTTATCATTTAAAAGCTTTTGAAGTGTA<br>AAGAAAGCAAGACAATAATAAGCCAAACGATGTGTTAAATA<br>GAAAAGTGCAATAACTACAAACACAAAATGATATACACTAA<br>TGCAGATTAAAGATATTATAGAACAAAATCAAATAGAATCG<br>TGATAAAAATCATTTTAAATGAGTGTCGGGGTGGAGGTTGTA<br>ACCCAAATGACGGTAGCCACAGCTATGCCCAGTTAAACAAG<br>CTTTACGTGTAGACAACACACCTGATATGCTGTATGATCCCA

							ACGTAAGGAGTTTTTTTAAGCACGAACTAGCAAAATAACGA<br>AAGCGGGAAAATGGAACAAAATAACACAGTATCTTGGATTA<br>GAACGGAAAGCTAAATGAGGAAAAAGACAATGACATATAA<br>CACAAGCTAAACAATGCAGTAGTAATAAAACAGAAACTAA<br>AACCTACTAAACCAACCTAAGAATTAAAGAACTACCTATAG<br>TAATTAAATATAACTAAAGAAATAATCTAGTGACCTGACATT<br>CTAAATTATATGTCGACATACCAAAAATTCCACTGCCTACTC<br>GCCGCTATAAACTAGATCACTCTTGGAATTAAAACTAACGGT<br>TCATTCTAACTAACAGCACATTCCCAGCTTACAGTACATATT<br>CCTTTACATAGGAACAAAATCTCCATCCTTAAAAGATCAACT<br>CAATTCACTTCCTCCTTAAAATAAAATCAAATTAATTGAAAC<br>AAAATTAACTACAGTACAAATTTGACTGGTACACACAATATA<br>ACTGGTAAACTTCCAAACGTGAGGTGTCTACTGGATGGCTAA<br>CCAGTTGAAAAGTTCTTTCCATATAAATGGAATACATCAAAC<br>ATCTCACGCCTGTATTTATGAATCTATCCCCAAGCAAGAAAC<br>ACAATCAGCTAAAAAAATGTTAACCTGTGTCTAAATTTGTTATT<br>TCAGCAACGGAAACTATAGGGTCATTAATATCTAGAACACA<br>ATCATCAGAGGAAACATATTTCATTCTATATAATTCCCCTTCA<br>AAATTATATCTTTGAAAGTAGAACTGGGAATATTAGATTTAA<br>AATGCTGATATTTACACGAATGAGAATAAAACGAAAATTAT<br>GGTCATGTCCTTTGTAATAAATATTAAAATGCAAAATCCCCC<br>GTCTTGTACGTGAATATTTATATCCAAAATGTTAGCAACTGA<br>ATTGCCATTGAGTATCTGTATGTTTCAATTTTAAATTTTTAGGT<br>TAAAATCCTAAATCCAGATCGTAATGCTTATCTTTACAAAAA<br>GTAAAAAAAAATAAAAAGACAACTAAACCAATGATGTATAT<br>ATAAAATAAGAAGTTCTTTACACTGCTAATTTTAGATTCATA<br>AATGTGCAAGTATATTTACTAATATTTTCCAATGCCAGGCGA<br>TTGGTAATTACGCCATTCGCCCCTTCAAGCTTCCCAATACTTT

							TTATGGGAGGAATTTAAAAAAAAATGAATACAGAGTACGAAT CATGGCGAGTACTGTTCATCGGTTGTCCAGAGTCACAAACAG CAAACGGTTTGATACGAAATAAATTCGCACCAAGACAGTCA CGCCCCCGTCAGGAGGCGAAATGCAACCACAGCCGATTTAC GAGGGAGAGACTTGAAATAGAAAACTTGGGATCGAAAAGAT AGTCTCATATATTTCCCGAAGCCTGATTGCTTATCAAGGAAA ACTAGTTTCTCTTAAACTCGCCTTTTTTTTTTTTTAAATTTAGC AGAATGATAAGTTAAAGCATTTTTTTGGCTTTTGGAGAACAG CGATACCTTTCTTGGCGAGGTGTCAACTTCTCGTTACCCCCTA GGCCACAATAATGAGCGAGGACCCAATGAAGAGTATTCGAA TTACGTAGGGAGTGTAGCGAGCGACTGTAGTATGTGCAGGTA TATATTTACTAAATATTGACGAAATCTTAATCCCCCCCCCCTC TGTCATAGCAATACCTTCGATTTGTTTCACCACGCTATCATTA TGCAAAACACAATTATCTACTACAACTAAAATTGAGCAATCT GAAATATCATTTGAATTCCACAACATTTTCCAATTGGTACAT ACATTTCCTTCCTTACTAGCCTTACTATTAATAAATCTTCAATT TCAATTGAATAAAACGTCTTCGAATGTGTGTATACAACTAAC CTCAAAATCCAGATTATATATCTCGCTGCTTATTACTTATGAA TCTACTCTTTTCTCTATGTTTTATTTTATAGAGAATGCAATTCA AATATTTCGTCAATTCCCTGCCAAACGTATTCATGTTTAAGTA TTTACACTACATGTTGTTTTTCTAAAATTAATTGGATCTTTGCG AAATTTAAGTGCAGCTATGATACACGGAAATCTTATGTTGTT AATTTCATATAACTGATCAGTTTGCGTGTGG

PhdEnzSeP 20	Q7M4I3	1,85E-06	47,500	PF00089	6997.48	MWRNGKAILIGIVSFGISCGTGTHPGVYTKLKLLMAHRQIIQNLLMSESKMFLQVLHCLELGV*	GGGGTGGAGTGGGATAGGGTGGGGAAGGTGTTCATCCTAAAAGTCTCCCTTTCCGCAGTTGCGCTACCGGCGCTAAGTATAAGCAATGACAATTGTATAAACTTCGTTACTTTAGCATATACTATTTTAAGAACAGTCCCTAAATATTTATTTATAGTAGCTCCACTTTTAATCAAATTATTTCGACTCTTTTCCTTCGTTCAGGTCGGGAGCCTTTAATACGGCTTCTGGAAAAGCCGTGCAAGGTATTCTCTTCCGGCCGGTTTGTGAAACTGATTTAAAATTCTTACTCTCTTCACGAGCTACTTTTCAGTTGGATTATGTTTTAATGATAAGTAAAAGGCTTAAAATTAGTAACACAGGACAATATAACGATATCGCTGCCACCCTATTTTCTCTGGCAGGGTGATTCCGGAGGACCTCTCGTCATGTGGAGGAATGGAAAAGCAATTTTGATTGGAATCGTATCTTTTGGAATATCATGCGGCACAGGAACGCATCCCGGAGTCTATACCAAGTTAAAGCTTTTAATGGCACACCGCCAGATTATACAAAACCTACTGATGTCCGAATCAAAGATGTTTTTGCAAGTCCTTCACTGTTTGGAATTGGGAGTCTGAATCATAAGGTCGTTTACCATACTACGATCTTGGATGCTATCGTCTAAAACATGAAGATTGCCTGTAACACTATGTTCTGCATTTTTATCTACAAAATCTTTAAATATTTCATCGCAGCAGCGACAAAAAATCCATTCCTCTACTCATCTCTGCCCATGTCCGCTTTTTAAGCTATAATTAAAAATGACTCTATCCAAAGGGGTTATCAAAAGGGATATTTGAAAGTTTTTACACACACACCTTCCACTCGTCATTGGATGCCATTATCGACAGTTTTGTTGTAGTAAAATTTAAACTCATTCACTTAAATACAGCAATTTTCATTGGATGCAGTGGTGCAGCCATGAAGCAGGGGGGGGGAGTTAAGGACAAACCTCTAGGAGCTAGAAAATCTGTACAAAAGTCCTTTCCCTAAAGACAAATTTTTTTTTCTTTCCCTAGAAAGGAATTCTCCATTTGTAGAGGAACCCCT

PhdEnzSeP 21	P84033	2,16E-41	41,538	PF00089	26347,65	GKKVTVRKCGRSSH GRPRIVGGEPVKEGT FPWIVTLHSKEDEDF KHYCGGSILNERWIL TAAHCLEFPVHPEL YEIYVGLHRLSRKAA KSVKRHGISKIILHD DYSPFYKNDIALLKT EDPIDFSGSEGFVNGI CLPAMNEKTPRRRA MIAGWGSTQSGGEY SDVLLTVPVPMVSG KICYKAYEAFLTEN MLCAGNAGIDSCQL KLLMALRHIIQNLL MSESKMFLQVLHCL ELGV*	TTTAGTTATCTTTGTGGGTATCAAGGTAGTTATGTTCTTTCATT TCTGTTTCGTCTTCATTGATGTACACATATAATGCGACTAAGG GCTACATTAGTCCGAAGCTAGGTGCAGCTCTTCTGCATAATT GTGGTTATTAATAGGTAAATTTGAATCGTATCTGTTCTTCATA AGTTTCCTGCGAATATGAGGTACAATGCAATTTACGTGCAAG AAATGTACTATACAAATGTATGTTTATAAATCTATCTTTGTAA ATTAATGTTAAAATAAAAATTGCTGGATAAAGTTTTTAATAA CGAACCAATGATTTAAAATGAGATGTACTATTATAAAAAAA AACTTCGACCGTTTAATGTACCTGTCAATATGGCGGTTTTCGG GGAAAACAGGTATGCAGTCAATGTAAATCTAGAGTTAAGTT GGAGAGTGCCGGTATTAAACCTATTCATGTCAAACATATGCG TCGGCGATCTCGAAATGTAGCTTATTTTTCAATTTTCGATCTC TCATTTGTCTTTAGGGATATGAAAAAATCGTGTAAAATCTTG ATATTCATTTGACAGTCATTTTATACGAAAGATGACTGAAGT GCGTATCAAATAACCACATGTCAGACAATACTTTCAGGACA GTGTAAAGCAGTATTACGCAATTACTTGCAGACTGACAGTCA TTTCTAAGACAGACATACTGTTACACACATCTGCAAAGGACT ACATAGTGCTACATTTAGTAAATTTCATCGTCTGGCGACATTT ATTGTTCTTGCCGGTAGGTCTCCTTTCGGAACAGGTGTCTCGT ATCTTCTAATAAATGCGAGAGCAGACGCTGCTTTCAAACGGC AGTCACCGGAAACCAAAAGCACCAAGAGACATGGTGGAAA ATTTTAAGTGGTAAGAACATTTGCATGCTTAGGTCTCGAGAT CCTCACCGTGATCTGAAAGTCTTATTTGAAAGACTGTTAAAT GCCTCACCTTATTTTGTACATTATTTAAAGATACGACTAATAA AACAGGTTATAGTAGCAGTACGCGCAGTGAAATTCTGAAGG AATGGTTCCATCGTATGTACTGCTGCGATAGGGCGCGAAACA ATATAAGAAATCGACAGTTATTTAGGTTTTAGATTTTTTTCA ATAATTTAAGGTATACCTTTAAAAACACGATTTCCTGTCAAA

							ATATTAATTTTTCAATTTTGTTCTAATATTAAAGCTTGTTCTTT<br>TATCTCTCCTAATCGGTTTAAATTTTGCTTCCGTCTTATTTCGA<br>ATATGCGATAACAGCTATTTTATTGTTGATTATTCGTAGCAAC<br>ATTTTAGAACTGGAAGTGAGGTAATTACATCGTAATGTTGTT<br>AAAAGCGAACGTCAAATTTGGACGATTTAATGTAAAGGAAA<br>AGCAGAAATTAGTATTTTCCCTTGTTTAATATATTTAGAAACA<br>TCCGATATCTCAAATTGAGTAATGAGAACTTCTCTCGCGCAG<br>CATTTACAGATAATACGAAAGGAAGCTTTCGTTTGCCTTGAA<br>AACCGTGAAAATAGTGCATAGGATGTTGCAAAACTATTAAG<br>ATGTATGGGCTTGAAGACTGTTTAAATGCATCGAAAATACTG<br>CAATATTCATGTTTCAGGGCTGTAAACATTTAGATATGAATC<br>GAATACAAGGACTGACACAGCATACCGCCTCTAATACAAAA<br>AAAATTAGAAAAATTGGGACAGCCCTCAGGAAGGTGAAAA<br>ATTACCGTTTTAGACAGCAAAAAGGGTATTTATCCTAAACTA<br>GGGTCAATTTAAAGCTTAATTATTATTTTAAAACTAGGATTTG<br>TGTTTAGATCTTGCTTTTTCTGCTTTTTTCCCCCTCAAAATGAG<br>ATTTTTCATCTTCTGCCTTTACAACATCGTAACAAAAGAAAA<br>AACTCATGCATGCAATACTTTAAAACATTTTGGCAGTACAAA<br>AGTTTTCCCAAGGGAATAGACCTTTCCCACGATTCTAGACGC<br>ATTAGTTTTTTTCCAATTTTTATTGAAAAAAAATTAATATATG<br>AATTGTTGTAATTTTTATGAATTTTCTCTTTCTCCGTACTTTAA<br>CCGTTATGGCTTATAAAGTAACAGCGCTATAATTACGGGAAT<br>GGTCTATTCCCCCAGAGGAAGTTATTATTACAATAGAAAGTT<br>TAAAATGAAAATATATAGATATGAATTTGAGTTATCGTGTTTT<br>GCTCAGTAGTTAACTTTGGGCTATATTTTACTACATTTTTTGGC<br>ATGAAAATCTAAAAGAATAGTGAAAACTATGCTCTTTGCTAC<br>ATTTTTCCAATTTTTAGCCATATAGGATTATAAAATACAGCTA<br>AGATAAATTTCAATGTTTAAAAGCCATCTCTTAAGGTATACG

							TATATCTTCATAGAACCAAACAGGCCCGGCAAGGCCTTTCGG<br>GGGGAAACCCCGCCGGTCAAGAAACACACCGCAGTTGGCAG<br>ACGCTTAGATGGCACCCAGACGCCACATCCCAGGGCGGGCT<br>GCACGAGGCTGGAGCCACACAAGCTCCGGTGCCGCAGACAG<br>ACAAGTTAATAGTTTCGAACAAATGGCGCGGGTCATGTACCA<br>AAACGGAGAAACGACAGGCAAGCAGGATTGTGAATAGAAA<br>TTCTAACACTGTGGCTTAACTATCTAACTTTCGTTCCAGGTTTT<br>GCGCTATTCTATTGTTTGGTCTCATAGTTGGAGATGCGTCAGG<br>TAACATTTTACTTGTTTACAGAATTATTGTTTTGTGTCCTTCAT<br>GATGCTAAGTTACTTACTTCCTTTTATAAGGCAAAAAAGTGA<br>CCGTACGTAAGTGTGGCAGGAGTTCGCACGGCCGGCCCAGG<br>ATAGTGGGTGGAGAACCGGTCAAGGAAGGAACTTTTCCGTG<br>GATAGTCACACTGCATTCAAAAGAGGACGAGGATTTTAAGC<br>ACTACTGCGGAGGCAGCATCCTGAATGAACGGTGGATTCTG<br>ACAGCCGCGCATTGCTTAGAATTCCCAGTTCATCCCGAATTG<br>TACGAGATCTACGTTGGATTGCATCGTTTATCGCGGAAAGCT<br>GCAAAAAGTGTTAAGAGACACGGGATTTCTAAGATCATTCTA<br>CACGATGACTACTCTCCTTTCTATAAGAATGATATCGCCCTCC<br>TAAAGACAGAGGATCCCATCGACTTCAGTGGATCAGAAGGA<br>TTCGTGAACGGGATCTGCCTCCCGGCGATGAACGAGAAAAC<br>ACCGAGAAGACGTGCTATGATAGCGGGCTGGGGGTCAACCC<br>AGTCTGGAGGAGAATACTCAGATGTGCTTCTTACCGTCCCTG<br>TGCCTATGGTATCTGGAAAAATCTGCTACAAAGCGTATGAGG<br>CATTTTTGACTGAAAATATGCTATGCGCTGGCAATGCGGGAA<br>TAGATTCATGTCAGTTAAAGCTTTTAATGGCACTCCGCCACA<br>TTATACAAAACCTACTGATGTCCGAATCAAAGATGTTTTTGC<br>AAGTCCTTCACTGTTTGGAATTGGGAGTCTGAATCATAAGGT<br>CGTTTACCATACTACGATCTTGGATGCTATCGTCTAAAACAT

							GAAGATTGCCTGTAACACTATGTTCTGCATTTTTATCTACAAA<br>ATCTTTAAATATTTCATCGCAGCAGCGACAAAAAATCCATTC<br>CTCTACTCATCTCTGCCCATGTCCGCTTTTTAAGCTATAATTA<br>AAAATGACTCTATCCAAAGGGGTTATCAAAAGGGATATTTG<br>AAAGTTTTTACACACACACCTTCCACTCGTCATTGGATGCCAT<br>TATCGACAGTTTTGTTGTAGTAAAATTTAAACTCATTCACTTA<br>AATACAGCAATTTTCATTGGATGCAGTGGTGCAGCCATGAAG<br>CAGGGGGGGGGAGTTAAGGACAAACCTCTAGGAGCTAGAA<br>AATCTGTACAAAAGTCCTTTCCCTAAAGACAAATTTTTTTTTT<br>CTTTCCCTAGAAAGGAATTCTCCATTTGTAGAGGAACCCCT

PhdEnzSeP 22	P84033	2,84E-51	42,308	PF00089	30192,07	GKKVTVRKCGRSSH GRPRIVGGEPVKEGT FPWIVTLHSKEDEDF KHYCGGSILNERWIL TAAHCLEFPVHPEL YEIYVGLHRLSRKAA KSVKRHGISKIILHD DYSPFYKNDIALLKT EDPIDFSGSEGFVNGI CLPAMNEKTPRRRA MIAGWGSTQSGGEY SDVLLTVPVPMVSG KICYKAYEAFLTEN MLCAGNAGIDSCQG DSGGPLVMWRNGK AILIGIVSFGISCGTGT HPGVYTKLKLLMAH RQIIQNLLMSESKMF LQVLHCLELGV*	CTCGGGTTTTCAGAGTGATGCGTTGAGGGCTTCTTGACAGATT GATGAAGTTGTCTAGGGGCCTGGGTGTCCCACTGCTTTTGGG TCTATAGTGTATGTGCATATTATACCTATTTTTTTCTGGAAAC GCGCGAAATTGACGAGTTTTCATAAAACTGTGGAATCACCGA TAATGATGCTGCAGATGGATCTCCCAAAAGCGGTTCGTCCGT CACCCAGTTATTAAATTGATAGGTACCCTATTTTTCAGCAAA GATACACATATGTAAAACTGTGAAAATGGTATCTTGTCAGTA ATCTGGCCGCAGAAACGGGGATAAATTCTGGTTTAGTCTCCC GCAATATGTTGGAACCCGTCAAATTGACGGGTATTCATAAAA TTGTGGAATCCCCGATAATGATGCTGCAGATGGATTAGTCAA AAGTGGTTCGTCCATCACGCAGTTATTAAAGAGATCGGTACC CTATCATTCAATAAAGCTACACGTATGTAAAACTATGAAAAT GGCATCTTGTCACAAATTGGTCTGCAGAAACAGCGATAAAC CCTGGTTCAGTTTGCCGCGACATGTTCAGGAAGCTTCCGGAA GCGAAGCTGTTGCCTCATTTCGTTCAGCCGTGGGTTCGTTTCG TTAGCCACTTGCCCAGAGTCACGTAATGTACACAACGGTGAA TCTGTCAAGTTAAAACGGTAGAAACAGGGCAGACAGACAGT TATGCTCTGTCGGATGTCTAACACTGGCAAGAGCCTGAAGTC TCGGAGAGTACGGGACGAGATTAACGCCGATGAAGGTTAAT CTCGGCTGCTCCACCTCTTACCTTCCGCAACTCGATGAAATG GTAATTTCCTTTAAGAGTCCGTCTTTTGGTCAAGAAGAATTTT TATGCAACGAAAGAACAGCGATCTTCTGGGTCTACAGATAAT AGCTGTACCCCAAATATGCAGTGCGCGAAGAGACAGATTCT TGTAGATGGCCTCTTCTCTCCAGTTTTCAAACTAACATCTTGA ACATAGCATCCATAAATGCGTAGTATTACATCACTAGAGCAA TATAAAGAAATTATTTTTCGATAGGCTGACCAACTGGCCAGT GATTTTAAGAACGCAAGACGAGTTTCGATCCCCGTTACCGGC CATACGAAATAATTCTAAAATAAGCAAACCTTGTCGAATCG

							GATATTGTGAAAGGAAATATAGATCTAATAATATTTGTGCAA<br>GGAGTGTGTTTGCGAAAAATGTACAGGCATGAGGGATAAGC<br>TGCAAAATGACTTCTTAACCATCCACAATTATGTAGAAGAGC<br>TGCTACTAGTTTCGGATTAACCGATGCAGTCCTTCGTCAAGG<br>CGAGGTGCATTTGATGTGTACGTCAGTGACGACGAGGCGCA<br>AATGCAGGGAGCATAATTGCTTTGATATGTATAAAGAGGATT<br>AAAATAATTGCCAGAAAAAGGGAGGCGGGTGATACGTCAG<br>GAAAGGGTGGGGAAAAGTGGGCAATACACCCCCTACTTTGA<br>TACATATACAGAGACTTGAGGAGATTATAAGAAAAAGTGCT<br>GATACGTCACAAATGGGGTGGGGCAACACGGGAAATAAACC<br>TGCCAACGCATGGGAGATGCATATAGACAAAGCAGTAAGAT<br>ATAGGAGGAATAAATAAAGTGAGAAATGAGTATAACGATTA<br>AGAAAAAACGATTTTTCAGGCAGAAGGGAAAGATAAATTAG<br>AAAGATCATTAACAAGCGGAATAGAGTTGAGATAAATCGAA<br>AGGTTTTCTGGAAAATCCAATCCCCTTTCTCGCCCTTTTGGAA<br>TGTATCCTCTTTTTCTCGTAACATCTTTAATTATCTTTATGGGT<br>ATCAAAGTAGTTATGTTCTTTCGTTTCTGTTTCGTCTTCATTGA<br>TGTACACATATAATGCGACTAAGGGCTACATTAGTCCGAAGC<br>TAGGTGCAGCTCTTCTGCATAATTGTGGTTATTAATAGGTAAA<br>TTTGAATCGTATCTGTTCTTCATAAGTTTCCTGCGAATATGAG<br>GTACAATGCAATTTACGTGCAAGAAATGTACTATACAAATGT<br>ATGTTTATAAATCTATCTTTGTAAATTAATGTTAAAATAAAAA<br>TTGCTGGATAAAGTTTTTAATAACGAACCAATGATTTAAAAT<br>GAGATGTACTATTATAAAAAAAAACTTCGACCGTTTAATGTA<br>CCTGTCAATATGGCGGTTTTCGGGGAAAACAGGTATGCAGTC<br>AATGTAAATCTAGAGTTAAGTTGGAGAGTGCCGGTATTAAAC<br>CTATTCATGTCAAACATATGCGTCGGCGATCTCGAAATGTAG<br>CTTATTTTTCAATTTTCGATCTCTCATTTGTCTTTAGGGATATG

							AAAAAATCGTGTAAAATCTTGATATTCATTTGACAGTCATTTT<br>ATACGAAAGATGACTGAAGTGCGTATCAAATAACCACATGT<br>CAGACAATACTTTCAGGACAGTGTAAAGCAGTATTACGCAA<br>TTACTTGCAGACTGACAGTCATTTCTAAGACAGACATACTGT<br>TACACACATCTGCAAAGGACTACATAGTGCTACATTTAGTAA<br>ATTTCATCGTCTGGCGACATTTATTGTTCTTGCCGGTAGGTCT<br>CCTTTCGGAACAGGTGTCTCGTATCTTCTAATAAATGCGAGA<br>GCAGACGCTGCTTTCAAACGGCAGTCACCGGAAACCAAAAG<br>CACCAAGAGACATGGTGGAAAATTTTAAGTGGTAAGAACAT<br>TTGCATGCTTAGGTCTCGAGATCCTCACCGTGATCTGAAAGT<br>CTTATTTGAAAGACTGTTAAATGCCTCACCTTATTTTGTACAT<br>TATTTAAAGATACGACTAATAAAACAGGTTATAGTAGCAGTA<br>CGCGCAGTGAAATTCTGAAGGAATGGTTCCATCGTATGTACT<br>GCTGCGATAGGGCGCGAAACAATATAAGAAATCGACAGTTA<br>TTTAGGTTTTAGATTTTTTTTCAATAATTTAAGGTATACCTTTA<br>AAAACACGATTTCCTGTCAAAATATTAATTTTTCAATTTTGTT<br>CTAATATTAAAGCTTGTTCTTTTATCTCTCCTAATCGGTTTAAA<br>TTTTGCTTCCGTCTTATTTCGAATATGCGATAACAGCTATTTTA<br>TTGTTGATTATTCGTAGCAACATTTTAGAACTGGAAGTGAGG<br>TAATTACATCGTAATGTTGTTAAAAGCGAACGTCAAATTTGG<br>ACGATTTAATGTAAAGGAAAAGCAGAAATTAGTATTTTCCCT<br>TGTTTAATATATTTAGAAACATCCGATATCTCAAATTGAGTA<br>ATGAGAACTTCTCTCGCGCAGCATTTACAGATAATACGAAAG<br>GAAGCTTTCGTTTGCCTTGAAAACCGTGAAAATAGTGCATAG<br>GATGTTGCAAAACTATTAAGATGTATGGGCTTGAAGACTGTT<br>TAAATGCATCGAAAATACTGCAATATTCATGTTTCAGGGCTG<br>TAAACATTTAGATATGAATCGAATACAAGGACTGACACAGC<br>ATACCGCCTCTAATACAAAAAAAATTAGAAAAATTGGGACA

							GCCCTCAGGAAGGTGAAAAATTACCGTTTTAGACAGCAAAA<br>AGGGTATTTATCCTAAACTAGGGTCAATTTAAAGCTTAATTA<br>TTATTTTAAAACTAGGATTTGTGTTTAGATCTTGCTTTTTCTGC<br>TTTTTTCCCCCTCAAAATGAGATTTTTCATCTTCTGCCTTTACA<br>ACATCGTAACAAAAGAAAAAACTCATGCATGCAATACTTTA<br>AAACATTTTGGCAGTACAAAAGTTTTCCCAAGGGAATAGAC<br>CTTTCCCACGATTCTAGACGCATTAGTTTTTTTCCAATTTTTAT<br>TGAAAAAAAATTAATATATGAATTGTTGTAATTTTTATGAATT<br>TTCTCTTTCTCCGTACTTTAACCGTTATGGCTTATAAAGTAAC<br>AGCGCTATAATTACGGGAATGGTCTATTCCCCCAGAGGAAGT<br>TATTATTACAATAGAAAGTTTAAAATGAAAATATATAGATAT<br>GAATTTGAGTTATCGTGTTTTGCTCAGTAGTTAACTTTGGGCT<br>ATATTTTACTACATTTTTTGGCATGAAAATCTAAAAGAATAGT<br>GAAAACTATGCTCTTTGCTACATTTTTCCAATTTTTAGCCATA<br>TAGGATTATAAAATACAGCTAAGATAAATTTCAATGTTTAAA<br>AGCCATCTCTTAAGGTATACGTATATCTTCATAGAACCAAAC<br>AGGCCCGGCAAGGCCTTTCGGGGGGAAACCCCGCCGGTCAA<br>GAAACACACCGCAGTTGGCAGACGCTTAGATGGCACCCAGA<br>CGCCACATCCCAGGGCGGGCTGCACGAGGCTGGAGCCACAC<br>AAGCTCCGGTGCCGCAGACAGACAAGTTAATAGTTTCGAAC<br>AAATGGCGCGGGTCATGTACCAAAACGGAGAAACGACAGG<br>CAAGCAGGATTGTGAATAGAAATTCTAACACTGTGGCTTAAC<br>TATCTAACTTTCGTTCCAGGTTTTGCGCTATTCTATTGTTTGGT<br>CTCATAGTTGGAGATGCGTCAGGTAACATTTTACTTGTTTACA<br>GAATTATTGTTTTGTGTCCTTCATGATGCTAAGTTACTTACTTC<br>CTTTTATAAGGCAAAAAAGTGACCGTACGTAAGTGTGGCAG<br>GAGTTCGCACGGCCGGCCCAGGATAGTGGGTGGAGAACCGG<br>TCAAGGAAGGAACTTTTCCGTGGATAGTCACACTGCATTCAA

							AAGAGGACGAGGATTTTAAGCACTACTGCGGAGGCAGCATC<br>CTGAATGAACGGTGGATTCTGACAGCCGCGCATTGCTTAGAA<br>TTCCCAGTTCATCCCGAATTGTACGAGATCTACGTTGGATTGC<br>ATCGTTTATCGCGGAAAGCTGCAAAAAGTGTTAAGAGACAC<br>GGGATTTCTAAGATCATTCTACACGATGACTACTCTCCTTTCT<br>ATAAGAATGATATCGCCCTCCTAAAGACAGAGGATCCCATC<br>GACTTCAGTGGATCAGAAGGATTCGTGAACGGGATCTGCCTC<br>CCGGCGATGAACGAGAAAACACCGAGAAGACGTGCTATGAT<br>AGCGGGCTGGGGGTCAACCCAGTCTGGAGGAGAATACTCAG<br>ATGTGCTTCTTACCGTCCCTGTGCCTATGGTATCTGGAAAAAT<br>CTGCTACAAAGCGTATGAGGCATTTTTGACTGAAAATATGCT<br>ATGCGCTGGCAATGCGGGAATAGATTCATGTCAGGGTGATTC<br>CGGAGGACCTCTCGTCATGTGGAGGAATGGAAAAGCAATTT<br>TGATTGGAATCGTATCTTTTGGAATATCATGCGGCACAGGAA<br>CGCATCCCGGAGTCTATACCAAGTTAAAGCTTTTAATGGCAC<br>ACCGCCAGATTATACAAAACCTACTGATGTCCGAATCAAAG<br>ATGTTTTTGCAAGTCCTTCACTGTTTGGAATTGGGAGTCTGAA<br>TCATAAGGTCGTTTACCATACTACGATCTTGGATGCTATCGTC<br>TAAAACATGAAGATTGCCTGTAACACTATGTTCTGCATTTTTA<br>TCTACAAAATCTTTAAATATTTCATCGCAGCAGCGACAAAAA<br>ATCCATTCCTCTACTCATCTCTGCCCATGTCCGCTTTTTAAGCT<br>ATAATTAAAAATGACTCTATCCAAAGGGGTTATCAAAAGGG<br>ATATTTGAAAGTTTTTACACACACACCTTCCACTCGTCATTGG<br>ATGCCATTATCGACAGTTTTGTTGTAGTAAAATTTAAACTCAT<br>TCACTTAAATACAGCAATTTTCATTGGATGCAGTGGTGCAGC<br>CATGAAGCAGGGGGGGGGAGTTAAGGACAAACCTCTAGGA<br>GCTAGAAAATCTGTACAAAAGTCCTTTCCCTAAAGACAAATT

							TTTTTTTTCTTTCCCTAGAAAGGAATTCTCCATTTGTAGAGGAACCCCT

PhdEnzSeP<br>23	P84033	1,82E-51	42,308	PF00089	34951.65	MREQTLLSNGSHRK<br>PKAPRDMVENFKW<br>FCAILLFGLIVGDAS<br>GKKVTVRKCGRSSH<br>GRPRIVGGEPVKEGT<br>FPWIVTLHSKEDEDF<br>KHYCGGSILNERWIL<br>TAAHCLEFPVHPEL<br>YEIYVGLHRLSRKAA<br>KSVKRHGISKIILHD<br>DYSPFYKNDIALLKT<br>EDPIDFSGSEGFVNGI<br>CLPAMNEKTPRRRA<br>MIAGWGSTQSGGEY<br>SDVLLTVPVPMVSG<br>KICYKAYEAFLTEN<br>MLCAGNAGIDSCQG<br>DSGGPLVMWRNGK<br>AILIGIVSFGISCGTGT<br>HPGVYTKLKLLMAH<br>RQIIQNLLMSESKMF<br>LQVLHCLELGV*	CTCGGGTTTTCAGAGTGATGCGTTGAGGGCTTCTTGACAGATT<br>GATGAAGTTGTCTAGGGGCCTGGGTGTCCCACTGCTTTTGGG<br>TCTATAGTGTATGTGCATATTATACCTATTTTTTTCTGGAAAC<br>GCGCGAAATTGACGAGTTTTCATAAAACTGTGGAATCACCGA<br>TAATGATGCTGCAGATGGATCTCCCAAAAGCGGTTCGTCCGT<br>CACCCAGTTATTAAATTGATAGGTACCCTATTTTTCAGCAAA<br>GATACACATATGTAAAACTGTGAAAATGGTATCTTGTCAGTA<br>ATCTGGCCGCAGAAACGGGGATAAATTCTGGTTTAGTCTCCC<br>GCAATATGTTGGAACCCGTCAAATTGACGGGTATTCATAAAA<br>TTGTGGAATCCCCGATAATGATGCTGCAGATGGATTAGTCAA<br>AAGTGGTTCGTCCATCACGCAGTTATTAAAGAGATCGGTACC<br>CTATCATTCAATAAAGCTACACGTATGTAAAACTATGAAAAT<br>GGCATCTTGTCACAAATTGGTCTGCAGAAACAGCGATAAAC<br>CCTGGTTCAGTTTGCCGCGACATGTTCAGGAAGCTTCCGGAA<br>GCGAAGCTGTTGCCTCATTTCGTTCAGCCGTGGGTTCGTTTCG<br>TTAGCCACTTGCCCAGAGTCACGTAATGTACACAACGGTGAA<br>TCTGTCAAGTTAAAACGGTAGAAACAGGGCAGACAGACAGT<br>TATGCTCTGTCGGATGTCTAACACTGGCAAGAGCCTGAAGTC<br>TCGGAGAGTACGGGACGAGATTAACGCCGATGAAGGTTAAT<br>CTCGGCTGCTCCACCTCTTACCTTCCGCAACTCGATGAAATG<br>GTAATTTCCTTTAAGAGTCCGTCTTTTGGTCAAGAAGAATTTT<br>TATGCAACGAAAGAACAGCGATCTTCTGGGTCTACAGATAAT<br>AGCTGTACCCCAAATATGCAGTGCGCGAAGAGACAGATTCT<br>TGTAGATGGCCTCTTCTCTCCAGTTTTCAAACTAACATCTTGA<br>ACATAGCATCCATAAATGCGTAGTATTACATCACTAGAGCAA<br>TATAAAGAAATTATTTTTCGATAGGCTGACCAACTGGCCAGT<br>GATTTTAAGAACGCAAGACGAGTTTCGATCCCCGTTACCGGC<br>CATACGAAATAATTCTAAAATAAGCAAACCTTGTCGAATCG

							GATATTGTGAAAGGAAATATAGATCTAATAATATTTGTGCAA<br>GGAGTGTGTTTGCGAAAAATGTACAGGCATGAGGGATAAGC<br>TGCAAAATGACTTCTTAACCATCCACAATTATGTAGAAGAGC<br>TGCTACTAGTTTCGGATTAACCGATGCAGTCCTTCGTCAAGG<br>CGAGGTGCATTTGATGTGTACGTCAGTGACGACGAGGCGCA<br>AATGCAGGGAGCATAATTGCTTTGATATGTATAAAGAGGATT<br>AAAATAATTGCCAGAAAAAGGGAGGCGGGTGATACGTCAG<br>GAAAGGGTGGGGAAAAGTGGGCAATACACCCCCTACTTTGA<br>TACATATACAGAGACTTGAGGAGATTATAAGAAAAAGTGCT<br>GATACGTCACAAATGGGGTGGGGCAACACGGGAAATAAACC<br>TGCCAACGCATGGGAGATGCATATAGACAAAGCAGTAAGAT<br>ATAGGAGGAATAAATAAAGTGAGAAATGAGTATAACGATTA<br>AGAAAAAACGATTTTTCAGGCAGAAGGGAAAGATAAATTAG<br>AAAGATCATTAACAAGCGGAATAGAGTTGAGATAAATCGAA<br>AGGTTTTCTGGAAAATCCAATCCCCTTTCTCGCCCTTTTGGAA<br>TGTATCCTCTTTTTCTCGTAACATCTTTAATTATCTTTATGGGT<br>ATCAAAGTAGTTATGTTCTTTCGTTTCTGTTTCGTCTTCATTGA<br>TGTACACATATAATGCGACTAAGGGCTACATTAGTCCGAAGC<br>TAGGTGCAGCTCTTCTGCATAATTGTGGTTATTAATAGGTAAA<br>TTTGAATCGTATCTGTTCTTCATAAGTTTCCTGCGAATATGAG<br>GTACAATGCAATTTACGTGCAAGAAATGTACTATACAAATGT<br>ATGTTTATAAATCTATCTTTGTAAATTAATGTTAAAATAAAAA<br>TTGCTGGATAAAGTTTTTAATAACGAACCAATGATTTAAAAT<br>GAGATGTACTATTATAAAAAAAAAACTTCGACCGTTTAATGTA<br>CCTGTCAATATGGCGGTTTTCGGGGAAAACAGGTATGCAGTC<br>AATGTAAATCTAGAGTTAAGTTGGAGAGTGCCGGTATTAAAC<br>CTATTCATGTCAAACATATGCGTCGGCGATCTCGAAATGTAG<br>CTTATTTTTCAATTTTCGATCTCTCATTTGTCTTTAGGGATATG

							AAAAAATCGTGTAAAATCTTGATATTCATTTGACAGTCATTTTATACGAAAGATGACTGAAGTGCGTATCAAATAACCACATGTCAGACAATACTTTCAGGACAGTGTAAAGCAGTATTACGCAATTACTTGCAGACTGACAGTCATTTCTAAGACAGACATACTGTTACACACATCTGCAAAGGACTACATAGTGCTACATTTAGTAAATTTCATCGTCTGGCGACATTTATTGTTCTTGCCGGTAGGTCTCCTTTCGGAACAGGTGTCTCGTATCTTCTAATAAATGCGAGAGCAGACGCTGCTTTCAAACGGCAGTCACCGGAAACCAAAAGCACCAAGAGACATGGTGGAAAATTTTAAGTGGTTTTGCGCTATTCTATTGTTTGGTCTCATAGTTGGAGATGCGTCAGGCAAAAAAGTGACCGTACGTAAGTGTGGCAGGAGTTCGCACGGCCGGCCCAGGATAGTGGGTGGAGAACCGGTCAAGGAAGGAACTTTTCCGTGGATAGTCACACTGCATTCAAAAGAGGACGAGGATTTTAAGCACTACTGCGGAGGCAGCATCCTGAATGAACGGTGGATTCTGACAGCCGCGCATTGCTTAGAATTCCCAGTTCATCCCGAATTGTACGAGATCTACGTTGGATTGCATCGTTTATCGCGGAAAGCTGCAAAAAGTGTTAAGAGACACGGGATTTCTAAGATCATTCTACACGATGACTACTCTCCTTTCTATAAGAATGATATCGCCCTCCTAAAGACAGAGGATCCCATCGACTTCAGTGGATCAGAAGGATTCGTGAACGGGATCTGCCTCCCGGCGATGAACGAGAAAACACCGAGAAGACGTGCTATGATAGCGGGCTGGGGGTCAACCCAGTCTGGAGGAGAATACTCAGATGTGCTTCTTACCGTCCCTGTGCCTATGGTATCTGGAAAAATCTGCTACAAAGCGTATGAGGCATTTTTGACTGAAAATATGCTATGCGCTGGCAATGCGGGAATAGATTCATGTCAGGGTGATTCCGGAGGACCTCTCGTCATGTGGAGGAATGGAAAAGCAATTTTGATTGGAATCGTATCTTTTGGAATATCATGCGGCACAGGAACGCATCCCGGAGTCTATACCAAGTTAAAGCTTTTAATGGCACACCGCCAGATTATACA

							AAACCTACTGATGTCCGAATCAAAGATGTTTTTGCAAGTCCT TCACTGTTTGGAATTGGGAGTCTGAATCATAAGGTCGTTTACC ATACTACGATCTTGGATGCTATCGTCTAAAACATGAAGATTG CCTGTAACACTATGTTCTGCATTTTTATCTACAAAATCTTTAA ATATTTCATCGCAGCAGCGACAAAAAATCCATTCCTCTACTC ATCTCTGCCCATGTCCGCTTTTTAAGCTATAATTAAAAATGAC TCTATCCAAAGGGGTTATCAAAAGGGATATTTGAAAGTTTTT ACACACACACCTTCCACTCGTCATTGGATGCCATTATCGACA GTTTTGTTGTAGTAAAATTTAAACTCATTCACTTAAATACAGC AATTTTCATTGGATGCAGTGGTGCAGCCATGAAGCAGGGGG GGGGAGTTAAGGACAAACCTCTAGGAGCTAGAAAATCTGTA CAAAAGTCCTTTCCCTAAAGACAAATTTTTTTTTCTTTCCCTA GAAAGGAATTCTCCATTTGTAGAGGAACCCCT

PhdEnzSeP 24	P84033	8,93E-55	42,387	PF00089	32825.83	MREQTLLSNGSHRK<br>PKAPRDMVENFKW<br>FCAILLFGLIVGDAS<br>GKKVTVRKCGRSSH<br>GRPRIVGGEPVKEGT<br>FPWIVTLHSKEDEDF<br>KHYCGGSILNERWIL<br>TAAHCLEFPVHPEL<br>YEIYVGLHRLSRKAA<br>KSVKRHGISKIILHD<br>DYSPFYKNDIALLKT<br>EDPIDFSGSEGFVNGI<br>CLPAMNEKTPRRRA<br>MIAGWGSTQSGGEY<br>SDVLLTVPVPMVSG<br>KICYKAYEAFLTEN<br>MLCAGNAGIDSCQG<br>DSGGPLVMWRNGK<br>AILIGIVSFGISCGTGT<br>HPGVYTKVSSYIAWI<br>EKTINSN*	CTCGGGTTTTCAGAGTGATGCGTTGAGGGCTTCTTGACAGATT<br>GATGAAGTTGTCTAGGGGCCTGGGTGTCCCACTGCTTTTGGG<br>TCTATAGTGTATGTGCATATTATACCTATTTTTTTCTGGAAAC<br>GCGCGAAATTGACGAGTTTTCATAAAACTGTGGAATCACCGA<br>TAATGATGCTGCAGATGGATCTCCCAAAAGCGGTTCGTCCGT<br>CACCCAGTTATTAAATTGATAGGTACCCTATTTTTCAGCAAA<br>GATACACATATGTAAAACTGTGAAAATGGTATCTTGTCAGTA<br>ATCTGGCCGCAGAAACGGGGATAAATTCTGGTTTAGTCTCCC<br>GCAATATGTTGGAACCCGTCAAATTGACGGGTATTCATAAAA<br>TTGTGGAATCCCCGATAATGATGCTGCAGATGGATTAGTCAA<br>AAGTGGTTCGTCCATCACGCAGTTATTAAAGAGATCGGTACC<br>CTATCATTCAATAAAGCTACACGTATGTAAAACTATGAAAAT<br>GGCATCTTGTCACAAATTGGTCTGCAGAAACAGCGATAAAC<br>CCTGGTTCAGTTTGCCGCGACATGTTCAGGAAGCTTCCGGAA<br>GCGAAGCTGTTGCCTCATTTCGTTCAGCCGTGGGTTCGTTTCG<br>TTAGCCACTTGCCCAGAGTCACGTAATGTACACAACGGTGAA<br>TCTGTCAAGTTAAAACGGTAGAAACAGGGCAGACAGACAGT<br>TATGCTCTGTCGGATGTCTAACACTGGCAAGAGCCTGAAGTC<br>TCGGAGAGTACGGGACGAGATTAACGCCGATGAAGGTTAAT<br>CTCGGCTGCTCCACCTCTTACCTTCCGCAACTCGATGAAATG<br>GTAATTTCCTTTAAGAGTCCGTCTTTTGGTCAAGAAGAATTTT<br>TATGCAACGAAAGAACAGCGATCTTCTGGGTCTACAGATAAT<br>AGCTGTACCCCAAATATGCAGTGCGCGAAGAGACAGATTCT<br>TGTAGATGGCCTCTTCTCTCCAGTTTTCAAACTAACATCTTGA<br>ACATAGCATCCATAAATGCGTAGTATTACATCACTAGAGCAA<br>TATAAAGAAATTATTTTTCGATAGGCTGACCAACTGGCCAGT<br>GATTTTAAGAACGCAAGACGAGTTTCGATCCCCGTTACCGGC<br>CATACGAAATAATTCTAAAATAAGCAAACCTTGTCGAATCG

							GATATTGTGAAAGGAAATATAGATCTAATAATATTTGTGCAA<br>GGAGTGTGTTTGCGAAAAATGTACAGGCATGAGGGATAAGC<br>TGCAAAATGACTTCTTAACCATCCACAATTATGTAGAAGAGC<br>TGCTACTAGTTTCGGATTAACCGATGCAGTCCTTCGTCAAGG<br>CGAGGTGCATTTGATGTGTACGTCAGTGACGACGAGGCGCA<br>AATGCAGGGAGCATAATTGCTTTGATATGTATAAAGAGGATT<br>AAAATAATTGCCAGAAAAAGGGAGGCGGGTGATACGTCAG<br>GAAAGGGTGGGGAAAAGTGGGCAATACACCCCCTACTTTGA<br>TACATATACAGAGACTTGAGGAGATTATAAGAAAAAGTGCT<br>GATACGTCACAAATGGGGTGGGGCAACACGGGAAATAAACC<br>TGCCAACGCATGGGAGATGCATATAGACAAAGCAGTAAGAT<br>ATAGGAGGAATAAATAAAGTGAGAAATGAGTATAACGATTA<br>AGAAAAAACGATTTTTCAGGCAGAAGGGAAAGATAAATTAG<br>AAAGATCATTAACAAGCGGAATAGAGTTGAGATAAATCGAA<br>AGGTTTTCTGGAAAATCCAATCCCCTTTCTCGCCCTTTTGGAA<br>TGTATCCTCTTTTTCTCGTAACATCTTTAATTATCTTTATGGGT<br>ATCAAAGTAGTTATGTTCTTTCGTTTCTGTTTCGTCTTCATTGA<br>TGTACACATATAATGCGACTAAGGGCTACATTAGTCCGAAGC<br>TAGGTGCAGCTCTTCTGCATAATTGTGGTTATTAATAGGTAAA<br>TTTGAATCGTATCTGTTCTTCATAAGTTTCCTGCGAATATGAG<br>GTACAATGCAATTTACGTGCAAGAAATGTACTATACAAATGT<br>ATGTTTATAAATCTATCTTTGTAAATTAATGTTAAAATAAAAA<br>TTGCTGGATAAAGTTTTTAATAACGAACCAATGATTTAAAAT<br>GAGATGTACTATTATAAAAAAAAACTTCGACCGTTTAATGTA<br>CCTGTCAATATGGCGGTTTTCGGGGAAAACAGGTATGCAGTC<br>AATGTAAATCTAGAGTTAAGTTGGAGAGTGCCGGTATTAAAC<br>CTATTCATGTCAAACATATGCGTCGGCGATCTCGAAATGTAG<br>CTTATTTTTCAATTTTCGATCTCTCATTTGTCTTTAGGGATATG

							AAAAAATCGTGTAAAATCTTGATATTCATTTGACAGTCATTTT<br>ATACGAAAGATGACTGAAGTGCGTATCAAATAACCACATGT<br>CAGACAATACTTTCAGGACAGTGTAAAGCAGTATTACGCAA<br>TTACTTGCAGACTGACAGTCATTTCTAAGACAGACATACTGT<br>TACACACATCTGCAAAGGACTACATAGTGCTACATTTAGTAA<br>ATTTCATCGTCTGGCGACATTTATTGTTCTTGCCGGTAGGTCT<br>CCTTTCGGAACAGGTGTCTCGTATCTTCTAATAAATGCGAGA<br>GCAGACGCTGCTTTCAAACGGCAGTCACCGGAAACCAAAAG<br>CACCAAGAGACATGGTGGAAAATTTTAAGTGGTTTTGCGCTA<br>TTCTATTGTTTGGTCTCATAGTTGGAGATGCGTCAGGCAAAA<br>AAGTGACCGTACGTAAGTGTGGCAGGAGTTCGCACGGCCGG<br>CCCAGGATAGTGGGTGGAGAACCGGTCAAGGAAGGAACTTT<br>TCCGTGGATAGTCACACTGCATTCAAAAGAGGACGAGGATTT<br>TAAGCACTACTGCGGAGGCAGCATCCTGAATGAACGGTGGA<br>TTCTGACAGCCGCGCATTGCTTAGAATTCCCAGTTCATCCCG<br>AATTGTACGAGATCTACGTTGGATTGCATCGTTTATCGCGGA<br>AAGCTGCAAAAAGTGTTAAGAGACACGGGATTTCTAAGATC<br>ATTCTACACGATGACTACTCTCCTTTCTATAAGAATGATATCG<br>CCCTCCTAAAGACAGAGGATCCCATCGACTTCAGTGGATCAG<br>AAGGATTCGTGAACGGGATCTGCCTCCCGGCGATGAACGAG<br>AAAACACCGAGAAGACGTGCTATGATAGCGGGCTGGGGGTC<br>AACCCAGTCTGGAGGAGAATACTCAGATGTGCTTCTTACCGT<br>CCCTGTGCCTATGGTATCTGGAAAAATCTGCTACAAAGCGTA<br>TGAGGCATTTTTGACTGAAAATATGCTATGCGCTGGCAATGC<br>GGGAATAGATTCATGTCAGGGTGATTCCGGAGGACCTCTCGT<br>CATGTGGAGGAATGGAAAAGCAATTTTGATTGGAATCGTATC<br>TTTTGGAATATCATGCGGCACAGGAACGCATCCCGGAGTCTA<br>TACCAAGGTATCTTCATACATAGCCTGGATCGAAAAGACGAT

							AAATTCTAATTAGTCCTAATCTGTGATCTCTGCTGTTCTTCAT AATTGTGGATATTATCTTTTGCATTTTAATGTTCATGTGCTATT AATAAAAATGATATTCAACCGGAAAAAGCGTGTGTCATCTGT AAGATGTTCGAAATGTATTCTTGAAGAAAACCGGTCACTTGC CTGACCGGTAGCGTTATGTAAACCCGCCCACCCCGACAAACT TTTTATCGTTTCATATATACGATACTATAAACTTCCTATACCTT ATCATTTAAAAGCTTTTGAAGTGTAAAGAAAGCAAGACAAT AATAAGCCAAACGATGTGTTAAATAGAAAAGTGCAATAACT ACAAACACAAAATGATATACACTAATGCAGATTAAAGATAT TATAGAACAAAATCAAATAGAATCGTGATAAAAATCATTTT AAATGAGTGTCGGGGTGGAGGTTGTAACCCAAATGACGGTA GCCACAGCTATGCCCAGTTAAACAAGCTTTACGTGTAGACAA CACACCTGATATGCTGTATGATCCCAACGTAAGGAGTTTTTTT AAGCACGAACTAGCAAAATAACGAAAGCGGGAAAATGGAA CAAAATAACACAGTATCTTGGATTAGAACGGAAAGCTAAAT GAGGAAAAAGACAATGACATATAACACAAGCTAAACAATG CAGTAGTAATAAAACAGAAACTAAAACCTACTAAACCAACC TAAGAATTAAAGAACTACCTATAGTAATTAAATATAACTAA AGAAATAATCTAGTGACCTGACATTCTAAATTATATGTCGAC ATACCAAAAATTCCACTGCCTACTCGCCGCTATAAACTAGAT CACTCTTGGAATTAAAACTAACGGTTCATTCTAACTAACAGC ACATTCCCAGCTTACAGTACATATTCCTTTACATAGGAACAA AATCTCCATCCTTAAAAGATCAACTCAATTCACTTCCTCCTTA AAATAAAATCAAATTAATTGAAACAAAATTAACTACAGTAC AAATTTGACTGGTACACACAATATAACTGGTAAACTTCCAAA CGTGAGGTGTCTACTGGATGGCTAACCAGTTGAAAAGTTCTT TCCATATAAATGGAATACATCAAACATCTCACGCCTGTATTT ATGAATCTATCCCCAAGCAAGAAACACAATCAGCTAAAAAA

							TGTTAACCTGTGTCTAAATTTGTTATTTCAGCAACGGAAACTA<br>TAGGGTCATTAATATCTAGAACACAATCATCAGAGGAAACA<br>TATTTCATTCTATATAATTCCCCTTCAAAATTATATCTTTGAA<br>AGTAGAACTGGGAATATTAGATTTAAAATGCTGATATTTACA<br>CGAATGAGAATAAAACGAAAATTATGGTCATGTCCTTTGTAA<br>TAAATATTAAAATGCAAAATCCCCCGTCTTGTACGTGAATAT<br>TTATATCCAAAATGTTAGCAACTGAATTGCCATTGAGTATCT<br>GTATGTTTCAATTTTAAATTTTTAGGTTAAAATCCTAAATCCA<br>GATCGTAATGCTTATCTTTACAAAAAGTAAAAAAAAATAAA<br>AAGACAACTAAACCAATGATGTATATATAAAATAAGAAGTT<br>CTTTACACTGCTAATTTTAGATTCATAAATGTGCAAGTATATT<br>TACTAATATTTTCCAATGCCAGGCGATTGGTAATTACGCCATT<br>CGCCCCTTCAAGCTTCCCAATACTTTTTATGGGAGGAATTTAA<br>AAAAAATGAATACAGAGTACGAATCATGGCGAGTACTGTTC<br>ATCGGTTGTCCAGAGTCACAAACAGCAAACGGTTTGATACG<br>AAATAAATTCGCACCAAGACAGTCACGCCCCCGTCAGGAGG<br>CGAAATGCAACCACAGCCGATTTACGAGGGAGAGACTTGAA<br>ATAGAAAACTTGGGATCGAAAAGATAGTCTCATATATTTCCC<br>GAAGCCTGATTGCTTATCAAGGAAAACTAGTTTCTCTTAAAC<br>TCGCCTTTTTTTTTTTTTAAATTTAGCAGAATGATAAGTTAAA<br>GCATTTTTTTGGCTTTTGGAGAACAGCGATACCTTTCTTGGCG<br>AGGTGTCAACTTCTCGTTACCCCCTAGGCCACAATAATGAGC<br>GAGGACCCAATGAAGAGTATTCGAATTACGTAGGGAGTGTA<br>GCGAGCGACTGTAGTATGTGCAGGTATATATTTACTAAATAT<br>TGACGAAATCTTAATCCCCCCCCCCTCTGTCATAGCAATACC<br>TTCGATTTGTTTCACCACGCTATCATTATGCAAAACACAATTA<br>TCTACTACAACTAAAATTGAGCAATCTGAAATATCATTTGAA<br>TTCCACAACATTTTCCAATTGGTACATACATTTCCTTCCTTACT

							AGCCTTACTATTAATAAATCTTCAATTTCAATTGAATAAAACGTCTTCGAATGTGTGTATACAACTAACCTCAAAATCCAGATTATATATCTCGCTGCTTATTACTTATGAATCTACTCTTTTCTCTATGTTTTATTTTATAGAGAATGCAATTCAAATATTTCGTCAATTCCCTGCCAAACGTATTCATGTTTAAGTATTTACACTACATGTTGTTTTTCTAAAATTAATTGGATCTTTGCGAAATTTAAGTGCAGCTATGATACACGGAAATCTTATGTTGTTAATTTCATATAACTGATCAGTTTGCGTGTGG

PhdEnzSeP 25	Q7Z269	5,56E-07	28,070	PF00089	70689.33<br><br>**68823.04**	MLTYTLVLAALSLSA<br>VFA**IKSKTCGPYPY**<br>**VELHHGTVRRTGR**<br>**FKYTFRCDEGYTLLF**<br>**SRSATVKCHRGEW**<br>**KPSGEPRCYGGEGR**<br>**CDEPPAVANAKVF**<br>**GDERHPGASVKYV**<br>**CHEGYTLLGNSELS**<br>**CARTGHWDRRAPT**<br>**CMDESEPVQNVAE**<br>**RLSNSFVTSMANYS**<br>**SDTPEGRLLDADSL**<br>**HLGLELYIVIDRSSS**<br>**VDPVQLEEAKRFIK**<br>**FLLQRFSVRNGMD**<br>**NPRGTRSSVIAFGT**<br>**DVQTIFSLQDEHIS**<br>**NPTLAAQAIDAIEP**<br>**NGGGTNLEGALTL**<br>**PILDSHNELLRPDA**<br>**KRALFLMTDGEPNI**<br>**ANPDITPQDIAQTL**<br>**RQQHDFEIFTVGIG**<br>**KGINMHLLNDLAS**<br>**EPPLSHVFMLEDYA**<br>**NLNDIMKIIQNPKP**<br>**KPPPISKDQCGYNA**	TCTATATATTGTTGAAATGAGTTTTTCTCTTTTGTCTTGATTGT<br>CCCTTTACATTGATATGAAAGGAAAATTTATGAGGAACTTTG<br>AAAAAAGGTTATTTGTACTTGTCGTTGTATTGATATGTTCTTC<br>TGTATTTCAAAGCAGCAAATTTTTTATTTATAACTTGACGGAA<br>GTTAATGTAGACTATTTAATACAAATATATTTTCAATGTGAA<br>ATCCAAGTACAATGGATAGCGGAAGGAAATGCATTATATAC<br>ATTTAAAAAAATATATTTCATTGAAATTACCAACACAAAAAG<br>AAGTCTGAAGAGTGTTCCTTAATCATGTTCGATAAAATGCAT<br>TGCTGAGTGAATGCCTCGAAGACATGCCTTTTTTCACGATTTC<br>ATTTCAGAGGGATTTTAATTCCTTAAGTTTCTCTCGATGAAGT<br>TGAAAGATTTTCCTTTGAAGCTTAAATATTTTGTTTTATCCAA<br>TATGATTATTTAAATATTTGCTATTAATCAAGGACTATTATCG<br>ACAAATACAGAGAATTCTTGTGAAGGAATGAAATATTTAAG<br>CGTATTTCCTCATGAAAAGGTAGTGTTTGCATCGCTACACTAT<br>TTAATGCAAGTTGTCGATTTACTTCCTGACATATATCGTGGAT<br>TTCGACGTTGTTACAACATAAAAACTTACAGTGTGTCTAAAA<br>AAAGTTACCAAGAGAGATACCTAAAGGACTAGTAAGAGCTA<br>TCGTTTTTTTTAATGCAGATGTATCGGAAAGCAGTATGGCGCT<br>AAATGGCGGAACAGCTTTTGGGATAAGCTCCCTCTTTCTGTG<br>GGAACATTCATTATTTGGAGACAGACAGAAAACGTCCATTA<br>CACAATGGTAGAGCATTGATAAATTAGTTCTAAATAATTTTG<br>GTCATACAGAAAAAAATCGAAAAATAAGTGCAGAAATTATAA<br>CTTTATAGTCGCAAGATTTTTTTTGTAATCAAAATCATCTGCC<br>GGGAAAATCACCTGCCAATACCTTTAACATAAACGATTGCG<br>AATGTGAGTCTTAATGAGAAATGCTTTGTTTAACAGACGATT<br>TGTCAATAAGTATCATCGTAATCGAAGCCAGAAAAATAAAC<br>TAATCTGAAGTGAAAGAAGATTCTATCTGAGACTATAGTTTT<br>AGCTGACTCTTTAATTCCACTCACTTTGATTTCGATTTCATGA

						**TDENQPWIVVLYIG<br>GTPFKICEGVLICSQ<br>WVLTAASCLQEGH<br>DHVNMQDVFVVL<br>GERHLLKKNKRQT<br>NFYVVDMRIHPRY<br>DPDQLIRNDLALLK<br>IYVPEDKESQYRPA<br>CLPLTDRVVPMHL<br>NLEINATISGWGST<br>SASSTTWARADDL<br>NHFKMFTTPVSLSI<br>DDECPVKKSGLSLL<br>CAGRGSRSCYGLP<br>GSPLMAADDTTGF<br>HHALGILSHRRKCV<br>QGQNQYVELTKHV<br>QWINYETKACQLK<br>HWGFNI***	ACGATGTGTCTGCGATTTCGTTCCCTTCAGGGCAGTTATAATG<br>CATCCCCCGGTTTTTAGGGTCATCTCACCCTTCCTGAAATAGG<br>GACAACTCCCCGAGAGTTGTCTCATCGTTTAGCGCCATACTC<br>CTTTTTTATAGACCTATAATAAAAATATCGCTACCGCTAGGA<br>GTTTTCGAGAAATTTCTCTTGTTAAGTCTAGTCGTTTCCTAGA<br>CACCTTGTAAATATCTTAGTATTTTCTCTCTTAATTATTACTGC<br>ATATTAATACGGTTAAACTTTTATGAAATCATATAATTGCCCC<br>AGAAGGCCCGTCGGATCACAAAAGGGGCGAAGGGTTATCTA<br>CTAAATGATTTTACTTTAAGCGTTATCGATTGAGTCTCTTTAG<br>TTTTCCATAATAGTTTTGTACAGCTGTCTTCATATGTTGAATCC<br>CCAATGCTTCAGCTGACAGGCTTTTGTTTCGTAATTTATCCAC<br>TGTACATGTTTGGTGAGTTCGACGTACTGATTTTGACCTTGTA<br>CGCATTTCCTGCGATGAGAAAGAATCCCAAGGGCGTGGTGG<br>AAGCCAGTAGTATCGTCTGCAGCCATTAGGGGTGAGCCGGG<br>CAATCCGTAGCAAGACCTCGACCCTCGTCCTGCGCAGAGAA<br>GAGAGAGACCGCTTTTTTTAACCGGGCACTCGTCATCTATGG<br>AGAGTGAAACGGGAGTAGTGAACATTTTGAAGTGGTTGAGG<br>TCGTCTGCCCTGGCCCAAGTAGTCGAAGAGGCCGAAGTACTT<br>CCCCAGCCCGAGATGGTGGCATTGATCTCCAGATTCAAATGC<br>ATCGGGACAACTCGATCTGTGAGCGGAAGGCATGCCGGCCT<br>GTACTGGGATTCCTTGTCCTCCGGGACATAGATCTTGAGGAG<br>CGCTAGATCGTTTCTGATCAGCTGATCTGGGTCGTAACGAGG<br>GTGGATTCTCATGTCCACCACGTAGAAGTTGGTTTGACGTTTA<br>TTTTTCTTTAGTAAATGTCTCTCACCAAGTACCACGAACACGT<br>CCTGCATGTTCACGTGATCGTGGCCTTCCTGCAGACAGGACG<br>CTGCCGTCAACACCCACTGGCTGCAGATGAGGACGCCCTCG<br>CAGATCTTGAAGGGAGTACCTCCGATATAGAGGACGACAAT<br>CCATGGCTGGTTTTCGTCGGTGGCGTTGTAGCCACACTGATCT

							TTGCTGATGGGCGGAGGTTTGGGTTTGGGGTTCTGGATAATCT<br>TCATAATATCATTGAGATTGGCGTAGTCCTCCAGCATGAACA<br>CGTGACTGAGGGGTGGCTCGCTCGCCAGATCGTTGAGGAGGT<br>GCATGTTGATACCTTTGCCGATACCCACAGTGAAGATCTCGA<br>AATCATGCTGTTGTCTGAGTGTCTGTGCGATGTCTTGGGGCGT<br>TATATCCGGGTTAGCAATATTCGGTTCCCCATCCGTCATCAG<br>GAAAAGGGCTCGCTTCGCGTCTGGTCTCAGCAACTCATTGTG<br>ACTGTCCAGAATAGGCAAAGTGAGAGCGCCTTCCAAGTTGG<br>TACCTCCACCGTTAGGCTCTATGGCGTCGATGGCCTGCGCAG<br>CTAGTGTGGGGTTGGATATGTGTTCATCTTGAAGACTGAAGA<br>TCGTCTGCACATCCGTTCCAAATGCTATTACCGAGGAACGTG<br>TTCCTCTTGGATTGTCCATTCCGTTCCTGACACTGAATCTTTGG<br>AGTAGGAATTTTATGAATCTCTTGGCCTCTTCCAATTGAACAG<br>GATCGACACTGCTTGACCTGTCGATCACTATATACAGCTCAA<br>GGCCTAAATGCAAGGAATCTGCGTCCAACAAACGTCCCTCTG<br>GTGTGTCTGACGAATAATTTGCCATAGATGTGACGAAGCTGT<br>TACTAAGTCTTTCGGCAACATTCTGAACTGGTTCAGATTCATC<br>CATGCAGGTAGGTGCCCGCCGGTCCCAATGTCCGGTTCGAGC<br>GCAGGAAAGTTCTGAGTTTCCGAGAAGTGTGTAGCCCTCGTG<br>GCACACGTATTTCACGCTAGCACCTGGGTGTCTCTCATCGCC<br>GAAGACCTTTGCATTGGCTACGGCGGGTGGTTCGTCGCACCG<br>TCCCTCTCCGCCATAGCATCTGGGTTCTCCGGAAGGTTTCCAT<br>TCTCCGCGGTGGCATTTAACGGTTGCACTCCTCGAAAACAGC<br>AGCGTGTATCCTTCATCGCAGCGGAAGGTGTACTTAAAGCGC<br>CCGGTGCGGCGCACAGTGCCATGATGGAGCTCCACGTAGGG<br>ATACGGCCCACAGGTTTTTGATTTGATGGCGAAGACGGCAGA<br>AAGCGAGAGGGCTGCCAGGACTAGAGTGTAAGTCAACATGT<br>TGCACAAAGAAAGAAGTAAACTGGAGTATGAAATTATTTCTT

							ACGATATGACCAGCGTCCAATACCGACAGCGATTGCATTTCC TGCTTCAG

PhdEnzSeP 26	P84033	7,63E-24	45,614	PF00089	11286.74	ADRIDFGGSEGFVN GICLPTQNEREPSSV AAVAGWGHTTKGG DTSDVLRAVQVPIIS RELCKAAYSSLHLPI TENMLCAGGMGLD ACQFDSGGPLVMW KDGRATLFGS	AGCCGATCGCATCGACTTCGGTGGATCGGAAGGATTCGTGA ATGGGATCTGTCTTCCGACGCAAAACGAGAGGGAACCCAGT TCCGTGGCTGCGGTAGCTGGGTGGGGACACACCACTAAAGG TGGCGACACCTCCGATGTGCTCCGCGCAGTCCAAGTACCGAT AATTTCCAGAGAATTATGCAAAGCAGCGTACAGCTCTCTGCA CCTACCCATCACTGAAAATATGTTATGCGCTGGCGGTATGGG ACTCGATGCCTGTCAGTTTGATTCCGGAGGACCTCTCGTTATG TGGAAAGATGGAAGGGCAACTTTGTTTGGAAGTG
PhdEnzSeP 27	P84033	2,45E-14	45,946	PF00089	7485.58	EARGGDTSDVLRAV QVPIISRELCKAAYSS LHLPITENMLCAGG MGLDACQFDSGGPL VMWKDGRATLFGS	GGACTGCATCGTCTGTCAAGGAAGGCGGCAAAGCAAGTTAA GAGGCACGAGGTGGCGACACCTCCGATGTGCTCCGCGCAGT CCAAGTACCGATAATTTCCAGAGAATTATGCAAAGCAGCGT ACAGCTCTCTGCACCTACCCATCACTGAAAATATGTTATGCG CTGGCGGTATGGGACTCGATGCCTGTCAGTTTGATTCCGGAG GACCTCTCGTTATGTGGAAAGATGGAAGGGCAACTTTGTTTG GAAGTG

PhdEnzSeP28	P84033	1,08E-10	46,269	PF00089	7956,23	LSLSLSLLGGDTSDVLRAVQVPIISRELCKAAYSSLHLPITENMLCAGGMGLDACQFDSGGPLVMWKDGRATLFGS	TGTGTCCGACAGCCCGCCAGCGCTCAGAATCCTCCATTCGTCTTCTGGAAGCCATAAAACACGTCTAGCTTGAAAACAAATCGTACGATGCCGGCTTCTTATACTGCCTGTTTTGCATATTTCTCGACGCGAAACCGTGATTGGCTGTTACCTAACATATTTGCATAATTCCTTCTACCTTTGCAGCAAATTTCAGCTTCTTTAATTCCGATTGGCTTACGGAAATTAACATTTTCCTCTCCGCGCTTCACATTTGGAAGTGAGTGTAAATTAAGTAATATATTATCTGTTTATTTAAAATCAGTATTTTGTTACATTTTTTAATGTTCTTCTTGTTGTTAAATTTATTTCTATAATGAGGTATTTATTGTTTTTTTTTTTTGAGAAAAAATACGAATGCTGTTCAAAAAGACTGTGTAAGTTGTTGTATATCTTCCCCATATGTTCTGAGGAATACCAATGAATTCAATCTTAAATCCCATCAGGGGATACGTAAAGGGCTTCCGTGCTAGGCAAGAGATTAAGAACCACTGATTTATACGAATGATATTTTTAGTCGATACTGGTTCCAGGGAAATTTATCCCAGGAGGGAGCATTTTATCTAAGAACAGTGGGAGAATAGAGGAAATTCCTAACCTTAACAATGTAAGGTCTTAAATGGGAAATTCAAGTATATATTTTTGACTCAAAATTAGATAGAAGGGTCATAATCGTATGGAATCGAAATTTTTCATAACTACACCCTTTTTTTCTTATCCCACGAGGGGTTACCAAGTTCATAATAGTAGCGCTCCTTGATTCAAGGGGAAAGAGAATTGCTCTCCCCACGGCGAGAGACTGATTTTGTACTCAGGTGTGTACCCCTCAATTCTGATCAAAAATTCCCAAGGGGGGAACTTGGAACTACCTGCAAGTCAACGCAGCGAGAAGCAGCGTTGAAGTTGTAATTATGCATTTATGACGTTCTAAAAATATTCTTTCATGTCGAAGTAATAAAAAAATTTCTAAAAATTAGGGTGAAATACGGAACTTTTTGACGACTTCCAGTTTTGAATGGCTACTTCCGGTTGGATGGATCGGTTTACGACCTATTTTTAAAAAGCTATAACTTTATAGCTGTTGCGAAAACATTCTGACTGAATAGCAGTGCACGCACTTCTCTAATGTAATAAGTTTTGCCGAGTG

							GCAACATTAGAAGAAGTAGGAGAGAACCCCCCCTTTTTTTTT<br>GCTGTGTTGTGTTGGCGAATTTCTCACGGATGTAGGCTTAGTT<br>TCGATGGTAGTATCTTAGATTTTTAGATGTGTTTGTATTCATA<br>GATTTGATTTCACCGCATTGGCAAGTGGCGGAAAGATTTGTA<br>ATTTGTCGATTTAATTGAATTAAAAACACTGTAATTCGTTAAT<br>GTGTTTTTCCTTGATATATAGAACTTACCACTGAACCATTTGA<br>TTCCGAACCATTGGAAGAAATATTTAACAATAGCTAATAGAC<br>GTACAATATAAAGGAAAACGTTTTTGTAAAGAGAAATGTGT<br>GAATTACAAGAAACACCCCCCCCCCCCGGGGGGTGGAAAAA<br>TAGTATTTTCAACGTAGTTGGATAAAGTTCTCTTTAAGGCGTG<br>GCGCCATTTTCGTCTTAAAGAATGAAAAATAACTCTTTCCGTT<br>TTACAGCGTGTGGCCATATAGTACATTCATGTCTCAAAATTTT<br>GAATTGTATTGTGCGTAATTTTATTAGAATTTTTTTTTATTCT<br>GAATTACAAAACTACTGTTTAACATCGAATCGTCACCGTTTA<br>TTCCAGTCCGGTACTTATTGCATAACCAGAATAAAAGCACTC<br>GTAATATATACAAGAGTAATTTAACCAAAGTCGGAACGCTTC<br>TGTTATTCCTGCTCACCTGTTCGCCAAGTTGTTCGTTCGATTGT<br>TCCACATCCGTTATCACGGCGCACATATTGTCGGCGACGAAA<br>TGTTGTCGATTTTCCCGACTATAACATTTTTGACGTTACAAAT<br>TAACACGAAATAGTTTACGGTATAAACACCAGTGAGTTATCA<br>TATAAATATCGAAACAACAACAATAACAACAGACTGCAAGC<br>GAAGAAATAATCCACAACATCCGCTGCCGCCCAGGATATGC<br>GAGTCGTGATATCAGAAAAGTTTTAGGGGACTTAGCTCTCTC<br>TCTCTCTCTCTCTTTTAGGTGGCGACACCTCCGATGTGCTCCG<br>CGCAGTCCAAGTACCGATAATTTCCAGAGAATTATGCAAAG<br>CAGCGTACAGCTCTCTGCACCTACCCATCACTGAAAATATGT<br>TATGCGCTGGCGGTATGGGACTCGATGCCTGTCAGTTTGATTC

							CGGAGGACCTCTCGTTATGTGGAAAGATGGAAGGGCAACTTT GTTTGGAAGTG

PhdEnzSeP 29	P84033	1,09E-10	46,269	PF00089	7956,23	LSLSLSLLGGDTSDV<br>LRAVQVPIISRELCK<br>AAYSSLHLPITENML<br>CAGGMGLDACQFD<br>SGGPLVMWKDGRA<br>TLFGS	TGTGTCCGACAGCCCGCCAGCGCTCAGAATCCTCCATTCGTC<br>TTCTGGAAGCCATAAAACACGTCTAGCTTGAAAACAAATCGT<br>ACGATGCCGGCTTCTTATACTGCCTGTTTTGCATATTTCTCGA<br>CGCGAAACCGTGATTGGCTGTTACCTAACATATTTGCATAAT<br>TCCTTCTACCTTTGCAGCAAATTTCAGCTTCTTTAATTCCGATT<br>GGCTTACGGAAATTAACATTTTCCTCTCCGCGCTTCACATTTG<br>GAAGTGAGTGTAAATTAAGTAATATATTATCTGTTTATTTAAA<br>ATCAGTATTTTGTTACATTTTTTAATGTTCTTCTTGTTGTTAAA<br>TTTATTTCTATAATGAGGTATTTATTGTTTTTTTTTTTTGAGAA<br>AAAATACGAATGCTGTTCAAAAAGACTGTGTAAGTTGTTGTA<br>TATCTTCCCCATATGTTCTGAGGAATACCAATGAATTCAATCT<br>TAAATCCCATCAGGGGATACGTAAAGGGCTTCCGTGCTAGGC<br>AAGAGATTAAGAACCACTGATTTATACGAATGATATTTTTAG<br>TCGATACTGGTTCCAGGGAAATTTATCCCAGGAGGGAGCATT<br>TTATCTAAGAACAGTGGGAGAATAGAGGAAATTCCTAACCTT<br>AACAATGTAAGGTCTTAAATGGGAAATTCAAGTATATATTTT<br>TGACTCAAAATTAGATAGAAGGGTCATAATCGTATGGAATC<br>GAAATTTTTCATAACTACACCCTTTTTTTCTTATCCCACGAGG<br>GGTTACCAAGTTCACAATAGTAGCGCTCTTTGATTCAAGGGG<br>AAAGAGAATTGCTCTCCCCACGGCGAGAGACTGATTTTGTAC<br>TCAGGTGTGTACCCCTCAATTCTGATCAAAAATTCCCAAGGG<br>GGGGAACTTGGAACGTCCTACCTCTCTCCTACCTACAAGTCA<br>ACGCAGCGAGAAGCAGCGTTGAAGTTGTAATTATGCATTTAT<br>GACGTTCTAAAAATATTCTTTCATGTCGAAGTAATAAAAAAA<br>TTTCTAAAAATTAGGGTGAAATACGGAACTTTTTGACGACTT<br>CCAGTTTTGAATGGCTACTTCCGGTTGGATGGATCGGTTTACG<br>ACCTATTTTTAAAAAGCTATAACTTTATAGCTGTTGCGAAAA<br>CATTCTGACTGAATAGCAGTGCACGCACTTCTCTAATGTAAT

							AAGTTTTGCCGAGTGGCAACATTAGAAGAAGTAGGAGAGAA<br>CCCCCCCTTTTTTTTTGCTGTGTTGTGTTGGCGAATTTCTCACG<br>GATGTAGGCTTAGTTTCGATGGTAGTATCTTAGATTTTTAGAT<br>GTGTTTGTATTCATAGATTTGATTTCACCGCATTGGCAAGTGG<br>CGGAAAGATTTGTAATTTGTCGATTTAATTGAATTAAAAACA<br>CTGTAATTCGTTAATGTGTTTTTCCTTGATATATAGAACTTAC<br>CACTGAACCATTTGATTCCGAACCATTGGAAGAAATATTTAA<br>CAATAGCTAATAGACGTACAATATAAAGGAAAACGTTTTTGT<br>AAAGAGAAATGTGTGAATTACAAGAAACACCCCCCCCCCC<br>GGGGGGTGGAAAAATAGTATTTTCAACGTAGTTGGATAAAG<br>TTCTCTTTAAGGCGTGGCGCCATTTTCGTCTTAAAGAATGAAA<br>AATAACTCTTTCCGTTTTACAGCGTGTGGCCATATAGTACATT<br>CATGTCTCAAAATTTTGAATTGTATTGTGCGTAATTTTATTAG<br>AATTTTTTTTTTATTCTGAATTACAAAACTACTGTTTAACATCG<br>AATCGTCACCGTTTATTCCAGTCCGGTACTTATTGCATAACCA<br>GAATAAAAGCACTCGTAATATATACAAGAGTAATTTAACCA<br>AAGTCGGAACGCTTCTGTTATTCCTGCTCACCTGTTCGCCAAG<br>TTGTTCGTTCGATTGTTCCACATCCGTTATCACGGCGCACATA<br>TTGTCGGCGACGAAATGTTGTCGATTTTCCCGACTATAACATT<br>TTTGACGTTACAAATTAACACGAAATAGTTTACGGTATAAAC<br>ACCAGTGAGTTATCATATAAATATCGAAACAACAACAATAA<br>CAACAGACTGCAAGCGAAGAAATAATCCACAACATCCGCTG<br>CCGCCCAGGATATGCGAGTCGTGATATCAGAAAAGTTTTAGG<br>GGACTTAGCTCTCTCTCTCTCTCTCTCTTTTAGGTGGCGACAC<br>CTCCGATGTGCTCCGCGCAGTCCAAGTACCGATAATTTCCAG<br>AGAATTATGCAAAGCAGCGTACAGCTCTCTGCACCTACCCAT<br>CACTGAAAATATGTTATGCGCTGGCGGTATGGGACTCGATGC

							CTGTCAGTTTGATTCCGGAGGACCTCTCGTTATGTGGAAAGA TGGAAGGGCAACTTTGTTTGGAAGTG

PhdEnzSeP 30	Q9TXD8	2.97E-25	39,394	PF00089	18307,81	NPRPQISKIILHEGYI<br>TNYNDIALLKTVDPI<br>DFGGSEGFVNGICLP<br>EMNEEFLNSRAMVA<br>GWDHTKHRGGESV<br>TLHSAVVPIIKPDKC<br>KSYRTHITRNMICAG<br>SSELFSCQWQADSGE<br>PLILWPYGRAVLVGI<br>VSSGTACGEKDEPG<br>VYTKVSPYINWIENII<br>NAD*	TTCCCCACACATTCCACAGGATTCGAGTCAACTTTTTTGATAA<br>ATTTATCTCGATGGCGACACTAGACATCTTCCGGAGACAACA<br>TTCGCTTCATCTTTTCCTTCAGTAATAATTTTAGGGGTGATTCA<br>AGTAAGGAAGGATCCGACGTTTGGTCCTTTTTTTTTGATAGAC<br>TCTACACGCATTCAGATTTGGGAATGCACGGTCGTGTTACGA<br>TTGGTTCAGCACAATGCTGTTTGTCTTCATTGGGTAACTGTGT<br>CATGAATGAGGAAAGAAATCGACCAACTGCAACATTATATT<br>CTATTTTCATTGAGGAGAATCATCCTACATCGCTTAACAACG<br>GTAAACATCACATGACATGCCAATCACTCAAAATTGTCGCTG<br>GAGGACGGCCTCTAATAGCTGAGGATTAATCAGCGTTGATG<br>ATATTCTCGATCCAGTTTATGTAGGGAGACACTTTGGTGTAC<br>ACACCGGGCTCATCTTTTTCGCCGCACGCCGTTCCAGAAGAC<br>ACAATTCCGACTAGAACAGCCCTTCCATACGGCCACAGGAT<br>GAGCGGTTCTCCGGAATCGGCCTGCCACTGACACGAAAATA<br>ACTCTGAACTTCCGGCACATATCATGTTTCTAGTTATATGCGT<br>CCTGTAGGATTTACATTTGTCTGGTTTTATTATGGGTACAACA<br>GCTGAATGAAGGGTAACAGATTCACCTCCACGATGCTTGGTG<br>TGATCCCATCCAGCGACCATAGCTCTTGAATTCAAAAATTCC<br>TCATTCATCTCCGGAAGGCAGATCCCGTTCACGAATCCTTCC<br>GATCCACCAAAGTCAATGGGATCAACTGTTTTCAGGAGAGC<br>AATGTCGTTATAGTTCGTGATGTATCCCTCGTGTAGGATGATT<br>TTGGAAATCTGGGGCCGTGGGTTCTAGCGAGGCTTTCCTAAT<br>TCCTTAATGGATTTCAGGGATAGATATTCCAGTACCGGCTGC<br>AGTGAGACGAGGCACTTCCTCTCCTATTGAAATTCGTAGCCT<br>AATTCCAATTTTGTTTCATCTTCAGCACATCCGTCATAATCCT<br>ATATTCTGTATCTTCTTCTCTTCTCTCCTCGATCGCAACTCCCA<br>CATTTATGCTGAAGGGAGGCGATTTCTGAAAAACCTATCACA<br>CTTGTCTTACCTTCCCCTGTCCAAAAACTCTTTGATAAAAAAA

							AAACTCGCTTTAACTCCTCCCAGATGGAGTCTACCCAATAGT<br>ATGATCCGGACATACCACTCAACGAATTCGTCATTGGCTAAT<br>GAGGGTTCTGCCTCCGACGTCGCTCCCCTTAATTCCTGATCAC<br>GAAGATCCGGTGAAAGTGTTTCATAACACGTGCGACGGACG<br>GAGAAACCTCTTCTCTTTTGACTGACCACCCAGCAGTCCGCA<br>GTACCCCCTGTATAGGCAACTAGGATCATAAAAACCCTATCC<br>AAACTACGTTTATGACCTCCTCGGATCATAGAGACTTGGCCT<br>GAGCATCTCGCATTTTAAGAGGGCTTTTGTACGAAATTTAAG<br>TTAGCATTTTGAATTCCTTGCCGTCCGGTCGTGGTTCCTTCTG<br>ACCACATTTCTTTACGTTTCCACATTCACACGTTTTCTCTTAAT<br>TCTTGACCAACCTAATATGGTCTGACATTTACAACTCAGCGT<br>CATGGATTCTGGCCCTGAGGTTCGAAAAAGATACATTATCCC<br>CTTGGCACCATTTATTTGTGAAGACTTCCGTTTTATTTGCGAA<br>TTGTGGAAAAAAATGGAAAATAGACCACCGCGTGCTACATG<br>AGGACCGATGGCAATTTGCTTATGTCTCTGTGGCGATCTCTCT<br>CAGGCTCCCGCGAAACGAGAACTTTAACTTTTGACGAACCTT<br>TACGTCATCACTCGGTTATGACGCCATCAAGCGTATCTCGTA<br>CTAATTATCATGTAAGTTTGGAATCTGGCAGCAGTCAGTTTTC<br>ATCCTTTCAATTGTATTTATACTGCCATGTTAAAGCAGTGTCA<br>TGCGACTTTCATAGGATGCGTTAAATGTTGCAGTTACATTATT<br>ATTGCTTGCCCAATGTTACTTAATAACTTGTTTACGTACTAGT<br>TCAGTGTTAGCTTGCTAGCATGTAGCTAACGTAATGTTCAAA<br>TATGTTTATGTTTAATGTGTTAAATCCTTTTAAATAAAAGGAC<br>GAATTTTGTCGTTCCGTCTGCATCTTTATTTTAACAGCTAGAA<br>ATATCACTTGGAATCGCCACAGCAAGCGTTTTATTTCAGCTTT<br>CTACTACAGTCTGGAGTTTAGTAAAATACAGGGCCGACAACT<br>AAACCCCTGTATCTCTAATGCACTCTGCATTGGGCCGTGTAA<br>TATTTAATTTATCGAGCTTTTTCTCGGCAGTTTAGTTTTTCGTG

							ATAAATTTACAGAAGTAAAATATTTTGCAAGAGTTGTATAGATTTCTGAAAAGATTAAACGCAGATATTTCTTCCGGAACCAATTTTTGGGGGGAGGGACGCATTCTAGAAGAGTATATAAATGTTAACAAGTAACTGTAGCCTAAGAACCTTTACGACGTTTCTGATAAACCGCAAGTTCAGCTCGCTCTTCTTGCAGTCCACACTCGAAATTTCTGCGTACTTTTTAAAAATAACAGACTCACCTTGTGCCGC

PhdEnzSeP 31	P84033	4,99E-22	34,545	PF00089	17715,15	ISKIILHEGYITNYNDIALLKTVDPIDFGGSEGFVNGICLPEMNEEFLNSRAMVAGWDHTKHRGGESVTLHSAVVPIIKPDKCKSYRTHITRNMICAGSSELFSCQWQADSGEPLILWPYGRAVLVGIVSSGTACGEKDEPGVYTKVSPYINWIENIINAD*	TTCCCCACACATTCCACAGGATTCGAGTCAACTTTTTTGATAAATTTATCTCGATGGCGACACTAGACATCTTCCGGAGACAACATTCGCTTCATCTTTTCCTTCAGTAATAATTTTAGGGGTGATTCAAGTAAGGAAGGATCCGACGTTTGGTCCTTTTTTTTTGATAGACTCTACACGCATTCAGATTTGGGAATGCACGGTCGTGTTACGATTGGTTCAGCACAATGCTGTTTGTCTTCATTGGGTAACTGTGTCATGAATGAGGAAAGAAATCGACCAACTGCAACATTATATTCTATTTTCATTGAGGAGAATCATCCTACATCGCTTAACAACGGTAAACATCACATGACATGCCAATCACTCAAAATTGTCGCTGGAGGACGGCCTCTAATAGCTGAGGATTAATCAGCGTTGATGATATTCTCGATCCAGTTTATGTAGGGAGACACTTTGGTGTACACACCGGGCTCATCTTTTTCGCCGCACGCCGTTCCAGAAGACACAATTCCGACTAGAACAGCCCTTCCATACGGCCACAGGATGAGCGGTTCTCCGGAATCGGCCTGCCACTGACACGAAAATAACTCTGAACTTCCGGCACATATCATGTTTCTAGTTATATGCGTCCTGTAGGATTTACATTTGTCTGGTTTTATTATGGGTACAACAGCTGAATGAAGGGTAACAGATTCACCTCCACGATGCTTGGTGTGATCCCATCCAGCGACCATAGCTCTTGAATTCAAAAATTCCTCATTCATCTCCGGAAGGCAGATCCCGTTCACGAATCCTTCCGATCCACCAAAGTCAATGGGATCAACTGTTTTCAGGAGAGCAATGTCGTTATAGTTCGTGATGTATCCCTCGTGTAGGATGATTTTGGAAATCTAGAAGAGTAGAAAGGGAAGCGACAGTAAAAATCGTATGGATTCGATCAAACCTGTTGAGTCGACTTTGTTCATCATACGGTGATCACAGCTTTTAAGAATATTTTTACCTTTATTTTCATTTCTCATAATTTACTGATTATTAAGAAAGACAAAAGCTTTCTATGCAGTATCTATTAAGTACATACTATCAGTAGATGGTGGTAGAAAAAACTAAGCGAGCCGTGGAATAGGGATACGAACCTAAAAACAGCCATCAAGAAACTCGGTGAAGCTAGAGGTTA

							GGTTAGAAGTCTCAAGCTGGCAGCCTTTAAAGCATACACATGTGGTCCGCGCCTAGTCCAATCCCCTACCTTTAACCCTGCGTCGCACAGTGGTTCCAAATGCTGAAAAACGGGACAAAAGTCGCTACAGGTTTGCCATTTTTTGCGTCGTTTTTAATGTAATTTTTCACGCTGAATCGAATGAAAACAACCGCAGGTCCCTACGACTAACCATTCTTGAGATATTGCGTCTTTCCCCCCTGTAAGGAAGAAAGGGGAAATCAGTAAAATTCAAACATGTTCATATTTTTTCAACATAGGTGTCGTTCTCTGGTTTTATTCTATTCAGCGTTCCAGAAA

PhdEnzSeP 32	Q9TXD8	3,03E-48	42,276	PF00089	30696.24<br>**28375.34**	MKNILGFCAILQLCF<br>VLGNALG**TGRVVT**<br>**VRNCGKSYGQSRIS**<br>**GGTPVREGLYPWM**<br>**VALHQEYGGKFYHI**<br>**CAGSILNERWILTA**<br>**AHCIKQPNDPQKY**<br>**DIFVGVHHLSRGTS**<br>**NRVARHKISKIILHE**<br>**GYITNYNDIALLKT**<br>**VDPIDFGGSEGFVN**<br>**GICLPEMNEEFLNS**<br>**RAMVAGWDHTKH**<br>**RGGESVTLHSAVVP**<br>**IIKPDKCKSYRTHIT**<br>**RNMICAGSSELFSC**<br>**QWQADSGEPLILW**<br>**PYGRAVLVGIVSSG**<br>**TACGEKDEPGVYT**<br>**KVSPYINWIENIINA**<br>**D***	TTCCCCACACATTCCACAGGATTCGAGTCAACTTTTTTGATAA<br>ATTTATCTCGATGGCGACACTAGACATCTTCCGGAGACAACA<br>TTCGCTTCATCTTTTCCTTCAGTAATAATTTTAGGGGTGATTCA<br>AGTAAGGAAGGATCCGACGTTTGGTCCTTTTTTTTTGATAGAC<br>TCTACACGCATTCAGATTTGGGAATGCACGGTCGTGTTACGA<br>TTGGTTCAGCACAATGCTGTTTGTCTTCATTGGGTAACTGTGT<br>CATGAATGAGGAAAGAAATCGACCAACTGCAACATTATATT<br>CTATTTTCATTGAGGAGAATCATCCTACATCGCTTAACAACG<br>GTAAACATCACATGACATGCCAATCACTCAAAATTGTCGCTG<br>GAGGACGGCCTCTAATAGCTGAGGATTAATCAGCGTTGATG<br>ATATTCTCGATCCAGTTTATGTAGGGAGACACTTTGGTGTAC<br>ACACCGGGCTCATCTTTTTCGCCGCACGCCGTTCCAGAAGAC<br>ACAATTCCGACTAGAACAGCCCTTCCATACGGCCACAGGAT<br>GAGCGGTTCTCCGGAATCGGCCTGCCACTGACACGAAAATA<br>ACTCTGAACTTCCGGCACATATCATGTTTCTAGTTATATGCGT<br>CCTGTAGGATTTACATTTGTCTGGTTTTATTATGGGTACAACA<br>GCTGAATGAAGGGTAACAGATTCACCTCCACGATGCTTGGTG<br>TGATCCCATCCAGCGACCATAGCTCTTGAATTCAAAAATTCC<br>TCATTCATCTCCGGAAGGCAGATCCCGTTCACGAATCCTTCC<br>GATCCACCAAAGTCAATGGGATCAACTGTTTTCAGGAGAGC<br>AATGTCGTTATAGTTCGTGATGTATCCCTCGTGTAGGATGATT<br>TTGGAAATCTTGTGCCGCGCAACTCGATTTGAAGTTCCCCTCG<br>ATAAATGATGCACGCCTACGAAGATGTCGTACTTTTGGGGAT<br>CATTTGGTTGCTTTATGCAATGTGCGGCTGTCAAAATCCACCG<br>TTCATTCAGGATGGAGCCTGCACAGATGTGATAAAATTTTCC<br>TCCATATTCCTGATGCAGTGCGACCATCCACGGATATAATCC<br>TTCCCTTACAGGTGTTCCACCTGAAATCCTGGATTGACCATA<br>ACTTTTACCACAGTTCCTTACTGTCACTACTCTGCCTGTACCA

							AGCGCATTTCCAAGTACGAAACACAGCTGCAGAATAGCACAAAACCCTAGAATGTTCTTCATCTTGTCCTAGAGTCAAGACCTGGTGCCTATATTGGAAAGTAGTGTCTGCTCATCATATTTATTAGAAGGCTCTGATGTAAAACATAGAGTGTCATGCAATCCATTCAGAAAGTAATTTCGTATGCTGTTCGTTGGGCCACGCTCTGAAAAGCATTGTGTGCCGTCGCAGTTATCTGAACATCCA

PhdEnzSeP 33	Q9TXD8	2,43E-48	42,276	PF00089	30696.24<br>**28375.34**	MKNILGFCAILQLCFVLGNALG**TGRVVTVRNCGKSYGQSRISGGTPVREGLYPWMVALHQEYGGKFYHICAGSILNERWILTAAHCIKQPNDPQKYDIFVGVHHLSRGTSNRVARHKISKIILHEGYITNYNDIALLKTVDPIDFGGSEGFVNGICLPEMNEEFLNSRAMVAGWDHTKHRGGESVTLHSAVVPIIKPDKCKSYRTHITRNMICAGSSELFSCQWQADSGEPLILWPYGRAVLVGIVSSGTACGEKDEPGVYTKVSPYINWIENIINAD***	TTCCCCACACATTCCACAGGATTCGAGTCAACTTTTTTGATAAATTTATCTCGATGGCGACACTAGACATCTTCCGGAGACAACATTCGCTTCATCTTTTCCTTCAGTAATAATTTTAGGGGTGATTCAAGTAAGGAAGGATCCGACGTTTGGTCCTTTTTTTTTGATAGACTCTACACGCATTCAGATTTGGGAATGCACGGTCGTGTTACGATTGGTTCAGCACAATGCTGTTTGTCTTCATTGGGTAACTGTGTCATGAATGAGGAAAGAAATCGACCAACTGCAACATTATATTCTATTTTCATTGAGGAGAATCATCCTACATCGCTTAACAACGGTAAACATCACATGACATGCCAATCACTCAAAATTGTCGCTGGAGGACGGCCTCTAATAGCTGAGGATTAATCAGCGTTGATGATATTCTCGATCCAGTTTATGTAGGGAGACACTTTGGTGTACACACCGGGCTCATCTTTTTCGCCGCACGCCGTTCCAGAAGACACAATTCCGACTAGAACAGCCCTTCCATACGGCCACAGGATGAGCGGTTCTCCGGAATCGGCCTGCCACTGACACGAAAATAACTCTGAACTTCCGGCACATATCATGTTTCTAGTTATATGCGTCCTGTAGGATTTACATTTGTCTGGTTTTATTATGGGTACAACAGCTGAATGAAGGGTAACAGATTCACCTCCACGATGCTTGGTGTGATCCCATCCAGCGACCATAGCTCTTGAATTCAAAAATTCCTCATTCATCTCCGGAAGGCAGATCCCGTTCACGAATCCTTCCGATCCACCAAAGTCAATGGGATCAACTGTTTTCAGGAGAGCAATGTCGTTATAGTTCGTGATGTATCCCTCGTGTAGGATGATTTTGGAAATCTTGTGCCGCGCAACTCGATTTGAAGTTCCCCTCGATAAATGATGCACGCCTACGAAGATGTCGTACTTTTGGGGATCATTTGGTTGCTTTATGCAATGTGCGGCTGTCAAAATCCACCGTTCATTCAGGATGGAGCCTGCACAGATGTGATAAAATTTTCCTCCATATTCCTGATGCAGTGCGACCATCCACGGATATAATCCTTCCCTTACAGGTGTTCCACCTGAAATCCTGGATTGACCATAACTTTTACCACAGTTCCTTACTGTCACTACTCTGCCTGTACCA

							AGCGCATTTCCAAGTACGAAACACAGCTGCAGAATAGCACAAAACCCTAGAATGTTCTTCATCTTGTCCTAGAGTCAAGACCTGGTGCCTATATTGGAAAGTAGTGTCTGCTCATCATATTTATTAGAAGGCTCTGATGTAAAACATCTGTTTCAAAAACAAAAAGTTACTTAAGAAAGGTATAAATGCCACCAGACGCTGGAAATTTAAAA

PhdEnzSeP 34	Q7M4I3	1,13E-32	32,510	PF00089	54839,13	YGGGYNPKPVVDSD<br>DEIKPAIGRPGDGGG<br>YGGGYNPNPVVDR<br>DDDRKPGRGRPSGG<br>GGYNPKPGVDRDD<br>YDIKPAVNNPKPGG<br>SRGGYNPKPDEDDY<br>TIKPARGGDGYPKD<br>DRKPIREDLGGYKGS<br>KDDDYNDYETRGD<br>GKNDDYEEDGKPVP<br>VVDGNCICVPYYQC<br>QDGHIVTDGAGIID<br>ARKRPEPDDELPLD<br>GKFKPPSCGPYHVC<br>CNTPETSTVKPYEHR<br>CGVRNPSGINSRILSP<br>SNKGEADFGEWPW<br>QAAVLKSEGKVNIF<br>QCGGVLIDKYHVLT<br>VAHCVFHLYQFNEY<br>PLKVRLGEWDTQTT<br>TEFLAHEDYRVSKIII<br>HPEFRNTSLWNDIA<br>VLRLQDPVLFAPHID<br>TVCLPQYDEVFSGQ<br>NCVVTGWGKDAYK<br>GGTFSNVMKEVALQ	TTTTTTTTTTAGATTCAATCTTTTTATTGGAAGTTCAATCAATT<br>TAACATATTTTTCCAATGGATAATGAATGAATAAATCAAAAT<br>CTTAAGCAGAAATGCCATTCATGTTGTCCTCAATACAACGAC<br>AACAGATGTAGATACTAGTCCTGTAGATTATAGTATAATGCC<br>ACGACTCAAATTCAATAGTGAAATATATAGTTCAAATAGTTC<br>CTGCAAATAGTTCCTATTAATTTCTGCTCTGCAACCATAAGA<br>GGCTTAATGAGTTTTTTACGCCCAGTTCCCCTCTAAAGAAAT<br>ATTGCGAATCAAATGCCCGTTACCTTTCGGGTAGAAGAGAGG<br>AATTTCAGTTAAAGCATCAACACGTAAAAACTAACATGTCGT<br>TATCGAATAAAAAGTAGTCTGAATTCAACGTAATAAGCCGTT<br>AATATCAAACTGAGAGCAATCCGGTTTGCGAGCATATACGTC<br>ACTAACGCTAAAAGGGTTAAATATTCCGATAAATTTTCTCGT<br>TCAGGATCTTAACTCATAAATTTGATTTCTAAGCTGTTTTACA<br>CAATAAAACTTAATTCCTTTTGAGCCATTCAGCTTTTGACCTC<br>TCAGATATAATTGATTGATAGAGTAGATGAATGCTGTTATTA<br>AATAATTTTCGATATTATTAATCATCAACTGAAACATACAAA<br>AAGAGGCAGTCGCATTGCGGTAGAGAAAGGCTTTCCTTAAA<br>ACAGTGGTTCAGAAATTTTGAAGGCTCGAGGAACCTTTAAAA<br>AGTTAACTTGATTTCACTTCTCTAAATGAATGTACCCCTCCAA<br>GGTTCTACGATAAGATTGAGGCGAAGCATACAGAAGAAACA<br>AAGTAATTATTATTTACCAGGAATTACAAAACTAAACTATGT<br>GCTGTTCATAGCGGTAACTTTTTTTTTTTCCCTGTTTTATCTTT<br>TCTTTTTGGCACTATTTCATTTTCCTTCTGTGTAGTGCTTTGTTT<br>CCTGTTGTATAATTTATGACGTTTATTACTGCAATCGCAGAGG<br>GCATGTCTAGTATTTATTTTTTACTTATTTTGCACGAACTTTAA<br>GAATATTCGCTCCGAGTACACGCGGCAAACGTGCTCGTAAA<br>ATCTGTGGAGTCGAAAGTCCTGTGGGCGTTAATTGCAGGTTC<br>TCATGGGTACATGGTTCAGACGTTTCCCTCCCTTCTGACCATG

						VIDNYKCEEMLRKT<br>RLGRFFQLDEGFLCA<br>GGEYGIDSCKGDGG<br>GPLVCYRKDKTYAL<br>AGIVSWGIDCGQPE<br>VPGVYVKIQKYLDFI<br>SKTTGVALQEYWPK<br>H*	TCTAAATTGGAGGGCCCTGGGAATGCCTATACAGAAGTCCTA<br>AACCTGAGAAAATTAGGTGGACCGGGCATTCTCCAGTTTCAA<br>AAAAAAAAAAAGAATATTCGCTACTCGTCTCAAGAAGCTTG<br>CTTAAGGCTCGGCTTCCCACATATCACAAATTCCCATGTTAA<br>GCTATGAGAATTTGGCTAAACCAAGTAGTGTGTACTGGCTTG<br>GAGAGTCGGAAGGCCTTTGACGAAAGCAGGTAGGAACTTTG<br>TGGTAATTAAGCCATCTCTAAGATGGAGTAAAATTAGGAAG<br>AAATCGATGGAAATTGATTTTTGAGTTATAATTGCACAACTT<br>ATATCACAGATTGATTACTTTTTGTGATTTTTAGTTGTTAAGAT<br>ATCAGTTCTGAGACCTAAATAAATTTGCTAGTTGTTATTACTA<br>CATTTTGAATGTTTTTTGAGCTTGGTGATTCTATATTTACGTAG<br>ATTAGATTTTGAAGTTTTGCTTGTACACGCGTACTCAAGTTAG<br>AATTGAAGGAATGTTGCTGCAAATTTATTTAGGATGGAGTTG<br>AAAGTATCCTTGGGATAATTTATCATGCGTTGGACCTTGCTGT<br>TTAGTTCTCTTATAGTAAGAAAAGAGTTTTGAGGTAGTCAGG<br>GTGAGGCCTAACGGCTTTGTTGTTGACCTAGGAAACATATCT<br>CGCATTAGTGGAAGAGGGTAGAATTTCACAAAAATATTAAA<br>TCTAAGGCTAAATACAGCTGGTATTACTAGCCGTTTCATATC<br>ATTAAAAACAATCGATTGATATTTCTGATCTCTCATTTTCCCT<br>GTTTGGGCTAAAGTCTTGATTTGTTATAAAGCAATGTCAACTC<br>TGCGTACAAAATCGTAGTGGTGGTCTCTCTTCTGACTTGTTTA<br>ACTTTTTTAATGCCGCTGTGCTGAGTTTTCAAGACTCCACCTG<br>TATTCTCAATTATCATTAACTTGTGTCTGTTTCCCTCCGATCAG<br>AGATGGAATGCTTAGCAAGGTATTCAGCAAAAGAAATTGCG<br>AAGTTTGACAAAAAGTTAACTGAACCAAAAGTGCTTAGCAG<br>CTGATTTTGAGAGTAGAGCCCTGCTGAAGCAAAATTTCTTCA<br>ATCCAAACCTTGATGAATTGCATGACCCTAAAACGACTACAT<br>ACCTTGTTCTGAGCAAGAAATCTCTTTTAGATTTAATTCTATT

							TCTATCAACTTATCTCTGTTAATACGGTTTTCAACTTTTGTCAA<br>CAAGATGACTCAAAAGCTAGAAAACAATTGAATGGTAGGCT<br>TGGCGTCGTTTAATGCTGCATCTCAAATAAACTGCTTGACTG<br>ACCATTTAAGGCACCAGCATCCACCACACCGAAGTGATTTTA<br>AAATTGTCCTCTTTTATATGTGTACTGAGTGGCTAGAGATAAT<br>TTTTATATACTTTTTAACGTATAATTTTTAAAATTTTAATTAAG<br>CTTAATTATTTCATTGCTTGTGTTAATTTCAATGAATATGCATT<br>TTTCAGTCTACACCCCAAGCCAAGTTTTAGGTACTAGACTGA<br>GAAGTTTGCACTTTCAAGCAAAAAATGCAAACTCACGTTGCT<br>TAAACATTAACTCGAGATCCAGATTCGTGGGAGGTATAGGTC<br>ATAAAATCTTTCCTAGAGAAGCGTGATAACATGTTAACGAAC<br>AACTGATCTAAAATATTATGTTTATCTAAGCTCTAGCTTTAAA<br>ATTGTGAGACAATCTTACAATTATTGTGTAAAAAGAGTAACA<br>CTGCTCTATAAGCTTATTCCGTCCAAAACTTATAATTTTCTCTT<br>TAATATTATGATTATATTCACAACTGAATGGCTCCTTAATTCA<br>CGTTTGTAGGATTCTAGTAGAGGAAACTAAAATTGTAAAATT<br>TCTTTTGATCTTTCCTAAAAATTTACAGCAAAAATTTTAATTTT<br>ATTGTCACACAGTTTTGTACAAACTTTTTTTGAGTTTTTCTCCT<br>ACAAACTCGCAACAATTGTCTGTCGAACCCCCAAAATTCCTC<br>GAACCACAGTTTCGAAACTACTGACTTGAAATAACGAAAGA<br>TTCCAGATTAGCTCTTGAGGAAACTATATTCTAACATCCCAG<br>ATTTATTTTTGAGATTTTAAACACATAGAAAAGACGTTTCTGT<br>GAGGAAACAGCTAATGCTTTGGCCAATATTCTTGTAAAGCAA<br>CTCCCGTTGTCTTGCTGATAAAATCTAAATATTTCTGGATCTT<br>TACGTAAACGCCAGGAACTTCTGGTTGCCCACAGTCAATGCC<br>CCATGAAACGATTCCGGCCAAGGCGTAGGTCTTATCTTTGCG<br>ATAGCAGACTAAAGGACCACCTCCATCACCTTTACAAGAAT<br>CAATTCCATATTCACCGCCGGCGCACAGGAAACCTTCGTCCA

							GTTGGAAGAATCTTCCCAATCTTGTTTTCCTCAGCATCTCTTC<br>ACATTTGTAGTTGTCGATTACTTGAAGAGCAACTTCTTTCATC<br>ACATTAGAGAAGGTACCGCCCTTGTAGGCGTCTTTTCCCCAT<br>CCAGTAACCACGCAGTTTTGGCCCGAAAACACTTCATCGTAC<br>TGGGGGAGGCACACAGTATCTATGTGAGGAGCAAACAGTAC<br>AGGATCTTGCAATCTCAGCACAGCAATGTCGTTCCAAAGGCT<br>AGTGTTGCGAAATTCTGGATGAATGATGATCTTTGATACTCTG<br>TAATCCTCGTGAGCCAAGAATTCTGTTGTTGTCTGTGTATCCC<br>ATTCACCCAGACGAACTTTCAGTGGATATTCATTGAACTGGT<br>ATAAGTGGAATACGCAATGAGCCACTGTGAGAACGTGATAT<br>TTATCGATAAGAACACCTCCACACTGGAATATATTCACTTTA<br>CCTTCAGATTTTAGAACAGCTGCTTGCCATGGCCATTCACCG<br>AAATCTGCTTCGCCCTTGTTGCTGGGCGATAAAATCCTACTGT<br>TGATTCCGCTTGGATTTCGAACTCCACAACGGTGTTCATAAG<br>GCTTTACGGTAGAAGTTTCTGGAGTGTTGCAGCACACGTGAT<br>AGGGTCCGCAAGATGGCGGTTTGAACTTGCCATCCAACGGA<br>AGTTCGTCATCAGGTTCCGGCCTCTTCCTTGCATCGATAATGC<br>CTGCACCATCAGTAACGATATGGCCATCTTGACATTGATAGT<br>AGGGAACACATATACAATTTCCATCCACGACCGGTACTGGTT<br>TACCATCTTCCTCGTAGTCATCATTTTTTCCATCACCTCTAGTT<br>TCGTAATCATTGTAGTCGTCATCTTTAGATCCCTTGTATCCAC<br>CTAAATCTTCCCTGATGGGTTTACGATCATCTTTAGGATAACC<br>ATCCCCACCACGTGCAGGCTTAATTGTGTAATCATCCTCGTC<br>AGGCTTAGGATTATAACCTCCTCTAGAGCCCCCTGGTTTGGG<br>GTTATTTACTGCTGGTTTGATATCGTAGTCATCTCTGTCCACTC<br>CTGGCTTTGGATTATATCCCCCTCCACCACTCGGCCTGCCTCT<br>GCCCGGCTTGCGATCGTCATCCCTATCTACTACAGGGTTCGG<br>ATTATAACCTCCACCATAACCACCTCCATCACCTGGTCTTCC

							AATAGCAGGTTTGATTTCATCATCGCTATCTACTACAGGCTTC GGATTATAACCTCCACCATA

PhdEnzSeP 35	P84033	8,04E-50	42,791	PF00089	28690.94 **24922.53**	MSTRGGGQRQAPDRCQMKLYWILQLCFVLGNASG**TGERVTVRNCGTLLNNQGRIVGGLPVREGLFPWMVTLHEKYDTKLHHVCGASILNERWVVTAAHCISDPNKPELFEIYVGLHRLSRKAAKQVKRHEISKIIVHEGFSRFHHAGHDIALLKIADRIDFGGSEGFVNGICLPTQNEGEPSSVALVAGWGHTTEGGDTSDVLRAVRVPLISRKLCAAKYSSRYGVGAITENMLCAGDVGLDSCQADSGGPLMVRKAGRATLYG**	TTCCATACAAAGTTGCCCTTCCAGCCTTTCGCACCATGAGGGGTCCTCCGGAATCAGCCTGACAGGAATCGAGCCCTACATCGCCAGCGCATAACATATTTTCAGTGATCGCACCCACTCCATAACGAGAGCTGTACTTTGCGGCGCATAATTTTCTGGAAATCAAGGGTACTCTAACTGCACGGAGCACATCGGAGGTGTCGCCACCTTCAGTGGTGTGTCCCCACCCGGCGACCAGAGCCACCGAGCTGGGTTCGCCCTCGTTCTGCGTCGGAAGACAGATCCCATTCACGAATCCTTCCGATCCACCGAAGTCGATGCGATCGGCTATTTTCAGAAGAGCGATGTCATGTCCAGCGTGGTGGAACCTGCTAAAGCCCTCGTGTACGATGATCTTGGAAATCTCGTGCCTCTTAACTTGCTTTGCCGCCTTCCTTGACAGACGATGCAGTCCAACGTAGATCTCAAACAATTCGGGTTTATTTGGGTCGGATATGCAATGTGCAGCTGTCACAACCCAACGTTCGTTCAGGATGGAGGCCCCACAGACGTGGTGTAATTTTGTATCGTATTTTTCATGCAGTGTGACCATCCATGGAAAAAGTCCTTCTCTGACAGGTAATCCACCTACGATCCTGCCCTGGTTATTTAGTAGCGTGCCACAGTTTCTTACTGTCACTCTTTCACCTGTACCAGACGCATTTCCAAGTACAAAACATAGCTGTAGGATCCAGTAAAGCTTCATCTGACATCTATCTGGTGCTTGCCGTTGGCCTCCTCCTCTCGTACTCATTGTAAGACCTTGAGAAACTGGAACTATCGGTGAGAATTTGAAAAGTGCACCACATGCTGAAATCTTCGAAAGCTTTCACGGAACATAATAATTGCAGATGTGCGTAAGTTTGACGTCTTTCATGCA
PhdEnzSeP 36	Q9TXD8	4,22E-12	44,000	PF00089	7476,56	EARGGDTSDVLRAVRVPLISRKLCAAKYSSRYGVGAITENMLCAGDVGLDSCQADSGGPLMVRKAGRATLYG	TTCCATACAAAGTTGCCCTTCCAGCCTTTCGCACCATGAGGGGTCCTCCGGAATCAGCCTGACAGGAATCGAGCCCTACATCGCCAGCGCATAACATATTTTCAGTGATCGCACCCACTCCATAACGAGAGCTGTACTTTGCGGCGCATAATTTTCTGGAAATCAAGGGTACTCTAACTGCACGGAGCACATCGGAGGTGTCGCCACCTCGTGCCTCTTAACTTGCTTTGCCGCCTTCCTTGACAGACGATGC

							AGTCCAACGTAGATCTCAAACAATTCGGGTTTATTTGGGGAAGGAAACAGACGCTTACTCGGATATGCAATGTGCAGCTGTCACAATCCAACGTTCGTTCAAGATGGAGGCCCCACAGACGTGGTGTAATTTTGTATCGTATTTTTCATGCAGTGTGACCAT
PhdEnzSeP 37	Q9TXD8	8,90E-40	41,000	PF00089	22897,82	VSDEFENIMCPDKPEVHEVYVGLHRLSRKAAKQVKRHEISKIILHEDYGKHRNEHDIALLKMADPIDFGGSEGFVNGICLPTQNETEPSLVAMVAGWGHTTEGGDTSDVLRSVRVPMLSRGECDKVYSSLYEGVESNIITGNMVCAGSTGLDSCQADSGGPLVLWKYGRATLYGIVSFGKGCGFPGFPGVYTRVSSYVDWIANKIHSDNS*	GTAACTTTATATTTAATGCATCGCTGAGAACCATTATACATTTCTTAGCAATGATACCCATTTTACAACATCATTATAACTAAGAACTGGAGAAATGCACTTGTACTTAAGAGTTGTCGGAGTGAATCTTGTTCGCTATCCAGTCTACGTACGAAGACACTCTGGTATAGACACCAGGAAATCCTGGAAAGCCGCAGCCCTTTCCAAAAGACACAATTCCATACAAAGTTGCCCTTCCATACTTCCACAAAACGAGAGGTCCTCCGGAATCAGCCTGACAAGAATCAAGTCCTGTACTGCCAGCGCATACCATATTTCCAGTGATTATATTACTCTCCACTCCTTCGTACAGAGAGCTGTAGACTTTGTCGCATTCTCCTCTGGAAAGCATGGGAACGCGAACCGAGCGGAGCACATCAGAGGTGTCGCCACCTTCAGTGGTGTGTCCCCATCCAGCAACCATAGCCACCAAGCTGGGTTCCGTCTCGTTCTGCGTTGGAAGACAGATCCCATTCACGAATCCTTCCGATCCACCGAAATCGATGGGATCCGCCATTTTCAGGAGGGCGATATCGTGTTCGTTTCGATGCTTGCCATAGTCCTCGTGTAGGATGATCTTGGAAATCTCGTGCCTCTTAACTTGCTTTGCCGCCTTCCTTGACAGACGATGCAGCCCAACGTAGACCTCGTGCACTTCGGGTTTATCTGGGCACATAATGTTCTCGAACTCGTCACTTACTT

PhdEnzSeP 38	Q9TXD8	4,37E-58	42,276	PF00089	32456.01<br>**30437.52**	MWFYWILQLCFML<br>GNA**SRIGLTVRNCG**<br>**KNLNNQGRIVGGL**<br>**PVREGFFPWMVTL**<br>**HEKYDKKFYHVCG**<br>**ASILNERWILTAAH**<br>**CIDYPDKPEVHEVY**<br>**VGLHRLSRKAAKQ**<br>**VKRHEISKIILHEDY**<br>**GKHRNEHDIALLK**<br>**MADPIDFGGSEGFV**<br>**NGICLPTQNETEPSL**<br>**VAMVAGWGHTTE**<br>**GGDTSDVLRSVRV**<br>**PMLSRGECDKVYSS**<br>**LYEGVESNIITGNM**<br>**VCAGSTGLDSCQA**<br>**DSGGPLVMWKNR**<br>**RITLFGIVSFGEGCG**<br>**LPGYPGVCTRVSAY**<br>**VNWIENKIHSDTSS**<br>**DFFSDSS***	ATTTTCATCCTTGGAAAATCTCCATATAAAAATATCTTTATTG<br>CAAATTTATTTATTTGAAAACGACGAATTTATCATTCCAGTCA<br>TCAACGACACAAAATCAATGACAACGTCTCTTTGACACAAA<br>ATATCAAAGCTGCCGAAAGGAATCATTAAAAACAAAATAAA<br>AACTTACGCAAGAAGTGCTCATTTCAGTAATAGAAAAAATT<br>ATCTTCCCTGACAATTAGATGATTTTTCAGTAAAAGATATATT<br>TGATCACATCTGGGACGGATGAAGGCGACTTTTCATGAAATA<br>CCAAACGTGCCCGACAGGAGAAGGGTCGTACGATCACGCTG<br>AGCAAGGAAGTGCGTCGACATTCGGAAGGATGTCACTGAAG<br>ACTCATGTTATGTGCTCCTGTAAGGTAGACTCAAAATCCTTCG<br>ACAGGAATGGCAGCGTAATAGGTCCCAGAATTTCGTTGACAT<br>GCCGTTGTTCCACGAGTGAACCACGGACAACCGTCACAGAG<br>GTCTGAAATTAAACAAATTGCGCCTCAGATCATCGGCGTTAG<br>GGACGGGCCCGTCTCTAACGCCTATGCGCTTGTCATCGAAGC<br>TCTGAAGACCACGGCACCAACGCAGTCGCTGCTGACGATGTT<br>CGGTAGCTAGTGGTATCCGGAGGATTGGCCATCTCGGGTGTA<br>GTCTACGCGCCTGTATAGATCTAGCAGTGATTTGGACGGACA<br>TTTCCATACCGGTAACTTGGTGAATATCGGATAGTGACACGT<br>CATGTGCTATTCGTGCAGACCTCTCACGAGTGCTCAGTGTTCC<br>CGTTCTGGGGTTTCCCAAAACGTTTAGTTAGCCGCTTGCTGCT<br>GAATTCTTCCATTCTCGACCCATCCCCATTAGCATCGTCCAAT<br>TGTGGCTAACCCCGTTCCAAATGAAGAATGGTGTGTTGATTG<br>GACCATCCGGTTTCCTTCGACCCGATTGGTTCGTCCCCTTTCG<br>AAATATCTAATTATCCGAAGCTGTCGTGGAATCGACGTCCAG<br>GCATTATCGCAATAAATGAAGATAAACGCTATCAAAATTAA<br>ATGCAGCTTTTGCCCTTCTGAGAGAGTTTTGAGCGCAATGTG<br>CATCGACATCCCATTGACACAAAATATCAGTACAGTAACAA<br>CGTATTTTGAGAGCCCTGGCCTCAAATTTTAATCATTTTTCAT

							ATTCGGTCTTAAGACACTTGCAGGTGAAGTTTCATCGCGATT<br>AGAGTATTCTTTCGTTGTATTTCGATATTTTTGTCAAACAATGT<br>ATTGTGTTTCTACATATGTCCGTTCGGTTATTGCTCCGTTATTG<br>TTCACTGCGTGTTCCATCCTAACTCTTATACTCCAAACAATGC<br>GCCAACGTGAACACACCAAAATACGAACAGACTTATGTCAC<br>AACCCAATATTTGCATAATTTTTCTTTTTTGTGGAAGAAAATG<br>CTGGAATTCTGTGGTATGTCTTCAATTTAAAACACAGGCATT<br>AAGAGGAAAACGATGAGACACACCTGTTTTGAATATAAAGC<br>TTTAAAGCAATAATTACATAAAATAACCCTTCTTGTTGTTTTT<br>TGAAGTTTGAAAAGTATCAAGATTTCTAAATTCTATCAAAAA<br>AGCTCACAGTATTTGACTTCACTTTTTAACCATTTAAATTTTTT<br>ATGTGATGCTGTAACCCCCCCCCCCCGAAAAAAAGGAGATG<br>TTTTTTTTGACAAATGTACTTACGATGTATATTTGTACATTGTT<br>GTACATTGTATGGTATATATAATAAGTTCGAGATAACGATCT<br>TTGCTGTGAAATTTCTGATTTAGTCGTATCTCTCATGCAGCCT<br>TCTAATAGTCTTAAATAGCACAACCTTTTGTCTTTATCAGTTA<br>GCTCTGTTTAATTAATCTGGAAAGAAACCTAACAAGTTGATA<br>TTTTATATTTAATCCATCGAAGAGAATTATTATGCATTTCGCA<br>GCAGTAGTACCCGTTTAACGACTTGATTATAACTAAGAACTG<br>TCACTGAAGAAGTCACTGGAGGTGTCGGAGTGAATCTTGTTC<br>TCTATCCAGTTTACGTATGCCGACACTCTGGTACAGACACCA<br>GGATATCCTGGAAGGCCGCAGCCCTCTCCGAAAGAGACAAT<br>TCCAAACAAAGTAATCCTTCTATTCTTCCACATAACGAGAGG<br>TCCTCCGGAATCAGCCTGACAAGAATCAAGTCCTGTACTGCC<br>AGCGCATACCATATTTCCAGTGATTATATTACTCTCCACTCCT<br>TCGTACAGAGAGCTGTAGACTTTGTCGCATTCTCCTCTGGAA<br>AGCATGGGAACGCGAACCGAGCGGAGCACATCAGAGGTGTC<br>GCCACCTTCAGTGGTGTGTCCCCATCCAGCAACCATAGCCAC

							CAAGCTGGGTTCCGTCTCGTTCTGCGTTGGAAGACAGATCCC ATTCACGAATCCTTCCGATCCACCGAAATCGATGGGATCCGC CATTTTCAGGAGGGCGATATCGTGTTCGTTTCGATGCTTGCCA TAGTCCTCGTGTAGGATGATCTTGGAAATCTCGTGCCTCTTAA CTTGCTTTGCCGCCTTCCTTGACAGACGATGCAGCCCAACGT AGACCTCGTGCACTTCGGGTTTATCTGGGTAATCTATGCAAT GTGCAGCTGTCAAAATCCAACGTTCGTTCAGGATGGAGGCGC CACAGACGTGGTAAAATTTTTTATCATATTTTTCATGCAGTGT GACCATCCACGGAAAGAATCCTTCCCTGACCGGTAATCCACC CACGATCCTGCCCTGGTTATTTAGATTCTTGCCACAGTTTCTT ACTGTCAGTCCTATACGAGACGCATTTCCAAGCATAAAACAT AGCTGCAGAATCCAGTAAAACCACATAATGTTCTCGAACTTG TCACTTACTTCATCTG

PhdEnzSeP 39	Q9TXD8	3,41E-58	42,683	PF00089	31538.05<br>**29519.56**	MWFYWILQLCFML<br>GNA**SRIGLTVRNCG**<br>**KNLNNQGRIVGGL**<br>**PVREGFFPWMVTL**<br>**HEKYDKKFYHVCG**<br>**ASILNERWILTAAH**<br>**CIDYPDKPEVHEVY**<br>**VGLHRLSRKAAKQ**<br>**VKRHEISKIILHEDY**<br>**GKHRNEHDIALLK**<br>**MADPIDFGGSEGFV**<br>**NGICLPTQNETEPSL**<br>**VAMVAGWGHTTE**<br>**GGDTSDVLRSVRV**<br>**PMLSRGECDKVYSS**<br>**LYEGVESNIITGNM**<br>**VCAGSTGLDSCQA**<br>**DSGGPLVLWKYGR**<br>**ATLYGIVSFGKGCG**<br>**FPGFPGVYTRVSSY**<br>**VDWIANKIHSDNS***	GTAACTTTATATTTAATGCATCGCTGAGAACCATTATACATTT<br>CTTAGCAATGATACCCATTTTACAACATCATTATAACTAAGA<br>ACTGGAGAAATGCACTTGTACTTAAGAGTTGTCGGAGTGAAT<br>CTTGTTCGCTATCCAGTCTACGTACGAAGACACTCTGGTATA<br>GACACCAGGAAATCCTGGAAAGCCGCAGCCCTTTCCAAAAG<br>ACACAATTCCATACAAAGTTGCCCTTCCATACTTCCACAAAA<br>CGAGAGGTCCTCCGGAATCAGCCTGACAAGAATCAAGTCCT<br>GTACTGCCAGCGCATACCATATTTCCAGTGATTATATTACTCT<br>CCACTCCTTCGTACAGAGAGCTGTAGACTTTGTCGCATTCTCC<br>TCTGGAAAGCATGGGAACGCGAACCGAGCGGAGCACATCAG<br>AGGTGTCGCCACCTTCAGTGGTGTGTCCCCATCCAGCAACCA<br>TAGCCACCAAGCTGGGTTCCGTCTCGTTCTGCGTTGGAAGAC<br>AGATCCCATTCACGAATCCTTCCGATCCACCGAAATCGATGG<br>GATCCGCCATTTTCAGGAGGGCGATATCGTGTTCGTTTCGATG<br>CTTGCCATAGTCCTCGTGTAGGATGATCTTGGAAATCTCGTGC<br>CTCTTAACTTGCTTTGCCGCCTTCCTTGACAGACGATGCAGCC<br>CAACGTAGACCTCGTGCACTTCGGGTTTATCTGGGTAATCTAT<br>GCAATGTGCAGCTGTCAAAATCCAACGTTCGTTCAGGATGGA<br>GGCGCCACAGACGTGGTAAAATTTTTTATCATATTTTTCATGC<br>AGTGTGACCATCCACGGAAAGAATCCTTCCCTGACCGGTAAT<br>CCACCCACGATCCTGCCCTGGTTATTTAGATTCTTGCCACAGT<br>TTCTTACTGTCAGTCCTATACGAGACGCATTTCCAAGCATAA<br>AACATAGCTGCAGAATCCAGTAAAACCACATAATGTTCTCG<br>AACTTGTCACTTACTTCA

PhdEnzSeP40	P84033	1,23E-22	43,609	PF00089	14042,94	ISKIIVHEGFSRFHHAGHDIALLKIADRIDFGGSEGFVNGICLPTQNEGEPSSVALVAGWGHTTEGGDTSDVLRAVRVPLISRKLCAAKYSSRYGVGAITENMLCAGDVGLDSCQADSGGPLMVRKAGRATLYG	TTCCATACAAAGTTGCCCTTCCAGCCTTTCGCACCATGAGGGGTCCTCCGGAATCAGCCTGACAGGAATCGAGCCCTACATCGCCAGCGCATAACATATTTTCAGTGATCGCACCCACTCCATAACGAGAGCTGTACTTTGCGGCGCATAATTTTCTGGAAATCAAGGGTACTCTAACTGCACGGAGCACATCGGAGGTGTCGCCACCTTCAGTGGTGTGTCCCCACCCGGCGACCAGAGCCACCGAGCTGGGTTCGCCCTCGTTCTGCGTCGGAAGACAGATCCCATTCACGAATCCTTCCGATCCACCGAAGTCGATGCGATCGGCTATTTTCAGAAGAGCGATGTCATGTCCAGCGTGGTGGAACCTGCTAAAGCCCTCGTGTACGATGATCTTGGAAATCTAAGAAAGTAACAGTGCTATGCAGTGGGACTGATTTTCGGGTGGGCAAACTGACCGCTTGCCCAGGACCCACTCCATTCAGGGCCCCATAATTTTCTAGTCTTCATTTAAAATTTTGTTTAAAGTATCCGACTACATTCGGAACAATTTTCTTTCCTAAAAAAAGTATATATTAATTATTTTTACTCTTTTTAGATCAATTTTGAAATAAAAAAATGCAAATATGTAAAGAT

PhdEnzSeP 41	Q9TXD8	1,55E-57	42,683	PF00089	29163,14	GLTVRNCGKNLNNQGRIVGGLPVREGFFPWMVTLHEKYDKKFYHVCGASILNERWILTAAHCIDYPDKPEVHEVYVGLHRLSRKAAKQVKRHEISKIILHEDYGKHRNEHDIALLKMADPIDFGGSEGFVNGICLPTQNETEPSLVAMVAGWGHTTEGGDTSDVLRSVRVPMLSRGECDKVYSSLYEGVESNIITGNMVCAGSTGLDSCQADSGGPLVLWKYGRATLYGIVSFGKGCGFPGFPGVYTRVSSYVDWIANKIHSDNS*	GTAACTTTATATTTAATGCATCGCTGAGAACCATTATACATTTCTTAGCAATGATACCCATTTTACAACATCATTATAACTAAGAACTGGAGAAATGCACTTGTACTTAAGAGTTGTCGGAGTGAATCTTGTTCGCTATCCAGTCTACGTACGAAGACACTCTGGTATAGACACCAGGAAATCCTGGAAAGCCGCAGCCCTTTCCAAAAGACACAATTCCATACAAAGTTGCCCTTCCATACTTCCACAAAACGAGAGGTCCTCCGGAATCAGCCTGACAAGAATCAAGTCCTGTACTGCCAGCGCATACCATATTTCCAGTGATTATATTACTCTCCACTCCTTCGTACAGAGAGCTGTAGACTTTGTCGCATTCTCCTCTGGAAAGCATGGGAACGCGAACCGAGCGGAGCACATCAGAGGTGTCGCCACCTTCAGTGGTGTGTCCCCATCCAGCAACCATAGCCACCAAGCTGGGTTCCGTCTCGTTCTGCGTTGGAAGACAGATCCCATTCACGAATCCTTCCGATCCACCGAAATCGATGGGATCCGCCATTTTCAGGAGGGCGATATCGTGTTCGTTTCGATGCTTGCCATAGTCCTCGTGTAGGATGATCTTGGAAATCTCGTGCCTCTTAACTTGCTTTGCCGCCTTCCTTGACAGACGATGCAGCCCAACGTAGACCTCGTGCACTTCGGGTTTATCTGGGTAATCTATGCAATGTGCAGCTGTCAAAATCCAACGTTCGTTCAGGATGGAGGCGCCACAGACGTGGTAAAATTTTTTATCATATTTTTCATGCAGTGTGACCATCCACGGAAAGAATCCTTCCCTGACCGGTAATCCACCCACGATCCTGCCCTGGTTATTTAGATTCTTGCCACAGTTTCTTACTGTCAGTCCTTATAAAATGAAGCAGAAATTAATCTTACAAAGGGTCCACGGATATAAAATAATAGTTCTCTATGTGCATAAAATGTTACCTATACGAGACGCATTTCCAAGCATAAAACATAGCTGCAGAATCCAGTAAAACCTAGAACGAAAGTCAGATGTTTCAAACCAATATAAGAATTTCGCAATTTACTGAACAATCCATCTCCTCTTAAACAACAACAACAACGAAAATCTGATTGTCAACTATTTTGAAA

PhdEnzSeP 42	Q9TXD8	5,17E-58	42,276	PF00089	30081,1	GLTVRNCGKNLNN<br>QGRIVGGLPVREGFF<br>PWMVTLHEKYDKK<br>FYHVCGASILNERWI<br>LTAAHCIDYPDKPE<br>VHEVYVGLHRLSRK<br>AAKQVKRHEISKIIL<br>HEDYGKHRNEHDIA<br>LLKMADPIDFGGSE<br>GFVNGICLPTQNETE<br>PSLVAMVAGWGHT<br>TEGGDTSDVLRSVRV<br>PMLSRGECDKVYSSL<br>YEGVESNIITGNMVC<br>AGSTGLDSCQADSG<br>GPLVMWKNRRITLF<br>GIVSFGEGCGLPGYP<br>GVCTRVSAYVNWIE<br>NKIHSDTSSDFFSDSS<br>*	ATTTTCATCCTTGGAAAATCTCCATATAAAAATATCTTTATTG<br>CAAATTTATTTATTTGAAAACGACGAATTTATCATTCCAGTCA<br>TCAACGACACAAAATCAATGACAACGTCTCTTTGACACAAA<br>ATATCAAAGCTGCCGAAAGGAATCATTAAAAACAAAATAAA<br>AACTTACGCAAGAAGTGCTCATTTCAGTAATAGAAAAAATT<br>ATCTTCCCTGACAATTAGATGATTTTTCAGTAAAAGATATATT<br>TGATCACATCTGGGACGGATGAAGGCGACTTTTCATGAAATA<br>CCAAACGTGCCCGACAGGAGAAGGGTCGTACGATCACGCTG<br>AGCAAGGAAGTGCGTCGACATTCGGAAGGATGTCACTGAAG<br>ACTCATGTTATGTGCTCCTGTAAGGTAGACTCAAAATCCTTCG<br>ACAGGAATGGCAGCGTAATAGGTCCCAGAATTTCGTTGACAT<br>GCCGTTGTTCCACGAGTGAACCACGGACAACCGTCACAGAG<br>GTCTGAAATTAAACAAATTGCGCCTCAGATCATCGGCGTTAG<br>GGACGGGCCCGTCTCTAACGCCTATGCGCTTGTCATCGAAGC<br>TCTGAAGACCACGGCACCAACGCAGTCGCTGCTGACGATGTT<br>CGGTAGCTAGTGGTATCCGGAGGATTGGCCATCTCGGGTGTA<br>GTCTACGCGCCTGTATAGATCTAGCAGTGATTTGGACGGACA<br>TTTCCATACCGGTAACTTGGTGAATATCGGATAGTGACACGT<br>CATGTGCTATTCGTGCAGACCTCTCACGAGTGCTCAGTGTTCC<br>CGTTCTGGGGTTTCCCAAAACGTTTAGTTAGCCGCTTGCTGCT<br>GAATTCTTCCATTCTCGACCCATCCCCATTAGCATCGTCCAAT<br>TGTGGCTAACCCCGTTCCAAATGAAGAATGGTGTGTTGATTG<br>GACCATCCGGTTTCCTTCGACCCGATTGGTTCGTCCCCTTTCG<br>AAATATCTAATTATCCGAAGCTGTCGTGGAATCGACGTCCAG<br>GCATTATCGCAATAAATGAAGATAAACGCTATCAAAATTAA<br>ATGCAGCTTTTGCCCTTCTGAGAGAGTTTTGAGCGCAATGTG<br>CATCGACATCCCATTGACACAAAATATCAGTACAGTAACAA<br>CGTATTTTGAGAGCCCTGGCCTCAAATTTTAATCATTTTTCAT

							ATTCGGTCTTAAGACACTTGCAGGCGAAGTTTCATCGCGATT<br>AGAATATTCTTTCGTTGTATTTCGATATTTTTGTGAAACAATG<br>TATTGTGTTTTTACATATGTCCGTTCGGTTATTGCTCCGTTATT<br>GTTCACTGCGTGTTCCATCCTAACTCTTATACTCCAAACAATG<br>CACCAAAATACGAACAGACTTATGTCACAACCCAATATTTGC<br>ATAATTTTTCTTTTTTGTGGAAGAAAATGCTGGAATTCTGTGG<br>TATGTCTTCAATTTAAAACACAGGCATTAAGAGGAAAACGAT<br>GAGACACACCTGTTTTGAATATAAAGCTTTAAAGCAATAATT<br>ACATAAAATAACCCTTCTTGTTGTTTTTTGAAGTTTGAAAAGT<br>ATCAAGATTTCTAAATTCTATCAAAAAAGCTCACAGTATTTG<br>ACTTCACTTTTTAACCATTTAAATTTTTTATGTGATGCTGTAAC<br>CCCCCCCCCCCGAAAAAAAGGAGATGTTTTTTTTGACAAATG<br>TACTTACGATGTATATTTGTACATTGTTGTACATTGTATGGTA<br>TATATAATAAGTTCGAGATAACGATCTTTGCTGTGAAATTTCT<br>GATTTAGTCGTATCTCTCATGCAGCCTTCTAATAGTCTTAAAT<br>AGCACAACCTTTTGTCTTTATCAGTTAGCTCTGTTTAATTAAT<br>CTGGAAAGAAACCTAACAAGTTGATATTTTATATTTAATCCA<br>TCGAAGAGAATTATTATGCATTTCGCAGCAGTAGTACCCGTT<br>TAACGACTTGATTATAACTAAGAACTGTCACTGAAGAAGTCA<br>CTGGAGGTGTCGGAGTGAATCTTGTTCTCTATCCAGTTTACGT<br>ATGCCGACACTCTGGTACAGACACCAGGATATCCTGGAAGG<br>CCGCAGCCCTCTCCGAAAGAGACAATTCCAAACAAAGTAAT<br>CCTTCTATTCTTCCACATAACGAGAGGTCCTCCGGAATCAGC<br>CTGACAAGAATCAAGTCCTGTACTGCCAGCGCATACCATATT<br>TCCAGTGATTATATTACTCTCCACTCCTTCGTACAGAGAGCTG<br>TAGACTTTGTCGCATTCTCCTCTGGAAAGCATGGGAACGCGA<br>ACCGAGCGGAGCACATCAGAGGTGTCGCCACCTTCAGTGGT<br>GTGTCCCCATCCAGCAACCATAGCCACCAAGCTGGGTTCCGT

							CTCGTTCTGCGTTGGAAGACAGATCCCATTCACGAATCCTTC<br>CGATCCACCGAAATCGATGGGATCCGCCATTTTCAGGAGGG<br>CGATATCGTGTTCGTTTCGATGCTTGCCATAGTCCTCGTGTAG<br>GATGATCTTGGAAATCTCGTGCCTCTTAACTTGCTTTGCCGCC<br>TTCCTTGACAGACGATGCAGCCCAACGTAGACCTCGTGCACT<br>TCGGGTTTATCTGGGTAATCTATGCAATGTGCAGCTGTCAAA<br>ATCCAACGTTCGTTCAGGATGGAGGCGCCACAGACGTGGTA<br>AAATTTTTTATCATATTTTTCATGCAGTGTGACCATCCACGGA<br>AAGAATCCTTCCCTGACCGGTAATCCACCCACGATCCTGCCC<br>TGGTTATTTAGATTCTTGCCACAGTTTCTTACTGTCAGTCCTTA<br>TAAAATGAAGCAGAAATTAATCTTACAAAGGGTCCACGGAT<br>ATAAAATAATAGTTCTCTATGTGCATAAAATGTTACCTATAC<br>GAGACGCATTTCCAAGCATAAAACATAGCTGCAGAATCCAG<br>TAAAACCTAGAACGAAAGTCAGATGTTTCAAACCAATATAA<br>GAATTTCGCAATTTACTGAACAATCCATCTCCTCTTAAACAA<br>CAACAACAACGAAAATCTGATTGTCAACTATTTTGAAA

PhdEnzSeP 43	Q9TXD8	8,38E-53	43,320	PF00089	31452.82<br><br>**29400.30**	MKLYWILQLCFILG<br>NASG**TGAEGVTVS**<br>**NCGVKQKNEGRIV**<br>**GGKPLREGLLPWM**<br>**VTLHVKYDKEFEHV**<br>**CGGSILNERWIVTA**<br>**AHCILDPDKPELHEI**<br>**YVGLHHLSKKAAK**<br>**QVKKHEISKIIPHED**<br>**YHSTPQIRDDIALL**<br>**KTADPIDFGGSEGF**<br>**VNGICLPTQNEIGP**<br>**TTIAAVAGWGHTT**<br>**HDGKTSDVLRGVR**<br>**VPIVSLEKCKSAYRF**<br>**YDRVITDKMICAG**<br>**DVGLDSCQADSGG**<br>**PLMVRKAGRATLY**<br>**GIVSFGEGCGSPGD**<br>**PGVYTKVSAYVDW**<br>**IENKINSDTTSDSN**<br>**DSS***	ATTTTCATCCTTGGAAAATCTCCATATAAAAATATCTTTATTG<br>CAAATTTATTTATTTGAAAACGACGAATTTATCATTCCAGTCA<br>TCAACGACACAAAATCAATGACAACGTCTCTTTGACACAAA<br>ATATCAAAGCTGCCGAAAGGAATCATTAAAAACAAAATAAA<br>AACTTACGCAAGAAGTGCTCATTTCAGTAATAGAAAAAATT<br>ATCTTCCCTGACAATTAGATGATTTTTCAGTAAAAGATATATT<br>TGATCACATCTGGGACGGATGAAGGCGACTTTTCATGAAATA<br>CCAAACGTTCTCGGCAGAAGAAGTGTCGTACGATCTCGCTGA<br>TCAAGGGACAGCGTCGACAGTGCGAAGGAAGTCACTGAAGA<br>CCCGCGCTGTGGGCTGCTGTAAAATAGATTCAGAATACTTCG<br>ACAGGAATGGCAACACAATAGGTCTTAGAATTTCGTTGACAT<br>ACCGTTGTTCTATGAGTGTGCTACGGAGGACCATCACAGAGG<br>CCTGAAAGTAACAAATGGTGTCTCAGATCATAGGCGTTAGG<br>GACGGGTCGTCCCTAACGTCTATGTGCTTGTCGTCGAAGTTC<br>AGTATTAGACCCCAGCACCATCGCAATCGCTGCTGACGATGT<br>TCGGTAGCAAGTGATAGAAATTTTTCATGTCAAGGCTGTAAA<br>GCCCCTCCTCCTCCCCGAAAGAAAAGGAGAGGTGTGTTGTAC<br>TTAAGATGTATACTTGTAAATCGTATGATATGTATAATAAGTT<br>CAAAATAACGATATTTGTTGTGAAATTTCTGATTTAGACGTA<br>CCTGTCATGCAGCCTTCTTATAGCCTTAAAAATAACACAACC<br>TTTTTTTCTTTATCAGATAGCTCTGTTTAATGAATGAGGAAAG<br>AAATGTAACAAGTCGTAACTTTATATTTAATGCATCGAAGAG<br>AACCATTATACATTTCTCAGCAATGGTATCGCTTTACGCCATC<br>ATTATAACTAAGAACTGTCATTAGAGTCACTGGTGGTGTCGG<br>AGTTAATCTTGTTCTCTATCCAGTCTACGTATGCAGACACTTT<br>GGTATAGACACCAGGATCTCCTGGAGAGCCGCACCCCTCTCC<br>GAAAGACACAATTCCATACAAAGTTGCCCTTCCAGCCTTTCG<br>CACCATGAGGGGTCCTCCGGAATCAGCCTGACAGGAATCGA

							GTCCTACATCGCCAGCGCAAATCATTTTGTCAGTGATCACTCT<br>ATCGTAAAATCTGTACGCTGATTTGCACTTTTCTAAGGAAACT<br>ATGGGTACTCTGACTCCACGAAGCACATCGGAGGTTTTGCCA<br>TCGTGAGTGGTGTGCCCCCATCCGGCGACCGCAGCTATCGTG<br>GTGGGTCCTATCTCGTTCTGCGTCGGAAGACAGATCCCATTC<br>ACGAACCCTTCTGATCCACCGAAGTCAATGGGATCCGCTGTT<br>TTCAGGAGGGCGATGTCGTCTCTGATCTGGGGTGTGCTGTGG<br>TAATCCTCGTGTGGGATGATCTTGGAAATCTCGTGCTTCTTAA<br>CTTGCTTTGCCGCCTTCTTTGACAGATGATGCAGTCCAACGTA<br>GATCTCATGTAATTCGGGTTTATCTGGGTCCAATATGCAATGT<br>GCAGCTGTCACAATCCAACGCTCGTTCAAGATGGAGCCGCC<br>ACAGACATGCTCAAATTCTTTATCGTATTTGACATGCAGAGT<br>GACCATCCACGGAAGTAGTCCTTCTCTGAGCGGTTTTCCGCC<br>CACGATTCTGCCCTCGTTTTTTTGTTTCACGCCACAGTTACTTA<br>CTGTTACGCCTTCGGCTCCTGTACCAGACGCATTTCCAAGTAT<br>AAAACATAGCTGCAGGATCCAGTAGAGCTTCATCTGCAATGT<br>ACCCGGTGCTTAGTGTCGACCTCCTCTTGTATCCATTTGAAGA<br>CCTTGAGAAACACTTGTTTTAAAATTGGAAATATCGGCGACA<br>ATTTGAAAACTGCACCACATGCTGGAAATTTTGAAAGCTTTG<br>ACGGAGCATAATAATTGCAGATGTTCAATATTTGATGTCTTG<br>CAGGCATCTCATCAGACATCTCATTTGCATGATGCAGATGAG<br>ATGTCTGACATTTGATGTCTTGCAGACGAGAGTCATTCAGGC<br>AATTTTAGAAGTTATTTCTTCTATTATTTAGCTATCCAGATTG<br>ACGTGAATTTTAATCATTTGCAAGACTACCTGCATTTCTGTGT<br>AAAGAAAAATGAGTGATTTTAGAAGAACGAAATAAACTCTC<br>CACAGATGTATACGTTTAATACGAGCAGTGCCGATATCGTAA<br>AACACTTTAGTTAAAATGTAAAAGAAGCTTTCCTCGTTGAAG<br>ATTGTTATATGAAACCTGAATTACAAACGGGTTCCGTTAGAG

							AGACTGTGAAAATGCAAAAAAATAAAGAAACTTACAGAGACTGAGCTTTAAAAATGTATCCCCATAAAAAAATACAAGGTACTTTCGATAATTTTTGGAACTCACGAAAAGTATAATGTTATAAAATTAAGATCGCAAAAATCGCTGTAGTTCTACTTTAGATGACTGATTATCCAGCAATAAAACATTGTTTTTCAATTGCTTTGTAGTACTTAAGCCACTCAATTTTCGCAAAGTTATAGTAGTCATTTACTTAGGGCGTTATTCACCAACCATTCGATCCGCGGACCAAACCGCGAGTCGGAGTTATCGTAATACCTAATGTACGCGATAACTCCGACTCGCGGCCCAATCCACGGATCAAATGGTTGGTGAATAAAGTGGTAAATTTAGAGGGACATCGGATTGTCCCAACTTGGGAAAAAACAAGCAAAGAAATATATGATATTATCAAAGGTTTTCGGATCAGAGACTTCATGTACGGATGATAGGGTAGTGCAAATGTTCTTCTCTTATTTAACGATTTATTAAAAGTGGGTGATTAGATCTTTCCTTATACGATATTTCCTAAAAGTTCTCTCCCCCAGATC

PhdEnzSeP 44	Q9TXD8	2,62E-51	43,256	PF00089	31052,58	MDDVSQGLHMGYK<br>RRSTAGTRYMSDEVS<br>DKFENIMWIYWILLL<br>CFILGNASRIGERVT<br>VRNCGKHLNHHGR<br>IVGGKPIKEGRFPW<br>MVTLHEKYDRKFEH<br>VCGGSILNARWIVT<br>AAHCITDPDEPEVLE<br>IYVGLHRLSRKAAK<br>QVKRHEISKIIVHEG<br>FDWIQLQDDIALLKT<br>ADPIDFGGSEGFVN<br>GICLPTQNEGEPSSV<br>ALVAGWGHTTEGG<br>DTSDVLRAVRVPLIS<br>RKLCAAKYSSRYGV<br>GAITENMLCAGDVG<br>LDSCQADSGGPLMV<br>RKAGRATLYG	TTCCATACAAAGTTGCCCTTCCAGCCTTTCGCACCATGAGGG<br>GTCCTCCGGAATCAGCCTGACAGGAATCGAGCCCTACATCGC<br>CAGCGCATAACATATTTTCAGTGATCGCACCCACTCCATAAC<br>GAGAGCTGTACTTTGCGGCGCATAATTTTCTGGAAATCAAGG<br>GTACTCTAACTGCACGGAGCACATCGGAGGTGTCGCCACCTT<br>CAGTGGTGTGTCCCCACCCGGCGACCAGAGCCACCGAGCTG<br>GGTTCGCCCTCGTTCTGCGTCGGAAGACAGATCCCATTCACG<br>AATCCTTCCGATCCACCGAAGTCGATGGGATCCGCTGTTTTC<br>AGAAGAGCGATGTCATCTTGGAGCTGGATCCAGTCAAAACC<br>CTCGTGTACGATGATCTTGGAAATCTCGTGCCTCTTAACTTGC<br>TTTGCCGCCTTCCTTGACAGACGATGCAGTCCAACGTAGATC<br>TCAAGCACTTCGGGTTCATCTGGGTCAGTTATGCAATGTGCA<br>GCTGTCACAATCCAACGTGCGTTCAAGATAGAGCCGCCACA<br>GACGTGCTCAAATTTTCTATCGTATTTTTCATGCAGTGTGACC<br>ATCCACGGAAAGCGTCCTTCCTTGATCGGTTTTCCACCCACG<br>ATCCTGCCGTGGTGATTGAGATGCTTGCCACAGTTTCTTACTG<br>TCACTCTTTCGCCTATTCGAGACGCATTTCCAAGTATAAAAC<br>ATAGCAGTAGAATCCAGTAAATCCACATAATGTTCTCGAACT<br>TGTCACTTACTTCATCTGACATGTATCTGGTGCCAGCCGTCGA<br>CCTCCTCTTGTACCCCATATGAAGACCTTGAGAAACATCATC<br>CATGGAAATAATCCTCTCTCTCTCTTCCTTTACCGGTATTCCA<br>CCAGAAATCTTGGGTTGACCATAACGTTTACCACAGTTCCTT<br>ACTATGACTACTCTGCCCTATAATGTTCTTCATCTTGTCCTAG<br>CGTCAAGACCTGGTGCCTATATTAGAAAG

PhdEnzSeP 45	P84033	8,96E-50	42,791	PF00089	28373,52	MSDEVSDKFESVMWLYWILQLCFVLGNASGTGERVTVRNCGTLLNNQGRIVGGLPVREGLFPWMVTLHEKYDTKLHHVCGASILNERWVVTAAHCISDPNKPELFEIYVGLHRLSRKAAKQVKRHEISKIIVHEGFSRFHHAGHDIALLKIADRIDFGGSEGFVNGICLPTQNEGEPSSVALVAGWGHTTEGGDTSDVLRAVRVPLISRKLCAAKYSSRYGVGAITENMLCAGDVGLDSCQADSGGPLMVRKAGRATLYG	TTCCATACAAAGTTGCCCTTCCAGCCTTTCGCACCATGAGGGGTCCTCCGGAATCAGCCTGACAGGAATCGAGCCCTACATCGCCAGCGCATAACATATTTTCAGTGATCGCACCCACTCCATAACGAGAGCTGTACTTTGCGGCGCATAATTTTCTGGAAATCAAGGGTACTCTAACTGCACGGAGCACATCGGAGGTGTCGCCACCTTCAGTGGTGTGTCCCCACCCGGCGACCAGAGCCACCGAGCTGGGTTCGCCCTCGTTCTGCGTCGGAAGACAGATCCCATTCACGAATCCTTCCGATCCACCGAAGTCGATGCGATCGGCTATTTTCAGAAGAGCGATGTCATGTCCAGCGTGGTGGAACCTGCTAAAGCCCTCGTGTACGATGATCTTGGAAATCTCGTGCCTCTTAACTTGCTTTGCCGCCTTCCTTGACAGACGATGCAGTCCAACGTAGATCTCAAACAATTCGGGTTTATTTGGGTCGGATATGCAATGTGCAGCTGTCACAACCCAACGTTCGTTCAGGATGGAGGCCCCACAGACGTGGTGTAATTTTGTATCGTATTTTTCATGCAGTGTGACCATCCATGGAAAAAGTCCTTCTCTGACAGGTAATCCACCTACGATCCTGCCCTGGTTATTTAGTAGCGTGCCACAGTTTCTTACTGTCACTCTTTCACCTGTACCAGACGCATTTCCAAGTACAAAACATAGCTGTAGGATCCAGTAAAGCCACATAACGCTCTCGAACTTGTCACTTACTTCATCTGACATCTATCTGGTGCTTGCCGTTGGCCTCCTCCTCTCGTACTCATTGTAAGACCTTGAGAAACTGGAACTATCGGTGAGAATTTGAAAAGTGCACCACATGCTGAAATCTTCGAAAGCTTTCACGGAACATAATAATTGCAGATGTGCGTAAGTTTGACGTCTTTCATGCA

PhdEnzSeP46	Q7M4I3	2,31E-08	51,163	PF00089	6355	DSGGPLVMWKDGRATLFGIVSFGEGCGLPGYPGVCTRVSAYVDWIENKIHSDTSSDSSGSS*	ATTTTCATCCTTGGAAAATCTCCATATAAAAATATCTTTATTGCAAATTTATTTATTTGAAAACGACGAATTTATCATTCCAGTCATCAACGACACAAAATCAATGACAACGTCTCTTTGACACAAAATATCAAAGCTGCCGAAAGGAATCATTAAAAACAAAATAAAAACTTACGCAAGAAGTGCTCATTTCAGTAATAGAAAAAATTATCTTCCCTGACAATTAGATGATTTTTCAGTAAAAGATATATTTGATCACATCTGGGACGGATGAAGGCGACTTTTCATGAAATACCAAACGTTCTCGGCAGAAGAAGTGTCGTACGATCTCGCTGATCAAGGGACAGCGTCGACAGTGCGAAGGAAGTCACTGAAGACCCGCGCTGTGGGCTGCTGTAAAATAGATTCAGAATACTTCGACAGGAATGGCAACACAATAGGTCTTAGAATTTCGTTGACATACCGTTGTTCTATGAGTGTGCTACGGAGGACCATCACAGAGGCCTGAAAGTAACAAATGGTGTCTCAGATCATAGGCGTTAGGGACGGGTCGTCCCTAACGTCTATGTGCTTGTCGTCGAAGTTCAGTATTAGACCCCAGCACCATCGCAATCGCTGCTGACGATGTTCGGTAGCAAGTGATAGAAATTTTTCATGTCAAGGCTGTAAAGCCCCTCCTCCTCCCCGAAAGAAAAGGAGAGGTGTGTTGTACTTAAGATGTATACTTGTAAATCGTATGATATGTATAATAAGTTCAAAATAACGATATTTGTTGTGAAATTTCTGATTTAGACGTACCTGTCATGCAGCCTTCTTATAGCCTTAAAAATAACACAACCTTTTTTTCTTTATCAGATAGCTCTGTTTAATGAATGAGGAAAGAAATGTAACAAGTCGTAACTTTATATTTAATGCATCGCTGAGAACCATTATACATTTCTTAGCAATGGCACCCATTTTACGACATCGTTACAACTAAGAACTGCCACTGGAGTCACTGGACGTGTCGGAGTGAATCTTGTTCTCTATCCAGTCTACGTACGCAGACACTCTGGTACAGACACCAGGATATCCTGGAAGGCCGCAGCCCTCTCCGAAAGACACAATTCCAAACAAAGTTGCCCTTCCATCTTTCCACATAACGAGAGGTCCTCCGGAATCAG

PhdEnzSeP 47	P84033	3,35E-51	43,925	PF00089	31052,58	MDDVSQGLHMGYKRRSTAGTRYMSDEVSDKFENIMWIYWILLLCFILGNASRIGERVTVRNCGKHLNHHGRIVGGKPIKEGRFPWMVTLHEKYDRKFEHVCGGSILNARWIVTAAHCITDPDEPEVLEIYVGLHRLSRKAAKQVKRHEISKIIVHEGFDWIQLQDDIALLKTADPIDFGGSEGFVNGICLPTQNEGEPSSVALVAGWGHTTEGGDTSDVLRAVRVPLISRKLCAAKYSSRYGVGAITENMLCAGDVGLDSCQADSGGPLMVRKAGRATLYG	TTCCATACAAAGTTGCCCTTCCAGCCTTTCGCACCATGAGGGGTCCTCCGGAATCAGCCTGACAGGAATCGAGCCCTACATCGCCAGCGCATAACATATTTTCAGTGATCGCACCCACTCCATAACGAGAGCTGTACTTTGCGGCGCATAATTTTCTGGAAATCAAGGGTACTCTAACTGCACGGAGCACATCGGAGGTGTCGCCACCTTCAGTGGTGTGTCCCCACCCGGCGACCAGAGCCACCGAGCTGGGTTCGCCCTCGTTCTGCGTCGGAAGACAGATCCCATTCACGAATCCTTCCGATCCACCGAAGTCGATGGGATCCGCTGTTTTCAGAAGAGCGATGTCATCTTGGAGCTGGATCCAGTCAAAACCCTCGTGTACGATGATCTTGGAAATCTCGTGCCTCTTAACTTGCTTTGCCGCCTTCCTTGACAGACGATGCAGTCCAACGTAGATCTCAAGCACTTCGGGTTCATCTGGGTCAGTTATGCAATGTGCAGCTGTCACAATCCAACGTGCGTTCAAGATAGAGCCGCCACAGACGTGCTCAAATTTTCTATCGTATTTTTCATGCAGTGTGACCATCCACGGAAAGCGTCCTTCCTTGATCGGTTTTCCACCCACGATCCTGCCGTGGTGATTGAGATGCTTGCCACAGTTTCTTACTGTCACTCTTTCGCCTATTCGAGACGCATTTCCAAGTATAAAACATAGCAGTAGAATCCAGTAAATCCACATAATGTTCTCGAACTTGTCACTTACTTCATCTGACATGTATCTGGTGCCAGCCGTCGACCTCCTCTTGTACCCCATATGAAGACCTTGAGAAACATCATCCATGGAAATAATCCTCTCTCTCTCTTCCTTTACCGGTATTCCACCAGAAATCTTGGGTTGACCATAACGTTTACCACAGTTCCTTACTATGACTACTCTGCCAAACGTATTTCCAAGTACGAAACACAGCTGCAGAACAGCACAAAACCTTTCAAATAAGAATGTGTGTTAAGA

PhdEnzSeP48	Q9TXD8	9,22E-53	43,320	PF00089	14042,94	ISKIIVHEGFSRFHHAGHDIALLKIADRIDFGGSEGFVNGICLPTQNEGEPSSVALVAGWGHTTEGGDTSDVLRAVRVPLISRKLCAAKYSSRYGVGAITENMLCAGDVGLDSCQADSGGPLMVRKAGRATLYG	TTCCATACAAAGTTGCCCTTCCAGCCTTTCGCACCATGAGGGGTCCTCCGGAATCAGCCTGACAGGAATCGAGCCCTACATCGCCAGCGCATAACATATTTTCAGTGATCGCACCCACTCCATAACGAGAGCTGTACTTTGCGGCGCATAATTTTCTGGAAATCAAGGGTACTCTAACTGCACGGAGCACATCGGAGGTGTCGCCACCTTCAGTGGTGTGTCCCCACCCGGCGACCAGAGCCACCGAGCTGGGTTCGCCCTCGTTCTGCGTCGGAAGACAGATCCCATTCACGAATCCTTCCGATCCACCGAAGTCGATGCGATCGGCTATTTTCAGAAGAGCGATGTCATGTCCAGCGTGGTGGAACCTGCTAAAGCCCTCGTGTACGATGATCTTGGAAATCTAAGAAAGTAACAGTGCTATGCAGTGGGACTGATTTTCGGGTGGGCAAACTGACCGCTTGCCCAGGACCCACTCCATTCAGGGCCCCATAATTTTCTAGTCTTCATTTAAAATTTTGTTTAAAGTATCCGACTACATTCGGAACAATTTTCTTTCCTCGTGCCTCTTAACTTGCTTTGCCGCCTTCCTTGACAGACGATGCAGTCCAACGTAGATCTCAAACAATTCGGGTTTATTTGGGGAAGGAAACAGACGCTTACTCGGATATGCAATGTGCAGCTGTCACAATCCAACGTTCGTTCAAGATGGAGGCCCCACAGACGTGGTGTAATTTTGTATCGTATTTTTCATGCAGTGTGACCAT

PhdEnzSeP49	P84033	3,46E-22	43,609	PF00089	14042,94	ISKIIVHEGFSRFHHAGHDIALLKIADRIDFGGSEGFVNGICLPTQNEGEPSSVALVAGWGHTTEGGDTSDVLRAVRVPLISRKLCAAKYSSRYGVGAITENMLCAGDVGLDSCQADSGGPLMVRKAGRATLYG	TTCCATACAAAGTTGCCCTTCCAGCCTTTCGCACCATGAGGGGTCCTCCGGAATCAGCCTGACAGGAATCGAGCCCTACATCGCCAGCGCATAACATATTTTCAGTGATCGCACCCACTCCATAACGAGAGCTGTACTTTGCGGCGCATAATTTTCTGGAAATCAAGGGTACTCTAACTGCACGGAGCACATCGGAGGTGTCGCCACCTTCAGTGGTGTGTCCCCACCCGGCGACCAGAGCCACCGAGCTGGGTTCGCCCTCGTTCTGCGTCGGAAGACAGATCCCATTCACGAATCCTTCCGATCCACCGAAGTCGATGCGATCGGCTATTTTCAGAAGAGCGATGTCATGTCCAGCGTGGTGGAACCTGCTAAAGCCCTCGTGTACGATGATCTTGGAAATCTAAGAAAGTAACAGTGCTATGCAGTGGGACTGATTTTCGGGTGGGCAAACTGACCGCTTGCCCAGGACCCACTCCATTCAGGGCCCCATAATTTTCTAGTCTTCATTTAAAATTTTGTTTAAAGTATCCGACTACATTCGGAACAATTTTCTTTCCTCGTGCCTCTTAACTTGCTTTGCCGCCTTCCTTGACAGACGATGCAGTCCAACGTAGATCTCAAACAATTCGGGTTTATTTGGGGAAGGAAACAGACGCTTACTCGGATATGCAATGTGCAGCTGTCACAATCCAACGTTCGTTCAAGATGGAGGCCCCACAGACGTGGTGTAATTTTGTATCGTATTTTTCATGCAGTGTGACCAT

PhdEnzSeP 50	Q9TXD8	3,04E-58	42,276	PF00089	30924,11	MLGNASRIGLTVRNCGKNLNNQGRIVGGLPVREGFFPWMVTLHEKYDKKFYHVCGASILNERWILTAAHCIDYPDKPEVHEVYVGLHRLSRKAAKQVKRHEISKIILHEDYGKHRNEHDIALLKMADPIDFGGSEGFVNGICLPTQNETEPSLVAMVAGWGHTTEGGDTSDVLRSVRVPMLSRGECDKVYSSLYEGVESNIITGNMVCAGSTGLDSCQADSGGPLVMWKNRRITLFGIVSFGEGCGLPGYPGVCTRVSAYVNWIENKIHSDTSSDFFSDSS*	ATTTTCATCCTTGGAAAATCTCCATATAAAAATATCTTTATTGCAAATTTATTTATTTGAAAACGACGAATTTATCATTCCAGTCATCAACGACACAAAATCAATGACAACGTCTCTTTGACACAAAATATCAAAGCTGCCGAAAGGAATCATTAAAAACAAAATAAAAACTTACGCAAGAAGTGCTCATTTCAGTAATAGAAAAAATTATCTTCCCTGACAATTAGATGATTTTTCAGTAAAAGATATATTTGATCACATCTGGGACGGATGAAGGCGACTTTTCATGAAATACCAAACGTGCCCGACAGGAGAAGGGTCGTACGATCACGCTGAGCAAGGAAGTGCGTCGACATTCGGAAGGATGTCACTGAAGACTCATGTTATGTGCTCCTGTAAGGTAGACTCAAAATCCTTCGACAGGAATGGCAGCGTAATAGGTCCCAGAATTTCGTTGACATGCCGTTGTTCCACGAGTGAACCACGGACAACCGTCACAGAGGTCTGAAATTAAACAAATTGCGCCTCAGATCATCGGCGTTAGGGACGGGCCCGTCTCTAACGCCTATGCGCTTGTCATCGAAGCTCTGAAGACCACGGCACCAACGCAGTCGCTGCTGACGATGTTCGGTAGCTAGTGGTATCCGGAGGATTGGCCATCTCGGGTGTAGTCTACGCGCCTGTATAGATCTAGCAGTGATTTGGACGGACATTTCCATACCGGTAACTTGGTGAATATCGGATAGTGACACGTCATGTGCTATTCGTGCAGACCTCTCACGAGTGCTCAGTGTTCCCGTTCTGGGGTTTCCCAAAACGTTTAGTTAGCCGCTTGCTGCTGAATTCTTCCATTCTCGACCCATCCCCATTAGCATCGTCCAATTGTGGCTAACCCCGTTCCAAATGAAGAATGGTGTGTTGATTGGACCATCCGGTTTCCTTCGACCCGATTGGTTCGTCCCCTTTCGAAATATCTAATTATCCGAAGCTGTCGTGGAATCGACGTCCAGGCATTATCGCAATAAATGAAGATAAACGCTATCAAAATTAAATGCAGCTTTTGCCCTTCTGAGAGAGTTTTGAGCGCAATGTGCATCGACATCCCATTGACACAAAATATCAGTACAGTAACAACGTATTTTGAGAGCCCTGGCCTCAAATTTTAATCATTTTTCAT

							ATTCGGTCTTAAGACACTTGCAGGCGAAGTTTCATCGCGATT<br>AGAATATTCTTTCGTTGTATTTCGATATTTTTGTGAAACAATG<br>TATTGTGTTTTTACATATGTCCGTTCGGTTATTGCTCCGTTATT<br>GTTCACTGCGTGTTCCATCCTAACTCTTATACTCCAAACAATG<br>CACCAAAATACGAACAGACTTATGTCACAACCCAATATTTGC<br>ATAATTTTTCTTTTTTGTGGAAGAAAATGCTGGAATTCTGTGG<br>TATGTCTTCAATTTAAAACACAGGCATTAAGAGGAAAACGAT<br>GAGACACACCTGTTTTGAATATAAAGCTTTAAAGCAATAATT<br>ACATAAAATAACCCTTCTTGTTGTTTTTTGAAGTTTGAAAAGT<br>ATCAAGATTTCTAAATTCTATCAAAAAAGCTCACAGTATTTG<br>ACTTCACTTTTTAACCATTTAAATTTTTTATGTGATGCTGTAAC<br>CCCCCCCCCCCGAAAAAAAGGAGATGTTTTTTTTGACAAATG<br>TACTTACGATGTATATTTGTACATTGTTGTACATTGTATGGTA<br>TATATAATAAGTTCGAGATAACGATCTTTGCTGTGAAATTTCT<br>GATTTAGTCGTATCTCTCATGCAGCCTTCTAATAGTCTTAAAT<br>AGCACAACCTTTTGTCTTTATCAGTTAGCTCTGTTTAATTAAT<br>CTGGAAAGAAACCTAACAAGTTGATATTTTATATTTAATCCA<br>TCGAAGAGAATTATTATGCATTTCGCAGCAGTAGTACCCGTT<br>TAACGACTTGATTATAACTAAGAACTGTCACTGAAGAAGTCA<br>CTGGAGGTGTCGGAGTGAATCTTGTTCTCTATCCAGTTTACGT<br>ATGCCGACACTCTGGTACAGACACCAGGATATCCTGGAAGG<br>CCGCAGCCCTCTCCGAAAGAGACAATTCCAAACAAAGTAAT<br>CCTTCTATTCTTCCACATAACGAGAGGTCCTCCGGAATCAGC<br>CTGACAAGAATCAAGTCCTGTACTGCCAGCGCATACCATATT<br>TCCAGTGATTATATTACTCTCCACTCCTTCGTACAGAGAGCTG<br>TAGACTTTGTCGCATTCTCCTCTGGAAAGCATGGGAACGCGA<br>ACCGAGCGGAGCACATCAGAGGTGTCGCCACCTTCAGTGGT<br>GTGTCCCCATCCAGCAACCATAGCCACCAAGCTGGGTTCCGT

							CTCGTTCTGCGTTGGAAGACAGATCCCATTCACGAATCCTTC<br>CGATCCACCGAAATCGATGGGATCCGCCATTTTCAGGAGGG<br>CGATATCGTGTTCGTTTCGATGCTTGCCATAGTCCTCGTGTAG<br>GATGATCTTGGAAATCTCGTGCCTCTTAACTTGCTTTGCCGCC<br>TTCCTTGACAGACGATGCAGCCCAACGTAGACCTCGTGCACT<br>TCGGGTTTATCTGGGTAATCTATGCAATGTGCAGCTGTCAAA<br>ATCCAACGTTCGTTCAGGATGGAGGCGCCACAGACGTGGTA<br>AAATTTTTTATCATATTTTTCATGCAGTGTGACCATCCACGGA<br>AAGAATCCTTCCCTGACCGGTAATCCACCCACGATCCTGCCC<br>TGGTTATTTAGATTCTTGCCACAGTTTCTTACTGTCAGTCCTAT<br>ACGAGACGCATTTCCAAGCATAAAACATAGCTGCAGAATCC<br>AGTAAAACCTAGAACGAAAGTCAGATGTTTCAAACCAATAT<br>AAGAATTTCGCAATTTACTGAACAATCCATCTCCTCTTAAAC<br>AACAACAACAACGAAAATCTGATTGTCAACTATTTTGAAA

PhdEnzSeP 51	Q9TXD8	6,70E-58	42,276	PF00089	34159.86<br>**30391.45**	MSTRGGGQRQAPD RCQMKLYWILQLCF VLGNASG**TGERVTV RNCGTLLNNQGRI VGGLPVREGLFPW MVTLHEKYDKKFY HVCGASILNERWIL TAAHCIDYPDKPEV HEVYVGLHRLSRK AAKQVKRHEISKIIL HEDYGKHRNEHDI ALLKMADPIDFGGS EGFVNGICLPTQNE TEPSLVAMVAGWG HTTEGGDTSDVLR SVRVPMLSRGECD KVYSSLYEGVESNII TGNMVCAGSTGLD SCQADSGGPLVMW KNRRITLFGIVSFGE GCGLPGYPGVCTR VSAYVNWIENKIHS DTSSDFFSDSS***	ATTTTCATCCTTGGAAAATCTCCATATAAAAATATCTTTATTG CAAATTTATTTATTTGAAAACGACGAATTTATCATTCCAGTCA TCAACGACACAAAATCAATGACAACGTCTCTTTGACACAAA ATATCAAAGCTGCCGAAAGGAATCATTAAAAACAAAATAAA AACTTACGCAAGAAGTGCTCATTTCAGTAATAGAAAAAATT ATCTTCCCTGACAATTAGATGATTTTTCAGTAAAAGATATATT TGATCACATCTGGGACGGATGAAGGCGACTTTTCATGAAATA CCAAACGTGCCCGACAGGAGAAGGGTCGTACGATCACGCTG AGCAAGGAAGTGCGTCGACATTCGGAAGGATGTCACTGAAG ACTCATGTTATGTGCTCCTGTAAGGTAGACTCAAAATCCTTCG ACAGGAATGGCAGCGTAATAGGTCCCAGAATTTCGTTGACAT GCCGTTGTTCCACGAGTGAACCACGGACAACCGTCACAGAG GTCTGAAATTAAACAAATTGCGCCTCAGATCATCGGCGTTAG GGACGGGCCCGTCTCTAACGCCTATGCGCTTGTCATCGAAGC TCTGAAGACCACGGCACCAACGCAGTCGCTGCTGACGATGTT CGGTAGCTAGTGGTATCCGGAGGATTGGCCATCTCGGGTGTA GTCTACGCGCCTGTATAGATCTAGCAGTGATTTGGACGGACA TTTCCATACCGGTAACTTGGTGAATATCGGATAGTGACACGT CATGTGCTATTCGTGCAGACCTCTCACGAGTGCTCAGTGTTCC CGTTCTGGGGTTTCCCAAAACGTTTAGTTAGCCGCTTGCTGCT GAATTCTTCCATTCTCGACCCATCCCCATTAGCATCGTCCAAT TGTGGCTAACCCCGTTCCAAATGAAGAATGGTGTGTTGATTG GACCATCCGGTTTCCTTCGACCCGATTGGTTCGTCCCCTTTCG AAATATCTAATTATCCGAAGCTGTCGTGGAATCGACGTCCAG GCATTATCGCAATAAATGAAGATAAACGCTATCAAAATTAA ATGCAGCTTTTGCCCTTCTGAGAGAGTTTTGAGCGCAATGTG CATCGACATCCCATTGACACAAAATATCAGTACAGTAACAA CGTATTTTGAGAGCCCTGGCCTCAAATTTTAATCATTTTTCAT

							ATTCGGTCTTAAGACACTTGCAGGCGAAGTTTCATCGCGATT<br>AGAATATTCTTTCGTTGTATTTCGATATTTTTGTGAAACAATG<br>TATTGTGTTTTTACATATGTCCGTTCGGTTATTGCTCCGTTATT<br>GTTCACTGCGTGTTCCATCCTAACTCTTATACTCCAAACAATG<br>CACCAAAATACGAACAGACTTATGTCACAACCCAATATTTGC<br>ATAATTTTTCTTTTTTGTGGAAGAAAATGCTGGAATTCTGTGG<br>TATGTCTTCAATTTAAAACACAGGCATTAAGAGGAAAACGAT<br>GAGACACACCTGTTTTGAATATAAAGCTTTAAAGCAATAATT<br>ACATAAAATAACCCTTCTTGTTGTTTTTTGAAGTTTGAAAAGT<br>ATCAAGATTTCTAAATTCTATCAAAAAAGCTCACAGTATTTG<br>ACTTCACTTTTTAACCATTTAAATTTTTTATGTGATGCTGTAAC<br>CCCCCCCCCCCGAAAAAAAGGAGATGTTTTTTTTGACAAATG<br>TACTTACGATGTATATTTGTACATTGTTGTACATTGTATGGTA<br>TATATAATAAGTTCGAGATAACGATCTTTGCTGTGAAATTTCT<br>GATTTAGTCGTATCTCTCATGCAGCCTTCTAATAGTCTTAAAT<br>AGCACAACCTTTTGTCTTTATCAGTTAGCTCTGTTTAATTAAT<br>CTGGAAAGAAACCTAACAAGTTGATATTTTATATTTAATCCA<br>TCGAAGAGAATTATTATGCATTTCGCAGCAGTAGTACCCGTT<br>TAACGACTTGATTATAACTAAGAACTGTCACTGAAGAAGTCA<br>CTGGAGGTGTCGGAGTGAATCTTGTTCTCTATCCAGTTTACGT<br>ATGCCGACACTCTGGTACAGACACCAGGATATCCTGGAAGG<br>CCGCAGCCCTCTCCGAAAGAGACAATTCCAAACAAAGTAAT<br>CCTTCTATTCTTCCACATAACGAGAGGTCCTCCGGAATCAGC<br>CTGACAAGAATCAAGTCCTGTACTGCCAGCGCATACCATATT<br>TCCAGTGATTATATTACTCTCCACTCCTTCGTACAGAGAGCTG<br>TAGACTTTGTCGCATTCTCCTCTGGAAAGCATGGGAACGCGA<br>ACCGAGCGGAGCACATCAGAGGTGTCGCCACCTTCAGTGGT<br>GTGTCCCCATCCAGCAACCATAGCCACCAAGCTGGGTTCCGT

							CTCGTTCTGCGTTGGAAGACAGATCCCATTCACGAATCCTTC<br>CGATCCACCGAAATCGATGGGATCCGCCATTTTCAGGAGGG<br>CGATATCGTGTTCGTTTCGATGCTTGCCATAGTCCTCGTGTAG<br>GATGATCTTGGAAATCTCGTGCCTCTTAACTTGCTTTGCCGCC<br>TTCCTTGACAGACGATGCAGCCCAACGTAGACCTCGTGCACT<br>TCGGGTTTATCTGGGTAATCTATGCAATGTGCAGCTGTCAAA<br>ATCCAACGTTCGTTCAGGATGGAGGCGCCACAGACGTGGTA<br>AAATTTTTTATCATATTTTTCATGCAGTGTGACCATCCATGGA<br>AAGAGTCCTTCTCTGACAGGTAATCCACCTACGATCCTGCCC<br>TGGTTATTTAGTAGCGTGCCACAGTTTCTTACTGTCACTCTTTC<br>ACCTGTACCAGACGCATTTCCAAGTACAAAACATAGCTGTAG<br>GATCCAGTAAAGCTTCATCTGACATCTATCTGGTGCTTGCCGT<br>TGGCCTCCTCCTCTCGTACTCATTGTAAGACCTTGAGAAACTG<br>GAACTATCGGTGAGAATTTGAAAAGTGCACCACATGCTGAA<br>ATCTTCGAAAGCTTTCACGGAACATAATAATTGCAGATGTGC<br>GTAAGTTTGACGTCTTTCATGCAGATGAGAGTCATTCAGACG<br>ATTTTAGGAGTTACTTTATCTCCTCTTTTATTTTTCTATCCGGA<br>TTACTTGACATGACTTTTAACCGTCTGCAAGATTCTACACACT<br>ATTTCTTTGTAAAGAAAAATGAGCGACTTTAAAAGGACAAA<br>ATGAAGTCTACACCTACACCCAGTGTTAGCCGATGCATTCCT<br>TCAACACGAACAGAGCCGACACCGTAAGACACTTCAGTTGA<br>AATGTAAAAGGAAACTTTCCTCGTTGAAGATCGTTTTATGAA<br>ACTTGAATTACAAGCGGGTTCCTTCAAAGAGTCTGATAAATT<br>ACAAAAAAAAGAAACTTACAGAGACTGAGCTTTAAAAATGT<br>ATCCCCATAAAAAAATACAAGGTACTTTCGATAATTTTTGGA<br>ACTCACGAAAAGTATAATGTTATAAAATTAAGATCGCAAAA<br>ATCGCTGTAGTTCTACTTTAGATGACTGATTATCCAGCAATAA<br>AACATTGTTTTTCAATTGCTTTGTAGTACTTAAGCCACTCAAT

							TTTCGCAAAGTTATAGTAGTCATTTACTTAGGGCGTTATTCACCAACCATTCGATCCGCGGACCAAACCGCGAGTCGGAGTTATCGTAATACCTAATGTACGCGATAACTCCGACTCGCGGCCCAATCCACGGATCAAATGGTTGGTGAATAAAGTGGTAAATTTAGAGGGACATCGGATTGTCCCAACTTGGGAAAAAACAAGCAAAGAAATATATGATATTATCAAAGGTTTTCGGATCAGAGACTTCATGTACGGATGATAGGGTAGTGCAAATGTTCTTCTCTTATTTAACGATTTATTAAAAGTGGGTGATTAGATCTTTCCTTATACGATATTTCCTAAAAGTTCTCTCCCCCAGATC

PhdEnzSeP 52	Q9TXD8	1,36E-19	46,602	PF00089	13547,2	HKSFLDDLSLSLSPSLLDGKTSDVLRGVRVPIVSLEKCKSAYRFYDRVITDKMICAGDVGLDSCQADSGGPLMVRKAGRATLYGIVSFGEGCGSPGDPGVYTKVSAYVDWIENKINSDTTSDSNDSS*	ATTTTCATCCTTGGAAAATCTCCATATAAAAATATCTTTATTGCAAATTTATTTATTTGAAAACGACGAATTTATCATTCCAGTCATCAACGACACAAAATCAATGACAACGTCTCTTTGACACAAAATATCAAAGCTGCCGAAAGGAATCATTAAAAACAAAATAAAAACTTACGCAAGAAGTGCTCATTTCAGTAATAGAAAAAATTATCTTCCCTGACAATTAGATGATTTTTCAGTAAAAGATATATTTGATCACATCTGGGACGGATGAAGGCGACTTTTCATGAAATACCAAACGTTCTCGGCAGAAGAAGTGTCGTACGATCTCGCTGATCAAGGGACAGCGTCGACAGTGCGAAGGAAGTCACTGAAGACCCGCGCTGTGGGCTGCTGTAAAATAGATTCAGAATACTTCGACAGGAATGGCAACACAATAGGTCTTAGAATTTCGTTGACATACCGTTGTTCTATGAGTGTGCTACGGAGGACCATCACAGAGGCCTGAAAGTAACAAATGGTGTCTCAGATCATAGGCGTTAGGGACGGGTCGTCCCTAACGTCTATGTGCTTGTCGTCGAAGTTCAGTATTAGACCCCAGCACCATCGCAATCGCTGCTGACGATGTTCGGTAGCAAGTGATAGAAATTTTTCATGTCAAGGCTGTAAAGCCCCTCCTCCTCCCCGAAAGAAAAGGAGAGGTGTGTTGTACTTAAGATGTATACTTGTAAATCGTATGATATGTATAATAAGTTCAAAATAACGATATTTGTTGTGAAATTTCTGATTTAGACGTACCTGTCATGCAGCCTTCTTATAGCCTTAAAAATAACACAACCTTTTTTTCTTTATCAGATAGCTCTGTTTAATGAATGAGGAAAGAAATGTAACAAGTCGTAACTTTATATTTAATGCATCGAAGAGAACCATTATACATTTCTCAGCAATGGTATCGCTTTACGCCATCATTATAACTAAGAACTGTCATTAGAGTCACTGGTGGTGTCGGAGTTAATCTTGTTCTCTATCCAGTCTACGTATGCAGACACTTTGGTATAGACACCAGGATCTCCTGGAGAGCCGCACCCCTCTCCGAAAGACACAATTCCATACAAAGTTGCCCTTCCAGCCTTTCGCACCATGAGGGGTCCTCCGGAATCAGCCTGACAGGAATCGA

							GTCCTACATCGCCAGCGCAAATCATTTTGTCAGTGATCACTCT<br>ATCGTAAAATCTGTACGCTGATTTGCACTTTTCTAAGGAAACT<br>ATGGGTACTCTGACTCCACGAAGCACATCGGAGGTTTTGCCA<br>TCTAAAAGAGAGGGAGAGAGAGAGAGCGATAAGTCATCTA<br>AAAAACTTTTGTGTTATCCCGGCTCACACATATCCTTGCCGAC<br>GCCATATTGTGGTTTACTTCTTCGCTTGCAGTCATTATTATTGT<br>CGTTGTTTCGATATTTGCATGATAACTCTGGTGGTTATAACAT<br>AAACTATTTTGTGTTAATTTGTAATGTCAAAAATGTTATAGCC<br>GGGAAAATCGATGAACCTTTCGACGCCGTGATAAACGATGT<br>CGAACAATGGAAAGGACAACTTGGCGAACAAGTGATCCATG<br>ATAAATGAACTGATTTCGTCGCAAGAAAATGTGAACCGGGA<br>CACCAGAAAAGTTCCGACTTTGGTTAAATGACTCTTGTCGTA<br>TATATTACGAGTGTTTTTATCCTGGTTATGCAATAAGTACCGT<br>GCTGGTATGAACGGTGACAATTCGACGTTAAACATTAGTTTT<br>GTCATTCAGAAATAAAAAAAAAATTCTAATAAAATATGGTA<br>TATATATATATATGTACTATATGACCAGACGCTGTAAAACAG<br>TAAGAGTGATTTTTCGCTCTTAAAAGTCGAAAAATGCACCAC<br>GCCTTAAAAAGAACTTTATCTGACTACGTTGAAAACGCTATT<br>TTTTCACCCACCGGGAATAAATTTTTTTTATTCGTGTTTCTTGT<br>AATTCACACATTCCTCTTTCGAAAACCGTTTTCCTTTACATTC<br>GTCCGTTAGCAATAAAGTAATAGCTTTTTAAAAATAGGTCGT<br>AAGCCGATGCATGAAACCGGAAGTAGCTTTAAAAATCTGGA<br>AGTCATCAAAATGTTCAGCATTTCACACTAACTTTTAGCATTT<br>TTTTATTATTTCGATTTGAAAGAACATTTTCACCGTGTTATGA<br>ATGCATCATTACAACTTTGATGCTGTTATTTTCTTTATTAAAA<br>AGATGGCGTGACTTATAAGTCGGGGGAACTTTAGACAGTCTT<br>GTTTAGCGGAAGATGTAGTTGATGCGAAGAAAAACTTTGGTT<br>GCGTATTTCTCAAAATTATTTTTCCCGTAAACGTTTTCCGTTA

							ACACGCTATCTCGAGAACCCATCAAGAAATTTATATAAAATT TTCAGGATATGTTTTATACGTTGTTGACAATGAAATGAACTA AAATCTTAGCACAGGATTTAAACATCTTTACATTTATGCGGA AAT

PhdEnzSeP 53	Q9TXD8	1,49E-20	30,364	PF00089	38343,03	QDVRICRFAAASLALGGWPTLCGWVGKGVPKVCCGLPLVLGPGLMKKVDEEITRKRECGTRIHSLGLSETRNKFFQILKTLPDVDISNPDKFDPPLDLGQESMLIPPVMFTGSRQGDFPWMVSIRKNGSHVCGGSLIDRKHILTAAHCVTGNKDQYAVHVGNLYVDDGYAYYISKITVHPKYQIGNSNNLAILALREEILSPLVPHVCLSGTNSTDVNLQGLSATLLRWGNATLDGTSRQKLRIIEGLNIVPIEDCKRSYVNEVIPEETGDLICTETNLNDKKLCEEDYGGPLMYKNNPENQMDHTIPWTMIGVDTFGSMCGNTKPLRLYTRVSSHLNWILETTNE*	TAATATTATACAAATCATTCATTCGTAGTTTCCAGTATCCAGTTTAAATGGCTCGATACTCTAGTGTATAGTCTCAATGGCTTCGTATTTCCGCACATGGATCCGAAGGTATCGACACCAATCATAGTCCATGGAATTGTGTGGTCCATTTGATTTTCAGGATTATTTTTGTACATAAGAGGGCCTCCATAGTCCTCCTCACACAATTTCTTATCGTTTAGGTTTGTTTCCGTGCAGATTAAATCTCCAGTTTCTTCCGGTATAACTTCATTGACGTAACTTCTTTTGCAATCTTCGATTGGAACAATATTTAAACCTTCTATTATTCTGAGTTTTTGCCTACTCGTACCATCAAGAGTCGCATTTCCCCACCGCAACAATGTGGCACTGAGACCTTGAAGATTCACATCAGTTGAGTTAGTTCCAGACAGACAGACATGAGGAACCAATGGAGATAAAATTTCTTCACGCAATGCTAAAATGGCCAAATTATTGGAATTTCCGATCTGATATTTAGGATGAACTGTGATTTTACTGATGTAATATGCATAGCCATCATCAACATATAAATTACCGACATGGACAGCGTATTGATCTTTATTTCCAGTAACACAGTGAGCTGCCGTCAAAATATGCTTTCTGTCAATGAGACTTCCACCACATACGTGAGATCCATTCTTCCGAATAGAAACCATCCACGGAAAATCCCCTTGTCTAGATCCTGTAAACATCACAGGAGGTATAAGCATAGACTCTTGCCCTAGATCTAATGGCGGATCAAATTTGTCTGGGTTACTTATGTCCACATCAGGGAGTGTCTTGAGAATCTGAAAGAACTTATTCCTAGTTTCTGAGAGACCAAGTGAATGTATTCTCGTCCCACATTCTCTCTTCCTCGTTATTTCTTCGTCCACTTTCTTCATGAGGCCTGGACCAAGTACGAGTGGCAATCCGCAGCAGACTTTGGGCACACCTTTCCCTACCCAGCCACAGAGGGTAGGCCAACCACCTAGTGCAAGGGACGCAGCGGCGAATCTACAGATCCGTACGTCCTG

PhdEnzSeP 54	Q9TXD8	5,12E-16	37,500	PF00089	12203,88	SHMRDSKIVGGGDA NFGEQPWQVAIVKQ SFLYRKISCGGALINR QWVVTAAHCVART QTSNLRVRLGEHDIK QTTEKYAHEEMAVR RKVVNPGYNPSNYQ HDIALLQL	AAGTCACATGAGAGACAGCAAAATCGTTGGAGGGGGGGAC GCAAATTTTGGTGAACAACCTTGGCAGGTGGCCATAGTGAA ACAGTCTTTCCTTTACCGGAAAATTTCGTGTGGAGGAGCTCT AATTAATCGCCAGTGGGTGGTTACTGCAGCACATTGTGTAGC CAGGACTCAAACATCAAACCTAAGAGTTCGCCTCGGTGAGC ACGACATCAAACAAACAACTGAGAAATATGCCCACGAAGA GATGGCAGTACGAAGGAAAGTAGTTAATCCTGGTTATAATCC TTCCAACTATCAGCATGATATTGCATTACTTCAGTTACA
PhdEnzSeP 55	Q9TXD8	4,11E-11	36,585	PF00089	8574,87	EAGQGDFPWMVSIR RLGNHVCGGSLIDR KHILSAAHCFFINGR KFNKTDFMVHVGNI YVDEGYPISVTKIAIH PQY	AAGAAGCTGGACAAGGAGATTTTCCATGGATGGTGTCCATTA GAAGACTAGGCAATCACGTGTGTGGAGGCAGTTTGATAGAT AGAAAACATATACTGTCAGCAGCACATTGTTTCTTTATTAAT GGTCGGAAGTTCAATAAAACTGATTTTATGGTGCACGTGGGC AACATATATGTTGACGAAGGTTATCCCATCTCAGTGACAAAA ATTGCTATTCATCCGCAGTATA
PhdEnzSeP 56	Q7M4I3	1,51E-16	31,579	PF00089	12792,87	KTVVGEWPWIVAIT AAEDNSSGILCGGA VLNSQMVITVAHCL FGFKKFRLYFGKYYY RNNLDDTHVLVRTS DKVIIHPDFKNTSLD SDIALIKFKPRIKFSER IQPICLPTGE	GACTCTCCTGTGGGCAAGCAAATGGGTTGGATCCGTTCCGAG AACTTAATTCTGGGCTTAAATTTGATCAAGGCGATGTCCGAG TCCAGGGAAGTGTTTTTGAAATCCGGGTGGATGATCACTTTG TCACTCGTTCGAACTAGCACGTGAGTGTCATCAAGGTTGTTTC TATAGTAATATTTCCCGAAGTAGAGTCTGAACTTTTTGAATCC GAACAGGCAGTGAGCAACTGTGATGACCATTTGGCTGTTCAG TACGGCTCCTCCGCAAAGTATGCCGCTGCTGTTGTCCTCTGCG GCCGTTATCGCCACGATCCAAGGCCATTCGCCAACCACTGTC TTTG

PhdEnzSeP 57	Q7M4I3	8,13E-19	32,308	PF00089	14941,13	WPWIVGIANRENNF TGQVICGGAILNSET IVTAAHCLPRNRRFT LYFGKHYRNNRLDD IQVLVRMSDRVIIHP DFVNETLDSDIALITF KPKMRFSERVQPICL PTTESTAKNIVTGRR GYVTGWGYTER	GTGGCCTTGGATTGTGGGAATCGCAAACCGAGAGAACAACT TCACCGGTCAAGTGATTTGCGGAGGAGCAATACTTAACAGC GAGACAATCGTAACGGCTGCTCACTGCCTGCCACGGAACAG AAGATTCACGCTCTACTTCGGGAAACATTACCGTAATAACAG ACTCGATGATATCCAAGTGTTAGTTCGAATGAGTGACAGAGT GATCATCCACCCGGATTTTGTGAACGAGACCCTGGACTCTGA TATCGCCTTGATCACATTTAAGCCCAAAATGAGATTCTCTGA GCGGGTCCAACCCATCTGTCTTCCCACAACAGAGTCTACTGC CAAGAATATCGTTACAGGAAGAAGAGGATACGTCACTGGAT GGGGCTATACTGAGAGG
PhdEnzSeP 58	Q7M4I3	7,63E-19	36,937	PF00089	12960,78	RGWKGIITGWGKTD TGLGNRYGTRLLQK VDVPIISNDLCESWH HEKGIEITISPEMMC AGYENGQKDACVG DSGGPLMVIIHGRW TVAGLVSAGFGCAQ SRQPGIYHRVPHTVE WLRE	CGAGGGTGGAAAGGAATTATCACTGGCTGGGGAAAGACTGA CACAGGACTCGGCAATCGTTATGGAACACGTCTCCTTCAGAA GGTGGATGTACCTATAATATCGAATGACCTGTGCGAAAGTTG GCATCACGAAAAAGGCATTGAGATAACCATTTCTCCTGAAAT GATGTGTGCAGGATATGAAAATGGACAGAAGGATGCATGCG TGGGAGACTCGGGAGGACCTCTGATGGTCATTATTCATGGCA GATGGACAGTTGCCGGTCTCGTTTCAGCTGGCTTCGGATGTG CCCAAAGCAGGCAGCCCGGAATATACCACAGAGTACCGCAC ACAGTGGAATGGCTACGAGAG

PhdEnzSeP 59	Q9TXD8	2,51E-52	40,455	PF00089	29482.55	HQRDCDKGPRHLCGGTILNEHWILTAAHCFRFPKNFWKYNVYIGVHSQARKDFKPSKRYEISNLIVHENYDGFKETHPNDIALLRTEEPITFKNFGGFVKPVSLPDSDTDPTGHAVVIGWGLNDEGFKVDILQAVDVPIVPRYDCSALYKKYTMTYITETQLCAGTAAKDAREHDSGGPLLQKNENGGYTLIGIVSYGKQKKKPYLPGVYTKVSSYTGWLKRNMANPGITTGKENDIRFSKYCKTKDGIPRLTRNSPIYW*	TCAAAAGCGAAATTTTTTCTTCTTCGTACATTTAAAGCTTTGTACTACCAATATATTGGAGAGTTCCGAGTCAGCCTAGGAATTCCATCTTTCGTCTTACAATATTTACTAAAACGGATATCATTCTCTTTTCCAGTAGTAATTCCAGGATTTGCCATGTTCCTTTTCAACCAGCCCGTGTAGGAAGATACTTTGGTGTAAACTCCAGGTAAGTACGGTTTTTTCTTTTGCTTTCCGTACGACACGATACCAATCAGAGTGTAACCACCGTTCTCATTTTTTTGCAAAAGAGGACCTCCAGAGTCATGCTCACGAGCGTCTTTAGCAGCAGTTCCAGCGCATAGCTGCGTTTCTGTAATATATGTCATTGTATATTTTTTGTATAGAGCACTGCAATCATACCGTGGAACAATTGGAACGTCCACAGCCTGCAATATATCAACTTTGAAACCTTCATCATTAAGCCCCCATCCGATGACGACCGCATGTCCTGTGGGATCTGTATCACTGTCTGGCAGGGAAACGGGCTTCACGAATCCTCCGAAATTCTTGAATGTTATGGGCTCTTCGGTCCGCAGCAGGGCAATATCGTTTGGATGTGTTTCCTTAAATCCGTCGTAATTCTCGTGTACGATCAAATTGGAGATCTCGTATCTCTTCGATGGCTTGAAGTCTTTTCTGGCCTGGCTATGGACACCTATGTACACATTATACTTCCAAAAATTTTTCGGGAACCTGAAACAGTGTGCAGCTGTTAAAATCCAGTGCTCGTTGAGTATGGTGCCTCCACATAGATGTCGGGGACCTTTGTCACAGTCTCTCTGATGG
PhdEnzSeP 60	P0CG03	4,67E-19	54,930	PF00089	7242.24	FSTMVCAGYKEGGKDSCQGDSGGPLTAVKNGRSTLVGLVSWGVGCARPKLPGVYTKISEYVNWIEQVTS*	GGCAGATCGTGTATTGAAAATGAATGAACACAGACATTTTTTACAGAATGCAGTTTCAACTTGTAACTTGCTCAATCCAGTTGACGTATTCTGAGATCTTAGTATATACTCCAGGAAGCTTCGGCCGTGCGCATCCAACACCCCAGGAAACTAAGCCAACCAACGTGGAGCGACCATTTTTGACTGCTGTTAGTGGACCACCAGAATCTCCCTGACACGAGTCTTTTCCGCCCTCTTTGTAGCCTGCACAAACCATGGTAGAAAAT

PhdEnzSeP 61	Q8MQS8	8,75E-11	39,726	PF00089	9619.17	ALLQLQRPIRFRRHVIPVCLPLYGEEFAGQKATVTGWGRTRYGVRDSPGILQKVELQVIDHNECQEWYKSIGRKETIFSTMVC	GCATTACTTCAGTTACAAAGACCTATTCGGTTCCGTAGGCATGTCATTCCAGTTTGCTTGCCACTATATGGAGAAGAATTTGCTGGCCAAAAAGCGACGGTAACAGGGTGGGGCAGAACTCGTTACGGTGTTCGAGATTCCCCTGGAATACTCCAAAAAGTAGAACTTCAAGTAATCGACCATAACGAATGCCAAGAATGGTACAAAAGCATTGGTAGAAAAGAGACTATATTTCTACCATGGTTTGTGC
PhdEnzSeP 62	A6MFK7	3,11E-16	57,143	PF00008<br>PF00594<br>PF00089	7783,7	NKYQSLKHELNQNVTRNMFCAGYSQEIIGDACNGDSGGPFLMSHEGRWYIVGIVSWGEGCGKRNKYGFY	GTAAAATCCATATTTATTTCGTTTGCCACAGCCTTCTCCCCAGCTGACAATACCTACTATATACCATCTTCCTTCATGGGACATTAAGAAAGGCCCTCCACTGTCACCATTGCAAGCATCACCAATAATTTCTTGGGAATATCCAGCACAAAACATATTTCGAGTAACATTTTGATTTAATTCATGTTTCAAAGACTGGTACTTATTTTAAAACTATTGGATGATATACACTGTTCAACATAATTACTAGCATAGTTCATGATTGCAAATAATTGTTCATCATTCATAAGCAGTGAAAAATCCCGCACCTGTTTCAAACTTTGCAAAGCTTCTGTATAATTTTTTATATGAAGCTCATCAACTGAGCTGATATTGCTTCGATCTTCATCATCTAGATTATCATTACTCTTATTTCCATTATTATCATCATCATCATCTATATCTTCAGTATCTTTA

PhdEnzSeP 63	A6MFK8	1,73E-50	35,294	PF00008<br>PF00594<br>PF00089	98480.64<br>**95762.32**	MRDIMGCVMQLLL<br>MAIFASWYADG**LQ**<br>**NAKRPPDSPLVRYT**<br>**RAAEGGCSSNLTV**<br>**KGSGGTLTSPNYPS**<br>**NYPSSIECRWLLTTT**<br>**EPNATVLLKIDSLSI**<br>**EPDTDCRLDYLQVY**<br>**LGATTLAPALGPFC**<br>**GNIYNRLLKTNAST**<br>**VLLVFRSDPVYEDK**<br>**GFSVDYFTQSRGDC**<br>**PTLLTDGSGSLRSP**<br>**GFPDSYPDHTDCW**<br>**TLIKAKEGKKITLFF**<br>**DSVDMEYGENCTF**<br>**DYIQIFDGEARNST**<br>**LMGQICRHSDVRSF**<br>**ESSSNSILVHFHSD**<br>**DSVNKQGYRARYS**<br>**SIDKRSSPVGECLW**<br>**ETGKYNGTIASPNY**<br>**PSNYPSSSNCSIWIL**<br>**APEGSTIAITFDALQ**<br>**LEMDTKCMFDFIQ**<br>**VEEGSATETKVLGR**<br>**FCGNDGEPKNLTST**<br>**RNEVHLQFHSDDF**	TTTTTTTTAAAGGAGAAACAAAACATTTTATTTTTACAGGTGG<br>ACAATAATTACACACATGCATGTTGCCCCACTCTTTTATCTTT<br>GCAGCCTCAAGTTCTTCACTTGCGAAAGAGTTTTGTCACGATT<br>TGATGATGCCCTGGATCCAGGATATGTAGTTGGGTATACGCG<br>TATAAAATCCGTATTTGTTCTCAGTGCCGCAGCCTTCTCCCCA<br>GCTGACAATGCCCACAATGTACCATCTCCCTAAGTTCGATAT<br>CAGGAACGGACCGCCGCTGTCGCCATGGCACGCGTCACCTA<br>GGATTTCTTGGGTGTATCCCGCGCAGAACATGTTTCGAGTGA<br>CCGTGTAATTTGTGGAGCGCAGGCATAAATTCTGGTCGACGA<br>TGGGCAACATGATTTCTTGTAGGAACCTCGGTGATGGGCCGT<br>TGTCCTTCAGCTTTCCCCAACCAATGACTGTCCCCATTTGCAA<br>CTTGGAGCCGCTCCCTTGCATTAGGGTCTCGCTGAGCCCCCT<br>GTCTCCCAGACAAGCGGGAAGGATGTAGTCCGTGAACTTGA<br>CGTGCTCCCTCATCCGGACCAACGCTAAGTCATTATCGAAGG<br>TGAGCCTGTTGTAGGCAGGGTGTACAACAATAGAGGAAGCG<br>TCCGCAATGCCAGTCCGGAACTCTTGTTCTTCTTCTCGTTCCC<br>TGTCATACTTTCCGAGCTTGATAACGACACGGTCCCAACGGA<br>GGTTCTTGTAGTTGATGAAGCAGTGGGCGGCTGTTGCGATCC<br>AGCGCTCGTTCAGGAGAGTCGCCCCACAAAACGACTTCAGA<br>TTGGGTTCCCAGAGCATTACTTGCCATGGGTAGGCGCCTTTTT<br>TGGCGTTGTGGCCCCCGATGATTTTTCCGTGGTCAACTTGAGG<br>AATTTCTTTGGATGGTTCTCCCATTTTGGGGACTCCGCAAATC<br>TCCTCTATCTTGATCCTTTGGTTGACCTGCAGTAAGGCTGATG<br>ACTTCTTCTCGCAGCCAATCGCCGTGTCACGGGCCACGCATG<br>TGTACACTCCAGCGTCGTCCAGTTTCACTCTGTCCATGAAAAT<br>GTAGCCGTTCTTGAGTACGGTTATATGTCCCTTGGACTCCAGA<br>GGTTGGCCGTCCTTCTCCCAAGACATCTGCACCTGTGTCCCG<br>GCAGCCAGGCATTCCAAATGGGCAAACTCACTCTCGGCGAA

						**<u>AEFSGFKLRYWFLG</u>**	GGAGGAATCTTTAGGAGTCTTCCGGAATACTATTCCGCAGGA
						**<u>TDHKEKTVSPSPAP</u>**	CTCATCGTCGTCATGCGGCTCGTTTTTCACGAGGACATAAGC
						**<u>SQKLTFSVVPHNVT</u>**	CTGCGCGGTGATGGTTTCCTCAGGAGAGATGGCAACGCAGG
						**<u>LAVSSSHMLHCST</u>**	AGTAGATACCAGATAGGGGATGGTCCATACTCTGTATCCACA
						**<u>QHPTAKLRWLKGD</u>**	GGGTGTTATTGGGAAGTACCTTCAGGCCTGGCATGGGGCTCC
						**<u>VFLTSGSPMPGLKV</u>**	CACTAGTGAGGAAAACGTCACCTTTCAACCATCGTAACTTGG
						**<u>LPNNTLWIQSMDH</u>**	CGGTAGGATGTTGAGTGCTGCAGTGCAACATGTGGGACGAA
						**<u>PLSGIYSCVAISPEE</u>**	CTCACGGCGAGAGTCACATTGTGAGGCACCACGCTGAACGT
						**<u>TITAQAYVLVKNEP</u>**	CAGTTTTTGAGAGGGGGCGGGACTAGGACTGACGGTCTTTTC
						**<u>HDDDESCGIVFRKT</u>**	TTTGTGATCTGTTCCGAGGAACCAATATCGCAGTTTGAATCCC
						**<u>PKDSSFAESEFAHLE</u>**	GAAAATTCTGCAAAATCGTCCGAGTGGAACTGTAAGTGCACT
						**<u>CLAAGTQVQMSWE</u>**	TCATTTCTGGTGGACGTTAGGTTCTTTGGCTCTCCGTCGTTTCC
						**<u>KDGQPLESKGHITV</u>**	ACAAAATCTTCCCAAAACTTTGGTTTCTGTAGCTGAGCCTTCC
						**<u>LKNGYIFMDRVKL</u>**	TCTACCTGGATAAAGTCGAACATGCACTTTGTGTCCATTTCCA
						**<u>DDAGVYTCVARDT</u>**	GCTGCAGGGCATCGAAAGTGATGGCGATAGTGCTGCCCTCTG
						**<u>AIGCEKKSSALLQV</u>**	GTGCCAGGATCCAGATACTACAGTTGGAGGAGCTGGGGTAG
						**<u>NQRIKIEEICGVPK</u>**	TTGGACGGATAGTTGGGACTGGCGATGGTACCATTGTACTTG
						**<u>MGEPSKEIPQVDH</u>**	CCAGTTTCCCAAAGGCATTCTCCGACAGGAGAACTCCTTTTG
						**<u>GKIIGGHNAKKGA</u>**	TCTATGGAAGAATACCTGGCGCGGTATCCCTGTTTATTGACC
						**<u>YPWQVMLWEPNLK</u>**	GAATCATCCGAATGGAAATGTACCAGAATAGAATTCGACGA
						**<u>SFCGATLLNERWIA</u>**	CGATTCGAAGCTTCTGACGTCCGAGTGTCGGCAGATCTGACC
						**<u>TAAHCFINYKNLR</u>**	CATGAGCGTGGAATTTCTCGCTTCGCCATCGAATATTTGTATG
						**<u>WDRVVIKLGKYDR</u>**	TAGTCGAAGGTGCAGTTCTCTCCGTATTCCATGTCAACGGAA
						**<u>EREEEQEFRTGIAD</u>**	TCGAAAAACAGGGTGATTTTTTTTCCCTCCTTCGCCTTGATTA
						**<u>ASSIVVHPAYNRLT</u>**	GCGTCCAACAGTCCGTGTGGTCTGGATAGCTGTCCGGGAATC
						**<u>FDNDLALVRMREH</u>**	CGGGACTCCTCAGCGAACCGGACCCATCTGTCAGCAATGTTG
						**<u>VKFTDYILPACLGD</u>**	GACAATCTCCTCTGGACTGAGTGAAGTAATCGACAGAGAAT
						**<u>RGLSETLMQGSGS</u>**	CCCTTGTCCTCGTAGACGGGATCCGATCGGAACACCAGCAG

						<u>**KLQMGTVIGWGKL<br>KDNGPSPRFLQEIM<br>LPIVDQNLCLRSTN<br>YTVTRNMFCAGYT<br>QEILGDACHGDSG<br>GPFLISNLGRWYIV<br>GIVSWGEGCGTEN<br>KYGFYTRIPNYISWI<br>QGIIKS***</u>	GACAGTGGACGCGTTAGTCTTGAGCAGCCTGTTGTAGATGTT<br>GCCGCAGAAGGGACCCAGGGCAGGCGCCAACGTCGTGGCCC<br>CCAGGTATACCTGCAAGTAGTCCAGTCTGCAGTCCGTGTCGG<br>GCTCTATACTGAGTGAATCAATCTTTAGCAGTACGGTCGCGT<br>TGGGTTCTGTGGTGGTGAGGAGCCAACGGCACTCTATGCTGC<br>TAGGGTAATTGCTGGGATAGTTCGGGCTCGTCAGGGTGCCAC<br>CACTGCCTTTCACAGTCAGGTTTGAAGAGCAGCCACCTTCAG<br>CTGCCCTTGTGTATCGGACCAATGGACTGTCCGGTGGTCGCTT<br>GGCGTTCTGTAATCCATCTGCGTACCAGGAAGCAAATATCGC<br>CATCAGAAGCAATTGCATTACACACCCCATAATGTCTCTCAT<br>CCAAAGTCGAACATCAGAGCAATCCAGGTGAGTGAAACGCA<br>CACGTTAGAAGTTCGCGGCAGAATTCATTCCGCGCATAATTT<br>TGCAAACAATTTAAGGCACATGCAACAGCTATTTCACCGCAT<br>GGTGGTCGCAGTTAGGATAATTGCCGCCAGACAATAAAAAG<br>GTTACCGGATTTTCTTTGTAAACGTTCGTTCGAGGAAAAAAC<br>ACACAGAGAGAGAAAGAATTGTTTTCGTTCGCTTCGCATGCG<br>AGTTCCGAACGCGAATGCAGAGAGTGAGAGAGAGAGAGGC<br>TGGCTCGAGAGAGAAAACAGGAACTACGTGCGCGG

PhdEnzSeP 64	A6MFK7	2,99E-35	34,979	PF00008 PF00594 PF00089	30366,63	MEISLLNSLFVSECGQSFMRTTSSKFVVGNGYKTVVGEWPWVVAIAIRQNNFTGRVICGGAVLNRRTIITAAHCLLQARQFTLYFGKYYRDISLDDAEVLNRTSDRVILHPNFANDTLDSDIALVTFKPEITFSSRIQPICLPTEESTGIVPGDSAFITGWGQTEKRLGSSELLMAFLPVVSNDACRESFRQYFPKFNLVLTQNMFCAGFSDARQFTCIGDSGSPIVSYDSVTDQYTLEGLVSFGVEGQCGLPGSYTIFTKVSRFLPWINRNI	TAAGAAATGTATAATTATGCACTTACTCCAAAACCTTCCCTACTCGTTATCCTAGTATGCAGATGTGATATTAGACCAAGAAATATGATGATTCGCATCCTATGAGATAAATTTAACAAATAGTCGACGAATCAGTCTTCTCAATATTTTCGGAACGTACTGTTATCTGAATAATACACTTCAGTAATGAAAATTTCTTTCTTCATCGAAAAGAAATTGTTGTCATTCACTTTCAGATTTTTTAATGGTGTCATTGCATAAAAACTCAGATGGAAATTTCTTTGCTCAATTCCCTATTCGTTTCAGAATGTGGACAATCCTTCATGAGAACCACCTCGAGTAAATTTGTTGTTGGCAACGGTTACAAGACAGTGGTCGGCGAGTGGCCTTGGGTTGTAGCGATCGCGATACGGCAGAACAACTTCACCGGTCGAGTGATTTGTGGAGGAGCAGTATTGAACAGACGGACGATCATAACGGCTGCTCACTGCCTGCTCCAAGCCAGACAGTTCACCCTTTATTTCGGAAAATACTACCGCGATATTAGCCTCGATGACGCCGAAGTCCTGAACCGAACAAGCGACAGGGTGATCCTCCACCCGAATTTTGCAAACGATACCTTGGACTCGGACATCGCCTTGGTTACATTTAAGCCCGAAATCACTTTCTCGAGTCGAATCCAACCTATTTGCCTGCCCACGGAAGAGTCTACGGGTATCGTTCCAGGAGACAGCGCATTCATCACTGGATGGGGTCAAACGGAGAAGAGGCTAGGATCGTCCGAGTTGTTGATGGCCTTCTTACCCGTTGTGTCGAACGACGCTTGCCGCGAATCATTCCGTCAGTACTTCCCGAAGTTCAATCTAGTGCTTACCCAGAACATGTTCTGCGCAGGATTCTCGGACGCACGTCAATTTACCTGCATAGGCGACAGTGGATCGCCAATAGTCTCCTATGATAGCGTGACAGACCAGTACACTCTGGAAGGTCTGGTCAGCTTCGGAGTCGAAGGACAGTGTGGTCTTCCTGGGTCGTACACCATCTTCACCAAAGTCAGTCGATTTCTACCATGGATTAACAGAAATATA

PhdEnzSeP 65	Q58L93	4,34E-28	30,453	PF00008<br><br>PF00594<br><br>PF00089	42353,85	PPDGLLEYICDDGYT LEGSTFLICRGMWT ADPPTCRRNVEPRN CMNPGPISNGRVLY HPPGNNRRSTQTTLI EPEYYPPDGLLEYIC DNGYTLEGSTFLTCR VVMWSAEPPTCRRD CGKSLVGMASNSLN GKILHGHSTSAGQW PWVVGIAEQETSSD VICGGALLNPQTVIT AAHCLKKADSEFTL YFGKYYRDISRDDEH VLVRRSDRVIIHPDF QYDTLESDIALITFTP KIKFSDRIQPICLPTN QSTAKNIKTEEGGYV AGWGRTETRLRSPD LRMAVLSVLTTEDC RESLSNNVANFTPVI TQNMFCADFPGTRQ STCIGDSGSPVVFYD EVTKRYTLEGLVSFG PNGNCSLAYTICTKV NQFVPWIMNNWNS K*	CCTCCAGACGGACTTTTGGAATACATTTGCGACGACGGATAT ACCTTGGAGGGCAGTACTTTCCTCATTTGCCGAGGAATGTGG ACAGCGGATCCACCCACTTGTCGAAGAAATGTCGAGCCAAG AAATTGTATGAATCCGGGACCCATTTCGAACGGGCGAGTACT GTACCATCCTCCGGGTAACAACCGACGAAGTACGCAGACCA CACTCATCGAACCGGAGTATTACCCTCCAGACGGACTTTTGG AATATATTTGCGACAACGGTTATACTTTGGAGGGCAGTACTT TCCTGACTTGCAGAGTAGTAATGTGGTCAGCGGAACCACCCA CTTGCCGAAGAGATTGTGGAAAGTCGTTAGTGGGAATGGCCT CGAATTCCCTGAATGGCAAAATCCTGCATGGCCATAGCACAT CGGCTGGCCAGTGGCCTTGGGTTGTGGGGATCGCGGAGCAG GAAACCAGCTCCGACGTGATCTGCGGAGGAGCCTTGCTGAA CCCCCAGACCGTCATCACTGCTGCTCACTGCCTGAAAAAAGC CGACAGCGAGTTCACGCTCTACTTCGGGAAGTATTACCGTGA TATCAGCCGTGATGACGAACACGTACTGGTGAGAAGAAGCG ACAGAGTGATAATCCACCCGGATTTTCAATACGACACCCTAG AATCAGACATCGCCTTGATCACCTTTACGCCCAAGATTAAGT TCTCAGACCGGATTCAACCCATCTGCCTTCCCACGAACCAGT CTACGGCCAAGAATATCAAAACAGAAGAGGGAGGATACGT CGCTGGATGGGGTCGAACAGAGACGAGGCTGAGATCGCCTG ATCTGAGAATGGCCGTATTATCCGTTCTGACCACAGAAGACT GTCGCGAGTCGTTAAGTAACAACGTCGCGAATTTCACGCCAG TAATTACCCAGAACATGTTCTGCGCTGATTTCCCGGGCACAC GACAGAGTACGTGCATAGGCGACAGTGGATCGCCAGTCGTC TTCTACGATGAAGTAACAAAACGGTACACCCTGGAAGGTCT GGTCAGCTTTGGGCCCAATGGAAATTGTAGTCTTGCGTACAC CATCTGCACGAAAGTTAATCAGTTTGTTCCATGGATTATGAA TAACTGGAATTCAAAATAGGACTCTATTTTAAATTAAATGAG

							TCTGTTCCATCTGCAGTTCCCTCCTGTTGCAAATGAATAGTTTAGGAAGCTTAGTACAAAGCAGAACATAATTATAACATGAGTTTATAACAGAAAGGACTCGTTTAATAAGGAATCTCTCGCAAATTCGGCGGAAAATTTCACTTCCTGTTATATTAGTTGTCAACGAAGAACAACTCCGGAGGCACTTTTTCGTCTCTCCTCCCGATTGACCTCAAAGCTATTTCCAAGAGGATAACTGCGGAGAAATAGGGTGAAATTTCAGAATGCGGAATTGTGTCACTTGCCTAGTTTCAATTTGTTATATTCTGTTACAGTGGAACAAGCAGAATCTCTTTGTAAAGGAGGAAGAATTTCCTTTTCTGAATGGGGATGAAAAAAAAAATAATGAAACTTCCATTTTTGCTGCCGACTTTTATATCCTGGAGAAGGGATGGACTCTAACCATTCCATCGTCGTGATTTAGTCACTCTACCGGTTGCGGTAGCTCGTTGTTGAAAATGAATAAACAGTCCCTTTGCAAGGCATACTGTA

PhdEnzSeP 66	P81428	1,69E-35	31,373	PF00008<br>PF00594<br>PF00089	59486,06	QGFPQGTRLQYSCN<br>TGYDIDGERSVFCLV<br>SGEWTSERPKCNSQS<br>REPAAPDTSLTYCPD<br>PGVDPNGNRNPSSP<br>PNHQGFPQGTRLQY<br>SCNPGYDIDGERSVF<br>CLVSGEWTSERPKC<br>NSQYREPGPDRSTKF<br>CRDPGLDPNGSRKP<br>SSPSNDQGFPQGTQL<br>EYFCNQGYDLEGEK<br>NMFCLASGEWLSRR<br>PSCTAEAATTSETTN<br>YCSNPGSILRGRVVY<br>LPPLNERNSPRLPNP<br>NEAGSVSYPQGSRL<br>QYSCDDGYSLDRSSS<br>VTCEASGRWSSAPPT<br>CTECGETAMGTITK<br>VINGNKTVAGQWP<br>WTVAIAVRQNDYTG<br>DVICGGAILNRQTVI<br>TAAHCLTQSSQFTLF<br>FGKYYRIHNLDDIN<br>VLNRTSDRLILHPDY<br>DNITMDSDIALITFRS<br>EITYSDRVKPICLPTG	ACCAAGGATTTCCCCAGGGTACCAGGTTGCAGTATTCATGCA<br>ACACAGGTTACGATATCGATGGCGAGAGAAGTGTGTTCTGCC<br>TCGTATCTGGAGAATGGACCTCAGAAAGACCGAAGTGTAAT<br>TCTCAGTCTCGTGAGCCAGCTGCTCCGGACACGAGCCTGACC<br>TACTGTCCAGACCCTGGGGTGGACCCGAACGGCAATCGAAA<br>TCCTAGTTCGCCTCCCAATCACCAAGGATTTCCCCAGGGTAC<br>CCGGTTGCAGTATTCATGCAACCCAGGTTACGATATCGATGG<br>CGAGAGAAGTGTGTTCTGCCTCGTATCTGGAGAATGGACCTC<br>AGAAAGACCGAAGTGTAATTCTCAGTATCGTGAACCAGGTC<br>CCGACAGGAGCACGAAATTTTGTCGAGATCCCGGTCTGGAC<br>CCGAACGGCAGTCGAAAGCCCTCGTCGCCCTCCAATGACCA<br>AGGTTTCCCCCAAGGCACACAACTGGAGTACTTTTGCAACCA<br>GGGTTACGATCTGGAGGGTGAGAAGAATATGTTCTGTCTTGC<br>ATCTGGAGAATGGTTGTCAAGAAGGCCGAGCTGTACCGCTG<br>AAGCTGCAACAACATCGGAAACGACCAATTACTGCAGTAAT<br>CCCGGATCAATCCTCAGAGGAAGAGTAGTGTACCTCCCGCCT<br>CTAAATGAACGGAATTCACCCAGACTCCCCAATCCTAATGA<br>AGCAGGGTCTGTATCGTATCCCCAAGGAAGCCGCTTGCAATA<br>TTCCTGTGACGATGGATACTCCCTGGATCGGTCGTCCAGTGT<br>AACATGCGAAGCGTCGGGACGATGGTCATCTGCTCCTCCCAC<br>CTGCACTGAGTGTGGAGAAACTGCTATGGGAACCATCACGA<br>AGGTCATTAACGGCAACAAGACGGTGGCTGGTCAATGGCCT<br>TGGACTGTGGCGATCGCGGTGCGACAGAACGACTACACCGG<br>TGATGTGATCTGTGGTGGAGCAATACTAAACAGACAGACAG<br>TCATAACCGCTGCTCACTGCCTGACGCAATCTTCACAGTTCA<br>CACTCTTTTTCGGAAAATATTATCGCATACATAATCTGGATGA<br>CATCAATGTGCTGAACCGAACGAGCGACAGACTGATACTCC<br>ACCCGGATTATGATAATATCACCATGGACTCGGACATCGCCT

						ESTAKNVVPGKKGF<br>VTGWGYTEWRQRSP<br>ELLMAHLPIESSDAC<br>RESFRENVPNFNLVL<br>TQNMFCAGYSDGR<br>QSTCIGDSGSPIVFYD<br>RRTERYILEGLVSFG<br>AEVQCGLPGTYSIFT<br>KVNRFVPWIIR	TGATTACCTTTAGGTCCGAAATCACTTATTCGGATCGCGTCAA<br>ACCTATTTGCCTGCCCACAGGAGAGTCTACGGCCAAGAATGT<br>CGTTCCAGGAAAGAAAGGATTCGTCACTGGATGGGGTTATA<br>CAGAGTGGAGACAGAGATCGCCCGAGCTGTTGATGGCCCAC<br>TTACCCATTGAGTCGAGCGACGCTTGCCGCGAGTCGTTTCGT<br>GAGAACGTCCCGAATTTCAATCTAGTGCTTACCCAGAACATG<br>TTCTGCGCAGGATACTCGGACGGACGACAGAGTACCTGCAT<br>CGGAGACAGTGGATCTCCAATCGTCTTCTACGATAGAAGGAC<br>AGAACGGTACATTCTGGAAGGTCTGGTCAGCTTCGGGGCCG<br>AGGTACAATGTGGTCTTCCTGGGACGTACTCAATCTTCACAA<br>AAGTTAATCGATTTGTTCCGTGGATTATCAGAG

PhdEnzSeP 67	Q4QXT9	6,00E-38	33,712	PF00008<br>PF00594<br>PF00089	111659.5<br>**109510.85**	MKIAWLIIGIFLSLSS<br>VCDG**HLILDDGFVG**<br>**VGLDLSKLGLGKSK**<br>**ADGGCPPSGNITCA**<br>**CAAEGYPISVEVPV**<br>**CSYSYRWKISCKPC**<br>**DKMTPEEVCPKYLL**<br>**CEQCHLHGGDSCV**<br>**TCPAGKFGTWCEN**<br>**TCACQNGGECERN**<br>**GKCLCPANFEGRN**<br>**CEKKKGCDSPVGIA**<br>**PPLQVAFQPPERPLT**<br>**AAYSCPPNYVLSGQ**<br>**AISTCISGKWTSGP**<br>**PSCRPKCPILSAPAN**<br>**GRLLFSGNDLVEGV**<br>**SADVECLQGYRLV**<br>**GQKTLTCSTGGRW**<br>**DHDLPLCEELASCP**<br>**DPGNVNNAERTIL**<br>**HRAVKVGGHFFQD**<br>**SQLQYKCLPGYEQ**<br>**MGTEKILCTFDGT**<br>**WSNQPPSCLKVAT**<br>**VVPDCETPGSDVID**<br>**EEGVSVRIFCPPECT**<br>**DEDFKLWGTSIYRV**	TTGAAAAGGTCGAAAGCACATTTTCGGTTTGTTTTGCAAGAA<br>GGTGGTAGCTGATTGGGTTCTGCAGAGTGATAAGCTGAAATA<br>CGGAGAACTAACGCAGGAAGTGCTCTTTATCTGGGTGAATCA<br>TGTGGTATAAATCTCCCGTTTAATAACTGACAGCGAATAAAG<br>CAAGATGAAGATCGCCTGGCTTATAATCGGTATTTTCTTATCT<br>CTGTCATCAGTCTGTGATGGTCATCTGATTCTGGATGATGGAT<br>TTGTGGGAGTTGGACTTGACCTCTCGAAACTCGGTCTCGGTA<br>AGAGTAAAGCAGATGGAGGCTGTCCTCCATCAGGGAACATC<br>ACGTGTGCTTGCGCTGCGGAGGGATATCCTATTTCAGTAGAA<br>GTGCCTGTGTGCAGCTATAGCTACCGCTGGAAGATTAGCTGT<br>AAGCCTTGCGATAAGATGACGCCAGAAGAGGTCTGTCCAAA<br>GTATCTTCTCTGTGAACAATGTCATCTCCATGGAGGAGACTCT<br>TGCGTGACCTGTCCCGCAGGCAAGTTCGGGACTTGGTGCGAA<br>AATACGTGTGCTTGTCAGAATGGTGGGGAATGTGAACGCAAT<br>GGAAAGTGCTTGTGCCCAGCAAATTTTGAAGGAAGAAACTG<br>CGAGAAAAAGAAAGGTTGTGATTCGCCTGTTGGTATCGCCCC<br>TCCATTGCAAGTTGCATTTCAACCACCAGAACGTCCTCTTACT<br>GCAGCTTATAGCTGCCCTCCAAACTATGTATTAAGTGGCCAA<br>GCAATTAGCACATGTATCTCTGGGAAATGGACTTCTGGACCT<br>CCTTCTTGTCGTCCGAAGTGTCCAATCCTTAGTGCACCTGCAA<br>ATGGAAGATTGCTCTTCTCTGGAAATGATCTGGTGGAGGGAG<br>TAAGCGCTGATGTTGAGTGCCTGCAAGGCTATCGGTTAGTAG<br>GACAGAAGACCTTAACATGCTCGACTGGAGGACGATGGGAT<br>CACGATCTTCCTCTGTGTGAGGAACTGGCATCGTGTCCCGAT<br>CCTGGAAACGTTAACAATGCAGAAAGAACAATTCTTCATCGT<br>GCAGTCAAAGTAGGTGGCCACTTCTTTCAAGACAGCCAACTT<br>CAGTACAAGTGCCTACCCGGTTACGAACAAATGGGGACTGA<br>GAAAATCTTGTGCACTTTCGATGGTACATGGTCAAATCAGCC

						**GSSVCQAAIHSAKI**	GCCTTCTTGTCTTAAAGTGGCAACTGTTGTACCTGACTGTGAA
						**TNSGGLVAVINNG**	ACTCCCGGATCTGATGTTATTGACGAAGAAGGCGTATCAGTG
						**PYSHFTGSDSNYIES**	AGGATTTTTTGTCCTCCGGAGTGTACTGATGAAGACTTCAAA
						**FSYPDKDESFRFDRL**	CTCTGGGGTACCTCTATATACAGGGTAGGATCATCAGTATGC
						**RAHVLARHSNECD**	CAAGCAGCCATTCACTCTGCCAAAATCACGAATAGTGGTGG
						**KGLSKLQNTCVYIS**	ATTGGTTGCAGTCATCAACAATGGGCCTTATTCCCATTTCACT
						**NLRRSYKDAKAVC**	GGAAGTGATTCTAACTACATAGAATCTTTTAGTTATCCAGAC
						**TNLGLHLEMPTDPE**	AAAGACGAAAGCTTTCGCTTCGATAGACTAAGAGCACATGTT
						**ERLRMITVLSSKGIS**	CTAGCTCGACACTCCAATGAATGTGACAAAGGGCTGTCGAA
						**SIWADDQIQVLPDL**	ACTGCAGAACACGTGTGTTTACATCTCAAATCTTCGCCGCTC
						**EYDSNTANPAAIDD**	GTACAAGGATGCTAAAGCTGTTTGTACAAATTTGGGTCTGCA
						**DLSCAVASVEELEF**	TCTGGAGATGCCAACTGATCCGGAGGAAAGGCTGAGGATGA
						**KPERRPCTELMNYA**	TCACAGTGCTCAGCAGTAAAGGTATATCATCTATTTGGGCTG
						**CFMKMDATHLAVC**	ATGATCAAATTCAAGTATTGCCTGATCTGGAATATGACAGTA
						**RDPGALANGKADP**	ATACAGCAAACCCTGCAGCTATAGACGATGACCTTTCTTGTG
						**IGRIDNIFYVGSSIE**	CCGTAGCCAGTGTAGAGGAGCTAGAATTTAAACCTGAGAGA
						**YSCAAQHYLKGAK**	AGACCATGTACAGAATTGATGAATTATGCATGCTTCATGAAA
						**TISCTGNGTWSGA**	ATGGATGCTACTCATTTAGCCGTATGCCGCGATCCAGGAGCG
						**KPVCTKVDACTDP**	TTAGCTAATGGGAAAGCAGATCCAATTGGTCGAATAGATAA
						**PVPIGGFVTYLPPIK**	CATCTTCTACGTGGGATCATCCATCGAATATTCTTGTGCAGCT
						**STSAQRSAIHQSRV**	CAACATTACCTGAAGGGAGCGAAAACTATTTCTTGTACAGGA
						**PASRTGRLPVGLAA**	AATGGAACCTGGTCTGGTGCGAAGCCTGTGTGCACGAAAGTT
						**PIPQNIQASTTPEPV**	GATGCCTGTACCGATCCTCCAGTTCCTATTGGCGGCTTTGTCA
						**TIPLPPGLHRVGTR**	CTTATTTACCCCCAATAAAATCGACTTCTGCTCAAAGATCAG
						**AMYDCESRYYKLIG**	CTATTCATCAATCAAGAGTTCCCGCTTCAAGAACAGGGCGTC
						**SRTRRCQEMGEWG**	TACCTGTGGGTCTAGCTGCACCTATACCTCAGAATATACAAG
						**GRPPTCMPVCGRS**	CCTCAACAACTCCAGAACCAGTTACAATCCCTTTGCCTCCTG
						**DSPRSPFIVNGNAT**	GATTACACCGAGTGGGCACTAGAGCAATGTATGATTGCGAA

						<u>**DIGQWPWQAGIAR<br>YLQDYDRWFLLCG<br>ASLLNELWIITAAH<br>CVTYAGTTLTIEPD<br>KFQVYLGKYHRTD<br>SKDDEYVQVRKIQE<br>IHIHPDYDPGLFDA<br>DIALIQLDSPVQLN<br>SRVQPVCLPTEQTT<br>RENIAEGKKGVVT<br>GWGMNENETYSET<br>LQQAVLPVISNANC<br>EKGYEESDLPLTVT<br>DNMFCAGYAEGRT<br>DACSGDSGGPIVFT<br>DDSSKERKWVLEGI<br>VSWGSPRGCGNKN<br>QYGGFTTVSRFLD<br>WIHLYF***</u>	TCCAGATACTACAAGCTCATTGGCTCCAGAACAAGAAGATG<br>CCAAGAAATGGGAGAATGGGGTGGAAGACCGCCCACCTGCA<br>TGCCAGTCTGTGGACGTTCAGATTCTCCCCGTTCACCATTTAT<br>AGTAAATGGAAATGCTACAGATATTGGACAATGGCCGTGGC<br>AAGCAGGAATTGCCAGATATTTGCAAGACTACGACCGATGG<br>TTCCTTCTTTGCGGCGCTTCTTTACTAAATGAATTGTGGATAA<br>TTACTGCTGCACATTGCGTCACATACGCAGGAACAACGCTGA<br>CTATAGAACCTGACAAATTTCAAGTATATTTAGGAAAGTATC<br>ATCGCACAGATTCAAAAGACGATGAATATGTACAAGTAAGA<br>AAGATACAAGAAATTCACATACATCCTGATTATGATCCAGGC<br>CTATTTGATGCAGACATAGCACTTATCCAACTTGATTCACCG<br>GTGCAACTTAATTCCCGTGTACAACCTGTATGTTTACCTACTG<br>AGCAGACTACAAGAGAGAACATAGCTGAAGGAAAAAAAGG<br>AGTGGTTACAGGATGGGGGATGAATGAAAATGAAACATACT<br>CAGAAACTTTACAACAAGCAGTTCTACCAGTAATATCTAATG<br>CAAATTGTGAAAAAGGATATGAAGAATCTGATCTACCTTTAA<br>CAGTGACAGACAATATGTTCTGTGCAGGCTATGCAGAAGGT<br>AGAACAGATGCCTGCAGTGGAGACAGTGGAGGCCCAATAGT<br>ATTTACAGATGATTCTTCAAAAGAAAGAAAATGGGTCTTAGA<br>AGGAATTGTCAGCTGGGGTAGTCCTAGAGGATGTGGAAATA<br>AAAATCAATATGGAGGTTTCACAACTGTTAGCAGATTTCTGG<br>ACTGGATTCATCTTTACTTCTAAATGCTGCATATTAAAATATC<br>ATGTCATACTAATCTTTCAGTGTTTTATATTAAATGCATTCTG<br>ACAAATACAAAAAAAAA

PhdEnzSeP 68	Q4QXT9	5,87E-38	33,712	PF00008<br>PF00594<br>PF00089	112099.02<br>**109510.85**	MSYPVFLCLKLLGIF<br>LSLSSVCDG**HLILDD**<br>**GFVGVGLDLSKLGL**<br>**GKSKADGGCPPSG**<br>**NITCACAAEGYPIS**<br>**VEVPVCSYSYRWKI**<br>**SCKPCDKMTPEEVC**<br>**PKYLLCEQCHLHGG**<br>**DSCVTCPAGKFGT**<br>**WCENTCACQNGGE**<br>**CERNGKCLCPANFE**<br>**GRNCEKKKGCDSP**<br>**VGIAPPLQVAFQPP**<br>**ERPLTAAYSCPPNY**<br>**VLSGQAISTCISGK**<br>**WTSGPPSCRPKCPI**<br>**LSAPANGRLLFSGN**<br>**DLVEGVSADVECL**<br>**QGYRLVGQKTLTC**<br>**STGGRWDHDLPLC**<br>**EELASCPDPGNVN**<br>**NAERTILHRAVKVG**<br>**GHFFQDSQLQYKC**<br>**LPGYEQMGTEKILC**<br>**TFDGTWSNQPPSCL**<br>**KVATVVPDCETPGS**<br>**DVIDEEGVSVRIFCP**<br>**PECTDEDFKLWGTS**	CTCATGTAACGAATGTATAACGCCTTTGTTCATATCCGGGAA<br>ATGTTTCCTGAAAGAGGAAGCCAAATTTTTTTTAGCCTCAG<br>ATCTCTGTTATACCTCGCCCAGGTTTGGCAATGAGTTATCCCG<br>TGTTTCTCTGCTTAAAATTGCTCGGTATTTTCTTATCTCTGTCA<br>TCAGTCTGTGATGGTCATCTGATTCTGGATGATGGATTTGTGG<br>GAGTTGGACTTGACCTCTCGAAACTCGGTCTCGGTAAGAGTA<br>AAGCAGATGGAGGCTGTCCTCCATCAGGGAACATCACATGT<br>GCTTGCGCTGCCGAGGGATATCCTATTTCAGTAGAAGTGCCT<br>GTGTGCAGCTATAGCTACCGCTGGAAGATTAGCTGTAAGCCT<br>TGCGATAAGATGACGCCAGAAGAGGTCTGTCCAAAGTATCTT<br>CTCTGTGAACAATGTCATCTCCATGGAGGAGACTCTTGCGTG<br>ACCTGTCCCGCAGGCAAGTTCGGGACTTGGTGCGAAAATAC<br>GTGTGCTTGTCAGAATGGTGGGGAATGTGAACGCAATGGAA<br>AGTGCTTGTGCCCAGCAAATTTTGAAGGAAGAAACTGCGAG<br>AAAAAGAAAGGTTGTGATTCGCCTGTTGGTATCGCCCCTCCA<br>TTGCAAGTTGCATTTCAACCACCAGAACGTCCTCTTACTGCA<br>GCTTATAGCTGCCCTCCAAACTATGTATTAAGTGGCCAAGCA<br>ATTAGCACATGTATCTCTGGGAAATGGACTTCTGGACCTCCTT<br>CTTGTCGTCCGAAGTGTCCAATCCTTAGTGCACCTGCAAATG<br>GAAGATTGCTCTTCTCTGGAAATGATCTGGTGGAGGGAGTAA<br>GCGCTGATGTTGAGTGCCTGCAAGGCTATCGGTTAGTAGGAC<br>AGAAGACCTTAACATGCTCGACTGGAGGACGATGGGATCAC<br>GATCTTCCTCTGTGTGAGGAACTGGCATCGTGTCCCGATCCTG<br>GAAACGTTAACAATGCAGAAAGAACAATTCTTCATCGTGCA<br>GTCAAAGTAGGTGGCCACTTCTTTCAAGACAGCCAACTTCAG<br>TACAAGTGCCTACCCGGTTACGAACAAATGGGGACTGAGAA<br>AATCTTGTGCACTTTCGATGGTACATGGTCAAATCAGCCGCC<br>TTCTTGTCTTAAAGTGGCAACTGTTGTACCTGACTGTGAAACT

						**IYRVGSSVCQAAIH**	CCCGGATCTGATGTTATTGACGAAGAAGGCGTATCAGTGAGG
						**SAKITNSGGLVAVI**	ATTTTTTGTCCTCCGGAGTGTACTGATGAAGACTTCAAACTCT
						**NNGPYSHFTGSDS**	GGGGTACCTCTATATACAGGGTAGGATCATCAGTATGCCAAG
						**NYIESFSYPDKDESF**	CAGCCATTCACTCTGCCAAAATCACGAATAGTGGTGGATTGG
						**RFDRLRAHVLARHS**	TTGCAGTCATCAACAATGGGCCTTATTCCCATTTCACTGGAA
						**NECDKGLSKLQNT**	GTGATTCTAACTACATAGAATCTTTTAGTTATCCAGACAAAG
						**CVYISNLRRSYKDA**	ACGAAAGCTTTCGCTTCGATAGACTAAGAGCACATGTTCTAG
						**KAVCTNLGLHLEM**	CTCGACACTCCAATGAATGTGACAAAGGGCTGTCGAAACTG
						**PTDPEERLRMITVLS**	CAGAACACGTGTGTTTACATCTCAAATCTTCGCCGCTCGTAC
						**SKGISSIWADDQIQ**	AAGGATGCTAAAGCTGTTTGTACAAATTTGGGTCTGCATCTG
						**VLPDLEYDSNTANP**	GAGATGCCAACTGATCCGGAGGAAAGGCTGAGGATGATCAC
						**AAIDDDLSCAVAS**	AGTGCTCAGCAGTAAAGGTATATCATCTATTTGGGCTGATGA
						**VEELEFKPERRPCTE**	TCAAATTCAAGTATTGCCTGATCTGGAATATGACAGTAATAC
						**LMNYACFMKMDA**	AGCAAACCCTGCAGCTATAGACGATGACCTTTCTTGTGCCGT
						**THLAVCRDPGALA**	AGCCAGTGTAGAGGAGCTAGAATTTAAACCTGAGAGAAGAC
						**NGKADPIGRIDNIF**	CATGTACAGAATTGATGAATTATGCATGCTTCATGAAAATGG
						**YVGSSIEYSCAAQH**	ATGCTACTCATTTAGCCGTATGCCGCGATCCAGGAGCGTTAG
						**YLKGAKTISCTGNG**	CTAATGGGAAAGCAGATCCAATTGGTCGAATAGATAACATC
						**TWSGAKPVCTKVD**	TTCTACGTGGGATCATCCATCGAATATTCTTGTGCAGCTCAAC
						**ACTDPPVPIGGFVT**	ATTACCTGAAGGGAGCGAAAACTATTTCTTGTACAGGAAATG
						**YLPPIKSTSAQRSAI**	GAACCTGGTCTGGTGCGAAGCCTGTGTGCACGAAAGTTGATG
						**HQSRVPASRTGRLP**	CCTGTACCGATCCTCCAGTTCCTATTGGCGGCTTTGTCACTTA
						**VGLAAPIPQNIQAS**	TTTACCCCCAATAAAATCGACTTCTGCTCAAAGATCAGCTAT
						**TTPEPVTIPLPPGLH**	TCATCAATCAAGAGTTCCCGCTTCAAGAACAGGGCGTCTACC
						**RVGTRAMYDCESR**	TGTGGGTCTAGCTGCACCTATACCTCAGAATATACAAGCCTC
						**YYKLIGSRTRRCQE**	AACAACTCCAGAACCAGTTACAATCCCTTTGCCTCCTGGATT
						**MGEWGGRPPTCMP**	ACACCGAGTGGGCACTAGAGCAATGTATGATTGCGAATCCA
						**VCGRSDSPRSPFIV**	GATACTACAAGCTCATTGGCTCCAGAACAAGAAGATGCCAA

						**NGNATDIGQWPW<br>QAGIARYLQDYDR<br>WFLLCGASLLNELW<br>IITAAHCVTYAGTT<br>LTIEPDKFQVYLGK<br>YHRTDSKDDEYVQ<br>VRKIQEIHIHPDYDP<br>GLFDADIALIQLDS<br>PVQLNSRVQPVCLP<br>TEQTTRENIAEGKK<br>GVVTGWGMNENE<br>TYSETLQQAVLPVIS<br>NANCEKGYEESDLP<br>LTVTDNMFCAGYA<br>EGRTDACSGDSGG<br>PIVFTDDSSKERKW<br>VLEGIVSWGSPRGC<br>GNKNQYGGFTTVS<br>RFLDWIHLYF***	GAAATGGGAGAATGGGGTGGAAGACCGCCCACCTGCATGCC<br>AGTCTGTGGACGTTCAGATTCTCCCCGTTCACCATTTATAGTA<br>AATGGAAATGCTACAGATATTGGACAATGGCCGTGGCAAGC<br>AGGAATTGCCAGATATTTGCAAGACTACGACCGATGGTTCCT<br>TCTTTGCGGCGCTTCTTTACTAAATGAATTGTGGATAATTACT<br>GCTGCACATTGCGTCACATACGCAGGAACAACGCTGACTAT<br>AGAACCTGACAAATTTCAAGTATATTTAGGAAAGTATCATCG<br>CACAGATTCAAAAGACGATGAATATGTACAAGTAAGAAAGA<br>TACAAGAAATTCACATACATCCTGATTATGATCCAGGCCTAT<br>TTGATGCAGACATAGCACTTATCCAACTTGATTCACCGGTGC<br>AACTTAATTCCCGTGTACAACCTGTATGTTTACCTACTGAGCA<br>GACTACAAGAGAGAACATAGCTGAAGGAAAAAAAGGAGTG<br>GTTACAGGATGGGGGATGAATGAAAATGAAACATACTCAGA<br>AACTTTACAACAAGCAGTTCTACCAGTAATATCTAATGCAAA<br>TTGTGAAAAAGGATATGAAGAATCTGATCTACCTTTAACAGT<br>GACAGACAATATGTTCTGTGCAGGCTATGCAGAAGGTAGAA<br>CAGATGCCTGCAGTGGAGACAGTGGAGGCCCAATAGTATTT<br>ACAGATGATTCTTCAAAAGAAAGAAAATGGGTCTTAGAAGG<br>AATTGTCAGCTGGGGTAGTCCTAGAGGATGTGGAAATAAAA<br>ATCAATATGGAGGTTTCACAACTGTTAGCAGATTTCTGGACT<br>GGATTCATCTTTACTTCTAAATGCTGCATATTAAAATATCATG<br>TCATACTAATCTTTCAGTGTTTTATATTAAATGCATTCTGACA<br>AATACAAAAAAAAAA

PhdEnzSeP 69	Q4QXT9	5,47E-38	33,712	PF00008<br>PF00594<br>PF00089	106173,93	QRIKQDEDRLAYNR<br>CSYSYRWKISCKPCD<br>KMTPEEVCPKYLLCE<br>QCHLHGGDSCVTCP<br>AGKFGTWCENTCA<br>CQNGGECERNGKC<br>LCPANFEGRNCEKK<br>KGCDSPVGIAPPLQV<br>AFQPPERPLTAAYSC<br>PPNYVLSGQAISTCIS<br>GKWTSGPPSCRPKC<br>PILSAPANGRLLFSG<br>NDLVEGVSADVECL<br>QGYRLVGQKTLTCS<br>TGGRWDHDLPLCEE<br>LASCPDPGNVNNAE<br>RTILHRAVKVGGHF<br>FQDSQLQYKCLPGY<br>EQMGTEKILCTFDGT<br>WSNQPPSCLKVATV<br>VPDCETPGSDVIDEE<br>GVSVRIFCPPECTDE<br>DFKLWGTSIYRVGSS<br>VCQAAIHSAKITNSG<br>GLVAVINNGPYSHF<br>TGSDSNYIESFSYPDK<br>DESFRFDRLRAHVL<br>ARHSNECDKGLSKL	TTGAAAAGGTCGAAAGCACATTTTCGGTTTGTTTTGCAAGAA<br>GGTGGTAGCTGATTGGGTTCTGCAGAGTGATAAGCTGAAATA<br>CGGAGAACTAACGCAGGAAGTGCTCTTTATCTGGGTGAATCA<br>TGTGGTATAAATCTCCCGTTTAATAACTGACAGCGAATAAAG<br>CAAGATGAAGATCGCCTGGCTTATAATCGGTGCAGCTATAGC<br>TACCGCTGGAAGATTAGCTGTAAGCCTTGCGATAAGATGACG<br>CCAGAAGAGGTCTGTCCAAAGTATCTTCTCTGTGAACAATGT<br>CATCTCCATGGAGGAGACTCTTGCGTGACCTGTCCCGCAGGC<br>AAGTTCGGGACTTGGTGCGAAAATACGTGTGCTTGTCAGAAT<br>GGTGGGGAATGTGAACGCAATGGAAAGTGCTTGTGCCCAGC<br>AAATTTTGAAGGAAGAAACTGCGAGAAAAAGAAAGGTTGTG<br>ATTCGCCTGTTGGTATCGCCCCTCCATTGCAAGTTGCATTTCA<br>ACCACCAGAACGTCCTCTTACTGCAGCTTATAGCTGCCCTCC<br>AAACTATGTATTAAGTGGCCAAGCAATTAGCACATGTATCTC<br>TGGGAAATGGACTTCTGGACCTCCTTCTTGTCGTCCGAAGTGT<br>CCAATCCTTAGTGCACCTGCAAATGGAAGATTGCTCTTCTCT<br>GGAAATGATCTGGTGGAGGGAGTAAGCGCTGATGTTGAGTG<br>CCTGCAAGGCTATCGGTTAGTAGGACAGAAGACCTTAACAT<br>GCTCGACTGGAGGACGATGGGATCACGATCTTCCTCTGTGTG<br>AGGAACTGGCATCGTGTCCCGATCCTGGAAACGTTAACAATG<br>CAGAAAGAACAATTCTTCATCGTGCAGTCAAAGTAGGTGGC<br>CACTTCTTTCAAGACAGCCAACTTCAGTACAAGTGCCTACCC<br>GGTTACGAACAAATGGGGACTGAGAAAATCTTGTGCACTTTC<br>GATGGTACATGGTCAAATCAGCCGCCTTCTTGTCTTAAAGTG<br>GCAACTGTTGTACCTGACTGTGAAACTCCCGGATCTGATGTT<br>ATTGACGAAGAAGGCGTATCAGTGAGGATTTTTGTCCTCCG<br>GAGTGTACTGATGAAGACTTCAAACTCTGGGGTACCTCTATA<br>TACAGGGTAGGATCATCAGTATGCCAAGCAGCCATTCACTCT

						QNTCVYISNLRRSYK	GCCAAAATCACGAATAGTGGTGGATTGGTTGCAGTCATCAAC
						DAKAVCTNLGLHLE	AATGGGCCTTATTCCCATTTCACTGGAAGTGATTCTAACTAC
						MPTDPEERLRMITVL	ATAGAATCTTTTAGTTATCCAGACAAAGACGAAAGCTTTCGC
						SSKGISSIWADDQIQ	TTCGATAGACTAAGAGCACATGTTCTAGCTCGACACTCCAAT
						VLPDLEYDSNTANP	GAATGTGACAAAGGGCTGTCGAAACTGCAGAACACGTGTGT
						AAIDDDLSCAVASV	TTACATCTCAAATCTTCGCCGCTCGTACAAGGATGCTAAAGC
						EELEFKPERRPCTEL	TGTTTGTACAAATTTGGGTCTGCATCTGGAGATGCCAACTGAT
						MNYACFMKMDAT	CCGGAGGAAAGGCTGAGGATGATCACAGTGCTCAGCAGTAA
						HLAVCRDPGALAN	AGGTATATCATCTATTTGGGCTGATGATCAAATTCAAGTATTG
						GKADPIGRIDNIFYV	CCTGATCTGGAATATGACAGTAATACAGCAAACCCTGCAGCT
						GSSIEYSCAAQHYLK	ATAGACGATGACCTTTCTTGTGCCGTAGCCAGTGTAGAGGAG
						GAKTISCTGNGTWS	CTAGAATTTAAACCTGAGAGAAGACCATGTACAGAATTGAT
						GAKPVCTKVDACTD	GAATTATGCATGCTTCATGAAAATGGATGCTACTCATTTAGC
						PPVPIGGFVTYLPPIK	CGTATGCCGCGATCCAGGAGCGTTAGCTAATGGGAAAGCAG
						STSAQRSAIHQSRVP	ATCCAATTGGTCGAATAGATAACATCTTCTACGTGGGATCAT
						ASRTGRLPVGLAAPI	CCATCGAATATTCTTGTGCAGCTCAACATTACCTGAAGGGAG
						PQNIQASTTPEPVTIP	CGAAAACTATTTCTTGTACAGGAAATGGAACCTGGTCTGGTG
						LPPGLHRVGTRAMY	CGAAGCCTGTGTGCACGAAAGTTGATGCCTGTACCGATCCTC
						DCESRYYKLIGSRTR	CAGTTCCTATTGGCGGCTTTGTCACTTATTTACCCCCAATAAA
						RCQEMGEWGGRPP	ATCGACTTCTGCTCAAAGATCAGCTATTCATCAATCAAGAGT
						TCMPVCGRSDSPRSP	TCCCGCTTCAAGAACAGGGCGTCTACCTGTGGGTCTAGCTGC
						FIVNGNATDIGQWP	ACCTATACCTCAGAATATACAAGCCTCAACAACTCCAGAAC
						WQAGIARYLQDYDR	CAGTTACAATCCCTTTGCCTCCTGGATTACACCGAGTGGGCA
						WFLLCGASLLNELW	CTAGAGCAATGTATGATTGCGAATCCAGATACTACAAGCTCA
						IITAAHCVTYAGTTL	TTGGCTCCAGAACAAGAAGATGCCAAGAAATGGGAGAATGG
						TIEPDKFQVYLGKYH	GGTGGAAGACCGCCCACCTGCATGCCAGTCTGTGGACGTTCA
						RTDSKDDEYVQVRK	GATTCTCCCCGTTCACCATTTATAGTAAATGGAAATGCTACA
						IQEIHIHPDYDPGLF	GATATTGGACAATGGCCGTGGCAAGCAGGAATTGCCAGATA

						DADIALIQLDSPVQL<br>NSRVQPVCLPTEQTT<br>RENIAEGKKGVVTG<br>WGMNENETYSETLQ<br>QAVLPVISNANCEK<br>GYEESDLPLTVTDN<br>MFCAGYAEGRTDA<br>CSGDSGGPIVFTDDS<br>SKERKWVLEGIVSW<br>GSPRGCGNKNQYG<br>GFTTVSRFLDWIHLY<br>F*	TTTGCAAGACTACGACCGATGGTTCCTTCTTTGCGGCGCTTCT<br>TTACTAAATGAATTGTGGATAATTACTGCTGCACATTGCGTC<br>ACATACGCAGGAACAACGCTGACTATAGAACCTGACAAATT<br>TCAAGTATATTTAGGAAAGTATCATCGCACAGATTCAAAAGA<br>CGATGAATATGTACAAGTAAGAAAGATACAAGAAATTCACA<br>TACATCCTGATTATGATCCAGGCCTATTTGATGCAGACATAG<br>CACTTATCCAACTTGATTCACCGGTGCAACTTAATTCCCGTGT<br>ACAACCTGTATGTTTACCTACTGAGCAGACTACAAGAGAGA<br>ACATAGCTGAAGGAAAAAAAGGAGTGGTTACAGGATGGGG<br>GATGAATGAAAATGAAACATACTCAGAAACTTTACAACAAG<br>CAGTTCTACCAGTAATATCTAATGCAAATTGTGAAAAAGGAT<br>ATGAAGAATCTGATCTACCTTTAACAGTGACAGACAATATGT<br>TCTGTGCAGGCTATGCAGAAGGTAGAACAGATGCCTGCAGT<br>GGAGACAGTGGAGGCCCAATAGTATTTACAGATGATTCTTCA<br>AAAGAAAGAAAATGGGTCTTAGAAGGAATTGTCAGCTGGGG<br>TAGTCCTAGAGGATGTGGAAATAAAAATCAATATGGAGGTTT<br>CACAACTGTTAGCAGATTTCTGGACTGGATTCATCTTTACTTC<br>TAAATGCTGCATATTAAAATATCATGTCATACTAATCTTTCAG<br>TGTTTTATATTAAATGCATTCTGACAAATACAAAAAAAAA

PhdEnzSeP 70	Q4QXT9	1,71E-35	35,931	PF00008<br>PF00594<br>PF00089	26424,87	PSQMDLQCGGSIIHR<br>NFILTAAHCMYSGD<br>WEQKTEKSIMVKLG<br>LTDIKNETYVQESEV<br>VKIFVHPKYRPGPTY<br>DYDIALLQLDRPVEF<br>NPFVRPICLPPKELLE<br>NSPLYSSNEFGWAT<br>GWGHQGVVSAGTN<br>DRMKSSQYLKELLM<br>PIQSRERCIQSLGDN<br>NVRVNFFTDRMFCA<br>GDGKSGNDTCKGDS<br>GGPLMQSQLNSEGY<br>LYWTQVGIVSWGIG<br>CGQENTYGYYSHVQ<br>RLRP	ATCCTTCTCAAATGGATCTCCAGTGCGGAGGTTCCATTATCC<br>ATAGAAACTTCATATTGACTGCAGCTCATTGCATGTACAGTG<br>GCGACTGGGAGCAAAAGACCGAAAAATCAATCATGGTAAA<br>ATTAGGTCTGACGGATATTAAAAATGAAACATACGTTCAAG<br>AATCCGAAGTCGTGAAGATCTTCGTCCACCCCAAGTATCGTC<br>CTGGTCCAACGTACGATTACGACATTGCTCTGCTCCAGCTAG<br>ACAGACCAGTTGAATTTAACCCTTTTGTGAGACCGATTTGTTT<br>GCCACCTAAAGAATTGTTGGAGAATAGTCCATTGTATAGCTC<br>GAATGAATTCGGCTGGGCAACAGGTTGGGGTCATCAAGGAG<br>TTGTCTCTGCGGGCACGAACGACAGGATGAAGTCCAGCCAG<br>TATCTTAAAGAGCTTCTGATGCCAATTCAATCCAGAGAAAGG<br>TGTATTCAAAGTTTGGGAGATAATAACGTCAGGGTGAATTTT<br>TTCACAGATCGCATGTTTTGCGCTGGCGATGGGAAAAGCGGA<br>AATGATACATGCAAAGGAGACAGTGGGGGACCCTTAATGCA<br>ATCACAGTTAAATTCGGAAGGCTATCTCTACTGGACTCAAGT<br>AGGAATTGTCAGCTGGGGCATAGGATGTGGTCAAGAAAACA<br>CATATGGATACTACAGTCATGTTCAGAGGCTCAGGCCGT
PhdEnzSeP 71	A6MFK7	6,46E-28	36,158	PF00008<br>PF00594<br>PF00089	21303,26	THPQYRHIKNFDYDI<br>ALLELKKPIEYNPFV<br>RPICLPPKELPDGSEF<br>YKLKAPVWATGWG<br>HRDTMEKDEDNPIV<br>SPDLKEIYLPIQKRED<br>CLHSLKDDIFNASHF<br>TERMFCAGTGKGRN<br>DTCKGDSGGPMMQ<br>SHVNAEGYHSWTQ<br>VGIVSWGLGCGLKQ	TACTCATCCCCAGTATCGCCATATAAAGAATTTTGATTACGA<br>CATTGCTCTGCTCGAACTGAAGAAACCGATTGAATACAATCC<br>GTTCGTTAGACCCATTTGTTTGCCACCCAAAGAATTACCTGA<br>CGGCAGTGAGTTCTACAAGCTGAAAGCTCCCGTTTGGGCAAC<br>TGGATGGGGTCACAGAGACACAATGGAAAAAGACGAAGAC<br>AATCCAATAGTTAGTCCGGATCTGAAGGAAATCTACCTCCCC<br>ATTCAAAAAAGAGAAGATTGTCTGCACAGCCTTAAAGACGA<br>TATTTTTAATGCTAGTCATTTCACAGAGCGGATGTTCTGCGCA<br>GGTACAGGAAAAGGAAGAAACGACACCTGCAAGGGCGACA<br>GCGGAGGGCCCATGATGCAGTCACACGTAAACGCGGAGGGC<br>TATCACAGCTGGACTCAAGTGGGCATTGTCAGCTGGGGCTTG

						EYGFYTHLQKLLPWVEETIENAMKRMT*	GGATGCGGTTTGAAGCAGGAGTATGGATTCTACACTCATTTGCAGAAGCTCTTGCCATGGGTCGAAGAGACCATCGAAAACGCCATGAAACGAATGACGTAGAATAAAGTTGTTACGTATTTTGAGTGCAAACGCAAAACATTGCTTATTATTTGACTTTAGCTGCAGATGACCATCGAAATGCTTTTGAGAG

PhdEnzSeP 72	B5U2W0	4,92E-44	40,161	PF12032<br><br>PF00089	51783.16<br><br>**48495.08**	MIRPYRFKGGTICVC<br>LWILFHLCTVCEC**RR**<br>**YDKCVTRDGRQGV**<br>**CVVKRYCREASTN**<br>**NNALLPCDGDAEN**<br>**HCCPLPTTQRSAMP**<br>**QPVPARPEVYRPNN**<br>**DEKAKQRPVAPKEP**<br>**DRDDRLNKTPPPG**<br>**DRRDPGQTRTSPRP**<br>**IYDDHRAPPSRRPD**<br>**NDRIYPAKDELSTE**<br>**PPRRLNPSKHKLDD**<br>**SSKAPIRFPEPYEFS**<br>**VPSPKVPACGVKPY**<br>**ELFIAGGEESERHE**<br>**WPWMTAIFRRHPD**<br>**SRPKTFLCGGSLINT**<br>**KYVLTAAHCFVNN**<br>**YVILPASSFVVRVG**<br>**SHFLDSGEEYTVAN**<br>**LVVHHNHSGSDFF**<br>**NDIALVRLASEVYIT**<br>**DKVAPICLPDPIMIR**<br>**DNLVGRMATVAG**<br>**WGDTAFRSGHTRIL**<br>**QHVTVPIVSGEECF**<br>**AAYSRVRGAAFLAR**	ACATAATGCCCTCTGAGAAAAGTCTTATTTTTTTTATGAGAGC<br>GATGCGTGATGCTCAAATCGCGTTCGCACTAATTGTTTAACC<br>AGCTTTAAAAGCAAGCCTATACTTGTTGTTTAAAGAAAGACA<br>TCTAAACCCCATTTTGGTGTCGATGTTTTTATTTCTCATTTTTG<br>TAATATTGTGATTGCTCCCGATATGCCTTTTATCAGTTTTTAGC<br>TCAACACATAAAAAATACTTCGCATTGATGATATTTATGTTGT<br>TAGTAAGTACAGTTTTATCAAAGTTAACCGCCTAACACGATT<br>AGGGACGTTAAGCGATTAAAGCTTCAGAATTTGCTTGTCGAA<br>AGGCATCGGCGAGCTGTTGGCGAGGCCTTGGAAAATGAATA<br>ATCGTGTATGAGCAACTTAAAACTCTTTCATGCTTCGATAGTG<br>CTCAAAGAACGTTAATCCAGTGAGTTTTAAAGTACGCAAGC<br>ATTGGTTGATCCCCCCATATGAAAGGACGCAACCCACAAAT<br>ATTTATAGTGCTTCCTGCTCAATTTCTGTGATACCCCAGATTT<br>CCTGTTTCCGTACACTATACACTACCATCATTCAGTGAGCTCT<br>AAAGTATATAAACATAAGTGTCGATAACCCGATGTAGTATA<br>GCCCCCCACCCAAATTTTTATAGCACCCCAATTTCTGTGATTC<br>CTCAGATTTCCCCGTTTTCATACATCATACCCTGCCCCCTTCC<br>ACAGTAACACTACAAACTATACTTACTGTAACACCACAAGA<br>TATCCCGTTTTTATTACCCCCCTAAAATCTTTATCTCGCGCAG<br>TTGACGTCCTAAACCCCTCTTGCAGGCACCGCTGAAAGATTC<br>TGGTTTACTAATCGAAAGGATGGCGTCTGGGGCAACTACGTC<br>CTCCAGAAAACGCAGAATTGCAGTGGCTTCAAAAATAGATT<br>TTGTGTTGTACTGTCGAAATTAAATTAGGGTTCCCCCTCTTTT<br>AATGTTTCCTCTGGTTAGAGTGTTTTATTTTAATGAGTGCAAT<br>ATTAGCTTAAAATTTCCCGTGATTTCTAACCTGGAGGAACCA<br>TTTGTGAGAAATCTATTGAGGTGGAGCAGCTTGCCACATACC<br>TATTGTAACTGAAAATGCCCAGTCTTAATTTATACTCTCAACG<br>CGCGCGACAGAAGGGTCCAGTGTCTACGACTAAAGCTCTTG

						<u>**GSDHVICAGLREG GKDACLGDSGGPL MLKMPDDRWIIIGI VSLGYKCAEPGYPG VYTRVTHYMSWIN TNMKKH***</u>	AAATATTGATTAGAGCAATCCAGGCTTCCGAATGACGACGA CTCGCGAATTTCAACCCTTCACAGGATTTGACGTATGCGGTT AAATTCTCCAGTGCGAACGCGACATTCGCTTCAGTGTTTCTTC ATGTTGGTGTTTATCCAAGACATGTAGTGCGTGACTCTGGTGT AGACGCCAGGATAGCCTGGCTCGGCGCACTTGTATCCTAGAG ACACTATTCCGATGATTATCCAACGGTCGTCCGGCATTTTCA ACATCAGGGGTCCTCCCGAATCACCCAAACAAGCGTCCTTTC CGCCTTCTCTGAGTCCGGCGCATATTACGTGGTCACTCCCTCT GGCCAGAAAAGCGGCCCCTCGTACCCTCGAATAGGCGGCAA AGCACTCTTCTCCGGACACGATAGGAACAGTCACGTGTTGAA GTATCCTCGTATGACCCGAGCGGAACGCAGTGTCTCCCCATC CGGCTACTGTGGCCATCCTACCGACGAGGTTGTCTCTGATCA TGATTGGATCCGGCAGGCAAATGGGCGCCACCTTGTCGGTGA TGTAGACCTCGCTGGCCAGCCGCACAAGCGCGATGTCGTTGA AGAAGTCCGACCCGGAGTGGTTATGGTGCACCACCAGATTG GCGACTGTGTACTCCTCGCCGCTGTCCAGGAAGTGCGAGCCC ACCCGAACCACAAACGAAGAGGCTGGCAGGATGACGTAGTT GTTGACGAAACAGTGGGCGGCGGTCAGAACGTACTTGGTGTT GATGAGGGAACCCCCACACAGGAACGTCTTGGGTCGGGAAT CCGGGTGTCGACGAAAGATGGCCGTCATCCATGGCCACTCGT GTCGCTCCGATTCTTCACCCCCGGCGATGAAGAGTTCATAGG GTTTCACTCCACAAGCGGGTACTTTGGGGCTTGGAACAGAGA ACTCATAGGGTTCCGGGAACCTGATGGGAGCTTTCGAGGAAT CGTCAAGCTTGTGTTTTGAGGGATTCAACCGCCTCGGAGGTT CCGTGCTCAGTTCGTCCTTCGCTGGATATATCCGGTCGTTATC CGGACGTCTGGAAGGTGGGGCACGGTGGTCGTCGTATATAG GTCGAGGCGAAGTTCTGGTCTGACCCGGGTCTCTTCTATCGC CCGGTGGAGGTGTCTTGTTCAGACGGTCATCTCTGTCGGGCTC

							TTTCGGCGCTACGGGACGTTGCTTGGCTTTCTCGTCGTTGTTG GGCCTGTACACTTCGGGTCTCGCCGGGACTGGCTGAGGCATC GCAGACCTCTGTGTTGTGGGTAAGGGGCAACAATGATTCTCT GCATCCCCATCGCAGGGCAGCAGGGCGTTGTTGTTGGTGCTG GCCTCCCGGCAGTACCGCTTTACCACGCACACTCCCTGCCTG CCATCTCTGGTGACGCACTTGTCATATCGGCGACACTCGCAA ACGGTGCAAAGATGAAACAGAATCCACAGGCAGACGCAAA TGGTTCCTCCCTTAAAGCGATAAGGTCGGATCATCGCAGGAG AAGAAACTCTCTATCCCGATTCTTTATGCATATTCCGCTTGTT GGTTTAAGACAGGCGTGCGTCTCTCGTGACGTCTGTCCAGCT TATGCTCTTGTCGTCTTTGAGGTCTCTCTCTACGATGTGTTGTT ATTTTATTTTTCCTCCTCGTGCTCCGAA

PhdEnzSeP 73	B5U2W0	5,71E-44	40,161	PF12032<br>PF00089	51783.16<br>**48495.08**	MIRPYRFKGGTICVC	GGAATAAGTGACATAATGCAAGGAACTTTCAGACCAATTTAT
						LWILFHLCTVCEC**RR**	CTTTATTGACGCATAAAACAAAACATGTTTCATTTACCGTCTT
						**YDKCVTRDGRQGV**	GAAGTTTAGAAGGAAACGTGAGCAAATAACAGTTTAGTTAT
						**CVVKRYCREASTN**	GCCATAGACTGGCGACTAAAATATTATAGACACATTCATTGA
						**NNALLPCDGDAEN**	ATGCAGTACGGTTAAAATTAATCAACGTTGGCGTGTAAAGTT
						**HCCPLPTTQRSAMP**	CATCGTCTGCGTAAATTTGTTCGCCATGGAATACTCTGTCAAG
						**QPVPARPEVYRPNN**	CAGAAATGCTTGGCATAATGCCCTCTGAGAAAAGTCTTATTT
						**DEKAKQRPVAPKEP**	TTTTTATGAGAGCGATGCGTGATGCTCAAATCGCGTTCGCAC
						**DRDDRLNKTPPPG**	TAATTGTTTAACCAGCTTTAAAAGCAAGCCTATACTTGTTGTT
						**DRRDPGQTRTSPRP**	TAAAGAAAGACATCTAAACCCCATTTTGGTGTCGATGTTTTT
						**IYDDHRAPPSRRPD**	ATTTCTCATTTTTGTAATATTGTGATTGCTCCCGATATGCCTTT
						**NDRIYPAKDELSTE**	TATCAGTTTTTAGCTCAACACATAAAAAATACTTCGCATTGA
						**PPRRLNPSKHKLDD**	TGATATTTATGTTGTTAGTAAGTACAGTTTTATCAAAGTTAAC
						**SSKAPIRFPEPYEFS**	CGCCTAACACGATTAGGGACGTTAAGCGATTAAAGCTTCAG
						**VPSPKVPACGVKPY**	AATTTGCTTGTCGAAAGGCATCGGCGAGCTGTTGGCGAGGCC
						**ELFIAGGEESERHE**	TTGGAAAATGAATAATCGTGTATGAGCAACTTAAAACTCTTT
						**WPWMTAIFRRHPD**	CATGCTTCGATAGTGCTCAAAGAACGTTAATCCAGTGAGTTT
						**SRPKTFLCGGSLINT**	TAAAGTACGCAAGCATTGGTTGATCCCCCCATATGAAAGGA
						**KYVLTAAHCFVNN**	CGCAACCCACAAATATTTATAGTGCTTCCTGCTCAATTTCTGT
						**YVILPASSFVVRVG**	GATACCCCAGATTTCCTGTTTCCGTACACTATACACTACCATC
						**SHFLDSGEEYTVAN**	ATTCAGTGAGCTCTAAAGTATATAAACATAAGTGTCGATAAC
						**LVVHHNHSGSDFF**	CCGATGTAGTATAGCCCCCCACCCAAATTTTTATAGCACCCC
						**NDIALVRLASEVYIT**	AATTTCTGTGATTCCTCAGATTTCCCCGTTTTCATACATCATA
						**DKVAPICLPDPIMIR**	CCCTGCCCCCTTCCACAGTAACACTACAAACTATACTTACTG
						**DNLVGRMATVAG**	TAACACCACAAGATATCCCGTTTTTATTACCCCCCTAAAATCT
						**WGDTAFRSGHTRIL**	TTATCTCGCGCAGTTGACGTCCTAAACCCCTCTTGCAGGCAC
						**QHVTVPIVSGEECF**	CGCTGAAAGATTCTGGTTTACTAATCGAAAGGATGGCGTCTG
						**AAYSRVRGAAFLAR**	GGGCAACTACGTCCTCCAGAAAACGCAGAATTGCAGTGGCT

						<u>**GSDHVICAGLREGGKDACLGDSGGPLMLKMPDDRWIIIGIVSLGYKCAEPGYPGVYTRVTHYMSWINTNMKKH***</u>	TCAAAAATAGATTTTGTGTTGTACTGTCGAAATTAAATTAGGGTTCCCCCTCTTTTAATGTTTCCTCTGGTTAGAGTGTTTTATTTTAATGAGTGCAATATTAGCTTAAAATTTCCCGTGATTTCTAACCTGGAGGAACCATTTGTGAGAAATCTATTGAGGTGGAGCAGCTTGCCACATACCTATTGTAACTGAAAATGCCCAGTCTTAATTTATACTCTCAACGCGCGCGACAGAAGGGTCCAGTGTCTACGACTAAAGCTCTTGAAATATTGATTAGAGCAATCCAGGCTTCCGAATGACGACGACTCGCGAATTTCAACCCTTCACAGGATTTGACGTATGCGGTTAAATTCTCCAGTGCGAACGCGACATTCGCTTCAGTGTTTCTTCATGTTGGTGTTTATCCAAGACATGTAGTGCGTGACTCTGGTGTAGACGCCAGGATAGCCTGGCTCGGCGCACTTGTATCCTAGAGACACTATTCCGATGATTATCCAACGGTCGTCCGGCATTTTCAACATCAGGGGTCCTCCCGAATCACCCAAACAAGCGTCCTTTCCGCCTTCTCTGAGTCCGGCGCATATTACGTGGTCACTCCCTCTGGCCAGAAAAGCGGCCCCTCGTACCCTCGAATAGGCGGCAAAGCACTCTTCTCCGGACACGATAGGAACAGTCACGTGTTGAAGTATCCTCGTATGACCCGAGCGGAACGCAGTGTCTCCCCATCCGGCTACTGTGGCCATCCTACCGACGAGGTTGTCTCTGATCATGATTGGATCCGGCAGGCAAATGGGCGCCACCTTGTCGGTGATGTAGACCTCGCTGGCCAGCCGCACAAGCGCGATGTCGTTGAAGAAGTCCGACCCGGAGTGGTTATGGTGCACCACCAGATTGGCGACTGTGTACTCCTCGCCGCTGTCCAGGAAGTGCGAGCCCACCCGAACCACAAACGAAGAGGCTGGCAGGATGACGTAGTTGTTGACGAAACAGTGGGCGGCGGTCAGAACGTACTTGGTGTTGATGAGGGAACCCCCACACAGGAACGTCTTGGGTCGGGAATCCGGGTGTCGACGAAAGATGGCCGTCATCCATGGCCACTCGTGTCGCTCCGATTCTTCACCCCCGGCGATGAAGAGTTCATAGGGTTTCACTCCACAAGCGGGTACTTTGGGG

							CTTGGAACAGAGAACTCATAGGGTTCCGGGAACCTGATGGGAGCTTTCGAGGAATCGTCAAGCTTGTGTTTTGAGGGATTCAACCGCCTCGGAGGTTCCGTGCTCAGTTCGTCCTTCGCTGGATATATCCGGTCGTTATCCGGACGTCTGGAAGGTGGGGCACGGTGGTCGTCGTATATAGGTCGAGGCGAAGTTCTGGTCTGACCCGGGTCTCTTCTATCGCCCGGTGGAGGTGTCTTGTTCAGACGGTCATCTCTGTCGGGCTCTTTCGGCGCTACGGGACGTTGCTTGGCTTTCTCGTCGTTGTTGGGCCTGTACACTTCGGGTCTCGCCGGGACTGGCTGAGGCATCGCAGACCTCTGTGTTGTGGGTAAGGGGCAACAATGATTCTCTGCATCCCCATCGCAGGGCAGCAGGGCGTTGTTGTTGGTGCTGGCCTCCCGGCAGTACCGCTTTACCACGCACACTCCCTGCCTGCCATCTCTGGTGACGCACTTGTCATATCGGCGACACTCGCAAACGGTGCAAAGATGAAACAGAATCCACAGGCAGACGCAAATGGTTCCTCCCTTAAAGCGATAAGGTCGGATCATCGCAGGAGAAGAAACTCTCTATCCCGATTCTTTATGCATATTCCGCTTGTTGGTTTAAGACAGGCGTGCGTCTCTCGTGACGTCTGTCCAGCTTATGCTCTTGTCGTCTTTGAGGTCTCTCTCTACGATGTGTTGTTATTTTATTTTTCCTCCTCGTGCTCCGAA

PhdEnzSeP 74	B5U2W0	6,20E-58	33,333	PF12032<br><br>PF00089	41812,13	STPNQNNTTEELKKAIAYLRASRAGSPVMFSQGNEAKAEECMITPGGAGRCVPLVNCSAVLRTIRSQNPIICRWINDMPVVCCPEPALPRRITDSSGCGKRIIPGMSSSRRVLRQVQPTTATANSTRNQKPTIAGGEASDLGAWPWMAGIYTMNFGIENFLCGAAIVNQYYLITAAHCFGMSGRSSARIQTSRYSIRVGSIKAKEGNQHFIEKIIVHPDFIPREYYNDIAVIRLKQPISFGMNVQPICLPISPEIRRKKLKGRSVTVAGWGDLDFGGRRATILQEVTIQVIDPPTCDKAYLEQRGTSIPRGITNQFLCAGVPEGGKDACQRDSGGPLMLLDNDVWILVGVVSFGFQCAQPGFPGVYTRVTSYMDWLQEVTTT*	GTTCGAATCTGTAAATCTTCATGCAATCAACTATATAAGGAAATGTTTTGTACAAGATATGAAAATAAATTATCCTTTTCTTCTTTCAAAAGTCGGTGGAAACAGCACCTCTTGCAGCAATTTAAGTTCACTAAAACGCGGCCGCAGACTATGTAGTGGTCACTTCTTGCAGCCAGTCCATATAGCTGGTTACTCTTGTATACACCCCAGGAAATCCAGGTTGAGCACACTGGAAACCAAAAGAGACTACTCCAACTAAGATCCAAACGTCATTGTCCAGAAGCATCAAAGGTCCGCCAGAATCTCGCTGGCAGGCGTCCTTTCCACCTTCTGGAACTCCTGCACACAGAAATTGATTCGTAATGCCTCGAGGTATGGACGTTCCTCTCTGTTCTAAATAGGCTTTATCGCAAGTCGGAGGATCTATCACTTGAATTGTTACTTCTTGCAGAATAGTTGCTCGTCTACCGCCAAAGTCCAGGTCTCCCCAACCAGCGACAGTCACACTTCTGCCTTTCAGCTTCTTTCTACGGATTTCGGGAGAAATAGGCAAGCATATGGGTTGAACATTCATTCCGAAGCTGATTGGTTGCTTTAAGCGGATCACCGCGATGTCGTTGTAGTACTCTCTCGGTATGAAATCGGGATGCACGATGATTTTCTCGATGAAATGCTGATTGCCTTCTTTGGCTTTGATACTGCCGACGCGGATAGAGTACCGGGAGGTTTGGATTCGTGCTGATGATCTTCCACTCATTCCAAAGCAATGGGCAGCAGTGATGAGGTAATATTGGTTGACGATAGCAGCACCACACAGGAAGTTCTCAATGCCAAAATTCATAGTATAAATACCAGCCATCCATGGCCAAGCTCCAAGATCTGAGGCTTCTCCTCCAGCAATGGTTGGTTTCTGGTTGCGAGTACTGTTTGCGGTTGCAGTGGTTGGTTGCACTTGTCGAAGAACTCTCCTCGAGCTACTCATTCCGGGAATTATACGTTTTCCGCAGCCGCTACTATCCGTAATTCTCCTCGGTAAGGCTGGTTCCGGGCAGCATACGACAGGCATATCATTTATCCATCGGCAAATGATAGGATTTTGGGAACGAATAGTTCTGAGGACGGCACTGCAGTTGACAAGCGGAACGCATCTGCCCGCGCCTCCAGGTGTTATCATACACT

							CTTCGGCTTTCGCTTCGTTACCTTGACTAAACATCACTGGTGA TCCAGCTCTTGATGCTCTAAGATAGGCAATAGCTTTCTTTAAT TCTTCTGTTGTATTATTTTGATTTGGAGTACTT

PhdEnzSeP<br>75	B5U2W0	3,89E-58	33,333	PF12032<br><br>PF00089	41783,05	STPNQNNTTEELKK<br>AIAYLRASRAGSPV<br>MFSQGNEAKAEEC<br>MITPGGAGRCVPLV<br>NCSAVLRTIRSQNPII<br>CRWINDMPVVCCPE<br>PALPRRITDSSGCGK<br>RIIPGMSSSRRVLRQV<br>QPTTATANSTRNQK<br>PTIAGGEASDLGAW<br>PWMAGIYTMNFGIE<br>NFLCGAAIVNQYYLI<br>TAAHCFGMSGRSSA<br>RIQTSRYSIRVGSIKA<br>KEGNQHFIEKIIVHP<br>DFIPREYYNDIAVIRL<br>KQPISFGMNVQPICL<br>PIAPEIRRKDLKGRS<br>VTVAGWGDLDFGG<br>RRATILQEVTIQVIDP<br>PTCDKAYLEQRGTSI<br>PRGITNQFLCAGVPE<br>GGKDACQRDSGGPL<br>MLLDNDVWILVGV<br>VSFGFQCAQPGFPG<br>VYTRVTSYMDWLQE<br>VTTT*	GTTCGAATCTGTAAATCTTCATGCAATCAACTATATAAGGAA<br>ATGTTTTGTACAAGATATGAAAATAAATTATCCTTTTCTTCTT<br>TCAAAAGTCGGTGGAAACAGCACCTCTTGCAGCAATTTAAGT<br>TCACTAAAACGCGGCCGCAGACTATGTAGTGGTCACTTCTTG<br>CAGCCAGTCCATATAGCTGGTTACTCTTGTATACACCCCAGG<br>AAATCCAGGTTGAGCACACTGGAAACCAAAAGAGACTACTC<br>CAACTAAGATCCAAACGTCATTGTCCAGAAGCATCAAAGGT<br>CCGCCAGAATCTCGCTGGCAGGCGTCCTTTCCACCTTCTGGA<br>ACTCCTGCACACAGAAATTGATTCGTAATGCCTCGAGGTATG<br>GACGTTCCTCTCTGTTCTAAATAGGCTTTATCGCAAGTCGGAG<br>GATCTATCACTTGAATTGTTACTTCTTGCAGAATAGTTGCTCG<br>TCTACCGCCAAAGTCCAGGTCTCCCCAACCAGCGACAGTCAC<br>ACTTCTGCCTTTTAAATCCTTTCTACGGATTTCGGGAGCAATA<br>GGCAAGCATATGGGTTGAACATTCATTCCGAAGCTGATTGGT<br>TGCTTTAAGCGGATCACCGCGATGTCGTTGTAGTACTCTCTCG<br>GTATGAAATCGGGATGCACGATGATTTTCTCGATGAAATGCT<br>GATTGCCTTCTTTGGCTTTGATACTGCCGACGCGGATAGAGT<br>ACCGGGAGGTTTGGATTCGTGCTGATGATCTTCCACTCATTCC<br>AAAGCAATGGGCAGCAGTGATGAGGTAATATTGGTTGACGA<br>TAGCAGCACCACACAGGAAGTTCTCAATGCCAAAATTCATA<br>GTATAAATACCAGCCATCCATGGCCAAGCTCCAAGATCTGA<br>GGCTTCTCCTCCAGCAATGGTTGGTTTCTGGTTGCGAGTACTG<br>TTTGCGGTTGCAGTGGTTGGTTGCACTTGTCGAAGAACTCTCC<br>TCGAGCTACTCATTCCGGGAATTATACGTTTTCCGCAGCCGCT<br>ACTATCCGTAATTCTCCTCGGTAAGGCTGGTTCCGGGCAGCA<br>TACGACAGGCATATCATTTATCCATCGGCAAATGATAGGATT<br>TTGGGAACGAATAGTTCTGAGGACGGCACTGCAGTTGACAA<br>GCGGAACGCATCTGCCCGCGCCTCCAGGTGTTATCATACACT

							CTTCGGCTTTCGCTTCGTTACCTTGACTAAACATCACTGGTGA TCCAGCTCTTGATGCTCTAAGATAGGCAATAGCTTTCTTTAAT TCTTCTGTTGTATTATTTTGATTTGGAGTACTT

PhdEnzSeP 76	B5U2W0	5,30E-31	37,868	PF12032<br><br>PF00089	30389.46<br><br>**28190.79**	MTTFLVFSVLCLGVWTSVRG**NTYECGLKSATGNATDGGWPWMAAINFRRRSGAYTPWCSGFLIDRRHILTSATCTEKREAGLYSARIGNVDQDEADEYFISQITVPEGYKAGSFYDDIAILKLTREVNTPNFVPICLPDEKIMDVVFSGASMTAAGWGGEEQGSQMSRNLKVLSHFHFVSNEECSRIFQNLIRSFRRTFPRGMTSGLLCASFPEGGKDVCGGDSGGPLMLKDRSNRWFAIGIISFGFRCGEPGIPGGYTRVSHYLDWIRQNTKD***	CGGCATTTGAGCTCAAGCACGAACAAAAATTTTAAATCCACATAAATACCCAAGAAGAATTTTCAGTAAGCATGAAGAAATGTTCTGTCATGAAATAAATATTCACTCAGAACTCAGTCTTTAGTATTCTGTCTTATCCAGTCCAAGTAATGAGACACTCTGGTATATCCACCAGGTATCCCAGGCTCACCACATCTAAAACCGAAGGAGATGATTCCAATAGCGAACCATCGATTACTCCTGTCTTTGAGCATAAGAGGTCCTCCAGAGTCACCCCCACAAACATCTTTACCACCTTCTGGAAATGACGCGCATAAAAGGCCACTTGTCATACCACGGGGAAAGGTACGTCGGAAACTTCTTATTAAATTTTGAAATATTCGACTGCATTCTTCGTTAGATACAAAGTGGAAATGGCTCAGTACCTTCAAATTACGGCTCATCTGGGAACCTTGTTCTTCACCTCCCCATCCTGCAGCTGTCATTGACGCGCCGCTGAAGACCACATCCATTATTTTTTCATCAGGCAAGCATATAGGAACAAAATTCGGAGTATTCACTTCTCTGGTCAATTTTAGTATTGCTATATCATCATAGAAGGAGCCTGCTTTGTAACCTTCTGGGACTGTTATTTGACTTATAAAGTATTCATCAGCTTCATCCTGATCGACATTTCCGATACGAGCCGAATAAAGTCCTGCCTCTCTCTTCTCAGTGCATGTGGCCGATGTCAGAATGTGTCTTCTGTCGATAAGAAATCCCGAACACCAAGGCGTGTAGGCTCCAGATCTCCGTCGAAAGTTGATGGCCGCCATCCATGGCCATCCCCCATCTGTCGCATTTCCGGTGGCGCTTTTGAGTCCGCATTCATAGGTATTGCCTCGTACACTGGTCCAAACTCCTAAGCATAAAACAGAGAAAACCAAGAATGTTGTCATCTCTCAGGTTTTGAGTTTGAAACTATTCTATCCGTCACAATGCTG

PhdEnzSeP 77	B5U2W0	2,87E-48	36,364	PF12032<br><br>PF00089	47169.66<br><br>**44621.42**	MKISCKQILFLSILCL<br>LGWIVSG**QDYDDP**<br>**DDMDPRFHERERS**<br>**CNYRRQQGFCRRR**<br>**SDCARPTRHICRFG**<br>**FNPVVCCLDQPEVK**<br>**TTTTTKQPVTTRRS**<br>**DVKPESVKSLDLTF**<br>**PGCGLRAPRIQGST**<br>**RPGSNNRNSNNRQ**<br>**RGFLSRGRRSILDD**<br>**FSYSNATRERRAIT**<br>**QPVIVGGVTAVAN**<br>**SWPWMVAIFKETN**<br>**ARSPKRFLCGASLIS**<br>**RKYVMSAAHCFDA**<br>**ERGNIDASKFFVVV**<br>**GAHSTKDGVEYPV**<br>**QSILIHPDYKQRQY**<br>**YNDISLLRIRNEVQL**<br>**TQKVYPVCVPSDG**<br>**LRDKIKPNNNNVT**<br>**VTGWGDTSFGGVS**<br>**SKVLQELTIPIVPLK**<br>**ECNEAFSKLLQSTFP**<br>**NGITNLFICAGVEE**<br>**GGKDACQGDSGG**<br>**PLVSILKDNSWVQL**	AAACAGCATGCGGTGACAGTCGGCTAAAAGCCTGATGGGAA<br>TTTTAAATTGTTTCCCGTAAATGTGAAAGAGGATTTCGAACA<br>GAGTTGCTGTCATTAGAGACTCGCAGAGTGCCGTGGGATCGC<br>CGAAAAAGGTGAAGAAGTAACTCCATAATGAAAATTTCATG<br>CAAGCAGATTTTGTTTCTTTCAATATTATGCCTGCTTGGCTGG<br>ATAGTCAGTGGCCAGGATTATGACGACCCAGACGACATGGA<br>TCCGAGGTTTCACGAGCGTGAACGTAGCTGTAACTATAGACG<br>TCAACAAGGATTTTGTCGGAGGCGATCGGACTGCGCTCGGCC<br>AACTAGGCATATTTGCAGATTCGGCTTTAACCCCGTCGTTTGT<br>TGCCTGGATCAGCCAGAGGTTAAAACTACTACAACAACGAA<br>GCAGCCCGTCACAACTAGGCGATCTGACGTTAAACCCGAGT<br>CAGTGAAATCTCTAGACCTAACGTTTCCAGGTTGTGGTTTAA<br>GAGCACCTAGAATTCAAGGAAGTACAAGACCAGGTTCAAAT<br>AACAGAAATTCAAACAACAGGCAAAGAGGTTTTCTTTCAAG<br>GGGGAGAAGATCCATCCTGGATGATTTTTCATATTCAAATGC<br>AACTAGAGAAAGAAGAGCTATTACACAACCTGTTATTGTTGG<br>AGGAGTCACTGCAGTGGCTAATTCTTGGCCGTGGATGGTTGC<br>AATATTCAAAGAAACGAATGCTAGGTCTCCTAAGCGATTCCT<br>TTGTGGGGCTTCCCTCATCAGCCGAAAGTACGTTATGAGTGC<br>TGCCCACTGTTTTGATGCAGAAAGGGGAAACATTGATGCATC<br>GAAGTTTTTCGTGGTCGTTGGAGCACACTCAACAAAAGACGG<br>CGTAGAGTATCCTGTGCAGAGCATCTTAATACACCCCGATTA<br>CAAACAGCGCCAGTACTACAACGATATCTCTCTGCTTCGTAT<br>TAGGAATGAAGTGCAATTGACGCAGAAAGTTTATCCAGTGTG<br>CGTGCCATCAGATGGACTCAGAGATAAGATAAAACCAAACA<br>ACAACAATGTAACAGTCACAGGATGGGGAGACACATCATTT<br>GGTGGAGTAAGCAGCAAAGTTTTACAAGAGCTTACAATACC<br>GATTGTTCCACTGAAAGAGTGCAACGAAGCCTTTTCAAAGCT

						**GIVSFGYGCAQPGYPGVYTRVSQYTKWLYDNTDLGKA***	TCTACAGTCAACCTTTCCAAATGGTATAACAAATCTGTTCATTTGTGCTGGCGTTGAAGAAGGAGGGAAAGATGCTTGCCAAGGTGACTCGGGTGGTCCGCTAGTTTCCATCTTGAAAGATAATTCTTGGGTGCAACTTGGAATTGTTTCTTTTGGCTACGGATGTGCTCAACCTGGATATCCTGGCGTATACACTAGAGTTTCTCAGTATACAAAATGGCTTTATGATAACACAGACCTTGGTAAAGCTTGAAACAGAAATAATGACTTCTGATGTTAGATGTGGCAGCTATAACTTGTTGAGAATTAAAATATTTATCATCTGTATTTTAAACAAAGTACTAAATAAAAGCTATTTTGAAAGCGAAAAAA

PhdEnzSeP<br>78	B5U2W0	5,91E-29	36,929	PF12032<br><br>PF00089	36427.31<br><br>**34070.44**	MQLRIYLLTAATFLACVASAFA**FVQLPGILPQRDDSKEKGTGESDKGAGGLLSNLGGAVPKDLPLLGDDSSEEEETGGSDKGLASNVRDVVPKGLREPVTNEGKDAESGQFPWSAALFRNGDFICTSSILSENIVLTAAHCIDSEEVEVYHAIANVNKNSPNVRIEFASITIHPDYSESGIKNDIAIFKTKKPIPFSENVQPICIAREDTAPMEYTNATAMGWGDTSRLRVVQMMPDNMKYFEQKIIPNDECRLHYFGGALDDNKLCAGDLLKGVCNGDSGGPLVHLSADKVPVLIGVLSYGNKVTGCGFLTPSVFARVSNFNAFIKKIAEEEKICEV***	CTCTGATCTTGCATAAAAGGGGGATTGCGAACAGCGAGTGGAACTTTTCGTTCGTTTGTCGCTTTCCAAGATGCAACTCCGGATTTATCTCCTTACGGCAGCTACATTTCTGGCGTGCGTTGCCTCGGCATTTGCGTTTGTTCAGCTACCAGGCATTCTACCCCAAAGAGACGATAGCAAAGAGAAAGGAACAGGAGAAAGTGATAAGGGGGCAGGAGGGTTATTGTCGAATCTCGGTGGTGCAGTACCAAAAGACCTTCCTCTTCTCGGAGATGACAGCAGTGAAGAAGAAGAAACTGGAGGAAGTGATAAGGGACTTGCGTCGAATGTTCGTGACGTGGTGCCAAAAGGGCTTAGAGAACCAGTAACAAACGAAGGGAAGGATGCCGAATCAGGACAGTTCCCGTGGTCGGCCGCTCTGTTCAGAAATGGGGATTTCATATGCACCAGCTCTATCCTGTCAGAAAATATTGTCTTGACCGCAGCTCACTGCATCGACAGCGAGGAAGTTGAAGTGTATCACGCCATCGCCAACGTCAATAAGAACTCGCCTAATGTTCGAATCGAGTTTGCCTCCATTACGATCCACCCAGATTACTCTGAATCCGGCATAAAGAACGACATAGCCATCTTCAAGACTAAGAAGCCCATCCCCTTCAGTGAAAACGTACAGCCCATTTGTATTGCCCGCGAGGACACGGCTCCGATGGAATACACCAATGCCACGGCCATGGGATGGGGTGACACTAGTAGACTCAGGGTTGTTCAAATGATGCCGGACAATATGAAGTACTTCGAACAGAAAATCATCCCTAACGATGAATGCAGACTGCATTATTTTGGAGGAGCGTTAGACGACAACAAGCTCTGCGCCGGTGACTTGCTGAAAGGAGTTTGCAACGGTGATTCCGGAGGTCCCCTGGTGCACCTGAGTGCCGACAAGGTTCCCGTCCTGATAGGTGTCCTCTCCTATGGAAACAAAGTCACCGGATGCGGTTTTCTCACGCCAAGTGTGTTCGCTCGGGTGAGCAATTTCAACGCCTTCATCAAAAAGATCGCCGAAGAAGAAAAAATTTGCGAAGTCTGATCACTTGCGTATTTTACCTTCAAATCCTTGAACAAACTAATCGCTAACAAACCCATGTAATAACTAATAGCCGTG

PhdEnzSeP 79	B5U2W0	1,31E-53	40,590	PF12032<br><br>PF00089	47795.27<br><br>**45201.02**	MRILEVCQLLVIFVF<br>LFIAVSSA**GRTKRQI**<br>**IFEDDNTNRLFNAD**<br>**CRTPDRRPGRCIRL**<br>**TSCRALSQSISRNRL**<br>**RRYFCGFEGDEPRV**<br>**CCPLSRDEVAPTTP**<br>**TVRTTLRTTPTTVP**<br>**PTRRPPNTPPTRRPP**<br>**NTPPTRRPTVRTTT**<br>**PIDEETAERNAGGR**<br>**PDAGSETRPAFLPG**<br>**SCGQSRHTNTRVV**<br>**GGSPAENGSWPW**<br>**MAVVYVRRSNGAL**<br>**VADCGGALVTNQ**<br>**HVVTAAHCVVTGR**<br>**RSIPLDPSRVVVRL**<br>**GTHNLTIPNEPGSIE**<br>**VDVESIQRHEQFES**<br>**RTYRNDIAVLKLRT**<br>**PIPFTPKIQPVCLPY**<br>**DSLRGEDLTSKGVI**<br>**VIGYGTTAFNGNSS**<br>**DVLMQAGFYIQSQ**<br>**EVCRRAYEPEVQIS**<br>**RVYLCAGMMDGS**<br>**KDACQGDSGGPLL**	CGGAGAGAGACATGTCCTCAGAGTCAAAGAACAACTCTCTA<br>AGAAAGATGCATCAGGTAAAAATCAGCAGCTTTCGCGAAGT<br>GACGTCACCGGTGCCTTTCCGGTGCCTTATCTATAGGAAACC<br>GGATTGCTGAGCAGGATGCCATTCTCTTTCAGAGCTCCAAGC<br>TGTGAAATAATAATTTAAGAAATATGAGAATCTTGGAAGTAT<br>GTCAGTTACTGGTAATTTTCGTATTTCTCTTCATAGCAGTTTCC<br>TCGGCAGGCAGGACAAAGCGACAAATTATCTTCGAAGATGA<br>CAACACCAACCGACTCTTCAACGCAGACTGTCGGACGCCGG<br>ACCGGAGGCCAGGCCGCTGCATTCGGCTGACGTCATGCAGG<br>GCTCTTAGCCAATCGATCAGTAGGAATAGACTGCGGCGCTAC<br>TTCTGCGGGTTCGAAGGGGATGAGCCAAGAGTATGCTGTCCG<br>CTGTCCAGAGATGAAGTCGCCCCGACAACCCCAACAGTGAG<br>GACGACTTTGAGAACCACTCCCACAACAGTGCCACCAACAA<br>GACGTCCTCCAAACACTCCTCCAACAAGACGTCCTCCCAACA<br>CTCCCCCAACGAGACGTCCTACAGTGAGGACAACCACACCG<br>ATCGATGAGGAAACGGCCGAGAGGAATGCAGGAGGAAGGC<br>CAGATGCGGGCTCTGAGACCAGGCCTGCTTTCCTGCCAGGAA<br>GCTGTGGACAATCGAGGCACACTAACACGCGAGTTGTGGGT<br>GGATCTCCAGCTGAGAATGGAAGCTGGCCTTGGATGGCCGT<br>AGTGTACGTGAGAAGGAGTAACGGCGCCCTTGTTGCCGACT<br>GTGGAGGTGCCCTGGTCACGAACCAGCATGTGGTGACCGCT<br>GCCCACTGTGTCGTCACTGGACGCAGATCTATCCCCCTGGAT<br>CCTTCGAGGGTGGTCGTTCGACTGGGAACCCACAACCTGACC<br>ATTCCGAACGAGCCTGGCTCCATCGAAGTCGACGTGGAGTCG<br>ATTCAGCGCCACGAACAGTTCGAGTCTCGCACTTACAGAAAT<br>GACATCGCGGTGCTGAAGCTGCGCACACCCATCCCTTTCACT<br>CCCAAAATACAGCCTGTCTGCCTACCCTACGACAGCTTGAGA<br>GGGGAGGACCTCACCTCAAAAGGGGTGATCGTCATTGGCTA

						**TIGRNNSYYLIGVV<br>SFGRQCGQPNYPG<br>VYTRVTEFLDWLAR<br>HLSN***	TGGCACTACGGCCTTCAATGGCAATTCGAGTGATGTTCTAAT<br>GCAAGCAGGCTTCTATATTCAGAGCCAAGAAGTGTGCAGAA<br>GAGCGTATGAACCCGAAGTGCAAATCTCTAGGGTTTATTTGT<br>GTGCAGGTATGATGGACGGATCCAAAGATGCTTGCCAGGGA<br>GACTCAGGAGGTCCACTACTGACAATTGGCAGGAATAACAG<br>CTACTATCTCATAGGAGTTGTATCATTTGGAAGACAATGTGG<br>GCAACCAAATTATCCTGGAGTTTACACGCGAGTTACAGAGTT<br>TCTGGACTGGTTAGCCAGACATCTTTCAAATTGAAACATCAT<br>TGAAAAAAAAAGACAAACTTGCAGAGAATATAAGATCGAA<br>AAATTCAAATGCAATAATTCCCTGTCGATAGTAAATATGAAT<br>ATCATAGCTGTCGTCTGTTCAGCAAATGTAGAAAAGTATATT<br>TGTAATACTGGCTCAGCATGGAATAATTAGACGATTTTTGCA<br>GGAGGGACATATTAGCAAGGAAATCATTACACACATCGCAA<br>GAAATAAAGAACTCTAAAAGTACTGATCATTCGATTGTTATT<br>ACTGTAAATATTCTGCAACAATAGATTAGAAGATTTCTTCCA<br>TTGTGATATGATTTTCATTTTCACTTTCTCTGAAGATACCTGA<br>ATCTAAATATTGCAGAGAAACGACATTTTACTTATTTTAAGA<br>GCACAGAGTATAAGATTCATGAAACGATATCATATGAGATA<br>TAAGAGATCTATATACAATTTGTATTTACACATTTCCTATTTT<br>CAAGTGGCAGAGGGTGGACGTTTATCATTTCATTGCAGATTA<br>GAAAATGCAGATTTCAAATATTTCATTCCGAAATTCTCTACT<br>GTGAAGAAGTTTAAAAAAAAAGTTTACGATGACAAGAATGTA<br>TTTCATCCTACTAAAATACTGTAATATATAATTTAGGTAGTTA<br>ACCCCCTTTGCAAAGCAAAATTTGACTAACAACAGATCTAAC<br>AAACTTTGGCAAATTTTGCGCAGTGCACAAAACGTGCACAA<br>GAAAGATTTTTACTGCAAAGATAGCGAGATGTGACAATTCA<br>ATTCCCGGAACGGTTCGTGTGGTGATTTGGTTGGCATCATGTG<br>CATAATCGTGTTTGTACGTGTTTTAACTAATGTACTGCGTTAA

							CCTCAAGCCGATACAACCATTTGTTAGTTGGTTATTGCGAAA<br>ACAACGAAGATCTATGTGTGAACTTTGCCATAAAAATAAGTG<br>ATTGAAACTTAGATGAATAATCTCTTGATTCACCTTTCTGCAA<br>ATAAGCACATGTTTTCGAAAGTAATTATGAGGGACGAAACCT<br>AATGTTTCCAATACGATCCCAAAGTAAAATCCCCATAATATA<br>CAGTGGAAAACCGCTAATTCTCCATGTCCAAATGTTCCATGT<br>CCCCCCACGTGTGTCCCATTTCCAGTTCAAGATCATGTGGTGC<br>GTTTCTTTGATCACACGATGGTAAGTCCACCGTGAATTTATTT<br>AAAGAGGACAAACGGTCGATCAGTAATGCAATTCAGAAGTT<br>CTGCCATGATTACCGCATTATGATTACACTTATTATGAACACT<br>ACTGTTAATTTTAATTACTATCAATTTTAATTTTCTATAGTGCG<br>TTGCGATTATAAAATTTTTACAAGCAGATGTCGTAGTTCAGTT<br>CTTTACCTGTCTTTACCAAATTAGACCAAGCTAACGACCAAT<br>AAGTTTAAATGGTGCCTACAAAACCAAGCAGTGAAGGAGAT<br>AAACAGAGCGATAAGAAACGTCTGATGCAATATTATTGCTC<br>ACAATTTATTTTAATTGCAGCTTTTGATCTCAAAATTATAAAG<br>AAATCTCTTGATGTTCGTCAATACATTAGTTAACGCAAGACT<br>GTGGACACATAGCATTATCAGTGAGAAGGTACAAGAATAAA<br>GAAATATATACCAGGTGGATGAAAAATAACTCTTTATCCAA<br>AATATTTAATAACTTTGATACTAATTTATCGATTTTAATGGGG<br>CAAGTTTTACATTGCACAGTAGGATGATACAACTTTACTACA<br>GTGTTAAAAATTCGCGTAGGCACAAGTGTTAAAATTCAGCAT<br>AGGCAACGAATGTTTTCCTCGATCCGAATGCTTTTACTGTAG<br>ACGTTTCCTCCGCTCACCCCTACAGGAAAAAGTCACATGGGG<br>TGAGGTAAGTACATCATCACTCAAAAATGAATGAGATGAAA<br>CCAGGAATTAGCATCACAGTTCTACCACTTTCGGAATCACAA<br>TAATTTTCTATTTCACGTAAATATTATAATTTTCAAAGAAATT<br>ATTAACAGTCTTCAATAGGGAGTTATTTTTCGCTCATTCTGTA

							CATATATATATATATATATACACTAAGATTACGAAATTCTGTT<br>CCAAGGAAAACACCTGAATTGTGGCCTGACAAGTGAATAAT<br>CCATCACGACACTCATTGTGAGAGTTTCTGGCCAAAATGTCG<br>ATTACCGAATTGGATTATCCTGCACAATCCTCGGCGTAGCCC<br>CTGTGATTTCTGACTGCGGCAATAAATTTTTCTGTCCAACACA<br>TAGGATTAAGTGACACAAAATTACACTAGAGAAATAGTTCA<br>AGGGTTGTTTTCAGAAATGGGAAGACGGTTTAAGCCAGTGTG<br>TAAATTCATAAAAGAACCTTCGAAGACGTCGGTAGGCACTA<br>ATGCACAGTTCAAAGCATATGGTTATTGTACAGTTACAATCC<br>GGGAATTACTGTCACACCTTGTAAATAAAGTTATAATGCAAA<br>TAATTTTGCATTGTAACTGCGTTAAATTTTATTACTTTTTATTT<br>CAACTTAAAAGTTTTTGCGCTTTCCTTTGAGAAATTGAATCTT<br>ATTTTAGTTAACAGTATAACAATAAAGATCTTAATACTTAATT<br>TTATATCTAGCTGTTATCAGCGCACACAGTACAAATTTTTCAT<br>TAAATGGAATACATGTATGGCTCTCTACTTCAATTTTTTTTCA<br>AAGAAAAAGCACTTTATTTGTTCCCAAT

PhdEnzSeP 80	B5U2W0	1,86E-53	40,590	PF12032<br><br>PF00089	47795.27<br><br>**45201.02**	MRILEVCQLLVIFVF<br>LFIAVSSA**GRTKRQI**<br>**IFEDDNTNRLFNAD**<br>**CRTPDRRPGRCIRL**<br>**TSCRALSQSISRNRL**<br>**RRYFCGFEGDEPRV**<br>**CCPLSRDEVAPTTP**<br>**TVRTTLRTTPTTVP**<br>**PTRRPPNTPPTRRPP**<br>**NTPPTRRPTVRTTT**<br>**PIDEETAERNAGGR**<br>**PDAGSETRPAFLPG**<br>**SCGQSRHTNTRVV**<br>**GGSPAENGSWPW**<br>**MAVVYVRRSNGAL**<br>**VADCGGALVTNQ**<br>**HVVTAAHCVVTGR**<br>**RSIPLDPSRVVVRL**<br>**GTHNLTIPNEPGSIE**<br>**VDVESIQRHEQFES**<br>**RTYRNDIAVLKLRT**<br>**PIPFTPKIQPVCLPY**<br>**DSLRGEDLTSKGVI**<br>**VIGYGTTAFNGNSS**<br>**DVLMQAGFYIQSQ**<br>**EVCRRAYEPEVQIS**<br>**RVYLCAGMMDGS**<br>**KDACQGDSGGPLL**	GATTTGTTTATTCTTCTGAATCACAAATTAAACAGAACGAGG<br>TTCTTAACCGAGAAAGATTTTACAGCAGTCTAATTTTCTTTCT<br>GTTTGTGAATTCTAGTTCTTGGAAATCGATAAAGACTATTGA<br>ACAAATAATAATTTAAGAAATATGAGAATCTTGGAAGTATGT<br>CAGTTACTGGTAATTTTCGTATTTCTCTTCATAGCAGTTTCCTC<br>GGCAGGCAGGACAAAGCGACAAATTATCTTCGAAGATGACA<br>ACACCAACCGACTCTTCAACGCAGACTGTCGGACGCCGGAC<br>CGGAGGCCAGGCCGCTGCATTCGGCTGACGTCATGCAGGGC<br>TCTTAGCCAATCGATCAGTAGGAATAGACTGCGGCGCTACTT<br>CTGCGGGTTCGAAGGGGATGAGCCAAGAGTATGCTGTCCGCT<br>GTCCAGAGATGAAGTCGCCCCGACAACCCCAACAGTGAGGA<br>CGACTTTGAGAACCACTCCCACAACAGTGCCACCAACAAGA<br>CGTCCTCCAAACACTCCTCCAACAAGACGTCCTCCCAACACT<br>CCCCCAACGAGACGTCCTACAGTGAGGACAACCACACCGAT<br>CGATGAGGAAACGGCCGAGAGGAATGCAGGAGGAAGGCCA<br>GATGCGGGCTCTGAGACCAGGCCTGCTTTCCTGCCAGGAAGC<br>TGTGGACAATCGAGGCACACTAACACGCGAGTTGTGGGTGG<br>ATCTCCAGCTGAGAATGGAAGCTGGCCTTGGATGGCCGTAGT<br>GTACGTGAGAAGGAGTAACGGCGCCCTTGTTGCCGACTGTGG<br>AGGTGCCCTGGTCACGAACCAGCATGTGGTGACCGCTGCCC<br>ACTGTGTCGTCACTGGACGCAGATCTATCCCCCTGGATCCTTC<br>GAGGGTGGTCGTTCGACTGGGAACCCACAACCTGACCATTCC<br>GAACGAGCCTGGCTCCATCGAAGTCGACGTGGAGTCGATTC<br>AGCGCCACGAACAGTTCGAGTCTCGCACTTACAGAAATGAC<br>ATCGCGGTGCTGAAGCTGCGCACACCCATCCCTTTCACTCCC<br>AAAATACAGCCTGTCTGCCTACCCTACGACAGCTTGAGAGG<br>GGAGGACCTCACCTCAAAAGGGGTGATCGTCATTGGCTATG<br>GCACTACGGCCTTCAATGGCAATTCGAGTGATGTTCTAATGC

						**<u>TIGRNNSYYLIGVVSFGRQCGQPNYPGVYTRVTEFLDWLARHLSN*</u>**	AAGCAGGCTTCTATATTCAGAGCCAAGAAGTGTGCAGAAGAGCGTATGAACCCGAAGTGCAAATCTCTAGGGTTTATTTGTGTGCAGGTATGATGGACGGATCCAAAGATGCTTGCCAGGGAGACTCAGGAGGTCCACTACTGACAATTGGCAGGAATAACAGCTACTATCTCATAGGAGTTGTATCATTTGGAAGACAATGTGGGCAACCAAATTATCCTGGAGTTTACACGCGAGTTACAGAGTTTCTGGACTGGTTAGCCAGACATCTTTCAAATTGAAACATCATTGAAAAAAAAAGACAAACTTGCAGAGAATATAAGATCGAAAAATTCAAATGCAATAATTCCCTGTCGATAGTAAATATGAATATCATAGCTGTCGTCTGTTCAGCAAATGTAGAAAAGTATATTTGTAATACTGGCTCAGCATGGAATAATTAGACGATTTTTGCAGGAGGGACATATTAGCAAGGAAATCATTACACACATCGCAAGAAATAAAGAACTCTAAAAGTACTGATCATTCGATTGTTATTACTGTAAATATTCTGCAACAATAGATTAGAAGATTTCTTCCATTGTGATATGATTTTCATTTTCACTTTCTCTGAAGATACCTGAATCTAAATATTGCAGAGAAACGACATTTTACTTATTTTAAGAGCACAGAGTATAAGATTCATGAAACGATATCATATGAGATATAAGAGATCTATATACAATTTGTATTTACACATTTCCTATTTTCAAGTGGCAGAGGGTGGACGTTTATCATTTCATTGCAGATTAGAAAATGCAGATTTCAAATATTTCATTCCGAAATTCTCTACTGTGAAGAAGTTTAAAAAAAAAGTTTACGATGACAAGAATGTATTTCATCCTACTAAAATACTGTAATATATAATTTAGGTAGTTAACCCCCTTTGCAAAGCAAAATTTGACTAACAACAGATCTAACAAACTTTGGCAAATTTTGCGCAGTGCACAAAACGTGCACAAGAAAGATTTTTACTGCAAAGATAGCGAGATGTGACAATTCAATTCCCGGAACGGTTCGTGTGGTGATTTGGTTGGCATCATGTGCATAATCGTGTTTGTACGTGTTTTAACTAATGTACTGCGTTAACCTCAAGCCGATACAACCATTTGTTAGTTGGTTATTGCGAAAACAA

							CGAAGATCTATGTGTGAACTTTGCCATAAAAATAAGTGATTG AAACTTAGATGAATAATCTCTTGATTCACCTTTCTGCAAATA AGCACATGTTTTCGAAAGTAATTATGAGGGACGAAACCTAAT GTTTCCAATACGATCCCAAAGTAAAATCCCCATAATATACAG TGGAAAACCGCTAATTCTCCATGTCCAAATGTTCCATGTCCC CCCACGTGTGTCCCATTTCCAGTTCAAGATCATGTGGTGCGTT TCTTTGATCACACGATGGTAAGTCCACCGTGAATTTATTTAAA GAGGACAAACGGTCGATCAGTAATGCAATTCAGAAGTTCTG CCATGATTACCGCATTATGATTACACTTATTATGAACACTACT GTTAATTTTAATTACTATCAATTTTAATTTTCTATAGTGCGTTG CGATTATAAAATTTTTACAAGCAGATGTCGTAGTTCAGTTCTT TACCTGTCTTTACCAAATTAGACCAAGCTAACGACCAATAAG TTTAAATGGTGCCTACAAAACCAAGCAGTGAAGGAGATAAA CAGAGCGATAAGAAACGTCTGATGCAATATTATTGCTCACA ATTTATTTTAATTGCAGCTTTTGATCTCAAAATTATAAAGAAA TCTCTTGATGTTCGTCAATACATTAGTTAACGCAAGACTGTGG ACACATAGCATTATCAGTGAGAAGGTACAAGAATAAAGAAA TATATACCAGGTGGATGAAAAATAACTCTTTATCCAAAATAT TTAATAACTTTGATACTAATTTATCGATTTTAATGGGGCAAGT TTTACATTGCACAGTAGGATGATACAACTTTACTACAGTGTT AAAAATTCGCGTAGGCACAAGTGTTAAAATTCAGCATAGGC AACGAATGTTTTCCTCGATCCGAATGCTTTTACTGTAGACGTT TCCTCCGCTCACCCCTACAGGAAAAAGTCACATGGGGTGAG GTAAGTACATCATCACTCAAAAATGAATGAGATGAAACCAG GAATTAGCATCACAGTTCTACCACTTTCGGAATCACAATAAT TTTCTATTTCACGTAAATATTATAATTTTCAAAGAAATTATTA ACAGTCTTCAATAGGGAGTTATTTTTCGCTCATTCTGTACATA TATATATATATATATACACTAAGATTACGAAATTCTGTTCCA

							AGGAAAACACCTGAATTGTGGCCTGACAAGTGAATAATCCA<br>TCACGACACTCATTGTGAGAGTTTCTGGCCAAAATGTCGATT<br>ACCGAATTGGATTATCCTGCACAATCCTCGGCGTAGCCCCTG<br>TGATTTCTGACTGCGGCAATAAATTTTTCTGTCCAACACATAG<br>GATTAAGTGACACAAAATTACACTAGAGAAATAGTTCAAGG<br>GTTGTTTTCAGAAATGGGAAGACGGTTTAAGCCAGTGTGTAA<br>ATTCATAAAAGAACCTTCGAAGACGTCGGTAGGCACTAATG<br>CACAGTTCAAAGCATATGGTTATTGTACAGTTACAATCCGGG<br>AATTACTGTCACACCTTGTAAATAAAGTTATAATGCAAATAA<br>TTTTGCATTGTAACTGCGTTAAATTTTATTACTTTTTATTTCAA<br>CTTAAAAGTTTTTGCGCTTTCCTTTGAGAAATTGAATCTTATTT<br>TAGTTAACAGTATAACAATAAAGATCTTAATACTTAATTTTAT<br>ATCTAGCTGTTATCAGCGCACACAGTACAAATTTTTCATTAA<br>ATGGAATACATGTATGGCTCTCTACTTCAATTTTTTTTCAAAG<br>AAAAAGCACTTTATTTGTTCCCAAT

PhdEnzSeP 81	B5U2W0	7,92E-11	26,103	PF12032<br>PF00089	70576,79	LTRCLSTMIRLTFYA<br>GILLLLTCFASAKHK<br>ICSPYPYVSFEHGSIQ<br>RLSFQKYGFRCDPGY<br>FLASPFTIRCYKGNW<br>TSLKEPTCLKKHGLC<br>DDPPVVDNSLTYGE<br>NRELGAKIKYICKEG<br>FTLLGHSELVCTSTG<br>HWNKRAPTCMDQS<br>EPLENVAERLKDSFV<br>TQLETYSADTPQGRL<br>LDKSSLLLGLELFLL<br>VDRSSSVDPVHLED<br>AKNFIKFLVRRFYVR<br>NGPDNNNGTRVAVI<br>AFGTDVQVIFDVDN<br>KNISNPTLAASAIDTI<br>QPNGGGTNMEGAL<br>THVLLDVINLRPKA<br>KRALFLMTDGEPNI<br>ANPEITPQDLAQELK<br>NFHDFEIFTIGIGKGI<br>NTQLLNELASEPPLS<br>HVFILQDYPDLNEV<br>MKIIQDTKPPPAPISK<br>DQCGYNATGEDRP<br>WLATLYIGNSPFKVC	AGGAAGTCTGCCAATCGTGCCAGTTGCGAAATTCCGGTCCCC<br>GAAGAAGACAGTAACTAACACGTTGTCTATCCACTATGATCA<br>GGCTCACTTTCTACGCGGGAATTCTTCTTCTGTTAACTTGTTTT<br>GCATCTGCCAAGCATAAGATTTGTTCGCCGTATCCGTATGTAT<br>CTTTCGAACATGGCTCCATCCAGAGGCTGTCTTTTCAGAAGT<br>ACGGATTCAGGTGCGATCCTGGGTATTTTCTTGCTTCTCCGTT<br>CACAATCCGCTGCTACAAAGGGAACTGGACCTCGCTAAAGG<br>AGCCGACCTGCCTCAAGAAGCATGGATTATGCGATGATCCTC<br>CAGTCGTTGACAATTCATTGACATACGGAGAAAACAGGGAA<br>TTGGGTGCGAAGATCAAATACATTTGTAAGGAGGGATTCACA<br>CTCCTGGGACATTCGGAACTGGTCTGTACTTCGACTGGTCACT<br>GGAACAAACGAGCACCCACCTGCATGGACCAGTCTGAACCT<br>CTGGAAAATGTTGCCGAGAGACTGAAAGACAGTTTCGTGAC<br>TCAGCTGGAAACATACTCTGCTGACACGCCCCAGGGACGAC<br>TCTTGGACAAGTCCTCTCTTCTGCTGGGATTGGAACTGTTCCT<br>CTTGGTAGACAGGTCCAGCAGCGTAGACCCCGTCCACCTTGA<br>AGACGCCAAGAATTTCATCAAGTTTTTGGTGCGGAGGTTCTA<br>TGTGAGGAACGGACCTGATAATAATAACGGTACACGAGTAG<br>CGGTGATTGCTTTCGGGACAGACGTGCAGGTAATATTTGATG<br>TGGACAACAAGAACATATCCAATCCCACTCTGGCTGCCAGTG<br>CCATAGACACCATACAGCCTAATGGAGGTGGTACAAATATG<br>GAAGGTGCACTGACTCACGTATTGCTGGATGTCATCAATTTG<br>CGGCCGAAAGCCAAAAGAGCCCTGTTCCTTATGACCGATGG<br>AGAACCGAATATCGCCAATCCTGAGATAACGCCCCAAGATT<br>TAGCTCAGGAATTGAAGAACTTTCACGATTTCGAGATCTTTA<br>CTATTGGAATAGGCAAAGGTATTAACACCCAACTTCTGAACG<br>AACTGGCGAGTGAACCACCGCTGAGTCACGTGTTCATTCTTC<br>AGGATTATCCAGACTTGAATGAAGTCATGAAAATAATCCAA

						GGVLICDQWVLTAG<br>SCLEEEEKPLNKQDI<br>FVVLGERHLLKDER<br>GQRNFYVVDIRFHP<br>LFDTNHIIRNNLALV<br>KLNLPASKYRPACLP<br>PTDQAIPLHLDLKIN<br>ASIAGWGTNKASKF<br>TMAQAGDLNFEMSS<br>TSVSLSEEQECPINRQ<br>GLTRLCAGRGSRAC<br>YGLTGSPLMAVNPA<br>THFQHVLGILADRT<br>HCAQGQNQYVELT<br>KYIVWINHATAGCH<br>LKHWGFTE*	GACACTAAACCTCCTCCTGCGCCAATAAGCAAGGACCAGTG<br>TGGTTACAATGCAACCGGCGAAGATCGACCTTGGCTTGCAAC<br>CCTCTATATAGGGAACAGCCCCTTCAAGGTATGCGGAGGCGT<br>GTTGATTTGTGACCAATGGGTGCTGACGGCTGGGTCTTGCCT<br>GGAGGAAGAAGAGAAGCCTCTTAATAAACAGGACATATTCG<br>TCGTACTCGGTGAACGACACCTGTTGAAAGACGAAAGAGGC<br>CAGAGGAATTTCTACGTTGTCGATATTCGGTTCCACCCACTCT<br>TTGATACAAATCACATCATTCGGAACAATCTGGCCCTCGTCA<br>AGCTAAATCTACCAGCATCCAAATACAGACCCGCCTGCCTCC<br>CTCCGACAGACCAAGCGATCCCTTTGCATCTGGACCTGAAGA<br>TCAACGCTTCTATTGCCGGTTGGGGCACAAATAAGGCATCTA<br>AATTTACTATGGCCCAGGCAGGAGACCTCAACTTCGAGATG<br>AGCTCAACGTCCGTCTCGCTGTCCGAAGAGCAAGAGTGTCCA<br>ATAAACAGACAGGGACTTACGAGGCTCTGCGCTGGACGAGG<br>GTCGAGGGCATGCTATGGCTTAACAGGATCGCCCTTAATGGC<br>AGTGAATCCGGCAACACACTTCCAGCATGTTCTTGGAATTTT<br>AGCTGACCGCACGCACTGCGCACAAGGTCAGAATCAGTACG<br>TCGAGCTCACGAAGTACATAGTCTGGATAAACCACGCCACT<br>GCGGGCTGCCATCTGAAGCACTGGGGGTTTACCGAGTGAGG<br>CTGTTTCACAGCATTCTTTTCACACGTCTGAATGTGGATCTCA<br>GATTGCAATGCGATAGGCGCCTATAGAGCAATCCAGTAGTAT<br>CAAATTGTGACACCATGTCAAATTGTGATCAATAACTCTACA<br>ACTCCATCCAATCGGCAGCCAAGGACTATCAGAAGTTAAAA<br>GAACACTCCGTCTGATCAGGAAACAAATAAGCAGAGGAACA<br>CACCAAATTTGCAAAACTTGTATTTCCTTTTCTCGGGGATTAA<br>ATGAAATCCTTTCACAATCAATAACGGTCAAATCAAACTGAC<br>ATTTGACCGAACATTTCATTCTGGAATCTTATGAAGTGACA

PhdEnzSeP 82	B5U2W0	7,29E-13	57,447	PF12032<br>PF00089	7800,68	SNSECKKSYRRVSGA QLPAGLTDDFICAG DQDGGRDACQRDS GGPLMYKDNHMNL PLDDPDIPWVLVGV VSF	CCAAATGACACAACACCTACCAGTACCCAAGGAATATCAGG ATCATCGAGTGGCAAGTTCATGTGGTTGTCCTTATACATCAG AGGTCCTCCAGAATCTCTCTGGCAAGCATCTCTTCCGCCATCT TGGTCACCGGCACAGATGAAATCATCCGTCAGTCCGGCTGGC AACTGAGCACCTGAAACTCTCCTGTAGCTCTTTTTGCATTCGC TGTTGGAG
PhdEnzSeP 83	B5U2W0	2,13E-31	35,341	PF12032<br>PF00089	37245,6	GIPKVCCGLPLVLGP TLSKKVEEEKARNR RCGTVTWQDDISET ERRFVEIHQSISPVDT SDPSKFNPPLERRLT EFHQAVPPVDTSDP PKFDLPLDLSYRFIVV LGKEAGQGDFPWM VSIRRLGNHWCGGS LIDRKHILSAAHCFV INGRKPDKTDFMVH VGNIHVDEGYPFSV AKIVIHPQYKPEQYY FDLAMLTLSEEILIPQ FAHICLPSPRLTAIDL TDKNTSVIGWGETSF GGLPPLVLNIAEDIP VVSNDECKKSYRRF AVDQLPLGVTDDFI CAGDRDGGRDSCR MDSGGPLMYKDNH	GGGAAACCAGGTTCACCGCACAGGAATCCAAATGACACAAC ACCTACCAGTACCCAAGGAATATCAGGATCATCGAGTGGCA AGTTCATGTGGTTGTCCTTATACATCAGAGGTCCTCCAGAATC CATCCGGCAAGAATCTCTTCCACCATCTCTGTCACCGGCGCA GATGAAATCATCCGTCACTCCCAATGGCAACTGATCAACCGC AAATCTTCTGTAGCTTTTTTTGCATTCATCGTTGGAGACTACT GGTATGTCCTCCGCTATGTTCAATACAAGTGGAGGTAGTCCG CCGAAAGAGGTTTCTCCCCATCCAATCACCGACGTATTCTTG TCTGTGAGATCTATAGCTGTTAGTCGTGGTGAAGGCAAACAA ATGTGTGCGAACTGCGGAATCAAAATTTCTTCGCTTAAAGTC AGCATGGCCAAGTCGAAGTAGTATTGTTCAGGTTTATACTGC GGATGAATAACAATTTTTGCCACTGAGAAGGGGTAACCTTCG TCAACATGTATATTACCCACGTGTACCATAAAATCTGTTTTAT CAGGCTTCCGACCATTAATAACGAAACAGTGTGCTGCTGACA GTATATGCTTTCTATCTATTAAACTGCCTCCGCACCAGTGATT GCCTAGTCTTCTAATGGACACCATCCATGGAAAATCTCCTTG TCCAGCTTCTTTGCCTAGAACAACTATAAACCTATAGCTGAG ATCTAGTGGAAGATCGAATTTAGGTGGATCGCTTGTGTCCAC TGGTGGAACGGCCTGATGAAATTCAGTGAGTCTTCTTTCTAAT GGTGGATTGAATTTAGACGGATCGCTTGTGTCAACTGGAGAA ATGGACTGATGAATTTCAACGAATCTTCTTTCTGTCTCAGAAA TATCGTCTTGCCATGTAACGGTTCCACATCTTCTATTTCTAGCT

						MNLPLDDPDIPWVL VGVVSFGFLCGEPGF	TTTTCCTCTTCAACCTTCTTTGAAAGCGTAGGCCCGAGTACTA AAGGTAAGCCGCAGCATACCTTTGGAATACCT
PhdEnzSeP 84	B5U2W0	4,11E-32	35,341	PF12032<br>PF00089	34301,17	GIPKVCCGLPLVLGP TLSKKVEEEKARNR RCGTVTWQDDISET ERRFVEIHQSISPVDT SDPSKFDPPLDLDTE FGITVVGGTVAEQG TFPWMVSIRRLGNH WCGGSLIDRKHILSA AHCFVINGRKPDKT DFMVHVGNIHVDE GYPFSVAKIVIHPQY KPEQYYFDLAMLTLS EEILIPQFAHICLPSP RLTAIDLTDKNTSVI GWGETSFGGLPPLV	GGGAAACCAGGTTCACCGCACAGGAATCCAAATGACACAAC ACCTACCAGTACCCAAGGAATATCAGGATCATCGAGTGGCA AGTTCATGTGGTTGTCCTTATACATCAGAGGTCCTCCAGAATC CATCCGGCAAGAATCTCTTCCACCATCTCTGTCACCGGCGCA GATGAAATCATCCGTCACTCCCAATGGCAACTGATCAACCGC AAATCTTCTGTAGCTTTTTTTGCATTCATCGTTGGAGACTACT GGTATGTCCTCCGCTATGTTCAATACAAGTGGAGGTAGTCCG CCGAAAGAGGTTTCTCCCCATCCAATCACCGACGTATTCTTG TCTGTGAGATCTATAGCTGTTAGTCGTGGTGAAGGCAAACAA ATGTGTGCGAACTGCGGAATCAAAATTTCTTCGCTTAAAGTC AGCATGGCCAAGTCGAAGTAGTATTGTTCAGGTTTATACTGC GGATGAATAACAATTTTTGCCACTGAGAAGGGGTAACCTTCG TCAACATGTATATTACCCACGTGTACCATAAAATCTGTTTTAT CAGGCTTCCGACCATTAATAACGAAACAGTGTGCTGCTGACA GTATATGCTTTCTATCTATTAAACTGCCTCCGCACCAGTGATT

						LNIAEDIPVVSNDEC KKSYRRFAVDQLPL GVTDDFICAGDRDG GRDSCRMDSGGPLM YKDNHMNLPLDDP DIPWVLVGVVSFGFL CGEPGF	GCCTAGTCTTCTAATGGACACCATCCATGGAAAAGTTCCTTG TTCAGCTACTGTGCCTCCAACAACTGTTATTCCAAATTCGGTG TCGAGATCTAATGGTGGATCGAATTTAGACGGATCGCTTGTG TCAACTGGAGAAATGGACTGATGAATTTCAACGAATCTTCTT TCTGTCTCAGAAATATCGTCTTGCCATGTAACGGTTCCACATC TTCTATTTCTAGCTTTTTCCTCTTCAACCTTCTTTGAAAGCGTA GGCCCGAGTACTAAAGGTAAGCCGCAGCATACCTTTGGAAT ACCT
PhdEnzSeP 85	B5U2W0	1,07E-24	36,719	PF12032<br><br>PF00089	22640,68	RFHYKSTKKPFSVTT EATTLKQSTTTESQL QEFTTLKPVVATVIG TAESSLQNTTFSAKF RCGTSGRDGRIVGG YEANPGQWPWMA AIFLDSPRGREYWCG GAVVSEWYILTAAH CLSDQRGRKYRKEQ LTVRLGDHHLFRTD DFMDPIEFKVAEAV PHPEFSRNGFYNDIA LLKLRHSISYTEFISPV CLPTPGLKANPL	AAAGATTTCATTATAAGTCTACAAAGAAACCATTTTCTGTTA CTACAGAAGCTACTACTCTGAAGCAAAGTACTACCACAGAA AGCCAACTACAGGAGTTTACAACTTTGAAACCAGTAGTTGCG ACTGTCATTGGGACAGCAGAATCTAGTCTTCAAAATACAACA TTTTCAGCAAAATTCAGATGTGGAACATCTGGAAGGGATGGT AGAATTGTTGGAGGATATGAAGCGAATCCTGGACAGTGGCC TTGGATGGCTGCAATTTTCTTAGATAGCCCCAGAGGAAGAGA ATATTGGTGCGGCGGGGCCGTAGTGAGCGAATGGTATATTTT AACAGCTGCCCATTGCCTCTCGGACCAAAGAGGACGAAAGT ACCGAAAGGAACAATTGACGGTGAGACTCGGAGATCACCAC CTCTTTCGGACAGATGATTTCATGGATCCAATCGAGTTTAAA GTGGCAGAAGCTGTTCCTCATCCGGAATTTTCTAGAAATGGA TTTTATAATGACATTGCTCTCCTAAAGCTGAGACACTCAATTT CCTACACCGAGTTCATAAGTCCTGTTTGTTTACCCACACCAG GACTAAAAGCGAATCCTCTTG

PhdEnzSeP 86	B5U2W0	9,81E-09	47,273	PF12032<br>PF00089	14708,96	ENGVVRPNVFILQD<br>YKTLAWLVQEITNG<br>TADYSQCGIGVKSIG<br>VNKGRIVGGKKAKE<br>PWPWMAALEMTIG<br>NDDPSRRTLKCGGSI<br>IHHNFVLTAAHCMY<br>NKMARRDKNEIRVK<br>LGLTSLKNDTYLQEF<br>EVQ	TCTGGACTTCAAATTCTTGAAGATACGTGTCATTCTTTAAAGA<br>AGTCAGGCCTAATTTGACTCTAATTTCGTTTTTATCCCTCCGA<br>GCCATTTTGTTGTACATGCAATGGGCAGCAGTCAGGACGAAG<br>TTATGATGAATGATGGAGCCTCCGCATTTCAGCGTTCGTCGG<br>GATGGGTCGTCATTTCCTATCGTCATTTCCAAAGCGGCCATCC<br>ATGGCCACGGTTCCTTGGCCTTCTTGCCTCCCACTATTCTGCC<br>TTTATTGACGCCTATAGATTTTACGCCAATACCGCACTGAGA<br>ATAATCTGCCGTCCCATTTGTGATCTCCTGCACGAGCCAGGC<br>GAGCGTCTTGTAGTCCTGGAGGATGAAGACGTTTGGACGCAC<br>TACCCCGTTCTCG
PhdEnzSeP 87	B5U2W0	2,37E-14	27,899	PF12032<br>PF00089	33423.97<br>**30459.38**	MELRNHLVFTITAVI<br>MYIASTKMIDA**SAL**<br>**NGSSELERIFRGKK**<br>**AAPGQFCFVCALYK**<br>**DRDFTCTSFIIGPTA**<br>**ILTAAHCVNDVGV**<br>**VPERYHLICNVNRN**<br>**SPEVRVNIASVTPH**<br>**PNYTENGFLHDLAV**<br>**LKATEPIRFSIEVCA**<br>**ACIASNKTELMQYE**<br>**YGTAIGYGDTTGT**<br>**GLLSSRDQRYADQ**<br>**RIRENDDCRPYYLG**<br>**MRPDDCMICAGG**<br>**DFTGICPGDSGGPL**<br>**AVPTEERRLSIESDG**<br>**SRHSDKDLRVEIFA**	GTTTAATAGACATAAGTTAATTATATTTTCCGAAGCAAGTCG<br>GGATCACAAATGCTTATACTACGCAAATGTCTTCCTCAGCGA<br>CCAACCGGATGAAGTCCTCGTAACAGCTCACCCGAGTCCAC<br>ACAGCCGGTACTACCGTGCCGCATGGAGCGTACCTGACTCCA<br>TAGGAGACGATACCTATCGCGATGTTACTCTGTTGCCTAGTTT<br>GATCGGCGAAAATTTCCACCCGTAAGTCCTTGTCGCTGTGTC<br>GGCTCCCGTCTGATTCAATTGATAATCGACGCTCTTCCGTAGG<br>CACTGCTAAAGGTCCTCCGGAATCACCAGGGCAAATTCCGGT<br>GAAATCTCCTCCGGCACAGATCATACAGTCATCTGGCCGCAT<br>TCCAAGATAGTATGGTCTGCAATCGTCGTTTTCCCTGATTCGC<br>TGATCTGCGTATCTCTGGTCTCTGGAAGACAACAATCCCGTT<br>CCCGTGGTGTCGCCATATCCGATTGCCGTACCATACTCGTATT<br>GCATCAGTTCCGTCTTGTTGCTAGCGATGCAAGCGGCACATA<br>CTTCGATACTGAAGCGGATGGGCTCGGTAGCCTTGAGGACGG<br>CTAAATCATGGAGGAAGCCGTTTTCAGTGTAGTTCGGGTGGG<br>GTGTCACGGAAGCAATATTGACTCGAACCTCAGGAGAGTTTC<br>TGTTGACGTTACAGATGAGGTGATACCTTTCCGGAACTACAC<br>CGACATCATTTACACAATGAGCTGCCGTCAGGATTGCAGTTG

						**DQTRQQSNIAIGIVSYGVRYAPCGTVVPAVWTRVSCYEDFIRLVAEEDICVV***	GCCCTATGATGAAGCTGGTGCAGGTGAAATCTCTGTCTTTGTACAGAGCGCACACAAAACAAAACTGTCCTGGTGCTGCTTTTTTTCCTCGAAAAATTCTTTCTAATTCACTACTTCCATTTAGTGCTGAAGCATCAATCATCTTCGTCGAAGCAATGTACATGATTACTGCAGTTATAGTAAAGACTAGGTGATTTCGGAGTTCCATCGTGGAGGCTGATGAACAG

PhdEnzSeP 88	B2D0J4	3,82E-157	37,112	PF00930<br>PF00326	93459.11	MSSVAGSTASPEKRL<br>PVEPPVGTKCSEMD<br>ALLLGRTRELVAAG<br>PDQKNWRGIGIALL<br>VIVLVCALIVAAIVL<br>LTPGDKGFNSRKSRI<br>TLEEVVKGQFSYRRF<br>NGSWISDYEFVYRDP<br>YGAIGLYDARNNSE<br>QVLMPNTTLRQHSIS<br>GYSVSPDRQYVLLA<br>HDMERIFRYTYKSQF<br>LLYNISSDHLTGLFP<br>KSPSMVLQFASWGP<br>SGNQLVYVHDNNV<br>FYLSDVSAKPRQLTS<br>SGTDGIIFNGVPDWV<br>YEEEILSTSNALWWS<br>PDGTKLCFATFNDT<br>DVDVLQYPYYGSYT<br>KITNVYPELVNLRYP<br>KAGKTNPTITIWIAD<br>LTSNKDLIVVEAPKE<br>YKDVDYYFTDVQW<br>VENSAVSVVWLKRS<br>QNSSIISLCRENDAW<br>ACKKNLQVDTNGH<br>GWIDLSKAIFSADSK	TTATGCATATTAAATTTCAATTCAATTAATTCTTAAATTCAAT<br>TAATACATTAACATTTTTGTTCAATAAATTATAATTAAAATCC<br>AATTTTAAAAATTTTCCTTCAAAAATTTTGCATCATGGAGTGG<br>TGCATTCGGAATCCTGATTCCAAAGGGTGGGGGTGGGGGTGG<br>TTCGATCCCCTTTTTCCAACGCCAATGGTTGTAAGCTCATCCA<br>TCAAACCGGAAGTAGCATTTCAAAACCGGAAGTCGTCCGAA<br>AGTTCAATTTTTCACTTATTTTTCGCAATTTTTTTATTATTACA<br>ATTTGAAATAATATTTTTACAATGTCATTAACGCATAACCAG<br>AACTTTAATGCAGTAATTTTCCTTATTAAAAAGAATGTGTGA<br>CTAATAAGTCTAGTGGATTTACACAATATTGTTGAGAGGAAG<br>AATCAGTCTACACGAAGAAAATTTTAATTGCATTTTTCTTAAA<br>ACTTATTTTTTATGTAAATGCTTTACCGTTAACATGATATCTC<br>AAGAATCAATACATAATATTTTTAGGGTATGCTTTATATGCTA<br>TTGACATGAAAATGACTCAAAATCTTTGATGTAGATTAAATA<br>TTTTAAAATTTATAGCTATTTATATGCGGAAAAGGTAACAAA<br>ATTTGGAAAAATATCCATATTATTATGTATTTGTGCCTGTAAT<br>TTCCTGGCAGTTTGAAATGTCTCTTAGATTTATTTTAGTTCATT<br>TTAAAGACCAGTGATACATATACATTGTGTCTAAAGGACATT<br>AAGATTATGCAATTACACTTTAGTGTAGAGTATTAGCTGTTTT<br>GATAAAAATTGACTAAAATTGACTAAAAATAACCCAATTTAT<br>AAAAATGTACGACATGCATAATTAAATTTTCACTCTTTTATTC<br>CAAAACTTACTTAAAATCAGTAAAATAAAAAAATATATATA<br>TATTATTTAAAAAAAAAAAAAATTGTTCCGAATGTAGTTTGGT<br>ACCTTAAATATCAATGCATATTTAGCCCACAATTGAGAATCG<br>TCGCCTTCATAAAAGGAGAAGAGGTGTTGATATTGTAGATAG<br>CATGAATATGATTTGTGCTCCACCCATCAGGTACCAATCTAC<br>GTGTGTTCATTTTTGTATCCAGCATATAAATCAACCCCTAAGT<br>TTTTGGCTCATTTTAGTGGTTCAAAAAGTTAACTTATAGACCA

						TYFLRLPGPPEGSLG<br>RFRHIAAVDIKTGSK<br>TFLTSGKYDTVTLLA<br>HNLEKKTLYYITTLE<br>GKPGERHIFGVTDTT<br>HKDPKNSTCLTCDI<br>GPDCLYNDAIFSPDF<br>TYYVLECLGPGIPRIE<br>LRSTESNNIVSILDIN<br>HDLKDFVDKKAMP<br>KIKNLQVPIDGNYH<br>ANVRLYLPPSLQEYE<br>ITKYPMLVEVYGGP<br>GSQMVSEKFNVNW<br>GSYLASKKNIIYTRID<br>GRGSGFQGDKFLHE<br>LYLKLGSVEVVDQIA<br>VTSYLKEKLPYVDSK<br>HVAIFGWSYGGYVS<br>ALALSSEDKVFTCGI<br>SVAPVTSWLYYDSV<br>YTERYMRMPTKDD<br>NLINYEKSDVMKKA<br>LSFKGKKYLLIHGTA<br>DDNVHWQQSMML<br>AKALTDAGVLFRM<br>QVYPDENHGLGHV<br>KLHLYQTMDDFLD	GAAAATACAGTAGATTATGCTTTTATACCCTTTCATTTATGAC<br>TGCAGTACTTTCTACACATGTGTACACACACACACACATAAA<br>ACAACTTCTTTAGAGAAATATTACAGAAATTAAATATTTAGC<br>GTTCTTTAATTTATACTTTTTTAATGTCCAACATTTGATGCTAG<br>CTACTATGCCTGAAGTACATAATTTTAATAATGACAAAATCA<br>TTAAGATAGTTGATTTTACTTTGACATAATTTTCATATGAATG<br>ATAAAGCACCAGTCTTTTCCCGTTTACACCATCTACTTATATA<br>TATATATACTTGTATTAAATATATATACATACATTATATGCAT<br>GTACATTTAAATCATTCCCTAGTTAGTAATATGATCAAATTAA<br>TATGATGTTCAATAAAATTTAGTGCTTAAAAAATAAATTAAA<br>TGTACATACATATTTCATTGAATGAAAAGGATAGTATTTTTCA<br>CAAACGTATCCAAACGTTATGCAAAAATTTTTGTATAGCAAA<br>TATTTTGGTAGAAAGCAAAGGTCACATTAGACAAATTGTACT<br>TTTCTTTGCCTCTTGTGCACTGTATTCAGAAAACCGCCTCAAT<br>TTCACATCTTAGAAAATATGATTTCCACGTCTCAGCACAAAT<br>GAAGAGTCTGAGATATACAAGCAGATGTATACTTATGAATG<br>ATGAACACAGTGATAGTTCTTTTTTCACGAAAGTATTAATAG<br>ATGTTGAGTTAAACCTATCTTTCAGAGTCTGGCGATGTGGAA<br>TGATAAGTGACATCATCCTTATAATTATCGGAAAAGCAATGA<br>TCTAAGAAATCATCCATTGTTTGATAAAGATGAAGTTTTACG<br>TGACCAAGTCCATGATTTTCATCAGGATACACCTGCATCCTG<br>AACAATACTCCAGCATCTGTTAAAGCTTTGGCTAACATCATT<br>GACTGCTGCCAGTGAACATTATCATCAGCTGTTCCATGTATA<br>AGCAAATACTTCTTTCCCTTAAAGCTCAGGGCTTTTTTCATCA<br>CGTCTGATTTTTCATAGTTTATAAGATTATCATCTTTTGTAGGC<br>ATTCGCATATATCTTTCAGTATAAACAGAATCGTAATAAAGC<br>CAACTAGTGACAGGAGCAACAGAAATTCCACAAGTGAAGAC<br>TTTATCTTCACTGGAAAGTGCCAATGCTGACACATAGCCACC

						HCFSDNYKDDVTYH STSPDSER*	ATAAGACCAGCCAAATATTGCAACATGCTTGCTATCCACGTA CGGTAACTTTTCTTTCAGATAACTTGTAACAGCAATTTGATCT ACAACTTCAACAGAGCCCAACTTAAGATAAAGTTCGTGCAA AAATTTGTCTCCTTGAAAACCACTTCCTCTACCATCAATGCG AGTATATATAATATTTTTTTTACTCGCAAGATAAGAGCCCCA GTTAACATTGAATTTTTCAGAAACCATTTGACTACCAGGTCC ACCATATACTTCAACAAGCATAGGATATTTGGTTATTTCATAT TCTTGCAAAGAAGGAGGTAAATAAAGACGGACATTGGCATG ATAATTTCCATCAATAGGCACCTGAAGGTTCTTGATTTTTGGC ATTGCTTTTTTGTCCACAAAATCTTTTAAGTCATGGTTAATAT CCAAAATAGATACTATATTGTTGGATTCAGTAGAACGAAGCT CTATACGAGGTATTCCTGGCCCCAAGCATTCTAAAACATAAT ACGTAAAATCTGGACTAAAGATTGCATCATTATACAGACAAT CAGGTCCAATATCACAAGTTAAACATGTTGAGTTCTTGGGAT CTTTGTGTGTTGTGTCTGTTACACCAAATATATGCCGTTCACC GGGTTTTCCTTCCAAAGTTGTGATGTAATACAAAGTCTTTTTT TCCAAATTATGAGCCAGGAGTGTAACAGTATCATATTTTCCA CTTGTTAGGAACGTCTTGGAGCCAGTCTTTATATCTACAGCTG CAATATGTCTGAAGCGACCTAGACTACCTTCAGGTGGCCCTG GGAGCCTCAAAAAGTAAGTCTTACTGTCAGCTGAAAATATTG CTTTTGACAGATCAATCCAACCATGACCATTAGTATCTACCT GAAGATTTTTTTACATGCCCATGCATCATTTTCTCTGCACAA ACTGATTATTGAAGAATTCTGAGATCTTTTTAGCCAAACAAC TGATACAGCACTGTTTTCAACCCACTGAACATCAGTGAAATA GTAATCAACATCTTTATATTCTTTAGGAGCTTCGACAACAATT AAATCTTTATTTGAAGTCAAATCTGCAATCCAAATAGTGATT GTTGGATTTGTTTTTCCTGCCTTAGGGTACCTTAGATTCACTA ATTCTGGATATACATTAGTAATTTTTGTGTATGAACCATAGTA

							AGGATACTGCAAAACATCAACATCAGTATCATTAAATGTTGC AAAACATAATTTTGTTCCATCTGGAGACCACCACAGAGCATT GCTAGTTGATAAAATTTCCTCTTCATATACCCAATCTGGAAC ACCATTAAAAATTATTCCATCAGTTCCACTAGATGTTAATTGT CGAGGTTTAGCAGACACATCAGACAAATAAAACACATTGTT ATCATGAACATAGACTAACTGGTTACCACTTGGACCCCAGGA AGCAAATTGTAAAACCATTGAAGGAGATTTAGGAAACAATC CTGTTAAGTGATCACTGCTGATGTTGTATAGCAAAAACTGAG ATTTATAGGTGTACCTAAAAATTCTTTCCATATCATGAGCAA GTAGAACATACTGACGATCCGGTGAAACAGAGTAACCAGAT ATTGAATGTTGTCTAAGTGTTGTATTTGGCATAAGCACTTGTT CAGAATTATTTCTGGCATCGTATAATCCAATAGCACCATATG GATCTCGGTAAACAAATTCATAATCTGAAATCCAAGAACCAT TGAATCTTCTATAACTAAATTGACCTTTAACTACTTCCTCTAA TGTTATTCTACTTTTCCTGGAGTTAAAACCTTTATCACCTGGA GTAAGCAGCACAATGGCAGCAACTATGAGAGCACAAACAA GCACAATTACTAGTAGAGCTATACCTATACCACGCCAATTTT TCTGATCAGGTCCAGCAGCAACAAGCTCCCTGGTTCTGCCCA GAAGAAGCGCGTCCATCTCACTACACTTTGTACCGACCGGGG GTTCCACGGGAAGCCGCTTTTCGGGGCTTGCAGTCGATCCCG CAACCGAACTCATTCTTTCTTCATAACCACCGCCTCGTTAACC GTTTTGCTGAGCATTCCACCGTCTCATGTTGTAGCGTATATTC GAGCCAAA

PhdEnzSeP 89	Q75WF2	6,87E-27	32,240	PF03265	20337.98	VFSYPDNALLNGQSILCVTFRTAELNEICHQLLYSHPNIYVHGITNEINDMLDDTAKTLFTDNPSFIRRDPLTRTASLKSLGNKVFVSFAKDNQYKDDLYSGIIAPGLKSSLLVETWRRGSGTVLPPACQSDYPVIDIVSINMTITNRRNSKVISFRSTEDHSKWAVSDNSGNSTRNWMCI	AAGTGTTTAGCTACCCTGACAATGCTTTACTAAATGGACAAAGTATCTTGTGTGTAACATTTAGAACTGCTGAATTAAATGAAATCTGCCATCAGTTACTATACAGTCACCCCAACATTTATGTGCATGGTATTACTAATGAAATAAATGACATGCTAGATGACACAGCAAAAACTTTATTTACTGACAATCCTTCTTTTATACGAAGAGACCCACTGACAAGAACAGCATCTTTAAAAAGCTTGGGTAATAAAGTTTTTGTGAGTTTTGCAAAGGATAATCAGTACAAAGATGATTTGTACAGTGGGATTATTGCACCTGGTCTAAAATCTTCATTGCTAGTAGAAACATGGCGAAGAGGAAGTGGTACTGTTCTTCCACCAGCATGTCAATCTGATTATCCTGTTATTGACATTGTTTCGATTAACATGACAATAACAAACAGAAGAAATAGCAAAGTAATCTCTTTCAGATCAACTGAAGACCACAGTAAATGGGCAGTATCTGACAACAGTGGAAACAGTACAAGAAACTGGATGTGCATT
Cholinesterases							
**Transcript ID**	**Uniprot ID reference protein**	**e-value**	**Identity (%)**	**PFAM domain**	**MW (Da)**	**Amino acid sequence**	**Nucleotide sequence**
PhdEnzCho 01	W4VSJ0	3,09E-27	51,163	PF00135	9560,92	IVVSINYRVASLGFLFFDRPDVPGNAGMFDQLMALEWIRDNIVYFGGNPHNVTLFGESAGAVSVGLHLLSPLSRHLFNQAVLQSGSPT	GCAGTAGGTGAACCGCTCTGTAGAACAGCCTGGTTAAATAAATGTCGGCTAAGTGGGGATAGTAAATGTAGGCCAACGCTCACTGCTCCCGCGCTTTCTCCAAAGAGAGTAACGTTGTGTGGATTACCACCGAAATAGACAATATTGTCTCTAATCCATTCGAGGGCCATCAGCTGATCGAACATGCCTGCATTGCCTGGGACATCGGGACGATCAAAGAAGAGGAAACCAAGAGATGCCACCCTATAGTTAATGGATACTACGAT

PhdEnzCho 02	W4VSJ0	8,01E-12	37,037	PF00135	13715,39	MDILGVYGEKQETF EFNDTFAKQFIQHFA GRKIAEGEENNKIIR SYLDRVKKESKYTYL QTTVDFVGDFMVTC GALFYADYHSLRDN PVYFYLFDYRPDSTP LAEWMGVAHFDEM Q	GATGGATATACTGGGTGTATACGGCGAAAAGCAAGAAACTT TTGAATTCAACGACACTTTCGCTAAACAGTTTATACAGCATTT TGCGGGGAGGAAGATAGCAGAAGGAGAAGAAAACAACAAA ATTATACGAAGTTACTTGGATCGTGTAAAAAAGGAATCAAA ATACACATACTTACAGACAACAGTAGATTTTGTAGGAGATTT CATGGTTACTTGCGGTGCATTATTTTATGCTGATTATCATTCTT TGAGAGACAATCCAGTGTATTTCTACTTATTCGACTATCGCCC AGATTCAACGCCACTGGCCGAGTGGATGGGAGTTGCTCATTT TGATGAAATGCAGT
PhdEnzCho 03	W4VSJ0	1,08E-07	28,448	PF00135	12706,56	QSGTAFHPMFNNN NGLLMMSQLVANH LGCADYSDSLQKNP KSVVDCMKALPSEK FAEADGVIMKNGG MLFPRTGDAFLPEN TIDSFKRGDFKDAEL LLGITRDEGSILLAFQ MMDI	AGTATATCCATCATTTGAAAAGCTAAGAGAATAGAACCTTCA TCTCGAGTTATTCCAAGGAGAAGTTCAGCGTCCTTAAAATCA CCCCTCTTAAAGGAATCAATGGTGTTTTCTGGGAGAAATGCG TCTCCAGTACGTGGGAATAGCATACCGCCGTTTTTCATGATA ACTCCGTCCGCTTCGGCAAATTTTTCAGAGGGAAGTGCTTTC ATGCAATCAACAACACTCTTCGGATTTTTCTGTAGAGAATCA CTATAATCTGCACATCCCAAATGGTTAGCAACTAACTGACTC ATCATCAGAAGTCCATTGTTATTATTAAACATGGGGTGAAAA GCAGTTCCACTTTG

PhdEnzCho 04	W4VSJ0	2,65E-105	35,185	PF00135	61497.38<br>**59798.16**	MKLLFLLLVFPLVN<br>G**DKTVTTSNGPVR**<br>**GISLAHKKVEAFLG**<br>**IPYAEPPVGSLRFAK**<br>**PVPKTTWENVYDA**<br>**NMLPPSCVQLFLGN**<br>**FYFTSDLTGDKMSE**<br>**DCLYLNLWVPEGG**<br>**SDLKPILLYIHGGAF**<br>**MIGSSNIKVYDGAT**<br>**LSEHGDVIVASINY**<br>**RVGSLGFFYGSIED**<br>**AAGSMGMYDQIM**<br>**AIQWIKDNAKHFG**<br>**GDPDNIVLFGESAG**<br>**GFSISMHMISPLSK**<br>**DLFKRAILQSGTAF**<br>**HPMFDDDNNGLIM**<br>**RSQVVANQSGCAD**<br>**NSDSVQKNPKRVV**<br>**DCMKSLPPEKIAET**<br>**DRVLFQTYGMLLPR**<br>**IGDAFLPENIIDSFK**<br>**KGDFKDTELLLGV**<br>**NRDEGSLFLAAQM**<br>**PDLLGVFGEKRGAL**<br>**EFNETFVRQNMLRF**<br>**LEGDENSKIPQSYL**	ATATAATTTTCTTATTATACCTCTGAAAAAAGTGCGTTTCTTT<br>CTCCAAATTGCTCTAATTAAATCAAGGAATTTCCAACAAAAT<br>GAAGCTCCTGTTTCTCCTTCTCGTTTTCCCTTTGGTAAATGGCG<br>ACAAAACTGTGACCACATCGAATGGACCAGTCCGAGGCATC<br>TCTCTAGCTCACAAGAAAGTCGAGGCTTTTCTTGGCATCCCA<br>TATGCAGAACCACCAGTGGGGTCACTAAGGTTCGCAAAGCC<br>AGTGCCCAAAACAACATGGGAAAATGTTTACGACGCTAATA<br>TGCTTCCACCTAGTTGCGTACAATTGTTCCTTGGAAACTTTTA<br>CTTCACTTCGGATCTCACTGGCGACAAGATGAGTGAGGATTG<br>CTTGTATCTTAACTTATGGGTACCTGAAGGTGGAAGTGATTTG<br>AAACCCATCCTATTGTACATCCACGGAGGAGCATTCATGATT<br>GGTTCGTCAAATATAAAAGTTTACGATGGTGCCACACTCTCG<br>GAACATGGAGATGTCATTGTGGCATCAATCAATTACAGAGTT<br>GGCTCTCTTGGATTTTTCTATGGTTCTATCGAAGATGCTGCTG<br>GAAGCATGGGAATGTACGATCAGATAATGGCCATCCAATGG<br>ATCAAAGACAACGCCAAGCATTTCGGTGGTGATCCTGATAAT<br>ATAGTACTGTTTGGTGAAAGTGCCGGTGGATTTTCGATATCTA<br>TGCACATGATATCACCCTTGTCCAAGGATCTCTTCAAAAGAG<br>CCATATTACAAAGTGGAACTGCTTTTCACCCAATGTTTGATG<br>ATGACAATAACGGTCTTATAATGAGAAGTCAGGTAGTTGCTA<br>ACCAGTCGGGATGTGCAGATAATAGCGATTCTGTACAGAAA<br>AATCCAAAACGTGTTGTTGACTGCATGAAATCTCTTCCTCCTG<br>AAAAAATTGCTGAAACGGACAGAGTTTTATTTCAAACATATG<br>GAATGCTTCTTCCACGTATAGGAGATGCATTTCTTCCAGAAA<br>ACATAATCGATTCCTTTAAGAAAGGCGATTTTAAGGACACGG<br>AACTTCTACTTGGCGTAAATCGAGACGAGGGTTCTCTCTTCTT<br>AGCTGCTCAAATGCCGGATTTATTGGGCGTATTTGGTGAAAA<br>GCGAGGAGCTCTTGAATTCAACGAGACTTTCGTTAGGCAGAA

						**DRVRKEPEYTYFQAAVDFIGDLAVTCNAIFQADYHSLKDNPVYFYVFDYRSASTPLAEWMGVTHFDEIQYVFGNPMHQNFSECEKEFSRHVMDMWVAFAKIGKPNIPGGVKWPLYTYQNPKYVMISKKDRVRVGPDDYRCESWRKFFRSVIDGDTIQKLKKSV***	CATGCTGCGTTTCTTAGAAGGAGATGAAAATAGCAAAATTCCTCAAAGTTACTTGGATCGTGTGAGAAAAGAGCCGGAATACACGTACTTTCAGGCAGCAGTTGATTTCATCGGTGATCTTGCAGTTACATGCAACGCAATATTTCAAGCTGACTATCATTCTTTGAAAGACAACCCAGTGTATTTCTATGTATTCGACTATCGTTCGGCTTCAACGCCACTGGCTGAGTGGATGGGAGTTACCCATTTTGATGAAATTCAGTATGTGTTTGGAAATCCAATGCATCAGAACTTTTCGGAATGCGAAAAGGAATTCAGCAGACATGTCATGGACATGTGGGTAGCTTTTGCGAAAATTGGAAAACCGAATATTCCAGGAGGAGTGAAATGGCCACTGTATACTTATCAAAATCCGAAATATGTTATGATCAGTAAGAAAGACAGAGTTCGTGTTGGACCTGATGACTATCGCTGTGAATCTTGGAGAAAGTTTTTCAGATCTGTAATCGATGGAGACACTATTCAAAAACTAAAAAAATCTGTTTGATACTCCACTTTTGCGGACTTCGAACTCTAAGCCTATCCTTCTGAACATGCACTGGCTCTTTTCGCTCCAGACTGCTATGCTTAACTATAAATGTCCAATTGATCTGTTTTATACTGTGCAAAATAATAACTAGCGGTGTTTATTATTAATACCATATTATGCATATATAAATTGTTAATTTAGCGATTATTAAAATTTGGATTTAAAAAAACCAGAACCAAAAGTATTAAAAATGGTCAACGTAAGATCTCCATAACTAACAGAAAATATTGTTCTATCAGAACTGAAGGTGA

PhdEnzCho<br>05	W4VSJ0	3,46E-<br>111	36,213	PF00135	61535.56<br><br>**59836.35**	MKLLFLLLVFPLVN<br>G**DKTVTTSNGPVR**<br>**GISLAHKKVEAFLG**<br>**IPYAEPPVGSLRFAK**<br>**PVPKTTWENVYDA**<br>**NMLPPSCVQLFLGN**<br>**FYFTSDLTGDKMSE**<br>**DCLYLNLWVPEGG**<br>**SDLKPILLYIHGGAF**<br>**MIGSSNIKVYDGAT**<br>**LSEHGDVIVASINY**<br>**RVGSLGFFYGSIVD**<br>**AAGSMGMYDQIM**<br>**AIQWIKDNAKHFG**<br>**GDPDNIVLFGESAG**<br>**GFSISMHMISPLSK**<br>**NLFKRAILQSGTAF**<br>**HPMFNDNNGLLM**<br>**MSQVIANQLGCAD**<br>**DSDSLQKNPESVV**<br>**NCMKALPPEKFAEA**<br>**DSILFKNVGMIVPR**<br>**IGDAFLPESTIDSIK**<br>**KGDFKDTELLLGVT**<br>**RDEGSLFLTFRMVD**<br>**SLGLFGEMHENLEF**<br>**NETFTKQSMLLLAN**<br>**MGEGEENNQILQS**	ATATAATTTTCTTATTATACCTCTGAAAAAAGTGCGTTTCTTT<br>CTCCAAATTGCTCTAATTAAATCAAGGAATTTCCAACAAAAT<br>GAAGCTCCTGTTTCTCCTTCTCGTTTTCCCTTTGGTAAATGGCG<br>ACAAAACTGTGACCACATCGAATGGACCAGTCCGAGGCATC<br>TCTCTAGCTCACAAGAAAGTCGAGGCTTTTCTTGGCATCCCA<br>TATGCAGAACCACCAGTGGGGTCACTAAGGTTCGCAAAGCC<br>AGTGCCCAAAACAACATGGGAAAATGTTTACGACGCTAATA<br>TGCTTCCACCTAGTTGCGTACAATTGTTCCTTGGAAACTTTTA<br>CTTCACTTCGGATCTCACTGGCGACAAGATGAGTGAGGATTG<br>CTTGTATCTTAACTTATGGGTACCTGAAGGTGGAAGTGATTTG<br>AAACCCATCCTATTGTACATCCACGGAGGAGCATTCATGATT<br>GGTTCGTCAAATATAAAAGTTTACGATGGTGCCACACTCTCG<br>GAACATGGAGATGTCATTGTGGCATCAATCAATTACAGAGTT<br>GGCTCTCTTGGATTTTTCTATGGTTCTATCGTAGATGCTGCTG<br>GAAGCATGGGAATGTACGATCAGATAATGGCCATCCAATGG<br>ATCAAAGACAACGCCAAGCATTTCGGTGGCGATCCAGATAA<br>TATAGTACTGTTTGGTGAAAGTGCCGGTGGATTTTCGATATCT<br>ATGCACATGATATCACCTTTGTCTAAGAATCTCTTCAAAAGA<br>GCCATATTACAAAGTGGAACTGCTTTTCACCCGATGTTTAAT<br>GATAACAACGGACTTCTGATGATGAGTCAGGTAATTGCCAAC<br>CAGTTAGGATGTGCAGATGATAGCGATTCTCTACAGAAAAAT<br>CCAGAGAGTGTTGTCAATTGTATGAAAGCCCTTCCTCCTGAA<br>AAATTTGCCGAAGCGGACAGCATTTTATTTAAAAACGTTGGA<br>ATGATCGTTCCACGTATCGGAGATGCATTTCTTCCAGAAAGC<br>ACAATCGATTCCATTAAAAAAGGCGATTTTAAGGACACAGA<br>ACTTCTCCTTGGAGTTACTCGAGACGAGGGTTCTCTCTTCCTA<br>ACTTTCCGAATGGTGGATTCATTGGGCTTATTCGGTGAAATGC<br>ATGAAAATCTTGAATTCAACGAGACTTTCACTAAGCAGTCCA

						**YLDRVKKEPEYTYLQAIIDFIGDLTITCGAVYQADYHSLKDNPVYFYVFDYRPASTPLAEWMGVAHFDEIQYVFGNPMHQDFTEYEKEFSRDLMDMWIAFAKTGKPNIPGGVKWPLYTYQNPKYVMISKKDRVRVGPDDYRCESWRKFFRSVIDGDTIQKLKKSV***	TGTTGCTTCTGGCAAATATGGGAGAAGGAGAAGAAAATAACCAAATCTTACAAAGTTACTTGGATCGTGTGAAAAAAGAGCCAGAATACACCTACTTGCAGGCAATAATAGATTTTATCGGTGATCTCACGATTACTTGCGGCGCAGTATATCAAGCTGACTATCATTCTTTAAAAGACAATCCAGTGTATTTCTATGTATTCGATTATCGTCCGGCTTCAACGCCATTGGCTGAATGGATGGGAGTTGCTCATTTTGATGAAATACAATATGTATTTGGAAATCCAATGCATCAGGACTTTACGGAATACGAAAAGGAATTCAGCAGAGATCTCATGGATATGTGGATCGCTTTTGCGAAGACGGGAAAACCGAATATTCCAGGAGGAGTGAAATGGCCACTGTATACTTATCAAAATCCGAAATATGTTATGATCAGTAAGAAAGACAGAGTTCGTGTTGGACCTGATGACTATCGCTGTGAATCTTGGAGAAAGTTTTTCAGATCTGTAATCGATGGAGACACTATTCAAAAACTAAAAAAATCTGTTTGATACTCCACTTTTGCGGACTTCGAACTCTAAGCCTATCCTTCTGAACATGCACTGGCTCTTTTCGCTCCAGACTGCTATGCTTAACTATAAATGTCCAATTGATCTGTTTTATACTGTGCAAAATAATAACTAGCGGTGTTTATTATTAATACCATATTATGCATATATAAATTGTTAATTTAGCGATTATTAAAATTTGGATTTAAAAAAACCAGAACCAAAAGTATTAAAAATGGTCAACGTAAGATCTCCATAACTAACAGAAAATATTGTTCTATCAGAACTGAAGGTGA

PhdEnzCho 06	W4VSJ0	9,91E-112	36,414	PF00135	61548.73<br>**59849.51**	MKLLFLLLVFPLVN<br>**GDKIVTTTNGPVR**<br>**GISLAPKKVEAFLGI**<br>**PYAEPPVGQLRFAK**<br>**PVPKRTWENVYEA**<br>**NMLPPSCVQLYLG**<br>**NLYFTPDLTGGGM**<br>**SEDCLYLNIWVPEG**<br>**GSDLKPIMLFIYGG**<br>**GFMHGSSNMKLYD**<br>**GAILSGHGDVIVAT**<br>**INYRVGSLGFFYGSI**<br>**EDAAGSMGMYDQI**<br>**MAIQWIKDNAKHF**<br>**GGDPDNIVLFGESA**<br>**GGFSISMHMISPLS**<br>**KNLFKRAILQSGTA**<br>**FHPMFNDNNGLLM**<br>**MSQVIANQLGCAD**<br>**DSDSLQKNPESVV**<br>**NCMKALPPEKFAEA**<br>**DSILFKNVGMIVPR**<br>**IGDAFLPESTIDSIK**<br>**KGDFKDTELLLGVT**<br>**RDEGSLFLTFRMVD**<br>**SLGLFGEMHENLEF**<br>**NETFTKQSMLLLAN**<br>**MGEGEENNQILQS**	GTTGAGGACTCTTTTAGAAACGATCTTATTGGGATGTAAAGC<br>TTTAAATTTTAGAGAAGCCCTGCAGTCTTTATTCAAAACTATC<br>ACGCTATATATATAGAAGTAATATACAAGTTATGTACTGTGA<br>TTATTTGACTTGCTTATTATACATCTAAAAAAAGTACATTTCT<br>TTCTCAACATTTGCTCTAATTAAATCAAGGAATTCCCGATAA<br>CATGAAGCTCCTGTTTCTCCTTCTCGTTTTCCCTTTGGTAAATG<br>GCGACAAAATTGTGACCACAACGAATGGACCAGTCAGAGGC<br>ATCTCTCTAGCTCCCAAAAAAGTCGAGGCTTTCCTTGGCATC<br>CCATATGCTGAACCACCAGTGGGGCAATTAAGATTTGCAAA<br>GCCAGTGCCCAAGAGAACATGGGAAAATGTTTACGAAGCTA<br>ATATGCTTCCACCTAGTTGCGTACAATTATACCTTGGAAATCT<br>TTACTTCACTCCGGATCTCACTGGCGGCGGGATGAGTGAGGA<br>TTGCTTGTATCTTAACATATGGGTACCCGAAGGTGGAAGTGA<br>TTTGAAACCCATCATGTTGTTTATCTATGGAGGAGGATTCATG<br>CATGGATCGTCAAATATGAAACTTTACGATGGTGCCATACTC<br>TCGGGGCATGGAGATGTCATAGTGGCAACAATCAACTACAG<br>AGTTGGCTCTCTTGGATTTTTTTATGGTTCAATCGAAGATGCT<br>GCTGGAAGCATGGGAATGTACGATCAGATAATGGCCATCCA<br>ATGGATCAAAGACAACGCCAAGCATTTCGGTGGCGATCCAG<br>ATAATATAGTACTGTTTGGTGAAAGTGCCGGTGGATTTTCGA<br>TATCTATGCACATGATATCACCTTTGTCTAAGAATCTCTTCAA<br>AAGAGCCATATTACAAAGTGGAACTGCTTTTCACCCGATGTT<br>TAATGATAACAACGGACTTCTGATGATGAGTCAGGTAATTGC<br>CAACCAGTTAGGATGTGCAGATGATAGCGATTCTCTACAGAA<br>AAATCCAGAGAGTGTTGTCAATTGTATGAAAGCCCTTCCTCC<br>TGAAAAATTTGCCGAAGCGGACAGCATTTTATTTAAAAACGT<br>TGGAATGATCGTTCCACGTATCGGAGATGCATTTCTTCCAGA<br>AAGCACAATCGATTCCATTAAAAAAGGCGATTTTAAGGACA

						**YLDRVKKEPEYTYL<br>QAIIDFIGDLTITCG<br>AVYQADYHSLKDN<br>PVYFYVFDYRPAST<br>PLAEWMGVAHFDE<br>IQYVFGNPMHQDF<br>TEYEKEFSRDLMDM<br>WIAFAKTGKPNIPG<br>GVKWPLYTYQNPK<br>YVMISKKDRVRVG<br>PDDYRCESWRKFFR<br>SVIDGDTIQKLKKS<br>V***	CAGAACTTCTCCTTGGAGTTACTCGAGACGAGGGTTCTCTCTT<br>CCTAACTTTCCGAATGGTGGATTCATTGGGCTTATTCGGTGAA<br>ATGCATGAAAATCTTGAATTCAACGAGACTTTCACTAAGCAG<br>TCCATGTTGCTTCTGGCAAATATGGGAGAAGGAGAAGAAAA<br>TAACCAAATCTTACAAAGTTACTTGGATCGTGTGAAAAAAGA<br>GCCAGAATACACCTACTTGCAGGCAATAATAGATTTTATCGG<br>TGATCTCACGATTACTTGCGGCGCAGTATATCAAGCTGACTA<br>TCATTCTTTAAAAGACAATCCAGTGTATTTCTATGTATTCGAT<br>TATCGTCCGGCTTCAACGCCATTGGCTGAATGGATGGGAGTT<br>GCTCATTTTGATGAAATACAATATGTATTTGGAAATCCAATG<br>CATCAGGACTTTACGGAATACGAAAAGGAATTCAGCAGAGA<br>TCTCATGGATATGTGGATCGCTTTTGCGAAGACGGGAAAACC<br>GAATATTCCAGGAGGAGTGAAATGGCCACTGTATACTTATCA<br>AAATCCGAAATATGTTATGATCAGTAAGAAAGACAGAGTTC<br>GTGTTGGACCTGATGACTATCGCTGTGAATCTTGGAGAAAGT<br>TTTTCAGATCTGTAATCGATGGAGACACTATTCAAAAACTAA<br>AAAAATCTGTTTGATACTCCACTTTTGCGGACTTCGAACTCTA<br>AGCCTATCCTTCTGAACATGCACTGGCTCTTTTCGCTCCAGAC<br>TGCTATGCTTAACTATAAATGTCCAATTGATCTGTTTTATACT<br>GTGCAAAATAATAACTAGCGGTGTTTATTATTAATACCATAT<br>TATGCATATATAAATTGTTAATTTAGCGATTATTAAAATTTGG<br>ATTTAAAAAAACCAGAACCAAAAGTATTAAAAATGGTCAAC<br>GTAAGATCTCCATAACTAACAGAAAATATTGTTCTATCAGAA<br>CTGAAGGTGA

PhdEnzCho 07	W4VSJ0	1,65E-104	35,196	PF00135	61480.56<br>**59781.34**	MKLLFLLLVFPLVN<br>G**DKIVTTTNGPVR**<br>**GISLAPKKVEAFLGI**<br>**PYAEPPVGQLRFAK**<br>**PVPKRTWENVYEA**<br>**NMLPPSCVQLYLG**<br>**NLYFTPDLTGGGM**<br>**SEDCLYLNIWVPEG**<br>**GSDLKPIMLFIYGG**<br>**GFMHGSSNMKLYD**<br>**GAILSGHGDVIVAT**<br>**INYRVGSLGFFYGSI**<br>**EDAAGSMGMYDQI**<br>**MAIQWIKDNAKHF**<br>**GGDPDNIVLFGESA**<br>**GGFSISMHMISPLS**<br>**KDLFKRAILQSGTA**<br>**FHPMFDDDNNGLI**<br>**MRSQVVANQSGC**<br>**ADNSDSVQKNPKR**<br>**VVDCMKSLPPEKIA**<br>**ETDRVLFQTYGMLL**<br>**PRIGDAFLPENIIDS**<br>**FKKGDFKDTELLLG**<br>**VNRDEGSLFLAAQ**<br>**MPDLLGVFGEKRG**<br>**ALEFNETFVRQNML**<br>**RFLEGDENSKIPQSY**	GTTGAGGACTCTTTTAGAAACGATCTTATTGGGATGTAAAGC<br>TTTAAATTTTAGAGAAGCCCTGCAGTCTTTATTCAAAACTATC<br>ACGCTATATATATAGAAGTAATATACAAGTTATGTACTGTGA<br>TTATTTGACTTGCTTATTATACATCTAAAAAAAGTACATTTCT<br>TTCTCAACATTTGCTCTAATTAAATCAAGGAATTCCCGATAA<br>CATGAAGCTCCTGTTTCTCCTTCTCGTTTTCCCTTTGGTAAATG<br>GCGACAAAATTGTGACCACAACGAATGGACCAGTCAGAGGC<br>ATCTCTCTAGCTCCCAAAAAAGTCGAGGCTTTCCTTGGCATC<br>CCATATGCTGAACCACCAGTGGGGCAATTAAGATTTGCAAA<br>GCCAGTGCCCAAGAGAACATGGGAAAATGTTTACGAAGCTA<br>ATATGCTTCCACCTAGTTGCGTACAATTATACCTTGGAAATCT<br>TTACTTCACTCCGGATCTCACTGGCGGCGGGATGAGTGAGGA<br>TTGCTTGTATCTTAACATATGGGTACCCGAAGGTGGAAGTGA<br>TTTGAAACCCATCATGTTGTTTATCTATGGAGGAGGATTCATG<br>CATGGATCGTCAAATATGAAACTTTACGATGGTGCCATACTC<br>TCGGGGCATGGAGATGTCATAGTGGCAACAATCAACTACAG<br>AGTTGGCTCTCTTGGATTTTTTTATGGTTCAATCGAAGATGCT<br>GCTGGAAGCATGGGAATGTACGATCAGATAATGGCCATCCA<br>ATGGATCAAAGACAACGCCAAGCATTTCGGTGGTGATCCTG<br>ATAATATAGTACTGTTTGGTGAAAGTGCCGGTGGATTTTCGA<br>TATCTATGCACATGATATCACCCTTGTCCAAGGATCTCTTCAA<br>AAGAGCCATATTACAAAGTGGAACTGCTTTTCACCCAATGTT<br>TGATGATGACAATAACGGTCTTATAATGAGAAGTCAGGTAGT<br>TGCTAACCAGTCGGGATGTGCAGATAATAGCGATTCTGTACA<br>GAAAAATCCAAAACGTGTTGTTGACTGCATGAAATCTCTTCC<br>TCCTGAAAAAATTGCTGAAACGGACAGAGTTTTATTTCAAAC<br>ATATGGAATGCTTCTTCCACGTATAGGAGATGCATTTCTTCCA<br>GAAAACATAATCGATTCCTTTAAGAAAGGCGATTTTAAGGAC

						**LDRVRKEPEYTYFQ<br>AAVDFIGDLAVTC<br>NAIFQADYHSLKD<br>NPVYFYVFDYRSAS<br>TPLAEWMGVTHFD<br>EIQYVFGNPMHQN<br>FSECEKEFSRHVMD<br>MWVAFAKIGKPNI<br>PGGVKWPLYTYQN<br>PKYVMISKKDRVR<br>VGPDDYRCESWRK<br>FFRSVIDGDTIQKL<br>KKSV***	ACGGAACTTCTACTTGGCGTAAATCGAGACGAGGGTTCTCTC<br>TTCTTAGCTGCTCAAATGCCGGATTTATTGGGCGTATTTGGTG<br>AAAAGCGAGGAGCTCTTGAATTCAACGAGACTTTCGTTAGGC<br>AGAACATGCTGCGTTTCTTAGAAGGAGATGAAAATAGCAAA<br>ATTCCTCAAAGTTACTTGGATCGTGTGAGAAAAGAGCCGGA<br>ATACACGTACTTTCAGGCAGCAGTTGATTTCATCGGTGATCTT<br>GCAGTTACATGCAACGCAATATTTCAAGCTGACTATCATTCT<br>TTGAAAGACAACCCAGTGTATTTCTATGTATTCGACTATCGTT<br>CGGCTTCAACGCCACTGGCTGAGTGGATGGGAGTTACCCATT<br>TTGATGAAATTCAGTATGTGTTTGGAAATCCAATGCATCAGA<br>ACTTTTCGGAATGCGAAAAGGAATTCAGCAGACATGTCATG<br>GACATGTGGGTAGCTTTTGCGAAAATTGGAAAACCGAATATT<br>CCAGGAGGAGTGAAATGGCCACTGTATACTTATCAAAATCC<br>GAAATATGTTATGATCAGTAAGAAAGACAGAGTTCGTGTTGG<br>ACCTGATGACTATCGCTGTGAATCTTGGAGAAAGTTTTTCAG<br>ATCTGTAATCGATGGAGACACTATTCAAAAACTAAAAAAAT<br>CTGTTTGATACTCCACTTTTGCGGACTTCGAACTCTAAGCCTA<br>TCCTTCTGAACATGCACTGGCTCTTTTCGCTCCAGACTGCTAT<br>GCTTAACTATAAATGTCCAATTGATCTGTTTTATACTGTGCAA<br>AATAATAACTAGCGGTGTTTATTATTAATACCATATTATGCAT<br>ATATAAATTGTTAATTTAGCGATTATTAAAATTTGGATTTAAA<br>AAAACCAGAACCAAAAGTATTAAAAATGGTCAACGTAAGAT<br>CTCCATAACTAACAGAAAATATTGTTCTATCAGAACTGAAGG<br>TGA

PhdEnzCho 08	W4VSJ0	1,64E-28	51,724	PF00135	9220,31	GGLGAGSGRMPLYDGRVLTAYGDVIVVTINYRVSALGFFTSETEEAPGNVGLYDMLEALKWTNENIAAFGGDNKRITVFGNSAGGISAG	ACCTGCAGATATTCCTCCGGCGCTGTTGCCGAACACCGTAATTCTTTTATTGTCTCCACCGAATGCAGCAATATTTTCGTTCGTCCATTTAAGAGCTTCCAACATGTCATACAAGCCGACGTTACCAGGAGCTTCTTCCGTTTCAGATGTGAAGAATCCAAGCGCGCTTACCCGGTAGTTTATTGTAACAACTATTACATCTCCATATGCAGTTAGAACACGTCCATCATATAGAGGCATCCGACCTGAACCGGCTCCCAAGCCGCC
PhdEnzCho 09	W4VSJ0	1,64E-28	51,724	PF00135	9220,31	GGLGAGSGRMPLYDGRVLTAYGDVIVVTINYRVSALGFFTSETEEAPGNVGLYDMLEALKWTNENIAAFGGDNKRITVFGNSAGGISAG	ACCTGCAGATATTCCTCCGGCGCTGTTGCCGAACACCGTAATTCTTTTATTGTCTCCACCGAATGCAGCAATATTTTCGTTCGTCCATTTAAGGGCTTCCAACATGTCATACAAACCGACGTTACCAGGAGCTTCTTCCGTTTCAGATGTGAAGAATCCAAGCGCGCTTACCCGGTAGTTTATTGTAACAACTATTACATCTCCATATGCAGTTAGAACACGTCCATCATATAGAGGCATCCGACCTGAACCGGCTCCCAAGCCGCC
PhdEnzCho 10	W4VSJ0	3,70E-24	33,898	PF00135	21004,2	SLMREYFQSIFPDPEGIIQHYLTGVADDDYDTVRSQIYHSLGDEFFHCPSKYFSEKFAEKNNNTYRYYFNHRRRNTRWAEWMKVAHFEEVEFVFGRALNPLANYTDYTKEEEELSLEIVKEWTNFAKTGEPGGMWRKFTESDPYVKVFELSDSDTSILEFHEENCEFFRPYFGF*	GTCCCTCATGAGAGAGTATTTCCAGTCAATATTTCCAGATCCGGAAGGAATAATCCAGCATTATCTCACTGGTGTTGCAGATGACGATTATGATACAGTAAGGAGTCAAATTTATCACTCTTTAGGGGATGAATTTTTTCACTGTCCAAGCAAATATTTTTCTGAGAAATTTGCGGAAAAAAATAATAATACCTACCGGTATTATTTCAATCACAGAAGAAGGAATACTCGCTGGGCAGAGTGGATGAAGGTTGCGCATTTTGAAGAAGTGGAGTTTGTCTTTGGACGCGCTTTAAATCCTCTGGCTAACTACACCGACTACACGAAAGAGGAAGAAGAATTAAGCCTTGAGATCGTAAAAGAGTGGACGAATTTTGCAAAGACAGGGGAACCCGGTGGAATGTGGCGGAAGTTCACTGAATCAGACCCATACGTCAAAGTTTTTGAATTGTCGGACAGCGATACGAGTATCTTGGAATTCCACGAAGAGAACTGCGAATTTTTTCGGCCTTATTTTGGATTTTAATCACATTCGTGTTAAAGGGAAATCGCTATGAATCTTTGTAAGTATTTGAATTGCGATGAAA

							GCATTCAAATATCGTTGAAATGTTAAGATTCATGCACTTCATTATAAATAATATATAATATTATATGGTAAATCTTTATGTGGTATATAAATGGAAACTAAATAAAATGAAGCTGGGAAACAATAAACAGG
PhdEnzCho11	W4VSJ0	2,05E-27	33,158	PF00135	24269,97	GSYLLANNFLQIVGPLGELNPRINKTFGESLMREYFQKIFPDPEEIIQYYLDGVADDDYDTVRSQVYHSIGDELFHCPSMYFSEKFAEKNNNTYRYYFNHRRRNTRWAEWMKVAHFEEVEFVFGRALNPLANYTDYTKEEEELSLEIVKEWTNFAKTGEPGGMWRKFTESDPYVKVFELSDSDTSILEFHEENCEFFRPYFGF*	GGATCATACCTACTTGCTAATAATTTTCTTCAGATCGTAGGCCCTTTGGGTGAATTAAATCCTCGCATTAATAAGACGTTTGGGGAGTCTCTCATGAGGGAATATTTCCAGAAAATATTTCCGGATCCGGAAGAAATAATTCAGTATTATCTCGATGGTGTTGCAGATGACGATTATGATACAGTAAGGAGTCAAGTTTATCACTCTATAGGGGATGAACTTTTTCACTGCCCAAGCATGTATTTTTCTGAGAAATTTGCGGAAAAAAATAATAATACCTACCGGTATTATTTCAATCACAGAAGAAGGAATACTCGCTGGGCAGAGTGGATGAAGGTTGCGCATTTTGAAGAAGTGGAGTTTGTCTTTGGACGCGCTTTAAATCCTCTGGCTAACTACACCGACTACACGAAAGAGGAAGAAGAATTAAGCCTTGAGATCGTAAAAGAGTGGACGAATTTTGCAAAGACAGGGGAACCCGGTGGAATGTGGCGGAAGTTCACTGAATCAGACCCATACGTCAAAGTTTTTGAATTGTCGGACAGCGATACGAGTATCTTGGAATTCCACGAAGAGAACTGCGAATTTTTTCGGCCTTATTTTGGATTTTAATCACATTCGTGTTAAAGGGAAATCGCTATGAATCTTTGTAAGTATTTGAATTGCGATGAAAGCATTCAAATATCGTTGAAATGTTAAGATTCATGCACTTCATTATAAATAATATATAATATTATATGGTAAATCTTTATGTGGT

							ATATAAATGGAAACTAAATAAAATGAAGCTGGGAAACAATAAACAGG

PhdEnzCho 12	W4VSJ0	8,79E-24	33,898	PF00135	21149,32	SLMREYFQSIFPDPE GIIQHYLTGVADDD YDTVRSQIYHSLGDE FFHCPSKYFSEKFAE KNNNTYRYYFNHRR RNTRWAEWMKVA HFEEVEFVFGRALNP LANYTDYTKEEEELS LEIVKDWTNFAKTG EPDEMWRKFSRSDP YVKVFELSDSDTSILE YHEENCEFFRPYFGF *	GTCCCTCATGAGAGAGTATTTCCAGTCAATATTTCCAGATCC GGAAGGAATAATCCAGCATTATCTCACTGGTGTTGCAGATGA CGATTATGATACAGTAAGGAGTCAAATTTATCACTCTTTAGG GGATGAATTTTTTCACTGTCCAAGCAAATATTTTTCTGAGAAA TTTGCGGAAAAAAATAATAATACCTACCGGTATTATTTCAAT CACAGAAGAAGGAATACTCGCTGGGCAGAGTGGATGAAGGT TGCGCATTTTGAAGAAGTGGAGTTTGTCTTTGGACGCGCTTTA AATCCTCTGGCCAATTACACCGACTACACCAAAGAGGAAGA AGAATTAAGCCTTGAGATCGTAAAAGATTGGACGAATTTTGC AAAGACAGGGGAACCCGATGAAATGTGGCGGAAGTTCTCTA GATCAGACCCATACGTCAAAGTTTTTGAATTGTCGGACAGCG ATACGAGTATCTTGGAATACCACGAAGAAAACTGCGAATTTT TTCGGCCTTATTTTGGATTTTAATCAAATTCATGTTAAAGAGA AATAACTATGAATCCCAAAAAGTTGTTTGAATTCTTCTCAATT GTTTGAATCAAATTCAAACAAGTCTGACAAGTTGTTTGAATT TTATTTTCATTCCATTTTATCTCCCTGAAACATTCAAATATCAT TGGAACATTAACAATTATGCACTTTATTATAAACAATGCAAA ATATTATAGGGTATACAGTATTATCGAGAAAGATTCGTATTA CAAGATTCAATTTTAAATGTTATTAAATAAAATGAAGCTAAA CCTTCAGGCTACAGCAATAAACAGTTCTATATATATATAAAG AAAGTTTGTTTATTTATTCGACTTAGTAATCACATTAATATTC CCATACACGATCTTTTGGTCACTCGTTATCGGTGACACGTTTT AATATATGTGTACATCCCATTTCATGCCCATCCATGGCTACTT CTATAGTTTTTCTGCACTTTTGTGCTTGCTCCCCTAATTTTTTTG AGCAGTTTAGCTGATTCGACGTATAAATTCCGTAATATAATC AAGTAGATCCTATAAGTATGAGATACCGTACCCCTCCAATCC AAAAATCAAATGGAAAAGCAGAATTAAGAAAGAATGTTTC ATTTGCCGAGGCAACCAAATTGCTAAAATCTGTAACCAAAG

							ATTTACGAAAAATATCCGATCTAAAGAAAAATCGACGGAAA<br>AAAAACCAACAAATGGCAATATTGCAGAAAATTTGAAGTCT<br>GGGTGTCTTAATTCTCATAATGCAAACATTAAAATTCAACCT<br>TTAAACTATGTTAATCTATTAACAGAGGGAAATTATACTAAA<br>GTTGTTTAGATAGTGGTTGCCAATTTGCTATTTTGAATAAATC<br>CTATGTTCCTATTTGGAAACCTGAAGATAAAAGCCATGGAGG<br>GGTCAATATGTATGTGTTTAGTTATGAATGAGATTGATTATTC<br>TTTTGTTTAGTTATTGTAGTTAGTTATCGATCGTATATTGTTGT<br>GTTGGCAAATGATTTGCTTTTAAATGTTAACGAAAGTTGCAG<br>GAAGGTGAAATTGAGATTGTGTTATCACAATTACATCAATCT<br>TGCATAGGGGTGTTGTGTACCCATTCCTACGCAAGGTTTCGGT<br>ATTTCGTTAGTCGGAATGGCAACGGCGAATTTTGTTTTTACGG<br>GCGGGCAGGAGTTAGCGAGAGTGAACTCGCGAGATTAGCAG<br>AGTGTTCATTTTTGCGTAAATAGTTCAAAATTATTTTGATTGG<br>GTATAGGTCTGTATGTGCGTATAAAGGTGTGTGCCTACATAA<br>TCTTCTTCAGTATCATCAATTGACTGTAGAATATTTTTTCGAT<br>GTGAAACGATACTACCGAAACTGTATACAAATATTTTTCGGT<br>GACAGCAGTCAGTATATGAATTTCTGGAATATATTTTATAGTT<br>ACATGATAAAGTGTACCTTTCTTAAACTGAAAAATTTAATTA<br>TTTGAAGAGTTACTTTTCAGACGGCGCGGCATCTGACATTGA<br>CGGTTTTATGATGTCAGAATCCATTAACGATTCAGCATTAAA<br>TATTTTAGATAAGAAGATTCCGTGGAAAAGAGATTATTGTTG<br>ATTTACGAATGAAGAAACACTTCTGGATTTAGTACACATATA<br>TAAGTCGAGGAACATTAGAAAAAAATTGCATCGTGATATTG<br>AAACTAACTTGTGAAGATTGCAGCTGAATCTACCTAAGAATA<br>ACAAACCTATAAGTAAACGACGCCGTCGTACAGTGAATTGC<br>TCGGCATTACGAAAATCTATATTAACACATTGATGACGGTAT<br>GCTCTGATAAGCATATAACGTGAATTCCCACGTATGTTTTTGA

							TTATTTATTAAATGATGGAATTGATTTGAAAGACCATATTTTA<br>TAATCTTTCCCGGGTTATATGTTCAAAATTTTAATCTTCCTCTC<br>CATGGAATCATAGCTTCATCCAAAGATACTTCTGTTGTTGTTC<br>TTCTTGTGGCTTATCTCATTTGCCTGACCGCAACATCAGATCA<br>TATCCATGACCTCTCACAACTGATCCAGAAACGCCCCAGAGT<br>TATAAAACAGATGGTTCTTGGTGCAACCAATGCACAATAGA<br>ACATGAGAGGGAGTCGTCTCAGTCAAGC

PhdEnzCho 13	W4VSJ0	8,72E-24	33,898	PF00135	21149,32	SLMREYFQSIFPDPE GIIQHYLTGVADDD YDTVRSQIYHSLGDE FFHCPSKYFSEKFAE KNNNTYRYYFNHRR RNTRWAEWMKVA HFEEVEFVFGRALNP LANYTDYTKEEEELS LEIVKDWTNFAKTG EPDEMWRKFSRSDP YVKVFELSDSDTSILE YHEENCEFFRPYFGF *	GTCCCTCATGAGAGAGTATTTCCAGTCAATATTTCCAGATCC GGAAGGAATAATCCAGCATTATCTCACTGGTGTTGCAGATGA CGATTATGATACAGTAAGGAGTCAAATTTATCACTCTTTAGG GGATGAATTTTTTCACTGTCCAAGCAAATATTTTTCTGAGAAA TTTGCGGAAAAAAATAATAATACCTACCGGTATTATTTCAAT CACAGAAGAAGGAATACTCGCTGGGCAGAGTGGATGAAGGT TGCGCATTTTGAAGAAGTGGAGTTTGTCTTTGGACGCGCTTTA AATCCTCTGGCCAATTACACCGACTACACCAAAGAGGAAGA AGAATTAAGCCTTGAGATCGTAAAAGATTGGACGAATTTTGC AAAGACAGGGGAACCCGATGAAATGTGGCGGAAGTTCTCTA GATCAGACCCATACGTCAAAGTTTTTGAATTGTCGGACAGCG ATACGAGTATCTTGGAATACCACGAAGAAAATTGCGAATTTT TTCGGCCTTATTTTGGATTTTAATCAAATTCATGTTAAAGAGA AATAACTATGAATCCCAAAAAGTTGTTTGAATTCTTCTCAATT GTTTGAATCAAATTCAAACAAGTCTGAAAAGTTGTTTGAATT TGATTTTCTCCCTGAAACATTCAAATATCATTGGAACATTAAC AATTATGCACTTTATTATAAACAATGCAAAATATTATAGGGT ATACAGTATTATCGAGAAAGATTCGTATTACAAGATTCAATT TTAAATGTTATTAAATAAAATGAAGCTAAACCTTCAGGCTAC AGCAATAAACAGTTCTATATATATATAAAGAAAGTTTGTTTA TTTATTCGACTTAGTAATCACATTAATATTCCCATACACGATC TTTTGGTCACTCGTTATCGGTGACACGTTTTAATATATGTGTA CATCCCATTTCATGCCCATCCATGGCTACTTCTATAGTTTTTCT GCACTTTTGTGCTTGCTCCCCTAATTTTTTTGAGCAGTTTAGCT GATTCGACGTATAAATTCCGTAATATAATCAAGTAGATCCTA TAAGTATGAGATACCGTACCCCTCCAATCCAAAAATCAAAT GGGAAAAGCAGAATTAAGAAAGAATGTTTCATTTGCCGAGG CAACCAAATTGCTAAAATCTGTAACCAAAGATTTACGAAAA

							ATATCCGATCTAAAGAAAAATCGACGGAAAAAAAACCAAC<br>AAATGGCAATATTGCAGAAAATTTGAAGTCTGGGTGTCTTAA<br>TTCTCATAATGCAAACATTAAAATTCAACCTTTAAACTATGTT<br>AATCTATTAACAGAGGGAAATTATACTAAAGTTGTTTAGATA<br>GTGGTTGCCAATTTGCTATTTTGAATAAATCCTATGTTCCTATT<br>TGGAAACCTGAAGATAAAAGCCATGGAGGGGTCAATATGTA<br>TGTGTTTAGTTATGAATGAGATTGATTATTCTTTTGTTTAGTTA<br>TTGTAGTTAGTTATCGATCGTATATTGTTGTGTTGGCAAATGA<br>TTTGCTTTTAAATGTTAACGAAAGTTGCAGGAAGGTGAAATT<br>GAGATTGTGTTATCACAATTACATCAATCTTGCATAGGGGTG<br>TTGTGTACCCATTCCTACGCAAGGTTTCGGTATTTCGTTAGTC<br>GGAATGGCAACGGCGAATTTTGTTTTTACGGGCGGGCAGGA<br>GTTAGCGAGAGTGAACTCGCGAGATTAGCAGAGTGTTCATTT<br>TTGCGTAAATAGTTCAAAATTATTTTGATTGGGTATAGGTCTG<br>TATGTGCGTATAAAGGTGTGTGCCTACATAATCTTCTTCAGTA<br>TCATCAATTGACTGTAGAATATTTTTTCGATGTGAAACGATAC<br>TACCGAAACTGTATACAAATATTTTTCGGTGACAGCAGTCAG<br>TATATGAATTTCTGGAATATATTTTATAGTTACATGATAAAGT<br>GTACCTTTCTTAAACTGAAAAATTTAATTATTTGAAGAGTTAC<br>TTTTCAGACGGCGCGGCATCTGACATTGACGGTTTTATGATGT<br>CAGAATCCATTAACGATTCAGCATTAAATATTTTAGATAAGA<br>AGATTCCGTGGAAAAGAGATTATTGTTGATTTACGAATGAAG<br>AAACACTTCTGGATTTAGTACACATATATAAGTCGAGGAACA<br>TTAGAAAAAAATTGCATCGTGATATTGAAACTAACTTGTGAA<br>GATTGCAGCTGAATCTACCTAAGAATAACAAACCTATAAGT<br>AAACGACGCCGTCGTACAGTGAATTGCTCGGCATTACGAAA<br>ATCTATATTAACACATTGATGACGGTATGCTCTGATAAGCAT<br>ATAACGTGAATTCCCACGTATGTTTTTGATTATTTATTAAATG

							ATGGAATTGATTTGAAAGACCATATTTTATAATCTTTCCCGGGTTATATGTTCAAAATTTTAATCTTCCTCTCCATGGAATCATAGCTTCATCCAAAGATACTTCTGTTGTTGTTCTTCTTGTGGCTTATCTCATTTGCCTGACCGCAACATCAGATCATATCCATGACCTCTCACAACTGATCCAGAAACGCCCCAGAGTTATAAAACAGATGGTTCTTGGTGCAACCAATGCACAATAGAACATGAGAGGGAGTCGTCTCAGTCAAGC

PhdEnzCho 14	W4VSJ0	1,20E-109	37,618	PF00135	63354,11	LKNCPQNKLSDISFL<br>NNAISILNSVFQSLH<br>LLFLLLPGTILASSVT<br>VNTPTGEVTGSVMT<br>YDDINLNVFLGIPFA<br>EPPVGNLRFKRTVPV<br>KPWTSPVIANSLPPA<br>CYQYSPDNYPWEDK<br>VQGQSEDCLYLNIW<br>APEISNSSEKKAVMF<br>WVFSGGFATGSGRM<br>PLYDGRVLTAIGDVI<br>VVAINYRLSVLGFFT<br>SETEEAPGNVGLYDI<br>LEALKWTNENIAAF<br>GGDNKRITVFGNSA<br>GGVATGLLCVSPLSK<br>GLFQRAIMQSGCPA<br>YFLNDHRAENLKLG<br>QKLAEKMECANENI<br>TLKKNPDDVIECLRS<br>KAPKDMVRTTFSLV<br>PVFPVITFFTISGDDF<br>LPVDPQKAIREGNF<br>NGVDSMIGVTQDEG<br>SFPFVNTFTQITGPFG<br>ELNPRINKTLGESLM<br>RDYFGTIFPDPEGIIQ	CGATAAACCTTTTGCAAGCAAGTTTCTCATGATCTGGCAATA<br>TTCTAGAAAGTGCAAACATGATTATGTAATATGAAAAATGCT<br>GTATATGAAACTCGAGGTATTTTTGATTTATTCTTTATACTTTC<br>ATATTACATATTCATCTTTACACTTTCATAACTATGCTTTCATG<br>AGTCTGCGTACATCAACATAACACATTAGAAAACTTTAATAT<br>CAATTCATATTCTCAATTTCATATTCTCAATTTAAGCTAGGAT<br>CCCCTTTATTTTGCAATTTAACTCAAAACTTCAAAGGAATAA<br>ACCTAAAATTATGCTTTTTTAACTACGGGAAGCTTCAAATTTT<br>ACGATAAACCTTTTGCAAGCAAGTTTCTCATGATCTGGCAAT<br>ATTCTAGAAAGTGCAAACATGATTATGTAATATGAAAAATGC<br>TGTATATGAAACTCAAGGTATTTTTGATTTATTTTTTATACTTT<br>CATATTACATAGTCATCTTTACACTTTCGTAATGCTGCGTATA<br>TCAACATTAGCAAACTTTAATATGCAATAAATAGCTATTAGG<br>ATAACCTTTTCTTTTAATAACGTCACTATGCCTCTTTTAAATA<br>GAATTGACTAGCTTCGATGTTTACTCGGAGCTTATATTATTCC<br>ATTCTTCTCTCAATATGGCTTTCAACATAGCCTTTGAAGCTAT<br>ATTGTGTTGGCAGATTCTGCGTTCCAGCAAATCTCAAAAACA<br>CTTTATCGGGTAAGTCAGGCGATTCTGGAGTTGTTTATGTGTA<br>GGAGTAGATAACTATTTTAGCACGTCATATAAGAACTAGATA<br>TTCATAACTTGAACAGTGTGCTTGGGGACCTTGTCCAATTAG<br>AAATAATAACTGTTGTCTATCTTAAGTACTCTGCATTAAATTT<br>CGTTTTAAAATGTTTAAATAATCCATTTTATTCAATTGTGTCCT<br>GAATAAAGTGTAGTTGTCCCACGCCTCAGTTTTAGATACACC<br>CCCAAATAATAATACTTTAGCCACCGTGTTTTACAGTTGGCA<br>TTAAATATTCTTACTTAAGCTCTTTAACAGGTTTTCGGTACAA<br>AAGCTTGAATCCTTTAATGTCAAATACGCAGAATCTGCTCTA<br>ATCGGAAAATATTACCAGTTGCAGAGCTCATTTTGCTTATTA<br>ATATATTCTTTTGCAACGTCAATGCTTAAATCTATTATCTCAG

						HYLSGVKDDDYATI<br>RSQVYHSLGDFMVM<br>CPSKYFAEKVAEKN<br>NKIYRYYFNHRPSST<br>PYAEWMGVMHCD<br>DMQFIFGRPLTPLSN<br>YTRKEEELSFEMVKE<br>WTNFAKRGEPGET<br>WRKFSKSDPYVKVF<br>ELSENRTNYLKSSEE<br>NCEFFRPYFGF*	GAAATGTAAAACAGCTAAAAGGGATGTTACAACAGTTTCTCT<br>TCGAGCCGTCACATTTATCTTTTGTTCTTTCGCCTCTTGTGTTT<br>TACCTATTCCTCGACATATTTATGGCTATTTTTCAGTTCACAA<br>CTCTCTCGGATGGCGTAATCTTTTCTTGAAATTTCTTCGAATTT<br>TCTCTCGTTTTATATATATCAAAGCGGTAATGCGTTTTGCATTT<br>TGTTCCGTTTATAGTAACGTGCGCACCTAATACTTTCACCTGC<br>TTCATGAAGGACTGTATCAAATAGTCCGAAGTATGCTGCAGC<br>TTTCCTGTCTTACTGTGGATCAGAATCTTTTTTTGCAGCTTTAT<br>GGTATTCACTGCCTCAGTGACGAGACTTTGAAAATTTCAATC<br>ATGTTTTAAAATTCCGCATATATCAACACGAAAATGGTGTCT<br>CCAAGGATTTGGAAAATTGAATTTCTACTCGCAAGTTACCTT<br>GGTGTTGCAATTAAAATGAGGTCCATCACACAAGGATGGGA<br>TAAAGTCGAACACGTAAACTGTATAGCCTTCTACAAATTTGT<br>CTTTCGAGAAGTAAATACCTCTGTTTTGATTAAAGTTGATAA<br>ACAATTTGGTGACAAGCGCGAATATATCTTTTAGAATCAAAA<br>TTCGGTTATAAATGTTTGCTTGGCACTGAGTGACCATCTACTT<br>AAACACTTAAAAAGTTGACCATGATAATGATGAAAAAAAGG<br>AAGAGGGCAGGTTATGTTTCCTATTTACCGTGGCATTCTTCAG<br>GGAGGTAATAATCACTCCTTTGGACATCAGTCACAAAAATAC<br>ATTGTCTTTCATAAAAGAAAATGTTCCAACTGAAACTGAGTA<br>TGCTTTATACAATACTCTGTCGATGGTATATTTCGAGAGACTT<br>TTTTCGAGAACATTGACATGTCCCAAGGATACGCCGGGACAT<br>TCACTGTCCTAATTAACAAGGAGGCATGCTCAGATACGATTT<br>TATACTGGTCTCATTTTGGGGGGGGAGACTAAACAAAAACTG<br>TGTCTGTTGTGGCTCATTTTGATTCGCAGATCATCAAATTTAA<br>TAAAAGTAGATTTTGTTGAAAACATTCGCATCATCTCCAACG<br>TTCGATTTTTAGCTGTAAGTTCATTACGCTTCGTCAAACCTAC<br>ATTTCTATCGATTTCTTTCAACGGGTTCGTCGAATGTAATTTG

							CTGTCTTTGTAAAAGCATTCAACGGGGGAGTAAACTTTTCTT<br>GACATTTTTTCCATATATCCAAATTCTTCAATATAGGTTCTGT<br>AGGGATAAGTATTGCTATAGACGACGAGACGACTCGCTTCTT<br>AAGTTGATTTCCACTCTGAAAAAAAGAGCGTGTAAAATTAA<br>ATTCACGGGGTCGACGAGTTCATTTTCAGGTAAAGCGGATCT<br>ATCCGACTTCATAATTTTGGCTTTTACGTGCAGTTAGGAAGAC<br>GATAAGTCAATGTAATCTTCACCATTGTTACATTCCACGGGT<br>CCACCATCGCGTAGGTTGGACAGGGGAGGATATTCAATAAA<br>ATGTCCTCTTCGATTGCGGTCTCGCATAAGCGGGATCATGAA<br>CAAATGGAAATCAGATTTTGCGCAAGCAACACAGTATTGAT<br>AGATGCGTGTCGATATGCTTAACCGAAAATAACGCGTGATTC<br>CATTTTCTTGGTTCTTGGTGAACTAATGCGTTATTCCAACGCT<br>TTCTCTTTTAAATTATGACCAGAAGTAATAATGGACTTCATTT<br>TTCAAGCTACTTTGTCCGTTAATTGGCTTCCAGCTTATTTCGA<br>ATAGCGTTTAACAGACATTTTTAGAACATTCAATATGCCCAC<br>ACTGCTGTGAAATCATCGTTTGCCGACTACTTTTATGCCCAGT<br>TTTAAAAGGGGAACTGTCACTCGATAAAAAGGACAAAAGAA<br>TCTGCCAAAACCATAAACTGTCTGAAATGTATCCCCTTTATA<br>ATAATTCAACTCGCCACGCTTTGCATAATCCTCGAAGGATTTT<br>GGACAACATTGATGAGGCACATACATCACGATAGAAACTTT<br>TGCCAAAAGTACAGTTTTACGTACGAGCGCTTGAGTTCAGAT<br>GACACAATTTCGCGTGTATGCATTCTTATAACGACATTCAAT<br>ACTTTTCTATTCCGTTGGAAGTAATGGAGTCTTTCATATTGTG<br>CTCAAATCATTTCCCCGTCGCTTCCGGTCACGCTCAAGATGC<br>GCAACAAGTGCGCTAACACATCTCCCACAATATCAGGCTCTA<br>CAATGTCCGAATATATCAACAGTAATCTATAATGAGCGCACA<br>GATATGCCGCTTACTGACTTTCGACGTTCTTTTCTTCATCATA<br>ATCATCATCGGTACTCTCAAGTTCAAACATAGCATTTGCGCG

							AGTCCGTCGTACAGTATCACTTTAGCACCCTGCAAAACAAAAACAGAATCTGCCCCTTCCGGTAGACGCACTATGTTAAGAGTTCCCGAAATTGGAATCGATGCAAAACTTTGAGGGTAGACAGAATATAGTATATAGACAAAATATGTCCAGATCGAAATAGAGTTTTAGGTCCAGATCAAGCTTTCAAGTTGTGAGTCTAGCATCGTTCTATGGTGGGAAATGTGTTAAAAATATTTTTTAAATGCTGCACCCTTTACTGTGTTGTTTACATGCAGTGTATATATTCAGCTATATACATGCAGTGTATATATATTGTTTATATTCAGCTACTCTTCTCGGCTACAGCGGCAGATAATTCAATTAGCCATCTCGCAAGAAAGGAAATATGACTGTGTTCCTGACTAAAGTACACCTGGCATGTATGCTTGTACCTAAAAGGTCCGAATTCGAGAATTTTCTCGAAAGATAATTAAGGAGCGTCAGGGAAATTGTTGGAGGTCAGTGATGAAAGGGCGACGATTGTTTTTCTGACGTCATAGGTCTGGGTTTTTTTTTTTACAACTTCCGTGTCGAAATTGCACTATTTCCTGCAGAGATCAGAGCTATATATTAACAGCGTTTCAGGGTATTACTGTGCTATGAATATATTCCAGATTTTATGCGTGTTTTAACGTATATCACTTGCAAAGATGTATTTTTATTGTCTTAAAATACTACCATGATGTGGTGTACCTCGGAAAAATAAACTTACTTTTACATATATAAGAGTGTTTATTGCTGTAGCAGGAAAGTTTACCTTCACTTTATTTAATGCTATATAATTGTACCTTACAAAATTTTGTAGCTTATAATAAAGTGAATAATCTTAATATTTCAACGATATTTGGAATGCTGTCAGCACAATTCAAACAACTTTCAGTGAATTATAGTGATTTCTCTTTAACAAGAATGCGATTAAAATCCAAAATAAGGTCGGAAAAATTCACAGTTTTCTTCGCTAGATTTCAAGTAATTTGTACGGTTTTCAGATAGTTCAAAAACTTTGACGTATGGATCTGACTTAGAGAACTTCCGCCACGTTTCACCGGGTTCCCCTCTCTTAGCAAAATTCGTCCACTCTTTCACCATCTCAAAGCTTAGTTCTTCTTCCTTTCGGGTGTAGTTGGACAGAGGAGTTAGAGGACG

							TCCAAAGATAAATTGCATATCGTCACAATGCATAACCCCCAT<br>CCACTCTGCGTACGGAGTACTCGATGGTCTGTGATTGAAATA<br>GTATCGGTAGATTTTATTATTTTTTTCTGCAACTTTCTCAGCAA<br>AATATTTGCTTGGGCACATAACCATGAAATCCCCAAGAGAGT<br>GATAAACTTGACTTCGTATTGTAGCATAATCGTCGTCTTTAAC<br>GCCACTGAGGTAATGCTGGATTATTCCTTCCGGATCTGGAAA<br>TATTGTCCCGAAATAATCTCTCATGAGAGACTCCCCTAACGT<br>CTTATTAATGCGAGGATTTAATTCACCAAAAGGGCCTGTGAT<br>CTGTGTAAAAGTGTTAACAAATGGGAATGATCCCTCGTCTTG<br>AGTAACTCCTATCATAGAATCAACACCGTTAAAGTTTCCTTCT<br>CTGATAGCTTTTTGTGGGTCTACCGGTAGAAAATCATCCCCA<br>CTGATGGTAAAGAAAGTTATCACTGGGAAGACGGGAACTAA<br>GGAAAACGTAGTTCTCACCATATCTTTTGGAGCTTTACTTCTT<br>AGGCATTCGATCACATCATCAGGATTTTTCTTCAGCGTTATGT<br>TTTCATTTGCACATTCCATCTTCTCTGCGAGCTTTTGTCCTAAT<br>TTAAGATTCTCAGCCCTATGGTCATTCAGAAAATATGCTGGA<br>CAACCACTTTGCATTATTGCTCTCTGGAAGAGACCTTTCGAA<br>AGAGGAGAAACACACAGCAAACCTGTAGCTACGCCGCCGGC<br>GCTGTTGCCGAACACCGTAATTCTTTTATTGTCTCCGCCGAAT<br>GCAGCAATGTTTTCGTTCGTCCATTTAAGGGCTTCCAAAATGT<br>CATACAAGCCGACGTTTCCAGGAGCTTCTTCGGTTTCAGATG<br>TGAAGAATCCGAGCACACTTAACCGGTAGTTTATCGCAACA<br>ACTATTACATCTCCAATTGCAGTTAGAACACGTCCATCATAT<br>AGAGGCATCCTACCTGAACCGGTTGCAAAGCCGCCTGAGAA<br>CACCCAAAACATGACGGCTTTTTTCTCGCTACTATTGGAAATT<br>TCTGGTGCCCATATATTCAAATACAAGCAGTCTTCGCTTTGAC<br>CCTGAACTTTATCTTCCCACGGATAGTTGTCCGGAGAGTACT<br>GGTAACAAGCAGGCGGTAAGCTGTTTGCTATTACAGGAGAG

							GTCCAAGGTTTGACAGGAACTGTTCGCTTAAAGCGTAAATTC CCCACTGGAGGCTCAGCAAACGGAATTCCGAGAAAGACATT TAGATTTATGTCGTCATAAGTCATAACAGAACCAGTCACTTC ACCTGTAGGAGTATTAACTGTAACGCTGGATGCTAAGATAGT TCCTGGTAGCAGTAAAAATAGTAGATGCAAGCTCTGAAAAA CAGAATTTAAGATACTTATTGCATTATTTAAAAAAGAAATAT CAGATAACTTATTTTGAGGACAATTTTTCAACTATTATTTAAT ATCCCTCAGAACTGTTACTATCAGCGACATAACCACAATTTT AACAATTCCCTCCTTCTCTGAAGCAAATTTCCATTTTCCAGAA AGTGAGCCTCTTTTGTACTAAAATGGATTTTGTTACATCTCTA GTTCTCAGAGTGCACTTACCAAGAGATCTTTTGGGAGAGATT ATTACTTCCCTCATCTTAATTCTTTGTTTATGATAGAGTTTTAT CTGTATGTAATTTCATTTTAATGACTAAAAGTTTTGGATAATT GTAAGTCCAATAACGCAACGCCTTATTTCAGAAATGCTTACA AAAAAAACTGAGGTAGTCACTGTTGCACTTGCACAACCTGTT GACAGTTGCTCATTGGAATTCCATGGTGGCAATGGCATCTTC CTCAGTCTTCAATTATTCTTTTTGTAGATTACCAGGTTTTGATT AATTACATTTACGTAAGTAGGAAATTACATATTGTGAAAAAT GATTTTAGATATTTTTAATAGCAGATAGACGGAATATTTCCG ATGATATAAATAACTGAATATTTACGACACGCTTTTGCACTA CAAGCGTTAAAGTTGTGGCAGTAATAAGATTGCAACAACTTT TGAGTAGTTTTACAAAGCGGAATCTAAAAAGTTAAGGAATT

PhdEnzCho 15	W4VSJ0	1,08E-109	37,618	PF00135	63218<br><br>**58747.62**	MSSPSSNVIAGAQGL<br>GAAMCGKLQKNVH<br>KSLHLLFLLLPGTILA<br>**SSVTVNTPTGEVTG**<br>**SVMTYDDINLNVFL**<br>**GIPFAEPPVGNLRF**<br>**KRTVPVKPWTSPVI**<br>**ANSLPPACYQYSPD**<br>**NYPWEDKVQGQSE**<br>**DCLYLNIWAPEISN**<br>**SSEKKAVMFWVFS**<br>**GGFATGSGRMPLY**<br>**DGRVLTAIGDVIVV**<br>**AINYRLSVLGFFTSE**<br>**TEEAPGNVGLYDIL**<br>**EALKWTNENIAAFG**<br>**GDNKRITVFGNSA**<br>**GGVATGLLCVSPLS**<br>**KGLFQRAIMQSGC**<br>**PAYFLNDHRAENLK**<br>**LGQKLAEKMECAN**<br>**ENITLKKNPDDVIE**<br>**CLRSKAPKDMVRT**<br>**TFSLVPVFPVITFFTI**<br>**SGDDFLPVDPQKAI**<br>**REGNFNGVDSMIG**<br>**VTQDEGSFPFVNTF**<br>**TQITGPFGELNPRIN**	CGATAAACCTTTTGCAAGCAAGTTTCTCATGATCTGGCAATA<br>TTCTAGAAAGTGCAAACATGATTATGTAATATGAAAAATGCT<br>GTATATGAAACTCGAGGTATTTTTGATTTATTCTTTATACTTTC<br>ATATTACATATTCATCTTTACACTTTCATAACTATGCTTTCATG<br>AGTCTGCGTACATCAACATAACACATTAGAAAACTTTAATAT<br>GCAATTCATATTCTCAATTTAAGCTAGGATCCCCTTTATTTTG<br>CAATTTAACTCAAAACTTCAAAGGAATAAACCTAAAATTATG<br>CTTTTTTAACTACGGGAAGCTTCAAATTTTACGATAAACCTTT<br>TGCAAGCAAGTTTCTCATGATCTGGCAATATTCTAGAAAGTG<br>CAAACATGATTATGTAATATGAAAAATGCTGTATATGAAACT<br>CAAGGTATTTTTGATTTATTTTTTATACTTTCATATTACATAGT<br>CATCTTTACACTTTCGTAATTCTGCGTATATCAACTGAAAACA<br>TTAGCAAACTTTAATATGCAATAAATAGCTATTAGGATAACC<br>TTTTCTTTTAATAACGTCACTATGCCTCTTTTAAATAGAATTG<br>ACTAGCTTCGATGTTTACTCGGAGCTTATATTATTCCATTCTTC<br>TCTCAATATGGCTTTCAACATAGCCTTTGAAGCTATATTGTGT<br>TGGCAGATTCTGCGTTCCAGCAAATCTCAAAAACACTTTATC<br>GGGTAAGTCAGGCGATTCTGGAGTTGTTTATGTGTAGGAGTA<br>GATAACTATTTTAGCACGTCATATAAGAACTAGATATTCATA<br>ACTTGAACAGTGTGCTTGGGGACCTTGTCCAATTAGAAATAA<br>TAACTGTTGTCTATCTTAAGTACTCTGCATTAAATTTCGTTTTA<br>AAATGTTTAAATAATCCATTTTATTCAATTGTGTCCTGAATAA<br>AGTGTAGTTGTCCCACGCCTCAGTTTTAGATACACCCCCAAA<br>TAATAATACTTTAGCCACCGTGTTTTACAGTTGGCATTAAATA<br>TTCTTACTTAAGCTCTTTAACAGGTTTTCGGTACAAAAGCTTG<br>AATCCTTTAATGTCAAATACGCAGAATCTGCTCTAATCGGAA<br>AATATTACCAGTTGCAGAGCTCATTTTGCTTATTAATATATTC<br>TTTTGCAACGTCAATGCTTAAATCTATTATCTCAGGAAATGTA

						**KTLGESLMRDYFGT<br>IFPDPEGIIQHYLSG<br>VKDDDYATIRSQV<br>YHSLGDFMVMCPS<br>KYFAEKVAEKNNKI<br>YRYYFNHRPSSTPY<br>AEWMGVMHCDD<br>MQFIFGRPLTPLSN<br>YTRKEEELSFEMVK<br>EWTNFAKRGEPGET<br>WRKFSKSDPYVKVF<br>ELSENRTNYLKSSEE<br>NCEFFRPYFGF***	AAACAGCTAAAAGGGATGTTACAACAGTTTCTCTTCGAGCCG<br>TCACATTTATCTTTTGTTCTTTCGCCTCTTGTGTTTTACCTATTC<br>CTCGACATATTTATGGCTATTTTTCAGTTCACAACTCTCTCGG<br>ATGGCGTAATCTTTTCTTGAAATTTCTTCGAATTTTCTCTCGTT<br>TTATATATATCAAAGCGGTAATGCGTTTTGCATTTTGTTCCGT<br>TTATAGTAACGTGCGCACCTAATACTTTCACCTGCTTCATGAA<br>GGACTGTATCAAATAGTCCGAAGTATGCTGCAGCTTTCCTGT<br>CTTACTGTGGATCAGAATCTTTTTTTGCAGCTTTATGGTATTCA<br>CTGCCTCAGTGACGAGACTTTGAAAATTTCAATCATGTTTTAA<br>AATTCCGCATATATCAACACGAAAATGGTGTCTCCAAGGATT<br>TGGAAAATTGAATTTCTACTCGCAAGTTACCTTGGTGTTGCA<br>ATTAAAATGAGGTCCATCACACAAGGATGGGATAAAGTCGA<br>ACACGTAAACTGTATAGCCTTCTACAAATTTGTCTTTCGAGA<br>AGTAAATACCTCTGTTTTGATTAAAGTTGATAAACAATTTGGT<br>GACAAGCGCGAATATATCTTTTAGAATCAAAATTCGGTTATA<br>AATGTTTGCTTGGCACTGAGTGACCATCTACTTAAACACTTA<br>AAAAGTTGACCATGATAATGATGAAAAAAGGAAGAGGGCA<br>GGTTATGTTTCCTATTTACCGTGGCATTCTTCAGGGAGGTAAT<br>AATCACTCCTTTGGACATCAGTCACAAAAATACATTGTCTTTC<br>ATAAAAGAAAATGTTCCAACTGAAACTGAGTATGCTTTATAC<br>AATACTCTGTCGATGGTATATTTCGAGAGACTTTTTTCGAGAA<br>CATTGACATGTCCCAAGGATACGCCGGGACATTCACTGTCCT<br>AATTAACAAGGAGGCATGCTCAGATACGATTTTATACTGGTC<br>TCATTTTGGGGGGGGAGACTAAACAAAAACTGTGTCTGTTGT<br>GGCTCATTTTGATTCGCAGATCATCAAATTTAATAAAAGTAG<br>ATTTTGTTGAAAACATTCGCATCATCTCCAACGTTCGATTTTT<br>AGCTGTAAGTTCATTACGCTTCGTCAAACCTACATTTCTATCG<br>ATTTCTTTCAACGGGTTCGTCGAATGTAATTTGCTGTCTTTGTA

							AAAGCATTCAACGGGGGAGTAAACTTTTCTTGACATTTTTTCC<br>ATATATCCAAATTCTTCAATATAGGTTCTGTAGGGATAAGTA<br>TTGCTATAGACGACGAGACGACTCGCTTCTTAAGTTGATTTCC<br>ACTCTGAAAAAAAGAGCGTGTAAAATTAAATTCACGGGGTC<br>GACGAGTTCATTTTCAGGTAAAGCGGATCTATCCGACTTCAT<br>AATTTTGGCTTTTACGTGCAGTTAGGAAGACGATAAGTCAAT<br>GTAATCTTCACCATTGTTACATTCCACGGGTCCACCATCGCGT<br>AGGTTGGACAGGGGAGGATATTCAATAAAATGTCCTCTTCGA<br>TTGCGGTCTCGCATAAGCGGGATCATGAACAAATGGAAATC<br>AGATTTTGCGCAAGCAACACAGTATTGATAGATGCGTGTCGA<br>TATGCTTAACCGAAAATAACGCGTGATTCCATTTTCTTGGTTC<br>TTGGTGAACTAATGCGTTATTCCAACGCTTTCTCTTTTAAATT<br>ATGACCAGAAGTAATAATGGACTTCATTTTTCAAGCTACTTT<br>GTCCGTTAATTGGCTTCCAGCTTATTTCGAATAGCGTTTAACA<br>GACATTTTTAGAACATTCAATATGCCCACACTGCTGTGAAAT<br>CATCGTTTGCCGACTACTTTTATGCCCAGTTTTAAAAGGGGA<br>ACTGTCACTCGATAAAAAGGACAAAAGAATCTGCCAAAACC<br>ATAAACTGTCTGAAATGTATCCCCTTTATAATAATTCAACTCG<br>CCACGCTTTGCATAATCCTCGAAGGATTTTGGACAACATTGA<br>TGAGGCACATACATCACGATAGAAACTTTTGCCAAAAGTAC<br>AGTTTTACGTACGAGCGCTTGAGTTCAGATGACACAATTTCG<br>CGTGTATGCATTCTTATAACGACATTCAATACTTTTCTATTCC<br>GTTGGAAGTAATGGAGTCTTTCATATTGTGCTCAAATCATTTC<br>CCCGTCGCTTCCGGTCACGCTCAAGATGCGCAACAAGTGCGC<br>TAACACATCTCCCACAATATCAGGCTCTACAATGTCCGAATA<br>TATCAACAGTAATCTATAATGAGCGCACAGATATGCCGCTTA<br>CTGACTTTCGACGTTCTTTTCTTCATCATAATCATCATCGGTA<br>CTCTCAAGTTCAAACATAGCATTTGCGCGAGTCCGTCGTACA

							GTATCACTTTAGCACCCTGCAAAACAAAAACAGAATCTGCC<br>CCTTCCGGTAGACGCACTATGTTAAGAGTTCCCGAAATTGGA<br>ATCGATGCAAAACTTTGAGGGTAGACAGAATATAGTATATA<br>GACAAAATATGTCCAGATCGAAATAGAGTTTTAGGTCCAGAT<br>CAAGCTTTCAAGTTGTGAGTCTAGCATCGTTCTATGGTGGGA<br>AATGTGTTAAAAATATTTTTTAAATGCTGCACCCTTTACTGTG<br>TTGTTTACATGCAGTGTATATATTCAGCTATATACATGCAGTG<br>TATATATATTGTTTATATTCAGCTACTCTTCTCGGCTACAGCG<br>GCAGATAATTCAATTAGCCATCTCGCAAGAAAGGAAATATG<br>ACTGTGTTCCTGACTAAAGTACACCTGGCATGTATGCTTGTAC<br>CTAAAAGGTCCGAATTCGAGAATTTTCTCGAAAGATAATTAA<br>GGAGCGTCAGGGAAATTGTTGGAGGTCAGTGATGAAAGGGC<br>GACGATTGTTTTTCTGACGTCATAGGTCTGGGTTTTTTTTTTA<br>CAACTTCCGTGTCGAAATTGCACTATTTCCTGCAGAGATCAG<br>AGCTATATATTAACAGCGTTTCAGGGTATTACTGTGCTATGA<br>ATATATTCCAGATTTTATGCGTGTTTTAACGTATATCACTTGC<br>AAAGATGTATTTTTATTGTCTTAAAATACTACCATGATGTGGT<br>GTACCTCGGAAAAATAAACTTACTTTTACATATATAAGAGTG<br>TTTATTGCTGTAGCAGGAAAGTTTACCTTCACTTTATTTAATG<br>CTATATAATTGTACCTTACAATATTGTGTAGCTTATAATAAAG<br>TGAATAATCTTAATATTTCAACGATATTTGGAATGCTGTCAGC<br>ACAATTCAAACAACTTTCAGTGAATTATAGTGATTTCTCTTTA<br>ACAAGAATGCGATTAAAATCCAAAATAAGGTCGGAAAAATT<br>CACAGTTTTCTTCGCTAGATTTCAAGTAATTTGTACGGTTTTC<br>AGATAGTTCAAAAACTTTGACGTATGGATCTGACTTAGAGAA<br>CTTCCGCCACGTTTCACCGGGTTCCCCTCTCTTAGCAAAATTC<br>GTCCACTCTTTCACCATCTCAAAGCTTAGTTCTTCTTCCTTTCG<br>GGTGTAGTTGGACAGAGGAGTTAGAGGACGTCCAAAGATAA

							ATTGCATATCGTCACAATGCATAACCCCCATCCACTCTGCGT<br>ACGGAGTACTCGATGGTCTGTGATTGAAATAGTATCGGTAGA<br>TTTTATTATTTTTTTCTGCAACTTTCTCAGCAAAATATTTGCTT<br>GGGCACATAACCATGAAATCCCCAAGAGAGTGATAAACTTG<br>ACTTCGTATTGTAGCATAATCGTCGTCTTTAACGCCACTGAGG<br>TAATGCTGGATTATTCCTTCCGGATCTGGAAATATTGTCCCGA<br>AATAATCTCTCATGAGAGACTCCCCTAACGTCTTATTAATGC<br>GAGGATTTAATTCACCAAAAGGGCCTGTGATCTGTGTAAAAG<br>TGTTAACAAATGGGAATGATCCCTCGTCTTGAGTAACTCCTA<br>TCATAGAATCAACACCGTTAAAGTTTCCTTCTCTGATAGCTTT<br>TTGTGGGTCTACCGGTAGAAAATCATCCCCACTGATGGTAAA<br>GAAAGTTATCACTGGGAAGACGGGAACTAAGGAAAACGTA<br>GTTCTCACCATATCTTTTGGAGCTTTACTTCTTAGGCATTCGAT<br>CACATCATCAGGATTTTTCTTCAGCGTTATGTTTTCATTTGCAC<br>ATTCCATCTTCTCTGCGAGCTTTTGTCCTAATTTAAGATTCTCA<br>GCCCTATGGTCATTCAGAAAATATGCTGGACAACCACTTTGC<br>ATTATTGCTCTCTGGAAGAGACCTTTCGAAAGAGGAGAAAC<br>ACACAGCAAACCTGTAGCTACGCCGCCGGCGCTGTTGCCGA<br>ACACCGTAATTCTTTTATTGTCTCCGCCGAATGCAGCAATGTT<br>TTCGTTCGTCCATTTAAGGGCTTCCAAAATGTCATACAAGCC<br>GACGTTTCCAGGAGCTTCTTCGGTTTCAGATGTGAAGAATCC<br>GAGCACACTTAACCGGTAGTTTATCGCAACAACTATTACATC<br>TCCAATTGCAGTTAGAACACGTCCATCATATAGAGGCATCCT<br>ACCTGAACCGGTTGCAAAGCCGCCTGAGAACACCCAAAACA<br>TGACGGCTTTTTTCTCGCTACTATTGGAAATTTCTGGTGCCCA<br>TATATTCAAATACAAGCAGTCTTCGCTTTGACCCTGAACTTTA<br>TCTTCCCACGGATAGTTGTCCGGAGAGTACTGGTAACAAGCA<br>GGCGGTAAGCTGTTTGCTATTACAGGAGAGGTCCAAGGTTTG

							ACAGGAACTGTTCGCTTAAAGCGTAAATTCCCCACTGGAGGC<br>TCAGCAAACGGAATTCCGAGAAAGACATTTAGATTTATGTCG<br>TCATAAGTCATAACAGAACCAGTCACTTCACCTGTAGGAGTA<br>TTAACTGTAACGCTGGATGCTAAGATAGTTCCTGGTAGCAGT<br>AAAAATAGTAGATGCAAGCTCTTATGAACGTTTTTCTGGAGC<br>TTTCCGCACATAGCGGCTCCTAAACCTTGGGCTCCAGCAATA<br>ACGTTAGACGAGGGAGACGACATGATGCTGCTGCCTAGATC<br>TCCAGTGTGCTGTGTCGTACACAAATGCGCCTATTTCCTGCTA<br>GTGTAGGTCTTCATCCGAATTATGCCCAAACTGGTTGTAGTTT<br>TACTGTGGATAAGAAGCTTTCTTAACCTAATTTGTTCTCCACA<br>GGCTCCTTGAGAGTACGATGTTAACACATTGATGATGGGAGA<br>CGCTAATTTGCGTGTTGAATACTTCATGCGGTATCCTATAAAA<br>ATGTTAACTACTATTAAATAAAACAGTAAAAAAAGCAATGG<br>ATGCTATGAACAAAATGTTACGAATGTGTTTTTACATGAGAA<br>TGCTTGAATAATAAGAAACTCCGCCATCAATATTGAAAAAAT<br>TTTTTTGAAAACCTTTTATTTAGTAATGAGAAGGCAATAAGCT<br>ACGGAAGATCAGTTTGTGGAATATAGAGCAGAAATTTATGC<br>AGATTTGTTGTCAGATGTACAAAATGATGCCGAATGTGAAAA<br>TAATATTATTCATAGTTGCATTGAAGTTGATTTTATTAAGAAT<br>AGAAGCACTCGTCCTTTGCCGAGATGTCATTTCACTAATGTCT<br>GGAAAAGTATTCATCTTTTTCAGGCTTTAATGAAAAAGATGA<br>ATACATATCTTTATCTTACGAAACAAGTTCATGGACTAAGCA<br>AGACAAAATTACTGTTCTGGAAGATTTTATAGGAAACCCTGG<br>AGTAAAGCAATTTCCATCCGATCATACTAGCATTAATGAAGT<br>CGTAGGTTTATTCTTTGGAAATGATTTCTTTGAAATGCTTTAC<br>CGTGAAAATGAATACAAATGAAATAAAAACTAAAGTGGGTT<br>GATGTAGAAATAAAGGAAATGAAACGATTTTTTTATCTTGTT<br>TTACTTATGGTACATGTAAAAAAGGATCGCATTAGAGATTAT

							TGGTCGACTGATTCATACATAGAAACTCCGATTTTTTTCAAAAGTAATGAGCCGGAACAGATTTGAACAAATTTTACAGTCGCTCCATTTCCGTAATAATGATTTTCAAACTAATTCGACAGATAGACTCTTCAAGTTAAGACCAATATTACATTACTGTGTAAAAAAATTTCAACTCATACATAAGCCAAATCAAGAAGTATCTTTTGGCAGAACATGGAGAACTGCTCCGATACATGATTGGTATGCCGGTTTTCGGCCTGGAGCATCCGTTGTAAAAGCAAGTAATAGAAAAGCACAAACTGCCTTGGCAAGGTTCCGCAGCGGCCATTTTCGGCCGATGAGGTTTTCTGCTGACAAGAGGGTCTACCCTTTAAGTTCAAAATGCAGCTTGACTGAGACGACTCCCTCTCATGTTCTATTGTGCATTGGTTGCACCAAGAACCATCTGTTTTATAACTCTGGGGCGTTTCTGGATCAGTTGTG

PhdEnzCho 16	W4VSJ0	1,20E-109	37,618	PF00135	63354,11	LKNCPQNKLSDISFL NNAISILNSVFQSLH LLFLLLPGTILASSVT VNTPTGEVTGSVMT YDDINLNVFLGIPFA EPPVGNLRFKRTVPV KPWTSPVIANSLPPA CYQYSPDNYPWEDK VQGQSEDCLYLNIW APEISNSSEKKAVMF WVFSGGFATGSGRM PLYDGRVLTAIGDVI VVAINYRLSVLGFFT SETEEAPGNVGLYDI LEALKWTNENIAAF GGDNKRITVFGNSA GGVATGLLCVSPLSK GLFQRAIMQSGCPA YFLNDHRAENLKLG QKLAEKMECANENI TLKKNPDDVIECLRS KAPKDMVRTTFSLV PVFPVITFFTISGDDF LPVDPQKAIREGNF NGVDSMIGVTQDEG SFPFVNTFTQITGPFG ELNPRINKTLGESLM RDYFGTIFPDPEGIIQ	CGATAAACCTTTTGCAAGCAAGTTTCTCATGATCTGGCAATA TTCTAGAAAGTGCAAACATGATTATGTAATATGAAAAATGCT GTATATGAAACTCGAGGTATTTTTGATTTATTCTTTATACTTTC ATATTACATATTCATCTTTACACTTTCATAACTATGCTTTCATG AGTCTGCGTACATCAACATAACACATTAGAAAACTTTAATAT CAATTCATATTCTCAATTTCATATTCTCAATTTAAGCTAGGAT CCCCTTTATTTTGCAATTTAACTCAAAACTTCAAAGGAATAA ACCTAAAATTATGCTTTTTTAACTACGGGAAGCTTCAAATTTT ACGATAAACCTTTTGCAAGCAAGTTTCTCATGATCTGGCAAT ATTCTAGAAAGTGCAAACATGATTATGTAATATGAAAAATGC TGTATATGAAACTCAAGGTATTTTTGATTTATTTTTTATACTTT CATATTACATAGTCATCTTTACACTTTCGTAATTCTGCGTATA TCAACTGAAAACATTAGCAAACTTTAATATGCAATAAATAGC TATTAGGATAACCTTTTCTTTTAATAACGTCACTATGCCTCTTT TAAATAGAATTGACTAGCTTCGATGTTTACTCGGAGCTTATAT TATTCCATTCTTCTCTCAATATGGCTTTCAACATAGCCTTTGA AGCTATATTGTGTTGGCAGATTCTGCGTTCCAGCAAATCTCA AAAACACTTTATCGGGTAAGTCAGGCGATTCTGGAGTTGTTT ATGTGTAGGAGTAGATAACTATTTTAGCACGTCATATAAGAA CTAGATATTCATAACTTGAACAGTGTGCTTGGGGACCTTGTC CAATTAGAAATAATAACTGTTGTCTATCTTAAGTACTCTGCAT TAAATTTCGTTTTAAAATGTTTAAATAATCCATTTTATTCAATT GTGTCCTGAATAAAGTGTAGTTGTCCCACGCCTCAGTTTTAG ATACACCCCCAAATAATAATACTTTAGCCACCGTGTTTTACA GTTGGCATTAAATATTCTTACTTAAGCTCTTTAACAGGTTTTC GGTACAAAAGCTTGAATCCTTTAATGTCAAATACGCAGAATC TGCTCTAATCGGAAAATATTACCAGTTGCAGAGCTCATTTTG CTTATTAATATATTCTTTTGCAACGTCAATGCTTAAATCTATT

						HYLSGVKDDDYATI<br>RSQVYHSLGDFMVM<br>CPSKYFAEKVAEKN<br>NKIYRYYFNHRPSST<br>PYAEWMGVMHCD<br>DMQFIFGRPLTPLSN<br>YTRKEEELSFEMVKE<br>WTNFAKRGEPGET<br>WRKFSKSDPYVKVF<br>ELSENRTNYLKSSEE<br>NCEFFRPYFGF*	ATCTCAGGAAATGTAAAACAGCTAAAAGGGATGTTACAACA<br>GTTTCTCTTCGAGCCGTCACATTTATCTTTTGTTCTTTCGCCTC<br>TTGTGTTTTACCTATTCCTCGACATATTTATGGCTATTTTTCAG<br>TTCACAACTCTCTCGGATGGCGTAATCTTTTCTTGAAATTTCTT<br>CGAATTTTCTCTCGTTTTATATATATCAAAGCGGTAATGCGTT<br>TTGCATTTTGTTCCGTTTATAGTAACGTGCGCACCTAATACTT<br>TCACCTGCTTCATGAAGGACTGTATCAAATAGTCCGAAGTAT<br>GCTGCAGCTTTCCTGTCTTACTGTGGATCAGAATCTTTTTTTGC<br>AGCTTTATGGTATTCACTGCCTCAGTGACGAGACTTTGAAAA<br>TTTCAATCATGTTTTAAAATTCCGCATATATCAACACGAAAA<br>TGGTGTCTCCAAGGATTTGGAAAATTGAATTTCTACTCGCAA<br>GTTACCTTGGTGTTGCAATTAAAATGAGGTCCATCACACAAG<br>GATGGGATAAAGTCGAACACGTAAACTGTATAGCCTTCTACA<br>AATTTGTCTTTCGAGAAGTAAATACCTCTGTTTTGATTAAAGT<br>TGATAAACAATTTGGTGACAAGCGCGAATATATCTTTTAGAA<br>TCAAAATTCGGTTATAAATGTTTGCTTGGCACTGAGTGACCA<br>TCTACTTAAACACTTAAAAAGTTGACCATGATAATGATGAAA<br>AAAAGGAAGAGGGCAGGTTATGTTTCCTATTTACCGTGGCAT<br>TCTTCAGGGAGGTAATAATCACTCCTTTGGACATCAGTCACA<br>AAAATACATTGTCTTTCATAAAAGAAAATGTTCCAACTGAAA<br>CTGAGTATGCTTTATACAATACTCTGTCGATGGTATATTTCGA<br>GAGACTTTTTTCGAGAACATTGACATGTCCCAAGGATACGCC<br>GGGACATTCACTGTCCTAATTAACAAGGAGGCATGCTCAGAT<br>ACGATTTTATACTGGTCTCATTTTGGGGGGGGAGACTAAACA<br>AAAACTGTGTCTGTTGTGGCTCATTTTGATTCGCAGATCATCA<br>AATTTAATAAAAGTAGATTTTGTTGAAAACATTCGCATCATC<br>TCCAACGTTCGATTTTTAGCTGTAAGTTCATTACGCTTCGTCA<br>AACCTACATTTCTATCGATTTCTTTCAACGGGTTCGTCGAATG

							TAATTTGCTGTCTTTGTAAAAGCATTCAACGGGGGAGTAAAC TTTTCTTGACATTTTTTCCATATATCCAAATTCTTCAATATAGG TTCTGTAGGGATAAGTATTGCTATAGACGACGAGACGACTCG CTTCTTAAGTTGATTTCCACTCTGAAAAAAAGAGCGTGTAAA ATTAAATTCACGGGGTCGACGAGTTCATTTTCAGGTAAAGCG GATCTATCCGACTTCATAATTTTGGCTTTTACGTGCAGTTAGG AAGACGATAAGTCAATGTAATCTTCACCATTGTTACATTCCA CGGGTCCACCATCGCGTAGGTTGGACAGGGGAGGATATTCA ATAAAATGTCCTCTTCGATTGCGGTCTCGCATAAGCGGGATC ATGAACAAATGGAAATCAGATTTTGCGCAAGCAACACAGTA TTGATAGATGCGTGTCGATATGCTTAACCGAAAATAACGCGT GATTCCATTTTCTTGGTTCTTGGTGAACTAATGCGTTATTCCA ACGCTTTCTCTTTTAAATTATGACCAGAAGTAATAATGGACTT CATTTTTCAAGCTACTTTGTCCGTTAATTGGCTTCCAGCTTATT TCGAATAGCGTTTAACAGACTTTTTAGAACATTCAATATGCC CACACTGCTGTGAAATCATCGTTTGCCGACTACTTTTATGCCC AGTTTTAAAAGGGGAACTGTCACTCGATAAAAAGGACAAAA GAATCTGCCAAAACCATAAACTGTCTGAAATGTATCCCCTTT ATAATAATTCAACTCGCCACGCTTTGCATAATCCTCGAAGGA TTTTGGACAACATTGATGAGGCACATACATCACGATAGAAA CTTTTGCCAAAAGTACAGTTTTACGTACGAGCGCTTGAGTTC AGATGACACAATTTCGCGTGTATGCATTCTTATAACGACATT CAATACTTTTCTATTCCGTTGGAAGTAATGGAGTCTTTCATAT TGTGCTCAAATCATTTCCCCGTCGCTTCCGGTCACGCTCAAGA TGCGCAACAAGTGCGCTAACACATCTCCCACAATATCAGGCT CTACAATGTCCGAATATATCAACAGTAATCTATAATGAGCGC ACAGATATGCCGCTTACTGACTTTCGACGTTCTTTTCTTCATC ATAATCATCATCGGTACTCTCAAGTTCAAACATAGCATTTGC

							GCGAGTCCGTCGTACAGTATCACTTTAGCACCCTGCAAAACA AAAACAGAATCTGCCCCTTCCGGTAGACGCACTATGTTAAGA GTTCCCGAAATTGGAATCGATGCAAAACTTTGAGGGTAGAC AGAATATAGTATATAGACAAAATATGTCCAGATCGAAATAG AGTTTTAGGTCCAGATCAAGCTTTCAAGTTGTGAGTCTAGCA TCGTTCTATGGTGGGAAATGTGTTAAAAATATTTTTTAAATGC TGCACCCTTTACTGTGTTGTTTACATGCAGTGTATATATTCAG CTATATACATGCAGTGTATATATATTGTTTATATTCAGCTACT CTTCTCGGCTACAGCGGCAGATAATTCAATTAGCCATCTCGC AAGAAAGGAAATATGACTGTGTTCCTGACTAAAGTACACCT GGCATGTATGCTTGTACCTAAAAGGTCCGAATTCGAGAATTT TCTCGAAAGATAATTAAGGAGCGTCAGGGAAATTGTTGGAG GTCAGTGATGAAAGGGCGACGATTGTTTTTCTGACGTCATAG GTCTGGGTTTTTTTTTTACAACTTCCGTGTCGAAATTGCACTA TTTCCTGCAGAGATCAGAGCTATATATTAACAGCGTTTCAGG GTATTACTGTGCTATGAATATATTCCAGATTTTATGCGTGTTTT AACGTATATCACTTGCAAAGATGTATTTTTATTGTCTTAAAAT ACTACCATGATGTGGTGTACCTCGGAAAAATAAACTTACTTT TACATATATAAGAGTGTTTATTGCTGTAGCAGGAAAGTTTAC CTTCACTTTATTTAATGCTATATAATTGTACCTTACAAAATTTT GTAGCTTATAATAAAGTGAATAATCTTAATATTTCAACGATA TTTGGAATGCTGTCAGCACAATTCAAACAACTTTCAGTGAAT TATAGTGATTTCTCTTTAACAAGAATGCGATTAAAATCCAAA ATAAGGTCGGAAAAATTCACAGTTTTCTTCGCTAGATTTCAA GTAATTTGTACGGTTTTCAGATAGTTCAAAAACTTTGACGTAT GGATCTGACTTAGAGAACTTCCGCCACGTTTCACCGGGTTCC CCTCTCTTAGCAAAATTCGTCCACTCTTTCACCATCTCAAAGC TTAGTTCTTCTTCCTTTCGGGTGTAGTTGGACAGAGGAGTTAG

							AGGACGTCCAAAGATAAATTGCATATCGTCACAATGCATAA<br>CCCCCATCCACTCTGCGTACGGAGTACTCGATGGTCTGTGAT<br>TGAAATAGTATCGGTAGATTTTATTATTTTTTTCTGCAACTTTC<br>TCAGCAAAATATTTGCTTGGGCACATAACCATGAAATCCCCA<br>AGAGAGTGATAAACTTGACTTCGTATTGTAGCATAATCGTCG<br>TCTTTAACGCCACTGAGGTAATGCTGGATTATTCCTTCCGGAT<br>CTGGAAATATTGTCCCGAAATAATCTCTCATGAGAGACTCCC<br>CTAACGTCTTATTAATGCGAGGATTTAATTCACCAAAAGGGC<br>CTGTGATCTGTGTAAAAGTGTTAACAAATGGGAATGATCCCT<br>CGTCTTGAGTAACTCCTATCATAGAATCAACACCGTTAAAGT<br>TTCCTTCTCTGATAGCTTTTTGTGGGTCTACCGGTAGAAAATC<br>ATCCCCACTGATGGTAAAGAAAGTTATCACTGGGAAGACGG<br>GAACTAAGGAAAACGTAGTTCTCACCATATCTTTTGGAGCTT<br>TACTTCTTAGGCATTCGATCACATCATCAGGATTTTTCTTCAG<br>CGTTATGTTTTCATTTGCACATTCCATCTTCTCTGCGAGCTTTT<br>GTCCTAATTTAAGATTCTCAGCCCTATGGTCATTCAGAAAAT<br>ATGCTGGACAACCACTTTGCATTATTGCTCTCTGGAAGAGAC<br>CTTTCGAAAGAGGAGAAACACACAGCAAACCTGTAGCTACG<br>CCGCCGGCGCTGTTGCCGAACACCGTAATTCTTTTATTGTCTC<br>CGCCGAATGCAGCAATGTTTTCGTTCGTCCATTTAAGGGCTTC<br>CAAAATGTCATACAAGCCGACGTTTCCAGGAGCTTCTTCGGT<br>TTCAGATGTGAAGAATCCGAGCACACTTAACCGGTAGTTTAT<br>CGCAACAACTATTACATCTCCAATTGCAGTTAGAACACGTCC<br>ATCATATAGAGGCATCCTACCTGAACCGGTTGCAAAGCCGCC<br>TGAGAACACCCAAAACATGACGGCTTTTTTCTCGCTACTATT<br>GGAAATTTCTGGTGCCCATATATTCAAATACAAGCAGTCTTC<br>GCTTTGACCCTGAACTTTATCTTCCCACGGATAGTTGTCCGGA<br>GAGTACTGGTAACAAGCAGGCGGTAAGCTGTTTGCTATTACA

							GGAGAGGTCCAAGGTTTGACAGGAACTGTTCGCTTAAAGCGTAAATTCCCCACTGGAGGCTCAGCAAACGGAATTCCGAGAAAGACATTTAGATTTATGTCGTCATAAGTCATAACAGAACCAGTCACTTCACCTGTAGGAGTATTAACTGTAACGCTGGATGCTAAGATAGTTCCTGGTAGCAGTAAAAATAGTAGATGCAAGCTCTGAAAAACAGAATTTAAGATACTTATTGCATTATTTAAAAAAGAAATATCAGATAACTTATTTTGAGGACAATTTTTCAACTATTATTTAATATCCCTCAGAACTGTTACTATCAGCGACATAACCACAATTTTAACAATTCCCTCCTTCTCTGAAGCAAATTTCCATTTTCCAGAAAGTGAGCCTCTTTTGTACTAAAATGGATTTTGTTACATCTCTAGTTCTCAGAGTGCACTTACCAAGAGATCTTTTGGGAGAGATTATTACTTCCCTCATCTTAATTCTTTGTTTATGATAGAGTTTTATCTGTATGTAATTTCATTTTAATGACTAAAAGTTTTGGATAATTGTAAGTCCAATAACGCAACGCCTTATTTCAGAAATGCTTACAAAAAAAACTGAGGTAGTCACTGTTGCACTTGCACAACCTGTTGACAGTTGCTCATTGGAATTCCATGGTGGCAATGGCATCTTCCTCAGTCTTCAATTATTCTTTTTGTAGATTACCAGGTTTTGATTAATTACATTTACGTAAGTAGGAAATTACATATTGTGAAAAATGATTTTAGATATTTTTAATAGCAGATAGACGGAATATTTCCGATGATATAAATAACTGAATATTTACGACACGCTTTTGCACTACAAGCGTTAAAGTTGTGGCAGTAATAAGATTGCAACAACTTTTGAGTAGTTTTACAAAGCGGAATCTAAAAAGTTAAGGAATT

PhdEnzCho 17	W4VSJ0	1,20E-109	37,618	PF00135	63354,11	LKNCPQNKLSDISFL NNAISILNSVFQSLH LLFLLLPGTILASSVT VNTPTGEVTGSVMT YDDINLNVFLGIPFA EPPVGNLRFKRTVPV KPWTSPVIANSLPPA CYQYSPDNYPWEDK VQGQSEDCLYLNIW APEISNSSEKKAVMF WVFSGGFATGSGRM PLYDGRVLTAIGDVI VVAINYRLSVLGFFT SETEEAPGNVGLYDI LEALKWTNENIAAF GGDNKRITVFGNSA GGVATGLLCVSPLSK GLFQRAIMQSGCPA YFLNDHRAENLKLG QKLAEKMECANENI TLKKNPDDVIECLRS KAPKDMVRTTFSLV PVFPVITFFTISGDDF LPVDPQKAIREGNF NGVDSMIGVTQDEG SFPFVNTFTQITGPFG ELNPRINKTLGESLM RDYFGTIFPDPEGIIQ	CGATAAACCTTTTGCAAGCAAGTTTCTCATGATCTGGCAATA TTCTAGAAAGTGCAAACATGATTATGTAATATGAAAAATGCT GTATATGAAACTCGAGGTATTTTTGATTTATTCTTTATACTTTC ATATTACATATTCATCTTTACACTTTCATAACTATGCTTTCATG AGTCTGCGTACATCAACATAACACATTAGAAAACTTTAATAT GCAATTCATATTCTCAATTTAAGCTAGGATCCCCTTTATTTTG CAATTTAACTCAAAACTTCAAAGGAATAAACCTAAAATTATG CTTTTTTAACTACGGGAAGCTTCAAATTTTACGATAAACCTTT TGCAAGCAAGTTTCTCATGATCTGGCAATATTCTAGAAAGTG CAAACATGATTATGTAATATGAAAAATGCTGTATATGAAACT CAAGGTATTTTTGATTTATTTTTTATACTTTCATATTACATAGT CATCTTTACACTTTCGTAATTCTGCGTATATCAACTGAAAACA TTAGCAAACTTTAATATGCAATAAATAGCTATTAGGATAACC TTTTCTTTTAATAACGTCACTATGCCTCTTTTAAATAGAATTG ACTAGCTTCGATGTTTACTCGGAGCTTATATTATTCCATTCTTC TCTCAATATGGCTTTCAACATAGCCTTTGAAGCTATATTGTGT TGGCAGATTCTGCGTTCCAGCAAATCTCAAAAACACTTTATC GGGTAAGTCAGGCGATTCTGGAGTTGTTTATGTGTAGGAGTA GATAACTATTTTAGCACGTCATATAAGAACTAGATATTCATA ACTTGAACAGTGTGCTTGGGGACCTTGTCCAATTAGAAATAA TAACTGTTGTCTATCTTAAGTACTCTGCATTAAATTTCGTTTTA AAATGTTTAAATAATCCATTTTATTCAATTGTGTCCTGAATAA AGTGTAGTTGTCCCACGCCTCAGTTTTAGATACACCCCCAAA TAATAATACTTTAGCCACCGTGTTTTACAGTTGGCATTAAATA TTCTTACTTAAGCTCTTTAACAGGTTTTCGGTACAAAAGCTTG AATCCTTTAATGTCAAATACGCAGAATCTGCTCTAATCGGAA AATATTACCAGTTGCAGAGCTCATTTTGCTTATTAATATATTC TTTTGCAACGTCAATGCTTAAATCTATTATCTCAGGAAATGTA

						HYLSGVKDDDYATI<br>RSQVYHSLGDFMVM<br>CPSKYFAEKVAEKN<br>NKIYRYYFNHRPSST<br>PYAEWMGVMHCD<br>DMQFIFGRPLTPLSN<br>YTRKEEELSFEMVKE<br>WTNFAKRGEPGET<br>WRKFSKSDPYVKVF<br>ELSENRTNYLKSSEE<br>NCEFFRPYFGF*	AAACAGCTAAAAGGGATGTTACAACAGTTTCTCTTCGAGCCG<br>TCACATTTATCTTTTGTTCTTTCGCCTCTTGTGTTTTACCTATTC<br>CTCGACATATTTATGGCTATTTTTCAGTTCACAACTCTCTCGG<br>ATGGCGTAATCTTTTCTTGAAATTTCTTCGAATTTTCTCTCGTT<br>TTATATATATCAAAGCGGTAATGCGTTTTGCATTTTGTTCCGT<br>TTATAGTAACGTGCGCACCTAATACTTTCACCTGCTTCATGAA<br>GGACTGTATCAAATAGTCCGAAGTATGCTGCAGCTTTCCTGT<br>CTTACTGTGGATCAGAATCTTTTTTTGCAGCTTTATGGTATTCA<br>CTGCCTCAGTGACGAGACTTTGAAAATTTCAATCATGTTTTAA<br>AATTCCGCATATATCAACACGAAAATGGTGTCTCCAAGGATT<br>TGGAAAATTGAATTTCTACTCGCAAGTTACCTTGGTGTTGCA<br>ATTAAAATGAGGTCCATCACACAAGGATGGGATAAAGTCGA<br>ACACGTAAACTGTATAGCCTTCTACAAATTTGTCTTTCGAGA<br>AGTAAATACCTCTGTTTTGATTAAAGTTGATAAACAATTTGGT<br>GACAAGCGCGAATATATCTTTTAGAATCAAAATTCGGTTATA<br>AATGTTTGCTTGGCACTGAGTGACCATCTACTTAAACACTTA<br>AAAAGTTGACCATGATAATGATGAAAAAAAGGAAGAGGGC<br>AGGTTATGTTTCCTATTTACCGTGGCATTCTTCAGGGAGGTAA<br>TAATCACTCCTTTGGACATCAGTCACAAAAATACATTGTCTTT<br>CATAAAAGAAAATGTTCCAACTGAAACTGAGTATGCTTTATA<br>CAATACTCTGTCGATGGTATATTTCGAGAGACTTTTTTCGAGA<br>ACATTGACATGTCCCAAGGATACGCCGGGACATTCACTGTCC<br>TAATTAACAAGGAGGCATGCTCAGATACGATTTTATACTGGT<br>CTCATTTTGGGGGGGGAGACTAAACAAAAACTGTGTCTGTTG<br>TGGCTCATTTTGATTCGCAGATCATCAAATTTAATAAAAGTA<br>GATTTTGTTGAAAACATTCGCATCATCTCCAACGTTCGATTTT<br>TAGCTGTAAGTTCATTACGCTTCGTCAAACCTACATTTCTATC<br>GATTTCTTTCAACGGGTTCGTCGAATGTAATTTGCTGTCTTTGT

							AAAAGCATTCAACGGGGGAGTAAACTTTTCTTGACATTTTTT<br>CCATATATCCAAATTCTTCAATATAGGTTCTGTAGGGATAAG<br>TATTGCTATAGACGACGAGACGACTCGCTTCTTAAGTTGATTT<br>CCACTCTGAAAAAAAGAGCGTGTAAAATTAAATTCACGGGG<br>TCGACGAGTTCATTTTCAGGTAAAGCGGATCTATCCGACTTC<br>ATAATTTTGGCTTTTACGTGCAGTTAGGAAGACGATAAGTCA<br>ATGTAATCTTCACCATTGTTACATTCCACGGGTCCACCATCGC<br>GTAGGTTGGACAGGGGAGGATATTCAATAAAATGTCCTCTTC<br>GATTGCGGTCTCGCATAAGCGGGATCATGAACAAATGGAAA<br>TCAGATTTTGCGCAAGCAACACAGTATTGATAGATGCGTGTC<br>GATATGCTTAACCGAAAATAACGCGTGATTCCATTTTCTTGGT<br>TCTTGGTGAACTAATGCGTTATTCCAACGCTTTCTCTTTTAAA<br>TTATGACCAGAAGTAATAATGGACTTCATTTTTCAAGCTACTT<br>TGTCCGTTAATTGGCTTCCAGCTTATTTCGAATAGCGTTTAAC<br>AGACATTTTTAGAACATTCAATATGCCCACACTGCTGTGAAA<br>TCATCGTTTGCCGACTACTTTTATGCCCAGTTTTAAAAGGGGA<br>ACTGTCACTCGATAAAAAGGACAAAAGAATCTGCCAAAACC<br>ATAAACTGTCTGAAATGTATCCCCTTTATAATAATTCAACTCG<br>CCACGCTTTGCATAATCCTCGAAGGATTTTGGACAACATTGA<br>TGAGGCACATACATCACGATAGAAACTTTTGCCAAAAGTAC<br>AGTTTTACGTACGAGCGCTTGAGTTCAGATGACACAATTTCG<br>CGTGTATGCATTCTTATAACGACATTCAATACTTTTCTATTCC<br>GTTGGAAGTAATGGAGTCTTTCATATTGTGCTCAAATCATTTC<br>CCCGTCGCTTCCGGTCACGCTCAAGATGCGCAACAAGTGCGC<br>TAACACATCTCCCACAATATCAGGCTCTACAATGTCCGAATA<br>TACCAACAGTAATCTATAATGAGCACACAGATATGCCGCTTA<br>CTGACTTTCGACGTTCTTTTCTTCATCATAATCATCATCGGTA<br>CTCTCAAGTTCAAACATAGCATTTGCGCGAGTCCGTCGTACA

							GTATCACTTTAGCACCCTGCAAAACAAAAACAGAATCTGCC<br>CCTTCCGGTAGACGCACTATGTTAAGAGTTCCCGAAATTGGA<br>ATCGATGCAAAACTTTGAGGGTAGACAGAATATAGTATATA<br>GACAAAATATGTCCAGATCGAAATAGAGTTTTAGGTCCAGAT<br>CAAGCTTTCAAGTTGTGAGTCTAGCATCGTTCTATGGTGGGA<br>AATGTGTTAAAAATATTTTTTAAATGCTGCACCCTTTACTGTG<br>TTGTTTACATGCAGTGTATATATTCAGCTATATACATGCAGTG<br>TATATATATTGTTTATATTCAGCTACTCTTCTCGGCTACAGCG<br>GCAGATAATTCAATTAGCCATCTCGCAAGAAAGGAAATATG<br>ACTGTGTTCCTGACTAAAGTACACCTGGCATGTATGCTTGTAC<br>CTAAAAGGTCCGAATTCGAGAATTTTCTCGAAAGATAATTAA<br>GGAGCGTCAGGGAAATTGTTGGAGGTCAGTGATGAAAGGGC<br>GACGATTGTTTTTCTGACGTCATAGGTCTGGGTTTTTTTTTTA<br>CAACTTCCGTGTCGAAATTGCACTATTTCCTGCAGAGATCAG<br>AGCTATATATTAACAGCGTTTCAGGGTATTACTGTGCTATGA<br>ATATATTCCAGATTTTATGCGTGTTTTAACGTATATCACTTGC<br>AAAGATGTATTTTTATTGTCTTAAAATACTACCATGATGTGGT<br>GTACCTCGGAAAAATAAACTTACTTTTACATATATAAGAGTG<br>TTTATTGCTGTAGCAGGAAAGTTTACCTTCACTTTATTTAATG<br>CTATATAATTGTACCTTACAAAATTTTGTAGCTTATAATAAAG<br>TGAATAATCTTAATATTTCAACGATATTTGGAATGCTGTCAGC<br>ACAATTCAAACAACTTTCAGTGAATTATAGTGATTTCTCTTTA<br>ACAAGAATGCGATTAAAATCCAAAATAAGGTCGGAAAAATT<br>CACAGTTTTCTTCGCTAGATTTCAAGTAATTTGTACGGTTTTC<br>AGATAGTTCAAAAACTTTGACGTATGGATCTGACTTAGAGAA<br>CTTCCGCCACGTTTCACCGGGTTCCCCTCTCTTAGCAAAATTC<br>GTCCACTCTTTCACCATCTCAAAGCTTAGTTCTTCTTCCTTTCG<br>GGTGTAGTTGGACAGAGGAGTTAGAGGACGTCCAAAGATAA

							ATTGCATATCGTCACAATGCATAACCCCCATCCACTCTGCGTACGGAGTACTCGATGGTCTGTGATTGAAATAGTATCGGTAGATTTTATTATTTTTTTCTGCAACTTTCTCAGCAAAATATTTGCTTGGGCACATAACCATGAAATCCCCAAGAGAGTGATAAACTTGACTTCGTATTGTAGCATAATCGTCGTCTTTAACGCCACTGAGGTAATGCTGGATTATTCCTTCCGGATCTGGAAATATTGTCCCGAAATAATCTCTCATGAGAGACTCCCCTAACGTCTTATTAATGCGAGGATTTAATTCACCAAAAGGGCCTGTGATCTGTGTAAAAGTGTTAACAAATGGGAATGATCCCTCGTCTTGAGTAACTCCTATCATAGAATCAACACCGTTAAAGTTTCCTTCTCTGATAGCTTTTTGTGGGTCTACCGGTAGAAAATCATCCCCACTGATGGTAAAGAAAGTTATCACTGGGAAGACGGGAACTAAGGAAAACGTAGTTCTCACCATATCTTTTGGAGCTTTACTTCTTAGGCATTCGATCACATCATCAGGATTTTTCTTCAGCGTTATGTTTTCATTTGCACATTCCATCTTCTCTGCGAGCTTTTGTCCTAATTTAAGATTCTCAGCCCTATGGTCATTCAGAAAATATGCTGGACAACCACTTTGCATTATTGCTCTCTGGAAGAGACCTTTCGAAAGAGGAGAAACACACAGCAAACCTGTAGCTACGCCGCCGGCGCTGTTGCCGAACACCGTAATTCTTTTATTGTCTCCGCCGAATGCAGCAATGTTTTCGTTCGTCCATTTAAGGGCTTCCAAAATGTCATACAAGCCGACGTTTCCAGGAGCTTCTTCGGTTTCAGATGTGAAGAATCCGAGCACACTTAACCGGTAGTTTATCGCAACAACTATTACATCTCCAATTGCAGTTAGAACACGTCCATCATATAGAGGCATCCTACCTGAACCGGTTGCAAAGCCGCCTGAGAACACCCAAAACATGACGGCTTTTTTCTCGCTACTATTGGAAATTTCTGGTGCCCATATATTCAAATACAAGCAGTCTTCGCTTTGACCCTGAACTTTATCTTCCCACGGATAGTTGTCCGGAGAGTACTGGTAACAAGCAGGCGGTAAGCTGTTTGCTATTACAGGAGAGGTCCAAGGTTTG

							ACAGGAACTGTTCGCTTAAAGCGTAAATTCCCCACTGGAGGC<br>TCAGCAAACGGAATTCCGAGAAAGACATTTAGATTTATGTCG<br>TCATAAGTCATAACAGAACCAGTCACTTCACCTGTAGGAGTA<br>TTAACTGTAACGCTGGATGCTAAGATAGTTCCTGGTAGCAGT<br>AAAAATAGTAGATGCAAGCTCTGAAAAACAGAATTTAAGAT<br>ACTTATTGCATTATTTAAAAAAGAAATATCAGATAACTTATTT<br>TGAGGACAATTTTTCAACTATTATTTAATATCCCTCAGAACTG<br>TTACTATCAGCGACATAACCACAATTTTAACAATTCCCTCCTT<br>CTCTGAAGCAAATTTCCATTTTCCAGAAAGTGAGCCTCTTTTG<br>TACTAAAATGGATTTTGTTACATCTCTAGTTCTCAGAGTGCAC<br>TTACCAAGAGATCTTTTGGGAGAGATTATTACTTCCCTCATCT<br>TAATTCTTTGTTTATGATAGAGTTTTATCTGTATGTAATTTCAT<br>TTTAATGACTAAAAGTTTTGGATAATTGTAAGTCCAATAACG<br>CAACGCCTTATTTCAGAAATGCTTACAAAAAAAAACTGAGGT<br>AGTCACTGTTGCACTTGCACAACCTGTTGACAGTTGCTCATTG<br>GAATTCCATGGTGGCAATGGCATCTTCCTCAGTCTTCAATTAT<br>TCTTTTTGTAGATTACCAGGTTTTGATTAATTACATTTACGTAA<br>GTAGGAAATTACATATTGTGAAAAATGATTTTAGATATTTTTA<br>ATAGCAGATAGACGGAATATTTCCGATGATATAAATAACTG<br>AATATTTACGACACGCTTTTGCACTACAAGCGTTAAAGTTGT<br>GGCAGTAATAAGATTGCAACAACTTTTGAGTAGTTTTACAAA<br>GCGGAATCTAAAAAGTTAAGGAATT

PhdEnzCho 18	W4VSJ0	8,75E-110	37,618	PF00135	63218<br>**58747.62**	MSSPSSNVIAGAQGL<br>GAAMCGKLQKNVH<br>KSLHLLFLLLPGTILA<br>**SSVTVNTPTGEVTG**<br>**SVMTYDDINLNVFL**<br>**GIPFAEPPVGNLRF**<br>**KRTVPVKPWTSPVI**<br>**ANSLPPACYQYSPD**<br>**NYPWEDKVQGQSE**<br>**DCLYLNIWAPEISN**<br>**SSEKKAVMFWVFS**<br>**GGFATGSGRMPLY**<br>**DGRVLTAIGDVIVV**<br>**AINYRLSVLGFFTSE**<br>**TEEAPGNVGLYDIL**<br>**EALKWTNENIAAFG**<br>**GDNKRITVFGNSA**<br>**GGVATGLLCVSPLS**<br>**KGLFQRAIMQSGC**<br>**PAYFLNDHRAENLK**<br>**LGQKLAEKMECAN**<br>**ENITLKKNPDDVIE**<br>**CLRSKAPKDMVRT**<br>**TFSLVPVFPVITFFTI**<br>**SGDDFLPVDPQKAI**<br>**REGNFNGVDSMIG**<br>**VTQDEGSFPFVNTF**<br>**TQITGPFGELNPRIN**	CGATAAACCTTTTGCAAGCAAGTTTCTCATGATCTGGCAATA<br>TTCTAGAAAGTGCAAACATGATTATGTAATATGAAAAATGCT<br>GTATATGAAACTCGAGGTATTTTTGATTTATTCTTTATACTTTC<br>ATATTACATATTCATCTTTACACTTTCATAACTATGCTTTCATG<br>AGTCTGCGTACATCAACATAACACATTAGAAAACTTTAATAT<br>GCAATTCATATTCTCAATTTAAGCTAGGATCCCCTTTATTTTG<br>CAATTTAACTCAAAACTTCAAAGGAATAAACCTAAAATTATG<br>CTTTTTTAACTACGGGAAGCTTCAAATTTTACGATAAACCTTT<br>TGCAAGCAAGTTTCTCATGATCTGGCAATATTCTAGAAAGTG<br>CAAACATGATTATGTAATATGAAAAATGCTGTATATGAAACT<br>CAAGGTATTTTTGATTTATTTTTTATACTTTCATATTACATAGT<br>CATCTTTACACTTTCGTAATTCTGCGTATATCAACTGAAAACA<br>TTAGCAAACTTTAATATGCAATAAATAGCTATTAGGATAACC<br>TTTTCTTTTAATAACGTCACTATGCCTCTTTTAAATAGAATTG<br>ACTAGCTTCGATGTTTACTCGGAGCTTATATTATTCCATTCTTC<br>TCTCAATATGGCTTTCAACATAGCCTTTGAAGCTATATTGTGT<br>TGGCAGATTCTGCGTTCCAGCAAATCTCAAAAACACTTTATC<br>GGGTAAGTCAGGCGATTCTGGAGTTGTTTATGTGTAGGAGTA<br>GATAACTATTTTAGCACGTCATATAAGAACTAGATATTCATA<br>ACTTGAACAGTGTGCTTGGGGACCTTGTCCAATTAGAAATAA<br>TAACTGTTGTCTATCTTAAGTACTCTGCATTAAATTTCGTTTTA<br>AAATGTTTAAATAATCCATTTTATTCAATTGTGTCCTGAATAA<br>AGTGTAGTTGTCCCACGCCTCAGTTTTAGATACACCCCCAAA<br>TAATAATACTTTAGCCACCGTGTTTTACAGTTGGCATTAAATA<br>TTCTTACTTAAGCTCTTTAACAGGTTTTCGGTACAAAAGCTTG<br>AATCCTTTAATGTCAAATACGCAGAATCTGCTCTAATCGGAA<br>AATATTACCAGTTGCAGAGCTCATTTTGCTTATTAATATATTC<br>TTTTGCAACGTCAATGCTTAAATCTATTATCTCAGGAAATGTA

						**KTLGESLMRDYFGT**<br>**IFPDPEGIIQHYLSG**<br>**VKDDDYATIRSQV**<br>**YHSLGDFMVMCPS**<br>**KYFAEKVAEKNNKI**<br>**YRYYFNHRPSSTPY**<br>**AEWMGVMHCDD**<br>**MQFIFGRPLTPLSN**<br>**YTRKEEELSFEMVK**<br>**EWTNFAKRGEPGET**<br>**WRKFSKSDPYVKVF**<br>**ELSENRTNYLKSSEE**<br>**NCEFFRPYFGF***	AAACAGCTAAAAGGGATGTTACAACAGTTTCTCTTCGAGCCG<br>TCACATTTATCTTTTGTTCTTTCGCCTCTTGTGTTTTACCTATTC<br>CTCGACATATTTATGGCTATTTTTCAGTTCACAACTCTCTCGG<br>ATGGCGTAATCTTTTCTTGAAATTTCTTCGAATTTTCTCTCGTT<br>TTATATATATCAAAGCGGTAATGCGTTTTGCATTTTGTTCCGT<br>TTATAGTAACGTGCGCACCTAATACTTTCACCTGCTTCATGAA<br>GGACTGTATCAAATAGTCCGAAGTATGCTGCAGCTTTCCTGT<br>CTTACTGTGGATCAGAATCTTTTTTTGCAGCTTTATGGTATTCA<br>CTGCCTCAGTGACGAGACTTTGAAAATTTCAATCATGTTTTAA<br>AATTCCGCATATATCAACACGAAAATGGTGTCTCCAAGGATT<br>TGGAAAATTGAATTTCTACTCGCAAGTTACCTTGGTGTTGCA<br>ATTAAAATGAGGTCCATCACACAAGGATGGGATAAAGTCGA<br>ACACGTAAACTGTATAGCCTTCTACAAATTTGTCTTTCGAGA<br>AGTAAATACCTCTGTTTTGATTAAAGTTGATAAACAATTTGGT<br>GACAAGCGCGAATATATCTTTTAGAATCAAAATTCGGTTATA<br>AATGTTTGCTTGGCACTGAGTGACCATCTACTTAAACACTTA<br>AAAAGTTGACCATGATAATGATGAAAAAAAGGAAGAGGGC<br>AGGTTATGTTTCCTATTTACCGTGGCATTCTTCAGGGAGGTAA<br>TAATCACTCCTTTGGACATCAGTCACAAAAATACATTGTCTTT<br>CATAAAAGAAAATGTTCCAACTGAAACTGAGTATGCTTTATA<br>CAATACTCTGTCGATGGTATATTTCGAGAGACTTTTTTCGAGA<br>ACATTGACATGTCCCAAGGATACGCCGGGACATTCACTGTCC<br>TAATTAACAAGGAGGCATGCTCAGATACGATTTTATACTGGT<br>CTCATTTTGGGGGGGGAGACTAAACAAAAACTGTGTCTGTTG<br>TGGCTCATTTTGATTCGCAGATCATCAAATTTAATAAAAGTA<br>GATTTTGTTGAAAACATTCGCATCATCTCCAACGTTCGATTTT<br>TAGCTGTAAGTTCATTACGCTTCGTCAAACCTACATTTCTATC<br>GATTTCTTTCAACGGGTTCGTCGAATGTAATTTGCTGTCTTTGT

							AAAAGCATTCAACGGGGGAGTAAACTTTTCTTGACATTTTTTCCATATATCCAAATTCTTCAATATAGGTTCTGTAGGGATAAGTATTGCTATAGACGACGAGACGACTCGCTTCTTAAGTTGATTTCCACTCTGAAAAAAAGAGCGTGTAAAATTAAATTCACGGGGTCGACGAGTTCATTTTCAGGTAAAGCGGATCTATCCGACTTCATAATTTTGGCTTTTACGTGCAGTTAGGAAGACGATAAGTCAATGTAATCTTCACCATTGTTACATTCCACGGGTCCACCATCGCGTAGGTTGGACAGGGGAGGATATTCAATAAAATGTCCTCTTCGATTGCGGTCTCGCATAAGCGGGATCATGAACAAATGGAAATCAGATTTTGCGCAAGCAACACAGTATTGATAGATGCGTGTCGATATGCTTAACCGAAAATAACGCGTGATTCCATTTTCTTGGTTCTTGGTGAACTAATGCGTTATTCCAACGCTTTCTCTTTTAAATTATGACCAGAAGTAATAATGGACTTCATTTTTCAAGCTACTTTGTCCGTTAATTGGCTTCCAGCTTATTTCGAATAGCGTTTAACAGACTTTTTAGAACATTCAATATGCCCACACTGCTGTGAAATCATCGTTTGCCGACTACTTTTATGCCCAGTTTTAAAAGGGGAACTGTCACTCGATAAAAAGGACAAAAGAATCTGCCAAAACCATAAACTGTCTGAAATGTATCCCCTTTATAATAATTCAACTCGCCACGCTTTGCATAATCCTCGAAGGATTTTGGACAACATTGATGAGGCACATACATCACGATAGAAACTTTTGCCAAAAGTACAGTTTTACGTACGAGCGCTTGAGTTCAGATGACACAATTTCGCGTGTATGCATTCTTATAACGACATTCAATACTTTTCTATTCCGTTGGAAGTAATGGAGTCTTTCATATTGTGCTCAAATCATTTCCCCGTCGCTTCCGGTCACGCTCAAGATGCGCAACAAGTGCGCTAACACATCTCCCACAATATCAGGCTCTACAATGTCCGAATATATCAACAGTAATCTATAATGAGCGCACAGATATGCCGCTTACTGACTTTCGACGTTCTTTTCTTCATCATAATCATCATCGGTACTCTCAAGTTCAAACATAGCATTTGCGCGAGTCCGTCGTACA

							GTATCACTTTAGCACCCTGCAAAACAAAAACAGAATCTGCC<br>CCTTCCGGTAGACGCACTATGTTAAGAGTTCCCGAAATTGGA<br>ATCGATGCAAAACTTTGAGGGTAGACAGAATATAGTATATA<br>GACAAAATATGTCCAGATCGAAATAGAGTTTTAGGTCCAGAT<br>CAAGCTTTCAAGTTGTGAGTCTAGCATCGTTCTATGGTGGGA<br>AATGTGTTAAAAATATTTTTTAAATGCTGCACCCTTTACTGTG<br>TTGTTTACATGCAGTGTATATATTCAGCTATATACATGCAGTG<br>TATATATATTGTTTATATTCAGCTACTCTTCTCGGCTACAGCG<br>GCAGATAATTCAATTAGCCATCTCGCAAGAAAGGAAATATG<br>ACTGTGTTCCTGACTAAAGTACACCTGGCATGTATGCTTGTAC<br>CTAAAAGGTCCGAATTCGAGAATTTTCTCGAAAGATAATTAA<br>GGAGCGTCAGGGAAATTGTTGGAGGTCAGTGATGAAAGGGC<br>GACGATTGTTTTTCTGACGTCATAGGTCTGGGTTTTTTTTTTA<br>CAACTTCCGTGTCGAAATTGCACTATTTCCTGCAGAGATCAG<br>AGCTATATATTAACAGCGTTTCAGGGTATTACTGTGCTATGA<br>ATATATTCCAGATTTTATGCGTGTTTTAACGTATATCACTTGC<br>AAAGATGTATTTTTATTGTCTTAAAATACTACCATGATGTGGT<br>GTACCTCGGAAAAATAAACTTACTTTTACATATATAAGAGTG<br>TTTATTGCTGTAGCAGGAAAGTTTACCTTCACTTTATTTAATG<br>CTATATAATTGTACCTTACAAAATTTTGTAGCTTATAATAAAG<br>TGAATAATCTTAATATTTCAACGATATTTGGAATGCTGTCAGC<br>ACAATTCAAACAACTTTCAGTGAATTATAGTGATTTCTCTTTA<br>ACAAGAATGCGATTAAAATCCAAAATAAGGTCGGAAAAATT<br>CACAGTTTTCTTCGCTAGATTTCAAGTAATTTGTACGGTTTTC<br>AGATAGTTCAAAAACTTTGACGTATGGATCTGACTTAGAGAA<br>CTTCCGCCACGTTTCACCGGGTTCCCCTCTCTTAGCAAAATTC<br>GTCCACTCTTTCACCATCTCAAAGCTTAGTTCTTCTTCCTTTCG<br>GGTGTAGTTGGACAGAGGAGTTAGAGGACGTCCAAAGATAA

							ATTGCATATCGTCACAATGCATAACCCCCATCCACTCTGCGT<br>ACGGAGTACTCGATGGTCTGTGATTGAAATAGTATCGGTAGA<br>TTTTATTATTTTTTTCTGCAACTTTCTCAGCAAAATATTTGCTT<br>GGGCACATAACCATGAAATCCCCAAGAGAGTGATAAACTTG<br>ACTTCGTATTGTAGCATAATCGTCGTCTTTAACGCCACTGAGG<br>TAATGCTGGATTATTCCTTCCGGATCTGGAAATATTGTCCCGA<br>AATAATCTCTCATGAGAGACTCCCCTAACGTCTTATTAATGC<br>GAGGATTTAATTCACCAAAAGGGCCTGTGATCTGTGTAAAAG<br>TGTTAACAAATGGGAATGATCCCTCGTCTTGAGTAACTCCTA<br>TCATAGAATCAACACCGTTAAAGTTTCCTTCTCTGATAGCTTT<br>TTGTGGGTCTACCGGTAGAAAATCATCCCCACTGATGGTAAA<br>GAAAGTTATCACTGGGAAGACGGGAACTAAGGAAAACGTA<br>GTTCTCACCATATCTTTTGGAGCTTTACTTCTTAGGCATTCGAT<br>CACATCATCAGGATTTTTCTTCAGCGTTATGTTTTCATTTGCAC<br>ATTCCATCTTCTCTGCGAGCTTTTGTCCTAATTTAAGATTCTCA<br>GCCCTATGGTCATTCAGAAAATATGCTGGACAACCACTTTGC<br>ATTATTGCTCTCTGGAAGAGACCTTTCGAAAGAGGAGAAAC<br>ACACAGCAAACCTGTAGCTACGCCGCCGGCGCTGTTGCCGA<br>ACACCGTAATTCTTTTATTGTCTCCGCCGAATGCAGCAATGTT<br>TTCGTTCGTCCATTTAAGGGCTTCCAAAATGTCATACAAGCC<br>GACGTTTCCAGGAGCTTCTTCGGTTTCAGATGTGAAGAATCC<br>GAGCACACTTAACCGGTAGTTTATCGCAACAACTATTACATC<br>TCCAATTGCAGTTAGAACACGTCCATCATATAGAGGCATCCT<br>ACCTGAACCGGTTGCAAAGCCGCCTGAGAACACCCAAAACA<br>TGACGGCTTTTTTCTCGCTACTATTGGAAATTTCTGGTGCCCA<br>TATATTCAAATACAAGCAGTCTTCGCTTTGACCCTGAACTTTA<br>TCTTCCCACGGATAGTTGTCCGGAGAGTACTGGTAACAAGCA<br>GGCGGTAAGCTGTTTGCTATTACAGGAGAGGTCCAAGGTTTG

							ACAGGAACTGTTCGCTTAAAGCGTAAATTCCCCACTGGAGGCTCAGCAAACGGAATTCCGAGAAAGACATTTAGATTTATGTCGTCATAAGTCATAACAGAACCAGTCACTTCACCTGTAGGAGTATTAACTGTAACGCTGGATGCTAAGATAGTTCCTGGTAGCAGTAAAAATAGTAGATGCAAGCTCTTATGAACGTTTTTCTGGAGCTTTCCGCACATAGCGGCTCCTAAACCTTGGGCTCCAGCAATAACGTTAGACGAGGGAGACGACATGATGCTGCTGCCTAGATCTCCAGTGTGCTGTGTCGTACACAAATGCGCCTATTTCCTGTTTGTAAAGCTCCTTCGTACACCAGTATCGTCAACAGCTATTTTTACATTAGTTATTCCGTTTCTGTCAGCAATCTTGCATCGCACAAATCTGCTAGTGTAGGTCTTCATCCGAATTATGCCCAAACTGGTTGTAGTTTTACTGTGGATAAGAAGCTTTCTTAACCTAATTTGTTCTCCACAGGCT

PhdEnzCho 19	W4VSJ0	1,20E-109	37,618	PF00135	63354,11	LKNCPQNKLSDISFL<br>NNAISILNSVFQSLH<br>LLFLLLPGTILASSVT<br>VNTPTGEVTGSVMT<br>YDDINLNVFLGIPFA<br>EPPVGNLRFKRTVPV<br>KPWTSPVIANSLPPA<br>CYQYSPDNYPWEDK<br>VQGQSEDCLYLNIW<br>APEISNSSEKKAVMF<br>WVFSGGFATGSGRM<br>PLYDGRVLTAIGDVI<br>VVAINYRLSVLGFFT<br>SETEEAPGNVGLYDI<br>LEALKWTNENIAAF<br>GGDNKRITVFGNSA<br>GGVATGLLCVSPLSK<br>GLFQRAIMQSGCPA<br>YFLNDHRAENLKLG<br>QKLAEKMECANENI<br>TLKKNPDDVIECLRS<br>KAPKDMVRTTFSLV<br>PVFPVITFFTISGDDF<br>LPVDPQKAIREGNF<br>NGVDSMIGVTQDEG<br>SFPFVNTFTQITGPFG<br>ELNPRINKTLGESLM<br>RDYFGTIFPDPEGIIQ	CGATAAACCTTTTGCAAGCAAGTTTCTCATGATCTGGCAATA<br>TTCTAGAAAGTGCAAACATGATTATGTAATATGAAAAATGCT<br>GTATATGAAACTCGAGGTATTTTTGATTTATTCTTTATACTTTC<br>ATATTACATATTCATCTTTACACTTTCATAACTATGCTTTCATG<br>AGTCTGCGTACATCAACATAACACATTAGAAAACTTTAATAT<br>GCAATTCATATTCTCAATTTAAGCTAGGATCCCCTTTATTTTG<br>CAATTTAACTCAAAACTTCAAAGGAATAAACCTAAAATTATG<br>CTTTTTTAACTACGGGAAGCTTCAAATTTTACGATAAACCTTT<br>TGCAAGCAAGTTTCTCATGATCTGGCAATATTCTAGAAAGTG<br>CAAACATGATTATGTAATATGAAAAATGCTGTATATGAAACT<br>CAAGGTATTTTTGATTTATTTTTTATACTTTCATATTACATAGT<br>CATCTTTACACTTTCGTAATTCTGCGTATATCAACTGAAAACA<br>TTAGCAAACTTTAATATGCAATAAATAGCTATTAGGATAACC<br>TTTTCTTTTAATAACGTCACTATGCCTCTTTTAAATAGAATTG<br>ACTAGCTTCGATGTTTACTCGGAGCTTATATTATTCCATTCTTC<br>TCTCAATATGGCTTTCAACATAGCCTTTGAAGCTATATTGTGT<br>TGGCAGATTCTGCGTTCCAGCAAATCTCAAAAACACTTTATC<br>GGGTAAGTCAGGCGATTCTGGAGTTGTTTATGTGTAGGAGTA<br>GATAACTATTTTAGCACGTCATATAAGAACTAGATATTCATA<br>ACTTGAACAGTGTGCTTGGGGACCTTGTCCAATTAGAAATAA<br>TAACTGTTGTCTATCTTAAGTACTCTGCATTAAATTTCGTTTTA<br>AAATGTTTAAATAATCCATTTTATTCAATTGTGTCCTGAATAA<br>AGTGTAGTTGTCCCACGCCTCAGTTTTAGATACACCCCCAAA<br>TAATAATACTTTAGCCACCGTGTTTTACAGTTGGCATTAAATA<br>TTCTTACTTAAGCTCTTTAACAGGTTTTCGGTACAAAAGCTTG<br>AATCCTTTAATGTCAAATACGCAGAATCTGCTCTAATCGGAA<br>AATATTACCAGTTGCAGAGCTCATTTTGCTTATTAATATATTC<br>TTTTGCAACGTCAATGCTTAAATCTATTATCTCAGGAAATGTA

						HYLSGVKDDDYATI<br>RSQVYHSLGDFMVM<br>CPSKYFAEKVAEKN<br>NKIYRYYFNHRPSST<br>PYAEWMGVMHCD<br>DMQFIFGRPLTPLSN<br>YTRKEEELSFEMVKE<br>WTNFAKRGEPGET<br>WRKFSKSDPYVKVF<br>ELSENRTNYLKSSEE<br>NCEFFRPYFGF*	AAACAGCTAAAAGGGATGTTACAACAGTTTCTCTTCGAGCCG<br>TCACATTTATCTTTTGTTCTTTCGCCTCTTGTGTTTTACCTATTC<br>CTCGACATATTTATGGCTATTTTTCAGTTCACAACTCTCTCGG<br>ATGGCGTAATCTTTTCTTGAAATTTCTTCGAATTTTCTCTCGTT<br>TTATATATATCAAAGCGGTAATGCGTTTTGCATTTTGTTCCGT<br>TTATAGTAACGTGCGCACCTAATACTTTCACCTGCTTCATGAA<br>GGACTGTATCAAATAGTCCGAAGTATGCTGCAGCTTTCCTGT<br>CTTACTGTGGATCAGAATCTTTTTTTGCAGCTTTATGGTATTCA<br>CTGCCTCAGTGACGAGACTTTGAAAATTTCAATCATGTTTTAA<br>AATTCCGCATATATCAACACGAAAATGGTGTCTCCAAGGATT<br>TGGAAAATTGAATTTCTACTCGCAAGTTACCTTGGTGTTGCA<br>ATTAAAATGAGGTCCATCACACAAGGATGGGATAAAGTCGA<br>ACACGTAAACTGTATAGCCTTCTACAAATTTGTCTTTCGAGA<br>AGTAAATACCTCTGTTTTGATTAAAGTTGATAAACAATTTGGT<br>GACAAGCGCGAATATATCTTTTAGAATCAAAATTCGGTTATA<br>AATGTTTGCTTGGCACTGAGTGACCATCTACTTAAACACTTA<br>AAAAGTTGACCATGATAATGATGAAAAAAAGGAAGAGGGC<br>AGGTTATGTTTCCTATTTACCGTGGCATTCTTCAGGGAGGTAA<br>TAATCACTCCTTTGGACATCAGTCACAAAAATACATTGTCTTT<br>CATAAAAGAAAATGTTCCAACTGAAACTGAGTATGCTTTATA<br>CAATACTCTGTCGATGGTATATTTCGAGAGACTTTTTTCGAGA<br>ACATTGACATGTCCCAAGGATACGCCGGGACATTCACTGTCC<br>TAATTAACAAGGAGGCATGCTCAGATACGATTTTATACTGGT<br>CTCATTTTGGGGGGGGAGACTAAACAAAAACTGTGTCTGTTG<br>TGGCTCATTTTGATTCGCAGATCATCAAATTTAATAAAAGTA<br>GATTTTGTTGAAAACATTCGCATCATCTCCAACGTTCGATTTT<br>TAGCTGTAAGTTCATTACGCTTCGTCAAACCTACATTTCTATC<br>GATTTCTTTCAACGGGTTCGTCGAATGTAATTTGCTGTCTTTGT

							AAAAGCATTCAACGGGGGAGTAAACTTTTCTTGACATTTTTT<br>CCATATATCCAAATTCTTCAATATAGGTTCTGTAGGGATAAG<br>TATTGCTATAGACGACGAGACGACTCGCTTCTTAAGTTGATTT<br>CCACTCTGAAAAAAAGAGCGTGTAAAATTAAATTCACGGGG<br>TCGACGAGTTCATTTTCAGGTAAAGCGGATCTATCCGACTTC<br>ATAATTTTGGCTTTTACGTGCAGTTAGGAAGACGATAAGTCA<br>ATGTAATCTTCACCATTGTTACATTCCACGGGTCCACCATCGC<br>GTAGGTTGGACAGGGGAGGATATTCAATAAAATGTCCTCTTC<br>GATTGCGGTCTCGCATAAGCGGGATCATGAACAAATGGAAA<br>TCAGATTTTGCGCAAGCAACACAGTATTGATAGATGCGTGTC<br>GATATGCTTAACCGAAAATAACGCGTGATTCCATTTTCTTGGT<br>TCTTGGTGAACTAATGCGTTATTCCAACGCTTTCTCTTTTAAA<br>TTATGACCAGAAGTAATAATGGACTTCATTTTTCAAGCTACTT<br>TGTCCGTTAATTGGCTTCCAGCTTATTTCGAATAGCGTTTAAC<br>AGACTTTTTAGAACATTCAATATGCCCACACTGCTGTGAAAT<br>CATCGTTTGCCGACTACTTTTATGCCCAGTTTTAAAAGGGGA<br>ACTGTCACTCGATAAAAAGGACAAAAGAATCTGCCAAAACC<br>ATAAACTGTCTGAAATGTATCCCCTTTATAATAATTCAACTCG<br>CCACGCTTTGCATAATCCTCGAAGGATTTTGGACAACATTGA<br>TGAGGCACATACATCACGATAGAAACTTTTGCCAAAAGTAC<br>AGTTTTACGTACGAGCGCTTGAGTTCAGATGACACAATTTCG<br>CGTGTATGCATTCTTATAACGACATTCAATACTTTTCTATTCC<br>GTTGGAAGTAATGGAGTCTTTCATATTGTGCTCAAATCATTTC<br>CCCGTCGCTTCCGGTCACGCTCAAGATGCGCAACAAGTGCGC<br>TAACACATCTCCCACAATATCAGGCTCTACAATGTCCGAATA<br>TATCAACAGTAATCTATAATGAGCGCACAGATATGCCGCTTA<br>CTGACTTTCGACGTTCTTTTCTTCATCATAATCATCATCGGTA<br>CTCTCAAGTTCAAACATAGCATTTGCGCGAGTCCGTCGTACA

							GTATCACTTTAGCACCCTGCAAAACAAAAACAGAATCTGCC<br>CCTTCCGGTAGACGCACTATGTTAAGAGTTCCCGAAATTGGA<br>ATCGATGCAAAACTTTGAGGGTAGACAGAATATAGTATATA<br>GACAAAATATGTCCAGATCGAAATAGAGTTTTAGGTCCAGAT<br>CAAGCTTTCAAGTTGTGAGTCTAGCATCGTTCTATGGTGGGA<br>AATGTGTTAAAAATATTTTTTAAATGCTGCACCCTTTACTGTG<br>TTGTTTACATGCAGTGTATATATTCAGCTATATACATGCAGTG<br>TATATATATTGTTTATATTCAGCTACTCTTCTCGGCTACAGCG<br>GCAGATAATTCAATTAGCCATCTCGCAAGAAAGGAAATATG<br>ACTGTGTTCCTGACTAAAGTACACCTGGCATGTATGCTTGTAC<br>CTAAAAGGTCCGAATTCGAGAATTTTCTCGAAAGATAATTAA<br>GGAGCGTCAGGGAAATTGTTGGAGGTCAGTGATGAAAGGGC<br>GACGATTGTTTTTCTGACGTCATAGGTCTGGGTTTTTTTTTTA<br>CAACTTCCGTGTCGAAATTGCACTATTTCCTGCAGAGATCAG<br>AGCTATATATTAACAGCGTTTCAGGGTATTACTGTGCTATGA<br>ATATATTCCAGATTTTATGCGTGTTTTAACGTATATCACTTGC<br>AAAGATGTATTTTTATTGTCTTAAAATACTACCATGATGTGGT<br>GTACCTCGGAAAAATAAACTTACTTTTACATATATAAGAGTG<br>TTTATTGCTGTAGCAGGAAAGTTTACCTTCACTTTATTTAATG<br>CTATATAATTGTACCTTACAAAATTTTGTAGCTTATAATAAAG<br>TGAATAATCTTAATATTTCAACGATATTTGGAATGCTGTCAGC<br>ACAATTCAAACAACTTTCAGTGAATTATAGTGATTTCTCTTTA<br>ACAAGAATGCGATTAAAATCCAAAATAAGGTCGGAAAAATT<br>CACAGTTTTCTTCGCTAGATTTCAAGTAATTTGTACGGTTTTC<br>AGATAGTTCAAAAACTTTGACGTATGGATCTGACTTAGAGAA<br>CTTCCGCCACGTTTCACCGGGTTCCCCTCTCTTAGCAAAATTC<br>GTCCACTCTTTCACCATCTCAAAGCTTAGTTCTTCTTCCTTTCG<br>GGTGTAGTTGGACAGAGGAGTTAGAGGACGTCCAAAGATAA

							ATTGCATATCGTCACAATGCATAACCCCCATCCACTCTGCGT<br>ACGGAGTACTCGATGGTCTGTGATTGAAATAGTATCGGTAGA<br>TTTTATTATTTTTTTCTGCAACTTTCTCAGCAAAATATTTGCTT<br>GGGCACATAACCATGAAATCCCCAAGAGAGTGATAAACTTG<br>ACTTCGTATTGTAGCATAATCGTCGTCTTTAACGCCACTGAGG<br>TAATGCTGGATTATTCCTTCCGGATCTGGAAATATTGTCCCGA<br>AATAATCTCTCATGAGAGACTCCCCTAACGTCTTATTAATGC<br>GAGGATTTAATTCACCAAAAGGGCCTGTGATCTGTGTAAAAG<br>TGTTAACAAATGGGAATGATCCCTCGTCTTGAGTAACTCCTA<br>TCATAGAATCAACACCGTTAAAGTTTCCTTCTCTGATAGCTTT<br>TTGTGGGTCTACCGGTAGAAAATCATCCCCACTGATGGTAAA<br>GAAAGTTATCACTGGGAAGACGGGAACTAAGGAAAACGTA<br>GTTCTCACCATATCTTTTGGAGCTTTACTTCTTAGGCATTCGAT<br>CACATCATCAGGATTTTTCTTCAGCGTTATGTTTTCATTTGCAC<br>ATTCCATCTTCTCTGCGAGCTTTTGTCCTAATTTAAGATTCTCA<br>GCCCTATGGTCATTCAGAAAATATGCTGGACAACCACTTTGC<br>ATTATTGCTCTCTGGAAGAGACCTTTCGAAAGAGGAGAAAC<br>ACACAGCAAACCTGTAGCTACGCCGCCGGCGCTGTTGCCGA<br>ACACCGTAATTCTTTTATTGTCTCCGCCGAATGCAGCAATGTT<br>TTCGTTCGTCCATTTAAGGGCTTCCAAAATGTCATACAAGCC<br>GACGTTTCCAGGAGCTTCTTCGGTTTCAGATGTGAAGAATCC<br>GAGCACACTTAACCGGTAGTTTATCGCAACAACTATTACATC<br>TCCAATTGCAGTTAGAACACGTCCATCATATAGAGGCATCCT<br>ACCTGAACCGGTTGCAAAGCCGCCTGAGAACACCCAAAACA<br>TGACGGCTTTTTTCTCGCTACTATTGGAAATTTCTGGTGCCCA<br>TATATTCAAATACAAGCAGTCTTCGCTTTGACCCTGAACTTTA<br>TCTTCCCACGGATAGTTGTCCGGAGAGTACTGGTAACAAGCA<br>GGCGGTAAGCTGTTTGCTATTACAGGAGAGGTCCAAGGTTTG

							ACAGGAACTGTTCGCTTAAAGCGTAAATTCCCCACTGGAGGC<br>TCAGCAAACGGAATTCCGAGAAAGACATTTAGATTTATGTCG<br>TCATAAGTCATAACAGAACCAGTCACTTCACCTGTAGGAGTA<br>TTAACTGTAACGCTGGATGCTAAGATAGTTCCTGGTAGCAGT<br>AAAAATAGTAGATGCAAGCTCTGAAAAACAGAATTTAAGAT<br>ACTTATTGCATTATTTAAAAAAGAAATATCAGATAACTTATTT<br>TGAGGACAATTTTTCAACTATTATTTAATATCCCTCAGAACTG<br>TTACTATCAGCGACATAACCACAATTTTAACAATTCCCTCCTT<br>CTCTGAAGCAAATTTCCATTTTCCAGAAAGTGAGCCTCTTTTG<br>TACTAAAATGGATTTTGTTACATCTCTAGTTCTCAGAGTGCAC<br>TTACCAAGAGATCTTTTGGGAGAGATTATTACTTCCCTCATCT<br>TAATTCTTTGTTTATGATAGAGTTTTATCTGTATGTAATTTCAT<br>TTTAATGACTAAAAGTTTTGGATAATTGTAAGTCCAATAACG<br>CAACGCCTTATTTCAGAAATGCTTACAAAAAAAAACTGAGGT<br>AGTCACTGTTGCACTTGCACAACCTGTTGACAGTTGCTCATTG<br>GAATTCCATGGTGGCAATGGCATCTTCCTCAGTCTTCAATTAT<br>TCTTTTTGTAGATTACCAGGTTTTGATTAATTACATTTACGTAA<br>GTAGGAAATTACATATTGTGAAAAATGATTTTAGATATTTTTA<br>ATAGCAGATAGACGGAATATTTCCGATGATATAAATAACTG<br>AATATTTACGACACGCTTTTGCACTACAAGCGTTAAAGTTGT<br>GGCAGTAATAAGATTGCAACAACTTTTGAGTAGTTTTACAAA<br>GCGGAATCTAAAAAGTTAAGGAATT

PhdEnzCho 20	W4VSJ0	8,78E-110	37,618	PF00135	63218.00<br><br>**58747.62**	MSSPSSNVIAGAQGL<br>GAAMCGKLQKNVH<br>KSLHLLFLLLPGTILA<br>**SSVTVNTPTGEVTG**<br>**SVMTYDDINLNVFL**<br>**GIPFAEPPVGNLRF**<br>**KRTVPVKPWTSPVI**<br>**ANSLPPACYQYSPD**<br>**NYPWEDKVQGQSE**<br>**DCLYLNIWAPEISN**<br>**SSEKKAVMFWVFS**<br>**GGFATGSGRMPLY**<br>**DGRVLTAIGDVIVV**<br>**AINYRLSVLGFFTSE**<br>**TEEAPGNVGLYDIL**<br>**EALKWTNENIAAFG**<br>**GDNKRITVFGNSA**<br>**GGVATGLLCVSPLS**<br>**KGLFQRAIMQSGC**<br>**PAYFLNDHRAENLK**<br>**LGQKLAEKMECAN**<br>**ENITLKKNPDDVIE**<br>**CLRSKAPKDMVRT**<br>**TFSLVPVFPVITFFTI**<br>**SGDDFLPVDPQKAI**<br>**REGNFNGVDSMIG**<br>**VTQDEGSFPFVNTF**<br>**TQITGPFGELNPRIN**	CGATAAACCTTTTGCAAGCAAGTTTCTCATGATCTGGCAATA<br>TTCTAGAAAGTGCAAACATGATTATGTAATATGAAAAATGCT<br>GTATATGAAACTCGAGGTATTTTTGATTTATTCTTTATACTTTC<br>ATATTACATATTCATCTTTACACTTTCATAACTATGCTTTCATG<br>AGTCTGCGTACATCAACATAACACATTAGAAAACTTTAATAT<br>CAATTCATATTCTCAATTTCATATTCTCAATTTAAGCTAGGAT<br>CCCCTTTATTTTGCAATTTAACTCAAAACTTCAAAGGAATAA<br>ACCTAAAATTATGCTTTTTTAACTACGGGAAGCTTCAAATTTT<br>ACGATAAACCTTTTGCAAGCAAGTTTCTCATGATCTGGCAAT<br>ATTCTAGAAAGTGCAAACATGATTATGTAATATGAAAAATGC<br>TGTATATGAAACTCAAGGTATTTTTGATTTATTTTTTATACTTT<br>CATATTACATAGTCATCTTTACACTTTCGTAATTCTGCGTATA<br>TCAACTGAAAACATTAGCAAACTTTAATATGCAATAAATAGC<br>TATTAGGATAACCTTTTCTTTTAATAACGTCACTATGCCTCTTT<br>TAAATAGAATTGACTAGCTTCGATGTTTACTCGGAGCTTATAT<br>TATTCCATTCTTCTCTCAATATGGCTTTCAACATAGCCTTTGA<br>AGCTATATTGTGTTGGCAGATTCTGCGTTCCAGCAAATCTCA<br>AAAACACTTTATCGGGTAAGTCAGGCGATTCTGGAGTTGTTT<br>ATGTGTAGGAGTAGATAACTATTTTAGCACGTCATATAAGAA<br>CTAGATATTCATAACTTGAACAGTGTGCTTGGGGACCTTGTC<br>CAATTAGAAATAATAACTGTTGTCTATCTTAAGTACTCTGCAT<br>TAAATTTCGTTTTAAAATGTTTAAATAATCCATTTTATTCAATT<br>GTGTCCTGAATAAAGTGTAGTTGTCCCACGCCTCAGTTTTAG<br>ATACACCCCCAAATAATAATACTTTAGCCACCGTGTTTTACA<br>GTTGGCATTAAATATTCTTACTTAAGCTCTTTAACAGGTTTTC<br>GGTACAAAAGCTTGAATCCTTTAATGTCAAATACGCAGAATC<br>TGCTCTAATCGGAAAATATTACCAGTTGCAGAGCTCATTTTG<br>CTTATTAATATATTCTTTTGCAACGTCAATGCTTAAATCTATT

						**KTLGESLMRDYFGT**	ATCTCAGGAAATGTAAAACAGCTAAAAGGGATGTTACAACA
						**IFPDPEGIIQHYLSG**	GTTTCTCTTCGAGCCGTCACATTTATCTTTTGTTCTTTCGCCTC
						**VKDDDYATIRSQV**	TTGTGTTTTACCTATTCCTCGACATATTTATGGCTATTTTTCAG
						**YHSLGDFMVMCPS**	TTCACAACTCTCTCGGATGGCGTAATCTTTTCTTGAAATTTCTT
						**KYFAEKVAEKNNKI**	CGAATTTTCTCTCGTTTTATATATATCAAAGCGGTAATGCGTT
						**YRYYFNHRPSSTPY**	TTGCATTTTGTTCCGTTTATAGTAACGTGCGCACCTAATACTT
						**AEWMGVMHCDD**	TCACCTGCTTCATGAAGGACTGTATCAAATAGTCCGAAGTAT
						**MQFIFGRPLTPLSN**	GCTGCAGCTTTCCTGTCTTACTGTGGATCAGAATCTTTTTTTGC
						**YTRKEEELSFEMVK**	AGCTTTATGGTATTCACTGCCTCAGTGACGAGACTTTGAAAA
						**EWTNFAKRGEPGET**	TTTCAATCATGTTTTAAAATTCCGCATATATCAACACGAAAA
						**WRKFSKSDPYVKVF**	TGGTGTCTCCAAGGATTTGGAAAATTGAATTTCTACTCGCAA
						**ELSENRTNYLKSSEE**	GTTACCTTGGTGTTGCAATTAAAATGAGGTCCATCACACAAG
						**NCEFFRPYFGF***	GATGGGATAAAGTCGAACACGTAAACTGTATAGCCTTCTACA
							AATTTGTCTTTCGAGAAGTAAATACCTCTGTTTTGATTAAAGT
							TGATAAACAATTTGGTGACAAGCGCGAATATATCTTTTAGAA
							TCAAAATTCGGTTATAAATGTTTGCTTGGCACTGAGTGACCA
							TCTACTTAAACACTTAAAAAGTTGACCATGATAATGATGAAA
							AAAAGGAAGAGGGCAGGTTATGTTTCCTATTTACCGTGGCAT
							TCTTCAGGGAGGTAATAATCACTCCTTTGGACATCAGTCACA
							AAAATACATTGTCTTTCATAAAAGAAAATGTTCCAACTGAAA
							CTGAGTATGCTTTATACAATACTCTGTCGATGGTATATTTCGA
							GAGACTTTTTTCGAGAACATTGACATGTCCCAAGGATACGCC
							GGGACATTCACTGTCCTAATTAACAAGGAGGCATGCTCAGAT
							ACGATTTTATACTGGTCTCATTTTGGGGGGGGAGACTAAACA
							AAAACTGTGTCTGTTGTGGCTCATTTTGATTCGCAGATCATCA
							AATTTAATAAAAGTAGATTTTGTTGAAAACATTCGCATCATC
							TCCAACGTTCGATTTTTAGCTGTAAGTTCATTACGCTTCGTCA
							AACCTACATTTCTATCGATTTCTTTCAACGGGTTCGTCGAATG

							TAATTTGCTGTCTTTGTAAAAGCATTCAACGGGGGAGTAAAC<br>TTTTCTTGACATTTTTTCCATATATCCAAATTCTTCAATATAGG<br>TTCTGTAGGGATAAGTATTGCTATAGACGACGAGACGACTCG<br>CTTCTTAAGTTGATTTCCACTCTGAAAAAAAGAGCGTGTAAA<br>ATTAAATTCACGGGGTCGACGAGTTCATTTTCAGGTAAAGCG<br>GATCTATCCGACTTCATAATTTTGGCTTTTACGTGCAGTTAGG<br>AAGACGATAAGTCAATGTAATCTTCACCATTGTTACATTCCA<br>CGGGTCCACCATCGCGTAGGTTGGACAGGGGAGGATATTCA<br>ATAAAATGTCCTCTTCGATTGCGGTCTCGCATAAGCGGGATC<br>ATGAACAAATGGAAATCAGATTTTGCGCAAGCAACACAGTA<br>TTGATAGATGCGTGTCGATATGCTTAACCGAAAATAACGCGT<br>GATTCCATTTTCTTGGTTCTTGGTGAACTAATGCGTTATTCCA<br>ACGCTTTCTCTTTTAAATTATGACCAGAAGTAATAATGGACTT<br>CATTTTTCAAGCTACTTTGTCCGTTAATTGGCTTCCAGCTTATT<br>TCGAATAGCGTTTAACAGACATTTTTAGAACATTCAATATGC<br>CCACACTGCTGTGAAATCATCGTTTGCCGACTACTTTTATGCC<br>CAGTTTTAAAAGGGGAACTGTCACTCGATAAAAAGGACAAA<br>AGAATCTGCCAAAACCATAAACTGTCTGAAATGTATCCCCTT<br>TATAATAATTCAACTCGCCACGCTTTGCATAATCCTCGAAGG<br>ATTTTGGACAACATTGATGAGGCACATACATCACGATAGAA<br>ACTTTTGCCAAAAGTACAGTTTTACGTACGAGCGCTTGAGTT<br>CAGATGACACAATTTCGCGTGTATGCATTCTTATAACGACAT<br>TCAATACTTTTCTATTCCGTTGGAAGTAATGGAGTCTTTCATA<br>TTGTGCTCAAATCATTTCCCCGTCGCTTCCGGTCACGCTCAAG<br>ATGCGCAACAAGTGCGCTAACACATCTCCCACAATATCAGG<br>CTCTACAATGTCCGAATATACCAACAGTAATCTATAATGAGC<br>ACACAGATATGCCGCTTACTGACTTTCGACGTTCTTTTCTTCA<br>TCATAATCATCATCGGTACTCTCAAGTTCAAACATAGCATTT

							GCGCGAGTCCGTCGTACAGTATCACTTTAGCACCCTGCAAAA<br>CAAAAACAGAATCTGCCCCTTCCGGTAGACGCACTATGTTAA<br>GAGTTCCCGAAATTGGAATCGATGCAAAACTTTGAGGGTAG<br>ACAGAATATAGTATATAGACAAAATATGTCCAGATCGAAAT<br>AGAGTTTTAGGTCCAGATCAAGCTTTCAAGTTGTGAGTCTAG<br>CATCGTTCTATGGTGGGAAATGTGTTAAAAATATTTTTTAAAT<br>GCTGCACCCTTTACTGTGTTGTTTACATGCAGTGTATATATTC<br>AGCTATATACATGCAGTGTATATATATTGTTTATATTCAGCTA<br>CTCTTCTCGGCTACAGCGGCAGATAATTCAATTAGCCATCTC<br>GCAAGAAAGGAAATATGACTGTGTTCCTGACTAAAGTACAC<br>CTGGCATGTATGCTTGTACCTAAAAGGTCCGAATTCGAGAAT<br>TTTCTCGAAAGATAATTAAGGAGCGTCAGGGAAATTGTTGGA<br>GGTCAGTGATGAAAGGGCGACGATTGTTTTTCTGACGTCATA<br>GGTCTGGGTTTTTTTTTTTACAACTTCCGTGTCGAAATTGCACT<br>ATTTCCTGCAGAGATCAGAGCTATATATTAACAGCGTTTCAG<br>GGTATTACTGTGCTATGAATATATTCCAGATTTTATGCGTGTT<br>TTAACGTATATCACTTGCAAAGATGTATTTTTATTGTCTTAAA<br>ATACTACCATGATGTGGTGTACCTCGGAAAAATAAACTTACT<br>TTTACATATATAAGAGTGTTTATTGCTGTAGCAGGAAAGTTTA<br>CCTTCACTTTATTTAATGCTATATAATTGTACCTTACAAAATT<br>TTGTAGCTTATAATAAAGTGAATAATCTTAATATTTCAACGAT<br>ATTTGGAATGCTGTCAGCACAATTCAAACAACTTTCAGTGAA<br>TTATAGTGATTTCTCTTTAACAAGAATGCGATTAAAATCCAA<br>AATAAGGTCGGAAAAATTCACAGTTTTCTTCGCTAGATTTCA<br>AGTAATTTGTACGGTTTTCAGATAGTTCAAAAACTTTGACGT<br>ATGGATCTGACTTAGAGAACTTCCGCCACGTTTCACCGGGTT<br>CCCCTCTCTTAGCAAAATTCGTCCACTCTTTCACCATCTCAAA<br>GCTTAGTTCTTCTTCCTTTCGGGTGTAGTTGGACAGAGGAGTT

							AGAGGACGTCCAAAGATAAATTGCATATCGTCACAATGCAT AACCCCCATCCACTCTGCGTACGGAGTACTCGATGGTCTGTG ATTGAAATAGTATCGGTAGATTTTATTATTTTTTTCTGCAACTT TCTCAGCAAAATATTTGCTTGGGCACATAACCATGAAATCCC CAAGAGAGTGATAAACTTGACTTCGTATTGTAGCATAATCGT CGTCTTTAACGCCACTGAGGTAATGCTGGATTATTCCTTCCGG ATCTGGAAATATTGTCCCGAAATAATCTCTCATGAGAGACTC CCCTAACGTCTTATTAATGCGAGGATTTAATTCACCAAAAGG GCCTGTGATCTGTGTAAAAGTGTTAACAAATGGGAATGATCC CTCGTCTTGAGTAACTCCTATCATAGAATCAACACCGTTAAA GTTTCCTTCTCTGATAGCTTTTTGTGGGTCTACCGGTAGAAAA TCATCCCCACTGATGGTAAAGAAAGTTATCACTGGGAAGAC GGGAACTAAGGAAAACGTAGTTCTCACCATATCTTTTGGAGC TTTACTTCTTAGGCATTCGATCACATCATCAGGATTTTTCTTCA GCGTTATGTTTTCATTTGCACATTCCATCTTCTCTGCGAGCTTT TGTCCTAATTTAAGATTCTCAGCCCTATGGTCATTCAGAAAAT ATGCTGGACAACCACTTTGCATTATTGCTCTCTGGAAGAGAC CTTTCGAAAGAGGAGAAACACACAGCAAACCTGTAGCTACG CCGCCGGCGCTGTTGCCGAACACCGTAATTCTTTTATTGTCTC CGCCGAATGCAGCAATGTTTTCGTTCGTCCATTTAAGGGCTTC CAAAATGTCATACAAGCCGACGTTTCCAGGAGCTTCTTCGGT TTCAGATGTGAAGAATCCGAGCACACTTAACCGGTAGTTTAT CGCAACAACTATTACATCTCCAATTGCAGTTAGAACACGTCC ATCATATAGAGGCATCCTACCTGAACCGGTTGCAAAGCCGCC TGAGAACACCCAAAACATGACGGCTTTTTTCTCGCTACTATT GGAAATTTCTGGTGCCCATATATTCAAATACAAGCAGTCTTC GCTTTGACCCTGAACTTTATCTTCCCACGGATAGTTGTCCGGA GAGTACTGGTAACAAGCAGGCGGTAAGCTGTTTGCTATTACA

							GGAGAGGTCCAAGGTTTGACAGGAACTGTTCGCTTAAAGCGTAAATTCCCCACTGGAGGCTCAGCAAACGGAATTCCGAGAAAGACATTTAGATTTATGTCGTCATAAGTCATAACAGAACCAGTCACTTCACCTGTAGGAGTATTAACTGTAACGCTGGATGCTAAGATAGTTCCTGGTAGCAGTAAAAATAGTAGATGCAAGCTCTTATGAACGTTTTTCTGGAGCTTTCCGCACATAGCGGCTCCTAAACCTTGGGCTCCAGCAATAACGTTAGACGAGGGAGACGACATGATGCTGCTGCCTAGATCTCCAGTGTGCTGTGTCGTACACAAATGCGCCTATTTCCTGTTTGTAAAGCTCCTTCGTACACCAGTATCGTCAACAGCTATTTTTACATTAGTTATTCCGTTTCTGTCAGCAATCTTGCATCGCACAAATCTGCTAGTGTAGGTCTTCATCCGAATTATGCCCAAACTGGTTGTAGTTTTACTGTGGATAAGAAGCTTTCTTAACCTAATTTGTTCTCCACAGGCT

PhdEnzCho 21	W4VSJ0	1,08E-109	37,618	PF00135	63218<br><br>**58747.62**	MSSPSSNVIAGAQGL<br>GAAMCGKLQKNVH<br>KSLHLLFLLLPGTILA<br>**SSVTVNTPTGEVTG**<br>**SVMTYDDINLNVFL**<br>**GIPFAEPPVGNLRF**<br>**KRTVPVKPWTSPVI**<br>**ANSLPPACYQYSPD**<br>**NYPWEDKVQGQSE**<br>**DCLYLNIWAPEISN**<br>**SSEKKAVMFWVFS**<br>**GGFATGSGRMPLY**<br>**DGRVLTAIGDVIVV**<br>**AINYRLSVLGFFTSE**<br>**TEEAPGNVGLYDIL**<br>**EALKWTNENIAAFG**<br>**GDNKRITVFGNSA**<br>**GGVATGLLCVSPLS**<br>**KGLFQRAIMQSGC**<br>**PAYFLNDHRAENLK**<br>**LGQKLAEKMECAN**<br>**ENITLKKNPDDVIE**<br>**CLRSKAPKDMVRT**<br>**TFSLVPVFPVITFFTI**<br>**SGDDFLPVDPQKAI**<br>**REGNFNGVDSMIG**<br>**VTQDEGSFPFVNTF**<br>**TQITGPFGELNPRIN**	CGATAAACCTTTTGCAAGCAAGTTTCTCATGATCTGGCAATA<br>TTCTAGAAAGTGCAAACATGATTATGTAATATGAAAAATGCT<br>GTATATGAAACTCGAGGTATTTTTGATTTATTCTTTATACTTTC<br>ATATTACATATTCATCTTTACACTTTCATAACTATGCTTTCATG<br>AGTCTGCGTACATCAACATAACACATTAGAAAACTTTAATAT<br>GCAATTCATATTCTCAATTTAAGCTAGGATCCCCTTTATTTTG<br>CAATTTAACTCAAAACTTCAAAGGAATAAACCTAAAATTATG<br>CTTTTTTAACTACGGGAAGCTTCAAATTTTACGATAAACCTTT<br>TGCAAGCAAGTTTCTCATGATCTGGCAATATTCTAGAAAGTG<br>CAAACATGATTATGTAATATGAAAAATGCTGTATATGAAACT<br>CAAGGTATTTTTGATTTATTTTTTATACTTTCATATTACATAGT<br>CATCTTTACACTTTCGTAATTCTGCGTATATCAACTGAAAACA<br>TTAGCAAACTTTAATATGCAATAAATAGCTATTAGGATAACC<br>TTTTCTTTTAATAACGTCACTATGCCTCTTTTAAATAGAATTG<br>ACTAGCTTCGATGTTTACTCGGAGCTTATATTATTCCATTCTTC<br>TCTCAATATGGCTTTCAACATAGCCTTTGAAGCTATATTGTGT<br>TGGCAGATTCTGCGTTCCAGCAAATCTCAAAAACACTTTATC<br>GGGTAAGTCAGGCGATTCTGGAGTTGTTTATGTGTAGGAGTA<br>GATAACTATTTTAGCACGTCATATAAGAACTAGATATTCATA<br>ACTTGAACAGTGTGCTTGGGGACCTTGTCCAATTAGAAATAA<br>TAACTGTTGTCTATCTTAAGTACTCTGCATTAAATTTCGTTTTA<br>AAATGTTTAAATAATCCATTTTATTCAATTGTGTCCTGAATAA<br>AGTGTAGTTGTCCCACGCCTCAGTTTTAGATACACCCCCAAA<br>TAATAATACTTTAGCCACCGTGTTTTACAGTTGGCATTAAATA<br>TTCTTACTTAAGCTCTTTAACAGGTTTTCGGTACAAAAGCTTG<br>AATCCTTTAATGTCAAATACGCAGAATCTGCTCTAATCGGAA<br>AATATTACCAGTTGCAGAGCTCATTTTGCTTATTAATATATTC<br>TTTTGCAACGTCAATGCTTAAATCTATTATCTCAGGAAATGTA

						**KTLGESLMRDYFGT<br>IFPDPEGIIQHYLSG<br>VKDDDYATIRSQV<br>YHSLGDFMVMCPS<br>KYFAEKVAEKNNKI<br>YRYYFNHRPSSTPY<br>AEWMGVMHCDD<br>MQFIFGRPLTPLSN<br>YTRKEEELSFEMVK<br>EWTNFAKRGEPGET<br>WRKFSKSDPYVKVF<br>ELSENRTNYLKSSEE<br>NCEFFRPYFGF***	AAACAGCTAAAAGGGATGTTACAACAGTTTCTCTTCGAGCCG<br>TCACATTTATCTTTTGTTCTTTCGCCTCTTGTGTTTTACCTATTC<br>CTCGACATATTTATGGCTATTTTTCAGTTCACAACTCTCTCGG<br>ATGGCGTAATCTTTTCTTGAAATTTCTTCGAATTTTCTCTCGTT<br>TTATATATATCAAAGCGGTAATGCGTTTTGCATTTTGTTCCGT<br>TTATAGTAACGTGCGCACCTAATACTTTCACCTGCTTCATGAA<br>GGACTGTATCAAATAGTCCGAAGTATGCTGCAGCTTTCCTGT<br>CTTACTGTGGATCAGAATCTTTTTTTGCAGCTTTATGGTATTCA<br>CTGCCTCAGTGACGAGACTTTGAAAATTTCAATCATGTTTTAA<br>AATTCCGCATATATCAACACGAAAATGGTGTCTCCAAGGATT<br>TGGAAAATTGAATTTCTACTCGCAAGTTACCTTGGTGTTGCA<br>ATTAAAATGAGGTCCATCACACAAGGATGGGATAAAGTCGA<br>ACACGTAAACTGTATAGCCTTCTACAAATTTGTCTTTCGAGA<br>AGTAAATACCTCTGTTTTGATTAAAGTTGATAAACAATTTGGT<br>GACAAGCGCGAATATATCTTTTAGAATCAAAATTCGGTTATA<br>AATGTTTGCTTGGCACTGAGTGACCATCTACTTAAACACTTA<br>AAAAGTTGACCATGATAATGATGAAAAAAAGGAAGAGGGC<br>AGGTTATGTTTCCTATTTACCGTGGCATTCTTCAGGGAGGTAA<br>TAATCACTCCTTTGGACATCAGTCACAAAAATACATTGTCTTT<br>CATAAAAGAAAATGTTCCAACTGAAACTGAGTATGCTTTATA<br>CAATACTCTGTCGATGGTATATTTCGAGAGACTTTTTTCGAGA<br>ACATTGACATGTCCCAAGGATACGCCGGGACATTCACTGTCC<br>TAATTAACAAGGAGGCATGCTCAGATACGATTTTATACTGGT<br>CTCATTTTGGGGGGGGAGACTAAACAAAAACTGTGTCTGTTG<br>TGGCTCATTTTGATTCGCAGATCATCAAATTTAATAAAAGTA<br>GATTTTGTTGAAAACATTCGCATCATCTCCAACGTTCGATTTT<br>TAGCTGTAAGTTCATTACGCTTCGTCAAACCTACATTTCTATC<br>GATTTCTTTCAACGGGTTCGTCGAATGTAATTTGCTGTCTTTGT

							AAAAGCATTCAACGGGGGAGTAAACTTTTCTTGACATTTTTT<br>CCATATATCCAAATTCTTCAATATAGGTTCTGTAGGGATAAG<br>TATTGCTATAGACGACGAGACGACTCGCTTCTTAAGTTGATTT<br>CCACTCTGAAAAAAAGAGCGTGTAAAATTAAATTCACGGGG<br>TCGACGAGTTCATTTTCAGGTAAAGCGGATCTATCCGACTTC<br>ATAATTTTGGCTTTTACGTGCAGTTAGGAAGACGATAAGTCA<br>ATGTAATCTTCACCATTGTTACATTCCACGGGTCCACCATCGC<br>GTAGGTTGGACAGGGGAGGATATTCAATAAAATGTCCTCTTC<br>GATTGCGGTCTCGCATAAGCGGGATCATGAACAAATGGAAA<br>TCAGATTTTGCGCAAGCAACACAGTATTGATAGATGCGTGTC<br>GATATGCTTAACCGAAAATAACGCGTGATTCCATTTTCTTGGT<br>TCTTGGTGAACTAATGCGTTATTCCAACGCTTTCTCTTTTAAA<br>TTATGACCAGAAGTAATAATGGACTTCATTTTTCAAGCTACTT<br>TGTCCGTTAATTGGCTTCCAGCTTATTTCGAATAGCGTTTAAC<br>AGACATTTTTAGAACATTCAATATGCCCACACTGCTGTGAAA<br>TCATCGTTTGCCGACTACTTTTATGCCCAGTTTTAAAAGGGGA<br>ACTGTCACTCGATAAAAAGGACAAAAGAATCTGCCAAAACC<br>ATAAACTGTCTGAAATGTATCCCCTTTATAATAATTCAACTCG<br>CCACGCTTTGCATAATCCTCGAAGGATTTTGGACAACATTGA<br>TGAGGCACATACATCACGATAGAAACTTTTGCCAAAAGTAC<br>AGTTTTACGTACGAGCGCTTGAGTTCAGATGACACAATTTCG<br>CGTGTATGCATTCTTATAACGACATTCAATACTTTTCTATTCC<br>GTTGGAAGTAATGGAGTCTTTCATATTGTGCTCAAATCATTTC<br>CCCGTCGCTTCCGGTCACGCTCAAGATGCGCAACAAGTGCGC<br>TAACACATCTCCCACAATATCAGGCTCTACAATGTCCGAATA<br>TATCAACAGTAATCTATAATGAGCGCACAGATATGCCGCTTA<br>CTGACTTTCGACGTTCTTTTCTTCATCATAATCATCATCGGTA<br>CTCTCAAGTTCAAACATAGCATTTGCGCGAGTCCGTCGTACA

							GTATCACTTTAGCACCCTGCAAAACAAAAACAGAATCTGCC<br>CCTTCCGGTAGACGCACTATGTTAAGAGTTCCCGAAATTGGA<br>ATCGATGCAAAACTTTGAGGGTAGACAGAATATAGTATATA<br>GACAAAATATGTCCAGATCGAAATAGAGTTTTAGGTCCAGAT<br>CAAGCTTTCAAGTTGTGAGTCTAGCATCGTTCTATGGTGGGA<br>AATGTGTTAAAAATATTTTTTAAATGCTGCACCCTTTACTGTG<br>TTGTTTACATGCAGTGTATATATTCAGCTATATACATGCAGTG<br>TATATATATTGTTTATATTCAGCTACTCTTCTCGGCTACAGCG<br>GCAGATAATTCAATTAGCCATCTCGCAAGAAAGGAAATATG<br>ACTGTGTTCCTGACTAAAGTACACCTGGCATGTATGCTTGTAC<br>CTAAAAGGTCCGAATTCGAGAATTTTCTCGAAAGATAATTAA<br>GGAGCGTCAGGGAAATTGTTGGAGGTCAGTGATGAAAGGGC<br>GACGATTGTTTTTCTGACGTCATAGGTCTGGGTTTTTTTTTTA<br>CAACTTCCGTGTCGAAATTGCACTATTTCCTGCAGAGATCAG<br>AGCTATATATTAACAGCGTTTCAGGGTATTACTGTGCTATGA<br>ATATATTCCAGATTTTATGCGTGTTTTAACGTATATCACTTGC<br>AAAGATGTATTTTTATTGTCTTAAAATACTACCATGATGTGGT<br>GTACCTCGGAAAAATAAACTTACTTTTACATATATAAGAGTG<br>TTTATTGCTGTAGCAGGAAAGTTTACCTTCACTTTATTTAATG<br>CTATATAATTGTACCTTACAAAATTTTGTAGCTTATAATAAAG<br>TGAATAATCTTAATATTTCAACGATATTTGGAATGCTGTCAGC<br>ACAATTCAAACAACTTTCAGTGAATTATAGTGATTTCTCTTTA<br>ACAAGAATGCGATTAAAATCCAAAATAAGGTCGGAAAAATT<br>CACAGTTTTCTTCGCTAGATTTCAAGTAATTTGTACGGTTTTC<br>AGATAGTTCAAAAACTTTGACGTATGGATCTGACTTAGAGAA<br>CTTCCGCCACGTTTCACCGGGTTCCCCTCTCTTAGCAAAATTC<br>GTCCACTCTTTCACCATCTCAAAGCTTAGTTCTTCTTCCTTTCG<br>GGTGTAGTTGGACAGAGGAGTTAGAGGACGTCCAAAGATAA

							ATTGCATATCGTCACAATGCATAACCCCCATCCACTCTGCGT<br>ACGGAGTACTCGATGGTCTGTGATTGAAATAGTATCGGTAGA<br>TTTTATTATTTTTTTCTGCAACTTTCTCAGCAAAATATTTGCTT<br>GGGCACATAACCATGAAATCCCCAAGAGAGTGATAAACTTG<br>ACTTCGTATTGTAGCATAATCGTCGTCTTTAACGCCACTGAGG<br>TAATGCTGGATTATTCCTTCCGGATCTGGAAATATTGTCCCGA<br>AATAATCTCTCATGAGAGACTCCCCTAACGTCTTATTAATGC<br>GAGGATTTAATTCACCAAAAGGGCCTGTGATCTGTGTAAAAG<br>TGTTAACAAATGGGAATGATCCCTCGTCTTGAGTAACTCCTA<br>TCATAGAATCAACACCGTTAAAGTTTCCTTCTCTGATAGCTTT<br>TTGTGGGTCTACCGGTAGAAAATCATCCCCACTGATGGTAAA<br>GAAAGTTATCACTGGGAAGACGGGAACTAAGGAAAACGTA<br>GTTCTCACCATATCTTTTGGAGCTTTACTTCTTAGGCATTCGAT<br>CACATCATCAGGATTTTTCTTCAGCGTTATGTTTTCATTTGCAC<br>ATTCCATCTTCTCTGCGAGCTTTTGTCCTAATTTAAGATTCTCA<br>GCCCTATGGTCATTCAGAAAATATGCTGGACAACCACTTTGC<br>ATTATTGCTCTCTGGAAGAGACCTTTCGAAAGAGGAGAAAC<br>ACACAGCAAACCTGTAGCTACGCCGCCGGCGCTGTTGCCGA<br>ACACCGTAATTCTTTTATTGTCTCCGCCGAATGCAGCAATGTT<br>TTCGTTCGTCCATTTAAGGGCTTCCAAAATGTCATACAAGCC<br>GACGTTTCCAGGAGCTTCTTCGGTTTCAGATGTGAAGAATCC<br>GAGCACACTTAACCGGTAGTTTATCGCAACAACTATTACATC<br>TCCAATTGCAGTTAGAACACGTCCATCATATAGAGGCATCCT<br>ACCTGAACCGGTTGCAAAGCCGCCTGAGAACACCCAAAACA<br>TGACGGCTTTTTTCTCGCTACTATTGGAAATTTCTGGTGCCCA<br>TATATTCAAATACAAGCAGTCTTCGCTTTGACCCTGAACTTTA<br>TCTTCCCACGGATAGTTGTCCGGAGAGTACTGGTAACAAGCA<br>GGCGGTAAGCTGTTTGCTATTACAGGAGAGGTCCAAGGTTTG

							ACAGGAACTGTTCGCTTAAAGCGTAAATTCCCCACTGGAGGC<br>TCAGCAAACGGAATTCCGAGAAAGACATTTAGATTTATGTCG<br>TCATAAGTCATAACAGAACCAGTCACTTCACCTGTAGGAGTA<br>TTAACTGTAACGCTGGATGCTAAGATAGTTCCTGGTAGCAGT<br>AAAAATAGTAGATGCAAGCTCTTATGAACGTTTTTCTGGAGC<br>TTTCCGCACATAGCGGCTCCTAAACCTTGGGCTCCAGCAATA<br>ACGTTAGACGAGGGAGACGACATGATGCTGCTGCCTAGATC<br>TCCAGTGTGCTGTGTCGTACACAAATGCGCCTATTTCCTGCTA<br>GTGTAGGTCTTCATCCGAATTATGCCCAAACTGGTTGTAGTTT<br>TACTGTGGATAAGAAGCTTTCTTAACCTAATTTGTTCTCCACA<br>GGCTCCTTGAGAGTACGATGTTAACACATTGATGATGGGAGA<br>CGCTAATTTGCGTGTTGAATACTTCATGCGGTATCCTATAAAA<br>ATGTTAACTACTATTAAATAAAACAGTAAAAAAAGCAATGG<br>ATGCTATGAACAAAATGTTACGAATGTGTTTTTACATGAGAA<br>TGCTTGAATAATAAGAAACTCCGCCATCAATATTGAAAAAAT<br>TTTTTTGAAAACCTTTTATTTAGTAATGAGAAGGCAATAAGCT<br>ACGGAAGATCAGTTTGTGGAATATAGAGCAGAAATTTATGC<br>AGATTTGTTGTCAGATGTACAAAATGATGCCGAATGTGAAAA<br>TAATATTATTCATAGTTGCATTGAAGTTGATTTTATTAAGAAT<br>AGAAGCACTCGTCCTTTGCCGAGATGTCATTTCACTAATGTCT<br>GGAAAAGTATTCATCTTTTTCAGGCTTTAATGAAAAAGATGA<br>ATACATATCTTTATCTTACGAAACAAGTTCATGGACTAAGCA<br>AGACAAAATTACTGTTCTGGAAGATTTTATAGGAAACCCTGG<br>AGTAAAGCAATTTCCATCCGATCATACTAGCATTAATGAAGT<br>CGTAGGTTTATTCTTTGGAAATGATTTCTTTGAAATGCTTTAC<br>CGTGAAAATGAATACAAATGAAATAAAAACTAAAGTGGGTT<br>GATGTAGAAATAAAGGAAATGAAACGATTTTTTTATCTTGTT<br>TTACTTATGGTACATGTAAAAAAGGATCGCATTAGAGATTAT

							TGGTCGACTGATTCATACATAGAAACTCCGATTTTTTTCAAAAGTAATGAGCCGGAACAGATTTGAACAAATTTTACAGTCGCTCCATTTCCGTAATAATGATTTTCAAACTAATTCGACAGATAGACTCTTCAAGTTAAGACCAATATTACATTACTGTGTAAAAAAATTTCAACTCATACATAAGCCAAATCAAGAAGTATCTTTTGGCAGAACATGGAGAACTGCTCCGATACATGATTGGTATGCCGGTTTTCGGCCTGGAGCATCCGTTGTAAAAGCAAGTAATAGAAAAGCACAAACTGCCTTGGCAAGGTTCCGCAGCGGCCATTTTCGGCCGATGAGGTTTTCTGCTGACAAGAGGGTCTACCCTTTAAGTTCAAAATGCAGCTTGACTGAGACGACTCCCTCTCATGTTCTATTGTGCATTGGTTGCACCAAGAACCATCTGTTTTATAACTCTGGGGCGTTTCTGGATCAGTTGTG

PhdEnzCho 22	W4VSJ0	1,20E-109	37,618	PF00135	63354,11	LKNCPQNKLSDISFL NNAISILNSVFQSLH LLFLLLPGTILASSVT VNTPTGEVTGSVMT YDDINLNVFLGIPFA EPPVGNLRFKRTVPV KPWTSPVIANSLPPA CYQYSPDNYPWEDK VQGQSEDCLYLNIW APEISNSSEKKAVMF WVFSGGFATGSGRM PLYDGRVLTAIGDVI VVAINYRLSVLGFFT SETEEAPGNVGLYDI LEALKWTNENIAAF GGDNKRITVFGNSA GGVATGLLCVSPLSK GLFQRAIMQSGCPA YFLNDHRAENLKLG QKLAEKMECANENI TLKKNPDDVIECLRS KAPKDMVRTTFSLV PVFPVITFFTISGDDF LPVDPQKAIREGNF NGVDSMIGVTQDEG SFPFVNTFTQITGPFG ELNPRINKTLGESLM RDYFGTIFPDPEGIIQ	CGATAAACCTTTTGCAAGCAAGTTTCTCATGATCTGGCAATA TTCTAGAAAGTGCAAACATGATTATGTAATATGAAAAATGCT GTATATGAAACTCGAGGTATTTTTGATTTATTCTTTATACTTTC ATATTACATATTCATCTTTACACTTTCATAACTATGCTTTCATG AGTCTGCGTACATCAACATAACACATTAGAAAACTTTAATAT CAATTCATATTCTCAATTTCATATTCTCAATTTAAGCTAGGAT CCCCTTTATTTTGCAATTTAACTCAAAACTTCAAAGGAATAA ACCTAAAATTATGCTTTTTTAACTACGGGAAGCTTCAAATTTT ACGATAAACCTTTTGCAAGCAAGTTTCTCATGATCTGGCAAT ATTCTAGAAAGTGCAAACATGATTATGTAATATGAAAAATGC TGTATATGAAACTCAAGGTATTTTTGATTTATTTTTTATACTTT CATATTACATAGTCATCTTTACACTTTCGTAATTCTGCGTATA TCAACTGAAAACATTAGCAAACTTTAATATGCAATAAATAGC TATTAGGATAACCTTTTCTTTTAATAACGTCACTATGCCTCTTT TAAATAGAATTGACTAGCTTCGATGTTTACTCGGAGCTTATAT TATTCCATTCTTCTCTCAATATGGCTTTCAACATAGCCTTTGA AGCTATATTGTGTTGGCAGATTCTGCGTTCCAGCAAATCTCA AAAACACTTTATCGGGTAAGTCAGGCGATTCTGGAGTTGTTT ATGTGTAGGAGTAGATAACTATTTTAGCACGTCATATAAGAA CTAGATATTCATAACTTGAACAGTGTGCTTGGGGACCTTGTC CAATTAGAAATAATAACTGTTGTCTATCTTAAGTACTCTGCAT TAAATTTCGTTTTAAAATGTTTAAATAATCCATTTTATTCAATT GTGTCCTGAATAAAGTGTAGTTGTCCCACGCCTCAGTTTTAG ATACACCCCCAAATAATAATACTTTAGCCACCGTGTTTTACA GTTGGCATTAAATATTCTTACTTAAGCTCTTTAACAGGTTTTC GGTACAAAAGCTTGAATCCTTTAATGTCAAATACGCAGAATC TGCTCTAATCGGAAAATATTACCAGTTGCAGAGCTCATTTTG CTTATTAATATATTCTTTTGCAACGTCAATGCTTAAATCTATT

						HYLSGVKDDDYATI<br>RSQVYHSLGDFMVM<br>CPSKYFAEKVAEKN<br>NKIYRYYFNHRPSST<br>PYAEWMGVMHCD<br>DMQFIFGRPLTPLSN<br>YTRKEEELSFEMVKE<br>WTNFAKRGEPGET<br>WRKFSKSDPYVKVF<br>ELSENRTNYLKSSEE<br>NCEFFRPYFGF*	ATCTCAGGAAATGTAAAACAGCTAAAAGGGATGTTACAACA<br>GTTTCTCTTCGAGCCGTCACATTTATCTTTTGTTCTTTCGCCTC<br>TTGTGTTTTACCTATTCCTCGACATATTTATGGCTATTTTTCAG<br>TTCACAACTCTCTCGGATGGCGTAATCTTTTCTTGAAATTTCTT<br>CGAATTTTCTCTCGTTTTATATATATCAAAGCGGTAATGCGTT<br>TTGCATTTTGTTCCGTTTATAGTAACGTGCGCACCTAATACTT<br>TCACCTGCTTCATGAAGGACTGTATCAAATAGTCCGAAGTAT<br>GCTGCAGCTTTCCTGTCTTACTGTGGATCAGAATCTTTTTTTGC<br>AGCTTTATGGTATTCACTGCCTCAGTGACGAGACTTTGAAAA<br>TTTCAATCATGTTTTAAAATTCCGCATATATCAACACGAAAA<br>TGGTGTCTCCAAGGATTTGGAAAATTGAATTTCTACTCGCAA<br>GTTACCTTGGTGTTGCAATTAAAATGAGGTCCATCACACAAG<br>GATGGGATAAAGTCGAACACGTAAACTGTATAGCCTTCTACA<br>AATTTGTCTTTCGAGAAGTAAATACCTCTGTTTTGATTAAAGT<br>TGATAAACAATTTGGTGACAAGCGCGAATATATCTTTTAGAA<br>TCAAAATTCGGTTATAAATGTTTGCTTGGCACTGAGTGACCA<br>TCTACTTAAACACTTAAAAAGTTGACCATGATAATGATGAAA<br>AAAAGGAAGAGGGCAGGTTATGTTTCCTATTTACCGTGGCAT<br>TCTTCAGGGAGGTAATAATCACTCCTTTGGACATCAGTCACA<br>AAAATACATTGTCTTTCATAAAAGAAAATGTTCCAACTGAAA<br>CTGAGTATGCTTTATACAATACTCTGTCGATGGTATATTTCGA<br>GAGACTTTTTTCGAGAACATTGACATGTCCCAAGGATACGCC<br>GGGACATTCACTGTCCTAATTAACAAGGAGGCATGCTCAGAT<br>ACGATTTTATACTGGTCTCATTTTGGGGGGGGAGACTAAACA<br>AAAACTGTGTCTGTTGTGGCTCATTTTGATTCGCAGATCATCA<br>AATTTAATAAAAGTAGATTTTGTTGAAAACATTCGCATCATC<br>TCCAACGTTCGATTTTTAGCTGTAAGTTCATTACGCTTCGTCA<br>AACCTACATTTCTATCGATTTCTTTCAACGGGTTCGTCGAATG

							TAATTTGCTGTCTTTGTAAAAGCATTCAACGGGGGAGTAAACTTTTCTTGACATTTTTTCCATATATCCAAATTCTTCAATATAGGTTCTGTAGGGATAAGTATTGCTATAGACGACGAGACGACTCGCTTCTTAAGTTGATTTCCACTCTGAAAAAAAGAGCGTGTAAAATTAAATTCACGGGGTCGACGAGTTCATTTTCAGGTAAAGCGGATCTATCCGACTTCATAATTTTGGCTTTTACGTGCAGTTAGGAAGACGATAAGTCAATGTAATCTTCACCATTGTTACATTCCACGGGTCCACCATCGCGTAGGTTGGACAGGGGAGGATATTCAATAAAATGTCCTCTTCGATTGCGGTCTCGCATAAGCGGGATCATGAACAAATGGAAATCAGATTTTGCGCAAGCAACACAGTATTGATAGATGCGTGTCGATATGCTTAACCGAAAATAACGCGTGATTCCATTTTCTTGGTTCTTGGTGAACTAATGCGTTATTCCAACGCTTTCTCTTTTAAATTATGACCAGAAGTAATAATGGACTTCATTTTTCAAGCTACTTTGTCCGTTAATTGGCTTCCAGCTTATTTCGAATAGCGTTTAACAGACATTTTTAGAACATTCAATATGCCCACACTGCTGTGAAATCATCGTTTGCCGACTACTTTTATGCCCAGTTTTAAAAGGGGAACTGTCACTCGATAAAAAGGACAAAAGAATCTGCCAAAACCATAAACTGTCTGAAATGTATCCCCTTTATAATAATTCAACTCGCCACGCTTTGCATAATCCTCGAAGGATTTTGGACAACATTGATGAGGCACATACATCACGATAGAAACTTTTGCCAAAAGTACAGTTTTACGTACGAGCGCTTGAGTTCAGATGACACAATTTCGCGTGTATGCATTCTTATAACGACATTCAATACTTTTCTATTCCGTTGGAAGTAATGGAGTCTTTCATATTGTGCTCAAATCATTTCCCCGTCGCTTCCGGTCACGCTCAAGATGCGCAACAAGTGCGCTAACACATCTCCCACAATATCAGGCTCTACAATGTCCGAATATACCAACAGTAATCTATAATGAGCACACAGATATGCCGCTTACTGACTTTCGACGTTCTTTTCTTCATCATAATCATCATCGGTACTCTCAAGTTCAAACATAGCATTT

							GCGCGAGTCCGTCGTACAGTATCACTTTAGCACCCTGCAAAA<br>CAAAAACAGAATCTGCCCCTTCCGGTAGACGCACTATGTTAA<br>GAGTTCCCGAAATTGGAATCGATGCAAAACTTTGAGGGTAG<br>ACAGAATATAGTATATAGACAAAATATGTCCAGATCGAAAT<br>AGAGTTTTAGGTCCAGATCAAGCTTTCAAGTTGTGAGTCTAG<br>CATCGTTCTATGGTGGGAAATGTGTTAAAAATATTTTTTAAAT<br>GCTGCACCCTTTACTGTGTTGTTTACATGCAGTGTATATATTC<br>AGCTATATACATGCAGTGTATATATATTGTTTATATTCAGCTA<br>CTCTTCTCGGCTACAGCGGCAGATAATTCAATTAGCCATCTC<br>GCAAGAAAGGAAATATGACTGTGTTCCTGACTAAAGTACAC<br>CTGGCATGTATGCTTGTACCTAAAAGGTCCGAATTCGAGAAT<br>TTTCTCGAAAGATAATTAAGGAGCGTCAGGGAAATTGTTGGA<br>GGTCAGTGATGAAAGGGCGACGATTGTTTTTCTGACGTCATA<br>GGTCTGGGTTTTTTTTTTTACAACTTCCGTGTCGAAATTGCACT<br>ATTTCCTGCAGAGATCAGAGCTATATATTAACAGCGTTTCAG<br>GGTATTACTGTGCTATGAATATATTCCAGATTTTATGCGTGTT<br>TTAACGTATATCACTTGCAAAGATGTATTTTTATTGTCTTAAA<br>ATACTACCATGATGTGGTGTACCTCGGAAAAATAAACTTACT<br>TTTACATATATAAGAGTGTTTATTGCTGTAGCAGGAAAGTTTA<br>CCTTCACTTTATTTAATGCTATATAATTGTACCTTACAAAATT<br>TTGTAGCTTATAATAAAGTGAATAATCTTAATATTTCAACGAT<br>ATTTGGAATGCTGTCAGCACAATTCAAACAACTTTCAGTGAA<br>TTATAGTGATTTCTCTTTAACAAGAATGCGATTAAAATCCAA<br>AATAAGGTCGGAAAAATTCACAGTTTTCTTCGCTAGATTTCA<br>AGTAATTTGTACGGTTTTCAGATAGTTCAAAAACTTTGACGT<br>ATGGATCTGACTTAGAGAACTTCCGCCACGTTTCACCGGGTT<br>CCCCTCTCTTAGCAAAATTCGTCCACTCTTTCACCATCTCAAA<br>GCTTAGTTCTTCTTCCTTTCGGGTGTAGTTGGACAGAGGAGTT

							AGAGGACGTCCAAAGATAAATTGCATATCGTCACAATGCAT<br>AACCCCCATCCACTCTGCGTACGGAGTACTCGATGGTCTGTG<br>ATTGAAATAGTATCGGTAGATTTTATTATTTTTTTCTGCAACTT<br>TCTCAGCAAAATATTTGCTTGGGCACATAACCATGAAATCCC<br>CAAGAGAGTGATAAACTTGACTTCGTATTGTAGCATAATCGT<br>CGTCTTTAACGCCACTGAGGTAATGCTGGATTATTCCTTCCGG<br>ATCTGGAAATATTGTCCCGAAATAATCTCTCATGAGAGACTC<br>CCCTAACGTCTTATTAATGCGAGGATTTAATTCACCAAAAGG<br>GCCTGTGATCTGTGTAAAAGTGTTAACAAATGGGAATGATCC<br>CTCGTCTTGAGTAACTCCTATCATAGAATCAACACCGTTAAA<br>GTTTCCTTCTCTGATAGCTTTTTGTGGGTCTACCGGTAGAAAA<br>TCATCCCCACTGATGGTAAAGAAAGTTATCACTGGGAAGAC<br>GGGAACTAAGGAAAACGTAGTTCTCACCATATCTTTTGGAGC<br>TTTACTTCTTAGGCATTCGATCACATCATCAGGATTTTTCTTCA<br>GCGTTATGTTTTCATTTGCACATTCCATCTTCTCTGCGAGCTTT<br>TGTCCTAATTTAAGATTCTCAGCCCTATGGTCATTCAGAAAAT<br>ATGCTGGACAACCACTTTGCATTATTGCTCTCTGGAAGAGAC<br>CTTTCGAAAGAGGAGAAACACACAGCAAACCTGTAGCTACG<br>CCGCCGGCGCTGTTGCCGAACACCGTAATTCTTTTATTGTCTC<br>CGCCGAATGCAGCAATGTTTTCGTTCGTCCATTTAAGGGCTTC<br>CAAAATGTCATACAAGCCGACGTTTCCAGGAGCTTCTTCGGT<br>TTCAGATGTGAAGAATCCGAGCACACTTAACCGGTAGTTTAT<br>CGCAACAACTATTACATCTCCAATTGCAGTTAGAACACGTCC<br>ATCATATAGAGGCATCCTACCTGAACCGGTTGCAAAGCCGCC<br>TGAGAACACCCAAAACATGACGGCTTTTTTCTCGCTACTATT<br>GGAAATTTCTGGTGCCCATATATTCAAATACAAGCAGTCTTC<br>GCTTTGACCCTGAACTTTATCTTCCCACGGATAGTTGTCCGGA<br>GAGTACTGGTAACAAGCAGGCGGTAAGCTGTTTGCTATTACA

							GGAGAGGTCCAAGGTTTGACAGGAACTGTTCGCTTAAAGCGT<br>AAATTCCCCACTGGAGGCTCAGCAAACGGAATTCCGAGAAA<br>GACATTTAGATTTATGTCGTCATAAGTCATAACAGAACCAGT<br>CACTTCACCTGTAGGAGTATTAACTGTAACGCTGGATGCTAA<br>GATAGTTCCTGGTAGCAGTAAAAATAGTAGATGCAAGCTCTG<br>AAAAACAGAATTTAAGATACTTATTGCATTATTTAAAAAAGA<br>AATATCAGATAACTTATTTTGAGGACAATTTTTCAACTATTAT<br>TTAATATCCCTCAGAACTGTTACTATCAGCGACATAACCACA<br>ATTTTAACAATTCCCTCCTTCTCTGAAGCAAATTTCCATTTTCC<br>AGAAAGTGAGCCTCTTTTGTACTAAAATGGATTTTGTTACATC<br>TCTAGTTCTCAGAGTGCACTTACCAAGAGATCTTTTGGGAGA<br>GATTATTACTTCCCTCATCTTAATTCTTTGTTTATGATAGAGTT<br>TTATCTGTATGTAATTTCATTTTAATGACTAAAAGTTTTGGAT<br>AATTGTAAGTCCAATAACGCAACGCCTTATTTCAGAAATGCT<br>TACAAAAAAAACTGAGGTAGTCACTGTTGCACTTGCACAAC<br>CTGTTGACAGTTGCTCATTGGAATTCCATGGTGGCAATGGCA<br>TCTTCCTCAGTCTTCAATTATTCTTTTTGTAGATTACCAGGTTT<br>TGATTAATTACATTTACGTAAGTAGGAAATTACATATTGTGA<br>AAAATGATTTTAGATATTTTTAATAGCAGATAGACGGAATAT<br>TTCCGATGATATAAATAACTGAATATTTACGACACGCTTTTGC<br>ACTACAAGCGTTAAAGTTGTGGCAGTAATAAGATTGCAACA<br>ACTTTTGAGTAGTTTTACAAAGCGGAATCTAAAAAGTTAAGG<br>AATT

PhdEnzCho 23	W4VSJ0	8,73E-110	37,618	PF00135	63218.00<br><br>**58747.62**	MSSPSSNVIAGAQGL<br>GAAMCGKLQKNVH<br>KSLHLLFLLLPGTILA<br>**SSVTVNTPTGEVTG**<br>**SVMTYDDINLNVFL**<br>**GIPFAEPPVGNLRF**<br>**KRTVPVKPWTSPVI**<br>**ANSLPPACYQYSPD**<br>**NYPWEDKVQGQSE**<br>**DCLYLNIWAPEISN**<br>**SSEKKAVMFWVFS**<br>**GGFATGSGRMPLY**<br>**DGRVLTAIGDVIVV**<br>**AINYRLSVLGFFTSE**<br>**TEEAPGNVGLYDIL**<br>**EALKWTNENIAAFG**<br>**GDNKRITVFGNSA**<br>**GGVATGLLCVSPLS**<br>**KGLFQRAIMQSGC**<br>**PAYFLNDHRAENLK**<br>**LGQKLAEKMECAN**<br>**ENITLKKNPDDVIE**<br>**CLRSKAPKDMVRT**<br>**TFSLVPVFPVITFFTI**<br>**SGDDFLPVDPQKAI**<br>**REGNFNGVDSMIG**<br>**VTQDEGSFPFVNTF**<br>**TQITGPFGELNPRIN**	CGATAAACCTTTTGCAAGCAAGTTTCTCATGATCTGGCAATA<br>TTCTAGAAAGTGCAAACATGATTATGTAATATGAAAAATGCT<br>GTATATGAAACTCGAGGTATTTTTGATTTATTCTTTATACTTTC<br>ATATTACATATTCATCTTTACACTTTCATAACTATGCTTTCATG<br>AGTCTGCGTACATCAACATAACACATTAGAAAACTTTAATAT<br>CAATTCATATTCTCAATTTCATATTCTCAATTTAAGCTAGGAT<br>CCCCTTTATTTTGCAATTTAACTCAAAACTTCAAAGGAATAA<br>ACCTAAAATTATGCTTTTTTAACTACGGGAAGCTTCAAATTTT<br>ACGATAAACCTTTTGCAAGCAAGTTTCTCATGATCTGGCAAT<br>ATTCTAGAAAGTGCAAACATGATTATGTAATATGAAAAATGC<br>TGTATATGAAACTCAAGGTATTTTTGATTTATTTTTTATACTTT<br>CATATTACATAGTCATCTTTACACTTTCGTAATTCTGCGTATA<br>TCAACTGAAAACATTAGCAAACTTTAATATGCAATAAATAGC<br>TATTAGGATAACCTTTTCTTTTAATAACGTCACTATGCCTCTTT<br>TAAATAGAATTGACTAGCTTCGATGTTTACTCGGAGCTTATAT<br>TATTCCATTCTTCTCTCAATATGGCTTTCAACATAGCCTTTGA<br>AGCTATATTGTGTTGGCAGATTCTGCGTTCCAGCAAATCTCA<br>AAAACACTTTATCGGGTAAGTCAGGCGATTCTGGAGTTGTTT<br>ATGTGTAGGAGTAGATAACTATTTTAGCACGTCATATAAGAA<br>CTAGATATTCATAACTTGAACAGTGTGCTTGGGGACCTTGTC<br>CAATTAGAAATAATAACTGTTGTCTATCTTAAGTACTCTGCAT<br>TAAATTTCGTTTTAAAATGTTTAAATAATCCATTTTATTCAATT<br>GTGTCCTGAATAAAGTGTAGTTGTCCCACGCCTCAGTTTTAG<br>ATACACCCCCAAATAATAATACTTTAGCCACCGTGTTTTACA<br>GTTGGCATTAAATATTCTTACTTAAGCTCTTTAACAGGTTTTC<br>GGTACAAAAGCTTGAATCCTTTAATGTCAAATACGCAGAATC<br>TGCTCTAATCGGAAAATATTACCAGTTGCAGAGCTCATTTTG<br>CTTATTAATATATTCTTTTGCAACGTCAATGCTTAAATCTATT

						**KTLGESLMRDYFGT<br>IFPDPEGIIQHYLSG<br>VKDDDYATIRSQV<br>YHSLGDFMVMCPS<br>KYFAEKVAEKNNKI<br>YRYYFNHRPSSTPY<br>AEWMGVMHCDD<br>MQFIFGRPLTPLSN<br>YTRKEEELSFEMVK<br>EWTNFAKRGEPGET<br>WRKFSKSDPYVKVF<br>ELSENRTNYLKSSEE<br>NCEFFRPYFGF***	ATCTCAGGAAATGTAAAACAGCTAAAAGGGATGTTACAACA<br>GTTTCTCTTCGAGCCGTCACATTTATCTTTTGTTCTTTCGCCTC<br>TTGTGTTTTACCTATTCCTCGACATATTTATGGCTATTTTTCAG<br>TTCACAACTCTCTCGGATGGCGTAATCTTTTCTTGAAATTTCTT<br>CGAATTTTCTCTCGTTTTATATATATCAAAGCGGTAATGCGTT<br>TTGCATTTTGTTCCGTTTATAGTAACGTGCGCACCTAATACTT<br>TCACCTGCTTCATGAAGGACTGTATCAAATAGTCCGAAGTAT<br>GCTGCAGCTTTCCTGTCTTACTGTGGATCAGAATCTTTTTTTGC<br>AGCTTTATGGTATTCACTGCCTCAGTGACGAGACTTTGAAAA<br>TTTCAATCATGTTTTAAAATTCCGCATATATCAACACGAAAA<br>TGGTGTCTCCAAGGATTTGGAAAATTGAATTTCTACTCGCAA<br>GTTACCTTGGTGTTGCAATTAAAATGAGGTCCATCACACAAG<br>GATGGGATAAAGTCGAACACGTAAACTGTATAGCCTTCTACA<br>AATTTGTCTTTCGAGAAGTAAATACCTCTGTTTTGATTAAAGT<br>TGATAAACAATTTGGTGACAAGCGCGAATATATCTTTTAGAA<br>TCAAAATTCGGTTATAAATGTTTGCTTGGCACTGAGTGACCA<br>TCTACTTAAACACTTAAAAAGTTGACCATGATAATGATGAAA<br>AAAAGGAAGAGGGCAGGTTATGTTTCCTATTTACCGTGGCAT<br>TCTTCAGGGAGGTAATAATCACTCCTTTGGACATCAGTCACA<br>AAAATACATTGTCTTTCATAAAAGAAAATGTTCCAACTGAAA<br>CTGAGTATGCTTTATACAATACTCTGTCGATGGTATATTTCGA<br>GAGACTTTTTTCGAGAACATTGACATGTCCCAAGGATACGCC<br>GGGACATTCACTGTCCTAATTAACAAGGAGGCATGCTCAGAT<br>ACGATTTTATACTGGTCTCATTTTGGGGGGGGAGACTAAACA<br>AAAACTGTGTCTGTTGTGGCTCATTTTGATTCGCAGATCATCA<br>AATTTAATAAAAGTAGATTTTGTTGAAAACATTCGCATCATC<br>TCCAACGTTCGATTTTTAGCTGTAAGTTCATTACGCTTCGTCA<br>AACCTACATTTCTATCGATTTCTTTCAACGGGTTCGTCGAATG

							TAATTTGCTGTCTTTGTAAAAGCATTCAACGGGGGAGTAAAC TTTTCTTGACATTTTTTCCATATATCCAAATTCTTCAATATAGG TTCTGTAGGGATAAGTATTGCTATAGACGACGAGACGACTCG CTTCTTAAGTTGATTTCCACTCTGAAAAAAAGAGCGTGTAAA ATTAAATTCACGGGGTCGACGAGTTCATTTTCAGGTAAAGCG GATCTATCCGACTTCATAATTTTGGCTTTTACGTGCAGTTAGG AAGACGATAAGTCAATGTAATCTTCACCATTGTTACATTCCA CGGGTCCACCATCGCGTAGGTTGGACAGGGGAGGATATTCA ATAAAATGTCCTCTTCGATTGCGGTCTCGCATAAGCGGGATC ATGAACAAATGGAAATCAGATTTTGCGCAAGCAACACAGTA TTGATAGATGCGTGTCGATATGCTTAACCGAAAATAACGCGT GATTCCATTTTCTTGGTTCTTGGTGAACTAATGCGTTATTCCA ACGCTTTCTCTTTTAAATTATGACCAGAAGTAATAATGGACTT CATTTTTCAAGCTACTTTGTCCGTTAATTGGCTTCCAGCTTATT TCGAATAGCGTTTAACAGACATTTTTAGAACATTCAATATGC CCACACTGCTGTGAAATCATCGTTTGCCGACTACTTTTATGCC CAGTTTTAAAAGGGGAACTGTCACTCGATAAAAAGGACAAA AGAATCTGCCAAAACCATAAACTGTCTGAAATGTATCCCCTT TATAATAATTCAACTCGCCACGCTTTGCATAATCCTCGAAGG ATTTTGGACAACATTGATGAGGCACATACATCACGATAGAA ACTTTTGCCAAAAGTACAGTTTTACGTACGAGCGCTTGAGTT CAGATGACACAATTTCGCGTGTATGCATTCTTATAACGACAT TCAATACTTTTCTATTCCGTTGGAAGTAATGGAGTCTTTCATA TTGTGCTCAAATCATTTCCCCGTCGCTTCCGGTCACGCTCAAG ATGCGCAACAAGTGCGCTAACACATCTCCCACAATATCAGG CTCTACAATGTCCGAATATATCAACAGTAATCTATAATGAGC GCACAGATATGCCGCTTACTGACTTTCGACGTTCTTTTCTTCA TCATAATCATCATCGGTACTCTCAAGTTCAAACATAGCATTT

							GCGCGAGTCCGTCGTACAGTATCACTTTAGCACCCTGCAAAA<br>CAAAAACAGAATCTGCCCCTTCCGGTAGACGCACTATGTTAA<br>GAGTTCCCGAAATTGGAATCGATGCAAAACTTTGAGGGTAG<br>ACAGAATATAGTATATAGACAAAATATGTCCAGATCGAAAT<br>AGAGTTTTAGGTCCAGATCAAGCTTTCAAGTTGTGAGTCTAG<br>CATCGTTCTATGGTGGGAAATGTGTTAAAAATATTTTTTAAAT<br>GCTGCACCCTTTACTGTGTTGTTTACATGCAGTGTATATATTC<br>AGCTATATACATGCAGTGTATATATATTGTTTATATTCAGCTA<br>CTCTTCTCGGCTACAGCGGCAGATAATTCAATTAGCCATCTC<br>GCAAGAAAGGAAATATGACTGTGTTCCTGACTAAAGTACAC<br>CTGGCATGTATGCTTGTACCTAAAAGGTCCGAATTCGAGAAT<br>TTTCTCGAAAGATAATTAAGGAGCGTCAGGGAAATTGTTGGA<br>GGTCAGTGATGAAAGGGCGACGATTGTTTTTCTGACGTCATA<br>GGTTTGGGTTTTTTTTTTTTTACAACTTCCGTGTCGAAATTGCA<br>CTATTTCCTGCAGAGATCAGAGCTATATATTAACAGCGTTTC<br>AGGGTATTACTGTGCTATGAATATATTCCAGATTTTATGCGTG<br>TTTTAACGTATATCACTTGCAAAGATGTATTTTTATTGTCTTA<br>AAATACTACCATGATGTGGTGTACCTCGGAAAAATAAACTTA<br>CTTTTACATATATAAGAGTGTTTATTGCTGTAGCAGGAAAGTT<br>TACCTTCACTTTATTTAATGCTATATAATTGTACCTTACAAAA<br>TTTTGTAGCTTATAATAAAGTGAATAATCTTAATATTTCAACG<br>ATATTTGGAATGCTGTCAGCACAATTCAAACAACTTTCAGTG<br>AATTATAGTGATTTCTCTTTAACAAGAATGCGATTAAAATCC<br>AAAATAAGGTCGGAAAAATTCACAGTTTTCTTCGCTAGATTT<br>CAAGTAATTTGTACGGTTTTCAGATAGTTCAAAAACTTTGAC<br>GTATGGATCTGACTTAGAGAACTTCCGCCACGTTTCACCGGG<br>TTCCCCTCTCTTAGCAAAATTCGTCCACTCTTTCACCATCTCA<br>AAGCTTAGTTCTTCTTCCTTTCGGGTGTAGTTGGACAGAGGAG

							TTAGAGGACGTCCAAAGATAAATTGCATATCGTCACAATGC<br>ATAACCCCCATCCACTCTGCGTACGGAGTACTCGATGGTCTG<br>TGATTGAAATAGTATCGGTAGATTTTATTATTTTTTTCTGCAA<br>CTTTCTCAGCAAAATATTTGCTTGGGCACATAACCATGAAAT<br>CCCCAAGAGAGTGATAAACTTGACTTCGTATTGTAGCATAAT<br>CGTCGTCTTTAACGCCACTGAGGTAATGCTGGATTATTCCTTC<br>CGGATCTGGAAATATTGTCCCGAAATAATCTCTCATGAGAGA<br>CTCCCCTAACGTCTTATTAATGCGAGGATTTAATTCACCAAA<br>AGGGCCTGTGATCTGTGTAAAAGTGTTAACAAATGGGAATG<br>ATCCCTCGTCTTGAGTAACTCCTATCATAGAATCAACACCGTT<br>AAAGTTTCCTTCTCTGATAGCTTTTTGTGGGTCTACCGGTAGA<br>AAATCATCCCCACTGATGGTAAAGAAAGTTATCACTGGGAA<br>GACGGGAACTAAGGAAAACGTAGTTCTCACCATATCTTTTGG<br>AGCTTTACTTCTTAGGCATTCGATCACATCATCAGGATTTTTC<br>TTCAGCGTTATGTTTTCATTTGCACATTCCATCTTCTCTGCGAG<br>CTTTTGTCCTAATTTAAGATTCTCAGCCCTATGGTCATTCAGA<br>AAATATGCTGGACAACCACTTTGCATTATTGCTCTCTGGAAG<br>AGACCTTTCGAAAGAGGAGAAACACACAGCAAACCTGTAGC<br>TACGCCGCCGGCGCTGTTGCCGAACACCGTAATTCTTTTATTG<br>TCTCCGCCGAATGCAGCAATGTTTTCGTTCGTCCATTTAAGGG<br>CTTCCAAAATGTCATACAAGCCGACGTTTCCAGGAGCTTCTT<br>CGGTTTCAGATGTGAAGAATCCGAGCACACTTAACCGGTAGT<br>TTATCGCAACAACTATTACATCTCCAATTGCAGTTAGAACAC<br>GTCCATCATATAGAGGCATCCTACCTGAACCGGTTGCAAAGC<br>CGCCTGAGAACACCCAAAACATGACGGCTTTTTTCTCGCTAC<br>TATTGGAAATTTCTGGTGCCCATATATTCAAATACAAGCAGT<br>CTTCGCTTTGACCCTGAACTTTATCTTCCCACGGATAGTTGTC<br>CGGAGAGTACTGGTAACAAGCAGGCGGTAAGCTGTTTGCTAT

							TACAGGAGAGGTCCAAGGTTTGACAGGAACTGTTCGCTTAA<br>AGCGTAAATTCCCCACTGGAGGCTCAGCAAACGGAATTCCG<br>AGAAAGACATTTAGATTTATGTCGTCATAAGTCATAACAGAA<br>CCAGTCACTTCACCTGTAGGAGTATTAACTGTAACGCTGGAT<br>GCTAAGATAGTTCCTGGTAGCAGTAAAAATAGTAGATGCAA<br>GCTCTTATGAACGTTTTTCTGGAGCTTTCCGCACATAGCGGCT<br>CCTAAACCTTGGGCTCCAGCAATAACGTTAGACGAGGGAGA<br>CGACATGATGCTGCTGCCTAGATCTCCAGTGTGCTGTGTCGT<br>ACACAAATGCGCCTATTTCCTGTTTGTAAAGCTCCTTCGTACA<br>CCAGTATCGTCAACAGCTATTTTTACATTAGTTATTCCGTTTCT<br>GTCAGCAATCTTGCATCGCACAAATCTAAAAATAAATATGCG<br>AGTCTGCTGATTCACACTTGAATTTATAAGGCTTTCGAAACA<br>AAAAAGAACATTCG

PhdEnzCho 24	W4VSJ0	2,02E-65	35,676	PF00135	43419,31	SLYVTSRTRNIHIENF VLGLYDILEALKWT NENIAAFGGDNKRI TVFGNSAGGVATGL LCVSPLSKGLFQRAI MQSGCPAYFLNDHR AENLKLGQKLAEK MECANENITLKKNP DDVIECLRSKAPKD MVRTTFSLVPVFPVI TFFTISGDDFLPVDP QKAIREGNFNGVDS MIGVTQDEGSFPFV NTFTQITGPFGELNP RINKTLGESLMRDYF GTIFPDPEGIIQHYLS GVKDDDYATIRSQV YHSLGDFMVMCPSK YFAEKVAEKNNKIY RYYFNHRPSSTPYAE WMGVMHCDDMQF IFGRPLTPLSNYTRKE EELSFEMVKEWTNF AKRGEPGETWRKFS KSDPYVKVFELSENR TNYLKSSEENCEFFR PYFGF*	CGATAAACCTTTTGCAAGCAAGTTTCTCATGATCTGGCAATA TTCTAGAAAGTGCAAACATGATTATGTAATATGAAAAATGCT GTATATGAAACTCGAGGTATTTTTGATTTATTCTTTATACTTTC ATATTACATATTCATCTTTACACTTTCATAACTATGCTTTCATG AGTCTGCGTACATCAACATAACACATTAGAAAACTTTAATAT CAATTCATATTCTCAATTTCATATTCTCAATTTAAGCTAGGAT CCCCTTTATTTTGCAATTTAACTCAAAACTTCAAAGGAATAA ACCTAAAATTATGCTTTTTTAACTACGGGAAGCTTCAAATTTT ACGATAAACCTTTTGCAAGCAAGTTTCTCATGATCTGGCAAT ATTCTAGAAAGTGCAAACATGATTATGTAATATGAAAAATGC TGTATATGAAACTCAAGGTATTTTTGATTTATTTTTTATACTTT CATATTACATAGTCATCTTTACACTTTCGTAATGCTGCGTATA TCAACATTAGCAAACTTTAATATGCAATAAATAGCTATTAGG ATAACCTTTTCTTTTAATAACGTCACTATGCCTCTTTTAAATA GAATTGACTAGCTTCGATGTTTACTCGGAGCTTATATTATTCC ATTCTTCTCTCAATATGGCTTTCAACATAGCCTTTGAAGCTAT ATTGTGTTGGCAGATTCTGCGTTCCAGCAAATCTCAAAAACA CTTTATCGGGTAAGTCAGGCGATTCTGGAGTTGTTTATGTGTA GGAGTAGATAACTATTTTAGCACGTCATATAAGAACTAGATA TTCATAACTTGAACAGTGTGCTTGGGGACCTTGTCCAATTAG AAATAATAACTGTTGTCTATCTTAAGTACTCTGCATTAAATTT CGTTTTAAAATGTTTAAATAATCCATTTTATTCAATTGTGTCCT GAATAAAGTGTAGTTGTCCCACGCCTCAGTTTTAGATACACC CCCAAATAATAATACTTTAGCCACCGTGTTTTACAGTTGGCA TTAAATATTCTTACTTAAGCTCTTTAACAGGTTTTCGGTACAA AAGCTTGAATCCTTTAATGTCAAATACGCAGAATCTGCTCTA ATCGGAAAATATTACCAGTTGCAGAGCTCATTTTGCTTATTA ATATATTCTTTTGCAACGTCAATGCTTAAATCTATTATCTCAG

							GAAATGTAAAACAGCTAAAAGGGATGTTACAACAGTTTCTCT<br>TCGAGCCGTCACATTTATCTTTTGTTCTTTCGCCTCTTGTGTTT<br>TACCTATTCCTCGACATATTTATGGCTATTTTTCAGTTCACAA<br>CTCTCTCGGATGGCGTAATCTTTTCTTGAAATTTCTTCGAATTT<br>TCTCTCGTTTTATATATATCAAAGCGGTAATGCGTTTTGCATTT<br>TGTTCCGTTTATAGTAACGTGCGCACCTAATACTTTCACCTGC<br>TTCATGAAGGACTGTATCAAATAGTCCGAAGTATGCTGCAGC<br>TTTCCTGTCTTACTGTGGATCAGAATCTTTTTTTGCAGCTTTAT<br>GGTATTCACTGCCTCAGTGACGAGACTTTGAAAATTTCAATC<br>ATGTTTTAAAATTCCGCATATATCAACACGAAAATGGTGTCT<br>CCAAGGATTTGGAAAATTGAATTTCTACTCGCAAGTTACCTT<br>GGTGTTGCAATTAAAATGAGGTCCATCACACAAGGATGGGA<br>TAAAGTCGAACACGTAAACTGTATAGCCTTCTACAAATTTGT<br>CTTTCGAGAAGTAAATACCTCTGTTTTGATTAAAGTTGATAA<br>ACAATTTGGTGACAAGCGCGAATATATCTTTTAGAATCAAAA<br>TTCGGTTATAAATGTTTGCTTGGCACTGAGTGACCATCTACTT<br>AAACACTTAAAAAGTTGACCATGATAATGATGAAAAAAAGG<br>AAGAGGGCAGGTTATGTTTCCTATTTACCGTGGCATTCTTCAG<br>GGAGGTAATAATCACTCCTTTGGACATCAGTCACAAAAATAC<br>ATTGTCTTTCATAAAAGAAAATGTTCCAACTGAAACTGAGTA<br>TGCTTTATACAATACTCTGTCGATGGTATATTTCGAGAGACTT<br>TTTTCGAGAACATTGACATGTCCCAAGGATACGCCGGGACAT<br>TCACTGTCCTAATTAACAAGGAGGCATGCTCAGATACGATTT<br>TATACTGGTCTCATTTTGGGGGGGGAGACTAAACAAAAACTG<br>TGTCTGTTGTGGCTCATTTTGATTCGCAGATCATCAAATTTAA<br>TAAAAGTAGATTTTGTTGAAAACATTCGCATCATCTCCAACG<br>TTCGATTTTTAGCTGTAAGTTCATTACGCTTCGTCAAACCTAC<br>ATTTCTATCGATTTCTTTCAACGGGTTCGTCGAATGTAATTTG

							CTGTCTTTGTAAAAGCATTCAACGGGGGAGTAAACTTTTCTT<br>GACATTTTTTCCATATATCCAAATTCTTCAATATAGGTTCTGT<br>AGGGATAAGTATTGCTATAGACGACGAGACGACTCGCTTCTT<br>AAGTTGATTTCCACTCTGAAAAAAAGAGCGTGTAAAATTAA<br>ATTCACGGGGTCGACGAGTTCATTTTCAGGTAAAGCGGATCT<br>ATCCGACTTCATAATTTTGGCTTTTACGTGCAGTTAGGAAGAC<br>GATAAGTCAATGTAATCTTCACCATTGTTACATTCCACGGGT<br>CCACCATCGCGTAGGTTGGACAGGGGAGGATATTCAATAAA<br>ATGTCCTCTTCGATTGCGGTCTCGCATAAGCGGGATCATGAA<br>CAAATGGAAATCAGATTTTGCGCAAGCAACACAGTATTGAT<br>AGATGCGTGTCGATATGCTTAACCGAAAATAACGCGTGATTC<br>CATTTTCTTGGTTCTTGGTGAACTAATGCGTTATTCCAACGCT<br>TTCTCTTTTAAATTATGACCAGAAGTAATAATGGACTTCATTT<br>TTCAAGCTACTTTGTCCGTTAATTGGCTTCCAGCTTATTTCGA<br>ATAGCGTTTAACAGACATTTTTAGAACATTCAATATGCCCAC<br>ACTGCTGTGAAATCATCGTTTGCCGACTACTTTTATGCCCAGT<br>TTTAAAAGGGGAACTGTCACTCGATAAAAAGGACAAAAGAA<br>TCTGCCAAAACCATAAACTGTCTGAAATGTATCCCCTTTATA<br>ATAATTCAACTCGCCACGCTTTGCATAATCCTCGAAGGATTTT<br>GGACAACATTGATGAGGCACATACATCACGATAGAAACTTT<br>TGCCAAAAGTACAGTTTTACGTACGAGCGCTTGAGTTCAGAT<br>GACACAATTTCGCGTGTATGCATTCTTATAACGACATTCAAT<br>ACTTTTCTATTCCGTTGGAAGTAATGGAGTCTTTCATATTGTG<br>CTCAAATCATTTCCCCGTCGCTTCCGGTCACGCTCAAGATGC<br>GCAACAAGTGCGCTAACACATCTCCCACAATATCAGGCTCTA<br>CAATGTCCGAATATATCAACAGTAATCTATAATGAGCGCACA<br>GATATGCCGCTTACTGACTTTCGACGTTCTTTTCTTCATCATA<br>ATCATCATCGGTACTCTCAAGTTCAAACATAGCATTTGCGCG

							AGTCCGTCGTACAGTATCACTTTAGCACCCTGCAAAACAAAA<br>ACAGAATCTGCCCCTTCCGGTAGACGCACTATGTTAAGAGTT<br>CCCGAAATTGGAATCGATGCAAAACTTTGAGGGTAGACAGA<br>ATATAGTATATAGACAAAATATGTCCAGATCGAAATAGAGTT<br>TTAGGTCCAGATCAAGCTTTCAAGTTGTGAGTCTAGCATCGTT<br>CTATGGTGGGAAATGTGTTAAAAATATTTTTTAAATGCTGCA<br>CCCTTTACTGTGTTGTTTACATGCAGTGTATATATTCAGCTAT<br>ATACATGCAGTGTATATATATTGTTTATATTCAGCTACTCTTC<br>TCGGCTACAGCGGCAGATAATTCAATTAGCCATCTCGCAAGA<br>AAGGAAATATGACTGTGTTCCTGACTAAAGTACACCTGGCAT<br>GTATGCTTGTACCTAAAAGGTCCGAATTCGAGAATTTTCTCG<br>AAAGATAATTAAGGAGCGTCAGGGAAATTGTTGGAGGTCAG<br>TGATGAAAGGGCGACGATTGTTTTTCTGACGTCATAGGTCTG<br>GGTTTTTTTTTTTACAACTTCCGTGTCGAAATTGCACTATTTCC<br>TGCAGAGATCAGAGCTATATATTAACAGCGTTTCAGGGTATT<br>ACTGTGCTATGAATATATTCCAGATTTTATGCGTGTTTTAACG<br>TATATCACTTGCAAAGATGTATTTTTATTGTCTTAAAATACTA<br>CCATGATGTGGTGTACCTCGGAAAAATAAACTTACTTTTACA<br>TATATAAGAGTGTTTATTGCTGTAGCAGGAAAGTTTACCTTCA<br>CTTTATTTAATGCTATATAATTGTACCTTACAAAATTTTGTAG<br>CTTATAATAAAGTGAATAATCTTAATATTTCAACGATATTTGG<br>AATGCTGTCAGCACAATTCAAACAACTTTCAGTGAATTATAG<br>TGATTTCTCTTTAACAAGAATGCGATTAAAATCCAAAATAAG<br>GTCGGAAAAATTCACAGTTTTCTTCGCTAGATTTCAAGTAATT<br>TGTACGGTTTTCAGATAGTTCAAAAACTTTGACGTATGGATCT<br>GACTTAGAGAACTTCCGCCACGTTTCACCGGGTTCCCCTCTCT<br>TAGCAAAATTCGTCCACTCTTTCACCATCTCAAAGCTTAGTTC<br>TTCTTCCTTTCGGGTGTAGTTGGACAGAGGAGTTAGAGGACG

							TCCAAAGATAAATTGCATATCGTCACAATGCATAACCCCCATCCACTCTGCGTACGGAGTACTCGATGGTCTGTGATTGAAATAGTATCGGTAGATTTTATTATTTTTTTCTGCAACTTTCTCAGCAAAATATTTGCTTGGGCACATAACCATGAAATCCCCAAGAGAGTGATAAACTTGACTTCGTATTGTAGCATAATCGTCGTCTTTAACGCCACTGAGGTAATGCTGGATTATTCCTTCCGGATCTGGAAATATTGTCCCGAAATAATCTCTCATGAGAGACTCCCCTAACGTCTTATTAATGCGAGGATTTAATTCACCAAAAGGGCCTGTGATCTGTGTAAAAGTGTTAACAAATGGGAATGATCCCTCGTCTTGAGTAACTCCTATCATAGAATCAACACCGTTAAAGTTTCCTTCTCTGATAGCTTTTTGTGGGTCTACCGGTAGAAAATCATCCCCACTGATGGTAAAGAAAGTTATCACTGGGAAGACGGGAACTAAGGAAAACGTAGTTCTCACCATATCTTTTGGAGCTTTACTTCTTAGGCATTCGATCACATCATCAGGATTTTTCTTCAGCGTTATGTTTTCATTTGCACATTCCATCTTCTCTGCGAGCTTTTGTCCTAATTTAAGATTCTCAGCCCTATGGTCATTCAGAAAATATGCTGGACAACCACTTTGCATTATTGCTCTCTGGAAGAGACCTTTCGAAAGAGGAGAAACACACAGCAAACCTGTAGCTACGCCGCCGGCGCTGTTGCCGAACACCGTAATTCTTTTATTGTCTCCGCCGAATGCAGCAATGTTTTCGTTCGTCCATTTAAGGGCTTCCAAAATGTCATACAAGCCTAGAACGAAATTCTCGATATGAATATTTCTTGTACGAGAAGTTACATACAAGGATTATTCTTGATAAAATATCAGTCATTTATTTTTTTAGATTTATCCATATAGGTTCTAAATAGAATATTTAATATTTAATATAAATATTAATGCCTAACAGCACAGGGCAGAA

PhdEnzCho 25	W4VSJ0	5,20E-15	33,571	PF00135	15697,49	KMECANENITLKSNPDDVTECLRSKDPKELVTTTFTLIPSFSAVTFFTITGDDFLPTDPQKAIREGNFNGVDSIIGVTQDEGSFLLANNFLQIAGPLGQLNSHINKTLGESLMREYFQSIFPDPEGIIQHYLTGVADDDYDT	CTGTATCATAATCGTCATCTGCAACACCAGTGAGATAATGCTGGATTATTCCTTCCGGATCTGGAAATATTGACTGGAAATACTCTCTCATGAGGGACTCCCCAAGTGTCTTGTTAATGTGAGAATTTAATTGACCAAGAGGGCCTGCGATCTGAAGAAAATTATTAGCAAGTAGGAATGATCCCTCATCTTGAGTAACTCCTATGATAGAATCAACACCGTTGAAGTTGCCTTCTCTGATAGCTTTCTGTGGGTCTGTAGGAAGAAAATCATCTCCAGTGATAGTAAAGAAAGTTACCGCTGAGAAGCTGGGAATTAAGGTAAACGTAGTTGTTACTAGTTCTTTTGGATCTTTACTTCTTAGACATTCGGTCACATCATCAGGATTCGACTTCAACGTTATGTTTTCGTTCGCACATTCCATCTTC

PhdEnzCho 26	W4VSJ0	8,76E-110	37,618	PF00135	63218.00<br><br>**58747.62**	MSSPSSNVIAGAQGL<br>GAAMCGKLQKNVH<br>KSLHLLFLLLPGTILA<br>**SSVTVNTPTGEVTG**<br>**SVMTYDDINLNVFL**<br>**GIPFAEPPVGNLRF**<br>**KRTVPVKPWTSPVI**<br>**ANSLPPACYQYSPD**<br>**NYPWEDKVQGQSE**<br>**DCLYLNIWAPEISN**<br>**SSEKKAVMFWVFS**<br>**GGFATGSGRMPLY**<br>**DGRVLTAIGDVIVV**<br>**AINYRLSVLGFFTSE**<br>**TEEAPGNVGLYDIL**<br>**EALKWTNENIAAFG**<br>**GDNKRITVFGNSA**<br>**GGVATGLLCVSPLS**<br>**KGLFQRAIMQSGC**<br>**PAYFLNDHRAENLK**<br>**LGQKLAEKMECAN**<br>**ENITLKKNPDDVIE**<br>**CLRSKAPKDMVRT**<br>**TFSLVPVFPVITFFTI**<br>**SGDDFLPVDPQKAI**<br>**REGNFNGVDSMIG**<br>**VTQDEGSFPFVNTF**<br>**TQITGPFGELNPRIN**	CGATAAACCTTTTGCAAGCAAGTTTCTCATGATCTGGCAATA<br>TTCTAGAAAGTGCAAACATGATTATGTAATATGAAAAATGCT<br>GTATATGAAACTCGAGGTATTTTTGATTTATTCTTTATACTTTC<br>ATATTACATATTCATCTTTACACTTTCATAACTATGCTTTCATG<br>AGTCTGCGTACATCAACATAACACATTAGAAAACTTTAATAT<br>CAATTCATATTCTCAATTTCATATTCTCAATTTAAGCTAGGAT<br>CCCCTTTATTTTGCAATTTAACTCAAAACTTCAAAGGAATAA<br>ACCTAAAATTATGCTTTTTTAACTACGGGAAGCTTCAAATTTT<br>ACGATAAACCTTTTGCAAGCAAGTTTCTCATGATCTGGCAAT<br>ATTCTAGAAAGTGCAAACATGATTATGTAATATGAAAAATGC<br>TGTATATGAAACTCAAGGTATTTTTGATTTATTTTTTATACTTT<br>CATATTACATAGTCATCTTTACACTTTCGTAATGCTGCGTATA<br>TCAACATTAGCAAACTTTAATATGCAATAAATAGCTATTAGG<br>ATAACCTTTTCTTTTAATAACGTCACTATGCCTCTTTTAAATA<br>GAATTGACTAGCTTCGATGTTTACTCGGAGCTTATATTATTCC<br>ATTCTTCTCTCAATATGGCTTTCAACATAGCCTTTGAAGCTAT<br>ATTGTGTTGGCAGATTCTGCGTTCCAGCAAATCTCAAAAACA<br>CTTTATCGGGTAAGTCAGGCGATTCTGGAGTTGTTTATGTGTA<br>GGAGTAGATAACTATTTTAGCACGTCATATAAGAACTAGATA<br>TTCATAACTTGAACAGTGTGCTTGGGGACCTTGTCCAATTAG<br>AAATAATAACTGTTGTCTATCTTAAGTACTCTGCATTAAATTT<br>CGTTTTAAAATGTTTAAATAATCCATTTTATTCAATTGTGTCCT<br>GAATAAAGTGTAGTTGTCCCACGCCTCAGTTTTAGATACACC<br>CCCAAATAATAATACTTTAGCCACCGTGTTTTACAGTTGGCA<br>TTAAATATTCTTACTTAAGCTCTTTAACAGGTTTTCGGTACAA<br>AAGCTTGAATCCTTTAATGTCAAATACGCAGAATCTGCTCTA<br>ATCGGAAAATATTACCAGTTGCAGAGCTCATTTTGCTTATTA<br>ATATATTCTTTTGCAACGTCAATGCTTAAATCTATTATCTCAG

						**KTLGESLMRDYFGT<br>IFPDPEGIIQHYLSG<br>VKDDDYATIRSQV<br>YHSLGDFMVMCPS<br>KYFAEKVAEKNNKI<br>YRYYFNHRPSSTPY<br>AEWMGVMHCDD<br>MQFIFGRPLTPLSN<br>YTRKEEELSFEMVK<br>EWTNFAKRGEPGET<br>WRKFSKSDPYVKVF<br>ELSENRTNYLKSSEE<br>NCEFFRPYFGF***	GAAATGTAAAACAGCTAAAAGGGATGTTACAACAGTTTCTCT<br>TCGAGCCGTCACATTTATCTTTTGTTCTTTCGCCTCTTGTGTTT<br>TACCTATTCCTCGACATATTTATGGCTATTTTTCAGTTCACAA<br>CTCTCTCGGATGGCGTAATCTTTTCTTGAAATTTCTTCGAATTT<br>TCTCTCGTTTTATATATATCAAAGCGGTAATGCGTTTTGCATTT<br>TGTTCCGTTTATAGTAACGTGCGCACCTAATACTTTCACCTGC<br>TTCATGAAGGACTGTATCAAATAGTCCGAAGTATGCTGCAGC<br>TTTCCTGTCTTACTGTGGATCAGAATCTTTTTTTGCAGCTTTAT<br>GGTATTCACTGCCTCAGTGACGAGACTTTGAAAATTTCAATC<br>ATGTTTTAAAATTCCGCATATATCAACACGAAAATGGTGTCT<br>CCAAGGATTTGGAAAATTGAATTTCTACTCGCAAGTTACCTT<br>GGTGTTGCAATTAAAATGAGGTCCATCACACAAGGATGGGA<br>TAAAGTCGAACACGTAAACTGTATAGCCTTCTACAAATTTGT<br>CTTTCGAGAAGTAAATACCTCTGTTTTGATTAAAGTTGATAA<br>ACAATTTGGTGACAAGCGCGAATATATCTTTTAGAATCAAAA<br>TTCGGTTATAAATGTTTGCTTGGCACTGAGTGACCATCTACTT<br>AAACACTTAAAAAGTTGACCATGATAATGATGAAAAAAAGG<br>AAGAGGGCAGGTTATGTTTCCTATTTACCGTGGCATTCTTCAG<br>GGAGGTAATAATCACTCCTTTGGACATCAGTCACAAAAATAC<br>ATTGTCTTTCATAAAAGAAAATGTTCCAACTGAAACTGAGTA<br>TGCTTTATACAATACTCTGTCGATGGTATATTTCGAGAGACTT<br>TTTTCGAGAACATTGACATGTCCCAAGGATACGCCGGGACAT<br>TCACTGTCCTAATTAACAAGGAGGCATGCTCAGATACGATTT<br>TATACTGGTCTCATTTTGGGGGGGGAGACTAAACAAAAACTG<br>TGTCTGTTGTGGCTCATTTTGATTCGCAGATCATCAAATTTAA<br>TAAAAGTAGATTTTGTTGAAAACATTCGCATCATCTCCAACG<br>TTCGATTTTTAGCTGTAAGTTCATTACGCTTCGTCAAACCTAC<br>ATTTCTATCGATTTCTTTCAACGGGTTCGTCGAATGTAATTTG

							CTGTCTTTGTAAAAGCATTCAACGGGGGAGTAAACTTTTCTT<br>GACATTTTTTCCATATATCCAAATTCTTCAATATAGGTTCTGT<br>AGGGATAAGTATTGCTATAGACGACGAGACGACTCGCTTCTT<br>AAGTTGATTTCCACTCTGAAAAAAAGAGCGTGTAAAATTAA<br>ATTCACGGGGTCGACGAGTTCATTTTCAGGTAAAGCGGATCT<br>ATCCGACTTCATAATTTTGGCTTTTACGTGCAGTTAGGAAGAC<br>GATAAGTCAATGTAATCTTCACCATTGTTACATTCCACGGGT<br>CCACCATCGCGTAGGTTGGACAGGGGAGGATATTCAATAAA<br>ATGTCCTCTTCGATTGCGGTCTCGCATAAGCGGGATCATGAA<br>CAAATGGAAATCAGATTTTGCGCAAGCAACACAGTATTGAT<br>AGATGCGTGTCGATATGCTTAACCGAAAATAACGCGTGATTC<br>CATTTTCTTGGTTCTTGGTGAACTAATGCGTTATTCCAACGCT<br>TTCTCTTTTAAATTATGACCAGAAGTAATAATGGACTTCATTT<br>TTCAAGCTACTTTGTCCGTTAATTGGCTTCCAGCTTATTTCGA<br>ATAGCGTTTAACAGACATTTTTAGAACATTCAATATGCCCAC<br>ACTGCTGTGAAATCATCGTTTGCCGACTACTTTTATGCCCAGT<br>TTTAAAAGGGGAACTGTCACTCGATAAAAAGGACAAAAGAA<br>TCTGCCAAAACCATAAACTGTCTGAAATGTATCCCCTTTATA<br>ATAATTCAACTCGCCACGCTTTGCATAATCCTCGAAGGATTTT<br>GGACAACATTGATGAGGCACATACATCACGATAGAAACTTT<br>TGCCAAAAGTACAGTTTTACGTACGAGCGCTTGAGTTCAGAT<br>GACACAATTTCGCGTGTATGCATTCTTATAACGACATTCAAT<br>ACTTTTCTATTCCGTTGGAAGTAATGGAGTCTTTCATATTGTG<br>CTCAAATCATTTCCCCGTCGCTTCCGGTCACGCTCAAGATGC<br>GCAACAAGTGCGCTAACACATCTCCCACAATATCAGGCTCTA<br>CAATGTCCGAATATATCAACAGTAATCTATAATGAGCGCACA<br>GATATGCCGCTTACTGACTTTCGACGTTCTTTTCTTCATCATA<br>ATCATCATCGGTACTCTCAAGTTCAAACATAGCATTTGCGCG

							AGTCCGTCGTACAGTATCACTTTAGCACCCTGCAAAACAAAA<br>ACAGAATCTGCCCCTTCCGGTAGACGCACTATGTTAAGAGTT<br>CCCGAAATTGGAATCGATGCAAAACTTTGAGGGTAGACAGA<br>ATATAGTATATAGACAAAATATGTCCAGATCGAAATAGAGTT<br>TTAGGTCCAGATCAAGCTTTCAAGTTGTGAGTCTAGCATCGTT<br>CTATGGTGGGAAATGTGTTAAAAATATTTTTTAAATGCTGCA<br>CCCTTTACTGTGTTGTTTACATGCAGTGTATATATTCAGCTAT<br>ATACATGCAGTGTATATATATTGTTTATATTCAGCTACTCTTC<br>TCGGCTACAGCGGCAGATAATTCAATTAGCCATCTCGCAAGA<br>AAGGAAATATGACTGTGTTCCTGACTAAAGTACACCTGGCAT<br>GTATGCTTGTACCTAAAAGGTCCGAATTCGAGAATTTTCTCG<br>AAAGATAATTAAGGAGCGTCAGGGAAATTGTTGGAGGTCAG<br>TGATGAAAGGGCGACGATTGTTTTTCTGACGTCATAGGTCTG<br>GGTTTTTTTTTTTACAACTTCCGTGTCGAAATTGCACTATTTCC<br>TGCAGAGATCAGAGCTATATATTAACAGCGTTTCAGGGTATT<br>ACTGTGCTATGAATATATTCCAGATTTTATGCGTGTTTTAACG<br>TATATCACTTGCAAAGATGTATTTTTATTGTCTTAAAATACTA<br>CCATGATGTGGTGTACCTCGGAAAAATAAACTTACTTTTACA<br>TATATAAGAGTGTTTATTGCTGTAGCAGGAAAGTTTACCTTCA<br>CTTTATTTAATGCTATATAATTGTACCTTACAAAATTTTGTAG<br>CTTATAATAAAGTGAATAATCTTAATATTTCAACGATATTTGG<br>AATGCTGTCAGCACAATTCAAACAACTTTCAGTGAATTATAG<br>TGATTTCTCTTTAACAAGAATGCGATTAAAATCCAAAATAAG<br>GTCGGAAAAATTCACAGTTTTCTTCGCTAGATTTCAAGTAATT<br>TGTACGGTTTTCAGATAGTTCAAAAACTTTGACGTATGGATCT<br>GACTTAGAGAACTTCCGCCACGTTTCACCGGGTTCCCCTCTCT<br>TAGCAAAATTCGTCCACTCTTTCACCATCTCAAAGCTTAGTTC<br>TTCTTCCTTTCGGGTGTAGTTGGACAGAGGAGTTAGAGGACG

							TCCAAAGATAAATTGCATATCGTCACAATGCATAACCCCCAT<br>CCACTCTGCGTACGGAGTACTCGATGGTCTGTGATTGAAATA<br>GTATCGGTAGATTTTATTATTTTTTTCTGCAACTTTCTCAGCAA<br>AATATTTGCTTGGGCACATAACCATGAAATCCCCAAGAGAGT<br>GATAAACTTGACTTCGTATTGTAGCATAATCGTCGTCTTTAAC<br>GCCACTGAGGTAATGCTGGATTATTCCTTCCGGATCTGGAAA<br>TATTGTCCCGAAATAATCTCTCATGAGAGACTCCCCTAACGT<br>CTTATTAATGCGAGGATTTAATTCACCAAAAGGGCCTGTGAT<br>CTGTGTAAAAGTGTTAACAAATGGGAATGATCCCTCGTCTTG<br>AGTAACTCCTATCATAGAATCAACACCGTTAAAGTTTCCTTCT<br>CTGATAGCTTTTTGTGGGTCTACCGGTAGAAAATCATCCCCA<br>CTGATGGTAAAGAAAGTTATCACTGGGAAGACGGGAACTAA<br>GGAAAACGTAGTTCTCACCATATCTTTTGGAGCTTTACTTCTT<br>AGGCATTCGATCACATCATCAGGATTTTTCTTCAGCGTTATGT<br>TTTCATTTGCACATTCCATCTTCTCTGCGAGCTTTTGTCCTAAT<br>TTAAGATTCTCAGCCCTATGGTCATTCAGAAAATATGCTGGA<br>CAACCACTTTGCATTATTGCTCTCTGGAAGAGACCTTTCGAA<br>AGAGGAGAAACACACAGCAAACCTGTAGCTACGCCGCCGGC<br>GCTGTTGCCGAACACCGTAATTCTTTTATTGTCTCCGCCGAAT<br>GCAGCAATGTTTTCGTTCGTCCATTTAAGGGCTTCCAAAATGT<br>CATACAAGCCGACGTTTCCAGGAGCTTCTTCGGTTTCAGATG<br>TGAAGAATCCGAGCACACTTAACCGGTAGTTTATCGCAACA<br>ACTATTACATCTCCAATTGCAGTTAGAACACGTCCATCATAT<br>AGAGGCATCCTACCTGAACCGGTTGCAAAGCCGCCTGAGAA<br>CACCCAAAACATGACGGCTTTTTTCTCGCTACTATTGGAAATT<br>TCTGGTGCCCATATATTCAAATACAAGCAGTCTTCGCTTTGAC<br>CCTGAACTTTATCTTCCCACGGATAGTTGTCCGGAGAGTACT<br>GGTAACAAGCAGGCGGTAAGCTGTTTGCTATTACAGGAGAG

							GTCCAAGGTTTGACAGGAACTGTTCGCTTAAAGCGTAAATTCCCCACTGGAGGCTCAGCAAACGGAATTCCGAGAAAGACATTTAGATTTATGTCGTCATAAGTCATAACAGAACCAGTCACTTCACCTGTAGGAGTATTAACTGTAACGCTGGATGCTAAGATAGTTCCTGGTAGCAGTAAAAATAGTAGATGCAAGCTCTTATGAACGTTTTTCTGGAGCTTTCCGCACATAGCGGCTCCTAAACCTTGGGCTCCAGCAATAACGTTAGACGAGGGAGACGACATGATGCTGCTGCCTAGATCTCCAGTGTGCTGTGTCGTACACAAATGCGCCTATTTCCTGTTTGTAAAGCTCCTTCGTACACCAGTATCGTCAACAGCTATTTTTACATTAGTTATTCCGTTTCTGTCAGCAATCTTGCATCGCACAAATCTGCTAGTGTAGGTCTTCATCCGAATTATGCCCAAACTGGTTGTAGTTTTACTGTGGATAAGAAGCTTTCTTAACCTAATTTGTTCTCCACAGGCT

PhdEnzCho 27	W4VSJ0	1,08E-109	37,618	PF00135	63218.00<br><br>**58747.62**	MSSPSSNVIAGAQGL<br>GAAMCGKLQKNVH<br>KSLHLLFLLLPGTILA<br>**SSVTVNTPTGEVTG**<br>**SVMTYDDINLNVFL**<br>**GIPFAEPPVGNLRF**<br>**KRTVPVKPWTSPVI**<br>**ANSLPPACYQYSPD**<br>**NYPWEDKVQGQSE**<br>**DCLYLNIWAPEISN**<br>**SSEKKAVMFWVFS**<br>**GGFATGSGRMPLY**<br>**DGRVLTAIGDVIVV**<br>**AINYRLSVLGFFTSE**<br>**TEEAPGNVGLYDIL**<br>**EALKWTNENIAAFG**<br>**GDNKRITVFGNSA**<br>**GGVATGLLCVSPLS**<br>**KGLFQRAIMQSGC**<br>**PAYFLNDHRAENLK**<br>**LGQKLAEKMECAN**<br>**ENITLKKNPDDVIE**<br>**CLRSKAPKDMVRT**<br>**TFSLVPVFPVITFFTI**<br>**SGDDFLPVDPQKAI**<br>**REGNFNGVDSMIG**<br>**VTQDEGSFPFVNTF**<br>**TQITGPFGELNPRIN**	CGATAAACCTTTTGCAAGCAAGTTTCTCATGATCTGGCAATA<br>TTCTAGAAAGTGCAAACATGATTATGTAATATGAAAAATGCT<br>GTATATGAAACTCGAGGTATTTTTGATTTATTCTTTATACTTTC<br>ATATTACATATTCATCTTTACACTTTCATAACTATGCTTTCATG<br>AGTCTGCGTACATCAACATAACACATTAGAAAACTTTAATAT<br>GCAATTCATATTCTCAATTTAAGCTAGGATCCCCTTTATTTTG<br>CAATTTAACTCAAAACTTCAAAGGAATAAACCTAAAATTATG<br>CTTTTTTAACTACGGGAAGCTTCAAATTTTACGATAAACCTTT<br>TGCAAGCAAGTTTCTCATGATCTGGCAATATTCTAGAAAGTG<br>CAAACATGATTATGTAATATGAAAAATGCTGTATATGAAACT<br>CAAGGTATTTTTGATTTATTTTTTATACTTTCATATTACATAGT<br>CATCTTTACACTTTCGTAATTCTGCGTATATCAACTGAAAACA<br>TTAGCAAACTTTAATATGCAATAAATAGCTATTAGGATAACC<br>TTTTCTTTTAATAACGTCACTATGCCTCTTTTAAATAGAATTG<br>ACTAGCTTCGATGTTTACTCGGAGCTTATATTATTCCATTCTTC<br>TCTCAATATGGCTTTCAACATAGCCTTTGAAGCTATATTGTGT<br>TGGCAGATTCTGCGTTCCAGCAAATCTCAAAAACACTTTATC<br>GGGTAAGTCAGGCGATTCTGGAGTTGTTTATGTGTAGGAGTA<br>GATAACTATTTTAGCACGTCATATAAGAACTAGATATTCATA<br>ACTTGAACAGTGTGCTTGGGGACCTTGTCCAATTAGAAATAA<br>TAACTGTTGTCTATCTTAAGTACTCTGCATTAAATTTCGTTTTA<br>AAATGTTTAAATAATCCATTTTATTCAATTGTGTCCTGAATAA<br>AGTGTAGTTGTCCCACGCCTCAGTTTTAGATACACCCCCAAA<br>TAATAATACTTTAGCCACCGTGTTTTACAGTTGGCATTAAATA<br>TTCTTACTTAAGCTCTTTAACAGGTTTTCGGTACAAAAGCTTG<br>AATCCTTTAATGTCAAATACGCAGAATCTGCTCTAATCGGAA<br>AATATTACCAGTTGCAGAGCTCATTTTGCTTATTAATATATTC<br>TTTTGCAACGTCAATGCTTAAATCTATTATCTCAGGAAATGTA

						**KTLGESLMRDYFGT<br>IFPDPEGIIQHYLSG<br>VKDDDYATIRSQV<br>YHSLGDFMVMCPS<br>KYFAEKVAEKNNKI<br>YRYYFNHRPSSTPY<br>AEWMGVMHCDD<br>MQFIFGRPLTPLSN<br>YTRKEEELSFEMVK<br>EWTNFAKRGEPGET<br>WRKFSKSDPYVKVF<br>ELSENRTNYLKSSEE<br>NCEFFRPYFGF***	AAACAGCTAAAAGGGATGTTACAACAGTTTCTCTTCGAGCCG<br>TCACATTTATCTTTTGTTCTTTCGCCTCTTGTGTTTTACCTATTC<br>CTCGACATATTTATGGCTATTTTTCAGTTCACAACTCTCTCGG<br>ATGGCGTAATCTTTTCTTGAAATTTCTTCGAATTTTCTCTCGTT<br>TTATATATATCAAAGCGGTAATGCGTTTTGCATTTTGTTCCGT<br>TTATAGTAACGTGCGCACCTAATACTTTCACCTGCTTCATGAA<br>GGACTGTATCAAATAGTCCGAAGTATGCTGCAGCTTTCCTGT<br>CTTACTGTGGATCAGAATCTTTTTTTGCAGCTTTATGGTATTCA<br>CTGCCTCAGTGACGAGACTTTGAAAATTTCAATCATGTTTTAA<br>AATTCCGCATATATCAACACGAAAATGGTGTCTCCAAGGATT<br>TGGAAAATTGAATTTCTACTCGCAAGTTACCTTGGTGTTGCA<br>ATTAAAATGAGGTCCATCACACAAGGATGGGATAAAGTCGA<br>ACACGTAAACTGTATAGCCTTCTACAAATTTGTCTTTCGAGA<br>AGTAAATACCTCTGTTTTGATTAAAGTTGATAAACAATTTGGT<br>GACAAGCGCGAATATATCTTTTAGAATCAAAATTCGGTTATA<br>AATGTTTGCTTGGCACTGAGTGACCATCTACTTAAACACTTA<br>AAAAGTTGACCATGATAATGATGAAAAAAAGGAAGAGGGC<br>AGGTTATGTTTCCTATTTACCGTGGCATTCTTCAGGGAGGTAA<br>TAATCACTCCTTTGGACATCAGTCACAAAAATACATTGTCTTT<br>CATAAAAGAAAATGTTCCAACTGAAACTGAGTATGCTTTATA<br>CAATACTCTGTCGATGGTATATTTCGAGAGACTTTTTTCGAGA<br>ACATTGACATGTCCCAAGGATACGCCGGGACATTCACTGTCC<br>TAATTAACAAGGAGGCATGCTCAGATACGATTTTATACTGGT<br>CTCATTTTGGGGGGGGAGACTAAACAAAAACTGTGTCTGTTG<br>TGGCTCATTTTGATTCGCAGATCATCAAATTTAATAAAAGTA<br>GATTTTGTTGAAAACATTCGCATCATCTCCAACGTTCGATTTT<br>TAGCTGTAAGTTCATTACGCTTCGTCAAACCTACATTTCTATC<br>GATTTCTTTCAACGGGTTCGTCGAATGTAATTTGCTGTCTTTGT

							AAAAGCATTCAACGGGGGAGTAAACTTTTCTTGACATTTTTT<br>CCATATATCCAAATTCTTCAATATAGGTTCTGTAGGGATAAG<br>TATTGCTATAGACGACGAGACGACTCGCTTCTTAAGTTGATTT<br>CCACTCTGAAAAAAAGAGCGTGTAAAATTAAATTCACGGGG<br>TCGACGAGTTCATTTTCAGGTAAAGCGGATCTATCCGACTTC<br>ATAATTTTGGCTTTTACGTGCAGTTAGGAAGACGATAAGTCA<br>ATGTAATCTTCACCATTGTTACATTCCACGGGTCCACCATCGC<br>GTAGGTTGGACAGGGGAGGATATTCAATAAAATGTCCTCTTC<br>GATTGCGGTCTCGCATAAGCGGGATCATGAACAAATGGAAA<br>TCAGATTTTGCGCAAGCAACACAGTATTGATAGATGCGTGTC<br>GATATGCTTAACCGAAAATAACGCGTGATTCCATTTTCTTGGT<br>TCTTGGTGAACTAATGCGTTATTCCAACGCTTTCTCTTTTAAA<br>TTATGACCAGAAGTAATAATGGACTTCATTTTTCAAGCTACTT<br>TGTCCGTTAATTGGCTTCCAGCTTATTTCGAATAGCGTTTAAC<br>AGACTTTTTAGAACATTCAATATGCCCACACTGCTGTGAAAT<br>CATCGTTTGCCGACTACTTTTATGCCCAGTTTTAAAAGGGGA<br>ACTGTCACTCGATAAAAAGGACAAAAGAATCTGCCAAAACC<br>ATAAACTGTCTGAAATGTATCCCCTTTATAATAATTCAACTCG<br>CCACGCTTTGCATAATCCTCGAAGGATTTTGGACAACATTGA<br>TGAGGCACATACATCACGATAGAAACTTTTGCCAAAAGTAC<br>AGTTTTACGTACGAGCGCTTGAGTTCAGATGACACAATTTCG<br>CGTGTATGCATTCTTATAACGACATTCAATACTTTTCTATTCC<br>GTTGGAAGTAATGGAGTCTTTCATATTGTGCTCAAATCATTTC<br>CCCGTCGCTTCCGGTCACGCTCAAGATGCGCAACAAGTGCGC<br>TAACACATCTCCCACAATATCAGGCTCTACAATGTCCGAATA<br>TATCAACAGTAATCTATAATGAGCGCACAGATATGCCGCTTA<br>CTGACTTTCGACGTTCTTTTCTTCATCATAATCATCATCGGTA<br>CTCTCAAGTTCAAACATAGCATTTGCGCGAGTCCGTCGTACA

							GTATCACTTTAGCACCCTGCAAAACAAAAACAGAATCTGCC<br>CCTTCCGGTAGACGCACTATGTTAAGAGTTCCCGAAATTGGA<br>ATCGATGCAAAACTTTGAGGGTAGACAGAATATAGTATATA<br>GACAAAATATGTCCAGATCGAAATAGAGTTTTAGGTCCAGAT<br>CAAGCTTTCAAGTTGTGAGTCTAGCATCGTTCTATGGTGGGA<br>AATGTGTTAAAAATATTTTTTAAATGCTGCACCCTTTACTGTG<br>TTGTTTACATGCAGTGTATATATTCAGCTATATACATGCAGTG<br>TATATATATTGTTTATATTCAGCTACTCTTCTCGGCTACAGCG<br>GCAGATAATTCAATTAGCCATCTCGCAAGAAAGGAAATATG<br>ACTGTGTTCCTGACTAAAGTACACCTGGCATGTATGCTTGTAC<br>CTAAAAGGTCCGAATTCGAGAATTTTCTCGAAAGATAATTAA<br>GGAGCGTCAGGGAAATTGTTGGAGGTCAGTGATGAAAGGGC<br>GACGATTGTTTTTCTGACGTCATAGGTCTGGGTTTTTTTTTTA<br>CAACTTCCGTGTCGAAATTGCACTATTTCCTGCAGAGATCAG<br>AGCTATATATTAACAGCGTTTCAGGGTATTACTGTGCTATGA<br>ATATATTCCAGATTTTATGCGTGTTTTAACGTATATCACTTGC<br>AAAGATGTATTTTTATTGTCTTAAAATACTACCATGATGTGGT<br>GTACCTCGGAAAAATAAACTTACTTTTACATATATAAGAGTG<br>TTTATTGCTGTAGCAGGAAAGTTTACCTTCACTTTATTTAATG<br>CTATATAATTGTACCTTACAATATTGTGTAGCTTATAATAAAG<br>TGAATAATCTTAATATTTCAACGATATTTGGAATGCTGTCAGC<br>ACAATTCAAACAACTTTCAGTGAATTATAGTGATTTCTCTTTA<br>ACAAGAATGCGATTAAAATCCAAAATAAGGTCGGAAAAATT<br>CACAGTTTTCTTCGCTAGATTTCAAGTAATTTGTACGGTTTTC<br>AGATAGTTCAAAAACTTTGACGTATGGATCTGACTTAGAGAA<br>CTTCCGCCACGTTTCACCGGGTTCCCCTCTCTTAGCAAAATTC<br>GTCCACTCTTTCACCATCTCAAAGCTTAGTTCTTCTTCCTTTCG<br>GGTGTAGTTGGACAGAGGAGTTAGAGGACGTCCAAAGATAA

							ATTGCATATCGTCACAATGCATAACCCCCATCCACTCTGCGTACGGAGTACTCGATGGTCTGTGATTGAAATAGTATCGGTAGATTTTATTATTTTTTTCTGCAACTTTCTCAGCAAAATATTTGCTTGGGCACATAACCATGAAATCCCCAAGAGAGTGATAAACTTGACTTCGTATTGTAGCATAATCGTCGTCTTTAACGCCACTGAGGTAATGCTGGATTATTCCTTCCGGATCTGGAAATATTGTCCCGAAATAATCTCTCATGAGAGACTCCCCTAACGTCTTATTAATGCGAGGATTTAATTCACCAAAAGGGCCTGTGATCTGTGTAAAAGTGTTAACAAATGGGAATGATCCCTCGTCTTGAGTAACTCCTATCATAGAATCAACACCGTTAAAGTTTCCTTCTCTGATAGCTTTTTGTGGGTCTACCGGTAGAAAATCATCCCCACTGATGGTAAAGAAAGTTATCACTGGGAAGACGGGAACTAAGGAAAACGTAGTTCTCACCATATCTTTTGGAGCTTTACTTCTTAGGCATTCGATCACATCATCAGGATTTTTCTTCAGCGTTATGTTTTCATTTGCACATTCCATCTTCTCTGCGAGCTTTTGTCCTAATTTAAGATTCTCAGCCCTATGGTCATTCAGAAAATATGCTGGACAACCACTTTGCATTATTGCTCTCTGGAAGAGACCTTTCGAAAGAGGAGAAACACACAGCAAACCTGTAGCTACGCCGCCGGCGCTGTTGCCGAACACCGTAATTCTTTTATTGTCTCCGCCGAATGCAGCAATGTTTTCGTTCGTCCATTTAAGGGCTTCCAAAATGTCATACAAGCCGACGTTTCCAGGAGCTTCTTCGGTTTCAGATGTGAAGAATCCGAGCACACTTAACCGGTAGTTTATCGCAACAACTATTACATCTCCAATTGCAGTTAGAACACGTCCATCATATAGAGGCATCCTACCTGAACCGGTTGCAAAGCCGCCTGAGAACACCCAAAACATGACGGCTTTTTTCTCGCTACTATTGGAAATTTCTGGTGCCCATATATTCAAATACAAGCAGTCTTCGCTTTGACCCTGAACTTTATCTTCCCACGGATAGTTGTCCGGAGAGTACTGGTAACAAGCAGGCGGTAAGCTGTTTGCTATTACAGGAGAGGTCCAAGGTTTG

							ACAGGAACTGTTCGCTTAAAGCGTAAATTCCCCACTGGAGGC<br>TCAGCAAACGGAATTCCGAGAAAGACATTTAGATTTATGTCG<br>TCATAAGTCATAACAGAACCAGTCACTTCACCTGTAGGAGTA<br>TTAACTGTAACGCTGGATGCTAAGATAGTTCCTGGTAGCAGT<br>AAAAATAGTAGATGCAAGCTCTTATGAACGTTTTTCTGGAGC<br>TTTCCGCACATAGCGGCTCCTAAACCTTGGGCTCCAGCAATA<br>ACGTTAGACGAGGGAGACGACATGATGCTGCTGCCTAGATC<br>TCCAGTGTGCTGTGTCGTACACAAATGCGCCTATTTCCTGCTA<br>GTGTAGGTCTTCATCCGAATTATGCCCAAACTGGTTGTAGTTT<br>TACTGTGGATAAGAAGCTTTCTTAACCTAATTTGTTCTCCACA<br>GGCTCCTTGAGAGTACGATGTTAACACATTGATGATGGGAGA<br>CGCTAATTTGCGTGTTGAATACTTCATGCGGTATCCTATAAAA<br>ATGTTAACTACTATTAAATAAAACAGTAAAAAAAGCAATGG<br>ATGCTATGAACAAAATGTTACGAATGTGTTTTTACATGAGAA<br>TGCTTGAATAATAAGAAACTCCGCCATCAATATTGAAAAAAT<br>TTTTTTGAAAACCTTTTATTTAGTAATGAGAAGGCAATAAGCT<br>ACGGAAGATCAGTTTGTGGAATATAGAGCAGAAATTTATGC<br>AGATTTGTTGTCAGATGTACAAAATGATGCCGAATGTGAAAA<br>TAATATTATTCATAGTTGCATTGAAGTTGATTTTATTAAGAAT<br>AGAAGCACTCGTCCTTTGCCGAGATGTCATTTCACTAATGTCT<br>GGAAAAGTATTCATCTTTTTCAGGCTTTAATGAAAAAGATGA<br>ATACATATCTTTATCTTACGAAACAAGTTCATGGACTAAGCA<br>AGACAAAATTACTGTTCTGGAAGATTTTATAGGAAACCCTGG<br>AGTAAAGCAATTTCCATCCGATCATACTAGCATTAATGAAGT<br>CGTAGGTTTATTCTTTGGAAATGATTTCTTTGAAATGCTTTAC<br>CGTGAAAATGAATACAAATGAAATAAAAACTAAAGTGGGTT<br>GATGTAGAAATAAAGGAAATGAAACGATTTTTTTATCTTGTT<br>TTACTTATGGTACATGTAAAAAAGGATCGCATTAGAGATTAT

							TGGTCGACTGATTCATACATAGAAACTCCGATTTTTTTCAAAAGTAATGAGCCGGAACAGATTTGAACAAATTTTACAGTCGCTCCATTTCCGTAATAATGATTTTCAAACTAATTCGACAGATAGACTCTTCAAGTTAAGACCAATATTACATTACTGTGTAAAAAAATTTCAACTCATACATAAGCCAAATCAAGAAGTATCTTTTGGCAGAACATGGAGAACTGCTCCGATACATGATTGGTATGCCGGTTTTCGGCCTGGAGCATCCGTTGTAAAAGCAAGTAATAGAAAAGCACAAACTGCCTTGGCAAGGTTCCGCAGCGGCCATTTTCGGCCGATGAGGTTTTCTGCTGACAAGAGGGTCTACCCTTTAAGTTCAAAATGCAGCTTGACTGAGACGACTCCCTCTCATGTTCTATTGTGCATTGGTTGCACCAAGAACCATCTGTTTTATAACTCTGGGGCGTTTCTGGATCAGTTGTG

PhdEnzCho 28	W4VSJ0	1,01E-63	34,959	PF00135	42055,19	MEYFLRPGLYDILGA<br>LKWVNQNIAAFGG<br>DDKRITVFGHSAGGI<br>ATGLLCVSPLSKGLF<br>QRAIMQSGSPAYFL<br>NNYRSRNMQLGQE<br>LAERMGCANENKT<br>LNENPDDVIECLRSK<br>DPNDLMTVTYSLVP<br>VFQEITFFTVTGDNF<br>MPIDPLKAIVQGNF<br>NGVDTLIGNAQDD<br>GSPFLVAFSRQIAGP<br>SGELNPRINKTVGES<br>LMREFFQTFLDPEAV<br>IQHYLSGVEDDDYD<br>TIRSQVYHSFGDFMF<br>ISTSKYFAEEIAEKNK<br>EVYRYYFSHRSSKTP<br>WAEWMGVAHFEEV<br>QFVFGRPLDSPSNYT<br>REEEELSFKIVKEWT<br>NFANEGKPSNMWQ<br>MFTRSDPYIEIFKVPQ<br>SSTSYLEAHEENCEF<br>FRPYFGF*	TTTTTTTTACACATAAAGGGATATTTATTACTGCAGCGTAATA<br>GTTTAGATTCGTTTTATTTAATATCTTAAACGTGTGTTTCTTAA<br>CGTTTCAGTCATATTTGAATGTCTTCAACACAATCCAAGCAA<br>ATTGTAGTGATTGGTAAAGATTTGCCTTTAAGAAGGTCGCGA<br>TTAAAAACCAAAATAAGGTCGGAAAAATTCGCAATTTTCTTC<br>GTGAGCTTCCAAGTAACTTGTACTGCTTTGAGGCACTTTAAA<br>AATTTCGATATATGGATCTGATCTAGTGAACATCTGCCACAT<br>ATTACTGGGTTTCCCTTCATTAGCAAAATTCGTCCACTCCTTG<br>ACAATTTTAAAACTTAGTTCTTCTTCCTCTCTAGTGTAGTTGG<br>ACGGAGAATCTAACGGACGACCAAAGACAAACTGCACTTCC<br>TCAAAATGCGCAACCCCCATCCACTCTGCCCACGGAGTTTTA<br>CTTGACCTGTGACTGAAATAATATCTGTAGACTTCTTTATTTT<br>TTTCTGCAATTTCCTCAGCAAAATATTTGCTTGTGCTTATAAA<br>CATGAAATCCCCAAAAGAGTGATAAACTTGACTTCGTATTGT<br>ATCATAATCGTCATCTTCAACACCACTGAGATAATGCTGAAT<br>TACTGCTTCCGGGTCTAGAAAAGTCTGGAAAAACTCTCTCAT<br>GAGAGACTCCCCAACCGTCTTATTAATGCGAGGATTTAACTC<br>ACCAGAAGGACCTGCGATTTGACGGGAAAATGCAACGAGAA<br>AGGGCGATCCATCATCTTGAGCATTTCCTATCAATGTATCAA<br>CACCGTTGAAGTTGCCTTGCACGATCGCTTTTAGTGGGTCTAT<br>AGGCATAAAATTGTCCCCAGTGACGGTAAAAAATGTTATCTC<br>CTGAAAAACAGGAACTAGGGAATATGTGACGGTCATCAAAT<br>CGTTTGGATCTTTACTTCTTAAACATTCGATTACGTCATCAGG<br>ATTTTCGTTCAACGTTTTGTTTTCATTTGCACATCCCATCCTCT<br>CTGCTAGCTCCTGTCCCAACTGCATATTCCGAGATCTATAGTT<br>GTTCAGAAAATATGCTGGAGAACCACTCTGCATGATCGCTCT<br>CTGGAAGAGACCTTTCGAAAGAGGAGAAACACACAGCAAA<br>CCTGTAGCTATGCCGCCGGCGCTGTGGCCGAACACCGTAATT

							CTTTTATCGTCTCCGCCGAATGCAGCAATGTTTTGGTTCACCC ATTTAAGGGCTCCCAAAATATCATACAAGCCGGGGCGGAGG AAGTACTCCATTTAAACCTCAGGTGGCAATTTGGAAAAATAA TCGAAACTAAATTAGTTGGCGTAATCAAACTTATTTAGACGA ACGACTGGCACTTCGATCTGAAAATGAGTCAAAGTTTTGGCC GTCATGAGAAAAACAGTATCCACGGAAAATATTCGGTGTCG GGGTGTCATAAAAATATTAGATTTACAATGAAT

PhdEnzCho 29	W4VSJ0	8,73E-110	37,618	PF00135	63218.00<br><br>**58747.62**	MSSPSSNVIAGAQGL<br>GAAMCGKLQKNVH<br>KSLHLLFLLLPGTILA<br>**SSVTVNTPTGEVTG**<br>**SVMTYDDINLNVFL**<br>**GIPFAEPPVGNLRF**<br>**KRTVPVKPWTSPVI**<br>**ANSLPPACYQYSPD**<br>**NYPWEDKVQGQSE**<br>**DCLYLNIWAPEISN**<br>**SSEKKAVMFWVFS**<br>**GGFATGSGRMPLY**<br>**DGRVLTAIGDVIVV**<br>**AINYRLSVLGFFTSE**<br>**TEEAPGNVGLYDIL**<br>**EALKWTNENIAAFG**<br>**GDNKRITVFGNSA**<br>**GGVATGLLCVSPLS**<br>**KGLFQRAIMQSGC**<br>**PAYFLNDHRAENLK**<br>**LGQKLAEKMECAN**<br>**ENITLKKNPDDVIE**<br>**CLRSKAPKDMVRT**<br>**TFSLVPVFPVITFFTI**<br>**SGDDFLPVDPQKAI**<br>**REGNFNGVDSMIG**<br>**VTQDEGSFPFVNTF**<br>**TQITGPFGELNPRIN**	CGATAAACCTTTTGCAAGCAAGTTTCTCATGATCTGGCAATA<br>TTCTAGAAAGTGCAAACATGATTATGTAATATGAAAAATGCT<br>GTATATGAAACTCGAGGTATTTTTGATTTATTCTTTATACTTTC<br>ATATTACATATTCATCTTTACACTTTCATAACTATGCTTTCATG<br>AGTCTGCGTACATCAACATAACACATTAGAAAACTTTAATAT<br>GCAATTCATATTCTCAATTTAAGCTAGGATCCCCTTTATTTTG<br>CAATTTAACTCAAAACTTCAAAGGAATAAACCTAAAATTATG<br>CTTTTTTAACTACGGGAAGCTTCAAATTTTACGATAAACCTTT<br>TGCAAGCAAGTTTCTCATGATCTGGCAATATTCTAGAAAGTG<br>CAAACATGATTATGTAATATGAAAAATGCTGTATATGAAACT<br>CAAGGTATTTTTGATTTATTTTTTATACTTTCATATTACATAGT<br>CATCTTTACACTTTCGTAATGCTGCGTATATCAACATTAGCAA<br>ACTTTAATATGCAATAAATAGCTATTAGGATAACCTTTTCTTT<br>TAATAACGTCACTATGCCTCTTTTAAATAGAATTGACTAGCTT<br>CGATGTTTACTCGGAGCTTATATTATTCCATTCTTCTCTCAATA<br>TGGCTTTCAACATAGCCTTTGAAGCTATATTGTGTTGGCAGAT<br>TCTGCGTTCCAGCAAATCTCAAAAACACTTTATCGGGTAAGT<br>CAGGCGATTCTGGAGTTGTTTATGTGTAGGAGTAGATAACTA<br>TTTTAGCACGTCATATAAGAACTAGATATTCATAACTTGAAC<br>AGTGTGCTTGGGGACCTTGTCCAATTAGAAATAATAACTGTT<br>GTCTATCTTAAGTACTCTGCATTAAATTTCGTTTTAAAATGTTT<br>AAATAATCCATTTTATTCAATTGTGTCCTGAATAAAGTGTAGT<br>TGTCCCACGCCTCAGTTTTAGATACACCCCCAAATAATAATA<br>CTTTAGCCACCGTGTTTTACAGTTGGCATTAAATATTCTTACT<br>TAAGCTCTTTAACAGGTTTTCGGTACAAAAGCTTGAATCCTTT<br>AATGTCAAATACGCAGAATCTGCTCTAATCGGAAAATATTAC<br>CAGTTGCAGAGCTCATTTTGCTTATTAATATATTCTTTTGCAA<br>CGTCAATGCTTAAATCTATTATCTCAGGAAATGTAAAACAGC

						**KTLGESLMRDYFGT<br>IFPDPEGIIQHYLSG<br>VKDDDYATIRSQV<br>YHSLGDFMVMCPS<br>KYFAEKVAEKNNKI<br>YRYYFNHRPSSTPY<br>AEWMGVMHCDD<br>MQFIFGRPLTPLSN<br>YTRKEEELSFEMVK<br>EWTNFAKRGEPGET<br>WRKFSKSDPYVKVF<br>ELSENRTNYLKSSEE<br>NCEFFRPYFGF***	TAAAAGGGATGTTACAACAGTTTCTCTTCGAGCCGTCACATT<br>TATCTTTTGTTCTTTCGCCTCTTGTGTTTTACCTATTCCTCGACA<br>TATTTATGGCTATTTTTCAGTTCACAACTCTCTCGGATGGCGT<br>AATCTTTTCTTGAAATTTCTTCGAATTTTCTCTCGTTTTATATA<br>TATCAAAGCGGTAATGCGTTTTGCATTTTGTTCCGTTTATAGT<br>AACGTGCGCACCTAATACTTTCACCTGCTTCATGAAGGACTG<br>TATCAAATAGTCCGAAGTATGCTGCAGCTTTCCTGTCTTACTG<br>TGGATCAGAATCTTTTTTTGCAGCTTTATGGTATTCACTGCCT<br>CAGTGACGAGACTTTGAAAATTTCAATCATGTTTTAAAATTC<br>CGCATATATCAACACGAAAATGGTGTCTCCAAGGATTTGGAA<br>AATTGAATTTCTACTCGCAAGTTACCTTGGTGTTGCAATTAAA<br>ATGAGGTCCATCACACAAGGATGGGATAAAGTCGAACACGT<br>AAACTGTATAGCCTTCTACAAATTTGTCTTTCGAGAAGTAAA<br>TACCTCTGTTTTGATTAAAGTTGATAAACAATTTGGTGACAA<br>GCGCGAATATATCTTTTAGAATCAAAATTCGGTTATAAATGT<br>TTGCTTGGCACTGAGTGACCATCTACTTAAACACTTAAAAAG<br>TTGACCATGATAATGATGAAAAAAAGGAAGAGGGCAGGTTA<br>TGTTTCCTATTTACCGTGGCATTCTTCAGGGAGGTAATAATCA<br>CTCCTTTGGACATCAGTCACAAAAATACATTGTCTTTCATAA<br>AAGAAAATGTTCCAACTGAAACTGAGTATGCTTTATACAATA<br>CTCTGTCGATGGTATATTTCGAGAGACTTTTTTCGAGAACATT<br>GACATGTCCCAAGGATACGCCGGGACATTCACTGTCCTAATT<br>AACAAGGAGGCATGCTCAGATACGATTTTATACTGGTCTCAT<br>TTTGGGGGGGGAGACTAAACAAAAACTGTGTCTGTTGTGGCT<br>CATTTTGATTCGCAGATCATCAAATTTAATAAAAGTAGATTTT<br>GTTGAAAACATTCGCATCATCTCCAACGTTCGATTTTTAGCTG<br>TAAGTTCATTACGCTTCGTCAAACCTACATTTCTATCGATTTC<br>TTTCAACGGGTTCGTCGAATGTAATTTGCTGTCTTTGTAAAAG

							CATTCAACGGGGGAGTAAACTTTTCTTGACATTTTTTCCATAT<br>ATCCAAATTCTTCAATATAGGTTCTGTAGGGATAAGTATTGCT<br>ATAGACGACGAGACGACTCGCTTCTTAAGTTGATTTCCACTC<br>TGAAAAAAAGAGCGTGTAAAATTAAATTCACGGGGTCGACG<br>AGTTCATTTTCAGGTAAAGCGGATCTATCCGACTTCATAATTT<br>TGGCTTTTACGTGCAGTTAGGAAGACGATAAGTCAATGTAAT<br>CTTCACCATTGTTACATTCCACGGGTCCACCATCGCGTAGGTT<br>GGACAGGGGAGGATATTCAATAAAATGTCCTCTTCGATTGCG<br>GTCTCGCATAAGCGGGATCATGAACAAATGGAAATCAGATT<br>TTGCGCAAGCAACACAGTATTGATAGATGCGTGTCGATATGC<br>TTAACCGAAAATAACGCGTGATTCCATTTTCTTGGTTCTTGGT<br>GAACTAATGCGTTATTCCAACGCTTTCTCTTTTAAATTATGAC<br>CAGAAGTAATAATGGACTTCATTTTTCAAGCTACTTTGTCCGT<br>TAATTGGCTTCCAGCTTATTTCGAATAGCGTTTAACAGACATT<br>TTTAGAACATTCAATATGCCCACACTGCTGTGAAATCATCGT<br>TTGCCGACTACTTTTATGCCCAGTTTTAAAAGGGGAACTGTC<br>ACTCGATAAAAAGGACAAAAGAATCTGCCAAAACCATAAA<br>CTGTCTGAAATGTATCCCCTTTATAATAATTCAACTCGCCACG<br>CTTTGCATAATCCTCGAAGGATTTTGGACAACATTGATGAGG<br>CACATACATCACGATAGAAACTTTTGCCAAAAGTACAGTTTT<br>ACGTACGAGCGCTTGAGTTCAGATGACACAATTTCGCGTGTA<br>TGCATTCTTATAACGACATTCAATACTTTTCTATTCCGTTGGA<br>AGTAATGGAGTCTTTCATATTGTGCTCAAATCATTTCCCCGTC<br>GCTTCCGGTCACGCTCAAGATGCGCAACAAGTGCGCTAACA<br>CATCTCCCACAATATCAGGCTCTACAATGTCCGAATATATCA<br>ACAGTAATCTATAATGAGCGCACAGATATGCCGCTTACTGAC<br>TTTCGACGTTCTTTTCTTCATCATAATCATCATCGGTACTCTCA<br>AGTTCAAACATAGCATTTGCGCGAGTCCGTCGTACAGTATCA

							CTTTAGCACCCTGCAAAACAAAAACAGAATCTGCCCCTTCCG<br>GTAGACGCACTATGTTAAGAGTTCCCGAAATTGGAATCGATG<br>CAAAACTTTGAGGGTAGACAGAATATAGTATATAGACAAAA<br>TATGTCCAGATCGAAATAGAGTTTTAGGTCCAGATCAAGCTT<br>TCAAGTTGTGAGTCTAGCATCGTTCTATGGTGGGAAATGTGTT<br>AAAAATATTTTTTAAATGCTGCACCCTTTACTGTGTTGTTTAC<br>ATGCAGTGTATATATTCAGCTATATACATGCAGTGTATATAT<br>ATTGTTTATATTCAGCTACTCTTCTCGGCTACAGCGGCAGATA<br>ATTCAATTAGCCATCTCGCAAGAAAGGAAATATGACTGTGTT<br>CCTGACTAAAGTACACCTGGCATGTATGCTTGTACCTAAAAG<br>GTCCGAATTCGAGAATTTTCTCGAAAGATAATTAAGGAGCGT<br>CAGGGAAATTGTTGGAGGTCAGTGATGAAAGGGCGACGATT<br>GTTTTTCTGACGTCATAGGTCTGGGTTTTTTTTTTACAACTTC<br>CGTGTCGAAATTGCACTATTTCCTGCAGAGATCAGAGCTATA<br>TATTAACAGCGTTTCAGGGTATTACTGTGCTATGAATATATTC<br>CAGATTTTATGCGTGTTTTAACGTATATCACTTGCAAAGATGT<br>ATTTTTATTGTCTTAAAATACTACCATGATGTGGTGTACCTCG<br>GAAAAATAAACTTACTTTTACATATATAAGAGTGTTTATTGCT<br>GTAGCAGGAAAGTTTACCTTCACTTTATTTAATGCTATATAAT<br>TGTACCTTACAAAATTTTGTAGCTTATAATAAAGTGAATAAT<br>CTTAATATTTCAACGATATTTGGAATGCTGTCAGCACAATTCA<br>AACAACTTTCAGTGAATTATAGTGATTTCTCTTTAACAAGAAT<br>GCGATTAAAATCCAAAATAAGGTCGGAAAAATTCACAGTTTT<br>CTTCGCTAGATTTCAAGTAATTTGTACGGTTTTCAGATAGTTC<br>AAAAACTTTGACGTATGGATCTGACTTAGAGAACTTCCGCCA<br>CGTTTCACCGGGTTCCCCTCTCTTAGCAAAATTCGTCCACTCT<br>TTCACCATCTCAAAGCTTAGTTCTTCTTCCTTTCGGGTGTAGTT<br>GGACAGAGGAGTTAGAGGACGTCCAAAGATAAATTGCATAT

							CGTCACAATGCATAACCCCCATCCACTCTGCGTACGGAGTACTCGATGGTCTGTGATTGAAATAGTATCGGTAGATTTTATTATTTTTTTCTGCAACTTTCTCAGCAAAATATTTGCTTGGGCACATAACCATGAAATCCCCAAGAGAGTGATAAACTTGACTTCGTATTGTAGCATAATCGTCGTCTTTAACGCCACTGAGGTAATGCTGGATTATTCCTTCCGGATCTGGAAATATTGTCCCGAAATAATCTCTCATGAGAGACTCCCCTAACGTCTTATTAATGCGAGGATTTAATTCACCAAAAGGGCCTGTGATCTGTGTAAAAGTGTTAACAAATGGGAATGATCCCTCGTCTTGAGTAACTCCTATCATAGAATCAACACCGTTAAAGTTTCCTTCTCTGATAGCTTTTTGTGGGTCTACCGGTAGAAAATCATCCCCACTGATGGTAAAGAAAGTTATCACTGGGAAGACGGGAACTAAGGAAAACGTAGTTCTCACCATATCTTTTGGAGCTTTACTTCTTAGGCATTCGATCACATCATCAGGATTTTTCTTCAGCGTTATGTTTTCATTTGCACATTCCATCTTCTCTGCGAGCTTTTGTCCTAATTTAAGATTCTCAGCCCTATGGTCATTCAGAAAATATGCTGGACAACCACTTTGCATTATTGCTCTCTGGAAGAGACCTTTCGAAAGAGGAGAAACACACAGCAAACCTGTAGCTACGCCGCCGGCGCTGTTGCCGAACACCGTAATTCTTTTATTGTCTCCGCCGAATGCAGCAATGTTTTCGTTCGTCCATTTAAGGGCTTCCAAAATGTCATACAAGCCGACGTTTCCAGGAGCTTCTTCGGTTTCAGATGTGAAGAATCCGAGCACACTTAACCGGTAGTTTATCGCAACAACTATTACATCTCCAATTGCAGTTAGAACACGTCCATCATATAGAGGCATCCTACCTGAACCGGTTGCAAAGCCGCCTGAGAACACCCAAAACATGACGGCTTTTTTCTCGCTACTATTGGAAATTTCTGGTGCCCATATATTCAAATACAAGCAGTCTTCGCTTTGACCCTGAACTTTATCTTCCCACGGATAGTTGTCCGGAGAGTACTGGTAACAAGCAGGCGGTAAGCTGTTTGCTATTACAGGAGAGGTCCAAGGTTTGACAGGAAC

							TGTTCGCTTAAAGCGTAAATTCCCCACTGGAGGCTCAGCAAACGGAATTCCGAGAAAGACATTTAGATTTATGTCGTCATAAGTCATAACAGAACCAGTCACTTCACCTGTAGGAGTATTAACTGTAACGCTGGATGCTAAGATAGTTCCTGGTAGCAGTAAAAATAGTAGATGCAAGCTCTTATGAACGTTTTTCTGGAGCTTTCCGCACATAGCGGCTCCTAAACCTTGGGCTCCAGCAATAACGTTAGACGAGGGAGACGACATGATGCTGCTGCCTAGATCTCCAGTGTGCTGTGTCGTACACAAATGCGCCTATTTCCTGTTTGTAAAGCTCCTTCGTACACCAGTATCGTCAACAGCTATTTTTACATTAGTTATTCCGTTTCTGTCAGCAATCTTGCATCGCACAAATCTGCTAGTGTAGGTCTTCATCCGAATTATGCCCAAACTGGTTGTAGTTTTACTGTGGATAAGAAGCTTTCTTAACCTAATTTGTTCTCCACAGGCT

PhdEnzCho 30	W4VSJ0	1,09E-109	37,618	PF00135	63218.00<br><br>**58747.62**	MSSPSSNVIAGAQGL<br>GAAMCGKLQKNVH<br>KSLHLLFLLLPGTILA<br>**SSVTVNTPTGEVTG**<br>**SVMTYDDINLNVFL**<br>**GIPFAEPPVGNLRF**<br>**KRTVPVKPWTSPVI**<br>**ANSLPPACYQYSPD**<br>**NYPWEDKVQGQSE**<br>**DCLYLNIWAPEISN**<br>**SSEKKAVMFWVFS**<br>**GGFATGSGRMPLY**<br>**DGRVLTAIGDVIVV**<br>**AINYRLSVLGFFTSE**<br>**TEEAPGNVGLYDIL**<br>**EALKWTNENIAAFG**<br>**GDNKRITVFGNSA**<br>**GGVATGLLCVSPLS**<br>**KGLFQRAIMQSGC**<br>**PAYFLNDHRAENLK**<br>**LGQKLAEKMECAN**<br>**ENITLKKNPDDVIE**<br>**CLRSKAPKDMVRT**<br>**TFSLVPVFPVITFFTI**<br>**SGDDFLPVDPQKAI**<br>**REGNFNGVDSMIG**<br>**VTQDEGSFPFVNTF**<br>**TQITGPFGELNPRIN**	CGATAAACCTTTTGCAAGCAAGTTTCTCATGATCTGGCAATA<br>TTCTAGAAAGTGCAAACATGATTATGTAATATGAAAAATGCT<br>GTATATGAAACTCGAGGTATTTTTGATTTATTCTTTATACTTTC<br>ATATTACATATTCATCTTTACACTTTCATAACTATGCTTTCATG<br>AGTCTGCGTACATCAACATAACACATTAGAAAACTTTAATAT<br>CAATTCATATTCTCAATTTCATATTCTCAATTTAAGCTAGGAT<br>CCCCTTTATTTTGCAATTTAACTCAAAACTTCAAAGGAATAA<br>ACCTAAAATTATGCTTTTTTAACTACGGGAAGCTTCAAATTTT<br>ACGATAAACCTTTTGCAAGCAAGTTTCTCATGATCTGGCAAT<br>ATTCTAGAAAGTGCAAACATGATTATGTAATATGAAAAATGC<br>TGTATATGAAACTCAAGGTATTTTTGATTTATTTTTTATACTTT<br>CATATTACATAGTCATCTTTACACTTTCGTAATTCTGCGTATA<br>TCAACTGAAAACATTAGCAAACTTTAATATGCAATAAATAGC<br>TATTAGGATAACCTTTTCTTTTAATAACGTCACTATGCCTCTTT<br>TAAATAGAATTGACTAGCTTCGATGTTTACTCGGAGCTTATAT<br>TATTCCATTCTTCTCTCAATATGGCTTTCAACATAGCCTTTGA<br>AGCTATATTGTGTTGGCAGATTCTGCGTTCCAGCAAATCTCA<br>AAAACACTTTATCGGGTAAGTCAGGCGATTCTGGAGTTGTTT<br>ATGTGTAGGAGTAGATAACTATTTTAGCACGTCATATAAGAA<br>CTAGATATTCATAACTTGAACAGTGTGCTTGGGGACCTTGTC<br>CAATTAGAAATAATAACTGTTGTCTATCTTAAGTACTCTGCAT<br>TAAATTTCGTTTTAAAATGTTTAAATAATCCATTTTATTCAATT<br>GTGTCCTGAATAAAGTGTAGTTGTCCCACGCCTCAGTTTTAG<br>ATACACCCCCAAATAATAATACTTTAGCCACCGTGTTTTACA<br>GTTGGCATTAAATATTCTTACTTAAGCTCTTTAACAGGTTTTC<br>GGTACAAAAGCTTGAATCCTTTAATGTCAAATACGCAGAATC<br>TGCTCTAATCGGAAAATATTACCAGTTGCAGAGCTCATTTTG<br>CTTATTAATATATTCTTTTGCAACGTCAATGCTTAAATCTATT

						**KTLGESLMRDYFGT<br>IFPDPEGIIQHYLSG<br>VKDDDYATIRSQV<br>YHSLGDFMVMCPS<br>KYFAEKVAEKNNKI<br>YRYYFNHRPSSTPY<br>AEWMGVMHCDD<br>MQFIFGRPLTPLSN<br>YTRKEEELSFEMVK<br>EWTNFAKRGEPGET<br>WRKFSKSDPYVKVF<br>ELSENRTNYLKSSEE<br>NCEFFRPYFGF***	ATCTCAGGAAATGTAAAACAGCTAAAAGGGATGTTACAACA<br>GTTTCTCTTCGAGCCGTCACATTTATCTTTTGTTCTTTCGCCTC<br>TTGTGTTTTACCTATTCCTCGACATATTTATGGCTATTTTTCAG<br>TTCACAACTCTCTCGGATGGCGTAATCTTTTCTTGAAATTTCTT<br>CGAATTTTCTCTCGTTTTATATATATCAAAGCGGTAATGCGTT<br>TTGCATTTTGTTCCGTTTATAGTAACGTGCGCACCTAATACTT<br>TCACCTGCTTCATGAAGGACTGTATCAAATAGTCCGAAGTAT<br>GCTGCAGCTTTCCTGTCTTACTGTGGATCAGAATCTTTTTTTGC<br>AGCTTTATGGTATTCACTGCCTCAGTGACGAGACTTTGAAAA<br>TTTCAATCATGTTTTAAAATTCCGCATATATCAACACGAAAA<br>TGGTGTCTCCAAGGATTTGGAAAATTGAATTTCTACTCGCAA<br>GTTACCTTGGTGTTGCAATTAAAATGAGGTCCATCACACAAG<br>GATGGGATAAAGTCGAACACGTAAACTGTATAGCCTTCTACA<br>AATTTGTCTTTCGAGAAGTAAATACCTCTGTTTTGATTAAAGT<br>TGATAAACAATTTGGTGACAAGCGCGAATATATCTTTTAGAA<br>TCAAAATTCGGTTATAAATGTTTGCTTGGCACTGAGTGACCA<br>TCTACTTAAACACTTAAAAAGTTGACCATGATAATGATGAAA<br>AAAAGGAAGAGGGCAGGTTATGTTTCCTATTTACCGTGGCAT<br>TCTTCAGGGAGGTAATAATCACTCCTTTGGACATCAGTCACA<br>AAAATACATTGTCTTTCATAAAAGAAAATGTTCCAACTGAAA<br>CTGAGTATGCTTTATACAATACTCTGTCGATGGTATATTTCGA<br>GAGACTTTTTTCGAGAACATTGACATGTCCCAAGGATACGCC<br>GGGACATTCACTGTCCTAATTAACAAGGAGGCATGCTCAGAT<br>ACGATTTTATACTGGTCTCATTTTGGGGGGGGAGACTAAACA<br>AAAACTGTGTCTGTTGTGGCTCATTTTGATTCGCAGATCATCA<br>AATTTAATAAAAGTAGATTTTGTTGAAAACATTCGCATCATC<br>TCCAACGTTCGATTTTTAGCTGTAAGTTCATTACGCTTCGTCA<br>AACCTACATTTCTATCGATTTCTTTCAACGGGTTCGTCGAATG

							TAATTTGCTGTCTTTGTAAAAGCATTCAACGGGGGAGTAAAC<br>TTTTCTTGACATTTTTTCCATATATCCAAATTCTTCAATATAGG<br>TTCTGTAGGGATAAGTATTGCTATAGACGACGAGACGACTCG<br>CTTCTTAAGTTGATTTCCACTCTGAAAAAAAGAGCGTGTAAA<br>ATTAAATTCACGGGGTCGACGAGTTCATTTTCAGGTAAAGCG<br>GATCTATCCGACTTCATAATTTTGGCTTTTACGTGCAGTTAGG<br>AAGACGATAAGTCAATGTAATCTTCACCATTGTTACATTCCA<br>CGGGTCCACCATCGCGTAGGTTGGACAGGGGAGGATATTCA<br>ATAAAATGTCCTCTTCGATTGCGGTCTCGCATAAGCGGGATC<br>ATGAACAAATGGAAATCAGATTTTGCGCAAGCAACACAGTA<br>TTGATAGATGCGTGTCGATATGCTTAACCGAAAATAACGCGT<br>GATTCCATTTTCTTGGTTCTTGGTGAACTAATGCGTTATTCCA<br>ACGCTTTCTCTTTTAAATTATGACCAGAAGTAATAATGGACTT<br>CATTTTTCAAGCTACTTTGTCCGTTAATTGGCTTCCAGCTTATT<br>TCGAATAGCGTTTAACAGACTTTTTAGAACATTCAATATGCC<br>CACACTGCTGTGAAATCATCGTTTGCCGACTACTTTTATGCCC<br>AGTTTTAAAAGGGGAACTGTCACTCGATAAAAAGGACAAAA<br>GAATCTGCCAAAACCATAAACTGTCTGAAATGTATCCCCTTT<br>ATAATAATTCAACTCGCCACGCTTTGCATAATCCTCGAAGGA<br>TTTTGGACAACATTGATGAGGCACATACATCACGATAGAAA<br>CTTTTGCCAAAAGTACAGTTTTACGTACGAGCGCTTGAGTTC<br>AGATGACACAATTTCGCGTGTATGCATTCTTATAACGACATT<br>CAATACTTTTCTATTCCGTTGGAAGTAATGGAGTCTTTCATAT<br>TGTGCTCAAATCATTTCCCCGTCGCTTCCGGTCACGCTCAAGA<br>TGCGCAACAAGTGCGCTAACACATCTCCCACAATATCAGGCT<br>CTACAATGTCCGAATATATCAACAGTAATCTATAATGAGCGC<br>ACAGATATGCCGCTTACTGACTTTCGACGTTCTTTTCTTCATC<br>ATAATCATCATCGGTACTCTCAAGTTCAAACATAGCATTTGC

							GCGAGTCCGTCGTACAGTATCACTTTAGCACCCTGCAAAACA<br>AAAACAGAATCTGCCCCTTCCGGTAGACGCACTATGTTAAGA<br>GTTCCCGAAATTGGAATCGATGCAAAACTTTGAGGGTAGAC<br>AGAATATAGTATATAGACAAAATATGTCCAGATCGAAATAG<br>AGTTTTAGGTCCAGATCAAGCTTTCAAGTTGTGAGTCTAGCA<br>TCGTTCTATGGTGGGAAATGTGTTAAAAATATTTTTTAAATGC<br>TGCACCCTTTACTGTGTTGTTTACATGCAGTGTATATATTCAG<br>CTATATACATGCAGTGTATATATATTGTTTATATTCAGCTACT<br>CTTCTCGGCTACAGCGGCAGATAATTCAATTAGCCATCTCGC<br>AAGAAAGGAAATATGACTGTGTTCCTGACTAAAGTACACCT<br>GGCATGTATGCTTGTACCTAAAAGGTCCGAATTCGAGAATTT<br>TCTCGAAAGATAATTAAGGAGCGTCAGGGAAATTGTTGGAG<br>GTCAGTGATGAAAGGGCGACGATTGTTTTTCTGACGTCATAG<br>GTCTGGGTTTTTTTTTTTACAACTTCCGTGTCGAAATTGCACTA<br>TTTCCTGCAGAGATCAGAGCTATATATTAACAGCGTTTCAGG<br>GTATTACTGTGCTATGAATATATTCCAGATTTTATGCGTGTTTT<br>AACGTATATCACTTGCAAAGATGTATTTTTATTGTCTTAAAAT<br>ACTACCATGATGTGGTGTACCTCGGAAAAATAAACTTACTTT<br>TACATATATAAGAGTGTTTATTGCTGTAGCAGGAAAGTTTAC<br>CTTCACTTTATTTAATGCTATATAATTGTACCTTACAATATTGT<br>GTAGCTTATAATAAAGTGAATAATCTTAATATTTCAACGATA<br>TTTGGAATGCTGTCAGCACAATTCAAACAACTTTCAGTGAAT<br>TATAGTGATTTCTCTTTAACAAGAATGCGATTAAAATCCAAA<br>ATAAGGTCGGAAAAATTCACAGTTTTCTTCGCTAGATTTCAA<br>GTAATTTGTACGGTTTTCAGATAGTTCAAAAACTTTGACGTAT<br>GGATCTGACTTAGAGAACTTCCGCCACGTTTCACCGGGTTCC<br>CCTCTCTTAGCAAAATTCGTCCACTCTTTCACCATCTCAAAGC<br>TTAGTTCTTCTTCCTTTCGGGTGTAGTTGGACAGAGGAGTTAG

							AGGACGTCCAAAGATAAATTGCATATCGTCACAATGCATAA CCCCCATCCACTCTGCGTACGGAGTACTCGATGGTCTGTGAT TGAAATAGTATCGGTAGATTTTATTATTTTTTTCTGCAACTTTC TCAGCAAAATATTTGCTTGGGCACATAACCATGAAATCCCCA AGAGAGTGATAAACTTGACTTCGTATTGTAGCATAATCGTCG TCTTTAACGCCACTGAGGTAATGCTGGATTATTCCTTCCGGAT CTGGAAATATTGTCCCGAAATAATCTCTCATGAGAGACTCCC CTAACGTCTTATTAATGCGAGGATTTAATTCACCAAAAGGGC CTGTGATCTGTGTAAAAGTGTTAACAAATGGGAATGATCCCT CGTCTTGAGTAACTCCTATCATAGAATCAACACCGTTAAAGT TTCCTTCTCTGATAGCTTTTTGTGGGTCTACCGGTAGAAAATC ATCCCCACTGATGGTAAAGAAAGTTATCACTGGGAAGACGG GAACTAAGGAAAACGTAGTTCTCACCATATCTTTTGGAGCTT TACTTCTTAGGCATTCGATCACATCATCAGGATTTTTCTTCAG CGTTATGTTTTCATTTGCACATTCCATCTTCTCTGCGAGCTTTT GTCCTAATTTAAGATTCTCAGCCCTATGGTCATTCAGAAAAT ATGCTGGACAACCACTTTGCATTATTGCTCTCTGGAAGAGAC CTTTCGAAAGAGGAGAAACACACAGCAAACCTGTAGCTACG CCGCCGGCGCTGTTGCCGAACACCGTAATTCTTTTATTGTCTC CGCCGAATGCAGCAATGTTTTCGTTCGTCCATTTAAGGGCTTC CAAAATGTCATACAAGCCGACGTTTCCAGGAGCTTCTTCGGT TTCAGATGTGAAGAATCCGAGCACACTTAACCGGTAGTTTAT CGCAACAACTATTACATCTCCAATTGCAGTTAGAACACGTCC ATCATATAGAGGCATCCTACCTGAACCGGTTGCAAAGCCGCC TGAGAACACCCAAAACATGACGGCTTTTTTCTCGCTACTATT GGAAATTTCTGGTGCCCATATATTCAAATACAAGCAGTCTTC GCTTTGACCCTGAACTTTATCTTCCCACGGATAGTTGTCCGGA GAGTACTGGTAACAAGCAGGCGGTAAGCTGTTTGCTATTACA

							GGAGAGGTCCAAGGTTTGACAGGAACTGTTCGCTTAAAGCGT<br>AAATTCCCCACTGGAGGCTCAGCAAACGGAATTCCGAGAAA<br>GACATTTAGATTTATGTCGTCATAAGTCATAACAGAACCAGT<br>CACTTCACCTGTAGGAGTATTAACTGTAACGCTGGATGCTAA<br>GATAGTTCCTGGTAGCAGTAAAAATAGTAGATGCAAGCTCTT<br>ATGAACGTTTTTCTGGAGCTTTCCGCACATAGCGGCTCCTAA<br>ACCTTGGGCTCCAGCAATAACGTTAGACGAGGGAGACGACA<br>TGATGCTGCTGCCTAGATCTCCAGTGTGCTGTGTCGTACACA<br>AATGCGCCTATTTCCTGCTAGTGTAGGTCTTCATCCGAATTAT<br>GCCCAAACTGGTTGTAGTTTTACTGTGGATAAGAAGCTTTCTT<br>AACCTAATTTGTTCTCCACAGGCTCCTTGAGAGTACGATGTTA<br>ACACATTGATGATGGGAGACGCTAATTTGCGTGTTGAATACT<br>TCATGCGGTATCCTATAAAAATGTTAACTACTATTAAATAAA<br>ACAGTAAAAAAAGCAATGGATGCTATGAACAAAATGTTACG<br>AATGTGTTTTTACATGAGAATGCTTGAATAATAAGAAACTCC<br>GCCATCAATATTGAAAAAATTTTTTTGAAAACCTTTTATTTAG<br>TAATGAGAAGGCAATAAGCTACGGAAGATCAGTTTGTGGAA<br>TATAGAGCAGAAATTTATGCAGATTTGTTGTCAGATGTACAA<br>AATGATGCCGAATGTGAAAATAATATTATTCATAGTTGCATT<br>GAAGTTGATTTTATTAAGAATAGAAGCACTCGTCCTTTGCCG<br>AGATGTCATTTCACTAATGTCTGGAAAAGTATTCATCTTTTTC<br>AGGCTTTAATGAAAAAGATGAATACATATCTTTATCTTACGA<br>AACAAGTTCATGGACTAAGCAAGACAAAATTACTGTTCTGG<br>AAGATTTTATAGGAAACCCTGGAGTAAAGCAATTTCCATCCG<br>ATCATACTAGCATTAATGAAGTCGTAGGTTTATTCTTTGGAA<br>ATGATTTCTTTGAAATGCTTTACCGTGAAAATGAATACAAAT<br>GAAATAAAAACTAAAGTGGGTTGATGTAGAAATAAAGGAA<br>ATGAAACGATTTTTTTATCTTGTTTTACTTATGGTACATGTAA

							AAAAGGATCGCATTAGAGATTATTGGTCGACTGATTCATACATAGAAACTCCGATTTTTTTCAAAAGTAATGAGCCGGAACAGATTTGAACAAATTTTACAGTCGCTCCATTTCCGTAATAATGATTTTCAAACTAATTCGACAGATAGACTCTTCAAGTTAAGACCAATATTACATTACTGTGTAAAAAAATTTCAACTCATACATAAGCCAAATCAAGAAGTATCTTTTGGCAGAACATGGAGAACTGCTCCGATACATGATTGGTATGCCGGTTTTCGGCCTGGAGCATCCGTTGTAAAAGCAAGTAATAGAAAAGCACAAACTGCCTTGGCAAGGTTCCGCAGCGGCCATTTTCGGCCGATGAGGTTTTCTGCTGACAAGAGGGTCTACCCTTTAAGTTCAAAATGCAGCTTGACTGAGACGACTCCCTCTCATGTTCTATTGTGCATTGGTTGCACCAAGAACCATCTGTTTTATAACTCTGGGGCGTTTCTGGATCAGTTGTG

PhdEnzCho 31	W4VSJ0	1,09E-109	37,618	PF00135	63218.00<br>**58747.62**	MSSPSSNVIAGAQGL<br>GAAMCGKLQKNVH<br>KSLHLLFLLLPGTILA<br>**SSVTVNTPTGEVTG**<br>**SVMTYDDINLNVFL**<br>**GIPFAEPPVGNLRF**<br>**KRTVPVKPWTSPVI**<br>**ANSLPPACYQYSPD**<br>**NYPWEDKVQGQSE**<br>**DCLYLNIWAPEISN**<br>**SSEKKAVMFWVFS**<br>**GGFATGSGRMPLY**<br>**DGRVLTAIGDVIVV**<br>**AINYRLSVLGFFTSE**<br>**TEEAPGNVGLYDIL**<br>**EALKWTNENIAAFG**<br>**GDNKRITVFGNSA**<br>**GGVATGLLCVSPLS**<br>**KGLFQRAIMQSGC**<br>**PAYFLNDHRAENLK**<br>**LGQKLAEKMECAN**<br>**ENITLKKNPDDVIE**<br>**CLRSKAPKDMVRT**<br>**TFSLVPVFPVITFFTI**<br>**SGDDFLPVDPQKAI**<br>**REGNFNGVDSMIG**<br>**VTQDEGSFPFVNTF**<br>**TQITGPFGELNPRIN**	CGATAAACCTTTTGCAAGCAAGTTTCTCATGATCTGGCAATA<br>TTCTAGAAAGTGCAAACATGATTATGTAATATGAAAAATGCT<br>GTATATGAAACTCGAGGTATTTTTGATTTATTCTTTATACTTTC<br>ATATTACATATTCATCTTTACACTTTCATAACTATGCTTTCATG<br>AGTCTGCGTACATCAACATAACACATTAGAAAACTTTAATAT<br>CAATTCATATTCTCAATTTCATATTCTCAATTTAAGCTAGGAT<br>CCCCTTTATTTTGCAATTTAACTCAAAACTTCAAAGGAATAA<br>ACCTAAAATTATGCTTTTTTAACTACGGGAAGCTTCAAATTTT<br>ACGATAAACCTTTTGCAAGCAAGTTTCTCATGATCTGGCAAT<br>ATTCTAGAAAGTGCAAACATGATTATGTAATATGAAAAATGC<br>TGTATATGAAACTCAAGGTATTTTTGATTTATTTTTTATACTTT<br>CATATTACATAGTCATCTTTACACTTTCGTAATTCTGCGTATA<br>TCAACTGAAAACATTAGCAAACTTTAATATGCAATAAATAGC<br>TATTAGGATAACCTTTTCTTTTAATAACGTCACTATGCCTCTTT<br>TAAATAGAATTGACTAGCTTCGATGTTTACTCGGAGCTTATAT<br>TATTCCATTCTTCTCTCAATATGGCTTTCAACATAGCCTTTGA<br>AGCTATATTGTGTTGGCAGATTCTGCGTTCCAGCAAATCTCA<br>AAAACACTTTATCGGGTAAGTCAGGCGATTCTGGAGTTGTTT<br>ATGTGTAGGAGTAGATAACTATTTTAGCACGTCATATAAGAA<br>CTAGATATTCATAACTTGAACAGTGTGCTTGGGGACCTTGTC<br>CAATTAGAAATAATAACTGTTGTCTATCTTAAGTACTCTGCAT<br>TAAATTTCGTTTTAAAATGTTTAAATAATCCATTTTATTCAATT<br>GTGTCCTGAATAAAGTGTAGTTGTCCCACGCCTCAGTTTTAG<br>ATACACCCCCAAATAATAATACTTTAGCCACCGTGTTTTACA<br>GTTGGCATTAAATATTCTTACTTAAGCTCTTTAACAGGTTTTC<br>GGTACAAAAGCTTGAATCCTTTAATGTCAAATACGCAGAATC<br>TGCTCTAATCGGAAAATATTACCAGTTGCAGAGCTCATTTTG<br>CTTATTAATATATTCTTTTGCAACGTCAATGCTTAAATCTATT

						**KTLGESLMRDYFGT**<br>**IFPDPEGIIQHYLSG**<br>**VKDDDYATIRSQV**<br>**YHSLGDFMVMCPS**<br>**KYFAEKVAEKNNKI**<br>**YRYYFNHRPSSTPY**<br>**AEWMGVMHCDD**<br>**MQFIFGRPLTPLSN**<br>**YTRKEEELSFEMVK**<br>**EWTNFAKRGEPGET**<br>**WRKFSKSDPYVKVF**<br>**ELSENRTNYLKSSEE**<br>**NCEFFRPYFGF***	ATCTCAGGAAATGTAAAACAGCTAAAAGGGATGTTACAACA<br>GTTTCTCTTCGAGCCGTCACATTTATCTTTTGTTCTTTCGCCTC<br>TTGTGTTTTACCTATTCCTCGACATATTTATGGCTATTTTTCAG<br>TTCACAACTCTCTCGGATGGCGTAATCTTTTCTTGAAATTTCTT<br>CGAATTTTCTCTCGTTTTATATATATCAAAGCGGTAATGCGTT<br>TTGCATTTTGTTCCGTTTATAGTAACGTGCGCACCTAATACTT<br>TCACCTGCTTCATGAAGGACTGTATCAAATAGTCCGAAGTAT<br>GCTGCAGCTTTCCTGTCTTACTGTGGATCAGAATCTTTTTTTGC<br>AGCTTTATGGTATTCACTGCCTCAGTGACGAGACTTTGAAAA<br>TTTCAATCATGTTTTAAAATTCCGCATATATCAACACGAAAA<br>TGGTGTCTCCAAGGATTTGGAAAATTGAATTTCTACTCGCAA<br>GTTACCTTGGTGTTGCAATTAAAATGAGGTCCATCACACAAG<br>GATGGGATAAAGTCGAACACGTAAACTGTATAGCCTTCTACA<br>AATTTGTCTTTCGAGAAGTAAATACCTCTGTTTTGATTAAAGT<br>TGATAAACAATTTGGTGACAAGCGCGAATATATCTTTTAGAA<br>TCAAAATTCGGTTATAAATGTTTGCTTGGCACTGAGTGACCA<br>TCTACTTAAACACTTAAAAAGTTGACCATGATAATGATGAAA<br>AAAAGGAAGAGGGCAGGTTATGTTTCCTATTTACCGTGGCAT<br>TCTTCAGGGAGGTAATAATCACTCCTTTGGACATCAGTCACA<br>AAAATACATTGTCTTTCATAAAAGAAAATGTTCCAACTGAAA<br>CTGAGTATGCTTTATACAATACTCTGTCGATGGTATATTTCGA<br>GAGACTTTTTTCGAGAACATTGACATGTCCCAAGGATACGCC<br>GGGACATTCACTGTCCTAATTAACAAGGAGGCATGCTCAGAT<br>ACGATTTTATACTGGTCTCATTTTGGGGGGGAGACTAAACA<br>AAAACTGTGTCTGTTGTGGCTCATTTTGATTCGCAGATCATCA<br>AATTTAATAAAAGTAGATTTTGTTGAAAACATTCGCATCATC<br>TCCAACGTTCGATTTTTAGCTGTAAGTTCATTACGCTTCGTCA<br>AACCTACATTTCTATCGATTTCTTTCAACGGGTTCGTCGAATG

							TAATTTGCTGTCTTTGTAAAAGCATTCAACGGGGGAGTAAAC<br>TTTTCTTGACATTTTTTCCATATATCCAAATTCTTCAATATAGG<br>TTCTGTAGGGATAAGTATTGCTATAGACGACGAGACGACTCG<br>CTTCTTAAGTTGATTTCCACTCTGAAAAAAAGAGCGTGTAAA<br>ATTAAATTCACGGGGTCGACGAGTTCATTTTCAGGTAAAGCG<br>GATCTATCCGACTTCATAATTTTGGCTTTTACGTGCAGTTAGG<br>AAGACGATAAGTCAATGTAATCTTCACCATTGTTACATTCCA<br>CGGGTCCACCATCGCGTAGGTTGGACAGGGGAGGATATTCA<br>ATAAAATGTCCTCTTCGATTGCGGTCTCGCATAAGCGGGATC<br>ATGAACAAATGGAAATCAGATTTTGCGCAAGCAACACAGTA<br>TTGATAGATGCGTGTCGATATGCTTAACCGAAAATAACGCGT<br>GATTCCATTTTCTTGGTTCTTGGTGAACTAATGCGTTATTCCA<br>ACGCTTTCTCTTTTAAATTATGACCAGAAGTAATAATGGACTT<br>CATTTTTCAAGCTACTTTGTCCGTTAATTGGCTTCCAGCTTATT<br>TCGAATAGCGTTTAACAGACATTTTTAGAACATTCAATATGC<br>CCACACTGCTGTGAAATCATCGTTTGCCGACTACTTTTATGCC<br>CAGTTTTAAAAGGGGAACTGTCACTCGATAAAAAGGACAAA<br>AGAATCTGCCAAAACCATAAACTGTCTGAAATGTATCCCCTT<br>TATAATAATTCAACTCGCCACGCTTTGCATAATCCTCGAAGG<br>ATTTTGGACAACATTGATGAGGCACATACATCACGATAGAA<br>ACTTTTGCCAAAAGTACAGTTTTACGTACGAGCGCTTGAGTT<br>CAGATGACACAATTTCGCGTGTATGCATTCTTATAACGACAT<br>TCAATACTTTTCTATTCCGTTGGAAGTAATGGAGTCTTTCATA<br>TTGTGCTCAAATCATTTCCCCGTCGCTTCCGGTCACGCTCAAG<br>ATGCGCAACAAGTGCGCTAACACATCTCCCACAATATCAGG<br>CTCTACAATGTCCGAATATATCAACAGTAATCTATAATGAGC<br>GCACAGATATGCCGCTTACTGACTTTCGACGTTCTTTTCTTCA<br>TCATAATCATCATCGGTACTCTCAAGTTCAAACATAGCATTT

							GCGCGAGTCCGTCGTACAGTATCACTTTAGCACCCTGCAAAA<br>CAAAAACAGAATCTGCCCCTTCCGGTAGACGCACTATGTTAA<br>GAGTTCCCGAAATTGGAATCGATGCAAAACTTTGAGGGTAG<br>ACAGAATATAGTATATAGACAAAATATGTCCAGATCGAAAT<br>AGAGTTTTAGGTCCAGATCAAGCTTTCAAGTTGTGAGTCTAG<br>CATCGTTCTATGGTGGGAAATGTGTTAAAAATATTTTTTAAAT<br>GCTGCACCCTTTACTGTGTTGTTTACATGCAGTGTATATATTC<br>AGCTATATACATGCAGTGTATATATATTGTTTATATTCAGCTA<br>CTCTTCTCGGCTACAGCGGCAGATAATTCAATTAGCCATCTC<br>GCAAGAAAGGAAATATGACTGTGTTCCTGACTAAAGTACAC<br>CTGGCATGTATGCTTGTACCTAAAAGGTCCGAATTCGAGAAT<br>TTTCTCGAAAGATAATTAAGGAGCGTCAGGGAAATTGTTGGA<br>GGTCAGTGATGAAAGGGCGACGATTGTTTTTCTGACGTCATA<br>GGTCTGGGTTTTTTTTTTTACAACTTCCGTGTCGAAATTGCACT<br>ATTTCCTGCAGAGATCAGAGCTATATATTAACAGCGTTTCAG<br>GGTATTACTGTGCTATGAATATATTCCAGATTTTATGCGTGTT<br>TTAACGTATATCACTTGCAAAGATGTATTTTTATTGTCTTAAA<br>ATACTACCATGATGTGGTGTACCTCGGAAAAATAAACTTACT<br>TTTACATATATAAGAGTGTTTATTGCTGTAGCAGGAAAGTTTA<br>CCTTCACTTTATTTAATGCTATATAATTGTACCTTACAAAATT<br>TTGTAGCTTATAATAAAGTGAATAATCTTAATATTTCAACGAT<br>ATTTGGAATGCTGTCAGCACAATTCAAACAACTTTCAGTGAA<br>TTATAGTGATTTCTCTTTAACAAGAATGCGATTAAAATCCAA<br>AATAAGGTCGGAAAAATTCACAGTTTTCTTCGCTAGATTTCA<br>AGTAATTTGTACGGTTTTCAGATAGTTCAAAAACTTTGACGT<br>ATGGATCTGACTTAGAGAACTTCCGCCACGTTTCACCGGGTT<br>CCCCTCTCTTAGCAAAATTCGTCCACTCTTTCACCATCTCAAA<br>GCTTAGTTCTTCTTCCTTTCGGGTGTAGTTGGACAGAGGAGTT

							AGAGGACGTCCAAAGATAAATTGCATATCGTCACAATGCAT<br>AACCCCCATCCACTCTGCGTACGGAGTACTCGATGGTCTGTG<br>ATTGAAATAGTATCGGTAGATTTTATTATTTTTTTCTGCAACTT<br>TCTCAGCAAAATATTTGCTTGGGCACATAACCATGAAATCCC<br>CAAGAGAGTGATAAACTTGACTTCGTATTGTAGCATAATCGT<br>CGTCTTTAACGCCACTGAGGTAATGCTGGATTATTCCTTCCGG<br>ATCTGGAAATATTGTCCCGAAATAATCTCTCATGAGAGACTC<br>CCCTAACGTCTTATTAATGCGAGGATTTAATTCACCAAAAGG<br>GCCTGTGATCTGTGTAAAAGTGTTAACAAATGGGAATGATCC<br>CTCGTCTTGAGTAACTCCTATCATAGAATCAACACCGTTAAA<br>GTTTCCTTCTCTGATAGCTTTTTGTGGGTCTACCGGTAGAAAA<br>TCATCCCCACTGATGGTAAAGAAAGTTATCACTGGGAAGAC<br>GGGAACTAAGGAAAACGTAGTTCTCACCATATCTTTTGGAGC<br>TTTACTTCTTAGGCATTCGATCACATCATCAGGATTTTTCTTCA<br>GCGTTATGTTTTCATTTGCACATTCCATCTTCTCTGCGAGCTTT<br>TGTCCTAATTTAAGATTCTCAGCCCTATGGTCATTCAGAAAAT<br>ATGCTGGACAACCACTTTGCATTATTGCTCTCTGGAAGAGAC<br>CTTTCGAAAGAGGAGAAACACACAGCAAACCTGTAGCTACG<br>CCGCCGGCGCTGTTGCCGAACACCGTAATTCTTTTATTGTCTC<br>CGCCGAATGCAGCAATGTTTTCGTTCGTCCATTTAAGGGCTTC<br>CAAAATGTCATACAAGCCGACGTTTCCAGGAGCTTCTTCGGT<br>TTCAGATGTGAAGAATCCGAGCACACTTAACCGGTAGTTTAT<br>CGCAACAACTATTACATCTCCAATTGCAGTTAGAACACGTCC<br>ATCATATAGAGGCATCCTACCTGAACCGGTTGCAAAGCCGCC<br>TGAGAACACCCAAAACATGACGGCTTTTTTCTCGCTACTATT<br>GGAAATTTCTGGTGCCCATATATTCAAATACAAGCAGTCTTC<br>GCTTTGACCCTGAACTTTATCTTCCCACGGATAGTTGTCCGGA<br>GAGTACTGGTAACAAGCAGGCGGTAAGCTGTTTGCTATTACA

							GGAGAGGTCCAAGGTTTGACAGGAACTGTTCGCTTAAAGCGT<br>AAATTCCCCACTGGAGGCTCAGCAAACGGAATTCCGAGAAA<br>GACATTTAGATTTATGTCGTCATAAGTCATAACAGAACCAGT<br>CACTTCACCTGTAGGAGTATTAACTGTAACGCTGGATGCTAA<br>GATAGTTCCTGGTAGCAGTAAAAATAGTAGATGCAAGCTCTT<br>ATGAACGTTTTTCTGGAGCTTTCCGCACATAGCGGCTCCTAA<br>ACCTTGGGCTCCAGCAATAACGTTAGACGAGGGAGACGACA<br>TGATGCTGCTGCCTAGATCTCCAGTGTGCTGTGTCGTACACA<br>AATGCGCCTATTTCCTGCTAGTGTAGGTCTTCATCCGAATTAT<br>GCCCAAACTGGTTGTAGTTTTACTGTGGATAAGAAGCTTTCTT<br>AACCTAATTTGTTCTCCACAGGCTCCTTGAGAGTACGATGTTA<br>ACACATTGATGATGGGAGACGCTAATTTGCGTGTTGAATACT<br>TCATGCGGTATCCTATAAAAATGTTAACTACTATTAAATAAA<br>ACAGTAAAAAAAGCAATGGATGCTATGAACAAAATGTTACG<br>AATGTGTTTTTACATGAGAATGCTTGAATAATAAGAAACTCC<br>GCCATCAATATTGAAAAAATTTTTTTGAAAACCTTTTATTTAG<br>TAATGAGAAGGCAATAAGCTACGGAAGATCAGTTTGTGGAA<br>TATAGAGCAGAAATTTATGCAGATTTGTTGTCAGATGTACAA<br>AATGATGCCGAATGTGAAAATAATATTATTCATAGTTGCATT<br>GAAGTTGATTTTATTAAGAATAGAAGCACTCGTCCTTTGCCG<br>AGATGTCATTTCACTAATGTCTGGAAAAGTATTCATCTTTTTC<br>AGGCTTTAATGAAAAAGATGAATACATATCTTTATCTTACGA<br>AACAAGTTCATGGACTAAGCAAGACAAAATTACTGTTCTGG<br>AAGATTTTATAGGAAACCCTGGAGTAAAGCAATTTCCATCCG<br>ATCATACTAGCATTAATGAAGTCGTAGGTTTATTCTTTGGAA<br>ATGATTTCTTTGAAATGCTTTACCGTGAAAATGAATACAAAT<br>GAAATAAAAACTAAAGTGGGTTGATGTAGAAATAAAGGAA<br>ATGAAACGATTTTTTTATCTTGTTTTACTTATGGTACATGTAA

							AAAAGGATCGCATTAGAGATTATTGGTCGACTGATTCATACATAGAAACTCCGATTTTTTTCAAAAGTAATGAGCCGGAACAGATTTGAACAAATTTTACAGTCGCTCCATTTCCGTAATAATGATTTTCAAACTAATTCGACAGATAGACTCTTCAAGTTAAGACCAATATTACATTACTGTGTAAAAAAATTTCAACTCATACATAAGCCAAATCAAGAAGTATCTTTTGGCAGAACATGGAGAACTGCTCCGATACATGATTGGTATGCCGGTTTTCGGCCTGGAGCATCCGTTGTAAAAGCAAGTAATAGAAAAGCACAAACTGCCTTGGCAAGGTTCCGCAGCGGCCATTTTCGGCCGATGAGGTTTTCTGCTGACAAGAGGGTCTACCCTTTAAGTTCAAAATGCAGCTTGACTGAGACGACTCCCTCTCATGTTCTATTGTGCATTGGTTGCACCAAGAACCATCTGTTTTATAACTCTGGGGCGTTTCTGGATCAGTTGTG

PhdEnzCho 32	W4VSJ0	1,09E-109	37,618	PF00135	63218.00<br><br>**58747.62**	MSSPSSNVIAGAQGL<br>GAAMCGKLQKNVH<br>KSLHLLFLLLPGTILA<br>**SSVTVNTPTGEVTG**<br>**SVMTYDDINLNVFL**<br>**GIPFAEPPVGNLRF**<br>**KRTVPVKPWTSPVI**<br>**ANSLPPACYQYSPD**<br>**NYPWEDKVQGQSE**<br>**DCLYLNIWAPEISN**<br>**SSEKKAVMFWVFS**<br>**GGFATGSGRMPLY**<br>**DGRVLTAIGDVIVV**<br>**AINYRLSVLGFFTSE**<br>**TEEAPGNVGLYDIL**<br>**EALKWTNENIAAFG**<br>**GDNKRITVFGNSA**<br>**GGVATGLLCVSPLS**<br>**KGLFQRAIMQSGC**<br>**PAYFLNDHRAENLK**<br>**LGQKLAEKMECAN**<br>**ENITLKKNPDDVIE**<br>**CLRSKAPKDMVRT**<br>**TFSLVPVFPVITFFTI**<br>**SGDDFLPVDPQKAI**<br>**REGNFNGVDSMIG**<br>**VTQDEGSFPFVNTF**<br>**TQITGPFGELNPRIN**	CGATAAACCTTTTGCAAGCAAGTTTCTCATGATCTGGCAATA<br>TTCTAGAAAGTGCAAACATGATTATGTAATATGAAAAATGCT<br>GTATATGAAACTCGAGGTATTTTTGATTTATTCTTTATACTTTC<br>ATATTACATATTCATCTTTACACTTTCATAACTATGCTTTCATG<br>AGTCTGCGTACATCAACATAACACATTAGAAAACTTTAATAT<br>CAATTCATATTCTCAATTTCATATTCTCAATTTAAGCTAGGAT<br>CCCCTTTATTTTGCAATTTAACTCAAAACTTCAAAGGAATAA<br>ACCTAAAATTATGCTTTTTTAACTACGGGAAGCTTCAAATTTT<br>ACGATAAACCTTTTGCAAGCAAGTTTCTCATGATCTGGCAAT<br>ATTCTAGAAAGTGCAAACATGATTATGTAATATGAAAAATGC<br>TGTATATGAAACTCAAGGTATTTTTGATTTATTTTTTATACTTT<br>CATATTACATAGTCATCTTTACACTTTCGTAATTCTGCGTATA<br>TCAACTGAAAACATTAGCAAACTTTAATATGCAATAAATAGC<br>TATTAGGATAACCTTTTCTTTTAATAACGTCACTATGCCTCTTT<br>TAAATAGAATTGACTAGCTTCGATGTTTACTCGGAGCTTATAT<br>TATTCCATTCTTCTCTCAATATGGCTTTCAACATAGCCTTTGA<br>AGCTATATTGTGTTGGCAGATTCTGCGTTCCAGCAAATCTCA<br>AAAACACTTTATCGGGTAAGTCAGGCGATTCTGGAGTTGTTT<br>ATGTGTAGGAGTAGATAACTATTTTAGCACGTCATATAAGAA<br>CTAGATATTCATAACTTGAACAGTGTGCTTGGGGACCTTGTC<br>CAATTAGAAATAATAACTGTTGTCTATCTTAAGTACTCTGCAT<br>TAAATTTCGTTTTAAAATGTTTAAATAATCCATTTTATTCAATT<br>GTGTCCTGAATAAAGTGTAGTTGTCCCACGCCTCAGTTTTAG<br>ATACACCCCCAAATAATAATACTTTAGCCACCGTGTTTTACA<br>GTTGGCATTAAATATTCTTACTTAAGCTCTTTAACAGGTTTTC<br>GGTACAAAAGCTTGAATCCTTTAATGTCAAATACGCAGAATC<br>TGCTCTAATCGGAAAATATTACCAGTTGCAGAGCTCATTTTG<br>CTTATTAATATATTCTTTTGCAACGTCAATGCTTAAATCTATT

						**KTLGESLMRDYFGT<br>IFPDPEGIIQHYLSG<br>VKDDDYATIRSQV<br>YHSLGDFMVMCPS<br>KYFAEKVAEKNNKI<br>YRYYFNHRPSSTPY<br>AEWMGVMHCDD<br>MQFIFGRPLTPLSN<br>YTRKEEELSFEMVK<br>EWTNFAKRGEPGET<br>WRKFSKSDPYVKVF<br>ELSENRTNYLKSSEE<br>NCEFFRPYFGF***	ATCTCAGGAAATGTAAAACAGCTAAAAGGGATGTTACAACA<br>GTTTCTCTTCGAGCCGTCACATTTATCTTTTGTTCTTTCGCCTC<br>TTGTGTTTTACCTATTCCTCGACATATTTATGGCTATTTTTCAG<br>TTCACAACTCTCTCGGATGGCGTAATCTTTTCTTGAAATTTCTT<br>CGAATTTTCTCTCGTTTTATATATATCAAAGCGGTAATGCGTT<br>TTGCATTTTGTTCCGTTTATAGTAACGTGCGCACCTAATACTT<br>TCACCTGCTTCATGAAGGACTGTATCAAATAGTCCGAAGTAT<br>GCTGCAGCTTTCCTGTCTTACTGTGGATCAGAATCTTTTTTTGC<br>AGCTTTATGGTATTCACTGCCTCAGTGACGAGACTTTGAAAA<br>TTTCAATCATGTTTTAAAATTCCGCATATATCAACACGAAAA<br>TGGTGTCTCCAAGGATTTGGAAAATTGAATTTCTACTCGCAA<br>GTTACCTTGGTGTTGCAATTAAAATGAGGTCCATCACACAAG<br>GATGGGATAAAGTCGAACACGTAAACTGTATAGCCTTCTACA<br>AATTTGTCTTTCGAGAAGTAAATACCTCTGTTTTGATTAAAGT<br>TGATAAACAATTTGGTGACAAGCGCGAATATATCTTTTAGAA<br>TCAAAATTCGGTTATAAATGTTTGCTTGGCACTGAGTGACCA<br>TCTACTTAAACACTTAAAAAGTTGACCATGATAATGATGAAA<br>AAAGGAAGAGGGCAGGTTATGTTTCCTATTTACCGTGGCATT<br>CTTCAGGGAGGTAATAATCACTCCTTTGGACATCAGTCACAA<br>AAATACATTGTCTTTCATAAAAGAAAATGTTCCAACTGAAAC<br>TGAGTATGCTTTATACAATACTCTGTCGATGGTATATTTCGAG<br>AGACTTTTTTCGAGAACATTGACATGTCCCAAGGATACGCCG<br>GGACATTCACTGTCCTAATTAACAAGGAGGCATGCTCAGATA<br>CGATTTTATACTGGTCTCATTTTGGGGGGGAGACTAAACAA<br>AAACTGTGTCTGTTGTGGCTCATTTTGATTCGCAGATCATCAA<br>ATTTAATAAAAGTAGATTTTGTTGAAAACATTCGCATCATCTC<br>CAACGTTCGATTTTTAGCTGTAAGTTCATTACGCTTCGTCAAA<br>CCTACATTTCTATCGATTTCTTTCAACGGGTTCGTCGAATGTA

							ATTTGCTGTCTTTGTAAAAGCATTCAACGGGGGAGTAAACTT<br>TTCTTGACATTTTTTCCATATATCCAAATTCTTCAATATAGGTT<br>CTGTAGGGATAAGTATTGCTATAGACGACGAGACGACTCGCT<br>TCTTAAGTTGATTTCCACTCTGAAAAAAAGAGCGTGTAAAAT<br>TAAATTCACGGGGTCGACGAGTTCATTTTCAGGTAAAGCGGA<br>TCTATCCGACTTCATAATTTTGGCTTTTACGTGCAGTTAGGAA<br>GACGATAAGTCAATGTAATCTTCACCATTGTTACATTCCACG<br>GGTCCACCATCGCGTAGGTTGGACAGGGGAGGATATTCAAT<br>AAAATGTCCTCTTCGATTGCGGTCTCGCATAAGCGGGATCAT<br>GAACAAATGGAAATCAGATTTTGCGCAAGCAACACAGTATT<br>GATAGATGCGTGTCGATATGCTTAACCGAAAATAACGCGTG<br>ATTCCATTTTCTTGGTTCTTGGTGAACTAATGCGTTATTCCAA<br>CGCTTTCTCTTTTAAATTATGACCAGAAGTAATAATGGACTTC<br>ATTTTTCAAGCTACTTTGTCCGTTAATTGGCTTCCAGCTTATTT<br>CGAATAGCGTTTAACAGACATTTTTAGAACATTCAATATGCC<br>CACACTGCTGTGAAATCATCGTTTGCCGACTACTTTTATGCCC<br>AGTTTTAAAAGGGGAACTGTCACTCGATAAAAAGGACAAAA<br>GAATCTGCCAAAACCATAAACTGTCTGAAATGTATCCCCTTT<br>ATAATAATTCAACTCGCCACGCTTTGCATAATCCTCGAAGGA<br>TTTTGGACAACATTGATGAGGCACATACATCACGATAGAAA<br>CTTTTGCCAAAAGTACAGTTTTACGTACGAGCGCTTGAGTTC<br>AGATGACACAATTTCGCGTGTATGCATTCTTATAACGACATT<br>CAATACTTTTCTATTCCGTTGGAAGTAATGGAGTCTTTCATAT<br>TGTGCTCAAATCATTTCCCCGTCGCTTCCGGTCACGCTCAAGA<br>TGCGCAACAAGTGCGCTAACACATCTCCCACAATATCAGGCT<br>CTACAATGTCCGAATATATCAACAGTAATCTATAATGAGCGC<br>ACAGATATGCCGCTTACTGACTTTCGACGTTCTTTTCTTCATC<br>ATAATCATCATCGGTACTCTCAAGTTCAAACATAGCATTTGC

							GCGAGTCCGTCGTACAGTATCACTTTAGCACCCTGCAAAACA<br>AAAACAGAATCTGCCCCTTCCGGTAGACGCACTATGTTAAGA<br>GTTCCCGAAATTGGAATCGATGCAAAACTTTGAGGGTAGAC<br>AGAATATAGTATATAGACAAAATATGTCCAGATCGAAATAG<br>AGTTTTAGGTCCAGATCAAGCTTTCAAGTTGTGAGTCTAGCA<br>TCGTTCTATGGTGGGAAATGTGTTAAAAATATTTTTTAAATGC<br>TGCACCCTTTACTGTGTTGTTTACATGCAGTGTATATATTCAG<br>CTATATACATGCAGTGTATATATATTGTTTATATTCAGCTACT<br>CTTCTCGGCTACAGCGGCAGATAATTCAATTAGCCATCTCGC<br>AAGAAAGGAAATATGACTGTGTTCCTGACTAAAGTACACCT<br>GGCATGTATGCTTGTACCTAAAAGGTCCGAATTCGAGAATTT<br>TCTCGAAAGATAATTAAGGAGCGTCAGGGAAATTGTTGGAG<br>GTCAGTGATGAAAGGGCGACGATTGTTTTTCTGACGTCATAG<br>GTCTGGGTTTTTTTTTTTACAACTTCCGTGTCGAAATTGCACTA<br>TTTCCTGCAGAGATCAGAGCTATATATTAACAGCGTTTCAGG<br>GTATTACTGTGCTATGAATATATTCCAGATTTTATGCGTGTTTT<br>AACGTATATCACTTGCAAAGATGTATTTTTATTGTCTTAAAAT<br>ACTACCATGATGTGGTGTACCTCGGAAAAATAAACTTACTTT<br>TACATATATAAGAGTGTTTATTGCTGTAGCAGGAAAGTTTAC<br>CTTCACTTTATTTAATGCTATATAATTGTACCTTACAATATTGT<br>GTAGCTTATAATAAAGTGAATAATCTTAATATTTCAACGATA<br>TTTGGAATGCTGTCAGCACAATTCAAACAACTTTCAGTGAAT<br>TATAGTGATTTCTCTTTAACAAGAATGCGATTAAAATCCAAA<br>ATAAGGTCGGAAAAATTCACAGTTTTCTTCGCTAGATTTCAA<br>GTAATTTGTACGGTTTTCAGATAGTTCAAAAACTTTGACGTAT<br>GGATCTGACTTAGAGAACTTCCGCCACGTTTCACCGGGTTCC<br>CCTCTCTTAGCAAAATTCGTCCACTCTTTCACCATCTCAAAGC<br>TTAGTTCTTCTTCCTTTCGGGTGTAGTTGGACAGAGGAGTTAG

							AGGACGTCCAAAGATAAATTGCATATCGTCACAATGCATAA<br>CCCCCATCCACTCTGCGTACGGAGTACTCGATGGTCTGTGAT<br>TGAAATAGTATCGGTAGATTTTATTATTTTTTTCTGCAACTTTC<br>TCAGCAAAATATTTGCTTGGGCACATAACCATGAAATCCCCA<br>AGAGAGTGATAAACTTGACTTCGTATTGTAGCATAATCGTCG<br>TCTTTAACGCCACTGAGGTAATGCTGGATTATTCCTTCCGGAT<br>CTGGAAATATTGTCCCGAAATAATCTCTCATGAGAGACTCCC<br>CTAACGTCTTATTAATGCGAGGATTTAATTCACCAAAAGGGC<br>CTGTGATCTGTGTAAAAGTGTTAACAAATGGGAATGATCCCT<br>CGTCTTGAGTAACTCCTATCATAGAATCAACACCGTTAAAGT<br>TTCCTTCTCTGATAGCTTTTTGTGGGTCTACCGGTAGAAAATC<br>ATCCCCACTGATGGTAAAGAAAGTTATCACTGGGAAGACGG<br>GAACTAAGGAAAACGTAGTTCTCACCATATCTTTTGGAGCTT<br>TACTTCTTAGGCATTCGATCACATCATCAGGATTTTTCTTCAG<br>CGTTATGTTTTCATTTGCACATTCCATCTTCTCTGCGAGCTTTT<br>GTCCTAATTTAAGATTCTCAGCCCTATGGTCATTCAGAAAAT<br>ATGCTGGACAACCACTTTGCATTATTGCTCTCTGGAAGAGAC<br>CTTTCGAAAGAGGAGAAACACACAGCAAACCTGTAGCTACG<br>CCGCCGGCGCTGTTGCCGAACACCGTAATTCTTTTATTGTCTC<br>CGCCGAATGCAGCAATGTTTTCGTTCGTCCATTTAAGGGCTTC<br>CAAAATGTCATACAAGCCGACGTTTCCAGGAGCTTCTTCGGT<br>TTCAGATGTGAAGAATCCGAGCACACTTAACCGGTAGTTTAT<br>CGCAACAACTATTACATCTCCAATTGCAGTTAGAACACGTCC<br>ATCATATAGAGGCATCCTACCTGAACCGGTTGCAAAGCCGCC<br>TGAGAACACCCAAAACATGACGGCTTTTTTCTCGCTACTATT<br>GGAAATTTCTGGTGCCCATATATTCAAATACAAGCAGTCTTC<br>GCTTTGACCCTGAACTTTATCTTCCCACGGATAGTTGTCCGGA<br>GAGTACTGGTAACAAGCAGGCGGTAAGCTGTTTGCTATTACA

							GGAGAGGTCCAAGGTTTGACAGGAACTGTTCGCTTAAAGCGT<br>AAATTCCCCACTGGAGGCTCAGCAAACGGAATTCCGAGAAA<br>GACATTTAGATTTATGTCGTCATAAGTCATAACAGAACCAGT<br>CACTTCACCTGTAGGAGTATTAACTGTAACGCTGGATGCTAA<br>GATAGTTCCTGGTAGCAGTAAAAATAGTAGATGCAAGCTCTT<br>ATGAACGTTTTTCTGGAGCTTTCCGCACATAGCGGCTCCTAA<br>ACCTTGGGCTCCAGCAATAACGTTAGACGAGGGAGACGACA<br>TGATGCTGCTGCCTAGATCTCCAGTGTGCTGTGTCGTACACA<br>AATGCGCCTATTTCCTGCTAGTGTAGGTCTTCATCCGAATTAT<br>GCCCAAACTGGTTGTAGTTTTACTGTGGATAAGAAGCTTTCTT<br>AACCTAATTTGTTCTCCACAGGCTCCTTGAGAGTACGATGTTA<br>ACACATTGATGATGGGAGACGCTAATTTGCGTGTTGAATACT<br>TCATGCGGTATCCTATAAAAATGTTAACTACTATTAAATAAA<br>ACAGTAAAAAAAGCAATGGATGCTATGAACAAAATGTTACG<br>AATGTGTTTTTACATGAGAATGCTTGAATAATAAGAAACTCC<br>GCCATCAATATTGAAAAAATTTTTTTGAAAACCTTTTATTTAG<br>TAATGAGAAGGCAATAAGCTACGGAAGATCAGTTTGTGGAA<br>TATAGAGCAGAAATTTATGCAGATTTGTTGTCAGATGTACAA<br>AATGATGCCGAATGTGAAAATAATATTATTCATAGTTGCATT<br>GAAGTTGATTTTATTAAGAATAGAAGCACTCGTCCTTTGCCG<br>AGATGTCATTTCACTAATGTCTGGAAAAGTATTCATCTTTTTC<br>AGGCTTTAATGAAAAAGATGAATACATATCTTTATCTTACGA<br>AACAAGTTCATGGACTAAGCAAGACAAAATTACTGTTCTGG<br>AAGATTTTATAGGAAACCCTGGAGTAAAGCAATTTCCATCCG<br>ATCATACTAGCATTAATGAAGTCGTAGGTTTATTCTTTGGAA<br>ATGATTTCTTTGAAATGCTTTACCGTGAAAATGAATACAAAT<br>GAAATAAAAACTAAAGTGGGTTGATGTAGAAATAAAGGAA<br>ATGAAACGATTTTTTTATCTTGTTTTACTTATGGTACATGTAA

							AAAAGGATCGCATTAGAGATTATTGGTCGACTGATTCATACA TAGAAACTCCGATTTTTTTCAAAAGTAATGAGCCGGAACAGA TTTGAACAAATTTTACAGTCGCTCCATTTCCGTAATAATGATT TTCAAACTAATTCGACAGATAGACTCTTCAAGTTAAGACCAA TATTACATTACTGTGTAAAAAAATTTCAACTCATACATAAGC CAAATCAAGAAGTATCTTTTGGCAGAACATGGAGAACTGCTC CGATACATGATTGGTATGCCGGTTTTCGGCCTGGAGCATCCG TTGTAAAAGCAAGTAATAGAAAAGCACAAACTGCCTTGGCA AGGTTCCGCAGCGGCCATTTTCGGCCGATGAGGTTTTCTGCTG ACAAGAGGGTCTACCCTTTAAGTTCAAAATGCAGCTTGACTG AGACGACTCCCTCTCATGTTCTATTGTGCATTGGTTGCACCAA GAACCATCTGTTTTATAACTCTGGGGCGTTTCTGGATCAGTTG TG

PhdEnzCho 33	W4VSJ0	8,78E-110	37,618	PF00135	63218.00<br><br>**58747.62**	MSSPSSNVIAGAQGL<br>GAAMCGKLQKNVH<br>KSLHLLFLLLPGTILA<br>**SSVTVNTPTGEVTG**<br>**SVMTYDDINLNVFL**<br>**GIPFAEPPVGNLRF**<br>**KRTVPVKPWTSPVI**<br>**ANSLPPACYQYSPD**<br>**NYPWEDKVQGQSE**<br>**DCLYLNIWAPEISN**<br>**SSEKKAVMFWVFS**<br>**GGFATGSGRMPLY**<br>**DGRVLTAIGDVIVV**<br>**AINYRLSVLGFFTSE**<br>**TEEAPGNVGLYDIL**<br>**EALKWTNENIAAFG**<br>**GDNKRITVFGNSA**<br>**GGVATGLLCVSPLS**<br>**KGLFQRAIMQSGC**<br>**PAYFLNDHRAENLK**<br>**LGQKLAEKMECAN**<br>**ENITLKKNPDDVIE**<br>**CLRSKAPKDMVRT**<br>**TFSLVPVFPVITFFTI**<br>**SGDDFLPVDPQKAI**<br>**REGNFNGVDSMIG**<br>**VTQDEGSFPFVNTF**<br>**TQITGPFGELNPRIN**	CGATAAACCTTTTGCAAGCAAGTTTCTCATGATCTGGCAATA<br>TTCTAGAAAGTGCAAACATGATTATGTAATATGAAAAATGCT<br>GTATATGAAACTCGAGGTATTTTTGATTTATTCTTTATACTTTC<br>ATATTACATATTCATCTTTACACTTTCATAACTATGCTTTCATG<br>AGTCTGCGTACATCAACATAACACATTAGAAAACTTTAATAT<br>CAATTCATATTCTCAATTTCATATTCTCAATTTAAGCTAGGAT<br>CCCCTTTATTTTGCAATTTAACTCAAAACTTCAAAGGAATAA<br>ACCTAAAATTATGCTTTTTTAACTACGGGAAGCTTCAAATTTT<br>ACGATAAACCTTTTGCAAGCAAGTTTCTCATGATCTGGCAAT<br>ATTCTAGAAAGTGCAAACATGATTATGTAATATGAAAAATGC<br>TGTATATGAAACTCAAGGTATTTTTGATTTATTTTTTATACTTT<br>CATATTACATAGTCATCTTTACACTTTCGTAATTCTGCGTATA<br>TCAACTGAAAACATTAGCAAACTTTAATATGCAATAAATAGC<br>TATTAGGATAACCTTTTCTTTTAATAACGTCACTATGCCTCTTT<br>TAAATAGAATTGACTAGCTTCGATGTTTACTCGGAGCTTATAT<br>TATTCCATTCTTCTCTCAATATGGCTTTCAACATAGCCTTTGA<br>AGCTATATTGTGTTGGCAGATTCTGCGTTCCAGCAAATCTCA<br>AAAACACTTTATCGGGTAAGTCAGGCGATTCTGGAGTTGTTT<br>ATGTGTAGGAGTAGATAACTATTTTAGCACGTCATATAAGAA<br>CTAGATATTCATAACTTGAACAGTGTGCTTGGGGACCTTGTC<br>CAATTAGAAATAATAACTGTTGTCTATCTTAAGTACTCTGCAT<br>TAAATTTCGTTTTAAAATGTTTAAATAATCCATTTTATTCAATT<br>GTGTCCTGAATAAAGTGTAGTTGTCCCACGCCTCAGTTTTAG<br>ATACACCCCCAAATAATAATACTTTAGCCACCGTGTTTTACA<br>GTTGGCATTAAATATTCTTACTTAAGCTCTTTAACAGGTTTTC<br>GGTACAAAAGCTTGAATCCTTTAATGTCAAATACGCAGAATC<br>TGCTCTAATCGGAAAATATTACCAGTTGCAGAGCTCATTTTG<br>CTTATTAATATATTCTTTTGCAACGTCAATGCTTAAATCTATT

						**KTLGESLMRDYFGT**<br>**IFPDPEGIIQHYLSG**<br>**VKDDDYATIRSQV**<br>**YHSLGDFMVMCPS**<br>**KYFAEKVAEKNNKI**<br>**YRYYFNHRPSSTPY**<br>**AEWMGVMHCDD**<br>**MQFIFGRPLTPLSN**<br>**YTRKEEELSFEMVK**<br>**EWTNFAKRGEPGET**<br>**WRKFSKSDPYVKVF**<br>**ELSENRTNYLKSSEE**<br>**NCEFFRPYFGF***	ATCTCAGGAAATGTAAAACAGCTAAAAGGGATGTTACAACA<br>GTTTCTCTTCGAGCCGTCACATTTATCTTTTGTTCTTTCGCCTC<br>TTGTGTTTTACCTATTCCTCGACATATTTATGGCTATTTTTCAG<br>TTCACAACTCTCTCGGATGGCGTAATCTTTTCTTGAAATTTCTT<br>CGAATTTTCTCTCGTTTTATATATATCAAAGCGGTAATGCGTT<br>TTGCATTTTGTTCCGTTTATAGTAACGTGCGCACCTAATACTT<br>TCACCTGCTTCATGAAGGACTGTATCAAATAGTCCGAAGTAT<br>GCTGCAGCTTTCCTGTCTTACTGTGGATCAGAATCTTTTTTTGC<br>AGCTTTATGGTATTCACTGCCTCAGTGACGAGACTTTGAAAA<br>TTTCAATCATGTTTTAAAATTCCGCATATATCAACACGAAAA<br>TGGTGTCTCCAAGGATTTGGAAAATTGAATTTCTACTCGCAA<br>GTTACCTTGGTGTTGCAATTAAAATGAGGTCCATCACACAAG<br>GATGGGATAAAGTCGAACACGTAAACTGTATAGCCTTCTACA<br>AATTTGTCTTTCGAGAAGTAAATACCTCTGTTTTGATTAAAGT<br>TGATAAACAATTTGGTGACAAGCGCGAATATATCTTTTAGAA<br>TCAAAATTCGGTTATAAATGTTTGCTTGGCACTGAGTGACCA<br>TCTACTTAAACACTTAAAAAGTTGACCATGATAATGATGAAA<br>AAAAGGAAGAGGGCAGGTTATGTTTCCTATTTACCGTGGCAT<br>TCTTCAGGGAGGTAATAATCACTCCTTTGGACATCAGTCACA<br>AAAATACATTGTCTTTCATAAAAGAAAATGTTCCAACTGAAA<br>CTGAGTATGCTTTATACAATACTCTGTCGATGGTATATTTCGA<br>GAGACTTTTTTCGAGAACATTGACATGTCCCAAGGATACGCC<br>GGGACATTCACTGTCCTAATTAACAAGGAGGCATGCTCAGAT<br>ACGATTTTATACTGGTCTCATTTTGGGGGGGGAGACTAAACA<br>AAAACTGTGTCTGTTGTGGCTCATTTTGATTCGCAGATCATCA<br>AATTTAATAAAAGTAGATTTTGTTGAAAACATTCGCATCATC<br>TCCAACGTTCGATTTTTAGCTGTAAGTTCATTACGCTTCGTCA<br>AACCTACATTTCTATCGATTTCTTTCAACGGGTTCGTCGAATG

							TAATTTGCTGTCTTTGTAAAAGCATTCAACGGGGGAGTAAAC<br>TTTTCTTGACATTTTTTCCATATATCCAAATTCTTCAATATAGG<br>TTCTGTAGGGATAAGTATTGCTATAGACGACGAGACGACTCG<br>CTTCTTAAGTTGATTTCCACTCTGAAAAAAAGAGCGTGTAAA<br>ATTAAATTCACGGGGTCGACGAGTTCATTTTCAGGTAAAGCG<br>GATCTATCCGACTTCATAATTTTGGCTTTTACGTGCAGTTAGG<br>AAGACGATAAGTCAATGTAATCTTCACCATTGTTACATTCCA<br>CGGGTCCACCATCGCGTAGGTTGGACAGGGGAGGATATTCA<br>ATAAAATGTCCTCTTCGATTGCGGTCTCGCATAAGCGGGATC<br>ATGAACAAATGGAAATCAGATTTTGCGCAAGCAACACAGTA<br>TTGATAGATGCGTGTCGATATGCTTAACCGAAAATAACGCGT<br>GATTCCATTTTCTTGGTTCTTGGTGAACTAATGCGTTATTCCA<br>ACGCTTTCTCTTTTAAATTATGACCAGAAGTAATAATGGACTT<br>CATTTTTCAAGCTACTTTGTCCGTTAATTGGCTTCCAGCTTATT<br>TCGAATAGCGTTTAACAGACTTTTTAGAACATTCAATATGCC<br>CACACTGCTGTGAAATCATCGTTTGCCGACTACTTTTATGCCC<br>AGTTTTAAAAGGGGAACTGTCACTCGATAAAAAGGACAAAA<br>GAATCTGCCAAAACCATAAACTGTCTGAAATGTATCCCCTTT<br>ATAATAATTCAACTCGCCACGCTTTGCATAATCCTCGAAGGA<br>TTTTGGACAACATTGATGAGGCACATACATCACGATAGAAA<br>CTTTTGCCAAAAGTACAGTTTTACGTACGAGCGCTTGAGTTC<br>AGATGACACAATTTCGCGTGTATGCATTCTTATAACGACATT<br>CAATACTTTTCTATTCCGTTGGAAGTAATGGAGTCTTTCATAT<br>TGTGCTCAAATCATTTCCCCGTCGCTTCCGGTCACGCTCAAGA<br>TGCGCAACAAGTGCGCTAACACATCTCCCACAATATCAGGCT<br>CTACAATGTCCGAATATATCAACAGTAATCTATAATGAGCGC<br>ACAGATATGCCGCTTACTGACTTTCGACGTTCTTTTCTTCATC<br>ATAATCATCATCGGTACTCTCAAGTTCAAACATAGCATTTGC

							GCGAGTCCGTCGTACAGTATCACTTTAGCACCCTGCAAAACA<br>AAAACAGAATCTGCCCCTTCCGGTAGACGCACTATGTTAAGA<br>GTTCCCGAAATTGGAATCGATGCAAAACTTTGAGGGTAGAC<br>AGAATATAGTATATAGACAAAATATGTCCAGATCGAAATAG<br>AGTTTTAGGTCCAGATCAAGCTTTCAAGTTGTGAGTCTAGCA<br>TCGTTCTATGGTGGGAAATGTGTTAAAAATATTTTTTAAATGC<br>TGCACCCTTTACTGTGTTGTTTACATGCAGTGTATATATTCAG<br>CTATATACATGCAGTGTATATATATTGTTTATATTCAGCTACT<br>CTTCTCGGCTACAGCGGCAGATAATTCAATTAGCCATCTCGC<br>AAGAAAGGAAATATGACTGTGTTCCTGACTAAAGTACACCT<br>GGCATGTATGCTTGTACCTAAAAGGTCCGAATTCGAGAATTT<br>TCTCGAAAGATAATTAAGGAGCGTCAGGGAAATTGTTGGAG<br>GTCAGTGATGAAAGGGCGACGATTGTTTTTCTGACGTCATAG<br>GTCTGGGTTTTTTTTTTTACAACTTCCGTGTCGAAATTGCACTA<br>TTTCCTGCAGAGATCAGAGCTATATATTAACAGCGTTTCAGG<br>GTATTACTGTGCTATGAATATATTCCAGATTTTATGCGTGTTTT<br>AACGTATATCACTTGCAAAGATGTATTTTTATTGTCTTAAAAT<br>ACTACCATGATGTGGTGTACCTCGGAAAAATAAACTTACTTT<br>TACATATATAAGAGTGTTTATTGCTGTAGCAGGAAAGTTTAC<br>CTTCACTTTATTTAATGCTATATAATTGTACCTTACAAAATTTT<br>GTAGCTTATAATAAAGTGAATAATCTTAATATTTCAACGATA<br>TTTGGAATGCTGTCAGCACAATTCAAACAACTTTCAGTGAAT<br>TATAGTGATTTCTCTTTAACAAGAATGCGATTAAAATCCAAA<br>ATAAGGTCGGAAAAATTCACAGTTTTCTTCGCTAGATTTCAA<br>GTAATTTGTACGGTTTTCAGATAGTTCAAAAACTTTGACGTAT<br>GGATCTGACTTAGAGAACTTCCGCCACGTTTCACCGGGTTCC<br>CCTCTCTTAGCAAAATTCGTCCACTCTTTCACCATCTCAAAGC<br>TTAGTTCTTCTTCCTTTCGGGTGTAGTTGGACAGAGGAGTTAG

							AGGACGTCCAAAGATAAATTGCATATCGTCACAATGCATAA<br>CCCCCATCCACTCTGCGTACGGAGTACTCGATGGTCTGTGAT<br>TGAAATAGTATCGGTAGATTTTATTATTTTTTTCTGCAACTTTC<br>TCAGCAAAATATTTGCTTGGGCACATAACCATGAAATCCCCA<br>AGAGAGTGATAAACTTGACTTCGTATTGTAGCATAATCGTCG<br>TCTTTAACGCCACTGAGGTAATGCTGGATTATTCCTTCCGGAT<br>CTGGAAATATTGTCCCGAAATAATCTCTCATGAGAGACTCCC<br>CTAACGTCTTATTAATGCGAGGATTTAATTCACCAAAAGGGC<br>CTGTGATCTGTGTAAAAGTGTTAACAAATGGGAATGATCCCT<br>CGTCTTGAGTAACTCCTATCATAGAATCAACACCGTTAAAGT<br>TTCCTTCTCTGATAGCTTTTTGTGGGTCTACCGGTAGAAAATC<br>ATCCCCACTGATGGTAAAGAAAGTTATCACTGGGAAGACGG<br>GAACTAAGGAAAACGTAGTTCTCACCATATCTTTTGGAGCTT<br>TACTTCTTAGGCATTCGATCACATCATCAGGATTTTTCTTCAG<br>CGTTATGTTTTCATTTGCACATTCCATCTTCTCTGCGAGCTTTT<br>GTCCTAATTTAAGATTCTCAGCCCTATGGTCATTCAGAAAAT<br>ATGCTGGACAACCACTTTGCATTATTGCTCTCTGGAAGAGAC<br>CTTTCGAAAGAGGAGAAACACACAGCAAACCTGTAGCTACG<br>CCGCCGGCGCTGTTGCCGAACACCGTAATTCTTTTATTGTCTC<br>CGCCGAATGCAGCAATGTTTTCGTTCGTCCATTTAAGGGCTTC<br>CAAAATGTCATACAAGCCGACGTTTCCAGGAGCTTCTTCGGT<br>TTCAGATGTGAAGAATCCGAGCACACTTAACCGGTAGTTTAT<br>CGCAACAACTATTACATCTCCAATTGCAGTTAGAACACGTCC<br>ATCATATAGAGGCATCCTACCTGAACCGGTTGCAAAGCCGCC<br>TGAGAACACCCAAAACATGACGGCTTTTTTCTCGCTACTATT<br>GGAAATTTCTGGTGCCCATATATTCAAATACAAGCAGTCTTC<br>GCTTTGACCCTGAACTTTATCTTCCCACGGATAGTTGTCCGGA<br>GAGTACTGGTAACAAGCAGGCGGTAAGCTGTTTGCTATTACA

							GGAGAGGTCCAAGGTTTGACAGGAACTGTTCGCTTAAAGCGT<br>AAATTCCCCACTGGAGGCTCAGCAAACGGAATTCCGAGAAA<br>GACATTTAGATTTATGTCGTCATAAGTCATAACAGAACCAGT<br>CACTTCACCTGTAGGAGTATTAACTGTAACGCTGGATGCTAA<br>GATAGTTCCTGGTAGCAGTAAAAATAGTAGATGCAAGCTCTT<br>ATGAACGTTTTTCTGGAGCTTTCCGCACATAGCGGCTCCTAA<br>ACCTTGGGCTCCAGCAATAACGTTAGACGAGGGAGACGACA<br>TGATGCTGCTGCCTAGATCTCCAGTGTGCTGTGTCGTACACA<br>AATGCGCCTATTTCCTGTTTGTAAAGCTCCTTCGTACACCAGT<br>ATCGTCAACAGCTATTTTTACATTAGTTATTCCGTTTCTGTCA<br>GCAATCTTGCATCGCACAAATCTGCTAGTGTAGGTCTTCATC<br>CGAATTATGCCCAAACTGGTTGTAGTTTTACTGTGGATAAGA<br>AGCTTTCTTAACCTAATTTGTTCTCCACAGGCT

PhdEnzCho 34	W4VSJ0	2,02E-65	35,676	PF00135	43419,31	SLYVTSRTRNIHIENFVLGLYDILEALKWTNENIAAFGGDNKRITVFGNSAGGVATGLLCVSPLSKGLFQRAIMQSGCPAYFLNDHRAENLKLGQKLAEKMECANENITLKKNPDDVIECLRSKAPKDMVRTTFSLVPVFPVITFFTISGDDFLPVDPQKAIREGNFNGVDSMIGVTQDEGSFPFVNTFTQITGPFGELNPRINKTLGESLMRDYFGTIFPDPEGIIQHYLSGVKDDDYATIRSQVYHSLGDFMVMCPSKYFAEKVAEKNNKIYRYYFNHRPSSTPYAEWMGVMHCDDMQFIFGRPLTPLSNYTRKEEELSFEMVKEWTNFAKRGEPGETWRKFSKSDPYVKVFELSENRTNYLKSSEENCEFFRPYFGF*	CGATAAACCTTTTGCAAGCAAGTTTCTCATGATCTGGCAATATTCTAGAAAGTGCAAACATGATTATGTAATATGAAAAATGCTGTATATGAAACTCGAGGTATTTTTGATTTATTCTTTATACTTTCATATTACATATTCATCTTTACACTTTCATAACTATGCTTTCATGAGTCTGCGTACATCAACATAACACATTAGAAAACTTTAATATGCAATTCATATTCTCAATTTAAGCTAGGATCCCCTTTATTTTGCAATTTAACTCAAAACTTCAAAGGAATAAACCTAAAATTATGCTTTTTTAACTACGGGAAGCTTCAAATTTTACGATAAACCTTTTGCAAGCAAGTTTCTCATGATCTGGCAATATTCTAGAAAGTGCAAACATGATTATGTAATATGAAAAATGCTGTATATGAAACTCAAGGTATTTTTGATTTATTTTTTATACTTTCATATTACATAGTCATCTTTACACTTTCGTAATTCTGCGTATATCAACTGAAAACATTAGCAAACTTTAATATGCAATAAATAGCTATTAGGATAACCTTTTCTTTTAATAACGTCACTATGCCTCTTTTAAATAGAATTGACTAGCTTCGATGTTTACTCGGAGCTTATATTATTCCATTCTTCTCTCAATATGGCTTTCAACATAGCCTTTGAAGCTATATTGTGTTGGCAGATTCTGCGTTCCAGCAAATCTCAAAAACACTTTATCGGGTAAGTCAGGCGATTCTGGAGTTGTTTATGTGTAGGAGTAGATAACTATTTTAGCACGTCATATAAGAACTAGATATTCATAACTTGAACAGTGTGCTTGGGGACCTTGTCCAATTAGAAATAATAACTGTTGTCTATCTTAAGTACTCTGCATTAAATTTCGTTTTAAAATGTTTAAATAATCCATTTTATTCAATTGTGTCCTGAATAAAGTGTAGTTGTCCCACGCCTCAGTTTTAGATACACCCCCAAATAATAATACTTTAGCCACCGTGTTTTACAGTTGGCATTAAATATTCTTACTTAAGCTCTTTAACAGGTTTTCGGTACAAAAGCTTGAATCCTTTAATGTCAAATACGCAGAATCTGCTCTAATCGGAAAATATTACCAGTTGCAGAGCTCATTTTGCTTATTAATATATTCTTTTGCAACGTCAATGCTTAAATCTATTATCTCAGGAAATGTA

							AAACAGCTAAAAGGGATGTTACAACAGTTTCTCTTCGAGCCG<br>TCACATTTATCTTTTGTTCTTTCGCCTCTTGTGTTTTACCTATTC<br>CTCGACATATTTATGGCTATTTTTCAGTTCACAACTCTCTCGG<br>ATGGCGTAATCTTTTCTTGAAATTTCTTCGAATTTTCTCTCGTT<br>TTATATATATCAAAGCGGTAATGCGTTTTGCATTTTGTTCCGT<br>TTATAGTAACGTGCGCACCTAATACTTTCACCTGCTTCATGAA<br>GGACTGTATCAAATAGTCCGAAGTATGCTGCAGCTTTCCTGT<br>CTTACTGTGGATCAGAATCTTTTTTTGCAGCTTTATGGTATTCA<br>CTGCCTCAGTGACGAGACTTTGAAAATTTCAATCATGTTTTAA<br>AATTCCGCATATATCAACACGAAAATGGTGTCTCCAAGGATT<br>TGGAAAATTGAATTTCTACTCGCAAGTTACCTTGGTGTTGCA<br>ATTAAAATGAGGTCCATCACACAAGGATGGGATAAAGTCGA<br>ACACGTAAACTGTATAGCCTTCTACAAATTTGTCTTTCGAGA<br>AGTAAATACCTCTGTTTTGATTAAAGTTGATAAACAATTTGGT<br>GACAAGCGCGAATATATCTTTTAGAATCAAAATTCGGTTATA<br>AATGTTTGCTTGGCACTGAGTGACCATCTACTTAAACACTTA<br>AAAAGTTGACCATGATAATGATGAAAAAAAGGAAGAGGGC<br>AGGTTATGTTTCCTATTTACCGTGGCATTCTTCAGGGAGGTAA<br>TAATCACTCCTTTGGACATCAGTCACAAAAATACATTGTCTTT<br>CATAAAAGAAAATGTTCCAACTGAAACTGAGTATGCTTTATA<br>CAATACTCTGTCGATGGTATATTTCGAGAGACTTTTTTCGAGA<br>ACATTGACATGTCCCAAGGATACGCCGGGACATTCACTGTCC<br>TAATTAACAAGGAGGCATGCTCAGATACGATTTTATACTGGT<br>CTCATTTTGGGGGGGGAGACTAAACAAAAACTGTGTCTGTTG<br>TGGCTCATTTTGATTCGCAGATCATCAAATTTAATAAAAGTA<br>GATTTTGTTGAAAACATTCGCATCATCTCCAACGTTCGATTTT<br>TAGCTGTAAGTTCATTACGCTTCGTCAAACCTACATTTCTATC<br>GATTTCTTTCAACGGGTTCGTCGAATGTAATTTGCTGTCTTTGT

							AAAAGCATTCAACGGGGGAGTAAACTTTTCTTGACATTTTTTCCATATATCCAAATTCTTCAATATAGGTTCTGTAGGGATAAGTATTGCTATAGACGACGAGACGACTCGCTTCTTAAGTTGATTTCCACTCTGAAAAAAAGAGCGTGTAAAATTAAATTCACGGGGTCGACGAGTTCATTTTCAGGTAAAGCGGATCTATCCGACTTCATAATTTTGGCTTTTACGTGCAGTTAGGAAGACGATAAGTCAATGTAATCTTCACCATTGTTACATTCCACGGGTCCACCATCGCGTAGGTTGGACAGGGGAGGATATTCAATAAAATGTCCTCTTCGATTGCGGTCTCGCATAAGCGGGATCATGAACAAATGGAAATCAGATTTTGCGCAAGCAACACAGTATTGATAGATGCGTGTCGATATGCTTAACCGAAAATAACGCGTGATTCCATTTTCTTGGTTCTTGGTGAACTAATGCGTTATTCCAACGCTTTCTCTTTTAAATTATGACCAGAAGTAATAATGGACTTCATTTTTCAAGCTACTTTGTCCGTTAATTGGCTTCCAGCTTATTTCGAATAGCGTTTAACAGACATTTTTAGAACATTCAATATGCCCACACTGCTGTGAAATCATCGTTTGCCGACTACTTTTATGCCCAGTTTTAAAAGGGGAACTGTCACTCGATAAAAAGGACAAAAGAATCTGCCAAAACCATAAACTGTCTGAAATGTATCCCCTTTATAATAATTCAACTCGCCACGCTTTGCATAATCCTCGAAGGATTTTGGACAACATTGATGAGGCACATACATCACGATAGAAACTTTTGCCAAAAGTACAGTTTTACGTACGAGCGCTTGAGTTCAGATGACACAATTTCGCGTGTATGCATTCTTATAACGACATTCAATACTTTTCTATTCCGTTGGAAGTAATGGAGTCTTTCATATTGTGCTCAAATCATTTCCCCGTCGCTTCCGGTCACGCTCAAGATGCGCAACAAGTGCGCTAACACATCTCCCACAATATCAGGCTCTACAATGTCCGAATATACCAACAGTAATCTATAATGAGCACACAGATATGCCGCTTACTGACTTTCGACGTTCTTTTCTTCATCATAATCATCATCGGTACTCTCAAGTTCAAACATAGCATTTGCGCGAGTCCGTCGTACA

							GTATCACTTTAGCACCCTGCAAAACAAAAACAGAATCTGCC<br>CCTTCCGGTAGACGCACTATGTTAAGAGTTCCCGAAATTGGA<br>ATCGATGCAAAACTTTGAGGGTAGACAGAATATAGTATATA<br>GACAAAATATGTCCAGATCGAAATAGAGTTTTAGGTCCAGAT<br>CAAGCTTTCAAGTTGTGAGTCTAGCATCGTTCTATGGTGGGA<br>AATGTGTTAAAAATATTTTTTAAATGCTGCACCCTTTACTGTG<br>TTGTTTACATGCAGTGTATATATTCAGCTATATACATGCAGTG<br>TATATATATTGTTTATATTCAGCTACTCTTCTCGGCTACAGCG<br>GCAGATAATTCAATTAGCCATCTCGCAAGAAAGGAAATATG<br>ACTGTGTTCCTGACTAAAGTACACCTGGCATGTATGCTTGTAC<br>CTAAAAGGTCCGAATTCGAGAATTTTCTCGAAAGATAATTAA<br>GGAGCGTCAGGGAAATTGTTGGAGGTCAGTGATGAAAGGGC<br>GACGATTGTTTTTCTGACGTCATAGGTCTGGGTTTTTTTTTTA<br>CAACTTCCGTGTCGAAATTGCACTATTTCCTGCAGAGATCAG<br>AGCTATATATTAACAGCGTTTCAGGGTATTACTGTGCTATGA<br>ATATATTCCAGATTTTATGCGTGTTTTAACGTATATCACTTGC<br>AAAGATGTATTTTTATTGTCTTAAAATACTACCATGATGTGGT<br>GTACCTCGGAAAAATAAACTTACTTTTACATATATAAGAGTG<br>TTTATTGCTGTAGCAGGAAAGTTTACCTTCACTTTATTTAATG<br>CTATATAATTGTACCTTACAAAATTTTGTAGCTTATAATAAAG<br>TGAATAATCTTAATATTTCAACGATATTTGGAATGCTGTCAGC<br>ACAATTCAAACAACTTTCAGTGAATTATAGTGATTTCTCTTTA<br>ACAAGAATGCGATTAAAATCCAAAATAAGGTCGGAAAAATT<br>CACAGTTTTCTTCGCTAGATTTCAAGTAATTTGTACGGTTTTC<br>AGATAGTTCAAAAACTTTGACGTATGGATCTGACTTAGAGAA<br>CTTCCGCCACGTTTCACCGGGTTCCCCTCTCTTAGCAAAATTC<br>GTCCACTCTTTCACCATCTCAAAGCTTAGTTCTTCTTCCTTTCG<br>GGTGTAGTTGGACAGAGGAGTTAGAGGACGTCCAAAGATAA

							ATTGCATATCGTCACAATGCATAACCCCCATCCACTCTGCGT<br>ACGGAGTACTCGATGGTCTGTGATTGAAATAGTATCGGTAGA<br>TTTTATTATTTTTTTCTGCAACTTTCTCAGCAAAATATTTGCTT<br>GGGCACATAACCATGAAATCCCCAAGAGAGTGATAAACTTG<br>ACTTCGTATTGTAGCATAATCGTCGTCTTTAACGCCACTGAGG<br>TAATGCTGGATTATTCCTTCCGGATCTGGAAATATTGTCCCGA<br>AATAATCTCTCATGAGAGACTCCCCTAACGTCTTATTAATGC<br>GAGGATTTAATTCACCAAAAGGGCCTGTGATCTGTGTAAAAG<br>TGTTAACAAATGGGAATGATCCCTCGTCTTGAGTAACTCCTA<br>TCATAGAATCAACACCGTTAAAGTTTCCTTCTCTGATAGCTTT<br>TTGTGGGTCTACCGGTAGAAAATCATCCCCACTGATGGTAAA<br>GAAAGTTATCACTGGGAAGACGGGAACTAAGGAAAACGTA<br>GTTCTCACCATATCTTTTGGAGCTTTACTTCTTAGGCATTCGAT<br>CACATCATCAGGATTTTTCTTCAGCGTTATGTTTTCATTTGCAC<br>ATTCCATCTTCTCTGCGAGCTTTTGTCCTAATTTAAGATTCTCA<br>GCCCTATGGTCATTCAGAAAATATGCTGGACAACCACTTTGC<br>ATTATTGCTCTCTGGAAGAGACCTTTCGAAAGAGGAGAAAC<br>ACACAGCAAACCTGTAGCTACGCCGCCGGCGCTGTTGCCGA<br>ACACCGTAATTCTTTTATTGTCTCCGCCGAATGCAGCAATGTT<br>TTCGTTCGTCCATTTAAGGGCTTCCAAAATGTCATACAAGCCT<br>AGAACGAAATTCTCGATATGAATATTTCTTGTACGAGAAGTT<br>ACATACAAGGATTATTCTTGATAAAATATCAGTCATTTATTTT<br>TTTAGATTTATCCATATAGGTTCTAAATAGAATATTTAATATT<br>TAATATAAATATTAATGCCTAACAGCACAGGGCAGAA

PhdEnzCho 35	W4VSJ0	1,20E-109	37,618	PF00135	63354,11	LKNCPQNKLSDISFL NNAISILNSVFQSLH LLFLLLPGTILASSVT VNTPTGEVTGSVMT YDDINLNVFLGIPFA EPPVGNLRFKRTVPV KPWTSPVIANSLPPA CYQYSPDNYPWEDK VQGQSEDCLYLNIW APEISNSSEKKAVMF WVFSGGFATGSGRM PLYDGRVLTAIGDVI VVAINYRLSVLGFFT SETEEAPGNVGLYDI LEALKWTNENIAAF GGDNKRITVFGNSA GGVATGLLCVSPLSK GLFQRAIMQSGCPA YFLNDHRAENLKLG QKLAEKMECANENI TLKKNPDDVIECLRS KAPKDMVRTTFSLV PVFPVITFFTISGDDF LPVDPQKAIREGNF NGVDSMIGVTQDEG SFPFVNTFTQITGPFG ELNPRINKTLGESLM RDYFGTIFPDPEGIIQ	CGATAAACCTTTTGCAAGCAAGTTTCTCATGATCTGGCAATA TTCTAGAAAGTGCAAACATGATTATGTAATATGAAAAATGCT GTATATGAAACTCGAGGTATTTTTGATTTATTCTTTATACTTTC ATATTACATATTCATCTTTACACTTTCATAACTATGCTTTCATG AGTCTGCGTACATCAACATAACACATTAGAAAACTTTAATAT GCAATTCATATTCTCAATTTAAGCTAGGATCCCCTTTATTTTG CAATTTAACTCAAAACTTCAAAGGAATAAACCTAAAATTATG CTTTTTTAACTACGGGAAGCTTCAAATTTTACGATAAACCTTT TGCAAGCAAGTTTCTCATGATCTGGCAATATTCTAGAAAGTG CAAACATGATTATGTAATATGAAAAATGCTGTATATGAAACT CAAGGTATTTTTGATTTATTTTTTATACTTTCATATTACATAGT CATCTTTACACTTTCGTAATGCTGCGTATATCAACATTAGCAA ACTTTAATATGCAATAAATAGCTATTAGGATAACCTTTTCTTT TAATAACGTCACTATGCCTCTTTTAAATAGAATTGACTAGCTT CGATGTTTACTCGGAGCTTATATTATTCCATTCTTCTCTCAATA TGGCTTTCAACATAGCCTTTGAAGCTATATTGTGTTGGCAGAT TCTGCGTTCCAGCAAATCTCAAAAACACTTTATCGGGTAAGT CAGGCGATTCTGGAGTTGTTTATGTGTAGGAGTAGATAACTA TTTTAGCACGTCATATAAGAACTAGATATTCATAACTTGAAC AGTGTGCTTGGGGACCTTGTCCAATTAGAAATAATAACTGTT GTCTATCTTAAGTACTCTGCATTAAATTTCGTTTTAAAATGTTT AAATAATCCATTTTATTCAATTGTGTCCTGAATAAAGTGTAGT TGTCCCACGCCTCAGTTTTAGATACACCCCCAAATAATAATA CTTTAGCCACCGTGTTTTACAGTTGGCATTAAATATTCTTACT TAAGCTCTTTAACAGGTTTTCGGTACAAAAGCTTGAATCCTTT AATGTCAAATACGCAGAATCTGCTCTAATCGGAAAATATTAC CAGTTGCAGAGCTCATTTTGCTTATTAATATATTCTTTTGCAA CGTCAATGCTTAAATCTATTATCTCAGGAAATGTAAAACAGC

						HYLSGVKDDDYATIRSQVYHSLGDFMVMCPSKYFAEKVAEKNNKIYRYYFNHRPSSTPYAEWMGVMHCDDMQFIFGRPLTPLSNYTRKEEELSFEMVKEWTNFAKRGEPGETWRKFSKSDPYVKVFELSENRTNYLKSSEENCEFFRPYFGF*	TAAAAGGGATGTTACAACAGTTTCTCTTCGAGCCGTCACATTTATCTTTTGTTCTTTCGCCTCTTGTGTTTTACCTATTCCTCGACATATTTATGGCTATTTTTCAGTTCACAACTCTCTCGGATGGCGTAATCTTTTCTTGAAATTTCTTCGAATTTTCTCTCGTTTTATATATATCAAAGCGGTAATGCGTTTTGCATTTTGTTCCGTTTATAGTAACGTGCGCACCTAATACTTTCACCTGCTTCATGAAGGACTGTATCAAATAGTCCGAAGTATGCTGCAGCTTTCCTGTCTTACTGTGGATCAGAATCTTTTTTTGCAGCTTTATGGTATTCACTGCCTCAGTGACGAGACTTTGAAAATTTCAATCATGTTTTAAAATTCCGCATATATCAACACGAAAATGGTGTCTCCAAGGATTTGGAAAATTGAATTTCTACTCGCAAGTTACCTTGGTGTTGCAATTAAAATGAGGTCCATCACACAAGGATGGGATAAAGTCGAACACGTAAACTGTATAGCCTTCTACAAATTTGTCTTTCGAGAAGTAAATACCTCTGTTTTGATTAAAGTTGATAAACAATTTGGTGACAAGCGCGAATATATCTTTTAGAATCAAAATTCGGTTATAAATGTTTGCTTGGCACTGAGTGACCATCTACTTAAACACTTAAAAAGTTGACCATGATAATGATGAAAAAAAGGAAGAGGGCAGGTTATGTTTCCTATTTACCGTGGCATTCTTCAGGGAGGTAATAATCACTCCTTTGGACATCAGTCACAAAAATACATTGTCTTTCATAAAAGAAAATGTTCCAACTGAAACTGAGTATGCTTTATACAATACTCTGTCGATGGTATATTTCGAGAGACTTTTTTCGAGAACATTGACATGTCCCAAGGATACGCCGGGACATTCACTGTCCTAATTAACAAGGAGGCATGCTCAGATACGATTTTATACTGGTCTCATTTTGGGGGGGGAGACTAAACAAAAACTGTGTCTGTTGTGGCTCATTTTGATTCGCAGATCATCAAATTTAATAAAAGTAGATTTTGTTGAAAACATTCGCATCATCTCCAACGTTCGATTTTTAGCTGTAAGTTCATTACGCTTCGTCAAACCTACATTTCTATCGATTTCTTTCAACGGGTTCGTCGAATGTAATTTGCTGTCTTTGTAAAAG

							CATTCAACGGGGGAGTAAACTTTTCTTGACATTTTTTCCATAT ATCCAAATTCTTCAATATAGGTTCTGTAGGGATAAGTATTGCT ATAGACGACGAGACGACTCGCTTCTTAAGTTGATTTCCACTC TGAAAAAAAGAGCGTGTAAAATTAAATTCACGGGGTCGACG AGTTCATTTTCAGGTAAAGCGGATCTATCCGACTTCATAATTT TGGCTTTTACGTGCAGTTAGGAAGACGATAAGTCAATGTAAT CTTCACCATTGTTACATTCCACGGGTCCACCATCGCGTAGGTT GGACAGGGGAGGATATTCAATAAAATGTCCTCTTCGATTGCG GTCTCGCATAAGCGGGATCATGAACAAATGGAAATCAGATT TTGCGCAAGCAACACAGTATTGATAGATGCGTGTCGATATGC TTAACCGAAAATAACGCGTGATTCCATTTTCTTGGTTCTTGGT GAACTAATGCGTTATTCCAACGCTTTCTCTTTTAAATTATGAC CAGAAGTAATAATGGACTTCATTTTTCAAGCTACTTTGTCCGT TAATTGGCTTCCAGCTTATTTCGAATAGCGTTTAACAGACATT TTTAGAACATTCAATATGCCCACACTGCTGTGAAATCATCGT TTGCCGACTACTTTTATGCCCAGTTTTAAAAGGGGAACTGTC ACTCGATAAAAAGGACAAAAGAATCTGCCAAAACCATAAA CTGTCTGAAATGTATCCCCTTTATAATAATTCAACTCGCCACG CTTTGCATAATCCTCGAAGGATTTTGGACAACATTGATGAGG CACATACATCACGATAGAAACTTTTGCCAAAAGTACAGTTTT ACGTACGAGCGCTTGAGTTCAGATGACACAATTTCGCGTGTA TGCATTCTTATAACGACATTCAATACTTTTCTATTCCGTTGGA AGTAATGGAGTCTTTCATATTGTGCTCAAATCATTTCCCCGTC GCTTCCGGTCACGCTCAAGATGCGCAACAAGTGCGCTAACA CATCTCCCACAATATCAGGCTCTACAATGTCCGAATATATCA ACAGTAATCTATAATGAGCGCACAGATATGCCGCTTACTGAC TTTCGACGTTCTTTTCTTCATCATAATCATCATCGGTACTCTCA AGTTCAAACATAGCATTTGCGCGAGTCCGTCGTACAGTATCA

							CTTTAGCACCCTGCAAAACAAAAACAGAATCTGCCCCTTCCG<br>GTAGACGCACTATGTTAAGAGTTCCCGAAATTGGAATCGATG<br>CAAAACTTTGAGGGTAGACAGAATATAGTATATAGACAAAA<br>TATGTCCAGATCGAAATAGAGTTTTAGGTCCAGATCAAGCTT<br>TCAAGTTGTGAGTCTAGCATCGTTCTATGGTGGGAAATGTGTT<br>AAAAATATTTTTTAAATGCTGCACCCTTTACTGTGTTGTTTAC<br>ATGCAGTGTATATATTCAGCTATATACATGCAGTGTATATAT<br>ATTGTTTATATTCAGCTACTCTTCTCGGCTACAGCGGCAGATA<br>ATTCAATTAGCCATCTCGCAAGAAAGGAAATATGACTGTGTT<br>CCTGACTAAAGTACACCTGGCATGTATGCTTGTACCTAAAAG<br>GTCCGAATTCGAGAATTTTCTCGAAAGATAATTAAGGAGCGT<br>CAGGGAAATTGTTGGAGGTCAGTGATGAAAGGGCGACGATT<br>GTTTTTCTGACGTCATAGGTCTGGGTTTTTTTTTTACAACTTC<br>CGTGTCGAAATTGCACTATTTCCTGCAGAGATCAGAGCTATA<br>TATTAACAGCGTTTCAGGGTATTACTGTGCTATGAATATATTC<br>CAGATTTTATGCGTGTTTTAACGTATATCACTTGCAAAGATGT<br>ATTTTTATTGTCTTAAAATACTACCATGATGTGGTGTACCTCG<br>GAAAAATAAACTTACTTTTACATATATAAGAGTGTTTATTGCT<br>GTAGCAGGAAAGTTTACCTTCACTTTATTTAATGCTATATAAT<br>TGTACCTTACAAAATTTTGTAGCTTATAATAAAGTGAATAAT<br>CTTAATATTTCAACGATATTTGGAATGCTGTCAGCACAATTCA<br>AACAACTTTCAGTGAATTATAGTGATTTCTCTTTAACAAGAAT<br>GCGATTAAAATCCAAAATAAGGTCGGAAAAATTCACAGTTTT<br>CTTCGCTAGATTTCAAGTAATTTGTACGGTTTTCAGATAGTTC<br>AAAAACTTTGACGTATGGATCTGACTTAGAGAACTTCCGCCA<br>CGTTTCACCGGGTTCCCCTCTCTTAGCAAAATTCGTCCACTCT<br>TTCACCATCTCAAAGCTTAGTTCTTCTTCCTTTCGGGTGTAGTT<br>GGACAGAGGAGTTAGAGGACGTCCAAAGATAAATTGCATAT

							CGTCACAATGCATAACCCCCATCCACTCTGCGTACGGAGTAC<br>TCGATGGTCTGTGATTGAAATAGTATCGGTAGATTTTATTATT<br>TTTTTCTGCAACTTTCTCAGCAAAATATTTGCTTGGGCACATA<br>ACCATGAAATCCCCAAGAGAGTGATAAACTTGACTTCGTATT<br>GTAGCATAATCGTCGTCTTTAACGCCACTGAGGTAATGCTGG<br>ATTATTCCTTCCGGATCTGGAAATATTGTCCCGAAATAATCTC<br>TCATGAGAGACTCCCCTAACGTCTTATTAATGCGAGGATTTA<br>ATTCACCAAAAGGGCCTGTGATCTGTGTAAAAGTGTTAACAA<br>ATGGGAATGATCCCTCGTCTTGAGTAACTCCTATCATAGAAT<br>CAACACCGTTAAAGTTTCCTTCTCTGATAGCTTTTTGTGGGTC<br>TACCGGTAGAAAATCATCCCCACTGATGGTAAAGAAAGTTA<br>TCACTGGGAAGACGGGAACTAAGGAAAACGTAGTTCTCACC<br>ATATCTTTTGGAGCTTTACTTCTTAGGCATTCGATCACATCAT<br>CAGGATTTTTCTTCAGCGTTATGTTTTCATTTGCACATTCCATC<br>TTCTCTGCGAGCTTTTGTCCTAATTTAAGATTCTCAGCCCTAT<br>GGTCATTCAGAAAATATGCTGGACAACCACTTTGCATTATTG<br>CTCTCTGGAAGAGACCTTTCGAAAGAGGAGAAACACACAGC<br>AAACCTGTAGCTACGCCGCCGGCGCTGTTGCCGAACACCGTA<br>ATTCTTTTATTGTCTCCGCCGAATGCAGCAATGTTTTCGTTCGT<br>CCATTTAAGGGCTTCCAAAATGTCATACAAGCCGACGTTTCC<br>AGGAGCTTCTTCGGTTTCAGATGTGAAGAATCCGAGCACACT<br>TAACCGGTAGTTTATCGCAACAACTATTACATCTCCAATTGC<br>AGTTAGAACACGTCCATCATATAGAGGCATCCTACCTGAACC<br>GGTTGCAAAGCCGCCTGAGAACACCCAAAACATGACGGCTT<br>TTTTCTCGCTACTATTGGAAATTTCTGGTGCCCATATATTCAA<br>ATACAAGCAGTCTTCGCTTTGACCCTGAACTTTATCTTCCCAC<br>GGATAGTTGTCCGGAGAGTACTGGTAACAAGCAGGCGGTAA<br>GCTGTTTGCTATTACAGGAGAGGTCCAAGGTTTGACAGGAAC

							TGTTCGCTTAAAGCGTAAATTCCCCACTGGAGGCTCAGCAAACGGAATTCCGAGAAAGACATTTAGATTTATGTCGTCATAAGTCATAACAGAACCAGTCACTTCACCTGTAGGAGTATTAACTGTAACGCTGGATGCTAAGATAGTTCCTGGTAGCAGTAAAAATAGTAGATGCAAGCTCTGAAAAACAGAATTTAAGATACTTATTGCATTATTTAAAAAAGAAATATCAGATAACTTATTTTGAGGACAATTTTTCAACTATTATTTAATATCCCTCAGAACTGTTACTATCAGCGACATAACCACAATTTTAACAATTCCCTCCTTCTCTGAAGCAAATTTCCATTTTCCAGAAAGTGAGCCTCTTTTGTACTAAAATGGATTTTGTTACATCTCTAGTTCTCAGAGTGCACTTACCAAGAGATCTTTTGGGAGAGATTATTACTTCCCTCATCTTAATTCTTTGTTTATGATAGAGTTTTATCTGTATGTAATTTCATTTTAATGACTAAAAGTTTTGGATAATTGTAAGTCCAATAACGCAACGCCTTATTTCAGAAATGCTTACAAAAAAAACTGAGGTAGTCACTGTTGCACTTGCACAACCTGTTGACAGTTGCTCATTGGAATTCCATGGTGGCAATGGCATCTTCCTCAGTCTTCAATTATTCTTTTTGTAGATTACCAGGTTTTGATTAATTACATTTACGTAAGTAGGAAATTACATATTGTGAAAAATGATTTTAGATATTTTTAATAGCAGATAGACGGAATATTTCCGATGATATAAATAACTGAATATTTACGACACGCTTTTGCACTACAAGCGTTAAAGTTGTGGCAGTAATAAGATTGCAACAACTTTTGAGTAGTTTTACAAAGCGGAATCTAAAAAGTTAAGGAATT

PhdEnzCho 36	W4VSJ0	2,02E-65	35,676	PF00135	43419,31	SLYVTSRTRNIHIENF<br>VLGLYDILEALKWT<br>NENIAAFGGDNKRI<br>TVFGNSAGGVATGL<br>LCVSPLSKGLFQRAI<br>MQSGCPAYFLNDHR<br>AENLKLGQKLAEK<br>MECANENITLKKNP<br>DDVIECLRSKAPKD<br>MVRTTFSLVPVFPVI<br>TFFTISGDDFLPVDP<br>QKAIREGNFNGVDS<br>MIGVTQDEGSFPFV<br>NTFTQITGPFGELNP<br>RINKTLGESLMRDYF<br>GTIFPDPEGIIQHYLS<br>GVKDDDYATIRSQV<br>YHSLGDFMVMCPSK<br>YFAEKVAEKNNKIY<br>RYYFNHRPSSTPYAE<br>WMGVMHCDDMQF<br>IFGRPLTPLSNYTRKE<br>EELSFEMVKEWTNF<br>AKRGEPGETWRKFS<br>KSDPYVKVFELSENR<br>TNYLKSSEENCEFFR<br>PYFGF*	CGATAAACCTTTTGCAAGCAAGTTTCTCATGATCTGGCAATA<br>TTCTAGAAAGTGCAAACATGATTATGTAATATGAAAAATGCT<br>GTATATGAAACTCGAGGTATTTTTGATTTATTCTTTATACTTTC<br>ATATTACATATTCATCTTTACACTTTCATAACTATGCTTTCATG<br>AGTCTGCGTACATCAACATAACACATTAGAAAACTTTAATAT<br>CAATTCATATTCTCAATTTCATATTCTCAATTTAAGCTAGGAT<br>CCCCTTTATTTTGCAATTTAACTCAAAACTTCAAAGGAATAA<br>ACCTAAAATTATGCTTTTTTAACTACGGGAAGCTTCAAATTTT<br>ACGATAAACCTTTTGCAAGCAAGTTTCTCATGATCTGGCAAT<br>ATTCTAGAAAGTGCAAACATGATTATGTAATATGAAAAATGC<br>TGTATATGAAACTCAAGGTATTTTTGATTTATTTTTTATACTTT<br>CATATTACATAGTCATCTTTACACTTTCGTAATTCTGCGTATA<br>TCAACTGAAAACATTAGCAAACTTTAATATGCAATAAATAGC<br>TATTAGGATAACCTTTTCTTTTAATAACGTCACTATGCCTCTTT<br>TAAATAGAATTGACTAGCTTCGATGTTTACTCGGAGCTTATAT<br>TATTCCATTCTTCTCTCAATATGGCTTTCAACATAGCCTTTGA<br>AGCTATATTGTGTTGGCAGATTCTGCGTTCCAGCAAATCTCA<br>AAAACACTTTATCGGGTAAGTCAGGCGATTCTGGAGTTGTTT<br>ATGTGTAGGAGTAGATAACTATTTTAGCACGTCATATAAGAA<br>CTAGATATTCATAACTTGAACAGTGTGCTTGGGGACCTTGTC<br>CAATTAGAAATAATAACTGTTGTCTATCTTAAGTACTCTGCAT<br>TAAATTTCGTTTTAAAATGTTTAAATAATCCATTTTATTCAATT<br>GTGTCCTGAATAAAGTGTAGTTGTCCCACGCCTCAGTTTTAG<br>ATACACCCCCAAATAATAATACTTTAGCCACCGTGTTTTACA<br>GTTGGCATTAAATATTCTTACTTAAGCTCTTTAACAGGTTTTC<br>GGTACAAAAGCTTGAATCCTTTAATGTCAAATACGCAGAATC<br>TGCTCTAATCGGAAAATATTACCAGTTGCAGAGCTCATTTTG<br>CTTATTAATATATTCTTTTGCAACGTCAATGCTTAAATCTATT

							ATCTCAGGAAATGTAAAACAGCTAAAAGGGATGTTACAACA<br>GTTTCTCTTCGAGCCGTCACATTTATCTTTTGTTCTTTCGCCTC<br>TTGTGTTTTACCTATTCCTCGACATATTTATGGCTATTTTTCAG<br>TTCACAACTCTCTCGGATGGCGTAATCTTTTCTTGAAATTTCTT<br>CGAATTTTCTCTCGTTTTATATATATCAAAGCGGTAATGCGTT<br>TTGCATTTTGTTCCGTTTATAGTAACGTGCGCACCTAATACTT<br>TCACCTGCTTCATGAAGGACTGTATCAAATAGTCCGAAGTAT<br>GCTGCAGCTTTCCTGTCTTACTGTGGATCAGAATCTTTTTTTGC<br>AGCTTTATGGTATTCACTGCCTCAGTGACGAGACTTTGAAAA<br>TTTCAATCATGTTTTAAAATTCCGCATATATCAACACGAAAA<br>TGGTGTCTCCAAGGATTTGGAAAATTGAATTTCTACTCGCAA<br>GTTACCTTGGTGTTGCAATTAAAATGAGGTCCATCACACAAG<br>GATGGGATAAAGTCGAACACGTAAACTGTATAGCCTTCTACA<br>AATTTGTCTTTCGAGAAGTAAATACCTCTGTTTTGATTAAAGT<br>TGATAAACAATTTGGTGACAAGCGCGAATATATCTTTTAGAA<br>TCAAAATTCGGTTATAAATGTTTGCTTGGCACTGAGTGACCA<br>TCTACTTAAACACTTAAAAAGTTGACCATGATAATGATGAAA<br>AAAAGGAAGAGGGCAGGTTATGTTTCCTATTTACCGTGGCAT<br>TCTTCAGGGAGGTAATAATCACTCCTTTGGACATCAGTCACA<br>AAAATACATTGTCTTTCATAAAAGAAAATGTTCCAACTGAAA<br>CTGAGTATGCTTTATACAATACTCTGTCGATGGTATATTTCGA<br>GAGACTTTTTTCGAGAACATTGACATGTCCCAAGGATACGCC<br>GGGACATTCACTGTCCTAATTAACAAGGAGGCATGCTCAGAT<br>ACGATTTTATACTGGTCTCATTTTGGGGGGGGAGACTAAACA<br>AAAACTGTGTCTGTTGTGGCTCATTTTGATTCGCAGATCATCA<br>AATTTAATAAAAGTAGATTTTGTTGAAAACATTCGCATCATC<br>TCCAACGTTCGATTTTTAGCTGTAAGTTCATTACGCTTCGTCA<br>AACCTACATTTCTATCGATTTCTTTCAACGGGTTCGTCGAATG

							TAATTTGCTGTCTTTGTAAAAGCATTCAACGGGGGAGTAAAC<br>TTTTCTTGACATTTTTTCCATATATCCAAATTCTTCAATATAGG<br>TTCTGTAGGGATAAGTATTGCTATAGACGACGAGACGACTCG<br>CTTCTTAAGTTGATTTCCACTCTGAAAAAAAGAGCGTGTAAA<br>ATTAAATTCACGGGGTCGACGAGTTCATTTTCAGGTAAAGCG<br>GATCTATCCGACTTCATAATTTTGGCTTTTACGTGCAGTTAGG<br>AAGACGATAAGTCAATGTAATCTTCACCATTGTTACATTCCA<br>CGGGTCCACCATCGCGTAGGTTGGACAGGGGAGGATATTCA<br>ATAAAATGTCCTCTTCGATTGCGGTCTCGCATAAGCGGGATC<br>ATGAACAAATGGAAATCAGATTTTGCGCAAGCAACACAGTA<br>TTGATAGATGCGTGTCGATATGCTTAACCGAAAATAACGCGT<br>GATTCCATTTTCTTGGTTCTTGGTGAACTAATGCGTTATTCCA<br>ACGCTTTCTCTTTTAAATTATGACCAGAAGTAATAATGGACTT<br>CATTTTTCAAGCTACTTTGTCCGTTAATTGGCTTCCAGCTTATT<br>TCGAATAGCGTTTAACAGACATTTTTAGAACATTCAATATGC<br>CCACACTGCTGTGAAATCATCGTTTGCCGACTACTTTTATGCC<br>CAGTTTTAAAAGGGGAACTGTCACTCGATAAAAAGGACAAA<br>AGAATCTGCCAAAACCATAAACTGTCTGAAATGTATCCCCTT<br>TATAATAATTCAACTCGCCACGCTTTGCATAATCCTCGAAGG<br>ATTTTGGACAACATTGATGAGGCACATACATCACGATAGAA<br>ACTTTTGCCAAAAGTACAGTTTTACGTACGAGCGCTTGAGTT<br>CAGATGACACAATTTCGCGTGTATGCATTCTTATAACGACAT<br>TCAATACTTTTCTATTCCGTTGGAAGTAATGGAGTCTTTCATA<br>TTGTGCTCAAATCATTTCCCCGTCGCTTCCGGTCACGCTCAAG<br>ATGCGCAACAAGTGCGCTAACACATCTCCCACAATATCAGG<br>CTCTACAATGTCCGAATATACCAACAGTAATCTATAATGAGC<br>ACACAGATATGCCGCTTACTGACTTTCGACGTTCTTTTCTTCA<br>TCATAATCATCATCGGTACTCTCAAGTTCAAACATAGCATTT

							GCGCGAGTCCGTCGTACAGTATCACTTTAGCACCCTGCAAAA<br>CAAAAACAGAATCTGCCCCTTCCGGTAGACGCACTATGTTAA<br>GAGTTCCCGAAATTGGAATCGATGCAAAACTTTGAGGGTAG<br>ACAGAATATAGTATATAGACAAAATATGTCCAGATCGAAAT<br>AGAGTTTTAGGTCCAGATCAAGCTTTCAAGTTGTGAGTCTAG<br>CATCGTTCTATGGTGGGAAATGTGTTAAAAATATTTTTTAAAT<br>GCTGCACCCTTTACTGTGTTGTTTACATGCAGTGTATATATTC<br>AGCTATATACATGCAGTGTATATATATTGTTTATATTCAGCTA<br>CTCTTCTCGGCTACAGCGGCAGATAATTCAATTAGCCATCTC<br>GCAAGAAAGGAAATATGACTGTGTTCCTGACTAAAGTACAC<br>CTGGCATGTATGCTTGTACCTAAAAGGTCCGAATTCGAGAAT<br>TTTCTCGAAAGATAATTAAGGAGCGTCAGGGAAATTGTTGGA<br>GGTCAGTGATGAAAGGGCGACGATTGTTTTTCTGACGTCATA<br>GGTCTGGGTTTTTTTTTTTACAACTTCCGTGTCGAAATTGCACT<br>ATTTCCTGCAGAGATCAGAGCTATATATTAACAGCGTTTCAG<br>GGTATTACTGTGCTATGAATATATTCCAGATTTTATGCGTGTT<br>TTAACGTATATCACTTGCAAAGATGTATTTTTATTGTCTTAAA<br>ATACTACCATGATGTGGTGTACCTCGGAAAAATAAACTTACT<br>TTTACATATATAAGAGTGTTTATTGCTGTAGCAGGAAAGTTTA<br>CCTTCACTTTATTTAATGCTATATAATTGTACCTTACAAAATT<br>TTGTAGCTTATAATAAAGTGAATAATCTTAATATTTCAACGAT<br>ATTTGGAATGCTGTCAGCACAATTCAAACAACTTTCAGTGAA<br>TTATAGTGATTTCTCTTTAACAAGAATGCGATTAAAATCCAA<br>AATAAGGTCGGAAAAATTCACAGTTTTCTTCGCTAGATTTCA<br>AGTAATTTGTACGGTTTTCAGATAGTTCAAAAACTTTGACGT<br>ATGGATCTGACTTAGAGAACTTCCGCCACGTTTCACCGGGTT<br>CCCCTCTCTTAGCAAAATTCGTCCACTCTTTCACCATCTCAAA<br>GCTTAGTTCTTCTTCCTTTCGGGTGTAGTTGGACAGAGGAGTT

							AGAGGACGTCCAAAGATAAATTGCATATCGTCACAATGCAT<br>AACCCCCATCCACTCTGCGTACGGAGTACTCGATGGTCTGTG<br>ATTGAAATAGTATCGGTAGATTTTATTATTTTTTTCTGCAACTT<br>TCTCAGCAAAATATTTGCTTGGGCACATAACCATGAAATCCC<br>CAAGAGAGTGATAAACTTGACTTCGTATTGTAGCATAATCGT<br>CGTCTTTAACGCCACTGAGGTAATGCTGGATTATTCCTTCCGG<br>ATCTGGAAATATTGTCCCGAAATAATCTCTCATGAGAGACTC<br>CCCTAACGTCTTATTAATGCGAGGATTTAATTCACCAAAAGG<br>GCCTGTGATCTGTGTAAAAGTGTTAACAAATGGGAATGATCC<br>CTCGTCTTGAGTAACTCCTATCATAGAATCAACACCGTTAAA<br>GTTTCCTTCTCTGATAGCTTTTTGTGGGTCTACCGGTAGAAAA<br>TCATCCCCACTGATGGTAAAGAAAGTTATCACTGGGAAGAC<br>GGGAACTAAGGAAAACGTAGTTCTCACCATATCTTTTGGAGC<br>TTTACTTCTTAGGCATTCGATCACATCATCAGGATTTTTCTTCA<br>GCGTTATGTTTTCATTTGCACATTCCATCTTCTCTGCGAGCTTT<br>TGTCCTAATTTAAGATTCTCAGCCCTATGGTCATTCAGAAAAT<br>ATGCTGGACAACCACTTTGCATTATTGCTCTCTGGAAGAGAC<br>CTTTCGAAAGAGGAGAAACACACAGCAAACCTGTAGCTACG<br>CCGCCGGCGCTGTTGCCGAACACCGTAATTCTTTTATTGTCTC<br>CGCCGAATGCAGCAATGTTTTCGTTCGTCCATTTAAGGGCTTC<br>CAAAATGTCATACAAGCCTAGAACGAAATTCTCGATATGAA<br>TATTTCTTGTACGAGAAGTTACATACAAGGATTATTCTTGATA<br>AAATATCAGTCATTTATTTTTTTAGATTTATCCATATAGGTTCT<br>AAATAGAATATTTAATATTTAATATAAATATTAATGCCTAAC<br>AGCACAGGGCAGAA

PhdEnzCho 37	W4VSJ0	2,02E-65	35,676	PF00135	43419,31	SLYVTSRTRNIHIENFVLGLYDILEALKWTNENIAAFGGDNKRITVFGNSAGGVATGLLCVSPLSKGLFQRAIMQSGCPAYFLNDHRAENLKLGQKLAEKMECANENITLKKNPDDVIECLRSKAPKDMVRTTFSLVPVFPVITFFTISGDDFLPVDPQKAIREGNFNGVDSMIGVTQDEGSFPFVNTFTQITGPFGELNPRINKTLGESLMRDYFGTIFPDPEGIIQHYLSGVKDDDYATIRSQVYHSLGDFMVMCPSKYFAEKVAEKNNKIYRYYFNHRPSSTPYAEWMGVMHCDDMQFIFGRPLTPLSNYTRKEEELSFEMVKEWTNFAKRGEPGETWRKFSKSDPYVKVFELSENRTNYLKSSEENCEFFRPYFGF*	CGATAAACCTTTTGCAAGCAAGTTTCTCATGATCTGGCAATATTCTAGAAAGTGCAAACATGATTATGTAATATGAAAAATGCTGTATATGAAACTCGAGGTATTTTTGATTTATTCTTTATACTTTCATATTACATATTCATCTTTACACTTTCATAACTATGCTTTCATGAGTCTGCGTACATCAACATAACACATTAGAAAACTTTAATATGCAATTCATATTCTCAATTTAAGCTAGGATCCCCTTTATTTTGCAATTTAACTCAAAACTTCAAAGGAATAAACCTAAAATTATGCTTTTTTAACTACGGGAAGCTTCAAATTTTACGATAAACCTTTTGCAAGCAAGTTTCTCATGATCTGGCAATATTCTAGAAAGTGCAAACATGATTATGTAATATGAAAAATGCTGTATATGAAACTCAAGGTATTTTTGATTTATTTTTTATACTTTCATATTACATAGTCATCTTTACACTTTCGTAATGCTGCGTATATCAACATTAGCAAACTTTAATATGCAATAAATAGCTATTAGGATAACCTTTTCTTTTAATAACGTCACTATGCCTCTTTTAAATAGAATTGACTAGCTTCGATGTTTACTCGGAGCTTATATTATTCCATTCTTCTCTCAATATGGCTTTCAACATAGCCTTTGAAGCTATATTGTGTTGGCAGATTCTGCGTTCCAGCAAATCTCAAAAACACTTTATCGGGTAAGTCAGGCGATTCTGGAGTTGTTTATGTGTAGGAGTAGATAACTATTTTAGCACGTCATATAAGAACTAGATATTCATAACTTGAACAGTGTGCTTGGGGACCTTGTCCAATTAGAAATAATAACTGTTGTCTATCTTAAGTACTCTGCATTAAATTTCGTTTTAAAATGTTTAAATAATCCATTTTATTCAATTGTGTCCTGAATAAAGTGTAGTTGTCCCACGCCTCAGTTTTAGATACACCCCCAAATAATAATACTTTAGCCACCGTGTTTTACAGTTGGCATTAAATATTCTTACTTAAGCTCTTTAACAGGTTTTCGGTACAAAAGCTTGAATCCTTTAATGTCAAATACGCAGAATCTGCTCTAATCGGAAAATATTACCAGTTGCAGAGCTCATTTTGCTTATTAATATATTCTTTTGCAACGTCAATGCTTAAATCTATTATCTCAGGAAATGTAAAACAGC

							TAAAAGGGATGTTACAACAGTTTCTCTTCGAGCCGTCACATT<br>TATCTTTTGTTCTTTCGCCTCTTGTGTTTTACCTATTCCTCGACA<br>TATTTATGGCTATTTTTCAGTTCACAACTCTCTCGGATGGCGT<br>AATCTTTTCTTGAAATTTCTTCGAATTTTCTCTCGTTTTATATA<br>TATCAAAGCGGTAATGCGTTTTGCATTTTGTTCCGTTTATAGT<br>AACGTGCGCACCTAATACTTTCACCTGCTTCATGAAGGACTG<br>TATCAAATAGTCCGAAGTATGCTGCAGCTTTCCTGTCTTACTG<br>TGGATCAGAATCTTTTTTTGCAGCTTTATGGTATTCACTGCCT<br>CAGTGACGAGACTTTGAAAATTTCAATCATGTTTTAAAATTC<br>CGCATATATCAACACGAAAATGGTGTCTCCAAGGATTTGGAA<br>AATTGAATTTCTACTCGCAAGTTACCTTGGTGTTGCAATTAAA<br>ATGAGGTCCATCACACAAGGATGGGATAAAGTCGAACACGT<br>AAACTGTATAGCCTTCTACAAATTTGTCTTTCGAGAAGTAAA<br>TACCTCTGTTTTGATTAAAGTTGATAAACAATTTGGTGACAA<br>GCGCGAATATATCTTTTAGAATCAAAATTCGGTTATAAATGT<br>TTGCTTGGCACTGAGTGACCATCTACTTAAACACTTAAAAAG<br>TTGACCATGATAATGATGAAAAAAAGGAAGAGGGCAGGTTA<br>TGTTTCCTATTTACCGTGGCATTCTTCAGGGAGGTAATAATCA<br>CTCCTTTGGACATCAGTCACAAAAATACATTGTCTTTCATAA<br>AAGAAAATGTTCCAACTGAAACTGAGTATGCTTTATACAATA<br>CTCTGTCGATGGTATATTTCGAGAGACTTTTTTCGAGAACATT<br>GACATGTCCCAAGGATACGCCGGGACATTCACTGTCCTAATT<br>AACAAGGAGGCATGCTCAGATACGATTTTATACTGGTCTCAT<br>TTTGGGGGGGGAGACTAAACAAAAACTGTGTCTGTTGTGGCT<br>CATTTTGATTCGCAGATCATCAAATTTAATAAAAGTAGATTTT<br>GTTGAAAACATTCGCATCATCTCCAACGTTCGATTTTTAGCTG<br>TAAGTTCATTACGCTTCGTCAAACCTACATTTCTATCGATTTC<br>TTTCAACGGGTTCGTCGAATGTAATTTGCTGTCTTTGTAAAAG

							CATTCAACGGGGGAGTAAACTTTTCTTGACATTTTTTCCATAT ATCCAAATTCTTCAATATAGGTTCTGTAGGGATAAGTATTGCT ATAGACGACGAGACGACTCGCTTCTTAAGTTGATTTCCACTC TGAAAAAAAGAGCGTGTAAAATTAAATTCACGGGGTCGACG AGTTCATTTTCAGGTAAAGCGGATCTATCCGACTTCATAATTT TGGCTTTTACGTGCAGTTAGGAAGACGATAAGTCAATGTAAT CTTCACCATTGTTACATTCCACGGGTCCACCATCGCGTAGGTT GGACAGGGGAGGATATTCAATAAAATGTCCTCTTCGATTGCG GTCTCGCATAAGCGGGATCATGAACAAATGGAAATCAGATT TTGCGCAAGCAACACAGTATTGATAGATGCGTGTCGATATGC TTAACCGAAAATAACGCGTGATTCCATTTTCTTGGTTCTTGGT GAACTAATGCGTTATTCCAACGCTTTCTCTTTTAAATTATGAC CAGAAGTAATAATGGACTTCATTTTTCAAGCTACTTTGTCCGT TAATTGGCTTCCAGCTTATTTCGAATAGCGTTTAACAGACATT TTTAGAACATTCAATATGCCCACACTGCTGTGAAATCATCGT TTGCCGACTACTTTTATGCCCAGTTTTAAAAGGGGAACTGTC ACTCGATAAAAAGGACAAAAGAATCTGCCAAAACCATAAA CTGTCTGAAATGTATCCCCTTTATAATAATTCAACTCGCCACG CTTTGCATAATCCTCGAAGGATTTTGGACAACATTGATGAGG CACATACATCACGATAGAAACTTTTGCCAAAAGTACAGTTTT ACGTACGAGCGCTTGAGTTCAGATGACACAATTTCGCGTGTA TGCATTCTTATAACGACATTCAATACTTTTCTATTCCGTTGGA AGTAATGGAGTCTTTCATATTGTGCTCAAATCATTTCCCCGTC GCTTCCGGTCACGCTCAAGATGCGCAACAAGTGCGCTAACA CATCTCCCACAATATCAGGCTCTACAATGTCCGAATATATCA ACAGTAATCTATAATGAGCGCACAGATATGCCGCTTACTGAC TTTCGACGTTCTTTTCTTCATCATAATCATCATCGGTACTCTCA AGTTCAAACATAGCATTTGCGCGAGTCCGTCGTACAGTATCA

							CTTTAGCACCCTGCAAAACAAAAACAGAATCTGCCCCTTCCG<br>GTAGACGCACTATGTTAAGAGTTCCCGAAATTGGAATCGATG<br>CAAAACTTTGAGGGTAGACAGAATATAGTATATAGACAAAA<br>TATGTCCAGATCGAAATAGAGTTTTAGGTCCAGATCAAGCTT<br>TCAAGTTGTGAGTCTAGCATCGTTCTATGGTGGGAAATGTGTT<br>AAAAATATTTTTTAAATGCTGCACCCTTTACTGTGTTGTTTAC<br>ATGCAGTGTATATATTCAGCTATATACATGCAGTGTATATAT<br>ATTGTTTATATTCAGCTACTCTTCTCGGCTACAGCGGCAGATA<br>ATTCAATTAGCCATCTCGCAAGAAAGGAAATATGACTGTGTT<br>CCTGACTAAAGTACACCTGGCATGTATGCTTGTACCTAAAAG<br>GTCCGAATTCGAGAATTTTCTCGAAAGATAATTAAGGAGCGT<br>CAGGGAAATTGTTGGAGGTCAGTGATGAAAGGGCGACGATT<br>GTTTTTCTGACGTCATAGGTCTGGGTTTTTTTTTTACAACTTC<br>CGTGTCGAAATTGCACTATTTCCTGCAGAGATCAGAGCTATA<br>TATTAACAGCGTTTCAGGGTATTACTGTGCTATGAATATATTC<br>CAGATTTTATGCGTGTTTTAACGTATATCACTTGCAAAGATGT<br>ATTTTTATTGTCTTAAAATACTACCATGATGTGGTGTACCTCG<br>GAAAAATAAACTTACTTTTACATATATAAGAGTGTTTATTGCT<br>GTAGCAGGAAAGTTTACCTTCACTTTATTTAATGCTATATAAT<br>TGTACCTTACAAAATTTTGTAGCTTATAATAAAGTGAATAAT<br>CTTAATATTTCAACGATATTTGGAATGCTGTCAGCACAATTCA<br>AACAACTTTCAGTGAATTATAGTGATTTCTCTTTAACAAGAAT<br>GCGATTAAAATCCAAAATAAGGTCGGAAAAAATTCACAGTTTT<br>CTTCGCTAGATTTCAAGTAATTTGTACGGTTTTCAGATAGTTC<br>AAAAACTTTGACGTATGGATCTGACTTAGAGAACTTCCGCCA<br>CGTTTCACCGGGTTCCCCTCTCTTAGCAAAATTCGTCCACTCT<br>TTCACCATCTCAAAGCTTAGTTCTTCTTCCTTTCGGGTGTAGTT<br>GGACAGAGGAGTTAGAGGACGTCCAAAGATAAATTGCATAT

							CGTCACAATGCATAACCCCCATCCACTCTGCGTACGGAGTACTCGATGGTCTGTGATTGAAATAGTATCGGTAGATTTTATTATTTTTTTCTGCAACTTTCTCAGCAAAATATTTGCTTGGGCACATAACCATGAAATCCCCAAGAGAGTGATAAACTTGACTTCGTATTGTAGCATAATCGTCGTCTTTAACGCCACTGAGGTAATGCTGGATTATTCCTTCCGGATCTGGAAATATTGTCCCGAAATAATCTCTCATGAGAGACTCCCCTAACGTCTTATTAATGCGAGGATTTAATTCACCAAAAGGGCCTGTGATCTGTGTAAAAGTGTTAACAAATGGGAATGATCCCTCGTCTTGAGTAACTCCTATCATAGAATCAACACCGTTAAAGTTTCCTTCTCTGATAGCTTTTTGTGGGTCTACCGGTAGAAAATCATCCCCACTGATGGTAAAGAAAGTTATCACTGGGAAGACGGGAACTAAGGAAAACGTAGTTCTCACCATATCTTTTGGAGCTTTACTTCTTAGGCATTCGATCACATCATCAGGATTTTTCTTCAGCGTTATGTTTTCATTTGCACATTCCATCTTCTCTGCGAGCTTTTGTCCTAATTTAAGATTCTCAGCCCTATGGTCATTCAGAAAATATGCTGGACAACCACTTTGCATTATTGCTCTCTGGAAGAGACCTTTCGAAAGAGGAGAAACACACAGCAAACCTGTAGCTACGCCGCCGGCGCTGTTGCCGAACACCGTAATTCTTTTATTGTCTCCGCCGAATGCAGCAATGTTTTCGTTCGTCCATTTAAGGGCTTCCAAAATGTCATACAAGCCTAGAACGAAATTCTCGATATGAATATTTCTTGTACGAGAAGTTACATACAAGGATTATTCTTGATAAAATATCAGTCATTTATTTTTTTAGATTTATCCATATAGGTTCTAAATAGAATATTTAATATTTAATATAAATATTAATGCCTAACAGCACAGGGCAGAA

PhdEnzCho 38	W4VSJ0	8,75E-110	37,618	PF00135	63218.00<br>**58747.62**	MSSPSSNVIAGAQGL<br>GAAMCGKLQKNVH<br>KSLHLLFLLLPGTILA<br>**SSVTVNTPTGEVTG**<br>**SVMTYDDINLNVFL**<br>**GIPFAEPPVGNLRF**<br>**KRTVPVKPWTSPVI**<br>**ANSLPPACYQYSPD**<br>**NYPWEDKVQGQSE**<br>**DCLYLNIWAPEISN**<br>**SSEKKAVMFWVFS**<br>**GGFATGSGRMPLY**<br>**DGRVLTAIGDVIVV**<br>**AINYRLSVLGFFTSE**<br>**TEEAPGNVGLYDIL**<br>**EALKWTNENIAAFG**<br>**GDNKRITVFGNSA**<br>**GGVATGLLCVSPLS**<br>**KGLFQRAIMQSGC**<br>**PAYFLNDHRAENLK**<br>**LGQKLAEKMECAN**<br>**ENITLKKNPDDVIE**<br>**CLRSKAPKDMVRT**<br>**TFSLVPVFPVITFFTI**<br>**SGDDFLPVDPQKAI**<br>**REGNFNGVDSMIG**<br>**VTQDEGSFPFVNTF**<br>**TQITGPFGELNPRIN**	CGATAAACCTTTTGCAAGCAAGTTTCTCATGATCTGGCAATA<br>TTCTAGAAAGTGCAAACATGATTATGTAATATGAAAAATGCT<br>GTATATGAAACTCGAGGTATTTTTGATTTATTCTTTATACTTTC<br>ATATTACATATTCATCTTTACACTTTCATAACTATGCTTTCATG<br>AGTCTGCGTACATCAACATAACACATTAGAAAACTTTAATAT<br>GCAATTCATATTCTCAATTTAAGCTAGGATCCCCTTTATTTTG<br>CAATTTAACTCAAAACTTCAAAGGAATAAACCTAAAATTATG<br>CTTTTTTAACTACGGGAAGCTTCAAATTTTACGATAAACCTTT<br>TGCAAGCAAGTTTCTCATGATCTGGCAATATTCTAGAAAGTG<br>CAAACATGATTATGTAATATGAAAAATGCTGTATATGAAACT<br>CAAGGTATTTTTGATTTATTTTTTATACTTTCATATTACATAGT<br>CATCTTTACACTTTCGTAATTCTGCGTATATCAACTGAAAACA<br>TTAGCAAACTTTAATATGCAATAAATAGCTATTAGGATAACC<br>TTTTCTTTTAATAACGTCACTATGCCTCTTTTAAATAGAATTG<br>ACTAGCTTCGATGTTTACTCGGAGCTTATATTATTCCATTCTTC<br>TCTCAATATGGCTTTCAACATAGCCTTTGAAGCTATATTGTGT<br>TGGCAGATTCTGCGTTCCAGCAAATCTCAAAAACACTTTATC<br>GGGTAAGTCAGGCGATTCTGGAGTTGTTTATGTGTAGGAGTA<br>GATAACTATTTTAGCACGTCATATAAGAACTAGATATTCATA<br>ACTTGAACAGTGTGCTTGGGGACCTTGTCCAATTAGAAATAA<br>TAACTGTTGTCTATCTTAAGTACTCTGCATTAAATTTCGTTTTA<br>AAATGTTTAAATAATCCATTTTATTCAATTGTGTCCTGAATAA<br>AGTGTAGTTGTCCCACGCCTCAGTTTTAGATACACCCCCAAA<br>TAATAATACTTTAGCCACCGTGTTTTACAGTTGGCATTAAATA<br>TTCTTACTTAAGCTCTTTAACAGGTTTTCGGTACAAAAGCTTG<br>AATCCTTTAATGTCAAATACGCAGAATCTGCTCTAATCGGAA<br>AATATTACCAGTTGCAGAGCTCATTTTGCTTATTAATATATTC<br>TTTTGCAACGTCAATGCTTAAATCTATTATCTCAGGAAATGTA

						**KTLGESLMRDYFGT<br>IFPDPEGIIQHYLSG<br>VKDDDYATIRSQV<br>YHSLGDFMVMCPS<br>KYFAEKVAEKNNKI<br>YRYYFNHRPSSTPY<br>AEWMGVMHCDD<br>MQFIFGRPLTPLSN<br>YTRKEEELSFEMVK<br>EWTNFAKRGEPGET<br>WRKFSKSDPYVKVF<br>ELSENRTNYLKSSEE<br>NCEFFRPYFGF***	AAACAGCTAAAAGGGATGTTACAACAGTTTCTCTTCGAGCCG<br>TCACATTTATCTTTTGTTCTTTCGCCTCTTGTGTTTTACCTATTC<br>CTCGACATATTTATGGCTATTTTTCAGTTCACAACTCTCTCGG<br>ATGGCGTAATCTTTTCTTGAAATTTCTTCGAATTTTCTCTCGTT<br>TTATATATATCAAAGCGGTAATGCGTTTTGCATTTTGTTCCGT<br>TTATAGTAACGTGCGCACCTAATACTTTCACCTGCTTCATGAA<br>GGACTGTATCAAATAGTCCGAAGTATGCTGCAGCTTTCCTGT<br>CTTACTGTGGATCAGAATCTTTTTTTGCAGCTTTATGGTATTCA<br>CTGCCTCAGTGACGAGACTTTGAAAATTTCAATCATGTTTTAA<br>AATTCCGCATATATCAACACGAAAATGGTGTCTCCAAGGATT<br>TGGAAAATTGAATTTCTACTCGCAAGTTACCTTGGTGTTGCA<br>ATTAAAATGAGGTCCATCACACAAGGATGGGATAAAGTCGA<br>ACACGTAAACTGTATAGCCTTCTACAAATTTGTCTTTCGAGA<br>AGTAAATACCTCTGTTTTGATTAAAGTTGATAAACAATTTGGT<br>GACAAGCGCGAATATATCTTTTAGAATCAAAATTCGGTTATA<br>AATGTTTGCTTGGCACTGAGTGACCATCTACTTAAACACTTA<br>AAAAGTTGACCATGATAATGATGAAAAAAAGGAAGAGGGC<br>AGGTTATGTTTCCTATTTACCGTGGCATTCTTCAGGGAGGTAA<br>TAATCACTCCTTTGGACATCAGTCACAAAAATACATTGTCTTT<br>CATAAAAGAAAATGTTCCAACTGAAACTGAGTATGCTTTATA<br>CAATACTCTGTCGATGGTATATTTCGAGAGACTTTTTTCGAGA<br>ACATTGACATGTCCCAAGGATACGCCGGGACATTCACTGTCC<br>TAATTAACAAGGAGGCATGCTCAGATACGATTTTATACTGGT<br>CTCATTTTGGGGGGGGAGACTAAACAAAAACTGTGTCTGTTG<br>TGGCTCATTTTGATTCGCAGATCATCAAATTTAATAAAAGTA<br>GATTTTGTTGAAAACATTCGCATCATCTCCAACGTTCGATTTT<br>TAGCTGTAAGTTCATTACGCTTCGTCAAACCTACATTTCTATC<br>GATTTCTTTCAACGGGTTCGTCGAATGTAATTTGCTGTCTTTGT

							AAAAGCATTCAACGGGGGAGTAAACTTTTCTTGACATTTTTT<br>CCATATATCCAAATTCTTCAATATAGGTTCTGTAGGGATAAG<br>TATTGCTATAGACGACGAGACGACTCGCTTCTTAAGTTGATTT<br>CCACTCTGAAAAAAAGAGCGTGTAAAATTAAATTCACGGGG<br>TCGACGAGTTCATTTTCAGGTAAAGCGGATCTATCCGACTTC<br>ATAATTTTGGCTTTTACGTGCAGTTAGGAAGACGATAAGTCA<br>ATGTAATCTTCACCATTGTTACATTCCACGGGTCCACCATCGC<br>GTAGGTTGGACAGGGGAGGATATTCAATAAAATGTCCTCTTC<br>GATTGCGGTCTCGCATAAGCGGGATCATGAACAAATGGAAA<br>TCAGATTTTGCGCAAGCAACACAGTATTGATAGATGCGTGTC<br>GATATGCTTAACCGAAAATAACGCGTGATTCCATTTTCTTGGT<br>TCTTGGTGAACTAATGCGTTATTCCAACGCTTTCTCTTTTAAA<br>TTATGACCAGAAGTAATAATGGACTTCATTTTTCAAGCTACTT<br>TGTCCGTTAATTGGCTTCCAGCTTATTTCGAATAGCGTTTAAC<br>AGACATTTTTAGAACATTCAATATGCCCACACTGCTGTGAAA<br>TCATCGTTTGCCGACTACTTTTATGCCCAGTTTTAAAAGGGGA<br>ACTGTCACTCGATAAAAAGGACAAAAGAATCTGCCAAAACC<br>ATAAACTGTCTGAAATGTATCCCCTTTATAATAATTCAACTCG<br>CCACGCTTTGCATAATCCTCGAAGGATTTTGGACAACATTGA<br>TGAGGCACATACATCACGATAGAAACTTTTGCCAAAAGTAC<br>AGTTTTACGTACGAGCGCTTGAGTTCAGATGACACAATTTCG<br>CGTGTATGCATTCTTATAACGACATTCAATACTTTTCTATTCC<br>GTTGGAAGTAATGGAGTCTTTCATATTGTGCTCAAATCATTTC<br>CCCGTCGCTTCCGGTCACGCTCAAGATGCGCAACAAGTGCGC<br>TAACACATCTCCCACAATATCAGGCTCTACAATGTCCGAATA<br>TACCAACAGTAATCTATAATGAGCACACAGATATGCCGCTTA<br>CTGACTTTCGACGTTCTTTTCTTCATCATAATCATCATCGGTA<br>CTCTCAAGTTCAAACATAGCATTTGCGCGAGTCCGTCGTACA

							GTATCACTTTAGCACCCTGCAAAACAAAAACAGAATCTGCC<br>CCTTCCGGTAGACGCACTATGTTAAGAGTTCCCGAAATTGGA<br>ATCGATGCAAAACTTTGAGGGTAGACAGAATATAGTATATA<br>GACAAAATATGTCCAGATCGAAATAGAGTTTTAGGTCCAGAT<br>CAAGCTTTCAAGTTGTGAGTCTAGCATCGTTCTATGGTGGGA<br>AATGTGTTAAAAATATTTTTTAAATGCTGCACCCTTTACTGTG<br>TTGTTTACATGCAGTGTATATATTCAGCTATATACATGCAGTG<br>TATATATATTGTTTATATTCAGCTACTCTTCTCGGCTACAGCG<br>GCAGATAATTCAATTAGCCATCTCGCAAGAAAGGAAATATG<br>ACTGTGTTCCTGACTAAAGTACACCTGGCATGTATGCTTGTAC<br>CTAAAAGGTCCGAATTCGAGAATTTTCTCGAAAGATAATTAA<br>GGAGCGTCAGGGAAATTGTTGGAGGTCAGTGATGAAAGGGC<br>GACGATTGTTTTTCTGACGTCATAGGTCTGGGTTTTTTTTTTA<br>CAACTTCCGTGTCGAAATTGCACTATTTCCTGCAGAGATCAG<br>AGCTATATATTAACAGCGTTTCAGGGTATTACTGTGCTATGA<br>ATATATTCCAGATTTTATGCGTGTTTTAACGTATATCACTTGC<br>AAAGATGTATTTTTATTGTCTTAAAATACTACCATGATGTGGT<br>GTACCTCGGAAAAATAAACTTACTTTTACATATATAAGAGTG<br>TTTATTGCTGTAGCAGGAAAGTTTACCTTCACTTTATTTAATG<br>CTATATAATTGTACCTTACAAAATTTTGTAGCTTATAATAAAG<br>TGAATAATCTTAATATTTCAACGATATTTGGAATGCTGTCAGC<br>ACAATTCAAACAACTTTCAGTGAATTATAGTGATTTCTCTTTA<br>ACAAGAATGCGATTAAAATCCAAAATAAGGTCGGAAAAATT<br>CACAGTTTTCTTCGCTAGATTTCAAGTAATTTGTACGGTTTTC<br>AGATAGTTCAAAAACTTTGACGTATGGATCTGACTTAGAGAA<br>CTTCCGCCACGTTTCACCGGGTTCCCCTCTCTTAGCAAAATTC<br>GTCCACTCTTTCACCATCTCAAAGCTTAGTTCTTCTTCCTTTCG<br>GGTGTAGTTGGACAGAGGAGTTAGAGGACGTCCAAAGATAA

							ATTGCATATCGTCACAATGCATAACCCCCATCCACTCTGCGT<br>ACGGAGTACTCGATGGTCTGTGATTGAAATAGTATCGGTAGA<br>TTTTATTATTTTTTTCTGCAACTTTCTCAGCAAAATATTTGCTT<br>GGGCACATAACCATGAAATCCCCAAGAGAGTGATAAACTTG<br>ACTTCGTATTGTAGCATAATCGTCGTCTTTAACGCCACTGAGG<br>TAATGCTGGATTATTCCTTCCGGATCTGGAAATATTGTCCCGA<br>AATAATCTCTCATGAGAGACTCCCCTAACGTCTTATTAATGC<br>GAGGATTTAATTCACCAAAAGGGCCTGTGATCTGTGTAAAAG<br>TGTTAACAAATGGGAATGATCCCTCGTCTTGAGTAACTCCTA<br>TCATAGAATCAACACCGTTAAAGTTTCCTTCTCTGATAGCTTT<br>TTGTGGGTCTACCGGTAGAAAATCATCCCCACTGATGGTAAA<br>GAAAGTTATCACTGGGAAGACGGGAACTAAGGAAAACGTA<br>GTTCTCACCATATCTTTTGGAGCTTTACTTCTTAGGCATTCGAT<br>CACATCATCAGGATTTTTCTTCAGCGTTATGTTTTCATTTGCAC<br>ATTCCATCTTCTCTGCGAGCTTTTGTCCTAATTTAAGATTCTCA<br>GCCCTATGGTCATTCAGAAAATATGCTGGACAACCACTTTGC<br>ATTATTGCTCTCTGGAAGAGACCTTTCGAAAGAGGAGAAAC<br>ACACAGCAAACCTGTAGCTACGCCGCCGGCGCTGTTGCCGA<br>ACACCGTAATTCTTTTATTGTCTCCGCCGAATGCAGCAATGTT<br>TTCGTTCGTCCATTTAAGGGCTTCCAAAATGTCATACAAGCC<br>GACGTTTCCAGGAGCTTCTTCGGTTTCAGATGTGAAGAATCC<br>GAGCACACTTAACCGGTAGTTTATCGCAACAACTATTACATC<br>TCCAATTGCAGTTAGAACACGTCCATCATATAGAGGCATCCT<br>ACCTGAACCGGTTGCAAAGCCGCCTGAGAACACCCAAAACA<br>TGACGGCTTTTTTCTCGCTACTATTGGAAATTTCTGGTGCCCA<br>TATATTCAAATACAAGCAGTCTTCGCTTTGACCCTGAACTTTA<br>TCTTCCCACGGATAGTTGTCCGGAGAGTACTGGTAACAAGCA<br>GGCGGTAAGCTGTTTGCTATTACAGGAGAGGTCCAAGGTTTG

							ACAGGAACTGTTCGCTTAAAGCGTAAATTCCCCACTGGAGGCTCAGCAAACGGAATTCCGAGAAAGACATTTAGATTTATGTCGTCATAAGTCATAACAGAACCAGTCACTTCACCTGTAGGAGTATTAACTGTAACGCTGGATGCTAAGATAGTTCCTGGTAGCAGTAAAAATAGTAGATGCAAGCTCTTATGAACGTTTTTCTGGAGCTTTCCGCACATAGCGGCTCCTAAACCTTGGGCTCCAGCAATAACGTTAGACGAGGGAGACGACATGATGCTGCTGCCTAGATCTCCAGTGTGCTGTGTCGTACACAAATGCGCCTATTTCCTGTTTGTAAAGCTCCTTCGTACACCAGTATCGTCAACAGCTATTTTTACATTAGTTATTCCGTTTCTGTCAGCAATCTTGCATCGCACAAATCTGCTAGTGTAGGTCTTCATCCGAATTATGCCCAAACTGGTTGTAGTTTTACTGTGGATAAGAAGCTTTCTTAACCTAATTTGTTCTCCACAGGCT

PhdEnzCho 39	W4VSJ0	8,70E-110	37,618	PF00135	63218.00<br>**58747.62**	MSSPSSNVIAGAQGL<br>GAAMCGKLQKNVH<br>KSLHLLFLLLPGTILA<br>**SSVTVNTPTGEVTG**<br>**SVMTYDDINLNVFL**<br>**GIPFAEPPVGNLRF**<br>**KRTVPVKPWTSPVI**<br>**ANSLPPACYQYSPD**<br>**NYPWEDKVQGQSE**<br>**DCLYLNIWAPEISN**<br>**SSEKKAVMFWVFS**<br>**GGFATGSGRMPLY**<br>**DGRVLTAIGDVIVV**<br>**AINYRLSVLGFFTSE**<br>**TEEAPGNVGLYDIL**<br>**EALKWTNENIAAFG**<br>**GDNKRITVFGNSA**<br>**GGVATGLLCVSPLS**<br>**KGLFQRAIMQSGC**<br>**PAYFLNDHRAENLK**<br>**LGQKLAEKMECAN**<br>**ENITLKKNPDDVIE**<br>**CLRSKAPKDMVRT**<br>**TFSLVPVFPVITFFTI**<br>**SGDDFLPVDPQKAI**<br>**REGNFNGVDSMIG**<br>**VTQDEGSFPFVNTF**<br>**TQITGPFGELNPRIN**	CGATAAACCTTTTGCAAGCAAGTTTCTCATGATCTGGCAATA<br>TTCTAGAAAGTGCAAACATGATTATGTAATATGAAAAATGCT<br>GTATATGAAACTCGAGGTATTTTTGATTTATTCTTTATACTTTC<br>ATATTACATATTCATCTTTACACTTTCATAACTATGCTTTCATG<br>AGTCTGCGTACATCAACATAACACATTAGAAAACTTTAATAT<br>GCAATTCATATTCTCAATTTAAGCTAGGATCCCCTTTATTTTG<br>CAATTTAACTCAAAACTTCAAAGGAATAAACCTAAAATTATG<br>CTTTTTTAACTACGGGAAGCTTCAAATTTTACGATAAACCTTT<br>TGCAAGCAAGTTTCTCATGATCTGGCAATATTCTAGAAAGTG<br>CAAACATGATTATGTAATATGAAAAATGCTGTATATGAAACT<br>CAAGGTATTTTTGATTTATTTTTTATACTTTCATATTACATAGT<br>CATCTTTACACTTTCGTAATTCTGCGTATATCAACTGAAAACA<br>TTAGCAAACTTTAATATGCAATAAATAGCTATTAGGATAACC<br>TTTTCTTTTAATAACGTCACTATGCCTCTTTTAAATAGAATTG<br>ACTAGCTTCGATGTTTACTCGGAGCTTATATTATTCCATTCTTC<br>TCTCAATATGGCTTTCAACATAGCCTTTGAAGCTATATTGTGT<br>TGGCAGATTCTGCGTTCCAGCAAATCTCAAAAACACTTTATC<br>GGGTAAGTCAGGCGATTCTGGAGTTGTTTATGTGTAGGAGTA<br>GATAACTATTTTAGCACGTCATATAAGAACTAGATATTCATA<br>ACTTGAACAGTGTGCTTGGGGACCTTGTCCAATTAGAAATAA<br>TAACTGTTGTCTATCTTAAGTACTCTGCATTAAATTTCGTTTTA<br>AAATGTTTAAATAATCCATTTTATTCAATTGTGTCCTGAATAA<br>AGTGTAGTTGTCCCACGCCTCAGTTTTAGATACACCCCCAAA<br>TAATAATACTTTAGCCACCGTGTTTTACAGTTGGCATTAAATA<br>TTCTTACTTAAGCTCTTTAACAGGTTTTCGGTACAAAAGCTTG<br>AATCCTTTAATGTCAAATACGCAGAATCTGCTCTAATCGGAA<br>AATATTACCAGTTGCAGAGCTCATTTTGCTTATTAATATATTC<br>TTTTGCAACGTCAATGCTTAAATCTATTATCTCAGGAAATGTA

						**KTLGESLMRDYFGT**<br>**IFPDPEGIIQHYLSG**<br>**VKDDDYATIRSQV**<br>**YHSLGDFMVMCPS**<br>**KYFAEKVAEKNNKI**<br>**YRYYFNHRPSSTPY**<br>**AEWMGVMHCDD**<br>**MQFIFGRPLTPLSN**<br>**YTRKEEELSFEMVK**<br>**EWTNFAKRGEPGET**<br>**WRKFSKSDPYVKVF**<br>**ELSENRTNYLKSSEE**<br>**NCEFFRPYFGF***	AAACAGCTAAAAGGGATGTTACAACAGTTTCTCTTCGAGCCG<br>TCACATTTATCTTTTGTTCTTTCGCCTCTTGTGTTTTACCTATTC<br>CTCGACATATTTATGGCTATTTTTCAGTTCACAACTCTCTCGG<br>ATGGCGTAATCTTTTCTTGAAATTTCTTCGAATTTTCTCTCGTT<br>TTATATATATCAAAGCGGTAATGCGTTTTGCATTTTGTTCCGT<br>TTATAGTAACGTGCGCACCTAATACTTTCACCTGCTTCATGAA<br>GGACTGTATCAAATAGTCCGAAGTATGCTGCAGCTTTCCTGT<br>CTTACTGTGGATCAGAATCTTTTTTTGCAGCTTTATGGTATTCA<br>CTGCCTCAGTGACGAGACTTTGAAAATTTCAATCATGTTTTAA<br>AATTCCGCATATATCAACACGAAAATGGTGTCTCCAAGGATT<br>TGGAAAATTGAATTTCTACTCGCAAGTTACCTTGGTGTTGCA<br>ATTAAAATGAGGTCCATCACACAAGGATGGGATAAAGTCGA<br>ACACGTAAACTGTATAGCCTTCTACAAATTTGTCTTTCGAGA<br>AGTAAATACCTCTGTTTTGATTAAAGTTGATAAACAATTTGGT<br>GACAAGCGCGAATATATCTTTTAGAATCAAAATTCGGTTATA<br>AATGTTTGCTTGGCACTGAGTGACCATCTACTTAAACACTTA<br>AAAAGTTGACCATGATAATGATGAAAAAAAGGAAGAGGGC<br>AGGTTATGTTTCCTATTTACCGTGGCATTCTTCAGGGAGGTAA<br>TAATCACTCCTTTGGACATCAGTCACAAAAATACATTGTCTTT<br>CATAAAAGAAAATGTTCCAACTGAAACTGAGTATGCTTTATA<br>CAATACTCTGTCGATGGTATATTTCGAGAGACTTTTTTCGAGA<br>ACATTGACATGTCCCAAGGATACGCCGGGACATTCACTGTCC<br>TAATTAACAAGGAGGCATGCTCAGATACGATTTTATACTGGT<br>CTCATTTTGGGGGGGGAGACTAAACAAAAACTGTGTCTGTTG<br>TGGCTCATTTTGATTCGCAGATCATCAAATTTAATAAAAGTA<br>GATTTTGTTGAAAACATTCGCATCATCTCCAACGTTCGATTTT<br>TAGCTGTAAGTTCATTACGCTTCGTCAAACCTACATTTCTATC<br>GATTTCTTTCAACGGGTTCGTCGAATGTAATTTGCTGTCTTTGT

							AAAAGCATTCAACGGGGGAGTAAACTTTTCTTGACATTTTTT CCATATATCCAAATTCTTCAATATAGGTTCTGTAGGGATAAG TATTGCTATAGACGACGAGACGACTCGCTTCTTAAGTTGATTT CCACTCTGAAAAAAAGAGCGTGTAAAATTAAATTCACGGGG TCGACGAGTTCATTTTCAGGTAAAGCGGATCTATCCGACTTC ATAATTTTGGCTTTTACGTGCAGTTAGGAAGACGATAAGTCA ATGTAATCTTCACCATTGTTACATTCCACGGGTCCACCATCGC GTAGGTTGGACAGGGGAGGATATTCAATAAAATGTCCTCTTC GATTGCGGTCTCGCATAAGCGGGATCATGAACAAATGGAAA TCAGATTTTGCGCAAGCAACACAGTATTGATAGATGCGTGTC GATATGCTTAACCGAAAATAACGCGTGATTCCATTTTCTTGGT TCTTGGTGAACTAATGCGTTATTCCAACGCTTTCTCTTTTAAA TTATGACCAGAAGTAATAATGGACTTCATTTTTCAAGCTACTT TGTCCGTTAATTGGCTTCCAGCTTATTTCGAATAGCGTTTAAC AGACATTTTTAGAACATTCAATATGCCCACACTGCTGTGAAA TCATCGTTTGCCGACTACTTTTATGCCCAGTTTTAAAAGGGGA ACTGTCACTCGATAAAAAGGACAAAAGAATCTGCCAAAACC ATAAACTGTCTGAAATGTATCCCCTTTATAATAATTCAACTCG CCACGCTTTGCATAATCCTCGAAGGATTTTGGACAACATTGA TGAGGCACATACATCACGATAGAAACTTTTGCCAAAAGTAC AGTTTTACGTACGAGCGCTTGAGTTCAGATGACACAATTTCG CGTGTATGCATTCTTATAACGACATTCAATACTTTTCTATTCC GTTGGAAGTAATGGAGTCTTTCATATTGTGCTCAAATCATTTC CCCGTCGCTTCCGGTCACGCTCAAGATGCGCAACAAGTGCGC TAACACATCTCCCACAATATCAGGCTCTACAATGTCCGAATA TATCAACAGTAATCTATAATGAGCGCACAGATATGCCGCTTA CTGACTTTCGACGTTCTTTTCTTCATCATAATCATCATCGGTA CTCTCAAGTTCAAACATAGCATTTGCGCGAGTCCGTCGTACA

							GTATCACTTTAGCACCCTGCAAAACAAAAACAGAATCTGCC<br>CCTTCCGGTAGACGCACTATGTTAAGAGTTCCCGAAATTGGA<br>ATCGATGCAAAACTTTGAGGGTAGACAGAATATAGTATATA<br>GACAAAATATGTCCAGATCGAAATAGAGTTTTAGGTCCAGAT<br>CAAGCTTTCAAGTTGTGAGTCTAGCATCGTTCTATGGTGGGA<br>AATGTGTTAAAAATATTTTTTAAATGCTGCACCCTTTACTGTG<br>TTGTTTACATGCAGTGTATATATTCAGCTATATACATGCAGTG<br>TATATATATTGTTTATATTCAGCTACTCTTCTCGGCTACAGCG<br>GCAGATAATTCAATTAGCCATCTCGCAAGAAAGGAAATATG<br>ACTGTGTTCCTGACTAAAGTACACCTGGCATGTATGCTTGTAC<br>CTAAAAGGTCCGAATTCGAGAATTTTCTCGAAAGATAATTAA<br>GGAGCGTCAGGGAAATTGTTGGAGGTCAGTGATGAAAGGGC<br>GACGATTGTTTTTCTGACGTCATAGGTTTGGGTTTTTTTTTTTT<br>ACAACTTCCGTGTCGAAATTGCACTATTTCCTGCAGAGATCA<br>GAGCTATATATTAACAGCGTTTCAGGGTATTACTGTGCTATG<br>AATATATTCCAGATTTTATGCGTGTTTTAACGTATATCACTTG<br>CAAAGATGTATTTTTATTGTCTTAAAATACTACCATGATGTGG<br>TGTACCTCGGAAAAATAAACTTACTTTTACATATATAAGAGT<br>GTTTATTGCTGTAGCAGGAAAGTTTACCTTCACTTTATTTAAT<br>GCTATATAATTGTACCTTACAAAATTTTGTAGCTTATAATAAA<br>GTGAATAATCTTAATATTTCAACGATATTTGGAATGCTGTCA<br>GCACAATTCAAACAACTTTCAGTGAATTATAGTGATTTCTCTT<br>TAACAAGAATGCGATTAAAATCCAAAATAAGGTCGGAAAAA<br>TTCACAGTTTTCTTCGCTAGATTTCAAGTAATTTGTACGGTTTT<br>CAGATAGTTCAAAAACTTTGACGTATGGATCTGACTTAGAGA<br>ACTTCCGCCACGTTTCACCGGGTTCCCCTCTCTTAGCAAAATT<br>CGTCCACTCTTTCACCATCTCAAAGCTTAGTTCTTCTTCCTTTC<br>GGGTGTAGTTGGACAGAGGAGTTAGAGGACGTCCAAAGATA

							AATTGCATATCGTCACAATGCATAACCCCCATCCACTCTGCG<br>TACGGAGTACTCGATGGTCTGTGATTGAAATAGTATCGGTAG<br>ATTTTATTATTTTTTTCTGCAACTTTCTCAGCAAAATATTTGCT<br>TGGGCACATAACCATGAAATCCCCAAGAGAGTGATAAACTT<br>GACTTCGTATTGTAGCATAATCGTCGTCTTTAACGCCACTGAG<br>GTAATGCTGGATTATTCCTTCCGGATCTGGAAATATTGTCCCG<br>AAATAATCTCTCATGAGAGACTCCCCTAACGTCTTATTAATG<br>CGAGGATTTAATTCACCAAAAGGGCCTGTGATCTGTGTAAAA<br>GTGTTAACAAATGGGAATGATCCCTCGTCTTGAGTAACTCCT<br>ATCATAGAATCAACACCGTTAAAGTTTCCTTCTCTGATAGCTT<br>TTTGTGGGTCTACCGGTAGAAAATCATCCCCACTGATGGTAA<br>AGAAAGTTATCACTGGGAAGACGGGAACTAAGGAAAACGT<br>AGTTCTCACCATATCTTTTGGAGCTTTACTTCTTAGGCATTCG<br>ATCACATCATCAGGATTTTTCTTCAGCGTTATGTTTTCATTTGC<br>ACATTCCATCTTCTCTGCGAGCTTTTGTCCTAATTTAAGATTCT<br>CAGCCCTATGGTCATTCAGAAAATATGCTGGACAACCACTTT<br>GCATTATTGCTCTCTGGAAGAGACCTTTCGAAAGAGGAGAA<br>ACACACAGCAAACCTGTAGCTACGCCGCCGGCGCTGTTGCC<br>GAACACCGTAATTCTTTTATTGTCTCCGCCGAATGCAGCAAT<br>GTTTTCGTTCGTCCATTTAAGGGCTTCCAAAATGTCATACAAG<br>CCGACGTTTCCAGGAGCTTCTTCGGTTTCAGATGTGAAGAAT<br>CCGAGCACACTTAACCGGTAGTTTATCGCAACAACTATTACA<br>TCTCCAATTGCAGTTAGAACACGTCCATCATATAGAGGCATC<br>CTACCTGAACCGGTTGCAAAGCCGCCTGAGAACACCCAAAA<br>CATGACGGCTTTTTTCTCGCTACTATTGGAAATTTCTGGTGCC<br>CATATATTCAAATACAAGCAGTCTTCGCTTTGACCCTGAACTT<br>TATCTTCCCACGGATAGTTGTCCGGAGAGTACTGGTAACAAG<br>CAGGCGGTAAGCTGTTTGCTATTACAGGAGAGGTCCAAGGTT

							TGACAGGAACTGTTCGCTTAAAGCGTAAATTCCCCACTGGAG<br>GCTCAGCAAACGGAATTCCGAGAAAGACATTTAGATTTATGT<br>CGTCATAAGTCATAACAGAACCAGTCACTTCACCTGTAGGAG<br>TATTAACTGTAACGCTGGATGCTAAGATAGTTCCTGGTAGCA<br>GTAAAAATAGTAGATGCAAGCTCTTATGAACGTTTTTCTGGA<br>GCTTTCCGCACATAGCGGCTCCTAAACCTTGGGCTCCAGCAA<br>TAACGTTAGACGAGGGAGACGACATGATGCTGCTGCCTAGA<br>TCTCCAGTGTGCTGTGTCGTACACAAATGCGCCTATTTCCTGT<br>TTGTAAAGCTCCTTCGTACACCAGTATCGTCAACAGCTATTTT<br>TACATTAGTTATTCCGTTTCTGTCAGCAATCTTGCATCGCACA<br>AATCTAAAAATAAATATGCGAGTCTGCTGATTCACACTTGAA<br>TTTATAAGGCTTTCGAAACAAAAAAGAACATTCG

PhdEnzCho 40	W4VSJ0	4,69E-21	52,941	PF00135	7626,52	QKFQSGEALSALGDIIVVTVSYRLGSLGFLYSGSNDAPGNVGLWDILAGLQWVNDHISAFGGDLSRITIAGES	CAGAAATTCCAGTCTGGAGAAGCTTTGTCAGCTCTGGGGGATATCATAGTGGTGACAGTCAGCTATAGACTAGGATCTTTGGGATTCTTGTATTCTGGATCAAATGATGCTCCCGGAAATGTTGGTTTATGGGACATTTTAGCCGGACTACAGTGGGTGAACGATCACATCAGTGCTTTTGGAGGAGATTTATCTCGCATAACAATCGCTGGAGAAAGTG
PhdEnzCho 41	W4VSJ0	1,93E-53	45,128	PF00135	22528.03<br>**20873.86**	MKILFLLLILPLASS**SSIVATTSGPMRGVSLAPKKVEAFYGIPYAEPPIGQLRFAKPVSKSTWKETYDADELPPSCMQVYIGDFYFTKALEGGEKMSEDCLYLNLWVPETKSDLKPILLFIYGGGFMGGTINMKIFDGANLSEQGDVIVATINYRVGSLGFFYGMTEDAAGSMGMYDQILAIQWIKDNAKHFGGDPDNIVLFGESA**	ATCACTTCCAACCAATCTGAGTGGTTTGAAGATTACTAGTTTTCCTGACAAAATGAAGATTCTGTTTCTCCTTCTGATTTTACCTTTGGCCAGCAGCAGCAGTATTGTTGCTACAACCAGTGGACCAATGAGAGGCGTATCTTTAGCTCCCAAGAAAGTCGAGGCATTTTACGGCATCCCATACGCTGAACCGCCAATAGGACAACTGAGGTTTGCAAAGCCAGTCTCTAAGTCAACCTGGAAGGAAACCTATGACGCTGATGAGTTGCCACCTAGTTGCATGCAAGTGTATATCGGAGATTTCTATTTCACTAAAGCTCTCGAAGGCGGAGAGAAGATGAGCGAAGACTGCCTGTACCTTAACTTATGGGTACCTGAGACTAAAAGTGATTTGAAACCCATCCTGCTGTTCATCTATGGAGGGGGATTCATGGGTGGAACGATAAATATGAAAATTTTCGATGGTGCCAATCTATCGGAACAGGGTGATGTCATAGTGGCGACAATCAATTACAGAGTTGGTTCTCTTGGATTTTTCTACGGTATGACCGAAGACGCTGCCGGAAGCATGGGTATGTACGATCAAATATTAGCAATCCAGTGGATCAAAGATAATGCAAAACATTTCGGCGGTGATCCAGACAATATAGTACTGTTTGGTGAAAGTGCCG
PhdEnzCho 42	B2D0J5	1,30E-06	31,884	PF00135	8879,14	RYIHSVRVFLGIPYAEPPVRRRSGENLQFKKPLEKQRFGDIDASRYKPACPQHQKHFSKGQGINTTSEDCLYLNIF	GTAGATATATACATTCAGTACGAGTGTTTCTAGGAATTCCATATGCAGAACCTCCTGTTAGGAGACGAAGTGGAGAAAATCTACAATTTAAGAAACCACTTGAGAAACAAAGATTTGGAGATATTGATGCCTCTCGTTATAAACCAGCTTGTCCCCAGCATCAAAAGCATTTTTCAAAAGGGCAAGGAATAAATACTACTTCAGAAGATTGTTTGTATCTCAATATATTTT

PhdEnzCho 43	W4VSJ0	8,43E-12	28,758	PF00135	16743,19	PLSKILFQRVIMQSAS<br>PLYSLNDHRSQNLQ<br>LGQTLAEKMECAD<br>QNKTLNENPDDVIE<br>CLRSKDPKELVMTTF<br>SLVPVFPLITFFTVVG<br>DDFLPTDPHEAIME<br>GKFYDVDAMIGFTQ<br>DDGAIILANFFLQIV<br>GPFGELNPRINKTFG<br>ETL	ATGAGAGTCTCCCCAAACGTCTTATTAATGCGAGGATTTAAT<br>TCCCCAAAAGGGCCTACTATCTGAAGAAAAAAATTAGCCAA<br>TATGATTGCTCCATCATCTTGAGTAAATCCTATCATAGCATCA<br>ACATCGTAGAATTTGCCTTCCATGATAGCTTCATGTGGATCTG<br>TAGGTAGAAAATCATCCCCAACAACGGTGAAAAATGTTATC<br>AGTGGGAAAACAGGGACTAAGGAAAACGTGGTCATCACCA<br>ATTCTTTTGGATCCTTACTTCTAAGACACTCGATCACATCATC<br>AGGATTTTCGTTCAACGTCTTGTTTTGATCTGCACATTCCATCT<br>TCTCTGCCAGCGTCTGTCCCAACTGAAGATTTTGAGACCTAT<br>GATCGTTCAGAGAGTACAGTGGCGAAGCACTTTGCATTATTA<br>CTCTCTGGAACAGAATTTTGGAAAGAGGAG
PhdEnzCho 44	W4VSJ0	8,75E-15	45,333	PF00135	8857,07	LDTFLGIPYAKPPIGK<br>YRFRHPKPIDPWKD<br>VFNATSNPNSCVQI<br>NDTQFGEFKGSTMW<br>NANSPMSEDCLTVS<br>VWVPRP	TCTTGGACACGTTCCTCGGGATACCATACGCGAAGCCACCGA<br>TAGGGAAATACAGATTCCGTCACCCAAAGCCTATAGATCCTT<br>GGAAGGACGTGTTCAATGCGACTAGTAACCCGAATTCCTGTG<br>TGCAGATCAATGACACGCAGTTCGGCGAATTCAAAGGGTCC<br>ACCATGTGGAACGCTAACAGTCCCATGAGCGAAGATTGTCT<br>AACGGTCAGCGTGTGGGTACCCAGGCCGAG
PhdEnzCho 45	W4VSJ0	2,10E-45	52,448	PF00135	15888,12	LEHWDGILDATIKPS<br>PCMQYSSRNFSWIPK<br>STPSEDCLFLNVWTP<br>SKCCCSGPEKLPVLV<br>WIYGGGFYSGSTDM<br>DVYDGSTLVSYSKAI<br>VVSINYRVGIFGFLN<br>SGTEEAPGNVGLMD<br>QNLALKWIKKNIGY<br>FGGDPGRITIVGE	TCTTGAACACTGGGACGGAATACTTGATGCCACGATAAAGC<br>CTTCCCCTTGCATGCAGTATTCAAGTCGGAACTTCAGCTGGA<br>TTCCCAAGTCAACCCCGTCTGAAGATTGCTTGTTTTTAAATGT<br>CTGGACTCCCTCTAAATGTTGTTGCAGTGGTCCAGAAAAACT<br>TCCCGTTTTGGTGTGGATTTATGGAGGCGGCTTCTATTCTGGA<br>TCCACAGATATGGATGTTTATGATGGAAGCACCCTTGTATCTT<br>ACAGCAAAGCAATTGTTGTAAGTATCAATTACAGAGTTGGCA<br>TATTTGGATTTTTGAACAGCGGAACAGAAGAAGCGCCGGGT<br>AACGTGGGACTTATGGATCAGAATTTAGCACTGAAGTGGATT<br>AAAAAGAACATAGGTTACTTTGGAGGAGATCCTGGCCGTATT<br>ACAATTGTGGGTGAG

PhdEnzCho46	W4VSJ0	1,29E-11	30,612	PF00135	11123,17	FGEKNNGTYRYYFNHRPTNTPYAQWMGVVHCEDMQFIFGRPLDSESNYTQAEKELSYQMATEWTNFAISGEPSKPWQTFTRSDPYIEVFETPES	ATTTGGCGAAAAAAATAATGGCACCTACAGATATTATTTCAATCACAGGCCAACGAATACTCCGTATGCACAATGGATGGGAGTTGTGCACTGTGAAGACATGCAGTTTATCTTTGGACGCCCTTTAGATTCTGAGTCCAACTACACTCAAGCGGAAAAAGAACTAAGCTACCAGATGGCTACAGAGTGGACAAATTTTGCTATTAGTGGGGAACCTAGTAAACCGTGGCAGACGTTCACGAGATCGGATCCATATATCGAAGTTTTTGAAACACCTGAAAGCG
				Metalloproteinases			
**Transcript ID**	**Uniprot ID reference protein**	**e-value**	**Identity (%)**	**PFAM domain**	**MW (Da)**	**Amino acid sequence**	**Nucleotide sequence**
PhdEnzMtP01	O93518	8,79E-34	46,923	PF08516<br>PF00200	18378,56	QCPDDKCIMAPSSSSTSPHHWSSCSLEYLDLAYSQGMDYCLKNHPVNIIGPVCGNGFLENGEECDCGLKEFCDNQCCNATSCQLFSNATCAMGGCCDLETCQVKKIATLCRDTVSECDLPEYCDGMSEFCPADTFQQNGKECGNGKAYCYDGHCQSHMDQCKLLWGQT	TCAATGTCCAGATGACAAGTGCATTATGGCTCCATCCTCTAGCTCTACAAGTCCTCATCACTGGTCTTCATGTTCTTTAGAGTATTTAGATCTTGCATATTCACAAGGAATGGATTACTGCCTAAAAAATCATCCTGTTAATATTATTGGTCCAGTGTGTGGTAATGGCTTTCTTGAAAATGGAGAAGAATGTGATTGTGGACTGAAAGAATTTTGTGACAATCAGTGCTGTAATGCCACTTCTTGCCAACTATTTTCCAATGCAACATGTGCAATGGGAGGATGCTGTGATCTTGAAACTTGCCAGGTGAAAAAGATAGCAACACTTTGTCGAGATACTGTGTCTGAATGTGATCTTCCAGAGTATTGTGACGGTATGTCTGAATTTTGTCCAGCTGATACTTTCCAACAAAATGGAAAAGAATGTGGTAATGGCAAAGCCTATTGTTATGATGGTCACTGTCAGTCTCATATGGACCAGTGTAAATTATTATGGGGACAGACTGG

PhdEnzMtP 02	P82942	9,02E-11	50,000	PF08516<br>PF17771<br>PF01421	7083,07	MARVGMAVPDSICTAKAVGVSEDSSIYEPHLIASTMTHMLGHNIGMSHDQNGTDCICDDWWGCIMA	TGGGGATGCATCATGGCCCGAGTTGGTATGGCAGTTCCTGATAGTATATGTACAGCAAAAGCAGTTGGAGTGAGTGAAGATTCTAGCATATATGAACCCCATCTCATAGCAAGTACAATGACCCACATGTTGGGACATAACATCGGAATGAGTCATGATCAAAATGGGACGGATTGTATATGCGATGATTGGTGGGGATGCATCATGGCCCG
PhdEnzMtP 03	Q9IAX6	1,91E-08	29,762	PF00200<br>PF01562<br>PF01421	10011.45	LKSIKVQNEEKLTVTFLAYNKEFILDLWMNSLLLPTHYFEKHHENGNHVVHSPMKENPSHCHYQGQIRGVNPSLVAVSTCNGLSGIIN	ATTAATAATACCACTTAATCCATTACAAGTACTAACTGCAACTAAGGAAGGATTTACTCCCCGTATTTGTCCTTGATAATGACAATGACTAGGATTTTCTTTCATAGGTGAATGCACAACATGATTTCCATTTTCATGATGTTTTTCAAAATAATGGGTGGGTAATAATAATGAATTCATCCACAAATCAAGAATAAATTCTTTGTTATAAGCTAAAAAAGTTACAGTCAACTTTTCTTCATTTTGTACCTTAATCGATTTAAG
PhdEnzMtP 04	Q5XUW8	2,39E-11	30,588	PF00200<br>PF01562<br>PF01421	10372.73	LDQAMFDNRNASRNEVVNDAIQIVNCVDMYFRTVNTRVSVVYVETWAHGDQMEINSDVRQTLLTFMEYASRKLYKVAKDATHLLTGHRF	AAACCTATGACCTGTCAATAGATGAGTGGCATCTTTTGCAACTTTGTACAATTTGCGTGAAGCATATTCCATAAAAGTGAGCAATGTTTGCCTAACATCACTATTAATTTCCATTTGATCTCCATGAGCCCAAGTTTCAACATATACCACAGAGACTCGAGTATTCACAGTACGAAAATACATATCAACACAGTTGACAATTTGGATGGCATCATTGACAACCTCATTCCTACTGGCATTGCGGTTATCAAACATAGCTTGGTCAAG
PhdEnzMtP 05	Q14FJ4	1,20E-08	31,507	PF01562<br>PF01421	9246,23	ISTQEIEHCYYHGTSKDYPGAIAAFRTCNGISGILHVGNDTFVIHPFYGGDQSKTHPHVIYRYFSETKEKHTCGNTNMHEW	AATTTCTACTCAGGAAATAGAACATTGTTATTATCATGGAACCTCAAAAGATTATCCTGGAGCCATTGCTGCCTTTCGCACATGTAATGGAATTAGTGGCATTCTTCATGTTGGAAATGATACATTTGTAATTCATCCATTTTATGGGGGAGATCAATCTAAAACACATCCACATGTTATTTATCGCTATTTTTCTGAAACCAAAGAAAAGCACACTTGTGGTAATACCAATATGCACGAATGGGG

PhdEnzMtP 06	Q9PW36	1,31E-10	37,000	PF01562<br>PF01421	13734,2	DDRDPSHYDTAILITREDLCRYPGACDTLGLAQSGMVCDSYSSCAIVEDNGLSAAFTIAHELGHVMSIPHDDDNKCARYHNEKKNLHVMARMLDYNSNPWSWSDCSREYLTTFFDVGYGNCL	ATTAAACAGTTGCCATAACCAACATCAAAAAATGTTGTTAAATATTCTCTGCTGCAATCTGACCAACTCCAAGGGTTGCTATTATAATCTAACATTCTAGCCATAACATGTAGGTTCTTCTTTTCATTATGATACCTTGCACACTTGTTATCATCATCATGAGGTATGCTCATTACATGTCCCAGCTCGTGTGCAATAGTAAATGCTGCACTGAGACCATTGTCTTCAACAATTGCACAAGAAGAATAAGAATCGCATACCATTCCAGACTGAGCCAAACCTAAAGTATCACATGCCCCAGGATATCGACAGAGATCTTCCCTTGTTATAAGGATGGCTGTATCATAGTGTGATGGGTCACGGTCATCA
PhdEnzMtP 07	Q10749	5,49E-46	44,444	PF08516<br>PF00200<br>PF01562<br>PF01421	25087,18	CIMAQTILGVNKIQPYHFSTCSLQDYINALRIGHGICLFNKPNQLEDFRSCGNSIVENGEECDCGSIDECTQTDPCCDPITCKLRVEAECSMGPCCADCKLRGPGHMCRLAVTECDIPEYCDGRDGQCPTDLFKKNGTPCKDGRGYCFQANCPTPDDQCEYLWGYGAVQSEYECFEQFNTQGSLNGNCGPDNQGGFIKCTEDNVRCGSLQCQRGSRAPMIAGKDKQYT	GATGCATCATGGCCCAAACCATCTTAGGAGTGAACAAAATCCAACCATATCATTTTTCAACTTGTAGTTTACAAGATTACATTAATGCACTCCGAATTGGCCATGGCATTTGTTTATTTAATAAACCAAATCAGCTGGAAGACTTTCGATCGTGTGGCAATAGTATTGTTGAAAATGGGGAAGAATGTGACTGTGGAAGTATAGATGAATGCACTCAAACAGATCCTTGTTGTGACCCCATTACTTGTAAGCTAAGAGTGGAAGCAGAATGCTCAATGGGACCTTGTTGTGCTGACTGCAAACTTCGAGGTCCCGGTCATATGTGTCGTCTTGCAGTCACAGAATGTGATATTCCTGAATACTGTGATGGAAGAGATGGTCAGTGTCCTACTGATTTGTTTAAAAAGAATGGTACCCCCTGCAAGGATGGCCGAGGTTACTGTTTTCAAGCCAATTGTCCAACACCTGATGATCAGTGTGAATATTTATGGGGTTATGGTGCTGTACAGTCGGAGTATGAGTGCTTTGAGCAGTTCAACACCCAGGGCAGTCTCAATGGAAATTGTGGGCCCGATAATCAGGGTGGTTTTATAAAATGTACTGAAGACAATGTAAGATGTGGTTCTCTTCAGTGCCAACGAGGCTCCCGTGCTCCGATGATAGCTGGAAAAGATAAACAATATACAC

PhdEnzMtP 08	F8RKW0	1,95E-22	24,816	PF08516<br>PF00200<br>PF01562<br>PF01421	61556.87<br>**59318.16**	MLWNVPICCFIGIFT<br>QVSQG**HPTIHSFAA**<br>**KPLEWDKSHSELVY**<br>**PQLHSPDSPGIKSSS**<br>**PNNHSILVLRMSLE**<br>**TFYIELKPNHGLLK**<br>**ANVSHGEDASKPC**<br>**HYHGNVLSHDDG**<br>**VAAISMCESKNRM**<br>**SGTIIVRNDAFVLR**<br>**PLDHLEDAESRLKF**<br>**QNSSKIGGVDPHVI**<br>**SKVDDTVTPFCGV**<br>**DSSGRFPNPEENEIS**<br>**SHRRFRRSSGAEPD**<br>**QHKGPKVIETAIYV**<br>**DYPLYRKFIFQRRKS**<br>**RSELRDTILTIINQV**<br>**QVIYNYKSLNQKFK**<br>**IVVVKLEYLTEGDD**<br>**VPDPVGGNIDEYLE**<br>**TFCAWQAPRNPPES**<br>**SENHWDHAIMLTG**<br>**YDLYKEINGKKNYK**<br>**VLGLAWVNGMCR**<br>**PKHSCTIEEGSSFEG**<br>**SFVIAHEMGHSLG**<br>**MMHDGVGNECDP**	GATTCCTCCCCCCCCTTTTGTTTTGGTCGGGCGGCGGATTAGT<br>ATTCAAAAAGTTCTTTAGCACGTTTTCGGAATAAATCACGGC<br>CAGGTTTCGATTCTCGAGCATACCGAGAAGATGCTATGGAAC<br>GTACCGATCTGTTGCTTTATTGGAATTTTTACTCAAGTATCAC<br>AGGGTCATCCGACAATCCACTCATTTGCCGCAAAACCACTCG<br>AATGGGACAAAAGTCACTCCGAACTAGTATATCCTCAGCTGC<br>ACTCTCCTGACAGTCCGGGAATAAAATCATCCAGTCCAAAC<br>AACCATTCCATCCTTGTGCTGCGAATGTCGCTGGAGACATTC<br>TACATCGAGCTCAAGCCAAATCACGGTCTCCTCAAGGCTAAC<br>GTGTCTCACGGTGAAGATGCCTCGAAACCCTGCCATTACCAC<br>GGAAACGTCTTGTCTCACGATGATGGCGTGGCTGCAATTTCT<br>ATGTGCGAATCCAAAAACAGAATGAGCGGTACCATCATAGT<br>TCGGAACGATGCTTTCGTCTTAAGGCCTTTGGATCATCTGGA<br>GGACGCGGAAAGTCGCTTGAAGTTCCAAAATTCTTCCAAGAT<br>CGGAGGAGTCGACCCCCACGTCATTAGTAAAGTGGACGACA<br>CTGTAACTCCTTTCTGCGGCGTGGATAGTTCTGGAAGATTTCC<br>CAATCCGGAAGAAAATGAAATTTCGTCTCATCGCCGATTCCG<br>CAGGTCCAGCGGCGCAGAACCTGATCAACACAAAGGGCCCA<br>AAGTAATCGAAACAGCAATCTACGTGGACTATCCGCTGTATC<br>GAAAGTTCATCTTCCAGAGGAGAAAGTCACGTTCAGAACTG<br>AGAGACACGATTCTCACAATTATAAACCAGGTGCAGGTAAT<br>CTACAATTACAAGTCACTGAACCAGAAATTCAAGATTGTTGT<br>CGTCAAACTCGAATATTTAACGGAAGGCGACGACGTTCCTG<br>ATCCTGTCGGTGGAAATATCGACGAATATTTAGAAACATTTT<br>GTGCGTGGCAGGCGCCTAGAAATCCGCCAGAATCATCAGAG<br>AATCACTGGGACCATGCTATCATGCTGACTGGTTATGATCTTT<br>ATAAAGAAATCAACGGCAAGAAAAACTACAAAGTGTTAGG<br>CCTGGCATGGGTTAACGGAATGTGCCGGCCCAAACACAGCT

						**TAYIMAEKTGPGRI TWSTCSRDYLQKFF QKGHGHCLDDEDP NSLKNSQYKFDGQ LPGHLFDLDAQCKL SLGRDYRPHVLPKV PFNNVCRQLWCISG LWASVAHPALEGSS CGGGRHCIQGKCK GERPNTPFLDESSLV EVQKMLVDFLDRL KMAFQLLLP***	GTACCATTGAGGAAGGGTCTAGCTTTGAAGGATCGTTTGTCA TCGCTCATGAAATGGGACACAGTTTAGGAATGATGCACGAT GGAGTGGGCAATGAATGCGATCCGACGGCTTACATAATGGC TGAAAAGACGGGACCGGGTCGAATTACGTGGTCTACATGCA GTAGAGACTATCTACAAAAGTTCTTTCAGAAAGGCCATGGTC ACTGTCTGGACGATGAGGATCCTAATTCACTAAAAAACAGC CAGTACAAATTCGATGGTCAACTTCCAGGACATCTTTTCGAC CTGGATGCTCAGTGCAAGCTGTCACTCGGCAGAGATTATCGA CCTCACGTCTTGCCCAAAGTTCCCTTCAACAATGTGTGCCGG CAACTGTGGTGCATCTCTGGACTCTGGGCATCCGTGGCTCAC CCAGCGCTGGAAGGAAGCTCCTGCGGTGGTGGAAGACACTG CATACAAGGCAAATGCAAGGGCGAACGACCCAACACTCCTT TTCTCGACGAAAGCTCTCTGGTGGAAGTACAAAAGATGTTGG TGGATTTCTTGGACAGGCTCAAAATGGCATTTCAATTGTTACT TCCTTGATATGCTAGCTGTGTTAGATTACTTATACGTTAATAA AATTATTTTTGTTCATTGTAAAAAAA

PhdEnzMtP 09	Q8JIR2	2,36E-90	30,707	PF08516<br>PF00200<br>PF01562<br>PF01421	83900.28<br>**81479.41**	MPHPPSLSARTLAV<br>ACALFAVAQS**ARD**<br>**AADPGLRSLLLNEQ**<br>**NQELIDNMGTRYE**<br>**VVHPFQIRKDWSR**<br>**GLSTRLSTVNGTTL**<br>**HVQETTLLIETFEYK**<br>**LQLDLELNTKLFAP**<br>**TLVQLLYLKDSTPIA**<br>**AHKLPENCYYHAK**<br>**VRNYPEAKAAFQT**<br>**CNGIRGIIYLAEKIFF**<br>**IHPLHGNHSGKHP**<br>**HLLYHYLSEEDFHC**<br>**GSTGALNVDHSED**<br>**GIRYTEKDDWHRN**<br>**KYIEMAVVLDQSLF**<br>**QKFKSPPSEVLSSA**<br>**VEIINHVDLLYRPLN**<br>**TSVSLVYLELWIED**<br>**QMPVSSNNMTATL**<br>**KQFKEYAVRRISRIS**<br>**VDATHLLTGVEFEN**<br>**KVKGLAFLDSICTN**<br>**NAVGLSRVHNIYQ**<br>**PHITAFILAHMLGH**<br>**NLGMSHDHSGCD**<br>**CPDVSGCVMEND**	TTTTTTAATATAAAAATTATTTTAATACAAAAATATATTTATA<br>CAAAATGATTCTCTACTTTAAAATCGTGCTTATTCTGTCCATT<br>CTTCATGTATGCCTGTAGAAAAAGAGCAGAATGACTATGAG<br>CACCAGTCCGACACCGATGACTATGGAAGCGGATATTACCG<br>TGATGGCCAGGTTCGGGTTCAGTCTCATCTCGTTCTGACCGTA<br>ATGGTGCGGATTTTCAGAGACTGGTCCAGAAGGGTCATAGTA<br>CTCGTCACCCACTCGCCATTCTTCATCTACCGTGCTGCAGTCC<br>GGCCCCTTCCAGCCCTCGTCGCAGAAGCACGTGTTGGCGTTG<br>GTACAAATCCCATGACCCGAGCAGGTATTCTGGAAGTCTTCC<br>ATAGGACACTTGCCGGATACAAGACTTTTTAGACTGGTGCAG<br>GTCTGGTTGAGGCACAGCTTTTGGTATCCGCACTTGGTTCCGT<br>CTCTCACAAGCCCCTCCCTGAGATCGGACGTGGAGAATGTCC<br>TGGCTTTGCATTCGTGCACGGTCCCTTGCGCATTGATCCGGTG<br>AACCACGAAGTGGACGGGGGCATCGTCCAGGTATACGGGCG<br>TCTTCTCACCCGAGCTGCACTGCAGGAAGCCGCAGTAAGAGT<br>TCTCTGCGGAGCATTTGGCGATTCCACCGCTTCCGTCCGTTCC<br>ACAATTACCGTTGGGAGTGCCCTGGACGTTCAGACGCTCGAA<br>GCACACCGAATCCGCGGCATCGGCCTCTGTTCCCCAGATGTC<br>CTGGCACTGGTGCTTCAAAACTGGGCATTCTCCCTGAAAGCA<br>GTATCCCAGGCCACCCGAACAAACTGCCCCGTTCCGCTTGAA<br>CACGTCCTCCGGGCACTGTCCATTTCTTCCGTCACAGAACTCC<br>GGGATGTCACATTCTCCTTTGGACGGCTTGCATAGGTGGTCTG<br>GCGACAGGAGCTTGCACTTCCTGCAGCAAGGTCCAGAGGAA<br>CACTGCGCATGCTTGATAAGTCTGCAGGTAAAGGGGTCGCA<br>GCACGGATTGGATCTCAAGCATTCCTCTACCGTACCGCAGTC<br>GCACTGTTCTGGTTCTTCGAGAATGCTGTTTCCGCATATCGAC<br>ATCCTCATGGTAGGCATATTATAGAGACAGGCTCCGTATCCC<br>TTGTCCAACGTTCGGTGGTACTGCAAGACGCTGCACTGGGAG

						**MPVLASTFFSQCSV<br>LQYHRTLDKGYGA<br>CLYNMPTMRMSIC<br>GNSILEEPEQCDCG<br>TVEECLRSNPCCDP<br>FTCRLIKHAQCSSG<br>PCCRKCKLLSPDHL<br>CKPSKGECDIPEFC<br>DGRNGQCPEDVFK<br>RNGAVCSGGLGYC<br>FQGECPVLKHQCQ<br>DIWGTEADAADSV<br>CFERLNVQGTPNG<br>NCGTDGSGGIAKC<br>SAENSYCGFLQCSS<br>GEKTPVYLDDAPV<br>HFVVHRINAQGTV<br>HECKARTFSTSDLR<br>EGLVRDGTKCGYQ<br>KLCLNQTCTSLKSL<br>VSGKCPMEDFQNT<br>CSGHGICTNANTCF<br>CDEGWKGPDCSTV<br>DEEWRVGDEYYDP<br>SGPVSENPHHYGQ<br>NEMRLNPNLAITVI<br>SASIVIGVGLVLIVI<br>LLFFYRHT***	AAAAAAGTCGATGCGAGGACTGGCATGTCGTTCTCCATAAC<br>GCAACCACTCACGTCTGGACAGTCACATCCTGAGTGGTCGTG<br>ACTCATTCCTAGGTTGTGTCCTAACATGTGAGCCAGAATGAA<br>GGCGGTGATGTGGGGCTGATAGATGTTGTGAACCCGGCTGAG<br>CCCGACTGCGTTATTAGTGCAGATGCTGTCGAGGAAAGCAA<br>GCCCCTTGACTTTGTTTTCGAACTCGACACCTGTGAGTAGATG<br>TGTAGCGTCCACCGATATCCTGCTGATCCGACGCACCGCGTA<br>CTCCTTGAATTGTTTTAGAGTGGCAGTCATGTTGTTACTAGAG<br>ACTGGCATCTGGTCCTCTATCCACAGCTCTAGGTACACCAGG<br>CTCACAGAGGTGTTGAGAGGACGGTACAGCAGGTCGACATG<br>GTTGATGATTTCGACGGCTGAAGACAGCACCTCGGACGGAG<br>GGGACTTGAACTTCTGGAACAAAGACTGATCCAACACAACT<br>GCCATTTCGATATACTTGTTTCTGTGCCAGTCATCTTTCTCCGT<br>ATAACGTATTCCATCCTCCGAATGATCGACGTTTAAGGCTCC<br>TGTGCTTCCACAGTGGAAGTCTTCTTCGGAGAGATAGTGGTA<br>GAGGAGGTGAGGATGTTTACCCGAATGATTTCCGTGCAAGG<br>GATGTATGAAAAAGATTTTCTCTGCCAGGTATATAATCCCTCT<br>GATGCCATTGCACGTCTGGAAGGCAGCTTTAGCTTCGGGGTA<br>GTTCCGCACCTTAGCGTGATAATAGCAGTTCTCAGGAAGTTT<br>GTGTGCTGCGATCGGAGTTGAGTCTTTTAAGTACAAGAGTTG<br>CACTAGGGTGGGGGCGAATAGCTTCGTGTTGAGTTCCAGGTC<br>CAATTGCAGTTTGTATTCGAAGGTCTCGATGAGGAGAGTCGT<br>TTCTTGCACGTGAAGAGTCGTTCCGTTTACAGTGGAAAGGCG<br>AGTGGAGAGGCCTCGACTCCAGTCCTTCCGAATCTGGAAAG<br>GGTGGACCACCTCGTAGCGGGTACCCATGTTGTCGATAAGCT<br>CCTGGTTCTGTTCGTTTAGGAGAAGTGAGCGCAGACCTGGGT<br>CTGCGGCATCCCTCGCCGATTGCGCTACCGCGAAGAGCGCGC

							AGGCGACCGCCAGCGTCCGAGCCGAGAGCGACGGAGGATGT GGCATGGCTGTGTGTATGTCAGCAAACGCACGCG

PhdEnzMtP 10	A8QL49	9,89E-22	30,303	PF08516<br>PF00200<br>PF01562<br>PF01421	57495,27	RDFDQDGRPDNVSF<br>LIKRIKVHTLDALKD<br>PVYRFPGNYGVEKFL<br>ELFSEEDYDAFCLAY<br>MFTYRDFEGGTLGL<br>AWTGDLKNAGGVC<br>EKNGHYRGSLKSLN<br>TGIVTLLNYGKYVPP<br>IVSHVTLAHEIGHNF<br>GSPHDPEDDVVCTP<br>GGDNGNYIMFARAT<br>SGDKKNNNKFSPCS<br>LRSINAVLNTKARTL<br>KGCFTEIQDSICGNG<br>VVEKSEQCDCGWEE<br>DCEESCCFPMRTNPP<br>RDEPPCKLRPNVICS<br>PSQGPCCSQDCSLKI<br>GEECRGDNGCRSPS<br>YCDGKGPQCPPSTN<br>KPNKTVCNEEFVCY<br>MGECTGSICVAYGL<br>ESCQCRRGPHDPAT<br>KACELCCKLPGDDY<br>SCKSSFDWNFSPYDV<br>PDLFAKPGTPCDNY<br>NGYCDVFQKCREVD<br>PSGPLATLRKLLLSN	TCTACTTCGACTTATACCTTGAGTCAAATCATGTATCAGTAAA<br>GAGATTTTGATCAAGATGGACGTCCAGATAATGTCTCTTTCCT<br>AATCAAGCGAATCAAAGTCCATACCTTAGATGCACTGAAAG<br>ATCCTGTGTATAGATTTCCTGGAAACTATGGTGTTGAAAAATT<br>TTTGGAGTTATTTTCAGAAGAAGATTATGATGCTTTCTGTTTA<br>GCTTATATGTTTACCTACAGAGATTTCGAAGGTGGCACTTTGG<br>GTTTAGCTTGGACGGGTGACTTAAAGAATGCTGGTGGTGTAT<br>GTGAAAAAAATGGGCATTATAGAGGCAGCTTGAAGAGTTTA<br>AATACTGGCATAGTTACGCTTTTGAATTACGGAAAGTATGTT<br>CCACCCATTGTGTCTCATGTTACATTAGCTCATGAAATTGGCC<br>ATAACTTTGGATCTCCTCACGATCCAGAGGATGATGTAGTGT<br>GTACACCTGGTGGAGACAATGGTAACTACATAATGTTTGCTC<br>GGGCAACATCTGGTGATAAGAAAAATAACAACAAATTTTCC<br>CCCTGTAGTTTACGTAGTATAAATGCCGTACTTAATACTAAG<br>GCCAGAACCTTAAAAGGTTGTTTCACAGAAATACAAGATTCT<br>ATTTGTGGAAATGGTGTAGTAGAAAAAAGTGAACAGTGTGA<br>CTGTGGCTGGGAGGAGGATTGTGAAGAGAGCTGTTGTTTTCC<br>AATGAGAACCAATCCTCCACGAGATGAACCTCCTTGTAAACT<br>CAGGCCGAATGTGATTTGCAGCCCTAGCCAAGGTCCATGCTG<br>CTCACAGGACTGCAGTCTGAAAATTGGTGAAGAATGTCGAG<br>GGGATAACGGATGTAGGAGTCCTAGCTACTGCGATGGAAAA<br>GGCCCTCAGTGTCCTCCATCCACAAACAAGCCAAATAAAAC<br>TGTGTGTAATGAAGAATTTGTGTGTTATATGGGTGAATGTACT<br>GGCTCTATATGTGTTGCTTATGGTTTAGAATCGTGTCAGTGTC<br>GCCGTGGACCGCATGATCCTGCAACAAAAGCTTGTGAATTAT<br>GCTGCAAATTGCCAGGGGATGATTACAGTTGCAAGTCATCAT<br>TTGATTGGAATTTTTCTCCATATGATGTTCCTGACCTTTTTGCC<br>AAACCTGGAACACCATGTGACAACTATAATGGATACTGTGA

						ESMASVKKWILLHWWVAVLLGLGIVMCMSVIVRLFGKQNETLKTVPENGQMNKTYGILRPCSNQPLVKARTQALATNSSIALAHATNHQKSTSNFVRTSVSSFSHKHKPLLMTGNGWV*	TGTTTTTCAGAAATGCAGAGAAAATTTTCAGGTTGATCCTTCAGGTCCTCTTGCTACACTTCGAAAACTTCTTTTATCAAATGAAAGTATGGCATCTGTCAAGAAATGGATTCTTTTGCATTGGTGGGTAGCTGTCCTTCTAGGCTTAGGGATTGTTATGTGTATGAGTGTTATTGTGCGGTTATTTGGAAAACAAAATGAAACTCTGAAAACAGTGCCTGAAAATGGCCAAATGAACAAAACTTATGGTATATTGCGTCCTTGCAGTAATCAACCTTTGGTCAAAGCCCGTACACAGGCACTCGCGACCAATTCAAGTATTGCCTTGGCTCATGCTACTAATCATCAGAAATCTACTAGCAATTTTGTCAGGACCAGTGTATCATCTTTCTCTCATAAACATAAACCACTTCTTATGACTGGGAATGGATGGGTATGACAGTAATTGTAATAAGTTGTCTGTCAAATGTATATATATGTGAAAGCCTGATCAGTTATATATTTTTTAACTTATATACACACATTTTCTTTTCTTGAACAGAGCCTAAGGTAAGGTTTGTTTTTATGCAGCTTGCATGCTTATATCTTTTATAATAAACTGAGTGACATATATTTTAAACTGTG

PhdEnzMtP 11	A8QL49	9,76E-22	30,303	PF08516<br>PF00200<br>PF01562<br>PF01421	57884,68	RDFDQDGRPDNVSF<br>LIKRIKVHTLDALKD<br>PVYRFPGNYGVEKFL<br>ELFSEEDYDAFCLAY<br>MFTYRDFEGGTLGL<br>AWTGDLKNAGGVC<br>EKNGHYRGSLKSLN<br>TGIVTLLNYGKYVPP<br>IVSHVTLAHEIGHNF<br>GSPHDPEDDVVCTP<br>GGDNGNYIMFARAT<br>SGDKKNNNKFSPCS<br>LRSINAVLNTKARTL<br>KGCFTEIQDSICGNG<br>VVEKSEQCDCGWEE<br>DCEESCCFPMRTNPP<br>RDEPPCKLRPNVICS<br>PSQGPCCSQDCSLKI<br>GEECRGDNGCRSPS<br>YCDGKGPQCPPSTN<br>KPNKTVCNEEFVCY<br>MGECTGSICVAYGL<br>ESCQCRRGPHDPAT<br>KACELCCKLPGDDY<br>SCKSSFDWNFSPYDV<br>PDLFAKPGTPCDNY<br>NGYCDVFQKCRENF<br>QVDPSGPLATLRKLL	TCTACTTCGACTTATACCTTGAGTCAAATCATGTATCAGTAAA<br>GAGATTTTGATCAAGATGGACGTCCAGATAATGTCTCTTTCCT<br>AATCAAGCGAATCAAAGTCCATACCTTAGATGCACTGAAAG<br>ATCCTGTGTATAGATTTCCTGGAAACTATGGTGTTGAAAAATT<br>TTTGGAGTTATTTTCAGAAGAAGATTATGATGCTTTCTGTTTA<br>GCTTATATGTTTACCTACAGAGATTTCGAAGGTGGCACTTTGG<br>GTTTAGCTTGGACGGGTGACTTAAAGAATGCTGGTGGTGTAT<br>GTGAAAAAAATGGGCATTATAGAGGCAGCTTGAAGAGTTTA<br>AATACTGGCATAGTTACGCTTTTGAATTACGGAAAGTATGTT<br>CCACCCATTGTGTCTCATGTTACATTAGCTCATGAAATTGGCC<br>ATAACTTTGGATCTCCTCACGATCCAGAGGATGATGTAGTGT<br>GTACACCTGGTGGAGACAATGGTAACTACATAATGTTTGCTC<br>GGGCAACATCTGGTGATAAGAAAAATAACAACAAATTTTCC<br>CCCTGTAGTTTACGTAGTATAAATGCCGTACTTAATACTAAG<br>GCCAGAACCTTAAAAGGTTGTTTCACAGAAATACAAGATTCT<br>ATTTGTGGAAATGGTGTAGTAGAAAAAAGTGAACAGTGTGA<br>CTGTGGCTGGGAGGAGGATTGTGAAGAGAGCTGTTGTTTTCC<br>AATGAGAACCAATCCTCCACGAGATGAACCTCCTTGTAAACT<br>CAGGCCGAATGTGATTTGCAGCCCTAGCCAAGGTCCATGCTG<br>CTCACAGGACTGCAGTCTGAAAATTGGTGAAGAATGTCGAG<br>GGGATAACGGATGTAGGAGTCCTAGCTACTGCGATGGAAAA<br>GGCCCTCAGTGTCCTCCATCCACAAACAAGCCAAATAAAAC<br>TGTGTGTAATGAAGAATTTGTGTGTTATATGGGTGAATGTACT<br>GGCTCTATATGTGTTGCTTATGGTTTAGAATCGTGTCAGTGTC<br>GCCGTGGACCGCATGATCCTGCAACAAAAGCTTGTGAATTAT<br>GCTGCAAATTGCCAGGGGATGATTACAGTTGCAAGTCATCAT<br>TTGATTGGAATTTTTCTCCATATGATGTTCCTGACCTTTTTGCC<br>AAACCTGGAACACCATGTGACAACTATAATGGATACTGTGA

						LSNESMASVKKWILL HWWVAVLLGLGIV MCMSVIVRLFGKQN ETLKTVPENGQMNK TYGILRPCSNQPLVK ARTQALATNSSIALA HATNHQKSTSNFVR TSVSSFSHKHKPLLM TGNGWV*	TGTTTTTCAGAAATGCAGAGAAAATTTTCAGGTTGATCCTTCA GGTCCTCTTGCTACACTTCGAAAACTTCTTTTATCAAATGAAA GTATGGCATCTGTCAAGAAATGGATTCTTTTGCATTGGTGGGT AGCTGTCCTTCTAGGCTTAGGGATTGTTATGTGTATGAGTGTT ATTGTGCGGTTATTTGGAAAACAAAATGAAACTCTGAAAAC AGTGCCTGAAAATGGCCAAATGAACAAAACTTATGGTATATT GCGTCCTTGCAGTAATCAACCTTTGGTCAAAGCCCGTACACA GGCACTCGCGACCAATTCAAGTATTGCCTTGGCTCATGCTAC TAATCATCAGAAATCTACTAGCAATTTTGTCAGGACCAGTGT ATCATCTTTCTCTCATAAACATAAACCACTTCTTATGACTGGG AATGGATGGGTATGACAGTAATTGTAATAAGTTGTCTGTCAA ATGTATATATATGTGAAAGCCTGATCAGTTATATATTTTTTAA CTTATATACACACATTTTCTTTTCTTGAACAGAGCCTAAGGTA AGGTTTGTTTTTATGCAGCTTGCATGCTTATATCTTTTATAATA AACTGAGTGACATATATTTTAAACTGTG

PhdEnzMtP 12	F8RKV9	3,13E-05	53,571	PF08516 PF00200 PF01562 PF01421	14813,62	CKVTSGTINEDIPDL GLVSDGTKCAEAKI CVNQTCVNVDVFIE PGKCPTNNVALVCS GHGVCSNINTCHCD QNWTSSDCSEPIKSG LDIVDHTNGPVGTIE KPGETGTFTTMKAIS ETVADWRNKTHKS NVPYGVGKKDV	AGGACATCCTTCTTGCCAACCCCATATGGTACATTTGATTTGT GAGTCTTATTTCTCCAGTCTGCAACAGTTTCGCTGATCGCTTT CATAGTGGTAAATGTACCTGTTTCTCCTGGTTTCTCTATAGTT CCAACAGGTCCGTTAGTGTGATCTACAATATCCAGCCCAGAC TTTATGGGTTCACTGCAATCTGAAGATGTCCAGTTTTGATCAC AGTGACAGGTATTAATATTGGAACAAACGCCATGTCCTGAGC ACACCAGGGCAACGTTGTTTGTTGGACATTTTCCAGGCTCGA TAAATACATCAACATTAACGCAGGTTTGATTGACACAGATCT TAGCCTCAGCACATTTAGTACCATCACTCACTAGGCCCAAGT CAGGAATGTCTTCATTGATTGTGCCAGAGGTTACTTTGCA
PhdEnzMtP 13	F8RKV9	3,24E-11	36,000	PF08516 PF00200 PF01562 PF01421	9038,19	VELIIVNDNKEFKEF EEDKNAVFERSKQIA NIVNGLYSPLNIYIAL VGVIVWSDHDEIKLS ADGDATLTNFLHYR RER	GTGGAGCTTATAATTGTGAATGATAACAAAGAGTTCAAAGA ATTTGAAGAAGACAAAAATGCTGTTTTTGAAAGAAGCAAGC AAATAGCAAACATTGTAAATGGGCTGTATTCTCCATTGAATA TTTACATCGCTTTAGTAGGTGTTATAGTGTGGTCAGACCACGA TGAAATCAAACTTTCTGCTGATGGGGATGCAACTTTGACTAA TTTTCTACATTACCGCAGAGAAAGG

PhdEnzMtP 14	A0FKN6	2,07E-58	48,259	PF01400	27903.77<br><br>**26222.75**	MFTAVGFLLLTAGSS<br>LA**TSRVYLGDLPIQ**<br>**NPDLFGGDILGVED**<br>**DEDRNAIVNKILLW**<br>**PGGIVPYEEDPGLK**<br>**ANVFKLTLQGAFD**<br>**QYKRDTCIKFVPRT**<br>**NEKDYIRLFSGEGC**<br>**YSHVGRTGGQQPV**<br>**SLGQGCGWMGTIV**<br>**HELGHALGFYHEQ**<br>**NRSDRDDWLIIFW**<br>**DNVKEGMEDQFFK**<br>**LKPHQNQLLTPFDY**<br>**SSIMLYGSYTFSKD**<br>**RKKLKTMVGKNNE**<br>**FLQEVISKYRLSKSD**<br>**IERVNTLYNCKM***	ATGCAATTGAAATACAGCTACAATGATGTCATGAATAAAAC<br>AAATTTCATTCCAGTTGATTTTATTTGCTCCAGATAAATATTC<br>TACGATGTTTACAGCAGTTGGTTTCCTCCTACTTACCGCAGGC<br>AGTAGCCTGGCAACATCGAGAGTTTATTTAGGAGATCTGCCG<br>ATACAAAATCCGGATCTATTTGGAGGAGATATTTTGGGAGTT<br>GAAGATGATGAAGATCGAAATGCTATAGTGAATAAAATACT<br>GCTTTGGCCTGGAGGAATCGTGCCATATGAAGAAGATCCTGG<br>TCTGAAAGCAAACGTCTTCAAGTTGACTCTTCAAGGGGCCTT<br>CGACCAGTACAAAAGAGATACGTGTATTAAATTCGTTCCCAG<br>AACTAACGAAAAGGACTACATCAGACTATTCTCTGGAGAGG<br>GATGCTATTCTCATGTGGGTCGAACTGGTGGGCAGCAACCTG<br>TATCACTTGGTCAGGGCTGTGGATGGATGGGAACCATTGTCC<br>ATGAACTGGGACATGCGCTTGGATTCTATCACGAACAGAAC<br>AGATCAGATAGGGATGATTGGTTGATCATTTTCTGGGATAAC<br>GTGAAAGAAGGTATGGAAGATCAGTTCTTCAAGCTGAAGCC<br>ACATCAAAATCAACTTTTGACTCCCTTTGATTACAGCTCCATA<br>ATGTTGTACGGCTCTTATACTTTCTCAAAGGATCGTAAGAAG<br>TTGAAGACGATGGTAGGCAAGAACAACGAGTTCCTCCAGGA<br>GGTGATTTCAAAATACCGACTCAGTAAGAGCGACATCGAGA<br>GAGTCAACACCCTGTACAACTGCAAGATGTGATAAATAAAT<br>TTCGGTAAATAAAAGGAACCTGAAAATGAAAAAAAA
PhdEnzMtP 15	A0FKN6	2,31E-37	45,098	PF01400	17174,43	RTCVRFTPRRYEQNY<br>IRIFAGQGCYSNVG<br>MIGGQQPVSLGQGC<br>MFKGTIVHELGHAI<br>GFFHEQNRSDRDQY<br>LTIYWQNIQQGMDS<br>QFFLLKPHENLLLTS	AGAACCTGCGTGCGATTCACCCCAAGGAGATACGAACAGAA<br>CTATATTAGGATATTTGCTGGTCAAGGATGCTATTCCAACGTT<br>GGTATGATTGGAGGACAACAACCAGTCTCCTTGGGACAGGG<br>CTGCATGTTCAAAGGAACTATCGTGCACGAACTGGGACACG<br>CCATCGGTTTCTTCCACGAGCAGAACAGGTCGGACCGTGACC<br>AGTACCTCACAATCTACTGGCAAAACATCCAACAAGGTATG<br>GATTCCCAGTTTTTCCTTCTGAAGCCTCACGAGAACTTGCTTT

						FDYNSIMLYGNTAFS<br>KDGRSNTMVAKTG<br>QRLLETYDKPGLSAS<br>DVQRVQK	TAACCAGCTTCGATTACAACTCCATTATGTTGTACGGCAACA<br>CTGCTTTCTCTAAGGATGGTCGATCCAACACCATGGTGGCTA<br>AGACAGGCCAACGTCTGTTGGAGACATACGATAAGCCAGGT<br>CTCAGTGCCAGCGACGTCCAAAGAGTCCAAAAA
PhdEnzMtP 16	A0FKN6	5,82E-37	45,098	PF01400	17158,43	RTCVRFTPRRYEQNY<br>IRIFAGQGCYSNVG<br>MIGGQQPVSLGQGC<br>MFKGTIVHELGHAI<br>GFFHEQNRSDRDQY<br>LTIYWQNIQQGMDT<br>QFFLLKPHENLLLTG<br>FDYNSIMLYGNTAFS<br>KDGRSNTMVAKTG<br>QRLLETYDKPGLSAS<br>DVQRVQK	AGAACCTGCGTGCGATTCACCCCAAGGAGATACGAACAGAA<br>CTATATTAGGATATTTGCTGGTCAAGGATGCTATTCCAACGTT<br>GGTATGATTGGAGGACAACAACCAGTCTCCTTGGGACAGGG<br>CTGCATGTTCAAAGGAACTATCGTGCACGAACTGGGACACG<br>CCATCGGTTTCTTCCACGAGCAGAACAGGTCGGACCGTGACC<br>AGTACCTCACAATCTACTGGCAAAACATCCAACAAGGCATG<br>GACACCCAATTCTTCCTTCTGAAACCTCACGAGAACTTGCTTT<br>TAACCGGCTTCGATTACAATTCCATTATGTTGTACGGCAACA<br>CTGCTTTCTCTAAGGATGGTCGATCCAACACCATGGTGGCTA<br>AGACAGGCCAACGTCTGTTGGAGACATACGATAAGCCAGGT<br>CTCAGTGCCAGCGACGTCCAAAGAGTCCAAAAA
PhdEnzMtP 17	A0FKN6	7,50E-12	32,941	PF01400	13269.22<br>**11785.39**	MYFVVFALLAVASA<br>**RDLRDNLSVDLGD**<br>**LPMQNPDLFGGDM**<br>**LGVEVVDRNVIPH**<br>**AHLRWTGKTVPYTI**<br>**DPAIAGYTSLINAAI**<br>**QDYHTRTCVRFTPR**<br>**KFESNYIRLFAGQG**<br>**CYSNVGMIGG**	TCCTCCAATCATACCAACGTTGGAGTAGCATCCTTGACCAGC<br>AAACAGCCTAATGTAGTTCGATTCGAATTTCCTTGGAGTGAA<br>TCGCACGCAGGTTCTTGTGTGATAGTCTTGTATGGCAGCATTG<br>ATGAGGCTCGTGTAACCAGCAATAGCAGGATCAATGGTGTA<br>TGGAACTGTCTTGCCGGTCCATCTTAGATGAGCATGGGGAAT<br>GACGTTTCGGTCCACAACTTCCACTCCCAGCATGTCTCCTCCG<br>AAGAGGTCGGGGTTCTGCATTGGCAAGTCTCCGAGATCGACC<br>GAGAGGTTATCTCTCAAGTCCCTGGCCGAAGCCACAGCGAG<br>AAGGGCAAACACTACGAAGTACATCTTGATTGTTCCACTGGA<br>GGAGACAATGGGCAGAATGTAATTTTCAGGAATCGCGCCCTT

							TTTATAATTCAAATCTTATCGAGGAGCATGGAAATTTTTTCACCTGTCGCTCCCAATCACGTTAGATAACTTGTTGCCG
PhdEnzMtP18	A0FKN6	1,92E-50	45,946	PF01400	21460,53	VVPYIQDPGITQTVAQAILTNAMWLYKRDTCIRFVPRTNEENYIRIFPGQGCYSHVGKTYGAQPVSLGQGCGYMGTVMHELAHALGFYHEQNRSDRDDWLTIFWDNIKEGMAPQFMKLKPDQNQLLTPFDYDSIMLYGSYTFSKEYGKLRTMEGKNGQFLKDVVRKYFMSKSDIQRIKTLYNCTN*	GCAGTGTACTTTATTAGTTATCAGACAACAAGATGTAAAACTTGTTTGAACACACTTTACGAGTCATCTATAAGTCATTATTGGCTATAAATCTCTACGTAAAGAGTTAAAAATTCCCCTACGAGAAAGGCATCCATCATTTATAGAGTTTCAGTTGGTGCAATTGTAGAGCGTTTTTATCCTCTGAATGTCACTCTTGCTCATGAAGTATTTGCGCACCACGTCTTTCAAAAATTGTCCGTTCTTTCCTTCCATGGTTCTCAGCTTGCCATATTCTTTCGAAAAAGTGTAAGACCCGTACAGCATGATTGAATCATAGTCGAAAGGAGTCAAAAGTTGGTTCTGGTCGGGCTTTAACTTCATGAACTGGGGCGCCATTCCTTCTTTGATATTATCCCAAAAAATTGTGAGCCAATCGTCTCTATCAGATCGGTTCTGTTCGTGGTAGAATCCGAGAGCATGTGCCAACTCATGCATTACCGTGCCCATATATCCGCAGCCTTGACCTAGAGAGACGGGCTGTGCGCCGTAGGTCTTGCCCACGTGAGAGTAACACCCTTGGCCTGGGAATATTCTGATGTAATTTTCTTCATTCGTCCTCGGAACGAAGCGGATGCAAGTGTCTCTTTTGTACAACCACATGGCATTCGTCAGAATGGCCTGGGCAACAGTTTGTGTTATTCCCGGATCTTGAATGTATGGTACCACA

PhdEnzMtP 19	A0FKN6	4,40E-38	48,889	PF01400	17928,39	KYRVNVNGISHFSD EEIFIVCYSHVGKTY GAQPVSLGQGCGY MGTVMHELAHALG FYHEQNRSDRDDWL TIFWDNIKEGMAPQ FMKLKPDQNQLLTP FDYDSIMLYGSYTFS KEYGKLRTMEGKNG QFLKDVVRKYFMSK SDIQRIKTLYNCTN*	GCAGTGTACTTTATTAGTTATCAGACAACAAGATGTAAAACT TGTTTGAACACACTTTACGAGTCATCTATAAGTCATTATTGGC TATAAATCTCTACGTAAAGAGTTAAAAATTCCCCTACGAGAA AGGCATCCATCATTTATAGAGTTTCAGTTGGTGCAATTGTAG AGCGTTTTTATCCTCTGAATGTCACTCTTGCTCATGAAGTATT TGCGCACCACGTCTTTCAAAAATTGTCCGTTCTTTCCTTCCAT GGTTCTCAGCTTGCCATATTCTTTCGAAAAAGTGTAAGACCC GTACAGCATGATTGAATCATAGTCGAAAGGAGTCAAAAGTT GGTTCTGGTCGGGCTTTAACTTCATGAACTGGGGCGCCATTC CTTCTTTGATATTATCCCAAAAAATTGTGAGCCAATCGTCTCT ATCAGATCGGTTCTGTTCGTGGTAGAATCCGAGAGCATGTGC CAACTCATGCATTACCGTGCCCATATATCCGCAGCCTTGACC TAGAGAGACGGGCTGTGCGCCGTAGGTCTTGCCCACGTGAG AGTAACACACTATGAATATTTCTTCATCACTGAAATGACTTA TCCCGTTTACATTTACTCTGTATTTCTACCTCCTTCGTTCGTCA AAAACAGCTGGGAAGTGCCTTACAGACGTGAATCACGTACT TTGCACTGGCTCATTTCATTTAAGGAAGATGTCATAAAAAAA AAACTCTTCTTACCCTTGGCCTGGG

PhdEnzMtP 20	K7Z9Q9	2,93E-29	44,755	PF01400	72948,42	SFVGKRGNGPQAISI<br>GKNCDKFGIVVHEL<br>GHVVGFWHEHTRP<br>DRDKHVQIINKNIM<br>TGQEYNFNKLTEEE<br>VTSLGLPYDYASIMH<br>YARNTFSKSTYLDTI<br>LPQEDPQRKRPEIGQ<br>RVRLSEGDISQTNLL<br>YKCPKCGKTLQNPS<br>GAFSSPDYGSSSPPPE<br>GEHCEWRITATQGE<br>RIILNITDIDLFKSEN<br>CDTDYLEVRDGYWY<br>KSPLLGKFCGDTKVP<br>DVLISSKYRMLVTYR<br>TSPNHAGHKGFKA<br>HYEAICGGDVIQEK<br>GILHSPNYPEDYWP<br>NKECTWRITVPENH<br>QVALKFQSFEIENHD<br>NCVYDYLEIRDGHE<br>STSPLLGRFCGYRNP<br>EDIRSSGNKMTVKFV<br>SDRSVQKAGFAADFI<br>KELDECKGDHGCEH<br>ECTNTLGAYKCECRI<br>GYELHSDGKKCEDA	GGATGGGGCACCTTGCTGCCACAGAACTTGCCCAGCGTTGGA<br>GACTCGTCGCTATCGCCATCGTAGGCCGCAATGCGGTCGTAC<br>GCACATTCTTGGTGGGGCTCCAGCTCGAAATCCTTAAACACC<br>AGTTTGATCCTGTGTCCAGGAGTCGTGGTAAAGAGCCAAGCG<br>CAATCTTTCCTGCTGGGATAATAATCAGGATAGTTGGGGCTG<br>ATAATCTCACCATGGGGTGAATTCATCTGGTGACTACAGCTT<br>CCTTCTTTACAATCGTGGAGATTCTCATGCAGTACAAATCCAT<br>TGTGACAAGAGCATGCATAACTTCCTATCGTATTCTTGCAGA<br>TGTGCTGACAACCTCCATTGTTTGTGGCACATTCATCTTTATC<br>AGTAAAGAAGAAAGCACCAAAGCCGCTCTTCTGGACAGAAT<br>TATCCGAGCTGAATTCTATTCTCAAGGAATTGCCTTCAGAGG<br>TGATAAGAGGGGGTAATCTCGAACCACAGAAGATTCCATGC<br>TTCCTGACCTCATCGTCGGCCATTTTGCTGCGTATGTCCACGC<br>TGTCATATTCACAGTCTTGATTGTTTCCTTCCAGATCGAAATG<br>CGTAAAATTAAGCGTGATTCTATACTGTGGAGGGGCTATGAT<br>TTCCCAGATGCAGATTTTGTTCGGGGGGTAGAGGTCGGGGAA<br>GGAAGGGCTGATGATCGTTCCATTAGTCTCTTCGATTACGCC<br>ACCACATGCATCTTCACATTTTTTACCATCTGAATGTAGCTCA<br>TATCCAATCCTGCATTCGCATTTGTAGGCTCCTAACGTATTGG<br>TACATTCATGTTCACAGCCGTGGTCACCTTTGCATTCATCCAG<br>TTCTTTTATGAAATCAGCGGCAAATCCTGCTTTCTGTACGGAT<br>CTGTCGGATACAAACTTGACTGTCATCTTGTTACCGGAGGAT<br>CGAATATCCTCCGGGTTTCTGTATCCACAGAAGCGACCCAGC<br>AAAGGGCTTGTGCTCTCATGGCCATCTCTGATTTCTAAGTAAT<br>CATACACACAGTTGTCATGGTTTTCAATCTCGAATGACTGGA<br>ACTTCAGAGCTACCTGATGGTTCTCAGGCACAGTAATTCGCC<br>ATGTACATTCCTTGTTTGGCCAATAATCTTCAGGGTAGTTGGG<br>ACTGTGTAGTATGCCTTTCTCCTGGATTACATCGCCACCACAA

						CGGVIEETNGTIISPSFPDLYPPNKICIWEIIAPPQYRITLNFTHFDLEGNNQDCEYDSVDIRSKMADDEVRKHGIFCGSRLPPLITSEGNSLRIEFSSDNSVQKSGFGAFFFTDKDECATNNGGCQHICKNTIGSYACSCHNGFVLHENLHDCKEGSCSHQMNSPHGEIISPNYPDYYPSRKDCAWLFTTTPGHRIKLVFKDFELEPHQECAYDRIAAYDGDSDESPTLGKFCGSKVPH	ATAGCTTCGTAGTGTGCTTTGAAACCCTTGTGGCCAGCGTGATTGGGCGACGTCCTGTAAGTGACCAACATGCGATACTTGCTGCTGATGAGCACATCAGGTACTTTTGTATCTCCGCAGAATTTGCCAAGCAGTGGCGACTTGTACCAATAACCGTCTCTCACTTCCAGGTAATCGGTGTCGCAATTCTCCGACTTGAAGAGATCAATATCTGTTATATTGAGAATGATGCGTTCACCCTGAGTCGCTGTTATTCGCCATTCACAGTGTTCGCCCTCTGGGGGTGGGGACGAACTCCCATAGTCTGGAGAAGAGAAGGCACCGCTCGGATTCTGGAGGGTTTTGCCACACTTTGGGCATTTGTAGAGAAGATTAGTCTGGGAAATGTCCCCTTCGCTTAGCCGGACCCTCTGTCCTATCTCTGGTCTTTTTCGCTGTGGGTCTTCCTGTGGCAGTATGGTGTCCAAGTAGGTACTCTTGGAGAAGGTGTTTCGGGCGTAGTGCATGATGCTGGCGTAGTCATACGGCAGGCCCAGTGACGTCACTTCCTCCTCAGTCAGTTTGTTGAAGTTGTACTCCTGACCGGTCATGATGTTCTTGTTGATTATTTGGACATGTTTATCCCGATCAGGCCGCGTATGTTCGTGCCAAAATCCGACCACATGACCAAGTTCATGCACCACGATGCCGAATTTGTCGCAGTTCTTGCCTATAGATATGGCTTGCGGGCCGTTTCCTCTCTTGCCCACGAAAGAAC

PhdEnzMtP 21	K7Z9Q9	9,91E-06	32,432	PF01400	26370,91	MEDSFRISKVLRRHE FLWIVGLVIGIAVCC AAESKKWTIEELTA ARFPHKVSNDIYLDP CKSDGFVGDIALSRL ENDVYEKELEEEEER EREEELAKMLAERA KNATELDSKRRHST TAKQDRKKFRSRNR MRHSGKNRRKSTKA HDRPKRAATARPER LWDRAVIPYDIESNF SGDHRALFKHAMR HWENYTCIQFVERE EHPNYIVFTERPCGC CSFVGKRG	TTCGACCGTATTGTTTACATCAAAGAACATCAGCTGAGAGAA AAGTTCAAGCGATTGATAAGCCGTGCTTCCGATTCGTTTTTGG CTATTGGCGTAACATTCTTGGTAAAAGCTGCTTGTTTAGCGAT GAGCAGATGACAATGAGGCGCCTGTAGTGAGAGGCGGTTAG TTTCCACGTCCGACGATGGAGGATTCTTTCCGCATTTCAAAA GTGCTGCGGAGGCACGAGTTTCTTTGGATTGTGGGACTTGTG ATCGGCATAGCAGTGTGTTGTGCCGCGGAATCTAAGAAATGG ACTATAGAGGAGTTAACAGCTGCAAGATTCCCACACAAAGT GTCCAACGACATTTACTTAGATCCATGCAAATCAGACGGTTT TGTGGGGGACATAGCCCTAAGTCGGTTGGAGAACGATGTCTA CGAGAAAGAACTGGAAGAGGAAGAGGAGCGAGAACGTGAG GAGGAACTCGCCAAAATGCTCGCCGAGCGCGCCAAAAACGC TACAGAATTGGACTCCAAAAGGCGGCATTCAACGACGGCGA AGCAAGACAGAAAAAAATTCAGGTCTCGAAATCGTATGCGG CATTCTGGCAAAAACAGGCGGAAAAGCACAAAAGCCCACG ATCGCCCGAAGCGTGCTGCAACAGCGAGGCCCGAGAGGTTA TGGGATAGAGCCGTCATTCCATACGATATAGAGTCCAATTTT AGTGGGGACCATCGTGCGCTCTTCAAACATGCTATGCGGCAT TGGGAGAATTATACCTGCATCCAGTTTGTGGAAAGGGAAGA GCATCCAAATTACATTGTTTTCACGGAAAGGCCTTGCGGGTG TTGTTCTTTCGTGGGCAAGAGAGGA

PhdEnzMtP 22	K7Z9Q9	7,52E-38	40,284	PF01400	108124.15<br><br>**105179.60**	MWVWRLAHGSVPL<br>AVVMVVAAVAGRG<br>GTA**QESSFPEASVR**<br>**GKHPRLVSTDLDM**<br>**DPGKAEIFEGDIIVD**<br>**YIPRRGSPMAQDAR**<br>**NFTNRHRRKGKGR**<br>**RGRHNRRRRPQRA**<br>**ATARSDRLWPHAV**<br>**IPYEIEANFSGEHRA**<br>**LFKQAMRYWENET**<br>**CVQFVERNPSQHP**<br>**DYIVFTERACGCCS**<br>**FVGRRGNGAQAISI**<br>**GKNCDKFGIVVHE**<br>**LGHVIGFWHEHTR**<br>**PDRDRHVQIINKNI**<br>**VAGQEYNFNKLTEE**<br>**EVNSLGLNYDYESI**<br>**MHYARNTFSRSTAL**<br>**DTILPVRGKFIEIGQ**<br>**RVRLSPGDVAQTK**<br>**MLYKCPECGRTYQ**<br>**APSAHIQSPGFETG**<br>**RTGRQHCEWRISAT**<br>**QGERIAFEVTILDM**<br>**ETSCEDSYLEIRDGY**<br>**WSKSPLLGRFCGSR**	AGGCCGAGAGAGGTGACTCGATGTGGGTCTGGCGGCTAGCG<br>CACGGCTCTGTCCCGCTGGCCGTGGTGATGGTCGTCGCAGCA<br>GTCGCGGGTCGGGGCGGGACGGCCCAGGAATCCTCCTTCCC<br>GGAGGCTTCCGTCCGAGGAAAGCACCCCAGGCTGGTGTCCA<br>CAGACCTCGACATGGATCCCGGAAAGGCAGAAATTTTCGAA<br>GGTGACATCATAGTGGACTACATTCCGAGAAGAGGATCTCCC<br>ATGGCTCAAGACGCGAGAAATTTCACAAACAGACATCGGAG<br>AAAAGGTAAAGGCCGGCGAGGAAGGCATAATCGAAGAAGA<br>CGTCCACAACGAGCGGCCACAGCTAGATCCGACAGACTTTG<br>GCCCCACGCAGTCATTCCTTATGAAATTGAAGCCAATTTCAG<br>CGGTGAACATCGAGCCCTCTTCAAACAAGCGATGCGATACT<br>GGGAAAATGAGACTTGCGTCCAATTTGTCGAGAGGAATCCCT<br>CGCAACATCCCGACTACATTGTCTTCACCGAGAGGGCGTGCG<br>GGTGTTGTTCATTTGTAGGACGAAGGGGCAATGGAGCCCAG<br>GCCATCTCGATCGGGAAGAACTGCGACAAATTCGGCATTGTG<br>GTTCACGAGCTAGGTCACGTGATCGGATTTTGGCACGAACAC<br>ACGCGACCGGATAGAGACAGACACGTGCAAATCATTAACAA<br>GAACATCGTTGCTGGTCAGGAGTACAACTTTAACAAGTTAAC<br>GGAAGAAGAAGTCAACTCCTTAGGTCTGAACTACGACTACG<br>AGAGCATAATGCACTACGCAAGGAACACCTTTTCGAGGAGT<br>ACTGCCCTTGACACCATATTGCCTGTGAGGGGTAAATTCATC<br>GAAATAGGACAAAGGGTACGACTCTCTCCGGGCGACGTGGC<br>TCAGACCAAGATGCTCTACAAGTGTCCAGAATGTGGTAGGA<br>CGTACCAGGCCCCTTCCGCACATATCCAGTCACCCGGTTTCG<br>AGACGGGTAGAACTGGACGTCAACACTGCGAGTGGCGCATC<br>TCTGCAACACAAGGAGAGCGTATAGCCTTTGAGGTGACCATC<br>TTAGACATGGAGACGTCATGCGAGGACAGCTACCTGGAAAT<br>ACGAGACGGATACTGGAGCAAGTCACCCCTGCTCGGCAGGT

						**TSGEKFVSTGHRIL**	TCTGCGGCAGTCGAACTTCGGGAGAGAAGTTCGTGTCCACGG
						**VTYHSSRTYSHHRG**	GCCACAGGATCCTAGTCACCTACCACTCTTCCAGGACGTACT
						**FQAKYEALCGGVLE**	CCCACCACAGGGGATTCCAGGCCAAATACGAAGCGCTGTGC
						**QPEGTLQSPNYPDE**	GGCGGAGTTCTGGAGCAACCCGAAGGTACTCTTCAGAGTCC
						**YRANKECIWKIVVE**	AAATTATCCGGATGAATACCGCGCCAATAAAGAATGCATAT
						**PESQVALTFQAFEV**	GGAAGATCGTGGTAGAGCCAGAAAGTCAAGTGGCCCTCACA
						**ELHDNCAYDFVEV**	TTCCAGGCTTTTGAAGTGGAGCTCCACGACAACTGCGCTTAC
						**RDGLELHSPLLARL**	GATTTCGTGGAGGTTCGGGACGGCCTCGAGCTGCATAGCCCC
						**CGYKIPEEVRSNSN**	TTACTGGCGAGACTTTGCGGCTACAAGATCCCAGAAGAGGT
						**TMLVRFVSDSSVLK**	GCGGTCCAACTCCAATACCATGCTTGTGCGGTTTGTCTCCGA
						**MGFSANFVSEIDEC**	CAGTTCGGTACTCAAAATGGGTTTCTCGGCAAACTTCGTGTC
						**ASRSHGCEQKCINT**	AGAGATCGACGAATGCGCGAGCCGCAGTCACGGTTGCGAGC
						**IGGYRCECEIGFELH**	AAAAGTGCATCAATACCATCGGTGGATATCGGTGCGAATGT
						**SDGKRCEDACGGV**	GAGATCGGATTCGAGCTGCACTCGGACGGCAAGAGGTGCGA
						**LKSGKGAITSPSFP**	AGACGCTTGCGGAGGGGTGCTGAAAAGCGGCAAGGGCGCC
						**DLYPSNKQCIWEIV**	ATCACTAGCCCCTCGTTCCCAGACTTGTACCCTTCGAACAAG
						**APPHFRITLNFTHFD**	CAGTGCATCTGGGAGATAGTCGCGCCCCCGCACTTCCGGATC
						**LEGTNQECEYDSVD**	ACACTGAATTTCACGCACTTCGACCTCGAGGGCACCAACCA
						**VRSRSSPDAEWRR**	AGAGTGCGAATACGATAGCGTGGATGTTCGCAGCCGATCCTC
						**QGTYCGSRLPPVLT**	TCCCGACGCGGAGTGGCGACGCCAGGGCACCTACTGTGGTTC
						**SEGNALRIEFVSDN**	CCGGTTGCCCCCTGTTCTCACCTCTGAGGGGAACGCCCTGCG
						**SVQKSGFAALYFTD**	CATCGAGTTCGTCTCGGACAACTCGGTGCAGAAGAGCGGATT
						**QDECASNNGGCQ**	CGCGGCCCTCTACTTCACGGATCAAGACGAATGTGCGTCGAA
						**QICKNTVGSYQCSC**	CAACGGCGGATGTCAGCAAATCTGCAAGAACACAGTGGGCA
						**HNGFVLHPNGHDC**	GCTACCAGTGTTCTTGCCACAATGGATTCGTGCTCCACCCCA
						**KEGSCVHHVSSTE**	ATGGACACGACTGTAAAGAAGGTTCTTGCGTCCACCACGTGT
						**GVVSSPNYPDQYPS**	CGTCAACAGAGGGCGTTGTTTCCAGCCCTAACTACCCGGATC
						**RKECTWKFETTPGH**	AGTATCCCAGTCGGAAGGAGTGCACCTGGAAATTCGAAACC

						**<u>RIKLVFDSFELEPHQ ECAYDHVALYDGD SADDPLLGRFCGSK VPHPILSTAHNML MAFRSDPSVQRNG FRATHTTVCGGRLS AGPVPAVLYSHAK YGDQNYGSRAECN WIVTASSGSGRIRL RFQSFDLEPEQDCA YDFLQVLDGFESSP SLGKFCGSKLPPTLT SSGFRLLICFQSDDS IAGKGFALAYSEVF S*</u>**	ACTCCAGGTCATAGGATAAAGCTGGTGTTCGACTCCTTCGAA TTGGAGCCTCATCAAGAGTGTGCCTACGATCATGTAGCCCTC TACGACGGTGACTCTGCTGACGACCCTCTGCTGGGGCGGTTT TGTGGCAGCAAAGTCCCCCATCCCATCTTATCAACAGCGCAC AATATGCTCATGGCATTCCGCTCAGATCCGTCGGTGCAGAGG AACGGGTTTAGAGCCACGCACACCACTGTGTGCGGAGGTCG TCTGAGCGCAGGACCCGTCCCGGCCGTCCTTTACTCTCATGC CAAGTACGGCGACCAGAACTATGGCAGCCGGGCCGAGTGCA ACTGGATAGTGACGGCATCTTCGGGGTCTGGCCGCATCCGGC TGCGATTCCAGAGCTTCGACCTGGAGCCAGAGCAGGACTGC GCCTATGACTTCCTGCAGGTGCTGGACGGCTTCGAAAGCAGC CCCAGTCTCGGCAAGTTCTGCGGCAGCAAGCTTCCTCCGACG CTGACGTCATCGGGCTTCCGGCTGTTGATCTGCTTCCAGTCGG ACGACAGCATCGCTGGCAAAGGATTTGCCCTCGCCTACTCAG AGGTCTTCTCGTGACCTCCTCCTCCTCCCACAACAGACA AACAGCCACCTTACTGCCAACAACGAGGTCCGATTCTTGCGG ATCCCGAACTCTCGTAGCTGCGATAGAGAGAGAGAGAGAGA GAAGAGAGTGCATGTGTCTCACGTGTGTGTCTCGTCCCATTGT GCGTGTCGTCCCAGTGTGTCGTCGGAGCAAGCGTCGTGTCGA GTTGGACTTAGTCGTTTCTATTGTTTGACTTGTTTACTTTTCTTT ACAAAAATAAACGAAGTTGAAAGACCCGA

PhdEnzMtP 23	K7Z9Q9	8,36E-29	32,520	PF01400	48421,56	HVISINPIHQDIITET<br>NPLETEDGEPLVEG<br>DIVLPKLPEYLENEV<br>EDELQERKGLLNVIS<br>LWPSARVTYNFHSS<br>VNRQTRDNIKAAIG<br>EWERETCLKFHESPL<br>GVSFIRFRTDKSGCW<br>SMVGRQNSLLNGQ<br>DVSIGPGCERKPVIV<br>HEIGHAIGLYHEQSR<br>GDRNRYINILWHNI<br>PSGLQSQFSTGLDFS<br>RGVEYDYTSVMHYS<br>PMAFSNKPFERNTIV<br>TKNPHYQRLIGTGK<br>KISFRDAKLVNKMY<br>SCNAFCPNLFNTSQS<br>CQNGGYLSPYRGNG<br>SCHCVCPPETSGPYC<br>EEQRKSDYYSPPICG<br>GNVTEEGEIQTPGYP<br>KRELPNDSCSWWIQ<br>APRGKKVMVTFQDF<br>SFYPRLSSRTSKYRGR<br>CLQERIEIRTRNMAE<br>GNMYCGEDIKPGTN<br>LASTGRNFMIIISTPH	TTTATCCTTTAGAATGATTTTGTGCATGTATGTGTGTGTGTG<br>TGTGTGTGTGTGTGTGCATGAAGTGGGGAACATCAGAATTTC<br>TTAATAGTTTTAGTTTGTACTATACAGTGTGGTGTTTCTGTGTT<br>TCCGTACAGGTTGAAACTTCAAACGTTTTGCCATAGAAACGA<br>GTCTCAGAACATTATTCTTATATTATTAAAAGATTAAGCCATA<br>TACAGAAACACAGATATTTTAGATATCTGTTATACTACTATA<br>ACCCATCTCAAATATTTTGAGTTTCTCCGCGGTTTTATATTGG<br>ATTTCTTCTTACAACATTTTGTTGAACTGAGCTCCAAATTTTAT<br>TTTTTTTCTCGGTGTTCCCTTAGCTTGCGTTTCTGTATGCACCA<br>CATAAGATTATTGATTCATAATAAACAATTTAAGTTCCCCTA<br>AATATCAGATGGACCTGCGTTCTTCAATATTGATAGTTTCCTC<br>GTTTAAAAGCTTCAGTTTGATAAATGCTGGATAAATAAACGG<br>AGAAATTAAATCTTCTGTTTATTAAATACCTTAAGAAAGTAA<br>TGTACATTCAAAAACATCAGAAAAACGCATTAATATTTCTTA<br>GTAAAATTTCTTATGAAAGGATTACTACACAGTAATTTAGAC<br>AACCTATTCTTTAATTTTTTTTATCTCAAGGCCTAGGATTTATT<br>TTTTTAAATGTAGCCAATTATCGACGGTGCCATCCCTTTAATT<br>ATACAAGTGCAATTATATGTGTTATTTAGAGAATGGAATACA<br>GAGATATTTACACGTGTAATTTTCTTCTATGTTCTGAACTCGTT<br>TATCTACAATGATAAGACCGACGGAGAGGTTAGAAATCTCA<br>TTACGTTCGTCCTGCCAGAGTAGTATTTATTACGTTCCTAATC<br>CATAGTCATTCATTTTTTTATGTTTCATACAACATCAGAACTTT<br>GAAGTTGAAACATATCATTTTATAAGTTACTATCTTTTGTTCT<br>TGCCCTTTGTGTTGTATTTTGCTTCAGTAGGAAAGTGTATTTTC<br>ATATACAGTGTCCGACATTAATAAGTATTATTGTACACTTCTC<br>ATATTTCGTACAAATTCTTTATTTGATGATAAGGTTATATATTT<br>ATATATCTGATACCAATGAATTACTTCTCGATTTGTATTTACA<br>TATTCGTTCAATGAAACTCATGGGTAGCTTTCTTTTTTCCTAA

						GYQSTRGLRAKVTFI<br>D*	ATTATTACTATAATTGTTATTATTTCATCTGACGAAAATTTGTT<br>GTAAATATTTGTCGGCTACAAAATTTGCTGAAATATAAAAAA<br>AATACTTAGATATTGGAATAGAAATTTTTTACTCATATCTAGG<br>ACAAAGGCTTTTCTTGTTTATCGGGTTAGAGGACTCTGTTAAC<br>AAATGTCAAAGGAAAATTTCAAAGTATTTAAAGGATTTCAAT<br>AATTTAAATATGCATATATTTCAATAAGTGCGGAGCTTTTGTG<br>AATTTATTTTAGAAATATTTATGTATTCAAGCATAAAGTTCAA<br>ATTAAATGCAAAATTCTTTTGCATTTTATCTTGCTTTCACCTTT<br>CTAGAAAAGATGTAGAGACGGTAAGAGAGTTTAAAATATAT<br>ACAATGCACTAGTCTTGACTTTTCTAATCTAATTAAGATCTTA<br>ATTTTACTTGTGAAACGTTCATACATTTTATAAAATTTAAGAT<br>ATCTTTTGCGAATTAAGCTAAATACTTATAGTGAACTTTACCT<br>AGAATAAATGGTAAGTATTAAATTCTATGAGTTAGATGTAAA<br>CTTCATAAATCTCAACTTCTGTTTGGGAATTTTGCTCATGTTAT<br>TCAATTCCATAAAATATTATTAAAGTTTGAGTTCTGATTAGTA<br>GATTTACTATTGAGTTTGCTGTAATTAAGTTCAAGAATAAAA<br>GAATCTCAAGGGAAAAAACATCATGAAATGTCATAAGCATC<br>ATATTCATTTTCATTAAAAGAAAAAAAAATCTCAAGGAAAA<br>AAACGCCATAAAATATCATAAACATCATATTCATTGCTTGCA<br>TTAAAATGTATAGCATATTCTGAAATCATTGCTTGATGATTTC<br>AGAATACGTAATTACAAGTCAGGTGATAATTTCCTTTACGTT<br>CAGAATTATTTTCTTTGTATAGACAATTTTTTTATGCTACGTAC<br>TAGGACCAAAAAGTTCGGAGACTGTGCTGATATCTCGAAGA<br>CGGATTGGAGGAGAGGGGAACTATTTTACCGAGGCGTAGAG<br>GCACATGTGACGCGAGATGTCGTGAGGCAAAAGTACGTCCG<br>CAGTGCAGGTTTTTACTAGACAATGCGTTTCAGTGAAGTGTA<br>GACGTTCGCGTTCGTCGAAAGCAGAAATGGGCAGTGATGGT<br>GAGCGTGATGAATGCAAGCGGATCTGTGCTGAACTGATTGAC

							GTCGTTGACAAAGATCCGACATTTCTTCCCAAAATTATCACC<br>GGTGGTGAGACGTGGTGTTTCTTGTACGACACACAAACGAA<br>AAGCCAGTCCTCACAGTGAAAGTCACCATCATCGCCGAAGT<br>CAAAACAGTTCCGTTTGGACCGTTCAAAGGGGAAGGCAATG<br>CTGGAGGTGTTTTTTGACCAGAAGGGCTTGGTGCATTACGAG<br>TTCATACCGGCAGGGGCCACAGTGAACAAGGAGAGGTACAC<br>GCATATCCTGACTCGCCTCAATTACGCTATCCTCCGGAAACG<br>TCCAGAACTCTGGAAAAGCAAGGATTTGGTGCTGCAACATG<br>ACAATGCCTCCCCCCCCCGCTCATCCGTCTTTCAGGGTGCAG<br>TCCGCACTGACCAAACAGGGAACGTCAACCCTTCCACACCC<br>TCTCTACTCACCTGATCTCGCTCGATGCAATTTTTACCTGTTCC<br>CCCGAATTTAAAACGAACTTAAGGGGCGCCGTTTCAAAAGT<br>GCATGTGAGGTTGAGGAAGCTTAGACGGCCGCACTGGAGAC<br>CATCTCGTCCAAAGGCTTCCAGAAATGTCTTCAGCAACTGTA<br>CTCACGCTGGCAAAAGTGATAGCTGCAGAAGGGGAGTACTT<br>CGAAGGTGATTGCGTATAACACGTGATATCAATAAATCCTAT<br>CCATCAGGATATCATCACTGAGACGAATCCTCTGGAGACGG<br>AGGATGGCGAACCTCTGGTGGAGGGTGACATTGTGTTGCCCA<br>AGCTTCCAGAGTATCTGGAGAACGAAGTCGAAGATGAATTG<br>CAAGAAAGGAAAGGTCTTCTGAATGTGATATCTCTGTGGCCT<br>AGCGCTAGAGTGACTTACAATTTTCATTCATCTGTTAACAGG<br>CAAACGAGAGACAACATTAAAGCTGCTATCGGCGAGTGGGA<br>GAGGGAGACTTGCCTAAAGTTTCACGAAAGCCCTTTAGGTGT<br>TTCTTTCATCCGTTTTCGCACCGATAAATCAGGATGCTGGTCC<br>ATGGTAGGTCGTCAAAATTCTCTTTTAAACGGTCAAGATGTTT<br>CAATAGGACCAGGATGTGAAAGAAAGCCTGTGATTGTCCAC<br>GAAATTGGTCACGCGATTGGTCTTTACCACGAACAATCTCGC<br>GGGGACCGCAATAGATACATCAACATCTTGTGGCACAACAT

							TCCATCGGGACTCCAGTCTCAGTTCTCAACAGGGTTAGATTTTTCGAGAGGAGTGGAGTATGACTACACATCAGTGATGCATTACAGTCCCATGGCATTCAGTAACAAACCGTTTGAGAGGAACACTATAGTGACAAAGAATCCTCATTACCAAAGACTGATTGGAACAGGCAAAAAAATATCATTCCGAGATGCCAAGCTTGTTAACAAAATGTATTCTTGTAACGCGTTTTGCCCAAATCTCTTCAACACATCCCAAAGCTGTCAAAACGGTGGCTACCTTTCACCATACCGAGGAAACGGCAGCTGCCACTGCGTCTGCCCTCCAGAGACTTCAGGACCTTACTGTGAAGAACAGAGAAAATCCGATTACTACAGTCCACCGATATGTGGAGGTAACGTAACAGAAGAAGGAGAAATTCAAACACCAGGATACCCTAAAAGAGAACTTCCAAATGATTCTTGCAGTTGGTGGATACAGGCTCCACGAGGGAAAAAAGTTATGGTAACATTCCAGGACTTCAGCTTCTACCCAAGATTATCGTCAAGGACCAGTAAGTACCGTGGACGGTGTCTACAAGAAAGAATCGAAATTAGAACAAGAAACATGGCGGAGGGTAATATGTATTGCGGTGAAGACATCAAACCAGGGACTAACCTAGCTTCTACAGGAAGAAATTTCATGATCATCATTTCTACACCTCACGGTTATCAGTCGACGAGAGGACTGAGAGCAAAGGTCACTTTCATCGACTGACCATGGAAAATATATGTACACG

PhdEnzMtP 24	K7Z9Q9	8,35E-29	32,520	PF01400	48421,56	HVISINPIHQDIITET<br>NPLETEDGEPLVEG<br>DIVLPKLPEYLENEV<br>EDELQERKGLLNVIS<br>LWPSARVTYNFHSS<br>VNRQTRDNIKAAIG<br>EWERETCLKFHESPL<br>GVSFIRFRTDKSGCW<br>SMVGRQNSLLNGQ<br>DVSIGPGCERKPVIV<br>HEIGHAIGLYHEQSR<br>GDRNRYINILWHNI<br>PSGLQSQFSTGLDFS<br>RGVEYDYTSVMHYS<br>PMAFSNKPFERNTIV<br>TKNPHYQRLIGTGK<br>KISFRDAKLVNKMY<br>SCNAFCPNLFNTSQS<br>CQNGGYLSPYRGNG<br>SCHCVCPPETSGPYC<br>EEQRKSDYYSPPICG<br>GNVTEEGEIQTPGYP<br>KRELPNDSCSWWIQ<br>APRGKKVMVTFQDF<br>SFYPRLSSRTSKYRGR<br>CLQERIEIRTRNMAE<br>GNMYCGEDIKPGTN<br>LASTGRNFMIIISTPH	TTTATCCTTTAGAATGATTTTGTGCATGTATGTGTGTGTGTG<br>TGTGTGTGTGTGCATGAAGTGGGGAACATCAGAATTTCTTAA<br>TAGTTTTAGTTTGTACTATACAGTGTGGTGTTTCTGTGTTTCCG<br>TACAGGTTGAAACTTCAAACGTTTTGCCATAGAAACGAGTCT<br>CAGAACATTATTCTTATATTATTAAAAGATTAAGCCATATAC<br>AGAAACACAGATATTTTAGATATCTGTTATACTACTATAACC<br>CATCTCAAATATTTTGAGTTTCTCCGCGGTTTTATATTGGATTT<br>CTTCTTACAACATTTTGTTGAACTGAGCTCCAAATTTTATTTTT<br>TTTCTCGGTGTTCCCTTAGCTTGCGTTTCTGTATGCACCACATA<br>AGATTATTGATTCATAATAAACAATTTAAGTTCCCCTAAATA<br>TCAGATGGACCTGCGTTCTTCAATATTGATAGTTTCCTCGTTT<br>AAAAGCTTCAGTTTGATAAATGCTGGATAAATAAACGGAGA<br>AATTAAATCTTCTGTTTATTAAATACCTTAAGAAAGTAATGTA<br>CATTCAAAAACATCAGAAAAACGCATTAATATTTCTTAGTAA<br>AATTTCTTATGAAAGGATTACTACACAGTAATTTAGACAACC<br>TATTCTTTAATTTTTTTTATCTCAAGGCCTAGGATTTATTTTTTT<br>AAATGTAGCCAATTATCGACGGTGCCATCCCTTTAATTATAC<br>AAGTGCAATTATATGTGTTATTTAGAGAATGGAATACAGAGA<br>TATTTACACGTGTAATTTTCTTCTATGTTCTGAACTCGTTTATC<br>TACAATGATAAGACCGACGGAGAGGTTAGAAATCTCATTAC<br>GTTCGTCCTGCCAGAGTAGTATTTATTACGTTCCTAATCCATA<br>GTCATTCATTTTTTTATGTTTCATACAACATCAGAACTTTGAA<br>GTTGAAACATATCATTTTATAAGTTACTATCTTTTGTTCTTGCC<br>CTTTGTGTTGTATTTTGCTTCAGTAGGAAAGTGTATTTTCATAT<br>ACAGTGTCCGACATTAATAAGTATTATTGTACACTTCTCATAT<br>TTCGTACAAATTCTTTATTTGATGATAAGGTTATATATTTATAT<br>ATCTGATACCAATGAATTACTTCTCGATTTGTATTTACATATT<br>CGTTCAATGAAACTCATGGGTAGCTTTCTTTTTTCCTAAATTA

						GYQSTRGLRAKVTFID*	TTACTATAATTGTTATTATTTCATCTGACGAAAATTTGTTGTAAATATTTGTCGGCTACAAAATTTGCTGAAATATAAAAAAATACTTAGATATTGGAATAGAAATTTTTTACTCATATCTAGGACAAAGGCTTTTCTTGTTTATCGGGTTAGAGGACTCTGTTAACAAATGTCAAAGGAAAATTTCAAAGTATTTAAAGGATTTCAATAATTTAAATATGCATATATTTCAATAAGTGCGGAGCTTTTGTGAATTTATTTTAGAAATATTTATGTATTCAAGCATAAAGTTCAAATTAAATGCAAAATTCTTTTGCATTTTATCTTGCTTTCACCTTTCTAGAAAAGATGTAGAGACGGTAAGAGAGTTTAAAATATATACAATGCACTAGTCTTGACTTTTCTAATCTAATTAAGATCTTAATTTTACTTGTGAAACGTTCATACATTTTATAAAATTTAAGATATCTTTTGCGAATTAAGCTAAATACTTATAGTGAACTTTACCTAGAATAAATGGTAAGTATTAAATTCTATGAGTTAGATGTAAACTTCATAAATCTCAACTTCTGTTTGGGAATTTTGCTCATGTTATTCAATTCCATAAAATATTATTAAAGTTTGAGTTCTGATTAGTAGATTTACTATTGAGTTTGCTGTAATTAAGTTCAAGAATAAAAGAATCTCAAGGGAAAAAACATCATGAAATGTCATAAGCATCATATTCATTTTCATTAAAAGAAAAAAAAATCTCAAGGAAAAAAACGCCATAAAATATCATAAACATCATATTCATTGCTTGCATTAAAATGTATAGCATATTCTGAAATCATTGCTTGATGATTTCAGAATACGTAATTACAAGTCAGGTGATAATTTCCTTTACGTTCAGAATTATTTTCTTTGTATAGACAATTTTTTTATGCTACGTACTAGGACCAAAAAGTTCGGAGACTGTGCTGATATCTCGAAGACGGATTGGAGGAGAGGGGAACTATTTTACCGAGGCGTAGAGGCACATGTGACGCGAGATGTCGTGAGGCAAAAGTACGTCCGCAGTGCAGGTTTTTACTAGACAATGCGTTTCAGTGAAGTGTAGACGTTCGCGTTCGTCGAAAGCAGAAATGGGCAGTGATGGTGAGCGTGATGAATGCAAGCGGATCTGTGCTGAACTGATTGACG

							TCGTTGACAAAGATCCGACATTTCTTCCCAAAATTATCACCG<br>GTGGTGAGACGTGGTGTTTCTTGTACGACACACAAACGAAA<br>AGCCAGTCCTCACAGTGAAAGTCACCATCATCGCCGAAGTC<br>AAAACAGTTCCGTTTGGACCGTTCAAAGGGGAAGGCAATGC<br>TGGAGGTGTTTTTTGACCAGAAGGGCTTGGTGCATTACGAGT<br>TCATACCGGCAGGGGCCACAGTGAACAAGGAGAGGTACACG<br>CATATCCTGACTCGCCTCAATTACGCTATCCTCCGGAAACGT<br>CCAGAACTCTGGAAAAGCAAGGATTTGGTGCTGCAACATGA<br>CAATGCCTCCCCCCCCCGCTCATCCGTCTTTCAGGGTGCAGTC<br>CGCACTGACCAAACAGGGAACGTCAACCCTTCCACACCCTC<br>TCTACTCACCTGATCTCGCTCGATGCAATTTTTACCTGTTCCC<br>CCGAATTTAAAACGAACTTAAGGGGCGCCGTTTCAAAAGTG<br>CATGTGAGGTTGAGGAAGCTTAGACGGCCGCACTGGAGACC<br>ATCTCGTCCAAAGGCTTCCAGAAATGTCTTCAGCAACTGTAC<br>TCACGCTGGCAAAAGTGATAGCTGCAGAAGGGGAGTACTTC<br>GAAGGTGATTGCGTATAACACGTGATATCAATAAATCCTATC<br>CATCAGGATATCATCACTGAGACGAATCCTCTGGAGACGGA<br>GGATGGCGAACCTCTGGTGGAGGGTGACATTGTGTTGCCCAA<br>GCTTCCAGAGTATCTGGAGAACGAAGTCGAAGATGAATTGC<br>AAGAAAGGAAAGGTCTTCTGAATGTGATATCTCTGTGGCCTA<br>GCGCTAGAGTGACTTACAATTTTCATTCATCTGTTAACAGGC<br>AAACGAGAGACAACATTAAAGCTGCTATCGGCGAGTGGGAG<br>AGGGAGACTTGCCTAAAGTTTCACGAAAGCCCTTTAGGTGTT<br>TCTTTCATCCGTTTTCGCACCGATAAATCAGGATGCTGGTCCA<br>TGGTAGGTCGTCAAAATTCTCTTTTAAACGGTCAAGATGTTTC<br>AATAGGACCAGGATGTGAAAGAAAGCCTGTGATTGTCCACG<br>AAATTGGTCACGCGATTGGTCTTTACCACGAACAATCTCGCG<br>GGGACCGCAATAGATACATCAACATCTTGTGGCACAACATTC

							CATCGGGACTCCAGTCTCAGTTCTCAACAGGGTTAGATTTTTCGAGAGGAGTGGAGTATGACTACACATCAGTGATGCATTACAGTCCCATGGCATTCAGTAACAAACCGTTTGAGAGGAACACTATAGTGACAAAGAATCCTCATTACCAAAGACTGATTGGAACAGGCAAAAAAATATCATTCCGAGATGCCAAGCTTGTTAACAAAATGTATTCTTGTAACGCGTTTTGCCCAAATCTCTTCAACACATCCCAAAGCTGTCAAAACGGTGGCTACCTTTCACCATACCGAGGAAACGGCAGCTGCCACTGCGTCTGCCCTCCAGAGACTTCAGGACCTTACTGTGAAGAACAGAGAAAATCCGATTACTACAGTCCACCGATATGTGGAGGTAACGTAACAGAAGAAGGAGAAATTCAAACACCAGGATACCCTAAAAGAGAACTTCCAAATGATTCTTGCAGTTGGTGGATACAGGCTCCACGAGGGAAAAAAGTTATGGTAACATTCCAGGACTTCAGCTTCTACCCAAGATTATCGTCAAGGACCAGTAAGTACCGTGGACGGTGTCTACAAGAAAGAATCGAAATTAGAACAAGAAACATGGCGGAGGGTAATATGTATTGCGGTGAAGACATCAAACCAGGGACTAACCTAGCTTCTACAGGAAGAAATTTCATGATCATCATTTCTACACCTCACGGTTATCAGTCGACGAGAGGACTGAGAGCAAAGGTCACTTTCATCGACTGACCATGGAAAATATATGTACACG

PhdEnzMtP 25	A0FKN6	1,25E-51	43,836	PF01400	27351.31<br>**25778.34**	MKIFLAIVLAATAAS<br>A**ITDKERSRLAMQ**<br>**NPDLFQGDILGFDP**<br>**DDKNVIPYSQMRW**<br>**ANKIVPYTIDSSLFL**<br>**QKNLILSAMKDYH**<br>**DNTCLRFKPRTTER**<br>**NYIKLFLGDGCYSY**<br>**VGMVNRGEQPVSL**<br>**GRGCHFRGTIVHEL**<br>**GHAIGFFHEQNRSD**<br>**RDKYLIIYWDNISK**<br>**GMESQFALLSPSQN**<br>**LLLTSFDHDSIMLY**<br>**GNYAFTKNRGSKT**<br>**MVARNGKSLLEPY**<br>**DKQGMTQSDIQRV**<br>**NKMYNC***	TTCAAAACAGTACAATGCATTCTCAACGATATTATCGATACT<br>TCTACAACACTAATTCAGGAAAGTTGTGATTTCGTCTTTATCC<br>ACTCAAGGACCGCAATGTCATTCCCGGATTCCAGCTCCGATG<br>GCCCGGGGGCGAAGTGGCGTACGAAATCGATCCGTCTCTCG<br>AGGCCATGGGATCGGAGCTGCCTGTTTAGATTTCCGGCAGCA<br>AAACTGACGATTTCCTTTCTGCGAAATTCCTCGGTTGCAGAC<br>ACAGTTCTAGAAAATGCATCGGTTCCCATGCGAGTAACGTTT<br>CAGAGTATCTGGCTGATAAAGCTGATCGGGCAATGTTTATTT<br>TTCTCTATAAATACCTCAACTAAAGGAGAACCTCAGTGTTCT<br>TTCCTTGCACAACCAGTTCACCATGAAGATATTCCTGGCCAT<br>CGTTCTTGCAGCAACGGCAGCTTCTGCCATTACAGACAAAGA<br>GAGAAGCAGGTTGGCTATGCAAAACCCTGATCTGTTCCAAG<br>GAGATATCTTAGGCTTTGATCCTGACGACAAAAATGTCATTC<br>CTTATTCACAGATGCGATGGGCCAATAAAATTGTTCCATACA<br>CCATCGATTCTTCCCTGTTCCTGCAGAAAAATCTGATCCTGTC<br>AGCGATGAAAGATTATCATGATAACACCTGCCTTCGTTTTAA<br>GCCCAGAACCACTGAAAGAAACTACATCAAGCTATTCTTGG<br>GAGACGGATGCTATTCGTATGTCGGAATGGTGAACAGAGGT<br>GAACAGCCTGTATCACTCGGAAGGGGTTGCCACTTCAGAGGT<br>ACCATTGTGCATGAGTTGGGTCATGCCATCGGTTTCTTCCACG<br>AACAGAACAGATCTGACCGTGACAAATACCTTATCATTTACT<br>GGGACAACATTTCTAAAGGTATGGAATCCCAGTTTGCCCTTT<br>TGTCTCCTAGTCAGAATCTCCTTTTGACCTCTTTCGACCACGA<br>CTCCATCATGTTGTACGGCAACTACGCCTTCACCAAAAACCG<br>CGGATCTAAGACCATGGTGGCCAGAAATGGAAAGTCCCTCTT<br>GGAGCCCTACGATAAGCAAGGAATGACACAAAGCGATATCC<br>AAAGAGTCAACAAAATGTACAACTGTTAAAAACTGAACAAG<br>AAATAAATGTAAATACTCTCAAAATAAATCAGGATTAAAGA

							ATGTTATACAATGACATTGAAAAATGAGTATCTAAGTAGTTT TGTTTAAATAAAC

PhdEnzMtP 26	A0FKN6	5,56E-51	43,836	PF01400	27351.31<br>**25778.34**	MKIFLAIVLAATAAS<br>A**ITDKERSRLAMQ**<br>**NPDLFQGDILGFDP**<br>**DDKNVIPYSQMRW**<br>**ANKIVPYTIDSSLFL**<br>**QKNLILSAMKDYH**<br>**DNTCLRFKPRTTER**<br>**NYIKLFLGDGCYSY**<br>**VGMVNRGEQPVSL**<br>**GRGCHFRGTIVHEL**<br>**GHAIGFFHEQNRSD**<br>**RDKYLIIYWDNISK**<br>**GMESQFALLSPSQN**<br>**LLLTSFDHDSIMLY**<br>**GNYAFTKNRGSKT**<br>**MVARNGKSLLEPY**<br>**DKQGMTQSDIQRV**<br>**NKMYNC***	TTCAAAACAGTACAATGCATTCTCAACGATATTATCGATACT<br>TCTACAACACTAATTCAGGAAAGTTGTGATTTCGTCTTTATCC<br>ACTCAAGGACCGCAATGTCATTCCCGGATTCCAGCTCCGATG<br>GCCCGGGGGCGAAGTGGCGTACGAAATCGATCCGTCTCTCG<br>ACGCGCATAAAGCTAAAATCCTGGACGCCATGAATCACTAC<br>GAAGGGTTGACCTGCGTTCGATTCAAGAAGAGAACGCTGGA<br>AAAAAACTACATCAGGATATTTGCTGGACAAGGTTGCTACTC<br>AAACGTAGGCATGATCAGCCGTGGCCAGCAACCGGTTTCATT<br>AGGAAATGGGTGCATCTTCAAGGGCACTATTGTCCATGAGTT<br>GGGACATGCTCTCGGATTTTTTCACGAACAGAACAGATCGGA<br>CCGAGATGATTATCTCATCATCTACTGGGAAAACATAAAGCA<br>AGATGCATCGGTTCCCATGCGAGTAACGTTTCAGAGTATCTG<br>GCTGATAAAGCTGATCGGGCAATGTTTATTTTTCTCTATAAAT<br>ACCTCAACTAAAGGAGAACCTCAGTGTTCTTTCCTTGCACAA<br>CCAGTTCACCATGAAGATATTCCTGGCCATCGTTCTTGCAGC<br>AACGGCAGCTTCTGCCATTACAGACAAAGAGAGAAGCAGGT<br>TGGCTATGCAAAACCCTGATCTGTTCCAAGGAGATATCTTAG<br>GCTTTGATCCTGACGACAAAAATGTCATTCCTTATTCACAGA<br>TGCGATGGGCCAATAAAATTGTTCCATACACCATCGATTCTT<br>CCCTGTTCCTGCAGAAAAATCTGATCCTGTCAGCGATGAAAG<br>ATTATCATGATAACACCTGCCTTCGTTTTAAGCCCAGAACCA<br>CTGAAAGAAACTACATCAAGCTATTCTTGGGAGACGGATGCT<br>ATTCGTATGTCGGAATGGTGAACAGAGGTGAACAGCCTGTAT<br>CACTCGGAAGGGGTTGCCACTTCAGAGGTACCATTGTGCATG<br>AGTTGGGTCATGCCATCGGTTTCTTCCACGAACAGAACAGAT<br>CTGACCGTGACAAATACCTTATCATTTACTGGGACAACATTT<br>CTAAAGGTATGGAATCCCAGTTTGCCCTTTTGTCTCCTAGTCA<br>GAATCTCCTTTTGACCTCTTTCGACCACGACTCCATCATGTTG

							TACGGCAACTACGCCTTCACCAAAAACCGCGGATCTAAGACCATGGTGGCCAGAAATGGAAAGTCCCTCTTGGAGCCCTACGATAAGCAAGGAATGACACAAAGCGATATCCAAAGAGTCAACAAAATGTACAACTGTTAAAAACTGAACAAGAAATAAATGTAAATACTCTCAAAATAAATCAGGATTAAAGAATGTTATACAATGACATTGAAAAATGAGTATCTAAGTAGTTTTGTTTAAATAAAC

PhdEnzMtP 27	A0FKN6	2,43E-31	49,630	PF01400	16913,2	MILFFSCYSNVGMIS RGQQPVSLGNGCIF KGTIVHELGHALGFF HEQNRSDRDDYLIIY WENIKQGLEDQFTK LKSHQNILMDTFDY NSVMLYGNTAFSKD GRSNTMVAKTGVRL YETYDKPGLSASDV KRVRKMYWCDEPW NKKD*	GTTTGACTTCACCGTAGAAACTTGCGGCTGTAGCAAAAAACT CATGAGGTGGGAGTGGCGATTTAAACAGCTCATTGTAATTCT TCTTAAAATCTGTCAATTTTGATACTAGTAATTTTTTTAGCTCG CAGAATTTTGATTCTTATCTTCTTCCCTATAAAGAAAATTATA TTGAAATTCATTTGATTATTTGAAATTATATAGAAAATGAAA GTACCGTTTGAAAGATAGTGGAGTGACTGTAAGTTAATATTA AAAAAACCGTTAGTGTTTAGTTTGTGTTTTTTTGACACTTGTG CCTTGTTGTTTGCTTGCTAAATCTTAGGATTTGTGTTTTTTAGT ACGTCAATGACAGAAGTGTTTTTCGACAACACTCTTTTGTGAT CTGAGTGTGTTTTAGGCGCAAAGCGACCGCGTGGTCAGTGGA ACAGGTGTGTCGGAGCAGGTTGTTTACAAACATGGAGCTGTT CGTCTTCAGCTTTCTAGCCGTAATGGTTCTCACCGCAGTGGTA CTGAAAACCATTTCTTGTTGGACGCAATGCTACAATCCAGCC TCATCAGCGGAAGTGAAAAAGTTTTTAGAGAAGAAGAAAAA AGAACTAAGTGCTTCTAAGAGGCAACTTGTACAATTAGAAAT TCCTATGGAAGATATTGAAAGAACCAGTACTCCAGTGCCAA AGGATGATAAGCATTTTCCTAGATCTGAATATCACAGCGATG TTTAACACTGCTGTAGGGGCCAAATACTAGAAAGTTTTAGTC CTGTTTTGGTGCATTTTAAATGACTTAAGTCACGAGTGAATTT TAGAGTCACTGAAAAATAGTTTACACATTTATTAATAACAGC AGTTATTACCAGTATTGTTATAACTGAATCCAGTATTTTGTTTT CAGATCTTTGAATCTCAGACATGAAAGAAATTCTTTTGAAAA TGGGCCTTAAGCTAAAAGCCTGTTAGCAGTATTTTTAATAAC TAGTACCTTTTAGGATGACGGATTAATTTAACCCTTAAAAGG TGTCATCTAAAAAAAAATCACGTTAACGGTATCATGGGTGTC ATTTCATCCCAGTAAATGAATTTTATTTTATAAATTTAATAAA GACATGTAAATGAGATTAGATATATTATAAGAATTACATATG TCATGAAAATTATAAAATTTATTTTTTACAGTTATTGCAAATA

							ATATTTCGGTGCCTTTTACAAATGAAGTTGGTACATATTGTAC<br>ATCTCACAGTATATTTTCTATCTTCATTCCTTGTACATATAAA<br>ACATCTCCCTTGCTTAGATGTGGATGATGGTATTGGCGCTGTT<br>TCCTCAATCTTTTCGTGAAGTATTTCTAATATTATTTTCCTCAG<br>ATTATGTGGCAAATTCGGTATTTTAATCGTTCTTTAAGAAATG<br>GTCCAACGAGAGAAAATCCTAATTCTCGCTTATTATCGCTTA<br>TAGTGTCCTCATAATCGCTAAAATCAACCGTTTCTAGCATAA<br>TTTTTTCAATTTCATCTTTTTTTTGGGAAAGCCATATTATAAAA<br>TTATCTTCACGGCAATAAAATAAGTAAAATAAAGTGAATAA<br>AAACACTTCTAAAATAATTATTTCATTACCTCTCAGCTTATAA<br>TTGTCTTACTAGAAACAAAAATTATAACTACGGTACTTAACA<br>TCAAATGAAATCTTCGCGAAATTTTAAAAAACTTAAACTTAC<br>GGTATCATGGATGAAATATCACCCCAGATCAGTTTTCTTTGTT<br>GTGCAGCCAACGAAGTACATAACAAAAGTGAACTCTTTTTG<br>ATAACGGTATCCCTACTGACGCCATAAATGATTGTAGGAAA<br>AATCAGTGTTACCAGTAATTCGGTTTTTTAACAATATTAGAA<br>AAACAATGTACATGGGGTGATTTTTCAGACATGATACCTTTT<br>AAGAGTTAAGATGTCCAAAGCATCAGCGAAAGAGACTTTAG<br>CATCACTAAAAGCAAATTACGTTCAAATTTAAATAAAAGAA<br>AACTTAATGTTCATTAATTTAAATGGACATTAGATGGACATG<br>GTCTTAAAAATGTCAGCCGAAAAAAAGTTTTTAACAAATGGT<br>ACTTCAGTGTGAAAATATAAAGGCACTGATTATAATGAAATG<br>CAACTTCTAAAATTCTATCACTGAATGAAAGGAACGATATTA<br>CTTATCTTATGACATTTATAGTCTGTCATTAAAATGAGATTAA<br>TCAGTAACTCTCAACAAAAACACAATCAGTTTGATCTAGTTC<br>CAATAAATATTTTCCTGTAGGCTAAAAATTTTGAACTTTCGTT<br>GGGCCCTGATTTCACATATAAATATATAATTTATTCCCAACTT<br>ATAATGAATATCTCCAGAGAAGTACATTGGTAAAAGATGATT

							TTATTTTTCAGTTGCTACTCAAACGTAGGCATGATCAGCCGTGGCCAGCAACCGGTTTCATTAGGAAATGGGTGCATCTTCAAGGGCACTATTGTCCATGAGTTGGGACATGCTCTCGGATTTTTTCACGAACAGAACAGATCGGACCGAGATGATTATCTCATCATCTACTGGGAAAACATAAAGCAAGGCTTAGAAGACCAGTTCACTAAACTGAAATCACACCAGAATATACTGATGGACACCTTTGACTACAATTCCGTCATGCTGTACGGAAACACTGCTTTCTCCAAGGACGGTCGGTCCAACACGATGGTGGCCAAGACAGGGGTACGTCTGTACGAGACATACGACAAGCCAGGCTTGAGCGCCAGTGATGTGAAACGGGTGAGGAAGATGTACTGGTGTGACGAACCTTGGAACAAGAAAGATTAAGTTAAAATGTGCTCAGATGAAATTAAAATTACTTTCTCACAAAAAAAAAAA

PhdEnzMtP 28	A0FKN6	2,37E-51	43,831	PF01400	27351.31<br>**25778.34**	MKIFLAIVLAATAAS<br>A**ITDKERSRLAMQ**<br>**NPDLFQGDILGFDP**<br>**DDKNVIPYSQMRW**<br>**ANKIVPYTIDSSLFL**<br>**QKNLILSAMKDYH**<br>**DNTCLRFKPRTTER**<br>**NYIKLFLGDGCYSY**<br>**VGMVNRGEQPVSL**<br>**GRGCHFRGTIVHEL**<br>**GHAIGFFHEQNRSD**<br>**RDKYLIIYWDNISK**<br>**GMESQFALLSPSQN**<br>**LLLTSFDHDSIMLY**<br>**GNYAFTKNRGSKT**<br>**MVARNGKSLLEPY**<br>**DKQGMTQSDIQRV**<br>**NKMYNC***	TTCAAAACAGTACAATGCATTCTCAACGATATTATCGATACT<br>TCTACAACACTAATTCAGGAAAGTTGTGATTTCGTCTTTATCC<br>ACTCAAGGACCGCAATGTCATTCCCGGATTCCAGCTCCGATG<br>GCCCGGGGGCGAAGTGGCGTACGAAATCGATCCGTCTCTCGT<br>TGCTACTCAAACGTAGGCATGATCAGCCGTGGCCAGCAACC<br>GGTTTCATTAGGAAATGGGTGCATCTTCAAGGGCACTATTGT<br>CCATGAGTTGGGACATGCTCTCGGATTTTTTCACGAACAGAA<br>CAGATCGGACCGAGATGATTATCTCATCATCTACTGGGAAAA<br>CATAAAGCAAGATGCATCGGTTCCCATGCGAGTAACGTTTCA<br>GAGTATCTGGCTGATAAAGCTGATCGGGCAATGTTTATTTTTC<br>TCTATAAATACCTCAACTAAAGGAGAACCTCAGTGTTCTTTC<br>CTTGCACAACCAGTTCACCATGAAGATATTCCTGGCCATCGT<br>TCTTGCAGCAACGGCAGCTTCTGCCATTACAGACAAAGAGA<br>GAAGCAGGTTGGCTATGCAAAACCCTGATCTGTTCCAAGGA<br>GATATCTTAGGCTTTGATCCTGACGACAAAAATGTCATTCCTT<br>ATTCACAGATGCGATGGGCCAATAAAATTGTTCCATACACCA<br>TCGATTCTTCCCTGTTCCTGCAGAAAAATCTGATCCTGTCAGC<br>GATGAAAGATTATCATGATAACACCTGCCTTCGTTTTAAGCC<br>CAGAACCACTGAAAGAAACTACATCAAGCTATTCTTGGGAG<br>ACGGATGCTATTCGTATGTCGGAATGGTGAACAGAGGTGAA<br>CAGCCTGTATCACTCGGAAGGGGTTGCCACTTCAGAGGTACC<br>ATTGTGCATGAGTTGGGTCATGCCATCGGTTTCTTCCACGAAC<br>AGAACAGATCTGACCGTGACAAATACCTTATCATTTACTGGG<br>ACAACATTTCTAAAGGTATGGAATCCCAGTTTGCCCTTTTGTC<br>TCCTAGTCAGAATCTCCTTTTGACCTCTTTCGACCACGACTCC<br>ATCATGTTGTACGGCAACTACGCCTTCACCAAAAACCGCGGA<br>TCTAAGACCATGGTGGCCAGAAATGGAAAGTCCCTCTTGGA<br>GCCCTACGATAAGCAAGGAATGACACAAAGCGATATCCAAA

							GAGTCAACAAAATGTACAACTGTTAAAAACTGAACAAGAAATAAATGTAAATACTCTCAAAATAAATCAGGATTAAAGAATGTTATACAATGACATTGAAAAATGAGTATCTAAGTAGTTTTGTTTAAATAAAC

PhdEnzMtP 29	A0FKN6	2,33E-31	49,630	PF01400	16913,2	MILFFSCYSNVGMIS RGQQPVSLGNGCIF KGTIVHELGHALGFF HEQNRSDRDDYLIIY WENIKQGLEDQFTK LKSHQNILMDTFDY NSVMLYGNTAFSKD GRSNTMVAKTGVRL YETYDKPGLSASDV KRVRKMYWCDEPW NKKD*	GTTTGACTTCACCGTAGAAACTTGCGGCTGTAGCAAAAAACT CATGAGGTGGGAGTGGCGATTTAAACAGCTCATTGTAATTCT TCTTAAAATCTGTCAATTTTGATACTAGTAATTTTTTTAGCTCG CAGAATTTTGATTCTTATCTTCTTCCCTATAAAGAAAATTATA TTGAAATTCATTTGATTATTTGAAATTATATAGAAAATGAAA GTACCGTTTGAAAGATAGTGGAGTGACTTACGTCAATGACAG AAGTGTTTTTCGACAACACTCTTTTGTGATCTGAGTGTGTTTT AGGCGCAAAGCGACCGCGTGGTCAGTGGAACAGGTGTGTCG GAGCAGGTTGTTTACAAACATGGAGCTGTTCGTCTTCAGCTTT CTAGCCGTAATGGTTCTCACCGCAGTGGTACTGAAAACCATT TCTTGTTGGACGCAATGCTACAATCCAGCCTCATCAGCGGAA GTGAAAAAGTTTTTAGAGAAGAAGAAAAAAGAACTAAGTGC TTCTAAGAGGCAACTTGTACAATTAGAAATTCCTATGGAAGA TATTGAAAGAACCAGTACTCCAGTGCCAAAGGATGATAAGC ATTTTCCTAGATCTGAATATCACAGCGATGTTTAACACTGCTG TAGGGGCCAAATACTAGAAAGTTTTAGTCCTGTTTTGGTGCA TTTTAAATGACTTAAGTCACGAGTGAATTTTAGAGTCACTGA AAAATAGTTTACACATTTATTAATAACAGCAGTTATTACCAG TATTGTTATAACTGAATCCAGTATTTTGTTTTCAGATCTTTGAA TCTCAGACATGAAAGAAATTCTTTTGAAAATGGGCCTTAAGC TAAAAGCCTGTTAGCAGTATTTTTAATAACTAGTACCTTTTAG GATGACGGATTAATTTAACCCTTAAAAGGTGTCATCTAAAAA AAAATCACGTTAACGGTATCATGGGTGTCATTTCATCCCAGT AAATGAATTTTATTTTATAAATTTAATAAAGACATGTAAATG AGATTAGATATATTATAAGAATTACATATGTCATGAAAATTA TAAAATTTATTTTTTACAGTTATTGCAAATAATATTTCGGTGC CTTTTACAAATGAAGTTGGTACATATTGTACATCTCACAGTAT ATTTTCTATCTTCATTCCTTGTACATATAAAACATCTCCCTTGC

							TTAGATGTGGATGATGGTATTGGCGCTGTTTCCTCAATCTTTT<br>CGTGAAGTATTTCTAATATTATTTTCCTCAGATTATGTGGCAA<br>ATTCGGTATTTTAATCGTTCTTTAAGAAATGGTCCAACGAGA<br>GAAAATCCTAATTCTCGCTTATTATCGCTTATAGTGTCCTCAT<br>AATCGCTAAAATCAACCGTTTCTAGCATAATTTTTTCAATTTC<br>ATCTTTTTTTTGGGAAAGCCATATTATAAAATTATCTTCACGG<br>CAATAAAATAAGTAAAATAAAGTGAATAAAAACACTTCTAA<br>AATAATTATTTCATTACCTCTCAGCTTATAATTGTCTTACTAG<br>AAACAAAAATTATAACTACGGTACTTAACATCAAATGAAAT<br>CTTCGCGAAATTTTAAAAAACTTAAACTTACGGTATCATGGA<br>TGAAATATCACCCCAGATCAGTTTTCTTTGTTGTGCAGCCAAC<br>GAAGTACATAACAAAAGTGAACTCTTTTTGATAACGGTATCC<br>CTACTGACGCCATAAATGATTGTAGGAAAAATCAGTGTTACC<br>AGTAATTCGGTTTTTTAACAATATTAGAAAAACAATGTACAT<br>GGGGTGATTTTTCAGACATGATACCTTTTAAGAGTTAAGATG<br>TCCAAAGCATCAGCGAAAGAGACTTTAGCATCACTAAAAGC<br>AAATTACGTTCAAATTTAAATAAAAGAAAACTTAATGTTCAT<br>TAATTTAAATGGACATTAGATGGACATGGTCTTAAAAATGTC<br>AGCCGAAAAAAAGTTTTTAACAAATGGTACTTCAGTGTGAA<br>AATATAAAGGCACTGATTATAATGAAATGCAACTTCTAAAAT<br>TCTATCACTGAATGAAAGGAACGATATTACTTATCTTATGAC<br>ATTTATAGTCTGTCATTAAAATGAGATTAATCAGTAACTCTCA<br>ACAAAAACACAATCAGTTTGATCTAGTTCCAATAAATATTTT<br>CCTGTAGGCTAAAAATTTTGAACTTTCGTTGGGCCCTGATTTC<br>ACATATAAATATATAATTTATTCCCAACTTATAATGAATATCT<br>CCAGAGAAGTACATTGGTAAAAGATGATTTTATTTTTCAGTT<br>GCTACTCAAACGTAGGCATGATCAGCCGTGGCCAGCAACCG<br>GTTTCATTAGGAAATGGGTGCATCTTCAAGGGCACTATTGTC

							CATGAGTTGGGACATGCTCTCGGATTTTTTCACGAACAGAAC AGATCGGACCGAGATGATTATCTCATCATCTACTGGGAAAAC ATAAAGCAAGGCTTAGAAGACCAGTTCACTAAACTGAAATC ACACCAGAATATACTGATGGACACCTTTGACTACAATTCCGT CATGCTGTACGGAAACACTGCTTTCTCCAAGGACGGTCGGTC CAACACGATGGTGGCCAAGACAGGGGTACGTCTGTACGAGA CATACGACAAGCCAGGCTTGAGCGCCAGTGATGTGAAACGG GTGAGGAAGATGTACTGGTGTGACGAACCTTGGAACAAGAA AGATTAAGTTAAAATGTGCTCAGATGAAATTAAAATTACTTT CTCACAAAAAAAAAAA

PhdEnzMtP 30	P0DM62	1,13E-04	51,145	PF01400	16631,97	FRLYPLKDRNVIPGFQLRWPGGEVAYEIDPSLDAHKAKILDAMNHYEGLTCVRFKKRTLEKNYIRIFAGQGCYSNVGMISRGQQPVSLGNGCIFKGTIVHELGHALGFFHEQNRSDRDDYLIIYWENIKQGLEDQFTKLKSHQN	TTCAAAACAGTACAATGCATTCTCAACGATATTATCGATACTTCTACAACACTAATTCAGGAAAGTTGTGATTTCGTCTTTATCCACTCAAGGACCGCAATGTCATTCCCGGATTCCAGCTCCGATGGCCCGGGGGCGAAGTGGCGTACGAAATCGATCCGTCTCTCGACGCGCATAAAGCTAAAATCCTGGACGCCATGAATCACTACGAAGGGTTGACCTGCGTTCGATTCAAGAAGAGAACGCTGGAAAAAAACTACATCAGGATATTTGCTGGACAAGGTTGCTACTCAAACGTAGGCATGATCAGCCGTGGCCAGCAACCGGTTTCATTAGGAAATGGGTGCATCTTCAAGGGCACTATTGTCCATGAGTTGGGACATGCTCTCGGATTTTTTCACGAACAGAACAGATCGGACCGAGATGATTATCTCATCATCTACTGGGAAAACATAAAGCAAGGCTTAGAAGACCAGTTCACTAAACTGAAATCACACCAGAAT
PhdEnzMtP 31	P0DM62	9,56E-23	60,870	PF01400	10054,31	MARGRSGVRNRSVSRCYSNVGMISRGQQPVSLGNGCIFKGTIVHELGHALGFFHEQNRSDRDDYLIIYWENIKQGLEDQFTKLKSHQN	TTCAAAACAGTACAATGCATTCTCAACGATATTATCGATACTTCTACAACACTAATTCAGGAAAGTTGTGATTTCGTCTTTATCCACTCAAGGACCGCAATGTCATTCCCGGATTCCAGCTCCGATGGCCCGGGGGCGAAGTGGCGTACGAAATCGATCCGTCTCTCGTTGCTACTCAAACGTAGGCATGATCAGCCGTGGCCAGCAACCGGTTTCATTAGGAAATGGGTGCATCTTCAAGGGCACTATTGTCCATGAGTTGGGACATGCTCTCGGATTTTTTCACGAACAGAACAGATCGGACCGAGATGATTATCTCATCATCTACTGGGAAAACATAAAGCAAGGCTTAGAAGACCAGTTCACTAAACTGAAATCACACCAGAAT

PhdEnzMtP 32	A0FKN6	4,80E-51	43,836	PF01400	27351.31<br>**25778.34**	MKIFLAIVLAATAAS<br>A**ITDKERSRLAMQ**<br>**NPDLFQGDILGFDP**<br>**DDKNVIPYSQMRW**<br>**ANKIVPYTIDSSLFL**<br>**QKNLILSAMKDYH**<br>**DNTCLRFKPRTTER**<br>**NYIKLFLGDGCYSY**<br>**VGMVNRGEQPVSL**<br>**GRGCHFRGTIVHEL**<br>**GHAIGFFHEQNRSD**<br>**RDKYLIIYWDNISK**<br>**GMESQFALLSPSQN**<br>**LLLTSFDHDSIMLY**<br>**GNYAFTKNRGSKT**<br>**MVARNGKSLLEPY**<br>**DKQGMTQSDIQRV**<br>**NKMYNC***	AAAAGCGCAAAAATATAGACTTCGACCCATTGCTTCTTTTTC<br>AGCGACATTCTAAAGCAAAGAATATTTTTAATGACGCAGAC<br>GATAATTAAACCCTATTCAGAATTGATGCCAGCCAGGTTTTG<br>TTTTTTTGTCCTTTAAATTTAATTCACAGAGAAAACCAATATC<br>GTTACAAAAATGCGTGAACTCTCTCTGTATTTACTAACAAAA<br>AACCAGTTTTATTTGTGTTTAGTCAGTAATATTTAACTTCATTT<br>GCGAACTCTTGCCTTTAGATAGTTACATTATGACAGACATAG<br>ACTTAGAATTTGAGTCTGAAGAATAAATTTGATTCCACGGTC<br>AATAATGATCTTCCCCTGTTATTTTCATTTGCATGTAATATTCG<br>AGAAAAGTGATAAACTTGATTGAGAAAAAAGCTGATATCAG<br>GGATTCATTTCACTTCCAGATGCATCGGTTCCCATGCGAGTA<br>ACGTTTCAGAGTATCTGGCTGATAAAGCTGATCGGGCAATGT<br>TTATTTTTCTCTATAAATACCTCAACTAAAGGAGAACCTCAGT<br>GTTCTTTCCTTGCACAACCAGTTCACCATGAAGATATTCCTGG<br>CCATCGTTCTTGCAGCAACGGCAGCTTCTGCCATTACAGACA<br>AAGAGAGAAGCAGGTTGGCTATGCAAAACCCTGATCTGTTC<br>CAAGGAGATATCTTAGGCTTTGATCCTGACGACAAAAATGTC<br>ATTCCTTATTCACAGATGCGATGGGCCAATAAAATTGTTCCA<br>TACACCATCGATTCTTCCCTGTTCCTGCAGAAAAATCTGATCC<br>TGTCAGCGATGAAAGATTATCATGATAACACCTGCCTTCGTT<br>TTAAGCCCAGAACCACTGAAAGAAACTACATCAAGCTATTC<br>TTGGGAGACGGATGCTATTCGTATGTCGGAATGGTGAACAGA<br>GGTGAACAGCCTGTATCACTCGGAAGGGGTTGCCACTTCAGA<br>GGTACCATTGTGCATGAGTTGGGTCATGCCATCGGTTTCTTCC<br>ACGAACAGAACAGATCTGACCGTGACAAATACCTTATCATTT<br>ACTGGGACAACATTTCTAAAGGTATGGAATCCCAGTTTGCCC<br>TTTTGTCTCCTAGTCAGAATCTCCTTTTGACCTCTTTCGACCAC<br>GACTCCATCATGTTGTACGGCAACTACGCCTTCACCAAAAAC

							CGCGGATCTAAGACCATGGTGGCCAGAAATGGAAAGTCCCTCTTGGAGCCCTACGATAAGCAAGGAATGACACAAAGCGATATCCAAAGAGTCAACAAAATGTACAACTGTTAAAAACTGAACAAGAAATAAATGTAAATACTCTCAAAATAAATCAGGATTAAAGAATGTTATACAATGACATTGAAAAATGAGTATCTAAGTAGTTTTGTTTAAATAAAC

PhdEnzMtP 33	A0FKN6	5,61E-52	43,836	PF01400	27351.31<br><br>**25778.34**	MKIFLAIVLAATAAS<br>A**ITDKERSRLAMQ<br>NPDLFQGDILGFDP<br>DDKNVIPYSQMRW<br>ANKIVPYTIDSSLFL<br>QKNLILSAMKDYH<br>DNTCLRFKPRTTER<br>NYIKLFLGDGCYSY<br>VGMVNRGEQPVSL<br>GRGCHFRGTIVHEL<br>GHAIGFFHEQNRSD<br>RDKYLIIYWDNISK<br>GMESQFALLSPSQN<br>LLLTSFDHDSIMLY<br>GNYAFTKNRGSKT<br>MVARNGKSLLEPY<br>DKQGMTQSDIQRV<br>NKMYNC***	TTCAAAACAGTACAATGCATTCTCAACGATATTATCGATACT<br>TCTACAACACTAATTCAGGAAAGTTGTGATTTCGTCTTTATCC<br>ACTCAAGGACCGCAATGTCATTCCCGGATTCCAGCTCCGATG<br>GCCCGGGGGCGAAGTGGCGTACGAAATCGATCCGTCTCTCG<br>ATGCATCGGTTCCCATGCGAGTAACGTTTCAGAGTATCTGGC<br>TGATAAAGCTGATCGGGCAATGTTTATTTTTCTCTATAAATAC<br>CTCAACTAAAGGAGAACCTCAGTGTTCTTTCCTTGCACAACC<br>AGTTCACCATGAAGATATTCCTGGCCATCGTTCTTGCAGCAA<br>CGGCAGCTTCTGCCATTACAGACAAAGAGAGAAGCAGGTTG<br>GCTATGCAAAACCCTGATCTGTTCCAAGGAGATATCTTAGGC<br>TTTGATCCTGACGACAAAAATGTCATTCCTTATTCACAGATG<br>CGATGGGCCAATAAAATTGTTCCATACACCATCGATTCTTCC<br>CTGTTCCTGCAGAAAAATCTGATCCTGTCAGCGATGAAAGAT<br>TATCATGATAACACCTGCCTTCGTTTTAAGCCCAGAACCACT<br>GAAAGAAACTACATCAAGCTATTCTTGGGAGACGGATGCTA<br>TTCGTATGTCGGAATGGTGAACAGAGGTGAACAGCCTGTATC<br>ACTCGGAAGGGGTTGCCACTTCAGAGGTACCATTGTGCATGA<br>GTTGGGTCATGCCATCGGTTTCTTCCACGAACAGAACAGATC<br>TGACCGTGACAAATACCTTATCATTTACTGGGACAACATTTC<br>TAAAGGTATGGAATCCCAGTTTGCCCTTTTGTCTCCTAGTCAG<br>AATCTCCTTTTGACCTCTTTCGACCACGACTCCATCATGTTGT<br>ACGGCAACTACGCCTTCACCAAAAACCGCGGATCTAAGACC<br>ATGGTGGCCAGAAATGGAAAGTCCCTCTTGGAGCCCTACGAT<br>AAGCAAGGAATGACACAAAGCGATATCCAAAGAGTCAACA<br>AAATGTACAACTGTTAAAAACTGAACAAGAAATAAATGTAA<br>ATACTCTCAAAATAAATCAGGATTAAAGAATGTTATACAATG<br>ACATTGAAAAATGAGTATCTAAGTAGTTTTGTTTAAATAAAC

PhdEnzMtP 34	A0FKN6	1,41E-17	39,837	PF01400	14972.2<br>**13013.77**	MKSIIYAVFMVCGTV<br>AWG**KTARRDPMIN**<br>**PGMFEGDMLGVRR**<br>**SKDRSAVPLDSQR**<br>**WSGGEIPYVVDQSF**<br>**DEGMRKALKAGM**<br>**DHISSRTCIRFVERT**<br>**LEHDYVKVFKGNG**<br>**CYSHWGRIGGQQD**<br>**LSLGIGCYDLGTV**	CTACCGTACCCAAGTCGTAGCACCCGATGCCCAGTGATAAGT<br>CTTGCTGACCCCCGATGCGACCCCAGTGGGAGTAACACCCGT<br>TTCCTTTGAAAACTTTAACGTAGTCATGTTCAAGGGTTCTCTC<br>CACAAACCGTATACATGTTCTGGAAGATATGTGGTCCATGCC<br>AGCTTTTAGTGCTTTCCGCATCCCTTCATCGAAAGATTGATCT<br>ACGACATAAGGAATTTCGCCTCCGGACCACCGCTGTGAATCG<br>AGAGGGACTGCACTTCTGTCCTTAGAACGTCTCACTCCCAGC<br>ATATCTCCCTCGAACATACCCGGATTTATCATCGGGTCTCTTC<br>TGGCTGTTTTTCCCCATGCTACTGTTCCACACACCATAAAAC<br>CGCGTAAATTATTGATTTCATACCGTTTGTGTGAG
PhdEnzMtP 35	K7Z9Q9	5,06E-11	45,455	PF01400	11366,58	GDAQALSLQKNGC<br>VHISTFLHEFMHAIG<br>FHHEQNRLDRDDC<br>VEVLWGNIDEDWH<br>SQYEKTDRRYFSDH<br>CGYDYYSIMHYRMQ<br>APGSRKDAFRVLR	CGGAGACGCACAAGCATTGTCGTTGCAGAAAAATGGTTGCG<br>TGCACATTTCTACATTCCTACATGAATTTATGCATGCTATTGG<br>GTTCCATCATGAGCAAAATCGTTTAGACAGGGACGATTGCGT<br>CGAAGTGCTCTGGGGAAATATTGATGAAGATTGGCATTCCCA<br>ATACGAAAAGACTGACCGCCGTTATTTCAGCGATCACTGTGG<br>GTACGATTACTACTCAATAATGCATTATCGCATGCAAGCACC<br>AGGCTCTCGAAAAGATGCTTTCAGGGTGTTAAGG
Hyalurnidases							
**Transcript ID**	**Uniprot ID reference protein**	**e-value**	**Identity (%)**	**PFAM domain**	**MW (Da)**	**Amino acid sequence**	**Nucleotide sequence**

PhdEnzHya 01	R4J7Z9	3,88E-87	54,077	PF01630	27587.56	KFFNVYRSYMLETLR LAKKLRPNAPWCYY LFPDCYNYKGEKAK DFRCSPATMKGNDK LTWLWEESTALCPSI YVYESHLDRYTFEQR TWRNNELLRETLRV ASRKAKIFPYVNYYQ KKMIPEQEMWRILA QAAAIGADRVVIWG SSASVGSEDHCKNL KRYIIDTLGPAAEKV AWRSDLCSNQICNG KGRCSFPNDDYAKA WKLFTDDTVKTFYA GDITCRCSENYSGRF CEKRI*	GGACCTAGGTTTCGGCCTCATATGGCCTCATCCGCAAAGTCC TGCCATATTACACTAGAGAAAGCAGCAATTCCAAAAGTGGA ATTCCTGCAAAACATTACTGGTAAGCGAAATAATATTCCGCC TTTTAAAACCAGCCGGGTCGGAAGACAGGGAATCACCCCAC AAGAATCCGTACAAGTTTTACCACTGGGAGATGAAATTAATT GCAAGTAACTCGCCTATGGGCGGAACAAAAGAATGCAAAA AATAAGCATCTGGAACTGCTTTCCACTACCAATCATAATCTC ACAGAACAAAAAATTAGTCACCCCTTGAGAAATCACGTTAA TACCGTGTAATTCCACTTCTGGCCTTGATAACCACCTGGATTC GGTTAGGAAGGGAGTCCACAAAGTTCTGCAGGTACGTCGCA TCCAGTTTAAACCAATTTCTGGGGTTTAAATATATTAAACCCC AGTTTAATTAGTTTAACCAGTTTAAACCAATCACGCAAATCC ACCAAATTTCGGGGATACTGATTTCGGCGTTTTACCCGCTGTT CCAAAAGTTCACAGATTTTCAATGGGGTTGACGTCAGGTGAT TTTGCAGGCCAGTCGAGGTGTGATAAGGTGTGTGGGTGTTCA TCACACCAGTCACATATGCATCCATCCCTGTGTACTTTACTGT TGTCATCTTGAAAAATAAGGGTGTCAAGAGCATACTCACCAC GAAGATGTTGACTGCAGGGAAGCACCTGATCATCGAGAATG TTAAAATAAACATGCTGGTTCATGTTAGTGGTCACCTCAATG AGTGGGCCCAATCCATAGTACGAGAAACACTCCCAAAACAT CACAGACCCTCCTCCGGTTTGAACCTGACCTTGCACACATTG AGGATGAAATACTTCATTTGGCCTTCGGTGCACTCAACGCCG TGCGTCATTTGAATGAAGGCAAAAACGGGATCCGTCAGACC ACATTATATGCCGCTAGCAGCTACTGTCCACGTTCGGTGATTT CTGGCCCATTAAAGACGTGCAACTTTATGTGCCTGTGTGAGC AATGGCCTCTTGCGAGGTACCCGATTCCAAATTTGCATTGCA TGCAATTCCCGTCGCAATGTTCTCTCGATAATAGGTTGGGAT GGACCTTCATTAAGGGACTGCTTCAATTCCTGTCGGGTTTGG

							AAGCGATTTTGAGTCACAAGCCGTAAAACTCTTCTCCGGTCC<br>CTCTCGGTTAGAATCTTTTTCCGCTCACAGTTCTGGCGTTGTG<br>TTTCATGACCACGCGTGTTACACCACTGCTTGTAGACACATT<br>GAACAGTCTGGTGCGATACATCAACAAATCCGGCAACTTCA<br>CTCACCGTATGGCCATGGGCACTGCCAAACACGATTGCACCT<br>TTCTGGCACTCTCTCACATACTTGCATTTTCCCATCTTCACAC<br>AAATTCCCGCTTGTAAAATAATGTCTCGCTCAGCGTTTCTGCC<br>TTATGATCACGTAGAGGCAGTACACGCGCAGTTCAGCACGTG<br>ACTCGAACGCTGCGTCATCTGTACCTGTTTAACGACTCCTCCG<br>TGCTTGTGCGCCAAAGTGACTAACTTTTTGTCCGGTGAGCTTA<br>CTATGAATGCATTTTAACTGAAAATTTTTTAATGTATACAGAT<br>CATACATGCTGGAAACTCTTCGTCTCGCAAAGAAACTTAGAC<br>CTAATGCTCCCTGGTGTTATTATCTATTTCCTGATTGCTATAAT<br>TATAAGGGAGAGAAAGCAAAAGATTTTCGGTGTAGTCCAGC<br>AACCATGAAAGGAAATGACAAATTAACATGGCTGTGGGAAG<br>AAAGTACTGCTCTGTGTCCGTCAATATATGTCTACGAAAGTC<br>ACCTTGATCGGTATACTTTTGAACAAAGAACTTGGAGAAATA<br>ACGAACTGCTTCGAGAAACTCTACGAGTTGCTTCAAGAAAA<br>GCTAAAATATTTCCATACGTCAATTATTACCAGAAGAAAATG<br>ATCCCAGAACAAGAAATGTGGAGAATACTAGCACAAGCAGC<br>AGCTATTGGAGCTGACAGAGTAGTAATATGGGGATCCTCAG<br>CATCAGTAGGTTCAGAAGATCATTGCAAGAATTTAAAACGTT<br>ACATAATAGATACTTTAGGACCGGCTGCTGAAAAAGTTGCAT<br>GGAGATCAGACTTATGTTCAAACCAGATATGTAATGGTAAA<br>GGTCGATGCTCGTTTCCTAATGATGATTATGCTAAAGCCTGG<br>AAACTCTTTACAGATGACACAGTAAAAACATTTTATGCAGGA<br>GACATCACATGCAGATGTTCTGAAAACTATTCTGGTCGATTTT<br>GTGAAAAAAGAATTTAATAGATATCATTCTGAAAAGAATAT

							CTTGAAAATTTGTCCAATAATTGTATAAAATAACATCATCTTTGTCGATGCATTTACCATTAATGACCATTGTAAAGGTTTTTATCTTTTTTAATG

PhdEnzHya 02	R4J7Z9	9,81E-119	52,090	PF01630	36003.18	LGNLEKHLVKASAD LEDVIPKKIFNGLGVI DWEAWRPVWEFN WDPMRIYQAESIKL AKQLNPSANSSAIQ AIAQSQWEKRAKSY MLETLRLAKKLRPN APWCYYLFPDCYNY KGEKAKDFRCSPAT MKGNDKLTWLWEE STALCPSIYVYESHL DRYTFEQRTWRNNE LLRETLRVASRKAKI FPYVNYYQKKMIPE QEMWRILAQAAAIG ADRVVIWGSSASVG SEDHCKNLKRYIIDT LGPAAEKVAWRSDL CSNQICNGKGRCSFP NDDYAKAWKLFTD DTVKTFYAGDITCRC SENYSGRFCEKRI*	ATTTTAATCATTCACCATGATATGAATCTAATTGTTTCTTTACT TTTCATTAGCTGGGAAATCTTGAAAAACATCTAGTAAAGGCT TCTGCGGATTTAGAAGACGTTATCCCTAAGAAAATTTTCAAT GGTCTAGGAGTGATTGACTGGGAAGCTTGGAGGCCAGTTTGG GAGTTTAACTGGGATCCAATGAGAATATACCAAGCTGAGTCC ATTAAACTAGCAAAGCAATTAAATCCATCGGCGAATTCTTCG GCCATTCAAGCAATAGCTCAAAGTCAGTGGGAAAAAAGAGC CAAATCATACATGCTGGAAACTCTTCGTCTCGCAAAGAAACT TAGACCTAATGCTCCCTGGTGTTATTATCTATTTCCTGATTGCT ATAATTATAAGGGAGAGAAAGCAAAAGATTTTCGGTGTAGT CCAGCAACCATGAAAGGAAATGACAAATTAACATGGCTGTG GGAAGAAAGTACTGCTCTGTGTCCGTCAATATATGTCTACGA AAGTCACCTTGATCGGTATACTTTTGAACAAAGAACTTGGAG AAATAACGAACTGCTTCGAGAAACTCTACGAGTTGCTTCAAG AAAAGCTAAAATATTTCCATACGTCAATTATTACCAGAAGAA AATGATCCCAGAACAAGAAATGTGGAGAATACTAGCACAAG CAGCAGCTATTGGAGCTGACAGAGTAGTAATATGGGGATCC TCAGCATCAGTAGGTTCAGAAGATCATTGCAAGAATTTAAAA CGTTACATAATAGATACTTTAGGACCGGCTGCTGAAAAAGTT GCATGGAGATCAGACTTATGTTCAAACCAGATATGTAATGGT AAAGGTCGATGCTCGTTTCCTAATGATGATTATGCTAAAGCC TGGAAACTCTTTACAGATGACACAGTAAAAACATTTTATGCA GGAGACATCACATGCAGATGTTCTGAAAACTATTCTGGTCGA TTTTGTGAAAAAAGAATTTAATAGATATCATTCTGAAAAGAA TATCTTGAAAATTTGTCCAATAATTGTATAAAATAACATCAT CTTTGTCGATGCATTTACCATTAATGACCATTGTAAAGGTTTT TATCTTTTTTAATG

PhdEnzHya 03	R4J7Z9	7,98E-87	54,077	PF01630	27587.56	KFFNVYRSYMLETLRLAKKLRPNAPWCYYLFPDCYNYKGEKAKDFRCSPATMKGNDKLTWLWEESTALCPSIYVYESHLDRYTFEQRTWRNNELLRETLRVASRKAKIFPYVNYYQKKMIPEQEMWRILAQAAIGADRVVIWGSSASVGSEDHCKNLKRYIIDTLGPAAEKVAWRSDLCSNQICNGKGRCSFPNDDYAKAWKLFTDDTVKTFYAGDITCRCSENYSGRFCEKRI*	CTTTCATGGAAAAATACTGAGAAACTAATTCAATTTAATTTGCACAAATTAATAATCTTCTTTTGGTTCAAAACGTGTTTTCTTCTTAGACATACACAGTTGAGCTATAGTGGGGAGAGTTCTTTTTACACAATGAAGTTCTTCAAAGTTTTTCTCCAAAGACTGAAGACTTGAACAGAATACTCTTGGAAAAAGTCTTACTTCTGAAGGGGTGTAGCCTTTTCCATAAAGAAAATTTATTATCTGGAGAGGGAAAAATATCATATATCAGTTTTATACAGTAAATATTAATTTTTAGTCTTTAACAAAACAAACTCAACATAGCAAATAGCTTAACTTGAAAGCAATGATAACACTGGCTAGCGTCAATTTTCTCCACAGTGAAATATTATGTGCTCTTTATAAGCTCACCAGACAAAAAATTAGTCACCCCTTGAGAAATCACGTTAATACCGTGTAATTCCACTTCTGGCCTTGATAACCACCTGGATTCGGTTAGGAAGGGAGTCCACAAAGTTCTGCAGGTACGTCGCATCCAGTTTAAACCAATTTCTGGGGTTTAAATATATTAAACCCCAGTTTAATTAGTTTAACCAGTTTAAACCAATCACGCAAATCCACCAAATTTCGGGGATACTGATTTCGGCGTTTTACCCGCTGTTCCAAAAGTTCACAGATTTTCAATGGGGTTGACGTCAGGTGATTTTGCAGGCCAGTCGAGGTGTGATAAGGTGTGTGGGTGTTCATCACACCAGTCACATATGCATCCATCCCTGTGTACTTTACTGTTGTCATCTTGAAAAATAAGGGTGTCAAGAGCATACTCACCACGAAGATGTTGACTGCAGGGAAGCACCTGATCATCGAGAATGTTAAAATAAACATGCTGGTTCATGTTAGTGGTCACCTCAATGAGTGGGCCCAATCCATAGTACGAGAAACACTCCCAAAACATCACAGACCCTCCTCCGGTTTGAACCTGACCTTGCACACATTGAGGATGAAATACTTCATTTGGCCTTCGGTGCACTCAACGCCGTGCGTCATTTGAATGAAGGCAAAAACGGGATCCGTCAGACCACATTATATGCCGCTAGCAGCTACTGTCCACGTTCGGTGATTTCTGGCCCATTAAAGACGTGCAACTTTATGTGCCTGTGTGAGCAATGGCCTCTTGC

							GAGGTACCCGATTCCAAATTTGCATTGCATGCAATTCCCGTC<br>GCAATGTTCTCTCGATAATAGGTTGGGATGGACCTTCATTAA<br>GGGACTGCTTCAATTCCTGTCGGGTTTGGAAGCGATTTTGAGT<br>CACAAGCCGTAAAACTCTTCTCCGGTCCCTCTCGGTTAGAAT<br>CTTTTTCCGCTCACAGTTCTGGCGTTGTGTTTCATGACCACGC<br>GTGTTACACCACTGCTTGTAGACACATTGAACAGTCTGGTGC<br>GATACATCAACAAATCCGGCAACTTCACTCACCGTATGGCCA<br>TGGGCACTGCCAAACACGATTGCACCTTTCTGGCACTCTCTC<br>ACATACTTGCATTTTCCCATCTTCACACAAATTCCCGCTTGTA<br>AAATAATGTCTCGCTCAGCGTTTCTGCCTTATGATCACGTAGA<br>GGCAGTACACGCGCAGTTCAGCACGTGACTCGAACGCTGCG<br>TCATCTGTACCTGTTTAACGACTCCTCCGTGCTTGTGCGCCAA<br>AGTGACTAACTTTTTGTCCGGTGAGCTTACTATGAATGCATTT<br>TAACTGAAAATTTTTTAATGTATACAGATCATACATGCTGGA<br>AACTCTTCGTCTCGCAAAGAAACTTAGACCTAATGCTCCCTG<br>GTGTTATTATCTATTTCCTGATTGCTATAATTATAAGGGAGAG<br>AAAGCAAAAGATTTTCGGTGTAGTCCAGCAACCATGAAAGG<br>AAATGACAAATTAACATGGCTGTGGGAAGAAAGTACTGCTC<br>TGTGTCCGTCAATATATGTCTACGAAAGTCACCTTGATCGGT<br>ATACTTTTGAACAAAGAACTTGGAGAAATAACGAACTGCTTC<br>GAGAAACTCTACGAGTTGCTTCAAGAAAAGCTAAAATATTTC<br>CATACGTCAATTATTACCAGAAGAAAATGATCCCAGAACAA<br>GAAATGTGGAGAATACTAGCACAAGCAGCAGCTATTGGAGC<br>TGACAGAGTAGTAATATGGGGATCCTCAGCATCAGTAGGTTC<br>AGAAGATCATTGCAAGAATTTAAAACGTTACATAATAGATA<br>CTTTAGGACCGGCTGCTGAAAAAGTTGCATGGAGATCAGACT<br>TATGTTCAAACCAGATATGTAATGGTAAAGGTCGATGCTCGT<br>TTCCTAATGATGATTATGCTAAAGCCTGGAAACTCTTTACAG

							ATGACACAGTAAAAACATTTTATGCAGGAGACATCACATGCAGATGTTCTGAAAACTATTCTGGTCGATTTTGTGAAAAAAGAATTTAATAGATATCATTCTGAAAAGAATATCTTGAAAATTTGTCCAATAATTGTATAAAATAACATCATCTTTGTCGATGCATTTACCATTAATGACCATTGTAAAGGTTTTTATCTTTTTTAATG

PhdEnzHya 04	R4J7Z9	9,57E-35	49,550	PF01630	13507.29	QKFYFQQEMWRILAQAAAIGADRVVIWGSSASVGSEDHCKNLKRYIIDTLGPAAEKVAWRSDLCSNQICNGKGRCSFPNDDYAKAWKLFTDDTVKTFYAGDITCRCSENYSGRFCEKRI*	ATATATTAAACATTTTTATTTTCAATAATTAAATTTACTTAAATATTATTTATTGCCACATGCTGTAGTTAACTTAAGCATATAAAACATCATTACCCCTTGCAGTGAATGGGATAATGTTAAAGTATGATATCTAGTTATTTTTTGAACGATACCAACTCTAAATACACATCAGTATACTGTGTTTTACGTAGCAGTCTGATTATTTACAGACATCTACAATCCCAACTCTTGTACCAATATTTTAATTAGAACAAGACGGCATAGAACAGTATTCTACTACATTATTTGAAACTGTGCAACTTTTTAACACTGCACCTGCAAAGTTAACTTGCAAGAAAACTAACAAAAATTCTACTTTCAGCAAGAAATGTGGAGAATACTAGCACAAGCAGCAGCTATTGGAGCTGACAGAGTAGTAATATGGGGATCCTCAGCATCAGTAGGTTCAGAAGATCATTGCAAGAATTTAAAACGTTACATAATAGATACTTTAGGACCGGCTGCTGAAAAAGTTGCATGGAGATCAGACTTATGTTCAAACCAGATATGTAATGGTAAAGGTCGATGCTCGTTTCCTAATGATGATTATGCTAAAGCCTGGAAACTCTTTACAGATGACACAGTAAAAACATTTTATGCAGGAGACATCACATGCAGATGTTCTGAAAACTATTCTGGTCGATTTTGTGAAAAAAGAATTTAATAGATATCATTCTGAAAAGAATATCTTGAAAATTTGTCCAATAATTGTATAAAATAACATCATCTTTGTCGATGCATTTACCATTAATGACCATTGTAAAGGTTTTTATCTTTTTTAATG

PhdEnzHya 05	R4J7Z9	7,84E-87	54,077	PF01630	27587.56	KFFNVYRSYMLETLR<br>LAKKLRPNAPWCYY<br>LFPDCYNYKGEKAK<br>DFRCSPATMKGNDK<br>LTWLWEESTALCPSI<br>YVYESHLDRYTFEQR<br>TWRNNELLRETLRV<br>ASRKAKIFPYVNYYQ<br>KKMIPEQEMWRILA<br>QAAAIGADRVVIWG<br>SSASVGSEDHCKNL<br>KRYIIDTLGPAAEKV<br>AWRSDLCSNQICNG<br>KGRCSFPNDDYAKA<br>WKLFTDDTVKTFYA<br>GDITCRCSENYSGRF<br>CEKRI*	CTTTCATGGAAAAATACTGAGAAACTAATTCAATTTAATTTG<br>CACAAATTAATAATCTTCTTTTGGTTCAAAACGTGTTTTCTTCT<br>TAGACATACACAGTTGAGCTATAGTGGGGAGAGTTCTTTTTA<br>CACAATGAAGTTCTTCAAAGTTTTTCTCCAAAGACTGAAGAC<br>TTGAACAGAATACTCTTGGAAAAAGTCTTACTTCTGAAGGGG<br>TGTAGCCTTTTCCATAAAGAAAATTTATTATCTGGAGAGGGA<br>AAAATATCATATATCAGTTTTATACAGTAAATATTAATTTTTA<br>GTCTTTAACAAAACAAACTCAACATAGCAAATAGCTTAACTT<br>GAAAGCAATGATAACACTGGCTAGCGTCAATTTTCTCCACAG<br>TGAAATATTATGTGCTCTTTATAAGCTCACCAGACAAAAAAT<br>TAGTCACCCCTTGAGAAATAACATTAATACGGTGTAACTCCG<br>CCCCTGGCTTAGACAACTGCCTGGATTCGGCTAGGAAGGGA<br>GTACACAAGTTTCTGCAGGTACGTCGCATCCAGTTTAAACCA<br>ATTTCTGGGGTTTAAATATATTAAACCCCAGTTTAATTAGTTT<br>AACCAGTTTAAACCAATCACGCAAATCCACCAAATTTCGGG<br>GATACTGATTTCGGCGTTTTACCCGCTGTTCCAAAAGTTCACA<br>GATTTTCAATGGGGTTGACGTCAGGTGATTTTGCAGGCCAGT<br>CGAGGTGTGATAAGGTGTGTGGGTGTTCATCACACCAGTCAC<br>ATATGCATCCATCCCTGTGTACTTTACTGTTGTCATCTTGAAA<br>AATAAGGGTGTCAAGAGCATACTCACCACGAAGATGTTGAC<br>TGCAGGGAAGCACCTGATCATCGAGAATGTTAAAATAAACA<br>TGCTGGTTCATGTTAGTGGTCACCTCAATGAGTGGGCCCAAT<br>CCATAGTACGAGAAACACTCCCAAAACATCACAGACCCTCC<br>TCCGGTTTGAACCTGACCTTGCACACATTGAGGATGAAATAC<br>TTCATTTGGCCTTCGGTGCACTCAACGCCGTGCGTCATTTGAA<br>TGAAGGCAAAAACGGGATCCGTCAGACCACATTATATGCCG<br>CTAGCAGCTACTGTCCACGTTCGGTGATTTCTGGCCCATTAA<br>AGACGTGCAACTTTATGTGCCTGTGTGAGCAATGGCCTCTTG

							CGAGGTACCCGATTCCAAATTTGCATTGCATGCAATTCCCGT<br>CGCAATGTTCTCTCGATAATAGGTTGGGATGGACCTTCATTA<br>AGGGACTGCTTCAATTCCTGTCGGGTTTGGAAGCGATTTTGA<br>GTCACAAGCCGTAAAACTCTTCTCCGGTCCCTCTCGGTTAGA<br>ATCTTTTTCCGCTCACAGTTCTGGCGTTGTGTTTCATGACCAC<br>GCGTGTTACACCACTGCTTGTAGACACATTGAACAGTCTGGT<br>GCGATACATCAACAAATCCGGCAACTTCACTCACCGTATGGC<br>CATGGGCACTGCCAAACACGATTGCACCTTTCTGGCACTCTC<br>TCACATACTTGCATTTTCCCATCTTCACACAAATTCCCGCTTG<br>TAAAATAATGTCTCGCTCAGCGTTTCTGCCTTATGATCACGTA<br>GAGGCAGTACACGCGCAGTTCAGCACGTGACTCGAACGCTG<br>CGTCATCTGTACCTGTTTAACGACTCCTCCGTGCTTGTGCGCC<br>AAAGTGACTAACTTTTTGTCCGGTGAGCTTACTATGAATGCA<br>TTTTAACTGAAAATTTTTTAATGTATACAGATCATACATGCTG<br>GAAACTCTTCGTCTCGCAAAGAAACTTAGACCTAATGCTCCC<br>TGGTGTTATTATCTATTTCCTGATTGCTATAATTATAAGGGAG<br>AGAAAGCAAAAGATTTTCGGTGTAGTCCAGCAACCATGAAA<br>GGAAATGACAAATTAACATGGCTGTGGGAAGAAAGTACTGC<br>TCTGTGTCCGTCAATATATGTCTACGAAAGTCACCTTGATCGG<br>TATACTTTTGAACAAAGAACTTGGAGAAATAACGAACTGCTT<br>CGAGAAACTCTACGAGTTGCTTCAAGAAAAGCTAAAATATTT<br>CCATACGTCAATTATTACCAGAAGAAAATGATCCCAGAACA<br>AGAAATGTGGAGAATACTAGCACAAGCAGCAGCTATTGGAG<br>CTGACAGAGTAGTAATATGGGGATCCTCAGCATCAGTAGGTT<br>CAGAAGATCATTGCAAGAATTTAAAACGTTACATAATAGAT<br>ACTTTAGGACCGGCTGCTGAAAAAGTTGCATGGAGATCAGA<br>CTTATGTTCAAACCAGATATGTAATGGTAAAGGTCGATGCTC<br>GTTTCCTAATGATGATTATGCTAAAGCCTGGAAACTCTTTACA

							GATGACACAGTAAAAACATTTTATGCAGGAGACATCACATG CAGATGTTCTGAAAACTATTCTGGTCGATTTTGTGAAAAAAG AATTTAATAGATATCATTCTGAAAAGAATATCTTGAAAATTT GTCCAATAATTGTATAAAATAACATCACCTTTGTTGTTTTTTTT TTAATCTTTTTTTTACATTAAAAAAGATAAAAACCTTTAC

PhdEnzHya<br>06	R4J7Z9	3,81E-87	54,077	PF01630	27587.56	KFFNVYRSYMLETLR<br>LAKKLRPNAPWCYY<br>LFPDCYNYKGEKAK<br>DFRCSPATMKGNDK<br>LTWLWEESTALCPSI<br>YVYESHLDRYTFEQR<br>TWRNNELLRETLRV<br>ASRKAKIFPYVNYYQ<br>KKMIPEQEMWRILA<br>QAAAIGADRVVIWG<br>SSASVGSEDHCKNL<br>KRYIIDTLGPAAEKV<br>AWRSDLCSNQICNG<br>KGRCSFPNDDYAKA<br>WKLFTDDTVKTFYA<br>GDITCRCSENYSGRF<br>CEKRI*	GGACCTAGGTTTCGGCCTCATATGGCCTCATCCGCAAAGTCC<br>TGCCATATTACACTAGAGAAAGCAGCAATTCCAAAAGTGGA<br>ATTCCTGCAAAACATTACTGGTAAGCGAAATAATATTCCGCC<br>TTTTAAAACCAGCCGGGTCGGAAGACAGGGAATCACCCCAC<br>AAGAATCCGTACAAGTTTTACCACTGGGAGATGAAATTAATT<br>GCAAGTAACTCGCCTATGGGCGGAACAAAAGAATGCAAAA<br>AATAAGCATCTGGAACTGCTTTCCACTACCAATCATAATCTC<br>ACAGAACAAAAAATTAGTCACCCCTTGAGAAATCACGTTAA<br>TACCGTGTAATTCCACTTCTGGCCTTGATAACCACCTGGATTC<br>GGTTAGGAAGGGAGTCCACAAAGTTCTGCAGGTACGTCGCA<br>TCCAGTTTAAACCAATTTCTGGGGTTTAAATATATTAAACCCC<br>AGTTTAATTAGTTTAACCAGTTTAAACCAATCACGCAAATCC<br>ACCAAATTTCGGGGATACTGATTTCGGCGTTTTACCCGCTGTT<br>CCAAAAGTTCACAGATTTTCAATGGGGTTGACGTCAGGTGAT<br>TTTGCAGGCCAGTCGAGGTGTGATAAGGTGTGTGGGTGTTCA<br>TCACACCAGTCACATATGCATCCATCCCTGTGTACTTTACTGT<br>TGTCATCTTGAAAAATAAGGGTGTCAAGAGCATACTCACCAC<br>GAAGATGTTGACTGCAGGGAAGCACCTGATCATCGAGAATG<br>TTAAAATAAACATGCTGGTTCATGTTAGTGGTCACCTCAATG<br>AGTGGGCCCAATCCATAGTACGAGAAACACTCCCAAAACAT<br>CACAGACCCTCCTCCGGTTTGAACCTGACCTTGCACACATTG<br>AGGATGAAATACTTCATTTGGCCTTCGGTGCACTCAACGCCG<br>TGCGTCATTTGAATGAAGGCAAAAACGGGATCCGTCAGACC<br>ACATTATATGCCGCTAGCAGCTACTGTCCACGTTCGGTGATTT<br>CTGGCCCATTAAAGACGTGCAACTTTATGTGCCTGTGTGAGC<br>AATGGCCTCTTGCGAGGTACCCGATTCCAAATTTGCATTGCA<br>TGCAATTCCCGTCGCAATGTTCTCTCGATAATAGGTTGGGAT<br>GGACCTTCATTAAGGGACTGCTTCAATTCCTGTCGGGTTTGG

							AAGCGATTTTGAGTCACAAGCCGTAAAACTCTTCTCCGGTCC<br>CTCTCGGTTAGAATCTTTTTCCGCTCACAGTTCTGGCGTTGTG<br>TTTCATGACCACGCGTGTTACACCACTGCTTGTAGACACATT<br>GAACAGTCTGGTGCGATACATCAACAAATCCGGCAACTTCA<br>CTCACCGTATGGCCATGGGCACTGCCAAACACGATTGCACCT<br>TTCTGGCACTCTCTCACATACTTGCATTTTCCCATCTTCACAC<br>AAATTCCCGCTTGTAAAATAATGTCTCGCTCAGCGTTTCTGCC<br>TTATGATCACGTAGAGGCAGTACACGCGCAGTTCAGCACGTG<br>ACTCGAACGCTGCGTCATCTGTACCTGTTTAACGACTCCTCCG<br>TGCTTGTGCGCCAAAGTGACTAACTTTTTGTCCGGTGAGCTTA<br>CTATGAATGCATTTTAACTGAAAATTTTTTAATGTATACAGAT<br>CATACATGCTGGAAACTCTTCGTCTCGCAAAGAAACTTAGAC<br>CTAATGCTCCCTGGTGTTATTATCTATTTCCTGATTGCTATAAT<br>TATAAGGGAGAGAAAGCAAAAGATTTTCGGTGTAGTCCAGC<br>AACCATGAAAGGAAATGACAAATTAACATGGCTGTGGGAAG<br>AAAGTACTGCTCTGTGTCCGTCAATATATGTCTACGAAAGTC<br>ACCTTGATCGGTATACTTTTGAACAAAGAACTTGGAGAAATA<br>ACGAACTGCTTCGAGAAACTCTACGAGTTGCTTCAAGAAAA<br>GCTAAAATATTTCCATACGTCAATTATTACCAGAAGAAAATG<br>ATCCCAGAACAAGAAATGTGGAGAATACTAGCACAAGCAGC<br>AGCTATTGGAGCTGACAGAGTAGTAATATGGGGATCCTCAG<br>CATCAGTAGGTTCAGAAGATCATTGCAAGAATTTAAAACGTT<br>ACATAATAGATACTTTAGGACCGGCTGCTGAAAAAGTTGCAT<br>GGAGATCAGACTTATGTTCAAACCAGATATGTAATGGTAAA<br>GGTCGATGCTCGTTTCCTAATGATGATTATGCTAAAGCCTGG<br>AAACTCTTTACAGATGACACAGTAAAAACATTTTATGCAGGA<br>GACATCACATGCAGATGTTCTGAAAACTATTCTGGTCGATTTT<br>GTGAAAAAAGAATTTAATAGATATCATTCTGAAAAGAATAT

							CTTGAAAATTTGTCCAATAATTGTATAAAATAACATCACCTTT GTTGTTTTTTTTTTAATCTTTTTTTTTACATTAAAAAAGATAAAA ACCTTTAC

PhdEnzHya 07	R4J7Z9	1,39E-65	52,198	PF01630	21410.38	ISLNCRLTWLWEESTALCPSIYVYESHLDRYTFEQRTWRNNELLRETLRVASRKAKIFPYVNYYQKKMIPEQEMWRILAQAAAIGADRVVIWGSSASVGSEDHCKNLKRYIIDTLGPAAEKVAWRSDLCSNQICNGKGRCSFPNDDYAKAWKLFTDDTVKTFYAGDITCRCSENYSGRFCEKRI*	TGTGTATTTTCTAATATGACATTTTCTGTATAATGTTGTAGGAATATCTCAATTCTAATTAATAAACTTTGCTTTAGTTGCGCAAACATCTGATGCTTTCATTATCGTTAAATTTCATTAAATTGCAGATTAACATGGCTGTGGGAAGAAAGTACTGCTCTGTGTCCGTCAATATATGTCTACGAAAGTCACCTTGATCGGTATACTTTTGAACAAAGAACTTGGAGAAATAACGAACTGCTTCGAGAAACTCTACGAGTTGCTTCAAGAAAAGCTAAAATATTTCCATACGTCAATTATTACCAGAAGAAAATGATCCCAGAACAAGAAATGTGGAGAATACTAGCACAAGCAGCAGCTATTGGAGCTGACAGAGTAGTAATATGGGGATCCTCAGCATCAGTAGGTTCAGAAGATCATTGCAAGAATTTAAAACGTTACATAATAGATACTTTAGGACCGGCTGCTGAAAAAGTTGCATGGAGATCAGACTTATGTTCAAACCAGATATGTAATGGTAAAGGTCGATGCTCGTTTCCTAATGATGATTATGCTAAAGCCTGGAAACTCTTTACAGATGACACAGTAAAAACATTTTATGCAGGAGACATCACATGCAGATGTTCTGAAAACTATTCTGGTCGATTTTGTGAAAAAAGAATTTAATAGATATCATTCTGAAAAGAATATCTTGAAAATTTGTCCAATAATTGTATAAAATAACATCATCTTTGTCGATGCATTTACCATTAATGACCATTGTAAAGGTTTTTATCTTTTTTAATG

PhdEnzHya 08	R4J7Z9	2,19E-105	51,625	PF01630	32592.46<br>**30802.18**	MKTRIIFLFLLGIVSG<br>**FEVYWNAPTIQCRA<br>TYKMDFIKLLRTYG<br>IQVNDGDKFQGNH<br>LTLFYEAQLGLYPRI<br>LKSGEMENGGIPQR<br>GDLTKHLDKASKD<br>LEKVIPWKNFNGLG<br>VIDWEAWRPTWEF<br>NWGPLKVYQNESI<br>KLAKQLNPTASASE<br>IEKIAERQWEDSTR<br>SYMLETIRLAKKLR<br>PDARWGYYLFPDC<br>YNYVGKKPKDFQC<br>SASIRKGNDKLAW<br>LWKDSSALCPSIYV<br>YESQLKSYSLEQRT<br>WRDNAKLRETLRV<br>ASKNAKIFPYINYFD<br>TK**	GTTTAGTGTCGAAATAATTAATGTATGGAAATATTTTAGCGTT<br>TTTTGAAGCTACGCGTAAAGTTTCTCTCAGCTTTGCGTTATCT<br>CTCCAAGTTCTTTGTTCCAAACTATAACTCTTGAGTTGACTCT<br>CATAGACGTATATCGAAGGACACAGGGCAGAGCTATCTTTC<br>CATAGCCATGCTAATTTGTCATTTCCTTTCCTGATTGATGCAG<br>AACACTGGAAATCCTTCGGTTTCTTTCCCACATAATTATAGCA<br>GTCAGGAAATAGATAATAGCCCCAACGAGCATCAGGTCTGA<br>GTTTCTTTGCCAAACGAATCGTTTCCAGCATGTATGATCTGGT<br>GCTGTCTTCCCATTGGCGTTCTGCTATTTTTTCAATTTCAGAAG<br>CACTTGCTGTTGGATTTAATTGCTTTGCTAGCTTAATGGATTC<br>ATTTTGGTAGACCTTTAATGGCCCCCAATTAAACTCCCAGGT<br>CGGTCTCCAAGCTTCCCAATCAATCACTCCTAGACCGTTGAA<br>ATTTTTCCAAGGGATAACCTTTTCTAAATCTTTAGAAGCCTTA<br>TCAAGATGTTTCGTGAGGTCTCCTCGCTGTGGTATTCCTCCGT<br>TTTCCATTTCTCCGGATTTTAATATTCTTGGATAGAGTCCAAG<br>TTGAGCCTCATAAAACAGCGTCAAATGGTTGCCTTGAAATTT<br>GTCTCCATCATTCACTTGTATTCCATAGGTTCTCAAAAGCTTG<br>ATGAAGTCCATCTTATAAGTTGCTCTACATTGAATGGTCGGG<br>GCATTCCAGTATACTTCAAATCCAGAAACAATCCCTAACAGG<br>AAGAGAAATATAATGCGAGTCTTCATATCGACTATATACTTC<br>AAATGAAACGAATTATTTGATTATCTCGATTTATATCATCCGC<br>TGACGAAACTCTCCGTCTGAGACAGGAACAAATGTTCATATT<br>TTAGTCTCTGGTATTATGCAGTAAAAAAAAAATAGCAATGAT<br>TATCTGTCAGGATATATTGCAATGCCATGTGCATGTTTTAAAT<br>TGCCGACTGACTTTAGCATGATAGCTTAGCACCACCTGTTAA<br>TATAGCCTTTATTTGATCTGTTAAAGAAGTAAAAAAATTTGA<br>AGGTTGAATGCGTATGGATTCACAGACATTTGGTCG

PhdEnzHya 09	R4J7Z9	1,78E-21	52,381	PF01630	10974.83<br>**8800.06**	MKIDMKTSIILLFLLGVISG**FEIYWNVPTMQCRHNYKMDFVKLLRTYGIRVNEGDKFQGESVTIFYEGQLGLYPHILKSGEMKNGGIPQVTSYFD***	GCCTGCCCTACGGTGTTTCTGAGGCTTTATATGCATCAATCAAATAAGAAGTTACCTGTGGAATACCTCCATTTTTCATTTCCCCTGATTTTAATATATGTGGATACAGTCCAAGTTGGCCCTCATAAAATATCGTCACACTCTCGCCTTGAAACTTGTCTCCTTCATTTACTCGAATTCCGTAGGTTCTCAAAAGCTTGACAAAGTCCATCTTATAGTTATGCCTGCATTGCATAGTCGGAACATTCCAGTATATTTCAAACCCGGAAATAACTCCTAACAGGAAAAGAAGAATAATGCTAGTCTTCATGTCGATCTTCATACGCTTTGAATGAAGCGGAACTGTTAGATTTCACCGATTTTATATTATCCGCTGATAAAATTCTTCTTCTGAGACAGGAACATATGTTTATACTTTAATCTGAGATATTATGCAGTAAAAGATAATAATAATTATCATCTGTTCGAATACACTTCAATGCTATTGTGTATGTTCTGAATAGGCAGCTATGATTGCTAAAGACTGTCTAAAAATGCAGATCATTATTGTTTTTGAACACTTAAATTCCTCTCAGTTTTAAATGTTGAAATACTAATGCATTCATTGACATATGATCCGTTTCTTAAGAAAGTGGTGTAAATATATTTTTGCAAGAATTGAGAACATTATACATAATTTAATGGGAACGCTTTATTTATAGACAATATATTATGGTTTAAAATATCCATATTCTTTGATCAACTGGAAATTCTCTACTAAACAAATATAATTATTTAATATGGGAGTGTATGTTTGTGTGTGGTGCAAGTTCAATTCCATTAGTCTAATTAAATACTGGATAAGCTAAGGAATAACTCAGGTGCGTTTGAAAATTACAGTCGCCATGTACTACAAAAAATTTTGCGTTTGTATATACATAAAAAAAAATCAAATCGTGACCTCTTTGAGCACTCGAACATGATGTTGACTTTATTTTAGTGTAACACATCTATGGAGGAAGCAGAATTTGAAAATTTTAATTTATTACAAACTTGAGGAGAAAGACCGCCAACATTTGGAAACATCACACAAACAGCTTTATGTAAAAATGCTAGACCCATTGCACAAAAAAATTTGTATATAATATTTTGCAGTTATTGCTTACATTACATTACATATATTACATGATACTCATAACGCATTGCATAG

							CTGCAGATGTATCACGAATATTTCTAAACACCTTCCACATAACTATTCGAAATGAAGATCGAGATCTGATATTAAACTTCTGATAATGTTAAACTCATAATGTTAATTCTCATAAAATGTCAATTT

PhdEnzHya 10	R4J7Z9	3,89E-27	51,818	PF01630	13193.67	RKFYFQQEFWRMLAQAASVGADGAAIWGSSASVNSENNCKNLKQYIIDILGPAAEKVASRSDLCSNQICNGRGRCTFPNDDDAKAWKLFIDDTVKTFNAGDITCRCSENYTGQFCNQSN*	AAGCTTCGCCTTGTTGCCGTAGGACTGTGCCCTAACTCGTGAAATTCCAGTACCAGAAACACTCTTTGTTCAATGGTATACATGTTTATACCTCCTTTCACGTTTCAGCGCTAGAACGGATCGCTTCGGCCTTCGGAGCACTATTTATAGGGACTCTTATTGCAATTACAGCGTGCAGCGCCATCTGTTGGCACCTTTTGTTGCTGGTTTCCGAACTTCTGTGTGCGGTTTTTACGGCTTTCGGAGTGGACATGCCGCTTGTTTTGTTTCACTGTCGTCCTTTGTTTTCGAGATGTTTAACTTTGAAATCAGGGAGTCCTTCGTTGGACACACTGTATGTATATATGGTTTTTATTAAAGGCAGATTTTTTTTATCTTTCTAAATTGTGATGAAACATTTAGTTGCAATGTGCAATCGGTAAAATACCCAAGAACAATTTCAAATAAATATAATTGAGACTTTAAATAAATTTTCAGGTAATTATTAATGCACAATTTTTATCATTTTAGTGCATATTGCTTGATTTTTAGGGCATGAATGCTTGCATATCTGGAGCAATTTTGGCGCTTGAATATACGATCTCTATTTATAAAAGTAACAGTAAAATATATTTTTGATGGCATCAAGCAATATTTTTCTGCATTTCATAAAATTTTTGGAACTAGATCTACAATGTACTTGCTACAAACTAACGAAAATTCTATTTTCAGCAAGAATTTTGGCGAATGCTAGCACAAGCAGCTTCTGTGGGAGCTGATGGAGCAGCAATATGGGGATCATCAGCATCAGTAAATTCGGAAAACAATTGCAAGAATCTAAAGCAGTACATTATAGATATTTTAGGTCCAGCTGCCGAGAAAGTAGCATCGAGATCAGATTTATGTTCGAATCAAATATGCAATGGTCGAGGTCGATGTACATTTCCTAATGATGATGATGCAAAAGCTTGGAAACTCTTTATTGACGACACTGTGAAAACATTTAATGCAGGAGATATCACATGCAGATGTTCTGAAAACTACACTGGCCAGTTTTGTAATCAAAGCAATTAATAGATTACGTATGAAAGACTATCTTTGAAATAAGCAGAATTGATGTATAAAAGAAATTTTCTATGACTAAATCTTTGTACTTTTAATTTTAAATCAAAAATAAATAAAATACAAAAATAATGAAACAGTTATC

							AACGCGAGTAACAGTTGTATATTTATAAACATTTTTTATAATC<br>CGATTCATATCGATAACTTTAGGAATATATTAATCTTATGCTG<br>TGCTCGAAACGCGCATCGTAAGTAACATAATCCAATATCTTT<br>TGACAAACAAAATTTAGATATACGTAAATATCTCCTTGAAAG<br>TTTAAAAGGTGTATCAAATGAAACAAGAGCGATATACAACG<br>TAATTTATCGCCTCACAATTTTCAATGCGAGTAACAATTGAA<br>TATCTATAAACCCTTCCTATAATCCGATTCATACAGATCGTTT<br>TAGGAATATATCAATCTTATGCACTACTCAAAATGTCCATCG<br>TAAATAACATAATCAAATATCTTATCATAAACAAAAATTAGA<br>TATACATAGATCCTCCCTGAAAGCTTAGAAGGTATATCAGGT<br>AAAACAAGAGTGATATACAACGTAATTTATCACCTCACAATT<br>GTCAATGCGAGTAACAATTGAATATCTATAAACCTTTCCTAT<br>AATCCGATTCATACAGATCGTTTTAGGAATATGTATCAATCTT<br>ATGCAGTACTCAAAATGTCCTTCGTAAATAACACAATCAAAT<br>ATCTTATCATAAACAAGAATTAGATGTATAGATCTCCCCCTG<br>AAAGCTTGAAAGGTATATCAGATGAAACAAGAGTGACAAGA<br>GCTTCAAAATCTTGACGCTTCATCATCGCTAATTATGGGCAC<br>ATTAAAAGTTTAATATATAACTAAGAAAAACAATTTTGATTT<br>ATTTAATCATGTCTTAAAATCAACAAAAATTTTTTCTTTATTC<br>CATTGATTCAACATGATACTCGAAGGGCATCATAATAATTTA<br>AAAATGTATAAATTTTTGACACTGTACATTTAAATCTGAATA<br>GATCCCATCGATTCGAATACAGCACTACTCTCATTGTTAATTA<br>ATTTTAATGATCTAAAAAAGCTTTTCTGAATGCCAAATGTGA<br>TCTTATACCTTATTAGACACATAATATATTCTTTTACCAGATA<br>TTTCAATAATTTTGATAACCTCCTGCACGTACTGGCGTTACAT<br>TACGTTACGCATTTAAAGAACGCATCTTAAAATATAATCTGT<br>TCTGAACATTAACACTTTAATACTCGCATTAAATCCTTGTTCT<br>TTTCTCGCTGTAAATGGGTAATACAAGAATTTTATTATCATAT

							TTGAATTCCCCATGGCTAAATTTATGAAAATTAAACTAAATT<br>GTTTTCAAAGAGAAAAAGGAAAGAAGTTTTCCTTTTACCAAT<br>CAGTGCATATACTGTATGGCAGGGAGCAACAAAAATTATTTT<br>TCCCCCGATTACTGTAATTTATAGACATCCTATATATTATACC<br>ATGGACACCCATACATTTTCCGGGGCAGTAGCAAATGTAATG<br>GTATCAGACTGAATTAAGAAACTTATATGCATCGGTATAATG<br>TATCGTATGCATAACATTGAAATTGAGGAGAGGAGGGGGAG<br>TGCCCATTCATGCCCCCCTATATAGGCGCCCATGGTATATACT<br>GTGATCCCTGTAAAACAGCATTCACTATGTGTAGATAATTGT<br>ATATATATCCAACTAAGCAAATACATTACAAAGGTTAATTAT<br>GTCTTTGTTATAACTACGTATGAAATATTCTGAGAGTATTACA<br>AATGATAGTTTTATGGGTCTCGTTACAAACATAATAATTCGA<br>TTTCCTTTTAATTCTCACACCACTTTACCTTCGCCTCTTCTCAT<br>GCGTTTGCTCCCTCTTGTCATGTTTATGCTTCACTTATTATGTT<br>CTACCGGCCATGAAAATCACTGGGTTTAACAAGGTGTTAGTA<br>AGATAATATGAATAATCATTCGGTTGATCTTCATTGGATGAA<br>ATATATAATTACGAAAGACTACATAATTTTTTTCACGATCATT<br>ACGTTCACAATTTTTATTTTTTCATGCAAAAGTTTATTAACTA<br>CTAATATAATTAATTAACTAAGTAGCTAGTAATATGATTCATT<br>ATAAAGATGTGAGTTTGAAGACAGCTATGATGCCATGTAGC<br>AAAATCGCGATACGATCATTTGTAGTTTTTCCCTGTGATGTAT<br>CTAACACTTCTTACACCATAGCCATTTTTTTGTGCCATCTTATT<br>GCTTTCAGCAGACGTATTTCAGACAACATTAAGAATGCTCCC<br>TAATGCAATCGAAATTACACATACGTTAGGCGCATATTTACC<br>AAATGGATCTCTTATTATGAAAGTGGTGTAAGTCCATTCTTCC<br>TTGTGTCGAGATGTTTAAGACTGTATTTCAAACGATTCAAAA<br>ATAATAATAGCGTCTATACTAGGTTTTCCCACGTTTCTATGAA

							TATGTGTAAACTAAATATGAAATTGACATTTTATGAGAATTA AC

PhdEnzHya 11	R4J7Z9	0	50,503	PF01630	45278.58<br><br>**43488.30**	MKTRIIFLFLLGIVSG<br>**FEVYWNAPTIQCRA<br>TYKMDFIKLLRTYG<br>IQVNDGDKFQGNH<br>LTLFYEAQLGLYPRI<br>LKSGEMENGGIPQR<br>GDLTKHLDKASKD<br>LEKVIPWKNFNGLG<br>VIDWEAWRPTWEF<br>NWGPLKVYQNESI<br>KLAKQLNPTASASE<br>IEKIAERQWEDSTR<br>SYMLETIRLAKKLR<br>PDARWGYYLFPDC<br>YNYVGKKPKDFQC<br>SASIRKGNDKLAW<br>LWKDSSALCPSIYV<br>YESQLKSYSLEQRT<br>WRDNAKLRETLRV<br>ASKNAKIFPYINYFD<br>TKLIPGQEFWRMLA<br>QAASVGADGAAIW<br>GSSASVNSENNCK<br>NLKQYIIDILGPAAE<br>KVASRSDLCSNQIC<br>NGRGRCTFPNDDD<br>AKAWKLFIDDTVK**	CGACCAAATGTCTGTGAATCCATACGCATTCAACCTTCAAAT<br>TTTTTTACTTCTTTAACAGATCAAATAAAGGCTATATTAACAG<br>GTGGTGCTAAGCTATCATGCTAAAGTCAGTCGGCAATTTAAA<br>ACATGCACATGGCATTGCAATATATCCTGACAGATAATCATT<br>GCTATTTTTTTTTTACTGCATAATACCAGAGACTAAAATATGA<br>ACATTTGTTCCTGTCTCAGACGGAGAGTTTCGTCAGCGGATG<br>ATATAAATCGAGATAATCAAATAATTCGTTTCATTTGAAGTA<br>TATAGTCGATATGAAGACTCGCATTATATTTCTCTTCCTGTTA<br>GGGATTGTTTCTGGATTTGAAGTATACTGGAATGCCCCGACC<br>ATTCAATGTAGAGCAACTTATAAGATGGACTTCATCAAGCTT<br>TTGAGAACCTATGGAATACAAGTGAATGATGGAGACAAATT<br>TCAAGGCAACCATTTGACGCTGTTTTATGAGGCTCAACTTGG<br>ACTCTATCCAAGAATATTAAAATCCGGAGAAATGGAAAACG<br>GAGGAATACCACAGCGAGGAGACCTCACGAAACATCTTGAT<br>AAGGCTTCTAAAGATTTAGAAAAGGTTATCCCTTGGAAAAAT<br>TTCAACGGTCTAGGAGTGATTGATTGGGAAGCTTGGAGACCG<br>ACCTGGGAGTTTAATTGGGGGCCATTAAAGGTCTACCAAAAT<br>GAATCCATTAAGCTAGCAAAGCAATTAAATCCAACAGCAAG<br>TGCTTCTGAAATTGAAAAAATAGCAGAACGCCAATGGGAAG<br>ACAGCACCAGATCATACATGCTGGAAACGATTCGTTTGGCA<br>AAGAAACTCAGACCTGATGCTCGTTGGGGCTATTATCTATTT<br>CCTGACTGCTATAATTATGTGGGAAAGAAACCGAAGGATTTC<br>CAGTGTTCTGCATCAATCAGGAAAGGAAATGACAAATTAGC<br>ATGGCTATGGAAAGATAGCTCTGCCCTGTGTCCTTCGATATA<br>CGTCTATGAGAGTCAACTCAAGAGTTATAGTTTGGAACAAAG<br>AACTTGGAGAGATAACGCAAAGCTGAGAGAAACTTTACGCG<br>TAGCTTCAAAAAACGCTAAAATATTTCCATACATTAATTATTT<br>CGACACTAAACTTATTCCAGGGCAAGAATTTTGGCGAATGCT

						<u>**TFNAGDITCRCSEN**</u><br><u>**YTGQFCNQSN***</u>	AGCACAAGCAGCTTCTGTGGGAGCTGATGGAGCAGCAATAT<br>GGGGATCATCAGCATCAGTAAATTCGGAAAACAATTGCAAG<br>AATCTAAAGCAGTACATTATAGATATTTTAGGTCCAGCTGCC<br>GAGAAAGTAGCATCGAGATCAGATTTATGTTCGAATCAAAT<br>ATGCAATGGTCGAGGTCGATGTACATTTCCTAATGATGATGA<br>TGCAAAAGCTTGGAAACTCTTTATTGACGACACTGTGAAAAC<br>ATTTAATGCAGGAGATATCACATGCAGATGTTCTGAAAACTA<br>CACTGGCCAGTTTTGTAATCAAAGCAATTAATAGATTACGTA<br>TGAAAGACTATCTTTGAAATAAGCAGAATTGATGTATAAAA<br>GAAATTTTCTATGACTAAATCTTTGTACTTTTAATTTTAAATC<br>AAAAATAAATAAAATACAAAAATAATGAAACAGTTATCAAC<br>GCGAGTAACAGTTGTATATTTATAAACATTTTTTATAATCCGA<br>TTCATATCGATAACTTTAGGAATATATTAATCTTATGCTGTGC<br>TCGAAACGCGCATCGTAAGTAACATAATCCAATATCTTTTGA<br>CAAACAAAATTTAGATATACGTAAATATCTCCTTGAAAGTTT<br>AAAAGGTGTATCAAATGAAACAAGAGCGATATACAACGTAA<br>TTTATCGCCTCACAATTTTCAATGCGAGTAACAATTGAATATC<br>TATAAACCCTTCCTATAATCCGATTCATACAGATCGTTTTAGG<br>AATATATCAATCTTATGCACTACTCAAAATGTCCATCGTAAA<br>TAACATAATCAAATATCTTATCATAAACAAAAATTAGATATA<br>CATAGATCCTCCCTGAAAGCTTAGAAGGTATATCAGGTAAA<br>ACAAGAGTGATATACAACGTAATTTATCACCTCACAATTGTC<br>AATGCGAGTAACAATTGAATATCTATAAACCTTTCCTATAAT<br>CCGATTCATACAGATCGTTTTAGGAATATGTATCAATCTTATG<br>CAGTACTCAAAATGTCCTTCGTAAATAACACAATCAAATATC<br>TTATCATAAACAAGAATTAGATGTATAGATCTCCCCCTGAAA<br>GCTTGAAAGGTATATCAGATGAAACAAGAGTGACAAGAGCT<br>TCAAAATCTTGACGCTTCATCATCGCTAATTATGGGCACATT

							AAAAGTTTAATATATAACTAAGAAAAACAATTTTGATTTATT<br>TAATCATGTCTTAAAATCAACAAAAATTTTTTCTTTATTCCAT<br>TGATTCAACATGATACTCGAAGGGCATCATAATAATTTAAAA<br>ATGTATAAATTTTTGACACTGTACATTTAAATCTGAATAGATC<br>CCATCGATTCGAATACAGCACTACTCTCATTGTTAATTAATTT<br>TAATGATCTAAAAAAGCTTTTCTGAATGCCAAATGTGATCTT<br>ATACCTTATTAGACACATAATATATTCTTTTACCAGATATTTC<br>AATAATTTTGATAACCTCCTGCACGTACTGGCGTTACATTAC<br>GTTACGCATTTAAAGAACGCATCTTAAAATATAATCTGTTCT<br>GAACATTAACACTTTAATACTCGCATTAAATCCTTGTTCTTTT<br>CTCGCTGTAAATGGGTAATACAAGAATTTTATTATCATATTTG<br>AATTCCCCATGGCTAAATTTATGAAAATTAAACTAAATTGTTT<br>TCAAAGAGAAAAAGGAAAGAAGTTTTCCTTTTACCAATCAGT<br>GCATATACTGTATGGCAGGGAGCAACAAAAATTATTTTTCCC<br>CCGATTACTGTAATTTATAGACATCCTATATATTATACCATGG<br>ACACCCATACATTTTCCGGGGCAGTAGCAAATGTAATGGTAT<br>CAGACTGAATTAAGAAACTTATATGCATCGGTATAATGTATC<br>GTATGCATAACATTGAAATTGAGGAGAGGAGGGGGAGTGCC<br>CATTCATGCCCCCCTATATAGGCGCCCATGGTATATACTGTG<br>ATCCCTGTAAAACAGCATTCACTATGTGTAGATAATTGTATA<br>TATATCCAACTAAGCAAATACATTACAAAGGTTAATTATGTC<br>TTTGTTATAACTACGTATGAAATATTCTGAGAGTATTACAAAT<br>GATAGTTTTATGGGTCTCGTTACAAACATAATAATTCGATTTC<br>[illegible]Ŷ[illegible]J[illegible]t[illegible]<br>[illegible] [illegible]ϖ[illegible]Ā[illegible]<br>[illegible]<br>[illegible]TAATCATTCGGTTGATCTTCATTGGATGAAATAT<br>ATAATTACGAAAGACTACATAATTTTTTTCACGATCATTACGT

							TCACAATTTTTATTTTTTCATGCAAAAGTTTATTAACTACTAAT ATAATTAATTAACTAAGTAGCTAGTAATATGATTCATTATAA AGATGTGAGTTTGAAGACAGCTATGATGCCATGTAGCAAAA TCGCGATACGATCATTTGTAGTTTTTCCCTGTGATGTATCTAA CACTTCTTACACCATAGCCATTTTTTTGTGCCATCTTATTGCTT TCAGCAGACGTATTTCAGACAACATTAAGAATGCTCCCTAAT GCAATCGAAATTACACATACGTTAGGCGCATATTTACCAAAT GGATCTCTTATTATGAAAGTGGTGTAAGTCCATTCTTCCTTGT GTCGAGATGTTTAAGACTGTATTTCAAACGATTCAAAAATAA TAATAGCGTCTATACTAGGTTTTCCCACGTTTCTATGAATATG TGTAAACTAAATATGAAATTGACATTTTATGAGAATTAAC

Carboxylesterases							
**Transcript ID**	**Uniprot ID reference protein**	**e-value**	**Identity (%)**	**PFAM domain**	**MW (Da)**	**Amino acid sequence**	**Nucleotide sequence**
PhdEnzCes 01	W4VSJ0	1,57E-21	37,815	PF00135	14433,11	RDDDYDTIRSQVYH SMGDDLLHCPSKYF AEKLEEKNNIYRYYF NHRPTTSPYAEWMG VTHCEDLQYTFGRP LTVLSNYTRMEELLS FEMVKEWTNFAKR GEPSETWRKFSRSDP YVKVF	TTAGAGATGACGATTATGATACAATAAGGAGTCAAGTTTACC ACTCTATGGGGGACGATCTTCTTCACTGTCCAAGCAAATATTT TGCTGAGAAATTGGAGGAAAAAAATAATATCTACCGATATT ATTTCAATCACAGACCAACGACTAGTCCGTACGCAGAGTGG ATGGGAGTTACGCATTGTGAAGATCTGCAGTATACCTTTGGA CGTCCATTAACTGTTTTGTCCAACTACACCCGAATGGAAGAA CTACTAAGCTTTGAGATGGTCAAAGAGTGGACGAATTTTGCT AAGAGAGGGGAACCCAGTGAGACGTGGCGGAAGTTTTCAAG ATCAGATCCATACGTCAAAGTTTTTG
PhdEnzCes 02	W4VSJ0	7,09E-22	37,815	PF00135	14460,24	RDDDYDTIRSQVYH SMGDDLLHCPSKYF AEKLEEKNNIYRYYF NHRPTTSPYAEWMG VMHCEDLEFIFGRPL TVLSNYTRMEELLSF EMVKEWTNFAKRG EPSETWRKFSRSDPY VKVF	TTAGAGATGACGATTATGATACAATAAGGAGTCAAGTTTACC ACTCTATGGGGGACGATCTTCTTCACTGTCCAAGCAAATATTT TGCTGAGAAATTGGAGGAAAAAAATAATATCTACCGATATT ATTTCAATCACAGACCAACGACTAGTCCGTACGCAGAGTGG ATGGGAGTTATGCATTGTGAAGATTTGGAATTTATCTTTGGAC GTCCATTAACTGTTTTGTCCAACTACACCCGAATGGAAGAAC TACTAAGCTTTGAGATGGTCAAAGAGTGGACGAATTTTGCTA AGAGAGGGGAACCCAGTGAGACGTGGCGGAAGTTTTCAAGA TCAGATCCATACGTCAAAGTTTTTG

PhdEnzCes 03	W4VSJ0	8,23E-50	26,549	PF00135	92987.86<br><br>**90574.86**	MFFVVYTIVLALVVL<br>VVPHVDG**DEVRRV**<br>**GSRVIATKYGSIKG**<br>**VLVTLPNRNIGPVE**<br>**VFLGIPYASPPVGQ**<br>**MRFMPPANPSQWR**<br>**GVRTADRFGPVCP**<br>**QRYPDIRNETESLR**<br>**HMPTGRLEYLKKLI**<br>**PFLRNESEDCLYLNI**<br>**YCPVKKEGARMPII**<br>**VFLHGDSYEWSSG**<br>**NPYDGTVLAGFGE**<br>**VIVITLNYRLGILGF**<br>**LPAMETASRGNYG**<br>**LMDIITALHWVQE**<br>**NIAQFGGDPRNVT**<br>**LLGHGYGASCAHL**<br>**LMLSPMSKGLFQR**<br>**VVLQSGSAFSPWA**<br>**VANEAMTFARHVA**<br>**RKLGCPVMNHPAL**<br>**VRCMRQRPISDIMR**<br>**VQLLIPEYLTAFGP**<br>**MVDGLVIPSDPEKS**<br>**FLNPPADHESSPKV**<br>**DILIGVTRIESFFYFG**<br>**ANEERYGIEAVRRD**	GACGGAACTTACTGTTATACTGTTCGAGTGCGGTTGTCAAGG<br>TCTTGTAAGACCAGCCTCTGGTTTTGCCGAAAGCGGGATACA<br>ATGCTGTGATCCCGAGGAAAGAAAAAAAATAGCCCTGTTGA<br>ATGCTCAAGAAGCCATGGCAAATATGCTTGGCTACAAAACA<br>AAAACTCATTCAAAAAATATACTTTCAAAAGTCATAATTGCT<br>TATTAAGTAGGGAAGACAGAAGGGTGTTCACATATAGAAAG<br>TTTGTAAATAGACAGTCTTAGAGCGATGATAATGAGTGTATA<br>TAGAAAAATTATTATAAATATGTTTCTATACATTTGATTATGT<br>TCGCAGTAACCTGAGAAACCAGTTGACATGTTTTTTGTTGTGT<br>ATACCATTGTGTTGGCATTGGTGGTGCTGGTTGTACCTCATGT<br>TGATGGAGATGAGGTAAGAAGAGTTGGCTCCAGAGTCATTG<br>CTACCAAATACGGTTCCATTAAAGGTGTGCTTGTGACACTTC<br>CAAACCGTAACATCGGACCAGTGGAGGTATTTCTTGGAATAC<br>CCTATGCTAGTCCTCCTGTGGGACAGATGCGTTTTATGCCCCC<br>AGCTAATCCCAGTCAGTGGAGGGGGGTACGGACCGCAGATA<br>GATTTGGCCCAGTTTGTCCCCAGCGATATCCTGATATCAGGA<br>ATGAAACTGAATCTTTACGCCACATGCCAACAGGACGATTA<br>GAGTATCTGAAGAAGCTTATTCCATTCCTTCGCAATGAAAGT<br>GAAGATTGCCTCTACTTAAACATATACTGCCCAGTTAAGAAG<br>GAAGGTGCTCGAATGCCGATTATTGTGTTCCTTCATGGTGATT<br>CATACGAATGGAGTTCAGGAAATCCTTATGATGGAACTGTAC<br>TCGCAGGATTTGGAGAAGTAATTGTAATTACTCTGAACTACA<br>GGCTTGGAATTTTGGGCTTTCTACCGGCTATGGAAACGGCTTC<br>GCGTGGAAATTATGGTTTGATGGATATCATAACCGCTCTGCA<br>TTGGGTCCAAGAAAATATAGCTCAATTTGGAGGAGATCCTCG<br>CAACGTGACTCTTCTAGGACATGGATATGGAGCATCGTGCGC<br>TCATCTTCTTATGCTTTCTCCCATGTCGAAAGGACTGTTTCAA<br>CGAGTGGTGTTACAAAGTGGCTCAGCATTCAGTCCTTGGGCA

						**RILRTLVRNLFTFHL**	GTAGCAAATGAAGCAATGACATTTGCTCGTCACGTGGCACG
						**QEIFLTIVNEYTDWS**	AAAATTGGGATGTCCAGTCATGAATCATCCTGCATTAGTGCG
						**RPVQHPVNILDGT**	TTGCATGCGACAACGTCCGATATCAGATATCATGCGAGTGCA
						**VDALTDALVVAPSI**	GTTGTTAATTCCTGAATACTTAACAGCTTTTGGACCAATGGTG
						**SSANLLSKLKHRTY**	GACGGATTGGTCATCCCATCAGATCCCGAAAAGTCGTTCCTA
						**LYVFNYPTEASDYP**	AATCCCCCAGCGGATCACGAATCCAGTCCTAAAGTTGATATA
						**QRLVCVHGEDLPYL**	CTCATTGGTGTTACTCGTATCGAGTCTTTCTTTTATTTTGGGGC
						**FGAPLVSKLAHFPN**	GAATGAAGAACGGTATGGCATTGAAGCCGTCCGTAGAGACC
						**NFSKPEVALSEAIM**	GAATTCTCAGGACACTTGTTCGCAACTTGTTTACTTTCCATTT
						**LYWSNFAKYGDPN**	ACAAGAAATATTTTTAACTATCGTGAACGAGTACACTGACTG
						**VVHDLDMPEKTRG**	GTCTCGTCCAGTACAGCATCCTGTTAATATATTAGATGGAAC
						**RFERFVWPLYDSVN**	AGTTGACGCTCTAACTGATGCTTTAGTTGTGGCACCATCAATC
						**QKYLSLAMKPKIRD**	AGTAGTGCAAATCTGTTATCAAAATTAAAACACAGGACGTAT
						**HYHAHRLSFWLSLI**	CTCTATGTATTTAACTATCCTACAGAAGCCAGTGATTACCCTC
						**PKLHRQGTTPGIAS**	AAAGATTAGTTTGTGTACATGGAGAAGACCTTCCTTACCTCTT
						**QHHLLDDHDNPST**	TGGCGCTCCTTTGGTTAGCAAGTTGGCACATTTCCCAAACAA
						**YDGIVRQGPVLPLS**	CTTCAGCAAACCAGAAGTAGCTCTGTCCGAAGCTATTATGTT
						**GAPARIPSTQLRVN**	GTATTGGTCAAATTTTGCTAAATATGGAGATCCCAACGTCGT
						**LEAPTASSAMEISVP**	ACATGATCTTGATATGCCGGAAAAAACAAGAGGACGTTTTG
						**HVFESSSTFYSSSQE**	AAAGATTTGTATGGCCATTATACGACAGTGTTAACCAAAAAT
						**DELPQTPVPSSTNV**	ACCTTTCTTTAGCTATGAAGCCAAAGATTCGGGACCATTATC
						**SGSLASSDTVMNF**	ATGCTCATAGACTTTCATTCTGGCTTAGCCTCATTCCAAAGCT
						**KDSSFTALRVTIIIG**	TCATCGGCAAGGAACTACACCTGGTATCGCTTCCCAACATCA
						**CSLLILNLCIFVGVY**	CCTCCTAGATGATCATGACAATCCTTCAACTTACGATGGAAT
						**RQRNENRVDSMM**	CGTAAGGCAAGGTCCAGTATTACCCCTATCAGGAGCTCCAGC
						**REVSSKRRGSELES**	TCGAATTCCATCTACACAACTTCGGGTCAATCTCGAGGCTCC
						**APSTIKSQIRAPPPS**	CACTGCAAGTTCTGCAATGGAAATTAGTGTGCCACATGTTTTT
						**PDLQRSLELPQFIPP**	GAGAGTAGCAGTACCTTTTATTCATCATCACAAGAAGACGA

						**PPVFIPTVSPTSTPTL IPLQDSQTKSALAS EGQHTGTSKVRWA PDVGCEADRDAV WL***	ACTGCCTCAGACGCCTGTACCGAGCTCAACTAACGTATCAGG CAGCTTGGCTTCTTCAGATACAGTCATGAACTTTAAAGATAG TTCTTTTACAGCTTTAAGAGTTACCATTATTATTGGCTGTTCAT TGCTCATTCTTAATCTCTGCATTTTCGTTGGAGTTTACCGTCAA AGAAATGAGAACAGAGTTGACTCCATGATGCGAGAGGTGAG CTCTAAACGTAGAGGATCAGAGCTGGAGAGTGCTCCTTCGAC GATCAAATCCCAAATTCGTGCCCCACCACCATCACCTGATCT TCAGCGTTCCCTGGAGCTACCGCAGTTCATTCCACCACCTCC GGTTTTTATTCCAACTGTTTCACCTACATCCACTCCAACTTTG ATTCCTTTACAAGACTCACAGACTAAGTCTGCATTAGCATCA GAAGGTCAACACACAGGCACAAGCAAAGTACGGTGGGCTCC AGATGTTGGTTGCGAAGCCGATAGGGATGCAGTATGGCTCTG ATGATAACTCTTTGCCTCCCCCCGTCGTCGAAAGAAGTTTCTA ATCGCTTCGTTGAGAGTGTACGCTACAAAACTTTTTAATGCCT GTTTTCACTGGGGGGGATGCAAAGGGATCTCAAATTTCTGTG TGCAAAAATATGTGTACTCTCGAAGAAAAATAAACAGTGTTT ATGTCTATATATAGTGGAAAATTGCAGTGCTGAAAATTGTCT GAAGAACAAAAAGGAAAACAAAAACTCATATCCAAGGATG ATCAGAATTTATTTGATAAAACATAAGAGGATTCATTTTTTCT CTTTCCTCTCTCAATACGTATTATTTAAGAAC

PhdEnzCes 04	W4VSJ0	8,16E-50	26,549	PF00135	92586.4<br>**90173.39**	MFFVVYTIVLALVVL<br>VVPHVDG**DEVRRV**<br>**GSRVIATKYGSIKG**<br>**VLVTLPNRNIGPVE**<br>**VFLGIPYASPPVGQ**<br>**MRFMPPANPSQWR**<br>**GVRTADRFGPVCP**<br>**QRYPDIRNETESLR**<br>**HMPTGRLEYLKKLI**<br>**PFLRNESEDCLYLNI**<br>**YCPVKKEGARMPII**<br>**VFLHGDSYEWSSG**<br>**NPYDGTVLAGFGE**<br>**VIVITLNYRLGILGF**<br>**LPAMETASRGNYG**<br>**LMDIITALHWVQE**<br>**NIAQFGGDPRNVT**<br>**LLGHGYGASCAHL**<br>**LMLSPMSKGLFQR**<br>**VVLQSGSAFSPWA**<br>**VANEAMTFARHVA**<br>**RKLGCPVMNHPAL**<br>**VRCMRQRPISDIMR**<br>**VQLLIPEYLTAFGP**<br>**MVDGLVIPSDPEKS**<br>**FLNPPADHESSPKV**<br>**DILIGVTRIESFFYFG**<br>**ANEERYGIEAVRRD**	GACGGAACTTACTGTTATACTGTTCGAGTGCGGTTGTCAAGG<br>TCTTGTAAGACCAGCCTCTGGTTTTGCCGAAAGCGGGATACA<br>ATGCTGTGATCCCGAGGAAAGAAAAAAAATAGCCCTGTTGA<br>ATGCTCAAGAAGCCATGGCAAATATGCTTGGCTACAAAACA<br>AAAACTCATTCAAAAAATATACTTTCAAAAGTCATAATTGCT<br>TATTAAGTAGGGAAGACAGAAGGGTGTTCACATATAGAAAG<br>TTTGTAAATAGACAGTCTTAGAGCGATGATAATGAGTGTATA<br>TAGAAAAATTATTATAAATATGTTTCTATACATTTGATTATGT<br>TCGCAGTAACCTGAGAAACCAGTTGACATGTTTTTTGTTGTGT<br>ATACCATTGTGTTGGCATTGGTGGTGCTGGTTGTACCTCATGT<br>TGATGGAGATGAGGTAAGAAGAGTTGGCTCCAGAGTCATTG<br>CTACCAAATACGGTTCCATTAAAGGTGTGCTTGTGACACTTC<br>CAAACCGTAACATCGGACCAGTGGAGGTATTTCTTGGAATAC<br>CCTATGCTAGTCCTCCTGTGGGACAGATGCGTTTTATGCCCCC<br>AGCTAATCCCAGTCAGTGGAGGGGGGTACGGACCGCAGATA<br>GATTTGGCCCAGTTTGTCCCCAGCGATATCCTGATATCAGGA<br>ATGAAACTGAATCTTTACGCCACATGCCAACAGGACGATTA<br>GAGTATCTGAAGAAGCTTATTCCATTCCTTCGCAATGAAAGT<br>GAAGATTGCCTCTACTTAAACATATACTGCCCAGTTAAGAAG<br>GAAGGTGCTCGAATGCCGATTATTGTGTTCCTTCATGGTGATT<br>CATACGAATGGAGTTCAGGAAATCCTTATGATGGAACTGTAC<br>TCGCAGGATTTGGAGAAGTAATTGTAATTACTCTGAACTACA<br>GGCTTGGAATTTTGGGCTTTCTACCGGCTATGGAAACGGCTTC<br>GCGTGGAAATTATGGTTTGATGGATATCATAACCGCTCTGCA<br>TTGGGTCCAAGAAAATATAGCTCAATTTGGAGGAGATCCTCG<br>CAACGTGACTCTTCTAGGACATGGATATGGAGCATCGTGCGC<br>TCATCTTCTTATGCTTTCTCCCATGTCGAAAGGACTGTTTCAA<br>CGAGTGGTGTTACAAAGTGGCTCAGCATTCAGTCCTTGGGCA

						**RILRTLVRNLFTFHL**	GTAGCAAATGAAGCAATGACATTTGCTCGTCACGTGGCACG
						**QEIFLTIVNEYTDWS**	AAAATTGGGATGTCCAGTCATGAATCATCCTGCATTAGTGCG
						**RPVQHPVNILDGT**	TTGCATGCGACAACGTCCGATATCAGATATCATGCGAGTGCA
						**VDALTDALVVAPSI**	GTTGTTAATTCCTGAATACTTAACAGCTTTTGGACCAATGGTG
						**SSANLLSKLKHRTY**	GACGGATTGGTCATCCCATCAGATCCCGAAAAGTCGTTCCTA
						**LYVFNYPTEASDYP**	AATCCCCCAGCGGATCACGAATCCAGTCCTAAAGTTGATATA
						**QRLVCVHGEDLPYL**	CTCATTGGTGTTACTCGTATCGAGTCTTTCTTTTATTTTGGGGC
						**FGAPLVSKLAHFPN**	GAATGAAGAACGGTATGGCATTGAAGCCGTCCGTAGAGACC
						**NFSKPEVALSEAIM**	GAATTCTCAGGACACTTGTTCGCAACTTGTTTACTTTCCATTT
						**LYWSNFAKYGDPN**	ACAAGAAATATTTTTAACTATCGTGAACGAGTACACTGACTG
						**VVHDLDMPEKTRG**	GTCTCGTCCAGTACAGCATCCTGTTAATATATTAGATGGAAC
						**RFERFVWPLYDSVN**	AGTTGACGCTCTAACTGATGCTTTAGTTGTGGCACCATCAATC
						**QKYLSLAMKPKIRD**	AGTAGTGCAAATCTGTTATCAAAATTAAAACACAGGACGTAT
						**HYHAHRLSFWLSLI**	CTCTATGTATTTAACTATCCTACAGAAGCCAGTGATTACCCTC
						**PKLHRQGTTPGIAS**	AAAGATTAGTTTGTGTACATGGAGAAGACCTTCCTTACCTCTT
						**QHHLLDDHDNPST**	TGGCGCTCCTTTGGTTAGCAAGTTGGCACATTTCCCAAACAA
						**YDGIVRQGPVLPLS**	CTTCAGCAAACCAGAAGTAGCTCTGTCCGAAGCTATTATGTT
						**GAPARIPSTQLRVN**	GTATTGGTCAAATTTTGCTAAATATGGAGATCCCAACGTCGT
						**LEAPTASSAMEISVP**	ACATGATCTTGATATGCCGGAAAAAACAAGAGGACGTTTTG
						**HVFESSSTFYSSSQE**	AAAGATTTGTATGGCCATTATACGACAGTGTTAACCAAAAAT
						**DELPQTPVPSSTNV**	ACCTTTCTTTAGCTATGAAGCCAAAGATTCGGGACCATTATC
						**SGSLASSDTVMNF**	ATGCTCATAGACTTTCATTCTGGCTTAGCCTCATTCCAAAGCT
						**KDSSFTALRVTIIIG**	TCATCGGCAAGGAACTACACCTGGTATCGCTTCCCAACATCA
						**CSLLILNLCIFVGVY**	CCTCCTAGATGATCATGACAATCCTTCAACTTACGATGGAAT
						**RQRNENRVDSMM**	CGTAAGGCAAGGTCCAGTATTACCCCTATCAGGAGCTCCAGC
						**RERRGSELESAPSTI**	TCGAATTCCATCTACACAACTTCGGGTCAATCTCGAGGCTCC
						**KSQIRAPPPSPDLQ**	CACTGCAAGTTCTGCAATGGAAATTAGTGTGCCACATGTTTTT
						**RSLELPQFIPPPPVFI**	GAGAGTAGCAGTACCTTTTATTCATCATCACAAGAAGACGA

						<u>**PTVSPTSTPTLIPLQDSQTKSALASEGQHTGTSKVRWAPDVGCEADRDAVWL***</u>	ACTGCCTCAGACGCCTGTACCGAGCTCAACTAACGTATCAGGCAGCTTGGCTTCTTCAGATACAGTCATGAACTTTAAAGATAGTTCTTTTACAGCTTTAAGAGTTACCATTATTATTGGCTGTTCATTGCTCATTCTTAATCTCTGCATTTTCGTTGGAGTTTACCGTCAAAGAAATGAGAACAGAGTTGACTCCATGATGCGAGAGCGTAGAGGATCAGAGCTGGAGAGTGCTCCTTCGACGATCAAATCCCAAATTCGTGCCCCACCACCATCACCTGATCTTCAGCGTTCCCTGGAGCTACCGCAGTTCATTCCACCACCTCCGGTTTTTATTCCAACTGTTTCACCTACATCCACTCCAACTTTGATTCCTTTACAAGACTCACAGACTAAGTCTGCATTAGCATCAGAAGGTCAACACACAGGCACAAGCAAAGTACGGTGGGCTCCAGATGTTGGTTGCGAAGCCGATAGGGATGCAGTATGGCTCTGATGATAACTCTTTGCCTCCCCCCGTCGTCGAAAGAAGTTTCTAATCGCTTCGTTGAGAGTGTACGCTACAAAACTTTTTAATGCCTGTTTTCACTGGGGGGGATGCAAAGGGATCTCAAATTTCTGTGTGCAAAAATATGTGTACTCTCGAAGAAAAATAAACAGTGTTTATGTCTATATATAGTGGAAAATTGCAGTGCTGAAAATTGTCTGAAGAACAAAAAGGAAAACAAAAACTCATATCCAAGGATGATCAGAATTTATTTGATAAAACATAAGAGGATTCATTTTTTCTCTTTCCTCTCTCAATACGTATTATTTAAGAAC

PhdEnzCes 05	W4VSJ0	9,73E-07	45,000	PF00135	6228,99	SLYAPSWRKNIDLYNFSLGLYDMLEALKWTNENIAAFGGDNKRITVFGNSAGGISAG	ACCTGCAGATATTCCTCCGGCGCTGTTGCCGAACACCGTAATTCTTTTATTGTCTCCACCGAATGCAGCAATATTTTCGTTCGTCCATTTAAGGGCTTCCAACATGTCATACAAACCTAGAGAAAAATTATAAAGATCAATATTTTTTCTCCAAGAAGGCGCATACAAGGATCATTCTTGATAAAAATTCAGTCATTTATTTTCTACATACATATAAGTTCTAAGCGGAATATTTAATATACATATTAATAGCTAACAGCGCAAGGTAGAGCGCTTATTCTTTTATCATAAAAACAATTGATATGAAAACAAATGTTAGGAACCACATGTATGTAAGTATATATGTCACGATACGAAACTGGGATTCCAATAAAAACATCTTTAGCTAATAACTTATTTCTAGGCTGAGTGAAACACATTTCGTTTCTACAAGTATCGAGACTAGCAGTCGGGTAAACATCATTTATTTGGATTATTCATCAAGTCACATCTCATCTGTATTATAATAGTATATTACAACAGGACAACTCTGAGGCCAAGCAACGAGTATAGAGATACAACAAATAATGAAGTGTACAGTATGGAGACAAATAACAAAGTATACAGCATGGCGACAAATAGCGAAGTATACAGAATGGTGACAAACAACAAAGTGTGGATCTCGGAAGAACTCATTCCCTCCTTTTTTCTCAATAGTTATTTTCTGATCTTAACACCAGGG
PhdEnzCes 06	W4VSJ0	1,95E-06	46,154	PF00135	4059,52	GLYDMLEALKWTNENIAAFGGDNKRITVFGNSAGGISAG	ACCTGCAGATATTCCTCCGGCGCTGTTGCCGAACACCGTAATTCTTTTATTGTCTCCACCGAATGCAGCAATATTTTCGTTCGTCCATTTAAGGGCTTCCAACATGTCATACAAACCTTAGAAACATTTTTCTGGAGTTCCCCCCACATAGCGGGTCCTAAACCTTGAACACTGGCAATTATGATAGACACGAGAGACGACATGATGCTGCTGCTTTGACCTGCACTGTGATATGTCGTACACAAATGCTTGCTTCCTGCTTATAAGAGGAAGAAGATGCTCCTGATTCATTTGCAAATGCACTCAATATGGCTGTCGTTTCTGGATGGCTTTAAACAAAGCAACTGGTCCTTCAAGCAAGCAACTCACTCACTTGGAGTTTCGTCGAGAAGTCACATTGGCTCGTTAACACGAAAAACCA

							AAGAAGGATGGGGCTCGACCTGGACCTGAAGTTCACAGCTC<br>TATAAAATTGAAAAAGTCTGATGGTCATTACCTGAAATAAAC<br>TTCAAGGTAGATGTGTAGTGTGTGGAAAAAACACACTATTGG<br>CATGTAATGAATGCAATAAACGAATAAAATTGCATTGTTTTC<br>CAGTGTACCATGTACTAACGACGTAGAAATGAATGGCAAAT<br>CATGCTTTAAATTTTACTTAATATTATTCATTAATTAAAAAAT<br>ATTCGTTTTATGCAAGAATATTAATAATTTCTTCAATATTTTGT<br>ATTAGAAACAC
PhdEnzCes<br>07	W4VSJ0	3,65E-27	37,349	PF00135	28191,58	MEYFLRPGLYDILGA<br>LKWVNQNIAAFGG<br>DDKRITVFGHSAGGI<br>ATGLLCVSPLSKGLF<br>QRAIMQSGSPAYFL<br>NNYRSRNMQLGQE<br>LAERMGCANENKT<br>LNENPDDVIECLRSK<br>DPNDLMTVTYSLVP<br>VFQEITFFTVTGDNF<br>MPIDPLKAIVQGNF<br>NGVDTLIGNAQDD<br>GSPFLVAFSRQIAGP<br>SGNPVICGRCSLDQI<br>HISKFLKCLKAVQVT<br>WKLTKKIANFSDLIL<br>VFNRDLLKGKSLPIT<br>TICLDCVEDIQI*	TTTTTTTTACACATAAAGGGATATTTATTACTGCAGCGTAATA<br>GTTTAGATTCGTTTTATTTAATATCTTAAACGTGTGTTTCTTAA<br>CGTTTCAGTCATATTTGAATGTCTTCAACACAATCCAAGCAA<br>ATTGTAGTGATTGGTAAAGATTTGCCTTTAAGAAGGTCGCGA<br>TTAAAAACCAAAATAAGGTCGGAAAAATTCGCAATTTTCTTC<br>GTGAGCTTCCAAGTAACTTGTACTGCTTTGAGGCACTTTAAA<br>AATTTCGATATATGGATCTGATCTAGTGAACATCTGCCACAT<br>ATTACTGGGTTTCCAGAAGGACCTGCGATTTGACGGAAAAT<br>GCAACGAGAAAGGGCGATCCATCATCTTGAGCATTTCCTATC<br>AATGTATCAACACCGTTGAAGTTGCCTTGCACGATCGCTTTTA<br>GTGGGTCTATAGGCATAAAATTGTCCCCAGTGACGGTAAAA<br>AATGTTATCTCCTGAAAAACAGGAACTAGGGAATATGTGAC<br>GGTCATCAAATCGTTTGGATCTTTACTTCTTAAACATTCGATT<br>ACGTCATCAGGATTTTCGTTCAACGTTTTGTTTTCATTTGCAC<br>ATCCCATCCTCTCTGCTAGCTCCTGTCCCAACTGCATATTCCG<br>AGATCTATAGTTGTTCAGAAAATATGCTGGAGAACCACTCTG<br>CATGATCGCTCTCTGGAAGAGACCTTTCGAAAGAGGAGAAA<br>CACACAGCAAACCTGTAGCTATGCCGCCGGCGCTGTGGCCG<br>AACACCGTAATTCTTTTATCGTCTCCGCCGAATGCAGCAATG

							TTTTGGTTCACCCATTTAAGGGCTCCCAAAATATCATACAAG<br>CCGGGGCGGAGGAAGTACTCCATTTAAACCTCAGGTGGCAA<br>TTTGGAAAAATAATCGAAACTAAATTAGTTGGCGTAATCAAA<br>CTTATTTAGACGAACGACTGGCACTTCGATCTGAAAATGAGT<br>CAAAGTTTTGGCCGTCATGAGAAAAACAGTATCCACGGAAA<br>ATATTCGGTGTCGGGGTGTCATAAAAATATTAGATTTACAAT<br>GAAT
PhdEnzCes<br>08	W4VSJ0	7,32E-60	46,798	PF00135	28175,38	MSPLVAIVISGDQSL<br>GAAMFGDIQKKVYK<br>SLQLLFLLLPATILSSS<br>VTVNTPTGVVTGSV<br>MDYGSKSLDVFLGIP<br>YAEPPVGNLRFKET<br>VPVKPWTTPVTANS<br>LPPACHQYSPYNYP<br>WEDNNPGQSEDCL<br>YLNIWAPKVSNSNE<br>KKAVMFWLHPGGF<br>SYGSGRLPLYDGRVL<br>AENGDVIVVTINYRL<br>GALGFFTSTSKEAPG	CCACTTTGCATTATTGCTCTCTGAAAGAGACCTTTCGAAAGA<br>GGGGATACACACAGTAAACCTGCAGTAATGCCACCGGAGCT<br>GCTGCCGAACACCGTAATTCTTTTATTGTCTCCGCCGAATGCA<br>GCAATGTTTTTGTTCACCCATTTAAGAGCTTCCAAAATGTCAT<br>ACAAGCCAACGTTCCCAGGAGCTTCTTTCGATGTAGATGTGA<br>AGAATCCAAGCGCTCCTAACCGGTAGTTTATCGTAACAACTA<br>TTACGTCTCCATTTTCAGCTAGAACACGTCCATCATATAGAG<br>GCAGCCTACCTGAACCATATGAAAAGCCGCCTGGGTGCAGC<br>CAAAACATGACGGCTTTCTTCTCATTGCTATTGGAAACTTTTG<br>GTGCCCATATATTCAAATATAAACAGTCTTCACTTTGTCCCGG<br>GTTATTGTCTTCCCACGGATAGTTGTACGGAGAGTACTGGTG<br>GCAAGCAGGCGGTAGGCTGTTTGCTGTTACAGGGGTAGTCCA<br>AGGTTTGACCGGAACTGTCTCCTTAAAGCGTAAATTCCCCAC<br>TGGAGGCTCAGCATACGGAATTCCGAGAAAGACGTCTAGAC

						NVGLYDILEALKWVNKNIAAFGGDNKRITVFGSSSGGITAGLLCVSPLSKGLFQRAIMQS	TTTTGCTGCCATAATCCATAACGGAACCAGTTACTACACCTGTAGGAGTATTAACTGTAACGCTGGATGATAAGATAGTTGCTGGTAGTAATAGAAACAATAGCTGTAAGCTCTTATAAACTTTTTTCTGGATGTCCCCGAACATAGCGGCTCCTAAACTTTGGTCTCCAGAAATTACGATCGCAACGAGAGGCGACATGATACTGCTTTTTGATCTCCACTGTGATATGTCGCATAGGAATGTCTCTATTATTCCCTATTTATAAGAGGATGAGGATACTTCTGTTTACATCTACCACATGATAAAATAAAATCACACGGTAGTTTTGACGCGACACTTCAAGTGTCTTTGAAGCGCATAACTATTGTGCCTTGTTCACTTGAGTTAACAGAT
PhdEnzCes09	B2D0J5	1,73E-24	36,207	PF00135	18876,47	LNIFSPVHEEIDSLVLDGYPVMIYIHDGNWDHGSGSLFPGHMLAASQKVVVVTFNYRLGPLGFLATSDTNSPGNYGMLDQVAAINWVYQNIKVFDGDPEKITVFGSGVGGVSASLLALSRLTKRKIKRVISQNGSPVADWAAISDPRFMYNTTEIVGEGLGCSQRDTAHLMECV	AACACATTCCATAAGATGAGCTGTATCCCTCTGAGAACATCCCAAACCTTCTCCAACAATTTCTGTGGTATTATACATAAATCTAGGATCACTTATAGCAGCCCAGTCAGCAACAGGAGATCCATTCTGAGAAATTACTCTCTTAATCTTTCTTTTTGTCAATCGTGATAAAGCTAAGAGTGAAGCACTGACTCCACCAACTCCAGATCCAAAGACAGTAATTTTTTCTGGATCACCATCAAAGACTTTTATGTTTTGATATACCCAATTGATAGCTGCCACTTGATCAAGCATACCATAATTCCCTGGAGAATTTGTATCACTAGTAGCTAAAAATCCTAGTGGTCCCAACCGGTAATTGAAAGTAACTACTACTTTCTGGGAAGCTGCAAGCATATGACCTGGAAACAAGGAGCCACTGCCATGGTCCCAGTTACCATCATGGATATAAATCATCACAGGATATCCATCTAGAACAAGACTATCGATTTCTTCATGAACTGGAGAAAATATATTGAGA
Hydrolases							

**Transcript ID**	**Uniprot ID reference protein**	**e-value**	**Identity (%)**	**PFAM domain**	**MW (Da)**	**Amino acid sequence**	**Nucleotide sequence**

PhdEnzHyd 01	B6EWW8	8,28E-141	44,234	PF02872<br><br>PF00149	71810.34<br><br>**69028.94**	MEFAVHRIFQFSLVL<br>IISIIVVHS**FNLTLLHT**<br>**NDVHSHFVEFNAF**<br>**GGRCTEKLAKEHK**<br>**CFGGFARQVTKVK**<br>**EVRSSRENVLFLNA**<br>**GDFYQGTVWYTLH**<br>**RWQIVAELVNKLSI**<br>**DVMSLGNHEFDDG**<br>**LKGLFPFLKTLKPK**<br>**VVVCNINASKVPEF**<br>**DEYVHPSTIVEVGG**<br>**ERIGIIGYLTPETKFL**<br>**SSAGDLEFEDEVECI**<br>**RREAKRLATEEGLN**<br>**KIIAVGHSGFAVDQ**<br>**DIAREVPEIDIVVG**<br>**GHTNTFLYTGDPPS**<br>**VELPQGPYPFVITR**<br>**DDGSDALVVQDFA**<br>**FGKYMGYLEVQFD**<br>**NEGRVTAWRGNPV**<br>**LLDASFAEDPETLEI**<br>**LEPYAQEVKRRVRQ**<br>**TVGKTNVLLRGDR**<br>**LTCRMRECNLGNFL**<br>**ADASLDFFIEQPTEK**<br>**GWNFVAIALWNSG**	AATATATATGAAAAACAATTTAAACCTGATTGTACAAGCGAT<br>TATAGAAAATTTCGAATTTTCATTAGAAAAGTCCCCTTCTCA<br>GAAACAAATGCGGTAATGTGCAATAATATTATCCAAATGGG<br>TGTCTCGTTTCTTCATCGTCTTCTTCCTGAAAAAATAGGTATC<br>ACTTTTGGAATTTCAGAAGGAAGTTTCATCAAATCATCCCCT<br>GCTTTAAGGATCCCAGAGCCGAAATCTTTGAAACCGGATGTT<br>ATATCTGTTAAAATATCTTTATCCGCAACTTCTGTTGTGACCG<br>AATCGGTTGGGGAAGTAGTTGCTTCGAGTCCAGTTGAGGTAG<br>TTGCTTCGCGTACAGCTGCGGTCAAGGGGACGGCTTTATCGT<br>CTTGTTCTTCTTTGCTTGAATCCTCATCGTCTCTCGAAGATAG<br>AATGGTAATTCTGTTTTCTAGGCCGGTGATGACAGGCGAACT<br>CTTCGCAGCGTATTTCGTGATAATGTCTACGTCTAGCTCATCT<br>GTATTTCTGTTGCTTATGGCATTCTTTTGAATAACATCAAAGC<br>CATCGCCGCCTTTGGCCATGTATGTATTCATCACCACTTCGTA<br>TACGCTTTCATCTTCAAGTGGTAAATACTGAGGCACTCTGCA<br>ATCTGCGCAACGAGCATATGCCTCTACTAGTCTTGAACCGAC<br>AGGTTGGGACATGTTGTAAGTTAGTCGAAGGCCAGAAACTTG<br>GAGAAGGCTTCCAGGTGGGTCTATGGCATAAGGATCGTAATC<br>TCTGACTGAATGTTCAAGAACTTGTCGAAGGTGCTTGCCCTTT<br>AAAGCTACAACATCCATGGTGTTTCCAAAAGGTGCCACGGC<br>AAGAACATCTTCTAGAGTGATATTTCCTCCATTAATCCTTTCA<br>TCAACAGATGCCCTAATGCCACCAGAATTCCAAAGAGCGAT<br>TGCCACAAAATTCCAACCTTTCTCCGTAGGCTGTTCGATGAA<br>GAAATCCAGGGAAGCATCAGCCAGGAAATTTCCTAAGTTAC<br>ATTCACGCATTCTGCAGGTCAGTCTGTCTCCCCTGAGAAGTA<br>CATTAGTCTTGCCTACTGTCTGTCGAACTCTTCTCTTCACTTCC<br>TGAGCATAGGGTTCTAGTATCTCCAACGTTTCTGGATCTTCTG<br>CAAATGAAGCGTCTAGTAACACTGGATTTCCACGCCAAGCG

						**GIRASVDERINGGN**<br>**ITLEDVLAVAPFGN**<br>**TMDVVALKGKHLR**<br>**QVLEHSVRDYDPY**<br>**AIDPPGSLLQVSGL**<br>**RLTYNMSQPVGSRL**<br>**VEAYARCADCRVP**<br>**QYLPLEDESVYEVV**<br>**MNTYMAKGGDGF**<br>**DVIQKNAISNRNT**<br>**DELDVDIITKYAAK**<br>**SSPVITGLENRITILS**<br>**SRDDEDSSKEEQD**<br>**DKAVPLTAAVREA**<br>**TTSTGLEATTSPTD**<br>**SVTTEVADKDILTD**<br>**ITSGFKDFGSGILKA**<br>**GDDLMKLPSEIPKV**<br>**IPIFSGRRR***	GTCACTCTGCCTTCGTTGTCAAACTGAACTTCGAGATATCCCA<br>TATACTTTCCGAAAGCAAAATCTTGAACCACCAAAGCATCA<br>GAGCCGTCGTCTCTAGTGATTACAAACGGATAGGGTCCTTGT<br>GGTAATTCTACTGATGGTGGATCTCCTGTATACAAAAATGTG<br>TTCGTATGACCTCCAACTACAATGTCTATTTCAGGCACTTCTC<br>TTGCGATATCCTGATCAACAGCGAATCCCGAATGCCCAACAG<br>CTATAATTTTGTTTAAACCTTCCTCAGTTGCCAGTCTCTTCGCT<br>TCTCTTCTTATGCATTCGACTTCATCTTCAAATTCTAAGTCACC<br>TGCTGAAGAAAGAAACTTTGTCTCGGGTGTCAGATATCCAAT<br>AATTCCAATCCTTTCGCCTCCGACTTCAACAATTGTCGATGGA<br>TGAACATATTCATCGAACTCTGGAACCTTGGATGCGTTGATA<br>TTACAGACAACCACCTTCGGTTTTAAGGTTTTCAGGAAGGGG<br>AAAAGACCTTTTAAGCCATCGTCAAATTCGTGGTTACCCAAC<br>GACATGACATCGATGGACAGCTTGTTCACAAGTTCCGCAACG<br>ATCTGCCAGCGGTGCAAAGTGTACCACACCGTCCCTTGGTAA<br>AAGTCACCAGCATTCAAGAATAGTACATTTTCTCGGCTGGAT<br>CTCACCTCCTTCACTTTAGTGACTTGTCTTGCAAATCCTCCAA<br>AACATTTGTGCTCTTTGGCAAGCTTCTCCGTACATCTTCCACC<br>GAACGCGTTAAATTCGACGAAATGAGAGTGCACATCATTCGT<br>ATGGAGCAGAGTCAGGTTAAATGAGTGAACCACAATAATAC<br>TGATAATTAAAACGAGAGAAAACTGAAAAATTCTGTGAACA<br>GCGAATTCCATCTTTTCGCTAGATGAAAGCGCG

PhdEnzHyd 02	J3RZ81	1,57E-37	31,596	PF00151<br>PF01477	41388.84	MSYIPNFFRCFAFMY SCVSLITFSRILLGLFQ NRCIDNLDCFYTGPP FFHPIFRPISVPPTLN LRTQFLLYTRMNPK NPQVLMPTEESLRTS TVDPNLETKILIHGY TVQLLPDDIRFEMK DELLENGPMNVLIV NWTADNQPPYPQA VSNARAVGAQVAQ LINYHVETMRLDPKS VHVIGHSLGGQMSG WIGERVPGLGRISSL DPAGPWFQKAPREV RLDETDANFVDVIH TNGGDNFMEGYGL HEPIGHLDFYPNGG NDQPGCFYEAVKRT DGEINRTTEMIAKSC SHSRANDYFLDSINR CQFAGIQCSSYRDYQ EGKCTPDNSAVALM GYHATKLPTQPEETS FYLLTSEEPPYCGES NEIV*	ATGTCTTATATTCCCAATTTCTTTCGTTGTTTCGCTTTCATGTA TTCCTGCGTATCACTAATCACTTTTTCCCGTATTCTTCTAGGCC TCTTCCAAAACAGATGCATCGACAATTTGGATTGCTTTTATAC GGGTCCTCCATTCTTCCACCCTATTTTTCGCCCCATAAGTGTG CCCCCTACTCTGAACTTGAGGACACAGTTTCTGCTCTATACCC GCATGAACCCTAAGAATCCACAGGTTTTAATGCCAACGGAA GAATCTCTGAGAACTTCAACCGTCGATCCCAATTTAGAGACG AAGATTTTGATTCATGGATACACTGTTCAACTACTACCGGAT GATATACGTTTTGAAATGAAAGATGAACTCCTAGAGAACGG TCCCATGAATGTCTTAATTGTTAACTGGACAGCGGACAATCA GCCTCCATACCCTCAGGCGGTGTCTAACGCGAGAGCAGTGG GAGCTCAAGTAGCCCAGTTGATAAATTATCACGTGGAGACC ATGCGCCTCGATCCCAAATCTGTCCACGTCATTGGACATAGC TTAGGAGGCCAGATGTCTGGTTGGATTGGCGAGAGGGTGCCT GGACTGGGAAGAATATCAAGTTTAGATCCTGCCGGTCCCTGG TTCCAGAAAGCACCACGTGAAGTTAGACTTGATGAAACAGA TGCTAATTTCGTCGACGTGATCCATACTAACGGAGGTGATAA TTTTATGGAAGGTTATGGATTACATGAACCTATTGGGCACTTG GATTTCTATCCGAATGGAGGTAACGACCAGCCTGGATGTTTC TACGAAGCCGTAAAGCGAACAGATGGAGAGATTAACAGAA CTACCGAAATGATAGCGAAATCTTGCAGCCACAGCCGAGCG AATGACTACTTCCTGGATTCCATCAACAGATGTCAATTCGCA GGGATCCAGTGCAGTAGTTACAGAGACTACCAAGAAGGGAA ATGCACTCCCGATAATTCTGCCGTTGCTCTGATGGGGTACCAT GCCACGAAACTTCCCACTCAACCTGAGGAGACTAGCTTTTAT TTGCTGACATCTGAAGAACCTCCTTACTGCGGGGAAAGCAAC GAAATTGTATGAAGAAGCTCAGTGAGCATTCACAGATTATAT TCTGTGACGTAGATAAGGCCGTCGCAGATCTCGTCTTGATGT

							TAAGACAACACTAAATGTTACAGACATTTTTATATTTATAGT<br>ATTCATGCAAAAGTCATGGACCAGACACCTTTGCAACGCAG<br>CACCGTTGGTGAGATTATATCATGTCATCTCCCCAGTGGTTAA<br>TTTTGCCAGCGAGCAAAGAATTCTGTAATCGGTATTATTTAAT<br>CATTTCAACGAAGACTGTGATTACTTGGACACCCATCTTGTA<br>TCAAGAAGCAACTGAGACAGAGGCGTAATTAAAAATTTTAA<br>ACCTTCTCGAAAAATTTTCGCGAGTCTCTCTGAAATGTCTTAT<br>ACAAATATCATTTAAACGATTTCTGGGATATTGAAAGCCCCC<br>TCAGTGCTGTAGAATCGCATTTGCAATTGCTTCTTAGGGGTAC<br>AGTGGTAACACCCCCTCCTCGGGGACCGTTAATGATAGCTGG<br>GGGATAACCCGAGGAGAGGGGTTACTTAACTCGCTAGGACT<br>CTGCTTAACTTCGGTTGCACTTGACATGGCTATTCACATAATA<br>ATGGTTTAGTCCAATAATGTCTCAACACCTTTTAGTCTCCATA<br>GTTCATTTTATTCTTGGCTGAACACTTATGTACACAGTATTTA<br>CATCCACTAGGGAGGGGGAGGGGATTCATGAAGTTCTCGGT<br>CACTCTTGCATATGAACGACATCGAGCCTGGGTGCCATCTTA<br>ATTAACTCCTTGTCAAACACAGTACGATTTACAAGTTGAAAT<br>CAATTACGCGTAAATACTTCCTAAACTCTCTTAAACACTTCTC<br>CTTGGTATCATACACTACATCTCACGTCGTATATGTGCCATCT<br>ACCTATCCTCGAATTATGTTCTGAGAAGCTCAACTTGAAACT<br>CAACCAGCCTCTGCACTCCTTGCGCCCTTCTGAGCTCTAAT

PhdEnzHyd 03	J3RZ81	3,51E-44	31,692	PF00151<br><br>PF01477	57435.20<br><br>**55409.69**	MKSWSAVLILMSAF<br>VLSCG**YKTFLRQILG**<br>**QALMGEWLSVLPT**<br>**KFHCLGTANNFTNF**<br>**LLFTRVNPDDYSPI**<br>**VPFNRDLLHKSDFD**<br>**PTVPTKFIIHGFVDS**<br>**IFLTDWMQRMKDA**<br>**LLSEDDYNVIIVDW**<br>**SCGNEFPYFQAVQK**<br>**SKIVSDELKNLITFL**<br>**MDSTGARTSSFHLI**<br>**GHSLGSHIAARVG**<br>**HAIPHLGRISGLDP**<br>**AGPLFYESTLEDRL**<br>**DPTDADFVDAIHT**<br>**DAGQGLLEGLGLR**<br>**SVAGHLDFYPNGG**<br>**NSQPGCHNSPFSIL**<br>**PRIGIHRAGRYYLT**<br>**CDHFRSTEFYIESIK**<br>**PLLQPRSSDDPMCV**<br>**PVGVACRDWETYE**<br>**QGRCADCVHPGDC**<br>**AVMGYHSDLFFDN**<br>**QPGNTYKDLYLKTS**<br>**EEKPFCLFQYQVIV**<br>**QTACDFESCDEGKY**	CCAAATCGAATTGCGCCTTACTTGCGAGCGGAAAGCGTTGTT<br>GTAGGCTAGTACGCCGAAAATTTCACATCATTTCAAAATGAA<br>GTCGTGGTCAGCTGTTTTGATTCTGATGTCGGCGTTCGTTTTA<br>AGTTGCGGATACAAGACGTTTCTCAGGCAGATATTGGGTCAA<br>GCCCTCATGGGTGAATGGTTGTCCGTTCTTCCGACCAAATTCC<br>ATTGTTTGGGTACAGCAAACAACTTTACCAACTTCTTGCTCTT<br>TACAAGAGTCAATCCGGATGACTACAGCCCTATCGTTCCATT<br>CAACAGGGACCTGCTCCACAAATCGGACTTCGATCCAACGG<br>TTCCGACGAAGTTCATCATTCATGGGTTTGTGGACAGCATATT<br>CCTTACTGACTGGATGCAGCGTATGAAAGATGCCCTTCTGTC<br>TGAGGATGACTACAACGTCATTATTGTTGACTGGAGTTGCGG<br>AAATGAATTTCCTTATTTCCAAGCGGTACAGAAATCAAAGAT<br>CGTCTCCGATGAATTGAAAAACCTCATTACATTCCTCATGGA<br>TTCCACGGGTGCTAGAACCTCCAGTTTCCACCTGATAGGTCA<br>CAGCCTGGGCAGCCACATCGCAGCGCGGGTCGGTCACGCCA<br>TTCCGCATCTGGGCAGGATTTCCGGACTCGATCCTGCTGGAC<br>CATTGTTTTACGAAAGTACCCTAGAAGACCGGTTGGACCCAA<br>CTGACGCAGATTTTGTCGATGCTATTCATACAGATGCCGGGC<br>AAGGTTTACTGGAAGGTCTTGGCTTGCGCTCAGTTGCTGGTC<br>ACTTAGACTTCTATCCAAATGGAGGTAATAGCCAGCCGGGTT<br>GTCATAACTCACCTTTTTCAATCCTACCAAGAATTGGAATCC<br>ACAGAGCTGGTCGCTATTACCTCACTTGCGATCACTTTCGTTC<br>TACGGAGTTTTACATCGAAAGTATTAAACCACTGCTGCAGCC<br>TAGATCTTCAGATGACCCCATGTGTGTACCTGTAGGCGTCGC<br>CTGCAGGGACTGGGAAACATACGAGCAAGGAAGATGCGCA<br>GATTGCGTCCATCCTGGTGACTGCGCAGTAATGGGTTATCAT<br>AGCGACTTGTTCTTTGACAACCAACCTGGAAATACCTATAAA<br>GACCTTTACCTCAAGACTTCAGAGGAAAAACCATTTTGCCTA

						**ETFTPGVLRLEMKVKNGKSTRLVLDDNSEHPMKPGAQYVYLATSRFPVGDVEGAKIWWGYASKPEVEAGPSPYMSGLNLKSVQINPITRLNEIDRFSQPEISVLCAPPNRTIGRNQYLNLSQDYC***	TTCCAGTATCAAGTAATCGTGCAGACAGCATGTGATTTTGAGTCCTGTGACGAGGGAAAGTACGAGACTTTTACACCCGGAGTGCTACGACTCGAAATGAAGGTGAAGAATGGGAAAAGCACTAGACTTGTTTTGGATGATAATTCTGAACACCCCATGAAGCCGGGAGCCCAGTATGTGTATTTGGCGACGAGTCGTTTCCCCGTTGGAGATGTGGAAGGGGCTAAAATTTGGTGGGGATATGCATCTAAGCCAGAGGTCGAAGCTGGGCCTTCACCTTACATGTCTGGTCTGAATCTAAAATCTGTGCAGATAAATCCTATCACGAGACTAAACGAAATTGACAGGTTTTCCCAGCCAGAAATAAGCGTTCTCTGTGCGCCCCCTAACAGGACTATTGGGAGAAATCAGTACTTAAATCTGAGCCAGGATTATTGCTGATATGAAGTCCAAGAGCGCCACTTCCATGACTTTTATACGTGAGAAATGGGCCATATTTATTTTAAAGACTATAGTCATTTCACCAAAGAGATAATTACACAAATATCACTGCTCAACATTGATACTGTTTAGTTTATAGTGATTCAGAG

PhdEnzHyd 04	J3RZ81	1,30E-14	32,624	PF00151<br>PF01477	24106.02	FHLIGHSMGAITCGFISRSLREMMGDDVLVGRITGLDPSVAPYLEAPPEERVTYRDAQTVDIIHSNGGSCYSSASGDIRQLGHADFYPNGGTRQISCYPQAFKSIIKFDIFYGIASIMRENLCFHFQSIQYFKSSINNKECQFTGVESPSYDVFLRESPHSPSESRAVMGMDYTRPQNDQTQRKYYLTTSLLCPYYDDSYLTDRKPQCAFQTN*	TTCCATCTTATCGGCCATAGTATGGGCGCAATTACATGCGGATTTATAAGCAGATCCCTGAGAGAGATGATGGGAGATGACGTCCTTGTGGGAAGAATAACAGGTCTCGATCCTTCTGTAGCTCCTTATCTAGAAGCACCACCAGAAGAGAGAGTGACTTACAGAGATGCCCAAACAGTAGATATCATTCACAGCAATGGTGGTTCGTGCTACTCTTCTGCTTCTGGAGACATACGGCAACTGGGCCATGCAGACTTCTACCCCAATGGTGGCACTCGTCAGATTAGTTGCTATCCGCAAGCATTTAAATCCATAATTAAATTCGATATCTTTTATGGTATAGCAAGCATCATGAGGGAAAATCTCTGCTTTCATTTTCAAAGCATTCAGTATTTTAAATCTTCTATAAATAACAAAGAATGCCAGTTTACAGGAGTAGAAAGTCCATCCTATGACGTCTTTCTGAGAGAGTCACCACACAGCCCGAGTGAATCACGAGCAGTCATGGGAATGGATTACACCAGGCCGCAAAATGACCAAACACAAAGAAAATATTACTTGACAACTAGTCTTCTCTGCCCTTATTACGATGACAGCTATCTTACGGACAGAAAACCCCAATGCGCATTTCAGACAAACTGATAGCATTGTACTTGATCAACCAGGATATTGGAACCATTGTGGCTTTAATTCTTTTGCAGTTTTAAGAGAAAATCATGCCGTGTTATACAGATATGCTACAAATTACTGCATATACAACTACTGCATATTACTGCAAATTACTGCATATGTGGCAAAATATATGATTGTAGCTTAATGAGGGTGAAGGGGGACAATATATGGGGTTCATATCTAAATTTGTG
PhdEnzHyd 05	J3RZ81	5,77E-11	41,818	PF00151<br>PF01477	7914.10	DPKVRLDPSDALFVDIIHTNSPPSTNVPGMLQPAGHVDFYPSPGWRRTGCNIMDGIKRLRRCKAVHATELF	GAAAAGCTCTGTCGCGTGGACAGCTTTGCATCGTCGAAGTCTTTTAATTCCATCCATTATATTACAACCAGTTCTTCTCCATCCAGGTGACGGGTAAAAGTCTACATGTCCTGCCGGCTGAAGCATACCAGGAACGTTTGTAGAGGGTGGGCTATTCGTATGGATAATATCAACGAAAAGAGCATCACTTGGATCAAGTCGAACTTTAGGATCCG

PhdEnzHyd 06	P0DPU3	3,84E-164	47,443		69453,87	MSRDGNLTQTVHGS<br>YVQLNTFDDKENEF<br>VQVRTSYPTLLPYKR<br>CLCTKRDTIIVLLVAI<br>GTCILITASLAIALPKI<br>HEKRQQYYDVSFTES<br>SSPLLTYDKAVVVSD<br>GVNCADIGKDILLK<br>GGSAVDAAIAALFC<br>GGVVNPQSMGLGG<br>GFLMTVYSRKTNSST<br>FIDGRETAPENASKD<br>MFQGNSSLSVEGGLS<br>IAIPGELRGYWAAH<br>EKFGNLTWKELIEPSI<br>QLCTHGFKVSAHLA<br>NHLQNYKEKILSDA<br>YLKEEFSNNETNDLY<br>KEGEILKRPNLGITL<br>NEIANKGADVLYTG<br>DLHETFLKDIRKCGG<br>IITETDLANYKPVIKS<br>AVKVNLTTAEALTL<br>HTVPPPGSGLILSLIL<br>NILSNYNMTAKDFK<br>DIDDAVLMYHRITE<br>AFKFAFAERTQLGD<br>EDFVDISELLTKLQS	GTCATTTTTTCAGTTTGTGTTTACTGTTTTTGTCGTGAAGATAT<br>GTCTTTCAACCGGCTAGTTAAATAGCTTTTGGTTTTAGTTAAT<br>GGTTAATATTTATTCCTAGACAAAGAAATTTCACTGCTTTTAT<br>TGAAATGAGTCGGGACGGTAACTTAACCCAAACGGTGCACG<br>GATCATATGTGCAACTAAATACATTCGATGATAAGGAAAAT<br>GAGTTTGTTCAAGTGAGAACATCATATCCAACGCTTTTACCA<br>TATAAAAGATGTTTATGCACCAAAAGGGATACAATTATTGTC<br>CTTCTGGTAGCTATAGGAACATGTATTTTAATCACAGCAAGT<br>TTAGCTATTGCACTACCAAAAATACATGAAAAACGGCAGCA<br>GTACTATGACGTTTCATTTACAGAATCATCATCTCCACTTCTT<br>ACATATGATAAAGCAGTTGTAGTGTCAGATGGAGTGAATTGT<br>GCAGATATAGGGAAGGATATTTTATTGAAAGGTGGTAGTGC<br>AGTTGACGCTGCCATTGCAGCCTTATTTTGTGGTGGTGTAGTG<br>AACCCACAGAGTATGGGACTTGGAGGAGGTTTTCTTATGACT<br>GTATATAGCAGAAAGACCAATTCCTCCACTTTTATTGATGGA<br>CGAGAAACTGCACCTGAAAATGCATCCAAGGACATGTTTCA<br>AGGGAATTCATCTTTATCTGTTGAAGGTGGGCTATCTATAGC<br>AATACCTGGCGAATTAAGAGGTTATTGGGCTGCTCATGAAAA<br>GTTTGGCAATCTCACTTGGAAAGAACTGATTGAACCATCTAT<br>TCAACTTTGCACTCATGGCTTTAAAGTATCTGCCCATCTAGCA<br>AATCACTTACAAAACTACAAAGAAAAGATTCTTTCTGATGCA<br>TATTTGAAAGAGGAATTTTCCAATAATGAAACCAATGATTTA<br>TATAAAGAAGGTGAGATTTTGAAACGTCCAAATCTTGGGATC<br>ACTCTGAACGAAATTGCGAACAAGGGTGCAGATGTCTTATAT<br>ACAGGAGATCTACATGAAACCTTTTTAAAAGACATTCGCAA<br>ATGTGGGGGAATAATCACTGAAACTGACCTTGCTAACTATAA<br>GCCTGTAATAAAATCTGCTGTGAAAGTAAATTTAACAACTGC<br>TGAAGCCTTAACTCTTCATACAGTTCCTCCTCCAGGCAGTGG

						KKYANSLWQQISDK<br>MTYSTSFYKPIMETV<br>NDHGTAHVSVLAE<br>NGDAVSVTSSINTYF<br>GSLCLSPSTGIILNNG<br>MDDFSSANITNYFG<br>VPPSPANNIVAGKR<br>PLSSMCPTIAVNAD<br>GDVHMVSGGAGGT<br>RMITSSALVMLRTL<br>WLGQDIKYATDAPR<br>IHHQLFPNVIQYEEG<br>FPQVYQDKLKKEYG<br>HLEQLDDHGSVFLG<br>IVRNGEKLQTNVDY<br>RKGGSVSGY*	ACTTATCCTCTCACTTATTCTCAATATATTGAGCAATTACAAC<br>ATGACAGCAAAAGATTTTAAGGATATAGATGATGCTGTTTTA<br>ATGTATCATCGAATTACTGAAGCTTTTAAGTTTGCATTTGCTG<br>AAAGAACTCAGCTTGGGGATGAAGATTTTGTTGATATTAGTG<br>AGTTGTTAACCAAGTTGCAGTCTAAAAAATATGCTAATTCAC<br>TATGGCAACAAATATCAGACAAAATGACTTATTCAACTAGTT<br>TTTACAAGCCTATTATGGAAACTGTGAATGATCATGGAACAG<br>CCCATGTGTCTGTACTTGCAGAAAATGGTGATGCTGTTTCTGT<br>AACTAGTAGTATAAATACTTATTTTGGTAGTCTTTGTTTATCA<br>CCTTCAACTGGTATAATATTAAACAATGGCATGGATGACTTT<br>TCCTCTGCTAACATCACAAATTATTTTGGAGTCCCTCCTTCCC<br>CTGCAAATAACATAGTAGCTGGAAAGCGTCCTCTGTCATCTA<br>TGTGTCCAACCATTGCAGTTAATGCAGACGGAGATGTTCATA<br>TGGTTAGTGGAGGAGCAGGTGGAACTCGAATGATTACATCTT<br>CTGCTTTGGTAATGCTTAGAACATTATGGTTAGGTCAAGATAT<br>TAAATATGCAACAGATGCACCACGTATTCATCATCAACTGTT<br>TCCAAATGTTATACAGTATGAAGAAGGTTTTCCTCAGGTATA<br>TCAGGATAAGTTGAAAAAAGAATATGGACATTTAGAGCAGT<br>TAGATGATCATGGAAGTGTATTCCTTGGGATAGTAAGAAATG<br>GTGAAAAGCTTCAAACGAATGTTGATTATAGGAAGGGTGGTT<br>CAGTAAGTGGGTACTAAACATTTGAAACTTGTGATTAAAATA<br>ACAATGTGCCTTATATCAAACACTGTAACTTTTACTGTGAACT<br>GTTTCTTTTGGGGCACTGGTGATATCTCTCAAGTATTTATTTCA<br>GTATTCATATATCTTTGATATGAAATGTACATTTGATTTTATTC<br>ATCTACTGTGAAATATTTGAAACACTG

PhdEnzHyd 07	P85843	1,34E-11	59,459		58961.27<br>**56886.59**	MVPRILLLLALSALLS<br>LKAVA**CSHHEPNFK**<br>**PGRTTIVHLFEWKW**<br>**GEIAEECESFLGPYG**<br>**YGGVQVSPPNENGI**<br>**VFEPSWKKEVKRP**<br>**WFERYQPVSYKLVT**<br>**RSGNENDFRDMVK**<br>**RCNDVGVRVYVDS**<br>**VINHMTGNIGAGQ**<br>**GTGGSSFDPDVPRY**<br>**DGVPFGPEHFNSRN**<br>**KCPTGSGDIEDYNN**<br>**KDQVRNCKLSGLN**<br>**DLDLGQEYVRNKIA**<br>**GYLNKLVSIGVAGF**<br>**RVDAAKHMWPGD**<br>**LSAVYNKLNDLNQ**<br>**TFFSPGTRPFIYQEVI**<br>**DLGGEAIKADEYV**<br>**GLGRVTEFRFGKHL**<br>**GDIIRKNYNQLLRY**<br>**LTNFGEGWSFVSGS**<br>**NALTFVDNHDNQR**<br>**GHGAGGYGTILTH**<br>**RQSKMYKMAVAF**<br>**MLAWPYGVPRVMS**<br>**SYSWHEIIVNGKDQ**	GAACACATCGCCAGAGACTGCAGGATGGTGCCTCGAATATT<br>GCTTCTCGCGCTGAGCGCGCTCCTTTCCCTGAAAGCAGTTGC<br>GTGTTCCCACCACGAACCAAACTTCAAGCCCGGAAGAACTA<br>CTATTGTTCACCTTTTTGAATGGAAGTGGGGCGAAATTGCAG<br>AAGAGTGTGAATCGTTTTTAGGCCCCTACGGATACGGAGGTG<br>TACAGGTCTCTCCCCCAAACGAAAATGGCATCGTTTTTGAAC<br>CTTCCTGGAAGAAGGAAGTAAAACGTCCCTGGTTCGAGAGA<br>TACCAACCCGTCAGTTACAAGCTCGTCACGAGGAGTGGGAA<br>CGAGAATGATTTCAGAGACATGGTGAAAAGATGCAATGACG<br>TTGGAGTGAGAGTCTACGTAGACAGTGTCATCAATCACATGA<br>CTGGCAATATTGGTGCTGGGCAAGGTACTGGCGGTAGTTCTT<br>TTGATCCTGATGTACCAAGATACGATGGCGTCCCATTCGGCC<br>CAGAACATTTTAATTCTAGGAATAAGTGTCCGACAGGATCGG<br>GAGACATTGAAGATTACAATAATAAAGACCAGGTACGAAAC<br>TGCAAGCTGTCTGGCTTGAATGATCTCGATCTTGGCCAGGAA<br>TACGTGAGAAATAAAATTGCCGGATATCTCAACAAACTCGT<br>AAGCATCGGAGTCGCTGGGTTCAGAGTAGACGCTGCCAAGC<br>ATATGTGGCCAGGCGATCTCTCAGCAGTGTATAATAAGCTAA<br>ACGATCTGAATCAGACGTTCTTCTCGCCAGGAACACGTCCTT<br>TCATCTACCAAGAAGTAATAGACTTGGGTGGTGAAGCTATCA<br>AGGCCGACGAGTACGTGGGTTTAGGCAGAGTAACGGAATTT<br>AGATTTGGAAAACATTTGGGTGATATTATTCGCAAAAATTAT<br>AACCAGCTTCTTCGATATTTAACCAACTTCGGCGAAGGATGG<br>TCATTTGTTTCCGGATCGAACGCATTGACTTTTGTGGACAATC<br>ACGACAATCAACGTGGACATGGCGCTGGTGGGTACGGCACC<br>ATTCTTACCCACAGGCAATCTAAGATGTACAAAATGGCCGTC<br>GCATTTATGTTAGCTTGGCCGTATGGAGTACCCCGAGTGATG<br>AGCAGTTACAGTTGGCACGAGATTATCGTGAACGGAAAAGA

						**NDWIGPPSDDNYTI**<br>**KNVIRNPDLTCGN**<br>**GWICEHRWRQIYN**<br>**MVKFRNVVGFENV**<br>**RHWWDNGYHQIA**<br>**FSRGNKGFIAINNE**<br>**DFPLSQHLNTGLPE**<br>**GTYCDIISGDKNEG**<br>**ECTGLSVEVSSSGQ**<br>**AHIVVDNSLEDPVV**<br>**AIHVEAKL***	CCAGAATGACTGGATAGGGCCTCCAAGTGATGATAATTATAC<br>CATCAAAAATGTCATACGAAATCCTGACTTGACGTGCGGCA<br>ATGGTTGGATATGCGAGCACAGGTGGCGACAGATTTACAAC<br>ATGGTTAAATTTAGGAACGTCGTGGGATTCGAAAATGTTCGT<br>CACTGGTGGGATAACGGATACCACCAGATAGCCTTTAGCCG<br>AGGCAACAAGGGTTTCATCGCTATAAACAATGAGGATTTCCC<br>CCTCAGCCAGCACCTGAATACGGGGCTACCGGAAGGGACAT<br>ATTGTGACATCATTTCCGGAGATAAGAATGAAGGCGAATGT<br>ACCGGACTAAGTGTCGAAGTGTCCAGTTCGGGTCAAGCTCAT<br>ATAGTTGTGGACAATTCGTTGGAGGATCCTGTGGTTGCTATTC<br>ATGTGGAGGCAAAGCTATGAAAATGGCATCACAGTAATCTG<br>TGATTTAAGGAGGACAGACTCACGGAACTTATTACTTTTGTA<br>GGACTCTTAAAGCAGTGGTTATTATGGATTGGTTTCTCAATAG<br>AAGTTTCCTTAATTGGAAGATATAAACTTTTGTGATAAGCAG<br>TTGAGGACCTGTAAAATAAAAATTGAGTTATACGATAAGTCT<br>ACATTTGTTATAGACTTTCAAAAATATTTATTACGTTCCACGA<br>GAGGCTTACATCAAATATGCTGTGAGGTCTTCTCGTAACGTA<br>TATATATATATGTATGTATGTGGTGTATGTCTTTTTTATGTATT<br>TTGAACCAAGATCAGTGTTAATTCAATTAACTTTATAACTCTG<br>AAAATTATATTAAAATGTTCTAACTATAGATTCTGCAATAAA<br>TTTGTGGTCCTTTCTGTATCTAAATTGTATTTTCCCATGGAGTT<br>ATAAAGGATGCATTTAAAAATATTTTTTATGAACTTGAAAAT<br>TAGATATATTTTTTTTTGTATCTAACTGTGCGAATACAGAGCA<br>ACTAGCAAATATTGCATTATGTCACAATGATATATTTATTGAT<br>CTTTGACGAGCTAGCATTCAGATCAATATTATAATAACTGCG<br>AACACTTATCACTTGAGTCTTGGCATGAAAATTTTGCACCAA<br>TCCTCTGGAGAAGCTTCTCTTTCACACCTTTTAAAAGCATTAA<br>AATATCTACTTGTATGATTATATGTAAAATAATCAATAAAAA

							TGACACTCCTTTCAGAGAACTTTGTTCTTATTTATTATTCTCAATTTCTGTCACTCTACGGAAGAGTGACAGAAGAGTGGAACATGTCTGCAACAAAAG

PhdEnzHyd 08	P0DPU6	0	46,409		19738,6	TSTINQYFFLLPSFGS<br>KILSPSTGIVLNDEM<br>DDFSSPNITNMFDLP<br>PSAKNNIKPGRRPM<br>SSMTPMILVDAGGE<br>AKLVLGGNGGTTITS<br>GVLQVAARSLFFGE<br>NIKQSIDAPRIHHQL<br>TPNIMHHEKSFPTAL<br>VDKLRKFGHDAQL<br>WTMGMMSIIMGVQ<br>RGEDGYLYANSDYR<br>KGGEVAGF*	ACTTCAACCATAAACCAGTACTTTTTTCTTTTACCCAGCTTCG<br>GCAGCAAAATACTTTCCCCTTCGACGGGCATCGTCCTCAACG<br>ACGAGATGGACGACTTCTCGTCCCCCAACATCACCAACATGT<br>TTGACCTGCCGCCTTCAGCCAAGAACAACATAAAGCCCGGC<br>AGGAGACCCATGTCTTCTATGACGCCAATGATCCTCGTCGAT<br>GCCGGCGGAGAAGCTAAACTGGTGCTCGGAGGAAACGGTGG<br>AACCACTATCACGAGCGGCGTGTTGCAGGTGGCGGCTCGAA<br>GTCTGTTCTTCGGCGAGAATATCAAGCAAAGCATCGATGCAC<br>CCAGGATCCACCACCAACTGACCCCTAACATCATGCACCAC<br>GAAAAGAGCTTTCCTACGGCACTAGTGGACAAACTTCGCAA<br>ATTCGGACACGATGCTCAGCTGTGGACCATGGGCATGATGA<br>GCATCATCATGGGTGTCCAGAGGGGCGAAGATGGTTATTTGT<br>ACGCCAATTCTGATTACAGAAAAGGCGGCGAAGTCGCCGGA<br>TTCTAAGATTCCGTTCGAAGGACGAGTACCGACCTAGTTGGA<br>CACCCATTGCCTTTGTGAATACATATCACGTGGTTTTAAACAC<br>ACTCGCAAAGTGATGCTATCTATACCTTTGTGAAATCTTCGTG<br>AGAGAGTTGTTACAAGCACTGTGAACTATTTTTTTTTTTCAGA<br>AGTCTATATCAATATCTCTCTCTGCATCTGAAGGAGTGGTCA<br>GTATTAGGTTTGTGGTATCACGTGCCGGTGCATTGCTGCACAC<br>GCCACACCAAAATACCTTTTATCTTCATCTTCATATCGTTGTA<br>CGAATTCCATTGTTCCTCATCTATTCGTGCTGTGTCTGTCTTTA<br>CGGCGGGAGAGTGCTCTTATAATTTCACGCGTTGTATCAGAG<br>CTCCTAAATATCCAGGCTGGATCTCCATCTTGTTGCCCACTGT<br>CGTCGAAACGCACTCCGCGCAGCAGCTTTCTCGCCGATTCGT<br>AAATATGATCTTTAAGCTGCATTTTTGGGTCGGTTCATGTAAG<br>TGGATTTATTTTTTCTCTCTGAAGCGAATGCGTATGCTTACGT<br>GAGGAAACGTTATTCAAAGTAAGAAAGCCTGCCATTCGAGT<br>GAATTTCATTCAACTCTAATATAAATTTTCGTGAGGTAGGGA

							TCGTGTGTTACTCAAATCTGATCAGATAACAGGAAGAATACA<br>GTTTTTAATTTCCTTCTGCGGAATGCTTAGTGTCTGCAAAATG<br>AACCAATTAAGTAACTTTTGAAGAAAAAGAGAATTTCTTTAA<br>ATATCGGGGTAATATTGGCAACCCCTTTTGACGACGTTGGGC<br>AACAAAATGCCCGATGCGCCAGCTCGGATATGTATATAGGA<br>GACTTAGTTTGTACTGTGGGCTCATTTCGTGAATGTGAGCGTG<br>TGTCATCAGTGATTTACTTTTTAAGGTATCGTAAACGCTTGAC<br>CCCTTAACATGATATCTCGAGAACGCATCGAGAAATTTACAT<br>AAAAATTTGAAGGTATGTTTTATGGGCTATTGACAATGAAAT<br>GAACTAAAATCTTGGATGTGGATTTAAATATTTTTTTAAATTC<br>GTAGTCGTTTTTATGAGAAACAGTTAACAAAATTTGGGAAAA<br>TATCCATTTTATCGTGTATCATTGCCCGCCATTTTCTCGCATTT<br>TGAAATGTCTCAGATTTTAGTTCATGTCAAAGACCGGTGATA<br>CGTGTGCCTCGTGTTCGAAGGAAATTAAGATTATTCGATTAC<br>AACTGAGTGTAGAGTGGTAGCCGGTTTGGTAGGAATTCGCTA<br>AAATTGACGAAAAATAACCCATTTTATAAAAATGCGTGACA<br>TGTACAATTAAATTTTCAGTTATTTTCGTTGTTTTAGTTCAAAG<br>TTTACCAAAAAACAGTAAACAAAATGTTCCGAACGTAATCG<br>GATGTCTTAAATCGGTAGAAATGAATGGAGACGCATTTATTC<br>ATTCGAGATGTCAAGTGAACGATGCTAACGTTCGATGAACTG<br>AATGTTCACTGAGCGTTGCCACATTTAGAAAGTAAATATTGT<br>GAGGTTCCGGATGCAAATTATCGAAATATGCATGATTTTGCC<br>ATCACGGAGGATAGCTACAGCAACATTAAAAATAGTAAGCA<br>TCCGACTTCCAAAAATTTAAACCACATTTCGAATGGGCCTCA<br>TTCGTTGTTTTTGAAGTCTGTTATCTGTTCTAATCTTCAGTAGA<br>CATTTTACTGTTTATTTTATTGCACACATTTTCAAACAAAATG<br>TGTTATACGGTGCGTAAAAGTTTCATTATTTACAGCAATATGC<br>AGTTAAAGAACATTAAGGTATATATGTATATCTTCGGAGACG

							ACTTTGTAAGATTATTTGAAATTTTTGAAACATTTTAAAAATTTATTCTGAAAATTATTTCAAATATGAATATTTTAATTGGAAAATTTTGTAAGATCTCGACTGATTTTATTTTTCCGTGAAATTTTGCTGAAGTACCGTTCTTAAATCAGGGAAGTATGGTTAAAATAAAAATTTCGCGAAAAAAAAAATGACCTAAATTTGGAATTTTTTATTGAAAGATCTTGCATAAGTAAATGGAGGGAGGGAGGTGGCCCTCAAACGGTATGTCCTGAGGCAGTGGCGCACTCAGGATTTAATCTTGGAGGGGGTCGAGTTGAGCGGAAAAAAATATTTTCAATGAATTTTCAGTAAAATTTATTAGGTAATTATAAGAATTTGGTTAGAGAATAAAATCTATTTTCTTTTTTTTTTAACAAAGCTCTTGAATTATATTAGTCGTGGTACATTATAGCAAAGCCAATCTGTTTAATCGTGATCCTGTCATATTTTTCTAGTGTTTCTTTTTCAGTGGAGGGGACCATGGCACCTTGTCCCTCCCCTCGAACAAGGGGTCCGCCGGTACGGTACCTACTTCTTCCCAGACTGAGTGTGCCACTGCCCTAAGGCAACACCTGCTAGAAAGCAGGTGTTGCCTAGGGCATGGTCATAGGAATGGAGGAAGCAGGAGCATAGGCAGCTCAAGCCAGTCCCTCTTCATTTAGAATAAAAATTTCCGAACGCAATATTTCACGATGCGTAAATTTCGTACGAAAATTTCTAAAATCAGATTTTAATTATTATTTACTGAGAAAAAAAAACGTTAATTTATTAGCAGAATTTAAGAAGTAATTGGATTGAAAATCAAGATGAATAA

Carboxypeptidases							
**Transcript ID**	**Uniprot ID reference protein**	**e-value**	**Identity (%)**	**PFAM domain**	**MW (Da)**	**Amino acid sequence**	**Nucleotide sequence**

PhdEnzCxp 01	C9WMM 5	1,47E-44	30,687	PF00450	51187.84<br>**49600.87**	MIGAVVFFSILYCAA<br>**SIKHPDEVESLPGL**<br>**NFPLNYRHYSGYLN**<br>**ATYGKFLHYWFVES**<br>**QRSPSTDPVVLWM**<br>**NGGPGCSSLDGFLS**<br>**ELGPLHVSSDGKTL**<br>**YNNTFSWNREANV**<br>**IFLEAPAGVGFSYSL**<br>**NKKYSTDDDTVSY**<br>**DNYVALQSFFEKFP**<br>**EFKKNEFYITGESYG**<br>**GIYIPTLSVRVLTGP**<br>**ANINFKGFAIGNGF**<br>**LDVANLTNSIVFFG**<br>**YYHGLIGPQLWSRL**<br>**EKYCCNGIAAPQYC**<br>**NFDSPASSQCSEAA**<br>**SEVSQIIQGSGLNV**<br>**YNLYEDCAHSSVG**<br>**NSRYLVDKRNMLR**<br>**LFPKSWKPYNLADD**<br>**PPCIDSDNIKKYLN**<br>**QPEVRKALHIPSSV**<br>**QDWDICSLDVEIGY**<br>**KRLYNTMRPQVLQ**<br>**LINSGKLRGLIYNG**<br>**DVDMACNFLGDE**	GATCTTTTTGATCATCCTGACGTCATGCGGTTTACCGGATGTT<br>TTTGTTTATACCGCTGAATAATCAATTGTTAATCATCGTCTCG<br>GGACATCATACGTAATTGTCATCAGTTATTTGATAAGTTTCAT<br>GATGAAACCCTATATCAAAGTTCCATTTCCTTTGTGTCACTGA<br>ACCTCAGTTACGAAATTTTGCAACAGAAAGTCCAAAGTTTAG<br>TCAATTTGCTTGTACCTGTGATAAACAAAAAAAATATTCATTT<br>GTTGCCGATTTAGAATTTTGATAGCGAATTTAAAGGCATTGTT<br>TAAATCAGTTTTAAGGATAAGTGGCTTGTGATTTTAATTTAAC<br>CCATTATCATCTTATTATTTATCTAATCTGTTATGTGAAAGGA<br>AAGAGGAAAGCGCCTTGGTTTTTTACTTTACTGGTGAGAAGA<br>TTACTATTTTAATTCTGTTCATAAGCCTTTCACAATTTTTTTCA<br>AGATGATTGGAGCAGTCGTCTTTTTCTCAATTCTATACTGTGC<br>AGCATCGATAAAACATCCTGATGAAGTGGAATCTTTACCTGG<br>CTTGAACTTTCCTCTGAATTACAGACATTATTCAGGATATCTA<br>AATGCGACTTACGGAAAATTTCTACATTACTGGTTTGTTGAG<br>AGCCAAAGATCTCCCTCTACTGATCCTGTTGTATTGTGGATGA<br>ATGGTGGACCAGGATGCAGTTCTCTTGATGGATTTCTCAGTG<br>AACTGGGCCCTTTGCATGTTTCATCTGATGGGAAAACTCTGT<br>ATAATAACACATTTTCTTGGAATAGAGAAGCAAATGTTATAT<br>TTTTAGAAGCTCCAGCAGGAGTAGGATTTTCATATTCCCTTAA<br>TAAGAAGTACAGTACTGATGATGATACAGTTTCATATGATAA<br>TTATGTTGCACTACAAAGCTTTTTTGAAAAATTTCCTGAGTTT<br>AAAAAGAATGAGTTTTATATAACTGGTGAAAGCTATGGAGG<br>AATTTATATTCCTACACTATCAGTAAGAGTTTTAACTGGGCCA<br>GCTAACATAAATTTCAAGGGTTTTGCTATTGGAAATGGGTTTT<br>TAGATGTTGCAAACTTGACTAATTCAATAGTGTTTTTTGGATA<br>TTATCATGGTTTAATAGGACCTCAATTATGGAGTCGTTTGGAA<br>AAATATTGCTGTAATGGAATTGCAGCTCCACAATACTGTAAT

						**WFANGLGFKVIENYEEWKYNDQVAGFVKYYDRLTYLTVKGSGHMVPQDKPGPAFKMITSFLNNQRL***	TTTGATTCGCCGGCTTCTTCCCAGTGTAGTGAAGCTGCCTCTGAAGTTAGCCAAATCATACAAGGATCTGGCCTTAATGTATATAATTTATATGAAGATTGTGCTCATTCATCTGTTGGTAACTCAAGATATTTGGTAGATAAAAGAAATATGCTTAGACTTTTCCCTAAATCCTGGAAACCTTATAACTTAGCGGATGATCCCCCATGCATTGATTCCGATAACATTAAGAAATACCTCAATCAACCAGAAGTTCGAAAAGCTCTCCATATACCTTCTTCTGTTCAAGACTGGGACATTTGCAGCTTGGATGTTGAAATAGGATACAAGCGTTTGTATAATACAATGAGACCCCAAGTTCTTCAGTTGATCAATTCTGGAAAACTCAGAGGGCTTATTTATAATGGTGATGTAGATATGGCATGTAATTTCTTAGGAGATGAGTGGTTTGCAAATGGTCTAGGTTTCAAGGTCATTGAGAATTATGAAGAATGGAAATATAATGATCAAGTTGCAGGATTTGTAAAATATTATGACCGATTAACATATCTGACTGTAAAGGGTTCTGGCCATATGGTGCCTCAAGATAAACCTGGTCCAGCTTTTAAAATGATCACCAGCTTCTTGAACAATCAGAGGTTGTAAACTAAGCTTACTGTTATACGTGACATAAAAGGTTGTTCCAAATGCTCTCTATAATTTGTAGCAGAGAGACTTACAATATTTACTTCAAGTCCGAAAGGATGAACTTTTGCTGAAAATTTACAGGAAATATTTTTTAGTGTAAAATTTGGTATATATTCCATGCGTCGAAATATAGATTGTAAATAAATTGAAATTTTTTTTTTGAAAAGAAACAAAAACAAA

PhdEnzCxp02	C9WMM5	1,20E-11	33,929	PF00450	13978,33	GQSSDVFEALSEDFMKPVIDSVDAILANESYIDLNIYSGQLDLIVDTMGTLQWVGKLKWPGMKKFNAAPMKPINFGEGRTGGFVKSFKQFSFFWILKAGHMVPEDAPVTALQMLDMILSKKKLKQ*	TGGTCAGTCAAGTGATGTTTTTGAAGCTTTATCTGAAGATTTTATGAAGCCTGTAATAGATTCAGTGGATGCTATTCTTGCCAATGAAAGTTACATTGATTTGAACATCTATTCTGGTCAGTTGGATCTCATTGTTGATACTATGGGCACTTTACAGTGGGTAGGAAAATTAAAGTGGCCTGGTATGAAAAAATTTAATGCAGCTCCTATGAAACCTATTAATTTTGGTGAAGGACGCACAGGTGGTTTTGTTAAATCTTTCAAGCAGTTTAGTTTCTTTTGGATTTTGAAAGCAGGCCATATGGTGCCCGAAGATGCTCCAGTTACTGCTTTGCAAATGCTTGATATGATTTTATCCAAAAAAAAATTAAAACAGTGATTGTATGAGTAGACTAGTACCATACGTAATGCCTTCATTTTCTCAGTGGTCTAAGATGTTATTTAACTAATGGAGATGGATTTTATATGTATATATTGTTTTCGTATACTACTGAGTATGTCTACTAATGTGCGTATAAATAAATTTAGTAAAGATATTTCCATTTTTCAAAATAAATTTACAAAATTTGTGGATGTAGA

PhdEnzCxp03	C9WMM5	3,26E-35	34,630	PF00450	39562.97<br>**37665.66**	MFIFLLALVSSALFSA<br>NA**ESKEAWGYVNV**<br>**RPNAYMFWWLYYT**<br>**DISADYASQPLVIW**<br>**LQGGPGSSSVGYG**<br>**NFMEIGPLDVNLQP**<br>**RNETWLKKCNLLFI**<br>**DNPVGSGFSYVTN**<br>**KSALATTNKQIARD**<br>**LVTTLSSVLQTVAE**<br>**FQKSPLYIFSESYGG**<br>**KMTVDFALELSQAI**<br>**KSKNISCNFKGIAL**<br>**GDSWISPLDSVAT**<br>**WAQYLYATSLLDN**<br>**AGVLSLKPYVVDIQ**<br>**KSLAVGQFKHATD**<br>**LFNEMQEAIGNLTH**<br>**SVDWYNILKDEGP**<br>**APSNNSVNKFPKN**<br>**HRLYKMFNRHVAP**<br>**LYSDKLAELMNGKI**<br>**REKLHDIPKNVTW**<br>**GGQSSDVFEALSED**<br>**FMKPVIDSVDAILA**<br>**NESYIDLNIYSGQ**	GTTTTGGTTCGAGTATTGATTTCATTCCGGTAGAAATACGTTG<br>TCGATTTGTTGTTCAAAGTTGTTTCTTCTGGGAGTTGATCTGCG<br>TGCGATAATTTCAAGTGCTTGTTCACAGTCACATGCTTGTTAT<br>CCGCACGTTTCTTTAGTAATTCTGCCCTGCGTATTGTATTTTGA<br>ATTTTAACAGAAGTTATGTTTATATTCTTATTAGCGTTAGTTTC<br>TTCAGCTCTTTTTTCAGCTAATGCTGAAAGTAAAGAAGCCTG<br>GGGTTATGTTAATGTTCGTCCAAATGCATATATGTTTTGGTGG<br>TTATATTACACAGACATCTCTGCAGACTATGCTAGTCAACCA<br>TTGGTAATATGGCTGCAGGGAGGACCAGGTTCTTCATCTGTT<br>GGATATGGAAATTTTATGGAGATTGGACCACTAGATGTTAAT<br>CTCCAACCTAGAAATGAGACCTGGTTGAAAAAATGTAATCTT<br>CTGTTTATCGATAATCCTGTTGGATCTGGTTTCAGTTATGTAA<br>CTAACAAATCAGCTCTTGCTACTACAAATAAGCAAATAGCCC<br>GTGATTTGGTTACAACACTGTCTTCTGTTCTTCAGACTGTTGCT<br>GAATTTCAGAAATCTCCACTCTACATATTCAGTGAATCATAT<br>GGAGGGAAAATGACTGTAGACTTTGCTTTAGAATTGTCTCAG<br>GCTATCAAATCAAAAAATATATCTTGTAATTTCAAAGGCATT<br>GCACTTGGAGATTCATGGATTTCTCCCCTAGACTCGGTGGCG<br>ACATGGGCACAGTATTTATATGCTACGTCTTTATTAGATAATG<br>CAGGAGTTTTGAGTCTGAAGCCTTACGTGGTTGATATACAGA<br>AATCTCTCGCTGTTGGGCAATTCAAACATGCAACAGACTTGT<br>TTAATGAAATGCAAGAAGCCATTGGAAATTTAACACATTCTG<br>TTGATTGGTATAACATTTTGAAAGATGAGGGTCCAGCTCCAA<br>GCAACAATTCAGTGAATAAGTTTCCAAAAAATCATAGATTGT<br>ACAAGATGTTCAATAGACATGTAGCTCCATTATATAGCGATA<br>AACTAGCAGAATTGATGAATGGAAAAATTCGAGAAAAATTG<br>CATGATATTCCAAAAAATGTTACCTGGGGTGGTCAGTCAAGT<br>GATGTTTTTGAAGCTTTATCTGAAGATTTTATGAAGCCTGTAA

							TAGATTCAGTGGATGCTATTCTTGCCAATGAAAGTTACATTGATTTGAACATCTATTCTGGTCAGT
PhdEnzCxp04	C9WMM5	1,48E-27	55,056	PF00450	10869,23	VRNQDQMAENMYEFLQQFFRLFYEYKNNDFYIIGESYGGKYVPALSYKIHMEGNNSNINFKGMAIGNGLCDPETMMDYGDYLYQLGLVDRKQ	GTCCGAAACCAGGATCAGATGGCAGAAAACATGTATGAATTTCTCCAGCAGTTTTTCAGACTTTTTTACGAATACAAGAACAATGACTTCTACATTATTGGAGAATCATACGGTGGGAAATACGTTCCAGCTCTTTCCTACAAAATCCACATGGAAGGAAACAATTCAAATATAAATTTCAAAGGCATGGCTATTGGCAATGGACTTTGCGATCCTGAAACTATGATGGATTACGGTGATTATCTGTACCAGCTTGGACTGGTTGACCGTAAGCAA

PhdEnzCxp 05	B1P1J3	3,95E-09	25,984	PF00031	51005.23<br><br>**49148.86**	MKSLILFSVFCMVLS<br>VG**QWKEITVDTEEV**<br>**TKTANFAVKYLSLR**<br>**MNSPYHAKLAHVH**<br>**KAEKQIVAGVNYR**<br>**LKLTLGYSTCKKSE**<br>**VKFEEINNCDFQAG**<br>**SFKKCVIKVYRNLQ**<br>**NLYEVTNFDCTKD**<br>**VSKKTVGKNAHHE**<br>**HIEKVMFEDFVAK**<br>**HKKVYEDVDEKLA**<br>**RFKIFQKNLQKIKFL**<br>**NDHERGTAKYGVT**<br>**HFSDWTEEEFRKNA**<br>**LGFRPDLLNTNNIA**<br>**PAAEIPQISIPDSFD**<br>**WRDKKVVSVVKN**<br>**QEQCGSCWAFSTT**<br>**GNIEGQWAIKKKN**<br>**LVSLSEQELVDCDK**<br>**VDQGCDGGLPSNA**<br>**YKEIIRLGGLEGEN**<br>**DYPYEAKDDTCEFK**<br>**KNKVKVYINGSVAI**<br>**SKNETEMAQWLVK**<br>**NGPISIGINANAMQ**<br>**FYYGGISHPWKFLC**	AGTTGCGTGAGTATTGTTTACATCGCACTGCTTGTTCAGATTT<br>TGTTAAAGTGGTATGTAAAATGAAGTCTTTAATTTTATTTAGT<br>GTGTTTTGTATGGTTTTGTCCGTAGGGCAATGGAAAGAAATC<br>ACTGTGGATACCGAAGAAGTAACGAAAACTGCCAATTTTGC<br>CGTAAAATACCTTTCGTTGCGAATGAATTCACCATATCATGC<br>TAAACTTGCTCATGTACACAAGGCTGAGAAACAAATTGTTGC<br>TGGTGTGAATTACAGACTGAAATTGACTCTTGGTTACAGTAC<br>TTGTAAAAAATCTGAAGTAAAATTTGAAGAGATTAATAATTG<br>TGATTTTCAAGCAGGATCATTTAAAAAATGTGTTATAAAAGT<br>ATACCGTAATTTGCAAAATTTGTATGAAGTCACAAATTTTGA<br>CTGCACTAAGGATGTGAGTAAAAAGACTGTTGGAAAGAATG<br>CCCACCATGAACACATTGAAAAAGTGATGTTTGAAGACTTTG<br>TTGCAAAGCACAAAAAAGTTTATGAGGATGTTGATGAAAAA<br>TTAGCAAGATTCAAAATCTTTCAGAAAAACTTGCAGAAAATT<br>AAGTTTTTAAATGACCATGAACGAGGTACAGCAAAGTATGG<br>AGTAACTCATTTTTCTGATTGGACTGAGGAGGAATTTCGCAA<br>GAATGCTCTTGGCTTCAGACCTGATCTTTTGAACACCAACAA<br>CATAGCTCCTGCTGCTGAAATTCCCCAAATATCAATCCCAGA<br>TTCTTTTGATTGGCGGGACAAAAAAGTTGTTAGTGTTGTAAA<br>AAATCAGGAACAATGTGGCTCATGTTGGGCATTTTCCACTAC<br>CGGAAACATTGAAGGGCAGTGGGCCATAAAGAAGAAAAAT<br>TTAGTTTCCCTCTCAGAGCAAGAGCTCGTTGACTGTGATAAG<br>GTTGATCAAGGATGCGATGGTGGATTGCCAAGTAATGCTTAC<br>AAAGAGATTATACGGTTAGGTGGACTTGAAGGAGAAAATGA<br>CTACCCATATGAAGCAAAAGATGATACATGTGAATTCAAGA<br>AGAACAAAGTGAAAGTTTATATCAATGGATCTGTGGCTATAT<br>CTAAGAATGAAACAGAAATGGCTCAGTGGCTTGTAAAGAAT<br>GGACCTATTTCAATTGGTATCAATGCCAATGCTATGCAATTCT

						<u>**SPDNLDHGVLIVGYGVHSYPIFKKTLPFWIIKNSWGETWGEQGYYRVYRGDGTCGLNLMATSSVVD***</u>	ATTATGGAGGGATATCTCATCCTTGGAAATTCTTATGCAGTCCTGATAATTTAGATCATGGTGTTCTCATTGTTGGTTATGGTGTACACTCATATCCTATTTTCAAGAAAACCTTGCCTTTCTGGATAATCAAAAACAGCTGGGGAGAGACATGGGGTGAACAGGGCTATTACAGAGTCTATCGTGGAGATGGAACTTGTGGACTGAACTTAATGGCTACCTCATCTGTTGTAGACTAGAATGTTAGTAGCCATTCTTTGCCTTTTTACTCCTGGTTGAATCTAGAAGTTGTGAAAATTATTTTCCCAGTTAGCAGCTTATTTTAAATAGTGACAGCCAAGTATTTAATTGTTGGTGTGAATTTGTACACAACTTAAAAATAGTTAGTTTTTAAAAAATTGTATATATTGCTCTCTGCTCAATTTCTTCCTATATTATTTAGTAAAAACTTTTTATACAAAAAAAAAA

PhdEnzCxp06	B1P1J3	2,84E-09	25,984	PF00031	25612,25	LLLLKFPKYQSQILLIGGTKKVVSVVKNQEQCGSCWAFSTTGNIEGQWAIKKKNLVSLSEQELVDCDKVDQGCDGGLPSNAYKEIIRLGGLEGENDYPYEAKDDTCEFKKNKVKVYINGSVAISKNETEMAQWLVKNGPISIGINANAMQFYYGGISHPWKFLCSPDNLDHGVLIVGYGVHSYPIFKKTLPFWIIKNSWGETWGEQGYYRVYRGDGTCGLNLMATSSVVD*	AGTTGCGTGAGTATTGTTTACATCGCACTGCTTGTTCAGATTTTGTTAAAGTGGTATGTAAAATGAAGTCTTTAATTTTATTTAGTGTGTTTTGTATGGTTTTGTCCGTAGGGCAATGGAAAGAAATCACTGTGGATACCGAAGAAGTAACGAAAACTGCCAATTTTGCCGTAAAATACCTTTCGTTGCGAATGAATTCACCATATCATGCTAAACTTGCTCATGTACACAAGGCTGAGAAACAAATTGTTGCTGGTGTGAATTACAGACTGAAATTGACTCTTGGTTACAGTACTTGTAAAAAATCTGAAGTAAAATTTGAAGAGATTAATAATTGTGATTTTCAAGCAGGATCATTTAAAAAATGTGTTATAAAAGTATACCGTAATTTGCAAAATTTGTATGAAGTCACAAATTTTGACTGCACTAAGGATGTGAGTAAAAAGACTGTTGGAAAGAATGCCCACCATGAACACATTGAAAAAGTGATGTTTGAAGACTTTGTTGCAAAGCACAAAAAAGTTTATGAGGATGTTGATGAAAAATTAGCAAGATTCAAAATCTTTCAGAAAAACTTGCAGAAAATTAAGTTTTTAAATGACCATGAACGAGGTACAGCAAAGTATGGAGTAACTCATTTTTCTGATTGGACTGAGGAGGAATTTCGCAAGAATGCTCTTGGCTTCAGACCTGATCTTTTGAACACCAACAACATAGCTCCTGCTGCTGAAATTCCCCAAATATCAATCCCAGATTCTTTTGATTGGCGGGACAAAAAAAGTTGTTAGTGTTGTAAAAAATCAGGAACAATGTGGCTCATGTTGGGCATTTTCCACTACCGGAAACATTGAAGGGCAGTGGGCCATAAAGAAGAAAAATTTAGTTTCCCTCTCAGAGCAAGAGCTCGTTGACTGTGATAAGGTTGATCAAGGATGCGATGGTGGATTGCCAAGTAATGCTTACAAAGAGATTATACGGTTAGGTGGACTTGAAGGAGAAAATGACTACCCATATGAAGCAAAAGATGATACATGTGAATTCAAGAAGAACAAAGTGAAAGTTTATATCAATGGATCTGTGGCTATATCTAAGAATGAAACAGAAATGGCTCAGTGGCTTGTAAAGAATGGACCTATTTCAATTGGTATCAATGCCAATGCTATGCAATTC

							TATTATGGAGGGATATCTCATCCTTGGAAATTCTTATGCAGTCCTGATAATTTAGATCATGGTGTTCTCATTGTTGGTTATGGTGTACACTCATATCCTATTTTCAAGAAAACCTTGCCTTTCTGGATAATCAAAAACAGCTGGGGAGAGACATGGGGTGAACAGGGCTATTACAGAGTCTATCGTGGAGATGGAACTTGTGGACTGAACTTAATGGCTACCTCATCTGTTGTAGACTAGAATGTTAGTAGCCATTCTTTGCCTTTTTACTCCTGGTTGAATCTAGAAGTTGTGAAAATTATTTTCCCAGTTAGCAGCTTATTTTAAATAGTGACAGCCAAGTATTTAATTGTTGGTGTGAATTTGTACACAACTTAAAAATAGTTAGTTTTTAAAAAATTGTATATATTGCTCTCTGCTCAATTTCTTCCTATATTATTTAGTAAAAACTTTTTATACAAAAAAAAAA
Phospholipases A2							
**Transcript ID**	**Uniprot ID reference protein**	**e-value**	**Identity (%)**	**PFAM domain**	**MW (Da)**	**Amino acid sequence**	**Nucleotide sequence**

PhdEnzPA201	A8CG90	1,52E-23	36,508	PF00068	17205,8	GRWCGVFSIVLFLLLRGHYASVIDLGNMLRLMTNRNPLDFINYGNWCGLGGSGYPLDELDRCCKIHDFCYDKASASTCLDEKPHVASYKWKYAAGKIHCNKRDNPCGLSTCRCDGKFAACAQVHQSSYDSRRKRTRTPILNLLSNAFTRNKK*	TTTTCTTTTATTTTTTATTTCTAGTAAATGCATTTGATAATAAGTTTAAAATTGGAGTTCTTGTTCTTTTCCTTCGTGAATCATATGAAGACTGGTGAACTTGAGCACATGCTGCAAACTTTCCATCACATCGACAGGTTGACAGACCACAGGGATTGTCACGTTTATTGCAGTGTATTTTCCCAGCTGCGTATTTCCATTTATAAGATGCCACGTGAGGCTTTTCGTCTAAGCATGTCGAGGCGGAAGCTTTGTCATAACAAAAGTCGTGAATCTTGCAGCACCTGTCCAATTCGTCAAGGGGATATCCACTGCCTCCGAGACCACACCAATTCCCGTAGTTTATGAAGTCCAAAGGATTCCGATTCGTCATTAATCGTAACATGTTTCCCAAGTCAATCACCGAGGCATAATGACCCCGCAGTAAGAGGAACAAAACGATCGAAAACACACCGCACCACCGTCCGG
PhdEnzPA202	P0DPT7	1,32E-22	40,336	PF00068	19065.31<br>**16847.70**	MSSAGWIFVGLLFISPFYEA**AEVKDESTVAKSRQRRSLVNLKDMVKAVTGRDGEDFINYGNWCGLGGSGRPVDPVDECCQNHDFCYDLATDGVCEEHGEKGVYQLEYKWRVADDGTAVCNDEKDQCKMAICFCDAIISNCLKSNIDAYNPENFHDLDIFQMIKDADWISS***	TCTGGATATATATATATTTCTTTTTAAAGTATATTCATTTAGAAATAAGAATACTTCGCATAACGCTCGTTTAAAATTTTGCAGTTATAATAATCTCGACACAGTATTATAACCGAAAGAATAAGTTAAAAAATTTGTCAAGTATATTCACACAGTTCTTGACATGAATTAAATAAAACAGCTTTATAGTAAAACAGACACTTTTTTTCGGACAAGGAAAAACAATGAGTATCACATGAAAACATTAAGACCGTTTCGTCATATATCATATGAGAAAGGTCCCGTTATATAGGTCATAATCAGCAGCTGATATCTCGGAGGATGTGATGTAGTTCGTTGTCTTTTAGGTGTTTATGACGTCAGGTCTAAGAGGAAATCCAGTCCGCATCCTTGATCATCTGGAAAATGTCCAAGTCGTGGAAATTCTCGGGGTTGTAGGCGTCGATATTACTCTTCAGGCAGTTCGAGATGATGGCGTCACAGAAGCAGATCGCCATTTTGCACTGATCCTTTTCATCGTTGCAGACAGCCGTTCCATCATCGGCCACTCTCCACTTGTATTCTAATTGGTAGACACCCTTCTCGCCGTGCTCTTCACACACCCCGTCAGTCGCCAAATCGTAGCAAAAGTCGTGATTTTGACAGCACTCGTCAACGGGATCGACAGGCCTCCCGG

							ATCCCCCTAGTCCGCACCAGTTTCCGTAGTTGATGAAGTCCTC<br>CCCGTCACGACCGGTCACCGCTTTCACCATGTCTTTCAGATTC<br>ACGAGGCTGCGCCGCTGCCTCGACTTTGCGACGGTGGACTCA<br>TCTTTCACCTCAGCTGCTTCGTAAAAGGGGCTTATGAAAAGT<br>AGCCCTACGAAAATCCAACCAGCAGACGACATTTCCAG
PhdEnzPA2<br>03	Q7M4I5	2,16E-35	43,478	PF05826	38193.93<br><br>**36006.20**	MVLLVNVFVLTLAW<br>TFVHC**EDQRDKLFL**<br>**IEKSSGDETKTLLV**<br>**VTWTESEDEIDAGC**<br>**EIFTDKELINEVLKS**<br>**SDAVNIRTLSADEM**<br>**ENLLGDCTEFTLKR**<br>**RRRESDDELPTTISA**<br>**RDITDDPQELRRRR**<br>**NRQRAPQNKRAEG**<br>**RNSRRVNSNNAKE**<br>**NSIETTTDETTLEYA**<br>**DAPATTPGYDGWP**<br>**VIFPGTKWCGAGDI**<br>**AKGYDDLGLHQDT**<br>**DRCCRAHDLCNDT**	AATCAACAAGAGTTTATTGTAACATTTCAACAGAATTTATAT<br>AAAGTGTAAACAGTATGTACAAAAGAAAAAAAGTAGTTTTC<br>TGAAACTCAACCCAGTTTTTTTTTCACAAAGTCAGTCATACC<br>GGGCGAGTTTATTAAAATGGCAGAGTTATCGTCAGAGGTCCC<br>GGGAAAGGAATCTTCTTGTACTTTTTGGCTGCAACCCACTGA<br>TATACTGGTTTAGAATTTGTGTCTTTTTCGTAGTCAAGGCATTT<br>GATCTTCAGAAGTGTCCTGTATTTTATACACTTCGACGTTCTG<br>GGATAATCCTCCTGGTAGCATCCTCTTCTCAGAAGGTTGAAG<br>TACATATTGCCGATGGTGTTGGATGTGACTGAATTTACGCGA<br>TCCAGGCAGCTGTAGAAATCGTTATCGCATTTGCAGCTTAAC<br>TTTGTGAAAGGTGATTTGTTGGTGAGGTTGTTTCTCGTTTCTCC<br>AGGAGCTAAAGTATCGTTGCACAAGTCATGCGCCCTGCAAC<br>ATCGGTCAGTGTCTTGATGCAGCCCCAGATCGTCGTAGCCTTT<br>TGCTATGTCTCCAGCTCCACACCATTTAGTACCTGGGAAAAT<br>TACTGGCCATCCATCGTATCCTGGTGTAGTTGCAGGGGCGTC<br>AGCGTACTCTAGTGTTGTTTCGTCAGTCGTCGTTTCTATAGAG

						**LAPGETRNNLTNKS**<br>**PFTKLSCKCDNDFY**<br>**SCLDRVNSVTSNTI**<br>**GNMYFNLLRRGCY**<br>**QEDYPRTSKCIKYR**<br>**TLLKIKCLDYEKDT**<br>**NSKPVYQWVAAKK**<br>**YKKIPFPGPLTITLPF**<br>*****	TTTTCTTTAGCATTATTAGAATTTACTCTGCGTGAATTCCTTCC<br>TTCTGCTCTCTTGTTCTGGGGCGCCCGCTGCCGATTTCTTCTTC<br>GCCTGAGCTCTTGGGGGTCGTCTGTGATATCCCTTGCTGAGAT<br>TGTTGTCGGCAGTTCATCGTCGCTTTCTCTCCTTCTCCTTTTTA<br>AAGTGAATTCTGTGCAATCTCCAAGAAGGTTTTCCATCTCGTC<br>TGCAGAGAGTGTACGTATATTCACGGCGTCAGATGATTTCAG<br>AACTTCATTGATGAGTTCTTTGTCAGTAAAAATCTCGCACCC<br>GGCGTCAATTTCGTCTTCGCTCTCTGTCCACGTAACTACCAGG<br>AGAGTTTTGGTCTCATCTCCTGATGACTTTTCGATTAAAAACA<br>GCTTGTCTCTTTGGTCTTCACAGTGAACAAACGTCCATGCCA<br>GAGTGAGTACAAAAACGTTGACTAGAAGGACCATAATTACT<br>TCGCTAGGCACGCGTACGAAATTCCACCGAAAACTCAGTCA<br>ATTTTTCAGATATTTTTTTCTACTTCGAAAGGCTTCTCTCA
PhdEnzPA204	P80003	3,49E-30	38,732	PF05826	30670.16<br><br>**26972.62**	MTMLKNSRGGSLAT<br>LLILLFVYVFEGLLSK<br>TVKA**QFEDYAPRPV**<br>**LFCDGKTLAEVILD**<br>**ENGNMEHCRLHEL**<br>**EEDKDAEPIIKGTG**<br>**LPTKYPSFKSMLDLI**<br>**NNCTKLAMPPTNF**<br>**TEDSPTNTTGVSSW**<br>**SPWSLWNGIVPGT**<br>**KWCGVGDIADSFE**<br>**QLGTEFDVDACCR**	CGGGTTGAAACCTGGGAATGACTATGTTAAAAAACAGCCGA<br>GGTGGATCCTTGGCTACGTTACTTATTTTGTTGTTTGTGTACGT<br>CTTTGAAGGCTTGCTGTCAAAAACTGTCAAAGCTCAGTTTGA<br>GGACTATGCGCCGAGGCCTGTGTTATTCTGTGATGGAAAAAC<br>TTTAGCAGAAGTGATCCTTGACGAAAACGGAAATATGGAGC<br>ACTGCAGATTGCATGAATTAGAGGAAGACAAGGATGCAGAA<br>CCCATCATTAAAGGCACCGGACTTCCAACCAAATACCCGAG<br>CTTCAAGAGCATGTTAGATCTTATCAACAACTGCACAAAACT<br>AGCCATGCCCCCAACGAATTTCACCGAAGACTCGCCCACCA<br>ACACTACAGGTGTAAGCTCATGGTCACCCTGGAGTCTGTGGA<br>ATGGAATTGTTCCCGGTACAAAATGGTGTGGAGTTGGAGACA<br>TAGCTGATTCTTTCGAGCAATTGGGCACAGAGTTTGATGTCG

						**AHDHCPVKLKAFR**<br>**TGYGLINLSLYTKS**<br>**HCECDREFYECLKN**<br>**SRNKLADAVGNFY**<br>**FNFLRVQCLREHRP**<br>**FVCVENRTEVDGG**<br>**QRCVRWIPDPNSK**<br>**KMQVTITPLKY***	ACGCGTGTTGCAGAGCCCATGACCATTGTCCAGTAAAGTTGA<br>AGGCATTCAGAACAGGATATGGTCTCATAAATCTTTCCCTTT<br>ACACTAAATCTCACTGTGAATGTGACAGAGAATTCTACGAAT<br>GCCTAAAAAATTCAAGAAATAAACTTGCAGACGCAGTTGGA<br>AATTTTTATTTCAACTTTCTGCGTGTACAGTGCCTACGAGAAC<br>ACAGACCATTCGTATGCGTGGAAAATAGAACAGAAGTAGAT<br>GGTGGGCAGCGGTGTGTAAGATGGATTCCAGATCCGAACAG<br>CAAAAAGATGCAAGTCACCATAACACCGTTGAAATATTAAG<br>CATCTAACAAAAGCG
PhdEnzPA2<br>05	Q7M4I6	1,62E-31	49,635	PF05826	34387.88<br><br>**32820.91**	MLAFLFAYLLALGA<br>A**DWKDVKDKQPLY**<br>**QKSTWRIYLDGSG**<br>**NRMVLTTEKNGVI**<br>**RDCYLSGDRYLIKS**<br>**ARRKISEAHMIRLG**<br>**EKSMNALINFCTYS**<br>**KFKHTFVGSSGEEV**<br>**WQEFGILSGLFIYPG**<br>**TKWCGAGNIAESY**<br>**DDLGPEIETDRCCR**<br>**DHDHCNDSIEAFG**<br>**SKHGLDNNAPFTK**<br>**SHCTCDIKFYDCLR**<br>**EANTTASNRIGRIYF**<br>**NYIQIQCFKLEYPII**	GTAAAGTCGTTGTAAGGTTTGCGAAATCCAATTTCAGTTTTAA<br>ATGGTTTTTCGCAACATTTGCAAAACAATATGCATGTTTCCTC<br>GCGGACTGAAAATCTAAAAACCTAACTCGGCGTTTCAAAAT<br>CACTGCGCATCTGTAAACAGTAATTTGACCTTACCGCAATAT<br>ATAACAAATCGTAATGTCGTAACCTAAATAAGATGATAAAG<br>TATTCGAGAGGAGATTGCCCATAAGGCTACGTATGCGGGCAT<br>AAAACACCCGCACGTATGGATAATTATTTCTCCCTCCTCCTTG<br>GAATGTAATAGTCACGGTGTATAAATACACGATAAAAGTTG<br>CCGGGAATCGGATAATTTTTGTCTGTTATGTTCTTATAAAATG<br>CTAGCTTTTCTTTTTGCCTATCTGTTGGCACTGGGAGCTGCCG<br>ATTGGAAAGATGTAAAAGATAAGCAACCACTATATCAGAAA<br>AGTACATGGAGAATATATTTAGATGGCAGTGGAAATCGAAT<br>GGTTCTTACAACGGAGAAAAATGGCGTAATACGGGATTGTTA<br>CCTATCCGGTGACAGGTACCTCATCAAGTCTGCGCGAAGAA<br>AGATATCCGAAGCGCATATGATAAGACTGGGTGAAAAGTCG<br>ATGAACGCATTAATAAACTTCTGCACCTACTCCAAGTTCAAA

						**GCNKHKGWVLPRCQEYELDEEQDKKWQFFDAKYYNSKQDNNEIKIGQEDVFDFILMCSHVKCNHEYGVENKGFYSS**	CATACCTTTGTTGGCTCTAGCGGGGAGGAAGTTTGGCAAGAATTTGGAATTCTCAGCGGTTTGTTCATCTATCCAGGAACAAAATGGTGTGGTGCTGGTAACATTGCCGAAAGTTACGATGACCTCGGGCCAGAGATCGAAACTGACCGCTGTTGTCGGGATCACGATCACTGCAACGACTCAATAGAAGCATTCGGCTCTAAGCATGGATTGGACAACAATGCACCTTTCACAAAATCCCACTGTACTTGTGATATAAAATTTTATGACTGCCTCAGAGAAGCTAATACGACGGCCTCAAACAGGATAGGCAGAATATATTTCAACTACATCCAAATACAATGCTTCAAACTGGAATACCCTATTATCGGCTGCAATAAACACAAAGGATGGGTTCTTCCTCGATGTCAAGAATATGAACTGGACGAAGAACAAGATAAAAAATGGCAATTTTTTGATGCGAAATACTACAACTCAAAACAGGACAACAACGAAATAAAAATAGGGCAGGAAGACGTCTTTGATTTCATCCTAATGTGCTCTCATGTGAAATGTAACCATGAATATGGAGTTGAAAATAAAGGTTTTTATTCCAGCA
Kinases							
**Transcript ID**	**Uniprot ID reference protein**	**e-value**	**Identity (%)**	**PFAM domain**	**MW (Da)**	**Amino acid sequence**	**Nucleotide sequence**

PhdEnzKin01	Q58L96	1,48E-05	36,111	PF00008<br>PF00594<br>PF00089	215443.72<br>**213105.83**	MLIRNILFIGLFFLTA<br>SLVSS**DRVQIDESLG**<br>**RFLGTLERYANTVN**<br>**DVVFLLDESGSIGV**<br>**DKFPQVKLFAKLTA**<br>**QRLSVSAEHSRIAV**<br>**VTFATETITHIDYIK**<br>**NPYGNNMCTLLKEI**<br>**DDIGYVAEWTHTD**<br>**KAMREADKILAQA**<br>**RPDSNKIIILLTDGQ**<br>**SNDGHSPVAISNSL**<br>**KARGIIIFAIGVAEV**<br>**NVAEINAVATSPD**<br>**HIYILKDFDYIVKV**<br>**NTYLVNDTREISMD**<br>**LAKNPSYCNSLCTE**<br>**HQDCCDEHANCYC**<br>**GTRTGRYECACHA**<br>**GYHGRGGYKGQC**<br>**HKCPKGTYKSESRN**<br>**AECTACPAHSTTDR**<br>**EGSTNFKDCVCETG**<br>**YEKIGNMCAPVRC**<br>**ASLSAPDGGILIPTH**<br>**CNNTYKTQCSFKC**<br>**REGYCQHSCKEYM**<br>**ALQSMNQHPPQLS**	TTTTTTTTGAACTTAAAATTACAAATTTATTTCAGATCTCATA<br>AAAGTATAGTACATTTCGAAATATCACATTTACAATATGAGC<br>AAAATATATACTTCTATTTTCTTACCACTCGAGAAAAAATAC<br>AGGAGAGATGAATTTTTTTTCCGCTTTTCAGATCTTGGGGACA<br>ATAATTGTAGCAGCGGTTTTCAGAAGCCCACTGAGCAACAG<br>ACCAGTTTTAATCACGAAAGACATTCATCTTGAAATGACTCA<br>CAAAGACGTTTGCCCACATAAAGATCACAGAAAGCGCATGA<br>GAATGAATGGTAAATAAAATCAGATTAGAAAGAGAGAAGA<br>TTCCCCGTTCTAAATTGTGGATTTCTTTTTTCTCATAGAGAATA<br>TAGTGATCAATCCACGGTCATAGTTACATCCTCAGTTAATGTT<br>TCAGTCAGTCACACAACCAGTCATTGTTCACGGCATTTAATG<br>TAAAGTCAGTCTTTCGCTAAATGATTCTCTTTCACAGTTATAG<br>AATTATATATAGCACATTTCGAAAATACATAGAAAATTGTTA<br>GAAAGTAACGAAACAAAATTTCTATGTTATAATTGAAAAAA<br>AAATTGGAATCCTACGTCAGTTTTTCAGTAATCCTACGTTAGA<br>AACTTTGATCCTGTAAATGTTATCAGTGCAAATAGCAAATAC<br>ACAAGAAGCAAACTTGCAGATGAAAAGTCTCTGGATGAACC<br>GCTGGTGAAGACGTTTTCTACATAACCTTCCGAGATAATATA<br>ACATTCATTTTCTCCTGTAGCCCATCTTTCTGACAGGTTGAAT<br>TTTCCGCTGCTGTACTTGTGGTATTCTTCTAACTTGCTGCAGAT<br>TCCGCACATCTTGCTGACGTGCGTGCTGGGTATTGAGATCAC<br>AGCCTCGTTTGTGAAATCCCAGTCCACCATTAAATCGGCGTT<br>CGTCTTCACGGAAATGCTGGAGTTCACTTCTTCGACATTCCAC<br>AATTCGTTCTCGAAAGGCACATCCTCGTTGACACCGTCGATC<br>ATTACGCTGCCGTCCGAAGAAATCGTCATCTTTTGGCAGTCTT<br>GGTAAATAACTAAGTTTGTTCTATTCGGCGGGAATTGGACCT<br>CGACCTTGAACCGCCCACCGTAACAGTCCTCGACGGCGGTAT<br>AGTTGCACCCACCTTGAATTTCGTACTTAGAGAAGTCAAAAG

						**RTCLESREWSGEEF**	CCATGATGCTGTCGTTGAGAGAGATCACGCAGAAGGCCCAC
						**HCEKMTCPALRFPH**	GCTACACATTTTGGACAGCATTCTCCAGGGGAGGTCATCAAA
						**NGMHNCSEKDFTF**	GTTTGTTGTGGGAGGCAAGAAGTTTCATTGCAGAATATGTCT
						**GTACSFQCNKGYEL**	TCACATTGCACGATTCCGCTATCGCAAATGCAGTTCTGACAG
						**SGSKIRTCLFNSWT**	TCTTTGACCCAAGTTTCGCCATTCACATAGGTCTCACCGTGGT
						**GDEATCTGIFCDPL**	CCAGGCAATAACCGGTATCCTTGCATTTCTTCCCATCGTCCGA
						**RTNARLHFKRTVC**	CATAATCATATCACTGGGACATTCACAGTGATACGAACCAGT
						**GTNPMPYRSICQYH**	TCCCACATCGTTCACGCAAATGTATTCACAACCGCCATTCTG
						**CAAGYILMANGNK**	ATCCAAGCATTCGTCGATATCGTATTCACAGCGTCTTCCTTCA
						**TEGVFTCEANKKW**	AACCCTGAATCGCAGCTGCAGAAGGAACTCTCGGCACCGGT
						**SKGYKDISCQDNEP**	GACACAAGGCTGTCCGTTTGAGCAAGCACCGTCATCGCAGG
						**PTIQCPKSMTVDAE**	CGTTAACGTTGTTCTTGCAACTGTGTTCTTTCTCCTGACTTGAG
						**AHKGNALVKWDSP**	TAGAAACAATCGCAGCTGTTTTCTGGGCAATGTCCAATATCT
						**KATDNVVGAVVK**	TTGCACTTATCGACATCCTGAGATATCTGTACTTCTCCGCTGA
						**VRIAEPKNLQKSPH**	CATACTGGCTCACAATGTACCAAGGGATCACATTTCCGACGA
						**YKKNKPSFYFPVGK**	AGCCACACGACAACGATATCTTCTTGATTTCCTCAGTTGTCAT
						**TKVVYEATDHSKLS**	GGCGTAGTCCCACACGTTAAATTCTGTAATATCACCGGCGAA
						**STCSFSVEVLDHESP**	TGCCTCTAGGACACTAAACATACTTCCAACGTCATCTTGTTCT
						**KVVFCPEDIEIYHNT**	TGGCCAAGGACAAAGATTCCTGGATGCATGTATTTATTTTCTC
						**NDPVPVEWEEPIFE**	CAACGTTCTTACCATCGGTAATGGTCTTCCCATCGACGTAGA
						**DNSKTLKSVESSRK**	AAGTCCATACTCCTCCCTCTTTATTCCACGTTATTGCATAATG
						**SGDKFGWGPAATV**	GTGCCAAGAGGAATCTCTGTTTGCAGAAAATTCTGTATGGAA
						**YYNATDAANNSAG**	TGTCTGCCCATGTATGTACAATACGAAGTTATTGGTGTCCAAT
						**CSFQVKVKHYPCPF**	AAAGTGAGAGCATTGTCTTCGAGATGATCTTGGTACTTATAG
						**YPPPANGVLTCDN**	GCGTAAGACACGGGTGTTGCTCGGCTGTTTGGATCCTTGAAA
						**WQDGRMCTVFCH**	CTTGTTTTCATGAAGAAAGACACTGTGATTGCGTACAATCGA
						**DGFQFAAGKGVER**	CGAACGTTATTCACCTCCACGTAGTTGGTGGTGGAGGGTGTG
						**NYYCKQGMQDGKI**	GGAAACGTTAAAGTATACTCGTGCATCAGTGCTTCTTCACAG

						**VGNWVVFRVGRK**	CGTGGTCCAGTAAACTTCTGCCGACAGGAACATGTTGCATTT
						**VKMPWPDCTIKQK**	GCGATTCCGTCGACACAGGTGCCGCCATTTTGGCATTTATGA
						**PASLNVTVGMNFG**	TCTTGGCAGTCGTCAATATTAACTTCGCAGAACTTCCCGATG
						**VDSCSEESLKKFRE**	AATCCCGGTCGACACGCGCAGTGATATTCGTCCACCCCTACA
						**KFYNIDAFKNDFFC**	ACACCTCCGATGCAGGTCCCACCGTTTTGGCAGTTGGCATTA
						**PKDRGCTPENVNIT**	TCACAAGCATCCGTGATTAATTCCTCTTCGCAGTTAGCACCGT
						**CSPVTTRARRSLRA**	GAAAGCCTTCAGTGCAACAGCACCTGTAGCCATTCTGCATAT
						**DSFDSIQFSDRRRR**	CGAAGCAAGTGCCGTTATTAAGGCAGGGGTTTGATTCACACT
						**DVPRQMLTVSFNV**	CGTAAATATTAGTCTCGCACATTTCTCCCATGAATCCAGGCA
						**QVFPNATNGDIPV**	GACATGCGCACTCATATGCATTCAATTTGTCCAGGCATTTGG
						**KPAMEPGDENEIDL**	CACCGTTGAGGCAGGGATTTGGATCACAGTCATCTCTATTTG
						**SSSPLMKDLENKISL**	TTTGACACTGGTGGTCAGTCCACCCTTCAAGGCATTCGCAGG
						**NVKDAYNVTNIPEP**	TGAAATTTCCAATTCCGTCAATGCAAGTTCCATTGTTTAGACA
						**EVNYGVIVMCSVG**	AGGACTTGAGGCGCAGTCGTCGAAATTAGTTTCGCAGTCTTT
						**QINANSSCLNCPAG**	GCCCAATAAACCATCTGGACACTCGCACTCATAATCTTTGTC
						**TYKDDVSETCIDCP**	TCGCTGAATGCATGGGGCATTGTACTGGCAAGGGCTGCGTTC
						**TATYQDEERQLSCK**	GACACAGATATCAAACTCCTTGCAGTCTTCGAAATGTTTGGC
						**PCPVGFTTHEVKAK**	ACCTGTGATCGTCGTGGTAGTTCGGTTGGGACAAACTGTGCA
						**SMDDCKGPCQPGT**	GTTAGTTTGGGTTGCTTGGTCCTGATACGAGCCAATTTCACAG
						**YSETTFEPCLACPLN**	TAATTGCAAGGTTCATACCCAGTATCTGAGAAAGTCCCTGGC
						**TYQDKATRKECKK**	TTGCAAGGTGCTACGCAGTCGCTAGATGAATTTGCTCCAACT
						**CPSGLYTWRVGAN**	CTCCAAGTATATAGACCGGAAGGACATTTCTTGCACTCCTTC
						**SSSDCVAPCKPGTF**	CTTGTTGCTTTATCCTGATATGTGTTCAACGGGCACGCAAGGC
						**SDTGYEPCNYCEIG**	AAGGCTCGAAAGTTGTCTCAGAGTAGGTTCCAGGCTGACAA
						**SYQDQATQTNCTV**	GGTCCTTTGCAGTCATCCATTGATTTGGCCTTCACTTCGTGCG
						**CPNRTTTTITGAKH**	TTGTGAAGCCTACAGGACAGGGCTTGCAGGATAACTGACGTT
						**FEDCKEFDICVERSP**	CTTCATCCTGATAAGTGGCCGTGGGACAGTCGATACATGTTT
						**CQYNAPCIQRDKD**	CACTTACGTCATCTTTGTAAGTTCCGGCAGGGCAGTTCAAGC

						**YECECPDGLLGKDC**<br>**ETNFDDCASSPCLN**<br>**NGTCIDGIGNFTCE**<br>**CLEGWTDHQCQTN**<br>**RDDCDPNPCLNGA**<br>**KCLDKLNAYECACL**<br>**PGFMGEMCETNIYE**<br>**CESNPCLNNGTCFD**<br>**MQNGYRCCCTEGF**<br>**HGANCEEELITDAC**<br>**DNANCQNGGTCIG**<br>**GVVGVDEYHCACR**<br>**PGFIGKFCEVNIDD**<br>**CQDHKCQNGGTC**<br>**VDGIANATCSCRQ**<br>**KFTGPRCEEALMHE**<br>**YTLTFPTPSTTNYVE**<br>**VNNVRRLYAITVSF**<br>**FMKTSFKDPNSRAT**<br>**PVSYAYKYQDHLE**<br>**DNALTLLDTNNFVL**<br>**YIHGQTFHTEFSAN**<br>**RDSSWHHYAITWN**<br>**KEGGVWTFYVDGK**<br>**TITDGKNVGENKY**<br>**MHPGIFVLGQEQD**<br>**DVGSMFSVLEAFA**<br>**GDITEFNVWDYAM**	ATGAACTGTTTGCGTTGATTTGACCCACTGAGCACATCACGA<br>TGACACCATAGTTCACTTCAGGCTCGGGAATATTTGTCACGTT<br>GTAAGCATCTTTGACGTTCAGAGATATTTTGTTTTCCAGATCC<br>TTCATTAATGGACTCGAAGATAAGTCTATCTCATTTTCATCGC<br>CAGGTTCCATCGCTGGTTTCACTGGGATATCGCCATTCGTTGC<br>GTTTGGAAACACTTGCACGTTGAACGAGACGGTAAGCATCTG<br>CCTCGGAACATCACGCCTCCTTCTGTCAGAGAATTGTATACT<br>GTCGAAGGAGTCAGCTCGCAAGCTACGTCTTGCGCGCGTTGT<br>GACAGGACTACAGGTAATGTTTACATTCTCTGGAGTGCATCC<br>TCTATCTTTCGGGCAGAAAAAATCGTTCTTGAAAGCATCAAT<br>ATTGTAGAACTTTTCCCGGAATTTCTTAAGTGACTCCTCCGAG<br>CACGAATCCACTCCAAAATTCATTCCAACTGTCACGTTGAGG<br>GAGGCTGGTTTCTGTTTTATCGTGCAGTCTGGCCACGGCATCT<br>TGACTTTTCTACCAACTCTGAAGACTACCCAATTTCCGACGAT<br>TTTTCCATCTTGCATTCCCTGCTTACAGTAATAGTTTCTTTCAA<br>CACCCTTTCCTGCAGCGAACTGAAAACCGTCGTGGCAAAAC<br>ACGGTGCACATTCTGCCATCTTGCCAGTTGTCGCAAGTCAAA<br>ACGCCATTGGCTGGTGGAGGGTAAAAAGGGCACGGATAATG<br>CTTGACTTTAACTTGGAAGCTGCATCCAGCACTGTTGTTAGCT<br>GCATCAGTGGCATTGTAGTAGACAGTAGCGGCTGGTCCCCAA<br>CCGAACTTGTCACCACTTTTACGAGAACTCTCCACGGATTTC<br>AGTGTCTTTGAATTGTCCTCGAATATTGGTTCTTCCCACTCTA<br>CAGGTACGGGATCATTGGTGTTGTGATAAATTTCAATATCTTC<br>AGGACAGAACACGACTTTGGGTGATTCGTGATCCAGAACTTC<br>GACGCTGAAAGAACAGGTGCTGGAGAGTTTGGAGTGATCGG<br>TCGCTTCGTATACCACTTTCGTCTTACCAACCGGAAAGTAGA<br>AAGAGGGCTTGTTCTTCTTATAATGAGGAGATTTTTGCAAATT<br>CTTTGGTTCCGCTATTCTGACTTTCACAACTGCCCCGACTACG

						**TTEEIKKISLSCGFV**	TTGTCCGTTGCTTTTGGCGAATCCCACTTCACGAGGCATTTC
						**GNVIPWYIVSQYVS**	CTTTATGAGCTTCTGCGTCGACTGTCATGGATTTGGGGCACTG
						**GEVQISQDVDKCK**	TATTGTGGGTGGTTCATTGTCTTGACAGCTTATGTCTTTGTATC
						**DIGHCPENSCDCFY**	CTTTCGACCACTTTTTGTTAGCCTCGCATGTAAACACGCCTTC
						**SSQEKEHSCKNNV**	CGTTTTATTACCGTTGGCCATCAAGATGTAACCAGCTGCACA
						**NACDDGACSNGQP**	GTGGTATTGGCAAATTGAGCGATAAGGCATAGGATTCGTGCC
						**CVTGAESSFCSCDS**	ACACACGGTCCGCTTGAAATGGAGACGTGCATTCGTTCTCAA
						**GFEGRRCEYDIDEC**	CGGATCACAGAAGATGCCTGTACAAGTTGCTTCATCTCCTGT
						**LDQNGGCEYICVN**	CCAGCTATTAAACAAACACGTGCGGATTTTCGACCCGGAAA
						**DVGTGSYHCECPS**	GTTCGTATCCTTTGTTACACTGGAAACTGCACGCCGTTCCGA
						**DMIMSDDGKKCK**	ATGTGAAGTCTTTCTCTGAACAATTATGCATTCCATTGTGTGG
						**DTGYCLDHGETYV**	AAAGCGAAGAGCTGGGCAAGTCATCTTTTCGCAGTGGAATTC
						**NGETWVKDCQNCI**	CTCTCCGCTCCATTCTCTACTTTCCAGGCAAGTTCTACTTAGTT
						**CDSGIVQCEDIFCN**	GTGGAGGATGCTGGTTCATGCTTTGCAGGGCCATGTATTCCTT
						**ETSCLPQQTLMTSP**	GCAAGAATGCTGGCAATAACCTTCTCTACATTTAAAAGAGCA
						**GECCPKCVAWAFC**	CTGTGTTTTGTAAGTGTTGTTGCAGTGGGTGGGGATCAAGATT
						**VISLNDSIMAFDFS**	CCTCCATCCGGAGCACTCAGCGATGCGCATCTCACGGGTGCG
						**KYEIQGGCNYTAVE**	CACATGTTTCCAATTTTCTCATATCCCGTTTCACAAACGCAGT
						**DCYGGRFKVEVQF**	CCTTGAAATTAGTACTCCCTTCTCTATCAGTTGTTGAATGGGC
						**PPNRTNLVIYQDCQ**	GGGGCATGCCGTACATTCTGCATTTCGAGATTCTGACTTGTA
						**KMTISSDGSVMID**	AGTGCCTTTAGGACACTTGTGGCATTGTCCTTTATAGCCCCCT
						**GVNEDVPFENELW**	CTCCCATGATATCCAGCGTGACATGCGCATTCATATCTTCCG
						**NVEEVNSSISVKTN**	GTTCTAGTTCCACAGTAGCAGTTGGCATGCTCGTCGCAACAG
						**ADLMVDWDFTNE**	TCCTGGTGTTCAGTGCAAAGGCTATTGCAGTAAGAGGGATTC
						**AVISIPSTHVSKMC**	TTCGCCAAATCCATGGAAATTTCTCTCGTATCATTAACTAAGT
						**GICSKLEEYHKYSS**	ACGTATTGACCTTGACAATGTAATCGAAATCTTTAAGGATGT
						**GKFNLSERWATGE**	AAATGTGGTCCGGAGAAGTGGCGACTGCGTTGATTTCTGCGA
						**NECYIISEGYVENVF**	CGTTCACTTCAGCAACGCCTATGGCGAAAATGATGATTCCCC

						**TSGSSRDFSSASLLLVYLLFALITFTGSKFLT***	TCGCTTTCAGAGAGTTGGATATAGCGACGGGACTATGGCCATCGTTACTCTGCCCGTCAGTCAACAGTATAATTATCTTATTGGAATCTGGGCGAGCCTGCGCAAGGATTTTGTCGGCCTCGCGCATAGCCTTGTCGGTATGCGTCCACTCAGCTACATATCCGATGTCGTCGATTTCTTTCAGTAGGGTGCACATGTTGTTGCCATAGGGATTCTTGATGTAGTCGATGTGCGTTATAGTCTCCGTTGCGAATGTCACCACTGCGATTCTGCTGTGCTCAGCCGACACGGAAAGCCTCTGGGCAGTTAGCTTAGCGAAGAGTTTCACTTGGGGGAATTTATCCACCCCGATGCTTCCTGACTCGTCGAGGAGGAACACGACATCGTTTACCGTATTCGCATAGCGTTCCAGCGTTCCCAGGAACCTCCCGAGGGATTCATCTATTTGCACTCGGTCGCTACTCACTAGAGATGCCGTTAAGAAAAACAGCCCGATGAACAAAATGTTCCTTATTAGCATTTTTTTCGGGAAGACAGCACGTAAATAGGCAATAATATTGAACAGACAATTAGTCAAAGCGAAACAGGCAGGTATGAAATTTTGCAATAATTAAGCGCCGAGCCCAATATTTTCTGCTCTTTACTCGCCCCGGCTCGAGCTTGGCGTGTTTAGCTCACAACTTCAGGAAACGCAAATAGAAAAGAGAGAGGAAATTTTGACAACCTTGAATACGGGTCGAAAGCATTCCGCTCAGAGCCG

PhdEnzKin02	Q58L96	1,87E-05	36,111	PF00008<br>PF00594<br>PF00089	211913.76<br>**209575.86**	MLIRNILFIGLFFLTASLVSS**DRVQIDESLGRFLGTLERYANTVNDVVFLLDESGSIGVDKFPQVKLFAKLTAQRLSVSAEHSRIAVVTFATETITHIDYIKNPYGNNMCTLLKEIDDIGYVAEWTHTDKAMREADKILAQARPDSNKIIILLTDGQSNDGHSPVAISNSLKARGIIIFAIGVAEVNVAEINAVATSPDHIYILKDFDYIVKVNTYLVNDTREISMDLAKNPSYCNSLCTEHQDCCDEHANCYCGTRTGRYECACHAGYHGRGGYKGQCHKCPKGTYKSESRNAECTACPAHSTTDREGSTNFKDCVCETGYEKIGNMCAPVRCASLSAPDGGILIPTHCNNTYKTQCSFKCREGYCQHSCKEYMALQSMNQHPPQLS**	GAAAAACGTGGTAAGAACCGTAACAAATCAAAATATTAAAATAAAGGAATAACATTCAATAAGAAATGCAAAAATTGCTCATCAGTCAAAGTAACATTTAATATCTGAAATGTTTATTTTGCACTATTAAATTACTTTTTATTGATTTTTTTTTTATTTCTAGGAATGTAACAGATGATCTAATGTATAAGGTTTATGCATTACATCCACTTTTCTTATTTCTTTAGACTCTTAATTACATTAACTACACCGTATATATGCATGCATATGTAATCTTAGTGCATAATCCTAACTTCTGGTTATTAAATCTAGTTTTTATTAAATTTACTTGTTTAAATCAAGGGCTACAATTCTGAAAACTATGAATTTTGAAACTGCAGATTTCTTCAAACTTCATACCGAAATTAATATGAAACATAACAAGATATATATAATTATCATAATTTTTAGCTAGAATACGCGCTGTGATTTTAAGCCAAATTACCTGAAGAAACTGGAAAAGCAAAGTAAGCAATTTTCCCCATTGTGTGCAAATCCTGTGTGTGTGTTCATAAATTTCATTTAGTTCACAAGTTTCATAATGTGTGTTCATACGTTTAATAAGTTTTATTAAGTTCATCAGGTTTTGGATATACGAATTAAATCTCATTTTTACCTCGAGAGTTAACTATATACATATTACATAGTATGGTGAGTTATTTTGAGTTAAACATATTTACGCCGTATATGGAAATTTTTAAGCAAATAAAATATTCTGTATTGTTTAAAAAATCTTAACTAAATTGTTAGAAAGAAATCTCTGATGAGACAATATGAAAATTAATAAAGTTACCATGATATTCTCGTGTTACCACGACATAGGATCTACGATTATTTCTAAAAATCATAAATGATTTGCTTTAAAAGATTAGGGTCTATTACCGATATTTCATACAGTTAGATAATTTTAAAATTTCAACATAATTAATTTTGAATGTAAGAAAGTATTTAGATAAAACAATTATTGAAGAAATTTAGATATGCGTCAAACCAATTTTAAAAAACATAATTATCTAATAACTTCTTTTACTGTGAAAAGGAAAAACATCATAAAATTTCAACACTGTCTGTGTCGTACTTTGACCATTTAACCCATTTACTCCGAACTACAGTATATTTCGTATATTTTTATGTTCTTAGAGCTACGGGCTATT

						**RTCLESREWSGEEF**	TTAGTCGTACCAATGTGGTGAGGCTTTTGGTAAATTTTCGTTT
						**HCEKMTCPALRFPH**	CACATGCGTAACTTAGCGGTCTCATTACAAAGGATCGAAATA
						**NGMHNCSEKDFTF**	TTGACAGCGAAGTGTTCGGCCCCGTGTATTTTAGAGGGGTCA
						**GTACSFQCNKGYEL**	TCTCAATATTGCAGAAAAACCTACTTCTTTGCAGATTTTTTAC
						**SGSKIRTCLFNSWT**	GCTGTTTATTGGTTCGATAAATTTTCTGCTTTCGTTCATCGTAT
						**GDEATCTGIFCDPL**	GATTTTGACTTTCATCGTGTTATAAATATACTTCTAGCATAAA
						**RTNARLHFKRTVC**	CTTCAATAAACCAGTAATATAATTATTTTATTATTGATTAAAA
						**GTNPMPYRSICQYH**	ATAATTATTAAATTCAGATTATTCTTCATTTAGATTATCTAAT
						**CAAGYILMANGNK**	AAGATTAGTAAAATCTCAAATGGGGATAGAGTTGTAAATAA
						**TEGVFTCEANKKW**	GAAAATGCTAACTGTTAGACAGATGTGTCACATATGAGAGTT
						**SKGYKDISCQDNEP**	GTAGGACTTTTTAAAGAGAGATTTATCTTAGTTGCTAGAGGT
						**PTIQCPKSMTVDAE**	GTTAAAGACTATTGTTTACAGCTTAGACATTGGTGCCTGGAT
						**AHKGNALVKWDSP**	ATAGCTAATTAAGCGGTCAATAATTAAGCTAACATTTTTTTA
						**KATDNVVGAVVK**	AAAATTTTCCTGTGAAAAGCTGGCTCCGCGTTTTGCATGAAA
						**VRIAEPKNLQKSPH**	ATTAAATAAGAAAAACACTTACTTTTTAAAGCGTATATTATG
						**YKKNKPSFYFPVGK**	TTTACTCCATAGATAATAAATAGTTCGAAACATATTTTGCACT
						**TKVVYEATDHSKLS**	CAAAATATTTCAGATGCTATTTTTGGAAAATGACAATATATTT
						**STCSFSVEVLDHESP**	AAGCATGGACTTAAATGAATTGTTAATAAATTCACAAGGAA
						**KVVFCPEDIEIYHNT**	ATAGGTTTTTGTGCAATTATGAAACGACTCCTGTGAAATACT
						**NDPVPVEWEEPIFE**	CCGCACCCCCGTCTTCTTATACTATAAAAAAGAGCGGGTTCC
						**DNSKTLKSVESSRK**	AAAGGTTGCCAAAATGAAATATTACCACAGGAATTAGCGAC
						**SGDKFGWGPAATV**	TGGATACGAGTGAAACAGCACAGTCGATCCAACAGTTGTGTT
						**YYNATDAANNSAG**	TAATTTTCCTGGCAGTAAATGGATTAAAATTGCCACGAAATA
						**CSFQVKVKHYPCPF**	TGATGAGACATATAGCTTGATATTTAATATAAAATACATATC
						**YPPPANGVLTCDN**	GTATGTGCTGCCTATGACAAATATAACTTAGCATGCGACATT
						**WQDGRMCTVFCH**	TCAGTGTTATTGATTTTTAAATACTTGATTGAAATATAATTAG
						**DGFQFAAGKGVER**	AAGTTAATTGTTTCAGTCATATGAGCAATTTTCATGTACATAA
						**NYYCKQGMQDGKI**	GAACTGCCTTTCAATCGCAACGCATTTAAAAATCGGAAAGA

						**VGNWVVFRVGRK**	ACAAGTGAATAAAAGTCACTCGAAATTAGCCTTACTGTAGCC
						**VKMPWPDCTIKQK**	CATCTTTCTGACAGGTTGAATTTTCCGCTGCTGTACTTGTGGT
						**PASLNVTVGMNFG**	ATTCTTCTAACTTGCTGCAGATTCCGCACATCTTGCTGACGTG
						**VDSCSEESLKKFRE**	CGTGCTGGGTATTGAGATCACAGCCTCGTTTGTGAAATCCCA
						**KFYNIDAFKNDFFC**	GTCCACCATTAAATCGGCGTTCGTCTTCACGGAAATGCTGGA
						**PKDRGCTPENVNIT**	GTTCACTTCTTCGACATTCCACAATTCGTTCTCGAAAGGCACA
						**CSPVTTRARRSLRA**	TCCTCGTTGACACCGTCGATCATTACGCTGCCGTCCGAAGAA
						**DSFDSIQFSDRRRR**	ATCGTCATCTTTTGGCAGTCTTGGTAAATAACTAAGTTTGTTC
						**DVPRQMLTVSFNV**	TATTCGGCGGGAATTGGACCTCGACCTTGAACCGCCCACCGT
						**QVFPNATNGDIPV**	AACAGTCCTCGACGGCGGTATAGTTGCACCCACCTTGAATTT
						**KPAMEPGDENEIDL**	CGTACTTAGAGAAGTCAAAAGCCATGATGCTGTCGTTGAGA
						**SSSPLMKDLENKISL**	GAGATCACGCAGAAGGCCCACGCTACACATTTTGGACAGCA
						**NVKDAYNVTNIPEP**	TTCTCCAGGGGAGGTCATCAAAGTTTGTTGTGGGAGGCAAGA
						**EVNYGVIVMCSVG**	AGTTTCATTGCAGAATATGTCTTCACATTGCACGATTCCGCTA
						**QINANSSCLNCPAG**	TCGCAAATGCAGTTCTGACAGTCTTTGACCCAAGTTTCGCCA
						**TYKDDVSETCIDCP**	TTCACATAGGTCTCACCGTGGTCCAGGCAATAACCGGTATCC
						**TATYQDEERQLSCK**	TTGCATTTCTTCCCATCGTCCGACATAATCATATCACTGGGAC
						**PCPVGFTTHEVKAK**	ATTCACAGTGATACGAACCAGTTCCCACATCGTTCACGCAAA
						**SMDDCKGPCQPGT**	TGTATTCACAACCGCCATTCTGATCCAAGCATTCGTCGATATC
						**YSETTFEPCLACPLN**	GTATTCACAGCGTCTTCCTTCAAACCCTGAATCGCAGCTGCA
						**TYQDKATRKECKK**	GAAGGAACTCTCGGCACCGGTGACACAAGGCTGTCCGTTTG
						**CPSGLYTWRVGAN**	AGCAAGCACCGTCATCGCAGGCGTTAACGTTGTTCTTGCAAC
						**SSSDCVAPCKPGTF**	TGTGTTCTTTCTCCTGACTTGAGTAGAAACAATCGCAGCTGTT
						**SDTGYEPCNYCEIG**	TTCTGGGCAATGTCCAATATCTTTGCACTTATCGACATCCTGA
						**SYQDQATQTNCTV**	GATATCTGTACTTCTCCGCTGACATACTGGCTCACAATGTACC
						**CPNRTTTTITGAKH**	AAGGGATCACATTTCCGACGAAGCCACACGACAACGATATC
						**FEDCKEFDICVERSP**	TTCTTGATTTCCTCAGTTGTCATGGCGTAGTCCCACACGTTAA
						**CQYNAPCIQRDKD**	ATTCTGTAATATCACCGGCGAATGCCTCTAGGACACTAAACA

						**YECECPDGLLGKDC**	TACTTCCAACGTCATCTTGTTCTTGGCCAAGGACAAAGATTC
						**ETNFDDCASSPCLN**	CTGGATGCATGTATTTATTTCTCCAACGTTCTTACCATCGGT
						**NGTCIDGIGNFTCE**	AATGGTCTTCCCATCGACGTAGAAAGTCCATACTCCTCCCTC
						**CLEGWTDHQCQTN**	TTTATTCCACGTTATTGCATAATGGTGCCAAGAGGAATCTCTG
						**RDDCDPNPCLNGA**	TTTGCAGAAAATTCTGTATGGAATGTCTGCCCATGTATGTAC
						**KCLDKLNAYECACL**	AATACGAAGTTATTGGTGTCCAATAAAGTGAGAGCATTGTCT
						**PGFMGEMCETNIYE**	TCGAGATGATCTTGGTACTTATAGGCGTAAGACACGGGTGTT
						**CESNPCLNNGTCFD**	GCTCGGCTGTTTGGATCCTTGAAACTTGTTTTCATGAAGAAAG
						**MQNGYRCCCTEGF**	ACACTGTGATTGCGTACAATCGACGAACGTTATTCACCTCCA
						**HGANCEEELITDAC**	CGTAGTTGGTGGTGGAGGGTGTGGGAAACGTTAAAGTATACT
						**DNANCQNGGTCIG**	CGTGCATCAGTGCTTCTTCACAGCGTGGTCCAGTAAACTTCTG
						**GVVGVDEYHCACR**	CCGACAGGAACATGTTGCATTTGCGATTCCGTCGACACAGGT
						**PGFIGKFCEVNIDD**	GCCGCCATTTTGGCATTTATGATCTTGGCAGTCGTCAATATTA
						**CQDHKCQNGGTC**	ACTTCGCAGAACTTCCCGATGAATCCCGGTCGACACGCGCAG
						**VDGIANATCSCRQ**	TGATATTCGTCCACCCCTACAACACCTCCGATGCAGGTCCCA
						**KFTGPRCEEALMHE**	CCGTTTTGGCAGTTGGCATTATCACAAGCATCCGTGATTAATT
						**YTLTFPTPSTTNYVE**	CCTCTTCGCAGTTAGCACCGTGAAAGCCTTCAGTGCAACAGC
						**VNNVRRLYAITVSF**	ACCTGTAGCCATTCTGCATATCGAAGCAAGTGCCGTTATTAA
						**FMKTSFKDPNSRAT**	GGCAGGGGTTTGATTCACACTCGTAAATATTAGTCTCGCACA
						**PVSYAYKYQDHLE**	TTTCTCCCATGAATCCAGGCAGACATGCGCACTCATATGCAT
						**DNALTLLDTNNFVL**	TCAATTTGTCCAGGCATTTGGCACCGTTGAGGCAGGGATTTG
						**YIHGQTFHTEFSAN**	GATCACAGTCATCTCTATTTGTTTGACACTGGTGGTCAGTCCA
						**RDSSWHHYAITWN**	CCCTTCAAGGCATTCGCAGGTGAAATTTCCAATTCCGTCAAT
						**KEGGVWTFYVDGK**	GCAAGTTCCATTGTTTAGACAAGGACTTGAGGCGCAGTCGTC
						**TITDGKNVGENKY**	GAAATTAGTTTCGCAGTCTTTGCCCAATAAACCATCTGGACA
						**MHPGIFVLGQEQD**	CTCGCACTCATAATCTTTGTCTCGCTGAATGCATGGGGCATTG
						**DVGSMFSVLEAFA**	TACTGGCAAGGGCTGCGTTCGACACAGATATCAAACTCCTTG
						**GDITEFNVWDYAM**	CAGTCTTCGAAATGTTTGGCACCTGTGATCGTCGTGGTAGTTC

						**TTEEIKKISLSCGFV**<br>**GNVIPWYIVSQYVS**<br>**GEVQISQDVDKCK**<br>**DIGHCPENSCDCFY**<br>**SSQEKEHSCKNNV**<br>**NACDDGACSNGQP**<br>**CVTGAESSFCSCDS**<br>**GFEGRRCEYDIDEC**<br>**LDQNGGCEYICVN**<br>**DVGTGSYHCECPS**<br>**DMIMSDDGKKCK**<br>**DTGYCLDHGETYV**<br>**NGETWVKDCQNCI**<br>**CDSGIVQCEDIFCN**<br>**ETSCLPQQTLMTSP**<br>**GECCPKCVAWAFC**<br>**VISLNDSIMAFDFS**<br>**KYEIQGGCNYTAVE**<br>**DCYGGRFKVEVQF**<br>**PPNRTNLVIYQDCQ**<br>**KMTISSDGSVMID**<br>**GVNEDVPFENELW**<br>**NVEEVNSSISVKTN**<br>**ADLMVDWDFTNE**<br>**AVISIPSTHVSKMC**<br>**GICSKLEEYHKYSS**<br>**GKFNLSERWATVRL**<br>**ISSDFYSLVLSDF***	GGTTGGGACAAACTGTGCAGTTAGTTTGGGTTGCTTGGTCCTG<br>ATACGAGCCAATTTCACAGTAATTGCAAGGTTCATACCCAGT<br>ATCTGAGAAAGTCCCTGGCTTGCAAGGTGCTACGCAGTCGCT<br>AGATGAATTTGCTCCAACTCTCCAAGTATATAGACCGGAAGG<br>ACATTTCTTGCACTCCTTCCTTGTTGCTTTATCCTGATATGTGT<br>TCAACGGGCACGCAAGGCAAGGCTCGAAAGTTGTCTCAGAG<br>TAGGTTCCAGGCTGACAAGGTCCTTTGCAGTCATCCATTGATT<br>TGGCCTTCACTTCGTGCGTTGTGAAGCCTACAGGACAGGGCT<br>TGCAGGATAACTGACGTTCTTCATCCTGATAAGTGGCCGTGG<br>GACAGTCGATACATGTTTCACTTACGTCATCTTTGTAAGTTCC<br>GGCAGGGCAGTTCAAGCATGAACTGTTTGCGTTGATTTGACC<br>CACTGAGCACATCACGATGACACCATAGTTCACTTCAGGCTC<br>GGGAATATTTGTCACGTTGTAAGCATCTTTGACGTTCAGAGA<br>TATTTTGTTTTCCAGATCCTTCATTAATGGACTCGAAGATAAG<br>TCTATCTCATTTTCATCGCCAGGTTCCATCGCTGGTTTCACTG<br>GGATATCGCCATTCGTTGCGTTTGGAAACACTTGCACGTTGA<br>ACGAGACGGTAAGCATCTGCCTCGGAACATCACGCCTCCTTC<br>TGTCAGAGAATTGTATACTGTCGAAGGAGTCAGCTCGCAAGC<br>TACGTCTTGCGCGCGTTGTGACAGGACTACAGGTAATGTTTA<br>CATTCTCTGGAGTGCATCCTCTATCTTTCGGGCAGAAAAAAT<br>CGTTCTTGAAAGCATCAATATTGTAGAACTTTTCCCGGAATTT<br>CTTAAGTGACTCCTCCGAGCACGAATCCACTCCAAAATTCAT<br>TCCAACTGTCACGTTGAGGGAGGCTGGTTTCTGTTTTATCGTG<br>CAGTCTGGCCACGGCATCTTGACTTTTCTACCAACTCTGAAG<br>ACTACCCAATTTCCGACGATTTTTCCATCTTGCATTCCCTGCTT<br>ACAGTAATAGTTTCTTTCAACACCCTTTCCTGCAGCGAACTG<br>AAAACCGTCGTGGCAAAACACGGTGCACATTCTGCCATCTTG<br>CCAGTTGTCGCAAGTCAAAACGCCATTGGCTGGTGGAGGGT

							AAAAAGGGCACGGATAATGCTTGACTTTAACTTGGAAGCTG<br>CATCCAGCACTGTTGTTAGCTGCATCAGTGGCATTGTAGTAG<br>ACAGTAGCGGCTGGTCCCCAACCGAACTTGTCACCACTTTTA<br>CGAGAACTCTCCACGGATTTCAGTGTCTTTGAATTGTCCTCGA<br>ATATTGGTTCTTCCCACTCTACAGGTACGGGATCATTGGTGTT<br>GTGATAAATTTCAATATCTTCAGGACAGAACACGACTTTGGG<br>TGATTCGTGATCCAGAACTTCGACGCTGAAAGAACAGGTGCT<br>GGAGAGTTTGGAGTGATCGGTCGCTTCGTATACCACTTTCGTC<br>TTACCAACCGGAAAGTAGAAAGAGGGCTTGTTCTTCTTATAA<br>TGAGGAGATTTTTGCAAATTCTTTGGTTCCGCTATTCTGACTTT<br>CACAACTGCCCCGACTACGTTGTCCGTTGCTTTTGGCGAATCC<br>CACTTCACGAGGGCATTTCCTTTATGAGCTTCTGCGTCGACTG<br>TCATGGATTTGGGGCACTGTATTGTGGGTGGTTCATTGTCTTG<br>ACAGCTTATGTCTTTGTATCCTTTCGACCACTTTTTGTTAGCCT<br>CGCATGTAAACACGCCTTCCGTTTTATTACCGTTGGCCATCAA<br>GATGTAACCAGCTGCACAGTGGTATTGGCAAATTGAGCGAT<br>AAGGCATAGGATTCGTGCCACACACGGTCCGCTTGAAATGG<br>AGACGTGCATTCGTTCTCAACGGATCACAGAAGATGCCTGTA<br>CAAGTTGCTTCATCTCCTGTCCAGCTATTAAACAAACACGTG<br>CGGATTTTCGACCCGGAAAGTTCGTATCCTTTGTTACACTGGA<br>AACTGCACGCCGTTCCGAATGTGAAGTCTTTCTCTGAACAAT<br>TATGCATTCCATTGTGTGGAAAGCGAAGAGCTGGGCAAGTC<br>ATCTTTTCGCAGTGGAATTCCTCTCCGCTCCATTCTCTACTTTC<br>CAGGCAAGTTCTACTTAGTTGTGGAGGATGCTGGTTCATGCTT<br>TGCAGGGCCATGTATTCCTTGCAAGAATGCTGGCAATAACCT<br>TCTCTACATTTAAAAGAGCACTGTGTTTTGTAAGTGTTGTTGC<br>AGTGGGTGGGGATCAAGATTCCTCCATCCGGAGCACTCAGC<br>GATGCGCATCTCACGGGTGCGCACATGTTTCCAATTTTCTCAT

							ATCCCGTTTCACAAACGCAGTCCTTGAAATTAGTACTCCCTTC<br>TCTATCAGTTGTTGAATGGGCGGGGCATGCCGTACATTCTGC<br>ATTTCGAGATTCTGACTTGTAAGTGCCTTTAGGACACTTGTGG<br>CATTGTCCTTTATAGCCCCCTCTCCCATGATATCCAGCGTGAC<br>ATGCGCATTCATATCTTCCGGTTCTAGTTCCACAGTAGCAGTT<br>GGCATGCTCGTCGCAACAGTCCTGGTGTTCAGTGCAAAGGCT<br>ATTGCAGTAAGAGGGATTCTTCGCCAAATCCATGGAAATTTC<br>TCTCGTATCATTAACTAAGTACGTATTGACCTTGACAATGTAA<br>TCGAAATCTTTAAGGATGTAAATGTGGTCCGGAGAAGTGGCG<br>ACTGCGTTGATTTCTGCGACGTTCACTTCAGCAACGCCTATGG<br>CGAAAATGATGATTCCCCTCGCTTTCAGAGAGTTGGATATAG<br>CGACGGGACTATGGCCATCGTTACTCTGCCCGTCAGTCAACA<br>GTATAATTATCTTATTGGAATCTGGGCGAGCCTGCGCAAGGA<br>TTTTGTCGGCCTCGCGCATAGCCTTGTCGGTATGCGTCCACTC<br>AGCTACATATCCGATGTCGTCGATTTCTTTCAGTAGGGTGCAC<br>ATGTTGTTGCCATAGGGATTCTTGATGTAGTCGATGTGCGTTA<br>TAGTCTCCGTTGCGAATGTCACCACTGCGATTCTGCTGTGCTC<br>AGCCGACACGGAAAGCCTCTGGGCAGTTAGCTTAGCGAAGA<br>GTTTCACTTGGGGGAATTTATCCACCCCGATGCTTCCTGACTC<br>GTCGAGGAGGAACACGACATCGTTTACCGTATTCGCATAGCG<br>TTCCAGCGTTCCCAGGAACCTCCCGAGGGATTCATCTATTTG<br>CACTCGGTCGCTACTCACTAGAGATGCCGTTAAGAAAAACA<br>GCCCGATGAACAAAATGTTCCTTATTAGCATTTTTTTCGGGAA<br>GACAGCACGTAAATAGGCAATAATATTGAACAGACAATTAG<br>TCAAAGCGAAACAGGCAGGTATGAAATTTTGCAATAATTAA<br>GCGCCGAGCCCAATATTTTCTGCTCTTTACTCGCCCCGGCTCG<br>AGCTTGGCGTGTTTAGCTCACAACTTCAGGAAACGCAAATAG

							AAAAGAGAGAGGAAATTTTGACAACCTTGAATACGGGTCGA AAGCATTCCGCTCAGAGCCG

PhdEnzKin03	Q58L93	4,30E-05	39,583	PF00008<br>PF00594<br>PF00089	9452,59	FYYSCICHPGWAGPS<br>CEINIDECSSNPCAN<br>GGQCIDQIDGFKCQ<br>CEPGFTGSRCQHPID<br>ACEAKPCQNGGTCF<br>DLINGFACQCRPGY<br>V	TTCTACTATAGCTGTATCTGTCACCCAGGATGGGCTGGTCCAT<br>CATGTGAAATTAATATTGATGAATGCAGCAGCAATCCGTGTG<br>CTAATGGTGGCCAATGTATTGATCAGATTGATGGATTTAAAT<br>GCCAGTGTGAGCCTGGATTTACTGGGTCTAGATGTCAACATC<br>CTATTGATGCCTGTGAAGCCAAACCTTGTCAGAATGGTGGTA<br>CTTGTTTTGATCTTATAAATGGCTTTGCCTGCCAATGTCGACC<br>AGGCTATGTTGG
PhdEnzKin04	A6MFK7	9,29E-06	39,474	PF00008<br>PF00594<br>PF00089	7662,56	LDNTFQCMCNPGFT<br>GELCEFNINECMSN<br>PCLNNGICTDTVNG<br>FRCQCPQGWTGERC<br>ELDIGGCSNDPCLN	ATTCAAACAAGGATCATTACTACAACCTCCTATGTCCAGTTC<br>ACATCGCTCACCCGTCCAGCCTTGAGGACACTGACATCTGAA<br>ACCATTTACTGTGTCCGTGCAAATACCATTGTTCAAACAAGG<br>GTTAGACATGCATTCATTGATGTTAAATTCACAGAGTTCACC<br>AGTGAACCCAGGATTGCACATGCATTGAAAAGTATTATCTAA<br>A
PhdEnzKin05	A6MFK8	2,78E-10	57,143	PF00008<br>PF00594<br>PF00089	8457,5	FNGATCFDRINGFFC<br>KCSQGFVGPLCQYN<br>IDECISNPCQNDGFC<br>IDLIANYTCDCMPGF<br>DGRNCEINIDDCASS<br>PCK	GTTTTAACGGTGCAACATGCTTTGATAGAATCAATGGATTTTT<br>CTGCAAATGCTCTCAAGGATTTGTTGGTCCTTTGTGCCAGTAC<br>AATATTGACGAGTGCATCAGTAACCCTTGCCAGAATGATGGA<br>TTTTGCATTGACTTGATAGCTAATTATACATGTGACTGCATGC<br>CTGGATTTGATGGAAGAAACTGCGAGATCAATATTGACGACT<br>GTGCTAGTTCACCGTGCAAA
Phosphatases							
**Transcript ID**	**Uniprot ID reference protein**	**e-value**	**Identity (%)**	**PFAM domain**	**MW (Da)**	**Amino acid sequence**	**Nucleotide sequence**
PhdEnzPho01	Q5BLY5	1,21E-07	40,678	PF00328	10666.42<br>**8461.61**	MLKLLFYVFTSLLLL<br>MCSS**SKQDSKLLFV**<br>**QILFRHGDRAPINL**<br>**YPNDPNPESFWVEG**	ACATTTACTTCATTGGGACTCGAAGTAGTAAAATTTTCATAC<br>ACATGTCTGAGAAATTTTCCTAGGGCATAATGCTGCTTTCTTC<br>CAAGCATAGTTAATTTTCCAAGTCCTTCAACCCAGAAACTTT<br>CAGGATTTGGATCATTTGGATACAAATTGATAGGTGCTCGAT

						**<u>LGKLTMLGRKQHYALGKFLRHVYENFTTSSPNEVN</u>**	CTCCATGTCTGAATAGTATTTGTACAAATAAAAGCTTGGAATCTTGTTTGGAACTTGAGCACATTAAAAGTAGCAAAGAAGTAAATACGTAAAATAAAAGTTTAAGCATTGTTGCATAAG
PhdEnzPho 02	Q5BLY5	8,68E-06	30,337	PF00328	11303.07	FSLLFQHSVPILGFLAAMDLGNFEHPPPCASVLSELYKHEGEYFVRYLYLNSTTPQREPQKVYSLAVPGCTEFCPLNYVIEYTRDVIPKNLERECLIM*	TGCATACTGAAATCGGCAGTGTCTAAATAAAAAAATACTTCTAGATCTTTGAGCACGTTGAAACACAAGAAATGCATAAACAATAATACTCAAGCTACTGTTATGGAATTTTAGCTCTACTTTCGAGACAATCTATAAAGTTAAGCTACAGTAGTTCCCGTTAAGGAGTTCTCATATGATGACTTGAACTTGTTACATGATTAAGCATTCTCGTTCCAAGTTCTTGGGTATTACATCTCGAGTGTATTCAATCACATAATTCAGGGGGCAGAATTCTGTGCATCCGGGAACTGCAAGTGAATAAACTTTTTGGGGTTCTCTTTGAGGCGTTGTCGAATTCAGGTATAAGTACCGGACAAAATATTCTCCTTCGTGTTTGTAGAGCTCAGACAAAACACTGGCACAAGGTGGCGGATGTTCAAAGTTTCCGAGATCCATGGCTGCTAAAAACCCCAAGATAGGTACAGAATGCTGAAAAAGTAAGGAAAA
PhdEnzPho 03	Q5BLY5	5,37E-05	32,911	PF00328	10476.93	NWQPISVLYVPKADDKYLETDSYCPRATEEQVRVLNSPEGQAFIAKYKEMFENLTINSGSSIIDWTSASYLHDVLFIEVMLIYIHMNLTLG*	TTAATTGGCAACCAATATCTGTTTTATATGTTCCAAAAGCAGATGATAAGTACCTTGAGACAGACAGTTATTGTCCACGTGCCACTGAAGAACAAGTTCGTGTTTTAAATTCTCCTGAGGGTCAAGCCTTTATTGCAAAATATAAGGAGATGTTTGAAAACCTGACCATTAACAGTGGTTCTTCTATAATAGATTGGACAAGTGCTTCCTACCTGCATGATGTTCTATTTATTGAAGTAATGTTAATTTACATACACATGAATTTAACGTTAGGATGATGTTTACTTGTTTTGAATTTGTCTTTTGTATGTGTTTGTGTAAGATATGGG

PhdEnzPho 04	J3SBP3	4,52E-39	27,333	PF01223<br>PF01663<br>PF01033	52308.71<br>**50090.06**	MALLTCLTVLFATLY<br>GNSAFS**YYSNDGAE**<br>**GGNKLLLILVDGCR**<br>**WDYPDQDTAGLPG**<br>**FRKLAENGVRAPYV**<br>**TPIFPSNSYPNWYTI**<br>**MTGLYAENHGFVQ**<br>**NMMFDSYYGDLFL**<br>**MAPNDTASIQHW**<br>**WEHAEPLWVTAEK**<br>**NGLRSALYWWDGC**<br>**QVEIRGKKPTYCRK**<br>**YKYVGYSWPTVND**<br>**DTKDALVTALELLS**<br>**NDEIQLVQLYYEPV**<br>**DFYGHKYGPNSDE**<br>**RKQSVKDIDNLLD**<br>**MVQREMDNRGLL**<br>**DKVNMVVVSDHG**<br>**MTSTDSRGLSVIDL**<br>**QQIIDISDIRYMVYY**<br>**GATTMLLPYDGKLE**<br>**KVYDALQGITGLH**<br>**VYKKEDIPEHYHIK**<br>**NSRLTLPLLLVASK**<br>**NYYIRGLDIPGKSIP**<br>**TGSSISPGSHGYDP**<br>**YEVPDMRGIFFARG**	TTTTTTCAGTAATAACTTTTACAACTTTTAAATTTTAGTGGGAT<br>AAAATAAATTGTGGAAAATATTTAGATTTTGAACATAAATAA<br>ATCATTTACCAATTTATTAATGCATCGGAAAACAGGGATATT<br>AAAAATAATTATATATAATTACTTGTGAATTCGCTATATTAGT<br>GCATAGTTAATAATTCTAAATATCGTATGCTTATGGCATAATC<br>ACTTTTTTAGACGAAGTGTGCATGTTGAAAATAATCGTTAAC<br>ATCCTTAACAATTTGATTTGCTACAACTGTCAATAATGAACTT<br>GCAATAAAGCTGCAGTTGCCCTCAAGTTTTGTGAGGAAATAA<br>AATAACAAGTGATGGTTCGAAAGTCAAAATACAAAGTTTAA<br>CGTAAGATGCTCTTGCAGGTGGTCGACGTCAACGCAGGCAA<br>AATAGAATTTGCAGATCTATTATTTGAATCGTTTGGCACGGTT<br>GCAGAAGAAATGTAGTCGAAATTTCTTTGCAAACTTAACGTT<br>CCTGTAGTGGAAAAAAAGGTATTTTAATAGGCTATTTGTATCA<br>TACAAACTTATGCAATTATAAAAATATATTTCGCTTTACCAG<br>GAATCAAGTCCATACTAAAAATTTTCTCGTCCTCGACCTTGAT<br>GTACAAAAAAATTTCAATTGATAAGTGTTCGTTAAAACTCTGG<br>TATAAAATAATATTTTGACGAACTATCACGTCGTAACGTCCA<br>AGTTTTTGCACAGGAAAATCATCAAGAGAAGAAAAGCAGCT<br>GCGATGACTTCCAACGACAGCTGCGTGTGTCTAGAAGCTGAC<br>GAGTAAGGCCTAGCTATGCTGATTCCCTTCGTGACACTCCTC<br>GTGCCGTTATTCGGCAAGGGCTCGACTCCTAATAAGTTGCAC<br>AAGATATTATATATATCCACCATTTGCAGTGGGGGTGATATG<br>TAATTTCTTCTAAGCCTTGGTCCTCTGGCGAAGAATATGCCCC<br>GCATGTCAGGCACCTCATAGGGGTCATATCCATGAGACCCTG<br>GTGATATGGAAGATCCGGTAGGAATGCTTTTCCCTGGAATAT<br>CCAGACCTCGTATGTAGTAATTTTTGGAAGCCACTAAGAGTA<br>AAGGAAGTGTTAAACGACTATTTTTAATGTGGTAATGCTCAG<br>GAATATCTTCTTTCTTATATACATGTAGACCCGTGATTCCTTG

						**<u>PRLRRNYISPPLQMVDIYNILCNLLGVEPLPNNGTRSVTKGISIARPYSSASRHTQLSLEVIAAAFLLLMIFLCKNLDVTT*</u>**	TAAAGCATCATAAACCTTTTCCAGTTTCCCGTCGTAAGGAAGTAGCATCGTCGTCGCACCATAGTACACCATGTAGCGTATGTCTGATATATCTATAATCTGTTGCAAATCAATGACGCTCAGGCCCCTAGAATCAGTGGACGTCATACCGTGGTCCGAAACCACGACCATGTTCACCTTATCGAGGAGTCCTCTGTTATCCATTTCTCTCTGCACCATATCTAAAAGATTGTCTATATCTTTCACCGACTGCTTGCGTTCGTCAGAATTTGGGCCATATTTGTGTCCGTAGAAGTCTACGGGTTCGTAGTACAGCTGAACTAGCTGTATCTCATCGTTGGAGAGGAGCTCTAAGGCTGTCACGAGAGCATCTTTTGTGTCGTCGTTGACTGTCGGCCAAGAATATCCGACGTATTTATATTTTCTGCAGTAGGTCGGCTTCTTTCCTCGTATTTCAACCTGGCATCCGTCCCACCAATACAGGGCACTTCTGAGGCCATTCTTTTCGGCAGTAACCCATAATGGCTCGGCGTGCTCCCACCAGTGCTGTATGGAAGCTGTGTCATTTGGAGCCATGAGGAAGAGGTCGCCGTAGTAAGAATCGAACATCATATTCTGTACAAAACCATGATTTTCTGCGTACAGTCCTGTCATTATCGTGTACCAGTTGGGGTAAGAGTTCGAAGGGAATATGGGCGTAACGTAGGGTGCCCTCACTCCATTCTCCGCAAGTTTTCGAAATCCGGGAAGTCCGGCCGTATCCTGATCTGGATAATCCCATCGGCAGCCATCCACGAGTATGAGTAATAATTTATTACCACCCTCGGCGCCATCGTTACTGTAGTAACTGAAAGCTGAATTTCCGTAGAGAGTCGCAAAAAGCACTGTGAGGCAAGTGAGCAAAGCCATGCAGTCCCGAAAAACTTTAAAACTCGAGCGCCTTCGAGTCGAAATTCTTGTGCGAGTTCAGGAGCCGGCGCGGAGAGTGTCAGCACGCTCTGCGG

PhdEnzPho 05	J3SBP3	3,43E-42	27,619	PF01223<br>PF01663<br>PF01033	56550,13	LLPCVLLSALLVSLIP<br>GFEGFPSMPGDSEKV<br>LLVLVDGVRWDYIN<br>DSSVYSGFKRIEDNG<br>VKAEYVTPVFPSNSY<br>PNWYTIVTGLYPEN<br>HGIVQNYMYDPELD<br>DLFLMALHSNASHA<br>HWWNGAEPVWVT<br>AEKRDLKTAVYWW<br>DGCQVIIRGRKSTTC<br>EEYANYWVWGKVN<br>RDTLQAMEEVLDKF<br>QKDNYRLGLVYYEA<br>VDANGHFNGPDSE<br>HRVNSLKDIDTILNSI<br>QDQIAKKGMEHQV<br>NLIVVSDHGMKAM<br>DQDTSKVINIEKSIDI<br>KDVKLMLDRGAVS<br>MVLPAPGKLEKLYN<br>DLKSANIEGLTVYKK<br>EDIPEHYHFKKHRL<br>VQPILLIADEGYVIK<br>ALSDPKRMKPFAERI<br>FKGYHGYDPYKVKD<br>MRTIFYARGPSFKKG<br>FLSPPLEMVDHYGAI	ACCCGCGGTTTCAAGAACACACATATGTATGACAGTCTTCCG<br>AGTTCGTTGTACTGCTTTTTAAAGCATTTTAAGTTACCAGAAC<br>TCACTTGAAGTTTAAATTAAAGTTTTCCAAGGACATTTGATAT<br>TAATGCAATGCATACCATTCATCAACTAAAATGATGAAACCT<br>GCTGAGAAGTGTCTTTTGTTTAAGCGATATGTTATCACCGTAT<br>TCTGCACCTGGTGCTCAACTTGCTCCTGAAGTCAAAGAACTC<br>GTCATGTAAAGTCTCTACACATGAGAGTTCCGCCGCAGTATG<br>GAACTTGTGAAAAACACCGTAGAAAGCAACAAGATGGAAA<br>TGAGCTGCCAATCCGGTATGGTGATTCCTACAGCCGATACAT<br>TTTTTCTCTTTCCTTTGAAAACACTTTTTGACTTCTTCATGCTCT<br>TGCTTTTACTGTGTATTACTACTTTCGACTTTTTATCCGGCCCT<br>ATGTGCGTTTCTGGCCTCAGAGCATTGCCCGTCAGCATCCTCT<br>GAGTTCTTGCCCACGAGCCATTGTTCGGCAAAGGTTCCATGC<br>CCATCGCTCGACAAATGGCTCCATAGTGATCTACCATCTCCA<br>AAGGCGGTGAAAGGAATCCTTTTTTGAATGAAGGCCCTCTAG<br>CATAAAATATTGTTCTCATGTCTTTCACTTTATATGGATCATA<br>TCCGTGATAGCCTTTGAATATTCTTTCTGCGAATGGCTTCATC<br>CTTTTGGGATCAGACAACGCTTTTATAACGTATCCTTCGTCTG<br>CAATCAGCAGTATAGGCTGTACAAGCCTGTGTTTCTTGAAAT<br>GGTAATGTTCAGGAATATCCTCTTTCTTATAGACTGTCAAACC<br>TTCGATGTTTGCACTTTTCAGATCGTTATACAGTTTTTCCAATT<br>TGCCAGGCGCAGGTAGCACCATAGACACTGCTCCTCTATCCA<br>ACATGAGTTTCACATCCTTGATGTCTATTGACTTTTCGATATT<br>AATCACTTTTGATGTATCTTGATCCATCGCTTTCATGCCATGG<br>TCTGAGACTACTATTAAGTTCACTTGATGTTCCATTCCTTTTTT<br>GGCTATTTGGTCTTGGATGCTGTTAAGAATGGTATCTATATCT<br>TTCAGAGAGTTTACTCTATGTTCAGAATCGGGCCCGTTAAAA<br>TGACCGTTTGCGTCCACCGCTTCGTAGTAGACGAGCCCCAGC

						CRAMGMEPLPNNGSWARTQRMLTGNALRPETHIGPDKKSKVVIHSKSKSMKKSKSVFKGKRKNVSAVGITIPDWQLISILLLSTVFFTSSILRRNSHV*	CTATAGTTGTCTTTCTGGAACTTGTCCAGTACTTCTTCCATAGCTTGCAGTGTGTCTCGGTTCACCTTTCCCCAGACCCAGTAGTTGGCGTATTCCTCGCATGTGGTAGACTTCCTGCCGCGAATGATAACTTGGCATCCGTCCCACCAGTACACCGCTGTCTTCAAGTCCCTCTTCTCAGCGGTCACCCAGACGGGCTCGGCACCGTTCCACCAGTGGGCATGCGATGCGTTCGAGTGCAGGGCCATCAAGAAGAGGTCGTCAAGTTCTGGATCGTACATGTAGTTCTGGACGATACCGTGGTTCTCTGGGTAGAGACCTGTCACGATGGTATACCAGTTGGGGTAGGAATTGGAGGGGAAGACGGGCGTCACATATTCCGCTTTCACCCCGTTATCCTCTATCCGCTTGAATCCCGAGTACACGCTGGAATCGTTAATGTAATCCCATCGGACACCGTCCACAAGCACGAGCAGTACTTTCTCCGAATCTCCGGGCATGGAGGGGAAACCCTCGAATCCGGGGATGAGGGAGACAAGGAGCGCCGAGAGCAGAACACACGGGAGAAGCA
Oxidoreductases							
**Transcript ID**	**Uniprot ID reference protein**	**e-value**	**Identity (%)**	**PFAM domain**	**MW (Da)**	**Amino acid sequence**	**Nucleotide sequence**

PhdEnzOxR 01	Q4JHE1	5,52E-24	24,897	PF01593	58963,85	MDSDVIVVGAGLSG<br>LSAAKWLSEAGVSVI<br>VLEAKDRVGGRTCT<br>KKDPKVNYVDIGGA<br>YVGPTQDRLLRLAK<br>EFGIENYKVNEVEDL<br>LHYKNGRRSRFRPC<br>QLPGRRNPLVNMDI<br>NNIMWLMDKMGE<br>QIPADAPWNAPKA<br>DEWDTMTYNRFLRE<br>NCWTQGAIDFYKNF<br>ININITSEAYEASLLSF<br>LWYIKQCGGTKRIFS<br>TTNGGQERKFIGGS<br>QQISIRMAEKLGSDR<br>VFINSPVVGIDQESE<br>QEVIVKTLNGKEYKT<br>KFIIFACPPMLQMKI<br>HFKPPLPPLRNQLLQ<br>RAPIGTVMKVIVYYS<br>STFWREKGYCGSFLI<br>EGDDNHPMFLTLDD<br>TKPDGSHPAIIGFILA<br>DKCRRLVLRTPEERK<br>NLVAKSIAEVTGCPE<br>GLKPIHYEEKNWME<br>EQYTGGCYTAMYPP	ATCGTTGCCCACCTCCCTCTCCTAAGATAAAAATATCACAAA<br>GAGAAGGTTTCAACCTTGAAATTGAAGCCTTGATATTTTACC<br>GTATACGCTAAAATGCATGCGAATGAAACAAATAAAAAAAA<br>ATCCAAGCCTTTTATCAATGAAAGTTAAACACTTCCAAAGAT<br>AAAATCCTGCGTTGAAGAAGCGAGTGACGAGTAAAAAGAG<br>GTAGATATTTGGAGGCTAGCTACTTCCTGGCTTCATTGCGCTG<br>CGTCACTACCAACCCCGCTTCCAGCAAAGAATAGCAGTTTTC<br>CCCTTCACAGCATGGATTCAGATGTGATAGTAGTGGGAGCTG<br>GACTTAGCGGTTTGAGCGCAGCGAAGTGGCTCTCTGAAGCGG<br>GTGTCAGTGTTATTGTGCTAGAAGCAAAGGATCGAGTTGGAG<br>GAAGAACGTGTACAAAAAAAGACCCCAAAGTGAACTACGT<br>AGACATAGGAGGTGCTTACGTTGGACCTACTCAAGATCGACT<br>TCTTCGTTTGGCCAAAGAGTTCGGCATTGAAAATTACAAGGT<br>TAATGAAGTAGAAGATCTCCTGCATTATAAAAATGGAAGAA<br>GATCGCGATTTCGACCTTGTCAGCTTCCTGGACGCCGAAATC<br>CTCTTGTTAATATGGATATTAATAATATTATGTGGCTCATGGA<br>TAAAATGGGAGAGCAGATACCTGCTGATGCTCCATGGAATG<br>CACCAAAAGCTGATGAGTGGGACACCATGACTTACAATCGG<br>TTTTTGAGAGAAAATTGTTGGACCCAGGGAGCTATAGATTTC<br>TACAAAAATTTCATCAACATTAACATAACATCTGAGGCATAT<br>GAAGCTTCTCTTCTTAGTTTCTTATGGTACATCAAGCAATGCG<br>GTGGAACTAAGAGAATATTCTCTACCACCAATGGCGGTCAA<br>GAAAGAAAATTTATAGGAGGGTCTCAACAAATTAGTATCAG<br>AATGGCTGAGAAGCTAGGAAGTGACCGTGTTTTCATAAATAG<br>CCCTGTAGTTGGAATTGATCAGGAATCAGAACAGGAAGTAA<br>TAGTGAAAACGTTGAATGGCAAAGAATACAAGACGAAATTT<br>ATTATCTTCGCATGCCCTCCAATGTTACAAATGAAAATTCACT<br>TCAAACCACCTCTACCTCCATTGAGAAACCAACTATTGCAAA

						GFFTRYGKFIRTPIDRLYFAGTETASQWSGYMEGAIQAGERAAREILYTMGKITRDQIWQQEPQSQDVIPQPFEITFAEKYTPSVPGFLKIVTLSTILGATAAAFLKCPHYFKR*	GAGCACCAATAGGCACAGTAATGAAAGTGATTGTTTACTATTCAAGTACTTTTTGGAGAGAAAAGGGATATTGCGGATCTTTTCTGATCGAAGGCGATGATAATCACCCAATGTTCCTTACTCTTGATGATACAAAGCCAGACGGATCACATCCAGCAATTATTGGGTTCATATTAGCTGACAAGTGCCGCCGATTAGTGCTACGTACACCGGAAGAACGTAAAAATTTAGTAGCAAAGAGTATAGCTGAAGTTACCGGTTGCCCAGAAGGTTTGAAGCCAATTCACTATGAGGAAAAAAATTGGATGGAAGAACAATATACTGGAGGCTGTTATACTGCTATGTATCCTCCAGGCTTCTTTACAAGATACGGAAAGTTTATAAGGACACCCATAGACAGACTGTATTTTGCTGGAACTGAAACTGCCTCCCAATGGTCTGGTTATATGGAAGGAGCCATCCAAGCTGGGGAAAGAGCAGCACGGGAAATTCTGTACACTATGGGAAAAATTACCCGAGACCAAATTTGGCAGCAAGAACCACAATCACAGGATGTAATTCCTCAACCATTTGAAATAACTTTTGCTGAAAAATACACTCCATCAGTACCTGGATTTCTAAAGATCGTCACCTTATCGACGATTTTAGGTGCAACTGCAGCAGCTTTCTTGAAATGTCCACACTACTTTAAACGATAAGAACGTTCAATATTTCTAAGCAAATGATCACCAGAAAACTTCGCAGTTTATATTGTTAAATTTTCTACTCTGTATACTTTTGATGTCAATAAAATTTATTAAATTTCAGGTTACTTGTAAAAAAA

PhdEnzOxR 02	Q6TGQ8	2,57E-05	27,027	PF01593	9573,93	MEKSIISKILIVGCGL TGSVTASLIRHEAAA DITIWEKEDHIGGRF STFRNPAEPKCSVDF GAQYISATLEFIRSH ARFYDDLLEK	AAAAAATAGATTTAAAGTGTCATTAAGTTTATTGCATTTTAA GTCACTTAATCAATTCCTACTATAGTTTGTTCTAAATAGCGTT CCAACATGGAAAAATCTATAATTTCTAAGATACTAATTGTTG GATGTGGTTTGACAGGAAGTGTCACAGCTAGTCTCATTAGAC ATGAGGCGGCTGCAGATATAACTATATGGGAAAAAGAAGAC CACATTGGTGGTCGTTTTTCCACTTTTCGAAATCCTGCTGAAC CTAAATGCAGTGTTGACTTTGGTGCCCAATATATTAGTGCAA CTTTAGAGTTTATTCGAAGCCATGCAAGATTTTATGATGATCT CCTGGAAAAAAA
PhdEnzOxR 03	P81383	1,34E-05	36,207	PF01593	13121.04	MTDTKRIAVIGAGP CGLAAAKSCLEENL DVVVFDKTDNIGGL WCYRDYDEDGIPSV MKSTVINTSKEMSA YSDFPPPPNYPNFM HNSMMFQYFKQYA EKFGILPHVQLLTEV VKVTQ	GTTTGAGTTACTTTTACAACTTCAGTCAGCAACTGTACATGAG GCAGAATCCCGAACTTCTCAGCATACTGCTTGAAATATTGAA ACATCATGGAATTGTGCATGAAATTTGGGTAATTCGGAGGAG GAGGAAAATCACTATAAGCACTCATCTCCTTACTGGTGTTGA TAACAGTCGATTTCATTACAGATGGTATTCCATCCTCATCATA GTCCCTGTAGCACCACAGACCACCAATGTTATCAGTTTTGTC AAAGACTACTACGTCAAGGTTTTCTTCCAGACAACTTTTGGC TGCAGCAAGACCACAAGGTCCGGCACCAATCACAGCTATAC GCTTTGTGTCTGTCATTTTCAGAGTTCGGTTAGATTCATGAAG GTAAAACGTCAACGATATTTCCAGAACTTCCGAACTCTGCTT ATCCTAGAATCTTCTATTTACCGTTTCCAGAAAAG
PhdEnzOxR 04	P81383	2,25E-10	56,250	PF01593	9558.67	MGSSDLYLQKKILVI GAGISGLACASHLR DYGFEDILILEAKDRI GGRTYTVSYREDQSS EEPVSDFIEFGAQWI HGEDGNVIYEL	AAAATGGGAAGCTCTGATCTATATCTGCAGAAGAAAATACTT GTTATTGGAGCTGGAATATCAGGTCTTGCATGTGCAAGCCAT TTAAGGGACTATGGATTTGAAGACATTTTAATTCTTGAAGCA AAAGACCGCATTGGTGGAAGAACTTACACAGTTAGTTATAG AGAAGACCAAAGCTCTGAAGAGCCAGTATCTGATTTTATTGA ATTTGGTGCACAATGGATCCATGGAGAGGATGGTAATGTTAT TTATGAACTTG

PhdEnzOxR 05	P81383	8,54E-05	40,741	PF01593	64294.14	RKAFDFLGAATDMS PAKRVAVIGAGPSG LTAIKSCKEVGFEPV CFERNGDPGGLWRY HDEDIEGVASVMKT TIINTSKEMSAFSDFP PPTEFPNFMHNSKM YEYFMLYAKQFDLL KHIRYYLEVVKVEPT KDHDETGRWIVSVK SSKTEVITREEFDAVI VCIGHHVFPKIPTFT GQEKFRGIITHSHSV KSCEQFNGKRVVVV GIGNSGVDAAVDST FVAKEVYLSTRRGS WIFSRVGKNGRPYD MDFASRKKLLMTSF MSYDAVCDSIEKEL NKNFDHEAYKLKPK HRVMSAHPTMNDA LPNCILSGRIVIKGDI KRFEENGIVFDGEEE VTEADVVILATGYII KFPFLNDVVRIEGNK VNLYKYCISPTLKHP TLALIGLIQPLGPIFP LSEMQCRWFAQVLL	CGGAAGGCATTTGATTTCTTAGGTGCTGCAACAGACATGAGT CCCGCTAAAAGAGTTGCCGTTATTGGAGCAGGCCCGAGTGG CTTAACAGCAATCAAATCCTGCAAAGAAGTCGGATTTGAGC CTGTTTGTTTTGAAAGAAATGGAGATCCTGGTGGATTGTGGA GATATCACGATGAGGATATTGAAGGAGTTGCATCTGTTATGA AAACCACTATCATCAACACCAGTAAAGAAATGAGTGCCTTT AGTGATTTTCCGCCACCCACAGAATTCCCCAATTTCATGCAC AACAGCAAGATGTATGAATATTTCATGCTGTATGCCAAACAA TTTGATCTGTTGAAACACATTAGGTATTACTTAGAGGTAGTTA AAGTTGAACCAACAAAAGATCATGACGAAACAGGTCGTTGG ATAGTGTCTGTAAAGAGTTCAAAGACAGAGGTCATCACTAG AGAAGAATTTGATGCTGTCATTGTCTGTATTGGGCATCACGTT TTCCCCAAGATTCCGACTTTCACAGGGCAAGAAAAGTTTCGT GGAATCATCACACACTCCCACAGTGTTAAAAGCTGTGAACA GTTCAATGGGAAAAGGGTTGTTGTCGTAGGCATTGGCAATTC TGGAGTAGACGCTGCTGTGGATTCCACTTTTGTTGCAAAAGA AGTTTATTTGAGCACCAGAAGAGGATCATGGATTTTTTCTCG GGTTGGAAAAAAATGGTAGACCTTACGATATGGATTTTGCTAG TCGGAAGAAGTTGTTAATGACATCCTTCATGTCATATGATGC CGTATGCGACAGTATTGAAAAAGAACTGAATAAAAATTTTG ACCATGAAGCTTACAAACTGAAACCTAAGCATCGTGTCATG AGTGCCCATCCAACAATGAATGACGCTTTGCCTAACTGTATA CTCAGTGGGAGAATAGTTATCAAAGGAGACATAAAAAGATT TGAAGAAAACGGAATAGTTTTCGATGGTGAGGAGGAAGTGA CTGAAGCTGATGTTGTAATTTTAGCTACGGGTTACATAATCA AATTCCCTTTTCTTAATGATGTAGTACGTATAGAAGGAAACA AAGTTAATTTATACAAATACTGCATATCACCTACTTTAAAGC ACCCTACATTGGCTTTGATAGGACTCATTCAACCTTTAGGGC

						GNIKFPSPQDMMNEIKAKNDANAKRYTDSTRHTIQVEWIPFMDELSSQMGAKPNMLKLALTDPKLFAACFSGPCLPYQYRLQGPHSWSGARDTILKYEQRVFAALNAKGKVIPKNYDTGSRKFWLLAVAFTSVVFAQKYFTFSKIFNDNITSFLSNYNLLPS*	CGATATTTCCGTTATCTGAAATGCAGTGCAGATGGTTTGCTCAAGTACTTCTCGGAAACATTAAATTTCCATCCCCGCAAGATATGATGAATGAAATAAAAGCGAAAAATGATGCCAATGCGAAAAGGTACACAGATTCTACAAGACACACAATTCAAGTTGAATGGATACCATTTATGGATGAACTTTCGTCTCAGATGGGAGCTAAACCTAACATGTTAAAATTAGCTTTAACTGATCCAAAGTTATTTGCAGCATGCTTCAGTGGCCCATGTTTGCCATACCAATATAGGCTTCAAGGCCCACATTCGTGGTCAGGTGCCAGAGATACTATACTTAAGTACGAACAGAGAGTTTTTGCTGCTTTGAACGCAAAAGGAAAAGTTATTCCCAAGAACTATGATACCGGCTCAAGAAAATTTTGGTTATTGGCTGTGGCTTTCACTAGCGTAGTGTTTGCACAAAAATACTTCACGTTTTCAAAGATCTTTAACGATAACATAACTTCATTCCTTTCAAATTATAACCTACTTCCCTCCTAAGTACTTTATTCGCACATATTATAATTTTCCTATAATACATGTAACATTCATTTGAAGTTTTTCTTTATTTCTTTCACATAAAGCAGTTAACGAGAACATGTTAAATATGTTAAATGTATCACTTACTTCTGGCGGGACTCATTAAAATCATTTGTCGAGATTTAAATTTGAATGAATTTCAAGAAATGAAAATTTTTGTCCGAGGAAGATTATATGGATTGGCG

Transferases

Transcript ID	Uniprot ID	e-value	Identity (%)	PFAM domain	MW (Da)	Amino acid sequence	Nucleotide sequence

	**reference protein**						
PhdEnzTra01	Q8MMH3	2,89E-42	31,769	PF01937	32890.50	LVKVLDTLCQDKNSIGLVHGEDGKEEAKKVIGRLSALQNEMITNKPIKPLISNGPDISLWNTYLDQQNSMCDLPAAWFDSAWLYVECYLYRRIREAFELSSVLKEYDPFYKQKLESLLIAIDDVEKLAHSLHQTVSNLQNSKDLYSLFVSYLEISLWGNKCDLSISAGKENTLFSYQVDDFKNKILVNNTEKVWNHLQEANERSSEVHIHIVLDNAGFELFGDFCFLHFLLSANLARKISIHVKQLPWFVSDTLDKDIDCLLETMMHSNRKVLVDLSNQWQANLAD	TCTTGTTAAAGTTTTAGATACTTTATGTCAAGATAAGAATTCAATTGGACTTGTTCATGGAGAGGATGGTAAAGAAGAAGCGAAAAAGGTTATTGGTCGATTGTCTGCTCTTCAGAATGAAATGATTACAAATAAACCTATTAAACCATTAATCAGTAATGGGCCTGATATCTCTCTATGGAATACCTATTTAGATCAACAAAACTCTATGTGTGATCTTCCTGCAGCTTGGTTTGATTCTGCTTGGTTATATGTAGAATGTTATCTTTATCGAAGAATTAGAGAAGCTTTTGAATTAAGTTCTGTGTTGAAAGAGTATGATCCCTTTTACAAGCAGAAATTAGAATCATTGTTAATAGCTATTGATGATGTCGAAAAGCTTGCCCATTCGCTGCATCAAACAGTATCAAATCTTCAGAATTCTAAAGATCTGTATAGCCTTTTTGTATCCTACTTAGAGATTTCTTTATGGGGCAACAAGTGTGATTTATCTATATCTGCTGGCAAAGAAAACACCCTCTTCAGTTATCAAGTTGATGATTTTAAAAATAAAATTCTTGTGAATAATACTGAGAAAGTATGGAATCACTTACAAGAAGCCAACGAAAGGTCGAGTGAAGTCCATATTCATATTGTCTTAGATAATGCTGGATTTGAATTGTTTGGCGATTTTTGCTTTCTACATTTTCTATTGTCAGCCAATTTAGCCAGAAAAATTAGCATACATGTAAAGCAACTACCATGGTTTGTATCCGATACGTTAGACAAAGACATTGATTGCTTATTAGAAACTATGATGCACAGTAATCGAAAGGTTTTGGTGGATTTAAGTAATCAGTGGCAAGCAAATTTGGCGGACGG

PhdEnzTra02	Q8MMH3	2,15E-21	53,409	PF01937	14185.15	QANLADGTWKVEKELFWTLPHDFSEMSSVDPKLYSKLSESDLVIFKGDLNYRKLTGDRQWDETTPFKIALNKFLPTSLVSLRTIKADVVVGLSSGMTQKLNSVSLNWKFSGEYALIQYECKEEQ*	GGCAAGCAAATTTGGCGGACGGAACGTGGAAAGTTGAAAAAGAATTATTTTGGACTCTTCCTCATGATTTCAGTGAAATGTCTTCTGTGGATCCCAAATTGTACAGCAAGTTAAGTGAAAGTGATCTGGTGATTTTTAAAGGAGATTTAAATTATCGGAAACTAACTGGAGATCGTCAGTGGGATGAAACCACGCCTTTTAAAATTGCTCTTAATAAATTCTTGCCAACTTCTTTAGTGTCTCTGCGTACCATTAAAGCAGATGTTGTAGTGGGACTTTCATCTGGCATGACTCAAAAGTTGAATAGTGTGTCTCTTAACTGGAAATTTTCCGGGGAATATGCTCTTATTCAATATGAATGTAAAGAAGAACAATAGTTTGATTTGATTTTTATTTAGCCTTTGTAATGTTTTTATTTTGCAACCCTTATAACTTTTCAGAATGCAAGTGCAGACAGTGATAAAGGGTCTAAACAAGAGTTTGGAATTCAAGCCTTATAGAAACAAATCGTAGCTAATAATTATTAGCTGTAAAATACTCTTAAAGATGCTTTTAAGATATCTGTCTGACTATGTTGAAAGGGTTATTTTAAAGAAAATTTCTTAACTTTTGAGTAATCCTGATTCAATGAATACCGTAGGATAAACTCTTTATTATTATCAGTATTCAGTGCTTTACAATTACGTACATATTTATTAACTGTTGTTGGATGCAGTGTATTTTTTAATTTTGATAAATGTTGTTGTTTAATGAGGGATTTTTTTTTCTGACTTAAGAATATGTTTTATTTAAAATTTTACTACAGAATGTAAATTATTTAGCGTGAAGTTATTTTTGATTATTCAGTTTGTTTTTTGAGTTTTGTTATCTTTTTACCAATTTCTCTCAAAAGTGGCAAACACCCATCAACAACTTTATTAGTGCCATTTTGTAACTTTTAAAAAGTATGTGCTACACGTACTAGTAGTTATAATGTGCTGGGTGTTTTTTTTTTTGTTTTTTTTTTTTTTTTT

PhdEnzTra03	A7ISW1	5,05E-79	44,606	PF04389	43460.46<br>**40954.25**	MLSISQLLIIPAILLLR<br>FLVGLS**VSNFSVRG**<br>**VSWSQEATKWHHT**<br>**PARLSDAELHTVST**<br>**VTSDYDDFNRTLD**<br>**AILITRVPGTEGHEK**<br>**VRNFILKEMRSLDW**<br>**DVEEDEFTDRTPHG**<br>**RKSFSNIITTLNPNA**<br>**CRFLTIACHYDSLH**<br>**NREYTFLGATDSAV**<br>**PCAMMIHAAKML**<br>**DAPLKEQKDKNNE**<br>**LTLQFIFFDGEEAFQ**<br>**RWSRTDSLYGSRHL**<br>**AAKWNRMTPFPSG**<br>**LYDGKHCKSKEYAS**<br>**YLDRMDVMVLLDL**<br>**IGAASPKFYSYFPDT**<br>**YGLYSNIVDIENRL**<br>**NSLHLVESHPPEGK**<br>**TNYFDSRSTLAFVE**<br>**DDHIPFMKRGVPII**<br>**HVIPSPFPQVWHRE**<br>**SDNLQNVHHPTVD**<br>**NLNKILRVFIAQYF**<br>**HLNMDVSRNFKIM**<br>**K***	CACGAATTTAAGACAGTCAACATGTCAATGTGAGCAGATTTC<br>GTGAGGTGATAGGTTTCGGTATTGAGGCAGTTTCTACTTATAT<br>ATGTTAACTTATATAACTTAAAAGTTTTTAGTTTCCCACTAGT<br>TTTTTAAACGATGCTCAGCATATCTCAACTGCTGATTATTCCT<br>GCCATATTACTTCTTCGCTTTTTAGTGGGCCTCTCGGTATCAA<br>ATTTTTCTGTGAGAGGTGTCAGCTGGTCTCAAGAAGCAACTA<br>AATGGCATCATACACCTGCAAGATTAAGTGATGCAGAATTA<br>CATACAGTATCTACAGTGACATCTGACTATGATGATTTTAAC<br>AGAACTTTAGATGCCATATTAATAACAAGAGTTCCTGGAACA<br>GAAGGACATGAAAAAGTCAGAAATTTTATTCTGAAAGAAAT<br>GAGATCGTTAGACTGGGATGTTGAAGAAGATGAGTTTACTGA<br>TCGAACACCTCATGGACGTAAATCATTTTCTAATATCATCAC<br>CACATTGAATCCTAATGCATGTAGATTTCTGACTATTGCATGT<br>CATTATGATTCTTTACATAATAGAGAATATACTTTCTTGGGTG<br>CTACAGACTCTGCAGTTCCTTGTGCAATGATGATTCATGCAG<br>CCAAGATGTTGGATGCACCTCTCAAGGAACAAAAAGATAAG<br>AACAATGAACTGACACTTCAGTTTATATTTTTTGATGGTGAA<br>GAAGCATTTCAAAGATGGAGTCGTACTGATTCTCTTTATGGG<br>TCAAGACATTTAGCTGCTAAATGGAATCGTATGACTCCTTTTC<br>CTAGCGGATTATATGATGGCAAGCATTGTAAATCTAAAGAAT<br>ATGCTTCATATTTGGATCGAATGGATGTCATGGTGTTACTGGA<br>TTTAATTGGAGCAGCCAGCCCCAAATTTTATAGTTATTTTCCA<br>GATACATATGGCTTGTATTCTAATATTGTTGACATTGAGAATC<br>GTCTCAACAGCTTACATCTAGTCGAATCCCATCCCCCTGAAG<br>GAAAAACAAATTACTTTGATAGTAGATCAACTTTGGCATTTG<br>TGGAAGATGATCACATCCCTTTTATGAAAAGAGGTGTACCAA<br>TTATTCACGTTATTCCTTCCCCTTTTCCACAAGTTTGGCACCG<br>GGAAAGTGACAATCTTCAAAATGTCCATCATCCTACTGTCGA

							CAATCTGAACAAAATTTTACGTGTATTTATAGCTCAGTATTTC CATTTAAATATGGATGTGTCGAGGAACTTTAAAATAATGAAA TGATATATCTATTTTATATACAAATACTTTGAAACGATGTGCA TTTTAAAAATTTGGTGCAGTTTATTTTTAAGAATCCATGGTTT ATTGAAATTTTCGAGCTATACTTTATTTTTGAATATCCCGTCT ACCATTCTGCATTTAATTGGTCTGTGTATACGCAACAAAATTT AAGTCGTGTTTTGTAATATAAAATCCACTGTTTAGGTATGTAT AAATGTTTTAGCTACATGTCCTATTCTGGATGTGTGTATTGTT CATAGTGATATAAATTTTTCATTGGAAACTTGGTGAAACCTCT AAAATGGTCATCTAAGAACATCTCTGGTACTTAAACAAAATT TTTTTAAAACTTTAACTATTCCTATTTCTTTTGAATTTGAATCT CGATTATTCTCTTTACAAATAGGAAAAAGAAATATTGCTACT AACTTTGAATCACTG

PhdEnzTra04	Q9YIB5	9,22E-15	59,184	PF04389	8357.77	MTYMYAYFIAGVPIIHVIPSPFPQVWHRESDNLQNVHHPTVDNLNKILRVFIAQYFHLNMDVSRNFKIMK*	TTTCCATTTATCTCTGTTCTGTTGTAGTCTTTCAGCTGTGATAATTTGATTGTACTAGGTTTCTGTGATGTCATTTATATTTGGGCACATAGTCTTAACACATTACAAGAAAGTACTCAATGTCATACATTTTATGCGTATGAACTGTATTTTTTCTAGAACCATTCATTACATTCAGGAATATTAAATCAATTTGTAATATTCTAGGTGCTTGTGAACAGAAAAGAAAAATCCAGGTTTTAACTATTAGTTTGAAAATTGATATACAAAATGACCTACATGTATGCTTATTTCATTGCAGGTGTACCAATTATTCACGTTATTCCTTCCCCTTTTCCACAAGTTTGGCACCGGGAAAGTGACAATCTTCAAAATGTCCATCATCCTACTGTCGACAATCTGAACAAAATTTTACGTGTATTTATAGCTCAGTATTTCCATTTAAATATGGATGTGTCGAGGAACTTTAAAATAATGAAATGATATATCTATTTTATATACAAATACTTTGAAACGATGTGCATTTTAAAAATTTGGTGCAGTTTATTTTTAAGAATCCATGGTTTATTGAAATTTTCGAGCTATACTTTATTTTTGAATATCCCGTCTACCATTCTGCATTTAATTGGTCTGTGTATACGCAACAAAATTTAAGTCGTGTTTTGTAATATAAAATCCACTGTTTAGGTATGTATAAATGTTTTAGCTACATGTCCTATTCTGGATGTGTGTATTGTTCATAGTGATATAAATTTTTCATTGGAAACTTGGTGAAACCTCTAAAATGGTCATCTAAGAACATCTCTGGTACTTAAACAAAATTTTTTTAAAACTTTAACTATTCCTATTTCTTTTGAATTTGAATCTCGATTATTCTCTTTACAAATAGGAAAAAGAAATATTGCTACTAACTTTGAATCACTG

PhdEnzTra05	P0DPU3	0	41,941		71509,76	MRSERNYSVAALNV<br>RGIHCFLTEPKRPKM<br>VNVITPLSSQKFYVN<br>ARDEPRIKRSLTFRA<br>KVSLFVAFVLAAVIV<br>TVVVVCFVLPRKEGE<br>QEESFWPTYVSQSKL<br>GRYRHAAVSTDAAP<br>CAPIGKDILERGGTA<br>IDAAIAVLVCMGAH<br>NPHSMGLGGGFLM<br>LYYNRSEERAYYIDA<br>REVAPRKSSVNMFR<br>GNATHAAEGGLAIA<br>VPGELAGYWEAYRL<br>FGFLPWEELFAPTIE<br>MCRSGIEVSAHLAR<br>GIKNNREKLKKYEA<br>LGKVFLKNGTDIYEE<br>GDHIKMLDLAKTLE<br>VISEKKMKALYGPSD<br>LADDFLKDLKEAGSI<br>IDRIDMQSYAPRML<br>DPVKVNLRRNTTLY<br>GVSPPGSGALLAFM<br>MAVLDGYSEMDESV<br>VGDEELTALTYHRIV<br>ETMKFGYAHRSHME	AAACTTATTATTCCGGATTTCGTCGTTTGCATGAGGAGCGAG<br>AGAAATTATTCAGTCGCGGCTCTTAATGTGCGTGGAATACAT<br>TGTTTCCTGACAGAACCGAAAAGGCCGAAGATGGTGAACGT<br>TATCACCCCGTTGAGTTCTCAAAAATTCTATGTCAACGCGAG<br>GGATGAGCCTAGAATTAAGCGGTCGCTGACTTTCCGGGCCAA<br>GGTCTCCCTTTTCGTCGCTTTCGTCCTCGCTGCGGTCATCGTCA<br>CCGTCGTCGTGGTGTGTTTTGTCCTACCCCGGAAAGAAGGGG<br>AGCAGGAGGAGTCTTTCTGGCCCACCTACGTCTCCCAGTCGA<br>AGCTGGGAAGGTATCGGCATGCTGCCGTGTCCACGGATGCTG<br>CCCCGTGCGCGCCCATTGGAAAGGACATTCTCGAGAGGGGT<br>GGGACTGCCATAGACGCCGCCATTGCCGTGCTGGTTTGCATG<br>GGTGCCCACAATCCACACAGCATGGGACTCGGAGGTGGATT<br>CCTCATGCTGTACTACAACAGGTCCGAGGAAAGGGCCTACT<br>ACATCGACGCCAGGGAGGTGGCTCCACGGAAATCGTCTGTG<br>AACATGTTCCGCGGAAATGCGACCCACGCTGCAGAAGGTGG<br>TCTCGCTATCGCTGTACCAGGAGAACTTGCAGGATACTGGGA<br>AGCTTACCGTCTGTTCGGATTCCTTCCTTGGGAAGAACTCTTC<br>GCTCCTACCATCGAGATGTGTCGCAGCGGTATTGAAGTCAGC<br>GCGCACCTTGCCAGAGGCATCAAAAACAACAGGGAAAAGTT<br>GAAGAAGTACGAAGCCCTCGGCAAAGTCTTTCTGAAAAACG<br>GCACGGACATTTACGAGGAGGGAGACCATATCAAGATGCTG<br>GATTTGGCAAAGACGTTAGAAGTCATTTCCGAAAAGAAGAT<br>GAAAGCCCTCTATGGACCGAGCGACCTCGCCGACGATTTTCT<br>GAAGGATCTGAAAGAAGCAGGGAGCATAATCGACCGCATA<br>GACATGCAGAGCTACGCTCCACGCATGCTCGATCCCGTAAA<br>AGTGAATCTCCGGAGGAACACAACCCTGTATGGTGTATCGCC<br>TCCCGGCAGCGGTGCTTTGTTAGCCTTCATGATGGCAGTGCT<br>AGACGGCTACTCCGAAATGGACGAATCCGTGGTCGGCGACG

						DDDSQEMEELIEKLT<br>SKEYADHVRGRIND<br>TRTFDKLSYYGDLW<br>NYAEDHGTAHLSVI<br>APNGDAVSVTSTIN<br>QYFGSKILSPSTGIVL<br>NDEMDDFSSPNITN<br>MFDLPPSAKNNIKP<br>GRRPMSSMTPMILV<br>DAGGEAKLVLGGN<br>GGTTITSGVLQVAAR<br>SLFFGENIKQSIDAPR<br>IHHQLTPNIMHHEK<br>SFPTALVDKLRKFGH<br>DAQLWTMGMMSII<br>MGVQRGEDGYLYA<br>NSDYRKGGEVAGF*	AAGAGCTGACTGCATTGACCTATCATCGCATTGTCGAGACGA<br>TGAAGTTTGGCTACGCGCACAGAAGTCACATGGAGGACGAC<br>GATTCCCAGGAAATGGAAGAGTTAATCGAGAAGCTTACGTC<br>CAAAGAGTACGCCGATCATGTTAGGGGACGTATCAACGACA<br>CCAGAACATTTGACAAACTGTCTTACTATGGGGACTTATGGA<br>ACTATGCTGAAGACCATGGCACAGCACACTTGTCCGTTATTG<br>CACCCAATGGAGATGCAGTCTCTGTCACTTCAACCATAAACC<br>AGTACTTCGGCAGCAAAATACTTTCCCCTTCGACGGGCATCG<br>TCCTCAACGACGAGATGGACGACTTCTCGTCCCCCAACATCA<br>CCAACATGTTTGACCTGCCGCCTTCAGCCAAGAACAACATAA<br>AGCCCGGCAGGAGACCCATGTCTTCTATGACGCCAATGATCC<br>TCGTCGATGCCGGCGGAGAAGCTAAACTGGTGCTCGGAGGA<br>AACGGTGGAACCACTATCACGAGCGGCGTGTTGCAGGTGGC<br>GGCTCGAAGTCTGTTCTTCGGCGAGAATATCAAGCAAAGCAT<br>CGATGCACCCAGGATCCACCACCAACTGACCCCTAACATCA<br>TGCACCACGAAAAGAGCTTTCCTACGGCACTAGTGGACAAA<br>CTTCGCAAATTCGGACACGATGCTCAGCTGTGGACCATGGGC<br>ATGATGAGCATCATCATGGGTGTCCAGAGGGGCGAAGATGG<br>TTATTTGTACGCCAATTCTGATTACAGAAAAGGCGGCGAAGT<br>CGCCGGATTCTAAGATTCCGTTCGAAGGACGAGTACCGACCT<br>AGTTGGACACCCATTGCCTTTGTGAATACATATCACGTGGTTT<br>TAAACACACTCGCAAAGTGATGCTATCTATACCTTTGTGAAA<br>TCTTCGTGAGAGAGTTGTTACAAGCACTGTGAACTATTTTTTT<br>TTTTCAGAAGTCTATATCAATATCTCTCTCTGCATCTGAAGGA<br>GTGGTCAGTATTAGGTTTGTGGTATCACGTGCCGGTGCATTGC<br>TGCACACGCCACACCAAAATACCTTTTATCTTCATCTTCATAT<br>CGTTGTACGAATTCCATTGTTCCTCATCTATTCGTGCTGTGTCT<br>GTCTTTACGGCGGGAGAGTGCTCTTATAATTTCACGCGTTGTA

							TCAGAGCTCCTAAATATCCAGGCTGGATCTCCATCTTGTTGCC<br>CACTGTCGTCGAAACGCACTCCGCGCAGCAGCTTTCTCGCCG<br>ATTCGTAAATATGATCTTTAAGCTGCATTTTTGGGTCGGTTCA<br>TGTAAGTGGATTTATTTTTCTCTCTGAAGCGAATGCGTATGC<br>TTACGTGAGGAAACGTTATTCAAAGTAAGAAAGCCTGCCATT<br>CGAGTGAATTTCATTCAACTCTAATATAAATTTTCGTGAGGTA<br>GGGATCGTGTGTTACTCAAATCTGATCAGATAACAGGAAGA<br>ATACAGTTTTTAATTTCCTTCTGCGGAATGCTTAGTGTCTGCA<br>AAATGAACCAATTAAGTAACTTTTGAAGAAAAAGAGAATTT<br>CTTTAAATATCGGGGTAATATTGGCAACCCCTTTTGACGACG<br>TTGGGCAACAAAATGCCCGATGCGCCAGCTCGGATATGTATA<br>TAGGAGACTTAGTTTGTACTGTGGGCTCATTTCGTGAATGTGA<br>GCGTGTGTCATCAGTGATTTACTTTTTAAGGTATCGTAAACGC<br>TTGACCCCTTAACATGATATCTCGAGAACGCATCGAGAAATT<br>TACATAAAAATTTGAAGGTATGTTTTATGGGCTATTGACAAT<br>GAAATGAACTAAAATCTTGGATGTGGATTTAAATATTTTTTA<br>AATTCGTAGTCGTTTTTATGAGAAACAGTTAACAAAATTTGG<br>GAAAATATCCATTTTATCGTGTATCATTGCCCGCCATTTTCTC<br>GCATTTTGAAATGTCTCAGATTTTAGTTCATGTCAAAGACCG<br>GTGATACGTGTGCCTCGTGTTCGAAGGAAATTAAGATTATTC<br>GATTACAACTGAGTGTAGAGTGGTAGCCGGTTTGGTAGGAAT<br>TCGCTAAAATTGACGAAAAATAACCCATTTTATAAAAATGCG<br>TGACATGTACAATTAAATTTTCAGTTATTTTCGTTGTTTTAGTT<br>CAAAGTTTACCAAAAAACAGTAAACAAAATGTTCCGAACGT<br>AATCGGATGTCTTAAATCGGTAGAAATGAATGGAGACGCAT<br>TTATTCATTCGAGATGTCAAGTGAACGATGCTAACGTTCGAT<br>GAACTGAATGTTCACTGAGCGTTGCCACATTTAGAAAGTAAA<br>TATTGTGAGGTTCCGGATGCAAATTATCGAAATATGCATGAT

							TTTGCCATCACGGAGGATAGCTACAGCAACATTAAAAATAG<br>TAAGCATCCGACTTCCAAAAATTTAAACCACATTTCGAATGG<br>GCCTCATTCGTTGTTTTTGAAGTCTGTTATCTGTTCTAATCTTC<br>AGTAGACATTTTACTGTTTATTTTATTGCACACATTTTCAAAC<br>AAAATGTGTTATACGGTGCGTAAAAGTTTCATTATTTACAGC<br>AATATGCAGTTAAAGAACATTAAGGTATATATGTATATCTTC<br>GGAGACGACTTTGTAAGATTATTTGAAATTTTTGAAACATTTT<br>AAAAATTTATTCTGAAAATTATTTCAAATATGAATATTTTAAT<br>TGGAAAATTTTGTAAGATCTCGACTGATTTTATTTTTCCGTGA<br>AATTTTGCTGAAGTACCGTTCTTAAATCAGGGAAGTATGGTT<br>AAAATAAAAATTTCGCGAAAAAAAAAATGACCTAAATTTGG<br>AATTTTTTATTGAAAGATCTTGCATAAGTAAATGGAGGGAGG<br>GAGGTGGCCCTCAAACGGTATGTCCTGAGGCAGTGGCGCAC<br>TCAGGATTTAATCTTGGAGGGGGTCGAGTTGAGCGGAAAAA<br>AATATTTTCAATGAATTTTCAGTAAAATTTATTAGGTAATTAT<br>AAGAATTTGGTTAGAGAATAAAATCTATTTTCTTTTTTTTTTA<br>ACAAAGCTCTTGAATTATATTAGTCGTGGTACATTATAGCAA<br>AGCCAATCTGTTTAATCGTGATCCTGTCATATTTTTCTAGTGTT<br>TCTTTTTCAGTGGAGGGGACCATGGCACCTTGTCCCTCCCCTC<br>GAACAAGGGGTCCGCCGGTACGGTACCTACTTCTTCCCAGAC<br>TGAGTGTGCCACTGCCCTAAGGCAACACCTGCTAGAAAGCA<br>GGTGTTGCCTAGGGCATGGTCATAGGAATGGAGGAAGCAGG<br>AGCATAGGCAGCTCAAGCCAGTCCCTCTTCATTTAGAATAAA<br>AATTTCCGAACGCAATATTTCACGATGCGTAAATTTCGTACG<br>AAAATTTCTAAAATCAGATTTTAATTATTATTTACTGAGAAA<br>AAAAAACGTTAATTTATTAGCAGAATTTAAGAAGTAATTGGA<br>TTGAAAATCAAGATGAATAA

Glutamate synthase							
**Transcript ID**	**Uniprot ID reference protein**	**e-value**	**Identity (%)**	**PFAM domain**	**MW (Da)**	**Amino acid sequence**	**Nucleotide sequence**

						MTPLAGRFLKVVVL<br>HPLLGPCSRSRPFPRS<br>KMSGPTLEYQGLPPP<br>RGLYHPQYEKEACG<br>VGFIVNINGISSHKLI<br>ADAATISKRMEHRG<br>ACACDNSTGDGAG<br>VTAAIPHSFYSQMLR<br>EELSISLPPKGQYAT<br>GILFLDTQTSSAAER<br>QFELLAKELHLEVLC<br>WRDVPVDSSILGYV<br>AKANEPLMRQVFVV<br>GPSMDADTFRREVF<br>VLRKYATHKIPTSDL<br>RFYICSLSPDTVVYK<br>GQLTSTQLWSYFTDL<br>QNPSFETYLALVHA<br>RFSTNTFPSWERAHP<br>MRILAHNGEINTLR<br>GNVNLMRAREGVM<br>ESKLFGDELHKLYPV<br>VEPNLSDSGCADNV<br>LEFLVMAGERTLPE<br>AVMTMVPEAWQN	TTTCGTACCACTTTTATTTGGCGACGTTTTATATAATGACTCC<br>ACTCGCCGGGCGTTTCCTAAAGGTTGTAGTGCTCCATCCGTTA<br>CTGGGTCCGTGTAGTAGAAGTCGCCCTTTTCCCCGGTCCAAG<br>ATGTCAGGTCCGACGCTGGAGTACCAGGGCTTGCCACCGCCC<br>AGAGGCCTCTACCACCCTCAGTACGAAAAAGAAGCGTGTGG<br>AGTCGGATTCATAGTCAACATCAACGGAATCTCTTCTCACAA<br>ACTCATCGCTGATGCCGCCACCATTTCAAAACGCATGGAACA<br>TCGCGGAGCCTGTGCCTGCGACAATAGCACTGGAGATGGGG<br>CAGGAGTCACAGCTGCCATACCCCACTCCTTCTACTCGCAGA<br>TGCTCAGGGAAGAACTAAGCATATCTCTTCCACCCAAAGGCC<br>AATACGCGACTGGCATATTATTTCTAGACACGCAGACCTCCA<br>GTGCAGCCGAAAGACAATTCGAGCTTTTAGCCAAAGAGCTT<br>CACTTGGAGGTTCTTTGTTGGCGCGACGTTCCCGTCGACAGTT<br>CCATCCTGGGTTATGTCGCCAAAGCCAATGAACCCCTTATGA<br>GGCAAGTTTTCGTCGTGGGACCTTCCATGGACGCTGACACAT<br>TCCGCAGAGAAGTTTTTGTCTTAAGAAAATACGCTACTCATA<br>AAATTCCTACTTCAGACCTTCGCTTCTATATATGTAGTTTATC<br>ACCCGACACTGTTGTATATAAAGGGCAACTAACATCCACTCA<br>ACTATGGAGTTACTTCACTGATCTCCAGAACCCTTCTTTCGAA<br>ACATATCTCGCTTTAGTGCACGCCAGATTCTCAACCAATACC<br>TTTCCCAGTTGGGAAAGAGCTCATCCAATGAGAATTTTAGCA<br>CATAATGGTGAAATCAATACTCTTCGTGGAAATGTCAACCTC<br>ATGCGTGCCCGAGAGGGTGTAATGGAAAGTAAACTATTTGG<br>AGACGAGTTACACAAGTTGTATCCAGTTGTAGAACCCAACCT<br>GTCCGACTCCGGTTGTGCCGATAATGTTCTTGAATTTTTGGTT<br>ATGGCTGGAGAACGAACCCTTCCAGAAGCTGTTATGACCATG<br>GTGCCTGAAGCTTGGCAAAATGATGATCAAATGAATGCTGA<br>CAAAAAAGCATTCTACAGGTGGAGTGGCTGTGTAATGGAAC<br>CTTGGGATGGTCCCGCCTTGTTGACATTTACTGATGGTAAGCT<br>GATAGGTGCTATTTTGGACAGAAATGGTTTGAGACCATCCAG<br>ATATTACTTAACCAAGGATAATCATATGGTGATGGCCAGTGA<br>AGTTGGTGTATTAGATCTGGAACATTCTAATGTTGTACAGAA<br>GGGACGGTTAAGACCTGGACGCATGTTGTTAGTTGACACTGA<br>[illegible]

PhdEnzGlS02	P81383	7,82E-05	54,054	PF01593	229151,9	MTPLAGRFLKVVVLHPLLGPCSRSRPFPRSKMSGPTLEYQGLPPPRGLYHPQYEKEACGVGFIVNINGISSHKLIADAATISKRMEHRGACACDNSTGDGAGVTAAIPHSFYSQMLREELSISLPPKGQYATGILFLDTQTSSAAERQFELLAKELHLEVLCWRDVPVDSSILGYVAKANEPLMRQVFVVGPSMDADTFRREVFVLRKYATHKIPTSDLRFYICSLSPDTVVYKGQLTSTQLWSYFTDLQNPSFETYLALVHARFSTNTFPSWERAHPMRILAHNGEINTLRGNVNLMRAREGVMESKLFGDELHKLYPVVEPNLSDSGCADNVLEFLVMAGERTLPEAVMTMVPEAWQNDDQMNADKKAFYRWSGCVMEPWDGPALLTFTDGKLIGAILD	TTTCGTACCACTTTTATTTGGCGACGTTTTATATAATGACTCCACTCGCCGGGCGTTTCCTAAAGGTTGTAGTGCTCCATCCGTTACTGGGTCCGTGTAGTAGAAGTCGCCCTTTTCCCCGGTCCAAGATGTCAGGTCCGACGCTGGAGTACCAGGGCTTGCCACCGCCCAGAGGCCTCTACCACCCTCAGTACGAAAAAGAAGCGTGTGGAGTCGGATTCATAGTCAACATCAACGGAATCTCTTCTCACAAACTCATCGCTGATGCCGCCACCATTTCAAAACGCATGGAACATCGCGGAGCCTGTGCCTGCGACAATAGCACTGGAGATGGGGCAGGAGTCACAGCTGCCATACCCCACTCCTTCTACTCGCAGATGCTCAGGGAAGAACTAAGCATATCTCTTCCACCCAAAGGCCAATACGCGACTGGCATATTATTTCTAGACACGCAGACCTCCAGTGCAGCCGAAAGACAATTCGAGCTTTTAGCCAAAGAGCTTCACTTGGAGGTTCTTTGTTGGCGCGACGTTCCCGTCGACAGTTCCATCCTGGGTTATGTCGCCAAAGCCAATGAACCCCTTATGAGGCAAGTTTTCGTCGTGGGACCTTCCATGGACGCTGACACATTCCGCAGAGAAGTTTTTGTCTTAAGAAAATACGCTACTCATAAAATTCCTACTTCAGACCTTCGCTTCTATATATGTAGTTTATCACCCGACACTGTTGTATATAAAGGGCAACTAACATCCACTCAACTATGGAGTTACTTCACTGATCTCCAGAACCCTTCTTTCGAAACATATCTCGCTTTAGTGCACGCCAGATTCTCAACCAATACCTTTCCCAGTTGGGAAAGAGCTCATCCAATGAGAATTTTAGCACATAATGGTGAAATCAATACTCTTCGTGGAAATGTCAACCTCATGCGTGCCCGAGAGGGTGTAATGGAAAGTAAACTATTTGGAGACGAGTTACACAAGTTGTATCCAGTTGTAGAACCCAACCTGTCCGACTCCGGTTGTGCCGATAATGTTCTTGAATTTTTGGTTATGGCTGGAGAACGAACCCTTCCAGAAGCTGTTATGACCATGGTGCCTGAAGCTTGGCAAAATGATGATCAAATGAATGCTGACAAAAAAGCATTCTACAGGTGGAGTGGCTGTGTAATGGAAC

						RNGLRPSRYYLTKD	CTTGGGATGGTCCCGCCTTGTTGACATTTACTGATGGTAAGCT
						NHMVMASEVGVLD	GATAGGTGCTATTTTGGACAGAAATGGTTTGAGACCATCCAG
						LEHSNVVQKGRLRP	ATATTACTTAACCAAGGATAATCATATGGTGATGGCCAGTGA
						GRMLLVDTENHLFI	AGTTGGTGTATTAGATCTGGAACATTCTAATGTTGTACAGAA
						KDEELKLQISRKRPL	GGGACGGTTAAGACCTGGACGCATGTTGTTAGTTGACACTGA
						GSWLKEMFTLEDLF	AAATCATCTTTTCATCAAAGATGAAGAATTAAAGTTACAAAT
						AAGFAAGHHTAHP	TTCTCGCAAACGCCCTTTAGGCAGCTGGTTAAAAGAAATGTT
						KLDQTKRTTSLAVE	TACACTAGAAGATCTGTTTGCTGCCGGTTTTGCAGCTGGACA
						DRRLALFAYTIETIN	TCATACTGCTCATCCCAAGTTAGATCAAACTAAGAGAACAA
						MLIVPMVETKKEAL	CTAGTTTAGCTGTTGAAGATAGGCGTCTTGCACTGTTTGCCTA
						GSMGNDAPLACLS	TACTATTGAAACAATAAATATGTTGATTGTACCCATGGTTGA
						MYQPLIYDYFKQLFA	AACAAAGAAAGAAGCCCTTGGTTCAATGGGAAATGATGCAC
						QVTNPPIDPFRERIV	CTTTAGCATGCCTGTCCATGTACCAGCCACTGATCTATGATTA
						MSLGCPIGPAANILE	CTTTAAACAACTCTTTGCTCAAGTGACAAATCCACCTATCGA
						PSAQQCCRLYLEQPI	TCCATTTCGTGAAAGAATAGTGATGTCATTGGGTTGTCCTATT
						LSLKDIEALKTTTYK	GGTCCAGCAGCTAACATCTTAGAACCTAGTGCTCAACAGTGC
						GWKTKVIDMVYPV	TGTCGGCTGTATTTGGAACAGCCTATATTATCTCTCAAAGAC
						NEAADGLLPALKRI	ATAGAAGCTTTGAAAACAACTACATATAAAGGATGGAAAAC
						CDEACSAAENGYSL	TAAAGTGATTGACATGGTTTATCCTGTAAATGAAGCAGCAGA
						VILSDRKAGRDYIPV	TGGCCTTTTGCCTGCCTTAAAAAGAATCTGTGATGAAGCTTG
						SALLALGATHHHLI	CAGTGCTGCTGAAAACGGATATTCTTTGGTTATACTTTCAGAC
						NNLQRMKVGLILET	AGAAAAGCTGGAAGAGATTATATTCCTGTTAGTGCATTACTA
						GEAREVHHMCVLL	GCTTTAGGTGCCACTCATCATCATTTGATTAATAATTTACAAA
						GYGADAICPYLVFES	GAATGAAAGTTGGTCTCATATTAGAAACTGGAGAAGCAAGA
						MAELQGEGVISETLT	GAAGTACATCACATGTGTGTATTATTGGGATATGGAGCTGAT
						EDMIFKNYVEAMER	GCCATATGCCCATACCTTGTGTTTGAAAGCATGGCTGAATTA
						GIAKVMAKMGISTL	CAAGGAGAAGGTGTCATTAGCGAGACTCTGACAGAGGACAT
						QSYKGAQIFEAVGLS	GATTTTTAAAAATTATGTTGAAGCGATGGAGCGAGGAATTGC

						EEVVDFCFKNTPSRI<br>GGVTFRVLAEENLE<br>RYRIAYSDRDCDNIV<br>LRNPGNYHWRSGG<br>EKHINDPLAIANLQ<br>DAARSKSQSAYANF<br>VETTMQNVRACTLR<br>GQFDLVKVPKPVDIS<br>QVEEAKEIVKRFATG<br>AMSFGSISLEAHKTL<br>AAAMNRVGGKSNT<br>GEGGEDPDRFIVTDS<br>NNNFRSAIKQVASG<br>RFGVTIEYLANADEL<br>QIKMAQGAKPGEG<br>GELPGYKVSKDIAKT<br>RHSIPGVGLISPPPH<br>HDIYSIEDLAELIYDL<br>KCANPTARISVKLVS<br>EVGVGVVASGVAKG<br>KAEHITISGHDGGTG<br>ASSWTGIKGAGLPW<br>ELGIAETHQTLVQN<br>DLRSRVVLQADGQI<br>RTGFDVVVAAILGA<br>DEFGFSTAPLIVLGC<br>TMMRKCHLNTCPV<br>GIATQDPELRKKFEG	CAAGGTAATGGCAAAAATGGGAATTTCTACTCTACAAAGTTA<br>TAAAGGGGCTCAAATTTTTGAAGCAGTTGGTCTGAGTGAAGA<br>AGTTGTGGATTTCTGTTTTAAAAATACACCCTCTCGCATTGGT<br>GGTGTGACATTTAGAGTTTTAGCTGAAGAAAATCTTGAACGC<br>TACCGTATTGCCTATAGTGATAGAGACTGTGACAATATTGTTT<br>TGAGAAATCCTGGAAATTACCACTGGAGAAGTGGAGGAGAG<br>AAACATATTAATGATCCTTTAGCTATAGCAAACTTACAGGAT<br>GCTGCCCGATCAAAAAGTCAAAGTGCCTATGCCAATTTTGTG<br>GAAACTACCATGCAGAATGTTAGAGCTTGCACTTTAAGAGGT<br>CAGTTTGACCTTGTAAAAGTACCTAAACCTGTAGATATTAGC<br>CAAGTGGAAGAGGCTAAAGAAATAGTGAAGCGCTTTGCTAC<br>TGGTGCCATGAGTTTTGGTAGTATATCATTAGAAGCTCATAA<br>AACTCTTGCTGCTGCAATGAATAGAGTTGGAGGCAAATCTAA<br>TACTGGAGAAGGTGGTGAAGATCCTGACAGATTCATTGTTAC<br>AGACTCAAATAACAATTTTCGCTCAGCTATCAAACAGGTTGC<br>ATCTGGCAGATTTGGGGTTACCATCGAATATCTTGCAAATGC<br>TGATGAACTTCAGATTAAGATGGCACAAGGAGCCAAACCTG<br>GGGAAGGTGGTGAACTTCCTGGTTATAAAGTCTCAAAAGAC<br>ATTGCTAAAACTAGACATTCTATTCCTGGAGTGGGACTTATA<br>AGTCCCCCTCCCCATCATGATATCTATTCTATTGAAGATTTAG<br>CTGAGCTCATCTATGATTTAAAATGTGCAAACCCTACAGCTC<br>GAATTTCTGTGAAACTTGTGTCAGAAGTTGGAGTAGGAGTTG<br>TTGCATCTGGTGTTGCCAAAGGAAAAGCAGAACATATCACC<br>ATATCAGGACATGATGGAGGAACAGGGGCCAGCAGTTGGAC<br>AGGAATAAAAGGAGCTGGTCTCCCTTGGGAACTTGGAATTG<br>CTGAGACTCATCAAACACTTGTACAAAATGATTTACGCTCGC<br>GGGTTGTTTTGCAAGCTGATGGACAGATTCGAACAGGATTCG<br>ACGTAGTGGTTGCTGCCATTCTTGGAGCCGATGAATTTGGCTT

						KPEHVVNYLFMLAE	TTCAACTGCACCTTTGATAGTCCTCGGATGTACAATGATGAG
						DVRSHMASLGIKRF	GAAGTGCCATTTGAACACCTGCCCTGTCGGCATTGCCACTCA
						QDLIGRTDLLKVYH	GGATCCAGAGCTCAGGAAGAAATTCGAAGGAAAGCCAGAA
						NKDNPKAQMLDYS	CATGTTGTGAATTACCTGTTCATGCTTGCTGAAGATGTACGTT
						AILMDALSLRPGTLI	CTCATATGGCGTCATTGGGTATAAAGAGATTCCAAGACTTGA
						VGGSLKQDFALENRI	TTGGACGCACAGATTTGCTGAAAGTTTACCATAATAAAGACA
						DNLLIKQAADVIDR	ATCCGAAAGCTCAGATGCTTGATTATTCTGCCATACTCATGG
						RKKDVTIHMDISNV	ATGCCTTGTCTTTGAGGCCTGGAACCTTAATTGTTGGTGGATC
						DRAFGSTLSYHIAKK	TCTTAAGCAAGACTTTGCTTTAGAAAACAGAATTGACAATCT
						HGSSGLPNNSIKIKL	GCTTATTAAACAAGCTGCTGATGTTATAGACAGGCGTAAAAA
						EGSAGQSFCAFLAP	AGATGTGACCATTCATATGGACATCTCCAATGTTGATAGAGC
						GVTVELEGDANDYV	ATTTGGATCAACACTAAGTTATCACATAGCGAAAAAACATG
						GKGLSGGTVIVYPPT	GTTCAAGTGGCTTACCTAATAATAGCATCAAAATAAAACTAG
						RLPQDFKSEENIIVG	AAGGATCAGCTGGTCAAAGTTTTTGTGCATTTTTAGCTCCTGG
						NVCLYGATDGKAFF	AGTAACAGTTGAGCTAGAAGGTGATGCTAATGACTATGTAG
						RGIAAERFCVRNSG	GAAAGGGATTATCTGGTGGAACTGTAATTGTATATCCCCCTA
						ATAVIEGVGDHGCE	CGCGTTTACCTCAGGATTTCAAATCTGAAGAAAATATTATTG
						YMTGGYCLILGPTG	TAGGCAATGTTTGCTTGTATGGTGCTACAGATGGAAAAGCCT
						RNFAAGMSGGIAYV	TCTTCAGAGGGATTGCTGCTGAACGTTTCTGTGTGAGGAACT
						LDENHKLESNSNLM	CTGGTGCTACTGCTGTTATTGAGGGTGTTGGTGACCATGGTTG
						MVDLLPLELPEDLSF	TGAGTACATGACTGGTGGCTACTGTCTGATTCTTGGTCCAACT
						VESLLREFFEKTGSEI	GGACGTAATTTTGCTGCAGGCATGTCTGGTGGTATTGCTTATG
						AQRILSNWPASAKSF	TCTTGGATGAGAATCATAAACTTGAATCTAATAGTAATTTGA
						VKVFPKEYQRALRQ	TGATGGTTGACTTATTGCCACTTGAATTGCCTGAAGACCTTAG
						AEEEKKQALTNGPT	TTTTGTAGAGAGCTTATTGAGAGAATTTTTTGAAAAAACAGG
						PAVETKPAVKDIED	TTCCGAAATTGCTCAGCGTATACTCTCTAACTGGCCAGCGTCT
						VIPKLDKIRGFMKYK	GCAAAATCTTTTGTTAAGGTGTTCCCTAAAGAGTACCAACGT
						RMTTPYREPADRLH	GCCTTACGCCAAGCTGAGGAAGAAAAGAAACAAGCTTTAAC

						DWDEIYDFKGVRKTI<br>KVQAARCMDCGVP<br>FCQSSHGCPLGNIIP<br>KWNDLVFKDDWRE<br>ALNQLLQTNNFPEF<br>TGRVCPAPCEGACV<br>LGINEPPVNIKHIEC<br>AIIDNAFDKGWMIP<br>QIPAVRTGKKIAIIGS<br>GPSGLAAAAQLNK<br>AGHTIHVYERNDRV<br>GGLLEYGIPTMKLG<br>KDVVKRRVDLLAAE<br>GITFHTNVNVGKDIS<br>GKSLLEETDALLLTV<br>GATWPRDLPIPGRD<br>LKGIHFAMNFLETW<br>QKHQLGNKIDYSKV<br>NAKDKNVVVIGGG<br>DTGCDCIATSLRQG<br>AKSIVTFEILPEPPEK<br>RADNNPWPQWPKV<br>FRVDYGHDEVKVK<br>WGDDPRRFCVLSEK<br>FLDNGNGDVSGISTI<br>KVEWKKDASGNWA<br>MEKIPGSEKVFKADL<br>VLIAMGFLSPEQYLL	AAATGGGCCAACTCCTGCTGTTGAAACTAAACCTGCTGTCAA<br>AGATATTGAAGACGTTATTCCTAAATTGGATAAAATAAGAG<br>GTTTTATGAAATACAAACGCATGACTACTCCATACCGTGAAC<br>CAGCTGATCGACTTCATGACTGGGATGAAATCTATGACTTTA<br>AGGGTGTGCGTAAAACTATAAAAGTACAAGCAGCCAGGTGT<br>ATGGATTGTGGTGTTCCATTCTGTCAGTCAAGTCATGGCTGCC<br>CTCTTGGAAATATCATACCTAAATGGAATGATCTTGTTTTCAA<br>GGATGACTGGAGAGAAGCTCTTAATCAATTACTGCAGACAA<br>ATAATTTTCCAGAATTCACTGGTCGAGTTTGTCCAGCCCCTTG<br>TGAGGGAGCTTGTGTTCTGGGTATCAATGAACCCCCAGTGAA<br>TATAAAGCATATTGAGTGTGCCATCATTGACAATGCCTTTGA<br>TAAAGGGTGGATGATTCCTCAAATTCCAGCTGTAAGAACTGG<br>TAAGAAAATTGCTATCATTGGGAGTGGACCGTCTGGTCTTGC<br>AGCTGCTGCACAACTAAACAAGGCTGGGCATACAATTCATG<br>TATATGAGAGAAATGACAGGGTTGGTGGTTTGTTGGAATATG<br>GAATTCCAACTATGAAGCTCGGTAAAGATGTTGTAAAAAGA<br>CGGGTAGACCTTCTTGCAGCCGAAGGTATTACTTTCCATACA<br>AATGTAAATGTAGGCAAAGACATTTCTGGAAAATCTCTTCTT<br>GAAGAGACTGATGCTCTGCTATTAACAGTTGGAGCTACTTGG<br>CCAAGAGACCTTCCAATACCAGGAAGAGATTTGAAAGGAAT<br>TCACTTTGCAATGAATTTCCTGGAGACCTGGCAAAAGCACCA<br>GTTGGGTAACAAAATTGACTATTCCAAGGTGAACGCCAAAG<br>ATAAAAATGTGGTTGTGATTGGCGGTGGTGACACTGGTTGTG<br>ACTGCATAGCTACTTCTCTCAGACAGGGTGCCAAAAGTATTG<br>TGACATTTGAAATTCTTCCTGAACCTCCAGAAAAGAGAGCTG<br>ACAATAACCCATGGCCTCAATGGCCAAAAGTGTTCAGAGTA<br>GATTATGGCCATGACGAGGTGAAAGTGAAATGGGGGGATGA<br>TCCAAGAAGATTCTGTGTTCTCAGTGAGAAATTCTTGGATAA

						NDVGVQQDPRKNV<br>ATPNGNYRTSIPKVY<br>AAGDCRRGQSLVV<br>HAISEGRQAARQID<br>ADLMQCSSLAGPGG<br>VIPYVNELSVAA*	TGGTAACGGCGATGTGTCAGGTATTAGTACCATAAAAGTTGA<br>ATGGAAGAAGGATGCTTCAGGAAATTGGGCGATGGAAAAAA<br>TTCCTGGCTCAGAAAAAGTATTCAAGGCGGATTTAGTTTTAA<br>TTGCTATGGGATTCCTTAGCCCTGAACAGTATCTTTTGAATGA<br>TGTTGGAGTACAACAAGATCCAAGAAAAAATGTTGCCACTC<br>CCAACGGAAACTATCGCACATCTATTCCCAAAGTTTATGCTG<br>CAGGAGATTGCCGCAGAGGCCAGTCATTAGTTGTACATGCG<br>ATTTCTGAAGGACGGCAGGCCGCCCGTCAGATTGATGCTGAT<br>CTTATGCAATGTTCCTCATTGGCTGGTCCTGGAGGTGTCATAC<br>CTTATGTCAATGAACTCTCTGTGGCTGCTTAAATATGTTTCTC<br>TGTAGTTTCATGAATCAAGAATGTACACTACAGCTGGTAGTT<br>ATGCTTATAAAACACAAGACTAATGAAATTGGCAGCAAATA<br>ATCATTAGTTTCTCATAGGTGGAACGATGTACCAGAATGGGA<br>CGAGTGTGTTCATCAGTGAGTGTTCATAGTTGTTGTATATTTA<br>AGGATTTGGCCTGTGTAATATTTTTAATTTTATGAGGAATGCA<br>TTGTTCGTTTATTACATCCGATGTGTTGGCTTTTTGTAAGTGAG<br>TCTAAGTTGTTGGTGTGTATATATATCTCAGATGTTTGCTGTC<br>CAGTTTGTTTTTTAAACATTGTGTAGCACTTGACTTCAATCAT<br>TAAACTCTACCTTGATGCCATTTAATTCTTGTATAGTTATCAT<br>ATTGCTTGTAGTGTATTGCTGGAATTTCATGCATTCGATAAAC<br>ATTTTAATTCACTTGTTTGCAGCATCTTCTATCCAGAAATATA<br>AAACTTGGCATGCACACATAGATATACTTAAATCTATTGAAG<br>TTTAAATAAAGGTTGTTTTACTGTAGATTCTACCAATTAGCCT<br>AAATTGCCATTGACTGTGCATGTTTGAGAAAGTTGCCGTTTTG<br>AAGTCTTCCATAAAAGAGGTGCTGACTGTTACGTTGGTTCCA<br>GCTAATACTTTTTTCTTCGCAGTAATAAATACAGCAAAACAA<br>ACTTTTATGCTTTTAATTTAGGAATATAGGGTTCTTGATAGGG<br>TCTGACTTTCACCCAAAAATGACCTTAAAATAAAAAAATTAAA

							AAAACAAAACAAAAAAAAGTGCGGTTTAGAAATTTCTGAAT<br>TGTTTTTACTGAATTAATTCTGAAACATTAGAAACTTAGGTTT<br>AGGTGATTTAAACAGCATACCATATCAGATATCAATGCCTGT<br>TTCTCTGAAAATTGCATCTTTTTAAAATATAGACACACGGAG<br>AGCCCTATGGTAAATTAATGTATCTATCTTACTTTTAAAAGAA<br>CAATGTGTGAAGTATTTTATCAGACTACTATAGAGAACAATC<br>ACACCTAGCCATTTATATTAAATATATTTCTTTAGTATTCATA<br>ATAGAGAGATAATAGTTTGTGTATAAGTGAGTATGTTGTGCC<br>TTGTTGAAAACAATAGTTTGCAAATGTATCTGTTTGCCTGGTA<br>TTGTAAATGGTAATTAAGATATTTTAGATGTATTTTCAGATTA<br>TAGCACCTCTTTTTAATAGCTTATAAATACCATTCTGCTCTAT<br>AATACTGATTTTGATCTAAGGTGCCAACCATTTCACAAATAA<br>CTGAATTTTAGTGAAATATGCAAGTATAATATTAAAGTAAAT<br>TGTATAAGCATAAAATGCTGTATAGGTGTAGCTAGAGTTAAA<br>AGAGGAAGACGAGTGAAATTAAACAGTATGATAGATGTGTT<br>GTTCTTTTCCTTGACACTGCAGGTAGAATTCGATACTACATCA<br>CTTATTGCACAATCACAATATAAACTTATATTAAAAAGTTGC<br>AAAGAGAGGTTATATCTCATAATTTTGTTTTACATGAAATAA<br>AGGTTGGCACCTTATTTTTTTACTATCTTTTTTGCCTTATCTGTC<br>AATACATTTGCAATTATTTTTATCCAGTGTTGACTTTATTGTTC<br>TATTTTACTATTTCAAGCTTTCGTGATGTACCACGATGTGTAA<br>GCATATCCCTGTAAACATTCCATAATAGTACTGTAATAAAGG<br>AATTGGGTTTATTACCCCATATACATTTAAACTGGCCTATTGA<br>AGTTTTCAAAGTTAAAATTAGATCAATCCTTAAATATACTGC<br>ACCAGAAAATAGAGCTTTAAGATTACTCGGTGTAAGAATAA<br>AATC

PhdEnzGlS03	P81383	7,87E-05	54,054	PF01593	228392,88	NLEVVVLHPLLGPC<br>SRSRPFPRSKMSGPT<br>LEYQGLPPPRGLYHP<br>QYEKEACGVGFIVNI<br>NGISSHKLIADAATIS<br>KRMEHRGACACDN<br>STGDGAGVTAAIPH<br>SFYSQMLREELSISLP<br>PKGQYATGILFLDTQ<br>TSSAAERQFELLAKE<br>LHLEVLCWRDVPVD<br>SSILGYVAKANEPLM<br>RQVFVVGPSMDADT<br>FRREVFVLRKYATH<br>KIPTSDLRFYICSLSP<br>DTVVYKGQLTSTQL<br>WSYFTDLQNPSFETY<br>LALVHARFSTNTFPS<br>WERAHPMRILAHN<br>GEINTLRGNVNLMR<br>AREGVMESKLFGDE<br>LHKLYPVVEPNLSDS<br>GCADNVLEFLVMA<br>GERTLPEAVMTMVP<br>EAWQNDDQMNAD<br>KKAFYRWSGCVMEP<br>WDGPALLTFTDGKL<br>IGAILDRNGLRPSRY	GAAAGGCATTACAGCCCGTAACCGCGCGATTTCACTTTCTCT<br>CCGAACCGGTGTTCTCGTGGATGCTTGCTATCTGGCAGGTGA<br>ATTTCGCAGACGAGCTCTTAAAACTTGGAAGTTGTAGTGCTC<br>CATCCGTTACTGGGTCCGTGTAGTAGAAGTCGCCCTTTTCCCC<br>GGTCCAAGATGTCAGGTCCGACGCTGGAGTACCAGGGCTTG<br>CCACCGCCCAGAGGCCTCTACCACCCTCAGTACGAAAAAGA<br>AGCGTGTGGAGTCGGATTCATAGTCAACATCAACGGAATCTC<br>TTCTCACAAACTCATCGCTGATGCCGCCACCATTTCAAAACG<br>CATGGAACATCGCGGAGCCTGTGCCTGCGACAATAGCACTG<br>GAGATGGGGCAGGAGTCACAGCTGCCATACCCCACTCCTTCT<br>ACTCGCAGATGCTCAGGGAAGAACTAAGCATATCTCTTCCAC<br>CCAAAGGCCAATACGCGACTGGCATATTATTTCTAGACACGC<br>AGACCTCCAGTGCAGCCGAAAGACAATTCGAGCTTTTAGCC<br>AAAGAGCTTCACTTGGAGGTTCTTTGTTGGCGCGACGTTCCC<br>GTCGACAGTTCCATCCTGGGTTATGTCGCCAAAGCCAATGAA<br>CCCCTTATGAGGCAAGTTTTCGTCGTGGGACCTTCCATGGAC<br>GCTGACACATTCCGCAGAGAAGTTTTTGTCTTAAGAAAATAC<br>GCTACTCATAAAATTCCTACTTCAGACCTTCGCTTCTATATAT<br>GTAGTTTATCACCCGACACTGTTGTATATAAAGGGCAACTAA<br>CATCCACTCAACTATGGAGTTACTTCACTGATCTCCAGAACC<br>CTTCTTTCGAAACATATCTCGCTTTAGTGCACGCCAGATTCTC<br>AACCAATACCTTTCCCAGTTGGGAAAGAGCTCATCCAATGAG<br>AATTTTAGCACATAATGGTGAAATCAATACTCTTCGTGGAAA<br>TGTCAACCTCATGCGTGCCCGAGAGGGTGTAATGGAAAGTA<br>AACTATTTGGAGACGAGTTACACAAGTTGTATCCAGTTGTAG<br>AACCCAACCTGTCCGACTCCGGTTGTGCCGATAATGTTCTTG<br>AATTTTTGGTTATGGCTGGAGAACGAACCCTTCCAGAAGCTG<br>TTATGACCATGGTGCCTGAAGCTTGGCAAAATGATGATCAAA

						YLTKDNHMVMASE<br>VGVLDLEHSNVVQK<br>GRLRPGRMLLVDTE<br>NHLFIKDEELKLQIS<br>RKRPLGSWLKEMFT<br>LEDLFAAGFAAGHH<br>TAHPKLDQTKRTTS<br>LAVEDRRLALFAYTI<br>ETINMLIVPMVETKK<br>EALGSMGNDAPLAC<br>LSMYQPLIYDYFKQL<br>FAQVTNPPIDPFRERI<br>VMSLGCPIGPAANIL<br>EPSAQQCCRLYLEQP<br>ILSLKDIEALKTTTYK<br>GWKTKVIDMVYPV<br>NEAADGLLPALKRI<br>CDEACSAAENGYSL<br>VILSDRKAGRDYIPV<br>SALLALGATHHHLI<br>NNLQRMKVGLILET<br>GEAREVHHMCVLL<br>GYGADAICPYLVFES<br>MAELQGEGVISETLT<br>EDMIFKNYVEAMER<br>GIAKVMAKMGISTL<br>QSYKGAQIFEAVGLS<br>EEVVDFCFKNTPSRI	TGAATGCTGACAAAAAAGCATTCTACAGGTGGAGTGGCTGT<br>GTAATGGAACCTTGGGATGGTCCCGCCTTGTTGACATTTACT<br>GATGGTAAGCTGATAGGTGCTATTTTGGACAGAAATGGTTTG<br>AGACCATCCAGATATTACTTAACCAAGGATAATCATATGGTG<br>ATGGCCAGTGAAGTTGGTGTATTAGATCTGGAACATTCTAAT<br>GTTGTACAGAAGGGACGGTTAAGACCTGGACGCATGTTGTTA<br>GTTGACACTGAAAATCATCTTTTCATCAAAGATGAAGAATTA<br>AAGTTACAAATTTCTCGCAAACGCCCTTTAGGCAGCTGGTTA<br>AAAGAAATGTTTACACTAGAAGATCTGTTTGCTGCCGGTTTT<br>GCAGCTGGACATCATACTGCTCATCCCAAGTTAGATCAAACT<br>AAGAGAACAACTAGTTTAGCTGTTGAAGATAGGCGTCTTGCA<br>CTGTTTGCCTATACTATTGAAACAATAAATATGTTGATTGTAC<br>CCATGGTTGAAACAAAGAAAGAAGCCCTTGGTTCAATGGGA<br>AATGATGCACCTTTAGCATGCCTGTCCATGTACCAGCCACTG<br>ATCTATGATTACTTTAAACAACTCTTTGCTCAAGTGACAAATC<br>CACCTATCGATCCATTTCGTGAAAGAATAGTGATGTCATTGG<br>GTTGTCCTATTGGTCCAGCAGCTAACATCTTAGAACCTAGTG<br>CTCAACAGTGCTGTCGGCTGTATTTGGAACAGCCTATATTATC<br>TCTCAAAGACATAGAAGCTTTGAAAACAACTACATATAAAG<br>GATGGAAAACTAAAGTGATTGACATGGTTTATCCTGTAAATG<br>AAGCAGCAGATGGCCTTTTGCCTGCCTTAAAAAGAATCTGTG<br>ATGAAGCTTGCAGTGCTGCTGAAAACGGATATTCTTTGGTTA<br>TACTTTCAGACAGAAAAGCTGGAAGAGATTATATTCCTGTTA<br>GTGCATTACTAGCTTTAGGTGCCACTCATCATCATTTGATTAA<br>TAATTTACAAAGAATGAAAGTTGGTCTCATATTAGAAACTGG<br>AGAAGCAAGAGAAGTACATCACATGTGTGTATTATTGGGAT<br>ATGGAGCTGATGCCATATGCCCATACCTTGTGTTTGAAAGCA<br>TGGCTGAATTACAAGGAGAAGGTGTCATTAGCGAGACTCTG

						GGVTFRVLAEENLE<br>RYRIAYSDRDCDNIV<br>LRNPGNYHWRSGG<br>EKHINDPLAIANLQ<br>DAARSKSQSAYANF<br>VETTMQNVRACTLR<br>GQFDLVKVPKPVDIS<br>QVEEAKEIVKRFATG<br>AMSFGSISLEAHKTL<br>AAAMNRVGGKSNT<br>GEGGEDPDRFIVTDS<br>NNNFRSAIKQVASG<br>RFGVTIEYLANADEL<br>QIKMAQGAKPGEG<br>GELPGYKVSKDIAKT<br>RHSIPGVGLISPPPH<br>HDIYSIEDLAELIYDL<br>KCANPTARISVKLVS<br>EVGVGVVASGVAKG<br>KAEHITISGHDGGTG<br>ASSWTGIKGAGLPW<br>ELGIAETHQTLVQN<br>DLRSRVVLQADGQI<br>RTGFDVVVAAILGA<br>DEFGFSTAPLIVLGC<br>TMMRKCHLNTCPV<br>GIATQDPELRKKFEG<br>KPEHVVNYLFMLAE	ACAGAGGACATGATTTTTAAAAATTATGTTGAAGCGATGGAG<br>CGAGGAATTGCCAAGGTAATGGCAAAAATGGGAATTTCTAC<br>TCTACAAAGTTATAAAGGGGCTCAAATTTTTGAAGCAGTTGG<br>TCTGAGTGAAGAAGTTGTGGATTTCTGTTTTAAAAATACACC<br>CTCTCGCATTGGTGGTGTGACATTTAGAGTTTTAGCTGAAGA<br>AAATCTTGAACGCTACCGTATTGCCTATAGTGATAGAGACTG<br>TGACAATATTGTTTTGAGAAATCCTGGAAATTACCACTGGAG<br>AAGTGGAGGAGAGAAACATATTAATGATCCTTTAGCTATAG<br>CAAACTTACAGGATGCTGCCCGATCAAAAAGTCAAAGTGCC<br>TATGCCAATTTTGTGGAAACTACCATGCAGAATGTTAGAGCT<br>TGCACTTTAAGAGGTCAGTTTGACCTTGTAAAAGTACCTAAA<br>CCTGTAGATATTAGCCAAGTGGAAGAGGCTAAAGAAATAGT<br>GAAGCGCTTTGCTACTGGTGCCATGAGTTTTGGTAGTATATCA<br>TTAGAAGCTCATAAAACTCTTGCTGCTGCAATGAATAGAGTT<br>GGAGGCAAATCTAATACTGGAGAAGGTGGTGAAGATCCTGA<br>CAGATTCATTGTTACAGACTCAAATAACAATTTTCGCTCAGC<br>TATCAAACAGGTTGCATCTGGCAGATTTGGGGTTACCATCGA<br>ATATCTTGCAAATGCTGATGAACTTCAGATTAAGATGGCACA<br>AGGAGCCAAACCTGGGGAAGGTGGTGAACTTCCTGGTTATA<br>AAGTCTCAAAAGACATTGCTAAAACTAGACATTCTATTCCTG<br>GAGTGGGACTTATAAGTCCCCCTCCCCATCATGATATCTATTC<br>TATTGAAGATTTAGCTGAGCTCATCTATGATTTAAAATGTGC<br>AAACCCTACAGCTCGAATTTCTGTGAAACTTGTGTCAGAAGT<br>TGGAGTAGGAGTTGTTGCATCTGGTGTTGCCAAAGGAAAAGC<br>AGAACATATCACCATATCAGGACATGATGGAGGAACAGGGG<br>CCAGCAGTTGGACAGGAATAAAAGGAGCTGGTCTCCCTTGG<br>GAACTTGGAATTGCTGAGACTCATCAAACACTTGTACAAAAT<br>GATTTACGCTCGCGGGTTGTTTTGCAAGCTGATGGACAGATT

						DVRSHMASLGIKRF	CGAACAGGATTCGACGTAGTGGTTGCTGCCATTCTTGGAGCC
						QDLIGRTDLLKVYH	GATGAATTTGGCTTTTCAACTGCACCTTTGATAGTCCTCGGAT
						NKDNPKAQMLDYS	GTACAATGATGAGGAAGTGCCATTTGAACACCTGCCCTGTCG
						AILMDALSLRPGTLI	GCATTGCCACTCAGGATCCAGAGCTCAGGAAGAAATTCGAA
						VGGSLKQDFALENRI	GGAAAGCCAGAACATGTTGTGAATTACCTGTTCATGCTTGCT
						DNLLIKQAADVIDR	GAAGATGTACGTTCTCATATGGCGTCATTGGGTATAAAGAGA
						RKKDVTIHMDISNV	TTCCAAGACTTGATTGGACGCACAGATTTGCTGAAAGTTTAC
						DRAFGSTLSYHIAKK	CATAATAAAGACAATCCGAAAGCTCAGATGCTTGATTATTCT
						HGSSGLPNNSIKIKL	GCCATACTCATGGATGCCTTGTCTTTGAGGCCTGGAACCTTA
						EGSAGQSFCAFLAP	ATTGTTGGTGGATCTCTTAAGCAAGACTTTGCTTTAGAAAAC
						GVTVELEGDANDYV	AGAATTGACAATCTGCTTATTAAACAAGCTGCTGATGTTATA
						GKGLSGGTVIVYPPT	GACAGGCGTAAAAAAGATGTGACCATTCATATGGACATCTC
						RLPQDFKSEENIIVG	CAATGTTGATAGAGCATTTGGATCAACACTAAGTTATCACAT
						NVCLYGATDGKAFF	AGCGAAAAAACATGGTTCAAGTGGCTTACCTAATAATAGCA
						RGIAAERFCVRNSG	TCAAAATAAAACTAGAAGGATCAGCTGGTCAAAGTTTTTGTG
						ATAVIEGVGDHGCE	CATTTTTAGCTCCTGGAGTAACAGTTGAGCTAGAAGGTGATG
						YMTGGYCLILGPTG	CTAATGACTATGTAGGAAAGGGATTATCTGGTGGAACTGTAA
						RNFAAGMSGGIAYV	TTGTATATCCCCCTACGCGTTTACCTCAGGATTTCAAATCTGA
						LDENHKLESNSNLM	AGAAAATATTATTGTAGGCAATGTTTGCTTGTATGGTGCTAC
						MVDLLPLELPEDLSF	AGATGGAAAAGCCTTCTTCAGAGGGATTGCTGCTGAACGTTT
						VESLLREFFEKTGSEI	CTGTGTGAGGAACTCTGGTGCTACTGCTGTTATTGAGGGTGTT
						AQRILSNWPASAKSF	GGTGACCATGGTTGTGAGTACATGACTGGTGGCTACTGTCTG
						VKVFPKEYQRALRQ	ATTCTTGGTCCAACTGGACGTAATTTTGCTGCAGGCATGTCTG
						AEEEKKQALTNGPT	GTGGTATTGCTTATGTCTTGGATGAGAATCATAAACTTGAATC
						PAVETKPAVKDIED	TAATAGTAATTTGATGATGGTTGACTTATTGCCACTTGAATTG
						VIPKLDKIRGFMKYK	CCTGAAGACCTTAGTTTTGTAGAGAGCTTATTGAGAGAATTTT
						RMTTPYREPADRLH	TTGAAAAAACAGGTTCCGAAATTGCTCAGCGTATACTCTCTA
						DWDEIYDFKGVRKTI	ACTGGCCAGCGTCTGCAAAATCTTTTGTTAAGGTGTTCCCTAA

						KVQAARCMDCGVPFCQSSHGCPLGNIIPKWNDLVFKDDWREALNQLLQTNNFPEFTGRVCPAPCEGACVLGINEPPVNIKHIECAIIDNAFDKGWMIPQIPAVRTGKKIAIIGSGPSGLAAAAQLNKAGHTIHVYERNDRVGGLLEYGIPTMKLGKDVVKRRVDLLAAEGITFHTNVNVGKDISGKSLLEETDALLLTVGATWPRDLPIPGRDLKGIHFAMNFLETWQKHQLGNKIDYSKVNAKDKNVVVIGGGDTGCDCIATSLRQGAKSIVTFEILPEPPEKRADNNPWPQWPKVFRVDYGHDEVKVKWGDDPRRFCVLSEKFLDNGNGDVSGISTIKVEWKKDASGNWAMEKIPGSEKVFKADLVLIAMGFLSPEQYLLNDVGVQQDPRKNV	AGAGTACCAACGTGCCTTACGCCAAGCTGAGGAAGAAAAGAAACAAGCTTTAACAAATGGGCCAACTCCTGCTGTTGAAACTAAACCTGCTGTCAAAGATATTGAAGACGTTATTCCTAAATTGGATAAAATAAGAGGTTTTATGAAATACAAACGCATGACTACTCCATACCGTGAACCAGCTGATCGACTTCATGACTGGGATGAAATCTATGACTTTAAGGGTGTGCGTAAAACTATAAAAGTACAAGCAGCCAGGTGTATGGATTGTGGTGTTCCATTCTGTCAGTCAAGTCATGGCTGCCCTCTTGGAAATATCATACCTAAATGGAATGATCTTGTTTTCAAGGATGACTGGAGAGAAGCTCTTAATCAATTACTGCAGACAAATAATTTTCCAGAATTCACTGGTCGAGTTTGTCCAGCCCCTTGTGAGGGAGCTTGTGTTCTGGGTATCAATGAACCCCCAGTGAATATAAAGCATATTGAGTGTGCCATCATTGACAATGCCTTTGATAAAGGGTGGATGATTCCTCAAATTCCAGCTGTAAGAACTGGTAAGAAAATTGCTATCATTGGGAGTGGACCGTCTGGTCTTGCAGCTGCTGCACAACTAAACAAGGCTGGGCATACAATTCATGTATATGAGAGAAATGACAGGGTTGGTGGTTTGTTGGAATATGGAATTCCAACTATGAAGCTCGGTAAAGATGTTGTAAAAAGACGGGTAGACCTTCTTGCAGCCGAAGGTATTACTTTCCATACAAATGTAAATGTAGGCAAAGACATTTCTGGAAAATCTCTTCTTGAAGAGACTGATGCTCTGCTATTAACAGTTGGAGCTACTTGGCCAAGAGACCTTCCAATACCAGGAAGAGATTTGAAAGGAATTCACTTTGCAATGAATTTCCTGGAGACCTGGCAAAAGCACCAGTTGGGTAACAAAATTGACTATTCCAAGGTGAACGCCAAAGATAAAAATGTGGTTGTGATTGGCGGTGGTGACACTGGTTGTGACTGCATAGCTACTTCTCTCAGACAGGGTGCCAAAAGTATTGTGACATTTGAAATTCTTCCTGAACCTCCAGAAAAGAGAGCTGACAATAACCCATGGCCTCAATGGCCAAAAGTGTTCAGAGTAGATTATGGCCATGACGAGGTGAAAGTGAAA

						ATPNGNYRTSIPKVY<br>AAGDCRRGQSLVV<br>HAISEGRQAARQID<br>ADLMQCSSLAGPGG<br>VIPYVNELSVAA*	TGGGGGGATGATCCAAGAAGATTCTGTGTTCTCAGTGAGAAA<br>TTCTTGGATAATGGTAACGGCGATGTGTCAGGTATTAGTACC<br>ATAAAAGTTGAATGGAAGAAGGATGCTTCAGGAAATTGGGC<br>GATGGAAAAAATTCCTGGCTCAGAAAAAGTATTCAAGGCGG<br>ATTTAGTTTTAATTGCTATGGGATTCCTTAGCCCTGAACAGTA<br>TCTTTTGAATGATGTTGGAGTACAACAAGATCCAAGAAAAA<br>ATGTTGCCACTCCCAACGGAAACTATCGCACATCTATTCCCA<br>AAGTTTATGCTGCAGGAGATTGCCGCAGAGGCCAGTCATTAG<br>TTGTACATGCGATTTCTGAAGGACGGCAGGCCGCCCGTCAGA<br>TTGATGCTGATCTTATGCAATGTTCCTCATTGGCTGGTCCTGG<br>AGGTGTCATACCTTATGTCAATGAACTCTCTGTGGCTGCTTAA<br>ATATGTTTCTCTGTAGTTTCATGAATCAAGAATGTACACTACA<br>GCTGGTAGTTATGCTTATAAAACACAAGACTAATGAAATTGG<br>CAGCAAATAATCATTAGTTTCTCATAGGTGGAACGATGTACC<br>AGAATGGGACGAGTGTGTTCATCAGTGAGTGTTCATAGTTGT<br>TGTATATTTAAGGATTTGGCCTGTGTAATATTTTTAATTTTATG<br>AGGAATGCATTGTTCGTTTATTACATCCGATGTGTTGGCTTTT<br>TGTAAGTGAGTCTAAGTTGTTGGTGTGTATATATATCTCAGAT<br>GTTTGCTGTCCAGTTTGTTTTTTAAACATTGTGTAGCACTTGAC<br>TTCAATCATTAAACTCTACCTTGATGCCATTTAATTCTTGTAT<br>AGTTATCATATTGCTTGTAGTGTATTGCTGGAATTTCATGCAT<br>TCGATAAACATTTTAATTCACTTGTTTGCAGCATCTTCTATCC<br>AGAAATATAAAACTTGGCATGCACACATAGATATACTTAAA<br>TCTATTGAAGTTTAAATAAAGGTTGTTTTACTGTAGATTCTAC<br>CAATTAGCCTAAATTGCCATTGACTGTGCATGTTTGAGAAAG<br>TTGCCGTTTTGAAGTCTTCCATAAAAGAGGTGCTGACTGTTAC<br>GTTGGTTCCAGCTAATACTTTTTTCTTCGCAGTAATAAATACA<br>GCAAAACAAACTTTTATGCTTTTAATTTAGGAATATAGGGTT

							CTTGATAGGGTCTGACTTTCACCCAAAAATGACCTTAAAATA<br>AAAAATTAAAAAAACAAAACAAAAAAAAGTGCGGTTTAGA<br>AATTTCTGAATTGTTTTTACTGAATTAATTCTGAAACATTAGA<br>AACTTAGGTTTAGGTGATTTAAACAGCATACCATATCAGATA<br>TCAATGCCTGTTTCTCTGAAAATTGCATCTTTTTAAAATATAG<br>ACACACGGAGAGCCCTATGGTAAATTAATGTATCTATCTTAC<br>TTTTAAAAGAACAATGTGTGAAGTATTTTATCAGACTACTAT<br>AGAGAACAATCACACCTAGCCATTTATATTAAATATATTTCT<br>TTAGTATTCATAATAGAGAGATAATAGTTTGTGTATAAGTGA<br>GTATGTTGTGCCTTGTTGAAAACAATAGTTTGCAAATGTATCT<br>GTTTGCCTGGTATTGTAAATGGTAATTAAGATATTTTAGATGT<br>ATTTTCAGATTATAGCACCTCTTTTTAATAGCTTATAAATACC<br>ATTCTGCTCTATAATACTGATTTTGATCTAAGGTGCCAACCAT<br>TTCACAAATAACTGAATTTTAGTGAAATATGCAAGTATAATA<br>TTAAAGTAAATTGTATAAGCATAAAATGCTGTATAGGTGTAG<br>CTAGAGTTAAAAGAGGAAGACGAGTGAAATTAAACAGTATG<br>ATAGATGTGTTGTTCTTTTCCTTGACACTGCAGGTAGAATTCG<br>ATACTACATCACTTATTGCACAATCACAATATAAACTTATATT<br>AAAAAGTTGCAAAGAGAGGTTATATCTCATAATTTTGTTTTA<br>CATGAAATAAAGGTTGGCACCTTATTTTTTTACTATCTTTTTTG<br>CCTTATCTGTCAATACATTTGCAATTATTTTTATCCAGTGTTGA<br>CTTTATTGTTCTATTTTACTATTTCAAGCTTTCGTGATGTACCA<br>CGATGTGTAAGCATATCCCTGTAAACATTCCATAATAGTACT<br>GTAATAAAGGAATTGGGTTTATTACCCCATATACATTTAAAC<br>TGGCCTATTGAAGTTTTCAAAGTTAAAATTAGATCAATCCTTA<br>AATATACTGCACCAGAAAATAGAGCTTTAAGATTTTATTCTT<br>ACACCGAGTAATC

PhdEnzGlS04	P81383	4,54E-05	54,054	PF01593	99443	LVFVSFFNFFLQDPE<br>LRKKFEGKPEHVVN<br>YLFMLAEDVRSHMA<br>SLGIKRFQDLIGRTD<br>LLKVYHNKDNPKA<br>QMLDYSAILMDALS<br>LRPGTLIVGGSLKQD<br>FALENRIDNLLIKQA<br>ADVIDRRKKDVTIH<br>MDISNVDRAFGSTLS<br>YHIAKKHGSSGLPN<br>NSIKIKLEGSAGQSF<br>CAFLAPGVTVELEG<br>DANDYVGKGLSGGT<br>VIVYPPTRLPQDFKS<br>EENIIVGNVCLYGAT<br>DGKAFFRGIAAERFC<br>VRNSGATAVIEGVG<br>DHGCEYMTGGYCLI<br>LGPTGRNFAAGMSG<br>GIAYVLDENHKLES<br>NSNLMMVDLLPLEL<br>PEDLSFVESLLREFFE<br>KTGSEIAQRILSNWP<br>ASAKSFVKVFPKEYQ<br>RALRQAEEEKKQAL<br>TNGPTPAVETKPAV<br>KDIEDVIPKLDKIRGF	CTTAAATGATTAGTCTTAAACCCTCTATATAAAATTTTAAAA<br>AATATACAAATTATGTAATTAATTCCCTTAATAAAGTGACTT<br>GAAAATACTCTATTGCCTCCTTAAGGGTTAATATGCTTATTTA<br>AGTGGGTGATGATTTCTTTTTATCTTCTTTATTCTTTCCTGTCA<br>GTCTTTCTGAGCACTTAAACCTCATTTCTATAAAACCTGATTT<br>ATGGCAAAAATTTAGCAAAATATTTTTGCAGATCTATTGTAA<br>ATGTAACAGTTTTAAAATTCTAGTGAATAGAAAGTGCAATAC<br>ACAAGTGATATTCATAAATGATTCAATCGCCCCTGCCAATGA<br>CTTGTTGCCCTGCTGTCTTCACTTTTTGTAAGTATCTAGAGTA<br>AGTTTTGACACCACATCCAGATGAATCTGCCTCTAACTCATA<br>CTGCATATTTTAGTCTTTCTCCCGAGGACAAAAGTCATCCAA<br>GAGAAGTGATTATCTTTTCCCATTTTCTTCTCTTCCTGCGCAG<br>GATTCGACGTAGTGGTTGCTGCCATTCTTGGAGCCGATGAAT<br>TTGGCTTTTCAACTGCACCTTTGATAGTCCTCGGATGTACAAT<br>GATGAGGAAGTGCCATTTGAACACCTGCCCTGTCGGCATTGC<br>CACTCAGGTGAGGAATTACTGCAAGAGAAGCGCAATCCATT<br>GAAGCAACCACATGGAATGCACTGTTTCCAGTTGGACGTCTC<br>GTGTAGCTGCTTTTAAACTGCTCTATTTTTCTTTTCAGTTTTTTG<br>CTATTTGTTCTTTTACTTAGCCTATTTAGCTTCCCGAGAGTATG<br>GCCTATCTGCTGGTCAACCAGTCTACTTGTAGAGAAGTAGTA<br>ATGGAGTGTGGTATCCATTATTCATTAATGATTTTGATTAGTT<br>TTTGTTAGTTTTTTTAACTTTTTTTTACAGGATCCAGAGCTCAG<br>GAAGAAATTCGAAGGAAAGCCAGAACATGTTGTGAATTACC<br>TGTTCATGCTTGCTGAAGATGTACGTTCTCATATGGCGTCATT<br>GGGTATAAAGAGATTCCAAGACTTGATTGGACGCACAGATTT<br>GCTGAAAGTTTACCATAATAAAGACAATCCGAAAGCTCAGA<br>TGCTTGATTATTCTGCCATACTCATGGATGCCTTGTCTTTGAG<br>GCCTGGAACCTTAATTGTTGGTGGATCTCTTAAGCAAGACTTT

						MKYKRMTTPYREPA<br>DRLHDWDEIYDFKG<br>VRKTIKVQAARCMD<br>CGVPFCQSSHGCPL<br>GNIIPKWNDLVFKD<br>DWREALNQLLQTN<br>NFPEFTGRVCPAPCE<br>GACVLGINEPPVNIK<br>HIECAIIDNAFDKG<br>WMIPQIPAVRTGKKI<br>AIIGSGPSGLAAAAQ<br>LNKAGHTIHVYERN<br>DRVGGLLEYGIPTM<br>KLGKDVVKRRVDLL<br>AAEGITFHTNVNVG<br>KDISGKSLLEETDAL<br>LLTVGATWPRDLPIP<br>GRDLKGIHFAMNFL<br>ETWQKHQLGNKIDY<br>SKVNAKDKNVVVIG<br>GGDTGCDCIATSLR<br>QGAKSIVTFEILPEPP<br>EKRADNNPWPQWP<br>KVFRVDYGHDEVKV<br>KWGDDPRRFCVLSE<br>KFLDNGNGDVSGIS<br>TIKVEWKKDASGN<br>WAMEKIPGSEKVFK	GCTTTAGAAAACAGAATTGACAATCTGCTTATTAAACAAGCT<br>GCTGATGTTATAGACAGGCGTAAAAAAGATGTGACCATTCAT<br>ATGGACATCTCCAATGTTGATAGAGCATTTGGATCAACACTA<br>AGTTATCACATAGCGAAAAAACATGGTTCAAGTGGCTTACCT<br>AATAATAGCATCAAAATAAAACTAGAAGGATCAGCTGGTCA<br>AAGTTTTTGTGCATTTTTAGCTCCTGGAGTAACAGTTGAGCTA<br>GAAGGTGATGCTAATGACTATGTAGGAAAGGGATTATCTGGT<br>GGAACTGTAATTGTATATCCCCCTACGCGTTTACCTCAGGATT<br>TCAAATCTGAAGAAAATATTATTGTAGGCAATGTTTGCTTGT<br>ATGGTGCTACAGATGGAAAAGCCTTCTTCAGAGGGATTGCTG<br>CTGAACGTTTCTGTGTGAGGAACTCTGGTGCTACTGCTGTTAT<br>TGAGGGTGTTGGTGACCATGGTTGTGAGTACATGACTGGTGG<br>CTACTGTCTGATTCTTGGTCCAACTGGACGTAATTTTGCTGCA<br>GGCATGTCTGGTGGTATTGCTTATGTCTTGGATGAGAATCATA<br>AACTTGAATCTAATAGTAATTTGATGATGGTTGACTTATTGCC<br>ACTTGAATTGCCTGAAGACCTTAGTTTTGTAGAGAGCTTATTG<br>AGAGAATTTTTTGAAAAAACAGGTTCCGAAATTGCTCAGCGT<br>ATACTCTCTAACTGGCCAGCGTCTGCAAAATCTTTTGTTAAGG<br>TGTTCCCTAAAGAGTACCAACGTGCCTTACGCCAAGCTGAGG<br>AAGAAAAGAAACAAGCTTTAACAAATGGGCCAACTCCTGCT<br>GTTGAAACTAAACCTGCTGTCAAAGATATTGAAGACGTTATT<br>CCTAAATTGGATAAAATAAGAGGTTTTATGAAATACAAACG<br>CATGACTACTCCATACCGTGAACCAGCTGATCGACTTCATGA<br>CTGGGATGAAATCTATGACTTTAAGGGTGTGCGTAAAACTAT<br>AAAAGTACAAGCAGCCAGGTGTATGGATTGTGGTGTTCCATT<br>CTGTCAGTCAAGTCATGGCTGCCCTCTTGGAAATATCATACC<br>TAAATGGAATGATCTTGTTTTCAAGGATGACTGGAGAGAAGC<br>TCTTAATCAATTACTGCAGACAAATAATTTTCCAGAATTCACT

						ADLVLIAMGFLSPEQ<br>YLLNDVGVQQDPRK<br>NVATPNGNYRTSIP<br>KVYAAGDCRRGQSL<br>VVHAISEGRQAARQ<br>IDADLMQCSSLAGP<br>GGVIPYVNELSVAA*	GGTCGAGTTTGTCCAGCCCCTTGTGAGGGAGCTTGTGTTCTGG<br>GTATCAATGAACCCCCAGTGAATATAAAGCATATTGAGTGTG<br>CCATCATTGACAATGCCTTTGATAAAGGGTGGATGATTCCTC<br>AAATTCCAGCTGTAAGAACTGGTAAGAAAATTGCTATCATTG<br>GGAGTGGACCGTCTGGTCTTGCAGCTGCTGCACAACTAAACA<br>AGGCTGGGCATACAATTCATGTATATGAGAGAAATGACAGG<br>GTTGGTGGTTTGTTGGAATATGGAATTCCAACTATGAAGCTC<br>GGTAAAGATGTTGTAAAAAGACGGGTAGACCTTCTTGCAGC<br>CGAAGGTATTACTTTCCATACAAATGTAAATGTAGGCAAAGA<br>CATTTCTGGAAAATCTCTTCTTGAAGAGACTGATGCTCTGCTA<br>TTAACAGTTGGAGCTACTTGGCCAAGAGACCTTCCAATACCA<br>GGAAGAGATTTGAAAGGAATTCACTTTGCAATGAATTTCCTG<br>GAGACCTGGCAAAAGCACCAGTTGGGTAACAAAATTGACTA<br>TTCCAAGGTGAACGCCAAAGATAAAAATGTGGTTGTGATTGG<br>CGGTGGTGACACTGGTTGTGACTGCATAGCTACTTCTCTCAG<br>ACAGGGTGCCAAAAGTATTGTGACATTTGAAATTCTTCCTGA<br>ACCTCCAGAAAAGAGAGCTGACAATAACCCATGGCCTCAAT<br>GGCCAAAAGTGTTCAGAGTAGATTATGGCCATGACGAGGTG<br>AAAGTGAAATGGGGGGATGATCCAAGAAGATTCTGTGTTCTC<br>AGTGAGAAATTCTTGGATAATGGTAACGGCGATGTGTCAGGT<br>ATTAGTACCATAAAAGTTGAATGGAAGAAGGATGCTTCAGG<br>AAATTGGGCGATGGAAAAAATTCCTGGCTCAGAAAAAGTAT<br>TCAAGGCGGATTTAGTTTTAATTGCTATGGGATTCCTTAGCCC<br>TGAACAGTATCTTTTGAATGATGTTGGAGTACAACAAGATCC<br>AAGAAAAAATGTTGCCACTCCCAACGGAAACTATCGCACAT<br>CTATTCCCAAAGTTTATGCTGCAGGAGATTGCCGCAGAGGCC<br>AGTCATTAGTTGTACATGCGATTTCTGAAGGACGGCAGGCCG<br>CCCGTCAGATTGATGCTGATCTTATGCAATGTTCCTCATTGGC

							TGGTCCTGGAGGTGTCATACCTTATGTCAATGAACTCTCTGTG<br>GCTGCTTAAATATGTTTCTCTGTAGTTTCATGAATCAAGAATG<br>TACACTACAGCTGGTAGTTATGCTTATAAAACACAAGACTAA<br>TGAAATTGGCAGCAAATAATCATTAGTTTCTCATAGGTGGAA<br>CGATGTACCAGAATGGGACGAGTGTGTTCATCAGTGAGTGTT<br>CATAGTTGTTGTATATTTAAGGATTTGGCCTGTGTAATATTTTT<br>AATTTTATGAGGAATGCATTGTTCGTTTATTACATCCGATGTG<br>TTGGCTTTTTGTAAGTGAGTCTAAGTTGTTGGTGTGTATATAT<br>ATCTCAGATGTTTGCTGTCCAGTTTGTTTTTTAAACATTGTGTA<br>GCACTTGACTTCAATCATTAAACTCTACCTTGATGCCATTTAA<br>TTCTTGTATAGTTATCATATTGCTTGTAGTGTATTGCTGGAATT<br>TCATGCATTCGATAAACATTTTAATTCACTTGTTTGCAGCATC<br>TTCTATCCAGAAATATAAAACTTGGCATGCACACATAGATAT<br>ACTTAAATCTATTGAAGTTTAAATAAAGGTTGTTTTACTGTAG<br>ATTCTACCAATTAGCCTAAATTGCCATTGACTGTGCATGTTTG<br>AGAAAGTTGCCGTTTTGAAGTCTTCCATAAAAGAGGTGCTGA<br>CTGTTACGTTGGTTCCAGCTAATACTTTTTTCTTCGCAGTAAT<br>AAATACAGCAAAACAAACTTTTATGCTTTTAATTTAGGAATA<br>TAGGGTTCTTGATAGGGTCTGACTTTCACCCAAAAATGACCT<br>TAAAATAAAAAATTAAAAAAACAAAACAAAAAAAAGTGCG<br>GTTTAGAAATTTCTGAATTGTTTTTACTGAATTAATTCTGAAA<br>CATTAGAAACTTAGGTTTAGGTGATTTAAACAGCATACCATA<br>TCAGATATCAATGCCTGTTTCTCTGAAAATTGCATCTTTTTAA<br>AATATAGACACACGGAGAGCCCTATGGTAAATTAATGTATCT<br>ATCTTACTTTTAAAAGAACAATGTGTGAAGTATTTTATCAGA<br>CTACTATAGAGAACAATCACACCTAGCCATTTATATTAAATA<br>TATTTCTTTAGTATTCATAATAGAGAGATAATAGTTTGTGTAT<br>AAGTGAGTATGTTGTGCCTTGTTGAAAACAATAGTTTGCAAA

							TGTATCTGTTTGCCTGGTATTGTAAATGGTAATTAAGATATTTTAGATGTATTTTCAGATTATAGCACCTCTTTTTAATAGCTTATAAATACCATTCTGCTCTATAATACTGATTTTGATCTAAGGTGCCAACCATTTCACAAATAACTGAATTTTAGTGAAATATGCAAGTATAA

Phosphodiesterases							
**Transcript ID**	**Uniprot ID reference protein**	**e-value**	**Identity (%)**	**PFAM domain**	**MW (Da)**	**Amino acid sequence**	**Nucleotide sequence**
						MATGLLLLFFACLLF	TTTTTTTTTTTGTTCATAAGTATTTAATGATATTTGGAAGTGA
						VHEISA**GKKLIVVLI**	AATAAAACAGTACAAGGGCAGAATATAACGAAATAATGAA
						**DGFKWDYINDPKL**	CAGACTGTGTACTGAAGTGTTCATAGAATTAGAAGGCACATA
						**ELKGIPKIIENGVRA**	AGATTCATCTTCGTCAGCAAGAATATCTTTCAGTATTTCCGTT
						**SYVNPIYPSISYPNW**	GATCCATTATTCGGCATAGGCTGGATGTCCAGAACAGAGCAC
						**YTIVTGLYPESHDM**	ATTATATTATAGAGGTCAATGTTACTCATTGGTTTCGACGTAT
						**VNNVMYDNATEEA**	ATCCTCTGCGAAATCCTGGACCACTTGCAAAGAATATTGTCC
				PF01223		**FLMLPVKGSTDPH**	TCATATCAGTTACTTCATTAGGATCAAATCCATGATGTCCATT
						**WWNGAEPIWVTAE**	GTACTGACCTGGATATTCTCCTAGTCTTTTAGTTGAATCATTC
						**KQGKNAAMYWWA**	AGGGCTTTTATGAAATATCCTTTTTCAGCTTTTATGAAAATGT
				PF01663	52398.11	**GCEVEIKGIQPMIC**	CATTCATTAAAGGACTTTTCTTCAAGTGAAAAGATATAGGAA
PhdEnzPhd 01	J3SBP3	3,40E-49	28,723			**ERHRYGPGTEEEKT**	TTTCTTCTCTTTTATATACTTGTATTCCACTTGTTTTAGCATCTT
					**50106.28**	**DYLERIDDIIEMFKP**	TCAAGTCATTATATAGCTTATCCAGCATACCGTCTTCTGGTAC
						**SRTVSTDRLSLVLM**	CAGCATCATTGTAGCTCCATAAGAAACTTGATATTTAATACT
						**YYGPLDYTGHYSGP**	AGGAGCAAATTTTTCCAAACTGATGTACTTCGTAGTTTCCGG
				PF01033		**GTSETRRALQDIDE**	ACGCACATCAGTCATTCCATGGTCGGATACAATCATAATATT
						**VLYKMLRKIEKAGL**	GACCTCTCTTTGCAATCCGGCCTTTTCAATCTTCCGAAGCATT
						**QREVNIMIVSDHG**	TTGTATAAGACTTCATCTATATCTTGCAAAGCTCTTCGCGTCT
						**MTDVRPETTKYISL**	CTGATGTTCCGGGCCCCGAATAATGACCAGTGTAATCTAAGG
						**EKFAPSIKYQVSYG**	GGCCATAATACATTAAGACTAAAGATAAACGATCCGTCGAA
						**ATMMLVPEDGML**	ACGGTACGCGATGGCTTGAACATTTCGATGATATCATCAATT
						**DKLYNDLKDAKTS**	CTTTCCAAGTAATCGGTTTCTCTTCCTCAGTTCCCGGGCCAT
						**GIQVYKREEIPISFH**	AACGATGCCTTTCACAAATCATAGGCTGAATACCCTTAATTT

						**LKKSPLMNDIFIKA**<br>**EKGYFIKALNDSTK**<br>**RLGEYPGQYNGHH**<br>**GFDPNEVTDMRTIF**<br>**FASGPGFRRGYTSK**<br>**PMSNIDLYNIMCSV**<br>**LDIQPMPNNGSTEI**<br>**LKDILADEDESYVP**<br>**SNSMNTSVHSLFIIS**<br>**LYSALVLFYFTSKYH**<br>*****	CAACTTCACAGCCGGCCCACCAATACATGGCGGCATTTTTGC<br>CCTGCTTTTCGGCTGTTACCCAGATAGGTTCTGCTCCGTTCCA<br>CCAGTGAGGATCGGTACTCCCTTTTACAGGCAACATGAGGAA<br>GGCTTCTTCAGTTGCGTTATCGTACATCACGTTATTAACCATG<br>TCATGACTCTCTGGGTAAAGACCTGTGACAATAGTGTACCAG<br>TTCGGGTATGATATTGAAGGATAAATTGGATTAACGTACGAA<br>GCTCTGACTCCATTTTCGATAATTTTCGGAATACCCTTTAGCT<br>CGAGTTTTGGATCATTGATATAATCCCACTTGAATCCATCAAT<br>TAAAACAACAATCAGTTTCTTACCAGCTGATATTTCGTGTAC<br>GAAAAGTAAACAGGCGAAAAATAGAAGCAATAGCCCGGTA<br>GCCATCGGAGAGATGCGATCGGGATACCAGTTTCTCTCAGAA<br>TATGTACACGCGCTTCTTTTCTGGTCAGAGACGCAATGTTGTT<br>TTTAAGAAGCGAGTTTCCTCTGTGTTTGAAACTAGACAGGTG<br>GCGACGCGGAAAGGTAATAGAGGATGAAAAGAAACAAATG<br>CTCAGGAAACTGCTCTCGTAATAGTGAACGAAAGAGA

PhdEnzPhd 02	J3SBP3	7,84E-138	37,428	PF01223<br>PF01663<br>PF01033	87248,44	PRGECFEELNLAVM<br>ELVERIQSFKRNFSSS<br>EDPNLPWYRQRRNI<br>MFLVIGLLVVFGVV<br>VLFFGGYFAGLAAN<br>KEPDDSAPESKPRVV<br>ALPPVEWEGICPKRE<br>DKCPSSFQGIPPLILV<br>SLDGFRPDFMDRGL<br>TPTIKNIASCGTHAP<br>YMFPVFPTKTFPNHF<br>SQVTGVFPTHHGIID<br>NTMYDPIKKRLFKV<br>GQPESLDRSWWLKE<br>PIWVTAQKQGKVTG<br>CFYWPGSEVDINHT<br>HPTYYKKYNSKVSFE<br>SRVDQVLAWLDLPV<br>ASRPHFITLYVNEPD<br>HALHYFGTRSKQAS<br>DALAKVDRMIERLIS<br>GLQQRSLINCVNVVI<br>MSDHGMSDTDCKR<br>VIDIQNYINVNNTFS<br>TVGPFGRIRPKGKRN<br>ATVVNEMITKLRCK<br>SPHMRAYPKEQLPV<br>RMHYAESPRIEPIFL	CAGAAACATCTCGCTTGGTCGCTAAGTTACAATGCCGGCTAT<br>TTTTCACGCTATTATTCTGAGCAGCTTCTACATTTAGGTGTTTA<br>GTACTGATATGGATGCTCAATTATCGACAAATCTTAAATGTA<br>AAATATTCCCATCTTTCGGTGAAATAAGAACTTGATATCTGC<br>ATCGAAAGAGACTGATCACGGTGTGTTGATCGTCCGGTTTAC<br>GCAGCTAACCTCGTGGCGAGTGTTTTGAAGAATTAAATCTTG<br>CAGTCATGGAGCTAGTGGAAAGGATTCAGAGCTTTAAAAGG<br>AATTTCAGTTCGTCGGAAGATCCAAATCTACCATGGTACAGA<br>CAAAGAAGAAACATTATGTTTCTGGTTATAGGTCTCTTGGTTG<br>TGTTTGGCGTTGTCGTTCTGTTTTTTGGAGGTTATTTTGCTGGT<br>TTGGCTGCGAATAAAGAACCTGATGATTCTGCACCTGAATCA<br>AAGCCAAGAGTTGTTGCACTTCCACCTGTAGAATGGGAAGGT<br>ATCTGTCCAAAGAGAGAGGATAAATGTCCATCAAGTTTTCAA<br>GGAATCCCCCCCTTGATACTGGTCTCCCTAGATGGATTTCGTC<br>CTGACTTCATGGACAGGGGCCTCACTCCTACCATAAAAAAC<br>ATTGCATCATGTGGAACACATGCTCCTTATATGTTTCCAGTAT<br>TTCCAACCAAAACATTTCCAAATCATTTCAGTCAAGTTACGG<br>GTGTATTTCCTACACATCATGGAATTATTGATAACACCATGTA<br>TGATCCAATCAAGAAAAGGCTGTTTAAAGTTGGGCAACCTG<br>AAAGTCTTGATCGATCTTGGTGGCTAAAAGAACCAATATGGG<br>TAACAGCTCAGAAACAAGGGAAGGTTACTGGCTGCTTCTATT<br>GGCCTGGATCTGAAGTAGATATTAATCATACACATCCTACAT<br>ACTATAAGAAATATAATTCAAAAGTATCATTTGAATCTAGAG<br>TAGATCAAGTGTTGGCTTGGTTGGATCTTCCTGTAGCTTCACG<br>TCCACATTTTATAACTCTTTATGTAAATGAACCAGACCATGCT<br>CTTCATTACTTTGGGACCAGATCAAAACAAGCAAGTGATGCT<br>CTTGCCAAAGTTGATCGCATGATTGAAAGATTAATTTCTGGA<br>TTGCAGCAAAGAAGTTTAATTAATTGTGTTAATGTTGTAATTA

						DIDAGWTVVTKSTE<br>FNDGICTGGAHGYD<br>NMFPDMRAIFLAHG<br>PSFKKNFTMRPFLNI<br>EVYEMLAELIDVTPN<br>PNNGTRGSMHYIMT<br>SPKTLSPQIEPESLAT<br>GDVPSDESVYNDRL<br>SASTCSCPESPKQLQ<br>VQNSVATKNAHLPF<br>GIPYSTEDSSLSLLYN<br>EDYIDAFSLKYKIPL<br>WSAFTLKGQKEYNV<br>VEDICWKGDARVPS<br>DSSSKCDDYTKITES<br>HQNIVQRPLYSPGFS<br>DPSSTEQATFITNSV<br>AKSFNHTNIIENPFN<br>KMLSKWVVESGELN<br>VVTGPAFDIFANGV<br>RPNEREFMKLFERN<br>GPVLIPTHIFVITTWC<br>STKTDSLKDCNPSKL<br>QSQGYLLPNQPYPA<br>NCEAPAEIISKNVAR	TGTCAGATCATGGAATGTCTGATACGGATTGCAAGCGTGTTA<br>TTGATATTCAAAATTATATTAATGTAAATAATACCTTTTCTAC<br>TGTTGGACCATTTGGCCGTATAAGACCCAAAGGAAAGCGGA<br>ATGCAACTGTGGTCAATGAAATGATAACCAAATTAAGGTGC<br>AAGAGTCCCCACATGAGAGCCTATCCTAAAGAGCAGTTGCC<br>AGTACGCATGCATTATGCTGAAAGCCCACGGATAGAGCCTA<br>TATTTTTGGATATTGATGCTGGATGGACTGTTGTCACTAAAAG<br>TACAGAGTTCAATGATGGCATCTGTACTGGTGGAGCTCATGG<br>GTATGACAACATGTTTCCAGATATGAGAGCTATCTTTTTAGCC<br>CATGGTCCTTCATTTAAAAAGAATTTCACCATGCGACCATTCT<br>TGAATATCGAGGTGTATGAGATGTTAGCTGAATTGATTGATG<br>TCACTCCAAATCCAAACAATGGTACAAGAGGTAGCATGCAT<br>TATATCATGACATCTCCAAAAACTTTATCACCTCAAATTGAG<br>CCAGAATCTCTAGCAACTGGAGATGTACCAAGTGATGAATCT<br>GTTTATAATGACAGACTGTCTGCTAGTACTTGTTCTTGTCCCG<br>AGTCTCCGAAACAACTGCAAGTTCAAAATAGTGTCGCTACTA<br>AAAATGCACACTTGCCTTTTGGGATTCCATACAGTACAGAAG<br>ATAGTTCACTTTCTTTATTGTACAATGAGGATTATATTGATGC<br>TTTTAGTTTGAAATACAAGATTCCACTGTGGTCTGCTTTTACT<br>CTTAAAGGACAAAAGGAATATAACGTGGTAGAGGATATCTG<br>TTGGAAAGGAGATGCCAGAGTTCCATCTGATAGTTCTTCAAA<br>ATGTGATGATTATACAAAGATCACCGAAAGTCATCAGAATAT<br>TGTACAGCGTCCTCTTTATTCACCAGGTTTCTCTGATCCTTCTA<br>GCACTGAACAGGCAACATTCATCACTAATTCAGTGGCTAAAT<br>CATTCAACCATACAAACATCATAGAAAATCCTTTTAATAAGA<br>TGCTCAGCAAATGGGTTGTTGAATCTGGTGAGTTGAATGTAG<br>TAACTGGACCAGCATTTGATATTTTTGCCAATGGTGTCAGAC<br>CAAATGAAAGAGAATTTATGAAATTGTTTGAAAGAAATGGT

							CCTGTGCTTATTCCAACTCACATTTTTGTCATCACAACATGGTGTAGCACAAAAACTGATTCGTTAAAAGATTGCAATCCATCCAAACTTCAGTCACAAGGCTATCTACTGCCAAATCAGCCTTATCCAGCCAATTGTGAAGCTCCTGCGGAGATAATTAGCAAGAATGTCGCAAGA

Phospholipase B							
**Transcript ID**	**Uniprot ID reference protein**	**e-value**	**Identity (%)**	**PFAM domain**	**MW (Da)**	**Amino acid sequence**	**Nucleotide sequence**

PhdEnzPLB 01	F8S101	2,03E-89	34,838	PF04916	62578.91<br>**60563.46**	MISYLLLFSCLTSIAL<br>SSA**IKNASVTYDRQ**<br>**TRKFSLHDSFVSNA**<br>**VAFANFQDEIFQTG**<br>**WSYLEVKTNEQYPD**<br>**PIQAYAAGIVEGYL**<br>**TRDLLNKHWYNTV**<br>**SDYCNGEVLYCQRL**<br>**AKFLQNNLDFINEN**<br>**IKVRRTHDVYWHQ**<br>**IALTLEQLKGLEDG**<br>**SKNITDGRPSTDID**<br>**VMGLLLINIMGDIE**<br>**DLEVVLHKNQKRV**<br>**FGSGSCSALVKVLP**<br>**NFKDLYFSQDTWSS**<br>**YNTMLRILKKYSLK**<br>**LHITMDNDASLIPG**<br>**HTCTFSSQPGMIYS**<br>**GDDFYLISSGLATM**<br>**ETTIGNGNASLWQ**<br>**YVKAEGTILEWQRN**<br>**IVANRLAKNGDQW**<br>**TKLFGIMNSGTYN**<br>**NQWMVLDYKKFKP**<br>**GQPLQDGLLWVLE**<br>**QLPGYLHSEDKTDV**<br>**LRNQSYWPSYNTA**	AAAAGACTTCACGTGCTGCTGAGTGTTTTGTTTTCGTAAATAA<br>AATGAGTTTGCAATTTTGGCCTCAATTAGATTGATAAGTTTGT<br>TGCTGAATATAAGAGATCTTCAAACATGATTTCATATCTCCTG<br>TTATTTAGTTGTTTGACTTCTATAGCATTGTCTAGTGCTATTAA<br>AAATGCATCTGTTACATATGATAGGCAAACAAGAAAGTTTTC<br>TCTTCATGATTCTTTTGTATCTAATGCAGTTGCCTTTGCAAACT<br>TTCAAGATGAAATTTTTCAAACCGGGTGGTCTTATCTTGAAGT<br>AAAAACTAATGAACAGTATCCTGATCCTATACAAGCCTATGC<br>TGCTGGTATTGTTGAAGGATATCTTACAAGAGATTTGCTAAA<br>TAAACACTGGTATAATACAGTTTCTGACTATTGTAATGGTGA<br>AGTTTTGTATTGCCAGAGATTAGCCAAATTTTTACAAAATAA<br>TTTGGATTTTATAAATGAAAATATCAAAGTAAGAAGAACCCA<br>TGATGTGTATTGGCATCAGATTGCTTTAACATTGGAACAGTTG<br>AAAGGGTTAGAAGATGGATCAAAGAATATTACTGATGGCCG<br>GCCTTCTACTGACATAGATGTTATGGGATTGCTATTGATTAAT<br>ATAATGGGTGATATAGAAGATCTGGAAGTTGTTTTGCACAAA<br>AATCAGAAGAGAGTGTTTGGAAGTGGATCATGCTCAGCATTA<br>GTAAAAGTTCTACCCAATTTTAAGGACTTGTATTTTTCTCAAG<br>ACACTTGGTCTTCTTATAACACAATGTTGAGAATTTTGAAGA<br>AATATAGTCTGAAGTTACATATTACAATGGATAATGATGCTT<br>CTCTAATACCAGGCCACACATGCACCTTTTCATCACAACCTG<br>GGATGATTTACTCTGGAGATGACTTTTATTTGATATCTTCAGG<br>ATTAGCTACTATGGAAACGACTATTGGAAATGGTAATGCAA<br>GTTTATGGCAATATGTAAAAGCTGAAGGAACTATTCTGGAAT<br>GGCAAAGAAATATTGTTGCAAATAGATTGGCTAAAAATGGA<br>GATCAGTGGACAAAACTCTTTGGAATAATGAACAGTGGCAC<br>TTACAATAACCAGTGGATGGTTTTGGATTATAAGAAATTTAA<br>ACCTGGCCAGCCTTTGCAAGATGGATTATTATGGGTTCTAGA

						**<u>YFKDIFNLSGGQLNVDKYGDWFTYEKNPRALIFKRDHSKVSDIDSMIKLMRYNDYTNDPLSRCNCTPPYSAENAISARCDLNPSNGTYPFAALGHRQHGATDMKLTNYEMFKRFEFVSFGGPTYDPLPPFQWSKSDFGKTVKHEGHPDLWKFKPIIHKWL*</u>**	ACAATTACCGGGTTACTTGCACAGTGAAGACAAAACCGATGTGTTAAGGAATCAGTCCTATTGGCCCTCATACAATACAGCGTATTTTAAGGATATTTTCAACTTAAGTGGTGGTCAGCTCAATGTTGATAAATATGGCGATTGGTTTACATATGAAAAAAACCCCCGTGCCTTAATTTTCAAACGGGATCATTCTAAAGTGTCTGACATTGACAGCATGATTAAGCTTATGAGGTACAATGATTATACCAATGATCCCTTATCACGCTGCAACTGCACACCTCCTTACAGTGCAGAAAATGCCATCTCTGCCCGTTGTGATCTTAATCCTTCCAATGGAACTTATCCATTTGCTGCTTTAGGCCATAGACAGCATGGAGCTACAGATATGAAGTTAACAAACTACGAGATGTTCAAAAGGTTTGAATTTGTATCATTTGGAGGCCCTACTTATGACCCACTACCACCATTCCAGTGGAGTAAATCAGATTTTGGCAAGACTGTTAAGCATGAAGGACATCCAGATTTATGGAAATTTAAGCCAATTATTCATAAATGGTTGTAAATTAAATATTCCTGTTTGTGGAAATTTGTTATTAATGATATTCTTATAGTGAGATATATATTTATAAAAATTATATGTGCATGCTATTTTTTTTCAGTGGTGGGCCCAGAGCATTGTTCCTTTCTAATTTTATTAATAACATTAATATTATAAGACATGCATAAATGAATGTTGATAAAAAATAAATTTTGTTTGTATTTATATTTTCATTAATATGAGCTTTG
Endonuclease							
**Transcript ID**	**Uniprot ID**	**e-value**	**Identity (%)**	**PFAM domain**	**MW (Da)**	**Amino acid sequence**	**Nucleotide sequence**

	reference protein						
PhdEnzEnd01	J3SBP3	3,45E-06	47,368	PF01223<br>PF01663<br>PF01033	4743,6	IISKNVARVDDIEKLTGISFYVGLPIYEAIRLRTMMPERSF*	TGACTTCAATATATAATTTATGATTCTCTGAGATGTGTATTTACATTTCTGAGTGTAAAAATATTTTCCCAAATATAAATTTCTTCGTGATTAAAAAGACCTTTCTGGCATCATAGTTCTTAGTCTAATGGCCTCATAGATAGGAAGTCCCACATAAAAGGATATTCCAGTCAATTTTTCAATGTCATCTACTCTTGCGACATTCTTGCTAATTATC
Ligase							
**Transcript ID**	**Uniprot ID reference protein**	**e-value**	**Identity (%)**	**PFAM domain**	**MW (Da)**	**Amino acid sequence**	**Nucleotide sequence**
PhdEnzLig01	Q2XXL4	6,00E-05	44,737	PF13765	12385,95	LTVSCDSYEHRVVLGNIGFSRGIHYWEVTIVKYDNNADPAFGIARFDVIKDLMLGKDDKGWSMYIDHQRSWFLHADCHENRTEGGIEIGSVIGILLDLEQHQLSFFVN	CATTTACAAAAAAGCTAAGTTGATGCTGCTCTAGGTCTAGAAGAATGCCAATAACAGAGCCTATTTCTATGCCACCTTCAGTTCTATTTTCATGACAGTCAGCATGAAGAAACCATGATCTTTGGTGATCAATATACATGCTCCAGCCTTTGTCATCTTTACCAAGCATGAGATCTTTAATTACATCAAATCGTGCTATACCAAAAGCAGGATCTGCATTATTATCATATTTAACTATTGTCACTTCCCAATAATGTATGCCTCTTGAGAAACCAATATTTCCTAATACAACTCTATGTTCATAACTGTCACAGCTAACAGTAAG
Lipase							
**Transcript ID**	**Uniprot ID reference protein**	**e-value**	**Identity (%)**	**PFAM domain**	**MW (Da)**	**Amino acid sequence**	**Nucleotide sequence**

PhdEnzLip01	P0CH47	7,63E-17	36,170	PF00151	9565,75	LHGFREKGDQDWIKDLIRELLTSDDNNIITVDWRMGAAQSYPQAAANTRTLGLVVADFIDYLQRKYRISPSDIHIIGNNLGAHAA	TTTTGCATGGATTTCGGGAAAAAGGAGACCAGGACTGGATTAAGGACCTCATTCGAGAGCTATTGACATCAGATGACAACAATATTATTACAGTCGATTGGCGCATGGGCGCAGCCCAATCTTACCCTCAAGCTGCTGCAAATACAAGAACTTTAGGACTTGTAGTGGCTGATTTTATTGACTACTTACAGCGTAAATATCGAATATCACCATCCGATATTCACATCATCGGCAATAACTTAGGAGCTCACGCTGCCGG
NEUROTOXINS							
Non specific toxins							
**Transcript ID**	**Uniprot ID reference protein**	**e-value**	**Identity (%)**	**PFAM domain**	**MW (Da)**	**Amino acid sequence**	**Nucleotide sequence**

PhdNtxNSp 01	P84016	1.61E-13	87.500	PF08116	7962.21<br><br>**6010.64**	MKLSIFLVLLLITLVR<br>P**EEANMNFLVEAES**<br>**GVPEINEERAFCRF**<br>**NGEQCTSDGQCCN**<br>**SRCINAFEGRICMG**<br>*	CACGAGATTAGCAGAAATATAGGTTAAGGAATATTGGTATA<br>AAGGGCCTAAATTTCGTTATTTAAACAATTACTGCAAATTGTT<br>AAAATGCGGAGTAATATGATGAAAAATACGACAAAACTGCT<br>ACCTTTCAAATGTAATCGAAAAATGTTCTGAGTTTTCAATTCT<br>GTTTTAAAAAAAATTTTGCTATGTTCTTTCCAAGAAATTCAAA<br>TATCATACAAACGTATCTCCGTTCAACGACCTGTCGGTTACG<br>ATGTGAGAAGAAATAGTATCCATATTTCTATTCTAAAATTTTA<br>CTTGTGCCGCCGAATGGCGTGCTGTTAGCTCTGGCTACTTCTC<br>TAGAGCAGGGTCCGGTTTGGACTCCGGATAAGACCTCCATGT<br>TTTATCTTGTCGTTCATACGGGTAGAATTGGTCGTCCATGTGA<br>TGCACCCATAATAAAATGGCCAAAAATTGTGGCAGTAATTGT<br>CTGAGTTCGGATCAATCATTGATTGCTCCAGGTCTGCCATAA<br>AATTGTTTTACTCATTAATATTTGGTTGTAAACGTTATTTCTAA<br>CGCGTTAAATATTATAGCTATCTGGCATCAAGGAGGTACATG<br>CTGTTCTTCACCTAAAATGGAGATTTTTCGTCTTTGGAAAATA<br>ACCCAACCACCAGGTGCTAGCTCTAGTTAGAGTGATACCTGA<br>AGTATGGTAACGAAACATTAAATTTTTGATTTTCGTAATCCAT<br>CTCCCCGAAAATGAGAGCATAGGAATTCGTTGTAACTTAAAC<br>TTGAAATGTTTGAACTAAATGGTTATTAACTCATTTAAATGA<br>AAGAAAGGTCTTTTGGTATTGTTCTTTATCCGGCACTAGCGAT<br>AGCAACAGAGGAAGGACGTTTATGATCAGTCTTCTCCTTAAA<br>GAGACTTAATCAGTAGGTAAGGGTCAACAAGATTTTTTTTAT<br>AAAAGTTTGGAATCCGATGCAAAAGAAACAAATAACACATA<br>CATACGCATATACAAACTTAATAATATCTTTAATTGTCTGAA<br>ATTGGAGCCCTTTCTTAAATTCAGGAATCATATTTTCGACTCG<br>TGAAATTTCTCTTTTTCAGTCTCAGTCATGATTTTCCTGTGGCG<br>CATCAAAGGCAGGTATAGGAATTTCTGGTGATGTTTCACATC<br>AACTTAGAAAGAAATTGCATGTGTCAATCACGAAAAGAAAC

							GAATTGGAGAGGTGGTGGATTTACGTTATTTTTATTACGCGAT<br>TGGCTTGCGGCTACTAATACATATCTCTCTCAGGTTAACCTTT<br>CCCATCTTGATCTCCTGTCAGTGAGTACCATGTTCGTATTCCT<br>TTTAGAAAAGTATCTGGTGCACAAATATAAGTAATTTGTCTTT<br>ACCTTTTATTCAACATGTTGAGTTGAACATGTGTTCTTTTTTCC<br>TCGGGATAGTTCCGACAACGAACAAGTACCTATATCACGAT<br>GTTATTCTCAATCAGATAAAAATCTGCGGACCTTTAACAGTT<br>GGAGCACGATTTTTAAATTGTCTGGAATTCATCCACTTACAA<br>AAGTCCTCCTCACACCTTTATTGTGATAGTCCGAATGCGAGC<br>ATGTAATGCAGGCGTATCGATGGCTTTTGCTCCAAACATTAA<br>CACCTGTAACGTTCAGATTGAAAATGCGTTAAACCATCTAAT<br>ACATTTTAATGAAATACAACCAAATATCGAATGCGTTCTTAC<br>GACTCTTAGCGTTTCACGTCATAATTTTCGAGTATCCAAAGCG<br>AATATGAACGCCCAAAACACTGTAACTGTCTCAAACGTGCC<br>ATGAAATGAAAGAAGACCGTTACATACACATACGAATTTAT<br>TGAAACCCTGCATTTCGTGTATTTATCCCAAATTATTAGGAAA<br>ATAGGTCAAACTAAAAACTTTACGTTAACTTAAAGATATGAA<br>ATTGTGCTTATCGTCTAGACAATTACAAAATTTAGTCATAGTA<br>ATTATTTTCATTACGTGTGTTTGTGAGTGTTATGTATATGATTT<br>TTTAATACCCTGTCACAGATTTTAAGCGGTAATAGAAGTAAT<br>TTTATGTACTTTTACTATCTCTTATTAAGCGAGTCGATGTACA<br>AATCATGGTGTATGTAAATCTTATCACGAAGGGTGTGGTAAC<br>GTCTTCTAAACAACAAATAAATTCTTCCAATGACCCAGATAT<br>TTGCGGTAGGAAAAGCTCTCATAACACAATTGATGAGGGTG<br>GCCGAGAGGCAACAGAAAAGAGTACTACATGAAGTAGTATT<br>ATATGCAAGATTTATAGACTTCAAGCTTTCAAGATTTCAAAT<br>GTTGCTCTCTTTACAAATGCTTGCCAGAGAGATATACGCAAT<br>CAAAAAAAGAGCGAAAATCATCTTTTTCTTCTTAGAGCTTAT

							TTTAAGCTCTAATATCGTTTCAAATCATATTGTTAATTAATTC<br>CTCTGAAGATGAAGGCAGTCACCTTAAATTTTTTGTTATGCCA<br>ATTGTGTTTAAAAGAAGGAAACGAAAAAATCAAAGAGAGA<br>ACAAATAATCATGGAAAAAGAACAAATACAAGGTAATGAA<br>AATAATGTAATTGAATTAAATAATAATGTCATTTAATGAATT<br>GCATTGAGTAACAATGTGGCTTCTAAATTTCGTTGATCCAAA<br>AGTACATCAAATTACAAAAATAAAAGACAAGATATATATCA<br>ACACGCACCATACTTTTCCTCAGGGGAATGAATAGTAGTAAA<br>AGAAAATTCCACTTTCTTCGAAATTTACGCTAATTGTGAACTG<br>AAAATTACCATTTTTCGGCTTAACTGCTTCAAGCAAATTAAA<br>GAACAACCAGAACCTTCTTGCATCATGTTCAAGTGTCAGATC<br>GATGCTGCAAAAACTGAAACCCATCACTTAATATCTTGATAA<br>CTGCGTAAAAAAGCACAACACTTCCCCTGAGTGCCGTCAAA<br>TGAAGGTAGGGAGAATGCTAATGTAAATGTGTAGACAGACA<br>ATCTCTAGGTGCAAGGTCATCAATATATATATAGTAACTATG<br>GATGTGAAGACTTTCTGCACACACACGGCAGAATGGATTGG<br>CTCAACATCGAATGAAGAGTCTTTTGGAGATATAGAGGATGC<br>GGAGAGGTAAATAGGCAGGAGATAATCGGTTGAGATTATGA<br>GACCGCTACAGGTAACGAAATGGTATAGATGTTAGTAGAAT<br>TAGAAATGCAACTCCCAGTGGCAATGGTATATGTGCACATGC<br>CATAATATAAATATGTCACATCGTGTCAAACCTTTCGAACCA<br>TATACGCCAGAAATGAAATGATCATCAGGATCGAAATGATT<br>CAGAAAATAGCCACAACGAAGTTAATGTTTGCACGGAGATT<br>TCTTTACGTTTTATTAAATACTATTTCCTGAAAATAGTGTAGC<br>ACTTTATTACGATAAACTACTGAATTTCAAAATAACTTGCAG<br>AATTTTTTAAAACACAGTTGCAAGAAATCAAATCATTTGCTTT<br>ACAAAATATATTTAATTCATCACAATGTATCAGAAAAATCTG<br>GGAACCCAAATTTTTATACATTAAATACTTAATACAGTAAAT

							TACTTATACATTAAATTACTTATAAATAATCGAATCACGTAAATAGAACGCAGTAAAAATCAAAATATATTGACTCTGGTACGTGAACTGTTTAATGGTTAGGGACACATGATGTTAGAAGTGTAGTTGGTGAGACTAACTCGACAATTTATGAATTTCATTTTGCAATGGATTTTCAACTATTATCAATATATACATACAATTTCAAACAGTATTATATGTAATCGTGTCATTTCTTTGCTAAAATAACACTCATTTCCCGTAATATATCATAATATCTGTAAATAGAATTATTGATATAATAAACATGCTTTTTTAGAAATTTAATCCTTTATGTTTTCTACACTTCTTTTCTTCTCTAATCTTCTTTTCATGATAGATTATTTCTCATATTTGTTCTTTCTGAAATCGGCCTAAGTTGCTGCTTAATTTTTGTTTCAGTAATGTTAATTTCTCTTGTTACTTAACTTGCGCATGTAAGCGAATAAAAAATTGAAAAATCTTAATTGCGGTTTTGCTTCTTACAATCAAAATATTTTTAATGTTTTGAAGTAGAACCAAAATGCTTAAATAAAAGCATTACCTTGTTTGTGCGTCTAGCATTTATGTCTCAGAAACACTAAAAATGTTCTAGTATCTTAAAGATTCATAAAGTTTATATTCATTTACTTAATTTATTGTTTCTATATTTTCGCAAGCGAAAACGTTATCGGCTACTGCATGCATTCAGTGTAATATGTGAAAGAATGAAATTCTAAATTTAAATACATAACTTATAATGAATGGAACCTACATTACTCTCATTGTACAGTATTCTAAAATGCACTCACAAGATATAATCCACAAATGCTAAAGAATCTGCAACTTACTAGAAAATCTTCAAATGCATTACTTTGCATCGTAAATATCACTGTTTCTTTTAAAAAGGTTCTCAAATATGATCAGAGTAGAGAAATTTTGAAATTGATCGCTTGATATCGATTCCATTATCAGTTAAAATGTTCGAGTATGGAGTAACTAAAAATTATTTGTTTAATTGATCAGTTTCGCTAACTGCAAATTTAGTTAAAGTGACAAAAAAAAAAGACTATTTACGAAAGAACAAAGAATATGGCACATTGGAAAACTCTATATTTTATGCTTTTTAGTTTGCCAAAATTTTAATTGAAGATAGAAACGAAAAACAGAA

							ATTGAGCAGGGACATTGCATATTTCTTTGCTCTGTATCTCAATTTAAAAGTTTTCTGGTGTCTTAATGTTATTTTTTCTTCATATTTCTTTAAACATGCATGTTTACTGATTGTTAATCAGACACGAAGAAGTATCATTATCAATAAATGAAGTAGAAGAATGAAGTAGTAGTAGAATGAATGATGATTAGAAGTATCATTATCAATTCCATTCGAACCATTGTCATTTTGTGTTTTTAACGTGGTTGTCAATATTATTTTCTGTTTTCCGGTTCCTTGTAAGTAGTGCATTTCTTTCTTGCGTATAGAGATATACTTTTGTAGCAAGGAATTTATTTTTTTTATTGTGCTGATAACAATCAGCAGAAAATACAATTATTTATTAGAGTAGTCACGAGCAGCAAGAAATAATTTCAAAATTTACGCCATTTTCATCAGTATTTCTTTACCCCATGCATATCCTTCCCTCAAACGCATTTATACATCTACTATTGCAGCATTGACCGTCGCTTGTACATTGCTCTCCGTTGAATCTACAAAATGCCCTTTCTTCATTGATTTCTGGAACTCCAGATTCTGCTTCAACTAGAAAATTCATATTCGCTTCTTCTGGGCGCACGAGAGTTATCAAAAGTAGAACGAGAAATATTGAAAGCTTCATGGTTCAACGTATGGAAATCTGTTCACTATAAATCTTCAGAGGTTATCAAAAATGTAACATCTAATTTGCAAAAGTCTTGGTTCTTATATGTATTTAAATGGACTATTACTTCATCATCAAATAGCAATGTGCATGGTAACGTCACTGCAGATGTTGAAATATAAAATACCTGATTGCTCATATGCATATGGTTTGATCACACACATTTCTTAAGAATAGCATTGAAACACTTCAGAGGATAGCAGCAGTCATCGTCGCTTTTACAGGGACGGCTGATGAAACTACAAAGGCCATCAAATAAACTAAACTTTCTTTCTTCATTGATTTCTGAAGCTTTGTCTTCTGCTTCAACAGGTTTTGGAAATCACTTCAGTAAAAATCTTCAGAGATTAGCAGAAATGTAAGATATAA

PhdNtxNSp 02	P84016	1.33E-15	87.500	PF08116	7946.21<br><br>**5994.64**	MKLSIFLVLLLITLVR P**EEANMNFLVEAEA GVPEINEERAFCRF NGEQCTSDGQCCN SRCINAFEGRICMG** *	TTTTATTGTGCTGATAACAATCAGCAGAAAATACAATTATTT ATTACAGTAATCACGAGCAGCAAGAAATAATTTCAAAATTT ACGCCATTTTCATCAGTATTTCTTTATCCCATGCATATCCTTCC CTCAAACGCATTTATACATCTACTATTGCAGCATTGACCGTC GCTTGTACATTGCTCGCCGTTGAATCTACAAAATGCCCGTTCT TCATTGATTTCGGGAACTCCAGCTTCTGCTTCAACTAGAAAA TTCATATTCGCTTCTTCTGGGCGCACAAGAGTTATCAAAAGT AGAACGAGAAATATTGAAAGCTTCATTGTTCAACGTATGGA AATCTGTTCACTATAAATCTTCAGAGGTTATCAAAAATGTAA CATCTAATTTGCAAAAGTCTTGGTTCTTATATGTATTT
PhdNtxNSp 03	P84018	1.05E-12	80.645	PF08116	6313,32	RFLVKRFPKLVEAEA GVPEINEERQFCSYP GEQCTSDGQCCYGK CRTAFKGMICMG*	CCATTTTCATCAGTATTTCTTTACCCCATGCATATCATTCCCTT AAACGCAGTCCTGCATTTACCATAGCAGCATTGACCGTCGCT TGTACATTGCTCGCCCGGGTAACTACAAAATTGCCTTTCTTCA TTGATTTCTGGAACTCCAGCTTCTGCTTCAACAAGCTTTGGAA ATCTCTTCACTAAAAATCTTCAGAGAATATCAGAAATGTAAC ATCTGATTTGCAAGAGTCTTGGTTCTTATATGTATTTAAATGG ACTATTACTTCATCATCAAATAGCAATGTGCATGGTAACGTC ACTGCAGATGTTGAAATATAAAATACCTGATTGCTCATATGC ATATG

PhdNtxNSp 04	P84016	7.37E-14	87.500	PF08116	7962.21<br>**6010.64**	MKLSIFLVLLLITLVRP**EEANMNFLVEAESGVPEINEERAFCRFNGEQCTSDGQCCNSRCINAFEGRICMG***	AATGCAACTCCCAGTGGCAATGGTATATGTGCACATGCCATA<br>ATATAAATATGTCACATCGTGCCAAATCTTTCGAACCATATA<br>CGCCAGAAATGAAATGATCATCAGGATCGAAATGATTCAGA<br>AAATAGCCACAACGAAGTTAATGTTTGCACGGAGATTTCTTT<br>ACGTTTTATTAAATACTATTTCCTGAAAATAGTGTAGCACTTT<br>ATTACGATAAACTACTGAATTTCAAAATAACTTGCAGAATTT<br>TTTAAAACACAGTTGCAAGAAATCAAATCATTTGCTTTACAA<br>AATATATTTAATTCATCACAATGTATCAGAAAAATCTGGGAA<br>CCCAAATTTTTATACATTAAATACTTAATACAGTAAATTACTT<br>ATACATTAAATTACTTATAAATAATCGAATCACGTAAATAGA<br>ACGCAGTAAAAATCAAAATATATTGACTCTGGTACGTGAACT<br>GTTTAATGGTTAGGGACACATGATGTTAGAAGTGTAGTTGGT<br>GAGACTAACTCGACAATTTATGAATTTCATTTTGCAATGGATT<br>TTCAACTATTATCAATATATACATACAATTTCAAACAGTATTA<br>TATGTAATCGTGTCATTTCTTTGCTAAAATAACACTCATTTCC<br>CGTAATATATCATAATATCTGTAAATAGAATTATTGATATAA<br>TAAACATGCTTTTTTAGAAATTTAATCCTTTATGTTTTCTACAC<br>TTCTTTTCTTCTCTAATCTTCTTTTCATGATAGATTATTTCTCAT<br>ATTTGTTCTTTCTGAAATCGGCCTAAGTTGCTGCTTAATTTTTG<br>TTTCAGTAATGTTAATTTCTCTTGTTACTTAACTTGCGCATGTA<br>AGCGAATAAAAAATTGAAAAATCTTAATTGCGGTTTTGCTTC<br>TTACAATCAAAATATTTTTAATGTTTTGAAGTAGAACCAAAA<br>TGCTTAAATAAAAGCATTACCTTGTTTGTGCGTCTAGCATTTA<br>TGTCTCAGAAACACTAAAAATGTTCTAGTATCTTAAAGATTC<br>ATAAAGTTTATATTCATTTACTTAATTTATTGTTTCTATATTTT<br>CGCAAGCGAAAACGTTATCGGCTACTGCATGCATTCAGTGTA<br>ATATGTGAAAGAATGAAATTCTAAATTTAAATACATAACTTA<br>TAATGAATGGAACCTACATTACTCTCATTGTACAGTATTCTA

							AAATGCACTCACAAGATATAATCCACAAATGCTAAAGAATC<br>TGCAACTTACTAGAAAATCTTCAAATGCATTACTTTGCATCGT<br>AAATATCACTGTTTCTTTTAAAAAGGTTCTCAAATATGATCAG<br>AGTAGAGAAATTTTGAAATTGATCGCTTGATATCGATTCCAT<br>TATCAGTTAAAATGTTCGAGTATGGAGTAACTAAAAATTATT<br>TGTTTAATTGATCAGTTTCGCTAACTGCAAATTTAGTTAAAGT<br>GACAAAAAAAAAAGACTATTTACGAAAGAACAAAGAATAT<br>GGCACATTGGAAAACTCTATATTTTATGCTTTTTAGTTTGCCA<br>AAATTTTAATTGAAGATAGAAACGAAAAACAGAAATTGAGC<br>AGGGACATTGCATATTTCTTTGCTCTGTATCTCAATTTAAAAG<br>TGCTCTGGTGTCTTAATGTTATTTTTTCTTCATATTTCTTTAAAC<br>ATGCATGTTTACTGATTTTTAATCAAAGACACGAAGAAGTAT<br>CATTATCAATAAATGAAGTAGAAGAATGAAGTAGTAGTATA<br>ATGAATGATTAGAAGTATCATTATCAATTCCATTCGAACCAT<br>TGTCATTTTGTGTTTTTAACGTGGTTGTCAATATTATTTTCTGTT<br>TTCCGGTTCCTTGTAAGTAGTGCATTTCTTTCTTGCGTATAGA<br>GATATACTTTTGTAGCAAGGAATTTATTTTTTTTATTGTGCTGA<br>TAACAATCAGCAGAAAATACAATTATTTATTAGAGTAGTCAC<br>GAGCAGCAAGAAATAATTTCAAAATTTACGCCATTTTCATCA<br>GTATTTCTTTACCCCATGCATATCCTTCCCTCAAACGCATTTA<br>TACATCTACTATTGCAGCATTGACCGTCGCTTGTACATTGCTC<br>TCCGTTGAATCTACAAAATGCCCTTTCTTCATTGATTTCTGGA<br>ACTCCAGATTCTGCTTCAACTAGAAAATTCATATTCGCTTCTT<br>CTGGGCGCACGAGAGTTATCAAAAGTAGAACGAGAAATATT<br>GAAAGCTTCATGGTTCAACGTATGGAAATCTGTTCACTATAA<br>ATCTTCAGAGGTTATCAAAAATGTAACATCTAATTTGCAAAA<br>GTCTTGGTTCTTATATGTATTTAAATGGACTATTACTTCATCAT<br>CAAATAGCAATGTGCATGGTAACGTCACTGCAGATGTTGAA

							ATATAAAATACCTGATTGCTCATATGCATATGGTTTGATCAC<br>ACACATTTCTTAAGAATAGCATTGAAACACTTCAGAGGATAG<br>CAGCAGTCATCGTCGCTTTTACAGGGACGGCTGATGAAACTA<br>CAAAGGCCATCAAATAAACTAAACTTTCTTTCTTCATTGATTT<br>CTGAAGCTTTGTCTTCTGCTTCAACAGGTTTTGGAAATCACTT<br>CAGTAAAAATCTTCAGAGATTAGCAGAAATGTAAGATATAA

PhdNtxNSp 05	P84018	6.61E-13	80.645	PF08116	7928.29<br>**5976.72**	MKLSIFLVLLLITLVR<br>P**EEANMNFLVEAEA**<br>**GVPEINEERQFCSYP**<br>**GEQCTSDGQCCYG**<br>**KCRTAFKGMICMG**<br>*	CCATTTTCATCAGTATTTCTTTACCCCATGCATATCATTCCCTT<br>AAACGCAGTCCTGCATTTACCATAGCAGCATTGACCGTCGCT<br>TGTACATTGCTCGCCCGGGTAACTACAAAATTGCCTTTCTTCA<br>TTGATTTCTGGAACTCCAGCTTCTGCTTCAACTAGAAAATTCA<br>TATTCGCTTCCTCTGGGCGCACGAGAGTTATCAAAAGTAGAA<br>CGAGAAATATTGAAAGCTTCATTGTTCAAGCTTTGGAAATCT<br>CTTCACTAAAAATCTTCAGAGAATATCAGAAATGTAACATCT<br>GATTTGCAAGAGTCTTGGTTCTTATATGTATTTAAATGGACTA<br>TTACTTCATCATCAAATAGCAATGTGCATGGTAACGTCACTG<br>CAGATGTTGAAATATAAAATACCTGATTGCTCATATGCATAT<br>G
PhdNtxNSp 06	P83915	4.11E-11	80.645	PF08116	6696,95	MHLIFTPQMSPLCRF<br>HHKILFFFYRQFCSY<br>PGEQCTSDGQCCYG<br>KCRTAFKGMICMG*	CCATTTTCATCAGTATTTCTTTACCCCATGCATATCATTCCCTT<br>AAACGCAGTCCTGCATTTACCATAGCAGCATTGACCGTCGCT<br>TGTACATTGCTCGCCCGGGTAACTACAAAATTGCCTATAAAA<br>GAAGAATAAGATTTTATGATGAAACCTACAAAGAGGAGACA<br>TTTGGGGAGTGAAAATTAAATGCATGTGCTACTTAAAGAATG<br>AAGATATATTAGATGTTATCTAAACATTTTAATTGGTTTAAAT<br>CACGTGACAAAATTCAGCAACGTTTAACATTTATCAGTTCTTT<br>TTTAGTAATGATATAATAAAAGAACCATACATTAATTATAGG<br>ACAGTCATCCAGTAAGACTCAATCTTAAATAGGAATTTGTTT<br>AAATGGATGTTTAATGGCCTGAGAACTGTTTGGACCACACAG<br>TCTGTCTTTCCGCAGTGAAACGACTTATATGTTGAATGCATTT<br>AATCGCAATGGATAAAATTTGGTGCTTAATATATTGATGGTA<br>TCGTATATAGTTCCTAACGTAAGAAATAATATTTTAACATTTT<br>TGGGAAATTAGTGTTTGTGAAATTGCTATATTTTCCTGAAAAT<br>AAATTCGACAATTTTGTTTAAGACAGTCATTAATATTCTTATT<br>CACTTATGAGAACATTTTACCTTCTGTCCTGCATTTTTCATTAT<br>CTATGACCCTTAAATGTTATATTTACGTATATAATACATTTAT

							GCTCTGAAAATTGTCAGATTACGTCATTATCTCCTTGAGAGGAAACTTATATAATACTCATAAATATTGAGGTGTTTCTCCATTGCATTACATGTCTGGCACATTTATTTTTGTGCTGTCCATCAAGCCGTTATTAGTTCTAATTTGCAAGATTGTGATTTTTTAAATTAGTGATATATCTTGTATATTTATAGTATGCATTACATTGCAATCATATTCAATTTCGTGAAAGTGAGTATATTCACATTTTACTATTCAAGTTCGATATACTTACCTTTCTTCATTGATTTCTGGAACTCCAGATTCTGCTTCAACTAGAAAATTCA

PhdNtxNSp07	P83915	7.21E-11	80.645	PF08116	6696,95	MHLIFTPQMSPLCRFHHKILFFFYRQFCSYPGEQCTSDGQCCYGKCRTAFKGMICMG*	CCATTTTCATCAGTATTTCTTTACCCCATGCATATCATTCCCTTAAACGCAGTCCTGCATTTACCATAGCAGCATTGACCGTCGCTTGTACATTGCTCGCCCGGGTAACTACAAAATTGCCTATAAAAGAAGAATAAGATTTTATGATGAAACCTACAAAGAGGAGACATTTGGGGAGTGAAAATTAAATGCATGTGCTACTTAAAGAATGAAGATATATTAGATGTTATCTAAACATTTTAATTGGTTTAAATCACGTGACAAAATTCAGCAACGTTTAACATTTATCAGTTCTTTTTTAGTAATGATATAATAAAAGAACCATACATTAATTATAGGACAGTCATCCAGTAAGACTCAATCTTAAATAGGAATTTGTTTAAATGGATGTTTAATGGCCTGAGAACTGTTTGGACCACACAGTCTGTCTTTCCGCAGTGAAACGACTTATATGTTGAATGCATTTAATCGCAATGGATAAAATTTGGTGCTTAATATATTGATGGTATCGTATATAGTTCCTAACGTAAGAAATAATATTTTAACATTTTTGGGAAATTAGTGTTTGTGAAATTGCTATATTTTCCTGAAAATAAATTCGACAATTTTGTTTAAGACAGTCATTAATATTCTTATTCACTTATGAGAACATTTTACCTTCTGTCCTGCATTTTTCATTATCTATGACCCTTAAATGTTATATTTACGTATATAATACATTTATGCTCTGAAAATTGTCAGATTACGTCATTATCTCCTTGAGAGGAAACTTATATAATACTCATAAATATTGAGGTGTTTCTCCATTGCATTACATATCTGGCACATTTATTTTCGTGCTGCACATCAAGCCGTTATCAGTTCTAATGTTTGCAAGATTAAGATATTGTAAATTGATGATATATCTTATTTATATTATGTACTACATTGCAATCGTGTTCATTTTTGTATTCAAGTTCCATATATACTTACCTTTCTTCATTGATTTCTGGAACTCCAGCTTCTGCTTCAACTAAAAATAGAAAAAAATATGGTTAATTATAGCGCATAGTTATTAGAAGACTGGAACTGGGTCATACAATTTATATGATTCCAGCGTGAATATTTACTGACATTCTTATGTGAAGTTTTTCTTAGAAATTCTGTCATCCATACTCTATATAATATCACAGAAGCAGCTGAATACCGCTGGC

							TCCATGTAATCGCAAAATCAAAGTTTAGGTAACATTAGGGTAACATTAGGTAACATTTTATCATACAAAGAAGCTGCACACTCTCGCAGAATGTTTAAGGAATAAACAGTATATTTAACAACTGCAAAGCCCTCGAAGTTGAATGGTAACACAGGCAAAGAGCGAGAGGCAGGAATTCTTTCGAGCTTAAAATGAGGAGTATAATTTTAAAGGGAGATTAAATTTATTTAGATGTCTTCAAAGGTTTTCAGTTCTCCTGATGCCAAACATGAAATGTATAAACTGCTACTAATCTGATTTTAGCACGAAGGTAGAGAGATACAAACACAGTTTTTTTATTAATACCGAACCATTCCCTCTCTCTGCAAAAATATTATATAATTTAATTCCGTGGAGTAATTTAATTACGCTGAGATGACAGAGTTGACAGATAAAATTATTAAAAATTATTTTCTCTCACGCATTAAGAGGAATGCTTCTTTTGATGTTTGCGAGAAAATAATTTGTGAAGAGACTTCCTCGAGTGTTTTACATTAGAATATTTTACATTAAATGTTTTTCATTTATAAAATAATATAGGTTACTTATACAGTAAAGCATATAACATATTACTTAAATATTTGTGCTTAATAACGACTTATAGGAAAAA

PhdNtxNSp 08	P84018	6.21E-13	80.645	PF08116	7928.29<br><br>**5976.72**	MKLSIFLVLLLITLVR P**EEANMNFLVEAEA GVPEINEERQFCSYP GEQCTSDGQCCYG KCRTAFKGMICMG** *	CCATTTTCATCAGTATTTCTTTACCCCATGCATATCATTCCCTT AAACGCAGTCCTGCATTTACCATAGCAGCATTGACCGTCGCT TGTACATTGCTCGCCCGGGTAACTACAAAATTGCCTTTCTTCA TTGATTTCTGGAACTCCAGCTTCTGCTTCAACTAGAAAATTCA TATTCGCTTCCTCTGGGCGCACGAGAGTTATCAAAAGTAGAA CGAGAAATATTGAAAGCTTCATTGTTCTGAAATCTTGATTTTT TTAAGCAAGCTTTGGAAATCTCTTCACTAAAAATCTTCAGAG AATATCAGAAATGTAACATCTGATTTGCAAGAGTCTTGGTTC TTATATGTATTTAAATGGACTATTACTTCATCATCAAATAGCA ATGTGCATGGTAACGTCACTGCAGATGTTGAAATATAAAATA CCTGATTGCTCATATGCATATG

PhdNtxNSp 09	P84016	6.99E-13	87.500	PF08116	5238,75	EFEAESGVPEINEER AFCRFNGEQCTSDG QCCNSRCINAFEGRI CMG	CACGAGATTAGCAGAAATATAGGTTAAGGAATATTGGTATA AAGGGCCTAAATTTCGTTATTTAAACAATTACTGCAAATTGTT AAAATGCGGAGTAATATGATGAAAAATACGACAAAACTGCT ACCTTTCAAATGTAATCGAAAAATGTTCTGAGTTTTCAATTCT GTTTTAAAAAAAATTTTGCTATGTTCTTTCCAAGAAATTCAAA TATCATACAAACGTATCTCCGTTCAACGACCTGTCGGTTACG ATGTGAGAAGAAATAGTATCCATATTTCTATTCTAAAATTTTA CTTGTGCCGCCGAATGGCGTGCTGTTAGCTCTGGCTACTTCTC TAGAGCAGGGTCCGGTTTGGACTCCGGATAAGACCTCCATGT TTTATCTTGTCGTTCATACGGGTAGAATTGGTCGTCCATGTGA TGCACCCATAATAAAATGGCCAAAAATTGTGGCAGTAATTGT CTGAGTTCGGATCAATCATTGATTGCTCCAGGTCTGCCATAA AATTGTTTTACTCATTAATATTTGGTTGTAAACGTTATTTCTAA CGCGTTAAATATTATAGCTATCTGGCATCAAGGAGGTACATG CTGTTCTTCACCTAAAATGGAGATTTTTCGTCTTTGGAAAATA ACCCAACCACCAGGTGCTAGCTCTAGTTAGAGTGATACCTGA AGTATGGTAACGAAACATTAAATTTTTGATTTTCGTAATCCAT CTCCCCGAAAATGAGAGCATAGGAATTCGTTGTAACTTAAAC TTGAAATGTTTGAACTAAATGGTTATTAACTCATTTAAATGA AAGAAAGGTCTTTTGGTATTGTTCTTTATCCGGCACTAGCGAT AGCAACAGAGGAAGGACGTTTATGATCAGTCTTCTCCTTAAA GAGACTTAATCAGTAGGTAAGGGTCAACAAGATTTTTTTTAT AAAAGTTTGGAATCCGATGCAAAAGAAACAAATAACACATA CATACGCATATACAAACTTAATAATATCTTTAATTGTCTGAA ATTGGAGCCCTTTCTTAAATTCAGGAATCATATTTTCGACTCG TGAAATTTCTCTTTTTCAGTCTCAGTCATGATTTTCCTGTGGCG CATCAAAGGCAGGTATAGGAATTTCTGGTGATGTTTCACATC AACTTAGAAAGAAATTGCATGTGTCAATCACGAAAAGAAAC

							GAATTGGAGAGGTGGTGGATTTACGTTATTTTTATTACGCGAT<br>TGGCTTGCGGCTACTAATACATATCTCTCTCAGGTTAACCTTT<br>CCCATCTTGATCTCCTGTCAGTGAGTACCATGTTCGTATTCCT<br>TTTAGAAAAGTATCTGGTGCACAAATATAAGTAATTTGTCTTT<br>ACCTTTTATTCAACATGTTGAGTTGAACATGTGTTCTTTTTTCC<br>TCGGGATAGTTCCGACAACGAACAAGTACCTATATCACGAT<br>GTTATTCTCAATCAGATAAAAATCTGCGGACCTTTAACAGTT<br>GGAGCACGATTTTTAAATTGTCTGGAATTCATCCACTTACAA<br>AAGTCCTCCTCACACCTTTATTGTGATAGTCCGAATGCGAGC<br>ATGTAATGCAGGCGTATCGATGGCTTTTGCTCCAAACATTAA<br>CACCTGTAACGTTCAGATTGAAAATGCGTTAAACCATCTAAT<br>ACATTTTAATGAAATACAACCAAATATCGAATGCGTTCTTAC<br>GACTCTTAGCGTTTCACGTCATAATTTTCGAGTATCCAAAGCG<br>AATATGAACGCCCAAAACACTGTAACTGTCTCAAACGTGCC<br>ATGAAATGAAAGAAGACCGTTACATACACATACGAATTTAT<br>TGAAACCCTGCATTTCGTGTATTTATCCCAAATTATTAGGAAA<br>ATAGGTCAAACTAAAAACTTTACGTTAACTTAAAGATATGAA<br>ATTGTGCTTATCGTCTAGACAATTACAAAATTTAGTCATAGTA<br>ATTATTTTCATTACGTGTGTTTGTGAGTGTTATGTATATGATTT<br>TTTAATACCCTGTCACAGATTTTAAGCGGTAATAGAAGTAAT<br>TTTATGTACTTTTACTATCTCTTATTAAGCGAGTCGATGTACA<br>AATCATGGTGTATGTAAATCTTATCACGAAGGGTGTGGTAAC<br>GTCTTCTAAACAACAAATAAATTCTTCCAATGACCCAGATAT<br>TTGCGGTAGGAAAAGCTCTCATAACACAATTGATGAGGGTG<br>GCCGAGAGGCAACAGAAAAGAGTACTACATGAAGTAGTATT<br>ATATGCAAGATTTATAGACTTCAAGCTTTCAAGATTTCAAAT<br>GTTGCTCTCTTTACAAATGCTTGCCAGAGAGATATACGCAAT<br>CAAAAAAAGAGCGAAAATCATCTTTTTCTTCTTAGAGCTTAT

							TTTAAGCTCTAATATCGTTTCAAATCATATTGTTAATTAATTC<br>CTCTGAAGATGAAGGCAGTCACCTTAAATTTTTTGTTATGCCA<br>ATTGTGTTTAAAAGAAGGAAACGAAAAAATCAAAGAGAGA<br>ACAAATAATCATGGAAAAAGAACAAATACAAGGTAATGAA<br>AATAATGTAATTGAATTAAATAATAATGTCATTTAATGAATT<br>GCATTGAGTAACAATGTGGCTTCTAAATTTCGTTGATCCAAA<br>AGTACATCAAATTACAAAAATAAAAGACAAGATATATATCA<br>ACACGCACCATACTTTTCCTCAGGGGAATGAATAGTAGTAAA<br>AGAAAATTCCACTTTCTTCGAAATTTACGCTAATTGTGAACTG<br>AAAATTACCATTTTTCGGCTTAACTGCTTCAAGCAAATTAAA<br>GAACAACCAGAACCTTCTTGCATCATGTTCAAGTGTCAGATC<br>GATGCTGCAAAAACTGAAACCCATCACTTAATATCTTGATAA<br>CTGCGTAAAAAAGCACAACACTTCCCCTGAGTGCCGTCAAA<br>TGAAGGTAGGGAGAATGCTAATGTAAATGTGTAGACAGACA<br>ATCTCTAGGTGCAAGGTCATCAATATATATATAGTAACTATG<br>GATGTGAAGACTTTCTGCACACACACGGCAGAATGGATTGG<br>CTCAACATCGAATGAAGAGTCTTTTGGAGATATAGAGGATGC<br>GGAGAGGTAAATAGGCAGGAGATAATCGGTTGAGATTATGA<br>GACCGCTACAGGTAACGAAATGGTATAGATGTTAGTAGAAT<br>TAGAAATGCAACTCCCAGTGGCAATGGTATATGTGCACATGC<br>CATAATATAAATATGTCACATCGTGTCAAACCTTTCGAACCA<br>TATACGCCAGAAATGAAATGATCATCAGGATCGAAATGATT<br>CAGAAAATAGCCACAACGAAGTTAATGTTTGCACGGAGATT<br>TCTTTACGTTTTATTAAATACTATTTCCTGAAAATAGTGTAGC<br>ACTTTATTACGATAAACTACTGAATTTCAAAATAACTTGCAG<br>AATTTTTTAAAACACAGTTGCAAGAAATCAAATCATTTGCTTT<br>ACAAAATATATTTAATTCATCACAATGTATCAGAAAAATCTG<br>GGAACCCAAATTTTTATACATTAAATACTTAATACAGTAAAT

							TACTTATACATTAAATTACTTATAAATAATCGAATCACGTAA<br>ATAGAACGCAGTAAAAATCAAAATATATTGACTCTGGTACGT<br>GAACTGTTTAATGGTTAGGGACACATGATGTTAGAAGTGTAG<br>TTGGTGAGACTAACTCGACAATTTATGAATTTCATTTTGCAAT<br>GGATTTTCAACTATTATCAATATATACATACAATTTCAAACA<br>GTATTATATGTAATCGTGTCATTTCTTTGCTAAAATAACACTC<br>ATTTCCCGTAATATATCATAATATCTGTAAATAGAATTATTGA<br>TATAATAAACATGCTTTTTTAGAAATTTAATCCTTTATGTTTTC<br>TACACTTCTTTTCTTCTCTAATCTTCTTTTCATGATAGATTATTT<br>CTCATATTTGTTCTTTCTGAAATCGGCCTAAGTTGCTGCTTAA<br>TTTTTGTTTCAGTAATGTTAATTTCTCTTGTTACTTAACTTGCG<br>CATGTAAGCGAATAAAAAATTGAAAAATCTTAATTGCGGTTT<br>TGCTTCTTACAATCAAAATATTTTTAATGTTTTGAAGTAGAAC<br>CAAAATGCTTAAATAAAAGCATTACCTTGTTTGTGCGTCTAG<br>CATTTATGTCTCAGAAACACTAAAAATGTTCTAGTATCTTAA<br>AGATTCATAAAGTTTATATTCATTTACTTAATTTATTGTTTCTA<br>TATTTTCGCAAGCGAAAACGTTATCGGCTACTGCATGCATTC<br>AGTGTAATATGTGAAAGAATGAAATTCTAAATTTAAATACAT<br>AACTTATAATGAATGGAACCTACATTACTCTCATTGTACAGT<br>ATTCTAAAATGCACTCACAAGATATAATCCACAAATGCTAA<br>AGAATCTGCAACTTACTAGAAAATCTTCAAATGCATTACTTT<br>GCATCGTAAATATCACTGTTTCTTTTAAAAAGGTTCTCAAATA<br>TGATCAGAGTAGAGAAATTTTGAAATTGATCGCTTGATATCG<br>ATTCCATTATCAGTTAAAATGTTCGAGTATGGAGTAACTAAA<br>AATTATTTGTTTAATTGATCAGTTTCGCTAACTGCAAATTTAG<br>TTAAAGTGACAAAAAAAAAAGACTATTTACGAAAGAACAA<br>AGAATATGGCACATTGGAAAACTCTATATTTTATGCTTTTTAG<br>TTTGCCAAAATTTTAATTGAAGATAGAAACGAAAAACAGAA

							ATTGAGCAGGGACATTGCATATTTCTTTGCTCTGTATCTCAATTTAAAAGTTTTCTGGTGTCTTAATGTTATTTTTTCTTCATATTTCTTTAAACATGCATGTTTACTGATTGTTAATCAGACACGAAGAAGTATCATTATCAATAAATGAAGTAGAAGAATGAAGTAGTAGTAGAATGAATGATGATTAGAAGTATCATTATCAATTCCATTCGAACCATTGTCATTTTGTGTTTTTAACGTGGTTGTCAATATTATTTTCTGTTTTCCGGTTCCTTGTAAGTAGTGCATTTCTTTCTTGCGTATAGAGATATACTTTTGTAGCAAGGAATTTATTTTTTTTATTGTGCTGATAACAATCAGCAGAAAATACAATTATTTATTAGAGTAGTCACGAGCAGCAAGAAATAATTTCAAAATTTACGCCATTTTCATCAGTATTTCTTTACCCCATGCATATCCTTCCCTCAAACGCATTTATACATCTACTATTGCAGCATTGACCGTCGCTTGTACATTGCTCTCCGTTGAATCTACAAAATGCCCTTTCTTCATTGATTTCTGGAACTCCAGATTCTGCTTCAAATTCTTATGTGAATTCCCTCTTGGAATTTCAGTCCTCCACAGTCTGTATAACGTCACAGCAGGAACTGAATACCGCTGAATCCCTAGAAAATTCATATTCGCTTCTTCTGGGCGCACGAGAGTTATCAAAAGTAGAACGAGAAATATTGAAAGCTTCATGGTTCAACGTATGGAAATCTGTTCACTATAAATCTTCAGAGGTTATCAAAAATGTAACATCTAATTTGCAAAAGTCTTGGTTCTTATATGTATTTAAATGGACTATTACTTCATCATCAAATAGCAATGTGCATGGTAACGTCACTGCAGATGTTGAAATATAAAATACCTGATTGCTCATATGCATATGGTTTGATCACACACATTTCTTAAGAATAGCATTGAAACACTTCAGAGGATAGCAGCAGTCATCGTCGCTTTTACAGGGACGGCTGATGAAACTACAAAGGCCATCAAATAAACTAAACTTTCTTTCTTCATTGATTTCTGAAGCTTTGTCTTCTGCTTCAACAGGTTTTGGAAATCACTTCAGTAAAAATCTTCAGAGATTAGCAGAAATGTAAGATATAA

PhdNtxNSp 10	P17727	4.15E-13	38.000	PF08396	12265.25<br>**10572.14**	MKCAVAFVCLFATVHA**LSVEKEKGLETFLPSARAEKKTCVKIGGECDPYKTQCQCCGDFTYCGCYWEIWFLGQCKCKYGNIATCFEKKTCENRSAWTSKPFNPKDCHTG***	AAAGAGTTTGAAAATTAAACAAGCCGATAAAAGAAGCTGTT<br>GCATTGTCATGGAAGTGTATAAATGGCATGTTTGGCCTTGCA<br>TTTTCATATTCGATTCAAACTTTAGGAAAAGATGAAGTGTGC<br>CGTGGCATTCGTTTGTCTTTTTGCAACTGTACATGCTTTATCTG<br>TTGAAAAAGAGAAAGGACTAGAAACCTTTCTGCCATCAGCC<br>CGAGCTGAAAAAAAAACATGCGTAAAAATTGGAGGCGAAT<br>GTGATCCCTACAAAACACAGTGCCAGTGTTGTGGCGATTTTA<br>CGTACTGTGGGTGTTATTGGGAGATCTGGTTTTTAGGCCAATG<br>CAAATGCAAGTATGGAAATATCGCGACCTGCTTTGAAAAGA<br>AGACCTGTGAAAATCGTTCCGCTTGGACAAGTAAGCCATTTA<br>ATCCAAAGGACTGTCACACTGGCTAATGTTACGAAGCTCAA<br>ATAAAAGATATCAATAACAACTGTATTTCATAAAATTATCAA<br>AAAAGATTATTGTTTTCATACAGTACGTTTTTATTGTTAGGAA<br>AATTCTAAATTCGGAACAAATGCAGTATAGGTCGTTTTGGCG<br>ATGTATTTTCTTTGGCGACATTTGTCGGCGCGATCGACGCTAA<br>CGCATAAAGATGGCGGTGTTTACGCCATCCTGACCGCGATGG<br>CTCGTCTGGAAAACAAAACAATGTAAAGAAAGCCGAAATTT<br>TAAAAAAATTCACATTTTTTATAATTCGAATAGCAAATCGGA<br>AGTTAAAGAAATTTTAGATATACTCATTGCTTGAATTCAGAA<br>AGCAAATTCGAAATTTAAAAAAATATCCAAATCTCTTGAATT<br>CGGATATCAAATCGGAAATTTAAAAAAATTAGATAGACACA<br>TTACTTGAATTCGAAAAACAAATTCGAAATTAAACAAAATAT<br>GCACAATTTTTCAATTCGGATAGCAAATTGGAAATTAAAATT<br>TTAGAAACATTACTTGAATTCGGAAAGCAAATCCGAAATTTA<br>AAAAAAATATCCATATTCTCTGAATTCGGTTATAAAATCAAA<br>AATTAAAAAAATTTAGACATATTACTTGAATTCGAATTTTAAA<br>AAAGGGTTAATGGACGAGCCGTCACTAGTCAATAACAAAGA<br>AGGAAAACAAATATTAATTAGCACTTACTTTATTGTGCTTTTA

							AATTTACGCTCCTATTTCACGGCAAACCCACTCATAGACATC TCATGGATCTTTACTCTCGACCAAATGTACATATTTTTTACAC ACCGGACATTCATATATAGAAGTAATTAATCTCCCTTTCATAT ACTATCTTTCTTACCCATACATTCCAATTTAGAAAAAAAAATT ATATTCAACTTAACAGAAGTCCACAACTGCGGAAATGGGAG CTTAAATCCACGTACCATTGCAACATCCAAACGAAAATGGT AGAAAACCAGTAGACCGGCTTGAATTTCCTTTATAACGGCCG CTGGTCACAAAATAATTTTACGAAATGAAAACCAATAGGAA TTAGGCGCAATCAATACGTAACAAAAGGGTTACCACAAGAG AAAAGAGCGAAATCGAACGAAAATGGTAAAGTTGCGATGCT TGGCGCTTTATCGCCAGTGTAACTTAAGACGTGGCCAAAACG ACCTATACTGCATCTGTACCCTAAATTCATCTAATACAACTGT TTACCAAATTCATTGAATGCTGTAACTATCTGTCGAGAGAAA TAAATCTCTGTGGCATTCGCTTTAAAAATTAATGTCCAAAAA TTACAGAAGTCCGAAA

PhdNtxNSp 11	P17727	3.81E-12	37.079	PF08396	12457,38	MEVYKWHVWPCIFI<br>FDSNFRRLETFLPSA<br>RAEKKTCVKIGGEC<br>DPYKTQCQCCGDFT<br>YCGCYWEIWFLGQC<br>KCKYGNIATCFEKK<br>TCENRSAWTSKPFN<br>PKDCHTG*	AAAGAGTTTGAAAATTAAACAAGCCGATAAAAGAAGCTGTT<br>GCATTGTCATGGAAGTGTATAAATGGCATGTTTGGCCTTGCA<br>TTTTCATATTCGATTCAAACTTTAGGAGACTAGAAACCTTTCT<br>GCCATCAGCCCGAGCTGAAAAAAAAACATGCGTAAAAATTG<br>GAGGCGAATGTGATCCCTACAAAACACAGTGCCAGTGTTGT<br>GGCGATTTTACGTACTGTGGGTGTTATTGGGAGATCTGGTTTT<br>TAGGCCAATGCAAATGCAAGTATGGAAATATCGCGACCTGC<br>TTTGAAAAGAAGACCTGTGAAAATCGTTCCGCTTGGACAAGT<br>AAGCCATTTAATCCAAAGGACTGTCACACTGGCTAATGTTAC<br>GAAGCTCAAATAAAAGATATCAATAACAACTGTATTTCATA<br>AAATTATCAAAAAAAGATTATTGTTTTCATACAGTACGTTTTTA<br>TTGTTAGGAAAATTCTAAATTCGGAACAAATGCAGTATAGGT<br>CGTTTTGGCGATGTATTTTCTTTGGCGACATTTGTCGGCGCGA<br>TCGACGCTAACGCATAAAGATGGCGGTGTTTACGCCATCCTG<br>ACCGCGATGGCTCGTCTGGAAAACAAAACAATGTAAAGAAA<br>GCCGAAATTTTAAAAAAATTCACATTTTTTATAATTCGAATA<br>GCAAATCGGAAGTTAAAGAAATTTTAGATATACTCATTGCTT<br>GAATTCAGAAAGCAAATTCGAAATTTAAAAAAATATCCAAA<br>TCTCTTGAATTCGGATATCAAATCGGAAATTTAAAAAAATTA<br>GATAGACACATTACTTGAATTCGAAAAACAAATTCGAAATT<br>AAACAAAATATGCACAATTTTTCAATTCGGATAGCAAATTGG<br>AAATTAAAATTTTAGAAACATTACTTGAATTCGGAAAGCAAA<br>TCCGAAATTTAAAAAAAATATCCATATTCTCTGAATTCGGTT<br>ATAAAATCAAAAATTAAAAAATTTAGACATATTACTTGAATT<br>CGAATTTTAAAAAAGGGTTAATGGACGAGCCGTCACTAGTC<br>AATAACAAAGAAGGAAAACAAATATTAATTAGCACTTACTT<br>TATTGTGCTTTTAAATTTACGCTCCTATTTCACGGCAAACCCA<br>CTCATAGACATCTCATGGATCTTTACTCTCGACCAAATGTAC

							ATATTTTTTACACACCGGACATTCATATATAGAAGTAATTAAT CTCCCTTTCATATACTATCTTTCTTACCCATACATTCCAATTTA GAAAAAAAAATTATATTCAACTTAACAGAAGTCCACAACTG CGGAAATGGGAGCTTAAATCCACGTACCATTGCAACATCCA AACGAAAATGGTAGAAAACCAGTAGACCGGCTTGAATTTCC TTTATAACGGCCGCTGGTCACAAAATAATTTTACGAAATGAA AACCAATAGGAATTAGGCGCAATCAATACGTAACAAAAGGG TTACCACAAGAGAAAAGAGCGAAATCGAACGAAAATGGTA AAGTTGCGATGCTTGGCGCTTTATCGCCAGTGTAACTTAAGA CGTGGCCAAAACGACCTATACTGCATCTGTACCCTAAATTCA TCTAATACAACTGTTTACCAAATTCATTGAATGCTGTAACTAT CTGTCGAGAGAAATAAATCTCTGTGGCATTCGCTTTAAAAAT TAATGTCCAAAAATTACAGAAGTCCGAAA

PhdNtxNSp 12	P17727	7.40E-26	54.955	PF08396	13201.18<br><br>**11130.57**	MKLLGIFLIASFAFVL<br>SFA**EEIIEGESPLETE**<br>**ETERRGCIEFGKECE**<br>**RGKGDCQCCRDN**<br>**GYCECKWFFGWNC**<br>**HCMVGTTSSSYGIC**<br>**LEKIKCPNRHTWTS**<br>**SKKECKRSCTAKHC**<br>**RG*****	TTTGGAGCATTTTCGTGCTAAAGAGCTGCATTTATAGGGAAA<br>ATTACGAAAATGAAACTTCTGGGGATATTTCTTATAGCAAGC<br>TTTGCTTTCGTACTGAGCTTCGCAGAAGAAATTATAGAAGGG<br>GAAAGTCCTCTTGAAACTGAAGAAACTGAAAGACGTGGCTG<br>CATCGAATTCGGGAAAGAATGCGAGAGGGGTAAAGGCGACT<br>GCCAGTGCTGCAGAGATAATGGATATTGCGAATGTAAGTGGT<br>TTTTCGGTTGGAACTGCCATTGCATGGTCGGCACTACATCATC<br>AAGTTACGGCATATGCCTGGAGAAGATTAAATGCCCGAACA<br>GACATACGTGGACCTCTTCCAAGAAGGAATGCAAAAGATCC<br>TGCACGGCGAAACATTGCAGGGGTTAAAATTAAGCTGTCTG<br>ATTTTGTTCACATGATTTGTATGGTATGTACTAATGCCAACTG<br>AAAACAAGTAACAATAAAAATAGAATAGTATGCACACTTGT<br>ACTTTTTAAGTTTTCTTATACCCGAGGTGAAGATGATGATCTG<br>TGTCTAAAATGTCTTTCGTAAACTCTGAATTCTGCTTCCCGAG<br>AAATTTATGAATGAATCAAGATCTCCTGCATAAGTATCAAGA<br>ATCCTGCTATTGAAAATACTTTCAATGCCGCGCCGTATAAAC<br>AGATAAATATGTCTGTTTACAGTGAGTAATCATAGAAACTGC<br>TGAACCTATTCAGAAAATTCTTTCATAAGTAGAGATCTTTATT<br>TCTCCCCCATGAACACAGAGTTGCATTTTTACAGTATGAAGA<br>GAGTGAGATTTGCAGGAAATATTTAAGAATGATTCGTCTAAG<br>CCGAGTCTTGAAATTTAGACTGCATTATGATACAATATAAAA<br>AAGTAGTCTCCAAGGAATAGTGGCTATCGCGAGTTGTGTTTT<br>TGCCCTGTAAGCTCGGGTTGAAGCTTGAGCAGCATGCGAATA<br>TATCTGTGCCAGC
PhdNtxNSp 13	P17727	3.31E-22	56.338	PF08396	7787,76	VASFAFVLSFAEEMI<br>EGESPLEAQRTERSG<br>CLELGKDCDGSKDN	GTAGCAAGCTTTGCTTTCGTACTCAGCTTCGCGGAAGAAATG<br>ATAGAAGGGGAAAGTCCTCTTGAAGCGCAACGAACCGAAAG<br>AAGTGGCTGCCTAGAACTCGGTAAGGATTGCGACGGGTCTA<br>AAGACAACTGCCAGTGCTGCAGAGATAATGCATTTTGCAGCT

						CQCCRDNAFCSCYN FFGYKFGCKCAVG	GTTATAACTTTTTTGGATACAAATTTGGTTGCAAGTGCGCAGT TGGTAA
PhdNtxNSp 14	P17727	4.11E-08	56.000	PF08396	7433.61	LVASFAFVLRFAEEM IEGESPLEAQGTERSL YLFSCQLAHSYIIGEI SNIFMLEYCIHREM WLV*	ATCGACATCGTATCACAGCTACAATATGCATTATCTCCGTAG CACTTGCAGATGTCCAAATCCAGATCGCATCTGCCACCCAAT TCAGCCGCCTGTTTCAGATTCTTGAGCTTCAACAGAATTTTCT CCAAATGTTCAGTTTCATTGAAGAAAATTCGCTGAAAAGAAA ATATCAGCAATCTAATCAGGCTATTCCATTTCCTCAATAGTCA TCTAATCGCACAATCACTCGACCAGCAATAGTGTTGAGTTGC TATCATACGGGTGTCCATGGACTCTTGCAACAAAAAATAGGG GTTATAGTACACAGTAAGACGAATAATAATTTCCATGGAACC TAGGATCGCAATCCATTCATAGTGCCAGAAACACAGCACGC AAAGCACGTCAGCTGCTGAAACGGCGTTGACGATTATTGTTC TGAAACATTTTTGTTATACATCGTGGGGGCCTCGGCACACTTT CCACCGCCATCCGTCATTTTTTAAACATGTCATTTCTCCAGCG ATCGATTGGATGAGGTGGTCCTGTGTTTTGGACTCCACGCTGC CCGGACCTGAATCCACTGGATTTTTTTTTTTGCTCATATGTCC GCTTTTTCTGAAGCAGTTGCTATACGAGCCACATTTCTCTATG AATACAGTACTCCAACATGAAAATGTTCGAAATCTCTCCAAT TATATATGAATGCGCCAGTTGGCAGGAAAACAAATACAAAC TTCTTTCGGTTCCTTGCGCTTCAAGAGGACTTTCCCCTTCTATC ATTTCTTCCGCGAATCTGAGTACGAAAGCAAAGCTTGCTACC AG

PhdNtxNSp 15	P17727	3.86E-08	56.000	PF08396	7433.61	LVASFAFVLRFAEEMIEGESPLEAQGTERSLYLFSCQLAHSYIIGEISNIFMLEYCIHREMWLV*	AATCAAACAGGTCGTATGTAGAGCGCATCATCACAGCATTTTCGTCCATGGAGAAACAATCCTGTTTCAGATTCTTGAGCTTCAACAGAATTTTCTCCAAATGTTCAGTTTCATTGAAGAAAATTCGCTGAAAAGAAAATATCAGCAATCTAATCAGGCTATTCCATTTCCTCAATAGTCATCTAATCGCACAATCACTCGACCAGCAATAGTGTTGAGTTGCTATCATACGGGTGTCCATGGACTCTTGCAACAAAAAAATAGGGGTTATAGTACACAGTAAGACGAATAATAATTTCCATGGAACCTAGGATCGCAATCCATTCATAGTGCCAGAAACACAGCACGCAAAGCACGTCAGCTGCTGAAACGGCGTTGACGATTATTGTTCTGAAACATTTTTGTTATACATCGTGGGGGCCTCGGCACACTTTCCACCGCCATCCGTCATTTTTTAAACATGTCATTTCTCCAGCGATCGATTGGATGAGGTGGTCCTGTGTTTTGGACTCCACGCTGCCCGGACCTGAATCCACTGGATTTTTTTTTTTGCTCATATGTCCGCTTTTTCTGAAGCAGTTGCTATACGAGCCACATTTCTCTATGAATACAGTACTCCAACATGAAAATGTTCGAAATCTCTCCAATTATATATGAATGCGCCAGTTGGCAGGAAAACAAATACAAACTTCTTTCGGTTCCTTGCGCTTCAAGAGGACTTTCCCCTTCTATCATTTCTTCCGCGAATCTGAGTACGAAAGCAAAGCTTGCTACCAG

PhdNtxNSp 16	P17727	5.36E-07	35.802	PF08396	13455.24	GATKRGKNVSDENLYRTLASAQAKYLFKEFSKIRKIWKCLYSGQECDPDKSHCQCCFDSSVCGCFWGVKAFGSCMCRSGTTASCFGKKDCENRSSWSTKAFDPEDCRRGSGHMRNTRRQ*	AAAAATTTTCTAATTTTAAAAGCAGGTCTGCAGCTGGAGAGAGTTTACGTATTATAATTATATCTTATTACTTTCTTTCTTAAAACATGCGCAATTCGTTAAAGATTTTTCTAAGACATTCGAGCTACTAAGGCGCCACGAAACGTGGAAAGAATGTTTCAGATGAGAATTTATACCGCACTTTGGCCTCCGCGCAAGCAAAGTATCTGTTCAAGGAATTTTCGAAGATTCGTAAAATTTGGAAGTGTCTTTACTCTGGTCAAGAATGTGACCCCGACAAGTCACACTGCCAGTGCTGCTTCGACTCATCTGTCTGTGGATGTTTCTGGGGAGTAAAAGCGTTTGGCAGCTGCATGTGTAGGTCAGGAACGACAGCATCTTGTTTCGGTAAGAAGGACTGCGAAAACCGTTCTTCGTGGTCAACTAAAGCATTTGATCCCGAAGACTGTAGGAGAGGATCAGGTCATATGCGAAACACAAGACGGCAGTGACCGCAGAATTTCCACCGATGATACCTATAATAAGAAGAAAATTTATTTTTTCTAAGAATCGCATTTTCTTCATGCAATGTGCAAAAATCTGTTGTATGGACCACCATTTAAGTTGGAATACCACATATCATGAATCAGTTCACACAGTACCGTTCGATTCATAAATACACGAATTAATTCCAGAAAAAAGCATTCGTGTCAGATTAAAAATCTCAATCAGCAATTTTTCCATTTATTAATAATAAAAAGTATTCTTCAAAAAAAA
PhdNtxNSp 17	P84093	4.25E-06	42.188	PF08396	10875.97	NSNSSILSSRKCLYSGQECDPDKSHCQCCFDSSVCECFWGIKAFGSCVCRSGTTTSCFDKKDCENRSSWSTKAFDPEDCRKEPRYFQQQNPWSSHG*	TAATAGTATGCTGTAATGTTTACGTTATGTTTACCAATGACTACCCTTTCGATTCGCTGTAAAATTCCAACAGCTCTATTCTCTCTTCCAGGAAATGTCTTTACTCTGGTCAAGAATGCGATCCCGATAAGTCACACTGCCAGTGCTGCTTCGACTCATCTGTCTGTGAATGTTTCTGGGGAATAAAAGCGTTTGGCAGCTGTGTGTGTAGGTCAGGAACGACAACATCTTGTTTCGATAAGAAGGACTGCGAAAACCGTTCTTCGTGGTCAACTAAAGCATTTGATCCCGAAGACTGTAGGAAAGAACCGAGATACTTTCAACAACAGAACCCTTGGAGTTCCCATGGATGATACCCATTCATCGTCGAAACTCTGACTTTTTTTAACAAGAAAAAATTGGTTTTTTTAGGAATCGCAGG

							TATATTTCTTCATGCTATGTGCAAAAAATCTTTTGTATGAACC ACCACTGAAGATGGAATACTGCATATCATGAATCAGTTCACA CAATACTGTTCGATTCATAAATACACGAATTAATTCCAGAAA AAAAGCATTCGTGTCAGATTAAAAATCTCAATCAGCAATTTT TCCATTTATTAATAATAAAAAGTATTCTTCAAAAAAAA
PhdNtxNSp 18	P84062	5.53E-06	36.765	PF08396	10782.37 **8622.75**	MWSLLLLIALFAVEEF VPGAA**QSKKCLYSG QECDPDKSHCQCC FDSSVCGCFWGVK AFGSCMCRSGTTA SCFGNVFTLVKNAI PISHTASAASTHLS VNVSGE***	AAAAATTTTCTAATTTTAAAAGCAGGTCTGCAGCTGGAGAGA GTTTACGTATTATAATTATATCTTATTACTTTCTTTCTTAAAAC ATGCGCAATTCGTTAAAGATTTTTCTAAGACATTCGAGCTACT AAGGCGCCACGAAACGTGGAAAGAATGTTTCAGATGAGAAT TTATACCGCACTTTGGCCTCCGCGCAAGCAAAGTATCTGTTC AAGGAATTTTCGAAGATTCGTAAAATTTGGGGAACCATGTGG TCTTTGTTACTCATTGCTCTTTTTGCAGTTGAAGAATTTGTTCC CGGTGCTGCTCAATCAAAGAAGTGTCTTTACTCTGGTCAAGA ATGTGACCCCGACAAGTCACACTGCCAGTGCTGCTTCGACTC ATCTGTCTGTGGATGTTTCTGGGGAGTAAAAGCGTTTGGCAG CTGCATGTGTAGGTCAGGAACGACAGCATCTTGTTTCGGAAA TGTCTTTACTCTGGTCAAGAATGCGATCCCGATAAGTCACAC TGCCAGTGCTGCTTCGACTCATCTGTCTGTGAATGTTTCTGGG GAATAAAAGCGTTTGGCAGCTGTGTGTGTAGGTCAGGAACG ACAACATCTTGTTTCGATAAGAAGGACTGCGAAAACCGTTCT TCGTGGTCAACTAAAGCATTTGATCCCGAAGACTGTAGGAAA GAACCGAGATACTTTCAACAACAGAACCCTTGGAGTTCCCAT GGATGATACCCATTCATCGTCGAAACTCTGACTTTTTTAACA AGAAAAAATTGGTTTTTTAGGAATCGCAGGTATATTTCTTCA

							TGCTATGTGCAAAAAATCTTTTGTATGAACCACCACTGAAGA TGGAATACTGCATATCATGAATCAGTTCACACAATACTGTTC GATTCATAAATACACGAATTAATTCCAGAAAAAAAGCATTC GTGTCAGATTAAAAATCTCAATCAGCAATTTTTCCATTTATTA ATAATAAAAAGTATTCTTCAAAAAAAA
PhdNtxNSp 19	P17727	9.35E-09	32.743	PF08396	11657.26<br><br>**9497.64**	MWSLLLIALFAVEEF VPGAA**QSKKCLYSG QECDPDKSHCQCC FDSSVCGCFWGVK AFGSCMCRSGTTA SCFGKKDCENRSS WSTKAFDPEDCRR GSGHMRNTRRQ***	AAAAATTTTCTAATTTTAAAAGCAGGTCTGCAGCTGGAGAGA GTTTACGTATTATAATTATATCTTATTACTTTCTTTCTTAAAAC ATGCGCAATTCGTTAAAGATTTTTCTAAGACATTCGAGCTACT AAGGCGCCACGAAACGTGGAAAGAATGTTTCAGATGAGAAT TTATACCGCACTTTGGCCTCCGCGCAAGCAAAGTATCTGTTC AAGGAATTTTCGAAGATTCGTAAAATTTGGGGAACCATGTGG TCTTTGTTACTCATTGCTCTTTTTGCAGTTGAAGAATTTGTTCC CGGTGCTGCTCAATCAAAGAAGTGTCTTTACTCTGGTCAAGA ATGTGACCCCGACAAGTCACACTGCCAGTGCTGCTTCGACTC ATCTGTCTGTGGATGTTTCTGGGGAGTAAAAGCGTTTGGCAG CTGCATGTGTAGGTCAGGAACGACAGCATCTTGTTTCGGTAA GAAGGACTGCGAAAACCGTTCTTCGTGGTCAACTAAAGCATT TGATCCCGAAGACTGTAGGAGAGGATCAGGTCATATGCGAA ACACAAGACGGCAGTGACCGCAGAATTTCCACCGATGATAC CTATAATAAGAAGAAAATTTATTTTTTCTAAGAATCGCATTTT

							CTTCATGCAATGTGCAAAAATCTGTTGTATGGACCACCATTT<br>AAGTTGGAATACCACATATCATGAATCAGTTCACACAGTACC<br>GTTCGATTCATAAATACACGAATTAATTCCAGAAAAAAGCAT<br>TCGTGTCAGATTAAAAATCTCAATCAGCAATTTTTCCATTTAT<br>TAATAATAAAAAGTATTCTTCAAAAAAAA
PhdNtxNSp 20	P17727	2.00E-07	32.353	PF08396	12332.87<br><br>**10173.25**	MWSLLLIALFAVEEF<br>VPGAA**QSKKCLYSG**<br>**QECDPDKSHCQCC**<br>**FDSSVCECFWGIKA**<br>**FGSCVCRSGTTTSC**<br>**FDKKDCENRSSWS**<br>**TKAFDPEDCRKEPR**<br>**YFQQQNPWSSHG***	AAAAATTTTCTAATTTTAAAAGCAGGTCTGCAGCTGGAGAGA<br>GTTTACGTATTATAATTATATCTTATTACTTTCTTTCTTAAAAC<br>ATGCGCAATTCGTTAAAGATTTTTCTAAGACATTCGAGCTACT<br>AAGGCGCCACGAAACGTGGAAAGAATGTTTCAGATGAGAAT<br>TTATACCGCACTTTGGCCTCCGCGCAAGCAAAGTATCTGTTC<br>AAGGAATTTTCGAAGATTCGTAAAATTTGGGGAACGATGTGG<br>TCTTTGTTACTCATTGCTCTTTTTGCAGTTGAAGAATTTGTTCC<br>CGGTGCTGCTCAATCAAAGAAATGTCTTTACTCTGGTCAAGA<br>ATGCGATCCCGATAAGTCACACTGCCAGTGCTGCTTCGACTC<br>ATCTGTCTGTGAATGTTTCTGGGGAATAAAAGCGTTTGGCAG<br>CTGTGTGTGTAGGTCAGGAACGACAACATCTTGTTTCGATAA<br>GAAGGACTGCGAAAACCGTTCTTCGTGGTCAACTAAAGCATT<br>TGATCCCGAAGACTGTAGGAAAGAACCGAGATACTTTCAAC<br>AACAGAACCCTTGGAGTTCCCATGGATGATACCCATTCATCG<br>TCGAAACTCTGACTTTTTTTAACAAGAAAAAATTGGTTTTTTT<br>AGGAATCGCAGGTATATTTCTTCATGCTATGTGCAAAAAATC<br>TTTTGTATGAACCACCACTGAAGATGGAATACTGCATATCAT

							GAATCAGTTCACACAATACTGTTCGATTCATAAATACACGAA<br>TTAATTCCAGAAAAAAAGCATTCGTGTCAGATTAAAAATCTC<br>AATCAGCAATTTTTCCATTTATTAATAATAAAAAGTATTCTTC<br>AAAAAAAA
PhdNtxNSp 21	P17727	2.96E-06	36.667	PF08396	13536.14	RICSRCCSIKTEAVTS<br>GSFGREFASIFLTNPS<br>VRKCLYSGQECDPD<br>KSHCQCCFDSSVCE<br>CFWGIKAFGSCVCRS<br>GTTTSCFDKKDCEN<br>RSSWSTKAFDPEDCR<br>KEPRYFQQQNPWSS<br>HG*	AAAAATTTTCTAATTTTAAAAGCAGGTCTGCAGCTGGAGAGA<br>GTTTACGTATTATAATTATATCTTATTACTTTCTTTCTTAAAAC<br>ATGCGCAATTCGTTAAAGATTTTTCTAAGACATTCGAGCTACT<br>AAGGCGCCACGAAACGTGGAAAGAATGTTTCAGATGAGAAT<br>TTATACCGCACTTTGGCCTCCGCGCAAGCAAAGTATCTGTTC<br>AAGGAATTTTCGAAGATTCGTAAAATTTGGGGAACCATGTGG<br>TCTTTGTTACTCATTGCTCTTTTTGCAGTTGAAGAATTTGTTCC<br>CGGTGCTGCTCAATCAAAACTGAAGCTGTGACGTCTGGATCC<br>TTCGGAAGAGAATTCGCCAGCATTTTCTTGACAAACCCTTCA<br>GTCAGGAAATGTCTTTACTCTGGTCAAGAATGCGATCCCGAT<br>AAGTCACACTGCCAGTGCTGCTTCGACTCATCTGTCTGTGAA<br>TGTTTCTGGGGAATAAAAGCGTTTGGCAGCTGTGTGTGTAGG<br>TCAGGAACGACAACATCTTGTTTCGATAAGAAGGACTGCGA<br>AAACCGTTCTTCGTGGTCAACTAAAGCATTTGATCCCGAAGA<br>CTGTAGGAAAGAACCGAGATACTTTCAACAACAGAACCCTT<br>GGAGTTCCCATGGATGATACCCATTCATCGTCGAAACTCTGA<br>CTTTTTTTAACAAGAAAAAATTGGTTTTTTTAGGAATCGCAGG

							TATATTTCTTCATGCTATGTGCAAAAAATCTTTTGTATGAACCACCACTGAAGATGGAATACTGCATATCATGAATCAGTTCACACAATACTGTTCGATTCATAAATACACGAATTAATTCCAGAAAAAAAGCATTCGTGTCAGATTAAAAATCTCAATCAGCAATTTTTCCATTTATTAATAATAAAAAGTATTCTTCAAAAAAAA
PhdNtxNSp 22	P17727	2.60E-07	36.585	PF08396	13859.85	MFQMRIYTALWPPRKQSICSRNFRRFVKFEFVPGAAQSKKCLYSGQECDPDKSHCQCCFDSSVCGCFWGVKAFGSCMCRSGTTASCFGKKDCENRSSWSTKAFDPEDCRRGSGHMRNTRRQ*	AAAAATTTTCTAATTTTAAAAGCAGGTCTGCAGCTGGAGAGAGTTTACGTATTATAATTATATCTTATTACTTTCTTTCTTAAAACATGCGCAATTCGTTAAAGATTTTTCTAAGACATTCGAGCTACTAAGGCGCCACGAAACGTGGAAAGAATGTTTCAGATGAGAATTTATACCGCACTTTGGCCTCCGCGCAAGCAAAGTATCTGTTCAAGGAATTTTCGAAGATTCGTAAAATTTGAATTTGTTCCCGGTGCTGCTCAATCAAAGAAGTGTCTTTACTCTGGTCAAGAATGTGACCCCGACAAGTCACACTGCCAGTGCTGCTTCGACTCATCTGTCTGTGGATGTTTCTGGGGAGTAAAAGCGTTTGGCAGCTGCATGTGTAGGTCAGGAACGACAGCATCTTGTTTCGGTAAGAAGGACTGCGAAAACCGTTCTTCGTGGTCAACTAAAGCATTTGATCCCGAAGACTGTAGGAGAGGATCAGGTCATATGCGAAACACAAGACGGCAGTGACCGCAGAATTTCCACCGATGATACCTATAATAAGAAGAAAATTTATTTTTTCTAAGAATCGCATTTTCTTCATGCAATGTGCAAAAAATCTGTTGTATGGACCACCATTTAAGT

							TGGAATACCACATATCATGAATCAGTTCACACAGTACCGTTCGATTCATAAATACACGAATTAATTCCAGAAAAAAGCATTCGTGTCAGATTAAAAATCTCAATCAGCAATTTTTCCATTTATTAATAATAAAAAGTATTCTTCAAAAAAAA
PhdNtxNSp23	P15969	7.78E-40	56.250	PF08396	12798.55 **10629.87**	MMKYALIFALLFASVYSFA**VEEDQAIEAEDVVPEEARSSLPDGAECDGHGSDCQCYGKWHKCSCPWFWPLRKGKCYCEMAYKHTCITKLSCPNRKEWGLDWRNEESQRSPC***	CCAATGTCGACTGTAAACATCTGCTAGCGGATATTGAGGTTTTGAACAAAAATGACAGTAGCAGACATTTTAGAAGAAAACGTGAATGGCATTTTAATGGAAGTGACATTCTATTGAGTACATGCAACATATTGTAACATAAAGAAAAAATCCCTATTGCATATATATATGTATAGATAGCTTTAAAAATATGCTGATTCAAATACTGTTTCAGGATTTGTGAGATTTTAGAATAAAGGACAATTTCAAGATGATGAAATACGCTTTGATTTTCGCACTCTTATTTGCTTCTGTATACTCCTTTGCAGTTGAAGAGGATCAAGCCATCGAAGCAGAGGACGTAGTTCCAGAGGAAGCACGCTCAAGTTTACCTGACGGAGCAGAGTGCGATGGCCATGGAAGCGATTGCCAGTGTTATGGTAAATGGCATAAGTGTAGCTGTCCATGGTTTTGGCCTTTGAGAAAAGGAAAATGTTACTGTGAGATGGCATATAAACATACTTGCATCACGAAACTATCCTGTCCTAACAGAAAAGAGTGGGGTCTAGACTGGAGGAACGAAGAGTCCCAAAGAAGCCCTTGCTAAATTTGTGAAATTCAGTTCAAAATTATTTTAATAAATTATCACAGCATGTCTTATGTAAAAAAAAAAAAA

PhdNtxNSp 24	P15969	6.80E-28	65.789	PF08396	70952.89	MADFRSDVVKLNN HNYSNWKYKVQLL LMKDNLFRKVIEGE RPIPDGDSNRKLIAD WDAADSKAQAYIGL TVEDDQLIHIRDKTT AKEMWHTLKEYHE KSTLTNKVHLMRTIC SLKLEEGGNIPQHIT RMQELFLKLRDIGEE SLSDKWSVAMLLSSL PRSYDPLITALETRPE KELTFAVVQQKVIAE YERKQHAEGISCNSS EKMMRSVSSKDFKKI CMKCYFCKKQGHM KKDCKKYLSWKESH PNKVNAIECKEHLF TFEIQSDGWILDSGA TRHVTSDRKFFTSLD TSYIDKIEVANGELV DVIGIGTGKLMFKN HKGAVTPVVVSNVL YAPKVVGNLISVRQL NLSNFSVNFSKDFCE ILKDGKQIGLANSVG NLYRLCEPQKIHTVI EHKNNCIHTWHRK	CCACAGTTCATATGGGGTTAATTTCTTTGCTCGAGTAAGAACT CTGTTTTGAATGAAATTAGCAGTCATCACAGCTTCACCCCAA AACTGATTTGGTAAGCCTGCATCAATCAGCATGCAACGTGCC ATTTCCACTAGAGTTCTATTTTTCCTCTCAGCTATGCCATTCTG CTCTGGGGAATAAGTTGCAGTTAATTGAGTCTGGATACCTTC ATTGGCTAAATATTTCCTTAAGTGGGTATTTAGATATTCACCT CCACGATCAGATCGAATAACCTTGACTTTTCTGTCAAACTTAT TTTTCACAAATGCTACATAATGTTTTAAAATGTCAGGAACTTC AGACTTATTTGCCAATAAATATACATGAGTATACCTTGAGAA ATCATCTATAAATGTGACCATATATCGCTTCCCACCTGGTGTC ACAGTTTGCATTGGTCCACAAACATCTGTATGGATCAAGTCT AGTGGGTTGGTTGACTCTCTAGAAGATTCTTTTGGAAATGGT AATCTGGTCATCTTTCCTTTCATGCATGTATCACATGACATTT GGATGCCACAATCAACTATCTTGAAATGATCTAAAAGTCCCT CAGCATGCATTTTCTTTATTGCGTCTGGATCACGATGTCCCAT TTTTCGATGCCATGTATGAATACAGTTGTTCTTATGTTCAATT ACTGTATGGATTTTCTGTGGTTCACATAATCTGTATAAGTTAC CAACTGAGTTAGCCAATCCTATCTGTTTTCCATCTTTGAGAAT TTCACAAAAGTCTTTACTGAAATTGACTGAAAAATTGCTTAA GTTTAGCTGTCGAACTGAAATCAAATTTCCAACAACCTTAGG AGCATAAAGCACATTGGATACAACAACTGGTGTAACTGCTC CTTTATGATTCTTAAACATCAGTTTTCCTGTCCCGATGCCAAT AACATCAACTAGTTCACCATTTGCAACTTCAATTTTATCAATG TATGATGTATCCAAGGATGTGAAAAACTTTCTGTCACTAGTT ACATGTCGTGTAGCTCCAGAATCCAAAATCCAACCATCTGAT TGAATTTCAAATGTAAACAAATGCTCTTTACATTCAATTGCAT TAACTTTATTTGGATGACTTTCTTTCCAACTTAAGTATTTCTTG CAATCCTTTTTCATGTGACCTTGCTTTTTACAAAAATAACATT

						MGHRDPDAIKKMHAEGLLDHFKIVDCGIQMSCDTCMKGKMTRLPFPKESSRESTNPLDLIHTDVCGPMQTVTPGGKRYMVTFIDDFSRYTHVYLLANKSEVPDILKHYVAFVKNKFDRKVKVIRSDRGGEYLNTHLRKYLANEGIQTQLTATYSPEQNGIAERKNRTLVEMARCMLIDAGLPNQFWGEAVMTANFIQNRVLTRAKKLTPYELW	TCATGCAAATTTTCTTAAAATCTTTACTAGAGACTGACCTCATCATCTTTTCTGAAGAGTTACAGGAAATCCCTTCTGCATGTTGTTTACGTTCGTACTCGGCAATGACCTTCTGCTGTACAACGGCAAACGTTAATTCTTTTTCTGGACGTGTTTCCAGTGCTGTAATTAAAGGATCATAGCTTCTAGGCAAACTGCTCAATAGCATCGCAACACTCCATTTATCCGATAACGATTCTTCACCAATATCTCGAAGTTTTAAAAACAACTCTTGCATGCGAGTAATATGCTGTGGAATATTACCGCCTTCTTCGAGCTTCAAAGAACAAATCGTTCGCATTAAATGAACTTTATTCGTTAACGTGCTTTTCTCGTGATATTCTTTAAGAGTATGCCACATTTCCTTAGCTGTGGTTTTATCCCTGATGTGGATAAGCTGATCATCTTCCACGGTAAGTCCGATGTAGGCTTGTGCTTTGCTGTCGGCTGCATCCCAATCGGCGATTAACTTTCTGTTTGAATCTCCATCAGGAATCGGTCTTTCACCTTCAATTACCTTTCTAAACAAGTTATCTTTCATTAAAAGCAGCTGAACTTTATATTTCCAGTTCGAATAATTATGATTATTCAACTTTACGACGTCACTTCGAAAGTCCGCCATTTTTCTACTTTCACCGTTTGTAACGAAAACCGGAAATGAACTGCTTCGCGTGTTGTACTTAAGTTTTAATTTATCTGACTGGGCCCATAACCTGTTGGGCTATTAAAACGGAAGCAACAGTACATAACTAAAACACGACTAAATGAAACAACTATTTAATTGAAAGGAAACCAACGTTACGTTATTACAATTCACACATAACAATACATAATGCAGTACCGGCACAGACACTGAATATTAAGTTGCCAAAACCAAAAAAGAAGTTGTGAAAACGATTAAGATTAAATAACTTCAACATGTTTTTCCTTGGGTTTCACTGCCATGCATTTTGGTTAATTTTCAGAAAAGTTTTTAACGTTAGTAAATATTCCAATCCAATAATATTTTTGTTTTAGAAACTCATATATTTTGACTGTTCCAAAATGAGGAGTATGAGCAACCTTTATACAATTTTTAATGAATTAAAATTATGTCGACTAATTCATTATCCTTTTTAAATTTATGGTCTGTAATGAGCTTTACCATG

							ACTAAATTTATGTGATTATCTAAGAAATATTGTTTGCTAATAT<br>TGAGTGTAAATATTGAGTGCAACATTTGAAACTGCTGTAGCG<br>GATACCTTGCTGGATTTCCCGAAAGAAATGGTCAAAGTTTTA<br>AACTCCGTGAATAACTAGGCTATAATATCCACAGGAAGATA<br>TGTATTTAATACCTTTTTGACACTTTTAAATCGAGATTTTTGA<br>AATTTCACAGAAAAATTTACTTTTATACTTACTTGTATACAGG<br>TGATTCCTCTCTTTACCTGAATTTCCTCCATTTAAAAATTTTCT<br>TTCTTCTTACATGCCAAAAGAGGAACAAAAGGTAGTTTTGGG<br>CTCAATAGGTAATCACCAGGCTCAATGATAATCATCAATCTA<br>TCCATATCTAATATCTTCATCCTAGGAAAATATTTTGTAATTT<br>AAGGGGACCAAAAAATTCTGAGTCTCTAGGCTCTTTTCCAGCA<br>TCCCATAACATATTTGTCATCAAGACCATGAATCAGATTTCTT<br>TATAATTTGGAGTGCTTAAGTTTAAAATAAAAGAATACGAAT<br>TTTAGATAAGTGTTTAAATGCATAAGGTTATGGTGGGGGTTC<br>CACCCCTCGGGGGAAACCCCTGTTTCGGTTGAGAGGGTCGTT<br>AATGGTTACCGCCGCCAAGTCCTCGACTAGCTCAGAATATTC<br>CTTGTTCCAGGCTGAACTCATGACTCTGGAACACTAAAGGTA<br>AGTACAGGCAATAAAGATCAAGTTATGCCAGTGCAAAGCCA<br>AATTTCTTTTATTGTCACCTTGACAATTGTACACGTTAGCAAA<br>CTGATGAAAGATGCACTGGTTCGTCTCCTATTGCAGCTAGAT<br>TAAGATTTTTATCTCCTCGCTTATTTTCAGTATCCTTCAGTATT<br>TCCTACAAGTCCTGTTTTAGCTTTCATTAATCACTTTCATTAAT<br>TTCATAGACCTCCTTTCTTTCGTGCCAAGACTGTAGTTTAAAC<br>GTCCACTTATGCTATCAGTCCCAGTTTGAAAGGGTCTTCAAA<br>AGTTAATTTAAATCTCTCTCATACAGATCTGCAAGTCACTTTA<br>TCACTAACACATTAAAAGAGTTCTTACATAGCAAAGAGATTC<br>TTACCCCTCGTTTGTGTAAATTCCCAGCTATTTTATAATTAGCT<br>TCTTGCATTCATTGAGCCTTTTTCACACTTCATCTGTCAATCAC

							ACTATATCATCTTTTTCCTACGGCACGTGCAGCTCCAACTGGATCGAATGCGTTATTCAGCCTTAGAGTACAGCTCAAGGTCGTTCAATTGCAAGCTGCATAAACTCCCGCCTGAATCTTCACTTCACAACAAAACGGCCTGAAATGCTTTCCCCTTTTGCTGCTAAAATTTATACTTTTGACTGGTATCGGAATTTGCGTAAAATGTTTTTTCTTCTTAAGTTAAAATTCTGTTTCTTTTAATTTTTAAATCCTTTACCTAAATTTTACTTTCAAACACCTGTCTTCTTTTTACAATGGAAAGTTTCAGAATGTATGCCCTCAAGTTTATTTCGTGCCCATGATATTACAAAAATGGTTAACAGTATGCCTTTGAAATATATTACATTTATTTATGCATATTGTATGTTAAAACAAATGTAAAATCTAAAAAAATATTAATCTTTAAAACACTTATTTATAAAAAAACATTTTCATATAAATAATTCCACCAATTTAGTTGAATGTAACTTTAAGAGACAAGAATTTTAGCTGTAAACAAATTTTATTTTGATTAGAAAATGCAAACTGCAGTTGAAGAATGTGTTTACGATATTCATGCTCAAATGAAGTTTCTTAAATCTAGGAAAATAAGATCTTATGATATATTAAAATAACTTACTTTATTTCGTTGTATTTTTTCAAGCTCAAGTTTACCTGACGGAGCAGAGTGCGATGGCCATGGAAGCGATTGCCAGTGTTATGGTAAATGGCATAAGTGTAGCTGTCCATGGTTTTGGCCTTTGAGAAAAGGAAAATGTTACTGTGAGATGGCATATAAACATACTTGCATCACGAAACTATCCTGTCCTAACAGAAAAGAGTGGGGTCTAGACTGGAGGAACGAAGAGTCCCAAAGAAGCCCTTGCTAAATTTGTGAAATTCAGTTCAAAATTATTTTAATAAATTATCACAGCATGTCTTATGTAAAAAAAAAAAAA

PhdNtxNSp 25	Q5Y4U3	1.35E-25	55.102	PF08396	6065,01	KWSYCKCPIWGLFG CSCVIGDSMVCVRK KEQCRNPEVMNTSP GGCFSSRSGNNRG*	GTACGGCTTATTTTTGAGAGAAAAATAACAGATTGACAAATT TTTATTTACTATATATACTAAGTGAAGACTCAATATCGGGACT ATTTCGAAAGTAAAATTTGAGAAATGCTGCCCTACTTGAAGA CGATGTAGCTCCAGAGGAAGCCAGGTCATGTTTCGAAGGTG GTAAGGATTGCAAAAATGATTGCCAGTGCTGTTGAAAGTGG AGCTATTGCAAATGCCCAATCTGGGGTCTATTCGGATGTTCTT GCGTTATCGGGGATTCCATGGTGTGCGTTCGAAAGAAGGAAC AGTGCAGAAATCCAGAAGTAATGAATACATCACCTGGAGGA TGTTTTTCTTCCCGTAGCGGCAATAACAGAGGTTGATGAGCT CAGCATTCAGTATAACACTGTTACCGGAAGCAATATTGATTT TCATTTCGATTTGTGACAGCAAAAATCTTTTAATTGTTTTGGA CTAAATTATGCATCCAGAAATTGAATTAATAAATTATTTGTTT AAAAAAAAAA
PhdNtxNSp 26	P81793	1.11E-34	77.778	PF02819	5423,28	EEARECADVYKECW YPEKPCCKDRACQC NLGMTCKCKATLG DLFGRR*	GTGCTTTCCCTTAGTGCTGGTTCCAAGGACATCTTTGAATGGA CGCGGAGTCCAAACAACCCACTGATTTAAGAAGAAGCTAGA GAATGTGCAGATGTTTATAAGGAGTGCTGGTATCCTGAGAAA CCTTGCTGTAAAGATCGAGCATGCCAATGCAATCTTGGCATG ACCTGCAAGTGCAAAGCAACTCTAGGAGACTTATTTGGAAG ACGATAATGAAACATGAAATACGACAATTTAAAACGGCAGA ATGAAAGTTTAAATATTTTAACAGCTAAGTAATACGACAATA ATGGATTTA
PhdNtxNSp 27	P81793	3.05E-48	87.500	PF02819	5423,28	EEARECADVYKECW YPEKPCCKDRACQC NLGMTCKCKATLG DLFGRR*	CTTTGGCACAGCTTCACACCAGGAACCTCCATAACGTTTTAC AATGTGGCTTAAAATACAAGTGTTTGTGCTTGCTATCACTTCG ATTACGATTGGAATACTGGCTGAACCATATTTAGGTCCAAAC AACCCACTGATTTAAGAAGAAGCTAGAGAATGTGCAGATGT TTATAAGGAGTGCTGGTATCCTGAGAAACCTTGCTGTAAAGA TCGAGCATGCCAATGCAATCTTGGCATGACCTGCAAGTGCAA AGCAACTCTAGGAGACTTATTTGGAAGACGATAATGAAACA

							TGAAATACGACAATTTAAAACGGCAGAATGAAAGTTTAAATATTTTAACAGCTAAGTAATACGACAATAATGGATTTA
PhdNtxNSp28	P84000	2.86E-15	90.909	PF02819	8475.92 **6042.79**	MFRKIISIVLVITLITLIGVHA**EQASNENVEVVQESARSCAEAYKSCDSLTCCNNRTCMCSMVGTNCTCRKSLAEILG***	CTGCCAGATGCACTGAATATAGAAGTTAAAGAAAAGTTTTCGTAGAGCATATATTTATAAATTCAGGAATCACGTATTAGAACTGAACGATCTTTGGGAACATTGAAAAACTAACTTTTCTAAAATCTTTTTACAAGATGATAACAATGAACATGTAGTAGAAGAAGCCAGAGGTTGCAGAGGCATAAAGACGTTTCATCATGTTCCGTAAAATAATCTCGATTGTTTTGGTTATCACCTTAATAACTCTAATTGGAGTACATGCTGAACAAGCTTCAAATGAAAACGTCGAAGTTGTTCAAGAAAGTGCCAGATCATGTGCAGAAGCCTACAAGTCGTGTGACTCTTTAACTTGTTGCAATAATCGTACCTGTATGTGTTCAATGGTTGGGACGAATTGCACATGCAGGAAATCTCTGGCGGAAATACTTGGTTAAAGTTTCATCGTTGAGTCATTTTTTCAGATTACTGAATATTGGCGCAAAATACCTGCAAATTACGACCAAATACGAAATTGTGTAATGTTACAATGATGTAATGTATGAAAATTTAATCCCCCATTTCTTCATATTTTGTGAAACTCAGAATTAATAATCTTATTAGACACAAAGAAAATAAGAATGAACTGTTGTATCCTTGAATCACTTTTGAAATAAAGCAATGTGACGCATATTATGACGTTGTTCTTAATCATCTAAAACCTATAAAATTTCGGAATTTTGCGCAACCGCCGTGATAACAAATGAA

PhdNtxNSp 29	P84000	1.33E-15	90.909	PF02819	8610.03<br><br>**6069.82**	MWLKMQLFVLAIALIALFEVHA**EPESNENVEVVQESARSCAEAYKSCDSLTCCNNRTCMCSMVGTNCTCRKSLAEILG***	AAGACGCTGGGCAATGTGGCTTAAAATGCAATTGTTTGTGCT<br>TGCTATCGCTTTAATAGCGTTGTTTGAAGTCCATGCTGAACCA<br>GAATCAAATGAAAACGTCGAAGTTGTTCAAGAAAGTGCCAG<br>ATCATGTGCAGAAGCCTACAAGTCGTGTGACTCTTTAACTTG<br>TTGCAATAATCGTACCTGTATGTGTTCAATGGTTGGGACGAA<br>TTGCACATGCAGGAAATCTCTGGCGGAAATACTTGGTTAAAG<br>TTTCATCGTTGAGTCATTTTTTCAGATTACTGAATATTGGCGC<br>AAAATACCTGCAAATTACGACCAAATACGAAATTGTGTAAT<br>GTTACAATGATGTAATGTATGAAAATTTAATCCCCCATTTCTT<br>CATATTTTGTGAAACTCAGAATTAATAATCTTATTAGACACA<br>AAGAAAATAAGAATGAACTGTTGTATCCTTGAATCACTTTTG<br>AAATAAAGCAATGTGACGCATATTATGACGTTGTTCTTAATC<br>ATCTAAAACCTATAAAATTTCGGAATTTTGCGCAACCGCCGT<br>GATAACAAATGAA
PhdNtxNSp 30	P84000	1.12E-13	90.625	PF02819	6088,15	HISCINLNCFHLFTCRCAEAYKSCDSLTCCNNRTCMCSMVGTNCTCRKSLAEILG*	TTACATGATTAAAGGGATTTTATATATTATGTAGCTTGAAAA<br>ATAACTACGTTTTACATTTTTAGACTTGTTCATTTTGCTAAATT<br>TTTGTGATAAATAAGTTCAATTTAAACCCATTACGAATAGAC<br>CTCGATCAAAGTGATTTTGTATTGCGTAGATGTTTCTTGGAAC<br>GTTTCAACCGCGTTATGTTATACTACAGCGCATTATGTATTAA<br>CAGCGTATTTCTGTACATATCTTAATATTGGTATTGTACTATA<br>GCAGGCATTAAGATACGTAAAAAGTAATTTTAACAGTGAGG<br>ATAGAAAACAGAGAATTTATATTTTTCACAGCTGGTAAGAGT<br>TCTTAAATGCGAATATAATATTAAAGTGAGTGCTTCATATATT<br>TGCAGCTTATTAATCTTTGATTTACTTGACACATTTCATGTATT<br>AATTTGAATTGCTTTCATTTATTTACTTGCAGATGTGCAGAAG<br>CCTACAAGTCGTGTGACTCTTTAACTTGTTGCAATAATCGTAC<br>CTGTATGTGTTCAATGGTTGGGACGAATTGCACATGCAGGAA<br>ATCTCTGGCGGAAATACTTGGTTAAAGTTTCATCGTTGAGTC

							ATTTTTTCAGATTACTGAATATTGGCGCAAAATACCTGCAAA<br>TTACGACCAAATACGAAATTGTGTAATGTTACAATGATGTAA<br>TGTATGAAAATTTAATCCCCCATTTCTTCATATTTTGTGAAAC<br>TCAGAATTAATAATCTTATTAGACACAAAGAAAATAAGAAT<br>GAACTGTTGTATCCTTGAATCACTTTTGAAATAAAGCAATGT<br>GACGCATATTATGACGTTGTTCTTAATCATCTAAAACCTATA<br>AAATTTCGGAATTTTGCGCAACCGCCGTGATAACAAATGAA
PhdNtxNSp 31	P84000	3.64E-15	90.909	PF02819	8475.92<br><br>**6042.79**	MFRKIISIVLVITLITLI<br>GVHA**EQASNENVE**<br>**VVQESARSCAEAYK**<br>**SCDSLTCCNNRTC**<br>**MCSMVGTNCTCRK**<br>**SLAEILG***	CTGCCAGATGCACTGAATATAGAAGTTAAAGAAAAGTTTTCG<br>TAGAGCATATATTTATAAATTCAGGAATCACGTATTAGAACT<br>GAACGATCTTTGGGAACATTGAAAAACTAACTTTTCTAAAAT<br>CTTTTTACAAGAAGACGCTGGGCAATGTGGCTTAAAATGCAA<br>TTGTTTGTGCTTGCTATCGCTTTAATAGCGTTGTTTGAAGTCCA<br>TGCTGAACCAGAATCAATTGCAGAGGCATAAAGACGTTTCAT<br>CATGTTCCGTAAAATAATCTCGATTGTTTTGGTTATCACCTTA<br>ATAACTCTAATTGGAGTACATGCTGAACAAGCTTCAAATGAA<br>AACGTCGAAGTTGTTCAAGAAAGTGCCAGATCATGTGCAGA<br>AGCCTACAAGTCGTGTGACTCTTTAACTTGTTGCAATAATCGT<br>ACCTGTATGTGTTCAATGGTTGGGACGAATTGCACATGCAGG<br>AAATCTCTGGCGGAAATACTTGGTTAAAGTTTCATCGTTGAG<br>TCATTTTTTCAGATTACTGAATATTGGCGCAAAATACCTGCAA<br>ATTACGACCAAATACGAAATTGTGTAATGTTACAATGATGTA<br>ATGTATGAAAATTTAATCCCCCATTTCTTCATATTTTGTGAAA<br>CTCAGAATTAATAATCTTATTAGACACAAAGAAAATAAGAA

							TGAACTGTTGTATCCTTGAATCACTTTTGAAATAAAGCAATGT GACGCATATTATGACGTTGTTCTTAATCATCTAAAACCTATA AAATTTCGGAATTTTGCGCAACCGCCGTGATAACAAATGAA
PhdNtxNSp 32	P83896	4.50E-20	97.368	PF17492	8744.22 **6995.89**	MKLAILLIVSVLVLA VA**SESVEENGEDYG TAEIMRKAKCADID QPCKSSCDCCETKG ACTCYKSGCVCRM GSFFTAWGKATC***	TAATGGCCTGTCGTTTACGAAATGATATTTTGCAACATTCCTT TTTGGAATGTTAATAAATGCATCAAAAGTAAAAAATATAGCT TGACAATTTAACTGCTAGACAGATAACGTTAGTGCCAGATGT TCTTGAAAACATTATTAAATGGGACATTTGATTCTTTTCTTAC GTAAATAAAAATAGGTATATATAAGAAAGGTCCTTGCAAAC AGAATTTATCAGATATCCAGCGAAATATCTGATATACATCTG ATATATACCAGCGAAATACTGTTGATACAAATCAGATAAGA AAGCATTTTACTTTGAGAATGAAGCTTGCAATCCTCCTCATC GTCTCTGTTTTGGTTCTTGCTGTTGCAAGTGAATCGGTTGAAG AAAATGGGGAAGATTATGGAACTGCAGAAATAATGAGAAA GGCGAAATGTGCTGATATAGACCAGCCTTGCAAAAGTTCTTG CGACTGCTGTGAAACGAAAGGAGCATGTACTTGCTACAAAA GTGGATGCGTATGCAGAATGGGCTCCTTTTTCACGGCCTGGG GAAAAGCTACTTGTTAACTGAGTATTGAGATGGCTTTATGTA TTCCTCGACAGAATAAATATTATTGCAT

PhdNtxNSp 33	O76198	4.24E-27	74.648	PF17492	7322.43	PSAVELSADFDVEELERGACLGQDKYCKETCDCCGPRGQCVCEGPCICRQGYFWIAWYKLFACKK*	ATATCTCCTCACTTCTCTCATTGGCTACAATTAACACATGCTCTATCTCGATAAATGTGACAACTCCTAGAAACAGGACTCTGGATTTTTCAGCTTACTAATCAGACCTCTATATCTTCTCTCCTAAAAATTGCCATTTCTCTTCTACCTAAATTGAATTTATAAATTGTATCCAAGTGTCACACTAAATTATAATTCGTTTTTTTAATACGGATTGTTATATTCCTCTAAATTCCCACTAAGGACATGATGACTGCAAGTTAATGACGGTTGCATCTATTATTTTTAAAATTCGCAAAGACCGTCATTTCTTGAGTCTGTAGGATTAATTATTTGCGATGGAAAACCAGTTTAGTGCCATTACATGCATATTTCCCTGACCGACTTTGATGTAAAATGAAAAACTAAATTTTAAACGTTAAAGAGGTATAGTGTGGATAGAAATGTGCATTACACTACATGAATTTTAATACACTGTTTTTATATATTTTATTTAATGTTTCATTTGCCCACAGAGGAATCACTATTTGATGGTGATTGCAACTTATTAAACTCGAGAATTCGCTAAGAATGTATAGAGGAGATTTATGATTATTACTGCTCGAAATATTTTCTTTATTTTTCTGTCTTTTCTCTGTCCTTAACTTTCGTATCAATACATATAATCACCTCATCACTGAATTAACATGCAGCCATTTTCTAAAATTTGACTCTTAGTGGTAGAGATAGAAAGTTATTTAAATTGCCAGATATTCAGGACAAGACTGAGCTCTGAAGTACGCATTTACATGCCTCAGAATGGGTAAATTTTCCATTATAGACAAAAATGAATGTGAACGCCTACACCTCAAATGCAATAATATTTATTACATCGTGTTATACATATATCCACCTCATTTTTTACACGCGAAAAGTTTATACCATGCTATCCAAAAGTAGCCTTGTCTGCAAATGCACGGTCCTTCGCAAACACATTGTCCTCTCGGACCACAGCAGTCGCAAGTTTCTTTGCAATATTTGTCTTGGCCAAGACATGCACCTCTCTCCAATTCTTCAACATCAAAATCTGCACTAAGCTCCACAGCACTGGGTG

PhdNtxNSp 34	O76198	9.78E-28	76.562	PF17492	10122.87	IENIFKYISDFFVISTKKCLLIIFLLFYTDFAVEESERGACAGQDKPCKETCDCCGERGECVCDLTRCICRQGYFWIAWHKLINCKK*	AATACGAATCAGAATGAAAGTTGCAATCCTCATCCTCTCTATTTTGGTGCTTGCTGCTGCAAGTGAATCCATTGAAGAATACCGTGAAGGTACGTTATTAAATGCAAACTATTATAGCAATACTTTATGTTCCATATTTCATATTAATACGTGTATGATTAACTATTTTTAATAATTATGGTACAACACAGACTTCAGTAGACCTAACGCCATGGAACGGTCCGCAAATGATTGGATCCCGACGGCACCCAGTGCTGTGGAGCGTAGTGCAGGTATAATACAGACTTATCCCTTCGTATAGCATTAAATAATTTACTGTAGAAATTTTTAGAATAAAATTCAATATTATAGCATTAAAAGTTCTGAGATCCAAAAATTTTAAGAAAAAATGCCTATTGATGTGTATAATGTTTAACGATATCCTGTATGAAAATTTTCATCGTTGTAAATCTTCAAATTTCATGCTACTATATTGTTAAGAACAGCACATTATAAAAAAGTAAGGAAAACTGTTTTGCCATATAACTTTTAGTTCACATGTCATTTGTTTTTTAGATGTTATATTTTATTAATCTTAACTTAAAGCTATGGAAAGCGAAAAAATTATTCGAATTTTAGTACCCGGAATGCATGTATCATTTTGTGTCTCGTCGGTGATGAGTTTTACTCAGTTTTTTACTTGTTACAGATTTAATGCAGAATTTTATTTTAATAAATATTAAATTGAGAATATTTTTAAATACATATCTGACTTCTTTGTAATAAGCACAAAGAAATGTCTTTTAATTATTTTTTTGCTTTTTTATACAGATTTTGCTGTTGAAGAATCGGAGAGAGGTGCATGTGCTGGCCAAGACAAGCCCTGCAAAGAAACTTGCGACTGCTGTGGAGAGAGAGGAGAATGTGTTTGCGACTTGACAAGGTGCATATGCAGGCAAGGTTACTTTTGGATAGCATGGCATAAACTTATTAACTGTAAGAAATGATATGGATTTATGTATACCATGATATAATAAATATTGTTGCATTTGA

PhdNtxNSp 35	O76198	1.06E-53	86.316	PF17492	12336.15<br><br>**10715.01**	MKVAILILSILVLAA<br>A**SESIEEYREDFSRP**<br>**NAMERSANDWIPT**<br>**APSAVERSADFAVE**<br>**ELERGACAGQDKP**<br>**CKETCDCCGERGEC**<br>**VCDLTRCICRQGYF**<br>**WIAWHKLINCKK***	ATAAAATTTGCATGTCGGAGAATTTTGATGCCTAAATGATAA<br>CGTCAGGTAAACAGATGTTCTTGAAATTTTATGAAATTGAAT<br>GTTAGATCCTATTTTTATTTCAATAAGAAGATATTAGGGATAT<br>ATAAGAGGACATCCGAACAGCAATTTATCAGATTCCCGTCG<br>GTCTTTGACAATACGAATCAGAATGAAAGTTGCAATCCTCAT<br>CCTCTCTATTTTGGTGCTTGCTGCTGCAAGTGAATCCATTGAA<br>GAATACCGTGAAGACTTCAGTAGACCTAACGCCATGGAACG<br>GTCCGCAAATGATTGGATCCCGACGGCACCCAGTGCTGTGGA<br>GCGTAGTGCAGATTTTGCTGTTGAAGAATTGGAGAGAGGTGC<br>ATGTGCTGGCCAAGACAAGCCCTGCAAAGAAACTTGCGACT<br>GCTGTGGAGAGAGAGGAGAATGTGTTTGCGACTTGACAAGG<br>TGCATATGCAGGCAAGGTTACTTTTGGATAGCATGGCATAAA<br>CTTATTAACTGTAAGAAATGATATGGATTTATGTATACCATG<br>ATATAATAAATATTGTTGCATTTGA
PhdNtxNSp 36	B6DCV2	2.62E-08	31.944	PF10530	9171.76<br><br>**7095.16**	MKSAIACFLFTILTTL<br>CFA**TAPEAQRQCVP**<br>**HGQRCNLIPDDTNI**<br>**CCDGLDCSCYHVYI**<br>**RGLKTDAKRCWCL**<br>**EKGMSGILEHK***	GTCCTTTCATTATTTATGCTCCAAAATACCGGACATTCCTTTTT<br>CTAAACACCAGCATCTCTTTGCGTCGGTTTTCAGCCCTCTTAT<br>ATATACGTGATAACAACTGCAATCCAATCCATCGCAGCATAT<br>ATTCGTATCATCTGGTATTAAGTTGCATCTTTGACCATGAGGC<br>ACGCATTGCCTTTGAGCTTCTGGAGCAGTGGCAAAGCAGAGT<br>GTAGTCAAAATCGTAAATAAAAAACAGGCAATAGCACTCTT<br>CATTTTCGGTTCAGATCTTTTAGGATAATATTTGTGACAACAA<br>CTCTGAAAAGTCTGGTAACATAATGGTTGTCAATCCCCCTTTT

PhdNtxNSp 37	B6DCW0	1.61E-25	59.459	PF10530	8578.2<br><br>**6221.17**	MKFLLCITIVVLAVVSLIEAKA**ESERKCIALETVCTNDTGNCCPGLKCECYQRFVKDVKEGLKCWCLENDVEYKPK***	CGATTATCTAAAAAGGGGTATAAAATCTGATATTCATGTACGCTTCCATCAAGAAAATTTTCATCTTGGTAAATAATTAAGATTATATTTGTTTGCATTCAAGTCCTTTCTTCTCCCAAAGATGAAATTTTTGCTCTGCATTACGATTGTAGTCCTTGCTGTGGTAAGCCTCATTGAAGCTAAGGCAGAAAGTGAAAGGAAATGCATCGCATTAGAAACAGTTTGCACGAATGATACAGGCAACTGCTGCCCTGGCTTGAAATGTGAATGTTATCAGAGATTTGTAAAAGATGTCAAGGAAGGACTAAAATGCTGGTGCCTCGAAAATGATGTTGAATACAAGCCGAAATAACTTAAATCTCAGACCATTGCTTTCTGCCATATGTCCTTCTAGAGACCTGGAAAGCTCCATTTGTTGACAAGTAATTTGAATAAAATAATTAGCATGCATGTCTCAGACTACTTATTATTTCGTGACATTACTTCTCTCTCGCTGCCTCATGAATATAAG
PhdNtxNSp 38	B6DCV6	1.42E-12	42.105	PF10530	9277.95<br><br>**7006.13**	MKIATFCALVIFTVFYCTFA**VPETERQCIKLEERCDLKEGDGAKCCEGLACKCYYPHVGGVKGGKRCWCFEKDIQYVYAGLK***	TACTGTTATCTTTAGGCGCACAATGATTCTTCGTTACATACTGATAAATTACCTTTCCTCTAATGTCATGCCTTCGTTCATATTGCAGTGACTTTTTACTTGTATTGATTTCTACCAGCATCTTCGTGAGGAATTAAATTATTGTGATATTAGCTGAAAAGTTGTGAGTTCAGAATGTTGTATGCATTGCCTTTATTAAGATGCTTATTTACATGGGTTTACGTGTGCATTATCTAGTACATCAAAATCTAACACAAATTTTGCAAGCAAAGAAGGTTTATTAAGTCATTTCAAATCAATAATGAATGGAATTTGCACAGTCATATTTCTTTATTTTAAACCAGCATAAACATATTGGATATCCTTCTCAAAACACCAGCATCTCTTTCCTCCTTTCACACCACCTACATGTGGATAATAACATTTGCAAGCTAGTCCTTCACAGCATTTGGCTCCATCACCTTCTTTCAAGTCACATCTTTCTTCAAGCTTTATGCACTGTCTTTCAGTTTCAGGTACTGCAAAAGTGCAATAGAAAACTGTGAAAATGACTAAAGCGCAGAATGTAGCAATCTTCATTTTCGAATCCTAGTGGCGAAAAACAAAATGTTATGCTACAG

PhdNtxNSp 39	B6DCV2	3.23E-13	43.284	PF10530	8804.42 **6802.89**	MKAAVVCTLLTVILAGCIEA**SPESERQCVHLEKRCDLNAGDKCCPGLACKCYFPVINGVKTEKRCWCFERDVIYTFAGAI***	AAAATATATAAGGCATATGTGGAGGGGCGATCTCGTTTTACAAACAGCATATTCATCGCGCTGTCAGTGTCAACTAACGGTTCTAAATCAACAAAATCCAGTAATGAAGGCCGCTGTAGTTTGCACCTTACTTACAGTGATTCTTGCTGGCTGTATTGAAGCATCACCTGAAAGTGAGAGGCAATGCGTACATCTCGAGAAACGATGTGATTTAAATGCAGGAGATAAATGCTGTCCAGGACTCGCTTGCAAGTGCTATTTTCCTGTTATAAACGGAGTGAAAACAGAAAAGAGATGTTGGTGCTTTGAAAGGGATGTCATTTACACATTTGCCGGTGCGATATAGATGAAAACAGGCTAAAATATGTAATTTCTAGTTATCAATTTTGAATGTAAAAAAATGAATTTCTTTAATAAATTCTTTATGAATTCCTTCAAAAAAAAAA
PhdNtxNSp 40	B6DD12	1.76E-12	40.000	PF10530	8853.53 **6775.82**	MKAAVVCTLLMVILACCIEA**SPESERQCEHLETRCDLNSGAKCCPGLACKCYFPVINGVKTAKRCWCFERDVVYEFAGLI***	TTTGAAACTGAGAGGTATATAAAGAAGCAGGAACCTTCTGAACAGTAGCAAATTCATCGCTCTGTCAGAGTCAACTAACGGTTCTAAATCAAGAAAATCCAGCTATGAAGGCCGCTGTAGTTTGCACCCTACTTATGGTGATTCTTGCTTGCTGCATTGAAGCTTCACCTGAAAGTGAGAGGCAATGCGAACATCTTGAGACAAGATGTGATTTAAATTCAGGCGCTAAATGCTGTCCAGGACTGGCTTGCAAGTGCTATTTTCCTGTTATAAACGGAGTGAAAACGGCAAAGAGATGTTGGTGCTTTGAAAGGGATGTCGTTTACGAATTTGCGGGTCTGATATAGATGGAAACAAGCTAAAATTTGTAATTTCCATCGATAAGTTTTGAGTGTAAAAATGAATTTCTTTAATAAATCTTTAGTATTTATGAATTTCTTTAATAAATCTTTAGTATTTATGAATTTCTTTAATAAATCTCATTTGCATTAAAAAAA

PhdNtxNSp 41	B3EWT0	3.61E-20	52.857	PF10530	10473.17	CGAPIVNSAVMPQY<br>ARVNIRGKNEDQGS<br>CIPKHHECTHQKDN<br>CCKGHLSLQYKCHC<br>YKVINDKGEESKRC<br>ACITPMSHKILEKTA<br>TVVKKLFE*	TCTAAACTTTCCAGTGACAAGTATGATAGATATTCAGTTTAA<br>AAAGGGAAAGAATTACAGCTTTGATTCTGCTACTCTTTCACT<br>ACCAGGTCCGAACTGAGTAAGATCTTACATTTCTCTTTCTGGA<br>GCATTCATTATGAAGTTACTTGGTCTCACGGTTTTGCTATTCA<br>TGGTTGTTCTCGACAACTGTTCTGGTGAAACGAATGAAGATT<br>TCAGAAGTGACGTCAGTCAGAGAAGTGTTCCAACTGATGAC<br>ATAAAAACTTTGCACTTCAGTGAGGAATTGAGGGAGCGACG<br>CTGGTGCGCTGATGTGGTGCTCCCATAGTAAACTCGGCTGTG<br>ATGCCGCAATATGCACGGGTGAATATCCGAGGAAAAAATGA<br>AGACCAAGGAAGCTGCATTCCCAAGCACCACGAATGTACCC<br>ACCAGAAAGATAACTGCTGTAAGGGACACCTGTCTCTCCAGT<br>ACAAATGTCACTGCTATAAGGTGATAAATGACAAAGGAGAA<br>GAGTCGAAACGGTGCGCCTGCATCACGCCCATGAGCCACAA<br>AATACTCGAAAAGACGGCCACAGTCGTAAAGAAACTTTTCG<br>AATGATGATATCTTCCCTTTCAGAAACGAGGTGCGAATAACT<br>GCATTTGCTCATATGATGTAATCTTTGATGCTTTTCAAACAGA<br>ACAATGTTTAATTAAATAATCAAATCTAAAAAAAAAAAAAA<br>AAAAAAAAA
PhdNtxNSp 42	B6DCV0	2.06E-24	38.333	PF10530	13957.06<br>**11808.36**	MKLLGLTVLLFMVV<br>LDNCSG**ETNEDFRS**<br>**DVSQRSVPTDDIKT**<br>**LHFSEELRKNEDQG**<br>**SCIPKHHECTHQKD**<br>**NCCKGHLSLQYKC**<br>**HCYKVINDKGEESK**<br>**RCACITPMSHKILE**<br>**KTATVVKKLFE***	TCTAAACTTTCCAGTGACAAGTATGATAGATATTCAGTTTAA<br>AAAGGGAAAGAATTACAGCTTTGATTCTGCTACTCTTTCACT<br>ACCAGGTCCGAACTGAGTAAGATCTTACATTTCTCTTTCTGGA<br>GCATTCATTATGAAGTTACTTGGTCTCACGGTTTTGCTATTCA<br>TGGTTGTTCTCGACAACTGTTCTGGTGAAACGAATGAAGATT<br>TCAGAAGTGACGTCAGTCAGAGAAGTGTTCCAACTGATGAC<br>ATAAAAACTTTGCACTTCAGTGAGGAATTGAGAAAAAATGA<br>AGACCAAGGAAGCTGCATTCCCAAGCACCACGAATGTACCC<br>ACCAGAAAGATAACTGCTGTAAGGGACACCTGTCTCTCCAGT<br>ACAAATGTCACTGCTATAAGGTGATAAATGACAAAGGAGAA

							GAGTCGAAACGGTGCGCCTGCATCACGCCCATGAGCCACAA<br>AATACTCGAAAAGACGGCCACAGTCGTAAAGAAACTTTTCG<br>AATGATGATATCTTCCCTTTCAGAAACGAGGTGCGAATAACT<br>GCATTTGCTCATATGATGTAATCTTTGATGCTTTTCAAACAGA<br>ACAATGTTTAATTAAATAATCAAATCTAAAAAAAAAAAAAA<br>AAAAAAAAA
PhdNtxNSp 43	B3EWT0	2.02E-19	52.857	PF10530	10224.85	MKNLYFEDNFQCIF<br>RKNEDQGSCIPKHH<br>ECTHQKDNCCKGH<br>LSLQYKCHCYKVIN<br>DKGEESKRCACITP<br>MSHKILEKTATVVK<br>KLFE*	CTCAACTAGTATTATTCTAACTAGTATTATTCTAACTAGTCAA<br>CTAGTATAACCTCAACTAGTATAACCTCAACTAGTATTATTCT<br>AACTAGTATTATTCTAACTAGTCAACTCAACTAGTCAATTAG<br>TAACAACTCAAGATGTTGTGTGACTTTTGACAGAGACTTTTG<br>ACAGAGACTGGTGACAGAGACTGGTGACTTTTGACAGAGCT<br>CGACGAGTGCTATAAAATAAAATGCTCAAACGGAAAACTTC<br>AAAAAAATATATAGTGAGAAAGTGTTGAATTTGGACAAATTT<br>TCGTTAACATATTAAAAATGTCGAAATATCTTCAAACGAACG<br>TGAATGCCTACTGTAAACGTTTATGGAGTGAAATAAGAAGA<br>AATGAGGAATCCAAAGAGGCCTTATGACGACAGCAGGACCC<br>ATGTGAAGATGAGACATCTTAGACAAGTTGATGTGAGGACC<br>AGTCGCCTCACACCTGTCGCTAATTCACCGGATGTGGATGAC<br>GAGAGTAAGACTGAACAGTTGGAATGCAAGGAAATAAATA<br>AAATGGCATAAAATATAACATAACATGCAAGTTCTCGGCGC<br>TAATTTCTTTCTTACCAACTTTGCAAACACCCTGTCCCGACAA<br>TACTTTCCTCCAAAAATCTTATATACTATCTATCAATTTTTTTT<br>TTGTTAAAAAACTTATAAAAATTTTTCACAAACACACGACTA<br>TAATTAACGCAAAATGAAAAATGAAAAATCTCTATTTTGAAG<br>ATAATTTCCAGTGTATTTTCAGAAAAAATGAAGACCAAGGA

							AGCTGCATTCCCAAGCACCACGAATGTACCCACCAGAAAGA<br>TAACTGCTGTAAGGGACACCTGTCTCTCCAGTACAAATGTCA<br>CTGCTATAAGGTGATAAATGACAAAGGAGAAGAGTCGAAAC<br>GGTGCGCCTGCATCACGCCCATGAGCCACAAAATACTCGAA<br>AAGACGGCCACAGTCGTAAAGAAACTTTTCGAATGATGATA<br>TCTTCCCTTTCAGAAACGAGGTGCGAATAACTGCATTTGCTC<br>ATATGATGTAATCTTTGATGCTTTTCAAACAGAACAATGTTTA<br>ATTAAATAATCAAATCTAAAAAAAAAAAAAAAAAAAAAA
PhdNtxNSp 44	B6DCV0	2.06E-24	38.333	PF10530	13957.06<br><br>**11808.36**	MKLLGLTVLLFMVV<br>LDNCSG**ETNEDFRS**<br>**DVSQRSVPTDDIKT**<br>**LHFSEELRKNEDQG**<br>**SCIPKHHECTHQKD**<br>**NCCKGHLSLQYKC**<br>**HCYKVINDKGEESK**<br>**RCACITPMSHKILE**<br>**KTATVVKKLFE*****	TCTAAACTTTCCAGTGACAAGTATGATAGATATTCAGTTTAA<br>AAAGGGAAAGAATTACAGCTTTGATTCTGCTACTCTTTCACT<br>ACCAGGTCCGAACTGAGTAAGATCTTACATTTCTCTTTCTGGA<br>GCATTCATTATGAAGTTACTTGGTCTCACGGTTTTGCTATTCA<br>TGGTTGTTCTCGACAACTGTTCTGGTGAAACGAATGAAGATT<br>TCAGAAGTGACGTCAGTCAGAGAAGTGTTCCAACTGATGAC<br>ATAAAAACTTTGCACTTCAGCGAGGAATTGAGAAAAAATGA<br>AGACCAAGGAAGCTGCATTCCCAAGCACCACGAATGTACCC<br>ACCAGAAAGATAACTGCTGTAAGGGACACCTGTCTCTCCAGT<br>ACAAATGTCACTGCTATAAGGTGATAAATGACAAAGGAGAA<br>GAGTCGAAACGGTGCGCCTGCATCACGCCCATGAGCCACAA<br>AATACTCGAAAAGACGGCCACAGTCGTAAAGAAACTTTTCG<br>AATGATGATATCTTCCCTTTCAGAAACGAGGTGCGAATAACT

							GCATTTGCTCATATGATGTAATCTTTGATGCTTTTCAAACAGAACAATGTTTAATTAAATAATCAAATCTAAAAAAAAAAAAAAAAAAAAAAA

PhdNtxNSp<br>45	B6DCU1	2.41E-21	61.194	PF10530	10378.94	MHIHDVLSPFYRSKK<br>CTERNHECTDDRHS<br>CCRGKLFKDVCVCF<br>YPEGGSNDTARANK<br>ELCTCQQPGHYKTIE<br>KVFDKTKVLVNKAK<br>GLVGL*	GTAATAAAAGTCTTGTAGAGACCGTTGACAGATAAAGTTGA<br>ACTGAAGAGATGGAACAATATTAACTTCAAAACGTCGTTCTT<br>TTATTTGAAGAAACACTAAATTAAATTTACATATTAAATACA<br>AGCTTGCAGCAAAATCTTTAAAAGAACCCAGCTACATGCTG<br>ATGAAATAGAACTGTGAAACAGTCAATTAAATAAATATGCA<br>AAGGATACATTACGTTGACAACAAAAATATATAAAAAAGAA<br>CAACTATATATGAAACACAAAGAATACTGGAACCTGGCTGA<br>TGCCAGATGTCGAAATTTATAAAAAACTAAATAAAATCGTTT<br>GCTGCATCGATTCAAAAAAAGCAGAATTGTTAAATGTTACAA<br>TTTCGGCATATGACGTCAGGGTCTGCAAGGGTTAGTGCAGAC<br>GATTTGTTCATGACCATCAAAGTTCATGATCAACTTTCGCGA<br>GAGCCTATAAGAGGTCGATACAAGATTATGAAATATAATTTA<br>ACGTAGGTGCAAGAAACATTAAAAAAATACTAAAAAAATCG<br>ATGCAAAAACTATTTAAGCATGCATTTAATTGTTTTGTTTTGA<br>AATTCATAAAAATTTTGTTTAGAGAAAACTAAGTTTTTTCTCT<br>TTATTCCTTTTTTCCATAAATTTGCATTAAGTGGTTGATATACC<br>AGGGAAAATCCTATTTAGCTCCTCAAAATGGAAGCATTCAA<br>AGCTTGAAACTTAGGACTCGAAAATATATCACACCCGAACG<br>AGATGCATTATTTATATAGGAGATTTATCGTGACCGAAGAAA<br>AATAACGTACAAACAAGAAAGCATAAAATTAGGGTTCCAGT<br>GAAGCCCGTTTCTGAAAATGCCTCTGCAATGACGGGCGCAC<br>ATTATTTCTTAGGAAATTATTTCGTATATACTACGCAGGAAA<br>AGAAGCGAAAGCTTCTGGCAGGATCTTCTAATCTATGCACAT<br>TCATGATGTGCTTTCCCCATTTTACAGATCCAAGAAATGTACC<br>GAAAGGAACCATGAGTGCACTGACGATCGTCACAGCTGCTG<br>CCGGGGAAAATTGTTCAAAGACGTCTGTGTGTGCTTTTACCC<br>GGAAGGCGGCAGCAACGATACGGCAAGGGCAAACAAGGAA<br>TTGTGCACTTGCCAGCAACCTGGGCATTACAAGACGATCGAA

							AAAGTCTTCGACAAAACCAAAGTATTGGTAAACAAGGCCAA<br>AGGACTCGTTGGCTTATAGGTCGTAGTGTAACATTTGATTTAT<br>ATGCCGAATAAACACAAAAGTTTGTAGCATTTTCCTTGAGTA<br>TGTATTTTATAAACAGACAAAGATCTATGACCTTATATTAAT<br>AACCGCTTGCAAAGTCTTTTCTTGATTGCTAGGTCAAGGCGA<br>AAATACTGTGTATGGGGGGGGAATCTATAATTTGTCAAAAC<br>TGCATTGATTACAGGATTCCAAAAGCGAACGATGCATTATTT<br>CAAACTCAAAATATATATCAAACAGCTTGAGAAGTAAATTG<br>CTAGATGTAATGAATTTGCGTGTCACATAAACAGACAGCGAC<br>TATCCTACGACTCTTTCAGTCCTTTTGCGCGTCTCACTTGCACT<br>AAAATAGAACACGGGTAAAACATAAATCTCGATATTTTTTAA<br>TAAATAAATGGGTTTTGATTTCGCTGTCCAAACAGGTAATTA<br>AAGAAAATACTGAACACTAACCTGGATATCTACTATTGAGC<br>GATATCTCCGAGGTTTTATTTGGAAGAAAAACGATGCGTTAT<br>GAGGGAAATATTTTTTTTATTATGCTGCTCCCATGATAAAGTC<br>GCGTAACAGATTTAGTGTTCATGGGCGAATGCTGCGAAAAA<br>AAAAATAAATAAATCATGTAATACTGGTTTCGTTTTCTTCTGG<br>TTCTTACTTATAGATTTATAATTAATTATCCCCTGTGTGCTCAT<br>CAACTGTGGATAGGGTTTCTTCAAGGCCAGTGGTAATGACAA<br>ATATTCAGTGACCACTGAGCGATTTATTTACAGGTTACAATTC<br>GTAAAGTACAAACACTAGAAGCAACTCACTTTTATAATGGG<br>AAAAAAATACGTTCATGCGTGCATCTTCATCAGTCGTCACTA<br>TAGAAGCAATTGATATGAAAATTATAACTACATAGTGAAAT<br>AATATGTTATTTTTTCATAGTTTCAAGAGCGCGTGACATCACA<br>TGCCCCCTCCAAATGGTTGTCAAGTAGGAGAGAAAGATGAG<br>AGGCTTGCCAAACGGCGAAATTAGTAACAACTCAAGATGTT<br>GTGTGACTTTTGACAGAGACTTTTGACAGAGACTGGTGACAG<br>AGACTGGTGACTAGTCAACTAGTAACAACTCAAGATGTCAA

							CTAGTCAATCTAACTAGTCAACTCAACTAGTATTATTCTAACT AGTATTATTCTAACTAGTCAACT

PhdNtxNSp 46	B6DCU1	2.23E-29	48.276	PF10530	14564.67<br>**12415.96**	MKLLGLTVLLFMVVLDNCSG**ETNEDFRSDVSQRSVPTDDIKTLHFSEELRSKKCTERNHECTDDRHSCCRGKLFKDVCVCFYPEGGSNDTARANKELCTCQQPGHYKTIEKVFDKTKVLVNKAKGLVGL***	TCTAAACTTTCCAGTGACAAGTATGATAGATATTCAGTTTAA<br>AAAGGGAAAGAATTACAGCTTTGATTCTGCTACTCTTTCACT<br>ACCAGGTCCGAACTGAGTAAGATCTTACATTTCTCTTTCTGGA<br>GCATTCATTATGAAGTTACTTGGTCTCACGGTTTTGCTATTCA<br>TGGTTGTTCTCGACAACTGTTCTGGTGAAACGAATGAAGATT<br>TCAGAAGTGACGTCAGTCAGAGAAGTGTTCCAACTGATGAC<br>ATAAAAACTTTGCACTTCAGTGAGGAATTGAGATCCAAGAA<br>ATGTACCGAAAGGAACCATGAGTGCACTGACGATCGTCACA<br>GCTGCTGCCGGGGAAAATTGTTCAAAGACGTCTGTGTGTGCT<br>TTTACCCGGAAGGCGGCAGCAACGATACGGCAAGGGCAAAC<br>AAGGAATTGTGCACTTGCCAGCAACCTGGGCATTACAAGAC<br>GATCGAAAAAGTCTTCGACAAAACCAAAGTATTGGTAAACA<br>AGGCCAAAGGACTCGTTGGCTTATAGGTCGTAGTGTAACATT<br>TGATTTATATGCCGAATAAACACAAAAGTTTGTAGCATTTTC<br>CTTGAGTATGTATTTTATAAACAGACAAAGATCTATGACCTT<br>ATATTAATAACCGCTTGCAAAGTCTTTTCTTGATTGCTAGGTC<br>AAGGCGAAAATACTGTGTATGGGGGGGGAATCTATAATTT<br>GTCAAAACTGCATTGATTACAGGATTCCAAAAGCGAACGAT<br>GCATTATTTCAAACTCAAAATATATATCAAACAGCTTGAGAA<br>GTAAATTGCTAGATGTAATGAATTTGCGTGTCACATAAACAG<br>ACAGCGACTATCCTACGACTCTTTCAGTCCTTTTGCGCGTCTC<br>ACTTGCACTAAAATAGAACACGGGTAAAACATAAATCTCGA<br>TATTTTTTAATAAATAAATGGGTTTTGATTTCGCTGTCCAAAC<br>AGGTAATTAAAGAAAATACTGAACACTAACCTGGATATCTA<br>CTATTGAGCGATATCTCCGAGGTTTTATTTGGAAGAAAAACG<br>ATGCGTTATGAGGGAAATATTTTTTTTATTATGCTGCTCCCAT<br>GATAAAGTCGCGTAACAGATTTAGTGTTCATGGGCGAATGCT<br>GCGAAAAAAAAAATAAATAAATCATGTAATACTGGTTTCGTT

							TTCTTCTGGTTCTTACTTATAGATTTATAATTAATTATCCCCTG TGTGCTCATCAACTGTGGATAGGGTTTCTTCAAGGCCAGTGG TAATGACAAATATTCAGTGACCACTGAGCGATTTATTTACAG GTTACAATTCGTAAAGTACAAACACTAGAAGCAACTCACTTT TATAATGGGAAAAAAATACGTTCATGCGTGCATCTTCATCAG TCGTCACTATAGAAGCAATTGATATGAAAATTATAACTACAT AGTGAAATAATATGTTATTTTTTCATAGTTTCAAGAGCGCGTG ACATCACATGCCCCCTCCAAATGGTTGTCAAGTAGGAGAGA AAGATGAGAGGCTTGCCAAACGGCGAAATTAGTAACAACTC AAGATGTTGTGTGACTTTTGACAGAGACTTTTGACAGAGACT GGTGACAGAGACTGGTGACTAGTCAACTAGTAACAACTCAA GATGTCAACTAGTCAATCTAACTAGTCAACTCAACTAGTATT ATTCTAACTAGTATTATTCTAACTAGTCAACT

PhdNtxNSp 47	B6DCU1	2.38E-21	61.194	PF10530	10378.94	MHIHDVLSPFYRSKKCTERNHECTDDRHSCCRGKLFKDVCVCFYPEGGSNDTARANKELCTCQQPGHYKTIEKVFDKTKVLVNKAKGLVGL*	GTAATAAAAGTCTTGTAGAGACCGTTGACAGATAAAGTTGAACTGAAGAGATGGAACAATATTAACTTCAAAACGTCGTTCTTTTATTTGAAGAAACACTAAATTAAATTTACATATTAAATACAAGCTTGCAGCAAAATCTTTAAAAGAACCCAGCTACATGCTGATGAAATAGAACTGTGAAACAGTCAATTAAATAAATATGCAAAGGATACATTACGTTGACAACAAAAATATATAAAAAAGAACAACTATATATGAAACACAAAGAATACTGGAACCTGGCTGATGCCAGATGTCGAAATTTACAAAAACTAAAAATCGTTTGCTACATCGATACAGAAAAGAATAATGACGTCACAGTCTACAAGAGTTAGTGCAGACGATTAGTTCATGACCATCAAAGTTCATGATCAACTTTCGCGAGAGCCTATAAGAGGTCGATACAAGATTATGAAATATAATTTAACGTAGGTGCAAGAAACATTAAAAAAATACTAAAAAAATCGATGCAAAAACTATTTAAGCATGCATTTAATTGTTTTGTTTTGAAATTCATAAAAATTTTGTTTAGAGAAAACTAAGTTTTTTCTCTTTATTCCTTTTTTCCATAAATTTGCATTAAGTGGTTGATATACCAGGGAAAATCCTATTTAGCTCCTCAAAATGGAAGCATTCAAAGCTTGAAACTTAGGACTCGAAAATATATCACACCCGAACGAGATGCATTATTTATATAGGAGATTTATCGTGACCGAAGAAAAATAACGTACAAACAAGAAAGCATAAAATTAGGGTTCCAGTGAAGCCCGTTTCTGAAAATGCCTCTGCAATGACGGGCGCACATTATTTCTTAGGAAATTATTTCGTATATACTACGCAGGAAAAGAAGCGAAAGCTTCTGGCAGGATCTTCTAATCTATGCACATTCATGATGTGCTTTCCCCATTTTACAGATCCAAGAAATGTACCGAAAGGAACCATGAGTGCACTGACGATCGTCACAGCTGCTGCCGGGGAAAATTGTTCAAAGACGTCTGTGTGTGCTTTTACCCGGAAGGCGGCAGCAACGATACGGCAAGGGCAAACAAGGAATTGTGCACTTGCCAGCAACCTGGGCATTACAAGACGATCGAAAAAGTCTTCGACAAAACCAAAGTATTGGT

							AAACAAGGCCAAAGGACTCGTTGGCTTATAGGTCGTAGTGT<br>AACATTTGATTTATATGCCGAATAAACACAAAAGTTTGTAGC<br>ATTTTCCTTGAGTATGTATTTTATAAACAGACAAAGATCTATG<br>ACCTTATATTAATAACCGCTTGCAAAGTCTTTTCTTGATTGCT<br>AGGTCAAGGCGAAAATACTGTGTATGGGGGGGGGAATCTAT<br>AATTTGTCAAAACTGCATTGATTACAGGATTCCAAAAGCGAA<br>CGATGCATTATTTCAAACTCAAAATATATATCAAACAGCTTG<br>AGAAGTAAATTGCTAGATGTAATGAATTTGCGTGTCACATAA<br>ACAGACAGCGACTATCCTACGACTCTTTCAGTCCTTTTGCGC<br>GTCTCACTTGCACTAAAATAGAACACGGGTAAAACATAAAT<br>CTCGATATTTTTTAATAAATAAATGGGTTTTGATTTCGCTGTC<br>CAAACAGGTAATTAAAGAAAATACTGAACACTAACCTGGAT<br>ATCTACTATTGAGCGATATCTCCGAGGTTTTATTTGGAAGAA<br>AAACGATGCGTTATGAGGGAAATATTTTTTTTATTATGCTGCT<br>CCCATGATAAAGTCGCGTAACAGATTTAGTGTTCATGGGCGA<br>ATGCTGCGAAAAAAAAAATAAATAAATCATGTAATACTGGT<br>TTCGTTTTCTTCTGGTTCTTACTTATAGATTTATAATTAATTAT<br>CCCCTGTGTGCTCATCAACTGTGGATAGGGTTTCTTCAAGGCC<br>AGTGGTAATGACAAATATTCAGTGACCACTGAGCGATTTATT<br>TACAGGTTACAATTCGTAAAGTACAAACACTAGAAGCAACT<br>CACTTTTATAATGGGAAAAAAAATACGTTCATGCGTGCATCTT<br>CATCAGTCGTCACTATAGAAGCAATTGATATGAAAATTATAA<br>CTACATAGTGAAATAATATGTTATTTTTTCATAGTTTCAAGAG<br>CGCGTGACATCACATGCCCCCTCCAAATGGTTGTCAAGTAGG<br>AGAGAAAGATGAGAGGCTTGCCAAACGGCGAAATTAGTAAC<br>AACTCAAGATGTTGTGTGACTTTTGACAGAGACTTTTGACAG<br>AGACTGGTGACAGAGACTGGTGACTAGTCAACTAGTAACAA

							CTCAAGATGTCAACTAGTCAATCTAACTAGTCAACTCAACTA GTATTATTCTAACTAGTATTATTCTAACTAGTCAACT

PhdNtxNSp 48	B6DCV7	9.62E-27	61.972	PF10530	8558.23<br>**6412.50**	MKYLLCITILILAVGS<br>LVEA**KAESERKCIEY<br>EKVCTYNTGNCCPS<br>MKCECYQRFIKGV<br>KETIKCWCIEKDVT<br>YK**	TCTTGGTCAATATTTAAGATTATATTTGTTTGAAGCCCATTAC<br>TTTTCCCAAAGATGAAATATCTGCTCTGCATTACTATCTTAAT<br>CCTTGCTGTGGGAAGCCTCGTTGAAGCTAAGGCAGAGAGTG<br>AAAGAAAATGCATCGAATATGAGAAAGTGTGCACTTATAAT<br>ACAGGCAACTGCTGTCCCAGCATGAAATGTGAATGTTATCAG<br>AGATTTATAAAAGGAGTCAAGGAAACAATAAAATGCTGGTG<br>CATTGAAAAAGATGTTACTTACAAG
PhdNtxNSp 49	P83901	1.29E-07	100.000		3513,94	GNVCYCPFDWFGGS<br>WQPKRRMSKNETC<br>QST*	GCGGGAACGTATGTTATTGCCCATTTGACTGGTTCGGTGGAA<br>GTTGGCAACCTAAGAGGAGAATGTCGAAGAATGAAACATGT<br>CAATCAACATGATGGCTTCCACAATTCTAATCTTGTTTTCTAC<br>TTTCTTAATTTACACTTACTTTTAACTGAGGGTCTTTATGCTTG<br>TAATTGACATGTGGAGAATAAAAGATGAATGGAGACAAGGA<br>ATTTAACTTTTGACAGTAAGGGCTCTGTCACATGTAAAAAAG<br>GAAGATTTGTAATTTATTTCATTTGAATTTCT
PhdNtxNSp 50	Q5Y4U4	5.11E-08	50.000		11304.19<br>**8611.74**	MKYLLKPLVVTVLL<br>LLCYAVMTNA**YVM<br>RDSSLDSYQEPNYE<br>ALRQYLMSTRKRSC<br>IRRGSSCDHRPSDC<br>CFNSSCRCNLWGT<br>NCRCQRAGLFQKW<br>GK***	GTGAAAATAGTTTGCAATCAAAGTGATTGATTTTAAGTTATCT<br>ATAAAAATGTATATTTGTTTTAAATACATGTGTATGAAATGC<br>ATGGAATAAATAATGCTGATAATTTCTTTAAGTTGTAAATGA<br>TATATACATCTAAGTAAGTCATTATTCCTAATGCATATCTTTG<br>CGAAATATTACATAGGAATGCAACGAAGGAGAATCTATAAT<br>ACTTTAATACATGTGGCGTTACAGGGTAGTGTAAAGAAGGTA<br>AGAAGCTGAGATGTGTAGAATGATTTTGAAAGAAATATTCAT<br>GTGTCAAACATAATGCTTATGCATTTTTCATAGAAAATGCAG<br>TCAGTTTAACTTCACATTCATTGAATCTAACAGTTTTTCAGCA<br>CAAGGAACGAGTAATAAATAACAAGCATGTTACCACAGAAT<br>GATAAGAACATCAATATACACAGAAGCAAAATAAAGTATGA<br>TGGTATTTCTGATTGGAAACATTTATAGCCGATGATTATTTTT<br>TCCGTCGGACTTTGAAAACCACATATAACGTAAAAAAGTAA<br>AACTGGGAAAACTGATTCTTTTTTGTCTTCAATTTTTAGCGAG

							TCCATATTCAATGTCGTGTTGAGGGTCATTTCGTTCTCACTTTC<br>CCCACTTCTGGAAGAGCCCGGCTCTCTGGCATCTGCAGTTGG<br>TTCCCCAAAGGTTGCATCTGCAGGAAGAGTTGAAGCAGCAG<br>TCACTCGGTCGGTGATCGCAGCTGCTGCCACGCCGAATGCAG<br>CTCCTTTTCCGTGTCGACATGAGGTACTGGCGAAGAGCTTCA<br>TAGTTCGGTTCCTGGTAGCTGTCCAGCGAACTGTCACGCATT<br>ACGTAGGCATTAGTCATGACAGCATAACACAAGAGCAGGAG<br>CACAGTCACAACCAAAGGCTTCAGTAGATATTTCATTCTTAT<br>ATGAAAACTGAAACTCGGGGTTTGCCTTTCTATTCTAATCCTG<br>GTTGAAGTGGTGAGTTCACTGATGTGCGGATCTTTAAATTCTC<br>CTTACATAGATAAATGGCCACTATTCTTCTTTTAACTACGTTT<br>AATGGGGTGTCA
PhdNtxNSp 51	Q5Y4U4	3.37E-08	50.000		12112.06<br><br>**9419.60**	MKYLLKPLVVTVLL<br>LLCYAVMTNA**YVM**<br>**RDSSLDSYQEPNYE**<br>**ALRQYLMSTRNLN**<br>**NIPNRRSCIRRGSSC**<br>**DHRPSDCCFNSSCR**<br>**CNLWGTNCRCQRA**<br>**GLFQKWGK***	GTGAAAATAGTTTGCAATCAAAGTGATTGATTTTAAGTTATCT<br>ATAAAAATGTATATTTGTTTTAAATACATGTGTATGAAATGC<br>ATGGAATAAATAATGCTGATAATTTCTTTAAGTTGTAAATGA<br>TATATACATCTAAGTAAGTCATTATTCCTAATGCATATCTTTG<br>CGAAATATTACATAGGAATGCAACGAAGGAGAATCTATAAT<br>ACTTTAATACATGTGGCGTTACAGGGTAGTGTAAAGAAGGTA<br>AGAAGCTGAGATGTGTAGAATGATTTTGAAAGAAATATTCAT<br>GTGTCAAACATAATGCTTATGCATTTTTCATAGAAAATGCAG<br>TCAGTTTAACTTCACATTCATTGAATCTAACAGTTTTTCAGCA<br>CAAGGAACGAGTAATAAATAACAAGCATGTTACCACAGAAT<br>GATAAGAACATCAATATACACAGAAGCAAAATAAAGTATGA<br>TGGTATTTCTGATTGGAAACATTTATAGCCGATGATTATTTTT<br>TCCGTCGGACTTTGAAAACCACATATAACGTAAAAAAGTAA<br>AACTGGGAAAACTGATTCTTTTTTGTCTTCAATTTTTAGCGAG

							TCCATATTCAATGTCGTGTTGAGGGTCATTTCGTTCTCACTTTCCCCACTTCTGGAAGAGCCCGGCTCTCTGGCATCTGCAGTTGGTTCCCCAAAGGTTGCATCTGCAGGAAGAGTTGAAGCAGCAGTCACTCGGTCGGTGATCGCAGCTGCTGCCACGCCGAATGCAGCTCCTTCTATTGGGAATGTTGTTAAGATTCCGTGTCGACATGAGGTACTGGCGAAGAGCTTCATAGTTCGGTTCCTGGTAGCTGTCCAGCGAACTGTCACGCATTACGTAGGCATTAGTCATGACAGCATAACACAAGAGCAGGAGCACAGTCACAACCAAAGGCTTCAGTAGATATTTCATTCTTATATGAAAACTGAAACTCGGGGTTTGCCTTTCTATTCTAATCCTGGTTGAAGTGGTGAGTTCACTGATGTGCGGATCTTTAAATTCTCCTTACATAGATAAATGGCCACTATTCTTCTTTTAACTACGTTTAATGGGGTGTCA

PhdNtxNSp 52	Q5Y4U4	2.98E-12	39.56		11839.73<br>**8232.27**	MNIILQESIRTTAMK<br>YLLLATIVVFLFVQA<br>LA**AVPYPPPLDRNL**<br>**SDDYNENVDMYM**<br>**KADKRACIRRGGG**<br>**CDGKPNDCCPNSS**<br>**CRCNLWGTNCRCE**<br>**RAGLFQQWGK*****	GTTGGTTTCGAGTCTGGACGCCTTTTTTGGAGGAGCCTTTTTT<br>AGCAAGCGGCGCAAGCACTGGAGATGGGCATAGCGAAATGT<br>GTCCTATGACGAAACGACAATGCGCCGGGCCACACAGCAGC<br>AGTTGTTCCCGAAATGAGTTAGGAACATGATCAAATTACTGT<br>CCTAATCTCAACACTTAACGCATCTTAATCTAGCACCTTAAA<br>TCAAACTTCTCAGAAGAAAGAAGGCTGAAAATTTATGAATA<br>TCATTTTGCAAGAATCCATCAGAACGACAGCCATGAAATACT<br>TGTTGTTAGCAACTATCGTTGTATTCCTCTTCGTTCAAGCTCTG<br>GCAGCAGTGCCGTACCCACCACCTCTTGATCGCAACCTCTCA<br>GATGACTATAATGAAAATGTGGACATGTACATGAAGGCGGA<br>CAAGCGCGCTTGCATCCGCAGAGGAGGTGGATGTGACGGAA<br>AACCCAATGATTGCTGCCCCAACTCATCCTGCAGATGCAATC<br>TCTGGGGAACCAACTGCAGATGCGAACGTGCAGGCCTTTTCC<br>AGCAGTGGGGCAAATAAGCAAGCACGGGTTAGCACAAGTCT<br>TTTACAGGTGTCAGCACCCAGGATGTTTTTGCTCGATGTACGT<br>GTTTTTATCTGTTTACTTCTCGATGTCTGTAGTACGTCGTCATT<br>GTTTTTCGTGATTTTCAAGCAGTTTGTAAATACTTAATGCATC<br>ATATCATCTGTCTTGGTTTATTGTTGTTTCCGTAGCAGAACGC<br>AGAGTTAGAGTGGTAACGATATGTTAACATTTATATTTTTTTT<br>TCAATAAAGTCTTTCTCCTTTCAGTCTTCAAAGTCGTTTATTA<br>ACTGTTTCTCGTAAATTTGTATACTGTTGTTCAATATAAAAGC<br>AAATTACTCGGAAATTTCAAACTCTATACTGCTATGAAAGAG<br>GCCTGATTTCATACCTAATTAAAAATCTCATGCTTTCGAAGA<br>AGTATTTGTCAAATATAACTTGTGAAAGCAGACCTACATAGT<br>AATTCCAAATTGATAATTATTAATTATCCTTTTAATATTCAAT<br>TATTCCAGGATAATTAAGTTTTGAGCATCTATGCAATCAGCTC<br>GTAGCCTTTATATATATATATATTCATAAACGTGAACATTTTC<br>CGTAATTAAATATTAAGCTTCGTGGCTCAAAAGCATTTCTAA

							GATTGATTAATAAAGATTATTTGCCGGATCATTAGTCTCAAA GAATGTACATTATAGAGCTATATTTTATAATATTCTCTTCCAT TTAAATGATATAGAAATGACTTATATGTAAATGCAATTTTGT ACAACATGAATTTTCGTATGTGTATGTTTAATACTGGAGTACC TTTAAATTATCAATATGGCTAAAATGTTGTAGCTTGCGATATA TTTATGAACTGATATATAGTGTATATATTGGAAAATAGAATG ATATTTTAGGTGAAGGTTTAGAATGAAGAGTTCAGCTAAATG CATTTAAACTTTATATGAAATAGAACGCCAGTCACTAAAAGA AGTTCAAATCTTTCCTTTTGAAAGCACATAGACAACAAAAGC ACATTATTTTTCTTTCTTTCTGCACTGAATTTGCACTTCACACT GTAAATTACAAACATGTGCACTCCTGAAACAAACTAAGTTAT TACCCAACACTTTAGAACAGAATTAATTTCAGACGTATGGAG TGAAAAATTCAGATGGACATTGATTGTAGAAATAACTGAAA GTTCAAGTCCGTGCATAATTTCCAAGGGTGTCTCAAAGAAAG GAAAATAATTCAATAAATATTATTACTTTAAATTTTACGGAT AAATAATTATGCTGTGAATAAATGTATATTAAACAGAAAGG GGAAATATGCTTTCATATGCTGGAATGTTTGTGTTGTAGTTAT AAGTTGTGTATAAAAATATTCATATGAAATAAAATCAGAAGT GTTTTCTAGATATTTGTATGAGTCACAAAAACTTTGGTTGTAC GTAAAAGATAATTCACAAAGGCCTCTGAGTAAAATAGTAAT GCTATAAGAATTAACAATTAATCCATGGAATTTTATAATTAT GATAATACATAGCAGCCGAAAGCAGTAGCTGAAATTTTGAA ATAAAAAATTATTTTGTATTTTTGAACAGCACTTGTACATATT ATTACAGATAATGCTTTCCATTAAAGTCTTCAATGTATGATAT ATTCTACTGTTATTTAACTGCACTGATTTGTATTTGTTGAATTT TGTGCCATTCTGTAACATTTTTCCTTGATTTACCCTAGAATTGT GCCGCTTCGTGACCTGTTGTGTTCTGTTGTCAATAAATTCATTT GCAGTCAGTACTTCATTCAAAA

PhdNtxNSp 53	Q5Y4U4	4.2E-12	39.56		11839.73<br>**8232.27**	MNIILQESIRTTAMKYLLLATIVVFLFVQALA**AVPYPPPLDRNLSDDYNENVDMYMKADKRACIRRGGGCDGKPNDCCPNSSCRCNLWGTNCRCERAGLFQQWGK***	TATTATCATTTATATATAAAGGTTTAATTATATTTCGAATGTATTTTGATAAAACATTTTTCAGATACAATCTGAAGTGCAAACATGGAAAGAAAGTCTTTGAATTTGTATGTTTGTAAATACATCTGTTTTCAATAAAGCATGATTGTAGTACACACTAAATGCGCACATACATATAAAATAAATGATAATAAAATTTTTTCGACGTGTACGAAAATTTCAATTTTTCATAGCAGAGGATAACATTACTTATAAATGACTTTTGATTCTAACTTTACATTCATATATTTATTTGTTTTCTGTGTATTATACTTTACGTAAGACAGAAAAATGAATATTTATAGATACGTTCTTCATAGTTTCCCACAGTGTGAAGTAAAAACAGTCCGTACATTATTATGATATTCGCAGATTGTAAAGCGAAATCTGTCTTTATATACCTATATAACCCCAAAAATATGATTTTGGAGACAGGGTTTTTGAAATGTGATGTCATTCTGATTTAAAATTCGTCTATAAGATAAACAATTCATGAATTGCAAGTACAGTGTAATTAAATGAATAGGAGTATAAACGTTGCCTCATTCATTTTAATAGTAAACATATCAGAGCTAAATTCTTCAGGTTATAAATTTGAATATGATGTTGCCTGACGTCAGATACTTCAGGACATCTACACAGTTTAAAATGCAGACTGCACTAACCTCTATCATCTATCGAAAATCCGGTAGTGTAGTATAATTCTTTTATTTTTTGGCAGTGTTCAACTGATAAAGAAACATTGAGCTTGAAAGATAATTTGACGAACCCCATTTAAATGAACATCACTGAATAAAAATTACATATTTCTGCAATAAAATTCTCATAATGATTATGTACCTCTCTGATAATATTATAATTTAATAATATAAGAAGATTTAACCACATTATCATTGTTATTATAGCCTAATAATATAATTTTTCTTGACTTTTAGGGCATAGCGAAATGTGTCCTATGACGAAACGACAATGCGCCGGGCCACACAGCAGCAGTTGTTCCCGAAATGAGTTAGGAACATGATCAAATTACTGTCCTAATCTCAACACTTAACGCATCTTAATCTAGCACCTTAAATCAAACTTCTCAGAAGAAAGAAGGCTGAAAATTTATGAATATCATTTTGCAAGAATCCATCAGAACGAC

							AGCCATGAAATACTTGTTGTTAGCAACTATCGTTGTATTCCTC TTCGTTCAAGCTCTGGCAGCAGTGCCGTACCCACCACCTCTT GATCGCAACCTCTCAGATGACTATAATGAAAATGTGGACATG TACATGAAGGCGGACAAGCGCGCTTGCATCCGCAGAGGAGG TGGATGTGACGGAAAACCCAATGATTGCTGCCCCAACTCATC CTGCAGATGCAATCTCTGGGGAACCAACTGCAGATGCGAAC GTGCAGGCCTTTTCCAGCAGTGGGGCAAATAAGCAAGCACG GGTTAGCACAAGTCTTTTACAGGTGTCAGCACCCAGGATGTT TTTGCTCGATGTACGTGTTTTTATCTGTTTACTTCTCGATGTCT GTAGTACGTCGTCATTGTTTTTCGTGATTTTCAAGCAGTTTGT AAATACTTAATGCATCATATCATCTGTCTTGGTTTATTGTTGTT TCCGTAGCAGAACGCAGAGTTAGAGTGGTAACGATATGTTA ACATTTATATTTTTTTTTCAATAAAGTCTTTCTCCTTTCAGTCTT CAAAGTCGTTTATTAACTGTTTCTCGTAAATTTGTATACTGTT GTTCAATATAAAAGCAAATTACTCGGAAATTTCAAACTCTAT ACTGCTATGAAAGAGGCCTGATTTCATACCTAATTAAAAATC TCATGCTTTCGAAGAAGTATTTGTCAAATATAACTTGTGAAA GCAGACCTACATAGTAATTCCAAATTGATAATTATTAATTAT CCTTTTAATATTCAATTATTCCAGGATAATTAAGTTTTGAGCA TCTATGCAATCAGCTCGTAGCCTTTATATATATATATATTCAT AAACGTGAACATTTTCCGTAATTAAATATTAAGCTTCGTGGC TCAAAAGCATTTCTAAGATTGATTAATAAAGATTATTTGCCG GATCATTAGTCTCAAAGAATGTACATTATAGAGCTATATTTT ATAATATTCTCTTCCATTTAAATGATATAGAAATGACTTATAT GTAAATGCAATTTTGTACAACATGAATTTTCGTATGTGTATGT TTAATACTGGAGTACCTTTAAATTATCAATATGGCTAAAATG TTGTAGCTTGCGATATATTTATGAACTGATATATAGTGTATAT ATTGGAAAATAGAATGATATTTTAGGTGAAGGTTTAGAATGA

							AGAGTTCAGCTAAATGCATTTAAACTTTATATGAAATAGAACGCCAGTCACTAAAAGAAGTTCAAATCTTTCCTTTTGAAAGCACATAGACAACAAAAGCACATTATTTTTCTTTCTTTCTGCACTGAATTTGCACTTCACACTGTAAATTACAAACATGTGCACTCCTGAAACAAACTAAGTTATTACCCAACACTTTAGAACAGAATTAATTTCAGACGTATGGAGTGAAAAATTCAGATGGACATTGATTGTAGAAATAACTGAAAGTTCAAGTCCGTGCATAATTTCCAAGGGTGTCTCAAAGAAAGGAAAATAATTCAATAAATATTATTACTTTAAATTTTACGGATAAATAATTATGCTGTGAATAAATGTATATTAAACAGAAAGGGGAAATATGCTTTCATATGCTGGAATGTTTGTGTTGTAGTTATAAGTTGTGTATAAAAATATTCATATGAAATAAAATCAGAAGTGTTTTCTAGATATTTGTATGAGTCACAAAAACTTTGGTTGTACGTAAAAGATAATTCACAAAGGCCTCTGAGTAAAATAGTAATGCTATAAGAATTAACAATTAATCCATGGAATTTTATAATTATGATAATACATAGCAGCCGAAAGCAGTAGCTGAAATTTTGAAATAAAAAATTATTTTGTATTTTTGAACAGCACTTGTACATATTATTACAGATAATGCTTTCCATTAAAGTCTTCAATGTATGATATATTCTACTGTTATTTAACTGCACTGATTTGTATTTGTTGAATTTTGTGCCATTCTGTAACATTTTTCCTTGATTTACCCTAGAATTGTGCCGCTTCGTGACCTGTTGTGTTCTGTTGTCAATAAATTCATTTGCAGTCAGTACTTCATTCAAAA

PhdNtxNSp 54	Q5Y4U4	2.72E-12	39.56		11258.02<br><br>**8232.27**	MESIRTTAMKYLLLATIVVFLFVQALA**AVPYPPPLDRNLSDDYNENVDMYMKADKRACIRRGGGCDGKPNDCCPNSSCRCNLWGTNCRCERAGLFQQWGK***	GTTGGTTTCGAGTCTGGACGCCTTTTTTGGAGGAGCCTTTTTT<br>AGCAAGCGGCGCAAGCACTGGAGATGGAATCCATCAGAAC<br>GACAGCCATGAAATACTTGTTGTTAGCAACTATCGTTGTATTC<br>CTCTTCGTTCAAGCTCTGGCAGCAGTGCCGTACCCACCACCT<br>CTTGATCGCAACCTCTCAGATGACTATAATGAAAATGTGGAC<br>ATGTACATGAAGGCGGACAAGCGCGCTTGCATCCGCAGAGG<br>AGGTGGATGTGACGGAAAACCCAATGATTGCTGCCCCAACT<br>CATCCTGCAGATGCAATCTCTGGGGAACCAACTGCAGATGC<br>GAACGTGCAGGCCTTTTCCAGCAGTGGGGCAAATAAGCAAG<br>CACGGGTTAGCACAAGTCTTTTACAGGTGTCAGCACCCAGGA<br>TGTTTTTGCTCGATGTACGTGTTTTTATCTGTTTACTTCTCGAT<br>GTCTGTAGTACGTCGTCATTGTTTTTCGTGATTTTCAAGCAGTT<br>TGTAAATACTTAATGCATCATATCATCTGTCTTGGTTTATTGTT<br>GTTTCCGTAGCAGAACGCAGAGTTAGAGTGGTAACGATATGT<br>TAACATTTATATTTTTTTTTCAATAAAGTCTTTCTCCTTTCAGT<br>CTTCAAAGTCGTTTATTAACTGTTTCTCGTAAATTTGTATACT<br>GTTGTTCAATATAAAAGCAAATTACTCGGAAATTTCAAACTC<br>TATACTGCTATGAAAGAGGCCTGATTTCATACCTAATTAAAA<br>ATCTCATGCTTTCGAAGAAGTATTTGTCAAATATAACTTGTGA<br>AAGCAGACCTACATAGTAATTCCAAATTGATAATTATTAATT<br>ATCCTTTTAATATTCAATTATTCCAGGATAATTAAGTTTTGAG<br>CATCTATGCAATCAGCTCGTAGCCTTTATATATATATATATTC<br>ATAAACGTGAACATTTTCCGTAATTAAATATTAAGCTTCGTG<br>GCTCAAAAGCATTTCTAAGATTGATTAATAAAGATTATTTGC<br>CGGATCATTAGTCTCAAAGAATGTACATTATAGAGCTATATT<br>TTATAATATTCTCTTCCATTTAAATGATATAGAAATGACTTAT<br>ATGTAAATGCAATTTTGTACAACATGAATTTTCGTATGTGTAT<br>GTTTAATACTGGAGTACCTTTAAATTATCAATATGGCTAAAA

							TGTTGTAGCTTGCGATATATTTATGAACTGATATATAGTGTATATATTGGAAAATAGAATGATATTTTAGGTGAAGGTTTAGAATGAAGAGTTCAGCTAAATGCATTTAAACTTTATATGAAATAGAACGCCAGTCACTAAAAGAAGTTCAAATCTTTCCTTTTGAAAGCACATAGACAACAAAAGCACATTATTTTTCTTTCTTTCTGCACTGAATTTGCACTTCACACTGTAAATTACAAACATGTGCACTCCTGAAACAAACTAAGTTATTACCCAACACTTTAGAACAGAATTAATTTCAGACGTATGGAGTGAAAAATTCAGATGGACATTGATTGTAGAAATAACTGAAAGTTCAAGTCCGTGCATAATTTCCAAGGGTGTCTCAAAGAAAGGAAAATAATTCAATAAATATTATTACTTTAAATTTTACGGATAAATAATTATGCTGTGAATAAATGTATATTAAACAGAAAGGGGAAATATGCTTTCATATGCTGGAATGTTTGTGTTGTAGTTATAAGTTGTGTATAAAAATATTCATATGAAATAAAATCAGAAGTGTTTTCTAGATATTTGTATGAGTCACAAAAACTTTGGTTGTACGTAAAAGATAATTCACAAAGGCCTCTGAGTAAAATAGTAATGCTATAAGAATTAACAATTAATCCATGGAATTTTATAATTATGATAATACATAGCAGCCGAAAGCAGTAGCTGAAATTTTGAAATAAAAAATTATTTTGTATTTTTGAACAGCACTTGTACATATTATTACAGATAATGCTTTCCATTAAAGTCTTCAATGTATGATATATTCTACTGTTATTTAACTGCACTGATTTGTATTTGTTGAATTTTGTGCCATTCTGTAACATTTTTCCTTGATTTACCCTAGAATTGTGCCGCTTCGTGACCTGTTGTGTTCTGTTGTCAATAAATTCATTTGCAGTCAGTACTTCATTCAAAA

PhdNtxNSp<br>55	P83893	5.11E-32	85.714		7889,77	KVLATQGEWCGTN<br>ADCGEGQCCTGGSY<br>NRNCQSLADDGKP<br>CQQPNKWNEYKFG<br>CPCKEGLICSVINYC<br>QKDE*	TAAAAAACCTGAAACTTTGCCAGTACACTCAATTGTGATGAC<br>AATAGCTTTTAATATTGTTTTTGCTCAGTTAACTATAATTGATT<br>GTAGTAATTAAAAATAATAAAGAAACATGTGCCGATAGATG<br>GCAGCACAAGACATGACGTGATAAGAACTGGTTACAGAATA<br>CAACATCTATTGCTATGTAAATTATGTTTTATATCTTTTTACAT<br>TGCACATTTCAGAAACAATCCTAGTGTTTTATGACAATATCC<br>ATATAGCACATAATGTAATTTATACATCCAAAAATGGCTATC<br>AAGTTCCAAGAAACGCTTGTAACGGACTTTCAACGAACATTC<br>GACGTTAGAATTTATACGTCGAAAACTAGTGACTTTTGTAAC<br>ATACTCTCTACATCTAGAAGCGGTATTCGGTATGACAGTAAT<br>TGTCGTTAATCCTACAGGTAATTTTGTATATGTGCTGAGGACC<br>GATTCTGCACTTGCAGAATGGGTGCAAAGCTATCCATATTGC<br>CCAAGTATACGACGTCCAAAACGCGGCATTCGACGAGACAC<br>ATAGAAGTGACGGAATCGTTTAGTATTCCACGTGTTTTGCAC<br>TTATCCTGAACGATTTTTCGACGCATATTGTGCTACTGGAGAT<br>ACCACTGCAGTTTTCATTTTCTCCATCATTATTTTGCATTAATT<br>TCTGTCTCGTCTCTCCTTATTGGCTATTTTTCCTACCATATGAG<br>TGACGTGACTAGCCCGGAGGACAGGAACACATAAAAGGGTA<br>AGCAATAAATAAGTAAAGCATGAGTGGAAGACGGTTCCATT<br>CCAGCCGCCGACAAATTTTTGCTAATACTTCACACACCTAGC<br>GATGGCAATCAGTAAGCACTGACTAATATATATGGCAAACG<br>TTTTCAGTAGTCCTAAATTAAATAAGTCGAGATGCAGCCAGA<br>AGGCCATAATTGACTTAGAAATATAGCAGTCGATAGAAAGA<br>TAACCAACTAACAATTTTGTTATAACATCTGGGTTACAATTTC<br>AAAAGACGAATTTTCAGGGGAGAGAAGGAATAATTTTTAGG<br>AAAGCACCCGAAATAGATAGGGATAGAACTATAGAACGTGT<br>TAAGAGAACGTGACACAGAAAACATGTTAATTATAGCCAAT<br>GAAAAGTGAAATAGAAAAGTCACGAGATTTGACGCATTAGT

							TATATAAGATTGAAGTTGCACCCTATTTCGTCAGTTCCTAACG<br>GTTCTACAAAACCGGAGCAAACAATTTAACACAGATTCTAAT<br>TGCCACAATGTTACCTGAAAGTCGTTGAGCGCAAGACAAGG<br>AAACTTGGATTTACAATTAATAGAAAGTACTGTTTTAAGTGA<br>AAATGGAAATTAATTAAATACATAAGTAATACATAATTAAT<br>AACATATATATAACAATTCCGATTTAAAATGTTAATCTCATA<br>CTAAATCATTTTTAAATTTGTGAAACTTGTGAAATTATTAATT<br>TTAATTATTTGAATTATTATTTTTTTTTATTTTGATTCTTAATTT<br>AATAACTGAAATCTTCATGTAACTAACGTAACCTAAATAAAT<br>TACTGCAACTGAATAATAATCACATAATAATAATATATTTAG<br>AGATAATGATTAAAATGTACAAGTGTTTCATACCACTAGTAG<br>TGATTTAATTTCGTTTTTAAAATGTTACATTTTTTTAATTCACT<br>GCCGAGCACAAAAGCACGTGCACCTCCATCATTATTACAAG<br>CAACTCTGGACTAGGATTTTCCTTTATTGGCTCTAGGTACAAA<br>TTATTGTTAACTACGCTGGAGGTGCATGTACTTTTACTTTCCC<br>AAGAACAGCCTTAAGGATGAGGAGATAAGAATACAAGGAT<br>CAGGAATTCTTAACCATAAGGATTTCTCAGTTATGGAAATGC<br>AGTTACTAAAACTAATGTCAATGTAGCAAGGACATATGACA<br>ACCTCTGGTTTGGGTGCTTTTAATAGATTACTAAAGCATCATC<br>AGCAATAAATAAAGAAACTTTTCAAGTATAAACTAAACTTA<br>ATATGATAATAAACAAAAGTAAATGCACTTACGTCTGTTTTT<br>ATTCATCTTTTTGACAATAATTTATAACGCTGCATATGAGCCC<br>TTCCTTGCATGGACAGCCGAATTTATACTCGTTCCACTTGTTC<br>GGTTGCTGGCAGGGTTTTCCATCATCAGCTAGTGACTGGCAG<br>TTTCTGTTATACGATCCTCCAGTGCAGCACTGTCCTTCACCAC<br>AGTCAGCGTTAGTACCGCACCATTCACCCTGGGTGGCCAGTA<br>CCTTTCACATTATAAAAATGTAGCTAAAATTATAATTCACTA

							CACATGAAAAACGATCTGGAAAGGAAAAAAGTGCTTTCAGA CATTGAAAGGATTCTGAGTGTTAAATAATAGAATAAAT

PhdNtxNSp 56	P83893	5.6E-32	85.714		9303.55<br>**7191.94**	MKYFVVTIFLVSLVLATQG**EWCGTNADCGEGQCCTGGSYNRNCQSLADDGKPCQQPNKWNEYKFGCPCKEGLICSVINYCQKDE***	TAAAAAACCTGAAACTTTGCCAGTACACTCAATTGTGATGACAATAGCTTTTAATATTGTTTTTGCTCAGTTAACTATAATTGATTGTAGTAATTAAAAATAATAAAGAAACATGTGCCGATAGATGGCAGCACAAGACATGACGTGATAAGAACTGGTTACAGAATACAACATCTATTGCTATGTAAATTATGTTTTATATCTTTTTACATTGCACATTTCAGAAACAATCCTAGTGTTTTATGACAATATCCATATAGCACATAATGTAATTTATACATCCAAAAATGGCTATCAAGTTCCAAGAAACGCTTGTAACGGACTTTCAACGAACATTCGACGTTAGAATTTATACGTCGAAAACTAGTGACTTTTGTAACATACTCTCTACATCTAGAAGCGGTATTCGGTATGACAGTAATTGTCGTTAATCCTACAGGTAATTTTGTATATGTGCTGAGGACCGATTCTGCACTTGCAGAATGGGTGCAAAGCTATCCATATTGCCCAAGTATACGACGTCCAAAACGCGGCATTCGACGAGACACATAGAAGTGACGGAATCGTTTAGTATTCCACGTGTTTTGCACTTATCCTGAACGATTTTTCGACGCATATTGTGCTACTGGAGATACCACTGCAGTTTTCATTTTCTCCATCATTATTTTGCATTAATTTCTGTCTCGTCTCTCCTTATTGGCTATTTTTCCTACCATATGAGTGACGTGACTAGCCCGGAGGACAGGAACACATAAAAGGGTAAGCAATAAATAAGTAAAGCATGAGTGGAAGACGGTTCCATTCCAGCCGCCGACAAATTTTTGCTAATACTTCACACACCTAGCGATGGCAATCAGTAAGCACTGACTAATATATATGGCAAACGTTTTCAGTAGTCCTAAATTAAATAAGTCGAGATGCAGCCAGAAGGCCATAATTGACTTAGAAATATAGCAGTCGATAGAAAGATAACCAACTAACAATTTTGTTATAACATCTGGGTTACAATTTCAAAAGACGAATTTTCAGGGGAGAGAAGGAATAATTTTTAGGAAAGCACCCGAAATAGATAGGGATAGAACTATAGAACGTGTTAAGAGAACGTGACACAGAAAACATGTTAATTATAGCCAATGAAAAGTGAAATAGAAAAGTCACGAGATTTGACGCATTAGT

							TATATAAGATTGAAGTTGCACCCTATTTCGTCAGTTCCTAACG<br>GTTCTACAAAACCGGAGCAAACAATTTAACACAGATTCTAAT<br>TGCCACAATGTTACCTGAAAGTCGTTGAGCGCAAGACAAGG<br>AAACTTGGATTTACAATTAATAGAAAGTACTGTTTTAAGTGA<br>AAATGGAAATTAATTAAATACATAAGTAATACATAATTAAT<br>AACATATATATAACAATTCCGATTTAAAATGTTAATCTCATA<br>CTAAATCATTTTTAAATTTGTGAAACTTGTGAAATTATTAATT<br>TTAATTATTTGAATTATTATTTTTTTTTATTTTGATTCTTAATTT<br>AATAACTGAAATCTTCATGTAACTAACGTAACCTAAATAAAT<br>TACTGCAACTGAATAATAATCACATAATAATAATATATTTAG<br>AGATAATGATTAAAATGTACAAGTGTTTCATACCACTAGTAG<br>TGATTTAATTTCGTTTTTAAAATGTTACATTTTTTTAATTCACT<br>GCCGAGCACAAAAGCACGTGCACCTCCATCATTATTACAAG<br>CAACTCTGGACTAGGATTTTCCTTTATTGGCTCTAGGTACAAA<br>TTATTGTTAACTACGCTGGAGGTGCATGTACTTTTACTTTCCC<br>AAGAACAGCCTTAAGGATGAGGAGATAAGAATACAAGGAT<br>CAGGAATTCTTAACCATAAGGATTTCTCAGTTATGGAAATGC<br>AGTTACTAAAACTAATGTCAATGTAGCAAGGACATATGACA<br>ACCTCTGGTTTGGGTGCTTTTAATAGATTACTAAAGCATCATC<br>AGCAATAAATAAAGAAACTTTTCAAGTATAAACTAAACTTA<br>ATATGATAATAAACAAAAGTAAATGCACTTACGTCTGTTTTT<br>ATTCATCTTTTTGACAATAATTTATAACGCTGCATATGAGCCC<br>TTCCTTGCATGGACAGCCGAATTTATACTCGTTCCACTTGTTC<br>GGTTGCTGGCAGGGTTTTCCATCATCAGCTAGTGACTGGCAG<br>TTTCTGTTATACGATCCTCCAGTGCAGCACTGTCCTTCACCAC<br>AGTCAGCGTTAGTACCGCACCATTCACCCTGGGTGGCCAGTA<br>CCAAGCTGACCAAAAAGATTGTCACAACAAAGTATTTCATGT<br>TAACCTGTTACGTGTTTCCCAGAAAATATAAGTTGTGCTTAG

							GTAAGCTTCTTTTAAAGCCCATAGCATCTGGAAATGCCTGCT AACGAGAGCTTCACAGATGTTTCCCAAAAGAAAGCAATGTC CATCACATGTTAAAATGATATTAAAAATACCTTAAAATCCAG CTATGATTCACTTCAACATGTGCTTTAACAAAAACAATTATC GATCTTGTTCCATTCTTCATGAAATATCATGGCAAGAAACTG ATTTATGTACAGCTGTGAAAACGCG

PhdNtxNSp 57	P0C2S8	5.95E-48	70		10585.3<br>**8164.26**	MKYTLLFLSVIALVH<br>IFMVEA**EEEAEFDAS<br>VPQERGCKARGDT<br>CEKDCDCCGCFYK<br>CHCPLDWFGGKW<br>HPLGCSCVYGDKYI<br>CEKKKKDCPNV***	TCGTCTACAAACCTTGTTACATCTCTAAAGAATAGCAGGAGC<br>AGTGAAACACAGGTTATTCAAGATGAAGTATACTTTATTGTT<br>TCTCTCTGTCATCGCTTTGGTGCATATTTTCATGGTAGAAGCC<br>GAAGAAGAAGCTGAATTCGATGCTTCAGTACCACAGGAGAG<br>AGGCTGCAAGGCTCGTGGAGATACTTGCGAAAAAGACTGTG<br>ATTGCTGCGGGTGCTTCTACAAATGTCATTGCCCCCTCGACTG<br>GTTCGGTGGAAAGTGGCATCCTCTTGGGTGCTCTTGCGTTTAT<br>GGGGATAAATATATATGCGAGAAGAAGAAGAAAGACTGTCC<br>AAATGTTTAGGCTTTGAGAATAAAAGATTTCAGGAGAAGCCT<br>GAAAATAACTGGACGGAGTTGAAGCATACGTTTGTGAAGAA<br>AAATTGAAAATATATATGTATTTCTGCAGGAAAATGATGTTG<br>TATTAATGATGAATCTATAAAATCAATGATTAAAGTTTATTC<br>ATGTTTTAAAAAAAA
PhdNtxNSp 58	P0C2S8	3.19E-56	95		10267.93<br>**7937.03**	MKYALLFLSVIALVH<br>IFAVEA**EDEPESDAL<br>VPQERGCLDIGKTC<br>KDDCECCGCGNVC<br>YCPFDWFGGSWQP<br>FGCSCAYGLKYVCA<br>HKKKKCPNV**	GGAAAATCGAAATGAGTTCAATTAATTTTCAACCTGATGGGA<br>AAACATGACAACTTTGACATAAGAATATGATTAAATTGGTAT<br>ATATTTGGGGATGGCAAACAAATAGCGATCAGTACACCTTCT<br>ACAAACCTTGAAGCGTTTTGCTACATCTCTAAAGATAGCAGG<br>AGCGGTGAAATACAGGATATTCAAGATGAAGTATGCATTATT<br>GTTTCTCTCTGTCATCGCTTTGGTGCATATTTTTGCAGTAGAA<br>GCCGAAGACGAACCTGAGTCCGACGCTTTAGTACCACAGGA<br>GAGAGGCTGCCTTGATATTGGAAAGACTTGCAAAGATGACT<br>GTGAATGCTGCGGGTGCGGGAACGTATGTTATTGCCCATTTG<br>ACTGGTTCGGTGGAAGTTGGCAACCCTTTGGGTGCTCTTGTGC<br>TTATGGGTTGAAATATGTGTGCGCACATAAGAAGAAAAAAT<br>GTCCAAATGTTTAAGCATGGAGAGTGAAATATTTCAGGAAA<br>AGCCTGAAAATAACTGGACGGAGTTGAAGCATACGTTTGTG<br>AAGAAAAAATTGAAAATATATATGTATTTCTGCAGGAAAATG

							ATGTTGTATTAATGATGAATCTATAAAATCAATGATTAAAGT TTATTCATGTTTTAAAAAAAA

PhdNtxNSp 59	P83895	5.93E-09	55.814		15454.78<br>**12842.54**	MWLRIHFFVFAVAVIAFFIACA**EPDSIHSTEHVEEDRRRSNFCLPELTKSFIMWLRIHLFVFAVAVTAFLVSCAEPDSIDSIELEEEEDRRGCAQAYKSCDSKPCCVVNGYHRTCKCNGFGSNCKCQKTMAERWG***	TAATAATAATTAAGATTACATATCAGGATCTATAGGATATTTCATTCAGCTAGGAATGAATGCAGCCACACAGCAACAATCAGATTGTAAAATAAATATATGTTGATAATTTGATGATGTTTTCATATCCCGTGCATCAAATATCAACATAGGAGCTAGCTTTTTCCGCACAAATAAACACAAAATAATTCGGGAAATATTTGTCGCGGGATGAAAAGGTGAGGGAAAAAGGAAACACCTAAAAGAGTGTGACCTTGAAAAAAAATAATATGAGGTGAAACAAGATAAAAGATTTCTTCTTACAGCCTGAAGCATACAAAAAATCCAATGCGAAATGAAGAGTCTGTATGAGAATGTATAAACATGATGTGTAGCGTAAGTGGTGCTATCAGTTAGAGTATCGAGTTAACAAAGGTGTTTAGTATATGCGGATTTGAGTTTATTGACAATAATTCCTTGAAAGATTTGATATATGTACGTATGTATCTATATAACGTACTATGAGGTTATTTTGTTTGCGCATCTAAGAAAATCTGAAGTAATTTACGTTGCTTTTGCGAAACAAAGAATTAAAACTTGAGTTCACTATATGAATCCAGAGAAGAGTGCGTGTCCATTTCACGAAGAGCTGGGAAAAGGAATGAAACAGGGAAGTCGACTAGAATGAAGGAAAACCAAATGTAGATGAATCAAAGTGAGGAGTATTAAGAATTGATAATTTGTGTTCTTTTACTTCACAGTTAAGAACTATAAACCAGTGGCTGTATAAAATTTTGCCATTTCTTCTCAATAAAATTCGAATACATTTTGGTATAAAATTTACGATGAAACCCTAATATCTGTAATATAGAAAGGCTAGATAAGAAACATATTAACCTAAAAATTTTATTTCCGACGGGAATAACATATTTATCACGAACAATTAATTCCTTAGACTGAGAATAACATAGGGCTGATGTTTCAAGATCGAAGCATGGTCTTGTTATACGATCAGGAACAAATTCTTTCTCGAGGACAAGTGGTTTTTGTTTCATATCTGGCTCTTGTATTTTAAATCTCTGGTAAAATGTGAGGAACTCAATGAAATCATTATATTATCTATAATGTCAGTCATGAAGAACTTTTAATTTTAGAAGAATGTATTGCTGCGATAGATAGTATCAGAGTTAGTTT

							TCTTGTCCTCTGACACTGAATCTTTCAAACCTCAGCAATTTTG<br>TGGTTATCAAGATGGGTTGTTATGTAATTCTTTATTTGAAACT<br>GAAAATTTTACATTCTTTATATTTACTTAACTTTACAAATTAC<br>AGAAAAAGATTCAGACTTCTTGTATTTTCATCACATTTGTGCA<br>GCAATAATTAACATCTGTTATTTTGTTACAAAATACAAATATT<br>TCGGATTATGTCAGTATTCTCTAGTATTCATAGTTGAAAACAT<br>ATTTTATCCCCAACGTTCGGCCATCGTTTTTTGGCATTTGCAA<br>TTACTTCCAAATCCATTACATTTGCAGGTTCTGTGATAGCCGT<br>TCACAACACAACAAGGTTTTGAGTCGCATGATTTATAGGCTT<br>GTGCACATCCTCTTCTGTCTTCTTCTTCTTCAAGTTCAATACTG<br>TCAATTGAGTCTGGTTCAGCACATGATACAAGAAAGGCAGTT<br>ACAGCGACAGCAAACACGAACAAGTGTATTCTAAGCCACAT<br>TATGAACGACTTCGTTAACTCTGGAAGGCAAAAGTTGCTTCT<br>TCTCCTGTCTTCTTCTACATGTTCAGTGCTGTGAATTGAGTCTG<br>GTTCAGCACATGCTATGAAAAAGGCAATTACAGCTACAGCA<br>AACACGAAGAAGTGTATTCTAAGCCACATTATGAAAGACTT<br>CGATAACTCTGCAAGGCAAAAATCCCTTCGACGGCGTCTGTA<br>AAGGAGTGATGTTTGCAAAGATCTTTCAACATTACTGCGCGG<br>TTGCTGTATTCTCAAATATATACTTCCCCTAAATTATTTCCTTT<br>CTAATATTCAATCCATATTGCAAAATATTGCGAAAATGCTTA<br>TTTCAATTATGAAT

PhdNtxNSp 60	P83895	6.11E-10	60.465		8621,82	MLKDLCKHHSFTDAVEVHSTEHVEEDRRRRGCAQAYKSCDSKPCCVVNGYHRTCQCNWFGSNCKCKKTLRELWG*	CTATTATTCTACTAGCTGTTGCTATTATTCTACTAGCTGCTACTATTACTCTACTAGCTGCTACTATTATTCTATTAGCTACTACTAGTCCGAAACTAGTAGCAGCTGTTCGGCGTCATCGTGGAGATTTGGAATAACTGTTTCTACATCTTATATTTTCCACAAGTCATCTGTGAGAATCAAAGTGAGGTCTGTTAAGAGTTGATGACTTGTGTTCTTCTACTCCATTGTTATATGGTATAAAACGAGCATGATCTTATTACATAAGCTGAAAAAACTTACTCGTGAACAGATGGTTTTTTTTGTTTATTATTGTTTGTTGAATTTTAAATCTCTCTTAAAATGGGAATGGAAAAGAAATCGCTATATTATCTATTACTGAAACTGTCATTCGCAAAAAACTTTTAATTTTATAAAAATGTATTGCTGCAATAGATATCAACAGTTAGTTTCCTTGTCCTCCAGTAAATTATCTGAAACTAAATCTTTCGCTTTATAGCAAATTTTAGTTATTAAGTAGGGTTGTTATGTAGTTCTTTATTTGAAATTGAAAACGAATGGAGGAGTAATATTTTACATTCATTACATTTAGTTAAATTTACTAATTACAGATATAGATACTCTCTTTTTATAGTTTCATCACTTTTGTGCAACATTATTTAATGTCTGTTATATTGTTACAAAATACTCATATTTCGGATTTTGTCAATATTTCATTAGTATACATAATTGAAACAGATTTTATCCCCACAATTCTCTAAGAGTTTTCTTGCATTTGCAATTTGATCCAAACCAATTGCATTGGCATGTTCTGTGATAGCCATTCACGACGCAACAAGGTTTTGAATCGCATGATTTATAGGCTTGTGCACATCCCCTTCTTCTCCTGTCTTCTTCTACATGTTCAGTGCTGTGAACTTCGACGGCGTCTGTAAAGGAGTGATGTTTGCAAAGATCTTTCAACATTACTGCGCGGTTGCTGTATTCTCAAATATATACTTCCCCTAAATTATTTCCTTTCTAATATTCAATCCATATTGCAAAATATTGCGAAAATGCTTATTTCAATTATGAAT

PhdNtxNSp 61	P83895	4.77E-09	55.814		14313,32	MLKDLCKHHSFTDAVEVHSTEHVEEDRRRSNFCLPELTKSFIMWLRIHLFVFAVAVTAFLVSCAEPDSIDSIELEEEDRRGCAQAYKSCDSKPCCVVNGYHRTCKCNGFGSNCKCQKTMAERWG*	TAATAATAATTAAGATTACATATCAGGATCTATAGGATATTTCATTCAGCTAGGAATGAATGCAGCCACACAGCAACAATCAGATTGTAAAATAAATATATGTTGATAATTTGATGATGTTTTCATATCCCGTGCATCAAATATCAACATAGGAGCTAGCTTTTTCCGCACAAATAAACACAAAATAATTCGGGAAATATTTGTCGCGGGATGAAAAGGTGAGGGAAAAAGGAAACACCTAAAAGAGTGTGACCTTGAAAAAAAATAATATGAGGTGAAACAAGATAAAAGATTTCTTCTTACAGCCTGAAGCATACAAAAAATCCAATGCGAAATGAAGAGTCTGTATGAGAATGTATAAACATGATGTGTAGCGTAAGTGGTGCTATCAGTTAGAGTATCGAGTTAACAAAGGTGTTTAGTATATGCGGATTTGAGTTTATTGACAATAATTCCTTGAAAGATTTGATATATGTACGTATGTATCTATATAACGTACTATGAGGTTATTTTGTTTGCGCATCTAAGAAAATCTGAAGTAATTTACGTTGCTTTTGCGAAACAAAGAATTAAAACTTGAGTTCACTATATGAATCCAGAGAAGAGTGCGTGTCCATTTCACGAAGAGCTGGGAAAAGGAATGAAACAGGGAAGTCGACTAGAATGAAGGAAAACCAAATGTAGATGAATCAAAGTGAGGAGTATTAAGAATTGATAATTTGTGTTCTTTTACTTCACAGTTAAGAACTATAAACCAGTGGCTGTATAAAATTTTGCCATTTCTTCTCAATAAAATTCGAATACATTTTGGTATAAAATTTACGATGAAACCCTAATATCTGTAATATAGAAAGGCTAGATAAGAAACATATTAACCTAAAAATTTTATTTCCGACGGGAATAACATATTTATCACGAACAATTAATTCCTTAGACTGAGAATAACATAGGGCTGATGTTTCAAGATCGAAGCATGGTCTTGTTATACGATCAGGAACAAATTCTTTCTCGAGGACAAGTGGTTTTTGTTTCATATCTGGCTCTTGTATTTTAAATCTCTGGTAAAATGTGAGGAACTCAATGAAATCATTATATTATCTATAATGTCAGTCATGAAGAACTTTTAATTTTAGAAGAATGTATTGCTGCGATAGATAGTATCAGAGTTAGTTT

							TCTTGTCCTCTGACACTGAATCTTTCAAACCTCAGCAATTTTG<br>TGGTTATCAAGATGGGTTGTTATGTAATTCTTTATTTGAAACT<br>GAAAATTTTACATTCTTTATATTTACTTAACTTTACAAATTAC<br>AGAAAAAGATTCAGACTTCTTGTATTTTCATCACATTTGTGCA<br>GCAATAATTAACATCTGTTATTTTGTTACAAAATACAAATATT<br>TCGGATTATGTCAGTATTCTCTAGTATTCATAGTTGAAAACAT<br>ATTTTATCCCCAACGTTCGGCCATCGTTTTTTGGCATTTGCAA<br>TTACTTCCAAATCCATTACATTTGCAGGTTCTGTGATAGCCGT<br>TCACAACACAACAAGGTTTTGAGTCGCATGATTTATAGGCTT<br>GTGCACATCCTCTTCTGTCTTCTTCTTCTTCAAGTTCAATACTG<br>TCAATTGAGTCTGGTTCAGCACATGATACAAGAAAGGCAGTT<br>ACAGCGACAGCAAACACGAACAAGTGTATTCTAAGCCACAT<br>TATGAACGACTTCGTTAACTCTGGAAGGCAAAAGTTGCTTCT<br>TCTCCTGTCTTCTTCTACATGTTCAGTGCTGTGAACTTCGACG<br>GCGTCTGTAAAGGAGTGATGTTTGCAAAGATCTTTCAACATT<br>ACTGCGCGGTTGCTGTATTCTCAAATATATACTTCCCCTAAAT<br>TATTTCCTTTCTAATATTCAATCCATATTGCAAAATATTGCGA<br>AAATGCTTATTTCAATTATGAAT

PhdNtxNSp 62	P83895	5.89E-09	55.814		9344.7<br>**6938.71**	MWLRIHLFVFAVAVTAFLVSC**AEPDSIDSIELEEEEDRRGCAQAYKSCDSKPCCVVNGYHRTCKCNGFGSNCKCQKTMAERWG***	TAATAATAATTAAGATTACATATCAGGATCTATAGGATATTT<br>CATTCAGCTAGGAATGAATGCAGCCACACAGCAACAATCAG<br>ATTGTAAAATAAATATATGTTGATAATTTGATGATGTTTTCAT<br>ATCCCGTGCATCAAATATCAACATAGGAGCTAGCTTTTTCCG<br>CACAAATAAACACAAAATAATTCGGGAAATATTTGTCGCGG<br>GATGAAAAGGTGAGGGAAAAAGGAAACACCTAAAAGAGTG<br>TGACCTTGAAAAAAAATAATATGAGGTGAAACAAGATAAAA<br>GATTTCTTCTTACAGCCTGAAGCATACAAAAAATCCAATGCG<br>AAATGAAGAGTCTGTATGAGAATGTATAAACATGATGTGTA<br>GCGTAAGTGGTGCTATCAGTTAGAGTATCGAGTTAACAAAGG<br>TGTTTAGTATATGCGGATTTGAGTTTATTGACAATAATTCCTT<br>GAAAGATTTGATATATGTACGTATGTATCTATATAACGTACT<br>ATGAGGTTATTTTGTTTGCGCATCTAAGAAAATCTGAAGTAA<br>TTTACGTTGCTTTTGCGAAACAAAGAATTAAAACTTGAGTTC<br>ACTATATGAATCCAGAGAAGAGTGCGTGTCCATTTCACGAAG<br>AGCTGGGAAAAGGAATGAAACAGGGAAGTCGACTAGAATG<br>AAGGAAAACCAAATGTAGATGAATCAAAGTGAGGAGTATTA<br>AGAATTGATAATTTGTGTTCTTTTACTTCACAGTTAAGAACTA<br>TAAACCAGTGGCTGTATAAAATTTTGCCATTTCTTCTCAATAA<br>AATTCGAATACATTTTGGTATAAAATTTACGATGAAACCCTA<br>ATATCTGTAATATAGAAAGGCTAGATAAGAAACATATTAAC<br>CTAAAAATTTTATTTCCGACGGGAATAACATATTTATCACGA<br>ACAATTAATTCCTTAGACTGAGAATAACATAGGGCTGATGTT<br>TCAAGATCGAAGCATGGTCTTGTTATACGATCAGGAACAAAT<br>TCTTTCTCGAGGACAAGTGGTTTTTGTTTCATATCTGGCTCTTG<br>TATTTTAAATCTCTGGTAAAATGTGAGGAACTCAATGAAATC<br>ATTATATTATCTATAATGTCAGTCATGAAGAACTTTTAATTTT<br>AGAAGAATGTATTGCTGCGATAGATAGTATCAGAGTTAGTTT

							TCTTGTCCTCTGACACTGAATCTTTCAAACCTCAGCAATTTTG<br>TGGTTATCAAGATGGGTTGTTATGTAATTCTTTATTTGAAACT<br>GAAAATTTTACATTCTTTATATTTACTTAACTTTACAAATTAC<br>AGAAAAAGATTCAGACTTCTTGTATTTTCATCACATTTGTGCA<br>ACAATAATTAACGTCTGTTATTTTGTTACAAAATACAAATATT<br>TCGGATTATGTCAGTATTCTCTAGTATTCATAGTTGAAAACAT<br>ATTTTATCCCCAACGTTCGGCCATCGTTTTTTGGCATTTGCAA<br>TTACTTCCAAATCCATTACATTTGCAGGTTCTGTGATAGCCGT<br>TCACAACACAACAAGGTTTTGAGTCGCATGATTTATAGGCTT<br>GTGCACATCCTCTTCTGTCTTCTTCTTCTTCAAGTTCAATACTG<br>TCAATTGAGTCTGGTTCAGCACATGATACAAGAAAGGCAGTT<br>ACAGCGACAGCAAACACGAACAAGTGTATTCTAAGCCACAT<br>TATGAACGACTTCGTTAACTCTGGAAGGCAAAAGTTGCTTAT<br>GGCAAAGGATAAATGGCAAAGGATTTGAATTGCCCCGAGAT<br>TGTTCAATATTGCTGCGCGGTTGCCGTATTTTAAATATATACT<br>TACTCCGAAATTATTTTATTTGTCATATTAAATACATATGGCA<br>GAAATGGATGTTTCAATTTTGAATGACTGGCGGGCCATCTGT<br>GTTTACTGATAAATATAATGCATAAACAGCTGTTTAATTTAA<br>ATTCTAAATACCCATTAATTTTGCTACATGTCAATTCAGAAAT<br>ATCTCAGACATTGTAACAAAGTATCTGTTTGAGGGATTGATA<br>GATATTGAAAATTGAAGTTAAAAATTAGTTTATTTGTATGGA<br>AATATCTGTATAGGCAATGCTATATATGAATCGTTTTTATTTA<br>TTTCATAAAGATAAACATTTTATTAGTTATATTTACTTATTTTG<br>CTTGTCCAGTTATTCGAAGTACATTTCAAGAATGGGGCACAC<br>AAGGGAAAGACAAAAGGTTAAATTACTAGATAAATATTTTTT<br>AGAGCATTTATTAGGTTTCACATATTAAACTGATATTACCCGT<br>TGTAGCGATTTTTGATACAGAAAAGAAAGAGCTAGTATCGC<br>GTATGGTAGTGCTGTTAACTATTGCATTATTCCGGGTATTTAG

							AGCCTACACCTGTGGAACAAAGTATAGTCAACAAAATAATTAACGACCTAAAATCAAAGTATAGTCAACAAATTAATTAATGAGCTAAAATCTACAAGTTTATATTATGCAAAGGCGTATGTGTTTAAATTCAAAACAGTAATACACACAACAGACGAAAATTTGAAAAGTACTTTAAAGTTCAGCTGGCAAATGTAGTAGATAGATAGATGCACGCTCACATGTATTCATATATGTCTATATATACATGCATACATTCTTATATGTTGCTGTTTCTTATAACCAATTTGTTTGCAGTAAGTTTTTAATCCATAATTTCATGGAATCAGTTAGTTAAAATATGCATTAAACCAAAAATAATGAAACATGTGATTGACTTAAATCATATTAATTTAAGAAGATAAAATATTACTCTAAATTTTTTCATAAATTCAATACGATAGATTGAACATACATACATATTT

PhdNtxNSp 63	P0C2S6	8.67E-12	53.571		9763.28 **7279.18**	MWLRIHFFVFAVAVIAFFIACA**EPDSIHSTEHVEEDRRRRGCAQAYKSCDSKPCCVVNGYHRTCQCNWFGSNCKCKKTLRELWG***	CTATTATTCTACTAGCTGTTGCTATTATTCTACTAGCTGCTACTATTACTCTACTAGCTGCTACTATTATTCTATTAGCTACTACTAGTCCGAAACTAGTAGCAGCTGTTCGGCGTCATCGTGGAGATTTGGAATAACTGTTTCTACATCTTATATTTTCCACAAGTCATCTGTGAGAATCAAAGTGAGGTCTGTTAAGAGTTGATGACTTGTGTTCTTCTACTCCATTGTTATATGGTATAAAACGAGCATGATCTTATTACATAAGCTGAAAAAACTTACTCGTGAACAGATGGTTTTTTTTGTTTATTATTGTTTGTTGAATTTTAAATCTCTCTTAAAATGGGAATGGAAAAGAAATCGCTATATTATCTATTACTGAAACTGTCATTCGCAAAAAACTTTTAATTTTATAAAAATGTATTGCTGCAATAGATATCAACAGTTAGTTTCCTTGTCCTCCAGTAAATTATCTGAAACTAAATCTTTCGCTTTATAGCAAATTTTAGTTATTAAGTAGGGTTGTTATGTAGTTCTTTATTTGAAATTGAAAACGAATGGAGGAGTAATATTTTACATTCATTACATTTAGTTAAATTTACTAATTACAGATATAGATACTCTCTTTTTATAGTTTCATCACTTTTGTGCAACATTATTTAATGTCTGTTATATTGTTACAAAATACTCATATTTCGGATTTTGTCAATATTTCATTAGTATACATAATTGAAACAGATTTTATCCCCACAATTCTCTAAGAGTTTTCTTGCATTTGCAATTTGATCCAAACCAATTGCATTGGCATGTTCTGTGATAGCCATTCACGACGCAACAAGGTTTTGAATCGCATGATTTATAGGCTTGTGCACATCCCCTTCTTCTCCTGTCTTCTTCTACATGTTCAGTGCTGTGAATTGAGTCTGGTTCAGCACATGCTATGAAAAAGGCAATTACAGCTACAGCAAACACGAAGAAGTGTATTCTAAGCCACATTAT
PhdNtxNSp 64	P0C2S9	9.33E-19	55.172		6800,7	LENEAEEKAPALREESARDCFGYGDSCKSNKNGCCWLFTCKC	ATGAAATAAACACGTTGGGAAATGCCTTTAATATTCATATTATGTTCATATAATAATCAGCAAGAAACATATTAGAATTGTTATCATGATTCATATTTTCTTGACTACCAAAACTTGCACTTGCACCACTCAGTTTTTTGTCCAAATGGATGCCAGCATTTACAGGTAA

						WHPFGQKTEWCKC KFW*	AAAGCCAACAGCAACCATTTTTGTTGCTCTTGCAGCTGTCTCC ATACCCGAAGCAGTCTCTGGCGCTTTCTTCTCGTAATGCCGGT GCTTTCTCTTCCGCTTCATTTTCCAAAA
PhdNtxNSp 65	P0C2S9	1.07E-11	71.154		6172.1 **4272.59**	MKFCILLALLLITVV RA**EEDILENEAEEIA PAVQDESGRGCIG HRKSCEHDKNGCC**	TGGAGTTCTAACGTGAGCGCTCCAACATCTGGAAAGAAAAT GAATGCGACATATCATAATTTAAAGATCAAATAACATCTGTA CTTTGATATAAAACCCAAAATCTTTTACAAAAGGAAGTAAAT ATATTTTTGCGAAATATCTGAAGAATTACGAGCGAGAAATAT CTTAAGCGTGAGCAATGAAGTTTTGTATTTTACTTGCTTTACT TTTGATAACGGTTGTACGAGCCGAAGAAGATATTTTGGAAAA TGAAGCGGAAGAGATAGCACCGGCAGTACAAGATGAAAGT GGCAGAGGATGCATCGGACATCGAAAGAGTTGCGAACACGA CAAAAACGGTTGTTGT
PhdNtxNSp 66	P83895	1.04E-08	76.923		3801,44	LFTISIYRCADAWES CDNRPCCVVNGWK RGCMC	ATCGTTGGATTCAAGTAAATGATTCTATTATCCCATGATCCAA TTAAACTGTGAGTCATCAAACAAAGGTGTTCCAATTAATCGA TAAATCAAATAAGCATATGATTCAATAAATTGCTCGTTCGAA TAAACGACGATGCTATTAACCGATGCATCTATTAACCAATGA TCCAATTAACGTTTGCCCAAAATTAAATTAATCGTTGGATTC AATTAAATTATTACATTATCCCATGATCCAATTGAAAAGCGA TGCATTAAACCAGGATTCTAATTAACCGATAAACCAATTTAA CATATGATTCAATTAACCGATGATTCAGCGTACCAGTGAAGC TTTTCCTTGTAAAGCTCCATCTTCCGTTTTGGCGTTACCACTTA TACAAATTCCCGCTATCGAAAAGGATGGCAATCTAACTGTGT TACTCTCATCTACGAAGTTTAACTCCAAAAATTAAAGAAATA AATGAATAATTTATATAAGTATTTCTGAAAATTTAGTACAAC GGTAACTCTGTCGCAGTGATTTTAGAATATTTATATTACATTT AAATTATTTGGCAAATTATACTACATCCTTTTAATGAGCACTA TATAATATCGTTTAATTGTTCACAATTTCTATTTACAGGTGTG

							CAGATGCTTGGGAATCCTGTGATAATCGACCTTGTTGTGTAGT GAATGGTTGGAAAAGAGGTTGCATGTGTAG
PhdNtxNSp 67	P83895	3.15E-09	76.923		3801,44	LFTISIYRCADAWES CDNRPCCVVNGWK RGCMC	TCCAATTAACCGATAAACCAAATGAACGTAGGATTCAATTA ACCGATGATCCAGCGTACCAGGGAGTCATTTCCGTGCAGAG CTCCAGCTTCCGTTTTACGTTACCCTAAGCAACGGACACTTCC GCTACAGAAAAGGTTAACGATCTCACTATGTAACTCTCATCT AAGAAGTTTAACTCGAAAAATTAAAGAAAGAAATGAATAAT TTATAGAATTATTCTGAAAATTTAGCAAAACGATAACTCTGT CGCAGTGATTTTAGAATATTTATATTACATTTAAATTATTTGG CAAATTATACTACATCCTTTTAATGAGCACTATATAATATCGT TTAATTGTTCACAATTTCTATTTACAGGTGTGCAGATGCTTGG GAATCCTGTGATAATCGACCTTGTTGTGTAGTGAATGGTTGG AAAAGAGGTTGCATGTGTAG
PhdNtxNSp 68	P83895	2.39E-13	64.286		10156.63<br><br>**7610.46**	MWFKMHLLLFAFA LPALLRASS**IPAESD SVDVADFVEEEDR GKCADAWESCDNR PCCVVNGWKRGC MCSLNLRNCDCVR SISEDIYNLFG***	TTTTTTCTTTCAATCAAATCCATCTGGCCTGCAATTCACTTAA CTATTGTTCGATTATATTTTAGCTTCGTTCGATAAGGAAAAAT ATATCCGTCAAGAGTATTTAAGAAACCGGAAAAATTATTCTA ATAGTGACTTATTCCTGAGCAAAGACCATATCCCATTTGCCA CAGATTTTAGAACAGAGGCATCAAGACGCTTCAAAATGTGG TTTAAAATGCACTTACTTTTGTTTGCTTTCGCTTTACCAGCATT ACTGCGTGCTTCATCAATACCAGCTGAATCAGATTCAGTTGA

							CGTCGCTGATTTTGTGGAAGAAGAAGACAGAGGAAAGTGTG<br>CAGATGCTTGGGAATCCTGTGATAATCGACCTTGTTGTGTAGT<br>GAATGGTTGGAAAAGAGGTTGCATGTGTAGTTTGAATTTGAG<br>AAACTGCGATTGCGTGAGATCTATTTCAGAAGATATTTATAA<br>TCTCTTTGGGTAATCTATTTGTAGATGAAAGAAGACTAATAA<br>TGGATTTTGACAAAATTCAAATCTATATCAAGTAATACAATA<br>TTAG
PhdNtxNSp<br>69	P83895	2.41E-13	64.286		10142.61<br><br>**7596.43**	MWFKMHLLLFAFA<br>LPALLRASS**IPAESD<br>SVDVADFVEEEDR<br>GKCADAWESCDNR<br>PCCVVNGWKRGC<br>MCSLNLRNCDCVR<br>SISEDVYNLFG***	TTTTTTCTTTCAATCAAATCCATCTGGCCTGCAATTCACTTAA<br>CTATTGTTCGATTATATTTTAGCTTTATTCGATAAAGAAAAAT<br>ATATCCTTCAAGAGTATTTAAGAAACAGGAAAAATTATTCTA<br>ATAGTGATTTATTCCTGAGCAAAGTCTATATTCCCTTTGGCAC<br>AGATTTTAGAGCAGAGGCATCAAGACGCTTCAAAATGTGGTT<br>TAAAATGCATTTACTTTTGTTTGCTTTCGCTTTACCAGCATTAC<br>TGCGTGCTTCATCAATACCAGCTGAATCAGATTCAGTTGACG<br>TCGCTGATTTTGTGGAAGAAGAAGACAGAGGAAAGTGTGCA<br>GATGCTTGGGAATCCTGTGATAATCGACCTTGTTGTGTAGTG<br>AATGGTTGGAAAAGAGGTTGCATGTGTAGTTTGAATTTGAGA<br>AACTGCGATTGCGTGAGATCTATATCAGAAGATGTTTATAAT<br>CTCTTTGGGTAATCTATTTGTAGATGAAAGAAGACTAATAAT<br>GGATTTTGACAAAATTCAAATCTATATCAAGTAATACAATAT<br>TAG
PhdNtxNSp<br>70	P83895	1.91E-12	71.429		5487,33	EDRGKCAGAWERC<br>DNLPCCKKNGWSR<br>TCMCSLNKRNCDCI<br>VTLGETIFG*	AAGAAGACAGAGGAAAGTGTGCAGGCGCTTGGGAAAGATG<br>CGATAACCTACCTTGTTGTAAAAAGAATGGTTGGTCAAGAAC<br>TTGCATGTGTAGTTTGAACAAAAGAAACTGTGATTGCATAGT<br>AACACTCGGAGAAACTATCTTTGGGTAATCTATTTATAGATG<br>AAAGACGAATAATAGTGGATTTTGACAAAATTCAAATGTAT<br>ATCAAGTAATACAATATTAGCTTTCGAAAACTGCTATTAATG

							AGATGTGGATAATGTAATTGCTGAATATTTTACTGAATAAAT<br>GAATGCAAATCTGATAAAAATATATA
PhdNtxNSp 71	P83895	2.67E-10	66.667		7801,95	FFACALPALLRSTAL<br>HAEPVSVDIVEFLEE<br>EDREKCAGAWVRC<br>DNLPCCKKNGWTR<br>TCTCSLNERNCYC	TGCAATAACAGTTTCTTTCGTTCAAGCTACACGTGCAAGTTCT<br>TGTCCAACCATTCTTTTTGCAACAAGGTAGGTTATCGCATCTT<br>ACCCAAGCGCCTGCACACTTTTCTCTGTCTTCTTCTTCTAAAA<br>ATTCAACGATGTCAACTGATACTGGTTCAGCATGCAGTGCTG<br>TAGAACGCAGTAATGCAGGTAAAGCGCAAGCAAAGAAAA
PhdNtxNSp 72	P84015	1.4E-17	100		7876.24<br>**5090.75**	MKMSFIFGLCITAIL<br>MLSFLDFSCA**ENVD**<br>**DQEAPQERGFCAQ**<br>**KGIKCHDIHCCTNL**<br>**KCVREGSNRVCRK**<br>**A***	TATTCAGATAGATTAAACCACTAATGTGATGGAAGGGGGCC<br>GCACTAATATAATTTCATTTAAAAAACAGAATTGCAATTAAA<br>ATCAACCCGTTTCAACTCGATTCAAACTATTTCAAGCAAGAT<br>CCTCATATAAACATTCGGCACTCTCCCCACAGAGCGGATCCA<br>GACTACGTCATCACGACGGACTTCCGGCTTCACTTTCTGAGC<br>AGCGTTGAACTCTACCTGGGTTACCAGTACCTATCACAGAGG<br>CCTAGCTATTAGAGCACCTATCAAGCAACAGTATCGAGTCGT<br>TGTGTTTCTAGTTGCATAACATGATATTAATATAACTAGTAAT<br>TTTTAAATGTTAATCTTCTATCTCTTGCTAGTTTTTAAAATGTT<br>GCAATCTGTTGTGAGCTAAGATTTCATTTTAAGATTTTTTTCGT<br>ATATGGGTCAGCTCATTTGTGTCTAGTCTTTTTTTGTTTCTCTC<br>AAACATTCTGCTAAGATTTCAAGCGTTCCAGGTGTTATACTTA<br>AGAGCACAATAATCATCCACAAAATCAAATTTATTTAAAAC<br>AAGGAGATAAGTATGATATATTAGAGATTCTGTTGTGAACTC<br>CGTCAATAGAAATATAATAATATAAATCATTGAATTTATTTA<br>TTTCCGGTTACATTCCTCTCCAACATTAATTGCAATTTTTGAA<br>TCAGCGTACAAGTTTCTTAATCATAATATTTTTAATAAAACTT<br>GTCTGGATTTAACTTTCTTATGCTTTCCGGCAGACACGATTAG

							AACCTTCTCGCACGCACTTGAGATTAGTGCAACAATGGATAT CATGGCATTTGATGCCTTTCTGAGCACAGAATCCGCGTTCTTG CGGAGCTTCTTGATCATCCACATTTTCTGCACAGGAAAAGTC CAGGAATGAAAGCATCAGAATTGCAGTGATGCACAATCCAA ATATAAATGACATTTTCATGATTGCGCAGGATTTGGTAGAAG TGCTTGTCTTAAAATA
PhdNtxNSp 73	P84015	3.06E-05	100		5371,45	NQLLRNLWSFSAKY LNICIILAVWHLLQR MQFCAQKGIKCHDI HC	CAACAATGGATATCATGGCATTTGATGCCTTTCTGAGCACAG AATTGCATTCTTTGGAGTAAATGCCAGACGGCGAGGATTATG CAGATATTTAAATACTTTGCAGAGAAACTCCACAAATTTCGG AGAAGTTGGTTTCACTTTGTGGAGCCGCGTTCTTGCGGAGCTT CTTGATCATCCACATTTTCTGCACAGGAAAAGTCCAGGAATG
PhdNtxNSp 74	P0C2S9	1.46E-33	94.34		7090,01	RPRARFSIAEDIAPA VHEESGRECIGHRRS CKENRNGCCSLYTC NCWYPTPGDQWCK CQLW*	CGACGTTGCTTGACGCGCCGCACGGAGGTCCCATTTTCCACC GGGCCATCCATTGAAACCATAATGGGTGTACGTATCTCAAGT AGCGGCTCTTGAACAAGTGCCAAGAAGGAAGAACTTTTTCTA CATGTTGTAAACAGTCCAACCTGGTGCTCTTTAACAAGGGAG ATACGGATGCTGCTGACCCGTCCAGGTATCTACCAATCTGCC TACTGACGGCCTAGAGCAAGGTTCTCGATAGCGGAAGATAT AGCACCTGCAGTACATGAAGAAAGTGGCAGAGAATGCATCG GACATAGAAGGAGCTGCAAAGAGAACAGAAATGGTTGTTGT AGCTTGTATACATGCAACTGCTGGTATCCGACACCAGGTGAT

							CAGTGGTGCAAGTGCCAGCTTTGGTAGTCAAGAAAACATGAATCCTGATAAAAATTAAATGTGTTTCAAG
PhdNtxNSp75	O76198	2.84E-05	94.118	PF17492	12427.16	SRKHILLSLHSSRKFLCCHQPVEEDKTFDCFLSYGSEDGDVIFAIVDELEKGENPEFTFCFHERNWKPGIPITDNIFQSVQNCRKTLIVLSEAFVKSMWFYLEFQA	GCAGCCTGAAACTCCAGGTAGAACCACATGCTTTTGACGAATGCCTCGGAGAGGACTATCAGTGTCTTCCTGCAGTTCTGTACGGATTGAAAAATGTTGTCGGTGATGGGTATTCCAGGTTTCCAGTTTCGCTCGTGGAAACAGAACGTAAATTCGGGATTCTCTCCTTTCTCAAGTTCGTCCACTATAGCGAATATCACGTCACCGTCCTCCGAACCGTAGGAGAGGAAGCAGTCGAACGTTTTATCTTCTTCGACTGGCTGGTGACAGCAAAGAAACTTGCGACTGCTGTGGAGAGAGAGGAGAATGTGTTTGCGACTTG
Calcium channel toxins							
**Transcript ID**	**Uniprot ID reference protein**	**e-value**	**Identity (%)**	**PFAM domain**	**MW (Da)**	**Amino acid sequence**	**Nucleotide sequence**
PhdNtxCav01	P86418	6,07E-11	62,857	PF07740	8264.65<br>**6305.08**	MKCSLFLILLLITLVRT**EDTNRNVAVEAEDRAPETNEERIACASHFSRCNSGKACCKGLRCRGPLENMGPMYCL**	TAAGCAGTACATTGGCCCCATATTTTCAAGTGGACCCCTGCATCTCAGACCTTTGCAGCATGCCTTGCCGCTATTACATCGACTGAAATGTGAAGCACAAGCGATCCTTTCTTCATTGGTTTCTGGAGCTCTGTCTTCTGCTTCAACTGCAACATTCCTATTCGTGTCTTCTGTGCGCACGAGCGTTATCAAAAGTAGAATAAGAAAAAGGGAACACTTCATTGTTCAGCTTTTGGAA

PhdNtxCav 02	P83996	2,35E-08	58,333	PF07740	5566.56	LKQKTRAPDISEERIDCVPRGRLCRSDKECCKGLRCRTNIWPYLCVR*	GGATTAGAAATAAACTTTTGTAACGTGCAATCTAATTTTTTATTGTGCTGATAACAATCATCAGAAAATACAATTATATAGTAGGGTAATCACGAGCAGCAGAAAATAATTTCAAAATTTATTTCATTATCATCAGTATGTCTTTACCGTACGCAGAGATATGGCCATATATTAGTCCTGCATCTCAGACCTTTGCAGCATTCCTTGTCGCTTCTACATAGCCGTCCACGGGGAACACAATCGATCCTTTCCTCACTGATATCTGGAGCTCTGGTCTTCTGCTTCAACTAGAAAATTCTTATTCGCTTCTTCTGTGCGAGTGTTATCAAAAGTAGAATGAGAAAAATTGAACGCTTCATTGTTCAGCTTTTGGAAATCGCCTCAGTAGAAATCTTCTTAGATTATTAGAAATGTCGCATATAATTTGCGGGAGTCTTGGCATTTATATATATAAATTATGTGTTATTTCATCATCGAACAAAAAGGTGCATACTAATATCA
PhdNtxCav 03	P86418	3,40E-08	60,000	PF07740	3461.11	HFSRCNSGKACCKGLRCRGPLENMGPMYCLQ*	TAGAGATATACTTTTGTAGCAAGGAATTTATTTTTTTATTGTGCTGATAACAATCATCAGAAAATACAATTATATAGTAGGGTAATCACGAGCAGCAGAAAATAATTTCAAAATTTATTTCATTGTCATCAGTATTTCTTTACTGTAAGCAGTACATTGGCCCCATATTTTCAAGTGGACCCCTGCATCTCAGACCTTTGCAGCATGCCTTGCCGCTATTACATCGACTGAAATGTG

PhdNtxCav<br>04	P81790	1,33E-40	86,458	PF08396	12788.75<br><br>**10468.73**	MKMKLLGIILLVSFP<br>FVLGFA**GIPIEEGEN**<br>**SVEVEEVERSCINH**<br>**GDECDGYKDDCQC**<br>**CRSNGFCNCSGVF**<br>**GYKWNCICDVGTT**<br>**ATSYHICMAKHKC**<br>**GRQTTCTKPCLSRR**<br>**CRNRG***	GTTACTTACCACTCAAGACTGTCAAATGAATCTGAATACCTT<br>GCTAAGAAATACTTCATTCAAACAATGTTTAAAGCCGTGTTT<br>TTCCTTTACTTGCATTTTGTGATTGATTTTTATGCCATCTGGCG<br>CAATGATATATATTGAGATTTCAAGCCTCTTCATATTGTAGAG<br>CTCTTTTGCCAGAGAAATTATGAAAATGAAACTTCTGGGAAT<br>CATTCTTCTAGTAAGCTTTCCTTTCGTGCTGGGTTTCGCCGGC<br>ATACCGATAGAGGAAGGGGAAAATTCTGTTGAAGTTGAAGA<br>AGTTGAAAGAAGCTGCATCAACCACGGGGATGAATGTGATG<br>GGTATAAAGACGACTGCCAGTGTTGCAGATCTAATGGTTTTT<br>GTAACTGTTCAGGGGTTTTTGGATATAAATGGAATTGTATTTG<br>CGATGTTGGTACTACAGCAACAAGCTACCATATATGCATGGC<br>GAAGCATAAGTGCGGTCGGCAGACCACTTGCACCAAGCCCT<br>GCTTGAGCAGACGCTGCAGAAACCGTGGATAAAATTGTTCTC<br>ACCAAGTCGTATTGTACGTTCGAATGTCTACGGAAGATAATG<br>CAACAATAAAAATAATTTAGCGCAGTTGTATTTATTATGCCTT<br>TTACATGTGAGGTCAAAATAATGTCATGTATATAAAATGCTT<br>TTAGGAAATTTCGAGTACTATTTCCTGAAGAATGCATGAGAA<br>TATCATATACCTCATGCGTTTGTTACAAAAAATCCTGGAGTG<br>AAAAATATTTTCATGCACACAGTCTATACGATTATTATAAAG<br>AGCGAATTTTGTTTAATTGTAATGCGCTGGGTAATCTTTGAAT<br>TTACTCTAACGATTGGAAAAAAAATCTTTCGCCTGTAGAAAT<br>TTGTATTTCATCTCGATGGGCGCAGACTTTTCATTATTATGGT<br>ATGTCAAGACAGGGGATCCACAGAAGAAGATTATTTAAGAA<br>AGGCGCTGGGGAGTCGAGTCGTGATACGCGGGCGGTATTAA<br>AGAATTTAAAATGTTGTTGCACAGCATAATCTTTAGCAAAGT<br>CTGTTTTACGGTTTAGGTTCGAGTTCGGATCCAGCCATTTCTT<br>ACAATCCTCAAAGAAATCAATTTTTGTTCAGTGGAGTGATGA<br>ACCCATAATAAACTAGGATTCGAAGCCGCGTCGTTCGTGGGC

							GAATAACCTGCAGATAAGTGCCTGCAGAATTAAAAATAAAG<br>AATCAAAAATTTACAATTAGTAATTTAAAATATTTCTGATTTG<br>GAACAAGTGATTATGGACATAGGTGAAATCTACGCAGACTA<br>AGCTATAAATTATATAGGTCTTGAAATGTTCTGAAATCCTAT<br>ATATCACTGTCACACTAGAAATTTATATTTATTGTCCATTCCG<br>CTTTATTTCCACTAACCGTTTAAATTATATAATAAAAAAACA<br>AATATTATTCCAATGCATTATTTTTGCTTTCTCTTCCCTATTCG<br>AATCACGCAACAGAACAGTACGCTATTTGACAATCGCTAAT<br>GAACAGGGAGATTTTGTGGCATAACTAATGGTGAACACTGGT<br>CCAGAGAATGCAATGTGCTCAATGTAGAGTAGAAGACCTAT<br>GGATCTTTATTAAACTGAAATAGTGTAGATTTCGCACCAGAG<br>GAAAAGAACAGTATAACAAAAGTAGAGGTTTCACATACCTT<br>CTTTGAATATTCGTAGAAAATGCCATTATTTAAATAAATAGC<br>AACAGATATCACAATATCAGTAGAGAGTATGACTGAGAAGA<br>TAATCGGTGCCTGAGAAGATAATCGGAGATTTCTTGCAAAGC<br>ACTTTTCGTTTCCTGCACCTTTTGGGGTGATGGTGTTCTCTACG<br>ACAGACGTTTTCCAATAACGCTCTAGACTTGTTCGCTAGGATT<br>GAGTTTCGGAGAGTATACAGGTTGTCATCTGGATGATATCGT<br>CACTCTCCAACACAGCAGTCACAACATTTGACTAGTGTGGAC<br>ACTCCTTGTCGTCCAGATACAGAAAATCGTGAATTATTGCGC<br>TATAGAAAAGACGAACATGACCCCGAAGAATCTGCTTAAAA<br>TATCTTCCTGTTGTATAGAACCTTGATATAAAGGTATGCAGCT<br>GTGTTCTAGCGTCATGCGTGACCCTTGCCATAACCATTTTGCC<br>AAAACCTAACGTTCAACATTTTGGACTTTTCATTGTTGGTAAC<br>GTGTTTCCTGTTTTCACTACCAGTTGGTGGCCGAAATCGCTTT<br>TCACAGCAAACCGGAATTCATCAACTTTGACTTTACCCGATA<br>GCCTCAGTGACGATGCTTCTCACACCACTGTAATCTGGCTTCC<br>CAATGGTGCAACGACAACGGAGCACATTTAACTGGCTTCCGT

							GGACAGAATTAAAAAAATTTAGTCTATCTAAGATGGTTCGAG AAGAGAAGAGGGTATATCCATAACTGTTCTGAAATCTGCAG CAATCTTTTCAGATTTAGTTTCTCTCTTTCTTTCGCTATAATGG AAACCCTTTCGCGTCTTGCTGCTGTGCTCTGTGTTGGGCCACC TCCCTGCACACATGGACGTGATCCTCTGCTCTTCTGATAGCTA TTTTCAATCGCGAGATTACACTCTTGAAGATTCCCGTTGCTAT CGCTACTACAGTAACACTTTGGTCCACGTCCAGGTGTTCAAC CGCTCGTCCACGATCGTACTCACTCATATGAGGCCATGAAGT CATTTTGCGACAGTAATTACTGTAAGCAGCAGTATTTCGCAA GGAAATTTGCGGAATAATTTACGAAAACGACATTTGTATTGC ATAGAGGAGTCACTGCAGCTTTACTTCTAATTAACAGCAACT CGTAACTCACTTCGTTTTCACAGATGATAAAATTTTCATTGCA GCACTTTGTTCAATAGTTCCTATGTCCCTTCGCACCTGTTTAG CCGCACGGCAAAACAAAAACCGATTGTTTCTTAGCAATTCGG AAACAATGTACTTCTCTACGAAAGATACCCGAGCAACAGTG TAAAATCATAGAAAGATAGTTCGTAATAATGACCATAAAGT AAATTTGGCCGTATCTGCCGTAATCTTTAGTTATGACGGTTGT GTATATGTACCTGAGGAAAATGAATTATTTTATTTCAATTGTA CATTACAGGGCCCGAGACAGCGGCAGGATCTTACGTGTGGA ATTCCCGCCGGTCAAGAAAAAGACCGCAGAAGGCAGCCACC CGACGACATCCGAAACCTTGACTCTCACAGGCCACATCCCA GGGCAGAACAGCACGAGATAAAAGCCCCACAAAGCAACAC ATCCAAGGGCACCGCAGATAGTCAATACAGAATTTGGAATA TAAAGAGCGGGAGAAAGACTCAAAGACACTCCGAGAGGCA AAAGAGGGAGATCGAATTCGCATTTAAACAGCCTGTATGCT AATTTTGCCGATTGCGAGAATTCGAAAAATTGCCTTATATTAT TTGACGCTTTTGCCGACGTCTGAAATTCCCATACTAGGAACA CTATGACTAATTCATTATCTTGAAAGAAGCTGGTTGGGGTAA

							ACAATGATAAACCAAATATTTGTTAACCTTCTGAACTCGTATGTCACACTGGAGGTGACAGTATCAACGTGTTACGAAACTCGTACGTTATGCTGGAGATGGAAATACTTACAGATAACTTGTGGCGATGAAAACAAATTTGAAAAAGCTTCTTGACCACAATAATGCGGGAACGCTGCAACTAGTTTCGAAGCATTAATTAATGCTTCGTCTTTTCTGACGTACGCACCTAATGTTTCTCTGCCTG

PhdNtxCav 05	P81790	6,93E-64	85,714	PF08396	12585.69<br>**10525.03**	MKLLGIILLVSFPFVLGFA**GIPIEKGENPLEVGELERGCINVGDFCDGEKDDCQCCRDNAYCSCSGIFGWKWNCRCEVGTTATSYGICMAKHKCGRQTTCTKPCLNRRCKNRMG***	TGAATACCTTGCTAAGAAATACTCCATTCAAACAATGTTTAATCTGTGTTTTTTCCTTTACCTGCATTTTGTTGTTGATTTTAATGCCACCTGGTGAATTGATATATATATAGTCGGTGAGATTTCAAGCTTCTTCTTAGTGTAGAGCTCTTTTGCCAGAGAAATTATCAAAATGAAACTTCTGGGAATCATTCTTCTAGTAAGCTTTCCTTTCGTGCTGGGTTTCGCCGGCATACCGATAGAGAAAGGGGAAAATCCTCTTGAAGTTGGAGAATTGGAGAGAGGCTGCATCAATGTCGGGGACTTTTGCGATGGGGAAAAAGACGACTGCCAGTGCTGCAGAGATAATGCTTATTGTAGCTGTTCAGGGATTTTTGGATGGAAATGGAATTGCAGGTGCGAGGTTGGTACTACAGCAACAAGCTACGGTATATGCATGGCGAAGCATAAGTGCGGTCGGCAGACCACTTGCACCAAGCCCTGCTTGAACAGACGCTGCAAAAACAGAATGGGATAAAATTGTTCTCATTAAGTTGTATTGTATGTACGAATGTGAACGGAAGAGATTGCAACAATAAAAATGAATTAACACATTAAAAAA
PhdNtxCav 06	P84093	2,21E-50	97,468	PF08396	13377.35<br>**11320.77**	MKLLGIFLVASFAFVLSFA**EEMIEGESPLEAHGTERRDCIELNNDCDGSKDDCQCCRDNAYCSCYNFFGIKSGCKCSVGNSVTGYSVCLKKLECPNRRAWTSWKKECTKPCIGKRCKNNG***	CACTTCGGGAGCATATCACAATATGAAAAATAAATGCAAATTTTACGTAATTTTTATTAGTTATGTATAATTTAAAGTTAGCTAGAGAAAGTACTTTAGAGAATATTTGCCCACTAATTCTGATAGCTGACATTTGATGGATTATACAAACGCTCCGTGTAGTTCCCCCTCAGTACATACAGTAACGGTATATACTCTGTGTTCACGGGGAAGAAATTAAGATTACTCTTGGTGAAAGGATTTTCTGACTCGTTTGAGTAGTTTCAGAGACTACTGAATACAACCATTTTTTTCCCTTTATAATGTTAGTGTAGTCAGTAAGGCCTGAATATATTATTGTCTCCAAGATTTCTTATTGCAGATGTAGGAGGTCTGGAATGTTTTAGACTTTAGGAAACAGAACTCGAAGTTTGCGAAAAGGATTTTAAATACAAGCCATCATTTTCTACTCAAATCCAAGAAAAGTAGAATTATATGTATGCTAATTTATTTTTCTTTGTTACATCCTTTCCGTTTACGGATAACAAACATTTAATGTGCACAAAATCT

							GACAGCGTCAATTTTATCCATTGTTTTTGCAACGTTTCCCGAT<br>GCAGGGCTTGGTGCATTCCTTCTTCCAAGAGGTCCAGGCACG<br>TCGGTTCGGGCACTCAAGCTTCTTCAAACATACACTGTATCC<br>GGTTACTGAATTACCAACTGAGCACTTGCAACCCGATTTAAT<br>TCCAAAAAAATTATAACAGCTGCAATATGCATTATCTCGGCA<br>GCACTGGCAGTCGTCTTTAGACCCATCGCAATCATTATTGAG<br>TTCGATGCAGTCCCTTCTTTCAGTTCCGTGAGCTTCAAGAGGA<br>CTTTCCCCTTCTATCATTTCTTCCGCGAAGCTGAGTACGAAAG<br>CAAAGCTTGCTACCAGAAATATCCCCAGAAGTTTCAT

PhdNtxCav 07	P84093	1,76E-44	91,139	PF08396	13521.52<br>**11464.94**	MKLLGIFLVASFAFVLSFA**EEMIEGESPLEAHGTERRDCIELNNDCDGSKDDCQCCRDNAYCSCYNFFGIKSGCKCSVGNSVTGYSVCLKKLECPNRRSWTTWKKECEKPCLTKRCRNKG***	TGTTTTCCCGGACATTTCCCCAAATTTTCTGATTTAGCAGGACAAGTTCCCTGCTATTTCATATATATAATTTTACTATTAGTATTAAAGATACACACATATACGAAGGTATTTTAATTTGTAAATATTGAGGAGAAAAGTAGAAAAACGTTATGGCTCCGTAAGGTCCCACTAAACTCCTGGAATCAAATAACTAAACTCCCGGAATCAAATAAATTGACTCCTTAAGAAACTCATATAAGGCTGACATCTCTGCTAATTTGATTTATTTTAAATTAAGATAATATAATTTTAACATTTCAATCATAAATTGGTAAAAAGAAATAGTTTTATCTCCTTCGATTTGATTTTATAACCTGCTTCCAAAATTCGGGTGTTAGGTCATAATGGTATCAAAGATAGACGGCGCAACATAGCAAAAGAGAATAAAAAAGAATTCTAGCTACGTTTGTTTGAATGTTTGCGTTTCATTTTATGATTGCAGTGTAAGTAGCATGAAAATATAGGCTAACGTTATAGCGCATAAATATGAAGAAAATATATTTAGCGGGACTAAATGATTTATTCACACTAAACTGTGTATAAATTATTAACCAAAATATCTGACCCTGGATATTATACTCTTGTTTTCTTTATTTTTTAGGATTCGTTAGAGGAATTTGTCAGGATTCTGAAAACAGTTTGAGCGAAGAATGGTTTATGACAAACTGCCCATTACAGCCCTCAGTGTAAGGAACTTACGAGCCAAAAACGTTCAAGTTAGAAAAGAGCGAAAGTAGCAACTCTTGAAAAAGCCTTGGAATATATGTGGCAGAAATTCGATGATAGAGAATTTTCAGGTGAGTTTAAAAATTATTTTTCGCCAGATTCTGGATCTGTTTGAGACGAAAATACCGTCGATGATAATGAAATGTCTAATGTTAATTCGAAAGATATTTGTTCCTGGGGACTTTGAATTCCATATGAAATTAAAACGCGAGGCAAATTCTCTGTTATTAATACATTTAAGAGAAATCAAAATTATATCAATGGAATAAGGGAGAACAGATGATCACATGATGGAAAATTGTTCAAATGCAAAATTTCAGGAAAATCGATTGATTTATTAGCAAAATAACCCTTTGAATTATTTAAAATATTCTTTGGCCTAGAAATTCAAGATAGTTTAATTTCAGAAAG

							TATGAATGCGGCACAAAAACAGGGTTACGTTAAATATTTGTG<br>GCTAAGCAAAATCTTGAACTTATCATTAGTCCCAGCTTACGA<br>AGTACATAAAGCAATAAATGGACAAATATCACTATTGGATTT<br>TTTGAGATATATTTTAAAATGTCGCTTACAAAATGTATTTAAC<br>GAATTTCACGAATTGTCACCTGGTTAAAAAATTATTCAGTAG<br>ACAACAATATTCTTTTCGATGAAGTTAATCATTTTACAGAAA<br>ATGTTGGGAAACTACGTAATGTCTGCACTAAAAATACGCGAC<br>TGTCCTGTAATAAATATGATGCAATGTTACATAAATCTTTCTC<br>GATGACATTGCATTAGGAATAAACGATTTTACGATAGCAGTT<br>TAAAAAATTTTTGTCGAAAAATAAAATTATTATTCATGAAAT<br>GGAAGAAAAATGAAATAAAAATTTTGGTCACGAATCTGTAA<br>AACAGAAAAAAAAATATGCAATTTTAAAATATAGGAATATG<br>GGAACGTGTAAAGGGAAGCGTATGATGTCAAATTACGTGAC<br>ATGCTCTCATTTCCGGAGCATATCCCAAGATGTAAAATCAAT<br>GCAAATTTTAAGTAATTTTATTATTATATATAAATTGAAGTAT<br>AATCAGCTAGAGAAAGTACTTTCGAGAATCTTTGCCTACTGC<br>TTCTGAATGCAGCATTAGTTGGATTATATAAACTCTCCCCGTT<br>GTTTCCCCCTCAGGTCATACCGTAATAATATATACTGTGTACA<br>TCGGGAAGAAATAAAGATTCCTCTTGGTGAAAGGATTTCCAG<br>GTAGTTTCAGAGACTATTCATTACAAAGTTTTTTTCTCTTTATA<br>ATGTTAGTATAGAGAGTGAGACGTAAAAATATTTTTGACTCC<br>AAGATTTCTTATTGCAGATGTAGGAGGTCTGGAACGCAGACT<br>TCAGGAAACAGAACTCTGAGTTTGCGGAAAGCATTTTAAATA<br>CAAGCCATCATTTTCACCTCAAATGTAAAATCATAATAATAC<br>ATGTACGCTAATTTATTTTTTTATTGTTACATATCTTCCGTTGG<br>CGCATACCAAACATTTAATGCGAACAAAATCTGACCGTCAA<br>TTTTATCCCTTGTTTCTGCAGCGTTTGGTCAAGCAGGGCTTCT<br>CGCATTCCTTCTTCCAAGTGGTCCACGAACGTCGGTTCGGGC

							ACTCAAGCTTCTTCAAACATACACTGTATCCGGTTACTGAATTACCAACTGAGCACTTGCAACCCGATTTAATTCCAAAAAAATTATAACAGCTGCAATATGCATTATCTCGGCAGCACTGGCAGTCGTCTTTAGACCCATCGCAATCATTATTGAGTTCGATGCAGTCCCTTCTTTCAGTTCCGTGAGCTTCAAGAGGACTTTCCCCTTCTATCATTTCTTCCGCGAAGCTGAGTACGAAAGCAAAGCTTGCTACCAGAAATATCCCCAGAAGTTTCAT

PhdNtxCav 08	P84093	5,19E-43	91,139	PF08396	10320,91	LKFLCFRDCIELNND CDGSKDDCQCCRD NAYCSCYNFFGIKSG CKCSVGNSVTGYSV CLKKLECPNRRSWT TWKKECEKPCLTKR CRNKG*	TGTTTTCCCGGACATTTCCCCAAATTTTCTGATTTAGCAGGAC AAGTTCCCTGCTATTTCATATATATAATTTTACTATTAGTATTA AAGATACACACATATACGAAGGTATTTTAATTTGTAAATATT GAGGAGAAAAGTAGAAAAACGTTATGGCTCCGTAAGGTCCC ACTAAACTCCTGGAATCAAATAACTAAACTCCCGGAATCAA ATAAATTGACTCCTTAAGAAACTCATATAAGGCTGACATCTC TGCTAATTTGATTTATTTTAAATTAAGATAATATAATTTTAAC ATTTCAATCATAAATTGGTAAAAAGAAATAGTTTTATCTCCTT CGATTTGATTTTATAACCTGCTTCCAAAATTCGGGTGTTAGGT CATAATGGTATCAAAGATAGACGGCGCAACATAGCAAAAGA GAATAAAAAAGAATTCTAGCTACGTTTGTTTGAATGTTTGCG TTTCATTTTATGATTGCAGTGTAAGTAGCATGAAAATATAGG CTAACGTTATAGCGCATAAATATGAAGAAAATATATTTAGCG GGACTAAATGATTTATTCACACTAAACTGTGTATAAATTATT AACCAAAATATCTGACCCTGGATATTATACTCTTGTTTTCTTT ATTTTTTAGGATTCGTTAGAGGAATTTGTCAGGATTCTGAAAA CAGTTTGAGCGAAGAATGGTTTATGACAAACTGCCCATTACA GCCCTCAGTGTAAGGAACTTACGAGCCAAAAACGTTCAAGT TAGAAAAGAGCGAAAGTAGCAACTCTTGAAAAAGCCTTGGA ATATATGTGGCAGAAATTCGATGATAGAGAATTTTCAGGTGA GTTTAAAAATTATTTTTCGCCAGATTCTGGATCTGTTTGAGAC GAAAATACCGTCGATGATAATGAAATGTCTAATGTTAATTCG AAAGATATTTGTTCCTGGGGACTTTGAATTCCATATGAAATT AAAACGCGAGGCAAATTCTCTGTTATTAATACATTTAAGAGA AATCAAAATTATATCAATGGAATAAGGGAGAACAGATGATC ACATGATGGAAAATTGTTCAAATGCAAAATTTCAGGAAAAT CGATTGATTTATTAGCAAAATAACCCTTTGAATTATTTAAAAT ATTCTTTGGCCTAGAAATTCAAGATAGTTTAATTTCAGAAAG

							TATGAATGCGGCACAAAAACAGGGTTACGTTAAATATTTGTG<br>GCTAAGCAAAATCTTGAACTTATCATTAGTCCCAGCTTACGA<br>AGTACATAAAGCAATAAATGGACAAATATCACTATTGGATTT<br>TTTGAGATATATTTTAAAATGTCGCTTACAAAATGTATTTAAC<br>GAATTTCACGAATTGTCACCTGGTTAAAAAATTATTCAGTAG<br>ACAACAATATTCTTTTCGATGAAGTTAATCATTTTACAGAAA<br>ATGTTGGGAAACTACGTAATGTCTGCACTAAAAATACGCGAC<br>TGTCCTGTAATAAATATGATGCAATGTTACATAAATCTTTCTC<br>GATGACATTGCATTAGGAATAAACGATTTTACGATAGCAGTT<br>TAAAAAATTTTTGTCGAAAAATAAAATTATTATTCATGAAAT<br>GGAAGAAAAATGAAATAAAAATTTTGGTCACGAATCTGTAA<br>AACAGAAAAAAAAATATGCAATTTTAAAATATAGGAATATG<br>GGAACGTGTAAAGGGAAGCGTATGATGTCAAATTACGTGAC<br>ATGCTCTCATTTCCGGAGCATATCCCAAGATGTAAAATCAAT<br>GCAAATTTTAAGTAATTTTATTATTATATATAAATTGAAGTAT<br>AATCAGCTAGAGAAAGTACTTTCGAGAATCTTTGCCTACTGC<br>TTCTGAATGCAGCATTAGTTGGATTATATAAACTCTCCCCGTT<br>GTTTCCCCCTCAGGTCATACCGTAATAATATATACTGTGTACA<br>TCGGGAAGAAATAAAGATTCCTCTTGGTGAAAGGATTTCCAG<br>GTAGTTTCAGAGACTATTCATTACAAAGTTTTTTTCTCTTTATA<br>ATGTTAGTATAGAGAGTGAGACGTAAAAATATTTTTGACTCC<br>AAGATTTCTTATTGCAGATGTAGGAGGTCTGGAACGCAGACT<br>TCAGGAAACAGAACTCTGAGTTTGCGGAAAGCATTTTAAATA<br>CAAGCCATCATTTTCACCTCAAATGTAAAATCATAATAATAC<br>ATGTACGCTAATTTATTTTTTTATTGTTACATATCTTCCGTTGG<br>CGCATACCAAACATTTAATGCGAACAAAATCTGACCGTCAA<br>TTTTATCCCTTGTTTCTGCAGCGTTTGGTCAAGCAGGGCTTCT<br>CGCATTCCTTCTTCCAAGTGGTCCACGAACGTCGGTTCGGGC

							ACTCAAGCTTCTTCAAACATACACTGTATCCGGTTACTGAATT<br>ACCAACTGAGCACTTGCAACCCGATTTAATTCCAAAAAAATT<br>ATAACAGCTGCAATATGCATTATCTCGGCAGCACTGGCAGTC<br>GTCTTTAGACCCATCGCAATCATTATTGAGTTCGATGCAGTCC<br>CTGAAACATAAGAATTTTAATCAGAAGAGATATAATTAAATT<br>ATGTATCCGCAGTATACGGTCTCAAGTAATCTCTCTAATCCTT<br>TGAATACAAAAGAGATCTGCCATCGGTTACACTGTAAAGTA<br>GAAAATTAGTTCCCTTGTACCAGTTATTGTCTGATTCTTCAAC<br>GACACTATTGTTCAGAAGTAAGAGCGTAACCAGACCTCGAA<br>TTGAAATGAAAAGGAGTGGAGCGTCGCCTATACGTTGTTGTC<br>GACGCCTGAGAGAGAAGAAAAGCTTAACTGTGAATAATGTG<br>TAAATAAATTTTAAGTTGGACCAACCTAGAACCCCATTAATA<br>TAATTTCTGTGTAAACGTGTATGGATAGAAAAAATAAAATAT<br>TTGTGCTTAACACCGATGATACAATATAACAAGAATTAACTT<br>CCGAATG

PhdNtxCav 09	P81790	2,85E-14	43,023	PF08396	12907.25 **9973.58**	MTMKRFSVLALTFTAMLNFFIVIAKG**DSIVGLGLEGERSCLKLDSPCDPDKGDCQCCRSNAFCGCALGYKVFGNCKCRVGDVRTCRKKQEKCKNWRAWTNYPIQCKRPPLKPYY***	TCACTGTTTAGTTGTTTATTTGTATTTCCAGCAGTGTGGATTACATATAGTCAAATGCACACAAAATGCCGCGAGAGACAGTTTTATCAGTAATAAGGCTTCAGAGGTGGGCGTTTGCATTGTATAGGATAGTTCGTCCAAGCTCGCCAGTTTTTGCACTTCTCCTGCTTTTTCCTGCAAGTTCTGACATCGCCGACTCTGCACTTGCAGTTTCCGAAGACTTTATACCCCAACGCACAACCACAAAACGCGTTACTCCGGCAGCACTGACAGTCGCCTTTATCAGGATCGCAGGGAGAGTCGAGCTTCAGACATGACCTTTCTCCCTCTAATCCTAAACCAACAATACTATCTCCTTTTGCAATAACTATGAAGAAATTTAACATCGCAGTGAACGTTAAAGCCAATACACTAAACCGTTTCATTGTCATTGCCGTTACTCGCGGTGCACCTGTCAGGTGATTCCCCCTCCAGCTGCTCAGATTTTTCGGCCTTG

PhdNtxCav 10	O76200	1,55E-09	92,000	PF02819	2799.41<br>**427.42**	MWFKIQVLVLAITLL TIGIQA**EPNS**	TGAATTTGGTTCAGCCTGTATTCCAATCGTAAGCAAAGTGAT<br>AGCAAGCACAAGCACTTGTATTTTAAACCACATTGTAAAAG<br>GTATTGGAGATTTCTGCAAAAATAAATTTATACTGTTTACATA<br>AATTGTTAGTAAGTACGAATATGCATCAAAAACATAATAAG<br>GAAATCTCAAATATTTTGCATCTTAAATATTATTTTATCGCTTT<br>GCAGTCAATGTTACGCCGAAGAGAACACAATTTTCAGATGA<br>AGTGTAAAATTTCTTCCTTAATTTCAAATATTAGGATCCTATT<br>AATGGAATTTTATCGTAGAAAAAAATAGAATGGCTTGACTTC<br>TTCTCTGCGCTTTACCTTTAATTACAGTTGGCCAGCCTGGCAT<br>ATAGACTAGAGTCTTTTTTATTATCACAGATATTTCCATTATG<br>ATGCCATTATGACATATTACAACGCTTGGTAATAAACTGCTG<br>ATTCTTATTATATTACATCGAAAATGATGAAAAAGATTATTA<br>TACCATCTCCTTGCACCACAATAATTCCAAAGTGATAAATAT<br>TTTGAGCAACATATACAAAGCATTAAAGCAAAATTGTAACA<br>GTTAATTATGAAATATATATTATTCAGAATACACGACCATCC<br>TATAATATTGTGCACCGGAAGTCAAAATAATATATTATTAAT<br>TAAAACTTAACTACCTCTAAGTTGGAGAGTGTTATTTGGTATT<br>TTTTCATTTAGACAATATCTCAATAACATTTCAGGTTAAATAT<br>CAAACCAAAGAAATATCTTTAATGTTTTCAATAACTGTATCT<br>CTCTCCTTACGATTAAAAACGCAATTTCATATAATTGTCGATA<br>TTATAAATGGTTTCATACCTCAAAATATAAAATACATTTTTTT<br>TCTTCTATAATTTTAATATCATCATGTTTTAATGCAGTCGAAA<br>ATGTACTAGGGTGCAAAGTTTTGCATTATTTCAGTAGGATTGC<br>CATGTTGTCTAAATGTGATAAAGTAAAGGTTAAATCGAAAG<br>AACAGAGTTTGAATGAAAGCATACTGTTTTTTAACTTCGTTTC<br>AGTTCTTTTCGTTTATCAGTTTGATTTTTAGTCAAATTTTAAAT<br>TTATTGGAGTAAGAGCAGGATAATTTACTGAGGCAATCAATC<br>ATTCAGAAATTCAGAAATCATTCGTCTGTAAATGACACTCAT

							TTTTCGCTAACCATCATAGTCTCATCATAGTCTCTATGAATGATTTTCTGTACATGACAAATATAATGTGCACAACCGCCAGTAAGTGCACATCTGGACACAAAAAATTTTTCTGTCTAGATACATGTGGGTTATAGGCAATAGTCTGCCAACAATAATAAAATCAATTGCATCAAGAAATTAGAAATAATTGCAGTTCTTAACTGATTTGTAAGGTATATAAATTCATTTCTACATGTTATACGTACATATTTTATAAAATCGTAGTAATAAGATAAAATATTGTTAGCTATTATCAGCAATCAATTTATACGTTTTGATAATGAGTATAATGTAAAACAAAATATAATTATTTTGAAATACATAGATGTTCAGGCAGAA

PhdNtxCav 11	O76200	1,64E-09	92,000	PF02819	2799.41<br>**427.42**	MWFKIQVLVLAITLLTIGIQA**EPNS**	TGAATTTGGTTCAGCCTGTATTCCAATCGTAAGCAAAGTGATAGCAAGCACAAGCACTTGTATTTTAAACCACATTGTAAAAGGTATTGGAGATTTCTGCAAAAATAAATTTATACTGTTTACATAAATTGTTAGTAAGTACGAATATGCATCAAAAACATAATAAGGAAATCTCAAATATTTTGCATCTTAAATATTATTTTATCGCTTTGCAGTCAATGTTACGCCGAAGAGAACACAATTTTCAGATGAAGTGTAAAATTTCTTCCTTAATTTCAAATATTAGGATCCTATTAATGGAATTTTATCGTAGAAAAAAATAGAATGGCTTGACTTCTTCTCTGCGCTTTACCTTTAATTACAGTTGGCCAGCCTGGCATATAGACTAGAGTCTTTTTTATTATCACAGATATTTCCATTATGATGCCATTATGACATATTACAACGCTTGGTAATAAACTGCTGATTCTTATTATATTACATCGAAAATGATGAAAAAGATTATTATACCATCTCCTTGCACCACAATAATTCCAAAGTGATAAATATTTTGAGCAACATATACAAAGCATTAAAGCAAAATTGTAACAGTTAATTATGAAATATATATTATTCAGAATACACGACCATCCTATAATATTGTGCACCGGAAGTCAAAATAATATATTATTAATTAAAACTTAACTACCTCTAAGTTGGAGAGTGTTATTTGGTATTTTTTCATTTAGACAATATCTCAATAACATTTCAGGTTAAATATCAAACCAAAGAAATATCTTTAATGTTTTCAATAACTGTATCTCTCTCCTTACGATTAAAAACGCAATTTCATATAATTGTCGATATTATAAATGGTTTCATACCTCAAAATATAAAATACATTTTTTTTCTTCTATAATTTTAATATCATCATGTTTTAATGCAGTCGAAAATGTACTAGGGTGCAAAGTTTTGCATTATTTCAGTAGGATTGCCATGTTGTCTAAATGTGATAAAGTAAAGGTTAAATCGAAAGAACAGAGTTTGAATGAAAGCATACTGTTTTTTAACTTCGTTTCAGTTCTTTTCGTTTATCAGTTTGATTTTTAGTCAAATTTTAAATTTATTGGAGTAAGAGCAGGATAATTTACTGAGGCAATCAATCATTCAGAAAGGCAGAAATCATTCGTCTGAAAATCACACAAT

							TTTTTTCGCTAATAATCAATGATTTTCTGTACATGGCAAATAT AATGTGCACAGCCGCCAGTAAATGCACATCTGAACACAAAC ATTTTTTTTGTCGACATACATGTGGGTTTTAGTCAATGGTCTGT AAAAAAGAATAAAACCAATTGCATGGACAACTCACAAATA ATTGCATTTCGGAACTGGTTTATAAAATATATAAATTCATTTT TGCACGTTATACGTACATATTTTATAAAAGCATAGTAATAAA ACAATAAGTCTGAAATTATTGTTAACTATCAACTGTAATCAG TTTATATGTTTTGATAATGATTATAATCTACAATAAAGAATAA TTATTCTGAAATACATAGATATTCAGGCAGAAGATAAATCTC TCAATTATTATATAAATTATCAAGAATTATATATTGGGTCCAA ACTATGAAAAAATATTTAGCAAAAGTTGCTGATTGAAATCC

PhdNtxCav 12	P81793	8,66E-26	78,182	PF02819	6176.25<br>**3836.30**	MWLKVQVFVLAITLITLGIQA**EPNSGPNIPLIQEEARACARAWTECSFPEKRCCE**	TTTTCACAGCAACGTTTCTCAGGAAACGAGCACTCCGTCCAA<br>GCACGTGCACATGCTCTTGCTTCTTCTTGAATCAGTGGGATGT<br>TTGGACCGGAATTTGGTTCAGCCTGTATTCCGAGCGTAATCA<br>AAGTGATAGCCAGCACAAACACTTGCACTTTAAGCCACATTG<br>TAAAACGTTATGGAGGTTCCACAGTTGCTGATTGAAATCCTC<br>AAATATTTCTTCCTTCTTTTTATTTTCGTAAGTTAAATTCTTAG<br>GGTATTAAAGTAAAATAATAAAGAATAATTTTAATCAGAAT<br>GTTAACTTGACAATAAAGAGATTTTAAGTACGAACTACTACC<br>TGGTGTAAAGCTGTGCCAAAGGGAATAATTCTTTACTCAGGA<br>ATAAATCACAATTAGAATAATTTTCGTGCTTTCTTAAATACTC<br>TTGAAGAAGATATTTTTCATTATCCAATGAAGCTAAAATATA<br>ATTGAAAAATAGTTAAGTGAATTGCAGGCCAGATGGATTTGA<br>TTGAAGGAAAATCTGTATAAGCAGATGTGCAGCTTTAGATAC<br>GTTTCATGCAA
PhdNtxCav 13	P81793	6,99E-20	63,636	PF02819	6115.27<br>**3482.96**	MWLKVQVFVLVITLIMFGIQAEA**NLGASIPLNKEDARECAGLYKECSSPEKPCCR**	TCTACAGCAAGGTTTCTCAGGAGACGAGCACTCCTTATAAAG<br>ACCTGCACATTCTCTAGCGTCTTCTTTGTTCAGTGGGATACTT<br>GCACCTAAATTTGCTTCAGCCTGTATTCCAAACATAATCAAA<br>GTGATAACAAGCACAAACACTTGTACTTTAAGCCACATTGTA<br>AAACGTTATGGAGGTTCCACAGTTGCTGATTGAAATCCTCAA<br>ATATTTCTTCCTTCTTTTTATTTTCGTAAGTTAAATTCTTAGGG<br>TATTAAAGTAAAATAATAAAGAATAATTTTAATCAGAATGTT<br>AACTTGACAATAAAGAGATTTTAAGTACGAACTACTACCTGG<br>TGTAAAGCTGTGCCAAAGGGAATAATTCTTTACTCAGGAATA<br>AATCACAATTAGAATAATTTTCGTGCTTTCTTAAATACTCTTG<br>AAGAAGATATTTTTCATTATCCAATGAAGCTAAAATATAATT<br>GAAAAATAGTTAAGTGAATTGCAGGCCAGATGGATTTGATTG<br>AAGGAAAATCTGTATAAGCAGATGTGCAGCTTTAGATACGTT<br>TCATGCAA

PhdNtxCav 14	P81793	1,68E-20	63,636	PF02819	6115.27<br>**3482.96**	MWLKVQVFVLVITLIMFGIQAEA**NLGASIPLNKEDARECAGLYKECSSPEKPCCR**	TCTACAGCAAGGTTTCTCAGGAGACGAGCACTCCTTATAAAGACCTGCACATTCTCTAGCGTCTTCTTTGTTCAGTGGGATACTTGCACCTAAATTTGCTTCAGCCTGTATTCCAAACATAATCAAAGTGATAACAAGCACAAACACTTGTACTTTAAGCCACATTGTAAAACGTTATGGAGGTTCCTGGTGTAAAGCTGTGCCAAAGGGAATAATTCTTTACTCAGGAATAAATCACAATTAGAATAATTTTCGTGCTTTCTTAAATACTCTTGAAGAAGATATTTTTCATTATCCAATGAAGCTAAAATATAATTGAAAAATAGTTAAGTGAATTGCAGGCCAGATGGATTTGATTGAAGGAAAATCTGTATAAGCAGATGTGCAGCTTTAGATACGTTTCATGCAA
PhdNtxCav 15	P81793	4,14E-21	63,636	PF02819	6115.27<br>**3482.96**	MWLKVQVFVLVITLIMFGIQAEA**NLGASIPLNKEDARECAGLYKECSSPEKPCCR**	TCTACAGCAAGGTTTCTCAGGAGACGAGCACTCCTTATAAAGACCTGCACATTCTCTAGCGTCTTCTTTGTTCAGTGGGATACTTGCACCTAAATTTGCTTCAGCCTGTATTCCAAACATAATCAAAGTGATAACAAGCACAAACACTTGTACTTTAAGCCACATTGTAAAACGTTATGGAGGTTCCTGGTGTCAAGCTGTGCCAAAGGTAATATTTTTTGCTTACGAATAAATCACTCCAGTCATGTTTCAATAATATCTTGCAATGATGCTTACATATCTTAGA
PhdNtxCav 16	P81793	4,14E-27	78,182	PF02819	6176.25<br>**3836.30**	MWLKVQVFVLAITLITLGIQA**EPNSGPNIPLIQEEARACARAWTECSFPEKRCCE**	TTTTCACAGCAACGTTTCTCAGGAAACGAGCACTCCGTCCAAGCACGTGCACATGCTCTTGCTTCTTCTTGAATCAGTGGGATGTTTGGACCGGAATTTGGTTCAGCCTGTATTCCGAGCGTAATCAAAGTGATAGCCAGCACAAACACTTGCACTTTAAGCCACATTGTAAAACGTTATGGAGGTTCCTGGTGTCAAGCTGTGCCAAAGGTAATATTTTTTGCTTACGAATAAATCACTCCAGTCATGTTTCAATAATATCTTGCAATGATGCTTACATATCTTAGA

PhdNtxCav<br>17	O76201	3,22E-08	88,462	PF02819	4419.28<br><br>**2122.29**	MCLKIQVFLLAIILIT<br>LGIQA**EPDSSMYHK<br>TQISLNEKD***	AAATATCTTCTCCAAGAGTATATATGTATCCGAAAAAATTAT<br>TCCATTAGCGAACTATCCTTGAGCAAAGAATATATTCTATTTG<br>GCACAGATTTACACCAGAAACCTCCAATACATTTTACAATGT<br>GCCTTAAAATACAAGTATTTCTGCTTGCTATCATTTTGATTAC<br>GCTTGGAATACAGGCTGAACCAGATTCAAGTATGTATCATAA<br>AACCCAAATTTCACTTAATGAAAAGGATTAGATTTGGTATAA<br>AATCATAGTAGTAAGTTTCAGAAAATGAAAGTTCCCAGGTCA<br>GAATTTCAGATGAATTTTTCGCACTACGCGTTGAGATTGCAA<br>GTAATGGCGTTCGGACTAAATAACAACGTAACTAATATAATT<br>AATGTGTAAATTTCTTACAGGACAATTAGAAGCATTGCTTTTT<br>GCGCACACACTCATACACACACACACACAGAGAGAGAGAG<br>AGAGAGAGAGAGAGAGAGAGAGAGAGAGAGA
PhdNtxCav<br>18	O76201	9,20E-42	93,939	PF02819	8925.63<br><br>**6557.62**	MWLKIQVFLIAITLIT<br>LGIQA**EPNSNPSNPL<br>IQEEARACAGLYKK<br>CGKGASPCCEDRPC<br>KCDLAMGNCICKK<br>KFIEFFGGGK***	GCCTGCAATTCACTTAACTATTTTACAATTATGTTTTACTTTCA<br>TTGGATAATGAAAAATACCTTCTCCAAGAGTATATAAGTATC<br>TGGAAAAATTATTCCATTAGCGAACTATCCCTGAGCAAAGA<br>ATATATTCTCTTTGGCACAGATTTACAACAGAAACCTCCAAT<br>ACATTTTACAATGTGGCTTAAAATACAAGTATTTCTGATTGCT<br>ATCACTTTGATTACGCTTGGAATACAAGCTGAACCAAATTCA<br>AATCCAAGCAACCCACTGATTCAAGAAGAAGCCAGAGCATG<br>TGCAGGTCTTTATAAGAAATGCGGGAAAGGTGCGAGTCCTTG<br>CTGTGAAGATCGACCATGTAAATGCGACCTTGCTATGGGCAA<br>CTGCATATGCAAAAAGAAATTTATCGAGTTCTTTGGAGGAGG<br>AAAATGAAACATCAAGAACGGCAGAATGAAGGTTTAATTAT<br>TTTAATAGCTAAGTAATATGATAATAATGAACAAGAATATGT<br>ATCAGGAGAAAACAATAATGTAAGCTATAAGAATATAGCTT<br>ATCTAAATAAAAATATGTTTTAAATGCAAAAAAAAAAAAAA<br>AAAAAAAAA

PhdNtxCav 19	O76201	2,57E-38	89,394	PF02819	8851.53<br><br>**6554.55**	MCLKIQVFLLAIILITLGIQA**EPDSSPNNSMTQEEARACAGLYKKCGKGASPCCEDRPCKCDLAMGNCICKKKFIEFFGGGK***	AAATATCTTCTCCAAGAGTATATATGTATCCGAAAAAATTATTCCATTAGCGAACTATCCTTGAGCAAAGAATATATTCTATTTGGCACAGATTTACACCAGAAACCTCCAATACATTTTACAATGTGCCTTAAAATACAAGTATTTCTGCTTGCTATCATTTTGATTACGCTTGGAATACAGGCTGAACCAGATTCAAGTCCAAACAACTCAATGACTCAGGAAGAAGCCAGAGCATGTGCAGGTCTTTATAAGAAATGCGGGAAAGGTGCGAGTCCTTGCTGTGAAGATCGACCATGTAAATGCGACCTTGCTATGGGCAACTGCATATGCAAAAAGAAATTTATCGAGTTCTTTGGAGGAGGAAAATGAAACATCAAGAACGGCAGAATGAAGGTTTAATTATTTTAATAGCTAAGTAATATGATAATAATGAACAAGAATATGTATCAGGAGAAAACAATAATGTAAGCTATAAGAATATAGCTTATCTAAATAAAAATATGTTTTAAATGCAAAAAAAAAAAAAAAAAAAAAAAA
PhdNtxCav 20	O76201	5,12E-14	100,000	PF02819	5874,98	YPTSFIHYRCAGLYKKCGKGASPCCEDRPCKCDLAMGNCICKKKFIEFFGGGK*	GTTACAAATATTCATTGATATATTACAATAAATAATTTAACGAATGGAGAATACTAGAGTAACATTTTGTAAATATTGCTTCTCGGTGTAAATTAAGTTGTGGAATGTAGATTATGATATCCAACTTCATTTATCCATTATAGATGTGCAGGTCTTTATAAGAAATGCGGGAAAGGTGCGAGTCCTTGCTGTGAAGATCGACCATGTAAATGCGACCTTGCTATGGGCAACTGCATATGCAAAAAGAAATTTATCGAGTTCTTTGGAGGAGGAAAATGAAACATCAAGAACGGCAGAATGAAGGTTTAATTATTTTAATAGCTAAGTAATATGATAATAATGAACAAGAATATGTATCAGGAGAAAACAATAATGTAAGCTATAAGAATATAGCTTATCTAAATAAAAATATGTTTTAAATGCAAAAAAAAAAAAAAAAAAAAAAAA

PhdNtxCav 21	O76200	1,05E-17	97,059	PF02819	5055,94	YPTSFIHCRCADVYERCGKGYKRCCEERPCKCNIVMDNCTCKK	TTTTTTGCACGTACAGTTGTCCATAACGATATTGCATTTGCATGGTCGTTCTTCACAGCAACGCTTGTAACCTTTTCCGCATCTCTCATAAACATCTGCGCATCTACAATGTATAAATGAAGTAGGATATCATAATCAACATTCCACAACTTAATTTAAACCGAGAAGCAATATTTACAAAATCTTACTCTAGTATTCTGCATTCGTGAAATTCATTGTTGCAATATATCAATGAATATTTGTAACATGGCAATAGAATAATTTATAACATATGAATGAAGAAGTATAATGTATATATTTTCAGTTAGTCACACATATACAATAAAGAATTTCTTGCTATTAAAATGACCTTTATTTCACCTATTTCTATGAACGGCTTCGAGTTTTCACATATTATTTATGAGAAATGGTTTGCATTCCTTTTACAGTTAAATTTATATGATAGAAATCTCGACGAAATTAGTTTAGTGACATGTATTATTCTATTTCTGAATGTTTGGAGATGCTAATTTTTTCATTTAA
PhdNtxCav 22	O76200	1,64E-16	97,059	PF02819	5055,94	YPTSFIHCRCADVYERCGKGYKRCCEERPCKCNIVMDNCTCKK	TTTTTTGCACGTACAGTTGTCCATAACGATATTGCATTTGCATGGTCGTTCTTCACAGCAACGCTTGTAACCTTTTCCGCATCTCTCATAAACATCTGCGCATCTACAATGTATAAATGAAGTAGGATATCATAATCAACATTCCACAACTTAATTTAAACCGAGAAGCAATATTTACAAAATCTTACTCTACTATTCTCTATTCGTGAGAATCATTATGGTAATATATCAATGAATATTTATAACATGTGAATAGAATATTTTATAACACATTAATGAAGCAGTATAATGTTTATAATTTCAGTTATTCACACATATACAATTAAGAATTTATTGCTATTAAAATTATCTTTATTTCACCTATTTCTATGAACAGCTTCGAGTATTCATGTATTATTTATCAGAAATTGTTTCCATTCTTTTTGCACCAAAACAATATGATAGAAATCCCGACGAAATTAGTCTACTGGCATGTGTTATTCCATTTCTGAATGATTAGAGATGCTAATTTTTTCATTTAATTGAAAGATATTGAGTTTTTCTTCTGTTATTTCCCTTCCAATTTGTATTTTTAATTAGCCAGTTTCGTAATCATTCTGTAAAATGTGGATTATCGATAACCATTATTAAGAGCATCGCTT

							TGATCTATTCTTACTGTTTAAGTCAGAAATAACGGAAATAAG<br>GATTAAAAGAGAATGAAACTGATGAGAAGACCGGGTCCATT<br>AGCTGCATTTATTGTAAATTCATACAAAAATCTCCATACATTT<br>CCATATAACAATTCATATACTTTAGATCAATAGATTATAAGA<br>GACATGCGAGAAAGGCTGTTGAAAAAATGTGCATGTATTTAT<br>TTTTCTTTCCGGATTAAAACCATACAATCTGGCATATAGGTAT<br>TTCCCCATACATTTATTTTTTTGAATAGCCTTAAAATGTTTTAT<br>ATTAAATTGTTTCATTCATAGAAATTCATTTCTGAAAATTCCG<br>TTTTGTGGGAACTTACAGGAAAAAAAATATTTTGGAATGCAT<br>AATGCCCACATCTTGATCTTCATCCATAATTAACAAAATAAT<br>TTACAGAATATTAGTATGAAACA
PhdNtxCav<br>23	O76200	2,11E-11	52,459	PF02819	3554.28<br><br>**1200.31**	MWFKIQVLVLAITLI<br>TIGIQA**EPNSSMYR**<br>**NC***	TATATATATATATATATATATATATATATATATATATATAT<br>ATGTGTGTGTGTGTGTGTAAACATTCATATGTGTGTGTGTG<br>CAATCAGCATTGTCTCTAATTTTCCTTGAAGGCATTTAGAAAT<br>AATACACGAAAATTACATTAGTTACCTTGCAAGGATGGACGT<br>TCGGTCTAAATAACAATCTCAACGCGTAGCCCGAAAAATTA<br>ATCTAAAATTCTGAAAATGTCAGATCTAGGAAATTTTTTTCTA<br>ACACTTTCTACTATGATTTTATACCAATTCTCATCCTTTTCATT<br>AAGTGGAATTTAGCAATTGCGATACATACTTGAATTTGGTTC<br>AGCCTGTATTCCAATCGTAATCAAAGTGATAGCAAGCACAA<br>GCACTTGTATTTTAAACCACATTGTAAAAGGTATTGGAGATT<br>TCTGGTGTAAAGATGTGCCAAAGAGGATATATTATCTGCTCA<br>GGAATAGTTCGCTCATGGAATAATTTTTTTTGGATTCTTATAT<br>ACTCTTGGAGAAGATATTTTTCTTTATCCTAGGTAGTTGAAAT

							ATAATAGCACAATACTTACGCGAATTACGTGCCAGGATGGAG
PhdNtxCav 24	O76200	1,22E-11	52,459	PF02819	6410,71	MLIQIFFQSTPSWHVIRKSPIPFTMWFKIQVLVLAITLITIGIQAEPNSSMYRNC*	TATATATATATATATATATATATATATATATATATATATATATATATATATATGTGTGTGTGTGTGTGTAAACATTCATATGTGTGTGTGTGTGCAATCAGCATTGTCTCTAATTTTCCTTGAAGGCATTTAGAAATAATACACGAAAATTACATTAGTTACCTTGCAAGGATGGACGTTCGGTCTAAATAACAATCTCAACGCGTAGCCCGAAAAATTAATCTAAAATTCTGAAAATGTCAGATCTAGGAAATTTTTTTCTAACACTTTCTACTATGATTTTATACCAATTCTCATCCTTTTCATTAAGTGGAATTTAGCAATTGCGATACATACTTGAATTTGGTTCAGCCTGTATTCCAATCGTAATCAAAGTGATAGCAAGCACAAGCACTTGTATTTTAAACCACATTGTAAAAGGTATTGGAGATTTGCGAATTACGTGCCAGGATGGAGTTGATTGAAAGAAAATCTGTATAAGCATATGTGCGGCCTTATACACTTTTCTTCTAAATTCATATTATAATCACCGAGAATATCTTCAGTGACATTTGAATATATATTCTAGAAGGACTTCAAAATACATGACAGAAACGTAGGCTAAAATTAAAAAAAA

PhdNtxCav 25	O76200	4,01E-54	95,181	PF02819	9366.98<br>**7013.01**	MWFKIQVLVLAITLITGIQA**EPNSSPNNPLIVEEDRGSCADVYERCGKGYKRCCEERPCKCNIVMDNCTCKKFISELFGFGK***	CTTATAGCTTACAAGAAAATGTATAATAAGATAACATTAAAT<br>TTCATTATTATCATATTACTTAGGTATTAAAATATTTAAATTT<br>ACATTCTGCAGGTTTAAATTGCCATATTTGGTGTTTCATTTTCC<br>GAATCCAAACAGCTCGCTTATAAATTTTTTGCACGTACAGTT<br>GTCCATAACGATATTGCATTTGCATGGTCGTTCTTCACAGCAA<br>CGCTTGTAACCTTTTCCGCATCTCTCATAAACATCTGCGCATG<br>ACCCTCTATCTTCTTCTACAATCAGTGGGTTGTTTGGACTTGA<br>ATTTGGTTCAGCCTGTATTCCAATCGTAATCAAAGTGATAGC<br>AAGCACAAGCACTTGTATTTTAAACCACATTGTAAAAGGTAT<br>TGGAGATTTCTGGTGTAAAGATGTGCCAAAGAGGATATATTA<br>TCTGCTCAGGAATAGTTCGCTCATGGAATAATTTTTTTGGATT<br>CTTATATACTCTTGGAGAAGATATTTTTCTTTATCCTAGGTAG<br>TTGAAATATAATAGCACAATACTTACGCGAATTACGTGCCAG<br>GATGGAGTTGATTGAAAGAAAATCTGTATAAGCATATGTGCG<br>GCCTTATACACTTTTCTTCTAAATTCATATTATAATCACCGAG<br>AATATCTTCAGTGACATTTGAATATATATTCTAGAAGGACTTC<br>AAAATACATGACAGAAACGTAGGCTAAAATTAAAAAAAA

PhdNtxCav 26	O76200	2,02E-16	97,059	PF02819	5055,94	YPTSFIHCRCADVYE RCGKGYKRCCEERP CKCNIVMDNCTCKK	TTTTTTGCACGTACAGTTGTCCATAACGATATTGCATTTGCAT GGTCGTTCTTCACAGCAACGCTTGTAACCTTTTCCGCATCTCT CATAAACATCTGCGCATCTACAATGTATAAATGAAGTAGGAT ATCATAATCAACATTCCACAACTTAATTTAAACCGAGAAGCA ATATTTACAAAATCTTACTCTACTATTCTCTATTCGTGAGAAT CATTATGGTAATATATCAATGAATATTTATAACATGTGAATA GAATATTTTATAACACATTAATGAAGCAGTATAATGTTTATA ATTTCAGTTATTCACACATATACAATTAAGAATTTATTGCTAT TAAAATTATCTTTATTTCACCTATTTCTATGAACAGCTTCGAG TATTCATGTATTATTTATCAGAAATTGTTTCCATTCTTTTTGCA CCAAAACAATATGATAGAAATCCCGACGAAATTAGTCTACT GGCATGTGTTATTCCATTTCTGAATGATTAGAGATGCTAATTT TTTCATTTAATTGAAAGATATTGAGTTTTTCTTCTGTTATTTCC CTTCCAATTTGTATTTTTAATTAGCCAGTTTCGTAATCATTCTG TAAAATGTGGATTATCGATAACCATTATTAAGAGCATCGCTT TGATCTATTCTTACTGTTTAAGTCAGAAATAACGGAAATAAG GATTAAAAGAGAATGAAACTGATGAGAAGACCGGGTCCATT AGCTGCATTTATTGTAAATTCATACAAAAATCTCCATACATTT CCATATAACAATTCATATACTTTAGATCAATAGATTATAAGA GACATGCGAGAAAGGCTGTTGAAAAAATGTGCATGTATTTAT TTTTCTTTCCGGATTAAAACCATACAATCTGGCATATAGGTAT TTCCCCATACATTTATTTTTTTGAATAGCCTTAAAATGTTTTAT ATTAAATTGTTTCATTCATAGAAATTCATTTCTGAAAATTCCG TTTTGTGGGAACTTACAGGAAAAAAATATTTTGGAATGCATA ATGCCCACATCTTGATCTTCATCCATAATTAACAAAATAATTT ACAGAATATTAGTATGAAACAGTTTTTAAGTTGAATCGGAAT ATAATTACGATATTTAAATAAGAAATAAAATTTGTGACTTAC

							GACCCTCTATCTTCTTCTACAATCAGTGGGTTGTTTGGACTTGAATTTGGTTCAGCCTGTA
PhdNtxCav 27	O76201	3,16E-25	78,182	PF02819	5039,94	MFHFPPPKNSINFFLYMQLPIARSHLHVRSSQQFLAPFPHFL*	GATGTTTCATTTTCCTCCTCCAAAAAACTCGATAAATTTCTTTTTGTATATGCAGTTGCCAATAGCAAGGTCGCATTTACATGTTCGATCTTCTCAGCAATTTCTCGCTCCTTTCCCGCATTTCTTATAAAGAACTGCACATGCTCTGTCTTCTTCTTGAATCAGTGGGTTGCATCGATTTGAATTTGTTTCAGCTTGTATTCCAAGCGTAAT
PhdNtxCav 28	O76200	1,36E-05	100,000	PF02819	4103,82	CAVRTALRYQLCILLFSGDVYERCGKGYKRCCEER	GTCGTTCTTCACAGCAACGCTTGTAACCTTTTCCGCATCTCTCATAAACATCTCCAGAAAAAAGCAAAATACATAGCTGATATCTTAAAGCTGTACGCACAGCACATCAAACTGCATCCTTCATGAGGATGTATAACTTTCTGGGTGCTAAAACTGTGTCTGACCTCTGCCAGACATAAGCTCTTTCTTTCCTAAACTAAAAATCTTCAAACACCACCCCTGTTGACTAAGAGAGACAGAGGAGGAAAGAAATGTCCACTCTGTTCACA

PhdNtxCav 29	O76200	3,09E-11	47,458	PF02819	8035,26	NGIKIFTINQGRNHP EIYGKENKTEEKCAP IYEKCDDSTKPCCD NRPCKCNIVKTSCM CKYTLGELFGRR*	AGAAAGATTAAATGGTTTAAGCTTCTGATACTTAATTTGCAA CTTTTATGCTATGATAACCGGAAGACAATTCTGAAATATGAA ATCACTTCCTTTACTGTATAAGATGCCTTCATGTTAAAACGGC ATTAAAATTTTCACGATAAATCAAGGTCGAAACCACCCAGA GATTTACGGAAAAGAGAATAAAACAGAAGAAAAGTGCGCA CCCATCTATGAGAAATGTGATGATTCTACAAAACCATGCTGC GATAACCGACCTTGCAAATGCAATATTGTGAAGACAAGTTGC ATGTGTAAGTATACACTGGGAGAGTTGTTTGGTAGACGATGA GGGTGAAGCAGTAGAAATAGGACTTTAAAATAGCAGGG
PhdNtxCav 30	O76201	1,62E-05	40,000	PF02819	2591,98	YFFRFGYGGKGASPC CEDRPCKC	TATATAACTAAATATGAAAATTTAATATTTCTTACGTAATCAC AATCAGAAAAACAAATACAACATGCAGAACAAAAAATCTT AAAAAATATTTTTAAGGGACAAAAATGCTAGATATCACTTTT TATTTCTACAACATGGAAGTTGCATAAAATTTCTAAATGTAT ATACAATTTTTCTTTTTAGGATTACTTTGGTCTCCCTATATAAA TTCAAGAATAAATTTTCCTTGCACGTTCAAAAAAAAAACTTT TTTTAATGATACTTTTTCAGATTTGGTTACGGGGGGAAAGGTG CGAGTCCTTGCTGTGAAGATCGACCATGTAAATGCG

PhdNtxCav 31	P81791	4,61E-28	92,453	PF10530	9560.06<br>**7680.54**	MKLCILLVVLLITVVRA**EEDIFENEAEEIAPAIKERSARGCIGHNESCKFDRHGCCWPWSCSCWNKEGQPESAVWCECNFKIGK***	GTTTGATATAAAACGGCAAAACGTTTACAAACATAAGTATATATATATATATTTGCTCAATATACCTGAGAATTACGAGGAAGAAATTTCTTAAGCGTGAACAATGAAGCTTTGTATTTTACTTGTTGTACTCTTGATAACGGTTGTACGAGCCGAAGAAGATATTTTCGAAAATGAAGCGGAAGAAATAGCACCCGCAATAAAAGAAAGAAGTGCCAGAGGATGCATCGGGCATAATGAGAGCTGCAAGTTCGACAGACATGGTTGTTGTTGGCCTTGGTCCTGTAGTTGTTGGAATAAGGAAGGACAACCTGAAAGTGCTGTGTGGTGCGAGTGCAATTTTAAGATAGGAAAGTAAAAAAATAAGAATCAAGATAAAAATTTTAATATGTTCCATGCTGATTATTATGCATATAAAAGAAATATTAAAGGCATTTAACAACATGTTTATTTCACACATATTTGTAACAAAAACACATTATATGATCTAGAGTGAATGTAATGCAGGTATAAATTTGGATTGAATGAAATATTTGCTTTAATCAAAATTTGACTTGCAAAGAAGTAGGCAAAATATTTACCTCATAATATGAAACAACGTTAAGCATGACAGTATAAAAACAATGATATTTTAAATTAAAAGACTAAGTAAAAGATGTTAAATATGTTGCAATATTTTAGGTAAAATTAGAGCTTGTGAAGATGCTGCCGTAATTAGAGTTCAAGCAAATTAATTTTTCTATAAGGAATTTTTCTCCATAATGTTCAATATACATTGTTTTTCAAGGAGTAAAATAAATTTTATTTTCTGCCTTAAAGAAGATTTCGGAGTAGAGATATTGATTATTATATATTTCAAATGGGGACATCCCTTAGTGTTTTAAGCTATAATTTTATATTAAAATTTAACTTGTTCAATAAATTTTTTGTTGAAAGGAAATATTTGCTTTGAAATTATTAATGCAGATTTTGTCGGTGTACAGTGAAAAGGAATTTTATCAGAATGTAGTCAGAAATAAATGCATTATCTTGAAAACAAATGAAGAGATTCAATCCATGTACAGATATGATGACTCAAAAAATTATTTTTGTGTGTGTAAATTTACGCTATTTTGGATGTGCAGAACGGACTGCTGCTTGTCACATATCAGCATTCTCAATCGAATGACTCCAATCGA

							ATTGACTCCAATCGAATATCACTTTGTAAGTTTATTTTTTTCA<br>ATTTTTGAATTATTTTGTCCCCAAATGTGAATTTTTTCATTAAT<br>AAAATTTTAAAGAATGAGCATCCATTGTTGAAGTTCAGAATG<br>CTCGATCGAAAATCGAGAGATTTGGATTCCAACCACTGGGTG<br>TTCACCGTATTTATCTGGATGAGGTGAGACGTACAAATAACA<br>CTCGCCATAATTACGTATGTGAAGTGCCTCCGTGGTGTTTAG<br>GCAACCCCGGACTCTCCTTGTAAAATAAATGTAATTAAAGAA<br>TGAGAACCCGTGGCACAACGTACAACATACTCGACCAGGAA<br>TCAAGAACCACGGGCTCTATCCATAGTGGGTGAAGTTATATT<br>TCAAGTGTGTGGATGAGTGAAACTTTATAAAATAAATTGCTT<br>TTTACAAATGTTAAAAATGATTCACCAAATGTAGTAAAACAA<br>CTTGAAGTGGTAGTTCTTCAAACCGTTGTTTCGCGAGTGCTCA<br>TGTTTCTTGAGAATTTCGTACATTTCCCTACTTTATGAAAAGT<br>TAGTTCCTCCTTCCATTCAATAATTCGTACGGTTACTGTGTAC<br>TTGTGAGCTAGCTGTAAGTAGGATAATTACAGCCTAATGGTA<br>AATGCAATTTTCTTTAAAAACCCCTTACTCAATCCAATAAGC<br>TTTCAATTCTTAATTCTTTGACTGTCGAGATATTCTTCGTGTAA<br>TTAAAAGAAAGCAAATCTTGAATCATTTAGTGTAAAACTATA<br>TTATATTTGCTCCTTTAGATATTTTTAATTTTGTTGGCTCCCATT<br>GGAAAAGTCGCTGTATCTTACGTATTTTCTTCTTTCTTTCGTAA<br>ATAACAATTATCATTGTAGATGTATTTATAAACATGTAATTTT<br>TAATTTAAAATAGAAAGGCTAATTACAAAGCTGATATTGAA<br>GAAACTTGCATGTTATTAACCAATTTGGGTTCCTTTGATAAAC<br>TAAAAAATCAAAGAAAC

PhdNtxCav 32	P81791	2,49E-25	82,692	PF10530	7871.91	DFHFLQFFFKFENTVFFYSARGCIGHNESCKFDRHGCCWPWSCSCWNKEGQPESAVWCECNFKIGK*	TGATTTTCATTTTCTCCAATTTTTTTTCAAGTTTGAAAATACAGTTTTCTTTTATAGTGCCAGAGGATGCATCGGGCATAATGAGAGCTGCAAGTTCGACAGACATGGTTGTTGTTGGCCTTGGTCCTGTAGTTGTTGGAATAAGGAAGGACAACCTGAAAGTGCTGTGTGGTGCGAGTGCAATTTTAAGATAGGAAAGTAAAAAAATAAGAATCAAGATAAAAATTTTAATATGTTCCATGCTGATTATTATGCATATAAAAGAAATATTAAAGGCATTTAACAACATGTTTATTTCACACATATTTGTAACAAAAACACATTATATGATCTAGAGTGAATGTAATGCAGGTATAAATTTGGATTGAATGAAATATTTGCTTTAATCAAAATTTGACTTGCAAAGAAGTAGGCAAAATATTTACCTCATAATATGAAACAACGTTAAGCATGACAGTATAAAAACAATGATATTTTAAATTAAAAGACTAAGTAAAAGATGTTAAATATGTTGCAATATTTTAGGTAAAATTAGAGCTTGTGAAGATGCTGCCGTAATTAGAGTTCAAGCAAATTAATTTTTCTATAAGGAATTTTTCTCCATAATGTTCAATATACATTGTTTTTCAAGGAGTAAAATAAATTTTATTTTCTGCCTTAAAGAAGATTTCGGAGTAGAGATATTGATTATTATATATTTCAAATGGGGACATCCCTTAGTGTTTTAAGCTATAATTTTATATTAAAATTTAACTTGTTCAATAAATTTTTTGTTGAAAGGAAATATTTGCTTTGAAATTATTAATGCAGATTTTGTCGGTGTACAGTGAAAAGGAATTTTATCAGAATGTAGTCAGAAATAAATGCATTATCTTGAAAACAAATGAAGAGATTCAATCCATGTACAGATATGATGACTCAAAAAATTATTTTTGTGTGTGTAAATTTACGCTATTTTGGATGTGCAGAACGGACTGCTGCTTGTCACATATCAGCATTCTCAATCGAATGACTCCAATCGAATTGACTCCAATCGAATATCACTTTGTAAGTTTATTTTTTTCAATTTTTGAATTATTTTGTCCCCAAATGTGAATTTTTTCATTAATAAAATTTTAAAGAATGAGCATCCATTGTTGAAGTTCAGAATGCTCGATCGAAAATCGAGAGATTTGGATTCCAACC

							ACTGGGTGTTCACCGTATTTATCTGGATGAGGTGAGACGTAC<br>AAATAACACTCGCCATAATTACGTATGTGAAGTGCCTCCGTG<br>GTGTTTAGGCAACCCCGGACTCTCCTTGTAAAATAAATGTAA<br>TTAAAGAATGAGAACCCGTGGCACAACGTACAACATACTCG<br>ACCAGGAATCAAGAACCACGGGCTCTATCCATAGTGGGTGA<br>AGTTATATTTCAAGTGTGTGGATGAGTGAAACTTTATAAAAT<br>AAATTGCTTTTTACAAATGTTAAAAATGATTCACCAAATGTA<br>GTAAAACAACTTGAAGTGGTAGTTCTTCAAACCGTTGTTTCG<br>CGAGTGCTCATGTTTCTTGAGAATTTCGTACATTTCCCTACTTT<br>ATGAAAAGTTAGTTCCTCCTTCCATTCAATAATTCGTACGGTT<br>ACTGTGTACTTGTGAGCTAGCTGTAAGTAGGATAATTACAGC<br>CTAATGGTAAATGCAATTTTCTTTAAAAACCCCTTACTCAATC<br>CAATAAGCTTTCAATTCTTAATTCTTTGACTGTCGAGATATTC<br>TTCGTGTAATTAAAAGAAAGCAAATCTTGAATCATTTAGTGT<br>AAAACTATATTATATTTGCTCCTTTAGATATTTTTAATTTTGTT<br>GGCTCCCATTGGAAAAGTCGCTGTATCTTACGTATTTTCTTCT<br>TTCTTTCGTAAATAACAATTATCATTGTAGATGTATTTATAAA<br>CATGTAATTTTTAATTTAAAATAGAAAGGCTAATTACAAAGC<br>TGATATTGAAGAAACTTGCATGTTATTAACCAATTTGGGTTCC<br>TTTGATAAACTAAAAAATCAAAGAAAC

PhdNtxCav 33	P81791	2,61E-28	92,453	PF10530	9560.06<br><br>**7680.54**	MKLCILLVVLLITVV<br>RA**EEDIFENEAEEIA**<br>**PAIKERSARGCIGH**<br>**NESCKFDRHGCCW**<br>**PWSCSCWNKEGQP**<br>**ESAVWCECNFKIGK**<br>*	GAAATTTCTTAAGCGTGGTAAGAACATGTTTATATTCCTCTTA<br>ATATAGAGTAAGACTTCAAGTAATTTAAACCATAAATTAATC<br>TGGAAGTATTATTCATGTTATAACTAAACAGCTATTTTAATTG<br>TTTCACATAATGATTAATTACAACGCAAGATTTCTTAACATA<br>AAAAATTTTTCACAATTAAACGCTCAGACAATTGAAGTTCAA<br>TCAAAGCAATGATGGATGTAACGAACTTATTGTGAATTCCCA<br>AACAATGAAGCTTTGTATTTTACTTGTTGTACTCTTGATAACG<br>GTTGTACGAGCCGAAGAAGATATTTTCGAAAATGAAGCGGA<br>AGAAATAGCACCCGCAATAAAAGAAAGAAGTGCCAGAGGA<br>TGCATCGGGCATAATGAGAGCTGCAAGTTCGACAGACATGG<br>TTGTTGTTGGCCTTGGTCCTGTAGTTGTTGGAATAAGGAAGGA<br>CAACCTGAAAGTGCTGTGTGGTGCGAGTGCAATTTTAAGATA<br>GGAAAGTAAAAAAATAAGAATCAAGATAAAAATTTTAATAT<br>GTTCCATGCTGATTATTATGCATATAAAAGAAATATTAAAGG<br>CATTTAACAACATGTTTATTTCACACATATTTGTAACAAAAA<br>CACATTATATGATCTAGAGTGAATGTAATGCAGGTATAAATT<br>TGGATTGAATGAAATATTTGCTTTAATCAAAATTTGACTTGCA<br>AAGAAGTAGGCAAAATATTTACCTCATAATATGAAACAACG<br>TTAAGCATGACAGTATAAAAACAATGATATTTTAAATTAAAA<br>GACTAAGTAAAAGATGTTAAATATGTTGCAATATTTTAGGTA<br>AAATTAGAGCTTGTGAAGATGCTGCCGTAATTAGAGTTCAAG<br>CAAATTAATTTTTCTATAAGGAATTTTTCTCCATAATGTTCAA<br>TATACATTGTTTTTCAAGGAGTAAAATAAATTTTATTTTCTGC<br>CTTAAAGAAGATTTCGGAGTAGAGATATTGATTATTATATAT<br>TTCAAATGGGGACATCCCTTAGTGTTTTAAGCTATAATTTTAT<br>ATTAAAATTTAACTTGTTCAATAAATTTTTTGTTGAAAGGAAA<br>TATTTGCTTTGAAATTATTAATGCAGATTTTGTCGGTGTACAG<br>TGAAAAGGAATTTTATCAGAATGTAGTCAGAAATAAATGCA

							TTATCTTGAAAACAAATGAAGAGATTCAATCCATGTACAGAT<br>ATGATGACTCAAAAAATTATTTTTGTGTGTGTAAATTTACGCT<br>ATTTTGGATGTGCAGAACGGACTGCTGCTTGTCACATATCAG<br>CATTCTCAATCGAATGACTCCAATCGAATTGACTCCAATCGA<br>ATATCACTTTGTAAGTTTATTTTTTTCAATTTTTGAATTATTTTG<br>TCCCCAAATGTGAATTTTTTCATTAATAAAATTTTAAAGAATG<br>AGCATCCATTGTTGAAGTTCAGAATGCTCGATCGAAAATCGA<br>GAGATTTGGATTCCAACCACTGGGTGTTCACCGTATTTATCTG<br>GATGAGGTGAGACGTACAAATAACACTCGCCATAATTACGT<br>ATGTGAAGTGCCTCCGTGGTGTTTAGGCAACCCCGGACTCTC<br>CTTGTAAAATAAATGTAATTAAAGAATGAGAACCCGTGGCA<br>CAACGTACAACATACTCGACCAGGAATCAAGAACCACGGGC<br>TCTATCCATAGTGGGTGAAGTTATATTTCAAGTGTGTGGATG<br>AGTGAAACTTTATAAAATAAATTGCTTTTTACAAATGTTAAA<br>AATGATTCACCAAATGTAGTAAAACAACTTGAAGTGGTAGTT<br>CTTCAAACCGTTGTTTCGCGAGTGCTCATGTTTCTTGAGAATT<br>TCGTACATTTCCCTACTTTATGAAAAGTTAGTTCCTCCTTCCA<br>TTCAATAATTCGTACGGTTACTGTGTACTTGTGAGCTAGCTGT<br>AAGTAGGATAATTACAGCCTAATGGTAAATGCAATTTTCTTT<br>AAAAACCCCTTACTCAATCCAATAAGCTTTCAATTCTTAATTC<br>TTTGACTGTCGAGATATTCTTCGTGTAATTAAAAGAAAGCAA<br>ATCTTGAATCATTTAGTGTAAAACTATATTATATTTGCTCCTTT<br>AGATATTTTTAATTTTGTTGGCTCCCATTGGAAAAGTCGCTGT<br>ATCTTACGTATTTTCTTCTTTCTTTCGTAAATAACAATTATCAT<br>TGTAGATGTATTTATAAACATGTAATTTTTAATTTAAAATAGA<br>AAGGCTAATTACAAAGCTGATATTGAAGAAACTTGCATGTTA<br>TTAACCAATTTGGGTTCCTTTGATAAACTAAAAAATCAAAGA<br>AAC

PhdNtxCav 34	P0C2S6	4,41E-07	46,429		5993,95	LRLPVFVFAVASIAF<br>LAACVETGSIHATEF<br>VEEENRRRGCAQAY<br>KSCDSKPCCVV	AACCGCATACTCATAGATAAAATCTTCGTAAAGATCATTTCT<br>TTAAACATTATTGAAGAGTTTTCGACATCGCAACATGATGTG<br>ACTTAGACTACCGGTATTTGTGTTTGCTGTCGCTTCAATTGCA<br>TTTTTAGCAGCATGTGTTGAAACAGGCTCAATTCACGCCACT<br>GAATTTGTAGAAGAAGAAAACAGAAGAAGGGGATGTGCAC<br>AAGCCTATAAATCATGCGATTCAAAACCTTGTTGCGTCGTGA
PhdNtxCav 35	A9XDG1	0	35,556		10272.94<br><br>**8238.33**	MKFCILLFVFFLTLV<br>DC**EKAILENENNAE**<br>**ETSALIQEESARECL**<br>**KHKEDCMNNQSG**<br>**CCFPWRCVCWTQSI**<br>**YNSHGVVIGSKRK**<br>**CGCKLW***	GCCACATCTGAGGAATTACAGGAAGAAATTTGTGAAATTGTT<br>TCAGGAAAATGAAGTTTTGTATTTTGCTCTTTGTGTTTTTCCTA<br>ACACTCGTAGACTGTGAAAAGGCAATCCTGGAAAATGAAAA<br>TAACGCAGAAGAAACATCAGCACTAATACAGGAAGAAAGT<br>GCCAGGGAATGCCTTAAACATAAGGAAGACTGCATGAATAA<br>TCAAAGTGGTTGCTGCTTTCCTTGGAGATGTGTGTGTTGGACA<br>CAGAGCATCTATAACTCACACGGCGTCGTGATTGGTTCGAAA<br>AGGAAGTGCGGTTGTAAGCTTTGGTAGTCAAGAAAATGCAG<br>CATAAAAACGTTTCAATCATAAAAATGTTTCAACGTATTTCA<br>ACCTAATTGTTATAAGTATGTTAAATAAATGCAAAATGCTCT<br>TTACCACAAGTTTATTTTTGGTTTCTGTGCAAAGATAAAAATT<br>CAGTGAGCTTAATGCTGGTATACATTCGGCTTGAATGAAATG<br>TGGGTTTTAAATAGGATTGAAGAAAAGTTAAGAATTCTGCCA

PhdNtxCav 36	A9XDG5	0,000000 00573	45		10501.29<br>**8543.69**	MKLCILLVLLLITFVR**SEKEILENEVNAEETLPIIAEGRRATKCIKEGKSCLDNEDGCCPLSTCECWEQRIPRKSTNWVKRCQCARPEQFQ***	CAAGCGCACTTTTATAATTGCAGGAGAAGGATTTTAGAAGCTTGAATAATGAAACTTTGCATTCTCCTCGTTCTGCTTTTGATAACGTTCGTACGCTCTGAAAAGGAGATCTTGGAAAATGAAGTTAATGCAGAAGAGACATTACCTATTATAGCGGAAGGAAGAAGAGCTACGAAATGTATCAAAGAGGGTAAATCCTGCTTGGACAACGAAGACGGTTGTTGTCCTCTTTCGACATGTGAATGCTGGGAACAAAGAATACCTAGAAAGAGCACTAATTGGGTCAAGAGGTGCCAGTGTGCGCGGCCAGAACAATTTCAATGATTTTCATATAAATTTTCAACGTATTTCATTTCGATTGCTGTAACTGTATTGAATTGAAATAAATTGTCCCTTTAAACAACATTATTCCGGGATTATATATAAGGTGTGTAAGTTATAGAACTTAGAGAGGAATGGGAATAAATCTAGACCGAATGAATTTCCCTTTCAGGGCAATTTTGACTCTGGAATGAAGTAACTAAAGAAATCTGTTCAAAGATGTGAATTAAGCGGAAGAAACATTATGAAAATGCAAAAATAATATAAAACATGAATAAGACGAATCTCTTAAAAATAAGATCTCTCCACACAGCTGCCAATAAGTTAATTGTTTAGAGCAACCATACACAAATCTGGATGAGATCCAGTTTCACAATACTGTTGAGCTGAGTGATCAAGTGCAACTCTTAGAATGAAAATGTACCAAAGAATTGTGTCAGAAAATGAGTAAGAAACACGAGGACAACCTCATGTGCCGATTATCAGGAGAAAATGTACAGGAATTTACGACAAATAAGATCGAAAATGACCTAGTCACCGCCATTATCCTCAGATTAGAAGATCCATGTTTGAACCATGGCTGTAACGAACAGTCGTTGCAACATGCAGAAATCTTAATTATTATAAACATATCCGATTAAATTTGAGTCAATGTAATACGGTATTACCGTGCAGACAAAATAGGAGTTCACTGGACATTTTTATGGATAAGTCTATATTTTTATCATTTTATGCAGATTTCATCTTTGGAGAAAAAAAGGCTACTGAATATTTTCTCTTGTAAAAGAAAGGATTTGCCTAGAAACTGGAAAACAAAAAATCTCAGTGTCAAAGGAAAGGAAGAAATC

							TATTGAAAAGTAATGCATCTTCACACAAACCAATAAGGCCATTGGTTAGACTATCAATAAGACTATCTTCCTATCAATGAATACCCATTTTGACGCTCAAAAATTAAATTTGCTGGCAACTAAAATATATATACATTTTATCCAGCAACTAATACTATGGCGTTGAAAATTATTTAACTTAAGAAACATCATTAAAGTTATTTTTCTGTTTAAATGAAAAAAAAAATATATAAAAGACTAGTCGTAAAGTTCGGTATTAGGTCCGAACGTATCGCGCGTATGGATGAGCACCCTCTACTGACTGTGTAAATGACGTAGTCAGTTGCGGAAAGTTTCTTGAAACGGGGAGACTGGAAATTTCTCTTGCTTCCAACTTCATATGTAAATATTGTGTAAATAAACTATTAGATTTTCGTATACACCCCAGGCG

PhdNtxCav 37	P0C2S6	0	92,683		5805,84	IYFRCADAYKLCNRPRTCCDGYNGYKRACICTGSNCKCKKSLREMAAAALSL*	TTTTCTAAAATCTTTTTACAAGATGATAACAATGAACATGTAGTAGAAGAAGCCAGAGGGTAAGTTACAATGTTTCTTTTCTTTCTGTGGATAGATTATATATAATATCACCTTTTAATTTAATTATGCTTCATAAACCAGAGAAAGAAACAGCATATATACATATAACATATTAATTAAAGGGATTAAAAAGATATGATTAGCAAAACAAAATGGTAAAATGTTTCCCTTGTATTTTTCCCTTCTTACTTGTTTTCCCTTTCACTATTTCTTTTTTAATGAGCATCTGTAAAATTAGCAACCATTTTAAAAATTCTGTACTGTATCACAAAAAGAAACAAGCGATTTTACTTTCAATGATGTGAAACATTTTAACATCATTTATGGCAGAGGTATAATGAGAAACGCGAATTAATAAGATTATAAATACGATCATCTGTGGACATAAATGAAAAGTATATTTTACTTATCGAAATGTATGTTTATGAAAATTTAAAATTAAACATGTGTACTACTAAATAGAAATTACATTGTAATTTAATTAATAATGAGTAGCTTTAATATAGAATGCAACATAGATACTGAAATTGATTACTTATACAAATTTTATAGATTCAGGTAAAAAAGAAATGATTGACACTATTCTGAACTTAGAACATGCAAAAACGTGAACTAACGAACATTTATTCCAACAGAAGTTTAAGTGTTAAAACTCTACAATAGTAATGAATGTTTTTCTTCGCGTACTTCCACACAATTACCTGTATTCCCTCTTGAAAAGGGGAGAGATTTAAGTTGAATTCGTGTTAGTTGTTGATATAAGGGGACATAAATATCTTAAATGCGCTCAATAAGGCAATTTTTTGCATTTTTGGACTATACATAGTTGTGAACATTTCGAATCATGGATTTTTATATATGATATCCTAAATTTTTTACTTTAATAAGTGGTTTTTACATCTTTCACATTCTATAAAATTAGTGTTTATTCACAAAACATTTATTCGTGTCTGATAATGATATACTTAGAGTTTGTTCGCAAAAATTATAACGCCAGCCCTTTCGTCAATGCCAACTATTCATTTGAGAATGCTGCGATGACTTTAATAGTGCAATATATTCATACGTTTCAAACGAGACAAATAATGTATGATATTTTAACATTTAAATTAAAATTATACAAACT

							AAAATAGTAATATAATACTACAGAAAAGTGTGGCTATCTTTCATAGTTCTTAAGATATCCCACTTGAAGTATTTAAAAAAAATCGAACTTTGAACCTTTCCTACCAAAATTTTACGTAAATTCCCCATATGTATTTTTAAACATATATTGAAAAATTATTGGAATAAGTTATGGGGTTGTATTAACTCATTAAAGAAAGTCTAAAAATTGCTAATAGTAATAATAATATGTATATATTATTATTATTATTATTGATATATAGAATATGTGTATATCTATGTAATGGAACTTAAGAGCCGGCCTAAAGAAAGTCCCTTTTTATAAGATATAATCGATACAGAAACCCCATTACCTTGTGACGTATTATGCAAATGCAGAACGTCGACTCAAGAAGAAGACAAAGATGCAAATTTTAACCAGTTAAATAGAGAGGGGATTTAGGTACGGGCAGTTTCATTTTTTGTAATTACTTTTGCTTCAGTTTACTTTAAGTTCAGTTATGTTATATTGTCTTTTCTGTTATTATGTTTAGGTAGCAGTATTTGGCTATTGCTGATGTTTTAAGTTTAGTAAATAGTGATTTATGTTTTATGGGTGAATAAAAATTCACTTTAACTTATTTGTGTGTCTTGTAATCATTCACTAATTACATCTGAGCTAGCCGTGTGGCCAACCGTTAGGAGGACTGAAGCCCCAACTATGCAAGACCCCGAGATGGAAGAGTTAAGTGGTGCGTGCTATTAAAGGGTGAGCATCCCCCTTGCACGCCACAACCTTACATCAGAAGACGTTAATTTGATATAAGCGGCTAACATGAACGGAAGTCTTAGCTCGCAAAAATATCAGAAAGATCCCCCCGCGCATCCATTTCCAGTGTCGGTGTATCCATTCCATATCAGATCTACACCTTTAGTTGTTTACCAGTCAAGCACGGTCAAGATCATCAAGATTATAAATAAGTAAACTTTAGTTCGGTTTAAGAATCTCTAATAGCATCAGACCCCTGGACCTCCCTGATGAAAATCCAAAATTAAACCACCAATTAAATCCCTTATGAGACCTTAGAGTATTTTCCGTGGGCTAAATGAGATGGTTCTTCCGAGATATAGATTGGGTTTTGTAACAGAGAGACAGAAAGATATTAGATTTTCAGTGAGAGCGCGATTTGATCTTGGTTCTCTCT

							TTCTGTTTTGTATATACGTAACGCTCGGCATTGTAACTGACAGCTAATACAATGTCTTATTGCTATCATCCGCACCTTCACTAAACGATGTGGAATTTAACTCTACGGTTTCGAGACTTAACTACTTAATCGAAACTTAATTGAACGGAGTGAAAACCCCGATCATCATATATATATATATATACATAAAAAAACAGAAAAATTGAAGGATTAGGACTACAAAGCAACTTGAAACGTTTTACTTCACGGTTTTGAGATATAACAAGTATATTTTACGATTCACTTTCATTTCACTTTTTATCGATATTTTATTTTTTTAAATTTATTTCAGGTGTGCCGATGCCTATAAGTTATGCAACCGTCCAAGAACTTGCTGCGATGGCTATAATGGCTATAAACGAGCCTGCATATGTACTGGATCAAATTGCAAATGCAAGAAATCACTGAGAGAGATGGCTGCTGCAGCTTTAAGCCTTTAATATTATTCTCTGTTACTAACATCCATAGAAGTTTGCTTAATATAATCGAAATATTTAGTAACTAGCTATCAGATGTAATTATTATAAATAAAATGCGAAAAAGAGAATCATGGTATACATTGTAAAATCATATAGAAATGT

PhdNtxCav 38	P0C2S6	0	91,25		9673.24<br>**7133.03**	MWLKMQLFVLAIALIALFEVHA**EPESNDNNEHVVEEARGCADAYKLCNRPRTCCDGYNGYKRACICTGSNCKCKKSLREMAAAALSL***	CTGCCAGATGCACTGAATATAGAAGTTAAAGAAAAGTTTTCGTAGAGCATATATTTATAAATTCAGGAATCACGTATTAGAACTGAACGATCTTTGGGAACATTGAAAAACTAACTTTTCTAAAATCTTTTTACAAGAAGACGCTGGGCAATGTGGCTTAAAATGCAATTGTTTGTGCTTGCTATCGCTTTAATAGCGTTGTTTGAAGTCCATGCTGAACCAGAATCAAATGATAACAATGAACATGTAGTAGAAGAAGCCAGAGGGTGTGCCGATGCCTATAAGTTATGCAACCGTCCAAGAACTTGCTGCGATGGCTATAATGGCTATAAACGAGCCTGCATATGTACTGGATCAAATTGCAAATGCAAGAAATCACTGAGAGAGATGGCTGCTGCAGCTTTAAGCCTTTAATATTATTCTCTGTTACTAACATCCATAGAAGTTTGCTTAATATAATCGAAATATTTAGTAACTAGCTATCAGATGTAATTATTATAAATAAAATGCGAAAAAGAGAATCATGGTATACATTGTAAAATCATATAGAAATGT
PhdNtxCav 39	P0C2S6	0,0000026	85,714		4864.93<br>**2324.73**	MWLKMQLFVLAIALIALFEVHA**EPESKDAGQCGLKCNCLCLLSL***	CTGCCAGATGCACTGAATATAGAAGTTAAAGAAAAGTTTTCGTAGAGCATATATTTATAAATTCAGGAATCACGTATTAGAACTGAACGATCTTTGGGAACATTGAAAAACTAACTTTTCTAAAATCTTTTTACAAGAAGACGCTGGGCAATGTGGCTTAAAATGCAATTGTTTGTGCTTGCTATCGCTTTAATAGCGTTGTTTGAAGTCCATGCTGAACCAGAATCAAAAGACGCTGGGCAATGTGGCTTAAAATGCAATTGTTTGTGCTTGCTATCGCTTTAATAGCGTTGTTTGAAGTCCATGCTGAACCAGAATCAA
PhdNtxCav 40	P0C2S6	0	90		5271.11<br>**2730.91**	MWLKMQLFVLAIALIALFEVHA**EPESNDNNEHVVEEARGCRGIKTF***	CTGCCAGATGCACTGAATATAGAAGTTAAAGAAAAGTTTTCGTAGAGCATATATTTATAAATTCAGGAATCACGTATTAGAACTGAACGATCTTTGGGAACATTGAAAAACTAACTTTTCTAAAATCTTTTTACAAGAAGACGCTGGGCAATGTGGCTTAAAATGCAATTGTTTGTGCTTGCTATCGCTTTAATAGCGTTGTTTGAAGTCCA

							TGCTGAACCAGAATCAAATGATAACAATGAACATGTAGTAG AAGAAGCCAGAGGTTGCAGAGGCATAAAGACGTTT
PhdNtxCav 41	P81792	0	71,429		8517,98	KVILYTIYIISKINFDF DFRACIRTGQICKDD CDCCDCEDECYCPP GSSLGFFKCSCALGG KYFCSHKKQKCKKA *	GGAAGTTATTTTGGAAAATCTTTGAATGCAAGTCGGCAATCA GTGAGAAATTTTTTTCCATAGGTTAACATTGAACAGGGTGAA TCCAATATGGCGTCTACCCAGCAGCCTTAACTGAGAACAAG ATTGTCCTGGATCAATTAAAGTGGCGTGGTCTTTGGACTTGGT TTAGAAGCTAAGGGGATGAGAAACAACAACAGTTTTTAATG GAGTGATTATTAACGTGATCATGATGCCAGTTTTAGAAAGTA ATTTTATACACAATATATATTATTTCAAAAATAAATTTTGATT TTGATTTCAGAGCTTGCATCCGTACGGGACAAATTTGCAAAG ATGACTGTGATTGCTGCGACTGCGAGGACGAATGTTATTGCC CCCCCGGGTCCTCCCTCGGATTCTTTAAGTGCTCGTGTGCACT TGGAGGTAAATATTTTTGTAGCCATAAGAAGCAAAAATGTA AAAAAGCTTAAGAATTGAGAGTAAAAGACTTCAGGAAAAGC CTGCAAATAACTGGACGGAATTGAAACATGCGTTTGTGAAA AGAAATTGAAAATTTATGTGTATTTCTGCCGGAAAATGAAGT CGCATTAATGATTAATCTATGAAATCAATGATTAAAATTTATT CATGTTTTAAAAAAAAAAAAAAAA

PhdNtxCav 42	P81792	0,000000 502	74,359		10668,27	RACIRTGQICKDDC DCCDCEDECYCPPG SSLGFFKCSCALGAE GSSISSLCGIPKRISRD FSIISSLVSKRRPFKDF LSSRRRRWTQTWRIR RS*	AGAGCTTGCATCCGTACGGGACAAATTTGCAAAGATGACTGT GATTGCTGCGACTGCGAGGACGAATGTTATTGCCCCCCCGGG TCCTCCCTCGGATTCTTTAAGTGCTCGTGTGCACTTGGAGCTG AAGGCAGTTCGATAAGCAGTTTGTGTGGAATACCCAAAAGA ATCTCACGTGACTTTTCCATTATCTCCTCTCTGGTTTCAAAAC GGCGTCCCTTTAAAGATTTTTTGAGTTCAAGAAGAAGACGTT GGACTCAAACCTGGAGGATAAGAAGGTCGTAGCACTGTTGC CAGTGACGTAGCCAGAAAAAAATTTCTGGGTGGGACCATAA TTTTTGATGAACACATTACATATCTTAATGCAACGCATCTTTG TTATGATACATTGAAAGTTATATAAAGGAACGAAATTTCGGG GGGCAGGCCCCCCTGGCCCACCCCCTGCCTACGTCCCTGAAT AAGATAATATAATTATTCAGTTCATTACATTACAGGAGAGCA TCCCAATATCTCTGAAACAAGAGCTAATAAAGCTTTTTCATA GCATTATTTGTGTTCATGATGTTAAATTCATACAGGATTGCAC ACTTTCACTTCAGAAACAACTTCATTGTTATCCAGTGTAATTA ATTAACATCTTAATCCATGTATGCCCTATGTGTCATTATTGGA ATGGTAGTGATGTTTTTTACATCCTACTGAGGTTTTGCCTAGT GTCCATTTTTGTAACATCACTTGGAGCTCAATACAGTATATAT TGTTTCTCTCTTCAAATTCTTGTTCCTTGAATTTTTCTAGGTAA CACATATGAATTTCATGCACAATTTAAAAATTTTAACCTCCTG
PhdNtxCav 43	P81792	0	74,359		13261,32	RSVHLLQTLKRCVTS LKNNRSGEIQVIQDE VCCVVSLCHRFVHIF VVEAEEEAEFDALV PQERACIRTGQICKD DCDCCDCEDECYCP PGSSLGFFKCSCALG	GGAAAACATGACAACTTTGACACAAGAATATGCTAATATTG GTATATATTCGGGGATGTCAAACACATAACGTTCAGTACACC TTTTACAAACCTTGAAGCGTTGCGTTACATCTCTAAAGAATA ACAGGAGCGGTGAAATACAGGTTATTCAAGATGAAGTGTGC TGTGTTGTTTCTCTCTGTCATCGCTTTGTGCATATTTTCGTAGT AGAAGCCGAAGAAGAGGCTGAATTCGATGCTTTAGTACCAC AGGAGAGAGCTTGCATCCGTACGGGACAAATTTGCAAAGAT GACTGTGATTGCTGCGACTGCGAGGACGAATGTTATTGCCCC

						GKYFCSHKKQKCKKA*	CCCGGGTCCTCCCTCGGATTCTTTAAGTGCTCGTGTGCACTTGGAGGTAAATATTTTTGTAGCCATAAGAAGCAAAAATGTAAAAAAGCTTAAGAATTGAGAGTAAAAGACTTCAGGAAAAGCCTGCAAATAACTGGACGGAATTGAAACATGCGTTTGTGAAAAGAAATTGAAAATTTATGTGTATTTCTGCCGGAAAATGAAGTCGCATTAATGATTAATCTATGAAATCAATGATTAAAATTTATTCATGTTTTAAAAAAAAAAAAAAAA
PhdNtxCav44	P81792	0	74,359		10762,34	NSYVIQDEVCCVVSLCHRFVHIFVVEAEEEAEFDALVPQERACIRTGQICKDDCDCCDCEDECYCPPGSSLGFFKCSCALGGKYFCSHKKQKCKKA*	GGAAAACATGACAACTTTGACACAAGAATATGCTAATATTGGTATATATTCGGGGATGTCAAACACATAACGTTCAGTACACCTTTTACAAACCTTGAAGCGTTGCGTTACATCTCTAAAGAATAACAGGAGCGGTGAAATACAGGTAACTGGCACTTGGTACAAAGAGCGGAATCTACTTGGAAAGAAGATATACAATTTGGTTAAATATTCTGTGCCGACGAAGAATTTGTATATGTACAAGTTAAAACTCCTATGTTATTCAAGATGAAGTGTGCTGTGTTGTTTCTCTCTGTCATCGCTTTGTGCATATTTTCGTAGTAGAAGCCGAAGAAGAGGCTGAATTCGATGCTTTAGTACCACAGGAGAGAGCTTGCATCCGTACGGGACAAATTTGCAAAGATGACTGTGATTGCTGCGACTGCGAGGACGAATGTTATTGCCCCCCCGGGTCCTCCCTCGGATTCTTTAAGTGCTCGTGTGCACTTGGAGGTAAATATTTTTGTAGCCATAAGAAGCAAAAATGTAAAAAAGCTTAAGAATTGAGAGTAAAAGACTTCAGGAAAAGCCTGCAAATAACTGGACGGAATTGAAACATGCGTTTGTGAAAAGAAATTGAAAATTTATGTGTATTTCTGCCGGAAAATGAAGTCGCATTAATGATTAATCTATGAAATCAATGATTAAAATTTATTCATGTTTTAAAAAAAAAAAAAAAA

PhdNtxCav 45	P81792	0	71,429		8517,98	KVILYTIYIISKINFDF DFRACIRTGQICKDD CDCCDCEDECYCPP GSSLGFFKCSCALGG KYFCSHKKQKCKKA *	ATTTTTTTGAAAATCATTTAGTGTGTCAAATTTGAGTTTTGCG ATTTCGTGAAAGAAACCGACTGACCGAAAACCAACCAAGTG AACAAGATAAGTAAAAGCATGTAAAATTCACCTTGTTGAAT ACTATTACTTCCAATAAATTGGTTTTCCATCATGAATATGACT TTGTCCATTTATGCTTCCAGAACACTGAAAAATTAAATGCGT ACGCATGGAACTCATAACTTTTCCCGAAAACAAAAAAAATA TGGGTATATAATTCGACCCGGGAAAATCGAAATTAACATTTT TCTTTGCATATGAAAATAATGTGAAAATTTCTTCCGCACTGA AAACAATTGAAATAACCTGTGTGTGACATATATTTCTGATCA GTAAAGGTTAAGTTAGAACTAAAGGATAGAGAAATGTTTAA TAAATTGAACTAAATTATTTAACTAGACTACTTAATTAGACT ATTGTTTATTTGAAAATTAGTTTTATTTCTATAGTTTCCTCCAC TACATTGTGGAACGAAAATAATACTTTACATTCTCCTATAGTT TCCAAATACAGTAACCAAATTGCATGAGAAATTTATGATCTC AGATAGTTTTGTATTATGTATGATATTCATATATTGTTTCTTTG CATATAAAATTTCACTAAAAGTTTGGTATCTCATACTGTAGA GGCTGAATTCGATGCTTTAGTACCACAGGAGAGGTAAGTCA GTTTTTATTTTTAACTTTTGAACTAAGCAATACAAACAAAAA AACTAATAAAAATTGTTAAGAATGTCGCACAGATATTTACAA ATTCCACGTTTATATTATTAGCAGTAATATTTGAGATAATGAT GTATTTTTGAACTTTTTATTTAAAATCTAGTATCATATTAAGG AAGTTCAGTATCTTTTCACAGGAAATGAACTTAACTTCGAGA GAATTTGAAACAGCTGAATTTTAATTTTGACACAATTAATGTT CAGGATTATTATTCTTGTATCCATAAATCTGCATTTAGATTCT AATTATAAATCAGTTTACTCGAATTAATCGACTCAGTTCTAA ATGTCAGAGATATAAGCATATTGACTATTTATATAACGTGTT GTTTAGTAATAATTATCCTTGTAATTTGATGAAACAAGTCATT GCGTTGTTGATAGCTATAGCTTTATAGATTGACAGAATTTAA

							ATTAATTTTTTATAACATGATCTAGAATAAAAAATTAATAAC<br>TTAAGTAAAATAAATTATTATCAGTTAACTTTCTGCAATAATC<br>CTTAATATTTACTTAATTTATTTTGTTACTTCTGATTTGAACTC<br>TATGAGTTACTATGGTGCATCTCCTACCTTCATCTCCAAGTTT<br>TTCTATTGGGCATTGCTGTGGGTTTTAACCTGCCACTCTGTTA<br>CTAAAAATTTGCTCAATGGCATTTCGGGTTTGTTGCACGTGAG<br>GATTTCGTGTGTCTCGTAACCCAATTAAAATTTATCCATATTC<br>GTTTCATTTTCTCTTCAGATCAGCCTAGCACCGAGATATGCTT<br>ATCGAGAAAATCATATATATATATGTAACTCACTTTTATTTTC<br>TGCTGATATTGTTGGTGCTCTTGAAAAATAACGCAATAATTTC<br>TTTCCCTCCCTCCTAATGTTTTATCCCGTGCTGGAGGTTTGGCC<br>CTGATCCCCCACCTGTGGTTGCGCCACTGCTTCTCACTCATTG<br>TAGAGAGATTACATTCACTTTCTTCGCATTTTTACCCATCTAT<br>GTGTCTATCTAACTATCTGTCTATCTCTATTTATAATATAAAC<br>GGTTTATACCGGCCCCATCCAGATCTGTCAGGGCAAACAACA<br>AAAACAGACAGACGAACTAATGTTTCGTCCTTATATTTTTGG<br>TTACATCTTCATATTTACATCCTAATTCTCCAGACCTGAAATT<br>AACAATTGTACTTAACTATCATATAAACTACTGTACTAAGCA<br>ATTTTATTTAAATTTAAAAATGCCAATATATCGATGTAAATAT<br>TACATGGACTGGTTGGCCATACACCAGTGGCTCCTCCCCAGT<br>TGACAGCGATGTTTTTATTTTTATTAAATGCTAATCTTCCAGA<br>AAATGTACCGTGATAATACACTATTTCTGGAACTCCTTAATTC<br>GTCAGGAATGCAACGGTGAAAATTCCATTGAAGTCAGTTCA<br>ATAGTTTTTAATTTATGTCTGTTCAAACGAATAAAAGAACAA<br>GCGTTTCAGTAGAATTGCAAATTGATTTTGCTCTTCCCAACTA<br>ACAATTAATTAAATCCATACTTAATATTTGTTAACGATGGCTT<br>TTGCAGAATTAATTTGATTTGGTTCCTCCCAATTAATTGCGAT<br>GTTTTTATTTTTATTATAACTATAAATCTCCACGGAAACATCG

							AATGATCACTGGAATTATCATTTTTTTTTTAAATAACAAATTT<br>GCAGTGTTTCATTCTTGTTTGATGAACATTTCATTGCAGACCA<br>GATTCCTGATTTGGGGTCCAAAAAAATACATTCGTCGATGTT<br>TCTATTAATCGTGCATTAATCCGTTAGTTGAGTGGTGCATAAC<br>CAAGTGGTGCTAATTGCATAATTCAAAAACTATGGAACCGAT<br>ATTATGGGTTCATTAATTTTTGAGTTATGCACTTTGAAACAAA<br>CCAACGCGCGAATAAATTTTAATATTTGTACAATTGTAAATT<br>GGTATATACCGTAGGAATTCATGTACCGAAATTTTCTATGGTT<br>TTAATATTAAAACTGTTAATCAAATGTCCGTGTGTAATTATAC<br>TTATTTTTTAATTAATATTTATGGAAAATATTTATTCGACAGC<br>GATGTATTCCCCTGGAAAAAATCGAAAAATAGATTTAAATAT<br>GTAGAAGACGCGGTCACTCGAAAGTGTATCCTCCCAATTGAC<br>AGTGATGCTTATATTTTTATTAACTGCTAATCTTCCATTAAAT<br>ATAGCTTGAAGACACTTACCGCATTTTACTCCTTAGCTTATGA<br>GGAATGCATCAGTAAAAGTGCCACCGAGATTGTTTCAGCAGT<br>TTTTGAATTACGCTTATTCAAACAAATAAATTACACGGTCAC<br>CTTGCGCCATTGTTGCAATGCAGTTTGTTAAATGTAGCTGCAG<br>GAAGAGAATATTTTTATTAACTGCTAAACTCCGAGAGACCGT<br>AGTGTGATTATACATACACCATGTTACTGAGTAGTTCTTGAG<br>GAATGCATAGGTGAAAATTCCATTGAAACCAGTCCAGTAGTT<br>TTTTGAGTTATGCGCATTTAAAGAAACAAACACACGCGGAA<br>ACAAACGTTACCTCTTTATATAACAGTATGGAGAAATACATT<br>TTAGTAGTGGAACTGCTAGTAGTGGAATTTGGTAGTTGCTCA<br>AACTCAATTCTATTCTACTAATAAATTTTGAGTTATTCTTGAT<br>AGGTAATAAAATGTACGCCTCAAAATAAAACTCATTCAAAA<br>TCTGTGACGGCCGAAAGTAAAGTTGGTTAGACTCTGGTGAGA<br>ACAGAGTCTGCTCGGGAAAATGAAGACCTCGTTGTTTTTTAC<br>AGAAGCTGATCACATGATCGGAAAGTGTAGATGCTAAGTCC

							AAGTATGGAAATAGTTTACGCAGTTTCTACACTCCTTAGCGCCAGAGGAAATTAGAAGTCCTACAAGCGCCAGCTAGTCTGGCTTTGAGGGAAAGCTTACTAATTACTATTACGTCAAAAAAGGAATGAGTCCAAATTAAATGTGTAAGGACAATTAGTAAAGGACTTATATATATATATTTAAATCTATTTCATTTTGACACTCACCCTTTGAAGTAATAGTGGTAAGTTCTTATATTGGAAAAATTTAATAACGTCTGTGGATGAGCTTCTTTCTTCACGGCACTCCTGATGTGTTGCAACAGCGTCTTATTGGGTCGCTGCACTCTATTTGAAAATTACTGAACTTACTTATAAACTGGATTGCATATCTTCGTAACAAATTGATAAAATATTTTTCTATTAATGGATAGTTGACAGAAATTTTATTAAAATTCAGAGAAAATTTTAAGAAAAGTCTTAAAAATTGATTTTCGAGAGTAAAATTGTAAAATGGGTAATTTTTCGTAAAAAATTAAGAAAAAATCACTCTTGTCAAGTCGAAGATTCACATTTGTGAAATCGTTTAGATTTTCCGTTTCTATCTCTTTTGTGCGATTGTAGGGAAGCGATCCCTTCACTTCTCCACATATTTGTTAGATCATTATTGAGATAGACGATTGATACATGATCGGGAAAAGGTATGTAAGGATGGGACTCAAAATATTTCGGTCATCGTTTATCTGTGTGATCCAATGGGCCACTGCTCATTACTTCTTACAGCTCATTTGGGAAGGTAATCGACTTTTGAAAAAAATTGAAGAGGCTTTAGGACGGGCATGTTGAAGATGGCGGGGAATTTAAATTTGAGCGGAGAACTTGGTAGGGGGCAATTTATGAAGGAAAGTTACTGGTGAAGAAGCCCGGGGCTCAGCTATCACCAAACTAGTAGCGTTTTTACTAGTAGCGAAAACGCTGCTAGAAGAAAGATTTATTTGGCTCTCCTCTCTACGAGTGTATTGGCTGCCGTTAACTGAGAACAAGATTGTCCTGGATCAATTAAAGTGGCGTGGTCTTTGGACTTGGTTTAGAAGCTAAGGGGATGAGAAACAACAACAGTTTTTAATGGAGTGATTATTAACGTGATCATGATGCCAGTTTTAGAAAGTAATTTTATACACAATATATATTATTTCAAA

							AATAAATTTTGATTTTGATTTCAGAGCTTGCATCCGTACGGGACAAATTTGCAAAGATGACTGTGATTGCTGCGACTGCGAGGACGAATGTTATTGCCCCCCCGGGTCCTCCCTCGGATTCTTTAAGTGCTCGTGTGCACTTGGAGGTAAATATTTTTGTAGCCATAAGAAGCAAAAATGTAAAAAAGCTTAAGAATTGAGAGTAAAAGACTTCAGGAAAAGCCTGCAAATAACTGGACGGAATTGAAACATGCGTTTGTGAAAAGAAATTGAAAATTTATGTGTATTTCTGCCGGAAAATGAAGTCGCATTAATGATTAATCTATGAAATCAATGATTAAAATTTATTCATGTTTTAAAAAAAAAAAAAAAA

PhdNtxCav46	P81792	0,00000103	74,359		10668,27	RACIRTGQICKDDCDCCDCEDECYCPPGSSLGFFKCSCALGAEGSSISSLCGIPKRISRDFSIISSLVSKRRPFKDFLSSRRRRWTQTWRIRRS*	AGAGCTTGCATCCGTACGGGACAAATTTGCAAAGATGACTGTGATTGCTGCGACTGCGAGGACGAATGTTATTGCCCCCCCGGGTCCTCCCTCGGATTCTTTAAGTGCTCGTGTGCACTTGGAGCTGAAGGCAGTTCGATAAGCAGTTTGTGTGGAATACCCAAAAGAATCTCACGTGACTTTTCCATTATCTCCTCTCTGGTTTCAAAACGGCGTCCCTTTAAAGATTTTTTGAGTTCAAGAAGAAGACGTTGGACTCAAACCTGGAGGATAAGAAGGTCGTAGCACTGTTGCCAGTGACGTAGCCAGAAAAAAATTTCTGGGTGGGACCATAATTTTTGATGAACACATTACATATCTTAATGCAACGCATCTTTGTTATGATACATTGAAAGTTATATAAAGGAACGAAATTTCGGGGGGCAGGCCCCCCTGGCCCACCCCCTGCCTACGACCCCCTGACTGTTGCAAGTACGAGCCAAAATCTGTTGTACTTTGAAAGCAGTATGTGGATGCGAATTATAGTGATGAAGCCTCCAATCTGTTTTCCCTGCTTCAGGTCGATTTCGTCCAACTTTCTCGCGCAAATGCCTCAAAAAAGTCGAGGCAAAATTGTCTCTCAAAGTTATTCTCCGATTTACTTTCACAACAGCAGCCACCTCTTCGCGTTTTACTTCAATTTTTGACGTTAGGGTCGTCCAGAACAGGGGAGGGGAGGTCATCTTGTGTGGAAGCTCGTCCGCCTTTGAAATGTTTACGCCGTCCAAAATATTTTGATCGTGACTTAGTCTTACTTCCAAAAGCTTGATTTATCAAGTCACAAGTCTCTTTGCCCGATTGGTTAAGTTTAACGCAAAATATGCTCGTTGCTCTTTAGAATAGGCTATTCTCTCGGAGTATGAAAATCGCGGCTGTCAAAATTTTGTCGCCGTGTAGATGTGTGTGTTATCTGTCAACACGAAACTTGACGAGAATATAGAAAAAAGTTCAAATAAGAGATCGGCATGCGGCGTGAATTTGAAATCTAAATATACAAAAAAAAAAAATCGATCCCTCAAGGTTTTGATCACACTTTGTACTAAATAGTCTGTATAGCAAGTTAGAAAAAGAGCTGGAAGTATTTCAATTTTCTGCCCTTGTGGGTCTACATGGACTGTATACATGCCAAATATTGGTCTGAA

							AGTCCTCCCAAAATGTGGTTAGCGTAACAAACACCAATAAA<br>ATTTTATGAACATTTGTAAAATGGGCATATCAAAGTATGTGA<br>AAAATTTTCATCATAGCAATGAACAATGACTTATTAAAATAA<br>CAAACATTGAAATAAACACTTACCACATATTCCCAAGGAAA<br>TTTTCGTCCAAATATTATCTCTTATTTATTTATTTATTTATTCCA<br>TATACAAGTTCGACCTTTCATCGTACAACGCCGGGTGGCGTT<br>TTACTTTCAAAATGAAAATCACTTCAAACTGTATGGTGTTGCT<br>TGCCGTATGTTTTATTTAAAATCTTTTTATCAAGGATGATCGT<br>AAAGCGATGAACAGACGGAGAAGAAATAAAGCAAAAAGA<br>ACAGAAATTCGCGCTGTATAGCAGGGCCGTAAACAGAGGGG<br>TG

PhdNtxCav 47	P81792	0,000000 893	74,359		9112,5	KVILYTIYIISKINFDFDFRACIRTGQICKDDCDCCDCEDECYCPPGSSLGFFKCSCALGGFKSIQCYGNRSALHDVAAKVN*	GGAAGTTATTTTGGAAAATCTTTGAATGCAAGTCGGCAATCAGTGAGAAATTTTTTTCCATAGGTTAACATTGAACAGGGTGAATCCAATATGGCGTCTACCCAGCAGCCTTAACTGAGAACAAGATTGTCCTGGATCAATTAAAGTGGCGTGGTCTTTGGACTTGGTTTAGAAGCTAAGGGGATGAGAAACAACAACAGTTTTTAATGGAGTGATTATTAACGTGATCATGATGCCAGTTTTAGAAAGTAATTTTATACACAATATATATTATTTCAAAAATAAATTTTGATTTTGATTTCAGAGCTTGCATCCGTACGGGACAAATTTGCAAAGATGACTGTGATTGCTGCGACTGCGAGGACGAATGTTATTGCCCCCCCGGGTCCTCCCTCGGATTCTTTAAGTGCTCGTGTGCACTTGGAGGCTTTAAATCCATACAGTGTTACGGGAACAGGTCTGCACTCCACGACGTTGCTGCAAAAGTGAATTAGGTCAGAAGGAAAAAAAAATAGAAAAGAGTAGATGTTAAGAATGTCTAGATTAGAAAGGATCTTTCAGTTATCTTCGGATGCCATCAACTATTCCAAAGTGTGTTTAATGGAAGCGTTTCATCTTCTAAAGGATCGAGTCCCGTTCCTGAGATATCGGAGATGGGAAGGATCAGAAGAGCCTCTCTGTGGAACGGACAGAGAGATGCAA

PhdNtxCav 48	P81792	0	71,429		8517,98	KVILYTIYIISKINFDFDFRACIRTGQICKDDCDCCDCEDECYCPPGSSLGFFKCSCALGGKYFCSHKKQKCKKA*	GGAAAACATGACAACTTTGACACAAGAATATGCTAATATTGGTATATATTCGGGGATGTCAAACACATAACGTTCAGTACACCTTTTACAAACCTTGAAGCGTTGCGTTACATCTCTAAAGAATAACAGGAGCGGTGAAATACAGGTAACTGGCACTTGGTACAAAGAGCGGAATCTACTTGGAAAGAAGATATACAATTTGGTTAAATATTCTGTGCCGACGAAGAATTTGTATATGTACAAGTTAAAACTCCTATGTTATTCAAGATGAAGTGTGCTGTGTTGTTTCTCTCTGTCATCGCTTTGTGCATATTTTCGTAGTAGAAGCCGAAGAAGAGGCTGAATTCGATGCTTTAGTACCACAGGAGAGGTAAGTCAGTTTTTATTTTTAACTTTTGAACTAAGCAATACAAACAAAAAAACTAATAAAAAATTGTTAAGAATGTCGCACAGATATTTACAAATTCCACGTTTATATTATTAGCAGTAATATTTGAGATAATGATGTATTTTTGAACTTTTTATTTAAAATCTAGTATCATATTAAGGAAGTTCAGTATCTTTTCACAGGAAATGAACTTAACTTCGAGAGAATTTGAAACAGCTGAATTTTAATTTTGACACAATTAATGTTCAGGATTATTATTCTTGTATCCATAAATCTGCATTTAGATTCTAATTATAAATCAGTTTACTCGAATTAATCGACTCAGTTCTAAATGTCAGAGATATAAGCATATTGACTATTTATATAACGTGTTGTTTAGTAATAATTATCCTTGTAATTTGATGAAACAAGTCATTGCGTTGTTGATAGCTATAGCTTTATAGATTGACAGAATTTAAATTAATTTTTTATAACATGATCTAGAATAAAAAATTAATAACTTAAGTAAAATAAATTATTATCAGTTAACTTTCTGCAATAATCCTTAATATTTACTTAATTTATTTTGTTACTTCTGATTTGAACTCTATGAGTTACTATGGTGCATCTCCTACCTTCATCTCCAAGTTTTTCTATTGGGCATTGCTGTGGGTTTTAACCTGCCACTCTGTTACTAAAAATTTGCTCAATGGCATTTCGGGTTTGTTGCACGTGAGGATTTCGTGTGTCTCGTAACCCAATTAAAATTTATCCATATTCGTTTCATTTTCTCTTCAGATCAGCCTAGCACCGAGATATGCTT

							ATCGAGAAAATCATATATATATATGTAACTCACTTTTATTTTC<br>TGCTGATATTGTTGGTGCTCTTGAAAAATAACGCAATAATTTC<br>TTTCCCTCCCTCCTAATGTTTTATCCCGTGCTGGAGGTTTGGCC<br>CTGATCCCCCACCTGTGGTTGCGCCACTGCTTCTCACTCATTG<br>TAGAGAGATTACATTCACTTTCTTCGCATTTTTACCCATCTAT<br>GTGTCTATCTAACTATCTGTCTATCTCTATTTATAATATAAAC<br>GGTTTATACCGGCCCCATCCAGATCTGTCAGGGCAAACAACA<br>AAAACAGACAGACGAACTAATGTTTCGTCCTTATATTTTTGG<br>TTACATCTTCATATTTACATCCTAATTCTCCAGACCTGAAATT<br>AACAATTGTACTTAACTATCATATAAACTACTGTACTAAGCA<br>ATTTTATTTAAATTTAAAAATGCCAATATATCGATGTAAATAT<br>TACATGGACTGGTTGGCCATACACCAGTGGCTCCTCCCCAGT<br>TGACAGCGATGTTTTTATTTTTATTAAATGCTAATCTTCCAGA<br>AAATGTACCGTGATAATACACTATTTCTGGAACTCCTTAATTC<br>GTCAGGAATGCAACGGTGAAAATTCCATTGAAGTCAGTTCA<br>ATAGTTTTTAATTTATGTCTGTTCAAACGAATAAAAGAACAA<br>GCGTTTCAGTAGAATTGCAAATTGATTTTGCTCTTCCCAACTA<br>ACAATTAATTAAATCCATACTTAATATTTGTTAACGATGGCTT<br>TTGCAGAATTAATTTGATTTGGTTCCTCCCAATTAATTGCGAT<br>GTTTTTATTTTTATTATAACTATAAATCTCCACGGAAACATCG<br>AATGATCACTGGAATTATCATTTTTTTTTTAAATAACAAATTT<br>GCAGTGTTTCATTCTTGTTTGATGAACATTTCATTGCAGACCA<br>GATTCCTGATTTGGGGTCCAAAAAAATACATTCGTCGATGTT<br>TCTATTAATCGTGCATTAATCCGTTAGTTGAGTGGTGCATAAC<br>CAAGTGGTGCTAATTGCATAATTCAAAAACTATGGAACCGAT<br>ATTATGGGTTCATTAATTTTTGAGTTATGCACTTTGAAACAAA<br>CCAACGCGCGAATAAATTTTAATATTTGTACAATTGTAAATT<br>GGTATATACCGTAGGAATTCATGTACCGAAATTTTCTATGGTT

							TTAATATTAAAACTGTTAATCAAATGTCCGTGTGTAATTATAC<br>TTATTTTTTAATTAATATTTATGGAAAATATTTATTCGACAGC<br>GATGTATTCCCCTGGAAAAAATCGAAAAATAGATTTAAATAT<br>GTAGAAGACGCGGTCACTCGAAAGTGTATCCTCCCAATTGAC<br>AGTGATGCTTATATTTTTATTAACTGCTAATCTTCCATTAAAT<br>ATAGCTTGAAGACACTTACCGCATTTTACTCCTTAGCTTATGA<br>GGAATGCATCAGTAAAAGTGCCACCGAGATTGTTTCAGCAGT<br>TTTTGAATTACGCTTATTCAAACAAATAAATTACACGGTCAC<br>CTTGCGCCATTGTTGCAATGCAGTTTGTTAAATGTAGCTGCAG<br>GAAGAGAATATTTTTATTAACTGCTAAACTCCGAGAGACCGT<br>AGTGTGATTATACATACACCATGTTACTGAGTAGTTCTTGAG<br>GAATGCATAGGTGAAAATTCCATTGAAACCAGTCCAGTAGTT<br>TTTTGAGTTATGCGCATTTAAAGAAACAAACACACGCGGAA<br>ACAAACGTTACCTCTTTATATAACAGTATGGAGAAATACATT<br>TTAGTAGTGGAACTGCTAGTAGTGGAATTTGGTAGTTGCTCA<br>AACTCAATTCTATTCTACTAATAAATTTTGAGTTATTCTTGAT<br>AGGTAATAAAATGTACGCCTCAAAATAAAACTCATTCAAAA<br>TCTGTGACGGCCGAAAGTAAAGTTGGTTAGACTCTGGTGAGA<br>ACAGAGTCTGCTCGGGAAAATGAAGACCTCGTTGTTTTTTAC<br>AGAAGCTGATCACATGATCGGAAAGTGTAGATGCTAAGTCC<br>AAGTATGGAAATAGTTTACGCAGTTTCTACACTCCTTAGCGC<br>CAGAGGAAATTAGAAGTCCTACAAGCGCCAGCTAGTCTGGC<br>TTTGAGGGAAAGCTTACTAATTACTATTACGTCAAAAAAGGA<br>ATGAGTCCAAATTAAATGTGTAAGGACAATTAGTAAAGGAC<br>TTATATATATATATTTAAATCTATTTCATTTTGACACTCACCCT<br>TTGAAGTAATAGTGGTAAGTTCTTATATTGGAAAAATTTAAT<br>AACGTCTGTGGATGAGCTTCTTTCTTCACGGCACTCCTGATGT<br>GTTGCAACAGCGTCTTATTGGGTCGCTGCACTCTATTTGAAA

							ATTACTGAACTTACTTATAAACTGGATTGCATATCTTCGTAAC<br>AAATTGATAAAATATTTTTCTATTAATGGATAGTTGACAGAA<br>ATTTTATTAAAATTCAGAGAAAATTTTAAGAAAAGTCTTAAA<br>AATTGATTTTCGAGAGTAAAATTGTAAAATGGGTAATTTTTC<br>GTAAAAAATTAAGAAAAAATCACTCTTGTCAAGTCGAAGAT<br>TCACATTTGTGAAATCGTTTAGATTTTCCGTTTCTATCTCTTTT<br>GTGCGATTGTAGGGAAGCGATCCCTTCACTTCTCCACATATTT<br>GTTAGATCATTATTGAGATAGACGATTGATACATGATCGGGA<br>AAAGGTATGTAAGGATGGGACTCAAAATATTTCGGTCATCGT<br>TTATCTGTGTGATCCAATGGGCCACTGCTCATTACTTCTTACA<br>GCTCATTTGGGAAGGTAATCGACTTTTGAAAAAAATTGAAGA<br>GGCTTTAGGACGGGCATGTTGAAGATGGCGGGGAATTTAAA<br>TTTGAGCGGAGAACTTGGTAGGGGGCAATTTATGAAGGAAA<br>GTTACTGGTGAAGAAGCCCGGGGCTCAGCTATCACCAAACT<br>AGTAGCGTTTTTACTAGTAGCGAAAACGCTGCTAGAAGAAA<br>GATTTATTTGGCTCTCCTCTCTACGAGTGTATTGGCTGCCGTT<br>AACTGAGAACAAGATTGTCCTGGATCAATTAAAGTGGCGTG<br>GTCTTTGGACTTGGTTTAGAAGCTAAGGGGATGAGAAACAAC<br>AACAGTTTTTAATGGAGTGATTATTAACGTGATCATGATGCC<br>AGTTTTAGAAAGTAATTTTATACACAATATATATTATTTCAAA<br>AATAAATTTTGATTTTGATTTCAGAGCTTGCATCCGTACGGGA<br>CAAATTTGCAAAGATGACTGTGATTGCTGCGACTGCGAGGAC<br>GAATGTTATTGCCCCCCCGGGTCCTCCCTCGGATTCTTTAAGT<br>GCTCGTGTGCACTTGGAGGTAAATATTTTTGTAGCCATAAGA<br>AGCAAAAATGTAAAAAAGCTTAAGAATTGAGAGTAAAAGA<br>CTTCAGGAAAAGCCTGCAAATAACTGGACGGAATTGAAACA<br>TGCGTTTGTGAAAAGAAATTGAAAATTTATGTGTATTTCTGCC

							GGAAAATGAAGTCGCATTAATGATTAATCTATGAAATCAATGATTAAAATTTATTCATGTTTTAAAAAAAAAAAAAAAA

PhdNtxCav 49	O76201	3,96E-05	73,077	PF02819	28118,6	LKKKTERCDTGFGN AFAKILDMRGYYVF ACCYDESSVGASNL KETCSPRLKVLPLDV TKDESVKKAMKAVE SNLGKSELWGIVNN AGVLKGFHTELCSM KDYEDCIAVNTLGM IRVTKVFLPFLRRSKG RVVNICSMSGRFRSP YLGPYTISKAAAITFT EVLRQEMREFGIRAI SIEPEFFKTPMCSEKY VQSVLDQSLAEVDE NIKSAYGTKYIEKAK EIYLTVFSHRCQDVK RVFDAVEAAISLQNP	GGCTGAACCAAATTCAAGTCCAAACAACCCACTGATTGAAG AAGAAGACAGAGCGGTGTGACACAGGCTTTGGAAATGCATT TGCTAAAATACTAGATATGAGAGGGTATTATGTTTTCGCTTGT TGTTACGACGAATCGAGTGTAGGTGCTTCCAATCTAAAGGAG ACGTGCTCACCTCGTCTAAAAGTCCTTCCGTTAGATGTGACA AAGGACGAAAGTGTGAAGAAAGCCATGAAAGCAGTTGAAA GCAATTTAGGCAAATCAGAACTCTGGGGAATTGTTAACAATG CTGGTGTCCTGAAAGGTTTTCACACAGAACTTTGTTCAATGA AAGATTATGAAGATTGCATTGCAGTAAATACTCTCGGAATGA TTAGAGTTACAAAAGTTTTCCTGCCATTTTTGAGACGCTCTAA AGGACGTGTAGTTAATATATGCAGTATGTCTGGAAGGTTCCG TTCTCCTTATCTTGGGCCGTATACAATTAGCAAAGCAGCTGC CATAACTTTTACTGAAGTTCTAAGGCAAGAGATGAGAGAATT CGGCATTCGGGCCATATCAATTGAACCAGAATTTTTTAAGAC ACCCATGTGCTCAGAAAAGTACGTGCAGAGTGTTTTGGATCA ATCCTTGGCAGAAGTAGACGAGAATATTAAGTCCGCATATG GTACTAAATACATAGAAAAAGCCAAAGAAATATATCTAACT GTATTTTCCCATCGCTGTCAAGATGTCAAACGCGTTTTTGATG CCGTAGAAGCCGCAATTTCACTTCAGAATCCTGC
Sodium channel toxins							
**Transcript ID**	**Uniprot ID reference protein**	**e-value**	**Identity (%)**	**PFAM domain**	**MW (Da)**	**Amino acid sequence**	**Nucleotide sequence**

PhdNtxNav 01	P29426	1,52E-09	84,375	PF07740	8424.91<br><br>**6457.38**	MRSSIFLILLLITLVRP **EEANMNYQVEAED RFPEISEERSFCIPFK PCKSDEHCCKKLKC KTTGLERLCRW***	GTAGGGTAGTCACGAGCAGCAGGATATAATTTCAAAATTTAC GTCGTTGTCATTAGTATTTCTTTACCATCTGCATAGCCTTTCGA GACCCGTAGTCTTGCACTTCAATTTTTTGCAGCAATGCTCGTC GCTTTTACATGGCTTGAATGGTATGCAAAATGACCTTTCTTCA CTGATTTCTGGAAATCTGTCTTCTGCTTCAACTTGATAATTCA TATTAGCTTCTTCTGGGCGCACGAGCGTTATCAAAAGTAGAA TGAGAAAAATTGAACTCCTCATTGTTCAAATTTTGGAAATCG CTTCACTAAAAA

PhdNtxNav 02	P17727	1,83E-09	92,000	PF08396	4305.84	IYLVASLARVLSSSEE MIEGENPLEDQRVD LTSCFPVGH	GATCTTAAAAATTGTTTGTTTGGGTGCGTACTGTCGCCTCTTG AAGTTTTGCCCATCTTTTATGAATCACCCAGTGTATTTTTCTCA TATTTGGTTATCGGTTAGCACCCTAATTGGACGAATAAATAT CCTCGTAAAATTTTCATACAGCATTATCCTTCCGAAAAGAAA AATAAATGATACATGAAAAGTTTCTTGTATTGAAAAAAATCT GATGCCTCAAGGAAGAAAGAAAAAATATTCATAAAATACAG CAGTAGAGGAAACAATCAATAATAACACTCCTCTGTGTGAT ACTTTCTTGAATTTCACACGGAAAGAGAACAGCGACATCGGT CTGAAACGCGAAGAAAGGATGAAAATGCGCATTGAGCACA ACTCATTCCTGTTGTCTTAACGTATTTTAATTGTTTGTTATTAA ATGTCATAATGTTTTCTAAAAATGTTTTTTTTTTTTTTTTTTT GTTATACGGAAAGAAAATTTCAAAAAAAAATTTCATTACGAC GACAGTGAATATTTTGTTCGAAATAATTCTAGGATCCTCACTT TCAGAAATACGTAAGAGGTATTGACATTAGAGATATCATAAT GAGATCATTTGCGCTCCATCGTCGAGTTGATTCACGAGTGTTT ATTTATGCATACAGTGTATAAGTCGAAAGTTTCAGATTTAATT TCGCTGTTCAAAAAGTCGAATTTTAGGGGTTTGCAGCTGTTAC CGCTAAATTTTCCATGTCTAATAACATAATGGTGAAGAGTGT CACCTAATAGATAAGTGTAGAATTCACCTCCTCGGAAAAAA ACAGGATATAGAATAACTAACAGCAGTTTAAAAGCCCAATA TCTTTATAAATATAAATCAACAATCGGCATTTAATTAAACTA TAAGCTAACTACAAAGCATTAGAAGATATTGTTCACCCAAAC ATTCGATGAAAGGCTCCTTGATGGAAGAAAGGGTCACTCAA ACGGAAAAAATTTGCCCCTCCAACATGCCACGCAATCGTGGA CTAAATTTCACCCTTATTAGGGCTCATCGGCATGGCATAGAC GATGATACGCTGGAGGCTTAGAATGCCAAAAGCACAGTAAA CAGATTGTCAGTTAGCAAAAAAACTTAACGTCTTTGGAACTC TATGCCCTCCAGCGTACCATGAGCTATGTTATGCTGATGAGC

							CCTAGTAAGGGCGAAATCAAAAAGCACGAGTGCCGGGTACG<br>CTAGAGATGCTAATTTTTGCATTAAGGTGTACGTTTAAGACCT<br>TTTCAAAACTTTTCAGTGGATCCTTCTGTAATTGAGATACGTT<br>ATTTATTATAATCTCTGAAAAATATCTCATTCCTCTCTTTTAG<br>AAAATTCCTTTCCTCACCTTGAATCAATTATTGTTGGGCGTGC<br>TAGGGTTCTCTGTATCGTGGATTGTGATTGGACGCTAAGGAA<br>AATGACTGTCGATTGTTATAGATTTCTGCACAGATCCGCCGG<br>TAGCGCTGTACGAATATTAAAATGGCATGCCTGAGATAAAA<br>AATTTTATTCTTAACATAAAAAACGGATATTTACAAGGAAGT<br>TAATAACATTCCCCTAACGAAGAAAGTAGAACGAAGAAGAA<br>AACAGGATCTACTGTTTTTCCCGTTGACTACGTTTTTTCACAA<br>GAGTGACAACATCAAAGACCATTTTCAAACTCGTGAATCATC<br>TTCCTCCAAATGCTTTGGGGTTCGCAGTGGAAAAGTTGCTCA<br>TGTTGAGGTGTTATTTTTTCAGTGCTCCACAGGCATTCTGTCTC<br>GGAGAATGCCTGCCTTGATTTAGAGGAGGATGTTTCGCTTAG<br>GACACAATAAAAGGGTATTTCACACAATGTTATTTAATAGAG<br>AAAGCGAATGGCTCTAATAATGATGAATAATGTAATTTTCTT<br>AGATCAACGAAGAAAAATGCAACGCACAAAGCTATCAGATT<br>ATTATCAAAGCTCTTGACAAAGACTGAATATTCTGGAATATC<br>GTTTTGTTAAAATATTAAATACATGTTAGATATATTTGAATCT<br>GGACGAATGTAGGAAATTTTTGGTTACAGGTTTCATTCAAAA<br>GTTAATTCTTGTTATAGGGTCTCATCGGTGGTAAGCACATATT<br>CCATTCTTTTTTCTATCCATAATCGTTTACACATGAATTATTTT<br>AATGTGGTTTTAGGTGGGTCGAACTAAAATTCATTTACACGT<br>ACTTCACAGTTAAGCATTTTTTTTCTCTCTAGCGTGGATAACA<br>ACGTAGATAAGCAACGCTCCACTCTCTTCCATTTGAATTCAA<br>GGTTTGGTTATAATCTTACTACGGAACACTATTGTCGTTCAAG<br>AATTAGACAATCCTGGTACAAATAAATTAATCTACTACTTTA

							CAATTTAACTGATAAAAATTTTCTATTGGATTCAAAAGATTGGATAAATTATTTGAGACCGCATTCTGCACACACACACACACACAATTATATCTCATCTATTTAAAAATCTTTTCTTTCAGGAGCTGCTTCCCAGTCGGTCATGAATGCGACGGGGATGCAAGCAACTGCAACTGCTGCGCATAGATATACTTGGTAGCAAGCCTTGCACGCGTGCTCAGCTCCTCGGAAGAAATGATAGAAGGAGAAAATCCTCTTGAAGATCAAAGAGTCGATTTAACGAGCTGCTTCCCAGTCGGTCATG

PhdNtxNav 03	P17727	2,66E-20	92,105	PF08396	5592.18	IYLVASLARVLSSSEE MIEGENPLEDQRVD LTSCFPVGHECDGD ASNCNCCA*	GAGCTGCTTCCCAGTCGGTCATGAATGCGACGGGGATGCAA GCAACTGCAACTGCTGCGCATAGATATACTTGGTAGCAAGCC TTGCACGCGTGCTCAGCTCCTCGGAAGAAATGATAGAAGGA GAAAATCCTCTTGAAGATCAAAGAGTCGATTTAACGAGCTGC TTCCCAGTCGGTCATGAATGCGACGGGGATGCAAGCAACTG CAACTGCTGCGCATAGATATACTTGGTAGCAAGCCTTGCACG CGTGCTCAGCTCCTCGGAAGAAATGATAGAAGGAGAAAATC CTCTTGAAGATCAAAGAGTCGATTTAACGAGCTGCTTCCCAG TCGGTCATG
PhdNtxNav 04	P17727	3,11E-32	94,643	PF08396	7112.21	ASNCNCCGDDVYC GCGFARWNCKCKV ADQSYAYGICKDKV NCPNRHLWPAKEC KKPCRRNCRG*	CTGTGATGTAAAATTACATCACACGCTCCCATTTCGGGAGCA TATCTCAAGATGTAAAGTAAATGCAACTTTTGCGTAATTTTTA TTATTGTGTATAATTTGAATTATAATTAGGTAGAAAAAGTAA TTTCGAGAGTCTTTGCCGACAGCGTCTGATAGCGGCATTAGA TGGATTACATAAACGATCCCCGTAGTTCTCCCTTAGGTCATTC CGTACTAGTATATACTCTGTGTGCATCCGGAGGGAATAAATA TTCCTCTTGGTGAAAGAATTTTCCGACGCGTTTGGGTAGTTTC AGAGACAACTCATTTCAACCATCTTTTTCTCTTTATAATGTTA GTGTAGACAGTGAGGTGTGAAAACATTTTTGACTCCAGGACT TCTTGTTGCAGATGGAGGACGTCGGGAATACTTTAACGCAGA CTTCAGGAAACTGAACTCGAAGTTTGCGAAAAGCGTTTTGAA TACAAGCCATGACTTTCAACTCAAATACGAAAAAAATAGAA ATGCATGTATGCTAATTTATTTTTATTGTTACGGTTCTTCCATT GGCGCATACCAAACATTTAATGCGAACAAAATCTGACAGCT TCAATTTTAACCGCGGCAATTTCTCCTGCAGGGCTTTTTGCAT TCCTTCGCGGGCCACAAATGTCGGTTCGGGCAGTTGACCTTG TCCTTGCATATACCGTAGGCATATGATTGATCAGCAACTTTGC ACTTGCAATTCCATCGAGCAAATCCACAGCCACAATACACAT CATCTCCGCAGCAGTTGCAGTTGCTTGCAT

PhdNtxNav<br>05	P17727	1,33E-32	94,643	PF08396	7112,21	ASNCNCCGDDVYC<br>GCGFARWNCKCKV<br>ADQSYAYGICKDKV<br>NCPNRHLWPAKEC<br>KKPCRRNCRG*	GTACAATTTAAAGTTAGTTAGAGAATTAGCTAGAGAAAGTA<br>CTTTCGAGAATCTTTGCCTACTGCTTCTGATAGCAGCATTAGT<br>TGGATTATATAAACTCTCCCCGTTGTTTCCCCCTCAGGTCATA<br>CCGTAATAATATATACTGTGTACATCGGGAAGAAATAAAGA<br>TTCCTCTTGGTGAAAGGATTTCCAGGTAGTTTCAGAGACTACT<br>CATTACAAACATTTTTTTCTCTTTATAATGTTAGTGTAGACAG<br>TGAGGTGTGAAAATATTTTTGACGCCAGGTTTTCTTATTGCAG<br>ATGTAGGAGGTCTGGAATGCTTTAACGCAAACTTCAGGAATC<br>AGAACTCTGAGTTTGCGGAAAGCATTTTAAATACAAGCCATC<br>ATTTTCAACTCAAATACGAAAAAATAGAAATATATGTATGCT<br>AATTTATTTTTATTCTTACATCTCTTCCGTTGGCGCATGCCAAA<br>CATTTAATGCGAACAAAATCTGACAGCTTCAATTTTAACCGC<br>GGCAATTTCTCCTGCAGGGCTTTTTGCATTCCTTCGCGGGCCA<br>CAAATGTCGGTTCGGGCAGTTGACCTTGTCCTTGCATATACC<br>GTAGGCATATGATTGATCAGCAACTTTGCACTTGCAATTCCA<br>TCGAGCAAATCCACAGCCACAATACACATCATCTCCGCAGC<br>AGTTGCAGTTGCTTGCAT
PhdNtxNav<br>06	P17727	2,65E-33	94,545	PF08396	6013.81<br>**3957.23**	MKLLGIFLVASFAFV<br>LSFA**EEMIEGENPLE**<br>**DQRVDLTSCFPVG**<br>**HECDGDASNCNC**	GCAGTTGCAGTTGCTTGCATCCCCGTCGCATTCATGACCGAC<br>TGGGAAGCAGCTCGTTAAATCGACTCTTTGATCTTCAAGAGG<br>ATTTTCTCCTTCTATCATTTCTTCCGCGAAGCTGAGCACGAAA<br>GCAAAGCTTGCTACCAAAAATATCCCCAGAAGTTTCATTTTC<br>GTAATATGTCTATAAAGGCAGCTATTCAACACGAAAATGCTC<br>GAAATTGCTCGAAATATATATGATTGCTACAGATGGCATGAA<br>AATGGATAGCACGATGAAAATAAAGAAAACAAAAATATAG<br>TAAACTTTGTTAGAAAGGCCTATGTCTATCAAAGTATTCAATT<br>AATTTTGCTGTCTTAAATGATACGTGATGTTACGAATGTAGA<br>ATGCAAGGTAAATTACATATTGATATATCAGAGGAAATAAA<br>ATGAAATTTATCGAGAATGGTTGACAG

PhdNtxNav 07	P17727	3,83E-33	94,545	PF08396	6013.81<br>**3957.23**	MKLLGIFLVASFAFV<br>LSFA**EEMIEGENPLE**<br>**DQRVDLTSCFPVG**<br>**HECDGDASNCNC**	GCAGTTGCAGTTGCTTGCATCCCCGTCGCATTCATGACCGAC<br>TGGGAAGCAGCTCGTTAAATCGACTCTTTGATCTTCAAGAGG<br>ATTTTCTCCTTCTATCATTTCTTCCGCGAAGCTGAGCACGAAA<br>GCAAAGCTTGCTACCAAAAATATCCCCAGAAGTTTCATTTTC<br>GTAATATCTCTTTAAAGGCAGCTATTCAACACGAAAATGCTC<br>GAAATTGCTCGAAATATATATGATTGCTACAGATGGCATGAA<br>AATGGATAGCTAAATAATAAAGCTAAATAATACTAAAATTT<br>GTTAGAAAGGCTTATGTCTAGCAAAGTATTCAAATTAATTTT<br>GCTGTCTTAAATGATACGTGATGTTACGAATGTAGAATGCAA<br>GGTAAATTACATATTGATATATCAGAGGAAATAAAATGAAA<br>TTTATCGAGAATGGTTGACAG
PhdNtxNav 08	P29425	2,13E-17	54,237	PF17492	9229.83<br>**7481.51**	MKLAILLIVSVLVLA<br>VA**SESVEENREDFG**<br>**AEELTRGKCAATFN**<br>**PCKEHCDCCGEKV**<br>**ECVCDTRRCICRET**<br>**HILMSWYKLITC*** 	GAATTTATCAGATATCCAGCGAAATATCTGATATACATCTGA<br>TATATACCAGCGAAATACTGTTGATACAAATCAGATAAGAA<br>AGCATTTCACTTTGAGAATGAAGCTTGCAATCCTCCTCATCGT<br>CTCTGTTTTGGTTCTTGCTGTTGCAAGTGAATCGGTTGAAGAA<br>AACAGGGAAGATTTTGGAGCTGAAGAATTAACGAGAGGTAA<br>ATGTGCTGCCACATTTAACCCTTGCAAAGAACATTGCGACTG<br>CTGTGGAGAGAAAGTAGAATGTGTTTGCGACACGAGAAGGT<br>GCATATGCAGGGAAACACACATTCTTATGTCATGGTATAAAC<br>TTATCACGTGTTAGAAATGAGGTGCATATATGTATAACACGA<br>TATAATAAATATTATTGCATTTGAAAAAAAAAAAAAAAAAA<br>AAAAAAAAAAAAAAAAAAAA
PhdNtxNav 09	P29425	2,60E-17	54,237	PF17492	9229.83<br>**7481.51**	MKLAILLIVSVLVLA<br>VA**SESVEENREDFG**<br>**AEELTRGKCAATFN**<br>**PCKEHCDCCGEKV**<br>**ECVCDTRRCICRET**<br>**HILMSWYKLITC***	GAATTTATCAGATATCCAGCGAAATATCTGATATACATCTGA<br>TATATACCAGCGAAATACTGTTGATACAAATCAGATAAGAA<br>AGCATTTCACTTTGAGAATGAAGCTTGCAATCCTCCTCATCGT<br>CTCTGTTTTGGTTCTTGCTGTTGCAAGTGAATCGGTTGAAGAA<br>AACAGGGAAGATTTTGGAGCTGAAGAATTAACGAGAGGTAA<br>ATGTGCTGCCACATTTAACCCTTGCAAAGAACATTGCGACTG

							CTGTGGAGAGAAAGTAGAATGTGTTTGCGACACGAGAAGGTGCATATGCAGGGAAACACACATTCTTATGTCATGGTATAAACTTATCACGTGTTAGAAATGAGGTGCATATATGTATAACACGATATAATAAATATTATTGCATTTGACGTGTAGGCGTTCACATTCATTTTTGTCTATAATGGAAAGTTTTCCCATCCTAAGGCA
PhdNtxNav 10	P29423	1,53E-06	90,000	PF17492	3832.34	VYIFFSYVHSSFFCSACAGQDKPCKETCDCCGER	TTTCATTACTCCTTATTGATTATTATGTTAATGATTTTTCAGTAGTTTCATTGAATTAAATTTTAAGTTCATATTTTATCTAATATTTTCTTTACGTTAATTGACTTAATTAAGCTTCTGACTATTTTCTAGTTAGGAGCAATTATAGTCGGTAAATTTAAAGCAGCCTGTTATGTGCTTGATATAACTATGACGATAATTTTTCATAATATGAATTCTATCAAATTAGTGACATGCTATTTTATATTATTCTTCGAGTAAAGACGGTTTATTGGGATTAACGGATTCATATTATTATTTTTAAACGTTGATTTGTGATAAAAGGTTCTGAAGTTTTAAAATTTAATAATTTCAAAATTTTAATCAGAATTCTTATCGATTTGTTTGTCTGCATTATTAATGATTATAATATCATCTATATCTATCTATCTATCTATTTATTTATCTATCTAACTATATATATATACTAAATTTTCCTTGTATTCTGCGAATGTCATGATTTAAATAATATATTTTTCCCCAACGGTCGCTGAACACACACGCCTAAATTAATTTCATAACGCGTTTATGCCTTCTACATGACAAATTAATAGATAAGCATTTTCTCTCCTTTTACTTGCGTTATTCTGATTGCGGATTTCGGAATCTCATCACTCAGCAATATTAGGGGTACCACCATCAGGGATATGATAAATTCTCATGCTTTAGGTTTACATATTTTTTTCTTACGTTCATTCATCTTTTTTTTGTAGTGCATGTGCTGGCCAAGACAAGCCCTGCAAAGAAACTTGCGACTGCTGTGGAGAGAGAGG

PhdNtxNav 11	O76199	1,90E-17	87,692	PF17492	9053.55<br><br>**7404.36**	MKVAILILSILVLAV<br>A**SETIEEYRDDFAVE**<br>**ELERDACAGQDQT**<br>**CKVTCDCCGERGE**<br>**CVCGGPCVCRQGN**<br>**FLIAWYKLINCKK****	TTTGCTGTAGAAGAATTGGAGAGGAGATTTTCTTCGTTCTTTG<br>ATAATACGAATCAGAATGAAAGTTGCAATCCTCATCCTCTCT<br>ATTTTGGTGCTTGCTGTTGCAAGTGAAACCATTGAAGAATAC<br>CGTGATGATTTTGCTGTAGAAGAATTGGAGAGAGATGCATGC<br>GCTGGCCAAGACCAGACCTGCAAGGTAACTTGCGACTGCTG<br>TGGTGAGAGAGGAGAATGTGTTTGCGGAGGACCTTGCGTTTG<br>CAGGCAAGGCAACTTTTTGATAGCATGGTATAAACTTATTAA<br>CTGTAAGAAATGATATGGATTTATGTATACCATGATATAATA<br>AATATTGTTGCATTTGACGTGTAGGAGTTCACTTTCATTTTTGT<br>CCGTAATAGAAAGTTTATCCATTCTGAGGCAGGTAAATGCGC<br>ACTGCAGAGCTCAGTCCTGACCTGAATATCTGGGAAATTAAT<br>CAACTTTCTATCGCTGAAACTCACAGTCAATGTTAGAAAAAT<br>AACTGCATGTTAATTTAGTGATGAGGTACGAGTAATTTTATGT<br>ACTCGTCGATACAATAAATATTACTGAAATTTTCCTGTTGGCG<br>TTCACTTTCATTTTTATCTGCATTGGAAAGTTTCTACATTCTGA<br>GTTTGGTAAAAGTATACCTGCAGGTCTTACTCTCGATCTGTAT<br>ATCTGGTTATTGAAAACAACCCTTATCTCTAAGGATGTGCTA<br>ACATACTCCCGGTTGATGTGTACACAGTGTTAACGGATTGCA<br>AAATACGGTAGCTTAACGTCATCAGTCAAAGTTGAACCAGA<br>CTGGCTAAAACTGAAATTCCTCCATTCTGAGTTTTGTAAAAG<br>CACACTGCTGAGCTCACTTTTGACTTGAATATCTAGGATGTG<br>AATCGAGGCACTATCATTAAGGATTTGCTCACATATTTGCGC<br>GATTTAGTACGTATATATGATAAATCTGGCAGTTTTAACCAA<br>TCTTTGAACACGGTAGTTTAACGTAATGTATCAATTTAAAAC<br>CCGATTGGTTCAAATAATTCCTGTTTCTTTTACCTTTTCAGTAT<br>AGTACATCAACTACTACCTACAAATTTCATATCATTTCGCTCA<br>TTTGAGGCAGATCAATGCATTATTTAGTTTTATAATTTACTCT<br>ACTGGATAATGAAAAAATGTATCCTCCTGCACTTCAAGACAC

							TTTTAAAATTAATGTTATTTTTTCATTTCTAAAAATAGCCGAC<br>GGAATTTAACTACCGAATGATTTACAGCCGTGTTATTTTATTG<br>CTGAAAGTGTTCGCTTTTCAAATTAACACCATTGTCATTAGGT<br>GTCTCGTGTGAGTTCCATATTGTTTTCCTTATTTTACTGTTCCT<br>AATTTGTGCGTGTCATTTGAGAAATATTTCATTTATTGGTGTA<br>CAGAAGCATTGCAAATATATCATGAGCAATATACGAAAGTTT<br>CTTCACCTGAAAACCGAAATATATACAAGGACATAATCGTA<br>CACACTGTATTATTGTTCAAAAAAGCAGAGGTTCCACGTTAC<br>AGCATGGAGTCGCAATACTTATCTCACCTCTGAAGAAATAAC<br>GATTTTTCTTGAAGAACATTTAACCTTAGCCTTACTTCTAGGG<br>GTATTTGGCACCCAGGTATTCCAAGACTTAACGAAACAGGA<br>GAACGCTGATCAATTTCAGAACAGAAGACTATAGCCGCAGA<br>CTTAAGCTTTCATCATTTAAAAGCAAAGGACGATTGGATAAA<br>AGTAATCGATAAAAAACAGTGAGAATGAAGAAATGAAGGA<br>ATATAAGAATGAAAATATGCTCGATATTTTCCATATTTTATAT<br>TTAAATAAATTATTTAGGATCCAAATTATATTATATTCCTTTT<br>AATGAAGTTTATTACTTCTCTTACTACTTGTACCACTGTACAT<br>ATTGATTTGACCCTTAAGAATCCAATTTTCTTCTTTAAAATGT<br>AAGATTATTCCTATTCAAGTATATTTACAATGTCTAATGCATT<br>GGAGAATAGACTACGAACAACTGACTTACTTATTCACCGAAT<br>AGTTCAGTGACACATTAACTGGACATCCAGGGAAGCTTAATT<br>CATTTCGGTCTCAGTTTGGGGAACACTTCGTTTACAGTCTTTC<br>TTTGTATAAAATATGCATTATATAAAAAATCTACTTACAGTC<br>GTGCTTAAAGTGGTTCAATATTGTTATTTAAAGGAATATAAA<br>TTATTCACTGCATTTGCATACATAAAACTGAGACTTATGTCCT<br>ATGCAAACAGACAAGATGTCTCTCTTCGATTCCAGGGTATAA<br>AATCTATTTTCGTTTATATGGCGAATCTTTGAGAAGTATAATA<br>CTCAAGTTGGAGCAAGCATGCAATGTATGAACGTTGCCTGAT

							CTCTGGTCAAAGAATACCAGTACAGTTTCTGTCAAAATTGTT<br>AATCCTGTGGTGCAAGGATAGGCATTTTTCTTTTTACGTGTAA<br>TAAAATCCCCCCCCCCCTCTATCGACAAAGAATAAATAAGT<br>ACATCTGTTGCAGCTTTATTTCTTATTTTTAAGCATAAATTATC<br>TCAAATTTACATTCCGCAGTTGAAGATATAAATGGCACAAAC<br>AAATACAATTTTAATGCAATTTGTAAGAGATCCATGAGTGTA<br>GCAGGTCTTAAGCCGTAGTTGTTGAGGTGAATTTAAAATCAC<br>GTAATTGGTGACGTGGGGTGAAAAAGGACTTCCCTTTTTAAT<br>AAACAAGATTGTGCGTATTATAAAAAAAAATATTACTTAATG<br>GAATTACATTCTTTAATCTTTCTTAAAGGGA

PhdNtxNav 12	O76199	1,34E-12	89,286	PF17492	9855.37<br><br>**8206.17**	MKVAILILSILVLAV<br>A**SETIEEYRDDFAVE<br>ELERDACAGQDQT<br>CKVTCDCCGERGE<br>CVCGGPCVCRQGN<br>FLIACLHRWSLAFE<br>AFWSPF***	TTTGCTGTAGAAGAATTGGAGAGGAGATTTTCTTCGTTCTTTG<br>ATAATACGAATCAGAATGAAAGTTGCAATCCTCATCCTCTCT<br>ATTTTGGTGCTTGCTGTTGCAAGTGAAACCATTGAAGAATAC<br>CGTGATGATTTTGCTGTAGAAGAATTGGAGAGAGATGCATGC<br>GCTGGCCAAGACCAGACCTGCAAGGTAACTTGCGACTGCTG<br>TGGTGAGAGAGGAGAATGTGTTTGCGGAGGACCTTGCGTTTG<br>CAGGCAAGGCAACTTTTTGATAGCATGTTTACATAGATGGTC<br>ACTTGCTTTTGAAGCATTTTGGTCACCTTTTTGACAAGAGGCC<br>TAAAGTGACCAGTCAACAGATTAAAGACGTTTCCAAACGAC<br>TGCAGACACTGATGAAAACTGCTAGAATATAATTGAAGAAG<br>GTCAGTGGAAAGACATTTCTACGCATTTCGATTTGAAAATAA<br>CAGCGCAAAACTTCATGATGCTGAAATTATGAGGGAAAAAC<br>TTTAAAATCAAATTTTAATTATTATTAAGGATATTGAAGGATT<br>TTTAATTTATTATTTGAATTGATGAAATAATTTGAATCAAA

PhdNtxNav 13	O76199	1,45E-17	87,692	PF17492	9053.55<br>**7404.36**	MKVAILILSILVLAV<br>A**SETIEEYRDDFAVE**<br>**ELERDACAGQDQT**<br>**CKVTCDCCGERGE**<br>**CVCGGPCVCRQGN**<br>**FLIAWYKLINCKK***	ATAAAATTTGCATGTCGGAGAATTTTGATGCCTAAATGATAA<br>CGTCAGGTAAACAGATGTTCTTGAAATTTTATGAAATTGAAT<br>GTTAGATCCTATTTTTATTTCAATAAGAAGATATTAGGGATAT<br>ATAAGAGGACATCCGAACAGCAATTTATCAGATTCCCGTCG<br>GTCTTTGACAATACGAATCAGAATGAAAGTTGCAATCCTCAT<br>CCTCTCTATTTTGGTGCTTGCTGTTGCAAGTGAAACCATTGAA<br>GAATACCGTGATGATTTTGCTGTAGAAGAATTGGAGAGAGAT<br>GCATGCGCTGGCCAAGACCAGACCTGCAAGGTAACTTGCGA<br>CTGCTGTGGTGAGAGAGGAGAATGTGTTTGCGGAGGACCTTG<br>CGTTTGCAGGCAAGGCAACTTTTTGATAGCATGGTATAAACT<br>TATTAACTGTAAGAAATGATATGGATTTATGTATACCATGAT<br>ATAATAAATATTGTTGCATTTGACGTGTAGGAGTTCACTTTCA<br>TTTTTGTCCGTAATAGAAAGTTTATCCATTCTGAGGCAGGTAA<br>ATGCGCACTGCAGAGCTCAGTCCTGACCTGAATATCTGGGAA<br>ATTAATCAACTTTCTATCGCTGAAACTCACAGTCAATGTTAG<br>AAAAATAACTGCATGTTAATTTAGTGATGAGGTACGAGTAAT<br>TTTATGTACTCGTCGATACAATAAATATTACTGAAATTTTCCT<br>GTTGGCGTTCACTTTCATTTTTATCTGCATTGGAAAGTTTCTAC<br>ATTCTGAGTTTGGTAAAAGTATACCTGCAGGTCTTACTCTCGA<br>TCTGTATATCTGGTTATTGAAAACAACCCTTATCTCTAAGGAT<br>GTGCTAACATACTCCCGGTTGATGTGTACACAGTGTTAACGG<br>ATTGCAAAATACGGTAGCTTAACGTCATCAGTCAAAGTTGAA<br>CCAGACTGGCTAAAACTGAAATTCCTCCATTCTGAGTTTTGT<br>AAAAGCACACTGCTGAGCTCACTTTTGACTTGAATATCTAGG<br>ATGTGAATCGAGGCACTATCATTAAGGATTTGCTCACATATT<br>TGCGCGATTTAGTACGTATATATGATAAATCTGGCAGTTTTAA<br>CCAATCTTTGAACACGGTAGTTTAACGTAATGTATCAATTTA<br>AAACCCGATTGGTTCAAATAATTCCTGTTTCTTTTACCTTTTC

							AGTATAGTACATCAACTACTACCTACAAATTTCATATCATTTC<br>GCTCATTTGAGGCAGATCAATGCATTATTTAGTTTTATAATTT<br>ACTCTACTGGATAATGAAAAAATGTATCCTCCTGCACTTCAA<br>GACACTTTTAAAATTAATGTTATTTTTTCATTTCTAAAAATAG<br>CCGACGGAATTTAACTACCGAATGATTTACAGCCGTGTTATT<br>TTATTGCTGAAAGTGTTCGCTTTTCAAATTAACACCATTGTCA<br>TTAGGTGTCTCGTGTGAGTTCCATATTGTTTTCCTTATTTTACT<br>GTTCCTAATTTGTGCGTGTCATTTGAGAAATATTTCATTTATTG<br>GTGTACAGAAGCATTGCAAATATATCATGAGCAATATACGA<br>AAGTTTCTTCACCTGAAAACCGAAATATATACAAGGACATA<br>ATCGTACACACTGTATTATTGTTCAAAAAAGCAGAGGTTCCA<br>CGTTACAGCATGGAGTCGCAATACTTATCTCACCTCTGAAGA<br>AATAACGATTTTTCTTGAAGAACATTTAACCTTAGCCTTACTT<br>CTAGGGGTATTTGGCACCCAGGTATTCCAAGACTTAACGAAA<br>CAGGAGAACGCTGATCAATTTCAGAACAGAAGACTATAGCC<br>GCAGACTTAAGCTTTCATCATTTAAAAGCAAAGGACGATTGG<br>ATAAAAGTAATCGATAAAAAACAGTGAGAATGAAGAAATG<br>AAGGAATATAAGAATGAAAATATGCTCGATATTTTCCATATT<br>TTATATTTAAATAAATTATTTAGGATCCAAATTATATTATATT<br>CCTTTTAATGAAGTTTATTACTTCTCTTACTACTTGTACCACTG<br>TACATATTGATTTGACCCTTAAGAATCCAATTTTCTTCTTTAA<br>AATGTAAGATTATTCCTATTCAAGTATATTTACAATGTCTAAT<br>GCATTGGAGAATAGACTACGAACAACTGACTTACTTATTCAC<br>CGAATAGTTCAGTGACACATTAACTGGACATCCAGGGAAGC<br>TTAATTCATTTCGGTCTCAGTTTGGGGAACACTTCGTTTACAG<br>TCTTTCTTTGTATAAAATATGCATTATATAAAAAATCTACTTA<br>CAGTCGTGCTTAAAGTGGTTCAATATTGTTATTTAAAGGAAT<br>ATAAATTATTCACTGCATTTGCATACATAAAACTGAGACTTA

							TGTCCTATGCAAACAGACAAGATGTCTCTCTTCGATTCCAGG<br>GTATAAAATCTATTTTCGTTTATATGGCGAATCTTTGAGAAGT<br>ATAATACTCAAGTTGGAGCAAGCATGCAATGTATGAACGTTG<br>CCTGATCTCTGGTCAAAGAATACCAGTACAGTTTCTGTCAAA<br>ATTGTTAATCCTGTGGTGCAAGGATAGGCATTTTTCTTTTTAC<br>GTGTAATAAAATCCCCCCCCCCCTCTATCGACAAAGAATAAA<br>TAAGTACATCTGTTGCAGCTTTATTTCTTATTTTTAAGCATAA<br>ATTATCTCAAATTTACATTCCGCAGTTGAAGATATAAATGGC<br>ACAAACAAATACAATTTTAATGCAATTTGTAAGAGATCCATG<br>AGTGTAGCAGGTCTTAAGCCGTAGTTGTTGAGGTGAATTTAA<br>AATCACGTAATTGGTGACGTGGGGTGAAAAAGGACTTCCCTT<br>TTTAATAAACAAGATTGTGCGTATTATAAAAAAAAATATTAC<br>TTAATGGAATTACATTCTTTAATCTTTCTTAAAGGGA

PhdNtxNav 14	P59367	4,96E-51	93,827	PF17492	9108.48<br>**7343.20**	MKFAIVFLSLLVLAF<br>A**NESIEENREEFPVE**<br>**ESARCADINGACKS**<br>**DCDCCGDSVTCDC**<br>**YWSDKCVCRESNF**<br>**KLGMAIRKKFC***	TTTTAACGTCCAAATTGTTAAATTTAAAGGCTTCAGCACCTTC<br>TCGATTGACTTGAAATTTTCGATTCTTTAACAGGTGTACAAAG<br>CTATATCATGACAACCTTTTGGAAACAGATGGTCTGAGAATT<br>CCTAATAAAATGAATGCAAATTTTTTACTTAAGCACAAAGTT<br>TTGGTGTATATATAAAAGAGCAGAACTTGCCTATAGCACAAT<br>ATCAGATTTCCAGGAAATATTTGATACAACTCAGCATCTCAC<br>TTTCAAAATGAAGTTTGCAATCGTCTTCCTCTCTCTTCTGGTA<br>CTTGCTTTTGCAAATGAATCCATTGAAGAAAATAGGGAAGA<br>GTTCCCTGTTGAAGAATCGGCGAGATGCGCCGATATTAACGG<br>TGCTTGCAAGAGTGATTGCGACTGCTGTGGAGACTCGGTAAC<br>CTGTGATTGTTATTGGTCGGACAAATGCGTATGTAGGGAGAG<br>TAATTTTAAACTCGGTATGGCAATCAGAAAGAAATTCTGTTA<br>AGACAGCAAACAAGTCCAGCATGTATGCATTATCTCGGTGG<br>ATTTACGTATCTATTTTTGTACCTCGATACAATAAACGTTGTT<br>GCATTTGAAAAAAAAAAAAAAAAAAAAAAAAA
PhdNtxNav 15	P59367	1,42E-51	96,296	PF17492	9033.41<br>**7316.18**	MKVAIVFLSLLVLAF<br>A**SESIEENREEFPVE**<br>**ESARCADINGACKS**<br>**DCDCCGDSVTCDC**<br>**YWSDKCVCRESNF**<br>**KLGMAIRKKFC***	GTTCAAACAAATTGACAGTAATAAGCTGTCTCGATTGACATG<br>ATATTTTAGATCCTTTAACATCTGTACAAAGCTACATCATGAT<br>AACCTTTTGGAAACAGATGGTCTGGGAATTTCTAGTAAATTC<br>TTTGCTATTTTATATTTAAGTAGAAAGTTTTGGTGTATATATAT<br>AAGAGCAGGACTTGCCAATAACACATTATCAGATTTCCAGA<br>AAATATTTGATATAACAACTCAGAATGAAGGTTGCAATCGTG<br>TTCCTCTCTCTGCTGGTACTTGCTTTTGCAAGTGAATCCATTG<br>AAGAAAATAGGGAAGAGTTCCCTGTTGAAGAATCGGCGAGA<br>TGCGCCGATATTAACGGTGCTTGCAAGAGTGATTGCGACTGC<br>TGTGGAGACTCGGTAACCTGTGATTGTTATTGGTCGGACAAA<br>TGCGTATGTAGGGAGAGTAATTTTAAACTCGGTATGGCAATC<br>AGAAAGAAATTCTGTTAAGACAGCAAACAAGTCCAGCATGT<br>ATGCATTATCTCGGTGGATTTACGTATCTATTTTTGTACCTCG

							ATACAATAAACGTTGTTGCATTTGAAAAAAAAAAAAAAAAAA AAAAAAAA
PhdNtxNav 16	P59367	7,86E-11	76,667	PF17492	6474.73	PGDLFLLPVRRPPRS TLFPYTTLFRSVTCD CYWSYKCVGRESNF KLVMAIRKKIC*	AGGACTGACCTGGAGATTTATTTTTATTGCCGGTACGGCGAC CACCGAGATCTACACTCTTTCCCTACACGACGCTCTTCCGATC TGTAACCTGTGATTGTTATTGGTCGTACAAATGCGTAGGTAG GGAGAGTAATTTTAAACTCGTTATGGCAATCAGAAAGAAAA TCTGTTAAGACAGCGAACAAGTTCAGTATGTATGCATTAT

PhdNtxNav 17	O76199	4,00E-06	69,231	PF17492	5501.23	YHNQVYGKFSYCRF TYCFPTFIDVFFFSAC AGQDQTCKVTCDC CGER	CCTCTCTCACCACAGCAGTCGCAAGTTACCTTGCAGGTCTGG TCTTGGCCAGCGCATGCACTGAAAAAAAAGACATCAATGAA TGTAGGAAAACAATATGTAAACCTACAGTATGAGAATTTACC GTATACCTGATTATGGTATCACTAATATTGCTGAGTGATGAA ATTGCGATGTCCGCAATCAGAATAATGCAAGTAAAAGGAGA GTAAATGCTTAGCTATTAATTTGTCATGTAGAAGGCATAAAT GCGTTATGCACTCAATTTAGGTGTGTGTGTGACGCGATCGTTG GGGAAAAATATATTACAAATATCATGGCATTCGCAGAATAC AGGGAAATTTTAGTGTATATATATCTGAAATAGCAGGGAACT TGTCCTGCTAAATCAGAAAATGTTGGGAAATGTCCGGGAAA AACAGAAGTTGCAGTCGGAAAAGAAATCGGTAAACAGGAA CGAAAGTTGTAATGCAATCATGATCGGGATGGAGTGTTAACA AGGGAAAACAATCGGGAATCTTCTCAAGATGTTTATTGGAG ACTCATATTAAAACATTTTAGATTTAAAACAGGAGCAAATCT ATTTAAGGGGTCCGGAAAAGTCTGGCGGTGGGGGGCTTCT ACAACATGATTGGAGGCGATGGCGTTTTAGCGGGCTCTGATG AAAATTATTGCACGGGGGCTGTGGTTCTTATCGCGAGGTTAG TAAAGCCAATTAGATTTAGTTTCTGTTAGGTCAGGTGACGAA AGTACACGACTGAGAGAGCAGTGTAAAAGAGCAGCGTTATA TAATTTTGAGAGAGAGGTATTTCGTGGGGATGGGGAGATCTT ATTTGGCTTGAAAACCGCCGGAAGTCTGTTGGTGACTTCGAG GATGCCAAAAAAGAAGCCAAAGCGAAAATGGAATGAAACA AAAGGAAACTGGCTCCAACAACAGGAAAAACTGGAAATTTA GGGAAAGGGGCCGAAAAATCATTACATATATATATCAACTT GATATGAACATCGCTTAGCAGCACTTGACTCTCCTTAGCGGA GCTGTTGGTTGAAGCACAAAGTTTCCGTCCTTAGACTTAACA TAGAAACTTTGACTTGGCCTGAAAGGCCATTCAATATATCAT ACTCAAATTTCATGATTTCACGCAGATGGTGAGGATCAAACG

							AATCTAAATGACACCAAAATCATAAACCTAATATCAAGCAA<br>AGGGGTCAAAGAACGCTGCAAGAAAATGCATGTAGCCTTTT<br>TATATTAAAAATAAAATCATGCGCAATGGAAAAGAATTCCA<br>AAAAAAAGGAAAAGAAATCGATTAAAATAAGAGTAGTATG<br>TCGTTGTATCCTATGGTGTGACGTCATGTCTAGTTAAAAAAG<br>ACAGTACGTTGTTGTAGCTTATGTTGACATGTGTTGTGACGTC<br>ATATCTAGTGCTGCCATCTATTGACACACTTTTCTTAATTATA<br>CTTAATACATAATATATTAACTAATATATCAAACT

PhdNtxNav 18	P59367	3,07E-07	100,000	PF17492	4210,75	SYFVIFYSYIYSFFISFRCADINGACKSDCDCCGDSV	TATACATTTTTATGGAAGCACAAAATTATTATATATTTATTTCTATGTTCCCGAATATAATGTTTGCCGTTTTTTCAAGATCTACTTTAAAAAATACCTCGAAATCTCATTCAGAGTTTCCGGATTTATAAGATAGTATTCTTTTTCCCCTCCAATACAAGTAAGGAGATCTACATCTTCACAATCGAAATCATTCTCAAAATTTCAGAAGATTTTATTAGCAAAGAAGTACAACTTTCAACGTCTGGATTTTCGATTTCAATAGCAGCAGTTCCATAAATTCCCATTAAATGTACTTCTTTATTAAAGCGGTAATGTAACCAAATATTGACTTAATTTGTTTTAATACTGTCTAAGTTTTTTTTTTAAATATAATAACTTAGTGTTAATTATTTATTTTTGTCCTTTAAAAAATAATTCGTTCATTGTTTTAAATTTCATTTATGTTTAAAATAGATCAGCATACTATTTACTTTTTCGGAAAGAATTTTAGAGCAAATACAAATTATCTCTCTTATATTGTTGTATATTTAATATAATACATGTATATATGTAATCAAAACTCATTTGTTTGCAACGCCACCAGTCGATAATGAACACTCCTATATTTCCTGGACAAAACCTATTATACCGTGAGCGTCGGGGTGTTTGATCCTATTTCGTTATATTTTATTCGTACATTTATTCATTTTTTATTTCTTTCAGATGCGCCGATATTAACGGTGCTTGCAAGAGTGATTGCGACTGCTGTGGAGACTCGGTAA

PhdNtxNav 19	P59367	8,72E-07	100,000	PF17492	4210,75	SYFVIFYSYIYSFFISFRCADINGACKSDCDCCGDSV	AATGCATCATAGGTCTTTGTAATAATTGTAGAGTAAAATAAATTACTTTTTCTTCTTTTTAACACAGAGTTCCCTGTTGAAGAATCGGCGAGGTGAGTAACTTTGAAAATTATTTATTTTTTGCAGACGACACTTTCATGAAAACCAAATTTTCATTTCTTTTAGATCTTCATTGGAAAATGTGTTTTGAATTTTTATACAGGATGACGTTTCCAAACAGCTAATTTAAATTAAATCATAAATTAGACAATAATATTCGTGAATAATTTTTTGTTTAAATTTTTGCAAGTTGCAATAACAGATGTAAAAATAACAATGAAATATTCCTTATGTCAGGATTGTACTCAATATCACAGACAATAATTCACTTATCCGACTTATATGGTTATATTGAAAAGAAAACACGGAAATTATCAGAGACGGAGGATTTTGGAGAAATTTTATCCAAGGTACATTTGAATGCATTTTTATATATCTATCTCGACTCAAATAGAAACCACGATTAAATCCCGACATTCTTAATTATTGAAATTTCTCGTTTTTAAACACTATATCTGCAACTTGAAGATACAGATTTTCCTTAGTTCATTTAGTTTTTACGCAAATTACAACTAGACAAATATAAATTTTTTTATTTCTTCATCAACCGAAAGCTCAACTATTTCTGTCCGTCAGAGACCTTAAAATTTTCACATAATTATTTGGAACTATCTGTCTTCGTTACTTTTATGTTTTAGGCTAATTTTACATCTTAAAAGTTTATTTATGGAGACTGCTCATGATTTTTTTCTAATGAGTTATCTTCCCTAAATATCCATGCAAAACCAATATAAATGCATAGCGGAATTTACTGAAAATCCAAATTTTTTATAACAATGGGCAAACGCAGGCTTGATATGTAAAGAATGCGCTGTAATATAAGGAACATTTATAGGTAAGTTATATACTCTAATGGCTGAGTTAAAGTTTTTCCGTGACTTTTATTTAAATTAATTAAAAAAAATTACATATTCTACTTCATAGTACATAAAAGTCACTATTTGCTATTTCCCATGTCAACAAACTCATATTTGTATATTTCAGGAGGGAGAAAGATTGCATCATAGTATTGTAGTTTATTCCACAGGGAATAGGTATACATTTTTATGGAAGCACAAAAAATATTACATATTTATTTCTATGTTCTCGAATTT

							AATGTTTGCCCTATTTTCAAGATCTACTTTAAAAAACACCTCGAAATCTCGTTCAGAATTTCCGGATTTATAAGATAGTACTCTTTTTCCCCCTCCAATACAAGTAACGAGACCTACATCCTCACAGTCGAATTTATTCTCAAAATTTCGCTACTAGATTTTCTCCTTAATTAATTGTTGAAACATTTTATTTGCAAATAAGTATAATTATCAACCCCTGGATTTTCGATTTCAATAGCTGCAGTTCGATAAATTCCCATTAAATGTACTTCTTTATTAAAGCGGTAATGTAACCAAATATTGACTTAATTTGTTTTAATACTGTCTAAGTTTTTTTTTTAAATATAATAACTTAGTGTTAATTATTTATTTTTGTCCTTTAAAAAATAATTCGTTCATTGTTTTAAATTTCATTTATGTTTAAAATAGATCAGCATACTATTTACTTTTTCGGAAAGAATTTTAGAGCAAATACAAATTATCTCTCTTATATTGTTGTATATTTAATATAATACATGTATATATGTAATCAAAACTCATTTGTTTGCAACGCCACCAGTCGATAATGAACACTCCTATATTTCCTGGACAAAACCTATTATACCGTGAGCGTCGGGGTGTTTGATCCTATTTCGTTATATTTTATTCGTACATTTATTCATTTTTTATTTCTTTCAGATGCGCCGATATTAACGGTGCTTGCAAGAGTGATTGCGACTGCTGTGGAGACTCGGTAA

PhdNtxNav 20	P61229	3,82E-16	75,676		9371.69 **7322.18**	MKAVTFLIASLLMLAAASES**LEERREEFHLEKSERGNKCGDINAPCTRDCDCCGERGSCDCYWSSSCKCREGTFLTAWYKLAAC***	ATTTGATAATAAGATTCAGGATGAAAGCCGTAACCTTCTTGATCGCCTCTCTTTTGATGCTTGCTGCTGCAAGTGAATCGCTTGAAGAACGGAGAGAAGAGTTTCATCTTGAAAAATCGGAGAGAGGAAACAAATGTGGTGATATTAACGCACCTTGCACAAGAGACTGTGACTGCTGTGGAGAGAGGGGATCCTGTGATTGTTACTGGTCCAGCAGTTGTAAATGTAGAGAAGGCACATTTCTTACAGCATGGTATAAACTGGCTGCCTGTTAACTCTGTGATGAGGTGAATGCATGTAAACACGGATACAATAAATACTATTGCCTTTGAAAAAA
PhdNtxNav 21	P61229	3,14E-16	75,676		9343.64 **7322.18**	MKAATFLIASLLMLAAASES**LEERREEFHLEKSERGNKCGDINAPCTRDCDCCGERGSCDCYWSSSCKCREGTFLTAWYKLAAC***	TGATAATACGACTCAGAATGAAAGCCGCAACCTTCTTGATCGCCTCTCTTTTGATGCTTGCTGCTGCAAGTGAATCGCTTGAAGAACGGAGAGAAGAGTTTCATCTTGAAAAATCGGAGAGAGGAAACAAATGTGGTGATATTAACGCACCTTGCACAAGAGACTGTGACTGCTGTGGAGAGAGGGGATCCTGTGATTGTTACTGGTCCAGCAGTTGTAAATGTAGAGAAGGCACATTTCTTACAGCATGGTATAAACTGGCTGCCTGTTAACTCTGTGATGAGGTGAATGCATGTAAACACGGATACAATAAATACTATTGCCTTTGAAAAAA
PhdNtxNav 22	P61229	0	92,941		9475.88 **7758.65**	MKVAIVFLSLLVLAFA**SESIEENREEFPVEESARCGDINAPCQSDCDCCGYSVTCDCYWSKDCKCRESNFPAGMAVRKAFCKNKI***	GTTCAAACAAATTGACAGTAATAAGCTGTCTCGATTGACATGATATTTTAGATCCTTTAACATCTGTACAAAGCTACATCATGATAACCTTTTGGAAACAGATGGTCTGGGAATTTCTAGTAAATTCTTTGCTATTTTATATTTAAGTAGAAAGTTTTGGTGTATATATATAAGAGCAGGACTTGCCAATAACACATTATCAGATTTCCAGAAAATATTTGATATAACAACTCAGAATGAAGGTTGCAATCGTGTTCCTCTCTCTGCTGGTACTTGCTTTTGCAAGTGAATCCATTGAAGAAAATAGGGAAGAGTTCCCTGTTGAAGAATCGGCGAGATGCGGCGATATTAACGCTCCCTGCCAAAGTGATTGCGATTGCTGTGGATATTCGGTAACCTGTGACTGTTATTGGTCAAAGGACTGTAAATGCAGAGAGAGTAATTTTCCTGCCGGCATGGCAGTC

							AGAAAAGCCTTCTGCAAAAACAAAATATAATAACAGTATTA<br>ATTCATTTTTGAAGTGAATCTGTGTATACCTCGTTACAATAAA<br>CGTTGTTGCATTTGAAAAAAAAAAAAAAAAAAAAAAAA
PhdNtxNav 23	P61229	0	92,941		9475.88<br><br>**7758.65**	MKVAIVFLSLLVLAF<br>A**SESIEENREEFPVE<br>ESARCGDINAPCQS<br>DCDCCGYSVTCDC<br>YWSKDCKCRESNFP<br>AGMAVRKAFCKNK<br>I***	GTTCAAACAAATTGACAGTAATAAGCTGTCTCGATTGACATG<br>ATATTTTAGATCCTTTAACATCTGTACAAAGCTACATCATGAT<br>AACCTTTTGGAAACAGATGGTCTGGGAATTTCTAGTAAATTC<br>TTTGCTATTTTATATTTAAGTAGAAAGTTTTGGTGTATATATAT<br>AAGAGCAGGACTTGCCAATAACACATTATCAGATTTCCAGA<br>AAATATTTGATATAACAACTCAGAATGAAGGTTGCAATCGTG<br>TTCCTCTCTCTGCTGGTACTTGCTTTTGCAAGTGAATCCATTG<br>AAGAAAATAGGGAAGAGTTCCCTGTTGAAGAATCGGCGAGA<br>TGCGGCGATATTAACGCTCCCTGCCAAAGTGATTGCGATTGC<br>TGTGGATATTCGGTAACCTGTGACTGTTATTGGTCAAAGGAC<br>TGTAAATGCAGAGAGAGTAATTTTCCTGCCGGCATGGCAGTC<br>AGAAAAGCCTTCTGCAAAAACAAAATATAATAACAGTATTA<br>ATTCATTTTTGAAGTGAATCTGTGTATACCTCGTTACAATAAA<br>CGTTGTTGCATTTGACTTCTGGCATTTCACATTTATTTTTTTCT<br>ATATTATATAAAATGCATATTGACTAAAGTCGGTAGGATGGA<br>AACTAACGAGTTCGCTCTTCACCTGAAAGTTTGGGCGCGTAA<br>TGGATTTTCTATGTCTAAATGTAACAGCAAATTTTAAAACAA<br>AATTGCGAAACGCACTTATGGACGTGACTTGCGTGACTTCTT<br>TTGCCTCTTACATCACTTTCGATGAACACTAAAACAAAAAAT<br>TAAATCAACAACTGATATTCACCTAATTGACATTGACTGAGT

							GTGCTTCCCTAGCAACAAACTGAAAAGCAATATGAACTAATTTGAATGGCTATCTGTCGAACTAATTTAAATGTATCTCTTTAAACACATTAAAGGAAATAATTTATTTTGTTAAGGAATAATACCTGAATTTACTTATATTTACTTCGTTCATAAGAGTTGCTTAACTCTAGTAAGAGAGGAAAATGCAATCTAACATGTTAATACGTAACTCGATCAGAGCAAATGAATTAAACCATCATTATAAAATATAAATCTTTCTAACAGATTTCATATTTGCAACAATGTTTCTACATATAGCTTTTGTAAGGAGATCGACGGTAAGAAAAATTTCCTGGTTAACTTATACTAATCCAGTTATCATTCATTTTGGTTTCATTCAAAAGTTAGAAAGATAGAAGTGACGGAAAATTAAACATCGGTAATTAATCAGGACATAATATTACATAATATTACAGGACATAATATTTTAATCGCCGTTTAATTAACGTTTCACTTATCCTCGTTTTCGCTAATTTTGGCGTCAATAACGTAAATATGAGTAAATTTGAACTGAGTACAGTTTAGC
PhdNtxNav 24	P61229	0	85,227		9810.34<br>**7945.94**	MKVAIFFILSLFVLAVA**SESIEEKREEFPVEESARGKCGDINAPCQSDCDCCGYSVTCDCYWSKDCKCRESNFAAGMALRKAFCKNKI***	GTTCAAACAAATTGACAGTAATAAGCTGTCTCGATTGACATGATATTTTAGATCCTTTAACATCTGTACAAAGCTACATCATGATAACCTTTTGGAAACAGATGGTCTGGGAATTTCTAGTAAATTCTTTGCTATTTTATATTTAAGTAGAAAGTTTTGGTGTATATATATAAGAGCAGGACTTGCCAATAACACATTATCAGATTTCCAGAAAATATTTGATATAACAACTCAGAATGAAGGTTGCAATCTTCTTCATCCTCTCTCTTTTCGTACTTGCTGTTGCAAGTGAATCCATTGAAGAAAAAAGGAAGAGTTCCCTGTTGAAGAATCCGCGAGAGGAAAATGCGGCGATATAAACGCTCCCTGCCAAAGTGATTGCGATTGCTGTGGATATTCGGTAACCTGTGACTGTTATTGGT

							CAAAGGACTGCAAATGCAGAGAGAGTAATTTTGCGGCCGGT ATGGCATTGAGAAAAGCCTTCTGCAAAAACAAAATATAATA ACAGTATTAATTCACTTTTGAAGTGAATCTCTGTATACCTCGG TAAAATAAACGTTGTTGCATTTGAAAAAAAAAAAAAAAAAA AAAAA
PhdNtxNav 25	P59368	0	90		10944,16	ITVHSHVFPTRCFRL FVPIKRNSFRVRSIHG VDEFNDAHLLCRES DNTSSFGTPRQTDN GSSTGQNPHQTSHM FASGTADAVMLTVD YSSWGCFLGRP*	CTTCAACAGGGAACTCTTCCCTAGTTTCTTCAATGGATTCACT TACAAAAGCAAGTACCAGAAGAGAGAAGAATACGATTGCA ACCTTCATTCTGAAAGTGAGATGCTAAATAATTAACAAATAT TTTTATCATTGTTTATCGATGGTTGTAGAATTCACATAATATA TTTTACTGAGCATGAAACACACTAATTATTGTAGAATGCTGA ACGAGATGGTCTAGTAAATGCATACGAGATGGTCTTTGAGCT AAGTTTAAATAGCATTTAGAGCATGAAAACATTCATTTGTGA AAATGCACTTCATAAAATTGCCATCCATGGAAAAACGTAAG TGACTGTTATTGATGTAATTATTGATGTTTGTGGTTCGGGTTTT ATTAATTTTGTTGAGCTGTGTTTTCTGTCCTCAGACTACTTGCG GCACATTTCTGAGAATACACTCTCTCGACAAGCATCCACACC CACTCAAGTGTTTGCGATGAAATGCCTAACAGAAGGTTGTTG TATCGTAAATTCTCCAGTGGGGGAAAAAACTTCATTAAGGTC TTCCAAGGAAACACCCCCAAGAAGAATAATCAACGGTCAAC ATCACCGCGTCGGCCGTGCCGGAGGCAAACATGTGTGACGTT TGGTGTGGATTTTGGCCGGTGGAAGAGCCATTGTCGGTTTGTC GCGGAGTACCGAATGAAGAGGTATTGTCGCTTTCGCGGCAC AGCAGATGTGCATCATTAAATTCGTCAACTCCGTGAATAGAC CGTACTCTAAATGAGTTTCTTTTGATCGGGACGAAAAGCCTG AAACACCTTGTGGGAAAGACATGACTGTGCACGGTGATTTA

							GAAATTCCCCGGGCAGGTCGGATCAAAGGAACACCGACCTG AGAAAATATCTTGAAAAAAAACTCATTTAGCTAAATTGAAG AAATTCTCACGTAGTCGTCGAATTTTGAAGGCGGAACCACTC CCGACCATTCAGAAGTGTTGAAATTAAAAGGCCGGAGATCG GGGCCTATTAAGTAAGCTGGGATTTAAATTCCCGACTCTAAA TCGCTAAGGCGATTTCCCTTTGTTTTAAACTCCACCTACAGAG

PhdNtxNav 26	P61229	0,000000 00862	95,455		7509,61	MCVIKTRLCATPLVE LEHPSFSAQNLLDPV QSCFVTFYSYFHSLFS FRCGDINAPCQSDC DCCGYSV	TTTTTAGAGGCTCAGGATTTTGTGAATTATTGGTGAATTGTTG TCAGTGAAATTTAGTACAATCTTGAGATAAGATTGCACTAAA TTTCACTGACAATAATTTACTAGTGATTTGCTGAAGTAGGGA GGGAAAGACGGATATTTTTAGAGGCTCAGGATTTTGTAGAAA TTTTGTACAAGGAATATTTGAATGCATTTTTATATATTTATCTG GATTGAAATACTAACCTCGATCAAATCGACCTTCTCAAATCT TTAAATTTCTCGATTTTAAATACTATATCTATAAATATCTGAT ACAGCTTTTTCTTAGTTAATTTAGTTTATACGCAAATCACAAG TAGACAAATAGATATTTTATCGTTTCTTCATGAAACGAAAGC TCGAATATTTCTATCCGGCAGATGGCTTAATATTTTACATAGT TATTCGGAACTATCTTTTCTACATTATTTTTATGTTTTAGGCTA ATTTTATTTCTTAAAAGGTTATTTCTGGAGATTTCTCATGATTT TTTTCTAAGGCGTTATCTGCACTAAATATGCATGTAAAACGA ATAAAAATGCTTAGAGGAATTTACTGAAAATCTAAATTTTCT ATAGCAATAGCCAAATGCAGGATTGATATGTAAAATACGAT AAGTAACATATGGAACTTTTAAAGGTATGTTATATTGTCTAAT TGCTGAATTTAATATTTTCCGTTTACTTTATTTAAATTAATTAC AAACAAAAATTACATATTCTGCTTTATAGAACATAAAAGTCA TTATTTGATATTTCCCATGTCAGCAAACTCATATTTGTATATTT CAGGAAGGTAAAAAGATTTCATCCTTATTCTTGCCTATTCCG CAGGGAATAGATATACATTTTTATGGAAGCACAAAAGTATTA CATATTTATTTCTATGTTCCCGAATATAATGTTTGCCGTTTTTT CAAGATCTACTTTAAAAATCACCTCGAAATCTCGTTCAGAGT TTCCGGATTTATAAGATAGTACTCTTTTTAACACTCCAATAGA AGTAACGAAATCTACATTTTCATAATCGAAATCATTCTCAAA ATTTCACACCGCGATTTTCGCTTTAATTAATTGTTGAAAGATT TAATTAGCAAAGAAGTATAATTATCAACCCCTGTATTTTCGA TTTCAATAGCAGCAGTTCGATAAATTCGCATTAAATATACTT

							CTTTATTAAGGTCATAATGTGACCAAATATTGACTTCATTTGTTTTAATATTGTTTAAGTTTTTTTAAATATACTAAGTTTTAATTATTTATTTTTGTCTTTTAAATATATAATTCATTTTTCATTATTTGTTTTAAGTTTTATTCATGTTTAAAATCAATCATCACAATATTTACTTTTTCGGAAAGAATTTTAGAGCAAATACAAATTATCTCTCTTTTATTGCTGTATATTTATTAGAATACATGTATGTGTGTAATCAAAACTCGTTTGTGTGCAACGCCTCTAGTCGAACTTGAGCATCCCTCATTTTCCGCGCAAAACCTTTTAGACCCTGTTCAATCGTGTTTTGTTACATTTTATTCCTACTTTCATTCATTATTTTCTTTCAGATGCGGCGATATTAACGCTCCCTGCCAAAGTGATTGCGATTGCTGTGGATATTCGGTAA
Atracotoxins							
**Transcript ID**	**Uniprot ID reference protein**	**e-value**	**Identity (%)**	**PFAM domain**	**MW (Da)**	**Amino acid sequence**	**Nucleotide sequence**

PhdNtxAtx01	Q8MTX1	5,94E-05	28,090	PF17556	10828.7 **9254.64**	MKIFILALCIVSAVA**LQPKHCKSADECAEDECCVNHIIFDFFHGHCHKFGSEGDNCVPQKKDDKKKPVMHHFLCPCKEGLNCEATDEATILGKIIKI**	TAGCGGCGTTAACATCGAGAGAAACCATAAAAATGAAAATCTTCATTCTTGCTCTGTGCATCGTTTCAGCTGTGGCACTTCAACCTAAACACTGCAAGTCTGCGGATGAATGTGCGGAGGATGAATGCTGTGTCAACCACATTATATTCGATTTTTCCATGGCCACTGTCATAAATTCGGAAGCGAAGGTGACAATTGCGTACCACAGAAAAAGGACGATAAGAAAAAACCGGTCATGCATCATTTCCTATGTCCTTGCAAGGAGGGTCTAAATTGTGAAGCAACTGATGAAGCAACAATTCTTGGAAAAATTATCAAGATAA
PhdNtxAtx02	Q8MTX1	1,59E-09	37,037	PF17556	11834,37	GGKDARKYSQEELAVEVKVNCEKNDDCAEEECCVQIHPFTGKVCKNLRREGEHCFTHYQGKDEDFYKFMCPCVKDLNCEPEDVIKEGDITRIINPKCVTKPES*	AAATCATATTTGACGTAAATCAGTCATATGCTTCTCTCCTTCGACAGAGCTCGTAATAGGAAAAATGCCTCTTAAGACTCTGGTTTCGTCACACATTTGGGGTTGATGATTCGAGTTATGTCTCCTTCTTTTATGACGTCTTCTGGCTCACAATTTAAATCTTTAACACATGGACACATGAATTTGTAAAAATCTTCATCTTTCCCCTGATAGTGCGTGAAACAGTGTTCCCCTTCCCTCCTGAGGTTTTTGCAAACTTTTCCTGTAAAAGGGTGGATCTGAACGCAGCATTCTTCTTCGGCACAGTCATCATTCTTTTCGCAATTTACTTTTACCTCCACTGCTAACTCCTCTTGGCTGTACTTTCTGGCGTCTTTTCCTCCTTATCCTCTAATGAGCTGCATCTTCGTCCAAAATGCGGGCTCCAATTAATGCGTCGTGACTCTTTTAATGAACGAATCATTGAAAAACCTCATTAGAGACAGATTCTCACTTAACATATAACGGAAGAGGAGTACAATGTTTCTTCGTGTCATG

PhdNtxAtx03	Q5D232	2,14E-05	28,409	PF17556	11170.88<br>**8972.22**	MQISIFLVISLCSAVALSGYT**RQCTQPSDCGPNECCVLGMQRYSIPSCQPLGQIDDTCLMYNEPDDKNLSYPNGNVKVYGVYRLFCPCAPSLRCMEAHCTE***	CGTCACCAGAAAAAACACAGCTCTATCAAAACAGAACCGGTGTGTGAACTTATAAGACGTGAGTTTTATTGCTATTGGATGAAGAACTTGTAACGAAAGCCTCACTACAAAAGGGCGCGAATAATGTAGTTTGCTATTAAATACTTCAGCTTGCGGGCAACAAATTTGTTTAGGAGGAAATCTGAAGCTGCCTTTTCATTGTGAAGGGAAATCATGCAGATTTCAATTTTCTTGGTTATTTCATTGTGTTCAGCGGTTGCTTTGAGTGGGTATACAAGACAGTGTACACAACCAAGCGACTGTGGACCTAATGAATGCTGTGTTCTCGGAATGCAACGTTATTCAATTCCTTCCTGCCAACCTCTGGGACAAATTGATGATACTTGCTTGATGTACAATGAACCAGATGATAAAAATTTAAGTTACCCCAATGGAAATGTTAAAGTTTATGGAGTGTATAGATTATTCTGCCCATGTGCACCTTCGTTACGTTGCATGGAAGCTCATTGTACTGAATAACTGAATGTAGATATTTTCAATTGTCTTTCAACTTTTGCTGAAACTTGCTTTGTTTTATGAAAGATGTCATTACAGTCCATTACACACATGTTTATATTTGTTACTTAAATGCTCACTTTTATATTGTAAGAAACTAAGTCTTCTGCTTATTTTTATATTTTGCTTTGTATTAACTTGCACCTTTATATACGACAGAAGTTGCTCATGATGTGCCAATAAATTCAATTCACAAATCCAATAAGCAAAATTGTCTCAATTGTTTAAAGAACTGTAAAACATTAATTATAAGATTTAATACAAAA

PhdNtxAtx04	Q5D232	1,80E-05	28,409	PF17556	11170.88<br>**8972.22**	MQISIFLVISLCSAVALSGYT**RQCTQPSDCGPNECCVLGMQRYSIPSCQPLGQIDDTCLMYNEPDDKNLSYPNGNVKVYGVYRLFCPCAPSLRCMEAHCTE***	GCAACAAATTTGTTTAGGAGGAAGTGGAATCTCCAGAAGTA<br>CCGAACATTTGCTCACGTCTTTGTAAAACAAGGAAATCTGAA<br>GCTGCCTTTTCATTGTGAAGGGAAATCATGCAGATTTCAATTT<br>TCTTGGTTATTTCATTGTGTTCAGCGGTTGCTTTGAGTGGGTAT<br>ACAAGACAGTGTACACAACCAAGCGACTGTGGACCTAATGA<br>ATGCTGTGTTCTCGGAATGCAACGTTATTCAATTCCTTCCTGC<br>CAACCTCTGGGACAAATTGATGATACTTGCTTGATGTACAAT<br>GAACCAGATGATAAAAATTTAAGTTACCCCAATGGAAATGTT<br>AAAGTTTATGGAGTGTATAGATTATTCTGCCCATGTGCACCTT<br>CGTTACGTTGCATGGAAGCTCATTGTACTGAATAACTGAATG<br>TAGATATTTTCAATTGTCTTTCAACTTTTGCTGAAACTTGCTTT<br>GTTTTATGAAAGATGTCATTACAGTCCATTACACACATGTTTA<br>TATTTGTTACTTAAATGCTCACTTTTATATTGTAAGAAACTAA<br>GTCTTCTGCTTATTTTTATATTTTGCTTTGTATTAACTTGCACCT<br>TTATATACGACAGAAGTTGCTCATGATGTGCCAATAAATTCA<br>ATTCACAAATCCAATAAGCAAAATTGTCTCAATTGTTTAAAG<br>AACTGTAAAACATTAATTATAAGATTTAATACAAAA
PhdNtxAtx05	Q5D232	1,82E-05	28,409	PF17556	11170.88<br>**8972.22**	MQISIFLVISLCSAVALSGYT**RQCTQPSDCGPNECCVLGMQRYSIPSCQPLGQIDDTCLMYNEPDDKNLSYPNGNVKVYGVYRLFCPCAPSLRCMEAHCTE***	ATATCAAGGAGGTTTTTATAGTAATAGTTTTAAACTCGTGTTT<br>GTCCTTTGAGTTTTCCCAGTATTCGAACAAGGATTTGAAATCT<br>GAAGCTGCCTTTTCATTGTGAAGGGAAATCATGCAGATTTCA<br>ATTTTCTTGGTTATTTCATTGTGTTCAGCGGTTGCTTTGAGTGG<br>GTATACAAGACAGTGTACACAACCAAGCGACTGTGGACCTA<br>ATGAATGCTGTGTTCTCGGAATGCAACGTTATTCAATTCCTTC<br>CTGCCAACCTCTGGGACAAATTGATGATACTTGCTTGATGTA<br>CAATGAACCAGATGATAAAAATTTAAGTTACCCCAATGGAA<br>ATGTTAAAGTTTATGGAGTGTATAGATTATTCTGCCCATGTGC<br>ACCTTCGTTACGTTGCATGGAAGCTCATTGTACTGAATAACT<br>GAATGTAGATATTTTCAATTGTCTTTCAACTTTTGCTGAAACT

							TGCTTTGTTTTATGAAAGATGTCATTACAGTCCATTACACACA<br>TGTTTATATTTGTTACTTAAATGCTCACTTTTATATTGTAAGAA<br>ACTAAGTCTTCTGCTTATTTTTATATTTTGCTTTGTATTAACTT<br>GCACCTTTATATACGACAGAAGTTGCTCATGATGTGCCAATA<br>AATTCAATTCACAAATCCAATAAGCAAAATTGTCTCAATTGT<br>TTAAAGAACTGTAAAACATTAATTATAAGATTTAATACAAAA
PhdNtxAtx0<br>6	Q8MTX1	6,79E-06	39,726	PF17556	12407,13	DIVASADCEKDTDC<br>ASDECCTLVTGGFYF<br>LGYRKTCAKLLSEGE<br>RCIMFEKTIKQYSGD<br>YYELCPCKPELVCQP<br>GDYVNYPWVGKFG<br>YNLRCAKSNGTVET<br>GVVEVSSIWM*	TTGTGCCAGAATCTATTTCAACTCAAAATATTAACGTTTTAAA<br>TTAAATAATTGATTTTCCATGTTATCACTGTTAGAAAAACAG<br>GTTTGTGAAAACCGACAAACTATACATCCTACCCCGCCTACT<br>AATAAACTTACTACTCGCCGTCTAATTTTCAGGGTATAAATA<br>TTGGTATTGAAATTCTCTCAACCCTTCTCTCCGTTTTTCTCCTT<br>TCAAATAGGACATAGTCGCTTCTGCAGATTGCGAGAAAGAC<br>ACGGACTGCGCCTCGGACGAATGCTGCACACTTGTGACTGGA<br>GGATTTTATTTCCTGGGATATCGTAAAACCTGCGCGAAACTC<br>CTATCAGAAGGTGAAAGATGTATAATGTTTGAAAAGACCAT<br>AAAACAGTACAGCGGAGATTACTATGAATTGTGCCCCTGTAA<br>GCCCGAACTAGTTTGTCAACCGGGAGATTACGTGAACTACCC<br>TTGGGTTGGCAAATTCGGATACAATCTACGCTGTGCTAAAAG<br>CAACGGAACAGTGGAAACAGGTGTAGTGGAAGTATCAAGTA<br>TATGGATGTGACAAGCGCGGATGGTACCTATAGGAATTATTG<br>CTACAGGCATTGCAATAATATTATACTTGTAGGTCACTAATA<br>ATAATCCTGAGTCTTTGAAATAAAAGCTATAAAAACATTTGG<br>GTTTCTACTTTACAAATTTTATGGGTTCATCTGTATTTTTTTAT<br>ATGCTTTTGCTTAACGTGCTCTGTTTGTTTGTTTCTTTCATCAA

							ATCGCAAAACTCAAATTTGACCCACTAAATGATTTCTAAAAA ATCTG
PROTEASE INHIBITORS							
Serine protease inhibitors							
**Transcript ID**	**Uniprot ID reference protein**	**e-value**	**Identity (%)**	**PFAM domain**	**MW (Da)**	**Amino acid sequence**	**Nucleotide sequence**
PhdPInSeP01	B2BS84	1,06E-24	40,845	PF00014	19432.63<br>**17000.60**	MLKIRHHLLLFIAGVICINSSA**SFRIPRLKRSTDLACSQPPRSGMCMALFIHYYYNETSNTCNKFVYGGCGGTENNFETGEDCERTCGGTIIDSDRDYAPIFVGPDDYWQYDDEACDAPPATGDCETSIIRYYFDGEKCKTFVYGGCDGNLNNFR**	ATCTTTCAAGGATAAAGTTTAGAAATAACTGGGAAATTAACTCAGGAGTTTATAATCTCTAGATGAAATATGTTGAAAATTAGACATCACTTACTTCTTTTTATTGCTGGTGTTATATGCATAAATTCTAGCGCTTCATTTCGGATCCCAAGATTGAAGCGTTCAACAGACCTTGCCTGTTCACAGCCTCCACGTTCTGGCATGTGCATGGCATTATTCATTCATTACTATTACAATGAAACCTCAAACACCTGCAATAAATTCGTTTACGGAGGATGTGGAGGAACTGAAAACAATTTCGAGACAGGAGAAGATTGTGAAAGAACATGTGGCGGAACAATTATAGACAGCGACAGAGATTACGCCCCAATATTTGTAGGACCAGATGATTATTGGCAATATGATGATGAAGCTTGTGATGCTCCACCTGCGACAGGAGACTGTGAGACTAGTATTATCCGCT

						**TEEQCLDRCGYRIS EDE***	ACTATTTTGATGGGGAAAAGTGCAAAACATTCGTTTACGGAG GTTGTGATGGAAACCTAAACAACTTCAGAACCGAGGAACAA TGTTTAGACAGATGTGGCTACAGGATCTCAGAGGATGAATAA GAATATAAAGAGGAATGGATCAAGAGCAGTGCAACATTTCA AAGGGCATTTAATTATTCTAAGATTGAAAATATGCAATATTT GGTTTCTG
PhdPInSeP02	B2BS84	7,63E-25	40,845	PF00014	17615,29	SLEITASFRIPRLKRST DLACSQPPRSGMCM ALFIHYYYNETSNTC NKFVYGGCGGTEN NFETGEDCERTCGG TIIDSDRDYAPIFVGP DDYWQYDDEACDA PPATGDCETSIIRYYF DGEKCKTFVYGGCD GNLNNFRTEEQCLD RCGYRISEDE*	TAAAGTTTAGAAATAACTGCTTCATTTCGGATCCCAAGATTG AAGCGTTCAACAGACCTTGCCTGTTCACAGCCTCCACGTTCT GGCATGTGCATGGCATTATTCATTCATTACTATTACAATGAA ACCTCAAACACCTGCAATAAATTCGTTTACGGAGGATGTGGA GGAACTGAAAACAATTTCGAGACAGGAGAAGATTGTGAAAG AACATGTGGCGGAACAATTATAGACAGCGACAGAGATTACG CCCCAATATTTGTAGGACCAGATGATTATTGGCAATATGATG ATGAAGCTTGTGATGCTCCACCTGCGACAGGAGACTGTGAG ACTAGTATTATCCGCTACTATTTTGATGGGGAAAAGTGCAAA ACATTCGTTTACGGAGGTTGTGATGGAAACCTAAACAACTTC AGAACCGAGGAACAATGTTTAGACAGATGTGGCTACAGGAT CTCAGAGGATGAATAAGAATATAAAGAGGAATGGATCAAG AGCAGTGCAACATTTCAAAGGGCATTTAATTATTCTAAGATT GAAAATATGCAATATTTGGTTTCTG

PhdPInSeP03	W4VSH9	8,78E-27	43,077	PF00014	21080.79<br>**18930.02**	MKKDLLLILLVIGVL<br>GPIYG**NCFSFLRSGI**<br>**SVGSENPCSLPPEE**<br>**GICDAVFMKYFYNT**<br>**SSKRCETFIYGGCG**<br>**GNENNFWTEEECT**<br>**NACQESSRRRRIISV**<br>**SIKNPLARDECEYPL**<br>**NIGRCFGSQKRFYF**<br>**NGEDCEEFVYKGCL**<br>**GNPNNFRTRRQCM**<br>**DKCGHKIDNGSGE**<br>**AEGNRDEDNRLFSF**<br>**SLL***	TTTTTTTACGGGATTAATTGTTTATTTAACTCATAGGTTAATAC<br>ATGACATATGTCACAAAATCATTTACTGATGATTCCTTAAAA<br>ATTAGAGAAGAGAAAAGGAGAAAAGTCTGTTATCTTCATCT<br>CTGTTACCTTCTGCTTCACCACTTCCGTTGTCTATTTTATGTCC<br>ACACTTATCCATGCACTGCCTTCTGGTACGAAAATTGTTTGGA<br>TTGCCGAGGCAGCCCTTATAAACGAACTCCTCACAATCCTCT<br>CCGTTGAAATAAAATCTCTTTTGAGAACCGAAGCATCTTCCA<br>ATATTCAGGGGGTATTCGCACTCGTCCCGAGCAAGAGGATTC<br>TTTATACTGACACTTATTATTCTCCTCCTTCTGCTTGATTCTTG<br>GCAAGCATTAGTGCATTCCTCTTCAGTCCAAAAGTTGTTTTCA<br>TTACCACCACACCCTCCATAAATAAAAGTCTCACATCTTTTA<br>GAAGAAGTATTGTAAAAGTATTTCATGAAAACTGCATCACAT<br>ATGCCCTCTTCCGGGGGTAAAGAACAAGGATTTTCGCTCCCT<br>ACTGATATCCCGCTTCGGAGAAAACTAAAACAGTTCCCATAA<br>ATAGGACCAAGCACGCCAATTACCAGTAGGATGAGCAAAAG<br>ATCTTTCTTCATTTTAGCTTATGGCGAATTCCTACCTATATCA<br>AAACGGTCAAGTCGCTTCCATCCATTCAGTTAAACTTTTTGA<br>AATTAATATAAACAGAAGAGCAAGCCTTTCTGCGTGCGATA<br>GTACGAAGGGAGGAAGTTGGAATTATGGCGGTTGATTG
PhdPInSeP04	B2ZBB6	1,36E-12	50,000	PF00014	13413.39<br>**11262.62**	MKKDLLLILLVIGVL<br>GPIYG**KSSRRRRIIS**<br>**VSIKNPLARDECEY**<br>**PLNIGRCFGSQKRF**<br>**YFNGEDCEEFVYKG**<br>**CLGNPNNFRTRRQ**<br>**CMDKCGHKIDNGS**<br>**GEAEGNRDEDNRL**<br>**FSFSLL***	TTTTTTTACGGGATTAATTGTTTATTTAACTCATAGGTTAATAC<br>ATGACATATGTCACAAAATCATTTACTGATGATTCCTTAAAA<br>ATTAGAGAAGAGAAAAGGAGAAAAGTCTGTTATCTTCATCT<br>CTGTTACCTTCTGCTTCACCACTTCCGTTGTCTATTTTATGTCC<br>ACACTTATCCATGCACTGCCTTCTGGTACGAAAATTGTTTGGA<br>TTGCCGAGGCAGCCCTTATAAACGAACTCCTCACAATCCTCT<br>CCGTTGAAATAAAATCTCTTTTGAGAACCGAAGCATCTTCCA<br>ATATTCAGGGGGTATTCGCACTCGTCCCGAGCAAGAGGATTC<br>TTTATACTGACACTTATTATTCTCCTCCTTCTGCTTGATTTCCC

							ATAAATAGGACCAAGCACGCCAATTACCAGTAGGATGAGCA<br>AAAGATCTTTCTTCATTTTAGCTTATGGCGAATTCCTACCTAT<br>ATCAAAACGGTCAAGTCGCTTCCATCCATTCAGTTAAACTTTT<br>TGAAATTAATATAAACAGAAGAGCAAGCCTTTCTGCGTGCG<br>ATAGTACGAAGGGAGGAAGTTGGAATTATGGCGGTTGATTG
PhdPInSeP0<br>5	C0LNR2	3,84E-06	38,095	PF00014	4693.5<br><br>1190.28	LRKSSEVLSYPFCLLS<br>PPLGFCEVSIKKYSN<br>TLIKNYFYSG*<br>ISSINNATHYA*	TATTGCATGATCAATTACTGTTTTTAGTCAAGCGTAATGCGTG<br>GCGTTATTGATGCTAGAGATTCATCCACTATAGAAGTAGTTTT<br>TAATTAGTGTATTACTGTACTTCTTAATAGATACTTCACAAAA<br>TCCTAAAGGAGGGGATAGCAGGCAAAAGGGATAGGAAAGA<br>ACTTCCGACGATTTTCTGAGTTAAGCAAACAAAAGGGACAGT<br>TGAAATTTCTAGAG
PhdPInSeP0<br>6	P0DN06	3,56E-09	42,254	PF00014	8562.82<br><br>**6590.27**	MKLFILLALIGFLTA<br>VRA**EYNGPRACLM**<br>**QKETGSCKANLIRF**<br>**YYNTGTTKCEKFIY**<br>**GGCGGNSNNFNSQ**<br>**EDCDAVCSS***	TATCGATACAAATGTATACAAGCTATCATATAATATATAACA<br>AATGAATGATTCACGCATATTCCTAATATATTAATTTAAAAC<br>TAGTAACAGGCGTATTTAGTAAACATTTATTGAAGTTTGTAC<br>ATAGTTGAGAGTGATTATTCTTCTCTACTCAGTAAATCTGAAA<br>TGGAAAATCAAAAGTTATTTAATCGTTAAGTTTATAAAATGC<br>TATAATTGTGCTTCCTGTAATGCTTGTAGGGAATAGAATGGA<br>GATTTCTTGTAGGTGAAATCTCTTCAGCTAAGAGGAGCAAAC<br>TGCATCACAGTCTTCCTGGCTGTTGAAATTGTTGGAGTTTCCT<br>CCACAGCCTCCATAGATAAATTTCTCGCACTTCGTAGTGCCC<br>GTGTTGTAGTAGAACCTGATGAGATTTGCTTTGCAGGAACCT<br>GTTTCTTTCTGCATTAGACAAGCTCGTGGACCATTATATTCGG<br>CTCTTACTGCGGTAAGGAAACCGATAAGAGCAAGTAATATG<br>AACAATTTCATTGTAACGTCTTTTGGCAACGGCTTAAA

PhdPInSeP07	Q6T269	2,26E-29	42,958	PF00014	134888,34	ATDCATSEYGCCPD<br>NVTLALGADFEGCN<br>ETSNATDVPQTTTET<br>PAECLNTLYGCCPD<br>EVTTALGPNLEGCC<br>FGLRFGCCPDNKTA<br>ALGPQSLGCGCQTF<br>PYGCCPDNITPAQG<br>PGFYGCSCENFHFG<br>CCQDNITPAKGPQY<br>QGCECQFMYYGCCP<br>DGITPVISDKGEGCG<br>CDSTRYGCCPDGRT<br>AATSADLTGCPCTT<br>LPYGCCPDGYTPAR<br>TSDLRDCPCESQRYG<br>CCLDRRTIARGPNYE<br>GCPCDTTRFGCCQD<br>GKTTAQGPGFEGCP<br>CETTAYGCCADNYT<br>PAQGPRFEGCPDVL<br>VPKPKVSTEVCSLPK<br>EPGPCRNYTVKWFF<br>DLAYGGCSRFWYGG<br>CDGNGNVFSTQEEC<br>ESICVKPDGPEACLL<br>PMVTGSCDGKVPSW<br>YFDAGSKTCETFTYG	GCTACAGACTGCGCAACGAGTGAATACGGTTGTTGTCCTGAC<br>AACGTAACACTTGCTTTGGGCGCTGACTTTGAAGGATGTAAC<br>GAGACAAGTAATGCAACCGATGTGCCTCAAACAACAACGGA<br>AACTCCTGCCGAATGCTTAAATACGTTGTACGGATGTTGCCC<br>GGACGAAGTCACCACAGCACTTGGACCTAATCTTGAAGGGT<br>GCTGCTTTGGTTTAAGATTTGGATGCTGCCCGGATAACAAAA<br>CTGCAGCTTTAGGACCTCAGTCTCTCGGATGTGGTTGTCAGA<br>CTTTTCCATATGGGTGCTGTCCTGATAATATAACTCCAGCTCA<br>AGGACCTGGTTTCTATGGCTGCAGCTGCGAAAATTTCCATTTC<br>GGCTGCTGCCAGGACAACATCACTCCAGCTAAAGGGCCCCA<br>ATACCAGGGCTGCGAATGTCAGTTCATGTATTACGGATGCTG<br>TCCTGATGGTATAACTCCTGTAATCAGCGACAAAGGAGAGG<br>GCTGCGGGTGCGACTCAACACGCTATGGATGTTGTCCTGACG<br>GGCGAACAGCAGCAACAAGCGCGGACTTGACTGGTTGTCCT<br>TGCACCACCCTGCCTTATGGATGTTGTCCTGATGGCTACACCC<br>CGGCTAGGACATCGGACTTGAGAGACTGTCCATGTGAATCTC<br>AACGTTACGGTTGCTGTTTGGACAGAAGGACGATTGCAAGG<br>GGTCCCAATTACGAGGGTTGTCCATGTGATACCACACGATTC<br>GGATGCTGCCAGGATGGCAAGACCACCGCGCAAGGGCCAGG<br>TTTCGAAGGATGTCCTTGTGAGACAACAGCCTATGGTTGCTG<br>CGCTGATAACTACACTCCAGCACAAGGCCCTCGTTTCGAAGG<br>CTGTCCTGACGTATTAGTGCCCAAACCAAAAGTGTCAACAGA<br>GGTATGCAGTTTGCCGAAAGAACCAGGCCCCTGTAGAAATT<br>ACACCGTTAAATGGTTCTTTGACCTAGCTTATGGAGGTTGCTC<br>GAGATTCTGGTATGGTGGTTGCGATGGCAACGGAAATGTCTT<br>TTCAACGCAAGAAGAATGTGAAAGCATCTGTGTAAAACCAG<br>ACGGACCAGAAGCATGTCTTTTGCCAATGGTGACTGGATCAT<br>GTGACGGCAAGGTTCCTTCTTGGTATTTTGATGCCGGTTCAAA

						GCLGNSNRFSTKTD<br>CVEKCINNQPPPKP<br>VDKCTQPKDEGGCD<br>SAIIKWYYNGKNER<br>CEQFYYGGCKGNSN<br>RFESRRECEKSCLPA<br>SNVTDSVCQLPHEIG<br>PCNEFRERWYFDYE<br>NGQCHRFIYGGCKG<br>NENNFGSFPECEKR<br>CGRDTASEPPTDLEF<br>KTDYCFQIQDSGPCS<br>NNEVNFYYDSADGV<br>CKEFYYGGCKGNRN<br>KFKTRRDCEKSCFKA<br>QDVCSLPAVKGPCS<br>GTFPQWYYDKSQDD<br>CYEFSFSGCQGNAN<br>RFNDRDSCQQRCKK<br>APVTTEAPVITSDVC<br>SLPREPGPCLGYYRA<br>WYYDFTENACKSFIY<br>GGCDGNENRFDRRR<br>DCEQRCVRKKTPKE<br>SKKPLPDLPPTKPAN<br>EDVCRLPVDPGRCN<br>EEYARWFYDAQSQS<br>CLPFVYGGCEGNKN	GACCTGTGAAACTTTCACTTATGGAGGTTGCTTAGGTAACAG<br>CAACAGATTTTCGACCAAAACAGACTGTGTCGAGAAATGTA<br>TCAACAACCAACCTCCTCCTAAACCAGTGGACAAGTGTACCC<br>AGCCGAAAGACGAAGGTGGATGCGACAGTGCAATCATCAAA<br>TGGTACTATAACGGAAAGAACGAGAGATGTGAACAGTTCTA<br>TTATGGTGGCTGCAAAGGAAATTCCAACCGTTTCGAGTCGAG<br>ACGGGAATGTGAGAAGTCCTGTTTGCCCGCCAGTAACGTGAC<br>CGATTCCGTGTGCCAACTTCCCCACGAGATTGGCCCTTGCAA<br>CGAGTTCCGAGAGCGCTGGTACTTCGACTATGAAAACGGCC<br>AGTGCCACAGGTTCATCTACGGAGGGTGCAAAGGAAACGAA<br>AACAACTTCGGCTCCTTCCCGGAGTGCGAGAAAAGATGCGG<br>ACGGGACACAGCATCCGAACCACCAACGGATCTGGAATTCA<br>AGACAGATTACTGCTTCCAAATCCAGGACAGTGGTCCTTGTT<br>CCAACAATGAAGTGAACTTTTATTACGATTCTGCCGACGGAG<br>TCTGCAAAGAATTCTACTACGGTGGTTGCAAAGGAAACAGA<br>AACAAGTTCAAAACCAGAAGAGATTGCGAGAAGTCCTGTTT<br>CAAAGCTCAAGATGTTTGCAGCTTGCCCGCCGTGAAAGGTCC<br>GTGCAGTGGTACCTTCCCTCAGTGGTACTATGATAAATCCCA<br>AGATGATTGCTACGAGTTCTCATTCAGCGGTTGCCAAGGAAA<br>CGCCAATCGCTTCAATGACAGAGACAGTTGCCAACAGAGGT<br>GTAAAAAGGCACCTGTCACTACAGAAGCTCCTGTGATCACA<br>AGTGATGTATGCAGCTTGCCGAGAGAGCCCGGACCTTGCCTG<br>GGTTATTACCGTGCGTGGTACTATGACTTTACGGAGAATGCC<br>TGCAAATCTTTCATTTATGGAGGATGCGACGGAAACGAGAAT<br>AGATTCGACAGACGTAGAGACTGTGAACAGCGGTGCGTGAG<br>GAAAAAAACTCCTAAAGAATCCAAGAAACCACTGCCTGATC<br>TACCACCCACCAAGCCTGCTAACGAAGATGTCTGCCGTCTAC<br>CCGTCGATCCCGGTCGGTGCAACGAAGAGTATGCCAGGTGG

						RFKTAEICLRFCSGIK<br>VKSGSEEVPYVTPSA<br>TTTTTTLAPTTCPPS<br>NCAELQCPYGIEETF<br>DGIGCSSCRCSNPCE<br>VHTCPEGSRCAIDV<br>MRTGDAVRTEPVCR<br>RVQKPGICPSSALSS<br>GPRDCELRCRDDAD<br>CRGEHKCCNNGCTF<br>TCMPPADESFVEAR<br>QPEPATILPGVNVTQ<br>GEIGQPAELPCNAM<br>GWPRPTVTWWRDT<br>TMLPLSSSRYEQMPD<br>YALLIRSVVYEDRGD<br>YSCHAHNGIGSGAT<br>FKITLIIDFSPDASAR<br>GDENDVNARESEPE<br>TPSYSVDPYQDVPVS<br>ASVSLVTSNPMAGSP<br>LQVDCTTEGPPETKV<br>VWQFGESQIVVDDR<br>RRILSNHTLFITKAD<br>VSDTGEYRCTAQYH<br>NNVATSSVYVDVKV<br>MPTESSCVDNPQFN<br>NCNLIVQANYCNN	TTTTACGACGCCCAGAGCCAGAGTTGCTTGCCGTTCGTGTAT<br>GGTGGCTGCGAAGGGAACAAGAACAGGTTTAAGACAGCCG<br>AAATCTGCTTAAGATTCTGTTCTGGTATCAAAGTTAAATCTGG<br>AAGTGAGGAAGTGCCGTACGTGACGCCTTCAGCAACTACGA<br>CCACAACGACACTCGCGCCGACAACGTGCCCGCCATCCAAC<br>TGCGCCGAACTCCAATGTCCGTACGGTATCGAGGAAACATTT<br>GACGGCATTGGGTGCTCCTCCTGCAGGTGTTCTAATCCATGC<br>GAGGTGCACACCTGTCCCGAGGGAAGTCGCTGTGCCATAGA<br>CGTCATGCGAACTGGTGATGCTGTACGCACAGAACCAGTTTG<br>TAGGAGAGTTCAAAAGCCAGGCATCTGCCCGTCAAGTGCATT<br>GAGTTCTGGGCCCCGGGACTGCGAGCTTAGATGCAGGGATG<br>ATGCTGATTGCCGTGGAGAACACAAGTGTTGCAACAATGGCT<br>GTACCTTCACTTGTATGCCACCTGCAGATGAATCCTTTGTAGA<br>AGCTAGACAACCAGAACCTGCAACAATCCTGCCCGGTGTGA<br>ATGTTACACAAGGCGAAATAGGGCAACCAGCCGAACTGCCG<br>TGCAATGCCATGGGCTGGCCAAGACCTACCGTAACTTGGTGG<br>AGGGATACTACTATGCTACCCCTGTCCAGTAGTAGATACGAA<br>CAAATGCCCGACTATGCGCTGCTTATAAGGTCTGTGGTATAT<br>GAAGATAGAGGCGATTACTCGTGCCATGCTCACAATGGCATC<br>GGGTCGGGTGCCACGTTCAAAATAACGCTGATCATTGACTTC<br>TCACCAGATGCATCCGCAAGAGGAGATGAAAACGATGTGAA<br>TGCGAGAGAATCGGAACCAGAGACTCCCTCATATTCTGTCGA<br>TCCATATCAAGATGTGCCAGTTTCAGCCTCTGTGAGTCTCGTT<br>ACCAGCAATCCAATGGCCGGATCTCCCTTACAAGTGGACTGT<br>ACGACGGAAGGGCCCCCAGAAACGAAAGTAGTTTGGCAGTT<br>TGGAGAGAGTCAGATCGTAGTGGACGACAGGCGAAGGATAT<br>TGTCCAATCATACCTTATTTATCACCAAAGCAGACGTAAGCG<br>ATACCGGAGAGTATCGATGTACTGCACAGTATCACAATAAC

						KYYGKFCCASCMRA<br>GQLQDMPQV*	GTAGCTACCTCCTCCGTCTATGTAGATGTGAAAGTAATGCCA<br>ACTGAATCGTCGTGTGTCGACAACCCTCAGTTCAACAACTGC<br>AATTTAATCGTCCAAGCTAACTACTGCAACAACAAATACTAT<br>GGAAAATTTTGCTGTGCTTCTTGTATGAGGGCTGGCCAGTTAC<br>AAGACATGCCTCAAGTGTGATATGGTGGTTAAAATATACAA<br>AAAAAAACTTTGGTCGGAAAATAACAGCATTTTTTTTTTTAA<br>ATGCTGATTTTCAGGTTATAAATGACAGACTTTTTGTTCCTTTT<br>GATATTTTCATGAACTGACCAGCATTGGAAAATTTCTATCAA<br>ACTTTTAAGAACTTGAATAGAAAAATATTTTTTACTAAAGTA<br>TACCTACGGCAGTGTGTGTGTGTATTGTGACTATTTCATTTAT<br>TTTACATAATTTGAATATAATTTGCGTAAGAATGTGTAAATAT<br>GAATATTCTATTTACATGGGTTTTTTTTTTTCCATTGAACACCC<br>TGAAAAGACAAAATGCATTTTTTTTTTAAAGACTTGTGAACT<br>CAATTCAAAAATTGAACGACTTTTTCAGTTAATTTGAATATTC<br>TTCATAAGAGACATCTATAGTAGAATAAGCTGATACTTTCGT<br>CCTTACCTGTTTGAATAGAATAATTACATCCACCTAGAAATT<br>CAAATATTATAACTGCAAGAAAATTGCAATTTCAAAGCTAA<br>AATTTCAAGCACAAATATTGATTCTCAAGCATTTTGTGCTATT<br>TACTACGTATGATAATTTTCCATGTGATTTTTTTTTTTTTAATC<br>AGGGTTCTTACATACAAACGTGGTATTCTATACTGTGGAAGA<br>GGAGTAAATTAAGCTCTAGTCAGGGTATTTTTAGGAAGGAAT<br>GATTTATTATATCAAAAAATTATATGTAGTATATTTCTATGAA<br>TATATGAAATTATCGGATTATGAGTATAATCTAGCCTGTTCGT<br>TCTGCATAACGTATCTTGTCAGGAAAAACTGTGTCATTCCTA<br>GTCTTAAAGAAATTGATATAAGCATTTCATTAATGAAAATGA<br>AAGTTTTTAATCATCATGGATATATTTTTTTTAATCGTGTGAA<br>ATAATATTTAGTTCATATCTCAATTTAAATTATAATCAATACT<br>TTTTGCTTAGTAACAATAGAAGTGAAATAAACCCGTGACAAC

							AGCAAAGTTTAAGATACGCGAAGTGGCAACATGAAAAAATTATTTTTTTAGCTCTAGGATAGAAGAACCGAAAAATGATTTTTTTCCCCCTATATCTGGACGTACATTACCTGTGCATTCTTTAAAATGTCAAAATACAAATGAAAAAGATAGATGAATAAAATAATGGCATACTGCTTTATTTTCCTCATTTCGTTACTCATTTTTCTAACTAGATTAATACTGTAGAGTTAAAATTTCTCTTTATTTCCCTATATAAGTAATTATAGATATGTATGCAATATCATTTAGTACATGTATCTTTTATGCAAATTTTTCCTCTTTATTAGTCAGTATCCGTAATTATATATTACACACGTTATTAGTATGAAATTGCAGGCGATAAAGAGATTATTCTCTAGTTGTTTACAGACTGAAAACTATTGTAAAATATTTAACTACTAAAACTATCAGCGAACAACTGTATTAGCAAAAAAAATTCCATAACTTTCCTTACTTCTTATTAATTAGGTTCCATGAGAAACCTGGTTTATGAAGCATGACTTAGGATACAATTAGTGTGAGTGCATTTCAAACATTGGGAAAAACAAAGCTGCTGTAAATATTAATGGATTAATAGCATAAAATTGATTAAAAAGCTAAATTTCTTCTCTTTATAAAGGGAGATGAAAATAATACATCTATAATTATAATTCATAGTAACACTAGAAAATTTTTGAAGATACACTCAAGACCTTTAAAATTATATATATATACTATATATATATATGTTATATTATTAATTATAATTATAAAATATTGTGTGATAT

PhdPInSeP08	P0DMW6	5,39E-16	55,172	PF00014	96030.47<br><br>**93894.83**	MVSVFAVVTVWVA<br>LMGQCRG**ADPSAG**<br>**DSEPRMIPQSFPGR**<br>**CRRAPPGVTIPKVP**<br>**GDNGFYIKISGNPE**<br>**KYQPGELYTVTLQG**<br>**YSSQFAVQKFIGFM**<br>**LVVEPKEVRNDISA**<br>**RPDVGSFQLFGDA**<br>**MCKYSEECPNAIVH**<br>**ASLIPKQDIQVMW**<br>**KAPQAGTNCVVFK**<br>**ATVQENRELWYMD**<br>**EGGLIRELCEEEQEN**<br>**QDEQPEIIEECCACD**<br>**EAKYELTFEGLWSR**<br>**HTHPKEFPKNEWLT**<br>**HFSDIIGASHTGDF**<br>**RMWEYGGIASEGV**<br>**KEVAERGITKRLESE**<br>**LKTESNKIRTIIKAR**<br>**GLWYPNAQGRTFA**<br>**VFRVDRLHHLVSLL**<br>**TMLGPSPDWIVGV**<br>**SALELCLRNCSWVA**<br>**EKVMNLYPWDAGT**<br>**DSGATYITSKMPTV**<br>**PQEKIRRITSSTPNN**	CTTTCGAAGGCGACGCCGCGGGCATCACTGATCTACCGCGTC<br>GTTCCGATAAAGGAAGAGTGTTTCCCTCTCTTGTGTTCCAACA<br>CGTGGATCGAGTATCGTTCGAATTTTCGAGACCTCGGAACGG<br>TTGAGGTGATCTCGAAATGGTGTCTGTGTTCGCGGTCGTGAC<br>GGTGTGGGTCGCACTGATGGGGCAGTGCAGGGGCGCCGACC<br>CCTCGGCGGGTGACTCCGAGCCCCGCATGATCCCCCAGAGCT<br>TCCCTGGGCGTTGCCGTAGGGCACCTCCCGGTGTGACGATTC<br>CGAAGGTGCCGGGAGACAACGGTTTCTACATCAAGATATCC<br>GGAAACCCGGAGAAGTACCAACCAGGAGAGCTGTACACAG<br>TGACCCTCCAAGGCTACAGCTCCCAGTTCGCCGTACAGAAGT<br>TCATCGGCTTCATGCTGGTGGTGGAGCCCAAAGAGGTGAGG<br>AACGACATCTCGGCCAGGCCGGACGTCGGATCCTTCCAGCTG<br>TTCGGCGACGCCATGTGCAAGTACTCGGAGGAGTGTCCGAAT<br>GCCATCGTGCACGCCTCGCTCATCCCCAAGCAGGACATACA<br>GGTGATGTGGAAGGCGCCGCAGGCAGGGACCAATTGCGTCG<br>TCTTCAAGGCCACCGTTCAGGAGAATAGAGAACTTTGGTACA<br>TGGACGAAGGAGGTCTCATACGGGAGCTCTGCGAGGAAGAG<br>CAAGAGAACCAGGATGAGCAGCCGGAGATCATCGAGGAGT<br>GCTGCGCATGCGATGAGGCCAAGTACGAACTGACCTTCGAA<br>GGGCTCTGGTCCAGGCACACGCACCCCAAGGAATTTCCCAA<br>AAACGAGTGGCTGACGCATTTCTCCGACATCATCGGCGCCTC<br>CCACACGGGCGACTTCCGGATGTGGGAGTACGGAGGCATCG<br>CGTCAGAGGGCGTGAAAGAAGTGGCCGAGAGAGGGATCAC<br>CAAAAGGCTCGAGTCCGAACTCAAGACCGAGTCCAACAAGA<br>TCCGCACAATCATAAAGGCTCGAGGCTTATGGTACCCTAACG<br>CCCAAGGCAGGACGTTCGCCGTGTTCCGAGTGGACCGCCTGC<br>ATCACCTGGTATCTCTCCTGACCATGCTGGGTCCGAGCCCAG<br>ACTGGATAGTGGGAGTGAGTGCCCTCGAGCTCTGCTTGCGGA

						**PLSPFYDSSGAPMK**	ACTGCTCCTGGGTGGCCGAGAAAGTCATGAACCTGTACCCCT
						**PVARLTVTRQRVY**	GGGATGCGGGAACGGATAGTGGAGCCACTTACATCACTTCG
						**DKSCDDYEPVTPTH**	AAGATGCCGACGGTGCCTCAAGAGAAGATCCGGCGCATCAC
						**SYGTNDEDDERME**	GTCGTCCACGCCTAACAACCCCCTGTCTCCGTTCTACGACTC
						**CRVSDWTDWSDCS**	ATCTGGAGCACCCATGAAACCCGTCGCCCGTCTCACTGTGAC
						**VTCGQGHRMRSRQ**	CCGCCAACGTGTCTACGACAAGTCCTGTGACGATTACGAACC
						**YINKPKADMAGCT**	CGTTACGCCTACTCATTCGTACGGGACCAACGACGAGGATG
						**VPLVDKEVCSQHC**	ACGAGAGGATGGAGTGCCGCGTCAGCGACTGGACGGACTGG
						**VGDVSCRTTEWGE**	AGCGATTGCAGCGTGACGTGCGGCCAGGGCCACCGGATGCG
						**WSECSVTCGKGLR**	CTCGAGGCAGTACATCAACAAACCGAAGGCGGACATGGCCG
						**TRNRKYLDNQARK**	GGTGCACGGTGCCGCTCGTGGACAAGGAGGTCTGCTCCCAG
						**KCALELMEKETCTG**	CACTGCGTCGGGGACGTGAGCTGCCGCACGACGGAATGGGG
						**MKMTCEEEDRVTN**	AGAGTGGTCCGAGTGCAGCGTGACCTGCGGCAAGGGACTCC
						**GRRRNKQKEKNKS**	GGACTCGAAACCGGAAGTACCTGGACAACCAGGCGCGGAA
						**SGDHPPPADESPDP**	GAAGTGTGCCCTCGAACTCATGGAGAAGGAAACCTGCACCG
						**KCAVTHWSEWSPC**	GCATGAAGATGACGTGCGAAGAGGAAGACAGGGTGACCAA
						**TVTCGKGLKVRTR**	CGGCAGGCGGAGGAACAAACAGAAGGAGAAGAACAAGTCT
						**LYLSPAALSMCNVE**	TCCGGAGACCACCCCCCGCCAGCGGACGAATCACCGGATCC
						**LMQKAPCTADKPD**	AAAGTGCGCCGTCACCCACTGGTCCGAGTGGTCGCCTTGCAC
						**CTVNLTEAKKNCM**	TGTGACATGCGGGAAAGGCCTGAAGGTCCGAACCCGACTCT
						**QPKEVGPCRGYFPR**	ACCTAAGCCCGGCCGCACTCAGCATGTGTAACGTGGAGCTG
						**WYYDVGRTMCLQF**	ATGCAGAAGGCCCCCTGCACGGCGGATAAGCCGGACTGCAC
						**IYGGCRGNRNNFER**	AGTCAACCTCACCGAGGCAAAAAAGAACTGCATGCAACCCA
						**YADCNRMCETMLR**	AGGAGGTGGGCCCCTGCCGTGGCTACTTCCCCCGCTGGTACT
						**APLSALTPLSTSPAL**	ACGACGTCGGGCGGACGATGTGCCTGCAGTTCATTTACGGCG
						**PMATKDQPPIIDCV**	GATGCCGGGGCAACAGGAACAACTTTGAGCGGTACGCGGAC
						**VTPWSDWTSCTTS**	TGCAACAGAATGTGCGAAACCATGCTCAGAGCTCCGCTCAG
						**CGNGRRERRRMIK**	TGCCCTGACGCCACTCTCCACTTCGCCCGCCCTGCCCATGGC

						**LNPENGGKPCPAKL<br>VQRRKCKDNPPCP<br>DRLGDKEAVDCQL<br>NPWSDWTPCSKTC<br>GQGGTQQRTKTVK<br>VPPKNNGAACGPK<br>IERKYCTLPPCPLY***	GACCAAGGACCAACCACCGATCATCGACTGCGTGGTCACTC<br>CGTGGTCAGACTGGACGTCGTGCACGACGAGCTGCGGGAAC<br>GGACGCCGGGAGAGGCGGCGAATGATCAAGCTCAACCCGG<br>AGAACGGAGGGAAGCCGTGCCCTGCCAAGCTGGTGCAACGC<br>AGGAAATGCAAGGACAACCCGCCGTGCCCTGACAGACTGGG<br>GGACAAAGAAGCCGTGGACTGCCAACTGAACCCGTGGTCTG<br>ACTGGACGCCGTGCTCCAAGACCTGCGGTCAGGGGGGAACC<br>CAGCAGAGGACGAAGACGGTCAAGGTGCCCCCCAAGAACA<br>ACGGGGCAGCCTGCGGACCCAAGATCGAGAGGAAGTACTGC<br>ACCCTGCCGCCCTGCCCACTCTACTGAGGACCGAACGCAAA<br>AGGCAGAAAATAAGAACGGACTGATGTCGGACCTGAGGGA<br>GCTCCACATATCATCGTTTCGGCGAAACAGGTAGTTGTGAAC<br>GATGTGGAAACTTAACTGACATTTGTACATAATTTAAAGGCG<br>AGCAGCGAGACTCCACGCAAGATTTTGGTCATTCAGCCCCTC<br>TCCTCTCGTCAGAAAGGATGTTCCCCAAAGCTCCACCTGTGG<br>TTCGTAATCATCTCGACGTATGCTGCGACAGACTGTGTCCCGT<br>TTTGGGATGGAAAAACTTTTTTCGTTTCTTCTCAGAAAACAGA<br>AATAGGGAAGTTCTACGTGTTGTGTTAGAATTTGTTGTGTGTT<br>TGAATAGAAAAGTTCTTAAATGATGTGCGCGTTCGGTGTGTA<br>AAGCGTAGGGGCAGTAGAACGGAAAGCGGATTTAGGACAC<br>GGACGACATTTTTATAGTGTCTCGAAATGTGCTTCTTAATCTC<br>TTGTCATATCACTTCATCTATAGGATAAAACACGGGCATAAA<br>TTATAATGAAAAGTGAAGCTTAATTGTATAGGTTAAGCCCTA<br>AAGATTTAAAAAATCGTCCTTTGTTTTCTTCGAGAAAATGTG<br>ATTAAAATTCTTTTGTCAGTCTACAACAAATCGACCTTCAGTG<br>ACAACTTCTTCCTCGCAAACACTTACAACATTGAGTTTAAAT<br>GAAGCGATCCACGGCAGTCAATAATTCACCGGCATCACTTCT<br>GCCGAATTTTTCATGCCCCTTTTTTTTTTTCCTTGGACTACAAG

							CTGCCAGTTCTGTGTCATGTAACTAAAAAAGAACACTGAACT TGGAGACTTTCTACGAGAATATGTCGACTTTATATATATATAT ATATATATATATATATATATATATAT

PhdPInSeP09	P0DMW6	4,01E-16	55,172	PF00014	89952.47<br>**87816.84**	MVSVFAVVTVWVA<br>LMGQCRG**ADPSAG**<br>**DSEPRMIPQSFPGR**<br>**CRRAPPGVTIPKVP**<br>**GDNGFYIKISGNPE**<br>**KYQPGELYTVTLQG**<br>**YSSQFAVQKFIGFM**<br>**LVVEPKEVRNDISA**<br>**RPDVGSFQLFGDA**<br>**MCKYSEECPNAIVH**<br>**ASLIPKQDIQVMW**<br>**KAPQAGTNCVVFK**<br>**ATVQENRELWYMD**<br>**EGGLIRELCEEEQEN**<br>**QDEQPEIIEECCACD**<br>**EAKYELTFEGLWSR**<br>**HTHPKEFPKNEWLT**<br>**HFSDIIGASHTGDF**<br>**RMWEYGGIASEGV**<br>**KEVAERGITKRLESE**<br>**LKTESNKIRTIIKAR**<br>**GLWYPNAQGRTFA**<br>**VFRVDRLHHLVSLL**<br>**TMLGPSPDWIVGV**<br>**SALELCLRNCSWVA**<br>**EKVMNLYPWDAGT**<br>**DSGATYITSKMPTV**<br>**PQEKIRRITSSTPNN**	CACAGAATGTGTTTAATTTTATTTTTAAATATATATATATATA<br>TATATATATATATATAAAGTCGACATATTCTCGTAGAAAGTC<br>TCCAAGTTCAGTGTTCTTTTTTAGTTACATGACACAGAACTGG<br>CAGCTTGTAGTCCAAGGAAAAAAAAAAAAGGGGCATGAAAA<br>ATTCGGCAGAAGTGATGCCGGTGAATTATTGACTGCCGTGGA<br>TCGCTTCATTTAAACTCAATGTTGTAAGTGTTTGCGAGGAAG<br>AAGTTGTCACTGAAGGTCGATTTGTTGTAGACTGACAAAAGA<br>ATTTTAATCACATTTTCTCGAAGAAAACAAAGGACGATTTTTT<br>AAATCTTTAGGGCTTAACCTATACAATTAAGCTTCACTTTTCA<br>TTATAATTTATGCCCGTGTTTTATCCTATAGATGAAGTGATAT<br>GACAAGAGATTAAGAAGCACATTTCGAGACACTATAAAAAT<br>GTCGTCCGTGTCCTAAATCCGCTTTCCGTTCTACTGCCCCTAC<br>GCTTTACACACCGAACGCGCACATCATTTAAGAACTTTTCTA<br>TTCAAACACACAACAAATTCTAACACAACACGTAGAACTTC<br>CCTATTTCTGTTTTCTGAGAAGAAACGAAAAAAGTTTTTCCAT<br>[illegible]<br>[illegible]<br>[illegible]<br>CTCGCTGCTCGCCTTTAAATTATGTACAAATGTCAGTTAAGTT<br>TCCACATCGTTCACAACTACCTGTTTCGCCGAAACGATGATA<br>TGTGGAGCTCCCTCAGGTCCGACATCAGTCCGTTCTTATTTTC<br>TGCCTTCTTTGTCCCCCAGTCTGTCAGGGCACGGCGGGTTGTC<br>CTTGCATTTCCTGCGTTGCACCAGCTTGGCAGGGCACGGCTTC<br>CCTCCGTTCTCCGGGTTGAGCTTGATCATTCGCCGCCTCTCCC<br>GGCGTCCGTTCCCGCAGCTCGTCGTGCACGACGTCCAGTCTG<br>ACCACGGAGTGACCACGCAGTCGATGATCGGTGGTTGGTCCT<br>TGGTCGCCATGGGCAGGGCGGGCGAAGTGGAGAGTGGCGTC<br>AGGGCACTGAGCGGAGCTCTGAGCATGGTTTCGCACATTCTG

						**PLSPFYDSSGAPMK**	TTGCAGTCCGCGTACCGCTCAAAGTTGTTCCTGTTGCCCCGGC
						**PVARLTVTRQRVY**	ATCCGCCGTAAATGAACTGCAGGCACATCGTCCGCCCGACGT
						**DKSCDDYEPVTPTH**	CGTAGTACCAGCGGGGGAAGTAGCCACGGCAGGGGCCCACC
						**SYGTNDEDDERME**	TCCTTGGGTTGCATGCAGTTCTTTTTGCCTCGGTGAGGTTGA
						**CRVSDWTDWSDCS**	CTGTGCAGTCCGGCTTATCCGCCGTGCAGGGGGCCTTCTGCA
						**VTCGQGHRMRSRQ**	TCAGCTCCACGTTACACATGCTGAGTGCGGCCGGGCTTAGGT
						**YINKPKADMAGCT**	AGAGTCGGGTTCGGACCTTCAGGCCTTTCCCGCATGTCACAG
						**VPLVDKEVCSQHC**	TGCAAGGCGACCACTCGGACCAGTGGGTGACGGCGCACTTT
						**VGDVSCRTTEWGE**	GGATCCGGTGATTCGTCCGCTGGCGGGGGGTGGTCTCCGGAA
						**WSECSVTCGKGLR**	GACTTGTTCTTCTCCTTCTGTTTGTTCCTCCGCCTGCCGTTGGT
						**TRNRKYLDNQARK**	CACCCTGTCTTCCTCTTCGCACGTCATCTTCATGCCGGTGCAG
						**KCALELMEKETCTG**	GTTTCCTTCTCCATGAGTTCGAGGGCACACTTCTTCCGCGCCT
						**MKMTCEEEDRVTN**	GGTTGTCCAGGTACTTCCGGTTTCGAGTCCGGAGTCCCTTGCC
						**GRRRNKQKEKNKS**	GCAGGTCACGCTGCACTCGGACCACTCTCCCCATTCCGTCGT
						**SGDHPPPADESPDP**	GCGGCAGCTCACGTCCCCGACGCAGTGCTGGGAGCAGACCT
						**KCAVTHWSEWSPC**	CCTTGTCCACGAGCGGCACCGTGCACCCGGCCATGTCCGCCT
						**TVTCGKGLKVRTR**	TCGGTTTGTTGATGTACTGCCTCGAGCGCATCCGGTGGCCCTG
						**LYLSPAALSMCNVE**	GCCGCACGTCACGCTGCAATCGCTCCAGTCCGTCCAGTCGCT
						**LMQKAPCTADKPD**	GACGCGGCACTCCATCCTCTCGTCATCCTCGTCGTTGGTCCCG
						**CTVNLTEAKKNCM**	TACGAATGAGTAGGCGTAACGGGTTCGTAATCGTCACAGGA
						**QPKEVGPCRGYFPR**	CTTGTCGTAGACACGTTGGCGGGTCACAGTGAGACGGGCGA
						**WYYDVGRTMCLQF**	CGGGTTTCATGGGTGCTCCAGATGAGTCGTAGAACGGAGAC
						**IYGGCRGNRNNFER**	AGGGGGTTGTTAGGCGTGGACGACGTGATGCGCCGGATCTTC
						**YADCNRMCETMLR**	TCTTGAGGCACCGTCGGCATCTTCGAAGTGATGTAAGTGGCT
						**APLSALTPLSTSPAL**	CCACTATCCGTTCCCGCATCCCAGGGGTACAGGTTCATGACT
						**PMATKDQPPIIDCV**	TTCTCGGCCACCCAGGAGCAGTTCCGCAAGCAGAGCTCGAG
						**VTPWSDWTSCTTS**	GGCACTCACTCCCACTATCCAGTCTGGGCTCGGACCCAGCAT
						**CGNGRRERRRMIK**	GGTCAGGAGAGATACCAGGTGATGCAGGCGGTCCACTCGGA

						**LNPENGGKPCPAKLVQRRKCKDNPPCPDRLGDKEGRK***	ACACGGCGAACGTCCTGCCTTGGGCGTTAGGGTACCATAAGCCTCGAGCCTTTATGATTGTGCGGATCTTGTTGGACTCGGTCTTGAGTTCGGACTCGAGCCTTTTGGTGATCCCTCTCTCGGCCACTTCTTTCACGCCCTCTGACGCGATGCCTCCGTACTCCCACATCCGGAAGTCGCCCGTGTGGGAGGCGCCGATGATGTCGGAGAAATGCGTCAGCCACTCGTTTTTGGGAAATTCCTTGGGGTGCGTGTGCCTGGACCAGAGCCCTTCGAAGGTCAGTTCGTACTTGGCCTCATCGCATGCGCAGCACTCCTCGATGATCTCCGGCTGCTCATCCTGGTTCTCTTGCTCTTCCTCGCAGAGCTCCCGTATGAGACCTCCTTCGTCCATGTACCAAAGTTCTCTATTCTCCTGAACGGTGGCCTTGAAGACGACGCAATTGGTCCCTGCCTGCGGCGCCTTCCACATCACCTGTATGTCCTGCTTGGGGATGAGCGAGGCGTGCACGATGGCATTCGGACACTCCTCCGAGTACTTGCACATGGCGTCGCCGAACAGCTGGAAGGATCCGACGTCCGGCCTGGCCGAGATGTCGTTCCTCACCTCTTTGGGCTCCACCACCAGCATGAAGCCGATGAACTTCTGTACGGCGAACTGGGAGCTGTAGCCTTGGAGGGTCACTGTGTACAGCTCTCCTGGTTGGTACTTCTCCGGGTTTCCGGATATCTTGATGTAGAAACCGTTGTCTCCCGGCACCTTCGGAATCGTCACACCGGGAGGTGCCCTACGGCAACGCCCAGGGAAGCTCTGGGGGATCATGCGGGGCTCGGAGTCACCCGCCGAGGGGTCGGCGCCCCTGCACTGCCCCATCAGTGCGACCCACACCGTCACGACCGCGAACACAGACACCATTTCGAGATCACCTCAACCGTTCCGAGGTCTCGAAAATTCGAACGATACTCGATCCACGTGTTGGAACACAAGAGAGGGAAACACTCTTCCTTTATCGGAACGACGCGGTAGATCAGTGATGCCCGCGGCGTCGCCTTCGAAAG

PhdPInSeP10	P0DMW6	5,42E-16	55,172	PF00014	96030.47<br><br>**93894.83**	MVSVFAVVTVWVA<br>LMGQCRG**ADPSAG**<br>**DSEPRMIPQSFPGR**<br>**CRRAPPGVTIPKVP**<br>**GDNGFYIKISGNPE**<br>**KYQPGELYTVTLQG**<br>**YSSQFAVQKFIGFM**<br>**LVVEPKEVRNDISA**<br>**RPDVGSFQLFGDA**<br>**MCKYSEECPNAIVH**<br>**ASLIPKQDIQVMW**<br>**KAPQAGTNCVVFK**<br>**ATVQENRELWYMD**<br>**EGGLIRELCEEEQEN**<br>**QDEQPEIIEECCACD**<br>**EAKYELTFEGLWSR**<br>**HTHPKEFPKNEWLT**<br>**HFSDIIGASHTGDF**<br>**RMWEYGGIASEGV**<br>**KEVAERGITKRLESE**<br>**LKTESNKIRTIIKAR**<br>**GLWYPNAQGRTFA**<br>**VFRVDRLHHLVSLL**<br>**TMLGPSPDWIVGV**<br>**SALELCLRNCSWVA**<br>**EKVMNLYPWDAGT**<br>**DSGATYITSKMPTV**<br>**PQEKIRRITSSTPNN**	CACAGAATGTGTTTAATTTTATTTTTAAATATATATATATATA<br>TATATATATATATATAAAGTCGACATATTCTCGTAGAAAGTC<br>TCCAAGTTCAGTGTTCTTTTTTAGTTACATGACACAGAACTGG<br>CAGCTTGTAGTCCAAGGAAAAAAAAAAAAGGGGCATGAAAA<br>ATTCGGCAGAAGTGATGCCGGTGAATTATTGACTGCCGTGGA<br>TCGCTTCATTTAAACTCAATGTTGTAAGTGTTTGCGAGGAAG<br>AAGTTGTCACTGAAGGTCGATTTGTTGTAGACTGACAAAAGA<br>ATTTTAATCACATTTTCTCGAAGAAAACAAAGGACGATTTTTT<br>AAATCTTTAGGGCTTAACCTATACAATTAAGCTTCACTTTTCA<br>TTATAATTTATGCCCGTGTTTTATCCTATAGATGAAGTGATAT<br>GACAAGAGATTAAGAAGCACATTTCGAGACACTATAAAAAT<br>GTCGTCCGTGTCCTAAATCCGCTTTCCGTTCTACTGCCCCTAC<br>GCTTTACACACCGAACGCGCACATCATTTAAGAACTTTTCTA<br>TTCAAACACACAACAAATTCTAACACAACACGTAGAACTTC<br>CCTATTTCTGTTTTCTGAGAAGAAACGAAAAAAGTTTTTCCAT<br>CCCAAAACGGGACACAGTCTGTCGCAGCATACGTCGAGATG<br>ATTACGAACCACAGGTGGAGCTTTGGGGAACATCCTTTCTGA<br>CGAGAGGAGAGGGGCTGAATGACCAAAATCTTGCGTGGAGT<br>CTCGCTGCTCGCCTTTAAATTATGTACAAATGTCAGTTAAGTT<br>TCCACATCGTTCACAACTACCTGTTTCGCCGAAACGATGATA<br>TGTGGAGCTCCCTCAGGTCCGACATCAGTCCGTTCTTATTTTC<br>TGCCTTTTGCGTTCGGTCCTCAGTAGAGTGGGCAGGGCGGCA<br>GGGTGCAGTACTTCCTCTCGATCTTGGGTCCGCAGGCTGCCC<br>CGTTGTTCTTGGGGGGCACCTTGACCGTCTTCGTCCTCTGCTG<br>GGTTCCCCCCTGACCGCAGGTCTTGGAGCACGGCGTCCAGTC<br>AGACCACGGGTTCAGTTGGCAGTCCACGGCTTCTTTGTCCCC<br>CAGTCTGTCAGGGCACGGCGGGTTGTCCTTGCATTTCCTGCGT<br>TGCACCAGCTTGGCAGGGCACGGCTTCCCTCCGTTCTCCGGG

						**PLSPFYDSSGAPMK**	TTGAGCTTGATCATTCGCCGCCTCTCCCGGCGTCCGTTCCCGC
						**PVARLTVTRQRVY**	AGCTCGTCGTGCACGACGTCCAGTCTGACCACGGAGTGACC
						**DKSCDDYEPVTPTH**	ACGCAGTCGATGATCGGTGGTTGGTCCTTGGTCGCCATGGGC
						**SYGTNDEDDERME**	AGGGCGGGCGAAGTGGAGAGTGGCGTCAGGGCACTGAGCG
						**CRVSDWTDWSDCS**	GAGCTCTGAGCATGGTTTCGCACATTCTGTTGCAGTCCGCGT
						**VTCGQGHRMRSRQ**	ACCGCTCAAAGTTGTTCCTGTTGCCCCGGCATCCGCCGTAAA
						**YINKPKADMAGCT**	TGAACTGCAGGCACATCGTCCGCCCGACGTCGTAGTACCAGC
						**VPLVDKEVCSQHC**	GGGGGAAGTAGCCACGGCAGGGGCCCACCTCCTTGGGTTGC
						**VGDVSCRTTEWGE**	ATGCAGTTCTTTTTTGCCTCGGTGAGGTTGACTGTGCAGTCCG
						**WSECSVTCGKGLR**	GCTTATCCGCCGTGCAGGGGGCCTTCTGCATCAGCTCCACGT
						**TRNRKYLDNQARK**	TACACATGCTGAGTGCGGCCGGGCTTAGGTAGAGTCGGGTTC
						**KCALELMEKETCTG**	GGACCTTCAGGCCTTTCCCGCATGTCACAGTGCAAGGCGACC
						**MKMTCEEEDRVTN**	ACTCGGACCAGTGGGTGACGGCGCACTTTGGATCCGGTGATT
						**GRRRNKQKEKNKS**	CGTCCGCTGGCGGGGGGTGGTCTCCGGAAGACTTGTTCTTCT
						**SGDHPPPADESPDP**	CCTTCTGTTTGTTCCTCCGCCTGCCGTTGGTCACCCTGTCTTCC
						**KCAVTHWSEWSPC**	TCTTCGCACGTCATCTTCATGCCGGTGCAGGTTTCCTTCTCCA
						**TVTCGKGLKVRTR**	TGAGTTCGAGGGCACACTTCTTCCGCGCCTGGTTGTCCAGGT
						**LYLSPAALSMCNVE**	ACTTCCGGTTTCGAGTCCGGAGTCCCTTGCCGCAGGTCACGC
						**LMQKAPCTADKPD**	TGCACTCGGACCACTCTCCCCATTCCGTCGTGCGGCAGCTCA
						**CTVNLTEAKKNCM**	CGTCCCCGACGCAGTGCTGGGAGCAGACCTCCTTGTCCACGA
						**QPKEVGPCRGYFPR**	GCGGCACCGTGCACCCGGCCATGTCCGCCTTCGGTTTGTTGA
						**WYYDVGRTMCLQF**	TGTACTGCCTCGAGCGCATCCGGTGGCCCTGGCCGCACGTCA
						**IYGGCRGNRNNFER**	CGCTGCAATCGCTCCAGTCCGTCCAGTCGCTGACGCGGCACT
						**YADCNRMCETMLR**	CCATCCTCTCGTCATCCTCGTCGTTGGTCCCGTACGAATGAGT
						**APLSALTPLSTSPAL**	AGGCGTAACGGGTTCGTAATCGTCACAGGACTTGTCGTAGAC
						**PMATKDQPPIIDCV**	ACGTTGGCGGGTCACAGTGAGACGGGCGACGGGTTTCATGG
						**VTPWSDWTSCTTS**	GTGCTCCAGATGAGTCGTAGAACGGAGACAGGGGGTTGTTA
						**CGNGRRERRRMIK**	GGCGTGGACGACGTGATGCGCCGGATCTTCTCTTGAGGCACC

						<u>**LNPENGGKPCPAKL VQRRKCKDNPPCP DRLGDKEAVDCQL NPWSDWTPCSKTC GQGGTQQRTKTVK VPPKNNGAACGPK IERKYCTLPPCPLY***</u>	GTCGGCATCTTCGAAGTGATGTAAGTGGCTCCACTATCCGTT CCCGCATCCCAGGGGTACAGGTTCATGACTTTCTCGGCCACC CAGGAGCAGTTCCGCAAGCAGAGCTCGAGGGCACTCACTCC CACTATCCAGTCTGGGCTCGGACCCAGCATGGTCAGGAGAG ATACCAGGTGATGCAGGCGGTCCACTCGGAACACGGCGAAC GTCCTGCCTTGGGCGTTAGGGTACCATAAGCCTCGAGCCTTT ATGATTGTGCGGATCTTGTTGGACTCGGTCTTGAGTTCGGACT CGAGCCTTTTGGTGATCCCTCTCTCGGCCACTTCTTTCACGCC CTCTGACGCGATGCCTCCGTACTCCCACATCCGGAAGTCGCC CGTGTGGGAGGCGCCGATGATGTCGGAGAAATGCGTCAGCC ACTCGTTTTTGGGAAATTCCTTGGGGTGCGTGTGCCTGGACCA GAGCCCTTCGAAGGTCAGTTCGTACTTGGCCTCATCGCATGC GCAGCACTCCTCGATGATCTCCGGCTGCTCATCCTGGTTCTCT TGCTCTTCCTCGCAGAGCTCCCGTATGAGACCTCCTTCGTCCA TGTACCAAAGTTCTCTATTCTCCTGAACGGTGGCCTTGAAGA CGACGCAATTGGTCCCTGCCTGCGGCGCCTTCCACATCACCT GTATGTCCTGCTTGGGGATGAGCGAGGCGTGCACGATGGCAT TCGGACACTCCTCCGAGTACTTGCACATGGCGTCGCCGAACA GCTGGAAGGATCCGACGTCCGGCCTGGCCGAGATGTCGTTCC TCACCTCTTTGGGCTCCACCACCAGCATGAAGCCGATGAACT TCTGTACGGCGAACTGGGAGCTGTAGCCTTGGAGGGTCACTG TGTACAGCTCTCCTGGTTGGTACTTCTCCGGGTTTCCGGATAT CTTGATGTAGAAACCGTTGTCTCCCGGCACCTTCGGAATCGT CACACCGGGAGGTGCCCTACGGCAACGCCCAGGGAAGCTCT GGGGGATCATGCGGGGCTCGGAGTCACCCGCCGAGGGGTCG GCGCCCCTGCACTGCCCCATCAGTGCGACCCACACCGTCACG ACCGCGAACACAGACACCATTTCGAGATCACCTCAACCGTTC CGAGGTCTCGAAAATTCGAACGATACTCGATCCACGTGTTGG

							AACACAAGAGAGGGAAACACTCTTCCTTTATCGGAACGACG CGGTAGATCAGTGATGCCCGCGGCGTCGCCTTCGAAAG

PhdPInSeP11	P0DMW6	4,05E-16	55,172	PF00014	86224.5<br><br>**84088.87**	MVSVFAVVTVWVA<br>LMGQCRG**ADPSAG**<br>**DSEPRMIPQSFPGR**<br>**CRRAPPGVTIPKVP**<br>**GDNGFYIKISGNPE**<br>**KYQPGELYTVTLQG**<br>**YSSQFAVQKFIGFM**<br>**LVVEPKEVRNDISA**<br>**RPDVGSFQLFGDA**<br>**MCKYSEECPNAIVH**<br>**ASLIPKQDIQVMW**<br>**KAPQAGTNCVVFK**<br>**ATVQENRELWYMD**<br>**EGGLIRELCEEEQEN**<br>**QDEQPEIIEECCACD**<br>**EAKYELTFEGLWSR**<br>**HTHPKEFPKNEWLT**<br>**HFSDIIGASHTGDF**<br>**RMWEYGGIASEGV**<br>**KEVAERGITKRLESE**<br>**LKTESNKIRTIIKAR**<br>**GLWYPNAQGRTFA**<br>**VFRVDRLHHLVSLL**<br>**TMLGPSPDWIVGV**<br>**SALELCLRNCSWVA**<br>**EKVMNLYPWDAGT**<br>**DSGATYITSKMPTV**<br>**PQEKIRRITSSTPNN**	CACAGAATGTGTTTAATTTTATTTTTAAATATATATATATATA<br>TATATATATATATATAAAGTCGACATATTCTCGTAGAAAGTC<br>TCCAAGTTCAGTGTTCTTTTTTAGTTACATGACACAGAACTGG<br>CAGCTTGTAGTCCAAGGAAAAAAAAAAAGGGGCATGAAAA<br>ATTCGGCAGAAGTGATGCCGGTGAATTATTGACTGCCGTGGA<br>TCGCTTCATTTAAACTCAATGTTGTAAGTGTTTGCGAGGAAG<br>AAGTTGTCACTGAAGGTCGATTTGTTGTAGACTGACAAAAGA<br>ATTTTAATCACATTTTCTCGAAGAAAACAAAGGACGATTTTTT<br>AAATCTTTAGGGCTTAACCTATACAATTAAGCTTCACTTTTCA<br>TTATAATTTATGCCCGTGTTTTATCCTATAGATGAAGTGATAT<br>GACAAGAGATTAAGAAGCACATTTCGAGACACTATAAAAAT<br>GTCGTCCGTGTCCTAAATCCGCTTTCCGTTCTACTGCCCCTAC<br>GCTTTACACACCGAACGCGCACATCATTTAAGAACTTTTCTA<br>TTCAAACACACAACAAATTCTAACACAACACGTAGAACTTC<br>CCTATTTCTGTTTTCTGAGAAGAAACGAAAAAAGTTTTTCCAT<br>CCCAAAACGGGACACAGTCTGTCGCAGCATACGTCGAGATG<br>ATTACGAACCACAGGTGGAGCTTTGGGGAACATCCTTTCTGA<br>CGAGAGGAGAGGGGCTGAATGACCAAAATCTTGCGTGGAGT<br>CTCGCTGCTCGCCTTTAAATTATGTACAAATGTCAGTTAAGTT<br>TCCACATCGTTCACAACTACCTGTTTCGCCGAAACGATGATA<br>TGTGGAGCTCCCTCAGGTCCGACATCAGTCCGTTCTTATTTTC<br>TGCGGCACGGCGGGTTGTCCTTGCATTTCCTGCGTTGCACCA<br>GCTTGGCAGGGCACGGCTTCCCTCCGTTCTCCGGGTTGAGCTT<br>GATCATTCGCCGCCTCTCCCGGCGTCCGTTCCCGCAGCTCGTC<br>GTGCACGACGTCCAGTCTGACCACGGAGTGACCACGCAGTC<br>GATGATCGGTGGTTGGTCCTTGGTCGCCATGGGCAGGGCGGG<br>CGAAGTGGAGAGTGGCGTCAGGGCACTGAGCGGAGCTCTGA<br>GCATGGTTTCGCACATTCTGTTGCAGTCCGCGTACCGCTCAA

						PLSPFYDSSGAPMK<br>PVARLTVTRQRVY<br>DKSCDDYEPVTPTH<br>SYGTNDEDDERME<br>CRVSDWTDWSDCS<br>VTCGQGHRMRSRQ<br>YINKPKADMAGCT<br>VPLVDKEVCSQHC<br>VGDVSCRTTEWGE<br>WSECSVTCGKGLR<br>TRNRKYLDNQARK<br>KCALELMEKETCTG<br>MKMTCEEEDRVPD<br>PKCAVTHWSEWSP<br>CTVTCGKGLKVRT<br>RLYLSPAALSMCNV<br>ELMQKAPCTADKP<br>DCTVNLTEAKKNC<br>MQPKEVGPCRGYF<br>PRWYYDVGRTMCL<br>QFIYGGCRGNRNN<br>FERYADCNRMCET<br>MLRAPLSALTPLSTS<br>PALPMATKDQPPII<br>DCVVTPWSDWTSC<br>TTSCGNGRRERRR<br>MIKLNPENGGKPCP	AGTTGTTCCTGTTGCCCCGGCATCCGCCGTAAATGAACTGCA<br>GGCACATCGTCCGCCCGACGTCGTAGTACCAGCGGGGGAAG<br>TAGCCACGGCAGGGGCCCACCTCCTTGGGTTGCATGCAGTTC<br>TTTTTTGCCTCGGTGAGGTTGACTGTGCAGTCCGGCTTATCCG<br>CCGTGCAGGGGGCCTTCTGCATCAGCTCCACGTTACACATGC<br>TGAGTGCGGCCGGGCTTAGGTAGAGTCGGGTTCGGACCTTCA<br>GGCCTTTCCCGCATGTCACAGTGCAAGGCGACCACTCGGACC<br>AGTGGGTGACGGCGCACTTTGGATCCGGCACCCTGTCTTCCT<br>CTTCGCACGTCATCTTCATGCCGGTGCAGGTTTCCTTCTCCAT<br>GAGTTCGAGGGCACACTTCTTCCGCGCCTGGTTGTCCAGGTA<br>CTTCCGGTTTCGAGTCCGGAGTCCCTTGCCGCAGGTCACGCT<br>GCACTCGGACCACTCTCCCCATTCCGTCGTGCGGCAGCTCAC<br>GTCCCCGACGCAGTGCTGGGAGCAGACCTCCTTGTCCACGAG<br>CGGCACCGTGCACCCGGCCATGTCCGCCTTCGGTTTGTTGAT<br>GTACTGCCTCGAGCGCATCCGGTGGCCCTGGCCGCACGTCAC<br>GCTGCAATCGCTCCAGTCCGTCCAGTCGCTGACGCGGCACTC<br>CATCCTCTCGTCATCCTCGTCGTTGGTCCCGTACGAATGAGTA<br>GGCGTAACGGGTTCGTAATCGTCACAGGACTTGTCGTAGACA<br>CGTTGGCGGGTCACAGTGAGACGGGCGACGGGTTTCATGGG<br>TGCTCCAGATGAGTCGTAGAACGGAGACAGGGGGTTGTTAG<br>GCGTGGACGACGTGATGCGCCGGATCTTCTCTTGAGGCACCG<br>TCGGCATCTTCGAAGTGATGTAAGTGGCTCCACTATCCGTTCC<br>CGCATCCCAGGGGTACAGGTTCATGACTTTCTCGGCCACCCA<br>GGAGCAGTTCCGCAAGCAGAGCTCGAGGGCACTCACTCCCA<br>CTATCCAGTCTGGGCTCGGACCCAGCATGGTCAGGAGAGAT<br>ACCAGGTGATGCAGGCGGTCCACTCGGAACACGGCGAACGT<br>CCTGCCTTGGGCGTTAGGGTACCATAAGCCTCGAGCCTTTAT<br>GATTGTGCGGATCTTGTTGGACTCGGTCTTGAGTTCGGACTCG

						<u>**AKLVQRRKCKDNPPCRRK***</u>	AGCCTTTTGGTGATCCCTCTCTCGGCCACTTCTTTCACGCCCTCTGACGCGATGCCTCCGTACTCCCACATCCGGAAGTCGCCCGTGTGGGAGGCGCCGATGATGTCGGAGAAATGCGTCAGCCACTCGTTTTTGGGAAATTCCTTGGGGTGCGTGTGCCTGGACCAGAGCCCTTCGAAGGTCAGTTCGTACTTGGCCTCATCGCATGCGCAGCACTCCTCGATGATCTCCGGCTGCTCATCCTGGTTCTCTTGCTCTTCCTCGCAGAGCTCCCGTATGAGACCTCCTTCGTCCATGTACCAAAGTTCTCTATTCTCCTGAACGGTGGCCTTGAAGACGACGCAATTGGTCCCTGCCTGCGGCGCCTTCCACATCACCTGTATGTCCTGCTTGGGGATGAGCGAGGCGTGCACGATGGCATTCGGACACTCCTCCGAGTACTTGCACATGGCGTCGCCGAACAGCTGGAAGGATCCGACGTCCGGCCTGGCCGAGATGTCGTTCCTCACCTCTTTGGGCTCCACCACCAGCATGAAGCCGATGAACTTCTGTACGGCGAACTGGGAGCTGTAGCCTTGGAGGGTCACTGTGTACAGCTCTCCTGGTTGGTACTTCTCCGGGTTTCCGGATATCTTGATGTAGAAACCGTTGTCTCCCGGCACCTTCGGAATCGTCACACCGGGAGGTGCCCTACGGCAACGCCCAGGGAAGCTCTGGGGGATCATGCGGGGCTCGGAGTCACCCGCCGAGGGGTCGGCGCCCCTGCACTGCCCCATCAGTGCGACCCACACCGTCACGACCGCGAACACAGACACCATTTCGAGATCACCTCAACCGTTCCGAGGTCTCGAAAATTCGAACGATACTCGATCCACGTGTTGGAACACAAGAGAGGGAAACACTCTTCCTTTATCGGAACGACGCGGTAGATCAGTGATGCCCGCGGCGTCGCCTTCGAAAG

PhdPInSeP12	P0DMW6	4,09E-16	55,172	PF00014	93114.34<br>**90978.71**	MVSVFAVVTVWVA<br>LMGQCRG**ADPSAG**<br>**DSEPRMIPQSFPGR**<br>**CRRAPPGVTIPKVP**<br>**GDNGFYIKISGNPE**<br>**KYQPGELYTVTLQG**<br>**YSSQFAVQKFIGFM**<br>**LVVEPKEVRNDISA**<br>**RPDVGSFQLFGDA**<br>**MCKYSEECPNAIVH**<br>**ASLIPKQDIQVMW**<br>**KAPQAGTNCVVFK**<br>**ATVQENRELWYMD**<br>**EGGLIRELCEEEQEN**<br>**QDEQPEIIEECCACD**<br>**EAKYELTFEGLWSR**<br>**HTHPKEFPKNEWLT**<br>**HFSDIIGASHTGDF**<br>**RMWEYGGIASEGV**<br>**KEVAERGITKRLESE**<br>**LKTESNKIRTIIKAR**<br>**GLWYPNAQGRTFA**<br>**VFRVDRLHHLVSLL**<br>**TMLGPSPDWIVGV**<br>**SALELCLRNCSWVA**<br>**EKVMNLYPWDAGT**<br>**DSGATYITSKMPTV**<br>**PQEKIRRITSSTPNN**	CACAGAATGTGTTTAATTTTATTTTTAAATATATATATATATA<br>TATATATATATATATATATATATAAAGTCGACATATTCTCGTA<br>GAAAGTCTCCAAGTTCAGTGTTCTTTTTTAGTTACATGACACA<br>GAACTGGCAGCTTGTAGTCCAAGGAAAAAAAAAAAGGGGC<br>ATGAAAAATTCGGCAGAAGTGATGCCGGTGAATTATTGACTG<br>CCGTGGATCGCTTCATTTAAACTCAATGTTGTAAGTGTTTGCG<br>AGGAAGAAGTTGTCACTGAAGGTCGATTTGTTGTAGACTGAC<br>AAAAGAATTTTAATCACATTTTCTCGAAGAAAACAAAGGAC<br>GATTTTTTAAATCTTTAGGGCTTAACCTATACAATTAAGCTTC<br>ACTTTTCATTATAATTTATGCCCGTGTTTTATCCTATAGATGA<br>AGTGATATGACAAGAGATTAAGAAGCACATTTCGAGACACT<br>ATAAAAATGTCGTCCGTGTCCTAAATCCGCTTTCCGTTCTACT<br>GCCCCTACGCTTTACACACCGAACGCGCACATCATTTAAGAA<br>CTTTTCTATTCAAACACACAACAAATTCTAACACAACACGTA<br>GAACTTCCCTATTTCTGTTTTCTGAGAAGAAACGAAAAAAGT<br>TTTTCCATCCCAAAACGGGACACAGTCTGTCGCAGCATACGT<br>CGAGATGATTACGAACCACAGGTGGAGCTTTGGGGAACATC<br>CTTTCTGACGAGAGGAGAGGGGCTGAATGACCAAAATCTTG<br>CGTGGAGTCTCGCTGCTCGCCTTTAAATTATGTACAAATGTCA<br>GTTAAGTTTCCACATCGTTCACAACTACCTGTTTCGCCGAAAC<br>GATGATATGTGGAGCTCCCTCAGGTCCGACATCAGTCCGTTC<br>TTATTTTCTGCCTTTTGCGTTCGGTCCTCAGTAGAGTGGGCAG<br>GGCGGCAGGGTGCAGTACTTCCTCTCGATCTTGGGTCCGCAG<br>GCTGCCCCGTTGTTCTTGGGGGGCACCTTGACCGTCTTCGTCC<br>TCTGCTGGGTTCCCCCCTGACCGCAGGTCTTGGAGCACGGCG<br>TCCAGTCAGACCACGGGTTCAGTTGGCAGTCCACGGCTTCTT<br>TGTCCCCCAGTCTGTCAGGGCACGGCGGGTTGTCCTTGCATTT<br>CCTGCGTTGCACCAGCTTGGCAGGGCACGGCTTCCCTCCGTT

						**PLSPFYDSSGAPMK**	CTCCGGGTTGAGCTTGATCATTCGCCGCCTCTCCCGGCGTCCG
						**PVARLTVTRQRVY**	TTCCCGCAGCTCGTCGTGCACGACGTCCAGTCTGACCACGGA
						**DKSCDDYEPVTPTH**	GTGACCACGCAGTCGATGATCGGTGGTTGGTCCTTGGTCGCC
						**SYGTNDEDDERME**	ATGGGCAGGGCGGGCGAAGTGGAGAGTGGCGTCAGGGCACT
						**CRVSDWTDWSDCS**	GAGCGGAGCTCTGAGCATGGTTTCGCACATTCTGTTGCAGTC
						**VTCGQGHRMRSRQ**	CGCGTACCGCTCAAAGTTGTTCCTGTTGCCCCGGCATCCGCC
						**YINKPKADMAGCT**	GTAAATGAACTGCAGGCACATCGTCCGCCCGACGTCGTAGT
						**VPLVDKEVCSQHC**	ACCAGCGGGGGAAGTAGCCACGGCAGGGGCCCACCTCCTTG
						**VGDVSCRTTEWGE**	GGTTGCATGCAGTTCTTTTTTGCCTCGGTGAGGTTGACTGTGC
						**WSECSVTCGKGLR**	AGTCCGGCTTATCCGCCGTGCAGGGGGCCTTCTGCATCAGCT
						**TRNRKYLDNQARK**	CCACGTTACACATGCTGAGTGCGGCCGGGCTTAGGTAGAGTC
						**KCALELMEKETCTG**	GGGTTCGGACCTTCAGGCCTTTCCCGCATGTCACAGTGCAAG
						**MKMTCEEEDRVPD**	GCGACCACTCGGACCAGTGGGTGACGGCGCACTTTGGATCC
						**PKCAVTHWSEWSP**	GGCACCCTGTCTTCCTCTTCGCACGTCATCTTCATGCCGGTGC
						**CTVTCGKGLKVRT**	AGGTTTCCTTCTCCATGAGTTCGAGGGCACACTTCTTCCGCGC
						**RLYLSPAALSMCNV**	CTGGTTGTCCAGGTACTTCCGGTTTCGAGTCCGGAGTCCCTTG
						**ELMQKAPCTADKP**	CCGCAGGTCACGCTGCACTCGGACCACTCTCCCCATTCCGTC
						**DCTVNLTEAKKNC**	GTGCGGCAGCTCACGTCCCCGACGCAGTGCTGGGAGCAGAC
						**MQPKEVGPCRGYF**	CTCCTTGTCCACGAGCGGCACCGTGCACCCGGCCATGTCCGC
						**PRWYYDVGRTMCL**	CTTCGGTTTGTTGATGTACTGCCTCGAGCGCATCCGGTGGCCC
						**QFIYGGCRGNRNN**	TGGCCGCACGTCACGCTGCAATCGCTCCAGTCCGTCCAGTCG
						**FERYADCNRMCET**	CTGACGCGGCACTCCATCCTCTCGTCATCCTCGTCGTTGGTCC
						**MLRAPLSALTPLSTS**	CGTACGAATGAGTAGGCGTAACGGGTTCGTAATCGTCACAG
						**PALPMATKDQPPII**	GACTTGTCGTAGACACGTTGGCGGGTCACAGTGAGACGGGC
						**DCVVTPWSDWTSC**	GACGGGTTTCATGGGTGCTCCAGATGAGTCGTAGAACGGAG
						**TTSCGNGRRERRR**	ACAGGGGGTTGTTAGGCGTGGACGACGTGATGCGCCGGATC
						**MIKLNPENGGKPCP**	TTCTCTTGAGGCACCGTCGGCATCTTCGAAGTGATGTAAGTG
						**AKLVQRRKCKDNP**	GCTCCACTATCCGTTCCCGCATCCCAGGGGTACAGGTTCATG

						**PCPDRLGDKEAVD CQLNPWSDWTPCS KTCGQGGTQQRTK TVKVPPKNNGAAC GPKIERKYCTLPPCP LY***	ACTTTCTCGGCCACCCAGGAGCAGTTCCGCAAGCAGAGCTCG AGGGCACTCACTCCCACTATCCAGTCTGGGCTCGGACCCAGC ATGGTCAGGAGAGATACCAGGTGATGCAGGCGGTCCACTCG GAACACGGCGAACGTCCTGCCTTGGGCGTTAGGGTACCATA AGCCTCGAGCCTTTATGATTGTGCGGATCTTGTTGGACTCGGT CTTGAGTTCGGACTCGAGCCTTTTGGTGATCCCTCTCTCGGCC ACTTCTTTCACGCCCTCTGACGCGATGCCTCCGTACTCCCACA TCCGGAAGTCGCCCGTGTGGGAGGCGCCGATGATGTCGGAG AAATGCGTCAGCCACTCGTTTTTGGGAAATTCCTTGGGGTGC GTGTGCCTGGACCAGAGCCCTTCGAAGGTCAGTTCGTACTTG GCCTCATCGCATGCGCAGCACTCCTCGATGATCTCCGGCTGC TCATCCTGGTTCTCTTGCTCTTCCTCGCAGAGCTCCCGTATGA GACCTCCTTCGTCCATGTACCAAAGTTCTCTATTCTCCTGAAC GGTGGCCTTGAAGACGACGCAATTGGTCCCTGCCTGCGGCGC CTTCCACATCACCTGTATGTCCTGCTTGGGGATGAGCGAGGC GTGCACGATGGCATTCGGACACTCCTCCGAGTACTTGCACAT GGCGTCGCCGAACAGCTGGAAGGATCCGACGTCCGGCCTGG CCGAGATGTCGTTCCTCACCTCTTTGGGCTCCACCACCAGCA TGAAGCCGATGAACTTCTGTACGGCGAACTGGGAGCTGTAG CCTTGGAGGGTCACTGTGTACAGCTCTCCTGGTTGGTACTTCT CCGGGTTTCCGGATATCTTGATGTAGAAACCGTTGTCTCCCG GCACCTTCGGAATCGTCACACCGGGAGGTGCCCTACGGCAA CGCCCAGGGAAGCTCTGGGGGATCATGCGGGGCTCGGAGTC ACCCGCCGAGGGGTCGGCGCCCCTGCACTGCCCCATCAGTG CGACCCACACCGTCACGACCGCGAACACAGACACCATTTCG AGATCACCTCAACCGTTCCGAGGTCTCGAAAATTCGAACGAT ACTCGATCCACGTGTTGGAACACAAGAGAGGGAAACACTCT

							TCCTTTATCGGAACGACGCGGTAGATCAGTGATGCCCGCGGC GTCGCCTTCGAAAG

PhdPInSeP13	P0DMW6	6,03E-16	55,172	PF00014	93114.34<br><br>**90978.71**	MVSVFAVVTVWVA<br>LMGQCRG**ADPSAG**<br>**DSEPRMIPQSFPGR**<br>**CRRAPPGVTIPKVP**<br>**GDNGFYIKISGNPE**<br>**KYQPGELYTVTLQG**<br>**YSSQFAVQKFIGFM**<br>**LVVEPKEVRNDISA**<br>**RPDVGSFQLFGDA**<br>**MCKYSEECPNAIVH**<br>**ASLIPKQDIQVMW**<br>**KAPQAGTNCVVFK**<br>**ATVQENRELWYMD**<br>**EGGLIRELCEEEQEN**<br>**QDEQPEIIEECCACD**<br>**EAKYELTFEGLWSR**<br>**HTHPKEFPKNEWLT**<br>**HFSDIIGASHTGDF**<br>**RMWEYGGIASEGV**<br>**KEVAERGITKRLESE**<br>**LKTESNKIRTIIKAR**<br>**GLWYPNAQGRTFA**<br>**VFRVDRLHHLVSLL**<br>**TMLGPSPDWIVGV**<br>**SALELCLRNCSWVA**<br>**EKVMNLYPWDAGT**<br>**DSGATYITSKMPTV**<br>**PQEKIRRITSSTPNN**	CACAGAATGTGTTTAATTTTATTTTTAAATATATATATATATA<br>TATATATATATATATATATATATATAAAGTCGACATATTCTCGTA<br>GAAAGTCTCCAAGTTCAGTGTTCTTTTTTAGTTACATGACACA<br>GAACTGGCAGCTTGTAGTCCAAGGAAAAAAAAAAAGGGGC<br>ATGAAAAATTCGGCAGAAGTGATGCCGGTGAATTATTGACTG<br>CCGTGGATCGCTTCATTTAAACTCAATGTTGTAAGTGTTTGCG<br>AGGAAGAAGTTGTCACTGAAGGTCGATTTGTTGTAGACTGAC<br>AAAAGAATTTTAATCACATTTTCTCGAAGAAAACAAAGGAC<br>GATTTTTTAAATCTTTAGGGCTTAACCTATACAATTAAGCTTC<br>ACTTTTCATTATAATTTATGCCCGTGTTTTATCCTATAGATGA<br>AGTGATATGACAAGAGATTAAGAAGCACATTTCGAGACACT<br>ATAAAAATGTCGTCCGTGTCCTAAATCCGCTTTCCGTTCTACT<br>GCCCCTACGCTTTACACACCGAACGCGCACATCATTTAAGAA<br>CTTTTCTATTCAAACACACAACAAATTCTAACACAACACGTA<br>GAACTTCCCTATTTCTGTTTTCTGAGAAGAAACGAAAAAAGT<br>TTTTCCATCCCAAAACGGGACACAGTCTGTCGCAGCATACGT<br>CGAGATGATTACGAACCACAGGTGGAGCTTTGGGGAACATC<br>CTTTCTGACGAGAGGAGAGGGGCTGAATGACCAAAATCTTG<br>CGTGGAGTCTCGCTGCTCGCCTTTAAATTATGTACAAATGTCA<br>GTTAAGTTTCCACATCGTTCACAACTACCTGTTTCGCCGAAAC<br>GATGATATGTGGAGCTCCCTCAGGTCCGACATCAGTCCGTTC<br>TTATTTTCTGCCTTTTGCGTTCGGTCCTCAGTAGAGTGGGCAG<br>GGCGGCAGGGTGCAGTACTTCCTCTCGATCTTGGGTCCGCAG<br>GCTGCCCCGTTGTTCTTGGGGGGCACCTTGACCGTCTTCGTCC<br>TCTGCTGGGTTCCCCCCTGACCGCAGGTCTTGGAGCACGGCG<br>TCCAGTCAGACCACGGGTTCAGTTGGCAGTCCACGGCTTCTT<br>TGTCCCCCAGTCTGTCAGGGCACGGCGGGTTGTCCTTGCATTT<br>CCTGCGTTGCACCAGCTTGGCAGGGCACGGCTTCCCTCCGTT

						**PLSPFYDSSGAPMK**	CTCCGGGTTGAGCTTGATCATTCGCCGCCTCTCCCGGCGTCCG
						**PVARLTVTRQRVY**	TTCCCGCAGCTCGTCGTGCACGACGTCCAGTCTGACCACGGA
						**DKSCDDYEPVTPTH**	GTGACCACGCAGTCGATGATCGGTGGTTGGTCCTTGGTCGCC
						**SYGTNDEDDERME**	ATGGGCAGGGCGGGCGAAGTGGAGAGTGGCGTCAGGGCACT
						**CRVSDWTDWSDCS**	GAGCGGAGCTCTGAGCATGGTTTCGCACATTCTGTTGCAGTC
						**VTCGQGHRMRSRQ**	CGCGTACCGCTCAAAGTTGTTCCTGTTGCCCCGGCATCCGCC
						**YINKPKADMAGCT**	GTAAATGAACTGCAGGCACATCGTCCGCCCGACGTCGTAGT
						**VPLVDKEVCSQHC**	ACCAGCGGGGGAAGTAGCCACGGCAGGGGCCCACCTCCTTG
						**VGDVSCRTTEWGE**	GGTTGCATGCAGTTCTTTTTTGCCTCGGTGAGGTTGACTGTGC
						**WSECSVTCGKGLR**	AGTCCGGCTTATCCGCCGTGCAGGGGGCCTTCTGCATCAGCT
						**TRNRKYLDNQARK**	CCACGTTACACATGCTGAGTGCGGCCGGGCTTAGGTAGAGTC
						**KCALELMEKETCTG**	GGGTTCGGACCTTCAGGCCTTTCCCGCATGTCACAGTGCAAG
						**MKMTCEEEDRVPD**	GCGACCACTCGGACCAGTGGGTGACGGCGCACTTTGGATCC
						**PKCAVTHWSEWSP**	GGCACCCTGTCTTCCTCTTCGCACGTCATCTTCATGCCGGTGC
						**CTVTCGKGLKVRT**	AGGTTTCCTTCTCCATGAGTTCGAGGGCACACTTCTTCCGCGC
						**RLYLSPAALSMCNV**	CTGGTTGTCCAGGTACTTCCGGTTTCGAGTCCGGAGTCCCTTG
						**ELMQKAPCTADKP**	CCGCAGGTCACGCTGCACTCGGACCACTCTCCCCATTCCGTC
						**DCTVNLTEAKKNC**	GTGCGGCAGCTCACGTCCCCGACGCAGTGCTGGGAGCAGAC
						**MQPKEVGPCRGYF**	CTCCTTGTCCACGAGCGGCACCGTGCACCCGGCCATGTCCGC
						**PRWYYDVGRTMCL**	CTTCGGTTTGTTGATGTACTGCCTCGAGCGCATCCGGTGGCCC
						**QFIYGGCRGNRNN**	TGGCCGCACGTCACGCTGCAATCGCTCCAGTCCGTCCAGTCG
						**FERYADCNRMCET**	CTGACGCGGCACTCCATCCTCTCGTCATCCTCGTCGTTGGTCC
						**MLRAPLSALTPLSTS**	CGTACGAATGAGTAGGCGTAACGGGTTCGTAATCGTCACAG
						**PALPMATKDQPPII**	GACTTGTCGTAGACACGTTGGCGGGTCACAGTGAGACGGGC
						**DCVVTPWSDWTSC**	GACGGGTTTCATGGGTGCTCCAGATGAGTCGTAGAACGGAG
						**TTSCGNGRRERRR**	ACAGGGGGTTGTTAGGCGTGGACGACGTGATGCGCCGGATC
						**MIKLNPENGGKPCP**	TTCTCTTGAGGCACCGTCGGCATCTTCGAAGTGATGTAAGTG
						**AKLVQRRKCKDNP**	GCTCCACTATCCGTTCCCGCATCCCAGGGGTACAGGTTCATG

						**PCPDRLGDKEAVD<br>CQLNPWSDWTPCS<br>KTCGQGGTQQRTK<br>TVKVPPKNNGAAC<br>GPKIERKYCTLPPCP<br>LY***	ACTTTCTCGGCCACCCAGGAGCAGTTCCGCAAGCAGAGCTCG<br>AGGGCACTCACTCCCACTATCCAGTCTGGGCTCGGACCCAGC<br>ATGGTCAGGAGAGATACCAGGTGATGCAGGCGGTCCACTCG<br>GAACACGGCGAACGTCCTGCCTTGGGCGTTAGGGTACCATA<br>AGCCTCGAGCCTTTATGATTGTGCGGATCTTGTTGGACTCGGT<br>CTTGAGTTCGGACTCGAGCCTTTTGGTGATCCCTCTCTCGGCC<br>ACTTCTTTCACGCCCTCTGACGCGATGCCTCCGTACTCCCACA<br>TCCGGAAGTCGCCCGTGTGGGAGGCGCCGATGATGTCGGAG<br>AAATGCGTCAGCCACTCGTTTTTGGGAAATTCCTTGGGGTGC<br>GTGTGCCTGGACCAGAGCCCTTCGAAGGTCAGTTCGTACTTG<br>GCCTCATCGCATGCGCAGCACTCCTCGATGATCTCCGGCTGC<br>TCATCCTGGTTCTCTTGCTCTTCCTCGCAGAGCTCCCGTATGA<br>GACCTCCTTCGTCCATGTACCAAAGTTCTCTATTCTCCTGAAC<br>GGTGGCCTTGAAGACGACGCAATTGGTCCCTGCCTGCGGCGC<br>CTTCCACATCACCTGTATGTCCTGCTTGGGGATGAGCGAGGC<br>GTGCACGATGGCATTCGGACACTCCTCCGAGTACTTGCACAT<br>GGCGTCGCCGAACAGCTGGAAGGATCCGACGTCCGGCCTGG<br>CCGAGATGTCGTTCCTCACCTCTTTGGGCTCCACCACCAGCA<br>TGAAGCCGATGAACTTCTGTACGGCGAACTGGGAGCTGTAG<br>CCTTGGAGGGTCACTGTGTACAGCTCTCCTGGTTGGTACTTCT<br>CCGGGTTTCCGGATATCTTGATGTAGAAACCGTTGTCTCCCG<br>GCACCTTCGGAATCGTCACACCGGGAGGTGCCCTACGGCAA<br>CGCCCAGGGAAGCTCTGGGGGATCATGCGGGGCTCGGAGTC<br>ACCCGCCGAGGGGTCGGCGCCCCTGCACTGCCCCATCAGTG<br>CGACCCACACCGTCACGACCGCGAACACAGACACCATTTCG<br>AGATCACCTCAACCGTTCCGAGGTCTCGAAAATTCGAACGAT<br>ACTCGATCCACGTGTTGGAACACAAGAGAGGGAAACACTCT

							TCCTTTATCGGAACGACGCGGTAGATCAGTGATGCCCGCGGC GTCGCCTTCGAAAG

PhdPInSeP14	P0DMW6	4,03E-16	55,172	PF00014	89952.47<br><br>**87816.84**	MVSVFAVVTVWVA<br>LMGQCRG**ADPSAG**<br>**DSEPRMIPQSFPGR**<br>**CRRAPPGVTIPKVP**<br>**GDNGFYIKISGNPE**<br>**KYQPGELYTVTLQG**<br>**YSSQFAVQKFIGFM**<br>**LVVEPKEVRNDISA**<br>**RPDVGSFQLFGDA**<br>**MCKYSEECPNAIVH**<br>**ASLIPKQDIQVMW**<br>**KAPQAGTNCVVFK**<br>**ATVQENRELWYMD**<br>**EGGLIRELCEEEQEN**<br>**QDEQPEIIEECCACD**<br>**EAKYELTFEGLWSR**<br>**HTHPKEFPKNEWLT**<br>**HFSDIIGASHTGDF**<br>**RMWEYGGIASEGV**<br>**KEVAERGITKRLESE**<br>**LKTESNKIRTIIKAR**<br>**GLWYPNAQGRTFA**<br>**VFRVDRLHHLVSLL**<br>**TMLGPSPDWIVGV**<br>**SALELCLRNCSWVA**<br>**EKVMNLYPWDAGT**<br>**DSGATYITSKMPTV**<br>**PQEKIRRITSSTPNN**	CACAGAATGTGTTTAATTTTATTTTTAAATATATATATATATA<br>TATATATATATATATATATATATAAAGTCGACATATTCTCGTA<br>GAAAGTCTCCAAGTTCAGTGTTCTTTTTTAGTTACATGACACA<br>GAACTGGCAGCTTGTAGTCCAAGGAAAAAAAAAAAGGGGC<br>ATGAAAAATTCGGCAGAAGTGATGCCGGTGAATTATTGACTG<br>CCGTGGATCGCTTCATTTAAACTCAATGTTGTAAGTGTTTGCG<br>AGGAAGAAGTTGTCACTGAAGGTCGATTTGTTGTAGACTGAC<br>AAAAGAATTTTAATCACATTTTCTCGAAGAAAACAAAGGAC<br>GATTTTTTAAATCTTTAGGGCTTAACCTATACAATTAAGCTTC<br>ACTTTTCATTATAATTTATGCCCGTGTTTTATCCTATAGATGA<br>AGTGATATGACAAGAGATTAAGAAGCACATTTCGAGACACT<br>ATAAAAATGTCGTCCGTGTCCTAAATCCGCTTTCCGTTCTACT<br>GCCCCTACGCTTTACACACCGAACGCGCACATCATTTAAGAA<br>CTTTTCTATTCAAACACACAACAAATTCTAACACAACACGTA<br>GAACTTCCCTATTTCTGTTTTCTGAGAAGAAACGAAAAAAGT<br>TTTTCCATCCCAAAACGGGACACAGTCTGTCGCAGCATACGT<br>CGAGATGATTACGAACCACAGGTGGAGCTTTGGGGAACATC<br>CTTTCTGACGAGAGGAGAGGGGCTGAATGACCAAAATCTTG<br>CGTGGAGTCTCGCTGCTCGCCTTTAAATTATGTACAAATGTCA<br>GTTAAGTTTCCACATCGTTCACAACTACCTGTTTCGCCGAAAC<br>GATGATATGTGGAGCTCCCTCAGGTCCGACATCAGTCCGTTC<br>TTATTTTCTGCCTTCTTTGTCCCCCAGTCTGTCAGGGCACGGC<br>GGGTTGTCCTTGCATTTCCTGCGTTGCACCAGCTTGGCAGGGC<br>ACGGCTTCCCTCCGTTCTCCGGGTTGAGCTTGATCATTCGCCG<br>CCTCTCCCGGCGTCCGTTCCCGCAGCTCGTCGTGCACGACGT<br>CCAGTCTGACCACGGAGTGACCACGCAGTCGATGATCGGTG<br>GTTGGTCCTTGGTCGCCATGGGCAGGGCGGGCGAAGTGGAG<br>AGTGGCGTCAGGGCACTGAGCGGAGCTCTGAGCATGGTTTCG

						**PLSPFYDSSGAPMK**	CACATTCTGTTGCAGTCCGCGTACCGCTCAAAGTTGTTCCTGT
						**PVARLTVTRQRVY**	TGCCCCGGCATCCGCCGTAAATGAACTGCAGGCACATCGTCC
						**DKSCDDYEPVTPTH**	GCCCGACGTCGTAGTACCAGCGGGGGAAGTAGCCACGGCAG
						**SYGTNDEDDERME**	GGGCCCACCTCCTTGGGTTGCATGCAGTTCTTTTTGCCTCGG
						**CRVSDWTDWSDCS**	TGAGGTTGACTGTGCAGTCCGGCTTATCCGCCGTGCAGGGGG
						**VTCGQGHRMRSRQ**	CCTTCTGCATCAGCTCCACGTTACACATGCTGAGTGCGGCCG
						**YINKPKADMAGCT**	GGCTTAGGTAGAGTCGGGTTCGGACCTTCAGGCCTTTCCCGC
						**VPLVDKEVCSQHC**	ATGTCACAGTGCAAGGCGACCACTCGGACCAGTGGGTGACG
						**VGDVSCRTTEWGE**	GCGCACTTTGGATCCGGTGATTCGTCCGCTGGCGGGGGGTGG
						**WSECSVTCGKGLR**	TCTCCGGAAGACTTGTTCTTCTCCTTCTGTTTGTTCCTCCGCCT
						**TRNRKYLDNQARK**	GCCGTTGGTCACCCTGTCTTCCTCTTCGCACGTCATCTTCATG
						**KCALELMEKETCTG**	CCGGTGCAGGTTTCCTTCTCCATGAGTTCGAGGGCACACTTCT
						**MKMTCEEEDRVTN**	TCCGCGCCTGGTTGTCCAGGTACTTCCGGTTTCGAGTCCGGA
						**GRRRNKQKEKNKS**	GTCCCTTGCCGCAGGTCACGCTGCACTCGGACCACTCTCCCC
						**SGDHPPPADESPDP**	ATTCCGTCGTGCGGCAGCTCACGTCCCCGACGCAGTGCTGGG
						**KCAVTHWSEWSPC**	AGCAGACCTCCTTGTCCACGAGCGGCACCGTGCACCCGGCC
						**TVTCGKGLKVRTR**	ATGTCCGCCTTCGGTTTGTTGATGTACTGCCTCGAGCGCATCC
						**LYLSPAALSMCNVE**	GGTGGCCCTGGCCGCACGTCACGCTGCAATCGCTCCAGTCCG
						**LMQKAPCTADKPD**	TCCAGTCGCTGACGCGGCACTCCATCCTCTCGTCATCCTCGTC
						**CTVNLTEAKKNCM**	GTTGGTCCCGTACGAATGAGTAGGCGTAACGGGTTCGTAATC
						**QPKEVGPCRGYFPR**	GTCACAGGACTTGTCGTAGACACGTTGGCGGGTCACAGTGA
						**WYYDVGRTMCLQF**	GACGGGCGACGGGTTTCATGGGTGCTCCAGATGAGTCGTAG
						**IYGGCRGNRNNFER**	AACGGAGACAGGGGGTTGTTAGGCGTGGACGACGTGATGCG
						**YADCNRMCETMLR**	CCGGATCTTCTCTTGAGGCACCGTCGGCATCTTCGAAGTGAT
						**APLSALTPLSTSPAL**	GTAAGTGGCTCCACTATCCGTTCCCGCATCCCAGGGGTACAG
						**PMATKDQPPIIDCV**	GTTCATGACTTTCTCGGCCACCCAGGAGCAGTTCCGCAAGCA
						**VTPWSDWTSCTTS**	GAGCTCGAGGGCACTCACTCCCACTATCCAGTCTGGGCTCGG
						**CGNGRRERRRMIK**	ACCCAGCATGGTCAGGAGAGATACCAGGTGATGCAGGCGGT

						**<u>LNPENGGKPCPAKLVQRRKCKDNPPCPDRLGDKEGRK*</u>**	CCACTCGGAACACGGCGAACGTCCTGCCTTGGGCGTTAGGGTACCATAAGCCTCGAGCCTTTATGATTGTGCGGATCTTGTTGGACTCGGTCTTGAGTTCGGACTCGAGCCTTTTGGTGATCCCTCTCTCGGCCACTTCTTTCACGCCCTCTGACGCGATGCCTCCGTACTCCCACATCCGGAAGTCGCCCGTGTGGGAGGCGCCGATGATGTCGGAGAAATGCGTCAGCCACTCGTTTTTGGGAAATTCCTTGGGGTGCGTGTGCCTGGACCAGAGCCCTTCGAAGGTCAGTTCGTACTTGGCCTCATCGCATGCGCAGCACTCCTCGATGATCTCCGGCTGCTCATCCTGGTTCTCTTGCTCTTCCTCGCAGAGCTCCCGTATGAGACCTCCTTCGTCCATGTACCAAAGTTCTCTATTCTCCTGAACGGTGGCCTTGAAGACGACGCAATTGGTCCCTGCCTGCGGCGCCTTCCACATCACCTGTATGTCCTGCTTGGGGATGAGCGAGGCGTGCACGATGGCATTCGGACACTCCTCCGAGTACTTGCACATGGCGTCGCCGAACAGCTGGAAGGATCCGACGTCCGGCCTGGCCGAGATGTCGTTCCTCACCTCTTTGGGCTCCACCACCAGCATGAAGCCGATGAACTTCTGTACGGCGAACTGGGAGCTGTAGCCTTGGAGGGTCACTGTGTACAGCTCTCCTGGTTGGTACTTCTCCGGGTTTCCGGATATCTTGATGTAGAAACCGTTGTCTCCCGGCACCTTCGGAATCGTCACACCGGGAGGTGCCCTACGGCAACGCCCAGGGAAGCTCTGGGGGATCATGCGGGGCTCGGAGTCACCCGCCGAGGGGTCGGCGCCCCTGCACTGCCCCATCAGTGCGACCCACACCGTCACGACCGCGAACACAGACACCATTTCGAGATCACCTCAACCGTTCCGAGGTCTCGAAAATTCGAACGATACTCGATCCACGTGTTGGAACACAAGAGAGGGAAACACTCTTCCTTTATCGGAACGACGCGGTAGATCAGTGATGCCCGCGGCGTCGCCTTCGAAAG

PhdPInSeP15	P0DMW6	5,43E-16	55,172	PF00014	96030.47<br>**93894.83**	MVSVFAVVTVWVA<br>LMGQCRG**ADPSAG**<br>**DSEPRMIPQSFPGR**<br>**CRRAPPGVTIPKVP**<br>**GDNGFYIKISGNPE**<br>**KYQPGELYTVTLQG**<br>**YSSQFAVQKFIGFM**<br>**LVVEPKEVRNDISA**<br>**RPDVGSFQLFGDA**<br>**MCKYSEECPNAIVH**<br>**ASLIPKQDIQVMW**<br>**KAPQAGTNCVVFK**<br>**ATVQENRELWYMD**<br>**EGGLIRELCEEEQEN**<br>**QDEQPEIIEECCACD**<br>**EAKYELTFEGLWSR**<br>**HTHPKEFPKNEWLT**<br>**HFSDIIGASHTGDF**<br>**RMWEYGGIASEGV**<br>**KEVAERGITKRLESE**<br>**LKTESNKIRTIIKAR**<br>**GLWYPNAQGRTFA**<br>**VFRVDRLHHLVSLL**<br>**TMLGPSPDWIVGV**<br>**SALELCLRNCSWVA**<br>**EKVMNLYPWDAGT**<br>**DSGATYITSKMPTV**<br>**PQEKIRRITSSTPNN**	CACAGAATGTGTTTAATTTTATTTTTAAATATATATATATATA<br>TATATATATATATATATATATATAAAGTCGACATATTCTCGTA<br>GAAAGTCTCCAAGTTCAGTGTTCTTTTTTAGTTACATGACACA<br>GAACTGGCAGCTTGTAGTCCAAGGAAAAAAAAAAAGGGGC<br>ATGAAAAATTCGGCAGAAGTGATGCCGGTGAATTATTGACTG<br>CCGTGGATCGCTTCATTTAAACTCAATGTTGTAAGTGTTTGCG<br>AGGAAGAAGTTGTCACTGAAGGTCGATTTGTTGTAGACTGAC<br>AAAAGAATTTTAATCACATTTTCTCGAAGAAAACAAAGGAC<br>GATTTTTTAAATCTTTAGGGCTTAACCTATACAATTAAGCTTC<br>ACTTTTCATTATAATTTATGCCCGTGTTTTATCCTATAGATGA<br>AGTGATATGACAAGAGATTAAGAAGCACATTTCGAGACACT<br>ATAAAAATGTCGTCCGTGTCCTAAATCCGCTTTCCGTTCTACT<br>GCCCCTACGCTTTACACACCGAACGCGCACATCATTTAAGAA<br>CTTTTCTATTCAAACACACAACAAATTCTAACACAACACGTA<br>GAACTTCCCTATTTCTGTTTTCTGAGAAGAAACGAAAAAAGT<br>TTTTCCATCCCAAAACGGGACACAGTCTGTCGCAGCATACGT<br>CGAGATGATTACGAACCACAGGTGGAGCTTTGGGGAACATC<br>CTTTCTGACGAGAGGAGAGGGGCTGAATGACCAAAATCTTG<br>CGTGGAGTCTCGCTGCTCGCCTTTAAATTATGTACAAATGTCA<br>GTTAAGTTTCCACATCGTTCACAACTACCTGTTTCGCCGAAAC<br>GATGATATGTGGAGCTCCCTCAGGTCCGACATCAGTCCGTTC<br>TTATTTTCTGCCTTTTGCGTTCGGTCCTCAGTAGAGTGGGCAG<br>GGCGGCAGGGTGCAGTACTTCCTCTCGATCTTGGGTCCGCAG<br>GCTGCCCCGTTGTTCTTGGGGGGCACCTTGACCGTCTTCGTCC<br>TCTGCTGGGTTCCCCCCTGACCGCAGGTCTTGGAGCACGGCG<br>TCCAGTCAGACCACGGGTTCAGTTGGCAGTCCACGGCTTCTT<br>TGTCCCCCAGTCTGTCAGGGCACGGCGGGTTGTCCTTGCATTT<br>CCTGCGTTGCACCAGCTTGGCAGGGCACGGCTTCCCTCCGTT

						**PLSPFYDSSGAPMK**	CTCCGGGTTGAGCTTGATCATTCGCCGCCTCTCCCGGCGTCCG
						**PVARLTVTRQRVY**	TTCCCGCAGCTCGTCGTGCACGACGTCCAGTCTGACCACGGA
						**DKSCDDYEPVTPTH**	GTGACCACGCAGTCGATGATCGGTGGTTGGTCCTTGGTCGCC
						**SYGTNDEDDERME**	ATGGGCAGGGCGGGCGAAGTGGAGAGTGGCGTCAGGGCACT
						**CRVSDWTDWSDCS**	GAGCGGAGCTCTGAGCATGGTTTCGCACATTCTGTTGCAGTC
						**VTCGQGHRMRSRQ**	CGCGTACCGCTCAAAGTTGTTCCTGTTGCCCCGGCATCCGCC
						**YINKPKADMAGCT**	GTAAATGAACTGCAGGCACATCGTCCGCCCGACGTCGTAGT
						**VPLVDKEVCSQHC**	ACCAGCGGGGGAAGTAGCCACGGCAGGGGCCCACCTCCTTG
						**VGDVSCRTTEWGE**	GGTTGCATGCAGTTCTTTTTTGCCTCGGTGAGGTTGACTGTGC
						**WSECSVTCGKGLR**	AGTCCGGCTTATCCGCCGTGCAGGGGGCCTTCTGCATCAGCT
						**TRNRKYLDNQARK**	CCACGTTACACATGCTGAGTGCGGCCGGGCTTAGGTAGAGTC
						**KCALELMEKETCTG**	GGGTTCGGACCTTCAGGCCTTTCCCGCATGTCACAGTGCAAG
						**MKMTCEEEDRVTN**	GCGACCACTCGGACCAGTGGGTGACGGCGCACTTTGGATCC
						**GRRRNKQKEKNKS**	GGTGATTCGTCCGCTGGCGGGGGGTGGTCTCCGGAAGACTTG
						**SGDHPPPADESPDP**	TTCTTCTCCTTCTGTTTGTTCCTCCGCCTGCCGTTGGTCACCCT
						**KCAVTHWSEWSPC**	GTCTTCCTCTTCGCACGTCATCTTCATGCCGGTGCAGGTTTCC
						**TVTCGKGLKVRTR**	TTCTCCATGAGTTCGAGGGCACACTTCTTCCGCGCCTGGTTGT
						**LYLSPAALSMCNVE**	CCAGGTACTTCCGGTTTCGAGTCCGGAGTCCCTTGCCGCAGG
						**LMQKAPCTADKPD**	TCACGCTGCACTCGGACCACTCTCCCCATTCCGTCGTGCGGC
						**CTVNLTEAKKNCM**	AGCTCACGTCCCCGACGCAGTGCTGGGAGCAGACCTCCTTGT
						**QPKEVGPCRGYFPR**	CCACGAGCGGCACCGTGCACCCGGCCATGTCCGCCTTCGGTT
						**WYYDVGRTMCLQF**	TGTTGATGTACTGCCTCGAGCGCATCCGGTGGCCCTGGCCGC
						**IYGGCRGNRNNFER**	ACGTCACGCTGCAATCGCTCCAGTCCGTCCAGTCGCTGACGC
						**YADCNRMCETMLR**	GGCACTCCATCCTCTCGTCATCCTCGTCGTTGGTCCCGTACGA
						**APLSALTPLSTSPAL**	ATGAGTAGGCGTAACGGGTTCGTAATCGTCACAGGACTTGTC
						**PMATKDQPPIIDCV**	GTAGACACGTTGGCGGGTCACAGTGAGACGGGCGACGGGTT
						**VTPWSDWTSCTTS**	TCATGGGTGCTCCAGATGAGTCGTAGAACGGAGACAGGGGG
						**CGNGRRERRRMIK**	TTGTTAGGCGTGGACGACGTGATGCGCCGGATCTTCTCTTGA

						**<u>LNPENGGKPCPAKLVQRRKCKDNPPCPDRLGDKEAVDCQLNPWSDWTPCSKTCGQGGTQQRTKTVKVPPKNNGAACGPKIERKYCTLPPCPLY*</u>**	GGCACCGTCGGCATCTTCGAAGTGATGTAAGTGGCTCCACTATCCGTTCCCGCATCCCAGGGGTACAGGTTCATGACTTTCTCGGCCACCCAGGAGCAGTTCCGCAAGCAGAGCTCGAGGGCACTCACTCCCACTATCCAGTCTGGGCTCGGACCCAGCATGGTCAGGAGAGATACCAGGTGATGCAGGCGGTCCACTCGGAACACGGCGAACGTCCTGCCTTGGGCGTTAGGGTACCATAAGCCTCGAGCCTTTATGATTGTGCGGATCTTGTTGGACTCGGTCTTGAGTTCGGACTCGAGCCTTTTGGTGATCCCTCTCTCGGCCACTTCTTTCACGCCCTCTGACGCGATGCCTCCGTACTCCCACATCCGGAAGTCGCCCGTGTGGGAGGCGCCGATGATGTCGGAGAAATGCGTCAGCCACTCGTTTTTGGGAAATTCCTTGGGGTGCGTGTGCCTGGACCAGAGCCCTTCGAAGGTCAGTTCGTACTTGGCCTCATCGCATGCGCAGCACTCCTCGATGATCTCCGGCTGCTCATCCTGGTTCTCTTGCTCTTCCTCGCAGAGCTCCCGTATGAGACCTCCTTCGTCCATGTACCAAAGTTCTCTATTCTCCTGAACGGTGGCCTTGAAGACGACGCAATTGGTCCCTGCCTGCGGCGCCTTCCACATCACCTGTATGTCCTGCTTGGGGATGAGCGAGGCGTGCACGATGGCATTCGGACACTCCTCCGAGTACTTGCACATGGCGTCGCCGAACAGCTGGAAGGATCCGACGTCCGGCCTGGCCGAGATGTCGTTCCTCACCTCTTTGGGCTCCACCACCAGCATGAAGCCGATGAACTTCTGTACGGCGAACTGGGAGCTGTAGCCTTGGAGGGTCACTGTGTACAGCTCTCCTGGTTGGTACTTCTCCGGGTTTCCGGATATCTTGATGTAGAAACCGTTGTCTCCCGGCACCTTCGGAATCGTCACACCGGGAGGTGCCCTACGGCAACGCCCAGGGAAGCTCTGGGGGATCATGCGGGGCTCGGAGTCACCCGCCGAGGGGTCGGCGCCCCTGCACTGCCCCATCAGTGCGACCCACACCGTCACGACCGCGAACACAGACACCATTTCGAGATCACCTCAACCGTTCCGAGGTCTCGAAAATTCGAACGATACTCGATCCAC

							GTGTTGGAACACAAGAGAGGGAAACACTCTTCCTTTATCGGA ACGACGCGGTAGATCAGTGATGCCCGCGGCGTCGCCTTCGA AAG

PhdPInSeP16	P0DMW6	3,99E-16	55,172	PF00014	89140.62<br><br>**87004.99**	MVSVFAVVTVWVA<br>LMGQCRG**ADPSAG**<br>**DSEPRMIPQSFPGR**<br>**CRRAPPGVTIPKVP**<br>**GDNGFYIKISGNPE**<br>**KYQPGELYTVTLQG**<br>**YSSQFAVQKFIGFM**<br>**LVVEPKEVRNDISA**<br>**RPDVGSFQLFGDA**<br>**MCKYSEECPNAIVH**<br>**ASLIPKQDIQVMW**<br>**KAPQAGTNCVVFK**<br>**ATVQENRELWYMD**<br>**EGGLIRELCEEEQEN**<br>**QDEQPEIIEECCACD**<br>**EAKYELTFEGLWSR**<br>**HTHPKEFPKNEWLT**<br>**HFSDIIGASHTGDF**<br>**RMWEYGGIASEGV**<br>**KEVAERGITKRLESE**<br>**LKTESNKIRTIIKAR**<br>**GLWYPNAQGRTFA**<br>**VFRVDRLHHLVSLL**<br>**TMLGPSPDWIVGV**<br>**SALELCLRNCSWVA**<br>**EKVMNLYPWDAGT**<br>**DSGATYITSKMPTV**<br>**PQEKIRRITSSTPNN**	CACAGAATGTGTTTAATTTTATTTTTAAATATATATATATATA<br>TATATATATATATATAAAGTCGACATATTCTCGTAGAAAGTC<br>TCCAAGTTCAGTGTTCTTTTTTAGTTACATGACACAGAACTGG<br>CAGCTTGTAGTCCAAGGAAAAAAAAAAAAGGGGCATGAAAA<br>ATTCGGCAGAAGTGATGCCGGTGAATTATTGACTGCCGTGGA<br>TCGCTTCATTTAAACTCAATGTTGTAAGTGTTTGCGAGGAAG<br>AAGTTGTCACTGAAGGTCGATTTGTTGTAGACTGACAAAAGA<br>ATTTTAATCACATTTTCTCGAAGAAAACAAAGGACGATTTTTT<br>AAATCTTTAGGGCTTAACCTATACAATTAAGCTTCACTTTTCA<br>TTATAATTTATGCCCGTGTTTTATCCTATAGATGAAGTGATAT<br>GACAAGAGATTAAGAAGCACATTTCGAGACACTATAAAAAT<br>GTCGTCCGTGTCCTAAATCCGCTTTCCGTTCTACTGCCCCTAC<br>GCTTTACACACCGAACGCGCACATCATTTAAGAACTTTTCTA<br>TTCAAACACACAACAAATTCTAACACAACACGTAGAACTTC<br>CCTATTTCTGTTTTCTGAGAAGAAACGAAAAAAGTTTTTCCAT<br>CCCAAAACGGGACACAGTCTGTCGCAGCATACGTCGAGATG<br>ATTACGAACCACAGGTGGAGCTTTGGGGAACATCCTTTCTGA<br>CGAGAGGAGAGGGGCTGAATGACCAAAATCTTGCGTGGAGT<br>CTCGCTGCTCGCCTTTAAATTATGTACAAATGTCAGTTAAGTT<br>TCCACATCGTTCACAACTACCTGTTTCGCCGAAACGATGATA<br>TGTGGAGCTCCCTCAGGTCCGACATCAGTCCGTTCTTATTTTC<br>TGCGGCACGGCGGGTTGTCCTTGCATTTCCTGCGTTGCACCA<br>GCTTGGCAGGGCACGGCTTCCCTCCGTTCTCCGGGTTGAGCTT<br>GATCATTCGCCGCCTCTCCCGGCGTCCGTTCCCGCAGCTCGTC<br>GTGCACGACGTCCAGTCTGACCACGGAGTGACCACGCAGTC<br>GATGATCGGTGGTTGGTCCTTGGTCGCCATGGGCAGGGCGGG<br>CGAAGTGGAGAGTGGCGTCAGGGCACTGAGCGGAGCTCTGA<br>GCATGGTTTCGCACATTCTGTTGCAGTCCGCGTACCGCTCAA

						**PLSPFYDSSGAPMK**	AGTTGTTCCTGTTGCCCCGGCATCCGCCGTAAATGAACTGCA
						**PVARLTVTRQRVY**	GGCACATCGTCCGCCCGACGTCGTAGTACCAGCGGGGAAG
						**DKSCDDYEPVTPTH**	TAGCCACGGCAGGGGCCCACCTCCTTGGGTTGCATGCAGTTC
						**SYGTNDEDDERME**	TTTTTTGCCTCGGTGAGGTTGACTGTGCAGTCCGGCTTATCCG
						**CRVSDWTDWSDCS**	CCGTGCAGGGGGCCTTCTGCATCAGCTCCACGTTACACATGC
						**VTCGQGHRMRSRQ**	TGAGTGCGGCCGGGCTTAGGTAGAGTCGGGTTCGGACCTTCA
						**YINKPKADMAGCT**	GGCCTTTCCCGCATGTCACAGTGCAAGGCGACCACTCGGACC
						**VPLVDKEVCSQHC**	AGTGGGTGACGGCGCACTTTGGATCCGGTGATTCGTCCGCTG
						**VGDVSCRTTEWGE**	GCGGGGGGTGGTCTCCGGAAGACTTGTTCTTCTCCTTCTGTTT
						**WSECSVTCGKGLR**	GTTCCTCCGCCTGCCGTTGGTCACCCTGTCTTCCTCTTCGCAC
						**TRNRKYLDNQARK**	GTCATCTTCATGCCGGTGCAGGTTTCCTTCTCCATGAGTTCGA
						**KCALELMEKETCTG**	GGGCACACTTCTTCCGCGCCTGGTTGTCCAGGTACTTCCGGTT
						**MKMTCEEEDRVTN**	TCGAGTCCGGAGTCCCTTGCCGCAGGTCACGCTGCACTCGGA
						**GRRRNKQKEKNKS**	CCACTCTCCCCATTCCGTCGTGCGGCAGCTCACGTCCCCGAC
						**SGDHPPPADESPDP**	GCAGTGCTGGGAGCAGACCTCCTTGTCCACGAGCGGCACCG
						**KCAVTHWSEWSPC**	TGCACCCGGCCATGTCCGCCTTCGGTTTGTTGATGTACTGCCT
						**TVTCGKGLKVRTR**	CGAGCGCATCCGGTGGCCCTGGCCGCACGTCACGCTGCAATC
						**LYLSPAALSMCNVE**	GCTCCAGTCCGTCCAGTCGCTGACGCGGCACTCCATCCTCTC
						**LMQKAPCTADKPD**	GTCATCCTCGTCGTTGGTCCCGTACGAATGAGTAGGCGTAAC
						**CTVNLTEAKKNCM**	GGGTTCGTAATCGTCACAGGACTTGTCGTAGACACGTTGGCG
						**QPKEVGPCRGYFPR**	GGTCACAGTGAGACGGGCGACGGGTTTCATGGGTGCTCCAG
						**WYYDVGRTMCLQF**	ATGAGTCGTAGAACGGAGACAGGGGGTTGTTAGGCGTGGAC
						**IYGGCRGNRNNFER**	GACGTGATGCGCCGGATCTTCTCTTGAGGCACCGTCGGCATC
						**YADCNRMCETMLR**	TTCGAAGTGATGTAAGTGGCTCCACTATCCGTTCCCGCATCC
						**APLSALTPLSTSPAL**	CAGGGGTACAGGTTCATGACTTTCTCGGCCACCCAGGAGCAG
						**PMATKDQPPIIDCV**	TTCCGCAAGCAGAGCTCGAGGGCACTCACTCCCACTATCCAG
						**VTPWSDWTSCTTS**	TCTGGGCTCGGACCCAGCATGGTCAGGAGAGATACCAGGTG
						**CGNGRRERRRMIK**	ATGCAGGCGGTCCACTCGGAACACGGCGAACGTCCTGCCTT

						**LNPENGGKPCPAKLVQRRKCKDNPPCRRK***	GGGCGTTAGGGTACCATAAGCCTCGAGCCTTTATGATTGTGCGGATCTTGTTGGACTCGGTCTTGAGTTCGGACTCGAGCCTTTTGGTGATCCCTCTCTCGGCCACTTCTTTCACGCCCTCTGACGCGATGCCTCCGTACTCCCACATCCGGAAGTCGCCCGTGTGGGAGGCGCCGATGATGTCGGAGAAATGCGTCAGCCACTCGTTTTTGGGAAATTCCTTGGGGTGCGTGTGCCTGGACCAGAGCCCTTCGAAGGTCAGTTCGTACTTGGCCTCATCGCATGCGCAGCACTCCTCGATGATCTCCGGCTGCTCATCCTGGTTCTCTTGCTCTTCCTCGCAGAGCTCCCGTATGAGACCTCCTTCGTCCATGTACCAAAGTTCTCTATTCTCCTGAACGGTGGCCTTGAAGACGACGCAATTGGTCCCTGCCTGCGGCGCCTTCCACATCACCTGTATGTCCTGCTTGGGGATGAGCGAGGCGTGCACGATGGCATTCGGACACTCCTCCGAGTACTTGCACATGGCGTCGCCGAACAGCTGGAAGGATCCGACGTCCGGCCTGGCCGAGATGTCGTTCCTCACCTCTTTGGGCTCCACCACCAGCATGAAGCCGATGAACTTCTGTACGGCGAACTGGGAGCTGTAGCCTTGGAGGGTCACTGTGTACAGCTCTCCTGGTTGGTACTTCTCCGGGTTTCCGGATATCTTGATGTAGAAACCGTTGTCTCCCGGCACCTTCGGAATCGTCACACCGGGAGGTGCCCTACGGCAACGCCCAGGGAAGCTCTGGGGGATCATGCGGGGCTCGGAGTCACCCGCCGAGGGGTCGGCGCCCCTGCACTGCCCCATCAGTGCGACCCACACCGTCACGACCGCGAACACAGACACCATTTCGAGATCACCTCAACCGTTCCGAGGTCTCGAAAATTCGAACGATACTCGATCCACGTGTTGGAACACAAGAGAGGGAAACACTCTTCCTTTATCGGAACGACGCGGTAGATCAGTGATGCCCGCGGCGTCGCCTTCGAAAG

PhdPInSeP17	P0DMW6	4,08E-16	55,172	PF00014	87036.35<br><br>**84900.72**	MVSVFAVVTVWVA<br>LMGQCRG**ADPSAG**<br>**DSEPRMIPQSFPGR**<br>**CRRAPPGVTIPKVP**<br>**GDNGFYIKISGNPE**<br>**KYQPGELYTVTLQG**<br>**YSSQFAVQKFIGFM**<br>**LVVEPKEVRNDISA**<br>**RPDVGSFQLFGDA**<br>**MCKYSEECPNAIVH**<br>**ASLIPKQDIQVMW**<br>**KAPQAGTNCVVFK**<br>**ATVQENRELWYMD**<br>**EGGLIRELCEEEQEN**<br>**QDEQPEIIEECCACD**<br>**EAKYELTFEGLWSR**<br>**HTHPKEFPKNEWLT**<br>**HFSDIIGASHTGDF**<br>**RMWEYGGIASEGV**<br>**KEVAERGITKRLESE**<br>**LKTESNKIRTIIKAR**<br>**GLWYPNAQGRTFA**<br>**VFRVDRLHHLVSLL**<br>**TMLGPSPDWIVGV**<br>**SALELCLRNCSWVA**<br>**EKVMNLYPWDAGT**<br>**DSGATYITSKMPTV**<br>**PQEKIRRITSSTPNN**	CACAGAATGTGTTTAATTTTATTTTTAAATATATATATATATA<br>TATATATATATATATAAAGTCGACATATTCTCGTAGAAAGTC<br>TCCAAGTTCAGTGTTCTTTTTTAGTTACATGACACAGAACTGG<br>CAGCTTGTAGTCCAAGGAAAAAAAAAAAAGGGGCATGAAAA<br>ATTCGGCAGAAGTGATGCCGGTGAATTATTGACTGCCGTGGA<br>TCGCTTCATTTAAACTCAATGTTGTAAGTGTTTGCGAGGAAG<br>AAGTTGTCACTGAAGGTCGATTTGTTGTAGACTGACAAAAGA<br>ATTTTAATCACATTTTCTCGAAGAAAACAAAGGACGATTTTTT<br>AAATCTTTAGGGCTTAACCTATACAATTAAGCTTCACTTTTCA<br>TTATAATTTATGCCCGTGTTTTATCCTATAGATGAAGTGATAT<br>GACAAGAGATTAAGAAGCACATTTCGAGACACTATAAAAAT<br>GTCGTCCGTGTCCTAAATCCGCTTTCCGTTCTACTGCCCCTAC<br>GCTTTACACACCGAACGCGCACATCATTTAAGAACTTTTCTA<br>TTCAAACACACAACAAATTCTAACACAACACGTAGAACTTC<br>CCTATTTCTGTTTTCTGAGAAGAAACGAAAAAAGTTTTTCCAT<br>CCCAAAACGGGACACAGTCTGTCGCAGCATACGTCGAGATG<br>ATTACGAACCACAGGTGGAGCTTTGGGGAACATCCTTTCTGA<br>CGAGAGGAGAGGGGCTGAATGACCAAAATCTTGCGTGGAGT<br>CTCGCTGCTCGCCTTTAAATTATGTACAAATGTCAGTTAAGTT<br>TCCACATCGTTCACAACTACCTGTTTCGCCGAAACGATGATA<br>TGTGGAGCTCCCTCAGGTCCGACATCAGTCCGTTCTTATTTTC<br>TGCCTTCTTTGTCCCCCAGTCTGTCAGGGCACGGCGGGTTGTC<br>CTTGCATTTCCTGCGTTGCACCAGCTTGGCAGGGCACGGCTTC<br>CCTCCGTTCTCCGGGTTGAGCTTGATCATTCGCCGCCTCTCCC<br>GGCGTCCGTTCCCGCAGCTCGTCGTGCACGACGTCCAGTCTG<br>ACCACGGAGTGACCACGCAGTCGATGATCGGTGGTTGGTCCT<br>TGGTCGCCATGGGCAGGGCGGGCGAAGTGGAGAGTGGCGTC<br>AGGGCACTGAGCGGAGCTCTGAGCATGGTTTCGCACATTCTG

						**PLSPFYDSSGAPMK**	TTGCAGTCCGCGTACCGCTCAAAGTTGTTCCTGTTGCCCCGGC
						**PVARLTVTRQRVY**	ATCCGCCGTAAATGAACTGCAGGCACATCGTCCGCCCGACGT
						**DKSCDDYEPVTPTH**	CGTAGTACCAGCGGGGGAAGTAGCCACGGCAGGGGCCCACC
						**SYGTNDEDDERME**	TCCTTGGGTTGCATGCAGTTCTTTTTGCCTCGGTGAGGTTGA
						**CRVSDWTDWSDCS**	CTGTGCAGTCCGGCTTATCCGCCGTGCAGGGGGCCTTCTGCA
						**VTCGQGHRMRSRQ**	TCAGCTCCACGTTACACATGCTGAGTGCGGCCGGGCTTAGGT
						**YINKPKADMAGCT**	AGAGTCGGGTTCGGACCTTCAGGCCTTTCCCGCATGTCACAG
						**VPLVDKEVCSQHC**	TGCAAGGCGACCACTCGGACCAGTGGGTGACGGCGCACTTT
						**VGDVSCRTTEWGE**	GGATCCGGCACCCTGTCTTCCTCTTCGCACGTCATCTTCATGC
						**WSECSVTCGKGLR**	CGGTGCAGGTTTCCTTCTCCATGAGTTCGAGGGCACACTTCTT
						**TRNRKYLDNQARK**	CCGCGCCTGGTTGTCCAGGTACTTCCGGTTTCGAGTCCGGAG
						**KCALELMEKETCTG**	TCCCTTGCCGCAGGTCACGCTGCACTCGGACCACTCTCCCCA
						**MKMTCEEEDRVPD**	TTCCGTCGTGCGGCAGCTCACGTCCCCGACGCAGTGCTGGGA
						**PKCAVTHWSEWSP**	GCAGACCTCCTTGTCCACGAGCGGCACCGTGCACCCGGCCAT
						**CTVTCGKGLKVRT**	GTCCGCCTTCGGTTTGTTGATGTACTGCCTCGAGCGCATCCGG
						**RLYLSPAALSMCNV**	TGGCCCTGGCCGCACGTCACGCTGCAATCGCTCCAGTCCGTC
						**ELMQKAPCTADKP**	CAGTCGCTGACGCGGCACTCCATCCTCTCGTCATCCTCGTCGT
						**DCTVNLTEAKKNC**	TGGTCCCGTACGAATGAGTAGGCGTAACGGGTTCGTAATCGT
						**MQPKEVGPCRGYF**	CACAGGACTTGTCGTAGACACGTTGGCGGGTCACAGTGAGA
						**PRWYYDVGRTMCL**	CGGGCGACGGGTTTCATGGGTGCTCCAGATGAGTCGTAGAAC
						**QFIYGGCRGNRNN**	GGAGACAGGGGGTTGTTAGGCGTGGACGACGTGATGCGCCG
						**FERYADCNRMCET**	GATCTTCTCTTGAGGCACCGTCGGCATCTTCGAAGTGATGTA
						**MLRAPLSALTPLSTS**	AGTGGCTCCACTATCCGTTCCCGCATCCCAGGGGTACAGGTT
						**PALPMATKDQPPII**	CATGACTTTCTCGGCCACCCAGGAGCAGTTCCGCAAGCAGA
						**DCVVTPWSDWTSC**	GCTCGAGGGCACTCACTCCCACTATCCAGTCTGGGCTCGGAC
						**TTSCGNGRRERRR**	CCAGCATGGTCAGGAGAGATACCAGGTGATGCAGGCGGTCC
						**MIKLNPENGGKPCP**	ACTCGGAACACGGCGAACGTCCTGCCTTGGGCGTTAGGGTAC
							CATAAGCCTCGAGCCTTTATGATTGTGCGGATCTTGTTGGACT

						<u>**AKLVQRRKCKDNP<br>PCPDRLGDKEGRK***</u>	CGGTCTTGAGTTCGGACTCGAGCCTTTTGGTGATCCCTCTCTC<br>GGCCACTTCTTTCACGCCCTCTGACGCGATGCCTCCGTACTCC<br>CACATCCGGAAGTCGCCCGTGTGGGAGGCGCCGATGATGTC<br>GGAGAAATGCGTCAGCCACTCGTTTTTGGGAAATTCCTTGGG<br>GTGCGTGTGCCTGGACCAGAGCCCTTCGAAGGTCAGTTCGTA<br>CTTGGCCTCATCGCATGCGCAGCACTCCTCGATGATCTCCGG<br>CTGCTCATCCTGGTTCTCTTGCTCTTCCTCGCAGAGCTCCCGT<br>ATGAGACCTCCTTCGTCCATGTACCAAAGTTCTCTATTCTCCT<br>GAACGGTGGCCTTGAAGACGACGCAATTGGTCCCTGCCTGCG<br>GCGCCTTCCACATCACCTGTATGTCCTGCTTGGGGATGAGCG<br>AGGCGTGCACGATGGCATTCGGACACTCCTCCGAGTACTTGC<br>ACATGGCGTCGCCGAACAGCTGGAAGGATCCGACGTCCGGC<br>CTGGCCGAGATGTCGTTCCTCACCTCTTTGGGCTCCACCACCA<br>GCATGAAGCCGATGAACTTCTGTACGGCGAACTGGGAGCTG<br>TAGCCTTGGAGGGTCACTGTGTACAGCTCTCCTGGTTGGTACT<br>TCTCCGGGTTTCCGGATATCTTGATGTAGAAACCGTTGTCTCC<br>CGGCACCTTCGGAATCGTCACACCGGGAGGTGCCCTACGGC<br>AACGCCCAGGGAAGCTCTGGGGGATCATGCGGGGCTCGGAG<br>TCACCCGCCGAGGGGTCGGCGCCCCTGCACTGCCCCATCAGT<br>GCGACCCACACCGTCACGACCGCGAACACAGACACCATTTC<br>GAGATCACCTCAACCGTTCCGAGGTCTCGAAAATTCGAACG<br>ATACTCGATCCACGTGTTGGAACACAAGAGAGGGAAACACT<br>CTTCCTTTATCGGAACGACGCGGTAGATCAGTGATGCCCGCG<br>GCGTCGCCTTCGAAAG

PhdPInSeP18	P0DMW6	4,06E-16	55,172	PF00014	86224.5<br><br>**84088.87**	MVSVFAVVTVWVA<br>LMGQCRG**ADPSAG**<br>**DSEPRMIPQSFPGR**<br>**CRRAPPGVTIPKVP**<br>**GDNGFYIKISGNPE**<br>**KYQPGELYTVTLQG**<br>**YSSQFAVQKFIGFM**<br>**LVVEPKEVRNDISA**<br>**RPDVGSFQLFGDA**<br>**MCKYSEECPNAIVH**<br>**ASLIPKQDIQVMW**<br>**KAPQAGTNCVVFK**<br>**ATVQENRELWYMD**<br>**EGGLIRELCEEEQEN**<br>**QDEQPEIIEECCACD**<br>**EAKYELTFEGLWSR**<br>**HTHPKEFPKNEWLT**<br>**HFSDIIGASHTGDF**<br>**RMWEYGGIASEGV**<br>**KEVAERGITKRLESE**<br>**LKTESNKIRTIIKAR**<br>**GLWYPNAQGRTFA**<br>**VFRVDRLHHLVSLL**<br>**TMLGPSPDWIVGV**<br>**SALELCLRNCSWVA**<br>**EKVMNLYPWDAGT**<br>**DSGATYITSKMPTV**<br>**PQEKIRRITSSTPNN**	CACAGAATGTGTTTAATTTTATTTTTAAATATATATATATATA<br>TATATATATATATATATATATATAAAGTCGACATATTCTCGTA<br>GAAAGTCTCCAAGTTCAGTGTTCTTTTTTAGTTACATGACACA<br>GAACTGGCAGCTTGTAGTCCAAGGAAAAAAAAAAAGGGGC<br>ATGAAAAATTCGGCAGAAGTGATGCCGGTGAATTATTGACTG<br>CCGTGGATCGCTTCATTTAAACTCAATGTTGTAAGTGTTTGCG<br>AGGAAGAAGTTGTCACTGAAGGTCGATTTGTTGTAGACTGAC<br>AAAAGAATTTTAATCACATTTTCTCGAAGAAAACAAAGGAC<br>GATTTTTTAAATCTTTAGGGCTTAACCTATACAATTAAGCTTC<br>ACTTTTCATTATAATTTATGCCCGTGTTTTATCCTATAGATGA<br>AGTGATATGACAAGAGATTAAGAAGCACATTTCGAGACACT<br>ATAAAAATGTCGTCCGTGTCCTAAATCCGCTTTCCGTTCTACT<br>GCCCCTACGCTTTACACACCGAACGCGCACATCATTTAAGAA<br>CTTTTCTATTCAAACACACAACAAATTCTAACACAACACGTA<br>GAACTTCCCTATTTCTGTTTTCTGAGAAGAAACGAAAAAAGT<br>TTTTCCATCCCAAAACGGGACACAGTCTGTCGCAGCATACGT<br>CGAGATGATTACGAACCACAGGTGGAGCTTTGGGGAACATC<br>CTTTCTGACGAGAGGAGAGGGGCTGAATGACCAAAATCTTG<br>CGTGGAGTCTCGCTGCTCGCCTTTAAATTATGTACAAATGTCA<br>GTTAAGTTTCCACATCGTTCACAACTACCTGTTTCGCCGAAAC<br>GATGATATGTGGAGCTCCCTCAGGTCCGACATCAGTCCGTTC<br>TTATTTTCTGCGGCACGGCGGGTTGTCCTTGCATTTCCTGCGTT<br>GCACCAGCTTGGCAGGGCACGGCTTCCCTCCGTTCTCCGGGT<br>TGAGCTTGATCATTCGCCGCCTCTCCCGGCGTCCGTTCCCGCA<br>GCTCGTCGTGCACGACGTCCAGTCTGACCACGGAGTGACCAC<br>GCAGTCGATGATCGGTGGTTGGTCCTTGGTCGCCATGGGCAG<br>GGCGGGCGAAGTGGAGAGTGGCGTCAGGGCACTGAGCGGA<br>GCTCTGAGCATGGTTTCGCACATTCTGTTGCAGTCCGCGTACC

						**PLSPFYDSSGAPMK**<br>**PVARLTVTRQRVY**<br>**DKSCDDYEPVTPTH**<br>**SYGTNDEDDERME**<br>**CRVSDWTDWSDCS**<br>**VTCGQGHRMRSRQ**<br>**YINKPKADMAGCT**<br>**VPLVDKEVCSQHC**<br>**VGDVSCRTTEWGE**<br>**WSECSVTCGKGLR**<br>**TRNRKYLDNQARK**<br>**KCALELMEKETCTG**<br>**MKMTCEEEDRVPD**<br>**PKCAVTHWSEWSP**<br>**CTVTCGKGLKVRT**<br>**RLYLSPAALSMCNV**<br>**ELMQKAPCTADKP**<br>**DCTVNLTEAKKNC**<br>**MQPKEVGPCRGYF**<br>**PRWYYDVGRTMCL**<br>**QFIYGGCRGNRNN**<br>**FERYADCNRMCET**<br>**MLRAPLSALTPLSTS**<br>**PALPMATKDQPPII**<br>**DCVVTPWSDWTSC**<br>**TTSCGNGRRERRR**<br>**MIKLNPENGGKPCP**	GCTCAAAGTTGTTCCTGTTGCCCCGGCATCCGCCGTAAATGA<br>ACTGCAGGCACATCGTCCGCCCGACGTCGTAGTACCAGCGG<br>GGGAAGTAGCCACGGCAGGGGCCCACCTCCTTGGGTTGCAT<br>GCAGTTCTTTTTGCCTCGGTGAGGTTGACTGTGCAGTCCGGC<br>TTATCCGCCGTGCAGGGGGCCTTCTGCATCAGCTCCACGTTA<br>CACATGCTGAGTGCGGCCGGGCTTAGGTAGAGTCGGGTTCGG<br>ACCTTCAGGCCTTTCCCGCATGTCACAGTGCAAGGCGACCAC<br>TCGGACCAGTGGGTGACGGCGCACTTTGGATCCGGCACCCTG<br>TCTTCCTCTTCGCACGTCATCTTCATGCCGGTGCAGGTTTCCTT<br>CTCCATGAGTTCGAGGGCACACTTCTTCCGCGCCTGGTTGTCC<br>AGGTACTTCCGGTTTCGAGTCCGGAGTCCCTTGCCGCAGGTC<br>ACGCTGCACTCGGACCACTCTCCCCATTCCGTCGTGCGGCAG<br>CTCACGTCCCCGACGCAGTGCTGGGAGCAGACCTCCTTGTCC<br>ACGAGCGGCACCGTGCACCCGGCCATGTCCGCCTTCGGTTTG<br>TTGATGTACTGCCTCGAGCGCATCCGGTGGCCCTGGCCGCAC<br>GTCACGCTGCAATCGCTCCAGTCCGTCCAGTCGCTGACGCGG<br>CACTCCATCCTCTCGTCATCCTCGTCGTTGGTCCCGTACGAAT<br>GAGTAGGCGTAACGGGTTCGTAATCGTCACAGGACTTGTCGT<br>AGACACGTTGGCGGGTCACAGTGAGACGGGCGACGGGTTTC<br>ATGGGTGCTCCAGATGAGTCGTAGAACGGAGACAGGGGGTT<br>GTTAGGCGTGGACGACGTGATGCGCCGGATCTTCTCTTGAGG<br>CACCGTCGGCATCTTCGAAGTGATGTAAGTGGCTCCACTATC<br>CGTTCCCGCATCCCAGGGGTACAGGTTCATGACTTTCTCGGC<br>CACCCAGGAGCAGTTCCGCAAGCAGAGCTCGAGGGCACTCA<br>CTCCCACTATCCAGTCTGGGCTCGGACCCAGCATGGTCAGGA<br>GAGATACCAGGTGATGCAGGCGGTCCACTCGGAACACGGCG<br>AACGTCCTGCCTTGGGCGTTAGGGTACCATAAGCCTCGAGCC<br>TTTATGATTGTGCGGATCTTGTTGGACTCGGTCTTGAGTTCGG

						**<u>AKLVQRRKCKDNPPCRRK*</u>**	ACTCGAGCCTTTTGGTGATCCCTCTCTCGGCCACTTCTTTCACGCCCTCTGACGCGATGCCTCCGTACTCCCACATCCGGAAGTCGCCCGTGTGGGAGGCGCCGATGATGTCGGAGAAATGCGTCAGCCACTCGTTTTTGGGAAATTCCTTGGGGTGCGTGTGCCTGGACCAGAGCCCTTCGAAGGTCAGTTCGTACTTGGCCTCATCGCATGCGCAGCACTCCTCGATGATCTCCGGCTGCTCATCCTGGTTCTCTTGCTCTTCCTCGCAGAGCTCCCGTATGAGACCTCCTTCGTCCATGTACCAAAGTTCTCTATTCTCCTGAACGGTGGCCTTGAAGACGACGCAATTGGTCCCTGCCTGCGGCGCCTTCCACATCACCTGTATGTCCTGCTTGGGGATGAGCGAGGCGTGCACGATGGCATTCGGACACTCCTCCGAGTACTTGCACATGGCGTCGCCGAACAGCTGGAAGGATCCGACGTCCGGCCTGGCCGAGATGTCGTTCCTCACCTCTTTGGGCTCCACCACCAGCATGAAGCCGATGAACTTCTGTACGGCGAACTGGGAGCTGTAGCCTTGGAGGGTCACTGTGTACAGCTCTCCTGGTTGGTACTTCTCCGGGTTTCCGGATATCTTGATGTAGAAACCGTTGTCTCCCGGCACCTTCGGAATCGTCACACCGGGAGGTGCCCTACGGCAACGCCCAGGGAAGCTCTGGGGGATCATGCGGGGCTCGGAGTCACCCGCCGAGGGGTCGGCGCCCCTGCACTGCCCCATCAGTGCGACCCACACCGTCACGACCGCGAACACAGACACCATTTCGAGATCACCTCAACCGTTCCGAGGTCTCGAAAATTCGAACGATACTCGATCCACGTGTTGGAACACAAGAGAGGGAAACACTCTTCCTTTATCGGAACGACGCGGTAGATCAGTGATGCCCGCGGCGTCGCCTTCGAAAG

PhdPInSeP19	P0DMW6	4,00E-16	55,172	PF00014	89140.62<br><br>**87004.99**	MVSVFAVVTVWVA<br>LMGQCRG**ADPSAG**<br>**DSEPRMIPQSFPGR**<br>**CRRAPPGVTIPKVP**<br>**GDNGFYIKISGNPE**<br>**KYQPGELYTVTLQG**<br>**YSSQFAVQKFIGFM**<br>**LVVEPKEVRNDISA**<br>**RPDVGSFQLFGDA**<br>**MCKYSEECPNAIVH**<br>**ASLIPKQDIQVMW**<br>**KAPQAGTNCVVFK**<br>**ATVQENRELWYMD**<br>**EGGLIRELCEEEQEN**<br>**QDEQPEIIEECCACD**<br>**EAKYELTFEGLWSR**<br>**HTHPKEFPKNEWLT**<br>**HFSDIIGASHTGDF**<br>**RMWEYGGIASEGV**<br>**KEVAERGITKRLESE**<br>**LKTESNKIRTIIKAR**<br>**GLWYPNAQGRTFA**<br>**VFRVDRLHHLVSLL**<br>**TMLGPSPDWIVGV**<br>**SALELCLRNCSWVA**<br>**EKVMNLYPWDAGT**<br>**DSGATYITSKMPTV**<br>**PQEKIRRITSSTPNN**	CACAGAATGTGTTTAATTTTATTTTTAAATATATATATATATA<br>TATATATATATATATATATATATAAAGTCGACATATTCTCGTA<br>GAAAGTCTCCAAGTTCAGTGTTCTTTTTTAGTTACATGACACA<br>GAACTGGCAGCTTGTAGTCCAAGGAAAAAAAAAAAAGGGGC<br>ATGAAAAAATTCGGCAGAAGTGATGCCGGTGAATTATTGACTG<br>CCGTGGATCGCTTCATTTAAACTCAATGTTGTAAGTGTTTGCG<br>AGGAAGAAGTTGTCACTGAAGGTCGATTTGTTGTAGACTGAC<br>AAAAGAATTTTAATCACATTTTCTCGAAGAAAACAAAGGAC<br>GATTTTTTAAATCTTTAGGGCTTAACCTATACAATTAAGCTTC<br>ACTTTTCATTATAATTTATGCCCGTGTTTTATCCTATAGATGA<br>AGTGATATGACAAGAGATTAAGAAGCACATTTCGAGACACT<br>ATAAAAATGTCGTCCGTGTCCTAAATCCGCTTTCCGTTCTACT<br>GCCCCTACGCTTTACACACCGAACGCGCACATCATTTAAGAA<br>CTTTTCTATTCAAACACACAACAAATTCTAACACAACACGTA<br>GAACTTCCCTATTTCTGTTTTCTGAGAAGAAACGAAAAAAGT<br>TTTTCCATCCCAAAACGGGACACAGTCTGTCGCAGCATACGT<br>CGAGATGATTACGAACCACAGGTGGAGCTTTGGGGAACATC<br>CTTTCTGACGAGAGGAGAGGGGCTGAATGACCAAAATCTTG<br>CGTGGAGTCTCGCTGCTCGCCTTTAAATTATGTACAAATGTCA<br>GTTAAGTTTCCACATCGTTCACAACTACCTGTTTCGCCGAAAC<br>GATGATATGTGGAGCTCCCTCAGGTCCGACATCAGTCCGTTC<br>TTATTTTCTGCGGCACGGCGGGTTGTCCTTGCATTTCCTGCGTT<br>GCACCAGCTTGGCAGGGCACGGCTTCCCTCCGTTCTCCGGGT<br>TGAGCTTGATCATTCGCCGCCTCTCCCGGCGTCCGTTCCCGCA<br>GCTCGTCGTGCACGACGTCCAGTCTGACCACGGAGTGACCAC<br>GCAGTCGATGATCGGTGGTTGGTCCTTGGTCGCCATGGGCAG<br>GGCGGGCGAAGTGGAGAGTGGCGTCAGGGCACTGAGCGGA<br>GCTCTGAGCATGGTTTCGCACATTCTGTTGCAGTCCGCGTACC

						**<u>PLSPFYDSSGAPMK</u>**	GCTCAAAGTTGTTCCTGTTGCCCCGGCATCCGCCGTAAATGA
						**<u>PVARLTVTRQRVY</u>**	ACTGCAGGCACATCGTCCGCCCGACGTCGTAGTACCAGCGG
						**<u>DKSCDDYEPVTPTH</u>**	GGGAAGTAGCCACGGCAGGGGCCCACCTCCTTGGGTTGCAT
						**<u>SYGTNDEDDERME</u>**	GCAGTTCTTTTTTGCCTCGGTGAGGTTGACTGTGCAGTCCGGC
						**<u>CRVSDWTDWSDCS</u>**	TTATCCGCCGTGCAGGGGGCCTTCTGCATCAGCTCCACGTTA
						**<u>VTCGQGHRMRSRQ</u>**	CACATGCTGAGTGCGGCCGGGCTTAGGTAGAGTCGGGTTCGG
						**<u>YINKPKADMAGCT</u>**	ACCTTCAGGCCTTTCCCGCATGTCACAGTGCAAGGCGACCAC
						**<u>VPLVDKEVCSQHC</u>**	TCGGACCAGTGGGTGACGGCGCACTTTGGATCCGGTGATTCG
						**<u>VGDVSCRTTEWGE</u>**	TCCGCTGGCGGGGGGTGGTCTCCGGAAGACTTGTTCTTCTCCT
						**<u>WSECSVTCGKGLR</u>**	TCTGTTTGTTCCTCCGCCTGCCGTTGGTCACCCTGTCTTCCTCT
						**<u>TRNRKYLDNQARK</u>**	TCGCACGTCATCTTCATGCCGGTGCAGGTTTCCTTCTCCATGA
						**<u>KCALELMEKETCTG</u>**	GTTCGAGGGCACACTTCTTCCGCGCCTGGTTGTCCAGGTACTT
						**<u>MKMTCEEEDRVTN</u>**	CCGGTTTCGAGTCCGGAGTCCCTTGCCGCAGGTCACGCTGCA
						**<u>GRRRNKQKEKNKS</u>**	CTCGGACCACTCTCCCCATTCCGTCGTGCGGCAGCTCACGTC
						**<u>SGDHPPPADESPDP</u>**	CCCGACGCAGTGCTGGGAGCAGACCTCCTTGTCCACGAGCG
						**<u>KCAVTHWSEWSPC</u>**	GCACCGTGCACCCGGCCATGTCCGCCTTCGGTTTGTTGATGTA
						**<u>TVTCGKGLKVRTR</u>**	CTGCCTCGAGCGCATCCGGTGGCCCTGGCCGCACGTCACGCT
						**<u>LYLSPAALSMCNVE</u>**	GCAATCGCTCCAGTCCGTCCAGTCGCTGACGCGGCACTCCAT
						**<u>LMQKAPCTADKPD</u>**	CCTCTCGTCATCCTCGTCGTTGGTCCCGTACGAATGAGTAGG
						**<u>CTVNLTEAKKNCM</u>**	CGTAACGGGTTCGTAATCGTCACAGGACTTGTCGTAGACACG
						**<u>QPKEVGPCRGYFPR</u>**	TTGGCGGGTCACAGTGAGACGGGCGACGGGTTTCATGGGTG
						**<u>WYYDVGRTMCLQF</u>**	CTCCAGATGAGTCGTAGAACGGAGACAGGGGGTTGTTAGGC
						**<u>IYGGCRGNRNNFER</u>**	GTGGACGACGTGATGCGCCGGATCTTCTCTTGAGGCACCGTC
						**<u>YADCNRMCETMLR</u>**	GGCATCTTCGAAGTGATGTAAGTGGCTCCACTATCCGTTCCC
						**<u>APLSALTPLSTSPAL</u>**	GCATCCCAGGGGTACAGGTTCATGACTTTCTCGGCCACCCAG
						**<u>PMATKDQPPIIDCV</u>**	GAGCAGTTCCGCAAGCAGAGCTCGAGGGCACTCACTCCCAC
						**<u>VTPWSDWTSCTTS</u>**	TATCCAGTCTGGGCTCGGACCCAGCATGGTCAGGAGAGATA
						**<u>CGNGRRERRRMIK</u>**	CCAGGTGATGCAGGCGGTCCACTCGGAACACGGCGAACGTC

						**LNPENGGKPCPAKLVQRRKCKDNPPCRRK***	CTGCCTTGGGCGTTAGGGTACCATAAGCCTCGAGCCTTTATGATTGTGCGGATCTTGTTGGACTCGGTCTTGAGTTCGGACTCGAGCCTTTTGGTGATCCCTCTCTCGGCCACTTCTTTCACGCCCTCTGACGCGATGCCTCCGTACTCCCACATCCGGAAGTCGCCCGTGTGGGAGGCGCCGATGATGTCGGAGAAATGCGTCAGCCACTCGTTTTTGGGAAATTCCTTGGGGTGCGTGTGCCTGGACCAGAGCCCTTCGAAGGTCAGTTCGTACTTGGCCTCATCGCATGCGCAGCACTCCTCGATGATCTCCGGCTGCTCATCCTGGTTCTCTTGCTCTTCCTCGCAGAGCTCCCGTATGAGACCTCCTTCGTCCATGTACCAAAGTTCTCTATTCTCCTGAACGGTGGCCTTGAAGACGACGCAATTGGTCCCTGCCTGCGGCGCCTTCCACATCACCTGTATGTCCTGCTTGGGGATGAGCGAGGCGTGCACGATGGCATTCGGACACTCCTCCGAGTACTTGCACATGGCGTCGCCGAACAGCTGGAAGGATCCGACGTCCGGCCTGGCCGAGATGTCGTTCCTCACCTCTTTGGGCTCCACCACCAGCATGAAGCCGATGAACTTCTGTACGGCGAACTGGGAGCTGTAGCCTTGGAGGGTCACTGTGTACAGCTCTCCTGGTTGGTACTTCTCCGGGTTTCCGGATATCTTGATGTAGAAACCGTTGTCTCCCGGCACCTTCGGAATCGTCACACCGGGAGGTGCCCTACGGCAACGCCCAGGGAAGCTCTGGGGGATCATGCGGGGCTCGGAGTCACCCGCCGAGGGGTCGGCGCCCCTGCACTGCCCCATCAGTGCGACCCACACCGTCACGACCGCGAACACAGACACCATTTCGAGATCACCTCAACCGTTCCGAGGTCTCGAAAATTCGAACGATACTCGATCCACGTGTTGGAACACAAGAGAGGGAAACACTCTTCCTTTATCGGAACGACGCGGTAGATCAGTGATGCCCGCGGCGTCGCCTTCGAAAG

PhdPInSeP20	P0DMW6	6,02E-16	55,172	PF00014	93114.34<br>**90978.71**	MVSVFAVVTVWVA<br>LMGQCRG**ADPSAG**<br>**DSEPRMIPQSFPGR**<br>**CRRAPPGVTIPKVP**<br>**GDNGFYIKISGNPE**<br>**KYQPGELYTVTLQG**<br>**YSSQFAVQKFIGFM**<br>**LVVEPKEVRNDISA**<br>**RPDVGSFQLFGDA**<br>**MCKYSEECPNAIVH**<br>**ASLIPKQDIQVMW**<br>**KAPQAGTNCVVFK**<br>**ATVQENRELWYMD**<br>**EGGLIRELCEEEQEN**<br>**QDEQPEIIEECCACD**<br>**EAKYELTFEGLWSR**<br>**HTHPKEFPKNEWLT**<br>**HFSDIIGASHTGDF**<br>**RMWEYGGIASEGV**<br>**KEVAERGITKRLESE**<br>**LKTESNKIRTIIKAR**<br>**GLWYPNAQGRTFA**<br>**VFRVDRLHHLVSLL**<br>**TMLGPSPDWIVGV**<br>**SALELCLRNCSWVA**<br>**EKVMNLYPWDAGT**<br>**DSGATYITSKMPTV**<br>**PQEKIRRITSSTPNN**	CACAGAATGTGTTTAATTTTATTTTTAAATATATATATATATA<br>TATATATATATATATAAAGTCGACATATTCTCGTAGAAAGTC<br>TCCAAGTTCAGTGTTCTTTTTTAGTTACATGACACAGAACTGG<br>CAGCTTGTAGTCCAAGGAAAAAAAAAAAAGGGGCATGAAAA<br>ATTCGGCAGAAGTGATGCCGGTGAATTATTGACTGCCGTGGA<br>TCGCTTCATTTAAACTCAATGTTGTAAGTGTTTGCGAGGAAG<br>AAGTTGTCACTGAAGGTCGATTTGTTGTAGACTGACAAAAGA<br>ATTTTAATCACATTTTCTCGAAGAAAACAAAGGACGATTTTTT<br>AAATCTTTAGGGCTTAACCTATACAATTAAGCTTCACTTTTCA<br>TTATAATTTATGCCCGTGTTTTATCCTATAGATGAAGTGATAT<br>GACAAGAGATTAAGAAGCACATTTCGAGACACTATAAAAAT<br>GTCGTCCGTGTCCTAAATCCGCTTTCCGTTCTACTGCCCCTAC<br>GCTTTACACACCGAACGCGCACATCATTTAAGAACTTTTCTA<br>TTCAAACACACAACAAATTCTAACACAACACGTAGAACTTC<br>CCTATTTCTGTTTTCTGAGAAGAAACGAAAAAAGTTTTTCCAT<br>CCCAAAACGGGACACAGTCTGTCGCAGCATACGTCGAGATG<br>ATTACGAACCACAGGTGGAGCTTTGGGGAACATCCTTTCTGA<br>CGAGAGGAGAGGGGCTGAATGACCAAAATCTTGCGTGGAGT<br>CTCGCTGCTCGCCTTTAAATTATGTACAAATGTCAGTTAAGTT<br>TCCACATCGTTCACAACTACCTGTTTCGCCGAAACGATGATA<br>TGTGGAGCTCCCTCAGGTCCGACATCAGTCCGTTCTTATTTTC<br>TGCCTTTTGCGTTCGGTCCTCAGTAGAGTGGGCAGGGCGGCA<br>GGGTGCAGTACTTCCTCTCGATCTTGGGTCCGCAGGCTGCCC<br>CGTTGTTCTTGGGGGGCACCTTGACCGTCTTCGTCCTCTGCTG<br>GGTTCCCCCCTGACCGCAGGTCTTGGAGCACGGCGTCCAGTC<br>AGACCACGGGTTCAGTTGGCAGTCCACGGCTTCTTTGTCCCC<br>CAGTCTGTCAGGGCACGGCGGGTTGTCCTTGCATTTCCTGCGT<br>TGCACCAGCTTGGCAGGGCACGGCTTCCCTCCGTTCTCCGGG

						**PLSPFYDSSGAPMK**	TTGAGCTTGATCATTCGCCGCCTCTCCCGGCGTCCGTTCCCGC
						**PVARLTVTRQRVY**	AGCTCGTCGTGCACGACGTCCAGTCTGACCACGGAGTGACC
						**DKSCDDYEPVTPTH**	ACGCAGTCGATGATCGGTGGTTGGTCCTTGGTCGCCATGGGC
						**SYGTNDEDDERME**	AGGGCGGGCGAAGTGGAGAGTGGCGTCAGGGCACTGAGCG
						**CRVSDWTDWSDCS**	GAGCTCTGAGCATGGTTTCGCACATTCTGTTGCAGTCCGCGT
						**VTCGQGHRMRSRQ**	ACCGCTCAAAGTTGTTCCTGTTGCCCCGGCATCCGCCGTAAA
						**YINKPKADMAGCT**	TGAACTGCAGGCACATCGTCCGCCCGACGTCGTAGTACCAGC
						**VPLVDKEVCSQHC**	GGGGGAAGTAGCCACGGCAGGGGCCCACCTCCTTGGGTTGC
						**VGDVSCRTTEWGE**	ATGCAGTTCTTTTTTGCCTCGGTGAGGTTGACTGTGCAGTCCG
						**WSECSVTCGKGLR**	GCTTATCCGCCGTGCAGGGGGCCTTCTGCATCAGCTCCACGT
						**TRNRKYLDNQARK**	TACACATGCTGAGTGCGGCCGGGCTTAGGTAGAGTCGGGTTC
						**KCALELMEKETCTG**	GGACCTTCAGGCCTTTCCCGCATGTCACAGTGCAAGGCGACC
						**MKMTCEEEDRVPD**	ACTCGGACCAGTGGGTGACGGCGCACTTTGGATCCGGCACC
						**PKCAVTHWSEWSP**	CTGTCTTCCTCTTCGCACGTCATCTTCATGCCGGTGCAGGTTT
						**CTVTCGKGLKVRT**	CCTTCTCCATGAGTTCGAGGGCACACTTCTTCCGCGCCTGGTT
						**RLYLSPAALSMCNV**	GTCCAGGTACTTCCGGTTTCGAGTCCGGAGTCCCTTGCCGCA
						**ELMQKAPCTADKP**	GGTCACGCTGCACTCGGACCACTCTCCCCATTCCGTCGTGCG
						**DCTVNLTEAKKNC**	GCAGCTCACGTCCCCGACGCAGTGCTGGGAGCAGACCTCCTT
						**MQPKEVGPCRGYF**	GTCCACGAGCGGCACCGTGCACCCGGCCATGTCCGCCTTCGG
						**PRWYYDVGRTMCL**	TTTGTTGATGTACTGCCTCGAGCGCATCCGGTGGCCCTGGCC
						**QFIYGGCRGNRNN**	GCACGTCACGCTGCAATCGCTCCAGTCCGTCCAGTCGCTGAC
						**FERYADCNRMCET**	GCGGCACTCCATCCTCTCGTCATCCTCGTCGTTGGTCCCGTAC
						**MLRAPLSALTPLSTS**	GAATGAGTAGGCGTAACGGGTTCGTAATCGTCACAGGACTTG
						**PALPMATKDQPPII**	TCGTAGACACGTTGGCGGGTCACAGTGAGACGGGCGACGGG
						**DCVVTPWSDWTSC**	TTTCATGGGTGCTCCAGATGAGTCGTAGAACGGAGACAGGG
						**TTSCGNGRRERRR**	GGTTGTTAGGCGTGGACGACGTGATGCGCCGGATCTTCTCTT
						**MIKLNPENGGKPCP**	GAGGCACCGTCGGCATCTTCGAAGTGATGTAAGTGGCTCCAC
						**AKLVQRRKCKDNP**	TATCCGTTCCCGCATCCCAGGGGTACAGGTTCATGACTTTCTC

						**PCPDRLGDKEAVD<br>CQLNPWSDWTPCS<br>KTCGQGGTQQRTK<br>TVKVPPKNNGAAC<br>GPKIERKYCTLPPCP<br>LY***	GGCCACCCAGGAGCAGTTCCGCAAGCAGAGCTCGAGGGCAC<br>TCACTCCCACTATCCAGTCTGGGCTCGGACCCAGCATGGTCA<br>GGAGAGATACCAGGTGATGCAGGCGGTCCACTCGGAACACG<br>GCGAACGTCCTGCCTTGGGCGTTAGGGTACCATAAGCCTCGA<br>GCCTTTATGATTGTGCGGATCTTGTTGGACTCGGTCTTGAGTT<br>CGGACTCGAGCCTTTTGGTGATCCCTCTCTCGGCCACTTCTTT<br>CACGCCCTCTGACGCGATGCCTCCGTACTCCCACATCCGGAA<br>GTCGCCCGTGTGGGAGGCGCCGATGATGTCGGAGAAATGCG<br>TCAGCCACTCGTTTTTGGGAAATTCCTTGGGGTGCGTGTGCCT<br>GGACCAGAGCCCTTCGAAGGTCAGTTCGTACTTGGCCTCATC<br>GCATGCGCAGCACTCCTCGATGATCTCCGGCTGCTCATCCTG<br>GTTCTCTTGCTCTTCCTCGCAGAGCTCCCGTATGAGACCTCCT<br>TCGTCCATGTACCAAAGTTCTCTATTCTCCTGAACGGTGGCCT<br>TGAAGACGACGCAATTGGTCCCTGCCTGCGGCGCCTTCCACA<br>TCACCTGTATGTCCTGCTTGGGGATGAGCGAGGCGTGCACGA<br>TGGCATTCGGACACTCCTCCGAGTACTTGCACATGGCGTCGC<br>CGAACAGCTGGAAGGATCCGACGTCCGGCCTGGCCGAGATG<br>TCGTTCCTCACCTCTTTGGGCTCCACCACCAGCATGAAGCCG<br>ATGAACTTCTGTACGGCGAACTGGGAGCTGTAGCCTTGGAGG<br>GTCACTGTGTACAGCTCTCCTGGTTGGTACTTCTCCGGGTTTC<br>CGGATATCTTGATGTAGAAACCGTTGTCTCCCGGCACCTTCG<br>GAATCGTCACACCGGGAGGTGCCCTACGGCAACGCCCAGGG<br>AAGCTCTGGGGGATCATGCGGGGCTCGGAGTCACCCGCCGA<br>GGGGTCGGCGCCCCTGCACTGCCCCATCAGTGCGACCCACA<br>CCGTCACGACCGCGAACACAGACACCATTTCGAGATCACCT<br>CAACCGTTCCGAGGTCTCGAAAATTCGAACGATACTCGATCC<br>ACGTGTTGGAACACAAGAGAGGGAAACACTCTTCCTTTATCG

							GAACGACGCGGTAGATCAGTGATGCCCGCGGCGTCGCCTTC GAAAG

PhdPInSeP2 1	W4VSH9	6,70E-18	39,000	PF00014	19536.67<br>**17422.98**	MMKNIVLVLLVVLV<br>ANASFG**ESFTYLRSR**<br>**RSSDEDESIDRCQL**<br>**PPVTGFCRAYFIMY**<br>**YYNVSSNTCNHFVF**<br>**GGCNGNGNRFLTG**<br>**EDCVEACGGTIDEH**<br>**TEIRSYHYDYDFESY**<br>**DEPCIYPKAVGNCD**<br>**AQIPRFYFDGEECKS**<br>**FFWGGCGGNFNNF**<br>**PTEDDCIQKCVQSP**<br>**EDI***	CGACATCTATTTCTCCATTTTCTTCATCAGATGTCCTCTGGCG<br>ATTGTACACATTTCTGTATACAGTCATCTTCTGTTGGAAAATT<br>GTTGAAATTTCCGCCACACCCTCCCCAAAAGAAGCTTTTGCA<br>TTCTTCTCCATCGAAGTAGAAACGAGGAATTTGAGCGTCACA<br>GTTACCCACCGCCTTGGGGTAAATGCAAGGTTCATCATAACT<br>TTCGAAATCATAGTCGTAATGGTAACTTCGTATCTCCGTGTGT<br>TCGTCAATCGTTCCTCCGCATGCCTCTACGCAGTCTTCACCTG<br>TCAGAAAACGGTTTCCATTTCCATTGCAACCTCCGAAAACAA<br>AGTGGTTGCACGTGTTTGAACTCACATTGTAGTAATACATAA<br>TGAAATATGCCCTACAAAATCCTGTTACTGGAGGTAATTGAC<br>AGCGGTCAATCGATTCATCTTCATCGCTCGATCTTCGGGATCG<br>CAGGTAAGTGAAGGATTCTCCAAATGAAGCATTAGCAACGA<br>GAACGACTAAAAGAACGAGTACAATATTTTTCATCATCCTTT<br>CTGCCTAAGAAGAAACAATGAATTTGCTTACACCAGTATAG<br>AAACTTAAGATTTCGTTTGACGAAG
PhdPInSeP2 2	W4VSH9	2,34E-29	35,882	PF00014	22053.26<br>**19990.64**	MKGNMLMVFLLAS<br>AIGLALS**NPFLPRPN**<br>**YDNIPQCMQPKES**<br>**GLCLAYFPSYYYNP**<br>**ATGNCEDFIYGGC**<br>**QGNANRFVTIQECL**<br>**NTCGGYKPETNEGF**<br>**TLITESAAVTESSEE**<br>**NGEQSEENICFQPS**<br>**VMGPCRANMPRFY**<br>**FNGDKCESFMYGG**<br>**CQGNDNNFRTVEE**<br>**CRQRCGNKSSEEVE**	AAGAGACTCAACCGCATCAGTTTTTCTCTCGCCTCTGCAATTC<br>CCTTACAGGTCAATTTTGAAGTTAAAATGAAAGGAAATATGT<br>TGATGGTTTTTCTCCTGGCTTCTGCAATTGGACTTGCGCTTTCA<br>AATCCATTTCTTCCGAGACCTAATTATGACAATATTCCACAAT<br>GTATGCAGCCAAAGGAGAGTGGCTTATGTCTGGCTTACTTCC<br>CATCTTATTACTATAACCCAGCTACCGGAAATTGCGAAGATT<br>TCATATATGGAGGTTGCCAAGGCAACGCCAATCGTTTCGTCA<br>CCATTCAAGAGTGCCTGAATACATGTGGAGGATATAAACCA<br>GAAACGAATGAAGGTTTTACGCTGATCACAGAATCCGCTGC<br>AGTGACAGAGTCATCTGAGGAAAATGGAGAACAGTCTGAGG<br>AGAATATTTGCTTCCAACCTTCTGTAATGGGACCTTGCAGAG<br>CTAACATGCCTAGGTTTTATTTCAACGGAGATAAATGTGAAT<br>CATTTATGTATGGTGGTTGTCAAGGAAATGATAACAATTTCA

						**SRSEDSSEEKSEETD<br>SKSEE***	GAACAGTGGAAGAATGCCGACAAAGGTGTGGCAATAAGTCT<br>TCCGAGGAAGTAGAATCACGTTCTGAAGATTCATCTGAAGA<br>AAAATCGGAGGAAACAGATTCCAAGTCTGAGGAATGAATCA<br>AAAGAAGGAAAATCGCATCCAAAAGAAGGATGATCATCGCT<br>TCCTCCAACAACGCATTATCTCATTCAAATAAATGCTTTTGAT<br>CTCAAATACGCATTATATCATATTTGATATTTATTTTTATATC<br>ATGTACTGTTTAAGTATATGTAGTTCAATAAAG
PhdPInSeP2<br>3	W4VSH9	5,49E-21	39,869	PF00014	20625.76<br><br>**18337.91**	MNLRFVLFCLVSAT<br>CMWISA**SFKLLRTK<br>RSTDIPSRCLHPPDS<br>GMCYAYFQNYYYN<br>AASGECETFVYGGC<br>QGNDNNFYSRGLC<br>EAVCKPTEDDGND<br>DYYDSVWESLEPLIE<br>GKDDCEYPPVTGE<br>CRALFYRYYFDGEK<br>CGIFVYGGCGGNP<br>NNFETEEECMERCG<br>PKPENENDENEVQ<br>DSL***	CGGTAGTATTATGATGATTATATTCACAAAATAATTCTCGTTT<br>GTAGAAATTTCGTGCTTCAAAATCTTGGTGCAGAAAATTTCT<br>CAGAGACTGTCTTGAACTTCATTTTCATCGTTTTCATTTTCAG<br>GTTTTGGCCCACATCTTTCCATGCACTCTTCCTCAGTTTCAAA<br>GTTGTTTGGATTTCCACCACAACCACCGTATACAAAGATGCC<br>GCATTTCTCTCCGTCGAAATAGTAACGGTAGAACAATGCTCT<br>GCATTCACCAGTTACAGGAGGGTACTCACAGTCATCCTTGCC<br>TTCAATTAAAGGTTCCAAACTCTCCCAGACTGAATCATAGTA<br>ATCATCGTTACCATCGTCCTCAGTTGGTTTGCATACTGCTTCG<br>CAGAGACCTCTAGAATAGAAGTTGTTGTCATTTCCTTGGCAT<br>CCACCATAGACGAATGTTTCGCATTCACCGGATGCTGCATTG<br>TAATAGTAGTTCTGAAAATAAGCGTAGCACATGCCTGAATCT<br>GGTGGATGAAGACATCGACTTGGAATATCAGTTGATCTCTTT<br>GTACGAAGGAGCTTGAAGGAGGCTGAAATCCACATGCAAGT<br>TGCAGATACCAGACAAAACAGTACGAATCGCAAATTCATTTT<br>TCTTCACTGAAATCAAGTTAAATGCCTCAATATATGTTCCTGT

							ATTCTAGATACGACTTTTTGCGGCCTTAGGTGATTTTGCTACT TCACAG
PhdPInSeP24	Q8T0W4	3,58E-13	45,098	PF00014	7517,21	RDYPRSVCDLPPEYG DCNSQIPRYYFDGER CTTFFYSGCGGNGN NFWSSVNCRTECGV RRSGINDI*	GTAAAAGCAACACTCGTATTTAAAATTAATGAACAAAAAAT GTGAAACATTCTTCACTTTAAAGAACAGAAATTTTAAGTTTTC AGCTCTCTGTGTTTGTTCCAAGTTTCTTCACTTGTTCTAAAGAT GATTACAAAAATTTCTCTGTATTTAAAAAGCGTAACTTCTCTC CACGAACTTAGTTACACTATATATCATTGATTCCGGATCGTCT AACGCCACACTCAGTTCTGCAATTGACGCTTGACCAGAAATT GTTACCATTGCCTCCGCAACCGCTGTAGAAGAATGTTGTACA TCTTTCACCATCAAAGTAGTAGCGGGGAATCTGAGAATTGCA ATCACCATATTCTGGAGGAAGGTCGCAAACTGATCTCGGATA ATCTCG
PhdPInSeP25	W4VSH9	5,96E-08	45,763	PF00014	13339,94	FPDSCVLEKHHGVR CKNGTAPGLKWYF DSETTQCLPFRYKGC GGNENRFSSSTSCES YCIPQDYGTCAMRG KAYQNKKGETVICS GTDSVSCPEEYACR	ATTTTTCTCAAACAGTTCTTCCGTGTTACGTGGGCAGCAAATA CCATAAAACGCAAGATGTCTGCACGCATATTCTTCTGGGCAT GATACAGAATCTGTTCCCGAGCAAATCACTGTTTCACCCTTTT TATTTTGATACGCTTTACCTCGCATTGCGCACGTCCCATAATC TTGTGGGATGCAGTATGATTCACAGCTCGTTGAACTCGAAAA ACGATTCTCGTTGCCGCCACAGCCTTTGTACCGAAATGGTAG ACACTGAGTTGTTTCGCTGTCAAAATACCACTTTAAACCAGG

						HLAFYGICCPRNTEE<br>LFEKN	CGCTGTTCCATTCTTACACCGAACGCCATGGTGTTTTTCCAGA<br>ACGCAAGAATCCGGAAA
PhdPInSeP2<br>6	P0DM56	1,85E-12	49,057	PF01826	9158.87<br><br>**7446.64**	MKFMILFAIIVFVSA<br>**QKRLRECEPNEFFN<br>NCGSACPVTCESLR<br>GPPQACNYMCVPR<br>CQCKSGLVRTKNW<br>RCVEPSVCALYA***	TTTTTTTTGAAAAGTGTCTCCAAGAAACAATACCATGAAGTTT<br>ATGATACTGTTTGCTATTATTGTTTTCGTGTCAGCTCAAAAAA<br>GGCTGAGAGAATGTGAACCGAATGAGTTTTTCAACAATTGTG<br>GCTCAGCATGTCCAGTTACCTGCGAGAGTCTTAGGGGACCTC<br>CTCAAGCATGCAATTACATGTGCGTCCCTAGATGTCAGTGTA<br>AAAGTGGTCTCGTTCGGACTAAGAACTGGAGATGCGTCGAA<br>CCTTCAGTTTGTGCCCTCTATGCTTGATGACTGGAGAGAGGA<br>ATCCGGAATCCATCCCAAGAAAACAATCTACAGAGG
PhdPInSeP2<br>7	P0DM56	1,85E-12	49,057	PF01826	9158.87<br><br>**7446.64**	MKFMILFAIIVFVSA<br>**QKRLRECEPNEFFN<br>NCGSACPVTCESLR<br>GPPQACNYMCVPR<br>CQCKSGLVRTKNW<br>RCVEPSVCALYA***	TTTTTTTTGAAAAGTGTCTCCAAGAAACAATACCATGAAGTTT<br>ATGATACTGTTTGCCATTATTGTTTTCGTGTCAGCCCAAAAAA<br>GGCTGAGAGAATGTGAACCGAATGAGTTTTTCAACAATTGTG<br>GCTCAGCATGTCCAGTTACCTGCGAGAGTCTTAGGGGACCTC<br>CTCAAGCATGCAATTACATGTGCGTCCCTAGATGTCAGTGTA<br>AAAGTGGTCTCGTTCGGACTAAGAACTGGAGATGCGTCGAA<br>CCTTCAGTTTGTGCCCTCTATGCTTGATGACTGGAGAGAGGA<br>ATCCGGAATCCATCCCAAGAAAACAATCTACAGAGG
PhdPInSeP2<br>8	P0DM56	5,60E-06	48,000	PF01826	11528,27	AAAHHVHTIFSFLF<br>HATANHNTMKYIA<br>LLLITAVVVVSAQMS<br>CGPREREVSCGTPCA<br>LDCDGYRPGGPTTW<br>DCVTPCIGRCECERG	TTTTTTTTCAAGCAAAAATAATGAGTCTTTATTATTAAGAGCA<br>ATTACATGGCTTTTAGAAGCTAAGATAGGATAAAAGAAGTGT<br>TTCTTTAATTCTTTCTCTACATATTTCTACATTCCCATGGATCC<br>TGGGAGTCTTTTCAGCCACTGCTCACGCAAATCAGCCGTATC<br>TTCTGCATTCCGAGGGCGTAACACATCTTCCTCTTCTGTCTCG<br>GACTAAACCCCTTTCACACTCGCATCGACCGATGCATGGGGT<br>GACACAGTCCCATGTAGTTGGACCTCCGGGTCTGTAACCGTC

						LVRDRRGRCVTPSEC RRYG*	GCAGTCGAGTGCACATGGTGTGCCGCAGCTGACTTCCCGTTC TCTCGGTCCGCAAGACATCTGCGCCGAGACAACTACGACTGC GGTAATTAAAAGTAATGCGATATATTTCATTGTATTGTGATTG GCAGTAGCATGAAATAAAAATGAAAAAATGGTGTGCACATG GTGTGCCGCAGCTG
PhdPInSeP29	P0DM56	5,51E-12	51,923	PF01826	10347,16	VLILFSLPATVKDAT MKYIILLFVIGVAVIS AQTRCGRNERYNAC GTACPLTCDNYRSG PGICTAVCNPGCECL RGFVRDRTGRCVMT SNCRRYG*	CAAGCATAAAAAGCTTCATCCCTAATGAGTTCTAATTTTATTT TCACTTCCGGCTACTGTTAAAGACGCTACAATGAAATATATT ATACTGCTGTTTGTTATTGGTGTCGCTGTCATTTCCGCACAGA CACGATGCGGGCGGAACGAGCGATATAACGCATGTGGTACA GCATGTCCACTTACCTGCGACAATTATAGGTCAGGTCCAGGA ATATGTACCGCCGTGTGTAATCCTGGATGCGAGTGTCTGAGG GGTTTCGTCCGGGACAGGACTGGACGATGTGTTATGACCTCG AATTGCCGAAGATATGGTTAATTAAAATGAAAAGTGACTGA AAAGAATCCAAGATCAATGGAATGCAATAATATACGAAGAA AGAAATAAAGAAACCTTGTTCTTATTCCTTTTTTGCTTATCAA AACCATGTAATTGCTTTTATTAATAAAGAAACTGCCGCTTCCT TAATGAAAAAAAA
PhdPInSeP30	P0DM56	1,88E-12	48,077	PF01826	10508,4	VLILFSLPATVKDAT MKYIILLFVIGVAVIS AQRQCGRNERYDEC GSACQLTCDNYRSG PRMCPAVCVPGCVC QMGFVRDRTGRCV MTSNCRRYG*	CAAGCATAAAAAGCTTCATCCCTAATGAGTTCTAATTTTATTT TCACTTCCGGCTACTGTTAAAGACGCTACAATGAAATATATT ATACTGCTGTTTGTTATTGGTGTCGCTGTCATTTCCGCACAGA GACAATGCGGGCGGAACGAGCGATACGACGAATGTGGTAGC GCATGTCAACTTACCTGCGACAATTATAGGTCAGGTCCAAGA ATGTGTCCCGCTGTGTGTGTTCCTGGATGCGTGTGTCAAATGG GCTTCGTCCGGGACAGGACTGGACGATGTGTTATGACCTCGA ATTGCCGAAGATATGGTTAATTAAAATGAAAAGTGACTGAA AAGGATCCAAGATCAATGGCAATGCAATAATATACGGAGAA AGAAATAAAGAAACCTTGTTATTCATTCCTTTTTTGCTTATAA

							AAACCATGTAATTGCTTTTATTAATAAAGAAACTGCCGCTTC<br>CTTAATGAAAAAAAA
PhdPInSeP3<br>1	P0DM56	1,81E-08	42,593	PF01826	17364,52	EYLLNCCLILLFLYIV<br>CGANEEFRSCGSPCP<br>VTCSNFHERTACVH<br>RCARGCFCKRGFVL<br>GPNDRCIKPENCPA<br>GLTTPDSVSVDTRRS<br>ETEEEVLMDETKTAP<br>SLSAKTTQSVTNKPT<br>QSDMTDTASLADST<br>KLSSSTATDSTIASTV<br>LLSETTNSASISTV	CGCAAATGTGTGAAGCTCAATTAAATTGCTTAAACTAGACTG<br>TTCGTCTTCGCCATGCTTTCGTTTCATCCGTACTCCTTAAGCG<br>AGTTCAGCTGTATACCTGTACGAATAATGAATACAAAACAA<br>AATTCTGTAAGGTACATTAAATCTTGAGAGATTCACTTAAAT<br>GCACCAGCTATATTACTTAACCGTTTTCTGTATTTTTCTTTTGC<br>TAAAAATATGTAAGAATATTTACTTAATTGTTGCTTAATATTA<br>TTATTTCTGTACATAGTGTGTGGAGCCAATGAGGAATTCCGTT<br>CGTGTGGTTCTCCTTGCCCTGTCACGTGTTCAAATTTCCATGA<br>AAGGACTGCTTGCGTTCACCGATGCGCAAGAGGATGTTTCTG<br>CAAACGAGGTTTCGTTTTAGGACCGAACGACAGGTGTATTAA<br>GCCAGAAAACTGTCCTGCCGGTTTGACGACTCCTGACAGCGT<br>TAGTGTTGATACCCGCAGAAGTGAAACAGAGGAAGAAGTCT<br>TAATGGATGAAACGAAGACAGCACCTAGTCTATCAGCTAAA<br>ACTACTCAAAGTGTCACGAATAAACCAACACAGAGTGATAT<br>GACTGATACTGCTTCACTTGCAGATTCCACAAAACTTTCTTCA<br>TCCACAGCAACGGATTCGACAATCGCTTCTACAGTTTTGCTTT<br>CCGAAACTACAAATTCGGCATCTATTTCTACAGTTT

PhdPInSeP32	P0DM56	9,38E-09	50,000	PF01826	71593,64	FSPQGGNLNFVCRH<br>EEEFHMCRSPCEPTC<br>SRVKVNCSQSCIAGC<br>FCRDGLIRASNRAC<br>VSPEECTKRTIPECKE<br>NEEFLECGSPCVVN<br>CSNINDPIFCTKPCH<br>SGCFCKPNFVRDSK<br>GNCVHPRKCSTAGY<br>GQCPMNEHYVRCV<br>NFCNTCWESGDCRS<br>HNCIPGCDCKPGFY<br>RNIYGQCVTIEECLA<br>PLYDSSYPGLPGNCA<br>ENEIYSDCVNLCND<br>CVSKGICNYEFYCTP<br>GCDCKSGYLRDERG<br>VCVPENFCLRRCSED<br>RVFNQCGPSCVSTCE<br>NYKRLLYHPCKQEC<br>VPGCFCPRNLVLGP<br>NGRCIEPKYCPSERP<br>VCPPDQQYYDCIPTC<br>RATCETYNDPCVISD<br>KSCIKGCFCMGGLV<br>RRQDGRCVIPKDCP<br>ARPPTCPPEEAFYQC<br>LPTCRTTCETLNTSIG	GACAAAAGGAAAATTGAGTTTCACCGTGAAGAATTATTCTA<br>GCGAAAGAGAAATGGTCATTTTATTTTCGATATCAAAAAAAC<br>TATGAAGCGGTTTTACGAAAATTTTAAGATAACAAAGTTCAA<br>TAATGTGAAGACAAAAAGTGATAAAAAGTTCAAAGAAAAA<br>AAAAATTTGTTTGCTGGTTTAAATTTGAGTAGTGAAGAGACT<br>TAAATTGTCTGAATACGTCAATACATATGTTTGCCTAGACCC<br>AGACGCAAGCAAAAGGAACCTATCCTAATTCTCACCGCAAG<br>GGGGGAACTTAAATTTTGTATGCAGACATGAAGAAGAATTTC<br>ACATGTGTCGATCTCCATGTGAACCAACATGCAGTAGAGTCA<br>AAGTGAATTGTTCCCAATCTTGTATTGCGGGTTGTTTCTGTAG<br>AGACGGGCTTATAAGAGCAAGTAATAGAGCATGTGTATCCC<br>CTGAAGAATGCACCAAACGTACAATACCTGAATGTAAAGAG<br>AACGAAGAATTCCTAGAATGTGGGTCCCCATGTGTCGTAAAT<br>TGTTCAAATATCAATGATCCTATATTCTGCACCAAGCCTTGCC<br>ATAGTGGTTGCTTCTGTAAGCCGAATTTTGTTCGAGATTCAAA<br>GGGGAATTGTGTTCATCCAAGAAAATGCAGTACGGCTGGCT<br>ACGGTCAATGTCCCATGAATGAACATTACGTAAGATGCGTCA<br>ATTTTTGTAACACATGCTGGGAGAGTGGTGATTGTCGAAGCC<br>ATAACTGCATTCCCGGATGCGATTGCAAACCTGGATTTTACA<br>GAAATATCTACGGGCAATGCGTAACCATCGAAGAATGCCTT<br>GCTCCATTATATGATTCTTCATATCCAGGCTTACCAGGTAACT<br>GTGCGGAGAACGAGATATATTCCGATTGCGTGAATTTGTGTA<br>ACGACTGCGTTTCCAAAGGGATATGTAACTACGAATTTTATT<br>GTACGCCTGGCTGCGATTGCAAATCCGGATATTTACGTGACG<br>AAAGAGGAGTCTGTGTACCGGAAAACTTTTGTTTAAGAAGAT<br>GCAGTGAAGATCGAGTTTTCAATCAATGTGGTCCATCTTGTGT<br>TAGCACATGTGAAAATTACAAGCGACTGCTTTATCATCCTTG<br>TAAACAAGAATGCGTACCCGGTTGCTTTTGTCCCAGAAACCT

						MCATECKPGCFCQP GFVRNKEGICVLPKE CPQPVRPTCPTGEVY YKCVPTCHNTCDN WNLPRRCVNICLPG CFCRRGFVRNRDGK CVRTRNCPIVCGAN EEFRSCGSPCPVTCS NFHERTACVHRCAR GCFCKRGFVLGPND RCIKPENCPAGLTTP DSVSVDTRRSETEEE VLMDETKTAPSLSA KTTQSVTNKPTQSD MTDTASLADSTKLSS STATDSTIASTVLLSE TTNSASISTV	TGTTTTAGGACCCAATGGGAGATGCATAGAACCTAAATATTG CCCTTCAGAAAGGCCCGTTTGCCCGCCTGATCAACAATATTA TGATTGCATTCCAACTTGCCGAGCCACATGTGAGACGTACAA CGATCCCTGTGTGATCTCTGATAAAAGCTGTATTAAAGGGTG TTTTTGCATGGGTGGATTAGTCCGCAGGCAAGATGGCAGATG TGTTATACCAAAAGATTGTCCAGCAAGACCCCCTACTTGTCC TCCAGAAGAAGCTTTCTACCAGTGTCTGCCAACTTGTCGAAC TACTTGTGAAACATTAAATACCTCAATTGGAATGTGTGCTAC TGAATGCAAGCCTGGGTGTTTTTGTCAACCAGGTTTTGTCCGG AATAAAGAAGGCATATGCGTTCTTCCTAAAGAATGTCCACA ACCTGTCAGGCCAACATGTCCTACTGGTGAAGTCTACTATAA ATGCGTCCCAACGTGCCATAACACTTGTGATAATTGGAACCT GCCAAGGCGTTGTGTCAATATATGCCTTCCCGGATGCTTTTGT CGAAGGGGTTTTGTTCGGAACCGCGATGGAAAGTGTGTGAG AACAAGAAATTGTCCAATAGTGTGTGGAGCCAATGAGGAAT TCCGTTCGTGTGGTTCTCCTTGCCCTGTCACGTGTTCAAATTTC CATGAAAGGACTGCTTGCGTTCACCGATGCGCAAGAGGATG TTTCTGCAAACGAGGTTTCGTTTTAGGACCGAACGACAGGTG TATTAAGCCAGAAAACTGTCCTGCCGGTTTGACGACTCCTGA CAGCGTTAGTGTTGATACCCGCAGAAGTGAAACAGAGGAAG AAGTCTTAATGGATGAAACGAAGACAGCACCTAGTCTATCA GCTAAAACTACTCAAAGTGTCACGAATAAACCAACACAGAG TGATATGACTGATACTGCTTCACTTGCAGATTCCACAAAACT TTCTTCATCCACAGCAACGGATTCGACAATCGCTTCTACAGTT TTGCTTTCCGAAACTACAAATTCGGCATCTATTTCTACAGTTT

PhdPInSeP3<br>3	P0DM56	8,43E-09	50,000	PF01826	66253.64<br><br>**64181.14**	MDFLLVTMLSVGIFV<br>GYNA**QPAAECKEN**<br>**EEFLECGSPCVVNC**<br>**SNINDPIFCTKPCHS**<br>**GCFCKPNFVRDSK**<br>**GNCVHPRKCSTAG**<br>**YGQCPMNEHYVRC**<br>**VNFCNTCWESGDC**<br>**RSHNCIPGCDCKPG**<br>**FYRNIYGQCVTIEEC**<br>**LAPLYDSSYPGLPG**<br>**NCAENEIYSDCVNL**<br>**CNDCVSKGICNYEF**<br>**YCTPGCDCKSGYLR**<br>**DERGVCVPENFCLR**<br>**RCSEDRVFNQCGPS**<br>**CVSTCENYKRLLYH**<br>**PCKQECVPGCFCPR**<br>**NLVLGPNGRCIEPK**<br>**YCPSERPVCPPDQQ**<br>**YYDCIPTCRATCET**<br>**YNDPCVISDKSCIK**<br>**GCFCMGGLVRRQD**<br>**GRCVIPKDCPARPP**<br>**TCPPEEAFYQCLPTC**<br>**RTTCETLNTSIGMC**<br>**ATECKPGCFCQPGF**<br>**VRNKEGICVLPKEC**	TAGACATACTCTGTAACTCTAAACATACGACAGGTGCCTGGC<br>AACGGTAAAGCTCGGAATGTGCAAGCCTAAGAGTTCGTGCC<br>TGTTTGTTAAATTAAACACGCTGCATTAGATTGTGATCATGGA<br>TTTCCTTCTGGTGACAATGCTCTCTGTCGGGATTTTTGTCGGAT<br>ATAATGCTCAACCAGCAGCTGAATGTAAAGAGAACGAAGAA<br>TTCCTAGAATGTGGGTCCCCATGTGTCGTAAATTGTTCAAATA<br>TCAATGATCCTATATTCTGCACCAAGCCTTGCCATAGTGGTTG<br>CTTCTGTAAGCCGAATTTTGTTCGAGATTCAAAGGGGAATTG<br>TGTTCATCCAAGAAAATGCAGTACGGCTGGCTACGGTCAATG<br>TCCCATGAATGAACATTACGTAAGATGCGTCAATTTTTGTAA<br>CACATGCTGGGAGAGTGGTGATTGTCGAAGCCATAACTGCAT<br>TCCCGGATGCGATTGCAAACCTGGATTTTACAGAAATATCTA<br>CGGGCAATGCGTAACCATCGAAGAATGCCTTGCTCCATTATA<br>TGATTCTTCATATCCAGGCTTACCAGGTAACTGTGCGGAGAA<br>CGAGATATATTCCGATTGCGTGAATTTGTGTAACGACTGCGTT<br>TCCAAAGGGATATGTAACTACGAATTTTATTGTACGCCTGGC<br>TGCGATTGCAAATCCGGATATTTACGTGACGAAAGAGGAGT<br>CTGTGTACCGGAAAACTTTTGTTTAAGAAGATGCAGTGAAGA<br>TCGAGTTTTCAATCAATGTGGTCCATCTTGTGTTAGCACATGT<br>GAAAATTACAAGCGACTGCTTTATCATCCTTGTAAACAAGAA<br>TGCGTACCCGGTTGCTTTTGTCCCAGAAACCTTGTTTTAGGAC<br>CCAATGGGAGATGCATAGAACCTAAATATTGCCCTTCAGAA<br>AGGCCCGTTTGCCCGCCTGATCAACAATATTATGATTGCATT<br>CCAACTTGCCGAGCCACATGTGAGACGTACAACGATCCCTGT<br>GTGATCTCTGATAAAAGCTGTATTAAAGGGTGTTTTTGCATG<br>GGTGGATTAGTCCGCAGGCAAGATGGCAGATGTGTTATACCA<br>AAAGATTGTCCAGCAAGACCCCCTACTTGTCCTCCAGAAGA<br>AGCTTTCTACCAGTGTCTGCCAACTTGTCGAACTACTTGTGAA

						PQPVRPTCPTGEVY<br>YKCVPTCHNTCDN<br>WNLPRRCVNICLPG<br>CFCRRGFVRNRDG<br>KCVRTRNCPIVCGA<br>NEEFRSCGSPCPVT<br>CSNFHERTACVHR<br>CARGCFCKRGFVL<br>GPNDRCIKPENCPA<br>GLTTPDSVSVDTRR<br>SETEEEVLMDETKT<br>APSLSAKTTQSVTN<br>KPTQSDMTDTASL<br>ADSTKLSSSTATDS<br>TIASTVLLSETTNSA<br>SISTV	ACATTAAATACCTCAATTGGAATGTGTGCTACTGAATGCAAG<br>CCTGGGTGTTTTTGTCAACCAGGTTTTGTCCGGAATAAAGAA<br>GGCATATGCGTTCTTCCTAAAGAATGTCCACAACCTGTCAGG<br>CCAACATGTCCTACTGGTGAAGTCTACTATAAATGCGTCCCA<br>ACGTGCCATAACACTTGTGATAATTGGAACCTGCCAAGGCGT<br>TGTGTCAATATATGCCTTCCCGGATGCTTTTGTCGAAGGGGTT<br>TTGTTCGGAACCGCGATGGAAAGTGTGTGAGAACAAGAAAT<br>TGTCCAATAGTGTGTGGAGCCAATGAGGAATTCCGTTCGTGT<br>GGTTCTCCTTGCCCTGTCACGTGTTCAAATTTCCATGAAAGGA<br>CTGCTTGCGTTCACCGATGCGCAAGAGGATGTTTCTGCAAAC<br>GAGGTTTCGTTTTAGGACCGAACGACAGGTGTATTAAGCCAG<br>AAAACTGTCCTGCCGGTTTGACGACTCCTGACAGCGTTAGTG<br>TTGATACCCGCAGAAGTGAAACAGAGGAAGAAGTCTTAATG<br>GATGAAACGAAGACAGCACCTAGTCTATCAGCTAAAACTAC<br>TCAAAGTGTCACGAATAAACCAACACAGAGTGATATGACTG<br>ATACTGCTTCACTTGCAGATTCCACAAAACTTTCTTCATCCAC<br>AGCAACGGATTCGACAATCGCTTCTACAGTTTTGCTTTCCGA<br>AACTACAAATTCGGCATCTATTTCTACAGTTT

PhdPInSeP34	P0DM56	8,31E-09	50,000	PF01826	64401,36	FSPQGAECKENEEFL<br>ECGSPCVVNCSNIN<br>DPIFCTKPCHSGCFC<br>KPNFVRDSKGNCVH<br>PRKCSTAGYGQCPM<br>NEHYVRCVNFCNTC<br>WESGDCRSHNCIPG<br>CDCKPGFYRNIYGQ<br>CVTIEECLAPLYDSSY<br>PGLPGNCAENEIYSD<br>CVNLCNDCVSKGIC<br>NYEFYCTPGCDCKS<br>GYLRDERGVCVPEN<br>FCLRRCSEDRVFNQ<br>CGPSCVSTCENYKRL<br>LYHPCKQECVPGCF<br>CPRNLVLGPNGRCIE<br>PKYCPSERPVCPPDQ<br>QYYDCIPTCRATCET<br>YNDPCVISDKSCIKG<br>CFCMGGLVRRQDG<br>RCVIPKDCPARPPTC<br>PPEEAFYQCLPTCRT<br>TCETLNTSIGMCATE<br>CKPGCFCQPGFVRN<br>KEGICVLPKECPQPV<br>RPTCPTGEVYYKCVP<br>TCHNTCDNWNLPR	GACAAAAGGAAAATTGAGTTTCACCGTGAAGAATTATTCTA<br>GCGAAAGAGAAATGGTCATTTTATTTTCGATATCAAAAAAAC<br>TATGAAGCGGTTTTACGAAAATTTTAAGATAACAAAGTTCAA<br>TAATGTGAAGACAAAAAGTGATAAAAAGTTCAAAGAAAAA<br>AAAAATTTGTTTGCTGGTTTAAATTTGAGTAGTGAAGAGACT<br>TAAATTGTCTGAATACGTCAATACATATGTTTGCCTAGACCC<br>AGACGCAAGCAAAAGGAACCTATCCTAATTCTCACCGCAAG<br>GGGCTGAATGTAAAGAGAACGAAGAATTCCTAGAATGTGGG<br>TCCCCATGTGTCGTAAATTGTTCAAATATCAATGATCCTATAT<br>TCTGCACCAAGCCTTGCCATAGTGGTTGCTTCTGTAAGCCGA<br>ATTTTGTTCGAGATTCAAAGGGGAATTGTGTTCATCCAAGAA<br>AATGCAGTACGGCTGGCTACGGTCAATGTCCCATGAATGAAC<br>ATTACGTAAGATGCGTCAATTTTTGTAACACATGCTGGGAGA<br>GTGGTGATTGTCGAAGCCATAACTGCATTCCCGGATGCGATT<br>GCAAACCTGGATTTTACAGAAATATCTACGGGCAATGCGTAA<br>CCATCGAAGAATGCCTTGCTCCATTATATGATTCTTCATATCC<br>AGGCTTACCAGGTAACTGTGCGGAGAACGAGATATATTCCG<br>ATTGCGTGAATTTGTGTAACGACTGCGTTTCCAAAGGGATAT<br>GTAACTACGAATTTTATTGTACGCCTGGCTGCGATTGCAAAT<br>CCGGATATTTACGTGACGAAAGAGGAGTCTGTGTACCGGAA<br>AACTTTTGTTTAAGAAGATGCAGTGAAGATCGAGTTTTCAAT<br>CAATGTGGTCCATCTTGTGTTAGCACATGTGAAAATTACAAG<br>CGACTGCTTTATCATCCTTGTAAACAAGAATGCGTACCCGGT<br>TGCTTTTGTCCCAGAAACCTTGTTTTAGGACCCAATGGGAGA<br>TGCATAGAACCTAAATATTGCCCTTCAGAAAGGCCCGTTTGC<br>CCGCCTGATCAACAATATTATGATTGCATTCCAACTTGCCGA<br>GCCACATGTGAGACGTACAACGATCCCTGTGTGATCTCTGAT<br>AAAAGCTGTATTAAAGGGTGTTTTTGCATGGGTGGATTAGTC

						RCVNICLPGCFCRRG FVRNRDGKCVRTRN CPIVCGANEEFRSCG SPCPVTCSNFHERTA CVHRCARGCFCKRG FVLGPNDRCIKPENC PAGLTTPDSVSVDTR RSETEEEVLMDETKT APSLSAKTTQSVTNK PTQSDMTDTASLAD STKLSSSTATDSTIAS TVLLSETTNSASISTV	CGCAGGCAAGATGGCAGATGTGTTATACCAAAAGATTGTCC AGCAAGACCCCCTACTTGTCCTCCAGAAGAAGCTTTCTACCA GTGTCTGCCAACTTGTCGAACTACTTGTGAAACATTAAATAC CTCAATTGGAATGTGTGCTACTGAATGCAAGCCTGGGTGTTTT TGTCAACCAGGTTTTGTCCGGAATAAAGAAGGCATATGCGTT CTTCCTAAAGAATGTCCACAACCTGTCAGGCCAACATGTCCT ACTGGTGAAGTCTACTATAAATGCGTCCCAACGTGCCATAAC ACTTGTGATAATTGGAACCTGCCAAGGCGTTGTGTCAATATA TGCCTTCCCGGATGCTTTTGTCGAAGGGGTTTTGTTCGGAACC GCGATGGAAAGTGTGTGAGAACAAGAAATTGTCCAATAGTG TGTGGAGCCAATGAGGAATTCCGTTCGTGTGGTTCTCCTTGCC CTGTCACGTGTTCAAATTTCCATGAAAGGACTGCTTGCGTTCA CCGATGCGCAAGAGGATGTTTCTGCAAACGAGGTTTCGTTTT AGGACCGAACGACAGGTGTATTAAGCCAGAAAACTGTCCTG CCGGTTTGACGACTCCTGACAGCGTTAGTGTTGATACCCGCA GAAGTGAAACAGAGGAAGAAGTCTTAATGGATGAAACGAA GACAGCACCTAGTCTATCAGCTAAAACTACTCAAAGTGTCAC GAATAAACCAACACAGAGTGATATGACTGATACTGCTTCACT TGCAGATTCCACAAAACTTTCTTCATCCACAGCAACGGATTC GACAATCGCTTCTACAGTTTTGCTTTCCGAAACTACAAATTCG GCATCTATTTCTACAGTTT

PhdPInSeP35	P0DM56	6,92E-09	52,000	PF01826	59744.22<br>**57446.46**	MDFLLVTMLSVGIFV<br>GYNAQP**AAGYGQC**<br>**PMNEHYVRCVNFC**<br>**NTCWESGDCRSHN**<br>**CIPGCDCKPGFYRN**<br>**IYGQCVTIEECLAPL**<br>**YDSSYPGLPGNCAE**<br>**NEIYSDCVNLCNDC**<br>**VSKGICNYEFYCTP**<br>**GCDCKSGYLRDER**<br>**GVCVPENFCLRRCS**<br>**EDRVFNQCGPSCVS**<br>**TCENYKRLLYHPCK**<br>**QECVPGCFCPRNLV**<br>**LGPNGRCIEPKYCP**<br>**SERPVCPPDQQYYD**<br>**CIPTCRATCETYND**<br>**PCVISDKSCIKGCFC**<br>**MGGLVRRQDGRC**<br>**VIPKDCPARPPTCPP**<br>**EEAFYQCLPTCRTT**<br>**CETLNTSIGMCATE**<br>**CKPGCFCQPGFVR**<br>**NKEGICVLPKECPQ**<br>**PVRPTCPTGEVYYK**<br>**CVPTCHNTCDNWN**<br>**LPRRCVNICLPGCF**<br>**CRRGFVRNRDGKC**	TAGACATACTCTGTAACTCTAAACATACGACAGGTGCCTGGC<br>AACGGTAAAGCTCGGAATGTGCAAGCCTAAGAGTTCGTGCC<br>TGTTTGTTAAATTAAACACGCTGCATTAGATTGTGATCATGGA<br>TTTCCTTCTGGTGACAATGCTCTCTGTCGGGATTTTTGTCGGAT<br>ATAATGCTCAACCAGCAGCTGGCTACGGTCAATGTCCCATGA<br>ATGAACATTACGTAAGATGCGTCAATTTTTGTAACACATGCT<br>GGGAGAGTGGTGATTGTCGAAGCCATAACTGCATTCCCGGAT<br>GCGATTGCAAACCTGGATTTTACAGAAATATCTACGGGCAAT<br>GCGTAACCATCGAAGAATGCCTTGCTCCATTATATGATTCTTC<br>ATATCCAGGCTTACCAGGTAACTGTGCGGAGAACGAGATAT<br>ATTCCGATTGCGTGAATTTGTGTAACGACTGCGTTTCCAAAG<br>GGATATGTAACTACGAATTTTATTGTACGCCTGGCTGCGATTG<br>CAAATCCGGATATTTACGTGACGAAAGAGGAGTCTGTGTACC<br>GGAAAACTTTTGTTTAAGAAGATGCAGTGAAGATCGAGTTTT<br>CAATCAATGTGGTCCATCTTGTGTTAGCACATGTGAAAATTA<br>CAAGCGACTGCTTTATCATCCTTGTAAACAAGAATGCGTACC<br>CGGTTGCTTTTGTCCCAGAAACCTTGTTTTAGGACCCAATGGG<br>AGATGCATAGAACCTAAATATTGCCCTTCAGAAAGGCCCGTT<br>TGCCCGCCTGATCAACAATATTATGATTGCATTCCAACTTGCC<br>GAGCCACATGTGAGACGTACAACGATCCCTGTGTGATCTCTG<br>ATAAAAGCTGTATTAAAGGGTGTTTTTGCATGGGTGGATTAG<br>TCCGCAGGCAAGATGGCAGATGTGTTATACCAAAAGATTGTC<br>CAGCAAGACCCCCTACTTGTCCTCCAGAAGAAGCTTTCTACC<br>AGTGTCTGCCAACTTGTCGAACTACTTGTGAAACATTAAATA<br>CCTCAATTGGAATGTGTGCTACTGAATGCAAGCCTGGGTGTT<br>TTTGTCAACCAGGTTTTGTCCGGAATAAAGAAGGCATATGCG<br>TTCTTCCTAAAGAATGTCCACAACCTGTCAGGCCAACATGTC<br>CTACTGGTGAAGTCTACTATAAATGCGTCCCAACGTGCCATA

						**VRTRNCPIVCGANEEFRSCGSPCPVTCSNFHERTACVHRCARGCFCKRGFVLGPNDRCIKPENCPAGLTTPDSVSVDTRRSETEEEVLMDETKTAPSLSAKTTQSVTNKPTQSDMTDTASLADSTKLSSSTATDSTIASTVLLSETTNSASISTV**	ACACTTGTGATAATTGGAACCTGCCAAGGCGTTGTGTCAATATATGCCTTCCCGGATGCTTTTGTCGAAGGGGTTTTGTTCGGAACCGCGATGGAAAGTGTGTGAGAACAAGAAATTGTCCAATAGTGTGTGGAGCCAATGAGGAATTCCGTTCGTGTGGTTCTCCTTGCCCTGTCACGTGTTCAAATTTCCATGAAAGGACTGCTTGCGTTCACCGATGCGCAAGAGGATGTTTCTGCAAACGAGGTTTCGTTTTAGGACCGAACGACAGGTGTATTAAGCCAGAAAACTGTCCTGCCGGTTTGACGACTCCTGACAGCGTTAGTGTTGATACCCGCAGAAGTGAAACAGAGGAAGAAGTCTTAATGGATGAAACGAAGACAGCACCTAGTCTATCAGCTAAAACTACTCAAAGTGTCACGAATAAACCAACACAGAGTGATATGACTGATACTGCTTCACTTGCAGATTCCACAAAACTTTCTTCATCCACAGCAACGGATTCGACAATCGCTTCTACAGTTTTGCTTTCCGAAACTACAAATTCGGCATCTATTTCTACAGTTT

PhdPInSeP36	P0DM56	5,09E-09	46,296	PF01826	9795,81	ASFQTVFTKLPNKKT<br>LLILAAVFVVAACYP<br>KKRIVCPAGERWNN<br>CATACPKTCANLKF<br>PPVYCAAVCKRGCE<br>CNPGLVRNSFGACV<br>PPIKC*	GGAAATCGAAATTTTTTTCTGACTGAAAAGGCCCCTTAAGTA<br>ATAACATTCCATGCTATTTACCAAGAATCCGGTCTTCGTTAA<br>AGGCTAATATCAGGATGAATAATACTGACATTTATCTGTAAA<br>AAAAAAATAGCACACACCCTGCAATTAGGTTTTAAATGATAT<br>TTATTTATCACTCAATATTTACAAGTACTAAAACTTTTAGATC<br>AAAGACACCAGCTAGAAATGGCTCATTAAGCAGCTGTTACA<br>CAAGAAAAGTGCTAGCAACAGCTTTCGGCACTGCGTACTTTA<br>AACGATGGTGTATTGTTACATGATTTTTAACACTTGATTGGAG<br>GAACGCATGCGCCGAAAGAATTCCTCACGAGCCCTGGATTG<br>CATTCGCACCCACGTTTGCAGACGGCGGCGCAGTAAACGGG<br>CGGGAACTTCAGATTCGCGCATGTTTTGGGGCATGCTGTTGC<br>GCAGTTATTCCATCTTTCACCTGCTGGACACACGATCCGTTTC<br>TTGGGGTAACAGGCAGCGACCACGAATACGGCGGCAAGGAT<br>CAACAGGGTCTTCTTGTTTGGTAGTTTGGTAAACACTGTCTGG<br>AATGATGCTCATCAGTAACTGCTGGCTCTATTTATTCTATTGG<br>ACCCTCATGATAATCGCGATTGCCTGAATTTATTGTAGCAGA<br>TCAAAGGTCACCAGACGCATATGTTGAAGAGCCTCGAATGT<br>GAAATTGAAACTGAATAAAACTGGAATTTAAAAAATTCTTTT<br>CTACTGAGTACCTGCTGGGAAATTTAACAAGTTGATAGGTT
PhdPInSeP37	P0DM56	3,52E-08	46,296	PF01826	8307.15<br><br>**6662.02**	MKTLLLILAAVFAVA<br>VC**IPKKQVPCPGGE<br>RWNVCAPTCPRTC<br>ANLKLPPVLCNPAC<br>KPGCECNPGLVRN<br>AFGTCVPPIKC***	TTTTTTTTAGATTTCAAATGATATTTATTTATCACTCATGATTT<br>ACAAGCACTAAAACATTTAGATCAAACACACCAGCAAGAAA<br>TGGCTTGTTAGGCAACTGTTACACAAGGAAGCACAAGAAAA<br>ATGCTAGAAGAAGCTTTCTGCACTGCTGGTGGGCTTTAAAGG<br>CTGGTGTATTGTTCAAGATTTTTAACACTTGATTGGAGGGACA<br>CATGTGCCGAAAGCATTCCTCACGAGCCCTGGATTGCATTCG<br>CACCCAGGTTTGCAGGCAGGGTTGCAGAGAACGGGAGGCAA<br>CTTCAGATTGGCGCATGTTCTAGGGCATGTTGGTGCACAGAC<br>ATTCCATCTTTCGCCTCCTGGACACGGGACCTGTTTCTTGGGA

							ATACAGACAGCGACTGCAAATACTGCGGCAAGGATCAACAGGGTCTTCATGTTTCGTAGTTTGGTAAATACTGTCTGGAATGATGTTCATCAGTAACTGCTGGCTCTGTTTATTCTCTTCAACCCTCACGAGAATCGCGATTGCCTGAATTTCTGAAAGGCAACCAGTCTGTTATACATAAATAAAACAAGAACAGCGTGTGAGAACGGAATATTTTGGGAGAGGAAACACCGAGTGGACATCCTCTTCATTTCTTTGAAAGGAATCAACGTATCTTACATTGTAGCAGATCAAAGGTCACC
PhdPInSeP38	P0DM56	4,45E-08	46,296	PF01826	8336.16 **6691.02**	MKTLLILAAVFVVAAC**NPKKRVPCPGGERWNVCAPTCPRTCANLKLPPVLCNPACKPGCECNPGLVRNAFGTCVPPIKC***	TTTTTTTTAGATTTCAAATGATATTTATTTATCACTCATGATTTACAAGCACTAAAACATTTAGATCAAACACACCAGCAAGAAATGGCTTGTTAGGCAACTGTTACACAAGGAAGCACAAGAAAAATGCTAGAAGAAGCTTTCTGCACTGCTGGTGGGCTTTAAAGGCTGGTGTATTGTTCAAGATTTTTAACACTTGATTGGAGGGACACATGTGCCGAAAGCATTCCTCACGAGCCCTGGATTGCATTCGCACCCAGGTTTGCAGGCAGGGTTGCAGAGAACGGGAGGCAACTTCAGATTGGCGCATGTTCTAGGGCATGTTGGTGCACAGACATTCCATCTTTCGCCTCCTGGACACGGGACCCGTTTCTTGGGGTTACAGGCAGCGACCACGAATACGGCGGCAAGAATCAACAGGGTCTTCATGTTGCTGAAAACTGGGTGGAATGATGTTCATCAGTAACTGCTGGCTCTGTTTATTCTCTTCAACCCTCACGAGAATCGCGATTGCCTGAATTTCTGAAAGGCAACCAGTCTGTTATACATAAATAAAACAAGAACAGCGTGTGAGAACGGAATATTTTGGGAGAGGAAACACCGAGTGGACATCCTCTTCATTTCTTTGAAAGGAATCAACGTATCTTACATTGTAGCAGATCAAAGGTCACC

PhdPInSeP39	P0DM56	6,25E-09	46,296	PF01826	8464.29<br><br>**6819.15**	MKTLLLILAAVFVVAAC**YPKKRIVCPAGERWNNCATACPKTCANLKFPPVYCAAVCKRGCECNPGLVRNSFGACVPPIKC***	GGAAATCGAAATTTTTTTCTGACTGAAAAGGCCCCTTAAGTAATAACATTCCATGCTATTTACCAAGAATCCGGTCTTCGTTAAAGGCTAATATCAGGATGAATAATACTGACATTTATCTGTAAAAAAAAAAATAGCACACACCCTGCAATTAGGTTTTAAATGATATTTATTTATCACTCAATATTTACAAGTACTAAAACTTTTAGATCAAAGACACCAGCTAGAAATGGCTCATTAAGCAGCTGTTACACAAGAAAAGTGCTAGCAACAGCTTTCGGCACTGCGTACTTTAAACGATGGTGTATTGTTACATGATTTTTAACACTTGATTGGAGGAACGCATGCGCCGAAAGAATTCCTCACGAGCCCTGGATTGCATTCGCACCCACGTTTGCAGACGGCGGCGCAGTAAACGGGCGGGAACTTCAGATTCGCGCATGTTTTGGGGCATGCTGTTGCGCAGTTATTCCATCTTTCACCTGCTGGACACACGATCCGTTTCTTGGGGTAACAGGCAGCGACCACGAATACGGCGGCAAGGATCAACAGGGTCTTCATGTTTGGTAGTTTGGTAAACACTGTCTGGAATGATGCTCATCAGTAACTGCTGGCTCTATTTATTCTATTGGACCCTCATGATAATCGCGATTGCCTGAATTTCTGAAAGGCAACCAGTCTGTTATACATAAATAAAACAAGAACAGCATGTGATAACGAAGCAATACTTGGAGAGGAAACACCGAGTGGACATCCTCTTCATTTCTTTGAAAGGAATCAACGTATCTTACATTGTAGCAGATCAAAGGTCACCAGACGCATATGTTGAAGAGCCTCGAATGTGAAATTGAAACTGAATAAAACTGGAATTTAAAAAATTCTTTTCTACTGAGTACCTGCTGGGAAATTTAACAAGTTGATAGGTT

PhdPInSeP40	Q8T0W5	4,14E-10	39,535	PF01826	8989.13<br><br>**6689.27**	MKQFAIIYLAVALMTVYLAAS**QTCDPEKEEEYNNCGTACPDTCENYKEDRPCTKQCVSGCFCSKGTVRTEPGGNCVDPSECQ***	TTGATAGTTTTAAGGGACCTTATAAGGCAATTGCAAACAGCA<br>TGTGATTTCTCAACTCGTGCAATTCCACTTGAAACTGGTTGGC<br>AAAAGAATCAAGGAATCACTTGGAAACAATATAGTACACAT<br>AGAAAATGAAACTTCTTTTGCCTACCCATTTGCAGGATGTGA<br>CGTTTTCTGCCATTGCCTTATAAGCAAATGGTATGGCAAAAT<br>GCCATGAGCTCGTTGAGGTACATTAAAAATGAAAAATTTCAG<br>GATCGTCTTCAGGACAGATAATCATATTTCAGGACATTTGAA<br>GATATCCTTATATTTTTTTAGCAATTTCAGGGAAATCGGAAA<br>AGTACGACACCCCATGAAAGGCTTGGATCTGTTGGAGAATAT<br>TTGAATTTCCTACTCCTTCCCGCGTGTCTCGTCTTAATTTTAAA<br>TTTTTAACTCTTCCTTATATTTATGGTACCATAATAAATGCATT<br>TTGGTTTTGTGATAATAACTGTTCAATATGGTAATTCAATATT<br>TACCTAATTTAGCTCTCATTTTAAGACACTTTTATTTAACGTGT<br>CATCGCTATTCAGAATTAAAGTAGGGTGCAAGAGTGAAGCA<br>GGGGTGCTGCGAAAAAGCATTGCAAGCTTGGGGTGCAATGT<br>TAAAAAACGGCTGAAAAACACTGTTTTAATAGACGTGCTTGT<br>TTCAAAAGCTGGTGTCACTGACATTCGGATGGATCAACGCAA<br>TTGCCACCGGGTTCTGTTCTCACGGTTCCTTTACTGCAGAAGC<br>ATCCACTCACACATTGTTTCGTGCAGGGGCGATCTTCCTTGTA<br>ATTCTCACAAGTGTCTGGGCATGCTGTTCCGCAATTGTTGTAT<br>TCTTCTTCTTTCTCGGGGTCGCATGTTTGACTTGCAGCCAAGT<br>AAACCGTCATCAGAGCAACAGCGAGATAAATTATCGCAAAC<br>TGCTTCATGGTTATGCAGAAGTTCTCCACCTGATGTTTTCGAT<br>ACTTGCTTTGGGCCTCGAGAAATCGGAAAGTTAAATAAACA<br>GATGGTTCTTACATATTTATATTGCAAAAAAAAAAAAAAAG<br>AAGTATTAATAATGGAGGTTAAGAAATTGATTGCTTAATGTG<br>TTGTT

PhdPInSeP41	A0A2R4SV19	1,09E-09	38,667	PF01826	8020.16<br>**5534.14**	MKQFAIIYLAVALMTVYLAASEG**CPDGEIYNECGTACPATCANLNPGPCTEQCNPGCFCPPGTVRNSNGDCVPPNQC*****	AGGACAGTACCTCGTTATCAGCAGTGCCAGTGCTATCCCAAACAATAATGGATTTGTTCTGAAAGGTGTAATCTAGACTATAAACATACCCTGCCACCGAACAGTCCATAAAAACAAAAGAAAATAGAGCAACAAAATCCGACCAATCTACTGTGCCTAAATGCAACGTGTGTGTGTGTACTTAAACGGGAGAAGTTTTCTGTCAATTTGATGTCTTTTTATGTTACATGTATGAAATGACAAGAAACGTCATCTTGGAAGAATAAATTCTAATTTATTAACCATCAGACTGCTATCCTTTCGCTGAGGCTTAAATGGAGTTCAGCTGCTTAAAAATTTCAGCAATAAAATGATAGAAGCATATTGTAACTATCGTTTGGCTCGCTTCGAAATCTGAAGACACTAGCATTGATTTGGAGGAACACAGTCGCCATTGGAGTTTCTCACAGTTCCTGGTGGACAGAAACATCCCGGATTGCATTGTTCGGTGCAGGGCCCGGGATTCAGATTTGCACACGTGGCCGGGCAGGCTGTTCCGCATTCGTTGTAGATTTCTCCATCGGGACATCCTTCACTTGCAGCCAAGTAAACCGTCATCAGAGCAACAGCGAGATAAATTATCGCAAACTGCTTCATGGTTATGCAGAAGTTCTCCACCTGATGTTTTCGATACTTGCTTTGGGCCTCGAGAAATCGGAAAGTTAAATAAACAGATGGTTCTTACATATTTATATTGCAAAAAAAAAAAAAAAGAAGTATTAATAATGGAGGTTAAGAAATTGATTGCTTAATGTGTTGTT

PhdPInSeP42	A0A2R4SV19	1,26E-09	39,189	PF01826	8034.19<br>**5534.14**	MKQFAIIYLAVALMTIYLAASEG**CPDGEIYNECGTACPATCANLNPGPCTEQCNPGCFCPPGTVRNSNGDCVPPNQC***	AGGACAGTACCTCGTTATCAGCAGTGCCAGTGCTATCCCAAA<br>CAATAATGGATTTGTTCTGAAAGGTGTAATCTAGACTATAAA<br>CATACCCTGCCACCGAACAGTCCATAAAAACAAAAGAAAAT<br>AGAGCAACAAAATCCGACCAATCTACTGTGCCTAAATGCAA<br>CGTGTGTGTGTGTACTTAAACGGGAGAAGTTTTCTGTCAATTT<br>GATGTCTTTTTATGTTACATGTATGAAATGACAAGAAACGTC<br>ATCTTGGAAGAATAAATTCTAATTTATTAACCATCAGACTGC<br>TATCCTTTCGCTGAGGCTTAAATGGAGTTCAGCTGCTTAAAA<br>ATTTCAGCAATAAAATGATAGAAGCATATTGTAACTATCGTT<br>TGGCTCGCTTCGAAATCTGAAGACACTAGCATTGATTTGGAG<br>GAACACAGTCGCCATTGGAGTTTCTCACAGTTCCTGGTGGAC<br>AGAAACATCCCGGATTGCATTGTTCGGTGCAGGGCCCGGGAT<br>TCAGATTTGCACACGTGGCCGGGCAGGCTGTTCCGCATTCGT<br>TGTAGATTTCTCCATCGGGACATCCTTCACTTGCAGCCAAGT<br>AAATCGTCATCAGAGCAACAGCGAGATAAATTATCGCAAAC<br>TGCTTCATGGTTATGCAGAAGTTCTCCACCTGATGTTTTCGAT<br>ACTTGCTTTGGGCCTCGAGAAATCGGAAAGTTAAATAAACA<br>GATGGTTCTTACATATTTATATTGCAAAAAAAAAAAAAAAG<br>AAGTATTAATAATGGAGGTTAAGAAATTGATTGCTTAATGTG<br>TTGTT

PhdPInSeP43	P0DM56	7,72E-08	43,333	PF01826	8212.83<br>**6567.69**	MKTLLILAAVFVVA<br>AC**NPGKRILCRFGE**<br>**RWNECGSACPRTC**<br>**GNLKAIVNCPAVC**<br>**KIGCECNPGLVRDS**<br>**AGNCVPPSKC***	TTTTTTTCAGATTTGGAATTATATTTATTTTTCACTCAAGAGTT<br>ACAAGCACTAAAACATTTAGATCAAACATTCCAGCAAGCAA<br>TGTCTTGGTAGGGAAATTTTGCGCAAGAAAGAAGCACAAGG<br>AAACTGCTAGCAGAAGCGTTTTGCGTTGCATACTTTCAATGC<br>TAAAGTAGTCTCGAGGACTTCTAACATTTGCTTGGAGGAACG<br>CAATTGCCGGCAGAATCTCTCACCAGGCCTGGATTGCACTCG<br>CAACCAATTTTGCAGACGGCGGGGCAGTTAACGATTGCCTTC<br>AGATTCCCGCATGTTCTGGGGCATGCTGATCCACATTCATTCC<br>ACCTTTCACCAAATCGACACAGGATCCGTTTGCCGGGGTTAC<br>AGGCAGCGACGACAAACACTGCGGCAAGAATCAACAGGGT<br>CTTCATGTTGGTGGAAACTCTCTGGAATAGTGATCATCAGTA<br>ACTGCTGGCTCTTTTTATTCTCTTGAACCCTCATGAGAATCGC<br>GATTCCCAGAATATCGAGTTAATCCTTCACGGCATCAGTCCA<br>TCTTGAGTCAACTGAACTTCATTATATTACTACTGACAAATA<br>AAATCCACAATAATGCGGGAACGCTGCAACTAGTTTCGGAC<br>TAACAGT
PhdPInSeP44	P0DM55	1,90E-13	33,010	PF01826	8994.25<br>**6633.36**	MKQFAVIHLVFALR<br>TVYLAAS**QRCDEKT**<br>**EVYMSCGTSCPDTC**<br>**ANYRDTNRPCPLIC**<br>**EAGCFCKDGTVRR**<br>**ESDGSCVNPNEC***	ATTTATCTCGCTGTTGCTCTGATGACGGTTTACTTGGCTGGGT<br>GCCGAAACTCTACATAACCATGAAGCAGTTCGCTGTAATCCA<br>TCTGGTCTTTGCTCTGAGGACAGTTTATTTAGCCGCGAGTCAA<br>AGATGTGACGAGAAGACAGAAGTGTACATGTCTTGCGGAAC<br>ATCTTGTCCGGATACGTGTGCAAATTACAGGGATACGAACCG<br>GCCCTGCCCCTTGATCTGCGAGGCTGGCTGCTTTTGTAAGGAT<br>GGAACCGTCAGAAGAGAATCCGACGGCTCTTGTGTTAATCCT<br>AATGAGTGCTAGGTCTGGAGCAAGGAGTATGAGAGAAACAC<br>GCTCTTATCGTTTTATTGCTATGATTGTTAAGTAGCTGACCTC<br>CACTTAAGCCTCGGCAGAAAAATGTAATTTTG

PhdPInSeP45	P0DM56	4,95E-07	44,444	PF01826	8408.77 **6721.61**	MKTLLIFATVIVVVAS**HHHKDHPCPEGEKWNDCGSDCPKTCEEPNGPPVCTLRCAVGCECEGGRLRNSKGKCVPPSMC***	TTTAGATTTTAAATGATATTTATTTTTCACTCTATTTACAATCACATATAGTTTTCAGAGCGCATGCTCCACACCAGCAATAGTTTGGTACTAAAATCTGCTACAGAAAAAGCACGAGGAAAACATTAGCAGAAACACTTCTTTTCTTTGGCGACGTGCATAGTTTAAGCACTAAAGTACGCTTTTAACACATGCTTGGAGGGACGCATTTCCCTTTAGAATTCCTCAAACGTCCTCCTTCGCACTCACAACCAACTGCACATCTCAAGGTGCACACGGGGGGACCATTCGGCTCCTCACACGTTTTGGGACAGTCGGAACCACAATCATTCCATTTTTCACCTTCCGGACATGGGTGGTCCTTGTGGTGGTGAGAGGCGACGACCACAATCACGGTGGCGAATATCAACAGCGTCTTCATGTTGGCGAAAGTTGTGGGAAATGGTGATCATCATTAACTGTTGGCTGCTTTTATTCCGTTGGCCCTCTTGAAAATCGTGACTGCCTATGAGTTACTGGACGCGAACTGTCTGTGGTACGTAACTAAAACAAAAACAGCATTCGCCAGCGAACAAAGAGTTTGGGGAAAACACAAGCGGATCTCCGTGGCAACTTTCCTGGAAAGAGTCAACTTATCTTTCTCTGCAGTTCATCAAGGTTGCCAATCAATTACATTCAAAGCTCGGACCTGAAAG
PhdPInSeP46	P0DM55	9,83E-11	45,763	PF01826	8801.08 **6798.58**	MKTFAFVFAAAVLLAVCFA**KKEECDTEKGEEFQTCGTACPDTCDNYNEIRPCTLQCVIGCGCPRGTVRRDDGYCVKPEEC***	TATAAAACTTCTTTATTGCCTAAAATTCGAACTAGCATTCTTCGGGTTTGACACAGTAACCGTCATCCCTCCTGACGGTTCCTCTGGGGCAACCACAACCGATCACACACTGCAGGGTGCATGGGCGGATCTCATTGTAGTTATCGCAGGTGTCTGGACAAGCTGTTCCACAGGTCTGGAATTCCTCTCCCTTCTCGGTGTCGCACTCTTCTTTCTTGGCGAAACAGACGGCAAGCAGGACGGCTGCGGCGAAGACAAAGGCAAAAGTTTTCATGGCTGGGTGGAACTTGAGGTAATAACGAAGACTGAGTTCTGAGGAAAAGGAATCCTTGATGAGTTGATGGCCTTTGTTGTTTTCTTTCGGAATTTATAGAAGGTCGCCTAAATGTTATCTCACAAACAGTTTAGGTGTTGTGGGA

							ATTGCTGCAGCCGTTATCTCATCCGCGAAGCGCTAACGGCCG GTAAACAGCTGTATCAG
PhdPInSeP47	P0DM56	1,66E-11	50,000	PF01826	8424.15 **6870.10**	MKILLLILAAVFVVAA **CNPHKRNRCPQGEIWNECGTACPLTCEMPKPPDVCITVCRVGCECKPGLLRNSAGKCVHPSKC***	GGAAATTTTACGCAAGAAAGAAGCACAAGGAAATTGCTGGCAGAAGTGTTTTGCGTTGCATACTTTCAATGCTAAACATGTCTCGAGGACTTTTTAACACTTGCTTGGATGAACGCATTTGCCGGCAGAATTCCTCAAGAGTCCTGGCTTGCACTCGCAACCAACTCTGCAGACTGTGATGCAGACATCGGGCGGTTTCGGCATCTCGCATGTTAAGGGACATGCTGTTCCACACTCATTCCATATTTCACCTTGTGGGCACCGGTTCCGTTTGTGGGGGTTACAGGCAGCGACTACAAACACTGCGGCAAGGATCAACAGGATCTTCATGTTGGTGGAAACTGTCTGGAATGGTGATCATCAG
PhdPInSeP48	P0DKM8	5,98E-08	40,476	PF00050	11946.32	GVVCKACKVSCEGVQCGKGMRCILRKQRPACVCSPSCSRKKRQMGEVCGSDGRTYKHICRLLKRQCRKNKQLTVEYFGKCQKSCDKVQCTGRKTCLLDQVLRPHCV	GAGGAGTCGTGTGCAAGGCTTGCAAAGTGTCTTGTGAAGGCGTCCAGTGTGGAAAGGGTATGCGGTGCATTTTAAGGAAACAGAGGCCAGCCTGCGTCTGTTCTCCCAGCTGTTCTCGCAAGAAAAGGCAAATGGGTGAAGTATGTGGATCAGATGGCAGAACGTACAAACATATATGCAGGCTGTTGAAAAGGCAGTGCCGGAAAAACAAACAGCTCACCGTGGAGTACTTCGGCAAATGTCAAAAGAGCTGTGACAAAGTGCAATGTACGGGTCGCAAAACTTGTCTTCTGGATCAAGTACTGAGACCCCACTGTGTTCG

PhdPInSeP49	P0DKM8	8,76E-06	59,259	PF00050	36678.11<br>**34641.60**	MVDAALQLLAAILL<br>VTSVRG**QLDSDVLQ**<br>**PDYEYENGQFFEDD**<br>**DGLLKDYIRIPSPKE**<br>**QKFLDGLIIEAGRH**<br>**VLMPELYLPKEEIEK**<br>**GKLKVIELGQPEIEE**<br>**SYMNQKSRRGDSN**<br>**CPDHCNIYKPVCGT**<br>**DGKVYESECHLRKE**<br>**NCGTNSVTSGPWQ**<br>**KCHGKHYLCPSRCL**<br>**DLNDPVCGSDGRI**<br>**YHNICILRKRNCGK**<br>**LIEVQPIRKCYENYR**<br>**QSKDMNGCPKDC**<br>**QEIYKPACGSDGVI**<br>**YYNECFLKKRTCNL**<br>**GIVELSMESCVHVP**<br>**KCPEVCLALYDPVC**<br>**GSDRRLYINHCRML**<br>**QENCGREIKAISRA**<br>**ACASSSSKIVQVSR**<br>**QGK***	GATACGGGTGCCAGTCCATTCGTCCAATAACAGTTCGACGTC<br>GCGCGAACCGGTGACACTGAACTTGTAGCACCCGCACTTTCG<br>GATTGACAGCGGAGGAGGTGAGAAACTATGGTGGATGCGGC<br>TCTGCAATTGCTGGCGGCGATTCTCTTGGTCACGTCGGTGAG<br>AGGTCAGCTGGATTCAGACGTATTGCAACCAGATTACGAGTA<br>TGAAAACGGACAGTTTTTGAAGACGACGATGGTTTGCTGAA<br>AGATTACATACGCATTCCCTCACCAAAGGAGCAAAAATTCTT<br>GGACGGACTCATCATCGAAGCAGGTAGGCATGTACTTATGCC<br>TGAGCTGTATTTACCGAAGGAAGAAATAGAAAAAGGTAAAC<br>TCAAAGTCATTGAACTGGGCCAGCCAGAAATCGAGGAAAGT<br>TACATGAACCAAAAATCTCGACGAGGAGACTCGAACTGTCC<br>GGATCATTGTAACATATACAAACCGGTCTGTGGGACAGATG<br>GAAAAGTGTATGAGAGTGAATGCCATTTGAGGAAGGAGAAT<br>TGCGGGACTAATAGTGTAACTTCAGGTCCTTGGCAGAAATGC<br>CATGGGAAGCATTACCTCTGCCCGTCTCGCTGTTTGGATCTGA<br>ATGACCCTGTGTGTGGCTCCGACGGTAGGATATACCACAATA<br>TCTGCATACTGAGGAAAAGGAATTGTGGAAAACTGATAGAA<br>GTTCAACCGATTCGGAAATGTTACGAAAATTATCGACAATCT<br>AAGGATATGAATGGTTGCCCAAAAGATTGTCAGGAGATATA<br>CAAACCAGCCTGTGGTAGCGATGGAGTCATCTATTACAATGA<br>ATGCTTTCTGAAAAAGAGAACATGCAACTTAGGCATTGTGGA<br>GCTATCGATGGAGAGTTGCGTCCATGTTCCAAAGTGCCCAGA<br>AGTTTGCCTTGCCCTGTATGACCCCGTCTGTGGTTCAGACAGG<br>CGTCTCTACATAAACCACTGCAGGATGTTACAAGAAAACTGC<br>GGGAGAGAAATTAAAGCGATATCAAGAGCTGCTTGCGCCAG<br>TTCTTCGTCCAAGATAGTACAAGTTTCAAGACAAGGAAAATA<br>ATGAAAAAGTTCCCTTCAAAGATATCATTTACGCACTTCTGC<br>ATACAGGGAAAAACAGTATTATTGCTATTAGTAAATATATTT

							TAAATAAACTTTGCTTCCGCACTCTTAAATTTCGTTCAAACTA<br>TATTTCTGAATAAGGGTTTTCAAGATTCAAATTTTGTTTTTGTA<br>CGAAGGAGGTACTATTAAAATACTTACATTTATGAATTTTTTT<br>TCACAATGTACGATATGCACAAAATTGAACAGGAATATAAG<br>TATTATTAAGAATTAATATGCATGCGTTAGGATTCTGGTTATC<br>AATTTTTTTATCATAAAAATATAAATTCACATTGAAGTTGCTT<br>AAGCTTAATTCACTATCTCTACGCTATATAAACTGATATTCAG<br>TTGGCGTTTTCCTGTATGCTTAACCATGATAAGAATGTTCATT<br>GTACATGTTCATACTCAGATATGCCTTAAAGATTTACCCTAAT<br>ATAATAACATTAAATATTATGTAACAGCGTAGTCCTAGTAAC<br>TAAGTTGTTATAAATTTTAAATGTTTACGTTGCATTTATAGTA<br>GATATTGAATGTCGTTAATAATTTTATAACAGATCATTTGAA<br>AATTAAAAAAAAAGTAGTTCTAACTGTAATCATCGC

PhdPInSeP50	P0DKM7	2,20E-05	37,037	PF00050	123142.09	MKRAGCHKGRELN<br>VQFYGTCEEIGEVTG<br>SDEDLPALKERKISS<br>CQDKQCKFGGVCDF<br>NSEGAPFCVCTFHC<br>PAIKEPVCGSDGRM<br>YDNECRMREESCLV<br>QKRIDIASREVCVDH<br>VDIACDGELPVVDP<br>ATGKDYFCGKGEGS<br>KLCPAKTVCHLTQY<br>FAKCCNVAPLIKTCE<br>DTPFGCCPNGRHPIL<br>GPNNGGCPSVCNC<br>NKLGSYSSTCDPVSK<br>QCPCKPGVGGLRCD<br>RCEPGYWGLHKISN<br>GNSGCAHCNCHPY<br>GSVRDDCEQTTGRC<br>VCKHGIQGMKCDM<br>CPIGSVLRPEGCIDES<br>QLNTTAASCAAMEC<br>FYGARCIEKDGLPRC<br>TCDFACAPEDSRDP<br>VCGFDGNTYGSECQ<br>MRLFSCRYHRAIRIR<br>YYGVCRKGYLGDLE<br>TTTVPPRLPKRRSYL	GATGAAAAGAGCTGGTTGCCACAAAGGAAGAGAGCTAAAC<br>GTACAATTTTATGGAACCTGCGAAGAGATCGGTGAAGTGACT<br>GGTTCTGATGAAGATCTTCCAGCTCTTAAGGAACGCAAGATC<br>AGCAGTTGCCAGGACAAGCAGTGTAAGTTCGGTGGGGTATG<br>CGACTTCAATTCCGAGGGAGCTCCCTTCTGTGTTTGTACCTTC<br>CATTGTCCAGCCATCAAGGAGCCTGTCTGCGGGTCCGATGGC<br>CGGATGTACGACAACGAATGTCGGATGAGAGAAGAATCTTG<br>TCTCGTCCAGAAACGTATAGACATCGCATCCAGGGAAGTCTG<br>CGTTGATCACGTGGACATTGCTTGTGATGGTGAACTTCCCGT<br>AGTTGACCCAGCGACTGGAAAAGACTATTTCTGTGGTAAAG<br>GAGAAGGATCAAAACTATGTCCAGCGAAGACAGTTTGCCAT<br>TTGACACAGTATTTTGCGAAATGTTGCAACGTGGCTCCTCTCA<br>TCAAGACGTGTGAAGATACGCCTTTCGGTTGCTGTCCAAATG<br>GACGTCACCCTATATTAGGACCAAACAACGGAGGATGTCCA<br>AGCGTTTGCAACTGCAACAAATTAGGTTCTTACAGCTCCACC<br>TGCGACCCGGTGTCGAAGCAGTGCCCCTGCAAACCGGGGGT<br>GGGAGGACTACGATGTGACCGATGCGAACCAGGCTACTGGG<br>GCCTACACAAGATTTCGAATGGCAACAGTGGATGCGCACAC<br>TGCAACTGTCATCCCTACGGATCTGTCAGAGATGATTGCGAG<br>CAGACGACCGGACGCTGTGTTTGCAAGCATGGCATCCAAGG<br>AATGAAATGTGACATGTGTCCAATCGGAAGCGTTCTCAGGCC<br>GGAAGGTTGTATTGACGAATCACAACTGAATACCACGGCAG<br>CTTCCTGTGCGGCCATGGAGTGTTTCTATGGGGCTCGCTGCAT<br>CGAAAAAGATGGACTGCCCCGCTGCACCTGCGACTTTGCGTG<br>TGCTCCAGAGGATAGCAGAGATCCAGTGTGCGGTTTCGATGG<br>TAATACGTACGGATCCGAATGCCAGATGAGGCTGTTCAGTTG<br>CAGATACCACAGAGCCATTAGAATAAGGTACTACGGAGTCT<br>GCCGTAAGGGATATTTAGGTGATCTGGAAACAACTACGGTG

						KRPDIKSTRAVTANL	CCACCGAGGCTGCCGAAAAGGAGAAGCTACTTGAAGAGGCC
						PENLFMTTRTPYYN	CGACATCAAATCGACGAGGGCTGTTACGGCAAACTTGCCAG
						MTTTVPPILAFAPDP	AAAACCTCTTTATGACTACACGCACGCCTTACTATAATATGA
						EVPIDIPSFSGKSYME	CGACGACCGTACCACCCATCCTTGCTTTCGCACCAGATCCAG
						MYKLEAYSRLSLEVE	AAGTCCCAATAGACATTCCTTCATTTTCTGGCAAGTCCTACAT
						FKTFANEGIILYNGQ	GGAAATGTATAAGCTGGAGGCCTACTCCCGGCTGTCTCTTGA
						TSSKSGDFVSLSVKD	AGTTGAGTTCAAGACATTTGCTAACGAAGGAATCATTTTATA
						GFVEFRYNLGSGSVV	CAACGGTCAGACGTCATCAAAATCGGGAGATTTCGTCTCCCT
						IRSSQKIDLGIYYKV	CAGCGTCAAAGATGGCTTTGTAGAATTCAGATATAACCTAGG
						MAKRYLKDGMLAIE	AAGCGGCTCCGTTGTCATCAGATCGTCACAGAAAATTGACCT
						GQETVSGKSLGTLNS	TGGAATATACTACAAAGTCATGGCTAAAAGGTACCTGAAGG
						LDLVENLYVGYVPG	ACGGAATGCTGGCTATAGAAGGACAAGAAACAGTTTCGGGA
						ASKVVFLNTGVENG	AAAAGTTTGGGAACGTTAAATTCCCTAGACCTAGTCGAGAAC
						LIGCIRRLKIDRQEV	TTGTACGTCGGATATGTACCTGGTGCTTCGAAAGTGGTGTTTT
						NLKMPSSKEILKSSGI	TAAATACTGGAGTCGAAAACGGACTTATTGGCTGTATCCGGC
						NECTLNACTSMPCK	GGCTGAAAATCGATCGGCAGGAAGTAAACCTAAAGATGCCT
						NGGTCISAGSKRYEC	TCATCGAAAGAAATTCTGAAGAGCTCAGGAATAAATGAATG
						RCKEGFSGENCEGTS	TACTTTAAATGCTTGCACCAGTATGCCATGTAAAAATGGAGG
						NPCDSNPCTDGSTC	AACTTGCATTTCGGCTGGCTCGAAACGATACGAGTGCCGCTG
						TVMPGGNFTCKCLD	TAAGGAAGGATTTTCAGGTGAGAACTGTGAAGGAACATCAA
						GRSGKLCDKTKTEV	ATCCCTGTGACAGCAATCCATGTACGGACGGCTCCACCTGTA
						YIPDFHSNSYLELPPL	CTGTAATGCCTGGTGGGAATTTTACCTGTAAATGCCTCGATG
						QNVKHAFSLEIWFL	GAAGATCGGGAAAATTGTGCGACAAAACGAAGACAGAGGT
						ARSENGVLFYNGQM	CTACATTCCAGATTTCCACAGTAACTCGTACCTGGAATTGCCT
						LNRKGDFVAVNLIN	CCTCTCCAGAACGTGAAACATGCTTTTTCGCTTGAAATATGGT
						RYIHFVYNLGSGSAN	TCTTAGCACGATCGGAGAACGGAGTCCTTTTCTACAATGGAC
						IVSRHPVTLNEWHA	AGATGCTCAACAGGAAGGGTGATTTTGTGGCTGTCAACCTCA
						VKISRLRRQGTLKVD	TCAATCGATATATACATTTTGTATACAATCTTGGAAGCGGAT

						NETTVIGKSKPPLAE<br>LNLLQPLYIGGVPDT<br>KHVHKESGMVRGL<br>DGAIMRLIVNGEQW<br>QSITAKAVRAHGVK<br>KYRGHPCTPDACLH<br>NGACTPELNSFICRC<br>PAKFVGKRCERRIRK<br>KGFHQAISFNGTTH<br>LNFRYQNFKGSRSR<br>KSNRFQVRFRTFHP<br>DGLLFWTSKGSSLN<br>GDYLAVALVGGFPE<br>LTYNLGKQKAPWA<br>VRSDQRLDDGMWH<br>SLTIIRKKRLGEIRVD<br>KTYIVSNMSEAGAT<br>DLNTDGLLWIGGSP<br>KLPLGLPSSYYQGFT<br>GCVQSIVIDKSPLHH<br>LLSGKETLRFCDISK<br>VNAPKH*	CGGCCAACATTGTATCTCGGCACCCAGTGACACTGAACGAAT<br>GGCATGCTGTCAAGATCAGCAGGCTCCGAAGACAAGGTACT<br>TTGAAAGTGGACAACGAGACTACAGTCATAGGCAAATCGAA<br>GCCACCATTGGCCGAACTAAACCTGCTGCAGCCCCTCTACAT<br>CGGCGGAGTGCCGGACACAAAACACGTTCACAAAGAGTCAG<br>GCATGGTCAGAGGACTAGACGGTGCCATTATGAGGCTAATC<br>GTGAATGGTGAGCAATGGCAGAGCATCACGGCGAAAGCAGT<br>TCGGGCCCACGGAGTTAAGAAGTACAGGGGACATCCCTGCA<br>CTCCAGACGCTTGCCTCCACAATGGCGCCTGCACTCCAGAAC<br>TAAACTCCTTCATTTGCAGGTGTCCAGCAAAGTTCGTCGGGA<br>AGCGATGCGAACGTCGCATAAGAAAAAAAGGTTTTCATCAG<br>GCAATCTCTTTTAATGGAACGACTCATCTCAATTTCCGTTATC<br>AAAACTTCAAAGGATCGCGAAGCCGCAAGTCCAACCGGTTT<br>CAAGTCCGATTCCGAACATTCCATCCTGATGGACTCCTTTTCT<br>GGACCAGCAAAGGATCCAGCTTGAATGGTGATTATCTCGCA<br>GTTGCACTGGTGGGAGGATTTCCTGAACTTACCTATAACTTG<br>GGCAAGCAAAAAGCTCCATGGGCCGTCAGAAGCGACCAAA<br>GACTCGACGACGGAATGTGGCATTCGTTAACGATAATTAGG<br>AAAAAGAGACTAGGAGAAATTAGAGTAGACAAGACATACA<br>TAGTCTCCAATATGTCTGAAGCTGGTGCTACAGATCTCAACA<br>CCGACGGTCTGCTGTGGATTGGTGGCTCACCAAAATTACCGC<br>TAGGATTGCCATCCAGCTACTATCAAGGATTCACTGGTTGCG<br>TACAATCAATTGTGATTGACAAGAGTCCTTTGCACCACCTGC<br>TGAGCGGAAAAGAGACTCTTCGATTCTGTGACATTTCGAAAG<br>TGAATGCTCCAAAGCATTAAAATGGTTATTGTTCGTCAATGC<br>ATTATGAATGTAATTGTGAAACGATGGAAGGAAACGCTTGTC<br>AGATGAACTAAATGAACTATGCAAAACTTTTTAAAATCTACG<br>AAGAATTACACAAGTTTTGAGCTTAAACATCAATAAACAAT

							GTCACGAGATGGAGAATAGAACTTTCGTTCTATAATATTCCT<br>GATGTTCGGAAAATAATGAAATTTGCGTCTCCTTTACAATATT<br>ATTCCGGCGTTTAGAAATACGCAGTTTAACGAGGAAATGAC<br>GGATAATACAAGAATTTTCCAGACTTAAACATTGTTCAAAAA<br>CACTTTCTTTATATCTGAACAGCATACAGAGTGTGATTAGACT<br>TACTTTATTTTATAACAGTATGCCTGTGTTTTAAAATATATTTT<br>CTTCAAAGCGGAAAATGATTTGCTTTATTCAAACAGAAGCAT<br>AAAAAAACAGTGTTTGGCATTCTATGACGTTAACAGTGAAA<br>ATATGCAATTCGCTTTTCTGAGACAGTATTGTCAATCTTGAAA<br>ATGGCACAATTGTGCTGAACGATGTTGTCGGACCTTTAACGG<br>TGAAATTTGTTTCAAAATGCGAGTTTTGAATCACAATGTTCTT<br>AGACGCCATTTCTACTCGCAGTCAGTCTTGTGTTACCATTCTC<br>CAGTCCTTGTTATTACTTACTTGTTTAAGCTTCGTGAATTAGA<br>GATGATTTTGCTTTCCAGAATCATGTCAAAGGCAGTTATCTGC<br>TTGTGATTTGTTATATTTGAAACTACACTGTAATGACTAACGT<br>AAGTTTTTCATACGTTACTGTAGTATTTATAGAAGACTATAAA<br>GTGCTATATATTATACTTCTTGTATTAGTATAAAAGCTAATAT<br>TTGATATATGTCAATATAGCATGCAAATGTTGTGAAATATCT<br>AAACATATCCGTATCTGAGAGTGAAAACTAAATACGGAAAT<br>ACTTTTAAAGAAGACTTGACTACACAAAGACATGATTCAGTG<br>TGAAGATTGAACTCGAACTCATATACATATGAGAAAGAAAA<br>ATGATTTGAAATTAAACTATGCTAAGAGTGTAAATATAAAAT<br>CCTATGTTTTCCTCGTTATTATTCATTCAAAGCTCTTGAGGGG<br>AAAACCAGTGAAAATTCATGTCTAGACTTGGTATTTTGTATTT<br>ACAATCACAATTAAACATTACTTAGTTTTTATTAAGTACTTCT<br>GGTTAAGGATGTTAATTAAAATGTTGAGATATTATTTCTTTTT<br>ACAAAGCAAGTGATTTCTATGATTATCTTCTAAAGAAAATTA<br>TCTGTAAT

PhdPInSeP51	P0DKM7	2,21E-05	37,037	PF00050	123142.09	MKRAGCHKGRELN<br>VQFYGTCEEIGEVTG<br>SDEDLPALKERKISS<br>CQDKQCKFGGVCDF<br>NSEGAPFCVCTFHC<br>PAIKEPVCGSDGRM<br>YDNECRMREESCLV<br>QKRIDIASREVCVDH<br>VDIACDGELPVVDP<br>ATGKDYFCGKGEGS<br>KLCPAKTVCHLTQY<br>FAKCCNVAPLIKTCE<br>DTPFGCCPNGRHPIL<br>GPNNGGCPSVCNC<br>NKLGSYSSTCDPVSK<br>QCPCKPGVGGLRCD<br>RCEPGYWGLHKISN<br>GNSGCAHCNCHPY<br>GSVRDDCEQTTGRC<br>VCKHGIQGMKCDM<br>CPIGSVLRPEGCIDES<br>QLNTTAASCAAMEC<br>FYGARCIEKDGLPRC<br>TCDFACAPEDSRDP<br>VCGFDGNTYGSECQ<br>MRLFSCRYHRAIRIR<br>YYGVCRKGYLGDLE<br>TTTVPPRLPKRRSYL	GATGAAAAGAGCTGGTTGCCACAAAGGAAGAGAGCTAAAC<br>GTACAATTTTATGGAACCTGCGAAGAGATCGGTGAAGTGACT<br>GGTTCTGATGAAGATCTTCCAGCTCTTAAGGAACGCAAGATC<br>AGCAGTTGCCAGGACAAGCAGTGTAAGTTCGGTGGGGTATG<br>CGACTTCAATTCCGAGGGAGCTCCCTTCTGTGTTTGTACCTTC<br>CATTGTCCAGCCATCAAGGAGCCTGTCTGCGGGTCCGATGGC<br>CGGATGTACGACAACGAATGTCGGATGAGAGAAGAATCTTG<br>TCTCGTCCAGAAACGTATAGACATCGCATCCAGGGAAGTCTG<br>CGTTGATCACGTGGACATTGCTTGTGATGGTGAACTTCCCGT<br>AGTTGACCCAGCGACTGGAAAAGACTATTTCTGTGGTAAAG<br>GAGAAGGATCAAAACTATGTCCAGCGAAGACAGTTTGCCAT<br>TTGACACAGTATTTTGCGAAATGTTGCAACGTGGCTCCTCTCA<br>TCAAGACGTGTGAAGATACGCCTTTCGGTTGCTGTCCAAATG<br>GACGTCACCCTATATTAGGACCAAACAACGGAGGATGTCCA<br>AGCGTTTGCAACTGCAACAAATTAGGTTCTTACAGCTCCACC<br>TGCGACCCGGTGTCGAAGCAGTGCCCCTGCAAACCGGGGGT<br>GGGAGGACTACGATGTGACCGATGCGAACCAGGCTACTGGG<br>GCCTACACAAGATTTCGAATGGCAACAGTGGATGCGCACAC<br>TGCAACTGTCATCCCTACGGATCTGTCAGAGATGATTGCGAG<br>CAGACGACCGGACGCTGTGTTTGCAAGCATGGCATCCAAGG<br>AATGAAATGTGACATGTGTCCAATCGGAAGCGTTCTCAGGCC<br>GGAAGGTTGTATTGACGAATCACAACTGAATACCACGGCAG<br>CTTCCTGTGCGGCCATGGAGTGTTTCTATGGGGCTCGCTGCAT<br>CGAAAAAGATGGACTGCCCCGCTGCACCTGCGACTTTGCGTG<br>TGCTCCAGAGGATAGCAGAGATCCAGTGTGCGGTTTCGATGG<br>TAATACGTACGGATCCGAATGCCAGATGAGGCTGTTCAGTTG<br>CAGATACCACAGAGCCATTAGAATAAGGTACTACGGAGTCT<br>GCCGTAAGGGATATTTAGGTGATCTGGAAACAACTACGGTG

						KRPDIKSTRAVTANL<br>PENLFMTTRTPYYN<br>MTTTVPPILAFAPDP<br>EVPIDIPSFSGKSYME<br>MYKLEAYSRLSLEVE<br>FKTFANEGIILYNGQ<br>TSSKSGDFVSLSVKD<br>GFVEFRYNLGSGSVV<br>IRSSQKIDLGIYYKV<br>MAKRYLKDGMLAIE<br>GQETVSGKSLGTLNS<br>LDLVENLYVGYVPG<br>ASKVVFLNTGVENG<br>LIGCIRRLKIDRQEV<br>NLKMPSSKEILKSSGI<br>NECTLNACTSMPCK<br>NGGTCISAGSKRYEC<br>RCKEGFSGENCEGTS<br>NPCDSNPCTDGSTC<br>TVMPGGNFTCKCLD<br>GRSGKLCDKTKTEV<br>YIPDFHSNSYLELPPL<br>QNVKHAFSLEIWFL<br>ARSENGVLFYNGQM<br>LNRKGDFVAVNLIN<br>RYIHFVYNLGSGSAN<br>IVSRHPVTLNEWHA<br>VKISRLRRQGTLKVD	CCACCGAGGCTGCCGAAAAGGAGAAGCTACTTGAAGAGGCC<br>CGACATCAAATCGACGAGGGCTGTTACGGCAAACTTGCCAG<br>AAAACCTCTTTATGACTACACGCACGCCTTACTATAATATGA<br>CGACGACCGTACCACCCATCCTTGCTTTCGCACCAGATCCAG<br>AAGTCCCAATAGACATTCCTTCATTTTCTGGCAAGTCCTACAT<br>GGAAATGTATAAGCTGGAGGCCTACTCCCGGCTGTCTCTTGA<br>AGTTGAGTTCAAGACATTTGCTAACGAAGGAATCATTTTATA<br>CAACGGTCAGACGTCATCAAAATCGGGAGATTTCGTCTCCCT<br>CAGCGTCAAAGATGGCTTTGTAGAATTCAGATATAACCTAGG<br>AAGCGGCTCCGTTGTCATCAGATCGTCACAGAAAATTGACCT<br>TGGAATATACTACAAAGTCATGGCTAAAAGGTACCTGAAGG<br>ACGGAATGCTGGCTATAGAAGGACAAGAAACAGTTTCGGGA<br>AAAAGTTTGGGAACGTTAAATTCCCTAGACCTAGTCGAGAAC<br>TTGTACGTCGGATATGTACCTGGTGCTTCGAAAGTGGTGTTTT<br>TAAATACTGGAGTCGAAAACGGACTTATTGGCTGTATCCGGC<br>GGCTGAAAATCGATCGGCAGGAAGTAAACCTAAAGATGCCT<br>TCATCGAAAGAAATTCTGAAGAGCTCAGGAATAAATGAATG<br>TACTTTAAATGCTTGCACCAGTATGCCATGTAAAAATGGAGG<br>AACTTGCATTTCGGCTGGCTCGAAACGATACGAGTGCCGCTG<br>TAAGGAAGGATTTTCAGGTGAGAACTGTGAAGGAACATCAA<br>ATCCCTGTGACAGCAATCCATGTACGGACGGCTCCACCTGTA<br>CTGTAATGCCTGGTGGGAATTTTACCTGTAAATGCCTCGATG<br>GAAGATCGGGAAAATTGTGCGACAAAACGAAGACAGAGGT<br>CTACATTCCAGATTTCCACAGTAACTCGTACCTGGAATTGCCT<br>CCTCTCCAGAACGTGAAACATGCTTTTTCGCTTGAAATATGGT<br>TCTTAGCACGATCGGAGAACGGAGTCCTTTTCTACAATGGAC<br>AGATGCTCAACAGGAAGGGTGATTTTGTGGCTGTCAACCTCA<br>TCAATCGATATATACATTTTGTATACAATCTTGGAAGCGGAT

						NETTVIGKSKPPLAE<br>LNLLQPLYIGGVPDT<br>KHVHKESGMVRGL<br>DGAIMRLIVNGEQW<br>QSITAKAVRAHGVK<br>KYRGHPCTPDACLH<br>NGACTPELNSFICRC<br>PAKFVGKRCERRIRK<br>KGFHQAISFNGTTH<br>LNFRYQNFKGSRSR<br>KSNRFQVRFRTFHP<br>DGLLFWTSKGSSLN<br>GDYLAVALVGGFPE<br>LTYNLGKQKAPWA<br>VRSDQRLDDGMWH<br>SLTIIRKKRLGEIRVD<br>KTYIVSNMSEAGAT<br>DLNTDGLLWIGGSP<br>KLPLGLPSSYYQGFT<br>GCVQSIVIDKSPLHH<br>LLSGKETLRFCDISK<br>VNAPKH*	CGGCCAACATTGTATCTCGGCACCCAGTGACACTGAACGAAT<br>GGCATGCTGTCAAGATCAGCAGGCTCCGAAGACAAGGTACT<br>TTGAAAGTGGACAACGAGACTACAGTCATAGGCAAATCGAA<br>GCCACCATTGGCCGAACTAAACCTGCTGCAGCCCCTCTACAT<br>CGGCGGAGTGCCGGACACAAAACACGTTCACAAAGAGTCAG<br>GCATGGTCAGAGGACTAGACGGTGCCATTATGAGGCTAATC<br>GTGAATGGTGAGCAATGGCAGAGCATCACGGCGAAAGCAGT<br>TCGGGCCCACGGAGTTAAGAAGTACAGGGGACATCCCTGCA<br>CTCCAGACGCTTGCCTCCACAATGGCGCCTGCACTCCAGAAC<br>TAAACTCCTTCATTTGCAGGTGTCCAGCAAAGTTCGTCGGGA<br>AGCGATGCGAACGTCGCATAAGAAAAAAAGGTTTTCATCAG<br>GCAATCTCTTTTAATGGAACGACTCATCTCAATTTCCGTTATC<br>AAAACTTCAAAGGATCGCGAAGCCGCAAGTCCAACCGGTTT<br>CAAGTCCGATTCCGAACATTCCATCCTGATGGACTCCTTTTCT<br>GGACCAGCAAAGGATCCAGCTTGAATGGTGATTATCTCGCA<br>GTTGCACTGGTGGGAGGATTTCCTGAACTTACCTATAACTTG<br>GGCAAGCAAAAAGCTCCATGGGCCGTCAGAAGCGACCAAA<br>GACTCGACGACGGAATGTGGCATTCGTTAACGATAATTAGG<br>AAAAAGAGACTAGGAGAAATTAGAGTAGACAAGACATACA<br>TAGTCTCCAATATGTCTGAAGCTGGTGCTACAGATCTCAACA<br>CCGACGGTCTGCTGTGGATTGGTGGCTCACCAAAATTACCGC<br>TAGGATTGCCATCCAGCTACTATCAAGGATTCACTGGTTGCG<br>TACAATCAATTGTGATTGACAAGAGTCCTTTGCACCACCTGC<br>TGAGCGGAAAAGAGACTCTTCGATTCTGTGACATTTCGAAAG<br>TGAATGCTCCAAAGCATTAAAATGGTTATTGTTCGTCAATGC<br>ATTATGAATGTAATTGTGAAACGATGGAAGGAAACGCTTGTC<br>AGATGAACTAAATGAACTATGCAAAACTTTTTAAAATCTACG<br>AAGAATTACACAAGTTTTGAGCTTAAACATCAATAAACAAT

							GTCACGAGATGGAGAATAGAACTTTCGTTCTATAATATTCCT<br>GATGTTCGGAAAATAATGAAATTTGCGTCTCCTTTACAATATT<br>ATTCCGGCGTTTAGAAATACGCAGTTTAACGAGGAAATGAC<br>GGATAATACAAGAATTTTCCAGACTTAAACATTGTTCAAAAA<br>CACTTTCTTTATATCTGAACAGCATACAGAGTGTGATTAGACT<br>TACTTTATTTTATAACAGTATGCCTGTGTTTTAAAATATATTTT<br>CTTCAAAGCGGAAAATGATTTGCTTTATTCAAACAGAAGCAT<br>AAAAAAACAGTGTTTGGCATTCTATGACATTTCTATGACGTT<br>AACAGTGAAAATATGCAATTCGCTTTTCTGAGACAGTATTGT<br>CAATCTTGAAAATGGCACAATTGTGCTGAACGATGTTGTCGG<br>ACCTTTAACGGTGAAATTTGTTTCAAAATGCGAGTTTTGAATC<br>ACAATGTTCTTAGACGCCATTTCTACTCGCAGTCAGTCTTGTG<br>TTACCATTCTCCAGTCCTTGTTATTACTTACTTGTTTAAGCTTC<br>GTGAATTAGAGATGATTTTGCTTTCCAGAATCATGTCAAAGG<br>CAGTTATCTGCTTGTGATTTGTTATATTTGAAACTACACTGTA<br>ATGACTAACGTAAGTTTTTCATACGTTACTGTAGTATTTATAG<br>AAGACTATAAAGTGCTATATATTATACTTCTTGTATTAGTATA<br>AAAGCTAATATTTGATATATGTCAATATAGCATGCAAATGTT<br>GTGAAATATCTAAACATATCCGTATCTGAGAGTGAAAACTAA<br>ATACGGAAATACTTTTAAAGAAGACTTGACTACACAAAGAC<br>ATGATTCAGTGTGAAGATTGAACTCGAACTCATATACATATG<br>AGAAAGAAAAATGATTTGAAATTAAACTATGCTAAGAGTGT<br>AAATATAAAATCCTATGTTTTCCTCGTTATTATTCATTCAAAG<br>CTCTTGAGGGGAAAACCAGTGAAAATTCATGTCTAGACTTGG<br>TATTTTGTATTTACAATCACAATTAAACATTACTTAGTTTTTAT<br>TAAGTACTTCTGGTTAAGGATGTTAATTAAAATGTTGAGATA<br>TTATTTCTTTTTACAAAGCAAGTGATTTCTATGATTATCTTCTA<br>AAGAAAATTATCTGTAAT

PhdPInSeP52	P0DKM7	3,61E-12	50,000	PF00050	36822.57	CDPCEFVRCPTYQVCQLDENRNPTCRCNDVCAPDFRTVCGSDGKTYANECSLRTEACKAQRNVRVIYSGDCSSGTNPCDLIKCGPGQECDIDQFGVASCQCPPSCEPRMQPVCGSNGRTFDNECELKRQSCLLRQELTGIHEGECGEHGPCHTHHCSYGAMCVVKNSLPVCECPTCAEEFEPVCGTDGISYMNACKLKKEACEQKADILADHEGLCNICHTRECDFYAVCESFGNGETRCVCPEVCIKVDAPVCGTDGISYANECELRVEACKKQQYIAVATKGSCDLCERVHCKHGARCESGRCVCPTDCPEAYEFVCSTDGRTHKNECEMKRAG	ATGCGATCCGTGCGAGTTCGTGAGGTGTCCAACGTATCAAGTGTGCCAATTGGACGAGAACCGGAATCCGACTTGCCGGTGCAACGATGTATGCGCGCCAGACTTCAGGACAGTCTGCGGTTCGGATGGAAAGACCTATGCAAACGAATGCTCACTGCGGACAGAAGCCTGCAAAGCACAGAGAAACGTACGCGTCATCTACTCGGGAGACTGCAGTTCAGGTACCAACCCATGCGATCTTATCAAGTGCGGTCCCGGACAAGAATGTGACATTGACCAATTCGGAGTCGCATCCTGCCAGTGTCCACCAAGCTGCGAGCCTAGGATGCAACCAGTTTGTGGGTCCAACGGACGCACTTTCGACAACGAGTGTGAGCTGAAACGGCAGTCCTGTCTCCTTAGACAAGAACTGACGGGTATACATGAAGGAGAATGTGGTGAACACGGACCTTGCCACACACACCACTGCTCTTACGGAGCCATGTGTGTGGTCAAAAATAGCTTGCCTGTTTGTGAGTGTCCGACTTGCGCCGAGGAATTCGAGCCTGTGTGTGGAACTGACGGTATATCCTACATGAATGCATGCAAGCTGAAGAAGGAGGCCTGCGAACAGAAAGCAGACATACTTGCCGATCATGAAGGGCTTTGTAACATCTGTCATACTCGGGAATGTGACTTTTACGCCGTGTGTGAAAGTTTTGGCAACGGAGAGACACGTTGTGTTTGTCCTGAAGTGTGCATAAAGGTGGATGCTCCAGTCTGTGGAACCGATGGTATATCATATGCAAATGAATGCGAACTGCGTGTCGAGGCTTGCAAGAAGCAACAATACATAGCTGTCGCAACCAAGGGATCGTGTGACCTATGCGAACGTGTCCACTGCAAGCACGGAGCCCGTTGTGAAAGTGGTCGATGCGTCTGCCCAACTGACTGTCCAGAGGCGTACGAATTCGTTTGCTCTACCGATGGCCGAACTCACAAGAACGAATGCGAGATGAAAAGAGCTGGTTG

PhdPInSeP53	P0DKT5	7,84E-09	47,826	PF00050	27089.79	GVVKVKRVIKGNSNFADNTIIVEGFGDARICHSDIRERDTRIFFVSEMDNGHLRLNSSVVRVTVTNLDRTVSEVKGLPMKERNILAESECERMYCAYGALCVVDKRTQQAHCRCQETCTDVFAPVCGSDGVTYSSDCQLRMASCSKQKRIFTKHQGPCDLKDPCEEKECHFGAECQPSLDGRSAECLCPAKCATYGDSRSRPVCGTDGKDYPNVCEMRRSSCLEMREIDVRYKGQCDPCEFV	GCGGAGTCGTCAAGGTGAAAAGGGTGATTAAAGGAAATTCTAATTTTGCTGATAATACAATCATCGTGGAAGGTTTCGGTGACGCTCGCATATGTCACAGTGACATCAGAGAAAGAGATACCAGAATATTTTTTGTGAGTGAGATGGACAACGGTCATTTAAGGCTGAATTCTTCCGTGGTCCGAGTAACGGTCACCAATCTGGATCGAACAGTCTCAGAAGTGAAAGGCCTCCCAATGAAGGAAAGAAATATCCTAGCAGAAAGCGAGTGTGAAAGGATGTACTGTGCTTATGGAGCTCTATGTGTGGTAGACAAACGCACTCAACAAGCCCATTGCCGTTGCCAGGAAACTTGTACGGACGTGTTTGCTCCCGTTTGCGGTAGCGATGGTGTAACCTACTCCAGTGATTGCCAACTTCGTATGGCATCCTGCTCCAAACAGAAAAGGATCTTCACGAAGCACCAAGGACCGTGCGATCTAAAGGACCCGTGTGAAGAGAAGGAATGTCATTTTGGAGCAGAGTGCCAACCCTCTCTGGACGGAAGGTCGGCAGAATGTCTATGCCCAGCGAAGTGTGCCACATACGGAGACAGTCGATCGAGGCCTGTCTGTGGTACGGACGGAAAGGATTACCCCAACGTATGCGAGATGAGGAGGTCCTCTTGTTTAGAAATGAGAGAAATTGACGTTCGATACAAGGGTCAATGCGATCCGTGCGAGTTCGTG

PhdPInSeP54	G4V4G1	3,45E-175	93,487	PF00219<br><br>PF00050	28621.53<br><br>**26632.92**	MWIPIIILVLIVPNIRC**ERKCGECDPEKCQPPSEECLAGLVKDLCGCCYVCGRREGELCDGDFLPIPYRNRGHGPCGENLECRPRTDLAPGDLPEALCVCLKTETLCGSDGKTYENECQLTEARYKQRDGLRAMDRGPCKSAPKIISPPEEASNYTGGNIAMSCEATGWPIPVFEWRVDIGDGNTIPLPSDDPKVSVQSRGGPSKYEVTSWLQLLSIQPKDDATYWCIAKNEEGESSAAARVVVLDFKGSQTSQKGRSNDL***	AGAATATCTTTGTGACAAGAAAAGCTTGGCGAGGGGAAAGTTATCCTTGGCGAGGAGAGTCCGAAAACTTTCCGCTAACCCTAGATTCCGTACTCCGAAAGTTGAAAAAGACCTGGGCGTGGAGAGAAAGAATTTCCCCCCTCGCATCTGGTCTGAGTTTGGAGTAAATAAAAGTAGAAAATATGTGGATCCCGATTATAATTCTAGTTTTGATTGTACCAAATATAAGGTGTGAACGAAAATGCGGTGAATGCGATCCCGAAAAATGCCAACCGCCTTCCGAGGAGTGTCTCGCGGGCCTGGTAAAAGACCTCTGCGGGTGCTGTTACGTATGCGGCCGGCGAGAAGGGGAGCTGTGCGACGGTGACTTCTTGCCGATACCATACCGTAACAGGGGACATGGCCCCTGCGGTGAGAACCTAGAATGCAGACCCCGCACCGACCTCGCCCCGGGAGATCTTCCCGAGGCCCTGTGCGTTTGCCTGAAAACCGAAACCTTGTGCGGCAGCGACGGCAAAACTTATGAAAATGAGTGCCAGCTAACCGAAGCGAGATATAAACAGAGGGATGGACTCAGGGCAATGGATAGGGGGCCATGCAAGAGCGCTCCAAAAATTATAAGTCCTCCCGAAGAAGCATCTAACTATACTGGTGGCAATATAGCTATGTCCTGTGAAGCTACAGGCTGGCCTATACCTGTATTTGAATGGAGAGTAGACATTGGTGATGGAAACACAATCCCTTTACCCAGTGATGATCCTAAAGTCTCGGTTCAATCAAGAGGTGGTCCTAGTAAATATGAAGTTACTTCATGGCTTCAGTTACTTAGCATTCAACCTAAAGATGATGCTACTTATTGGTGCATTGCCAAAAATGAAGAAGGAGAATCATCAGCTGCTGCTAGAGTTGTAGTTTTGGATTTTAAAGGAAGCCAAACTTCACAGAAGGGAAGAAGCAATGATTTGTAATCAGATGGACTGAATTTTATATGCATGGTGCTTTCACACAACTGCCATTTTATTTTTTATTTGAAAATTATGTATGTTACTGTAGTTTATACATTTTTATTATTTTGTGAAATGGAAATTATTTTTAAGTGTATGTATATCTTATTTTTAAAACTATAAAATTTGAAAGAATTGTTATTGCTATTCTCACTAAAATGCAGTACTTA

							AGTTTTTACAAATATGGTAAAATGTAAATAAATACAACATATTTGTGTGTTATAAATA
PhdPInSeP55	D5KXH3	0,0000564	33,333		7661,43	DNGLRCKRSSYKGDCKDYEIRVYCECEEEVTCPTGQEWNSCAFNCDKVCQGMRAYLDEEKACDQDQ	AGATAACGGTTTACGATGCAAGCGCAGTTCCTACAAGGGGGATTGCAAAGATTACGAAATACGAGTCTATTGTGAATGTGAGGAAGAAGTGACTTGCCCAACTGGTCAAGAATGGAATTCTTGTGCCTTCAACTGCGATAAAGTTTGCCAAGGCATGAGGGCATATTTAGATGAGGAAAAGGCTTGTGATCAGGATCAGGA

PhdPInSeP56	P83998	3,09E-06	77,273	PF00299	8054.57<br>**6089.04**	MKRSIFLILLMITFVSA**EEGNMNFPVEEEARAPEISEERMPCPKILKPCESDKDCCTGLKCRDLLFGKVCV***	GGATTAGAAATAAACTTTTGTAACGTGCAATCTAATTTTTATTGTGCTGATAACAATCATCAGAAAAAAAATATTTAGTAGGGTAGTCACGAGTAGCAGGAAATAATTTCAAAATTTACGTCATTGTCATCAGTATTTCTTTAAACGCATACCTTTCCGAAAAGCAGATCCCTGCACTTCAAACCTGTGCAGCAATCCTTGTCGCTTTCACATGGCTTGAGGATTTTAGGACAAGGCATCCTTTCTTCACTTATTTCTGGAGCTCTGGCTTCTTCTTCAACTGGAAAATTCATATTCCCCTCTTCTGCGCTCACGAACGTTATCATAAGTAGAATGAGAAAAATTGAACGCTTCATTATACAGCTTTTGAAAATCGCTTCAGAAGAAATCTTCAGAGATTATCAGAAATGCAGCATCTAATTTGCAACAGTCTTGGAATTTATATATATAAATGGGGTTTCATTTCATCATCAAATGACAGGTGCATACTAACCTCACTCCAGATGTTAAAGTACAAAGTATAAAAATAGCTATTCAAATATTAAATAGCTAATTATTCAACAGATGATTTAGCTTCTCATTCGTGACAAAGTAATTTAAAGATAAAAGTGTAATAATAAAACTGCTGTCATTCCAATAAAACCAGAACATATTTTGTGAGGAGAGGCATCTGTTTTGAAAGCTATTTCGCGC
PhdPInSeP57	P83998	1,11E-18	97,059	PF00299	8691.31<br>**6739.74**	MKLSVFLILLLITLVRP**EDANMDFPVEAEDRAPEINEERMPCPKILKQCKSDEDCCRGWKCFGFSIKDKLCISIR***	TTTTGTGCGATTAATGTGGTTTTTACTATTTTTTCAGCTTTCCCGTTACGTGTAAATAATGCATTTTTTTATGCTTGTTTCAGATAATAACCGAGATTAAGAAATACATTTCTGTAACGTGCAATTCATTTTTCTATTGTGCTGATAACAATAAACAAAAAATACAATTTTTAGTAGGGTAGTCACGAGCAGCAAGAAATAATTTCAAAATTTATGTCATTGTCATCAGTATTACTTTATCTTATAGATATGCACAGCTTATCCTTAATCGAAAATCCGAAGCACTTCCAACCTCTGCAGCAATCCTCGTCGCTTTTACATTGCTTGAGGATTTTAGGACAGGGCATCCTTTCTTCATTGATTTCTGGAGCTCTGTCTTCTGCTTCAACTGGAAAATCCATATTCGCATCTTCTGGGCGCACGAGCGTTATCAAAAGTAGAATGAGAAAAACTGAAAGCTTCATTGTA

							CAGGTTTTGGAAATCGTTTCAATGAAAACCCAGAGATTATCA<br>GTAATATAACATCTAAATAGCAAGTCTTGACATTTATATATCT<br>ATATAAGTGGGGTATCATTTCATCATCAAATAACAGGTGCTT<br>A
PhdPInSeP5<br>8	P83998	1,33E-17	97,059	PF00299	8691.31<br><br>**6739.74**	MKLSVFLILLLITLVR<br>P**EDANMDFPVEAE<br>DRAPEINEERMPCP<br>KILKQCKSDEDCCR<br>GWKCFGFSIKDKLC<br>ISIR***	GGAACCTACATTACTCTCATTGTGCAGCGTTCTAAAATGCAC<br>TCACAAGATATAATCCACAAATGCTAAAGAATCTGCAACTT<br>ACTAGAAAATCTTCAAATGCATTACTTTGCATCGTAAATATC<br>ACTGTTTCTTTTAAAAAGGTTCTCAAATATGATCAGAGTAGA<br>GAAATTTTGAAATTGATCGCTTGATATCGATTCCATTATCAGT<br>TAAAATGTTCGAGTATGGAGTAACTAAAAATTATTTGTTTAA<br>TTGATCAGTTTCGCTAACTGCAAATTTAGTTAAAGTGACAAA<br>AAAAAAAGACTATTTACGAAAGAACAAAGAATATGGCACAT<br>TGGAAAACTCTATATTTTATGCTTTTTAGTTTGCCAAAATTTT<br>AATTGAAGATAGAAACGAAAAACAGAAATTGAGCAGGGAC<br>ATTGCATATTTCTTTGCTCTGTATCTCAATTTAAAAGTTTTCTG<br>GTGTCTTAATGTTATTTTTTCTTCATATTTCTTTAAACATGCAT<br>GTTTACTGATTGTTAATCAGACACGAAGAAGTATCATTATCA<br>ATAAATGAAGTAGAAGAATGAAGTAGTAGTAGAATGAATGA<br>TGATTAGAAGTATCATTATCAATTCCATTCGAACCATTGTCAT<br>TTTGTGTTTTTAACGTGGTTGTCAATATTATTTTCTGTTTTCCG<br>GTTCCTTGTAAGTAGTGCATTTCTTTCTTGCGTATAGAGATAT<br>ACTTTTGTAGCAAGGAATTTATTTTTTTTATTGTGCTGATAAC<br>AATCAGCAGAAAATACAATTATTTATTAGAGTAGTCACGAGC<br>AGCAAGAAATAATTTCAAAATTTATGTCATTGTCATCAGTAT

							TACTTTATCTTATAGATATGCACAGCTTATCCTTAATCGAAAATCCGAAGCACTTCCAACCTCTGCAGCAATCCTCGTCGCTTTTACATTGCTTGAGGATTTTAGGACAGGGCATCCTTTCTTCATTGATTTCTGGAGCTCTGTCTTCTGCTTCAACTGGAAAATCCATATTCGCATCTTCTGGGCGCACGAGCGTTATCAAAAGTAGAATGAGAAAAACTGAAAGCTTCATTGTACAGGTTTTGGAAATCGTTTCAATGAAAACCCAGAGATTATCAGTAATATAACATCTAAATAGCAAGTCTTGACATTTATATATCTATATAAGTGGGGTATCATTTCATCATCAAATAACAGGTGCTTA

PhdPInSeP59	P83998	2,78E-05	77,273	PF00299	9288,83	MCSKLAYWVYKESCWRSGNISLIVVMDKKSNLSELQLGIIIGFRDNSDSISQTLHFVNCFSSVVVKVYNYRTIRPIPNIPT*	GGATTAGAAATAAACTTTTGTAACGTGCAATCTAATTTTTATTGTGCTGATAACAATCATCAGAAAAAAAATATTTAGTAGGGTAGTCACGAGTAGCAGGAAATAATTTCAAAATTTACGTCATTGTCATCAGTATTTCTTTAAACGCATACCTTTCCGAAAAGCAGATCCCTGCACTTCAAACCTGTGCAGCAATCCTTGTCGCTTTCACATGGCTTGAGGATTTTAGGACAAGGCATCCTTTCTTCACTTATTTCTGGAGCTCTGGCTTCTTCTTCAACTATAAAAAGAAAAATAAAAGGTTAATAATAGCGCATAGTTATTAGAAGTCTGGAAGTGGTTCATACAATTTTTGCTCATCTTTTGCCACTCTAATTTTGGGAATTCTGTCTTTCATACTCTTTATAACGTCACGGCAGGAGCTGAATACCAGTAATTGCATGTAACCGTGAAATCGAAGTTTTACTTATAGGTAACATTTTAATATATAAAGTTCAAGAAACCACTCACTCCTGCAGGAGTGTTTAAGAAATAAGTAGTATATTTAACAACTACAATGCTCTCGAAGTTGAATGGCAGCATAGGCAGAAAGTCTCAGATCTTCTTGGAAGAAGTGCGATTTCTTTCGAGCTTAAAATGAGGAGTATAATCTTATAGAGAGATTAGGATTATTTTATGAATTCAAATGTTTTCAGTTCTCCTTATGCCATACATGGAATGTATAAAATGCTACTAATATGATTTAGCATGAAAGTGCAGAGATACAAACACTGTATTAATACCGAACCATTCCCTATCTCTGCAAAAATACTATATATTTTAATTCTATGGACGAAGTTAATGACGCTGGGATGATAGATAAAATTAACAGAAATTATTTTTTCCCACGCATTAAGATGAATGCTTTCTTTTGATGCGAGAAAATAATTTGTAGAGATTTCTCTTTCACGCAGTGGAGTATTTTACATTAATTCTTTCCATTTACTAAAAATTTTTTACATAACATATTACTTATATATTTGTGCTTGTAGATTTTTCGGCTGATGCATCTTCTTTAGCATAGGTGCAGTGACCACCAGTTTGTTTCGGAGCCCTATATGCAGCAGGATTCGATTAATTTTCGGTTTAGACACACATTGGTTAATTACCTGGTCCATTTGGTACATGTATCGTTCCTCTGGGCCGTGTT

							GGTGGGGTCTCAGACACATTCGTAGTGGACGTTCATTTCTTGGAAATCAATGGAACTGAGTGCTTCTCATTTTTCACGTCGGTATATTCGGAATGGGGCGAATTGTCCTATAGTTATACACTTTTACCACAACAGAAGAGAAACAGTTCACAAAATGAAGTGTTTGAGAAATACTATCGCTGTTGTCACGAAAACCGATAATAATTCCTAACTGCAACTCTGATAAATTGCTCTTCTTATCCATAACAACAATTAGCGATATGTTTCCAGAACGCCAGCAACTTTCCTTATAAACCCAATAAGCCAATTTACTACACATGTCTTCATTAGCTACGCTCTGCTGATGTTAAATGAAGAAGTTAGTATTATAATCAGTTTCGACTTTGCAGGTTTAATGAAAGTGAGTTCACTCGCAGTTTCTTAATTAGCGGCAATTAAACAGATACTGGAAAAGGAATTTATTTATAAAATATAAATCGGTTTTCCGTTTTCAGAAATTTTAATTTCCAGTACGCGGTGCACTGCAACAATAAACCACACTTCCTCTTTCGTGATGTTGACGTTGATTAGAATAAGTCATAAAGAAGTACATAGATGCTCAATATTGCAATTTTGAGAAAAAGAACAATCAAGACACTGTTACTTGGATCACCGTCAGACAATTTATTCTTCTAGAAAATGACACGAATATGCCATATTACAGGACAAGACAGTCG

						MSFVKSSVKSASYKH<br>TDDGVLSTTTTSSHV<br>SSATMDSSSVTVHH<br>KSTKLTRTAGEREEE<br>ESVSVSYAAKKSKISS<br>AYEADDSSASLIRSA<br>VYSESYMTDVSSEHK<br>GIDVYVAIMDFKPM<br>ESDDEAVTMKEGQQ<br>VEVIDASKPRRWLV<br>RVLPMTADESVKEG<br>WVPACYLEKSTAYD<br>TLGSYVVTEAELDPK<br>ELEALQNREAIVKEL<br>VETEEDFAKDMLFV<br>VENYYKQMDNPRLP<br>KEFRDRKGSIFGNFK<br>DISDFHNNVLMEGI<br>HYHAKEPTKLGKTF<br>LRLERDFDMHANYC<br>WNEARAQQLLREG<br>PLKEYFDDHSRMID<br>DDKLLADHLKLPIQ<br>RLNDYQLLLKELIKY<br>STRLKEDTADLQKA<br>LDFIHSLNKRTKDLQ<br>FIQAIQGYQGDLLKI<br>GKIIRHEAFDALEEN<br>QTTASERYVFLFKGR<br>LFVTEKRSVAEKEIY<br>HVTNLFKLQDVEIVE<br>NVDGDPMGILFRSH<br>KKSRPGFPLTLRARS<br>[illegible]	TTCACAGCTCCATGCGGATTCGCGCGTAGCCTCAGGGATAGA<br>CCGAGACTCGAGCCTTACTTCGGTCACAGACTTTTCGGGCGC<br>AGCCTTTCCCTCGGAGGGCTGCCAACAAAGCAGCAACAGGA<br>TGTCCTTCGTGAAATCCTCTGTCAAGAGTGCCTCATACAAGC<br>ACACGGATGATGGTGTTTTGTCAACAACAACAACTTCGTCCC<br>ATGTCTCATCAGCTACCATGGATTCTTCATCGGTCACCGTCCA<br>TCATAAGTCCACCAAACTAACCAGGACAGCTGGTGAGAGGG<br>AAGAGGAAGAGAGCGTGTCTGTATCCTACGCGGCCAAGAAA<br>TCCAAGATCAGTTCCGCCTATGAGGCCGACGACTCCAGCGCG<br>TCCCTCATCAGGTCTGCTGTTTATTCTGAATCCTATATGACGG<br>ACGTGTCCTCAGAACACAAGGGCATCGATGTGTATGTGGCTA<br>TTATGGATTTCAAGCCCATGGAGAGCGATGACGAAGCAGTT<br>ACCATGAAAGAAGGACAACAGGTTGAAGTGATCGATGCTTC<br>CAAGCCTAGGAGATGGCTGGTACGTGTTCTTCCAATGACTGC<br>CGACGAATCAGTGAAGGAAGGTTGGGTACCGGCCTGTTACTT<br>GGAGAAATCTACGGCTTACGACACCCTCGGCAGCTATGTAGT<br>GACAGAGGCTGAACTCGATCCCAAAGAACTCGAAGCCTTGC<br>AGAATAGAGAGGCCATTGTGAAGGAGCTCGTGGAAACGGAG<br>GAAGATTTTGCAAAGGATATGCTCTTTGTGGTGGAGAACTAC<br>TATAAGCAGATGGACAATCCTAGGCTGCCCAAAGAATTCAG<br>AGATAGGAAGGGTTCTATCTTCGGAAACTTCAAAGACATATC<br>CGATTTTCATAACAATGTATTGATGGAAGGAATCCACTATCA<br>TGCTAAGGAGCCTACAAAACTCGGAAAGACATTTTTGCGTCT<br>GGAGCGAGATTTCGATATGCATGCCAATTACTGCTGGAATGA<br>AGCTCGGGCGCAGCAGCTGTTGAGAGAAGGACCGCTGAAGG<br>AGTATTTTGACGATCACAGCCGCATGATAGACGATGATAAAT<br>TGCTAGCCGACCATCTAAAACTACCTATCCAGAGACTCAACG<br>ACTACCAGCTTCTTCTCAAGGAACTGATCAAATATTCAACTC<br>GACTGAAAGAGGACACGGCCGATCTGCAGAAGGCCTTAGAC<br>TTCATCCACAGCCTCAACAAGCGAACCAAAGACCTGCAGTT<br>CATCCAGGCAATACAAGGATACCAAGGTGATCTACTCAAGA<br>TAGGAAAAATCATCAGACACGAAGCTTTCGATGCCCTCGAA<br>GAGAACCAGACTACAGCTTCCGAGCGGTATGTCTTCCTCTTC<br>[illegible]

						MSFVKSSVKSASYKH	TTCACAGCTCCATGCGGATTCGCGCGTAGCCTCAGGGATAGA
						TDDGVLSTTTTSSHV	CCGAGACTCGAGCCTTACTTCGGTCACAGACTTTTCGGGCGC
						SSATMDSSSVTVHH	AGCCTTTCCCTCGGAGGGCTGCCAACAAAGCAGCAACAGGA
						KSTKLTRTAGEREEE	TGTCCTTCGTGAAATCCTCTGTCAAGAGTGCCTCATACAAGC
						ESVSVSYAAKKSKISS	ACACGGATGATGGTGTTTTGTCAACAACAACAACTTCGTCCC
						AYEADDSSASLIRSA	ATGTCTCATCAGCTACCATGGATTCTTCATCGGTCACCGTCCA
						VYSESYMTDVSSEHK	TCATAAGTCCACCAAACTAACCAGGACAGCTGGTGAGAGGG
						GIDVYYVAIMDFKPM	AAGAGGAAGAGAGCGTGTCTGTATCCTACGCGGCCAAGAAA
						ESDDEAVTMKEGQQ	TCCAAGATCAGTTCCGCCTATGAGGCCGACGACTCCAGCGCG
						VEVIDASKPRRWLV	TCCCTCATCAGGTCTGCTGTTTATTCTGAATCCTATATGACGG
						RVLPMTADESVKEG	ACGTGTCCTCAGAACACAAGGGCATCGATGTGTATGTGGCTA
						WVPACYLEKSTAYD	TTATGGATTTCAAGCCCATGGAGAGCGATGACGAAGCAGTT
						TLGSYVVTEAELDPK	ACCATGAAAGAAGGACAACAGGTTGAAGTGATCGATGCTTC
						ELEALQNREAIVKEL	CAAGCCTAGGAGATGGCTGGTACGTGTTCTTCCAATGACTGC
						VETEEDFAKDMLFV	CGACGAATCAGTGAAGGAAGGTTGGGTACCGGCCTGTTACTT
						VENYYKQMDNPRLP	GGAGAAATCTACGGCTTACGACACCCTCGGCAGCTATGTAGT
						KEFRDRKGSIFGNFK	GACAGAGGCTGAACTCGATCCCAAAGAACTCGAAGCCTTGC
						DISDFHNNVLMEGI	AGAATAGAGAGGCCATTGTGAAGGAGCTCGTGGAAACGGAG
						HYHAKEPTKLGKTF	GAAGATTTTGCAAAGGATATGCTCTTTGTGGTGGAGAACTAC
						LRLERDFDMHANYC	TATAAGCAGATGGACAATCCTAGGCTGCCCAAAGAATTCAG
						WNEARAQQLLREG	AGATAGGAAGGGTTCTATCTTCGGAAACTTCAAAGACATATC
						PLKEYFDDHSRMID	CGATTTTCATAACAATGTATTGATGGAAGGAATCCACTATCA
						DDKLLADHLKLPIQ	TGCTAAGGAGCCTACAAAACTCGGAAAGACATTTTTGCGTCT
						RLNDYQLLLKELIKY	GGAGCGAGATTTCGATATGCATGCCAATTACTGCTGGAATGA
						STRLKEDTADLQKA	AGCTCGGGCGCAGCAGCTGTTGAGAGAAGGACCGCTGAAGG
						LDFIHSLNKRTKDLQ	AGTATTTTGACGATCACAGCCGCATGATAGACGATGATAAAT
						FIQAIQGYQGDLLKI	TGCTAGCCGACCATCTAAAACTACCTATCCAGAGACTCAACG
						GKIIRHEAFDALEEN	ACTACCAGCTTCTTCTCAAGGAACTGATCAAATATTCAACTC
						QTTASERYVFLFKGR	GACTGAAAGAGGACACGGCCGATCTGCAGAAGGCCTTAGAC
						LFVTEKRSVAEKEIY	TTCATCCACAGCCTCAACAAGCGAACCAAAGACCTGCAGTT
						HVTNLFKLQDVEIVE	CATCCAGGCAATACAAGGATACCAAGGTGATCTACTCAAGA
						NVDGDPMGILFRSH	TAGGAAAAATCATCAGACACGAAGCTTTCGATGCCCTCGAA
						KKSRPGFPLTLRARS	GAGAACCAGACTACAGCTTCCGAGCGGTATGTCTTCCTCTTC
						[illegible]	[illegible]

						MDKPRFSKTLRGVT	AAACAAGCTTTTTGCGGACGGTGCGAAGCATTGCTGAATTCC
						CVLGETAVLECETEG	TTGGCTCCATGCCGCTGCCGATCTAACACTTTATTCCTCTGTG
						SPVPSVTWLKDNSKI	GGCAAAAGTAGATCTACTACTTAACATTTTAGCCGAAGAATA
						AKSSRIKTSSQGQKH	TACCCTGTGATATTGCACATTTACAAGAGAACAGGGAAAAA
						TLTLSDATSDDGGLY	AATATACTGCTGTAGTTCTCATAGATTGGCAAGGGCTGCTAT
						TVIASSSKGTASCSAP	TTTAGTCCAAGTGAAATGTGATATTTTCTCGTAAAATAATAC
						LNLKIPLLGVPKEDS	AGCTTCTTAATAGAGGAATTCACGCATCACCTACGGGATAAT
						RPTTPGGTVLPHAP	TTGATAATTCTGCTTGACTCTATCTTCTCTTCCTTCTGTGTTAT
						VFKVKLNKETQLLE	ATTTGTGGTCATACATTCCAGGATACATTTTTTCCTTTTTCACT
						GTSVRFELVVRGNPE	GTTGAAGATTTAGGAGATCTCGAACCCAGCATCGAACGACC
						PQVKFFKDGKELKL	TATCGAACTTAAAGATACTGATAACTTAGAACTTAAACATAA
						DDRVRVSYESKEVFE	GGCCGAAGAGTTAGAAGAGCCAAGTTTCAAAAAACCGAAA
						LILDHVVAKDAGTY	GTCGAATCTTTGAAGAGACAAGAATCCGTGAAGAAGAAACC
						SCVATNSEGEDKTV	TGCGAAACCGGGTGACTCTGCGCCGCAAACTCCTACAGAGG
						GNLSVVKNKDVFKG	AAGAAACTAAACCCCCAGCGCTTTCCCGGCAAGAGTCAGTG
						LEELEEEREPSPRPKT	AAAAAGAAATCTAAGACAGACTCTACCCCTCAGACACCTAC
						PKFKWFKDGQYFEA	GGACACTGACAAACCTCCCAAACTCTCCAGGCAGGAGTCCG
						NERFQVIFNEEEDSL	TGAAGAAAAAATCCAAATCGGCTGAATCGTCAGCACCACAA
						ALMFQHVTPEDAGL	ACACCCGTTGAAGAGACTGCAGCACCTGCCTTAAAGCGACA
						YTCVASTSTGSKIAC	AGATTCGGTGAAAAAGAAACCCAAGTCTGCAGAATCCTCTG
						SAELTVQGVIRQVEA	TTCCGCAGACGCCTACGGACGAAACCCCAGCTCCAGCGTTGT
						PGIKAHLSDAEATE	CTAGACAAGATTCTGTTAAAAAGTCGAAAAAGACATCGGTC
						GGSAMLELKITGFPK	ACTGACAAAGGCGTAGATTTGAAGGAAACCGTACCCGAAGA
						PKVTWYHKNKEVST	AACTGTTTCCGGCATCACTCGACAAGAATCGGTAAGCAAGA
						GGRFRFLFADEESFT	AAGATAAAAAATCTTCAGTGACTGAGAAGGGTGAGGTCCAG
						LVIKNVKKDDSGKY	CCTGAGACTAAGGTGAAGAGACAAGACTCTCGGGATAAGAA
						TVKAVNEMGEAESS	GAGCACCGAGGCCACCACAAAACAGCCCGTGAAGCGTCAG
						CTLSVKSAPSFIKGLK	GATTCAGGAAAGGATATCTCGGAAAAGAAACGAGAGGTCG
						DMSVMAEETIKFEA	ATCCGATGGATAAGCCACGTTTTTCCAAGACTCTCAGAGGAG
						QIDGNPAPEIKWYK	TTACATGTGTTCTGGGCGAGACGGCCGTCTTAGAATGCGAAA
						DGQQLSPSDRIKISSD	CTGAAGGCAGTCCTGTGCCTTCTGTGACATGGCTGAAAGACA
						SGKISLQILKAKPTDS	ATTCCAAGATAGCTAAATCATCACGCATTAAGACCTCTTCTC
						GSYSCVLTNSIGSQA	AGGGCCAGAAGCATACGCTGACGCTGAGCGATGCGACATCG
						[illegible]	[illegible]

PhdPInSeP63	G4V4G1	1,55E-05	27,523	PF00219<br><br>PF00050	21148,95	TDWGSDFVELTWPAPTNDGGAPVSSYIIQKRVRGSNLWEKGTEVPGSDTKGKVPYLIEGQDYEFRIIAVNKGGESEPSEASEFITAKPRFLAPKIITPMKDVEVKNGGVLHIEIKFIGTPTPEISWTLDDKPLVTDKRVTVSNYENYTLVHTINTKRLDTGLYCLKLKNDSGTDEGILKVTVLDKPGTPEGP	AGGTCCTTCTGGAGTACCTGGTTTGTCTAATACAGTTACTTTCAATATGCCTTCGTCAGTACCAGAATCATTTTTCAATTTCAAACAATATAAGCCAGTATCCAGCCTCTTTGTGTTGATAGTATGGACCAGAGTGTAGTTTTCATAATTAGACACAGTTACTCTCTTATCTGTTACCAAAGGTTTGTCATCAAGAGTCCAAGAGATTTCTGGTGTTGGAGTTCCAATGAATTTAATTTCAATATGCAACACTCCACCATTCTTCACTTCCACATCTTTCATTGGTGTAATAATCTTAGGTGCCAAGAATCTGGGCTTAGCAGTGATGAACTCAGATGCCTCACTAGGTTCACTTTCACCTCCTTTGTTGACAGCAATAATCCTGAATTCATAGTCCTGTCCTTCAATGAGGTAAGGAACTTTGCCCTTAGTATCCGATCCAGGAACTTCTGTTCCTTTCTCCCATAAGTTAGATCCACGGACTCTCTTTTGAATGATGTAACTAGACACTGGTGCACCACCATCATTTGTAGGTGCTGGCCAAGTAAGTTCAACAAAGTCACTACCCCAGTCAGTA

						MSEDASGSDARIESV	AAACAAGCTTTTTGCGGACGGTGCGAAGCATTGCTGAATTCC
						DFNEDNATLASESL	TTGGCTCCATGCCGCTGCCGATCTAACACTTTATTCCTCTGTG
						RSDDSVTPPASEVDP	GGCAAAAGTAGATCTACTACTTAACATTTTAGCCGAAGAATA
						MDKPRFSKTLRGVT	TACCCTGTGATATTGCACATTTACAAGAGAACAGGGAAAAA
						CVLGETAVLECETEG	AATATACTGCTGTAGTTCTCATAGATTGGCAAGGGCTGCTAT
						SPVPSVTWLKDNSKI	TTTAGTCCAAGTGAAATGTGATATTTTCTCGTAAAATAATAC
						AKSSRIKTSSQGQKH	AGCTTCTTAATAGAGGAATTCACGCATCACCTACGGGATAAT
						TLTLSDATSDDGGLY	TTGATAATTCTGCTTGACTCTATCTTCTCTTCCTTCTGTGTTAT
						TVIASSSKGTASCSAP	ATTTGTGGTCATACATTCCAGGATACATTTTTTCCTTTTTCACT
						LNLKIPLLGVPKEDS	GTTGAAGATTTAGGAGATCTCGAACCCAGCATCGAACGACC
						RPTTPGGTVLPHAP	TATCGAACTTAAAGATACTGATAACTTAGAACTTAAACATAA
						VFKVKLNKETQLLE	GGCCGAAGAGTTAGAAGAGCCAAGTTTCAAAAAACCGAAA
						GTSVRFELVVRGNPE	GTCGAATCTTTGAAGAGACAAGAATCCGTGAAGAAGAAACC
						PQVKFFKDGKELKL	TGCGAAACCGGGTGACTCTGCGCCGCAAACTCCTACAGAGG
						DDRVRVSYESKEVFE	AAGAAACTAAACCCCCAGCGCTTTCCCGGCAAGAGTCAGTG
						LILDHVVAKDAGTY	AAAAAGAAATCTAAGACAGACTCTACCCCTCAGACACCTAC
						SCVATNSEGEDKTV	GGACACTGACAAACCTCCCAAACTCTCCAGGCAGGAGTCCG
						GNLSVVKNKDVFKG	TGAAGAAAAAATCCAAATCGGCTGAATCGTCAGCACCACAA
						LEELEEEREPSPRPKT	ACACCCGTTGAAGAGACTGCAGCACCTGCCTTAAAGCGACA
						PKFKWFKDGQYFEA	AGATTCGGTGAAAAAGAAACCCAAGTCTGCAGAATCCTCTG
						NERFQVIFNEEEDSL	TTCCGCAGACGCCTACGGACGAAACCCCAGCTCCAGCGTTGT
						ALMFQHVTPEDAGL	CTAGACAAGATTCTGTTAAAAAGTCGAAAAAGACATCGGTC
						YTCVASTSTGSKIAC	ACTGACAAAGGCGTAGATTTGAAGGAAACCGTACCCGAAGA
						SAELTVQGVIRQVEA	AACTGTTTCCGGCATCACTCGACAAGAATCGGTAAGCAAGA
						PGIKAHLSDAEATE	AAGATAAAAAATCTTCAGTGACTGAGAAGGGTGAGGTCCAG
						GGSAMLELKITGFPK	CCTGAGACTAAGGTGAAGAGACAAGACTCTCGGGATAAGAA
						PKVTWYHKNKEVST	GAGCACCGAGGCCACCACAAAACAGCCCGTGAAGCGTCAG
						GGRFRFLFADEESFT	GATTCAGGAAAGGATATCTCGGAAAAGAAACGAGGTGAGG
						LVIKNVKKDDSGKY	ATCTGATTCCTAAGATAATCATGTCTGAGGATGCTTCAGGTTC
						TVKAVNEMGEAESS	TGATGCAAGAATAGAGAGTGTGGATTTTAACGAAGATAATG
						CTLSVKSAPSFIKGLK	CTACCCTAGCCGAGCGAGAGCCTCCGTTCGGATGACAGTGTGA
						DMSVMAEETIKFEA	CCCCTCCTGCAAGTGAGGTCGATCCGATGGATAAGCCACGTT
						QIDGNPAPEIKWYK	TTTCCAAGACTCTCAGAGGAGTTACATGTGTTCTGGGCGAGA
						[illegible]	[illegible]

PhdPInSeP65	P0DM56	1,24E-05	45,902	PF01826	151169,14	ETVHCDPLPCTHPEE<br>LQGHCCPVCAPEIL<br>CHYLGKTYEAWENF<br>IDPTNPCSECVCIEG<br>MVSCHLVLCLNSEC<br>PNPQLGHCCQSCDG<br>CSYGEKNYINHISFS<br>DPFNECRDCKCNN<br>GVVSCQQKSCTSPSC<br>QHPTLMESECCPTC<br>GDALECIFKDNSYM<br>DGETFYDPDDSCVE<br>CFCQFPDVSCHVYD<br>CDEDSCSDPLRSHCC<br>KSCSDCTFMDFTYEA<br>GSTFTNVQNPCEEC<br>VCQEGRVTCFEKPC<br>PAVLCTHPVHSDCC<br>FVCNEGCLFENRIYN<br>ELDVFQDPENPCQE<br>CFCQNGSILCQNKK<br>CAKVTCKYPARGEC<br>CSVCHDCFYKNELV<br>KNRQKIDHDGSDVC<br>KECLCSNGNIECQH<br>KFCPTVYCSDPVIQD<br>CCPVCHLGCFYGGEI<br>YKFGAEFDHPNDSC	GAAACAGTTCACTGTGATCCTCTGCCATGTACTCATCCGGAA<br>GAATTACAAGGCCATTGCTGTCCAGTGTGCGCCCCAGAAATC<br>CTTTGTCATTACCTTGGAAAAACATACGAAGCCTGGGAAAAC<br>TTCATAGACCCCACAAATCCCTGCAGTGAATGTGTTTGCATC<br>GAAGGGATGGTTAGTTGTCACCTGGTATTGTGCCTCAATTCG<br>GAATGTCCAAATCCTCAGTTGGGACATTGTTGTCAATCTTGTG<br>ATGGTTGTTCATATGGAGAGAAGAATTACATCAATCACATCT<br>CATTCAGCGATCCATTCAATGAGTGTCGTGATTGCAAATGCA<br>ATAATGGAGTTGTGTCATGCCAGCAAAAATCGTGTACTTCTC<br>CTTCATGCCAACATCCAACGCTGATGGAAAGTGAATGTTGTC<br>CAACCTGTGGAGATGCATTAGAGTGTATTTCAAAGACAATT<br>CCTACATGGATGGAGAAACCTTTTACGATCCTGATGATTCAT<br>GTGTTGAGTGCTTTTGCCAGTTTCCCGATGTTTCATGTCACGT<br>CTATGATTGTGATGAAGACAGCTGCAGTGATCCACTTCGATC<br>ACACTGTTGTAAAAGTTGTTCAGATTGTACCTTTATGGACTTT<br>ACCTATGAAGCTGGTTCTACGTTTACAAATGTTCAGAATCCC<br>TGCGAGGAGTGTGTCTGTCAGGAAGGAAGAGTTACTTGCTTT<br>GAGAAACCTTGTCCTGCTGTCCTGTGTACCCATCCTGTTCATT<br>CCGATTGTTGTTTCGTGTGCAACGAAGGATGTTTGTTTGAAAA<br>TAGGATTTATAATGAACTCGATGTGTTTCAAGATCCTGAAAA<br>TCCTTGCCAAGAATGTTTTTGCCAAAATGGCTCTATTCTTTGT<br>CAGAATAAAAAATGTGCCAAGGTTACTTGTAAATATCCAGC<br>AAGAGGCGAGTGCTGCTCAGTTTGTCATGATTGCTTCTACAA<br>GAACGAACTAGTTAAAAACAGACAAAAAATTGATCACGATG<br>GTTCCGATGTATGTAAAGAATGCCTTTGCTCTAATGGTAATAT<br>TGAATGCCAACATAAATTTTGTCCAACTGTTTACTGTTCTGAT<br>CCAGTCATCCAAGACTGTTGTCCTGTCTGCCATCTAGGTTGCT<br>TCTATGGTGGCGAGATTTACAAATTTGGCGCTGAATTTGATC

						SECACMEGSVHCDQ<br>KPCEHLSCKHLITDS<br>QHCCPYCPDYCIYD<br>GQRYGNNSVFADAT<br>DVCKECKCLSGDVF<br>CERKMCPKINCLHP<br>ALGECCLECEKCDF<br>HGEIYTDGKNFTNP<br>NNSCETCLCTKGQV<br>ACRRNPCVQCLHPL<br>LTDFNCCPLCDGCF<br>YKGINYTNQELFIDP<br>GNACEECICKSGTVT<br>CHELSCSLPSCSNPV<br>YLPEHCCPVCPGCSF<br>EGTQFEENEEFTHPG<br>NVCEKCICKDSHVA<br>CKTQICHRNCTHPT<br>PDPCCPICDNCMFQ<br>TIRYTNGERFNPDPC<br>QTCECKNGNVLCIS<br>EQCPELHCQLRIRLP<br>GVCCETCRGCFYEG<br>NEYENGETWFSSTN<br>PCLSCSCNGGTVSC<br>QNIVCPIQCSEPVPV<br>PGLCCPICPECEKDG<br>KKYKSGEVFSSDDYP	ACCCAAATGACTCTTGTTCTGAATGTGCTTGCATGGAAGGTT<br>CGGTTCATTGCGATCAAAAACCATGTGAACATCTGAGTTGCA<br>AGCACCTGATTACTGACTCACAACACTGCTGCCCTTATTGTC<br>CAGATTATTGCATTTATGATGGCCAAAGATATGGCAATAATT<br>CTGTATTCGCAGACGCAACAGATGTTTGTAAAGAATGCAAAT<br>GCTTGTCAGGTGATGTATTCTGTGAGCGTAAGATGTGCCCTA<br>AGATCAACTGTTTGCATCCAGCTCTTGGAGAATGTTGCTTGG<br>AATGCGAAAAGTGCGACTTCCATGGTGAAATTTACACAGAT<br>GGAAAAAACTTCACAAATCCTAATAATTCTTGCGAAACTTGT<br>CTCTGCACGAAAGGCCAGGTAGCCTGTAGGAGAAATCCTTGT<br>GTCCAGTGCCTTCATCCACTTCTAACTGATTTCAACTGCTGCC<br>CATTGTGTGATGGATGTTTCTATAAAGGAATAAATTACACCA<br>ACCAAGAACTTTTTATTGATCCTGGAAACGCCTGTGAAGAAT<br>GTATCTGCAAGAGCGGCACTGTGACATGTCATGAGTTATCAT<br>GCAGTTTGCCATCTTGTTCGAATCCTGTTTACCTTCCGGAACA<br>CTGTTGCCCTGTGTGTCCAGGATGCAGTTTTGAAGGCACTCA<br>GTTTGAAGAAAATGAAGAGTTTACACATCCTGGTAATGTGTG<br>TGAAAAATGCATTTGTAAGGATAGTCACGTGGCTTGCAAGAC<br>ACAAATATGTCATCGAAACTGCACGCATCCAACACCTGATCC<br>TTGTTGCCCTATTTGTGACAACTGCATGTTTCAGACTATCAGA<br>TATACAAATGGAGAAAGATTTAATCCTGATCCATGTCAAACG<br>TGTGAATGTAAGAATGGCAATGTATTGTGCATCTCTGAGCAA<br>TGTCCAGAACTTCATTGTCAGTTAAGGATACGGCTTCCAGGC<br>GTATGTTGCGAAACTTGTCGAGGTTGCTTTTATGAGGGAAAT<br>GAGTATGAAAATGGGGAAACTTGGTTTTCCTCCACAAACCCT<br>TGTTTATCTTGTTCATGCAACGGTGGCACTGTATCTTGCCAAA<br>ATATTGTTTGTCCAATTCAATGTTCAGAACCAGTACCAGTTCC<br>TGGCCTATGCTGTCCAATTTGTCCAGAATGCGAAAAAGATGG

						CSSCTCDSGSIFCIRH<br>ECPTLVGCPEEAIKP<br>PGEGECCPTCAAFG<br>TSCTRNQIGKLVYPS<br>GDPCFSCQCTENFM<br>WVCSKEFCPHLNCP<br>VDEIQTLETECCPKC<br>KVCLSEGLETHYHG<br>DTWTSPNDVCSSCF<br>CDYGFIKCNKIACPP<br>VRCETGQIEFLSSTEC<br>CSICISSSELSCDYGG<br>SYYEHRQIWNPDPC<br>TTCQCLEGKMNCV<br>NERCLPLHCSSDEIK<br>SADPGQCCPKCIPRP<br>ATCIAFGDPHYKTFD<br>GAAVSFQGTCRYVL<br>TADCSTQQFNVLVE<br>NNDRGFNGVSWTH<br>RVVVVMQNASVDL<br>RAGPSIYVRGKHIEH<br>LPYFEDPVLYVEKTG<br>SSVLVTTNVGVKVL<br>WNGDGYVEVIVSGA<br>YRDKTCGLCGNFNN<br>FPEDDMRTPTNEIVS<br>SEAKFGNSWKNPGV	TAAAAAATACAAATCAGGTGAAGTCTTTTCTTCGGACGACTA<br>TCCTTGTAGTTCCTGCACTTGTGATTCTGGAAGTATATTTTGC<br>ATACGTCACGAATGCCCGACGCTAGTTGGCTGCCCTGAAGA<br>GGCCATTAAACCACCTGGAGAAGGAGAATGTTGCCCGACAT<br>GTGCAGCATTTGGAACATCTTGTACAAGAAATCAAATTGGAA<br>AATTAGTATACCCTTCAGGAGACCCATGCTTCTCTTGTCAATG<br>TACAGAAAATTTTATGTGGGTATGCTCGAAAGAGTTTTGTCCT<br>CATCTCAACTGCCCAGTGGATGAAATCCAAACTTTAGAGACA<br>GAGTGTTGTCCAAAGTGCAAAGTGTGCCTTTCTGAAGGATTG<br>GAGACCCATTATCATGGAGATACATGGACTTCCCCAAATGAC<br>GTCTGTAGCTCTTGTTTTTGCGACTATGGCTTCATTAAATGCA<br>ACAAGATTGCATGCCCTCCAGTTCGGTGTGAAACTGGGCAGA<br>TCGAATTTCTTTCTTCTACAGAATGCTGCTCAATTTGTATCTCT<br>TCTAGTGAACTTTCTTGTGATTACGGGGGATCTTATTACGAAC<br>ATAGACAAATATGGAACCCAGATCCTTGCACAACATGTCAG<br>TGTTTAGAAGGAAAAATGAACTGCGTCAATGAACGATGTCTT<br>CCTCTTCATTGTTCTTCAGATGAAATTAAATCGGCTGATCCTG<br>GCCAGTGTTGTCCCAAATGCATCCCTCGTCCAGCAACGTGCA<br>TTGCTTTTGGAGATCCTCATTACAAAACTTTTGATGGTGCAGC<br>TGTCAGTTTTCAAGGGACGTGTCGTTATGTTCTCACAGCAGAT<br>TGTTCTACGCAGCAGTTCAATGTGCTGGTGGAAAATAATGAT<br>CGTGGCTTCAATGGTGTTTCATGGACTCACAGGGTCGTCGTTG<br>TTATGCAGAACGCATCAGTAGATTTACGAGCAGGACCATCC<br>ATATATGTTCGAGGAAAACACATAGAACATCTGCCGTATTTT<br>GAAGATCCTGTCCTTTACGTAGAAAAAACTGGATCTTCAGTT<br>CTTGTCACTACCAACGTAGGAGTCAAGGTGCTATGGAATGGT<br>GATGGTTATGTCGAAGTGATTGTTTCTGGTGCCTATAGAGAT<br>AAAACTTGTGGATTGTGTGGAAATTTTAATAATTTTCCTGAGG

						QPEGCTESVDIDPCSKGGYRVRKIANSKCSILKQSLFASCHRFIPPEPYFAACVYDVCACSENDDCLCDILSAYSQECSKAGVIVHWRSEGLCGIRCSREEGFFFDECGPPCPRTCENRNVPIGILAAQCFKPCVPGCQCSADKVLHNGRCIRPDKCP*	ACGATATGCGAACACCTACGAATGAAATTGTATCATCAGAAGCTAAATTTGGAAACAGTTGGAAAAATCCTGGCGTTCAACCTGAGGGTTGTACTGAATCTGTGGACATTGATCCTTGCTCAAAAGGAGGATACAGGGTCCGTAAAATAGCAAATAGCAAATGTTCCATTTTGAAACAAAGTTTATTCGCTTCCTGTCATCGATTCATTCCTCCTGAGCCCTATTTCGCGGCTTGTGTTTATGATGTTTGTGCTTGCTCAGAAAATGACGACTGCCTCTGTGATATTTTGTCAGCTTACTCCCAGGAATGTTCAAAAGCCGGTGTTATTGTTCACTGGAGAAGTGAAGGACTCTGTGGTATCCGTTGTTCCCGTGAAGAAGGATTTTTTTTTGATGAATGTGGTCCTCCGTGTCCCAGAACATGTGAGAATCGAAACGTTCCCATCGGAATTTTAGCTGCGCAGTGTTTCAAGCCATGTGTTCCAGGATGTCAGTGTTCCGCTGACAAAGTGCTTCATAATGGGCGATGTATTCGCCCTGACAAGTGCCCTTAAATTCAAAGTGTTTCCATCTGTTTTTATGAAGTCCTAAGTCACCCATTTGCGTCCACTGCGAGAGTTTTCTGAAATTTGATCTTCCCCGATTGGCTGTAGTTGAATAATGTGAATGCAATACAATTTTAATAATATTATTTTTGATTCTATATTACAATGTGCATGATATCACTTTCTATGACAAATTATGGTATATAACAATGCAAGTTTTTAAAAAATGAAAATATAACTATAGATCCATTTTTATGTATATTTTTTCTAGGAATGTCACGTCATTATTAAGTTGCATCGATATATACTATTAG

PhdPInSeP66	G4V4G1	2,82E-10	31,405	PF00219<br>PF00050	165766.14<br>**162818.45**	MGARLIPGLRTLVFQ<br>ICLLVLLEMAHG**QC**<br>**PAKCLCFRTTVRCM**<br>**FLQLDSVPEVPAET**<br>**TILDLRFNKIREIPR**<br>**NAFKSLKDLNTLLL**<br>**NNNHITKIEDEAFA**<br>**GLEELRYLYLYKNKI**<br>**KTIGSTAFKGLRNL**<br>**EQLYIHFNKIERLEP**<br>**DTFADLPSLERLFLH**<br>**NNRLSKIPYGAFKH**<br>**LKSLRRLRLDSNALI**<br>**CDCEIMWLAEMLK**<br>**ESHAATQAAATCE**<br>**YPNNLQGKSLMAL**<br>**NSEDFRCKRPKLTE**<br>**EPNDVDVSFGGTA**<br>**YFTCKADGDPKPDI**<br>**VWLHNSNEISTEEE**<br>**ERYSILTDGTLMITE**<br>**TQDLDAGVYECMA**<br>**KNPAGETKSRAAK**<br>**MHYLKGRENKSKG**<br>**ILHFTQQPTDSTVP**<br>**VGSTIVLPCSATSDI**<br>**DTSINWTQNGIALP**<br>**RIFRYYISSSGDLTIK**	AGTAGATATAAAATATGCAGTCTTAATGTATATCTATACAAT<br>AAATTTCTTATTTGTAAGCTATAATGAAAAAAAAGTTGCACA<br>AACTAAATTTCCCTACTATGTGAGACAACTGCTTATTTACATG<br>TTAAAACAAACAATAACAAAAAAAAAACAATTATTGGCAGT<br>CAAATAGCTATTCAATTCAAATTCAACAAACTGGGCAACATT<br>CTCCATCAATAATTCTTGGCTTTTGACATTCACTAGGCTGACA<br>TGTTTCAACCCAGCAAGTTATCTGGTTATTCTCACAACGACAT<br>TTTGTGCATTTTTCTTTTTGCCACAATTCTCCTGAATGTCTTTTC<br>TTTCTGTCGTGATCAAAACATCCACCTTCATTTCGAAATTTAC<br>TCTCAGCTTCTAACTTTCTTACTTTTCGACTTAATTTTTGAACA<br>GTTTTTTCAAGTCTTCCAACTAATTCTTCAATACCTTCAATTCT<br>CTCTTCTGTTATGTCAAAATCATCTTCTTGCCAATCACGTGAT<br>GTCTTCCTTGTTGCATTACGATACCTATGAGATTCATCAGCAT<br>GGTCATAACGATCGACGAAAGATCTTCTATTGCGAGTGTTGA<br>ATCTATTTATTTCATTACTTTGGGATGCACTGCATTCTTGACA<br>GCATTCTGTCCATACATTTAACTCAATTTTAGGTATAGATGAA<br>CAGTCAGCAAAATTGGGATTTTGATGTTGAGGTACTGTAAAC<br>ACATCTTTGGTAACAACAGTGATATTGTCACCACTATCACAC<br>AGCACTCGGCCTAAACTGCTCTGTTTTATCTGAACTAATTGGT<br>CTGGAGAAAAGATTCCTGGATTCTCATACCAAAATCTGTCAC<br>CACTTCGCAGTCTTTTAAACTGATCAATCAGCATACAAAGAA<br>ATGTTGGTCCAACTTTTGCATCTTTGACAGGTTCTTCACTAAT<br>TCCTCCAACCCAAACATCAATGTTGTCTGGATGGCCATAAAG<br>ACTTTTAATTTTTCTCAATAAAGCTAGATCTCGTATTTCTCCAT<br>GCAAATCTTCAAACGTAGTTGCAACTGACAGATTACAGTATC<br>TTCTCCATTCATTGTATGATGGGAGACCATGATCTCTACCTCT<br>CTGTATATTTAAGGCTGCTAAATCTTGAGCTACTAAGTGGGC<br>CGGCGTAAAGAGATGTTCTGTTAAATCTGAATTCAACAATTG

						**NVQRNDAGVYQCI**	ATCAGGCCTCTTTAATTTGGCTGCTCCATAAATCAAACCTCTC
						**ASNSESSIAVSVKID**	ATCAATGGATCAATGCCACCTTCCGTTAAAAGCCTGAATGGT
						**IAAPPVFLERPRDQE**	GCAAAAAAGGCTTTTCTTAGAGGAATATTACCTTGAGGTATT
						**AIEGNNVELLCQVE**	GGCTGAAATGTTTCATTCAAACGTAGCAATTCAGGATTTATC
						**GIPPPVLTWTRRDL**	AGAGTATGACCAAATCTCAAAGCAGCTGTTGCAAATACATTT
						**PLRTGSRLQLLKDS**	GAAATAGATGGATTGATGGTGGGATCAAATCCTTCATATTGT
						**SVLSIQRVNKFDEG**	CCAAGTTGAGCATAGCCATCTTTCCCCAATACTTTAGGTAAC
						**TYTCHAENPAGVR**	CAATGTGCATAAGTTATGTGCTGCATTACAGCTCCAACAATT
						**NSSALLIIREKVPPV**	TTTCTTGCCTCATGATAGATTTTATCATTATCCCAATGAGGAT
						**FTRTPEDQHTNIGS**	TAAAACTGTACAGGTCTCTAGCAATACGATTGTGTTCTCTCAT
						**SVELPCHAKGQPLP**	CCATAGTGTGTGCATTGCCAAAAGTCCAAGTTGCTCATTGGC
						**HIKWKKDGILMSSE**	TCTGACATCACCTGCCAAGAAGCAATCAATACTACTTTCAGT
						**SNHHRVSSNGDLY**	CAAATCTCTTTTGCAGTCAATGGGTTGCCCATCATTAAATGGC
						**MFNIQPADDGVYE**	AGCAATGGCTTTCCTGTGTGCATTAGCACTCCCGATCTTAAC
						**CIAENDIGTASVSM**	AGCCCTCTTCGAGTGCTCATTTCTCTCAGATGACGGGCAATTC
						**RLTVVSDSPYIGRP**	GCTCATTACTTCCATAAACATTAGAAGCATCAATAAATGCTG
						**GDRYIVSAFDEARQ**	TTAGCTGGTTAACCTGTTCACGAGGCTGGACAGTATTAAAAA
						**EVDNALNHTLFELF**	ATACAGATGTCAACCCAGATCCACAAACAGCACTGGAACGA
						**GDSHQRTPGELLQ**	ACAAATTCCATGCATCTGACTCTATTAATTCGTCTGTCATCCA
						**AFRFPTNEARESAR**	ATGGTACTTCAATAGGAAAACATGGAGTGGCAAGTTCACAT
						**AAEIYERALEIVWK**	GAAGTAGCACAATCCACTCCATCAACAAAACTTGCATGACTT
						**HAENGAQFNLSEF**	ATAGATGGCATTGAAAAGTCAAGATCATGGTCTAAAAATTGT
						**NYKDILSLQKLELL**	CCCCACTGCATAAGCATATGTGAATATTCGCTATCTTTGGAC
						**ANLSGCLVHRESSS**	ACAAAATCTGTTGATACAATTCTAGTTGAAATTAGGCGGGCA
						**CSDMCFHSKYRSFD**	CTTGGTTTAGAAAATCCATTATACAGATGTGATCTAGACCAA
						**GRCNNFNNPRWGS**	CCAATGGGTGTATTAAATCCATTTTCATACAGAGCAGGCAAC
						**SLTPFVRLLPALYEN**	AAACGGACAAATGGAGTTAAGGAGGATCCCCATCTTGGATT
						**GFNTPIGWSRSHLY**	ATTAAAGTTGTTGCACCGCCCATCAAAGCTTCGATATTTGCTG

						**NGFSKPSARLISTRI**	TGAAAGCACATATCTGAACAACTTGAAGATTCTCGATGGAC
						**VSTDFVSKDSEYSH**	AAGACAACCAGACAAATTAGCAAGTAATTCTAACTTTTGTAG
						**MLMQWGQFLDHD**	GGATAATATATCTTTGTAGTTGAATTCTGATAAGTTGAATTGA
						**LDFSMPSISHASFV**	GCCCCATTTTCTGCATGCTTCCAGACTATTTCTAAAGCTCTCT
						**DGVDCATSCELATP**	CGTAGATTTCTGCAGCTCTTGCAGACTCACGAGCTTCATTGGT
						**CFPIEVPLDDRRINR**	TGGAAATCGGAAAGCCTGTAACAATTCTCCAGGAGTTCGTTG
						**VRCMEFVRSSAVC**	GTGAGAATCACCAAATAACTCGAACAAAGTATGATTCAAAG
						**GSGLTSVFFNTVQP**	CATTATCAACTTCTTGACGAGCTTCATCAAATGCTGAAACTA
						**REQVNQLTAFIDAS**	TGTACCTATCCCCAGGTCTTCCAATATAAGGACTATCTGACA
						**NVYGSNERIARHLR**	CAACAGTTAGTCTCATTGACACAGATGCTGTTCCAATATCGT
						**EMSTRRGLLRSGVL**	TTTCTGCTATGCATTCATATACTCCATCATCTGCTGGTTGGAT
						**MHTGKPLLPFNDG**	ATTAAACATATAGAGATCACCATTGGAGCTTACTCTGTGATG
						**QPIDCKRDLTESSID**	ATTTGATTCTGATGACATGAGTATACCATCTTTTTTCCATTTTA
						**CFLAGDVRANEQL**	TATGTGGCAAAGGTTGACCTTTGGCATGACATGGTAGTTCAA
						**GLLAMHTLWMREH**	CACTTGATCCAATATTTGTATGCTGGTCTTCAGGTGTTCGAGT
						**NRIARDLYSFNPHW**	AAATACTGGCGGTACTTTTTCTCTGATAATAAGTAAAGCACT
						**DNDKIYHEARKIVG**	GGAGTTCCTTACTCCGGCAGGATTTTCCGCATGGCATGTATA
						**AVMQHITYAHWLP**	GGTTCCTTCATCAAATTTATTAACTCGTTGAATGCTTAGAACA
						**KVLGKDGYAQLGQ**	GAAGAATCTTTCAATAATTGCAATCTGCTTCCCGTTCTTAAAG
						**YEGFDPTINPSISNV**	GAAGATCCCTTCTTGTCCATGTCAAAACTGGTGGGGTATTC
						**FATAALRFGHTLIN**	CTTCTACTTGGCACAAGAGCTCTACGTTGTTTCCTTCAATGGC
						**PELLRLNETFQPIPQ**	CTCCTGATCCCTTGGTCTCTCGAGGAACACCGGTGGGGCTGC
						**GNIPLRKAFFAPFRL**	AATGTCAATTTTCACTGAAACTGCAATGCTAGATTCTGAATT
						**LTEGGIDPLMRGLI**	GGATGCAATGCACTGGTAAACTCCAGCATCATTTCGCTGAAC
						**YGAAKLKRPDQLL**	ATTTTTTATTGTCAGATCACCTGAAGAGGAGATATAATAGCG
						**NSDLTEHLFTPAHL**	GAAAATTCTTGGCAATGCAATGCCATTCTGTGTCCAGTTGAT
						**VAQDLAALNIQRG**	GGATGTGTCAATATCACTTGTAGCACTGCAAGGAAGAACTAT
						**RDHGLPSYNEWRR**	TGTAGATCCCACAGGTACTGTACTATCAGTTGGTTGTTGAGTA

						**YCNLSVATTFEDLH<br>GEIRDLALLRKIKSL<br>YGHPDNIDVWVGG<br>ISEEPVKDAKVGPT<br>FLCMLIDQFKRLRS<br>GDRFWYENPGIFSP<br>DQLVQIKQSSLGRV<br>LCDSGDNITVVTK<br>DVFTVPQHQNPNF<br>ADCSSIPKIELNVW<br>TECCQECSASQSNE<br>INRFNTRNRRSFVD<br>RYDHADESHRYRN<br>ATRKTSRDWQEDD<br>FDITEERIEGIEELVG<br>RLEKTVQKLSRKVR<br>KLEAESKFRNEGGC<br>FDHDRKKRHSGEL<br>WQKEKCTKCRCEN<br>NQITCWVETCQPSE<br>CQKPRIIDGECCPV<br>C***	AAATGTAGTATACCTTTACTTTTATTTTCTCTTCCTTTCAAATA<br>ATGCATTTTAGCTGCCCGAGATTTTGTTTCACCTGCAGGATTT<br>TTTGCCATGCACTCATATACTCCTGCATCTAAATCTTGAGTTT<br>CAGTAATCATGAGGGTACCATCTGTAAGTATACTGTACCTTT<br>CTTCTTCTTCAGTTGATATTTCATTACTATTATGCAACCATAC<br>AATATCAGGCTTTGGATCTCCATCAGCTTTACAGGTAAAATA<br>AGCTGTTCCCCCAAATGATACATCCACGTCATTTGGTTCTTCT<br>GTTAGTTTAGGTCTTTTGCATCGGAAATCCTCACTGTTTAATG<br>CCATCAAAGATTTTCCTTGAAGATTATTAGGATACTCACAAG<br>TTGCTGCAGCTTGTGTAGCTGCATGGCTTTCTTTAAGCATTTCT<br>GCAAGCCACATGATTTCACAATCACATATCAAAGCGTTAGA<br>ATCTAATCTCAAACGTCTGAGTGATTTTAGATGCTTGAAGGC<br>TCCATATGGTATTTTAGACAATCTGTTATTATGAAGGAATAGT<br>CTTTCAAGGGATGGTAGATCTGCGAAAGTATCTGGTTCTAGT<br>CTTTCAATCTTATTGAAATGTATATACAGCTGCTCTAAATTTC<br>TCAGTCCTTTGAAAGCTGTGCTTCCAATAGTCTTAATTTTATTT<br>TTATATAAATATAAGTAGCGAAGCTCTTCCAGTCCTGCAAAA<br>GCTTCATCTTCAATCTTGGTAATATGATTATTATTGAGGAGTA<br>GAGTGTTCAGATCTTTCAAGCTCTTGAAAGCATTCCTTGGAAT<br>TTCTCGGATTTTATTGAACCTCAAGTCCAGTATTGTGGTTTCG<br>GCCGGCACCTCGGGGACACTATCCAGTTGCAGGAACATGCA<br>TCTGACCGTCGTCCGGAAGCACAGGCACTTTGCAGGGCACTG<br>TCCGTGGGCCATTTCCAAGAGGACCAACAGGCAGATCTGAA<br>ACACCAGAGTCCGGAGCCCCGGGATCAGTCGGGCCCCCATC<br>TTTTTTCTTCCTGGACAAAAAAGAAGACAACCAAAATGTTAA<br>GATCTCTCCGGCAGTGTTTGTCGGTCTCTTCCCCGCACGAACC<br>GG

PhdPInSeP67	G4V4G1	8,25E-05	29,333	PF00219<br>PF00050	15910,26	ENPLLCDCNVKWIYDIVNTDTSQIRMLNSDTMLCDRPVEVHQTQLKNLKKSQIPDSCPPVLVGSINSTVDKKIDDTHIFQCRAHGIPVPNVHWILPNGAILNQTSNDIHIHMKSPGSLGIYHLKPKDSGTYVCVAENPLGEAM	TGAAAATCCCCTTTTGTGTGATTGTAATGTTAAATGGATATATGATATAGTGAATACGGACACGAGTCAGATAAGGATGTTAAATTCTGATACTATGTTATGTGACCGGCCAGTAGAAGTACATCAAACTCAGCTCAAAAATTTGAAAAAGTCCCAAATTCCAGACAGCTGCCCTCCAGTACTCGTAGGCTCCATCAACAGCACAGTAGATAAAAAGATAGATGATACGCACATCTTTCAGTGCAGGGCTCACGGAATACCCGTTCCTAACGTCCACTGGATTCTACCAAATGGTGCAATCCTAAATCAAACGTCCAATGACATCCACATCCATATGAAAAGTCCTGGTAGTCTGGGAATTTATCACCTCAAACCAAAAGACTCTGGTACTTACGTCTGCGTAGCAGAAAACCCTTTAGGTGAAGCTATGAG
PhdPInSeP68	G4V4G1	2,56E-20	55,224	PF00219<br>PF00050	7311,24	LSQNPLCGSDGVTYENICQLTEARYRRRDGLRAVSAEPCIKPPKIITAPQNVRNSTDSSAAMRCEAS	GTTTGAGTCAAAATCCTCTATGCGGGAGCGACGGAGTCACATACGAAAACATATGTCAACTGACTGAAGCCAGATATCGCAGAAGAGATGGTCTCCGAGCTGTATCGGCGGAGCCTTGCATTAAACCTCCAAAAATAATTACTGCGCCTCAAAATGTGCGAAACTCTACAGACAGTTCAGCTGCTATGAGGTGTGAAGCAAGTGC

PhdPInSeP69	Q58L91	6,95E-30	30,060	PF07732<br><br>PF00754	243425,84	MITVIQCRQVGKAAGELQGGVVCDLDIGLKCNPEFLFGDEQCGDYEVRFFCDCPTMAPATEIETLPPIIASSPVYMACAYWSEWINEHHPSGEGGGIKKSGVKGGGGSRGDTEKAMLLKLQREYNFCMEGYISDIECREAVTDLDYTETGDKKLVCSVVSGFRCRGRDQPNHRCLDYKIRYFCSCTETSTVTETTVKPTTPMIIVPPCNSYYRVVDGPLPLPDSSLKASSSRDTSSGPQSARLSSTYNARSAGAWIAGKVNDLQFIEIDLGRIQPVYGVVTKGRKSHQEWVKSYKVLYSRDGDAFYYVQDPGAPNGEKVFTGNYDSDSEVEHVFVKPFEARFVRIQPVTFYMSIAMRVDVLGCATGMDIFMTTSTTPAPLEPLCTGEMGMYTGHIHDEQITTSS	ATTGTTCAGGAAGACTGTTGGACCGAATGGATCAATATTGATGAACCAAGAGGAGGAGGGGGAGATCTGGAAGTACTGGGCAAAATCAGACAGAAATATGAATTCTGCCTGGATCCTGTGAAAATAGAATGTCGAACAGCTGTCAGTCACCAACTTCCATTTGATGTTGGACAAAGTGTTACTTGCGACGTAAACGAGGGTCTCATTTGTTGGAATAAAGAAAATAAACCTGAAGAGTGCTACGATTATGAGATCAGATTTTTCTGCCCGTGCCCAACTCAAGCCACGACTACAACGACGCCCATTCCAACATTACCACCAGGACCTTGTGTGTATGGCTGGACGGAGTGGTACAACAGTCACATACCAGATGGGCGTGGTGATTATGAAACTGTTCAGAGTTCAAGAATTTCGAATTCAATCTGCTCCAATGACATGATAACAGTGATACAATGTAGACAAGTTGGGAAGGCTGCTGGTGAACTCCAAGGTGGCGTAGTTTGTGACTTGGATATTGGACTGAAATGTAATCCTGAATTTTTGTTTGGCGATGAACAGTGTGGCGATTATGAAGTCAGATTCTTTTGTGATTGCCCAACTATGGCTCCTGCTACTGAAATAGAAACTTTGCCTCCGATAATTGCCTCTTCCCCAGTTTACATGGCATGTGCATATTGGTCTGAATGGATCAACGAACATCACCCAAGTGGAGAAGGTGGTGGCATCAAGAAAAGTGGAGTGAAAGGCGGTGGTGGAAGTAGAGGTGATACAGAGAAGGCCATGCTTCTGAAACTACAAAGAGAATACAACTTTTGCATGGAAGGTTACATATCAGACATCGAATGCCGAGAAGCTGTCACAGATCTGGATTACACTGAGACAGGTGATAAAAAGCTCGTTTGCAGCGTTGTTAGTGGCTTTAGATGCAGAGGAAGGGACCAACCAAATCACAGATGTCTAGATTACAAAATTAGATACTTCTGCTCATGCACTGAAACGTCAACAGTTACTGAAACCACAGTGAAACCTACCACTCCTATGATAATTGTTCCACCCTGTAATAGCTATTATAGGGTTGTGGATGGACCACTTCCTCTTCCAGATTCAAGCTTGAAAGCATCTAGTAGTAGAGACACCTCTAGTGGACCTCAGAGTGCTAGGCTTAGTT

						NRNWSTDGRYARL	CTACATACAACGCTAGATCAGCTGGTGCCTGGATTGCTGGTA
						NTLQTEDHTGGWV	AGGTTAATGATTTGCAGTTCATCGAAATCGACCTTGGTCGCA
						ADELNDKQYIQVDF	TTCAGCCTGTGTATGGTGTAGTTACTAAAGGCCGAAAGAGTC
						LEPRNLTGLKTQGR	ATCAAGAATGGGTTAAGTCTTATAAAGTTCTGTACAGTAGAG
						DMVPQWVTAFYISY	ATGGAGATGCTTTCTACTATGTTCAAGATCCCGGGGCTCCAA
						SQDGVKWNDITDEH	ATGGAGAAAAGGTTTTCACTGGAAATTACGATAGCGATTCGG
						GTVIFPGNYDSSTTVI	AGGTAGAGCATGTATTCGTGAAACCTTTCGAAGCCCGCTTTG
						TYFDRPIRARFLRIHP	TTCGTATTCAGCCTGTGACCTTCTATATGTCAATTGCCATGAG
						KFWENWIAMRFEIL	AGTGGATGTACTGGGATGTGCTACAGGAATGGATATCTTCAT
						GCYEPTNLVKDEIAT	GACTACTTCCACTACACCAGCACCTCTAGAACCTCTCTGTAC
						TPAHVIIPEKCIEPM	TGGCGAAATGGGCATGTACACTGGTCATATACACGATGAGC
						GFENYKLPDTLISVSS	AAATTACGACTTCCAGTAACAGAAATTGGAGCACAGATGGA
						SLGPNSGASRIRLNT	CGCTACGCTAGATTGAACACTCTTCAAACTGAGGATCACACT
						HHEGEETGGWIAAG	GGAGGATGGGTAGCAGACGAACTGAATGATAAACAGTATAT
						YDYMPKIYIDFNEER	ACAGGTTGATTTCCTGGAGCCGCGCAATTTAACTGGATTGAA
						NLTGILTQGLEDDDL	AACTCAAGGTCGCGATATGGTTCCACAGTGGGTGACAGCATT
						WVVSYKVAYSVDN	CTACATTAGCTACAGCCAAGATGGCGTGAAATGGAACGACA
						HTWDFATDAENRGI	TAACAGATGAACACGGCACAGTGATATTCCCTGGTAATTATG
						VFNGNIDRNSEVLRL	ACAGTAGTACTACAGTCATCACGTATTTTGATCGTCCAATAA
						FSRMITARFLEIIILDY	GAGCTCGCTTTTTACGTATTCATCCAAAATTCTGGGAGAACT
						HIAPALRMEILGCFIP	GGATAGCAATGCGTTTCGAAATTTTGGGATGTTATGAGCCGA
						YPVYTPPPVLLTTTEI	CTAATTTAGTCAAAGACGAAATTGCTACTACTCCAGCGCATG
						CVQYGSWISLSDPAS	TCATTATTCCAGAAAAATGCATTGAACCAATGGGATTTGAAA
						SSFGDEEPIDQVIAAS	ATTATAAGCTACCAGATACATTAATTTCTGTGTCTTCTTCTCT
						GVCRNPFEIECRTQV	CGGACCAAACAGTGGAGCCTCAAGAATAAGACTCAATACAC
						TKKDWSETGQVVTC	ATCATGAAGGGGAAGAAACAGGTGGATGGATAGCAGCAGG
						DLTRGLVCKNAHQS	TTATGACTATATGCCAAAAATCTATATTGATTTTAATGAAGA
						SYQCYNFEVRIKCW	AAGGAATCTGACTGGTATCTTAACTCAAGGCCTGGAAGATG

						TCGVTEVTTPIPLEM<br>CPEIPDSFRESCPESC<br>PYGFACDGATCVPS<br>VDCPCFKNGKRFEPS<br>NMAITEDCYSCECIL<br>GGYSRCEPIHCPECL<br>PGQKSDMDSNCHC<br>GCVGCAVGTLLCPT<br>SAECIKEELWCDGVI<br>DCPDDEIGCPTTLAP<br>TTTTLPTTTTEAPLIC<br>STDYAPDTDTCEML<br>ANVFETFDGLTYQY<br>DICDHVLMKESSSGL<br>YSVSVHKLCTSESVH<br>FCYRHLVIDVDGIIIK<br>IGPGIDDVTIKDTRV<br>PVANLWLVSQRFPD<br>FELKKSGNTLVFRSF<br>KYNFDVTWDSLQD<br>ARITVSRCLVHNIVG<br>LCGLYNKYLQDDLT<br>MPNGNLASGSQQFG<br>DSWAIGSLEKCMPP<br>KCPPETMKTAIDLC<br>NKLREEPFNSSCFEY<br>VKMENRIEACVVFM<br>CECIRRPVEAKISPSK	ATGATTTATGGGTTGTCAGCTACAAAGTCGCCTATAGTGTGG<br>ACAACCATACATGGGACTTCGCTACAGACGCCGAAAATCGT<br>GGCATAGTATTCAATGGAAATATTGACAGAAATTCTGAAGTG<br>TTGAGACTGTTCAGTAGAATGATAACGGCACGATTCTTAGAA<br>ATTATCATCCTAGATTACCACATCGCTCCTGCATTGCGAATG<br>GAAATATTGGGATGTTTCATACCATACCCTGTGTATACACCG<br>CCTCCTGTTCTTCTCACCACTACGGAAATATGTGTTCAGTACG<br>GCTCTTGGATTAGCTTGTCCGATCCTGCCTCTTCTTCATTCGGT<br>GACGAAGAACCTATTGACCAGGTCATAGCTGCCTCTGGTGTC<br>TGCCGAAATCCCTTCGAAATCGAGTGCCGCACGCAAGTAAC<br>CAAAAAAGATTGGTCGGAGACTGGACAAGTTGTAACCTGCG<br>ACCTTACTCGTGGCCTGGTCTGTAAAAACGCGCATCAGTCCT<br>CTTATCAGTGCTACAACTTTGAAGTGAGGATCAAGTGCTGGA<br>CGTGCGGAGTAACAGAAGTGACGACTCCTATTCCACTAGAA<br>ATGTGCCCCGAAATTCCCGATAGTTTCAGAGAAAGCTGTCCA<br>GAATCTTGTCCTTATGGCTTTGCTTGTGATGGAGCTACTTGCG<br>TTCCCAGTGTTGATTGCCCATGCTTCAAAAATGGAAAGCGTT<br>TCGAACCGTCAAATATGGCCATTACTGAAGACTGCTACAGCT<br>GCGAATGTATATTAGGAGGATACTCAAGATGTGAGCCTATTC<br>ATTGCCCAGAATGCTTGCCAGGCCAAAAATCTGATATGGATT<br>CTAACTGCCATTGCGGTTGTGTCGGTTGTGCCGTAGGAACTCT<br>GCTATGTCCGACAAGTGCAGAATGCATAAAAGAGGAACTAT<br>GGTGCGATGGCGTCATAGACTGTCCAGATGACGAAATAGGG<br>TGTCCAACAACTCTTGCACCTACTACGACAACTTTACCAACA<br>ACTACGACAGAGGCGCCGCTCATTTGCTCAACAGACTATGCT<br>CCAGATACAGACACCTGCGAAATGTTGGCAAATGTGTTCGA<br>GACATTCGATGGTCTGACTTATCAGTATGATATATGTGATCA<br>CGTTCTGATGAAAGAATCTTCTTCTGGACTATATTCAGTATCA

						DVLYDFFGVDTCKC<br>QAYESFVEACEYSKQ<br>GPVQEWRILHDCTP<br>DCTEGMEWQDCGP<br>GCELSCDNYHQRSEI<br>CNSEICVPGCYCPPG<br>LVRHHNRCVKPKM<br>CQDCVCRGHGDPN<br>YITFDGRYYAFQGN<br>CTYILAQHQISDDEA<br>KNFRILATNVECPEE<br>PHTSCTQGLTIYWN<br>GHVIEKFKNKPVYF<br>NQKPLSPEDSPLEYE<br>GISITYVPNKSTLIHI<br>QEINLAVRYFDQMY<br>GFNIELPAFYYYNKT<br>EGLCGICNFIQSDDL<br>YHRDGYVTEDIEDF<br>AYSWLEEHETRKNC<br>ELPRVEVPEPPQGIC<br>NFTVSPCEIFLDSTLY<br>SKSCQNDVTYSQKP<br>EASMCRSKFQYAQQ<br>CCERGVSLVEWLKL<br>SGCESSCPEGMVYEC<br>TSACQKTCDNYKTY<br>QDEYCSLMPLYTCV	GTTCACAAATTGTGCACATCTGAAAGCGTCCACTTCTGTTAC<br>CGGCACCTAGTTATAGATGTTGACGGAATTATAATCAAAATA<br>GGTCCAGGCATCGACGATGTAACCATTAAAGACACCAGGGT<br>ACCTGTAGCCAATCTGTGGCTAGTTTCCCAGAGATTCCCGGA<br>TTTTGAGTTGAAGAAAAGTGGAAACACCTTAGTCTTCAGATC<br>CTTCAAATACAATTTTGATGTCACGTGGGACTCGTTGCAAGA<br>TGCAAGAATTACGGTATCTAGATGCTTGGTTCATAATATAGT<br>GGGTTTGTGCGGATTGTATAACAAGTATTTGCAGGACGATCT<br>CACAATGCCAAATGGTAATTTGGCATCTGGAAGTCAGCAATT<br>TGGCGACAGTTGGGCGATTGGATCTCTAGAAAAATGCATGCC<br>ACCGAAGTGTCCTCCAGAAACTATGAAAACAGCTATAGACC<br>TTTGCAATAAACTTAGAGAGGAGCCTTTTAATTCTTCTTGCTT<br>CGAATATGTTAAGATGGAGAACAGAATAGAAGCTTGTGTTGT<br>ATTCATGTGCGAATGTATACGTCGACCTGTTGAAGCTAAAAT<br>TTCGCCATCAAAAGACGTGTTGTATGATTTCTTTGGCGTTGAT<br>ACATGCAAATGTCAAGCTTATGAAAGCTTCGTAGAAGCTTGT<br>GAGTATTCCAAACAAGGACCTGTCCAAGAATGGAGAATTCT<br>ACATGACTGTACGCCCGATTGTACGGAAGGAATGGAATGGC<br>AAGATTGCGGTCCCGGATGCGAGCTGAGTTGCGATAACTACC<br>ACCAACGATCAGAGATTTGTAATTCAGAGATTTGCGTTCCTG<br>GTTGTTACTGTCCTCCAGGTCTTGTCCGACACCACAACCGGT<br>GCGTCAAGCCAAAGATGTGCCAAGACTGCGTCTGCCGTGGTC<br>ACGGAGATCCTAATTACATTACCTTCGATGGCCGTTACTATG<br>CCTTCCAAGGAAACTGTACCTACATTCTTGCCCAACACCAAA<br>TATCAGATGACGAAGCAAAGAATTTCCGAATCTTAGCCACTA<br>ACGTAGAGTGTCCTGAGGAGCCACATACATCTTGCACACAA<br>GGCTTAACGATCTACTGGAATGGGCACGTCATCGAAAAGTTC<br>AAAAACAAACCGGTGTATTTTAATCAAAAACCTCTGAGTCCC

						CPEGEVLKLGKCVK<br>TIQCEVCDAQGHLI<br>GEVWQEGPCKTCEC<br>FSDLSISCVIKECPEP<br>PLCSVKETLQRLNRS<br>AEECCDAYLCMAM<br>AEKTCPEPPLEECPP<br>GEAYVRDYHDGCPI<br>YRCECIPELCPPPIVP<br>LLDDGENFTAIAIDC<br>CTQFRIICDIDLCIPPP<br>KCNPGFKLSTYEGRC<br>CNKYTCIPRNDVCL<br>YQHKYDVVNGTQV<br>HLPLEEYYDMEYEP<br>GSSWQDGLCRKCSC<br>VEINGQFTYSCKEEI<br>CPNPNDFPDAEKYF<br>REELRIPGACCPKYR<br>RTACIVQGVKYQIDE<br>EWPSPEGNPCKSYR<br>CVLDNKGEAVRLEK<br>TTICDTACPDYAVYV<br>EATPESGKCCGMCK<br>PIACEENGILYEDGQ<br>TWVSTAKPCFTAEC<br>LVTENGTRITYRGES<br>CPIMPDNCAPENVK	GAAGATTCTCCTCTTGAGTACGAAGGAATTAGCATCACTTAC<br>GTGCCAAATAAAAGTACCTTGATTCACATTCAAGAAATCAAC<br>TTGGCTGTTCGCTACTTTGATCAAATGTACGGCTTCAACATAG<br>AACTTCCTGCATTCTATTACTACAACAAGACTGAGGGATTGT<br>GTGGTATATGCAACTTTATTCAGTCAGACGACTTATACCACC<br>GTGATGGCTATGTAACCGAAGACATCGAAGACTTCGCTTACA<br>GCTGGTTGGAAGAACACGAGACCAGAAAGAACTGCGAGTTG<br>CCTCGAGTAGAGGTTCCTGAACCTCCACAAGGCATCTGTAAC<br>TTTACGGTCAGCCCCTGCGAAATCTTCCTTGATTCGACTCTTT<br>ACTCGAAGTCATGTCAGAATGACGTTACCTACAGCCAAAAG<br>CCAGAAGCCTCCATGTGCAGATCTAAGTTCCAGTATGCTCAG<br>CAATGTTGCGAGAGAGGAGTGTCGCTGGTGGAGTGGTTGAA<br>ACTCAGCGGTTGCGAGAGTTCTTGTCCCGAAGGAATGGTTTA<br>CGAATGCACCTCAGCTTGTCAGAAAACATGCGATAACTATA<br>AGACTTACCAAGACGAATACTGCAGCCTCATGCCTCTATACA<br>CGTGTGTGTGTCCAGAGGGTGAAGTTCTGAAACTTGGCAAAT<br>GTGTGAAAACTATTCAATGTGAAGTTTGCGATGCTCAAGGAC<br>ATCTAATAGGGGAAGTGTGGCAGGAAGGACCTTGCAAGACA<br>TGCGAATGCTTTTCAGATTTGTCGATCAGTTGCGTGATCAAGG<br>AATGTCCAGAGCCTCCACTGTGCAGTGTGAAAGAAACGCTC<br>CAGAGATTGAACAGATCAGCTGAAGAATGTTGTGATGCTTAC<br>CTATGCATGGCTATGGCGGAAAAGACCTGTCCTGAACCACCT<br>TTAGAAGAATGTCCTCCTGGGGAAGCATACGTAAGAGATTAT<br>CATGACGGATGTCCGATATACAGATGTGAATGTATTCCAGAA<br>TTATGTCCTCCTCCTATCGTACCTTTGTTGGACGATGGTGAAA<br>ATTTCACTGCAATAGCAATAGACTGCTGCACGCAGTTCAGAA<br>TTATATGCGATATAGATTTATGTATCCCTCCACCCAAGTGCA<br>ACCCTGGTTTCAAGTTATCCACTTACGAAGGTCGATGCTGCA

						SDPKGCCTYCKIPTE<br>TCTAVEVPLYETRGF<br>FGYVDKRKGFCSNE<br>QVLRGLTKCGGQCG<br>TESHYSDLIGDFQSV<br>CNCCLPRESRNRTIT<br>LDCEDGSRVEKVYM<br>QPITCQCLSCSGSSRY<br>LDTLNKVEPYRK*	ACAAGTACACGTGCATTCCAAGGAACGATGTATGTCTATATC<br>AGCATAAGTATGACGTTGTGAATGGTACTCAAGTACATCTCC<br>CACTTGAAGAATATTATGATATGGAATATGAGCCTGGATCAT<br>CATGGCAAGATGGATTGTGCCGAAAATGCTCTTGCGTTGAAA<br>TAAATGGACAGTTTACTTACAGCTGCAAAGAGGAGATTTGCC<br>CGAATCCTAACGACTTCCCTGATGCAGAAAAATATTTCAGAG<br>AAGAGCTTCGAATCCCTGGAGCCTGCTGTCCAAAATACAGA<br>CGAACAGCTTGTATCGTTCAAGGAGTGAAATATCAAATCGAC<br>GAGGAATGGCCGTCACCCGAAGGCAATCCTTGCAAGTCCTA<br>CCGCTGCGTGCTCGACAATAAAGGTGAAGCTGTCAGACTGG<br>AGAAAACAACCATATGTGACACAGCTTGTCCAGATTATGCTG<br>TATATGTTGAAGCCACTCCTGAATCTGGTAAATGTTGCGGGA<br>TGTGCAAGCCAATAGCCTGCGAAGAAAATGGCATCCTATAC<br>GAAGATGGTCAGACGTGGGTCTCAACAGCCAAACCTTGTTTT<br>ACGGCTGAGTGTTTGGTAACAGAGAATGGAACACGAATCAC<br>ATACAGAGGTGAAAGCTGTCCTATAATGCCAGACAACTGTG<br>CTCCGGAAAATGTCAAGTCCGATCCGAAAGGTTGCTGCACTT<br>ATTGTAAAATACCGACAGAAACTTGCACAGCTGTCGAAGTA<br>CCGCTTTACGAGACAAGGGGATTCTTTGGATACGTGGACAAA<br>CGAAAAGGATTCTGTTCCAATGAGCAAGTATTAAGAGGTCTA<br>ACGAAATGTGGAGGACAATGTGGTACAGAATCACACTATTC<br>TGATCTGATAGGAGACTTCCAGAGTGTCTGTAATTGCTGTTTG<br>CCACGGGAATCACGAAATAGGACAATTACCCTAGACTGCGA<br>AGATGGATCTAGAGTGGAAAAAGTATACATGCAGCCAATCA<br>CATGTCAGTGCTTATCTTGTTCTGGAAGCAGCCGCTACCTCGA<br>CACCCTGAATAAAGTGGAGCCTTACAGAAAATAGCATTTCG<br>CAGAAGTAAAATGCTTCAGAAAATTTTATGCTGTTAACTGTT<br>GCTCTGCTAAAAAGCTTGAACGCATCTGTTTCTGGACATCTAT

							TAACTACTAACAGTGTTATCTTCTGTGATCTGCAACTTTTCAC TAATAAAAATGCAATTCGTTTATTGTTTAAAAAAAA

PhdPInSeP70	Q7SZN0	9,57E-37	31,210	PF07732<br><br>PF00754	278812,15	YLEVKFSDEGTITGVI<br>TKGREDKPNWVRSF<br>MIAYSDDGKNWEK<br>VKNEVGQPEIFRAN<br>TNQDERVTNMLPSP<br>VQAQYIRFHPVSWKI<br>KISMQVEILGCLPEE<br>PVTKEEEEEEEEEETP<br>EIEECKEPMGLENGL<br>LMDSQLSASSSYSSRF<br>TPDSARLGSDSVWA<br>TAVQDDNQYLQVD<br>FIDEQKIAGIITKGRE<br>DIPQWVTAYTVQFS<br>KDGVIWNKVKDDN<br>GVDQEFPANYDPFS<br>TVTNLFPNTVRARLL<br>RIRPTKWKNWISMQ<br>LEILGCYHPEPCRDP<br>MGIENGIITDHQLTA<br>SSEISESLSANNGRLT<br>SPTAWSPEISDKNPF<br>LQVDLLEPINLTAVV<br>TKGNRELQQWVTSF<br>NVAYSNDSVDWKK<br>VVDTKGKIKEFIGNE<br>DQDSAVISLLPIPVV<br>ARYVRIIPLEMHRQI	AATATCTTGAGGTAAAATTCTCCGACGAAGGAACTATAACTG<br>GAGTCATAACGAAAGGCCGAGAAGATAAACCAAATTGGGTC<br>CGATCGTTTATGATTGCATACAGTGACGATGGAAAAAACTGG<br>GAGAAAGTGAAAAACGAAGTTGGCCAGCCGGAGATATTCCG<br>GGCCAACACCAACCAAGACGAACGCGTTACCAACATGCTTC<br>CGTCACCTGTACAGGCACAATACATTCGTTTCCATCCAGTCT<br>CATGGAAAATCAAAATAAGCATGCAAGTAGAAATTTTAGGA<br>TGTTTACCTGAAGAACCAGTGACAAAAGAAGAAGAAGAAG<br>AAGAAGAAGAAGAAGAGACTCCTGAAATTGAAGAATGCAA<br>GGAACCAATGGGCCTTGAAAATGGCTTATTGATGGACAGTC<br>AACTCTCGGCCTCATCCAGCTATAGTTCAAGGTTTACTCCAG<br>ACTCGGCACGCCTAGGTTCTGACTCAGTTTGGGCAACTGCAG<br>TTCAAGATGATAATCAATATTTGCAGGTCGACTTCATTGACG<br>AGCAAAAGATTGCTGGTATAATCACGAAAGGCAGGGAAGAT<br>ATACCGCAGTGGGTAACAGCTTACACTGTGCAGTTCAGCAA<br>AGATGGTGTCATTTGGAACAAAGTAAAGGATGACAATGGTG<br>TTGATCAAGAGTTCCCCGCCAATTATGATCCATTTTCTACGGT<br>TACAAACCTATTCCCAAATACTGTTAGAGCGAGACTTTTAAG<br>GATACGTCCAACGAAATGGAAAAATTGGATAAGCATGCAGC<br>TAGAAATCTTAGGATGTTACCATCCAGAACCTTGCCGAGACC<br>CTATGGGAATAGAAAATGGTATAATCACTGACCATCAACTG<br>ACAGCTTCATCTGAAATATCCGAATCACTGTCTGCGAACAAC<br>GGACGTCTCACTTCTCCCACGGCTTGGTCACCTGAGATTTCTG<br>ACAAAAATCCATTCCTGCAGGTTGATTTGCTTGAACCCATCA<br>ACCTCACTGCAGTAGTAACAAAAGGCAATCGAGAGCTTCAA<br>CAGTGGGTCACATCATTTAATGTTGCCTACAGTAATGATTCC<br>GTGGACTGGAAGAAGGTGGTAGACACTAAGGGTAAGATAAA<br>AGAATTCATTGGAAACGAAGATCAAGATTCAGCTGTCATCA

						ALRMDLLGCFHEKV<br>CLEPMGLENGVLAD<br>AQITASSSSDPDTTPS<br>HARLSDKSGWQPD<br>KLDTHPYLQIDFLEP<br>RNVSAVVTKGMED<br>ADNWVTKFKVVVS<br>DDRRKWYPLITDDG<br>DDVVFKANEDRDTP<br>VVNTFPEEVEARFFR<br>IVPLDFENAVGLRVE<br>LLGCYHPHECQEPM<br>GMESGSVFDFQISAS<br>SFLDDEFSPAKARLD<br>SDTAWVADVNDDE<br>PFIEVDFLVPTNITGV<br>MTRGRNDANEWVK<br>AYKIEYSEDGVNNF<br>VSDKDAQGKEMEYL<br>ANSDSESTVTNTFEE<br>PILARLLRIIPVSYEK<br>KIALRFEVLGCFHKT<br>ECMEPMGLENGLLE<br>DSHISATSYKSEDTN<br>PTKLRLNSDASWMP<br>DTIDDDQFIQVDFID<br>PKNVSAVVTKGDAT<br>EPVWITAYQVEYSED	GTCTACTTCCCATCCCAGTCGTTGCGAGATATGTTCGAATCAT<br>TCCATTGGAAATGCACAGACAAATTGCTCTAAGAATGGACCT<br>TTTGGGCTGCTTCCATGAAAAAGTCTGTCTCGAACCCATGGG<br>TCTGGAAAATGGCGTTCTTGCGGATGCACAGATAACTGCTTC<br>ATCATCATCTGATCCTGATACTACCCCGTCTCATGCCCGACTC<br>AGTGATAAATCTGGTTGGCAGCCGGACAAATTAGACACTCAT<br>CCTTATCTTCAGATTGACTTCTTAGAACCCCGTAATGTCAGTG<br>CAGTGGTTACCAAGGGCATGGAAGACGCTGACAATTGGGTT<br>ACAAAATTCAAGGTTGTGGTCAGTGACGACAGGCGCAAATG<br>GTATCCCCTGATCACAGATGATGGTGATGATGTGGTATTTAA<br>AGCCAATGAAGACAGAGATACTCCTGTTGTCAACACTTTTCC<br>AGAAGAGGTAGAAGCTCGATTTTTCCGTATCGTTCCTCTCGA<br>TTTCGAAAATGCTGTTGGCCTCCGAGTGGAACTTCTAGGATG<br>TTATCACCCTCATGAATGTCAAGAACCCATGGGAATGGAGA<br>GCGGCTCCGTTTTTGATTTCCAGATTTCCGCTTCATCTTTCCTA<br>GATGATGAATTCAGTCCAGCGAAAGCTAGGCTCGACAGCGA<br>TACTGCTTGGGTAGCGGATGTAAATGACGACGAACCATTCAT<br>TGAGGTTGACTTTTTGGTGCCAACTAATATAACTGGTGTAATG<br>ACTCGAGGCAGAAATGATGCCAACGAATGGGTTAAAGCGTA<br>CAAAATTGAATACAGTGAAGATGGAGTTAACAATTTCGTTTC<br>TGATAAAGATGCTCAAGGAAAAGAGATGGAATATCTAGCAA<br>ATTCTGACAGTGAATCAACTGTTACCAATACTTTCGAAGAGC<br>CAATACTTGCTCGATTACTGCGCATAATACCAGTATCATATG<br>AAAAGAAGATAGCCTTGAGATTTGAAGTTCTAGGATGTTTCC<br>ACAAAACTGAATGCATGGAGCCTATGGGTCTTGAAAATGGA<br>CTCTTAGAAGACAGCCACATCTCTGCTACGTCCTACAAATCA<br>GAAGACACGAATCCGACAAAATTACGCCTCAATAGTGATGC<br>TTCCTGGATGCCAGATACCATAGATGATGATCAATTCATTCA

						GNTWSSIEDTMGNI<br>KEFPANSDNDSPVIN<br>VFPETVEARYIRIIPV<br>KWKNGIALRAEILG<br>CYHPYGEEAGTELTT<br>LPEIVMPYAGVYTEP<br>PMLVACPDPMESEIS<br>LLTGARIQASSSTPD<br>GGPTRVLLHTEAAK<br>GQSGGWVPNVDDL<br>HPVLFVQLPEKRWL<br>TSMYIQGREDEENWI<br>KSLRVVASPDNSTW<br>MPVFGPDGDEIIEAN<br>EDQANIVGLYFKDP<br>TEAKYIKIIPETWHR<br>WPSFRLDLRGCRMP<br>EATVLPVSTPAAYPE<br>LCPELLNYPQLAQY<br>CPEDCPEGQLCDGE<br>GCVDPVDCACVRD<br>GKIYKVSDRLEDRLC<br>RECDCALGGHSICSE<br>KQCEPCEVGQTSVL<br>NDDCSCTCEECPEG<br>QILCPSDPQCIPEER<br>WCDGTIDCEDDEID<br>CFSTTEEPVVTVIGP	AGTCGACTTCATAGATCCCAAAAATGTGTCGGCTGTTGTCAC<br>CAAAGGTGATGCAACTGAACCTGTGTGGATCACGGCATATC<br>AAGTAGAATACAGTGAGGACGGAAACACCTGGTCATCAATA<br>GAAGATACGATGGGAAATATTAAAGAATTTCCAGCAAACTC<br>AGACAATGATTCCCCTGTTATTAACGTCTTTCCCGAAACAGT<br>AGAAGCAAGATACATTAGGATCATTCCTGTTAAATGGAAAA<br>ACGGAATAGCTTTACGAGCGGAAATTCTTGGTTGTTACCATC<br>CATACGGCGAGGAAGCCGGAACAGAATTAACAACTCTTCCG<br>GAAATTGTTATGCCTTACGCTGGTGTTTATACTGAACCCCCAA<br>TGCTTGTTGCGTGTCCGGATCCGATGGAATCGGAGATAAGTT<br>TGTTGACGGGTGCTAGAATTCAAGCAAGCTCCTCGACTCCCG<br>ATGGAGGACCCACTCGAGTTTTACTGCATACAGAAGCCGCTA<br>AAGGCCAAAGTGGAGGATGGGTACCAAATGTTGATGATTTG<br>CATCCAGTTCTTTTTGTTCAGCTACCAGAAAAGAGATGGTTG<br>ACTTCAATGTATATTCAAGGACGAGAAGATGAAGAAAATTG<br>GATCAAGTCTCTGCGTGTTGTTGCAAGCCCAGACAATAGCAC<br>TTGGATGCCTGTATTTGGTCCGGATGGAGATGAAATAATCGA<br>AGCTAACGAAGATCAAGCAAATATAGTTGGATTGTATTTCAA<br>GGACCCCACAGAAGCCAAATATATTAAGATCATCCCAGAAA<br>CATGGCACAGGTGGCCTAGCTTCAGATTAGACTTAAGAGGTT<br>GTCGTATGCCTGAAGCTACAGTATTACCAGTCTCTACACCTG<br>CTGCATATCCTGAACTATGCCCAGAGCTCCTTAATTATCCTCA<br>ACTTGCTCAATATTGTCCAGAAGACTGCCCAGAAGGACAATT<br>ATGCGATGGAGAAGGATGTGTAGACCCTGTAGACTGCGCAT<br>GCGTGCGCGATGGAAAAATATATAAAGTGAGCGACAGACTA<br>GAAGATCGACTCTGCAGAGAGTGCGACTGCGCCTTGGGGGG<br>TCATTCAATATGCAGTGAGAAGCAGTGTGAACCCTGCGAAGT<br>GGGCCAAACATCAGTCTTAAATGATGACTGTAGTTGCACTTG

						DGIPVAVVPPTEEGR<br>LKPAKKHRTKKPAG<br>TTTTPVVPFTVKPSV<br>PGNGTCNVMGNHV<br>ETFDGQDIIYDVCN<br>HVVFKDIKNEKYNV<br>TIHKECNYVSECKH<br>WIEVYYMNQHIKVQ<br>SDLNVEVNGHSYTT<br>SQLWRLSKSTKMNL<br>VAKKVGEQVMITSL<br>ADSFTIIYDIRGHLEI<br>DVAPELLQKVSGLC<br>GYYSGRSDDDQRKP<br>NGKKAFTSREFGES<br>WAIKERGKECKPLV<br>CPHEQMSKAMELC<br>HKLKERPFIACGRLL<br>PLNQFIEFCMSTTCE<br>CLLNKKNSENKCKC<br>DGLLPFVEACESKLG<br>PMAVRNWRLIHEC<br>HTECPPGMEWNDC<br>GPSCQLTCDSTSEDT<br>LQECSGRCVPGCFCP<br>AGTVLHEEECIPPEE<br>CRDQVCQGFGDPHF<br>KTFDGLFFPFQAVGS	CGAGGAATGTCCAGAAGGGCAGATACTGTGTCCTTCAGATCC<br>ACAATGCATCCCTGAAGAACGATGGTGTGATGGTACAATAG<br>ACTGTGAGGACGATGAAATTGACTGCTTCTCTACAACTGAAG<br>AACCGGTGGTTACAGTGATAGGGCCAGATGGAATACCAGTT<br>GCTGTCGTACCACCGACTGAAGAGGGCAGACTGAAACCAGC<br>GAAAAAACATCGAACAAAAAAACCAGCTGGTACTACAACT<br>ACTCCAGTTGTTCCATTCACTGTGAAACCCTCAGTCCCAGGA<br>AACGGTACGTGCAACGTCATGGGCAATCATGTGGAAACATT<br>CGATGGCCAGGATATTATATATGATGTATGCAATCACGTCGT<br>CTTTAAAGATATAAAAAATGAAAAATACAACGTTACAATTC<br>ACAAAGAATGCAACTATGTGTCAGAATGCAAGCACTGGATT<br>GAAGTCTATTACATGAATCAACACATAAAGGTACAATCCGA<br>TCTTAATGTCGAAGTTAATGGCCATAGTTACACTACTTCACA<br>ATTATGGCGTCTAAGCAAGAGTACCAAAATGAATCTAGTTGC<br>GAAAAAAGTAGGAGAACAAGTTATGATAACATCCCTTGCTG<br>ACAGTTTCACTATTATCTATGATATTAGAGGACACCTTGAAA<br>TTGATGTCGCTCCAGAGCTGCTTCAGAAAGTTTCTGGCTTGTG<br>TGGATACTATTCTGGAAGATCAGATGACGACCAACGAAAAC<br>CAAATGGCAAAAAGGCTTTTACAAGCAGGGAATTCGGGGAG<br>AGTTGGGCGATCAAAGAGAGAGGAAAAGAATGCAAACCTCT<br>TGTATGTCCCCATGAACAGATGTCCAAAGCAATGGAGCTGTG<br>TCACAAACTGAAGGAAAGACCTTTCATTGCGTGTGGAAGACT<br>ACTGCCTCTGAATCAGTTCATTGAATTCTGCATGAGTACGACT<br>TGTGAATGTCTACTGAACAAGAAGAACTCTGAGAACAAATG<br>CAAATGCGACGGTTTGTTACCTTTCGTAGAAGCTTGCGAATC<br>CAAGCTAGGGCCGATGGCAGTCAGGAACTGGAGACTTATAC<br>ATGAATGTCACACGGAATGTCCTCCTGGAATGGAATGGAAT<br>GACTGTGGTCCATCCTGCCAGTTAACTTGCGATAGTACTTCTG

						FLIVSDTGKNFEVKG	AAGACACGCTTCAAGAATGTTCTGGCAGGTGTGTTCCAGGTT
						LSKKCGLFNPITCLV	GCTTCTGTCCCGCAGGAACAGTGCTTCACGAGGAAGAGTGTA
						GMEISYKRHKISIQKS	TTCCACCAGAAGAGTGTCGTGATCAAGTATGCCAAGGATTTG
						KELRLDRKRLSMDK	GCGATCCACACTTCAAAACGTTCGATGGACTGTTTTTCCCGTT
						LPLYEDGMVILGYPG	CCAAGCAGTGGGATCATTTTTGATCGTCAGCGATACAGGCAA
						RTFVIVIPSINFEARY	GAATTTTGAAGTCAAGGGACTGTCGAAGAAGTGTGGTCTATT
						YEENSGFAVRVPSRR	TAATCCCATAACTTGTCTTGTGGGAATGGAAATCTCTTATAA
						YFNKTRGLCGNCNG	GAGGCACAAGATATCGATTCAAAAGAGCAAAGAGCTTCGAT
						RQDDELKNEPLNEY	TAGACAGAAAGAGATTATCAATGGACAAGCTACCTTTGTAC
						VCSWQTDGNKEDC	GAAGATGGCATGGTTATCCTCGGGTACCCTGGGAGAACATTT
						MKELEELPELAQRV	GTTATAGTTATTCCATCGATCAACTTTGAAGCTAGATATTACG
						PICEKLKDPVFEACH	AAGAAAATTCAGGATTTGCAGTAAGAGTGCCATCCAGAAGA
						PLIDVNIYIDACNFD	TACTTTAATAAAACAAGAGGATTGTGCGGTAACTGCAATGGC
						ATHSGDNQASFCKT	CGGCAAGATGACGAATTAAAGAATGAACCACTTAATGAATA
						AMEYGRQCCQADV	CGTTTGTAGCTGGCAAACCGATGGTAACAAAGAAGACTGTA
						SIEDWPRELGCDIDC	TGAAAGAACTCGAAGAACTTCCCGAGTTAGCTCAGCGTGTA
						PSGMSFEQCHKGCP	CCAATCTGTGAAAAACTCAAAGATCCAGTATTTGAAGCTTGT
						QTCLSKKNESGDSSC	CATCCTCTCATAGATGTGAATATTTATATCGACGCATGTAATT
						KQLRTDGCFCPEGKI	TCGATGCTACCCACAGTGGGGATAACCAGGCGTCTTTCTGCA
						LKDDGCVDIELCETC	AAACAGCTATGGAATATGGCAGGCAGTGTTGCCAAGCTGAT
						DEEGHVPGDKWDD	GTTAGTATAGAAGACTGGCCTCGGGAATTAGGATGCGATATC
						GPCKHCECHEDRTV	GATTGCCCGTCTGGTATGTCCTTCGAACAGTGTCATAAAGGA
						ECIVDNCPHGPVCS	TGCCCTCAGACATGTCTTAGTAAGAAGAATGAATCCGGTGAT
						NLETYVKQVNEEKE	TCATCTTGCAAACAACTGCGAACAGACGGGTGTTTCTGCCCT
						SCCESYVCVPKEEKC	GAAGGAAAGATCCTGAAAGATGATGGATGCGTCGATATTGA
						PTMELPKCKKGEVA	ATTATGCGAAACATGTGATGAAGAAGGCCATGTGCCTGGTG
						KVHTSEEDTCPVLK	ACAAGTGGGACGATGGACCATGCAAACATTGCGAGTGTCAT
						CECDPLLCAPLVWP	GAAGACCGAACTGTTGAATGCATTGTGGACAACTGCCCCCAT

						EDLEDGQKAVMAN<br>DSCCPLVEVICFDEF<br>CPDKPYCEEHLELVE<br>AKGECCTTYKCKPP<br>DDVCAYSHMFKIVG<br>GFQSLVEQIEGDTTIY<br>QPGEEWEDGLCQKC<br>SCIESNGQHVPLCV<br>MEHCTTLEDHPENK<br>EYVLQEMKIPDKCC<br>PDIFKIKCRDDDGNV<br>YEPGEQWQDEDDPC<br>VTFLCEQEGTGEVLK<br>IKSVINCTECPRTAIL<br>FPPSKALGQCCGFC<br>KPSKCEDGEDLHEV<br>GDTWPNPDDACKM<br>AECVSEYGKISVVYT<br>KQPCPKIPEDCPPDQ<br>IVLDENECCQVCNL<br>TSEVSCSSGPIPDEET<br>VGFFKYNEPSEGGIC<br>VNERPVSKVLQCKG<br>ACHSQSFYSLEDGDF<br>KDTCKCCKVNVTTN<br>RTVELKCPNGNYIR<br>KTFLQPETCQCSKCA<br>PKSGDSKQNSFKPQS	GGACCTGTCTGCTCAAATCTCGAAACCTACGTTAAACAAGTC<br>AACGAAGAAAAGGAATCATGCTGTGAATCCTACGTATGCGT<br>GCCTAAGGAAGAAAAATGCCCGACCATGGAATTACCCAAGT<br>GCAAAAAGGGAGAAGTAGCAAAAGTGCACACAAGTGAAGA<br>GGATACTTGCCCCGTTCTAAAATGCGAATGTGATCCACTTTTA<br>TGTGCTCCCCTTGTGTGGCCAGAAGATTTAGAAGATGGTCAA<br>AAAGCAGTCATGGCAAACGACTCTTGTTGTCCTCTTGTCGAA<br>GTTATCTGTTTCGATGAATTTTGTCCTGACAAACCATACTGTG<br>AAGAACATCTGGAACTTGTCGAAGCGAAAGGAGAATGTTGC<br>ACTACTTATAAATGCAAGCCTCCAGATGATGTTTGTGCATATT<br>CTCATATGTTCAAAATCGTGGGTGGCTTCCAGTCACTTGTGG<br>AACAAATAGAAGGGGATACTACTATATATCAGCCGGGAGAG<br>GAATGGGAAGATGGCCTATGTCAGAAATGTAGCTGTATTGA<br>AAGCAATGGACAGCACGTGCCACTGTGCGTCATGGAGCATT<br>GTACCACCCTGGAAGATCATCCTGAAAATAAGGAATATGTTC<br>TGCAAGAGATGAAAATACCGGATAAATGCTGTCCAGACATT<br>TTTAAAATTAAATGTCGAGACGATGATGGAAATGTGTACGAG<br>CCAGGGGAACAGTGGCAAGATGAAGATGATCCTTGCGTAAC<br>ATTCCTTTGCGAACAAGAAGGAACTGGAGAAGTCCTCAAAA<br>TCAAAAGCGTCATCAACTGCACGGAATGTCCTAGAACCGCT<br>ATTCTCTTCCCGCCCTCAAAAGCACTGGGGCAGTGTTGCGGT<br>TTCTGCAAGCCGTCCAAATGTGAAGATGGAGAAGATTTGCAT<br>GAGGTTGGAGATACGTGGCCCAACCCAGATGATGCCTGTAA<br>AATGGCAGAATGTGTGAGCGAATACGGAAAGATTTCGGTTG<br>TCTATACGAAACAGCCATGCCCAAAAATTCCGGAAGATTGC<br>CCTCCGGACCAAATTGTTTTGGATGAAAATGAATGCTGTCAA<br>GTTTGCAATCTAACTAGTGAGGTCAGTTGCAGTTCGGGTCCT<br>ATACCAGATGAGGAAACCGTAGGCTTTTTCAAGTACAACGA

						PWNQQNQWYTESEFVIAQRPVDNERFTQQRDIDEDIQQLQQNQVQQQVEFEDI*	ACCATCGGAAGGCGGAATTTGTGTTAACGAAAGACCTGTTTCTAAAGTTCTCCAATGCAAAGGCGCTTGCCACTCCCAATCTTTCTATTCACTAGAGGATGGAGATTTCAAAGATACGTGCAAGTGCTGCAAAGTCAATGTCACAACAAATCGTACTGTTGAACTCAAATGTCCCAATGGCAACTATATTCGAAAAACTTTCCTTCAACCAGAAACGTGCCAGTGCTCGAAATGTGCCCCGAAGTCAGGAGATTCCAAACAAAACAGTTTCAAACCACAGTCACCATGGAATCAACAAAATCAGTGGTATACTGAGTCTGAATTCGTAATTGCTCAGCGACCTGTAGATAACGAACGCTTCACACAGCAGAGAGATATTGATGAAGATATCCAACAGTTACAGCAAAATCAAGTGCAACAGCAAGTGGAATTCGAAGACATTTAAAACGACTATATTGATGTACACGTAGTTGATGCAAACGACAAATTAATCAATGTGACTTGAAAGTGATACTCTATTTAATAAAGTCACAAGTTGGTTTAAAAAAAA

PhdPInSeP71	Q7SZN0	9,32E-37	31,210	PF07732<br><br>PF00754	278669,01	YLEVKFSDEGTITGVI<br>TKGREDKPNWVRSF<br>MIAYSDDGKNWEK<br>VKNEVGQPEIFRAN<br>TNQDERVTNMLPSP<br>VQAQYIRFHPVSWKI<br>KISMQVEILGCLPEE<br>PVTKEEEEEEEEETPE<br>IEECKEPMGLENGLL<br>MDSQLSASSSYSSRFT<br>PDSARLGSDSVWAT<br>AVQDDNQYLQVDFI<br>DEQKIAGIITKGREDI<br>PQWVTAYTVQFSKD<br>GVIWNKVKDDNGV<br>DQEFPANYDPFSTVT<br>NLFPNTVRARLLRIR<br>PTKWKNWISMQLEI<br>LGCYHPEPCRDPMG<br>IENGIITDHQLTASSE<br>ISESLSANNGRLTSPT<br>AWSPEISDKNPFLQV<br>DLLEPVNLTAVVTK<br>GNRELQQWVTSFNV<br>AYSNDSVDWKKVV<br>DTKGKIKEFIGNEDQ<br>DSAVISLLPIPVVARY<br>VRIIPLEMHRQIALR	AATATCTTGAGGTAAAATTCTCCGACGAAGGAACTATAACTG<br>GAGTCATAACGAAAGGCCGAGAAGATAAACCAAATTGGGTC<br>CGATCGTTTATGATTGCATACAGTGACGATGGAAAAAACTGG<br>GAGAAAGTGAAAAACGAAGTTGGCCAGCCGGAGATATTCCG<br>GGCCAACACCAACCAAGACGAACGCGTTACCAACATGCTTC<br>CGTCACCTGTACAGGCACAATACATTCGTTTCCATCCAGTCT<br>CATGGAAAATCAAAATAAGCATGCAAGTAGAAATTTTAGGA<br>TGTTTACCTGAAGAACCAGTGACAAAAGAAGAAGAAGAAG<br>AAGAAGAAGAAGAGACTCCTGAAATTGAAGAATGCAAGGA<br>ACCAATGGGCCTTGAAAATGGCTTATTGATGGACAGTCAACT<br>CTCGGCCTCATCCAGCTATAGTTCAAGGTTTACTCCAGACTC<br>GGCACGCCTAGGTTCTGACTCAGTTTGGGCAACTGCAGTTCA<br>AGATGATAATCAATATTTGCAGGTCGACTTCATTGACGAGCA<br>AAAGATTGCTGGTATAATCACGAAAGGCAGGGAAGATATAC<br>CGCAGTGGGTAACAGCTTACACTGTGCAGTTCAGCAAAGAT<br>GGTGTCATTTGGAACAAAGTAAAGGATGACAATGGTGTTGAT<br>CAAGAGTTCCCCGCCAATTATGATCCATTTTCTACGGTTACA<br>AACCTATTCCCAAATACTGTTAGAGCGAGACTTTTAAGGATA<br>CGTCCAACGAAATGGAAAAATTGGATAAGCATGCAGCTAGA<br>AATCTTAGGATGTTACCATCCAGAACCTTGCCGAGACCCTAT<br>GGGAATAGAAAATGGTATAATCACTGACCATCAACTGACAG<br>CTTCATCTGAAATATCCGAATCACTGTCTGCGAACAACGGAC<br>GTCTCACTTCTCCCACGGCTTGGTCACCTGAGATTTCTGACAA<br>AAATCCATTCCTGCAGGTTGATTTGCTTGAACCCGTCAACCTC<br>ACTGCAGTGGTAACAAAAGGCAATCGAGAGCTTCAACAGTG<br>GGTCACATCATTTAATGTTGCCTACAGTAATGATTCCGTGGA<br>CTGGAAGAAGGTGGTAGACACTAAGGGTAAGATAAAAGAAT<br>TCATTGGAAACGAAGATCAAGATTCAGCTGTCATCAGTCTAC

						MDLLGCFHEKVCLE<br>PMGLENGVLADAQI<br>TASSSSDPDTTPSHA<br>RLSDKSGWQPDKLD<br>THPYLQIDFLEPRNV<br>SAVVTKGMEDADN<br>WVTKFKVVVSDDRR<br>KWYPLITDDGDDVV<br>FKANEDRDTPVVNT<br>FPEEVEARFFRIVPLD<br>FENAVGLRVELLGC<br>YHPHECQEPMGMES<br>GSVFDFQISASSFLD<br>DEFSPAKARLDSDT<br>AWVADVNDDEPFIE<br>VDFLVPTNITGVMT<br>RGRNDANEWVKAY<br>KIEYSEDGVNNFVSD<br>KDAQGKEMEYLAN<br>SDSESTVTNTFEEPIL<br>ARLLRIIPVSYEKKIA<br>LRFEVLGCFHKTEC<br>MEPMGLENGLLEDS<br>HISATSYKSEDTNPT<br>KLRLNSDASWMPDT<br>IDDDQFIQVDFIDPK<br>NVSAVVTKGDATEP<br>VWITAYQVEYSEDG	TTCCCATCCCAGTCGTTGCGAGATATGTTCGAATCATTCCATT<br>GGAAATGCACAGACAAATTGCTCTAAGAATGGACCTTTTGG<br>GCTGCTTCCATGAAAAAGTCTGTCTCGAACCCATGGGTCTGG<br>AAAATGGCGTTCTTGCGGATGCACAGATAACTGCTTCATCAT<br>CATCTGATCCTGATACTACCCCGTCTCATGCCCGACTCAGTG<br>ATAAATCTGGTTGGCAGCCGGACAAATTAGACACTCATCCTT<br>ATCTTCAGATTGACTTCTTAGAACCCCGTAATGTCAGTGCAGT<br>GGTTACCAAGGGCATGGAAGACGCTGACAATTGGGTTACAA<br>AATTCAAGGTTGTGGTCAGTGACGACAGGCGCAAATGGTAT<br>CCCCTGATCACAGATGATGGTGATGATGTGGTATTTAAAGCC<br>AATGAAGACAGAGATACTCCTGTTGTCAACACTTTTCCAGAA<br>GAGGTAGAAGCTCGATTTTTCCGTATCGTTCCTCTCGATTTCG<br>AAAATGCTGTTGGCCTCCGAGTGGAACTTCTAGGATGTTATC<br>ACCCTCATGAATGTCAAGAACCCATGGGAATGGAGAGCGGC<br>TCCGTTTTTGATTTCCAGATTTCCGCTTCATCTTTCCTAGATGA<br>TGAATTCAGTCCAGCGAAAGCTAGGCTCGACAGCGATACTG<br>CTTGGGTAGCGGATGTAAATGACGACGAACCATTCATTGAG<br>GTTGACTTTTTGGTGCCAACTAATATAACTGGTGTAATGACTC<br>GAGGCAGAAATGATGCCAACGAATGGGTTAAAGCGTACAAA<br>ATTGAATACAGTGAAGATGGAGTTAACAATTTCGTTTCTGAT<br>AAAGATGCTCAAGGAAAAGAGATGGAATATCTAGCAAATTC<br>TGACAGTGAATCAACTGTTACCAATACTTTCGAAGAGCCAAT<br>ACTTGCTCGATTACTGCGCATAATACCAGTATCATATGAAAA<br>GAAGATAGCCTTGAGATTTGAAGTTCTAGGATGTTTCCACAA<br>AACTGAATGCATGGAGCCTATGGGTCTTGAAAATGGACTCTT<br>AGAAGACAGCCACATCTCTGCTACGTCCTACAAATCAGAAG<br>ACACGAATCCGACAAAATTACGCCTCAATAGTGATGCTTCCT<br>GGATGCCAGATACCATAGATGATGATCAATTCATTCAAGTCG

						NTWSSIEDTMGNIKE<br>FPANSDNDSPVINVF<br>PETVEARYIRIIPVKW<br>KNGIALRAEILGCYH<br>PYGEEAGTELTTLPEI<br>VMPYAGVYTEPPML<br>VACPDPMESEISLLT<br>GARIQASSSTPDGGP<br>TRVLLHTEAAKGQS<br>GGWVPNVDDLHPV<br>LFVQLPEKRWLTSM<br>YIQGREDEENWIKSL<br>RVVASPDNSTWMPV<br>FGPDGDEIIEANEDQ<br>ANIVGLYFKDPTEA<br>KYIKIIPETWHRWPS<br>FRLDLRGCRMPEAT<br>VLPVSTPAAYPELCP<br>ELLNYPQLAQYCPE<br>DCPEGQLCDGEGCV<br>DPVDCACVRDGKIY<br>KVSDRLEDRLCREC<br>DCALGGHSICSEKQ<br>CEPCEVGQTSVLND<br>DCSCTCEECPEGQIL<br>CPSDPQCIPEERWCD<br>GTIDCEDDEIDCFST<br>TEEPVVTVIGPDGIP	ACTTCATAGATCCCAAAAATGTGTCGGCTGTTGTCACCAAAG<br>GTGATGCAACTGAACCTGTGTGGATCACGGCATATCAAGTAG<br>AATACAGTGAGGACGGAAACACCTGGTCATCAATAGAAGAT<br>ACGATGGGAAATATTAAAGAATTTCCAGCAAACTCAGACAA<br>TGATTCCCCTGTTATTAACGTCTTTCCCGAAACAGTAGAAGC<br>AAGATACATTAGGATCATTCCTGTTAAATGGAAAAACGGAA<br>TAGCTTTACGAGCGGAAATTCTTGGTTGTTACCATCCATACG<br>GCGAGGAAGCCGGAACAGAATTAACAACTCTTCCGGAAATT<br>GTTATGCCTTACGCTGGTGTTTATACTGAACCCCCAATGCTTG<br>TTGCGTGTCCGGATCCGATGGAATCGGAGATAAGTTTGTTGA<br>CGGGTGCTAGAATTCAAGCAAGCTCCTCGACTCCCGATGGA<br>GGACCCACTCGAGTTTTACTGCATACAGAAGCCGCTAAAGG<br>CCAAAGTGGAGGATGGGTACCAAATGTTGATGATTTGCATCC<br>AGTTCTTTTTGTTCAGCTACCAGAAAAGAGATGGTTGACTTC<br>AATGTATATTCAAGGACGAGAAGATGAAGAAAATTGGATCA<br>AGTCTCTGCGTGTTGTTGCAAGCCCAGACAATAGCACTTGGA<br>TGCCTGTATTTGGTCCGGATGGAGATGAAATAATCGAAGCTA<br>ACGAAGATCAAGCAAATATAGTTGGATTGTATTTCAAGGACC<br>CCACAGAAGCCAAATATATTAAGATCATCCCAGAAACATGG<br>CACAGGTGGCCTAGCTTCAGATTAGACTTAAGAGGTTGTCGT<br>ATGCCTGAAGCTACAGTATTACCAGTCTCTACACCTGCTGCA<br>TATCCTGAACTATGCCCAGAGCTCCTTAATTATCCTCAACTTG<br>CTCAATATTGTCCAGAAGACTGCCCAGAAGGACAATTATGC<br>GATGGAGAAGGATGTGTAGACCCTGTAGACTGCGCATGCGT<br>GCGCGATGGAAAAATATATAAAGTGAGCGACAGACTAGAA<br>GATCGACTCTGCAGAGAGTGCGACTGCGCCTTGGGGGTCAT<br>TCAATATGCAGTGAGAAGCAGTGTGAACCCTGCGAAGTGGG<br>CCAAACATCAGTCTTAAATGATGACTGTAGTTGCACTTGCGA

						VAVVPPTEEGRLKP<br>AKKHRTKKPAGTTT<br>TPVVPFTVKPSVPGN<br>GTCNVMGNHVETF<br>DGQDIIYDVCNHVV<br>FKDIKNEKYNVTIHK<br>ECNYVSECKHWIEV<br>YYMNQHIKVQSDLN<br>VEVNGHSYTTSQLW<br>RLSKSTKMNLVAKK<br>VGEQVMITSLADSFT<br>IIYDIRGHLEIDVAPE<br>LLQKVSGLCGYYSG<br>RSDDDQRKPNGKK<br>AFTSREFGESWAIKE<br>RGKECKPLVCPHEQ<br>MSKAMELCHKLKER<br>PFIACGRLLPLNQFIE<br>FCMSTTCECLLNKK<br>NSENKCKCDGLLPF<br>VEACESKLGPMAVR<br>NWRLIHECHTECPP<br>GMEWNDCGPSCQL<br>TCDSTSEDTLQECSG<br>RCVPGCFCPAGTVL<br>HEEECIPPEECRDQV<br>CQGFGDPHFKTFDG<br>LFFPFQAVGSFLIVSD	GGAATGTCCAGAAGGGCAGATACTGTGTCCTTCAGATCCACA<br>ATGCATCCCTGAAGAACGATGGTGTGATGGTACAATAGACT<br>GTGAGGACGATGAAATTGACTGCTTCTCTACAACTGAAGAAC<br>CGGTGGTTACAGTGATAGGGCCAGATGGAATACCAGTTGCTG<br>TCGTACCACCGACTGAAGAGGGCAGACTGAAACCAGCGAAA<br>AAACATCGAACAAAAAAACCAGCTGGTACTACAACTACTCC<br>AGTTGTTCCATTCACTGTGAAACCCTCAGTCCCAGGAAACGG<br>TACGTGCAACGTCATGGGCAATCATGTGGAAACATTCGATGG<br>CCAGGATATTATATATGATGTATGCAATCACGTCGTCTTTAA<br>AGATATAAAAAATGAAAAATACAACGTTACAATTCACAAAG<br>AATGCAACTATGTGTCAGAATGCAAGCACTGGATTGAAGTCT<br>ATTACATGAATCAACACATAAAGGTACAATCCGATCTTAATG<br>TCGAAGTTAATGGCCATAGTTACACTACTTCACAATTATGGC<br>GTCTAAGCAAGAGTACCAAAATGAATCTAGTTGCGAAAAAA<br>GTAGGAGAACAAGTTATGATAACATCCCTTGCTGACAGTTTC<br>ACTATTATCTATGATATTAGAGGACACCTTGAAATTGATGTC<br>GCTCCAGAGCTGCTTCAGAAAGTTTCTGGCTTGTGTGGATAC<br>TATTCTGGAAGATCAGATGACGACCAACGAAAACCAAATGG<br>CAAAAAGGCTTTTACAAGCAGGGAATTCGGGGAGAGTTGGG<br>CGATCAAAGAGAGAGGAAAAGAATGCAAACCTCTTGTATGT<br>CCCCATGAACAGATGTCCAAAGCAATGGAGCTGTGTCACAA<br>ACTGAAGGAAAGACCTTTCATTGCGTGTGGAAGACTACTGCC<br>TCTGAATCAGTTCATTGAATTCTGCATGAGTACGACTTGTGA<br>ATGTCTACTGAACAAGAAGAACTCTGAGAACAAATGCAAAT<br>GCGACGGTTTGTTACCTTTCGTAGAAGCTTGCGAATCCAAGC<br>TAGGGCCGATGGCAGTCAGGAACTGGAGACTTATACATGAA<br>TGTCACACGGAATGTCCTCCTGGAATGGAATGGAATGACTGT<br>GGTCCATCCTGCCAGTTAACTTGCGATAGTACTTCTGAAGAC

						TGKNFEVKGLSKKC<br>GLFNPITCLVGMEIS<br>YKRHKISIQKSKELRL<br>DRKRLSMDKLPLYE<br>DGMVILGYPGRTFVI<br>VIPSINFEARYYEENS<br>GFAVRVPSRRYFNKT<br>RGLCGNCNGRQDD<br>ELKNEPLNEYVCSW<br>QTDGNKEDCMKEL<br>EELPELAQRVPICEK<br>LKDPVFEACHPLIDV<br>NIYIDACNFDATHS<br>GDNQASFCKTAMEY<br>GRQCCQADVSIEDW<br>PRELGCDIDCPSGMS<br>FEQCHKGCPQTCLS<br>KKNESGDSSCKQLR<br>TDGCFCPEGKILKDD<br>GCVDIELCETCDEEG<br>HVPGDKWDDGPCK<br>HCECHEDRTVECIV<br>DNCPHGPVCSNLET<br>YVKQVNEEKESCCE<br>SYVCVPKEEKCPTM<br>ELPKCKKGEVAKVH<br>TSEEDTCPVLKCECD<br>PLLCAPLVWPEDLE	ACGCTTCAAGAATGTTCTGGCAGGTGTGTTCCAGGTTGCTTCT<br>GTCCCGCAGGAACAGTGCTTCACGAGGAAGAGTGTATTCCA<br>CCAGAAGAGTGTCGTGATCAAGTATGCCAAGGATTTGGCGA<br>TCCACACTTCAAAACGTTCGATGGACTGTTTTTCCCGTTCCAA<br>GCAGTGGGATCATTTTTGATCGTCAGCGATACAGGCAAGAAT<br>TTTGAAGTCAAGGGACTGTCGAAGAAGTGTGGTCTATTTAAT<br>CCCATAACTTGTCTTGTGGGAATGGAAATCTCTTATAAGAGG<br>CACAAGATATCGATTCAAAAGAGCAAAGAGCTTCGATTAGA<br>CAGAAAGAGATTATCAATGGACAAGCTACCTTTGTACGAAG<br>ATGGCATGGTTATCCTCGGGTACCCTGGGAGAACATTTGTTA<br>TAGTTATTCCATCGATCAACTTTGAAGCTAGATATTACGAAG<br>AAAATTCAGGATTTGCAGTAAGAGTGCCATCCAGAAGATAC<br>TTTAATAAAACAAGAGGATTGTGCGGTAACTGCAATGGCCG<br>GCAAGATGACGAATTAAAGAATGAACCACTTAATGAATACG<br>TTTGTAGCTGGCAAACCGATGGTAACAAAGAAGACTGTATG<br>AAAGAACTCGAAGAACTTCCCGAGTTAGCTCAGCGTGTACC<br>AATCTGTGAAAAACTCAAAGATCCAGTATTTGAAGCTTGTCA<br>TCCTCTCATAGATGTGAATATTTATATCGACGCATGTAATTTC<br>GATGCTACCCACAGTGGGGATAACCAGGCGTCTTTCTGCAAA<br>ACAGCTATGGAATATGGCAGGCAGTGTTGCCAAGCTGATGTT<br>AGTATAGAAGACTGGCCTCGGGAATTAGGATGCGATATCGA<br>TTGCCCGTCTGGTATGTCCTTCGAACAGTGTCATAAAGGATG<br>CCCTCAGACATGTCTTAGTAAGAAGAATGAATCCGGTGATTC<br>ATCTTGCAAACAACTGCGAACAGACGGGTGTTTCTGCCCTGA<br>AGGAAAGATCCTGAAAGATGATGGATGCGTCGATATTGAAT<br>TATGCGAAACATGTGATGAAGAAGGCCATGTGCCTGGTGAC<br>AAGTGGGACGATGGACCATGCAAACATTGCGAGTGTCATGA<br>AGACCGAACTGTTGAATGCATTGTGGACAACTGCCCCCATGG

						DGQKAVMANDSCC<br>PLVEVICFDEFCPDK<br>PYCEEHLELVEAKGE<br>CCTTYKCKPPDDVC<br>AYSHMFKIVGGFQS<br>LVEQIEGDTTIYQPG<br>EEWEDGLCQKCSCI<br>ESNGQHVPLCVME<br>HCTTLEDHPENKEY<br>VLQEMKIPDKCCPDI<br>FKIKCRDDDGNVYE<br>PGEQWQDEDDPCV<br>TFLCEQEGTGEVLKI<br>KSVINCTECPRTAILF<br>PPSKALGQCCGFCK<br>PSKCEDGEDLHEVG<br>DTWPNPDDACKMA<br>ECVSEYGKISVVYTK<br>QPCPKIPEDCPPDQI<br>VLDENECCQVCNLT<br>SEVSCSSGPIPDEETV<br>GFFKYNEPSEGGICV<br>NERPVSKVLQCKGA<br>CHSQSFYSLEDGDFK<br>DTCKCCKVNVTTNR<br>TVELKCPNGNYIRKT<br>FLQPETCQCSKCAPK<br>SGDSKQNSFKPQSP	ACCTGTCTGCTCAAATCTCGAAACCTACGTTAAACAAGTCAA<br>CGAAGAAAAGGAATCATGCTGTGAATCCTACGTATGCGTGC<br>CTAAGGAAGAAAAATGCCCGACCATGGAATTACCCAAGTGC<br>AAAAAGGGAGAAGTAGCAAAAGTGCACACAAGTGAAGAGG<br>ATACTTGCCCCGTTCTAAAATGCGAATGTGATCCACTTTTATG<br>TGCTCCCCTTGTGTGGCCAGAAGATTTAGAAGATGGTCAAAA<br>AGCAGTCATGGCAAACGACTCTTGTTGTCCTCTTGTCGAAGTT<br>ATCTGTTTCGATGAATTTTGTCCTGACAAACCATACTGTGAAG<br>AACATCTGGAACTTGTCGAAGCGAAAGGAGAATGTTGCACT<br>ACTTATAAATGCAAGCCTCCAGATGATGTTTGTGCATATTCTC<br>ATATGTTCAAAATCGTGGGTGGCTTCCAGTCACTTGTGGAAC<br>AAATAGAAGGGGATACTACTATATATCAGCCGGGAGAGGAA<br>TGGGAAGATGGCCTATGTCAGAAATGTAGCTGTATTGAAAGC<br>AATGGACAGCACGTGCCACTGTGCGTCATGGAGCATTGTACC<br>ACCCTGGAAGATCATCCTGAAAATAAGGAATATGTTCTGCA<br>AGAGATGAAAATACCGGATAAATGCTGTCCAGACATTTTTAA<br>AATTAAATGTCGAGACGATGATGGAAATGTGTACGAGCCAG<br>GGGAACAGTGGCAAGATGAAGATGATCCTTGCGTAACATTC<br>CTTTGCGAACAAGAAGGAACTGGAGAAGTCCTCAAAATCAA<br>AAGCGTCATCAACTGCACGGAATGTCCTAGAACCGCTATTCT<br>CTTCCCGCCCTCAAAAGCACTGGGGCAGTGTTGCGGTTTCTG<br>CAAGCCGTCCAAATGTGAAGATGGAGAAGATTTGCATGAGG<br>TTGGAGATACGTGGCCCAACCCAGATGATGCCTGTAAAATG<br>GCAGAATGTGTGAGCGAATACGGAAAGATTTCGGTTGTCTAT<br>ACGAAACAGCCATGCCCAAAAATTCCGGAAGATTGCCCTCC<br>GGACCAAATTGTTTTGGATGAAAATGAATGCTGTCAAGTTTG<br>CAATCTAACTAGTGAGGTCAGTTGCAGTTCGGGTCCTATACC<br>AGATGAGGAAACCGTAGGCTTTTTCAAGTACAACGAACCAT

						WNQQNQWYTESEF VIAQRPVDNERFTQ QRDIDEDIQQLQQN QVQQQVEFEDI*	CGGAAGGCGGAATTTGTGTTAACGAAAGACCTGTTTCTAAAG TTCTCCAATGCAAAGGCGCTTGCCACTCCCAATCTTTCTATTC ACTAGAGGATGGAGATTTCAAAGATACGTGCAAGTGCTGCA AAGTCAATGTCACAACAAATCGTACTGTTGAACTCAAATGTC CCAATGGCAACTATATTCGAAAAACTTTCCTTCAACCAGAAA CGTGCCAGTGCTCGAAATGTGCCCCGAAGTCAGGAGATTCC AAACAAAACAGTTTCAAACCACAGTCACCATGGAATCAACA AAATCAGTGGTATACTGAGTCTGAATTCGTAATTGCTCAGCG ACCTGTAGATAACGAACGCTTCACACAGCAGAGAGATATTG ATGAAGATATCCAACAGTTACAGCAAAATCAAGTGCAACAG CAAGTGGAATTCGAAGACATTTAAAACGACTATATTGATGTA CACGTAGTTGATGCAAACGACAAATTAATCAATGTGACTTGA AAGTGATACTCTATTTAATAAAGTCACAAGTTGGTTTAAAAA AAA

Peptidase inhibitors							
**Transcript ID**	**Uniprot ID reference protein**	**e-value**	**Identity (%)**	**PFAM domain**	**MW (Da)**	**Amino acid sequence**	**Nucleotide sequence**
PhdPInPep01	Q5Y4V9	1,73E-10	38,824		9194.68<br>**7019.97**	MKQAVFLIFTFALVCTMASA**EEGYCPERSQKVCIRNQILCCSDNDCEAGKVCCYENCGTECRPVSSKPTSGRKVVPDKNCKKE**	GTTTCTTTTTTGCAGTTCTTGTCAGGAACCACTTTTCGTCCACTGGTTGGTTTTGAAGAGACTGGTCGACACTCGGTACCGCAGTTCTCGTAACAACATACTTTTCCAGCTTCACAGTCGTTATCCGAACAGCACAAGATCTGATTTCGAATACACACTTTTTGGCTTCTCTCAGGACAGTAGCCTTCTTCTGCTGAAGCCATAGTGCATACTAAAGCAAACGTAAAGATAAGGAAGACAGCTTGCTTCATCTTTTGATAGTTAGGTAACCAGAATTTAGAGACACTTCTTGAAAAGTAACGTCTGCTTCAAGAGATAAAGAGAAGCAGACAGATGCTCTGAGTCCAAGCCCCTAATTATATCGAAACCCGAGGATTGAAATTTCACTTTTCTTTCCACTCACTTCCAATCATATATCTCCTTCTTGCTGCGCCTTCCAGTGCCAAGCTACTGCCGAGATTCGAACGTCTGCATTCAAGGACTTTCGCCCAGGTAATTCTGTGCAAGCCGTCACTGTTG
PhdPInPep02	Q5Y4V9	1,05E-10	38,824		9194.68<br>**7019.97**	MKQAVFLIFTFALVCTMASA**EEGYCPERSQKVCIRNQILCCSDNDCEAGKVCCYENCGTECRPVSSKPTSGRKVVPDKNCKKE**	GTTTCTTTTTTGCAGTTCTTGTCAGGAACCACTTTTCGTCCACTGGTTGGTTTTGAAGAGACTGGTCGACACTCGGTACCGCAGTTCTCGTAACAACATACTTTTCCAGCTTCACAGTCGTTATCCGAACAGCACAAGATCTGATTTCGAATACACACTTTTTGGCTTCTCTCAGGACAGTAGCCTTCTTCTGCTGAAGCCATAGTGCATACTAAAGCAAACGTAAAGATAAGGAAGACAGCTTGCTTCATCTTTTGATAGTTAGGTAACCAGAATTTAGAGACACTTCTTGAAAAGTAACGTCTGCTTCAAGAGATAAAGAGAAGCAGACAGATGCTCTGAGTCCAAGCCCCTAATTATATCGAAACCCGAGGATTGAA

							ATTTCACTTTCCTATTCCTTTCTCTCACCCAATCAGAACTTCCT TGTCCTGCTCACTATGCCATTGG
PhdPInPep03	B6DCY1	1,50E-06	47,727		10963	SGIVTCNFIVIKITSPI FLQLDETFARSEKAV SVPLNSGGFCPPRRI RVCVWAQNQCCSN GDCPAGQLCCEENC GNECRPSQQWQSN GRLVPFRPACTIP*	TCTCGTTTCACTTTATTAAAAACATTTTGCTAAGCTCGTGTTC ACCTCTATTTCACCTCATTCTACGAAATGCTATCTCGCTCTAT AACAACAGGGAAGAATGTGATTACGGCAGGATACTCTATTG TTCTGTTGATGGAATAAATACAGATATATTTCAGCGAAAGTA AACATTATCGTCGATCAGGTGATCAGGAATAGTTACCTGCAA TTTCATTGTAATCAAAATAACTAGCCCAATTTTTCTTCAATTA GACGAGACATTCGCCCGCTCAGAAAAAGCAGTTTCAGTGCC TCTAAACTCAGGGGGATTCTGTCCTCCGAGGCGCATAAGGGT TTGCGTCTGGGCGCAGAACCAGTGTTGTTCAAATGGAGACTG TCCAGCTGGGCAGCTCTGCTGTGAGGAGAATTGCGGTAACG AGTGCCGACCTTCCCAGCAGTGGCAAAGCAATGGACGCCTG GTGCCATTTCGTCCTGCGTGCACAATACCTTAATCATGATCTG TCGATTCTTGTTAATGATAACTGATAATCTTCTAATAAAAGCT CTAG

PhdPInPep04	Q5Y4V9	3,53E-07	39,394		9385.96<br><br>**7283.29**	MKRAVVFLTLIIALACVTSA**QRRGGFCPPRRIRVCVWAQNQCCSNGDCPAGQLCCEENCGNECRPSQQWQSNGRLVPFRPACTIP***	TCTCGTTTCACTTTATTAAAAACATTTTGCTAAGCTCGTGTTC<br>ACCTCTATTTCACCTCATTCTACGAAATGCTATCTCGCTCTAT<br>AACAACAGGGAAGAATGTGATTACGGCAGGATACTCTATTG<br>TTCTGTTGATGGAATAAATACAGATATATTTCAGCGAAAGTA<br>AACATTATCGTCGATCAGGTGATCAGGAATAGTTACCTGCAA<br>TTTCATTGTAATCAAAATAACTAGCCCAATTTTTCTTCAATTA<br>GACGAGACATTCGCCCGCTCAGAAAAAGCAGTTTCAGTGCC<br>TCTAAACTCAGGCTAAACAATTCTTAAACGATGAAGAGAGC<br>TGTCGTTTTCCTGACCCTTATTATTGCTCTGGCATGCGTCACAT<br>CGGCGCAGAGAAGAGGGGGATTCTGTCCTCCGAGGCGCATA<br>AGGGTTTGCGTCTGGGCGCAGAACCAGTGTTGTTCAAATGGA<br>GACTGTCCAGCTGGGCAGCTCTGCTGTGAGGAGAATTGCGGT<br>AACGAGTGCCGACCTTCCCAGCAGTGGCAAAGCAATGGACG<br>CCTGGTGCCATTTCGTCCTGCGTGCACAATACCTTAATCATGA<br>TCTGTCGATTCTTGTTAATGATAACTGATAATCTTCTAATAAA<br>AGCTCTAG
PhdPInPep05	Q5Y4V9	3,33E-07	39,394		9385.96<br><br>**7283.29**	MKRAVVFLTLIIALACVTSA**QRRGGFCPPRRIRVCVWAQNQCCSNGDCPAGQLCCEENCGNECRPSQQWQSNGRLVPFRPACTIP***	TCTCGTTTCACTTTATTAAAAACATTTTGCTAAGCTCGTGTTC<br>ACCTCTATTTCACCTCATTCTACGAAATGCTATCTCGCTCTAT<br>AACAACAGGGAAGAATGTGATTACGGCAGGATACTCTATTG<br>TTCTGTTGATGGAATAAATACAGATATATTTCAGCGAAAGTA<br>AACATTATCGTCGATCAGGTGATCAGGAATAGTTACCTGCAA<br>TTTTATTGTAATATTACTGAATTAGATGTTATATTCGCACACT<br>CATAAAAAGCAGTTTCAGTGCCTCTAAACTCAGGCTAAACA<br>ATTCTTAAACGATGAAGAGAGCTGTCGTTTTCCTGACCCTTAT<br>TATTGCTCTGGCATGCGTCACATCGGCGCAGAGAAGAGGGG<br>GATTCTGTCCTCCGAGGCGCATAAGGGTTTGCGTCTGGGCGC<br>AGAACCAGTGTTGTTCAAATGGAGACTGTCCAGCTGGGCAG<br>CTCTGCTGTGAGGAGAATTGCGGTAACGAGTGCCGACCTTCC

							CAGCAGTGGCAAAGCAATGGACGCCTGGTGCCATTTCGTCCTGCGTGCACAATACCTTAATCATGATCTGTCGATTCTTGTTAATGATAACTGATAATCTTCTAATAAAAGCTCTAG

PhdPInPep06	W4VSH9	3,79E-24	37,879	PF00014	72872,81	FEENTPEAFSIEFCKF<br>EPDAGPCSGRKIQW<br>FYDKDGVCKQFYYG<br>GCRGNGNRFNTKK<br>DCEVKCAASQDICK<br>LPKIRGPCSGSFVQW<br>YFDSEHKECREFSYS<br>GCQGNGNRFNEKD<br>TCEIQCKKWPAADS<br>HESQDACSLPKDPG<br>PCLGYFLMWYHDA<br>DENACRSFIYGGCH<br>GNDNRFTTMQECE<br>AKCHTNSAINIRKA<br>KEKQSSTAAASFITN<br>EDICRQAPDEGHCG<br>YSQSRWYYDPHKG<br>MCLPFVYKGCGGN<br>KNRFKSDDLCMRFC<br>AGVQGSEVVGDAA<br>ARDVVPFEINKNCP<br>ASNCATLQCPHGIE<br>EIVDVRGCISCLCSN<br>PCENHNCPEGSQCV<br>VEKYRKSDGEFRSQP<br>LCRLKQKTGTCPSSP<br>SDHLLNGSCHDRCQ<br>SDADCPGGLKCCFV	GATGAATTATGATACTTGGCTTCAAGAAACTTTTAAGATTAA<br>ACTGAAAATTTAAGTGCATAAGAAAAATTTATTGTAACAGG<br>ATTTTTGCTTCCATTTTCTCTATTTACAAAGTTAATATGAAAAT<br>ATGTAATAAAATTTTTATAAGTTGTTAATTAAAAACACATAG<br>TGAAGAAATCATTGAATGCTATATAAAACCTTTACACTGGAG<br>CAACAAGTATTTCCAACTGTATTTCTTCAAGAGTCATTTTTAA<br>ATATTCAGCACAAATCTCATACTTTTTGAATTAATTCCAAAA<br>GCAGTATAGTAGCAAAAAGTGTCAACATTACACAGTTTTGTA<br>CAACAATGATGAAATCCAAATGAATGTCAAGTTCTGAGTCCT<br>CAACAACCAGACTGTGAGAAAAATGTCTTTCATAGTCTCGAT<br>GAACTCTTTTAATTTATTAACTATTTTTAGTGTGGGTTGACTAT<br>GAGTTACCAACATTATCTGTGTGGAGTTGGCCTGCCAATGCA<br>CAGGAGAGACAACAGAATTTGGCATATGTTGGATTGATGCA<br>ATAGTTTTGTTTCACAATCATGGCACAGCTGGTGAAAAATGG<br>ACTGTCTTTACATGTCGAAGGTACAGAAATATCATGAACTTG<br>TATATTTATTGAAGCTGACGCTTTTCCATGCTGGTTCTGAGCT<br>TCACAGATATAATCACCACTGTCATTATATTGAGCGGATGAA<br>ATGAACAAAGTATGATTACTGAAGATCTCTCTGTGCTCATTA<br>GATTCCAGTGGTTTGCTATCTTTTCCGAGGTGCCATGTTACTT<br>CCATAGGGGGTGTTCCACTTACAATACAGTTCAACTGCAGAG<br>GAGTGCCTAGTTTGTAGGCTGGTGTTATGAGGGATATATTTAC<br>TGTAGTGTCCTCAAATACAATCAAATTGAAAAATTGTGTTAC<br>TGCTTTTCCTACACCATTGTCAGCTGTACAAGAATACATTCCT<br>TCATTCGAGTTGTCCACCGACATTATATGCAAGGAGCCATCA<br>ACAGATATTTTATAATCACCAGCTTCAGTGTGTAGTGGATGA<br>TTCTTATGATGCCAAGTTACTCTCGGTGAAGGATAACCTTTGA<br>CAATGCATCTCAACAGAGCAGGGGTTCCTTTTTTAACAGCAA<br>CATCTCTCCTAGCAGCCAAAATTTGAGGTGGTGTTGGAGTTT

						SCAKICVLPVLPDEEYSVEENSIEDTETPTPPQILAARRDVAVKKGTPALLRCIVKGYPSPRVTWHHKNHPLHTEAGDYKISVDGSLHIMSVDNSNEGMYSCTADNGVGKAVTQFFNLIVFEDTTVNISLITPAYKLGTPLQLNCIVSGTPPMEVTWHLGKDSKPLESNEHREIFSNHTLFISSAQYNDSGDYICEAQNQHGKASASINIQVHDISVPSTCKDSPFFTSCAMIVKQNYCINPTYAKFCCLSCALAGQLHTDNVGNS*	CAGTATCTTCAATGCTGTTTTCTTCAACAGAATATTCTTCATCTGGCAGAACTGGCAAAACACAAATCTTTGCACATGAAACAAAGCAACATTTCAAACCACCCGGGCAATCAGCATCGGACTGGCATCGATCGTGGCACGAGCCATTCAGTAAATGATCAGAAGGAGATGATGGACATGTACCTGTCTTTTGTTTTAAACGACACAAAGGCTGTGATCTAAATTCTCCATCACTTTTTCGATACTTCTCAACCACACACTGAGATCCTTCAGGGCAGTTATGATTCTCGCATGGATTTGAGCATAAGCAAGAAATGCATCCTCTCACATCAACTATTTCTTCAATGCCATGAGGACATTGTAAAGTTGCACAATTTGAAGCAGGACAGTTCTTATTGATTTCAAATGGTACCACATCCCTCGCAGCAGCATCACCAACAACTTCTGAACCTTGGACTCCAGCACAGAAACGCATGCACAGATCATCACTCTTGAATCTGTTCTTATTTCCTCCGCAACCTTTGTAAACAAAAGGCAAGCACATTCCCTTATGGGGATCATAATACCAACGGCTCTGAGAATAACCACAGTGACCTTCATCGGGAGCTTGTCGACAGATATCTTCATTTGTAATAAATGAAGCAGCAGCAGTAGATGATTGTTTTTCTTTAGCTTTTCTTATATTAATTGCACTGTTCGTGTGACATTTCGCTTCACACTCCTGCATAGTTGTAAATCTATTATCATTTCCGTGGCATCCACCATATATGAAACTTCTACAAGCATTTTCATCCGCATCATGGTACCACATGAGGAAATATCCCAAGCATGGACCTGGATCTTTTGGTAGACTGCAAGCATCCTGAGATTCGTGACTGTCAGCTGCAGGCCATTTCTTGCACTGAATTTCACAAGTATCCTTCTCATTGAACCTATTCCCATTGCCCTGGCAACCACTGTATGAAAATTCTCTGCATTCTTTGTGTTCTGAATCAAAATACCACTGCACAAATGAACCACTACAAGGACCCCTTATTTTTGGAAGCTTGCAAATATCTTGAGAAGCAGCACATTTGACTTCACAGTCTTTCTTAGTGTTGAATCTATTACCATTTCCTCTACAACCTCCATAATAAAACTGCTTGCAAACACCATCTTTGTCATAGAACCATTGAATTTTTCT

							ACCACTGCAAGGACCAGCATCTGGCTCAAATTTGCAGAATTC TATACTAAATGCTTCGGGAGTATTTTCTTCAAAG

PhdPInPep07	Q6T269	2,95E-30	36,364	PF00014	43009,19	PYGCCHDGTTPARA<br>PNFGGCQPSTTDVC<br>SMPKERGPCHNFSV<br>KWYFDVTYGGCSRF<br>WYGGCDGNGNQFN<br>SQEECERVCVTPEGP<br>QACSLPKVVGPCKG<br>SYQNWYYEPETDSC<br>HTFNYSGCLGNNNR<br>FESKKMCESTCVKQ<br>AVLDACEQPMDVG<br>PCKTITQRWFFDRAE<br>DVCKPFLYGGCKGN<br>HNNFETEKECMLRC<br>VTSPPKDPTTVYNAS<br>VGSRVTMVCKAMPS<br>TVDDEPILFLWYKG<br>DSAYPIHGVDKREES<br>GGKSLVASRGTDPRF<br>AIQHNDLMTALMIN<br>KIRREDEGQYRCRV<br>DYKIGPTTNRFMRL<br>HIEEPSICNLPHAVG<br>NCYQFRERWYYNSH<br>EKKCQRFYYSGCGG<br>NENNFATYLSCEKQ<br>CEKSFEENTPE	CTTCGGGAGTATTTTCTTCAAAGGACTTCTCGCATTGTTTTTC<br>ACATGATAAATAAGTAGCAAAATTATTTTCATTGCCTCCACA<br>ACCACTGTAGTAAAATCTTTGGCATTTCTTTTCATGAGAATTA<br>TAGTACCATCTTTCTCTAAACTGGTAACAGTTACCAACTGCAT<br>GGGGCAAATTACAGATACTAGGTTCCTCGATATGTAGCCTCA<br>TAAATCTGTTAGTGGTAGGTCCAATTTTGTAATCAACTCGGC<br>ATCTATATTGCCCTTCATCTTCCCTCCTAATTTTGTTGATCATC<br>AAAGCTGTCATAAGATCATTATGTTGAATGGCGAATCTTGGA<br>TCAGTTCCCCGACTAGCAACTAGGCTCTTTCCTCCACTCTCCT<br>CTCTTTTATCAACACCATGAATAGGATATGCCGAATCACCTTT<br>GTACCATAAAAATAATATAGGTTCATCATCCACTGTTGAAGG<br>CATGGCTTTGCAGACCATAGTTACCCGTGAACCAACACTAGC<br>ATTGTATACAGTTGTAGGATCTTTAGGTGGGGAAGTAACACA<br>TCGCAGCATACATTCCTTTTCAGTTTCGAAATTATTATGATTT<br>CCTTTGCATCCTCCATATAAGAAAGGCTTGCAGACATCTTCG<br>GCTCTGTCAAAGAACCACCTCTGGGTAATCGTTTTGCAAGGT<br>CCAACATCCATTGGTTGCTCACAAGCATCTAAAACAGCTTGT<br>TTGACGCAAGTGCTTTCACACATTTTCTTGCTTTCAAATCTATT<br>ATTATTACCAAGACAACCACTATAGTTGAATGTGTGGCAGGA<br>ATCTGTTTCAGGTTCATAATACCAATTCTGATAAGAACCTTTA<br>CATGGTCCAACAACTTTAGGCAAGGAACAGGCTTGTGGCCCT<br>TCAGGAGTAACACAAACTCTTTCACATTCTTCTTGAGAGTTG<br>AACTGATTGCCATTTCCATCACAACCACCATACCAGAAACGA<br>CTGCAGCCACCATACGTAACGTCAAAGTACCATTTAACACTA<br>AAATTATGGCAAGGTCCTCTTTCTTTTGGCATGCTACACACAT<br>CAGTAGTACTAGGTTGGCATCCACCAAAGTTTGGAGCTCTTG<br>CTGGAGTAGTACCATCATGACAACATCCATATGG

PhdPInPep08	P0DQE8	1,66E-09	35,556		12210.98<br>**9296.33**	MVCSHGVAPCSSALLCVLLLVLIVTMTTG**QINPNEQPCLAAGGVCAHESACSEDRRANGTRLCPTQEDQGAVCCHGVPDDETSCRGHGGECLSAARCGNSKILGQFECKEGTNCCLLI*****	TTTTTTTTGATATTTGAAGTATAACTTTAATATTTCATTTCTTATTTCCCTTTATACAAATTACAATCCTGTTAATGACATATATAGAGATTCAGTGCTGAATATGCTTCTTTATTAGATTAAAAGGCAACAATTGGTCCCTTCTTTGCACTCGAATTGCCCTAATATCTTAGAATTGCCACATCTCGCTGCTGACAAACATTCGCCTCCATGTCCTCTGCAAGATGTTTCATCATCCGGAACACCATGGCAACAGACAGCTCCTTGGTCTTCTTGCGTTGGACAAAGTCTGGTCCCATTCGCCCTTCTGTCTTCCGAACAAGCACTTTCGTGTGCGCACACACCGCCTGCTGCTAAACACGGCTGTTCGTTCGGATTGATTTGTCCTGTTGTCATGGTGACGATGAGGACGAGAAGCAAAACACAGAGCAACGCAGAGCTGCAGGGAGCCACACCATGCGAGCACACCATAGCTGCCAGCACCAAGCTTCCCGATGAGAGCGTACAAATATGTCCAGGTCAGAACGTTTCTAGCGGGCCGTCTGTTCTCTTCTGTATCAGAGAGCGAAGTGCTGCTTCCACTTTTACGATGAATTCCTCAATAATGCTGTTTACAAGGCCTCCAATCTCGAGGCCACTGTTTCAAAACTGCATCATTGTATTGTTAAGCTAGGCAAGGAGACCTTTATAGTCTCTTGCCACGGATGAG
Cystein protease inhibitors							
**Transcript ID**	**Uniprot ID reference protein**	**e-value**	**Identity (%)**	**PFAM domain**	**MW (Da)**	**Amino acid sequence**	**Nucleotide sequence**

PhdPInCyP 01	B1P1J3	7,29E-20	31,343	PF00031	15462.68<br><br>**13540.31**	MNLLVFVIMATLYQ<br>ANV**GEMVGGWKN**<br>**VTNPNSAEFQNIMY**<br>**FAAGKITDSLDSQY**<br>**RMRPTELLSAKQQL**<br>**VAGMKYEFNMKLQ**<br>**LTECKTNENSDVDI**<br>**KECRYLRCGAVQN**<br>**CNAVVWTRSWLNE**<br>**TTLTNFSCNKETSS**<br>**C***	CTATTATTTAAATTTAAATAGACAAGCGCTGCAATCACAGCA<br>ACAAAATACGTCACAGCTACCGTAATAAACGGTACCGCCCA<br>ATGACCAATCAGATAAAGAGTGCGTATTTTCCGGGAGTTGAT<br>TTTAGTGTAATTTGCACGCCTCAAAATTTCTCGTTTCAGTAAC<br>CGTGGCTCTGTTGTGTTTACATTGTAGAAACGTCATGGTGCAG<br>GAAGTTGTATAAATTTATTGTAGTTTAGAAACCGTGAGTTTCT<br>TAAAATCTGAACGTGCAGTATAGGAAAAACTGTTTCTTTGTA<br>AAGATGAATTTGTTAGTATTTGTTATTATGGCCACTTTATATC<br>AGGCTAATGTGGGGGAGATGGTAGGTGGATGGAAAAATGTT<br>ACTAATCCGAATTCTGCAGAATTTCAAAATATAATGTATTTTG<br>CTGCGGGGAAAATAACTGATTCATTAGATAGCCAGTACAGA<br>ATGCGACCTACTGAACTTTTATCTGCTAAGCAACAGCTTGTTG<br>CTGGAATGAAATATGAATTCAATATGAAACTTCAATTAACAG<br>AATGTAAAACAAATGAAAATAGTGATGTTGACATCAAGGAA<br>TGTAGATATTTACGATGTGGAGCTGTCCAGAACTGTAATGCT<br>GTTGTGTGGACCAGATCCTGGTTAAATGAAACTACACTTACC<br>AATTTTTCTTGCAATAAAGAAACTAGTTCCTGCTAAATATTCA<br>ACAATAAATGAACTGTAATTGGATTCTGAAATTTTGCATTAT<br>GTAATTTTTTACAGTTATAATCTGTTAATCTTAGTGAAATTAC<br>CCCCAGCTTCAATTTATAAACTAATTTCTTGTTCATAAAAATG<br>ATTAATCTATTCATATTTGGAGTTTTTAATATATTTAAAACTTT<br>TATTTATTAACTCTAATATATTGATGCATTTCTCGGATCTTATT<br>GTTTCAATGTTATATATATATATACTCGGTGAAATATTTATAA<br>ATGTATAGTATTTTTTGTAATTATGAG

PhdPInCyP 02	B1P1J3	2,65E-14	34,409	PF00031	15018.4<br>**13024.90**	MWKALVLFGFLAV<br>ALCASA**GLLGGFQ**<br>**DVNVDDEGVVKA**<br>**ANEASRQLSKQFA**<br>**GKYHHRLTKVLKA**<br>**KQQVVAGMNYQM**<br>**DIVVGMTTCKKDE**<br>**VEFDDAQDCDFQD**<br>**SISTYKKCTVTVFM**<br>**DLKGKHKLSSSGCI**<br>**LASKKDL***	GCGAGCTGTCCGTTTTCCGTGGGAAAGGCACCGTCTGCTTCC<br>TTCCGCATAAGAGCGGGCGGCTTGCCACGGCCGGGCACTCTC<br>TTTTGCTCTTCTAGACTCGACACACCGCAAACATGTGGAAAG<br>CTTTAGTCCTCTTCGGATTCTTAGCGGTCGCTTTGTGCGCCTC<br>GGCCGGCCTCTTGGGTGGGTTCCAGGATGTGAACGTGGACGA<br>TGAGGGCGTCGTTAAAGCCGCCAACGAGGCCTCACGCCAGC<br>TTTCCAAGCAATTCGCCGGCAAATACCACCACAGGCTTACAA<br>AGGTCCTGAAAGCCAAGCAACAGGTCGTTGCTGGCATGAAC<br>TACCAGATGGACATCGTCGTCGGCATGACCACCTGCAAGAA<br>GGACGAAGTTGAGTTCGACGACGCCCAAGACTGCGACTTCC<br>AAGATAGCATAAGCACCTACAAGAAATGCACGGTAACAGTC<br>TTCATGGACCTAAAGGGCAAACACAAGCTGTCCTCCTCCGGA<br>TGCATCCTCGCCTCCAAGAAAGACCTTTGAACGCTTAGTTGT<br>CATCTTTTACTTCGTATGTAATAATAAACAGTAAAACCGACT<br>CGAA
PhdPInCyP 03	B1P1J3	2,67E-11	30,088	PF00031	15223.55<br>**12983.80**	MWKALLFFGFLVVA<br>WSATVA**IPGGYND**<br>**AKVDDEGVVKAA**<br>**NEATRQLSKNFASK**<br>**YHHTLRKILKAEIQ**<br>**VVAGLNYRLKIIVG**<br>**MTNCKKEEVKFDD**<br>**AQDCEYQDGVSTY**<br>**KKCEIIVYRALTGE**<br>**HSLKASGCTLASKN**<br>**EV***	TGAAGGAGAAGTACAAGCGAAGCGTTCAGTTCAGACTTCGT<br>TCTTGGAGGCGAGCGTGCAACCGGAGGCCTTCAGAGAGTGTT<br>CGCCGGTCAGGGCCCTGTAGACTATGATTTCGCATTTCTTGTA<br>GGTACTCACGCCATCTTGATATTCGCAGTCCTGGGCGTCGTC<br>GAACTTGACTTCTTCCTTCTTGCAGTTGGTCATGCCGACGATG<br>ATCTTCAGTCTGTAGTTCAGGCCAGCAACAACCTGTATCTCG<br>GCCTTCAGGATCTTTCTAAGCGTGTGGTGGTATTTGCTGGCGA<br>AATTCTTGGAAAGCTGGCGGGTCGCCTCGTTAGCGGCCTTCA<br>CCACGCCCTCATCGTCCACTTTCGCGTCATTGTAACCTCCTGG<br>AATGGCGACCGTGGCGCTCCAAGCGACCACCAAGAAACCAA<br>AAAAGAGTAAAGCCTTCCACATGTTTGCGATATCGAGTCTCG<br>AAGGAGAAGCGGTTTGCCACGGCCTGCACGCACTTCTCCTTC<br>GAGACTCGA

Metalloproteinase inhibitors							
**Transcript ID**	**Uniprot ID reference protein**	**e-value**	**Identity (%)**	**PFAM domain**	**MW (Da)**	**Amino acid sequence**	**Nucleotide sequence**
PhdPInMtP01	P0DKM9	2,61E-08	46,667	PF07648	24885.86 **22826.31**	MRANVIIGILLFLSTVVDC**RRRRYKECSEERLKIREGAADIVLTGSVKRLYSGLADIYSGEVLVKRVVKGGHIKAGETLIVEGFGNKDICTSQPLAKDSKIFLLSQLQTGRFRLNSSMIQINSQNLDKITATVKGIPYRRRPPIADEPCEKKYCKFNGDCYEENRRALCRCPTSCKEHYNPICGSDGFTYNTECQMRVDSCRKKKRIYVRHEGLCSQSK***	TGCGTGAGGTCATTGATGCGATTATTCAAATTGTGCTACATCTTCCTACATTGGTCAAAGAAAATTAGTTAAAATAAACATAGAATATCGATCAATTCAAGACTGTGTATTTCACTTGGATTGTGAACAAAGACCTTCGTGACGCACGTATATTCTCTTTTTCTTACGACAAGAGTCTACTCTCATTTGACATTCCGTGTTATAAGTGAAACCATCGCTTCCACAAATGGGATTGTAATGTTCTTTGCAACTGGTTGGACATCGGCAGAGTGCTCGTCGATTTTCTTCATAACAGTCCCCATTGAACTTACAGTATTTCTTTTCACATGGTTCATCAGCTATTGGCGGTCTCCTTCTGTACGGTATTCCTTTCACAGTTGCAGTGATTTTATCCAAGTTCTGGGAATTGATTTGTATCATACTCGAATTTAATCGGAACCTGCCTGTTTGCAGTTGACTCAGAAGGAATATTTTGGAATCCTTCGCAAGAGGTTGGCTGGTACATATGTCCTTATTTCCAAATCCTTCTACTATAAGGGTCTCTCCAGCTTTAATATGGCCTCCTTTTACTACTCGTTTCACGAGGACTTCCCCGCTATATATATCAGCCAAGCCGCTGTACAGCCGTTTCACGCTACCTGTCAAAACTATATCTGCTGCACCTTCGCGGATTTTTAGCCTTTCTTCCGAACATTCTTTGTATCGTCGCCTTCGACAGTCAACCACGGTTGATAAAAACAACAGAATTCCTATAATCACATTTGCCCGCATAGCGCTTGAATGGAGAACCCGATCAGAAATTTCAGGCTCTCAAAACACTTGTTATTTTGAAGTAGGTCGTACGCGACGCG
FIBRINOGEN-LIKE PEPTIDES							
Techylectins							

**Transcript ID**	**Uniprot ID reference protein**	**e-value**	**Identity (%)**	**PFAM domain**	**MW (Da)**	**Amino acid sequence**	**Nucleotide sequence**

PhdFibTec01	D8VNS7	2,76E-43	40,183	PF01391<br>PF00147	65125.29<br>**62795.39**	MNLPVFVQALLLCV<br>LASLVSCN**RERRLAE**<br>**ILDGINRIEEHLKKE**<br>**ERLREITDIVRIDAT**<br>**VDKLNKKFDIVID**<br>**MLDNSPSLASPRSG**<br>**GQGALIPRDLSTNL**<br>**EVIDNRLSNLESTV**<br>**AKVEESTSKIDLYA**<br>**ARIEEIESRTTQMK**<br>**DDTNLKLRKMSNV**<br>**INALYEMNKDMKV**<br>**NMGGRSETPEQSA**<br>**GDSGSNYASTFLVE**<br>**NLEEMERKMKEQL**<br>**SVLATDLRSEITSLK**<br>**MVMSTLGSNCQSS**<br>**SESEDEEWTPRDNA**<br>**RSIPQSSRSIQKVEN**<br>**TLDNLVESLGRQAR**<br>**EIREEIHTGMKEIDT**<br>**KLNNLTLIAHTHQT**<br>**CDPLETAESIASRQS**<br>**VRAVLPIQSVGEKQ**<br>**SGRSETCTKSVQNV**<br>**PNPKSCSELRKGGA**<br>**TCDGIYVIFPKGVR**<br>**AVRVLCDMTTDGG**	GGGAGGGGGAACTTTTTCGGCTCTGGCGTTCATTAGCCGTTG<br>CAGTCCAGTCGAGACGCTGCCATTCACAATTAACGTCGCTGT<br>TTGCGTCATCTGTGTTGATTCACCACTTAAGGATAAGTCAAA<br>GAATAATACCACGTGGATCCCTGGTGACTGTCAACAAAGTA<br>ATCAAGCATATTTCCCAGAAACCGGGGGAACTTCGCTGTGTT<br>TTCAGTTCTTCCGATCCTTTCGACTGCGCTTCAAAATTGCAGT<br>CCTGCTCTCGTTCGACACATTGCTGATTCTCGCTCGCAGAACA<br>GACGGTCCCTACCGGTCCTTCTGGCGTGTGCATAAAGCTAAA<br>AGGAACACGGCGAAATAATCTTTTAATTGTATGAAGAAGTA<br>CGTCGTGCTAGCAACAGAATAGTGCTCATTCAAAAGTGTGTA<br>AATTGCTGAAATTTGAAAGAATACATCATGAATCTACCGGTG<br>TTTGTGCAAGCTCTTTTACTCTGTGTTCTCGCTTCTTTAGTATC<br>CTGCAATAGAGAGAGAAGATTAGCTGAAATATTAGATGGCA<br>TAAACAGAATTGAGGAACACTTGAAGAAAGAAGAAAGACTT<br>AGAGAGATTACTGATATAGTGCGGATTGATGCAACGGTGGA<br>TAAGCTGAATAAAAAGTTTGATATCGTGATTGACATGCTTGA<br>CAATTCTCCTTCCCTTGCAAGTCCTCGCAGCGGAGGGCAAGG<br>TGCTCTTATACCTCGAGACCTTTCCACGAACTTGGAAGTTATT<br>GATAACAGGCTAAGCAATTTGGAATCTACTGTAGCTAAAGTC<br>GAAGAAAGCACGTCTAAAATCGACTTATATGCAGCCAGAAT<br>TGAAGAAATAGAAAGCCGAACGACCCAAATGAAAGATGAT<br>ACGAACTTGAAATTGCGGAAGATGTCGAATGTTATAAACGCT<br>CTGTACGAGATGAACAAAGACATGAAAGTCAACATGGGAGG<br>GCGAAGCGAAACTCCGGAGCAAAGTGCTGGGGATTCCGGTT<br>CTAATTACGCATCTACATTTTTAGTGGAGAATTTAGAAGAAA<br>TGGAACGGAAAATGAAAGAACAACTTAGCGTTCTTGCGACT<br>GACCTCAGATCAGAAATAACGAGTCTGAAAATGGTTATGTC<br>AACGCTGGGCAGTAACTGCCAGTCATCTTCCGAATCGGAAG

						**GWTVLMRRGDFG<br>DKMTTFHKNWPSY<br>KNGFGDSEGEFWL<br>GNDVMHLMTTEEE<br>NMLRIHLSSFDND<br>DINLDYASFAVGNE<br>SSFYRLHIGHPTGA<br>AARTPAANSLRYH<br>NNHPFSTFDRKND<br>GFEQNCAATYKGG<br>WWFNGCYFGFLTG<br>EYFRATDKRETWQ<br>GIMWYDWKGNQS<br>LKGAEMMIRPKNF<br>TITV***	ACGAAGAATGGACGCCAAGAGACAACGCACGTTCTATTCCC<br>CAATCAAGCAGATCTATTCAGAAAGTAGAAAACACTTTGGA<br>CAACTTGGTGGAAAGTTTAGGGCGACAAGCTCGAGAAATTC<br>GAGAGGAAATTCACACTGGCATGAAAGAGATTGACACTAAG<br>TTAAACAATTTGACTCTGATTGCTCATACGCATCAGACTTGTG<br>ACCCTCTGGAAACTGCCGAATCGATCGCCAGCCGTCAGTCCG<br>TTCGAGCAGTATTGCCAATCCAATCTGTGGGTGAAAAACAGT<br>CCGGAAGAAGTGAAACTTGCACTAAAAGCGTTCAAAATGTG<br>CCTAATCCGAAATCTTGCTCAGAGCTGAGAAAAGGAGGAGC<br>TACTTGTGATGGTATTTACGTAATTTTTCCCAAAGGAGTAAGA<br>GCTGTTAGAGTACTTTGCGACATGACAACAGATGGTGGTGGA<br>TGGACAGTTCTCATGAGGAGAGGAGATTTTGGTGACAAAAT<br>GACGACCTTCCACAAAAACTGGCCCAGCTACAAAAATGGTT<br>TTGGTGACTCTGAAGGTGAATTCTGGCTGGGCAATGACGTCA<br>TGCACCTCATGACCACGGAAGAAGAAAACATGCTTCGAATC<br>CATTTATCTTCTTTCGACAATGATGACATAAACTTGGACTACG<br>CATCTTTCGCAGTTGGAAATGAGAGCTCGTTCTATCGCCTGC<br>ACATAGGTCATCCTACAGGAGCTGCAGCGAGAACTCCAGCA<br>GCTAACAGCTTGCGCTACCACAACAACCATCCTTTCAGCACG<br>TTCGACCGCAAAAATGACGGATTCGAGCAGAATTGTGCTGCT<br>ACCTACAAAGGAGGATGGTGGTTTAACGGCTGCTACTTTGGT<br>TTCCTGACGGGAGAATATTTCAGAGCAACCGACAAGAGAGA<br>AACGTGGCAAGGAATCATGTGGTATGACTGGAAAGGTAACC<br>AGTCTCTCAAAGGGGCTGAAATGATGATTAGGCCCAAGAAC<br>TTCACGATTACAGTCTAATTTATATTCATTTAAGTAGATTGCA<br>GGAGAAAGACATTATATGCTGTACCGAGACCATTATTGTACA<br>AGACCATTATACACTGTAGTGAATTCGAGCTGTGCCAAATAT<br>ACTTCATGCTGTGAAATGAATTTTAGAGCCTTTTGACATGTAT

							AGTTTTCTAATATATAGTGAGTTAACAGTATTTAATGGATGTTTTCATAGAAGATGTACTTTTTATTATTTTATTCTGAAATAAAATAAAGA

PhdFibTec02	D8VNT0	4,11E-42	44,785	PF01391<br>PF00147	20931,14	FDSGQEYFYKDWNA<br>YKHGFGDLMKDFW<br>LGNDNIFVLTNQRA<br>SVVKFTLADWEGNT<br>TYATYDEFWIDDEM<br>RNYMLHVSSYRGTA<br>GDDFSYHNEQAFST<br>NDRDNDKKSDHCA<br>VEYKGAWWYKSCY<br>HANLNGMYFKKAL<br>PSKKHGISWGGFRG<br>RSYPLKETVIKIRPQD<br>FQRKTNDITPH*	TTTTGACAGTGGACAAGAATATTTTTATAAAGATTGGAACGC<br>TTATAAACATGGTTTCGGAGATCTAATGAAGGATTTCTGGCT<br>AGGTAATGACAATATATTTGTTCTAACAAACCAAAGAGCGTC<br>AGTTGTGAAATTTACATTGGCTGACTGGGAAGGAAATACCAC<br>TTACGCTACCTACGACGAATTCTGGATAGACGACGAGATGCG<br>GAATTACATGTTGCATGTCTCCAGCTACAGAGGAACAGCAG<br>GTGACGATTTTTCTTATCATAATGAACAAGCGTTCAGTACTA<br>ACGATCGAGACAATGACAAGAAGAGTGACCATTGTGCTGTT<br>GAGTATAAGGGAGCTTGGTGGTATAAAAGCTGTTATCACGCA<br>AACCTTAACGGAATGTATTTTAAAAAAGCTCTTCCGTCTAAG<br>AAACATGGAATCTCATGGGGAGGTTTCAGAGGACGATCTTA<br>CCCTCTGAAAGAGACTGTCATAAAAATTCGGCCTCAAGATTT<br>TCAGAGGAAGACAAATGATATTACTCCACATTAAAATTGCTT<br>TTCACCCAGCTTGTTATAGATATTTGAATTTCAAGACAATTTA<br>TTTAAACAAGCATATGAATTAGTTCAAGAGAAGACCATCTAC<br>TTTTGCATAATATTTTCTATTAGAGTTCCATGCATGTTTCAAA<br>GTAATATCCATAGTTTTTTACTCTGGATTTTCTCTCGTTATTTTT<br>CAAGAATGAAAAATACATATACGTTATAATTATATCAAACA<br>ATATATATACTATATTTTATATTTCTGTATACACATACAAA

PhdFibTec03	D8VNT0	8,88E-43	47,239	PF01391<br><br>PF00147	20620,71	FDSGQEYFYKDWNAYKHGFGDLIKDFWLGNDKLFALTNQRASVVKFSLVDWEGNTTYATYDEFWIDDELRNYTLHVSDYRGTAGDSFSSHNERAFSTKDRDNDKSSTHCAVKYKGAWWYDACYYSNLNAVYFKDVVSTPRGGIIWRSFGGSAYTLKETVIKIRPQDFQKKPNDVTPH*	TTTTGACAGTGGACAAGAATATTTTTATAAAGATTGGAACGCTTATAAACATGGTTTCGGAGATCTAATAAAGGATTTCTGGCTAGGTAATGACAAGTTATTTGCTCTAACAAACCAAAGGGCATCCGTTGTGAAATTTTCATTGGTTGATTGGGAAGGAAATACGACTTACGCTACATATGACGAATTCTGGATAGACGACGAACTGCGGAATTACACATTGCATGTGTCCGATTACAGAGGAACAGCTGGTGACAGTTTTTCAAGTCATAATGAACGAGCGTTCAGCACTAAGGATCGAGACAACGACAAGAGCAGTACACATTGTGCTGTTAAATATAAGGGAGCTTGGTGGTATGACGCATGTTATTACTCAAACCTTAACGCTGTGTATTTTAAAGATGTTGTTTCGACTCCTCGAGGTGGAATCATATGGCGAAGTTTTGGAGGATCAGCATACACGCTGAAAGAGACTGTGATAAAAATTCGTCCTCAAGATTTTCAGAAGAAGCCAAATGATGTTACTCCACATTAAAATTGCTTTCAACCCAGTATATTATGGATATATGCATTTCAAGAACAATTTATTCAAACAAGTATATGAATTAGGTCAAGAGATAAGCATATACTTTTGAACAATATTTTGCATTACCGTTCCAGTGGAGTTCTATGGATGTTTCAATGGAAACTCCATAGATATTTTCACTGGATTTTCTCATGATATTTTTCATTCTTGTAAAATACATGTAAGGTATAGTCATATAACTATGAATAAAATATACTATATGTTAAAAAA

PhdFibTec04	D8VNT0	3,62E-39	45,732	PF01391<br>PF00147	20840,85	FDSGQEYFYKDWNAYKHGFGDLMKDFWLGNDNIFVLTNQRASVVKFTLADWEGNTTYATYDEFWIDDEMRNYTLYVSGYRGTAGDDLSYHNEEAFSTNDRDNDKSIRHCAAEYKAAWWYHNCYSSNLNGQYFKNVLPTKKEGIAWGGFRGGSAYSLKETVIKIRPQDFQRKTNDITPH*	TTTTGACAGTGGACAAGAATATTTTTATAAAGATTGGAACGCTTATAAACATGGTTTCGGAGATCTAATGAAGGATTTCTGGCTAGGTAATGACAATATATTTGTTCTAACAAACCAAAGAGCGTCAGTTGTGAAATTTACATTGGCTGACTGGGAAGGAAATACCACTTACGCTACCTACGACGAATTCTGGATAGACGACGAGATGCGGAATTACACGTTGTATGTGTCCGGCTACAGAGGAACAGCTGGTGACGATTTATCTTATCATAATGAAGAAGCGTTCAGTACTAACGATCGAGACAATGACAAGTCCATTAGGCATTGTGCTGCTGAGTATAAAGCAGCTTGGTGGTATCACAACTGTTATTCCTCAAACCTTAACGGGCAATATTTTAAAAACGTTCTTCCGACTAAGAAAGAAGGAATCGCATGGGGAGGTTTCAGAGGAGGATCAGCATACTCGCTGAAAGAGACTGTCATAAAAATTCGGCCTCAAGATTTTCAGAGGAAGACAAATGATATTACTCCACATTAAAATTGCTTTTCACCAACTTTGTTATAGATATATTCATTTCAGGAACAATTTATTTAAACAAGCATATGAATTAGTTCAAGAGATAACCCACTTTTTTGCATTATATTTTCTATTAGAGTTCTAGTTGAGTTCTACGCATGTTTCAAAGGAATATCCATAGTTTTTTACACTGGATTTTCCATGGATATGTTTCAAGAATGAAAAATACATATACGTTATAATTATATCAAACAATATATATACTATATTTTATATTTCTGTATACACATACAAAT

PhdFibTec0<br>5	D8VNT0	1,09E-44	47,170	PF01391<br><br>PF00147	20267,4	YFYKDWKSYKHGFG<br>DLMKDFWLGNDKL<br>FTLTNQRASVVKFSL<br>VDWEGNTTYATYDE<br>FWIDDELRNYTLHV<br>SGYRGTAGDDFSYH<br>NEQAFSTKDRDNDK<br>STVHCAVKYNGAW<br>WYDKCHRTNLNSLY<br>FQNVLSTKEDGISWR<br>GFRGTTYSLKETVIKI<br>RPQDFQKKPNDVTP<br>H*	TATTTTTATAAAGATTGGAAATCTTATAAACATGGTTTCGGAG<br>ATCTAATGAAGGATTTCTGGCTAGGTAATGACAAGTTATTTA<br>CTCTAACAAACCAAAGGGCGTCCGTTGTGAAATTTTCATTGG<br>TTGATTGGGAAGGAAATACGACTTACGCTACATATGACGAAT<br>TCTGGATAGACGACGAACTGCGGAATTACACATTGCATGTGT<br>CCGGCTACAGAGGAACAGCTGGTGACGATTTTTCTTATCATA<br>ACGAACAAGCGTTCAGCACTAAGGATCGAGACAACGACAA<br>GAGCACTGTACATTGTGCTGTTAAATACAATGGAGCTTGGTG<br>GTATGACAAATGTCATCGCACAAACCTTAACAGTCTGTACTT<br>TCAAAATGTTCTTTCGACTAAGGAAGATGGAATCTCATGGCG<br>AGGTTTCAGAGGAACAACATACTCACTGAAAGAGACTGTGA<br>TAAAAATTCGTCCTCAAGATTTTCAGAAGAAGCCAAATGATG<br>TTACTCCACATTAAAATTGCTTTCAACCCAGTATATTATGGAT<br>ATATGCATTTCAAGAACAATTTATTCAAACAAGTATATGAAT<br>TAGGTCAAGAGATAAGCATATACTTTTGAACAATATTTTGCA<br>TTACCGTTCCAGTGGAGTTCTATGGATGTTTCAATGGAAACTC<br>CATAGATATTTTCACTGGATTTTCTCATGATATTTTTCATTCTT<br>GTAAAATACATGTAAGGTATAGTCATATAACTATGAATAAA<br>ATATACTATATGTTAAAAAAA
PhdFibTec0<br>6	D8VNT0	3,58E-25	53,659	PF01391<br><br>PF00147	11005,03	TAGDSFSDTNGFPFT<br>TKDQDNDKHDRNC<br>AVIYKGAWWFYNC<br>HTSNLNGMYLKGA<br>HGSDADGVDWLAF<br>RGYYYSLKDTVIKIRP<br>RDFQKNSDDITPH*	GAACAGCTGGTGATTCCTTTTCGGACACTAATGGATTTCCATT<br>TACCACCAAGGATCAAGACAATGACAAGCATGATAGGAACT<br>GTGCTGTTATTTATAAAGGGGCTTGGTGGTTTTATAATTGCCA<br>TACTTCGAATCTTAATGGAATGTACTTGAAGGGCGCTCATGG<br>AAGTGACGCTGATGGAGTTGACTGGCTAGCTTTTAGAGGTTA<br>CTACTATTCTCTGAAAGATACTGTGATAAAAATTCGCCCTAG<br>AGATTTCCAGAAGAATTCAGATGATATTACACCACATTAACC<br>ACTTTCCATTCGTTGTATCTTGATTGATAACCGACATTTATTTC<br>CAGAGATTGATCAGAACAATGGTTTCAGTCATTTTAAGAAAT

							TAACAATGATTTCCAAGTTTACACATGGATTCCTAACTGCAA<br>TTAGCAATTTCCATCAAACTAAATAATTATAGTATTGTAATA<br>ATTATAAGAGTTGAATAATGAATTCTATATATATAATGTTTGT<br>GTTTTTATATGTATATATTTTATGTGTG
PhdFibTec07	D8VNT0	3,14E-64	44,978	PF01391<br><br>PF00147	35169.15<br><br>**32732.30**	MYSNTLIFLTLWSFVI<br>YSANA**DETECTSPL**<br>**LKNIIDQTQSSVVL**<br>**TLLNSAENLLEDAK**<br>**RYVAIKTRCSVTES**<br>**TCEKPMDCAEIMEK**<br>**NPFANSGVYKIWPR**<br>**SRVINNPIDVYCDM**<br>**EDDGGAWTVLQRR**<br>**GEFDSGSDYFYKD**<br>**WKSYKEGFGDKKK**<br>**DFWLGNDNIFALT**<br>**NQRPNVVKFSLSD**<br>**WEKNETYATYDEF**<br>**WIEDESRKYTMHVS**<br>**GYRGTAGDSFSVT**<br>**NGLPFTTKDQDND**<br>**KHHKNCAVQFKG**<br>**AWWYAGCHSSNL**<br>**NGMYLKGAHGSD**<br>**ADGVDWLAFRGYY**	TCTCATGCAATGTATTCGAATACTTTAATTTTCTTAACTCTCTG<br>GAGTTTCGTGATTTACAGTGCTAACGCAGATGAAACAGAATG<br>CACTTCGCCGCTGTTAAAGAACATAATCGACCAAACGCAAT<br>CATCTGTTGTATTGACTCTCTTAAATAGTGCTGAAAACCTTCT<br>GGAGGATGCAAAGAGATATGTAGCAATAAAAACCAGATGCT<br>CTGTTACTGAATCAACGTGTGAAAAACCAATGGACTGCGCTG<br>AAATAATGGAGAAAAATCCCTTTGCAAACAGTGGTGTATAC<br>AAAATCTGGCCTCGCAGTCGGGTGATTAACAATCCGATCGAT<br>GTCTATTGTGACATGGAGGATGACGGAGGAGCTTGGACGGT<br>ACTGCAGAGACGAGGAGAATTCGATAGTGGGAGCGATTATT<br>TTTATAAAGACTGGAAATCTTATAAAGAAGGATTCGGCGATA<br>AAAAGAAAGATTTTTGGCTGGGTAATGACAACATCTTTGCTC<br>TCACGAACCAACGGCCGAATGTCGTAAAATTTTCTTTGAGTG<br>ATTGGGAAAAAAATGAAACGTATGCAACATATGATGAATTC<br>TGGATTGAAGACGAATCTCGTAAATACACCATGCATGTGAGT<br>GGATACAGAGGAACAGCTGGTGATTCTTTTTCTGTTACTAAT<br>GGATTACCATTTACCACCAAAGATCAAGACAATGACAAGCA<br>TCATAAGAACTGTGCAGTTCAGTTTAAAGGGGCTTGGTGGTA<br>TGCAGGTTGCCATTCTTCGAATCTTAATGGAATGTACTTGAA<br>GGGCGCTCATGGAAGTGACGCTGATGGAGTTGACTGGCTAG<br>CTTTTAGAGGTTACTACTATTCTCTGAAAGATACTGTGATAAA

						**YSLKDTVIKIRPRDF QKNSDDITPH***	AATTCGCCCTAGAGATTTCCAGAAGAATTCAGATGATATTAC ACCACATTAACCACTTTCCATTCGTTGTATCTTGATTGATAAC CGACATTTATTTCCAGAGATTGATCAGAACAATGGTTTCAGT CATTTTAAGAAATTAACAATGATTTCCAAGTTTACACATGGA TTCCTAACTGCAATTAGCAATTTCCATCAAACTAAATAATTAT AGTATTGTAATAATTATAAGAGTTGAATAATGAATTCTATAT ATATAATGTTTGTGTTTTTATATGTATATATTTTATGTGTG

PhdFibTec08	D8VNT0	4,67E-62	43,668	PF01391<br><br>PF00147	35243.35<br><br>**32806.50**	MYSNTLIFLTLWSFVIYSANA**GETECTSLLLKNMTDKSQSSVVLNLLNNARNLIEDAKEYVARKTRCSVNKSTCEKPMDCAEIMEKNPFSNSGVYKIWPRSRIITNPIDVYCDMEDDGGAWTALQRRGNFDSGKEYFYKDWKSYKEGFGDKKKDFWLGNDNIFALTNQRPNVVKFSLSDWEKNETYATYDEFWIEDESRKYTMHVSGYRGTAGDSFSVTNGLPFTTKDQDNDKHHKNCAVQFKGAWWYAGCHSSNLNGMYLKGAHGSDADGVDWLAFRGYYYSLKDTVIKIRPRDFQKNSDDITPH***	CAAACACTTGCACTTCTTGGAATATTTCAATACTAACGTCATATTACTACGATACGTTTCGATTTATTTTATTCTCATATTTTGGTATTTAAAGAGTGAAAGTTCCTTGAAGTTTAGTGTCTCACAGCGATCGTGACGGACGTTTTATCTCTCTCTCATGCAATGTATTCGAATACTTTAATTTTCTTAACTCTCTGGAGTTTCGTGATTTACAGTGCCAACGCAGGTGAAACAGAATGCACTTCGCTGCTGTTAAAGAACATGACCGACAAATCACAATCTTCTGTTGTATTGAATCTGTTAAATAATGCTAGAAATCTTATCGAAGATGCAAAGGAATATGTAGCCAGAAAAACCAGATGCTCTGTTAATAAATCAACGTGTGAAAAACCAATGGACTGCGCTGAAATAATGGAGAAAAATCCCTTTTCAAATAGTGGTGTTTACAAAATCTGGCCGCGCAGTCGGATAATTACTAATCCGATCGATGTCTATTGTGATATGGAGGATGACGGAGGTGCTTGGACGGCACTGCAGAGACGAGGAAATTTCGATAGTGGGAAAGAATATTTTTATAAAGACTGGAAATCTTATAAAGAAGGATTCGGCGATAAAAAGAAAGATTTTTGGCTGGGTAATGACAACATCTTTGCTCTCACGAACCAACGGCCGAATGTCGTAAAATTTTCTTTGAGTGATTGGGAAAAAAATGAAACGTATGCAACATATGATGAATTCTGGATTGAAGACGAATCTCGTAAATACACCATGCATGTGAGTGGATACAGAGGAACAGCTGGTGATTCTTTTTCTGTTACTAATGGATTACCATTTACCACCAAAGATCAAGACAATGACAAGCATCATAAGAACTGTGCAGTTCAGTTTAAAGGGGCTTGGTGGTATGCAGGTTGCCATTCTTCGAATCTTAATGGAATGTACTTGAAGGGCGCTCATGGAAGTGACGCTGATGGAGTTGACTGGCTAGCTTTTAGAGGTTACTACTATTCTCTGAAAGATACTGTGATAAAAATTCGCCCTAGAGATTTCCAGAAGAATTCAGATGATATTACACCACATTAACCACTTTCCATTCGTTGTATCTTGATTGATAACCGACATTTATTTCCAGAGATTGATCAGAACAATGGTTTCAGTCATTTTAAGAAATTAACAAT

							GATTTCCAAGTTTACACATGGATTCCTAACTGCAATTAGCAATTTCCATCAAACTAAATAATTATAGTATTGTAATAATTATAAGAGTTGAATAATGAATTCTATATATATAATGTTTGTGTTTTTATATGTATATATTTTATGTGTG
PhdFibTec09	D8VNT0	5,29E-15	52,542	PF01391<br>PF00147	8490,53	KDVNCAEHHKGGWWYGACQFSNLNGLYHTGKHESLDGINWYTWKGHNESMESTEIKIRPKTFRLPNLTMNTPL*	ATAAGGATGTAAATTGTGCCGAACACCATAAAGGAGGATGGTGGTATGGTGCATGTCAATTTTCCAATCTGAACGGGCTCTACCATACGGGAAAACATGAAAGTCTTGATGGAATAAACTGGTATACCTGGAAAGGCCATAACGAGTCCATGGAATCCACTGAAATAAAGATTCGACCAAAAACGTTCAGGCTGCCAAATCTGACCATGAATACTCCTTTGTGAAATTTTACAAATGCGTTCCTTAAGAAAGCTATAATACTGATATATG

PhdFibTec10	D8VNT0	4,37E-16	54,545	PF01391<br>PF00147	12032,13	SLNRNTFSFFSGDSFS<br>STNERQFTTKDKDN<br>DSHDNGNCAVQYK<br>GAWWYTNCHNSNL<br>NGLYLKGAHENKD<br>GVIWGTFRGHRISLK<br>DTTIKIRPRDFKKNS<br>DATTPH*	TTTATGTTTTATAATTAATAAAGACATAGCATATGTTTATTCA<br>TTTTTATGACAATGATGAACTGAAGTGATTTGAAGCTTAAAC<br>CGAAATACGTTTTCCTTTTTTTCAGGCGATTCATTTTCGAGTA<br>CTAATGAAAGACAGTTTACTACTAAAGATAAAGACAATGAT<br>AGTCATGATAATGGGAATTGTGCTGTCCAGTATAAAGGTGCT<br>TGGTGGTATACCAACTGCCATAATTCAAACCTTAACGGACTG<br>TACTTGAAAGGAGCTCATGAAAATAAGGATGGAGTAATATG<br>GGGCACCTTTAGAGGACACCGCATATCTCTGAAGGATACTAC<br>CATAAAGATTCGCCCTAGAGATTTCAAGAAGAATTCAGATG<br>CTACTACACCGCATTAACTGCTTTCCATCCCTAGTAGCTCGAC<br>TGAAAACAACATTTTCTTTCAAAACTCGATTGCAACAATGAT<br>TTCCATTAGTGAAAAATCAACAACTACCGTTAAATTTCAGCA<br>TTCAGCAAATTCTATCAAATGGAACAGAATGGTATCATCATA<br>AAAATTCTTATTTTGCTCTGATATATATATATATATATGACAC<br>ATTTATATATATGTATGTGTGTGTGTGTGTGTGAAAGAGAG<br>AGAGAGAGAGAGAGAGTAATAAAATATTAAAAAGACTTAG<br>ACTTTAGTTTAAATTAATATTCTTATTTCATATCTGGTTTACAC<br>ATCACAATTACAGACTATGAACAAAATTTTCTAGACTTACAT<br>CCATACCTTAATCGCTTAATCGATATCGAGCAACTGACTGAT<br>TTTACAGCAGTTACAATCCTTTCTGAAAGACTTTAAGACACC<br>TTATCGGATAAAGACATCTTAAACACCACGCCGGAGAAGAT<br>GCATGAGAATGGACATTATTGGACTTTTGAAACGGAGGTTTT<br>AAGTGAAACTTACTTTTTTGGGAAGCAACAGATACATCATCC<br>ATAACAGCAACTGTTACTTTCATCAATTTTTGGAACTAGTGG<br>GTGATTTCACGCATTCCATTTTAGTTGTTCAGCTTTTGACCGT<br>AAAATAAATCTGTGGTGTTGTTGCTATCAGTGACTTTGCTAGC<br>ATATAAATTTTTGGATGAATAATTATATATGAAAAATTTTTTC<br>TGACCACTCTTAGGTTAATTTGAAAGCAAAAGCATGGGACAC

							ATACACATACTACAAGGTACTGATAAAATGCCGTATCAATTG<br>GATGAACAAGGTATTGTCTTAGTATATTAGAAAGAATTCATA<br>CTGTTAAGAACAAAACTAATATTAATATCCAAACTACAGGTA<br>GAGCTTATCGGTAAATTTAAAAGTAAAAGCACGTTTACTTTT<br>ACCCTGTCAACAGGAATTTTAGCCTGTAAAACTAGACAAATG<br>CTCTCTGATTATGAATTGATATGTTCCACTATACTTTGAGAAA<br>AGCATTTATTCATAGTTTACAATTGTAATGCATATATGAATTA<br>ATAAATGAAATTTTCATTTAAAGTAATTCTGTTGCACTTTATA<br>CATCACATATACGTTGTAAAAATTTAGAAAGTACTTCACTAA<br>TTCTCCTTAATGTGCATTATACTTTTTAATTTGAACTCATTCCC<br>TTTTTGACATAACAATGATCTGTAGGTTTTCACTCAGAGATAA<br>ACTGGTGGGTGCTTGCATGCTTTCAGTGCTCATCTGGTACTAA<br>GGGGTGTGGAATCTATAAATGCTACACAAAACAATGGAGCC<br>TTCATTTCTGTGAATAGGGTCTGAACTCGCCATCTATACACTT<br>TACTCTTTACAGCAAAAGATTCCAGTTGTGTTTTATTTGAGGA<br>TGATACACGCCTTTTAGTTATCCTGTACATTATGCTATCTATCT<br>CCAATAAATCATGTTAGGTTCATGTAACTGCAAAGTTTCTTTG<br>CTTAAAATACTTTTATATTTCCTGAGGACGTGATACTGTTTAG<br>ACTCTTAATGGACTATATTGCCTTGCTGTTCATCTTTACAAAT<br>CTAAAATTTTCCCAAGCCCTTTTTGTAGCCTCCACAGACTTTC<br>CAAATTGATGGACAGATGCCACCTTCTAATTTGCTCTGCCCA<br>GAATGGGACAACTATATCAGTTTGTTACTGGGAAGCTCAACA<br>TCTGCTTATGGGTATATCTAATGGCTAATGCAAATTTCTTTCT<br>GCTCGCATATACCCTTTTGAGCCATATTGGAAATATATACAC<br>ACACATACATAAAATCCTCTATATTTTTACTCAGAAATATAT<br>ATATTCCTGCATAAAAATAAAATTATTCAATTTAAAAAAATT<br>AAGTCACTTACTATCCGAAACAATTTATGAATACATTCATAT<br>ACATACATGCCTGTGCACAATGTACAATCAGAAATTAGAACT

							ACATAAGCATATTCCAAGCATATCATACCATATATTTTCATA ACTGCAACCTAAGACAATATAAATAAAAAAAAAAATTCACA TTTTTATACAAACTTCCACAAATTCTTTTTAAAAATGTATTGA TAAAATCTATATTGAA

PhdFibTec1<br>1	D8VNT0	1,47E-16	54,545	PF01391<br><br>PF00147	12032,13	SLNRNTFSFFSGDSFS<br>STNERQFTTKDKDN<br>DSHDNGNCAVQYK<br>GAWWYTNCHNSNL<br>NGLYLKGAHENKD<br>GVIWGTFRGHRISLK<br>DTTIKIRPRDFKKNS<br>DATTPH*	TTTATGTTTTATAATTAATAAAGACATAGCATATGTTTATTCA<br>TTTTTATGACAATGATGAACTGAAGTGATTTGAAGCTTAAAC<br>CGAAATACGTTTTCCTTTTTTTCAGGCGATTCATTTTCGAGTA<br>CTAATGAAAGACAGTTTACTACTAAAGATAAAGACAATGAT<br>AGTCATGATAATGGGAATTGTGCTGTCCAGTATAAAGGTGCT<br>TGGTGGTATACCAACTGCCATAATTCAAACCTTAACGGACTG<br>TACTTGAAAGGAGCTCATGAAAATAAGGATGGAGTAATATG<br>GGGCACCTTTAGAGGACACCGCATATCTCTGAAGGATACTAC<br>CATAAAGATTCGCCCTAGAGATTTCAAGAAGAATTCAGATG<br>CTACTACACCGCATTAACTGCTTTCCATCCCTAGTAGCTCGAC<br>TGAAAACAACATTTTCTTTCAAAACTCGATTGCAACAATGAT<br>TTCCATTAGTGAAAAATCAACAACTACCGTTAAATTTCAGCA<br>TTCAGCAAATTCTATCAAATGGAACAGAATGGTATCATCATA<br>AAAATTCTTATTTTGCTCTGATATATATATATATATATGACAC<br>ATTTATATATATGTATGTGTGTGTGTGTGTGTGAAAGAGAG<br>AGAGAGAGAGAGAGAGTAATAAAATATTAAAAAGACTTAG<br>ACTTTAGTTTAAATTAATATTCTTATTTCATATCTGGTTTACAC<br>ATCACAATTACAGACTATGAACAAAATTTTCTAGACTTACAT<br>CCATACCTTAATCGCTTAATCGATATCGAGCAACTGACTGAT<br>TTTACAGCAGTTACAATCCTTTCTGAAAGACTTTAAGACACC<br>TTATCGGATAAAGACATCTTAAACACCACGCCGGAGAAGAT<br>GCATGAGAATGGACATTATTGGACTTTTGAAACGGAGGTTTT<br>AAGTGAAACTTACTTTTTTGGGAAGCAACAGATACATCATCC<br>ATAACAGCAACTGTTACTTTCATCAATTTTTGGAACTAGTGG<br>GTGATTTCACGCATTCCATTTTAGTTGTTCAGCTTTTGACCGT<br>AAAATAAATCTGTGGTGTTGTTGCTATCAGTGACTTTGCTAGC<br>ATATAAATTTTTGGATGAATAATTATATATGAAAATTTATTTC<br>TCTCCCTGACCACTCTGAGGTAAATTTAAAAGTAAGAGCATT

							GAAGCGCGTATAAATACTACGTGGTATTGATAAAATGACAT GTCAATTTTGTGAGCAAGTTATCTTAGTATATTAGAAAGAATT CACGCTATTAAGATCGAAACGAAAAAAAACTAATGGCATTT AGCTTGCGCCCCATGCT

PhdFibTec12	D8VNS7	3,12E-54	45,815	PF01391<br><br>PF00147	74181.52<br><br>**71893.67**	MVTSLTSIACVLLLG<br>FIPSIRA**RHHDLRRE**<br>**LASVKDMVAGIER**<br>**KLDSLSTTDTGKRI**<br>**RSIETTLTSLLTKVA**<br>**DINRKSQLLSEIHQ**<br>**DNDARKDILQRISR**<br>**SLEEMGRKQSVTSG**<br>**KKSDVEIERLDKLS**<br>**ENVQALFQIFGGM**<br>**NRKIDDLQTRVER**<br>**MPNVCPTSDLKFFI**<br>**TKKIDSLTASTALLS**<br>**ECLECDRESLKDVL**<br>**HSFLRHPLDNIAKK**<br>**CNSGDTERSVGRIL**<br>**ESKWKDMIDSVQS**<br>**LIHIKLEGFNAKLDE**<br>**AIQECNCLNDKNA**<br>**KFTAHDIAYSHDTP**<br>**QELRAAAKEDANT**<br>**PRETPGRIFRKLWR**<br>**KMTEPINKVGEKIE**<br>**SLAQTIEISNQRHH**<br>**NETLSKLREWTGKS**<br>**KGGDSCTKQINAIF**<br>**NTTKDTAQRIGYIE**<br>**SRQLNMQNSVSKIL**	GTACAAGTTGTCTCCTCTTCTAAACCAAGAGCTCGCAAATGA<br>GGTGAAAATGCAAATGTCAACATGATGAATAGTCGAATGAA<br>GACCAGTAGCCAAAATGAAAAGTATCAATAAGCTGATTGTTT<br>GACCGATAAATTCAAGTATCAAAATGGTGACTTCTCTTACTT<br>CTATAGCTTGTGTTCTCTTGCTGGGCTTTATTCCGAGCATCAG<br>AGCAAGACATCATGATTTACGTAGGGAATTGGCATCAGTGA<br>AAGATATGGTTGCAGGCATAGAAAGAAAACTTGACAGTTTA<br>TCTACTACTGATACAGGGAAAAGAATTCGAAGTATAGAAAC<br>AACTCTCACTTCTTTGTTAACAAAGGTAGCTGATATTAATAG<br>GAAAAGTCAACTACTGAGTGAAATACATCAAGACAATGATG<br>CTCGAAAAGATATCCTACAAAGGATATCACGGAGTTTAGAA<br>GAAATGGGACGTAAGCAATCCGTAACAAGTGGAAAGAAAA<br>GTGATGTAGAAATTGAACGCTTGGATAAACTTTCGGAAAATG<br>TTCAAGCCCTTTTTCAGATATTTGGAGGAATGAATAGAAAAA<br>TTGATGATTTACAGACGCGAGTAGAGAGAATGCCCAATGTTT<br>GTCCAACTAGTGACTTGAAATTTTTCATTACAAAAAAGATTG<br>ACTCTCTAACAGCTTCAACAGCATTACTCTCTGAATGTCTTGA<br>ATGCGACCGAGAATCTTTGAAAGATGTCCTTCACTCTTTCTTA<br>CGGCATCCATTAGATAATATTGCTAAGAAATGTAACTCAGGG<br>GATACTGAAAGAAGTGTCGGGAGGATATTAGAATCGAAATG<br>GAAAGATATGATTGATTCTGTACAGTCTCTTATACATATCAA<br>ACTGGAAGGTTTCAATGCAAAATTAGACGAAGCAATTCAAG<br>AGTGCAATTGCTTGAATGACAAAAATGCTAAATTTACAGCAC<br>ACGATATAGCATATTCTCACGATACCCCTCAAGAACTTAGAG<br>CTGCTGCTAAGGAAGATGCAAATACTCCCCGGGAAACTCCT<br>GGAAGAATATTTAGAAAATTGTGGCGAAAAATGACTGAACC<br>TATAAACAAAGTTGGTGAAAAGATTGAATCACTAGCGCAGA<br>CTATTGAAATATCAAATCAAAGGCATCATAATGAAACTTTAT

						**SEVERVKSTIQVGN<br>SAFIPGVTDAYIEGP<br>VEGYLATSCADLQT<br>QGTVRNGIYTIKPK<br>DAPEPFLAYCDLETE<br>GGGWTVIQRRGNF<br>GEEFRQNFTQNWD<br>MYKRGFGDPQREF<br>WIGNEKMHLLSSQ<br>EDVKLRVELEDFEG<br>NSSFAEYSRFRVEDE<br>TKQYRLSVGEYRG<br>NATDSLSLHDGKM<br>FSTHDKDNDEVAS<br>CCNCADTFKGGW<br>WYYRCFEANLNGP<br>YHTDPTENGYFLGII<br>WERWRGDYSLKSS<br>EMKIRPLSFEDLQD<br>P***	CAAAACTCAGGGAATGGACAGGAAAATCAAAAGGCGGCGA<br>TTCGTGTACAAAGCAAATTAATGCCATTTTTAATACAACAAA<br>GGATACAGCTCAAAGAATAGGGTATATCGAATCGCGCCAGT<br>TGAACATGCAGAATTCAGTGTCAAAGATTTTGTCGGAAGTCG<br>AAAGGGTTAAAAGCACAATTCAGGTTGGAAATTCTGCCTTTA<br>TCCCTGGAGTAACGGATGCGTACATCGAAGGACCAGTTGAA<br>GGATATCTAGCTACGAGTTGTGCAGATCTTCAAACTCAAGGA<br>ACTGTTAGAAATGGAATTTATACCATAAAACCGAAAGATGC<br>ACCCGAACCTTTCTTGGCGTACTGCGATCTCGAAACCGAGGG<br>AGGAGGCTGGACGGTGATTCAGAGAAGAGGCAATTTCGGTG<br>AAGAATTCCGCCAAAATTTCACTCAGAACTGGGACATGTAC<br>AAACGAGGTTTTGGCGACCCCCAGCGGGAATTTTGGATTGGA<br>AACGAGAAGATGCACCTGCTGTCTTCACAAGAAGACGTCAA<br>ACTGCGAGTGGAACTCGAAGATTTTGAAGGAAATTCCTCATT<br>CGCCGAGTATTCAAGATTTAGAGTTGAAGATGAGACTAAGC<br>AATACAGATTGTCAGTAGGTGAATATCGTGGGAACGCTACC<br>GATTCGCTCAGCCTTCATGATGGAAAAATGTTCTCCACCCAT<br>GATAAAGACAATGATGAAGTTGCATCATGTTGTAACTGTGCG<br>GATACATTTAAAGGTGGATGGTGGTATTACAGATGTTTCGAA<br>GCAAATCTAAATGGTCCATACCACACTGACCCTACTGAGAAT<br>GGTTATTTTCTAGGAATAATTTGGGAAAGATGGCGGGGTGAT<br>TATTCACTTAAATCTTCGGAAATGAAAATACGACCCCTATCT<br>TTTGAGGATTTACAAGATCCATGAATGGAATACATATGCATT<br>TCTAATTTTCTAGTCTTCCATGGTACTGAAGTCATTATTTATTG<br>GAGACACTTTCCATCTTCGCATTTATATCTAAAATCATAAGTT<br>AAAGAAGTTTTAGACATCATATAAAATAAAGAATGAAATCG<br>AAACGACAGTTAAGGAGACGAAACGATATGAGAGGAAATA<br>CGAGAAATTTACAGAATTAAACTGGTATATTTGTATGTTGAT

							CGTAAATGTGTGTGGTTGGATTATGAAGAATGATGACTGCGT TTAACGGACTCCGACATTGGTTTTATCGCTCCAATCTTCAGCC ATGGCCCATCTTTCGTATATATATATTTATATATATATGTATA TAAATCTACACACACACACACACACATATATAT

PhdFibTec1<br>3	D8VNT0	1,73E-29	53,000	PF01391<br><br>PF00147	13424,69	RNVLEGRDESRKYT<br>MHVSGYKGTAGDSF<br>SGTNGLPFSTKDQD<br>NDTYDKHCAVEFKG<br>AWWYSNCHSSNLN<br>GMYLKGAHASYAD<br>GVEWHGFRGHYYSL<br>KDTVIKIRPKDFHKN<br>SDDITPH*	TTTTTTTTAATGGTTAATATGTGTTTTTATTAAAACCTTAAGGA<br>CAGACCCTACATATATATATTTCATAATGTTAAAATAAGAAG<br>TATTACAGTGCTATAAATGTTCCATTTGTCAGAAATTGATGA<br>ATGCTGAAGTTTGGAACTCGTTGCTGATTTGAAATCATTGTCC<br>AATCAGTTTCTAAAAGTAAGCCAAGGTACTGGAGATGAAAA<br>GCAATTAATGCGGTGTAATGTCATCTGAATTCTTATGGAAAT<br>CCTTAGGACGAATCTTGATCACGGTATCTTTCAGTGAATAAT<br>AGTGACCTCTGAAGCCGTGCCATTCAACACCGTCAGCGTAAC<br>TTGCATGAGCTCCCTTCAAGTACATTCCGTTAAGATTTGAGG<br>AATGGCAATTTGAATACCACCAAGCCCCTTTAAACTCCACAG<br>CACAGTGCTTATCATACGTGTCATTGTCTTGATCTTTAGTGGA<br>AAACGGTAATCCATTAGTACCAGAAAAAGAATCACCAGCTG<br>TTCCTTTGTACCCGCTCACATGCATGGTGTATTTACGAGATTC<br>GTCTCTCCCTTCAAGTACATTCCGTTA
PhdFibTec1<br>4	D8VNS7	4,19E-06	47,619	PF01391<br><br>PF00147	11021.7<br><br>**9073.26**	MYLELLLAFVFCCA<br>GAMA**TSSCEQKITV**<br>**FHLDTAVDMISKA**<br>**KFNIPGCNKSGNN**<br>**VTRGALDCSDILLM**<br>**GYNKSGVYTIHPVS**<br>**RVTDDKPLEVYCD**<br>**METNGGG**	ATCCTCCTCCGTTCGTTTCCATATCGCAATATACCTCGAGTGG<br>CTTATCATCCGTCACTCTACTCACAGGATGTATTGTGTACACT<br>CCACTTTTATTGTAGCCCATGAGGAGAATGTCACTGCAATCG<br>AGAGCTCCTCTTGTCACATTGTTTCCACTCTTATTACATCCTG<br>GAATATTAAATTTCGCTTTCGAAATCATGTCAACTGCTGTATC<br>CAAATGAAACACGGTAATTTTCTGCTCACAACTTGAAGTCGC<br>CATGGCACCTGCGCAGCAAAATACGAAGGCCAGAAGTAGTT<br>CCAAATACATGGTCTTTTTCCACTCACAAAATTCGCAGATTAT<br>ACGCAAGAATGGAACAGTTGACGGATAATTTAACTTCTGAC<br>ACTTGGCTA

PhdFibTec15	D8VNT0	1,07E-08	39,394	PF01391 PF00147	10287,56	NAFKDGQNCAITYKGGWWYTSCYQVNINGLYNDQSSTGISWGATAANIMVWTEMKIRPADFAQKSATINLNTVKRNSVDKSYLTDFLFSRK*	AATGCTTTTAAGGACGGTCAGAATTGCGCTATTACCTACAAGGGCGGATGGTGGTATACAAGCTGTTATCAAGTCAATATCAACGGTTTGTACAACGATCAGTCATCAACCGGAATAAGTTGGGGTGCAACCGCTGCGAATATAATGGTATGGACGGAGATGAAAATAAGACCAGCAGACTTTGCACAGAAATCTGCAACCATCAACTTAAACACGGTTAAAAGAAATTCGGTAGATAAAAGTTATTTGACGGATTTTCTTTTTTCTAGAAAATAGTCCGCCAGATTTACAATTAATAATTTGAAAACG
PhdFibTec16	D8VNS8	3,65E-33	51,786	PF01391 PF00147	12561,63	GNDNIFALTSQRANTVKFSLSDWEGNKTYAAYDEFWIDDEAHNYTMHVGGYRGTAGDSFSFTNGIPFTTKDRDNDKYKTNCAVSFKGAWWYTACHTSNLNGLYLKGAHESY	GGGAATGACAATATTTTTGCCCTGACCAGCCAAAGAGCCAATACTGTGAAATTTTCTCTATCAGATTGGGAAGGAAATAAGACTTATGCAGCATATGACGAATTCTGGATAGATGACGAAGCACACAATTATACAATGCATGTTGGAGGATACAGAGGTACTGCAGGTGATAGCTTCTCTTTTACCAATGGAATTCCATTTACTACGAAGGACAGAGATAACGATAAATATAAAACGAACTGTGCTGTTTCTTTCAAAGGCGCATGGTGGTATACGGCCTGCCATACTTCGAATCTTAATGGATTGTATTTAAAAGGTGCTCATGAATCTTAT
PhdFibTec17	D8VNT0	4,46E-29	47,863	PF01391 PF00147	14654,05	TDWEGNTTYATFDDFWIDNESRNYTLHATGYNGTAGDSLSRHNGQAFSTKDKDNDRIRDNCAVMRKGAWWYTDCNASNLNGMHFKGALTTDYEGIEWMSFKGGKYSLKDTVIKIRPKDFQKDLGSSVTP	GCGGAGTTACTGAGGAGCCAAGGTCCTTCTGGAAATCCTTAGGACGAATTTTTATCACAGTATCTTTCAGAGAATACTTCCCTCCTTTGAAAGACATCCATTCGATTCCTTCGTAATCCGTTGTAAGAGCTCCCTTAAAATGCATTCCGTTAAGATTTGAAGCATTACAATCTGTATACCACCAAGCTCCTTTACGCATAACAGCACAGTTATCACGGATCCTGTCATTGTCTTTATCTTTAGTACTAAATGCTTGTCCATTATGGCGAGATAAAGAATCACCAGCTGTTCCATTGTAACCTGTTGCATGCAATGTGTAATTCCTAGATTCGTTATCAATCCAGAAATCGTCGAATGTCGCATAGGTTGTATTTCCCTCCCAATCTGTCA

PhdFibTec18	P85031	2,96E-44	52,000	PF00147	18148,71	SFGDVKEDFWLGND<br>NIYALSNQKPYSVRF<br>DLEDSEGSIRFAVYD<br>TFWVDNERLNYTIHI<br>QGYSGDAGDSMGTE<br>HDGKMFSTADKDN<br>DIHDSNNCAVAYKG<br>GWWYGSCHYSNLN<br>GFYHGGKHESYADG<br>VNWHSWKGYHESL<br>KSTAIKIRRAKSSTIQ<br>DMK*	TCTTTTGGCGACGTAAAAGAAGATTTCTGGCTAGGAAACGAC<br>AATATATATGCCTTGAGTAACCAAAAGCCGTATTCCGTTCGA<br>TTTGACTTAGAAGATTCTGAAGGAAGCATACGTTTTGCTGTCT<br>ATGATACATTTTGGGTAGACAACGAAAGACTAAACTATACC<br>ATACATATACAGGGATACAGTGGAGATGCAGGCGATTCCAT<br>GGGTACGGAACATGATGGTAAGATGTTCTCAACAGCAGATA<br>AAGACAATGACATTCATGATTCCAATAACTGTGCTGTAGCCT<br>ACAAAGGAGGATGGTGGTATGGTAGCTGTCACTATTCCAATT<br>TAAATGGTTTCTATCATGGTGGAAAGCACGAGAGTTATGCGG<br>ACGGAGTAAACTGGCACTCGTGGAAAGGTTACCATGAATCA<br>CTGAAATCAACAGCAATCAAAATAAGAAGAGCAAAATCTTC<br>CACTATACAAGATATGAAATAAGAAGTGAAGATCATCGAGA<br>GATTTGTTTGAAGTCCTACAATTATTTGTTTAGTTTATTCTGTA<br>ATTTGATATAAAATTTTTAAAAACAATAGTGGATAAGATATG<br>TGCTTGCATCGTAGAAATTGTGCATTTCAACATATCAGTAAA<br>TGCCACGAATTTGAATTAATTAATGCAATAGATAATAAGTAG<br>TACTGCATATTCTCAGTCTTTTCTGTCATGTATGATACTGAAC<br>ATGTAAAAAAATTTTTTTAGTGTTATAGTATTTGGGTCTGGGT<br>CGAAATCCTGACAGCCTAAATCCCGTCAAGCCTAAATCCCG
PhdFibTec19	P85031	2,85E-13	72,973	PF00147	7858.04<br><br>**5778.51**	MIHRAFLISLLFHLSV<br>EA**IQGNWTSEPSFT**<br>**ATPEFSSVCERKGK**<br>**ATGSLNLALQLIAD**<br>**VRDNFPVCPSSPLP**	GGGAAGTGGTGATGAAGGACAAACTGGGAAATTGTCTCGCA<br>CGTCTGCTATGAGTTGAAGAGCTAAATTCAAGCTGCCTGTTG<br>CTTTGCCTTTCCTTTCACACACACTTGAAAATTCTGGAGTTGC<br>AGTAAAAGATGGTTCACTTGTCCAGTTCCCCTGAATCGCTTC<br>GACAGAAAGGTGGAACAGGAGAGAAATTAGAAACGCTCTG<br>TGAATCATTCTGAGACCAAACTGAAAACGTAGACCTAAACT<br>CTCATGGATATTCACACGTTAGGATTAG

PhdFibTec20	P85031	1,30E-55	40,625	PF00147	31688.45<br>**29507.86**	MFQYAVTLLIFIDVTLSHS**ACCEDEVRSKSLLDAASDFISKAKAVMPKCSEEEACLKQNIDCADLYRSGHNKSGIYNIAPMSRLFHKKQIQVYCDMETDGGGWTVIQRRGDFGKRDDYFNQGWAEYKIGFGTVDTDFWLGNENIFALTNQQAYTVRFDLEDRKGVRAFAKYDNFWIDDENHLFTLHISGFTGNAGDGMVYHNGMKFTTKDRDNDLRSDSNCGQTWKSGWWFNSCYDGYSSLNGVYNAKTNGGIDWMPFRWEESLRWTEIKIKPKF***	TTTTATTTCCCAGATACACAGATATTTCTGAAATATGTGACTCAGTAAGTGGAGCTCATGTCGCTTTCAGATTTTAGGGGAATAACGCTTTGTAAAAATATTTGTTGAAATTAAATATGTGTTAACAAAAGAGTGATATCTGGCATCTTAAAATTTAGGTTTTATTTTTATTTCTGTCCAGCGTAAGGATTCTTCCCATCTGAATGGCATCCAATCGATTCCTCCATTTGTCTTGGCATTGTAGACCCCATTCAAACTGCTGTATCCATCGTAGCATGAATTAAACCACCATCCACTTTTCCAGGTTTGACCACAGTTGCTATCTGACCTAAGATCATTATCTCTGTCCTTTGTTGTAAATTTCATTCCATTGTGGTATACCATTCCATCCCCTGCATTTCCAGTGAAACCAGAAATATGTAGTGTGAACAGGTGGTTCTCGTCGTCAATCCAAAAATTGTCATACTTGGCAAATGCTCTTACTCCCTTTCTATCCTCCAAATCAAAGCGCACGGTGTAAGCCTGTTGATTGGTCAAAGCAAAGATATTTTCGTTTCCCAGCCAGAAGTCAGTGTCCACAGTGCCGAATCCAATTTTGTATTCTGCCCACCCTTGGTTGAAATAGTCATCTCGTTTCCCAAAGTCTCCTCTTCTTTGTATTACAGTCCATCCACCACCATCGGTTTCCATGTCACAATAGACTTGAATCTGTTTCTTATGGAATAATCTACTCATGGGAGCGATGTTGTATATACCACTTTTATTGTGACCACTTCTGTACAGATCGGCGCAGTCTATATTTTGCTTAAGACATGCTTCTTCTTCAGAACATTTAGGCATAACTGCCTTTGCCTTAGAAATAAAGTCACTTGCTGCATCCAAGAGTGATTTAGACCTCACCTCGTCTTCACAGCATGCTGAATGACTTAAGGTTACGTCGATAAATATCAGCAGTGTGACAGCGTATTGAAACATGGTTCAAAGAAAACCGTATCCCCTGAAATTCGCCGATGTCTGG

PhdFibTec21	P85031	5,35E-46	51,875	PF00147	20404,43	GSGQDYFYRDWQSY<br>KHGFGDLKKDFWL<br>GNENIFALTNQRSN<br>VVKFTLTDWEENTT<br>YATFDVFWVDDESR<br>KYTLHATGYSGIAG<br>DSLSRHNGQAFSTK<br>DQDNDKSSGNCAIS<br>YKGAWWYEECHAS<br>NLNGLYFTEAFTPSE<br>LGIVWRSFKGRYYSL<br>KDSVIKIRPTSFQKNS<br>AALLGPQL*	TGGCAGCGGACAAGATTATTTTATAGAGACTGGCAGTCTTA<br>TAAACATGGCTTCGGAGATCTAAAGAAGGATTTCTGGCTAGG<br>TAATGAGAATATCTTCGCGCTAACAAATCAAAGATCGAATGT<br>TGTGAAATTTACGTTGACTGACTGGGAGGAAAATACGACTTA<br>TGCAACTTTCGATGTATTTTGGGTTGATGACGAATCTCGGAA<br>ATATACACTGCATGCCACGGGATACAGTGGAATAGCTGGTG<br>ATTCATTGTCTCGTCATAATGGACAAGCTTTTAGCACTAAAG<br>ATCAAGACAATGACAAGAGCAGTGGTAACTGTGCTATTTCTT<br>ACAAAGGCGCTTGGTGGTATGAAGAATGTCATGCTTCAAATC<br>TTAATGGACTGTATTTTACAGAAGCATTTACACCTTCGGAACT<br>CGGAATTGTATGGAGATCATTTAAAGGCAGATACTATTCACT<br>GAAAGATTCTGTGATAAAAATTCGTCCTACTAGTTTCCAGAA<br>GAATTCAGCTGCATTACTCGGTCCCCAGTTGTAATATTAAGG<br>AAACTGAAAACGTTTTTTGAAACGAGATTGTGACTGGCTCAA<br>GGAATATTAACACACTTTTGAAATGATCATTTTGCATGAGAC<br>GAAATACTTATAGAATTGTCATTTAGCCTTAATATATTTCAGA<br>AAGGAACATCTGGAAAAATCATTCTTATAAGACAATTTACTA<br>CATTTTTATGTATACAACATAACAACTGTCATGACAGGGAGC<br>GAAAATATGTCCCTTATAAAG
PhdFibTec22	P85031	2,78E-25	59,211	PF00147	12174.95<br>**10039.35**	MIHRAFLISLLFYLTV<br>ES**IPGNGTSELSFIA**<br>**TPEFSSECETKGKAT**<br>**GSLNLALQLIADVR**<br>**DNYPVCPSSPLPIDC**<br>**EDVLRRGQNKSGV**<br>**YTVLPRSRAIPNPIE**<br>**VYCDLITD**	AAAAGCAGCATTTATCACAGATGTTCCTAATCCTAACGTGTG<br>AACATCCATGAGAATTTAGGTCTACGCTTTGAGTTTGGTCTCA<br>GAATGATTCACAGAGCGTTTTTAATTTCGCTCCTCTTCTACCT<br>TACTGTCGAATCGATTCCAGGGAACGGGACAAGTGAATTGTC<br>TTTTATTGCAACTCCAGAATTTTCAAGTGAATGTGAAACGAA<br>AGGAAAAGCAACAGGAAGCTTGAATTTAGCTCTTCAACTCA<br>TAGCAGACGTGCGAGACAATTACCCAGTGTGTCCTTCATCAC<br>CACTTCCCATAGACTGTGAAGATGTATTACGACGTGGTCAGA<br>ACAAGAGTGGTGTCTACACAGTTCTACCACGGAGCAGAGCA

							ATACCAAATCCGATCGAAGTTTACTGTGATTTGATCACTGACGG
PhdFibTec23	P85031	5,25E-31	74,576	PF00147	8561,48	WLVNRITDDKSLLVYCDMQTDGGGWTIIQRRGNYQNPADYFFKDWKSYKEGFGDIERDFWLGNDNIFALTNQ	TATGGCTTGTGAATCGAATAACGGATGATAAATCACTACTTGTATACTGTGATATGCAAACCGATGGAGGTGGTTGGACCATAATACAGAGAAGAGGAAATTATCAGAACCCAGCTGACTATTTCTTCAAAGACTGGAAAAGTTATAAAGAAGGATTTGGAGATATCGAAAGAGATTTTTGGTTGGGAAATGATAACATCTTTGCTTTGACGAACCAGA

PhdFibTec24	P85031	2,76E-56	47,706	PF00147	34563.26<br>**32709.01**	MFAIVAVFILCSIASSEA**AENGKCDSELSKNVTEYSRASFIVTLLNNAGNLLEEAKRQASVRTSCMGSQSSSCEKPMDCFEIYEKDQTAKSGVYKIWPRSRVSSAAVNVYCDMNDDSGTWTLIQRRGDFDSEQNYFYQDWSSYKNGFGDPLKDFWLGNDNIFALTNQRANSVKFVLADWEGNTTYAIYDEFWIDDEINKYRAHVKGHRGTAGDSFSGTNGVPFSTKDRDNDSYEKNCAEQFKGAWWYTACHASNLNGLYLKGPHKSHADGVEWQAFRGHYYSLKDTVIKIRPRDFTAPRMDVTPE***	TATTAACGCCTTATTCAGCATTTCATTCGTAGAAATCGGAAAAGGACGTGATTATCAGCTCTCATTTACGCAGAAACTGTCTCAGGTAATTAGATAGTGCGTAAGTTCATATTACAGATCGTCGGATTTCTAGCTGGATTAGCCGTGTCGAGAAAATTTCTTGGAAATGTTGCGAAATCTGCAATATTGCATCAGTCTGAAGTCACCGAACGAGAGCTGCGTAGCTAACGGTGATTCCCCTAGCTTCTCATCATAAGCGGAAAGGAAAATTTGGATTCAGCAATGTTCGCAATAGTGGCTGTTTTCATTTTGTGTTCAATTGCTTCTTCTGAAGCAGCTGAAAACGGAAAGTGTGATTCTGAGTTATCCAAAAACGTGACAGAATATTCGCGTGCATCTTTTATTGTCACTCTGTTAAATAATGCCGGAAATCTTCTCGAAGAAGCGAAGCGACAGGCGTCAGTTAGAACAAGTTGTATGGGTTCTCAGTCATCATCATGTGAAAAGCCGATGGATTGTTTTGAAATCTATGAGAAGGATCAAACTGCTAAAAGTGGAGTCTATAAAATTTGGCCTCGCAGCAGAGTATCATCTGCAGCTGTCAATGTGTATTGCGACATGAATGACGATAGCGGAACTTGGACGCTGATACAGAGGCGTGGAGATTTCGATAGTGAGCAAAATTACTTCTATCAAGATTGGTCAAGTTATAAAAATGGATTTGGAGATCCCCTAAAGGATTTCTGGCTTGGAAACGACAATATTTTTGCTCTGACCAACCAGAGAGCAAATTCTGTCAAATTTGTATTAGCTGACTGGGAGGGAAATACTACTTATGCAATATACGACGAATTCTGGATTGATGATGAAATAAATAAGTACAGAGCGCATGTGAAAGGCCACAGAGGGACGGCAGGAGATTCTTTCTCTGGAACTAACGGAGTACCATTCTCAACTAAAGATAGAGACAATGACAGCTACGAGAAAAATTGCGCAGAACAATTCAAAGGAGCCTGGTGGTATACAGCCTGTCATGCCTCCAACCTGAACGGGCTATATTTGAAAGGACCTCACAAAAGTCATGCTGACGGTGTGGAGTGGCAGGCTTTCAGGGGCCATTACTATTCCCTGAAAGACACCGTTATCAAAATAAGGCCAAGAGATTTCACA

							GCACCCAGAATGGATGTTACACCTGAATAATAGGTGAATTCT<br>GATGGTGATCATAATATACGATGTGCGAAGTTATATGGTACA<br>ATGATTGAAGTTTAATAAAAATTACCCATAATCTCTTATCTAG<br>TAATTTCGAAACATACACATTCTGCAAATAACTGTTGAAATC<br>AGAAAACTCTGAACTAAATCAATATGTTGATCACCCTCCTTC<br>GCACTGACTACTTATCTGATCTTTGCTTTCCGCAATTTCCAGC<br>ACGTCTCCATTAGAATATGAAACTATTTCTAGATTAAAGCCG<br>CTGCAAGAAGGGAGGTCGCACATGACGGAACAAGCGACAG<br>AAACCCTCATGAGTGAACAAACTGTTTTGTAACCCAATTTTT<br>CCGTTTTAAATAACTGTTTTTTTATGTAATTTTTGTCATTTGTG<br>CCTCTCAGTGTGTGAGAGAGATTTTTCCATAA

PhdFibTec25	P85031	5,74E-72	49,794	PF00147	34850,13	MNCSMASLLDRLKLGFFINVLLILLTFIDLNHTQNCVTPILPKLYLDSAMTLLQMAKDSYPSEYAGRMARPADCYDVLNLGNNTSGIYNIWPLSRVTGGRSVQVYCDMNTDGGGWTVIQRRGDFQRAVDFFYKDWESYKNGFGDLDKDFWLGNNNIFAISNQKQYSARFDLKAVDGEKRYAHYDFFWIDDENHKYMLHIRDYSGDAGDSMYPKHDNQMFSTKDQKNDNHTTVNCAVSFKGAWWYNACHDSNLNGLYLNGIHESHANGVNWKSWKGHKESLKETEIKLRPKDCKRSQTDVTPQ*	TTTTTTTTTGAATCTTCAAAAACTTGTTTATTACCATCTTCAGCAGAAGCGAATTACTGCTGTTCTATTATTGGGGTGTTACATCAGTTTGGCTTCTTTTGCAATCTTTTGGCCTGAGTTTTATTTCTGTTTCTTTTAAAGATTCTTTATGACCTTTCCAAGATTTCCAGTTGACTCCATTCGCGTGACTCTCATGTATTCCATTCAAGTACAGGCCGTTGAGGTTCGAATCGTGACATGCGTTGTACCACCAAGCTCCTTTGAAAGAAACCGCACAATTCACAGTAGTATGGTTGTCATTTTTCTGGTCTTTTGTTGAAAACATCTGATTATCGTGTTTTGGATACATAGAATCTCCGGCATCTCCACTGTAGTCTCTGATGTGTAGCATATATTTGTGGTTTTCATCATCTATCCAGAAGAAGTCATAATGAGCATATCTCTTTTCACCGTCTACAGCTTTCAGATCAAATCGAGCACTATATTGCTTCTGGTTGCTGATGGCAAAAATATTGTTGTTACCTAACCAGAAATCTTTATCTAAATCACCAAATCCATTTTTGTAACTTTCCCAGTCTTTGTAAAAAAAATCTACAGCCCGCTGAAAATCTCCTCTTCTCTGGATGACCGTCCATCCACCGCCATCGGTATTCATGTCACAATATACTTGCACGGATCTTCCCCCTGTGACACGACTAAGGGGCCATATGTTGTATATGCCACTTGTATTGTTACCCAAATTCAGCACATCATAGCAGTCGGCAGGTCTTGCCATTCTACCAGCGTACTCTGAGGGATAGGAATCCTTTGCCATTTGCAAGAGTGTCATAGCAGAGTCCAGATACAGCTTTGGAAGTATAGGAGTCACACAATTCTGGGTATGATTTAGGTCAATAAAAGTCAGCAATATAAGTAACACATTTATGAAGAAACCTAATTTCAATCTGTCTAGTAGCGACGCCATGCTGCAATTCATTGTGCTTTTCCAAAATAAACGGATTTCTCCAGTAATTTCCGTACCTTAAATAGAAAACCACTTAGACACCAAGCGTTGCAGCTGTTACGCTCCCTATCCGCTCCTCGGATGACTTTCGCTTTTGGAACAGGTGTTTTCACGCATGCGTCAGAGTGAAAAGGAATTATAGCTAGATCAGTTTTCGCTTCCGAACTGACACAAACCACAGAAATTG

							GTATCTGTACCTCCACAATTCGCCGAGAACGACATAATGTCC CGCTTTTGGAACAG

PhdFibTec26	P85031	1,33E-57	44,248	PF00147	33913.85<br>**31847.36**	MFRTVFLLLLSTAVL<br>SSDA**ARESSCGTAD**<br>**VVKTETLLETAAEL**<br>**LDKMRDNLEQVSA**<br>**NADCSCPPKPMDC**<br>**DEVYKCGNNRSGI**<br>**YEIWPRNRIIVKSVK**<br>**VFCDMETNGGGW**<br>**TVIQRRGEYKRPKD**<br>**FFSKSWLDYKSGFG**<br>**DPRRDFWFGNDNI**<br>**YAMTNQKKYYLRV**<br>**DLADFENRTRYAFY**<br>**DQFWIENEGQNYK**<br>**LHIYDFSGDAGDSL**<br>**AKAHDGSMFSTFD**<br>**RDNDNSTEHCARK**<br>**HSGGWWFNGCLAS**<br>**NLNGLYQGGSVRS**<br>**ADGVVWSSWKKN**<br>**HESLRITEMKIRSA**<br>**DFQTNMLDMDVEP**<br>**A***	GAAAGACTGCGTACGCGAAATATCATTCCCACATCCGATATC<br>CAATCGGTAAATGGTTGCACGAGTTCCTTTTGGTAGGTCCTAT<br>ATATTTTACTCGTTCCATTGTTTCGCGAAGCTGATAGCTGCTT<br>TCGACATTTGATGCTTGAAAAGTAAAGAGATTGTTTTGCAAC<br>TTGCAAACTAGCGTCTGCTTGCTTGTAAAGGTAACCATGTTTC<br>GAACAGTATTTCTGCTTCTACTCTCCACAGCTGTCCTCAGCAG<br>CGACGCAGCTCGAGAAAGTTCGTGTGGGACGGCTGATGTGG<br>TGAAAACTGAAACATTGCTAGAAACTGCAGCTGAACTCCTG<br>GACAAGATGAGAGATAATCTGGAACAAGTGTCAGCTAATGC<br>AGACTGCAGCTGCCCCCCGAAACCCATGGACTGCGACGAGG<br>TGTACAAATGTGGCAATAATCGAAGTGGTATTTATGAAATAT<br>GGCCAAGAAACAGAATAATCGTCAAATCTGTTAAGGTATTTT<br>GCGATATGGAGACCAATGGAGGCGGCTGGACGGTAATCCAA<br>AGGCGTGGTGAGTACAAGAGACCGAAGGACTTTTTTCGAA<br>ATCTTGGCTCGACTATAAATCAGGATTCGGAGATCCTCGCCG<br>AGATTTTTGGTTTGGAAACGACAACATTTACGCTATGACGAA<br>CCAGAAGAAATATTACCTGAGGGTAGACCTCGCCGACTTCG<br>AAAACAGGACAAGATATGCATTCTACGATCAGTTTTGGATTG<br>AAAATGAAGGACAGAATTACAAGCTGCACATCTATGATTTC<br>AGTGGAGATGCAGGTGACTCTTTAGCCAAAGCCCATGATGG<br>ATCCATGTTTTCCACTTTCGACCGCGACAACGACAACAGCAC<br>GGAGCATTGTGCAAGAAAACACAGTGGTGGTTGGTGGTTCA<br>ACGGCTGCCTAGCTTCCAACTTGAACGGACTTTATCAGGGAG<br>GTTCTGTCAGAAGTGCAGACGGCGTTGTGTGGTCTTCCTGGA<br>AGAAAAATCATGAATCACTGAGAATTACTGAGATGAAGATC<br>AGGTCTGCCGACTTCCAGACAAACATGTTAGACATGGATGTC<br>GAACCTGCTTAAATGGGGAGTGTCTCGTACCTTTGTATTGTGC<br>ATTCAGATGTAATAAGAATAGAATTTTTTTTAATGTTATGTAT

							CGTGAAATGACTGTCAATAAACGCCAAAGTTTTTAGAAAAAAA

PhdFibTec27	P85031	3,53E-72	54,587	PF00147	28707,63	MDCSDVLQKGYNKSGVYIIWPINRVLGDKSLEVYCDMQTDGGGWTLIQRRGNFQRANDYFYKDWKSYKEGFGDVEKDFWLGNDNIYALTNQRLYSARFDLRDVENSTRYAHYDSFWIENENHKYTLYIADYSGDAGDSMIDQHNNKKFSTQDQDNDNQNEENCAQKYKGGWWYNVCHSANLNGLYLRGKHESFADGVNWLSWKGYHESLESTEIKIRPKNFRPPRVTLKPHVPAHVNSSDINTPM*	TTTTTTCATATTTTATTAAGCTGTGAGTTAACAGAAGAGAATTTCGTCATTCTTAGAAAAGTTTAATGCCACATGAAAATTCTCTCAATTTTACAGCGATATCCATTCACATTGGAGTGTTTATATCAGAGGAGTTCACATGGGCAGGCACGTGAGGTTTCAAGGTTACACGAGGTGGCCTGAAATTTTTGGGTCGAATTTTTATTTCGGTCGATTCCAAAGATTCGTGATAGCCTTTCCAGGACAACCAATTTACTCCGTCAGCAAAACTTTCATGTTTACCCCTGAGGTACAAACCATTGAGATTGGCGGAGTGGCATACATTATACCACCATCCCCCTTTGTACTTCTGGGCGCAATTTTCCTCATTCTGATTGTCATTATCTTGGTCTTGCGTGGAAAATTTCTTATTATTGTGTTGATCTATCATGGAATCACCTGCATCTCCACTATAATCAGCAATATATAGAGTGTACTTATGATTTTCATTCTCAATCCAGAAACTATCGTAATGGGCGTATCGAGTGCTGTTCTCCACGTCTCTCAAATCAAATCTCGCAGAATAGAGTCTCTGGTTGGTCAAGGCATATATATTGTCGTTTCCCAACCAAAAATCTTTTTCAACATCGCCGAAACCTTCTTTGTAACTTTTCCAATCTTTGTAAAAATAATCATTCGCTCTTTGAAAATTCCCTCTTCTTTGTATTAAGGTCCATCCTCCTCCATCCGTTTGCATATCACAGTATACTTCGAGAGATTTATCTCCAAGCACTCTATTTATAGGCCATATTATGTACACCCCACTTTTATTATAACCCTTCTGGAGAACGTCAGAGCAATCCATGGCTCCTCGGACAATACTTTTTCCACAGTTATCACATTTTCCACCTAATGAAATTCACAGCTTATTGGTTAGAATCATATACCTCATAGAAAATTTAGTTGCTCATCAAATTTCCGACTGATATTGCGCGAGAAACAGTGACAAATGAGTGAATCTATAATTTTTTGAACAAAACTTATATTAATTGAGAACGCATGTCACATGACGCAGTG

PhdFibTec28	P85031	1,73E-75	51,220	PF00147	35224.41<br>**33024.55**	MARILAILVVLCLCCRDIKA**NSSCNPDVSKSYLDLAVDMISKAKQSMPTCDNCGKSIVRGAMDCSDVLQKGYNKSGVYIIWPINRVLGDKSLEVYCDMQTDGGGWTLIQRRGNFQRANDYFYKDWKSYKEGFGDVEKDFWLGNDNIYALTNQRLYSARFDLRDVENSTRYAHYDSFWIENENHKYTLYIADYSGDAGDSMIDQHNNKKFSTQDQDNDNQNEENCAQKYKGGWWYNVCHSANLNGLYLRGKHESFADGVNWLSWKGYHESLESTEIKIRPKNFRPPRVTLKPHVPAHVNSSDINTPM***	TTTTTTCATATTTTATTAAGCTGTGAGTTAACAGAAGAGAATTTCGTCATTCTTAGAAAAGTTTAATGCCACATGAAAATTCTCTCAATTTTACAGCGATATCCATTCACATTGGAGTGTTTATATCAGAGGAGTTCACATGGGCAGGCACGTGAGGTTTCAAGGTTACACGAGGTGGCCTGAAATTTTTGGGTCGAATTTTTATTTCGGTCGATTCCAAAGATTCGTGATAGCCTTTCCAGGACAACCAATTTACTCCGTCAGCAAAACTTTCATGTTTACCCCTGAGGTACAAACCATTGAGATTGGCGGAGTGGCATACATTATACCACCATCCCCCTTTGTACTTCTGGGCGCAATTTTCCTCATTCTGATTGTCATTATCTTGGTCTTGCGTGGAAAATTTCTTATTATTGTGTTGATCTATCATGGAATCACCTGCATCTCCACTATAATCAGCAATATATAGAGTGTACTTATGATTTTCATTCTCAATCCAGAAACTATCGTAATGGGCGTATCGAGTGCTGTTCTCCACGTCTCTCAAATCAAATCTCGCAGAATAGAGTCTCTGGTTGGTCAAGGCATATATATTGTCGTTTCCCAACCAAAAATCTTTTTCAACATCGCCGAAACCTTCTTTGTAACTTTTCCAATCTTTGTAAAAATAATCATTCGCTCTTTGAAAATTCCCTCTTCTTTGTATTAAGGTCCATCCTCCTCCATCCGTTTGCATATCACAGTATACTTCGAGAGATTTATCTCCAAGCACTCTATTTATAGGCCATATTATGTACACCCCACTTTTATTATAACCCTTCTGGAGAACGTCAGAGCAATCCATGGCTCCTCGGACAATACTTTTTCCACAGTTATCACAAGTTGGCATACTCTGTTTCGCTTTGCTAATCATATCAACTGCTAAATCTAAATAAGACTTGGAAACGTCCGGGTTACAACTGGAATTTGCTTTGATATCTCTGCAACACAGACAAAGGACTACTAGAATTGCCAAGATTCTTGCCATTTTCCACCTAATGAAATTCACAGCTTATTGGTTAGAATCATATACCTCATAGAAAATTTAGTTGCTCATCAAATTTCCGACTGATATTGCGCGAGAAACAGTGACAAATGAGTGAATCTATAA

							TTTTTTGAACAAAACTTATATTAATTGAGAACGCATGTCACAT<br>GACGCAGTG
PhdFibTec2<br>9	P85031	3,79E-34	59,000	PF00147	25220.07<br><br>**23020.41**	MSRYLSLLVVVSLCY<br>ANSKA**TSNCEEKVS**<br>**LTYLDVAADMIDK**<br>**ARHHIPDCADCKC**<br>**DQSRRVMDQSDEQ**<br>**QMRYNISGTHSGS**<br>**NLTRGAMDCSELL**<br>**QMGNTKSGVYTIW**<br>**PVSRITDDKPQLVY**<br>**CDMQTDEGGWTII**<br>**QRRGNYQNPDDYF**<br>**FKDWSSYKQGFGH**	TAATTAGAAATGTGCAATGTGTATTTGCAATTTTCGTCGTCGA<br>TCCAGAAAGCATCGTAATGGGCGTACCTGGTATTGTTCTCCA<br>CGTCTCTCAAATCAAACCTCACGGAGTAAAGTTTCTGATTGG<br>TCAAGGCAAATATGTTGTCGTTTCCCAACCAAAATTCTCTTTC<br>GATGTGCCCAAAACCTTGTTTATAACTGCTCCAGTCTTTGAAG<br>AAATAGTCGTCTGGATTCTGATAATTTCCTCTTCTTTGTATTAT<br>GGTCCAACCACCTTCATCGGTTTGCATATCACAGTATACCAG<br>TTGTGGTTTATCATCCGTTATTCGACTCACAGGCCATATAGTA<br>TACACACCACTTTTAGTATTACCCATTTGGAGTAATTCGGAA<br>CAATCCATGGCTCCTCGGGTGAGATTACTTCCGCTGTGAGTA<br>CCACTTATATTATAACGCATTTGCTGCTCGTCAGATTGATCCA<br>TTACTCTTCGGCTCTGATCACATTTACAGTCCGCACAGTCTGG

						**IEREFWLGNDNIFA LTNQKLYSVRFDLR DVENNTRYAHYDA FWIDDENCKYTLHI SN**	AATATGATGTCTCGCTTTGTCAATCATGTCAGCCGCTACATCC AGATAAGTTAGTGACACCTTCTCCTCACAATTTGAAGTTGCTT TGCTATTTGCATAGCAAAGAGAAACGACTACTAGCAATGAC AAGTATCTCGACATTTCACTCCCAAGAAATTCACAAAATAAA TAGCGCAGTTAGAAAATTTTGCTGCTCATTACAATTGCGGAT GAAGACCGCACTCGAAATTAA
PhdFibTec30	P85031	1,17E-67	54,497	PF00147	32201,5	ARHHIPDCADCKCD QSRRVMDQSDEQQ MRHNISGTSSGSNLT RGAMDCSDLLQMG HNRSGVYTIWPVSRI TEDKSLLVYCDMQT DGGGWTIIQRRGNY QNPVDYFFKEWKSY KEGFGDIEKEFWLG NDNIFALTNQKLYS ARFDLRDVENNTRY AHYDAFWIDDENY NYTLHISNYSGDAG ESLLEQHHNQKFST KDQDNDNSKSVHC AQLAKGGWWYGA CQHANLNGMNRRG KHKESDKYGISWYS	ATCTGTTTAAAGACACGTGATGCAGACCGATGAACTTCTTGG GTCGAATTTTTATTTCAGTCGATTCCATAGATTCATCGTGGCC TTTCCAGCTATACCAACTTATTCCATATTTATCAGATTCTTTAT GTTTTCCCCTCCGATTCATCCCATTCAGATTGGCATGTTGACA TGCACCATACCACCATCCCCCTTTAGCTAATTGGGCACAATG TACAGATTTAGAATTATCATTATCCTGGTCTTTTGTGGAGAAT TTCTGATTGTGATGTTGTTCCAATAGTGATTCACCTGCGTCTC CGCTGTAATTAGAAATGTGCAATGTGTAGTTGTAATTTTCGTC GTCGATCCAGAAAGCATCGTAATGGGCGTACCTGGTATTGTT CTCCACGTCTCTCAAATCAAACCTCGCTGAATAAAGTTTCTG ATTGGTCAAGGCAAATATGTTGTCATTTCCTAACCAAAATTCT TTTTCGATGTCCCCGAAACCTTCTTTATAACTTTTCCACTCTTT GAAGAAATAGTCAACTGGATTCTGATAATTTCCTCTTCTTTGT ATGATAGTCCAACCACCTCCATCGGTTTGCATATCACAGTAT ACAAGTAGTGATTTATCTTCAGTTATTCGACTCACAGGCCAT ATAGTATACACACCACTTCTGTTATGACCCATTTGGAGTAAG TCGGAACAATCCATGGCTCCTCGAGTCAGATTACTTCCGCTG CTAGTACCACTTATATTATGACGCATTTGCTGCTCGTCAGATT

						WKGHDESMESTEIKI<br>RPKKFIGLHHVSLNR	GATCCATTACTCTTCGGCTCTGATCACATTTACAGTCCGCACA<br>GTCTGGAATATGATGTCTCGCTT
PhdFibTec3<br>1	P85031	2,21E-57	59,155	PF00147	25422,92	ARHHIPDCADCKCD<br>QSRRVMDQSDEQQ<br>MRHNISGTSSGSNLT<br>RGAMDCSDLLQMG<br>HNRSGVYTIWPVSRI<br>TEDKSLLVYCDMQT<br>DGGGWTIIQRRGNY<br>QNPVDYFFKEWKSY<br>KEGFGDIEKEFWLG<br>NDNIFALTNQKLYS<br>ARFDLRDVENNTRY<br>AQYEAFWIDEENCK<br>YTLHISNYSGDAGDS<br>LIGQHNNQKFTTKD<br>QDNDVYEHVNCAQ<br>◎◎◎◎◎◎ [illegible]	GCACTATACCACCATGCCCCTTTAGCTAATTGGGCACAATTT<br>ACATGTTCATAAACATCATTATCCTGGTCTTTTGTGGTAAATT<br>TCTGATTGTTATGTTGTCCGATCAGTGAATCGCCTGCGTCTCC<br>GCTGTAATTAGAAATGTGCAATGTGTATTTGCAATTTTCCTCG<br>TCGATCCAGAAAGCCTCGTATTGAGCGTACCTGGTATTGTTCT<br>CCACGTCTCTCAAATCAAACCTCGCTGAATAAAGTTTCTGAT<br>TGGTCAAGGCAAATATGTTGTCGTTTCCTAACCAAAATTCTTT<br>TTCGATGTCCCCGAAACCTTCTTTATAACTTTTCCACTCTTTGA<br>AGAAATAGTCAACTGGATTCTGATAATTTCCTCTTCTTTGTAT<br>GATAGTCCAACCACCTCCATCGGTTTGCATATCACAGTATAC<br>AAGTAGTGATTTATCTTCAGTTATTCGACTCACAGGCCATATA<br>GTATACACACCACTTCTGTTATGACCCATTTGGAGTAAGTCG<br>GAACAATCCATGGCTCCTCGAGTCAGATTACTTCCGCTGCTA<br>GTACCACTTATATTATGACGCATTTGCTGCTCGTCAGATTGAT<br>CCATTACTCTTCGGCTCTGATCACATTTACAGTCCGCACAGTC<br>TGGAATATGATGTCTCGCTT

PhdFibTec32	P85031	1,76E-66	53,968	PF00147	26669,46	SDLLQMGNNRSGVYTIWPVSRITEDKSLLVYCDMQTDGGGWTIIQRRGNYQNPVDYFFKEWKSYKEGFGDIEKEFWLGNDNIFALTNQKLYSARFDLRDVENNTRYAQYEAFWIDEENCKYTLHISNYSGDAGESLFERHNNQKFSTKDQDNDASKSGDCALGSKGGWWYGACHTANLNGMNRRGKHKESDKYGISWYSWKGHDESMESTEIKIRPKKFIGLHHVSLNR	ATCTGTTTAAAGACACGTGATGCAGACCGATGAACTTCTTGGGTCGAATTTTTATTTCAGTCGATTCCATAGATTCATCGTGGCCTTTCCAGCTATACCAACTTATTCCATATTTATCAGATTCTTTATGTTTTCCCCTCCGATTCATCCCATTCAGATTGGCAGTGTGACATGCACCATACCACCAACCCCCTTTAGATCCTAGGGCGCAATCTCCAGATTTAGAAGCATCATTATCCTGGTCTTTTGTGGAAAATTTCTGATTGTTATGACGTTCGAACAGTGATTCACCTGCGTCTCCGCTGTAATTAGAAATGTGCAATGTGTATTTGCAATTTTCCTCGTCGATCCAGAAAGCCTCGTATTGAGCGTACCTGGTATTGTTCTCCACGTCTCTCAAATCAAACCTCGCTGAATAAAGTTTCTGATTGGTCAAGGCAAATATGTTGTCGTTTCCTAACCAAAATTCTTTTTCGATGTCCCCGAAACCTTCTTTATAACTTTTCCACTCTTTGAAGAAATAGTCAACTGGATTCTGATAATTTCCTCTTCTTTGTATGATAGTCCAACCACCTCCATCGGTTTGCATATCACAGTATACAAGTAGTGATTTATCTTCAGTTATTCGACTCACAGGCCATATAGTGTACACACCACTTCTATTATTACCCATTTGGAGTAAGTCGGAAC

PhdFibTec33	P85031	2,14E-61	53,439	PF00147	31837,08	ARHHIPDCADCKCDQSRRVMDQSDEQQMRHNISGTSSGSNLTRGAMDCSDLLQMGHNRSGVYTIWPVSRITEDKSLLVYCDMQTDGGGWTIIQRRGNYQNPVDYFFKEWKSYKEGFGDIEKEFWLGNDNIFALTNQKLYSARFDLRDVENNTRYAQYEAFWIDEENCKYTLHISNYSGDAGESLFERHNNQKFSTKDQDNDASKSGDCALGSKGGWWYGACHTANLNGMNRRGKHKDSDKNVISWSSWKGDDESLESIEIKIRPKKFIGPHHVNNPV*	GATTTATCAATGATTTGGCGCTAAATGTTAAAATTGAAAGTAAGGCGAAGTAACTCTAACTATCACTATTATCTAAAGTTCCATTCTTGGCGATCAAATGCAGGGTGGTGTTTCTGGTGCCTTCGAGTTAAAGTAATTTAGGTCTGTCAGTAGATAGATGTGCAAATAAGGGACACTTTTCAGAATATTGAGTTAGCGTCATAAATTTAACTGTTAAAATAAGATGAGGGGGGATAACTGTCGAAATTTTTGAATTCAAATTATACGATGTACAACGATTGGATTTGGTATTTATGTTTATTATCGTATGACTTTCCTTTTGTGTTAAGGTAAATTAATTTAAATTTTAGAGTTTAGACGGACCATTGTCGTTCCCTATTGACGAACTGCAACTGCATATATATAAAGAACCGACATGCAGATTCATATAAATATACACATAAGAAATGTTAATCAGTGCTTGAATAGTCAGCCTATGAGTGTGTCGTATAAGTGTTTACGCTTGTGTAAGCAAGAGACATTTTGCTGTTTTATGCATTATTTTTCATAAATTTAGGGATTTAAAAATGCATCAATGACGCGCCAATGTATTTTACGAAAAATTAGTAAGGCCTGTCCACATAGCCAATATGTGAATGTTTGTTTTGCGCAATTTTCTAAATAATTATAGTGTTTTTACAAGATATTAACTTCATGTCTCTAATGAAAGTTGTTTTTTCGTTAAAAAAAATTTATCCTTTTTATAAAAGGTCCTTAAAGAAGCATGAGTATACTCGCGAATTTAAAACATGTCAGTTATAAATATTCAGACAGGATTGTTTACGTGATGAGGCCCGATGAACTTCTTGGGTCGAATCTTTATTTCAATCGATTCCAAAGATTCATCATCGCCTTTCCAGCTACTCCAGCTTATTACATTTTTATCAGAATCTTTATGTTTTCCCCTTCGATTCATCCCATTCAGATTGGCAGTGTGACATGCACCATACCACCAACCCCCTTTAGATCCTAGGGCGCAATCTCCAGATTTAGAAGCATCATTATCCTGGTCTTTTGTGGAAAATTTCTGATTGTTATGACGTTCGAACAGTGATTCACCTGCGTCTCCGCTGTAATTAGAAATGTGCAATGTGTATTTGCAATTTTCCTCGTCGATCCAGAAAGCCTCGTATTGAGCGTACCTGGTATTG

							TTCTCCACGTCTCTCAAATCAAACCTCGCTGAATAAAGTTTCTGATTGGTCAAGGCAAATATGTTGTCGTTTCCTAACCAAAATTCTTTTTCGATGTCCCCGAAACCTTCTTTATAACTTTTCCACTCTTTGAAGAAATAGTCAACTGGATTCTGATAATTTCCTCTTCTTTGTATGATAGTCCAACCACCTCCATCGGTTTGCATATCACAGTATACAAGTAGTGATTTATCTTCAGTTATTCGACTCACAGGCCATATAGTATACACACCACTTCTGTTATGACCCATTTGGAGTAAGTCGGAACAATCCATGGCTCCTCGAGTCAGATTACTTCCGCTGCTAGTACCACTTATATTATGACGCATTTGCTGCTCGTCAGATTGATCCATTACTCTTCGGCTCTGATCACATTTACAGTCCGCACAGTCTGGAATATGATGTCTCGCTT

PhdFibTec34	P85031	2,08E-48	42,857	PF00147	34442,96	EALRKNASSYSNVFGIIFLTKFDTRSLVHFRIANRLASAIPKCSDTQASQAACVKESLDCADLFRNGYNKSGIYNITLVNRLFYKKRLEVYCDMETDGGGWTVIQRRGDFQREKHYFYRNWTEYKEGFGVKEEDFWLGLLYIYAITNQKEYSARFDLQDGRRHKRYAHYDIFWVDDEHHFFTLHIANYTGDAGDSMISEGRYNHNNQKFSTYDNDNDNSDDTSCAEKYQGAWWYNACHRSNLNGLDYKGKYRGVADGRHWATWKQEEEENEKEEKDKDKKDKDKKDKDKKD	GCTGATGAGACGGTTCTTTATAAGAAGCGTTACGTAAAAATGCATCTTCCTACTCGAATGTATTTGGAATTATTTTCCTCACAAAATTCGACACAAGAAGTTTAGTTCATTTCAGAATCGCGAATCGTCTTGCTTCCGCAATTCCAAAATGTTCTGACACGCAGGCCAGTCAAGCAGCATGTGTGAAGGAAAGCTTAGATTGCGCTGATTTGTTCAGAAATGGTTACAATAAGAGTGGTATATACAACATCACGCTCGTGAACAGGCTATTCTACAAGAAACGCCTTGAAGTCTATTGTGATATGGAAACAGATGGAGGTGGATGGACTGTAATTCAAAGGAGGGGAGATTTTCAGAGGGAGAAACATTACTTTTATAGAAACTGGACTGAATATAAAGAAGGATTTGGAGTTAAGGAAGAAGATTTTTGGTTAGGTCTCCTCTACATATATGCTATAACAAATCAGAAAGAGTATTCAGCAAGATTTGATTTGCAAGATGGAAGGCGCCATAAAAGATATGCACATTACGACATCTTTTGGGTTGACGATGAACACCATTTCTTCACTCTGCACATTGCAAACTACACAGGAGATGCAGGAGATTCTATGATCTCAGAAGGTCGTTACAATCACAATAATCAAAAATTTTCTACCTACGACAATGATAACGACAATTCAGATGACACTTCGTGTGCTGAGAAGTATCAAGGAGCGTGGTGGTACAATGCATGCCACAGATCCAATCTGAATGGGCTAGACTACAAAGGAAAGTACAGAGGTGTAGCGGATGGAAGACACTGGGCAACCTGGAAACAGGAAGAGGAAGAAAATGAAAAGGAGGAAAAGGATAAGGACAAAAAGGATAAGGACAAAAAGGATAAGGACAAAAAGGAT

PhdFibTec3<br>5	P85031	2,65E-52	40,873	PF00147	35215.84<br><br>**33064.22**	MFQYAILLLSFVSMC<br>KTSS**HTSCCEQEVR**<br>**TKSLLDTAIDLISNA**<br>**KSNIPSCSDTQASQ**<br>**AACVKESLDCADLF**<br>**RNGYNKSGIYNITL**<br>**VNRLFYKKRLEVYC**<br>**DMETDGGGWTVI**<br>**QRRGDFQREKHYF**<br>**YRNWTEYKEGFGV**<br>**KEEDFWLGLLYIYAI**<br>**TNQKEYSARFDLQ**<br>**DGRRHKRYAHYDI**<br>**FWVDDEHHFFTLHI**<br>**ANYTGDAGDSMIS**<br>**EGRYNHNNQKFST**<br>**YDNDNDNSDDTSC**<br>**AEKYQGAWWYNA**<br>**CHRSNLNGLDYKG**<br>**KYRGVADGRHWA**<br>**TWKQEEEENEKEEK**<br>**DKDKKDKDKKDK**<br>**DKKD**	GCTGATGAGACGGTTCTTTATAAGAAGCGTTACGTAAAAATG<br>CATCTTCCTACTCGAATGTATTTGGAATTATTTTCCTCACAAA<br>ATTCGACACAAGAAGTTTAGTTCATTTCAGAATCGCGAATCG<br>TCTTGCTTCCGCAATTCCAAAATGTTCCAGTACGCTATCCTAC<br>TGTTGTCATTCGTAAGCATGTGTAAGACCTCAAGTCATACGT<br>CATGCTGTGAGCAGGAGGTCCGCACTAAATCGTTATTGGATA<br>CAGCGATTGACTTAATTTCAAACGCAAAGTCAAATATTCCTA<br>GTTGTTCTGACACGCAGGCCAGTCAAGCAGCATGTGTGAAG<br>GAAAGCTTAGATTGCGCTGATTTGTTCAGAAATGGTTACAAT<br>AAGAGTGGTATATACAACATCACGCTCGTGAACAGGCTATTC<br>TACAAGAAACGCCTTGAAGTCTATTGTGATATGGAAACAGAT<br>GGAGGTGGATGGACTGTAATTCAAAGGAGGGGAGATTTTCA<br>GAGGGAGAAACATTACTTTTATAGAAACTGGACTGAATATA<br>AAGAAGGATTTGGAGTTAAGGAAGAAGATTTTTGGTTAGGTC<br>TCCTCTACATATATGCTATAACAAATCAGAAAGAGTATTCAG<br>CAAGATTTGATTTGCAAGATGGAAGGCGCCATAAAAGATAT<br>GCACATTACGACATCTTTTGGGTTGACGATGAACACCATTTCT<br>TCACTCTGCACATTGCAAACTACACAGGAGATGCAGGAGAT<br>TCTATGATCTCAGAAGGTCGTTACAATCACAATAATCAAAAA<br>TTTTCTACCTACGACAATGATAACGACAATTCAGATGACACT<br>TCGTGTGCTGAGAAGTATCAAGGAGCGTGGTGGTACAATGC<br>ATGCCACAGATCCAATCTGAATGGGCTAGACTACAAAGGAA<br>AGTACAGAGGTGTAGCGGATGGAAGACACTGGGCAACCTGG<br>AAACAGGAAGAGGAAGAAAATGAAAAGGAGGAAAAGGAT<br>AAGGACAAAAAGGATAAGGACAAAAAGGATAAGGACAAA<br>AAGGAT

PhdFibTec36	P85031	1,10E-54	38,710	PF00147	36081.85<br>**33930.23**	MFQYAILLLSFVSMCKTSS**HTSCCEQEVRTKSLLDTAIDLISNAKSNIPSCSDTQASQAACVKESLDCADLFRNGYNKSGIYNITLVNRLFYKKRLEVYCDMETDGGGWTVIQRRGDFQREKHYFYRNWTEYKEGFGVKEEDFWLGLLYIYAITNQKEYSARFDLQDGRRHKRYAHYDIFWVDDEHHFFTLHIANYTGDAGDSMISEGRYNHNNQKFSTYDNDNDNSNDTSCAEQYKGAWWYNACHRSHLNGLDYKGKYRGVANGMHWATWEQEKNGKEVESWQVADRESLEATEIKVRPRNFKDTDD***	GCTGATGAGACGGTTCTTTATAAGAAGCGTTACGTAAAAATGCATCTTCCTACTCGAATGTATTTGGAATTATTTTCCTCACAAAATTCGACACAAGAAGTTTAGTTCATTTCAGAATCGCGAATCGTCTTGCTTCCGCAATTCCAAAATGTTCCAGTACGCTATCCTACTGTTGTCATTCGTAAGCATGTGTAAGACCTCAAGTCATACGTCATGCTGTGAGCAGGAGGTCCGCACTAAATCGTTATTGGATACAGCGATTGACTTAATTTCAAACGCAAAGTCAAATATTCCTAGTTGTTCTGACACGCAGGCCAGTCAAGCAGCATGTGTGAAGGAAAGCTTAGATTGCGCTGATTTGTTCAGAAATGGTTACAATAAGAGTGGTATATACAACATCACGCTCGTGAACAGGCTATTCTACAAGAAACGCCTTGAAGTCTATTGTGATATGGAAACAGATGGAGGTGGATGGACTGTAATTCAAAGGAGGGGAGATTTTCAGAGGGAGAAACATTACTTTTATAGAAACTGGACTGAATATAAAGAAGGATTTGGAGTTAAGGAAGAAGATTTTTGGTTAGGTCTCCTCTACATATATGCTATAACAAATCAGAAAGAGTATTCAGCAAGATTTGATTTGCAAGATGGAAGGCGCCATAAAAGATATGCACATTACGACATCTTTTGGGTTGACGATGAACACCATTTCTTCACTCTGCACATTGCAAACTACACAGGAGATGCAGGAGATTCTATGATCTCAGAAGGTCGTTACAATCACAATAATCAAAAATTTTCTACCTACGACAATGATAACGACAATTCAAATGACACTTCGTGTGCTGAGCAGTATAAAGGAGCGTGGTGGTACAATGCATGCCACAGATCCCACCTGAATGGGCTAGACTACAAAGGAAAGTACAGAGGTGTAGCAAATGGAATGCATTGGGCAACCTGGGAACAGGAAAAAAATGGTAAGGAAGTGGAAAGCTGGCAAGTTGCCGATCGTGAATCTTTGGAAGCTACAGAAATTAAAGTTAGACCGAGGAATTTCAAAGATACCGACGATTAAACAGATACTTTTATGTAATGCATATTCTTAGCGGATTATCGCAACAAATATATATATGTAAAGTATGTGTGTATAATCAAGATACAAGTGACTT

							TTAACTGAAATAAATGTAGATATACAGAATTATCCAATGATC AAATAAAATCTTCTATTTG

PhdFibTec3<br>7	P85031	8,21E-51	40,310	PF00147	35308,97	EALRKNASSYSNVF<br>GIIFLTKFDTRSLVHF<br>RIANRLASAIPKCSD<br>TQASQAACVKESLD<br>CADLFRNGYNKSGI<br>YNITLVNRLFYKKRL<br>EVYCDMETDGGGW<br>TVIQRRGDFQREKH<br>YFYRNWTEYKEGFG<br>VKEEDFWLGLLYIYA<br>ITNQKEYSARFDLQD<br>GRRHKRYAHYDIFW<br>VDDEHHFFTLHIAN<br>YTGDAGDSMISEGR<br>YNHNNQKFSTYDN<br>DNDNSNDTSCAEQY<br>KGAWWYNACHRS<br>HLNGLDYKGKYRG<br>VANGMHWATWEQ<br>EKNGKEVESWQVA<br>DRESLEATEIKVRPR<br>NFKDTDD*	GCTGATGAGACGGTTCTTTATAAGAAGCGTTACGTAAAAATG<br>CATCTTCCTACTCGAATGTATTTGGAATTATTTTCCTCACAAA<br>ATTCGACACAAGAAGTTTAGTTCATTTCAGAATCGCGAATCG<br>TCTTGCTTCCGCAATTCCAAAATGTTCTGACACGCAGGCCAG<br>TCAAGCAGCATGTGTGAAGGAAAGCTTAGATTGCGCTGATTT<br>GTTCAGAAATGGTTACAATAAGAGTGGTATATACAACATCAC<br>GCTCGTGAACAGGCTATTCTACAAGAAACGCCTTGAAGTCTA<br>TTGTGATATGGAAACAGATGGAGGTGGATGGACTGTAATTCA<br>AAGGAGGGGAGATTTTCAGAGGGAGAAACATTACTTTTATA<br>GAAACTGGACTGAATATAAAGAAGGATTTGGAGTTAAGGAA<br>GAAGATTTTTGGTTAGGTCTCCTCTACATATATGCTATAACAA<br>ATCAGAAAGAGTATTCAGCAAGATTTGATTTGCAAGATGGA<br>AGGCGCCATAAAAGATATGCACATTACGACATCTTTTGGGTT<br>GACGATGAACACCATTTCTTCACTCTGCACATTGCAAACTAC<br>ACAGGAGATGCAGGAGATTCTATGATCTCAGAAGGTCGTTA<br>CAATCACAATAATCAAAAATTTTCTACCTACGACAATGATAA<br>CGACAATTCAAATGACACTTCGTGTGCTGAGCAGTATAAAGG<br>AGCGTGGTGGTACAATGCATGCCACAGATCCCACCTGAATG<br>GGCTAGACTACAAAGGAAAGTACAGAGGTGTAGCAAATGG<br>AATGCATTGGGCAACCTGGGAACAGGAAAAAAATGGTAAG<br>GAAGTGGAAAGCTGGCAAGTTGCCGATCGTGAATCTTTGGA<br>AGCTACAGAAATTAAAGTTAGACCGAGGAATTTCAAAGATA<br>CCGACGATTAAACAGATACTTTTATGTAATGCATATTCTTAGC<br>GGATTATCGCAACAAATATATATATGTAAAGTATGTGTGTAT<br>AATCAAGATACAAGTGACTTTTAACTGAAATAAATGTAGATA<br>TACAGAATTATCCAATGATCAAATAAAATCTTCTATTTG

PhdFibTec38	P85031	2,54E-119	76,000	PF00147	26884,26	VCPSPPLPIDCEEVLQRGQSKSGVYKVLPRSRAIPNPIEVYCDMITDGGGWTVIQRRGNFLRPKDYFYQDWQAYKKGFGSIEKDFWLGNDNIFGLSNQRMCSVRFDLKDVEGSRRHALYDTFWIDDEDHKYMLHINGYKGDAGDSMIGVHNEQKFSTKDQRNDNFPGATSCAQLYKGGWWYNRCHVSNLNGQYLKGKHKSYADGVIWRSWKGYHESLGWTEIKIRSKDFRK*	TTTTAAGCATTTAAGTTATTTATCAACATAAAATTTCTTAATGATAACCGTTATTTAGGAAATAGCATTTGCCAATATACTTAGAGAAAGGTGTTTTCTCTGTCTCTTCTTGATCGGTGAATGGAATTATTTTCTGAAATCTTTACTGCGTATTTTTATTTCCGTCCATCCTAATGATTCATGGTAACCTTTCCAGGAACGCCAGATGACACCATCTGCATAGCTCTTATGTTTGCCTTTCAGGTATTGTCCATTTAAGTTCGAAACATGGCACCGATTATACCACCATCCACCTTTGTATAGTTGTGCGCAACTGGTAGCGCCTGGAAAATTGTCATTTCTCTGATCCTTTGTTGAAAATTTTTGTTCATTATGTACACCTATCATGGAATCACCTGCATCTCCCTTATAACCATTGATGTGCAGCATATACTTATGATCCTCGTCATCAATCCAAAACGTATCGTAGAGGGCGTGACGTCGACTGCCTTCAACATCCTTGAGGTCAAATCTTACTGAGCACATTCTCTGGTTACTCAGGCCAAAAATGTTGTCATTGCCTAACCAAAAATCTTTTTCAATGCTGCCAAATCCCTTCTTGTATGCTTGCCAGTCTTGGTAAAAGTAATCTTTTGGTCTAAGGAAATTACCCCTTCTTTGTATTACTGTCCATCCACCTCCGTCAGTGATCATATCACAGTAAACTTCGATCGGATTTGGTATTGCTCTGCTCCGTGGTAGAACTTTGTAGACACCACTCTTGCTCTGACCACGTTGTAATACTTCTTCACAGTCTATGGGAAGTGGTGGTGAAGGACAAAC

PhdFibTec39	P85031	4,12E-100	83,908	PF00147	21264,68	TVIQRRGDFHGPIDYFYKDWQSYKKGFGDNEKEFWLGNENIFGLSNQRMCSIRFDLKDVAGNQRYALYDTFRIDDEDHKYMLHINGYKGDAGDSMIGVHNEQKFSTKDQRNDNFPGATSCAQLYKGGWWYNRCHVSNLNGQYLKGKHKSYADGVIWRSWKGYHESLGWTEIKIRSKDFRK*	TTTTAAGCATTTAAGTTATTTATCAACATAAAATTTCTTAATGATAACCGTTATTTAGGAAATAGCATTTGCCAATATACTTAGAGAAAGGTGTTTTCTCTGTCTCTTCTTGATCGGTGAATGGAATTATTTTCTGAAATCTTTACTGCGTATTTTTATTTCCGTCCATCCTAATGATTCATGGTAACCTTTCCAGGAACGCCAGATGACACCATCTGCATAGCTCTTATGTTTGCCTTTCAGGTATTGTCCATTTAAGTTCGAAACATGGCACCGATTATACCACCATCCACCTTTGTATAGTTGTGCGCAACTGGTAGCGCCTGGAAAATTGTCATTTCTCTGATCCTTTGTTGAAAATTTTTGTTCATTATGTACACCTATCATGGAATCACCTGCATCTCCCTTATAACCATTGATGTGCAGCATATACTTATGATCCTCGTCATCAATACGAAACGTATCGTAGAGGGCGTAACGCTGATTGCCTGCCACATCCTTGAGGTCAAATCTTATTGAGCACATTCTCTGGTTACTAAGGCCAAAAATGTTTTCATTGCCTAGCCAAAATTCTTTTTCATTGTCGCCAAATCCTTTCTTGTACGATTGCCAGTCTTTGTAAAAGTAATCTATTGGTCCATGGAAATCACCCCTTCTTTGTATTACTGTCC
PhdFibTec40	P85031	2,21E-51	46,193	PF00147	30195.81<br><br>**27778.79**	MLMKIIFLLTFCGFLSYSVHA**CESQCVSLLLKNLTDKAQSSIVVTLIKNAQILLDDANKYATMQTKCPVAESICEKPMDCSEIMEKNPMASSGIYKVWPRSRVMTGSIEVYCDMEDAGGAWIVLQRRGDFGSGQDYFYRDWQSYKHGFGDLKKDFWLGNENIFA**	GGAAGTTATAACCGAAGATTTTGTCAGACACTTTCTCTTCACTACCATGCTCATGAAAATAATCTTTCTGTTAACTTTCTGCGGTTTCCTAAGTTACAGCGTTCATGCGTGTGAATCACAATGCGTTTCGCTTCTTTTGAAGAACTTGACTGATAAAGCACAATCTTCAATTGTAGTAACTCTTATAAAGAACGCTCAGATTCTTTTGGACGATGCAAACAAGTATGCAACTATGCAAACGAAATGCCCTGTTGCTGAATCAATATGCGAAAAACCGATGGACTGCTCTGAAATAATGGAGAAAAATCCCATGGCTAGTAGCGGCATATACAAGGTGTGGCCTCGCAGTCGGGTGATGACTGGTTCGATTGAAGTGTACTGCGACATGGAGGACGCTGGAGGAGCTTGGATAGTACTTCAAAGGCGAGGAGATTTTGGCAGCGGACAAGATTATTTTTATAGAGATTGGCAGTCTTATAAACATGGTTTCGGAGATCTGAAGAA

						**<u>LANQRANVVKFTLSDWEGNTTYATFDDFWIDDESENYKLHASRYSGTAGDSLSRHNGHAFSTNDKDNDSSKSVNCAASNKGAWWYTNCHDSNLNGLYFKDSRS</u>**	GGATTTTTGGCTAGGTAATGAGAACATCTTCGCGCTGGCCAATCAAAGGGCGAATGTTGTGAAATTCACCCTGAGTGACTGGGAAGGAAATACAACTTATGCTACGTTCGACGATTTCTGGATTGATGACGAATCTGAGAATTACAAACTGCATGCGTCGAGGTACAGCGGAACAGCTGGTGATTCTTTGTCTCGTCATAATGGACATGCATTTTCTACTAACGATAAAGATAATGACAGTAGCAAGTCCGTGAACTGTGCTGCTTCAAATAAAGGGGCGTGGTGGTATACAAATTGTCATGATTCAAATCTTAATGGATTGTATTTTAAAGACTCTCGCTCG

PhdFibTec41	P85031	9,70E-12	36,782	PF00147	14293.95<br>**11876.92**	MLMKIIFLLTFCGFLSYSVHA**CESQCVSLLLKNLTDKAQSSIVVTLIKNAQILLDDANKYATMQTKCPVAESICEKPMDCSEIMEKNPMASSGIYKVWPRSRVMTGSIEVYCDMEDAGGAWIIFLMTM***	GGAAGTTATAACCGAAGATTTTGTCAGACACTTTCTCTTCACTACCATGCTCATGAAAATAATCTTTCTGTTAACTTTCTGCGGTTTCCTAAGTTACAGCGTTCATGCGTGTGAATCACAATGCGTTTCGCTTCTTTTGAAGAACTTGACTGATAAAGCACAATCTTCAATTGTAGTAACTCTTATAAAGAACGCTCAGATTCTTTTGGACGATGCAAACAAGTATGCAACTATGCAAACGAAATGCCCTGTTGCTGAATCAATATGCGAAAAACCGATGGACTGCTCTGAAATAATGGAGAAAAATCCCATGGCTAGTAGCGGCATATACAAGGTGTGGCCTCGCAGTCGGGTGATGACTGGTTCGATTGAAGTGTACTGCGACATGGAGGACGCTGGAGGAGCTTGGATAATTTTTCTAATGACGATGTAGAAGTTTTCACATCGAAATTAAGAGATACTGCTGAAATTATTAAAGACGCCAAAACAAGTATTGAAGATGAAGTGGTGAGCGAAAGTGCTTGTGATGAAGTTGCAACCATGAGTTAATTATCACCATGAATCAAACAAGATATGAATCTATACCTGGTACTTCAAAGGCGAGGAGATTTTGGCAGCGGACAAGATTATTTTTATAGAGATTGGCAGTCTTATAAACATGGTTTCGGAGATCTGAAGAAGGATTTTTGGCTAGGTAAATTAAAATAAAATTAGGCATATTTGATGACTTTACAATTGTGAAATCGCAAAGCTCTTTCTCACGAGAACAGAACTTAAAAATTCTAATGTGTTCTGCCAGCATCGCACGCACATCAAAGGTTCCTCATTATCACCGATCGCAGAGCTGATCTTCCCAGGAATTACAGGAACTTAAAACTTCGCTGATGTTGAACACATGTTTGGAAAAGGTGTATCGGCGGTTTCTCACGATTTTTCAAAAGGCCATGCTTGGCGGCCCGAAATCGACGCAGAATAGCACATGGCGAGTTTTCCCAGAGGGAGAATGTATCGGTCGTTCCCCCCCCCCCTCTCCTGCGAACACAGGAGGACGTGGGCCGAACGCTGTGAGAAAAAGGTTATTTTCCGAGTTCCGTCCGAAATTACTGTAATGAGCGTGGGACCCCACGTTAATGAGGGATGCGAGGTGATGTCGCATCTTGGCGAACAAACGTTCGT

							CAGTCATTAACGAGATTTGCTAGGAGTTAGTTGACTCGAGAT<br>GGAGAGCTGAATGATTTCTTGGCCGATATTTCGCCGAGACCT<br>TCTGTATAACACTCGGGCTGATAGTGAAATCAGCGGGACTCG<br>CCTTGCAGTCCGATTTTGCCGCCCCGGCAGTAAAGTTTTTCTT<br>CGTTCGACACTGAATTGTGCCGACGTTCAGAGCAGAATTTCC<br>CTCTGTAAAGCTCCAGCTGAACTAATTTCAATAGTGGGAGTT<br>ATACTCCGTGAACTTAGCCGAAGCACTAGAGATTGCCGTGCT<br>CGAGACCTGCAGCGAAACTGTGTGACGACGCCTAAATCCCT<br>GAGGAGCGAGATATCCCGATGACCTATCTAAAGAATCCTCG<br>AATCCATGACTGAACTCTCGATGACTGAATTATACGGAAGAT<br>GAAACGGACAATAACCCTGAAATGAATTGGAATCTTGTAAA<br>TTAAATATATTAATCAAGCGAACTATGCAAACTAGTTCAAAT<br>TAATAAAATCTATTTCTCTGATCGAATAGCTGGTAATGTTCAC<br>TACGTCACAGGTGCTTACCTCACAGCCTGATTACTGCACCTCT<br>AATGAACGCTGGATTATTGATTCTGGTTGCGACAGACATATG<br>ACATCATGCTCTGAAATGTCTGCAACTTACACTGAATTACCA<br>CCGTGTACTGTAAAAACAGGAGGTACAAAGCTTCATAGTGTT<br>GGCGAAGGGTCTATAATTTTTAACATTGAACGGTAAAATAAA<br>ATTGTTGAGACCACT

PhdFibTec42	P85031	9,33E-49	42,523	PF00147	20043,86	MNLYLVLQRRGDFGSGQDYFYRDWQSYKHGFGDLKKDFWLGNENIFALANQRANVVKFTLSDWEGNTTYATFDDFWIDDESEKYKLYATGYSGTAGDSLSRHNGKAFSTKDQDNDSSKDRNCAAAYKGGWWYADCHDSNLNGLYFKDSRSSKFDGIEWKGYKGWNYSLKDTVI	GGAAGTTATAACCGAAGATTTTGTCAGACACTTTCTCTTCACTACCATGCTCATGAAAATAATCTTTCTGTTAACTTTCTGCGGTTTCCTAAGTTACAGCGTTCATGCGTGTGAATCACAATGCGTTTCGCTTCTTTTGAAGAACTTGACTGATAAAGCACAATCTTCAATTGTAGTAACTCTTATAAAGAACGCTCAGATTCTTTTGGACGATGCAAACAAGTATGCAACTATGCAAACGAAATGCCCTGTTGCTGAATCAATATGCGAAAAACCGATGGACTGCTCTGAAATAATGGAGAAAAATCCCATGGCTAGTAGCGGCATATACAAGGTGTGGCCTCGCAGTCGGGTGATGACTGGTTCGATTGAAGTGTACTGCGACATGGAGGACGCTGGAGGAGCTTGGATAATTTTTCTAATGACGATGTAGAAGTTTTCACATCGAAATTAAGAGATACTGCTGAAATTATTAAAGACGCCAAAACAAGTATTGAAGATGAAGTGGTGAGCGAAAGTGCTTGTGATGAAGTTGCAACCATGAGTTAATTATCACCATGAATCAAACAAGATATGAATCTATACCTGGTACTTCAAAGGCGAGGAGATTTTGGCAGCGGACAAGATTATTTTTATAGAGATTGGCAGTCTTATAAACATGGTTTCGGAGATCTGAAGAAGGATTTTTGGCTAGGTAATGAGAACATCTTCGCGCTGGCCAATCAAAGGGCGAATGTTGTGAAATTCACCCTGAGTGACTGGGAAGGAAATACAACTTATGCTACGTTCGACGATTTCTGGATTGATGACGAATCTGAGAAATACAAACTGTATGCGACGGGATACAGCGGAACAGCTGGTGATTCTTTGTCTCGTCATAATGGAAAGGCATTTTCTACTAAAGATCAAGACAATGACAGTAGCAAGGACCGGAACTGTGCTGCTGCTTATAAAGGGGGGTGGTGGTACGCAGATTGTCATGATTCCAATCTTAATGGATTGTATTTTAAAGACTCTCGCTCGTCTAAATTCGACGGAATCGAATGGAAAGGATATAAAGGCTGGAATTATTCTCTGAAAGATACTGTGATA

PhdFibTec43	P85031	8,69E-60	45,982	PF00147	32948.02<br>**30530.99**	MLMKIIFLLTFCGFLSYSVHA**CESQCVSLLLKNLTDKAQSSIVVTLIKNAQILLDDANKYATMQTKCPVAESICEKPMDCSEIMEKNPMASSGIYKVWPRSRVMTGSIEVYCDMEDAGGAWIVLQRRGDFGSGQDYFYRDWQSYKHGFGDLKKDFWLGNENIFALANQRANVVKFTLSDWEGNTTYATFDDFWIDDESEKYKLYATGYSGTAGDSLSRHNGKAFSTKDQDNDSSKDRNCAAAYKGGWWYADCHDSNLNGLYFKDSRSSKFDGIEWKGYKGWNYSLKDTVI**	GGAAGTTATAACCGAAGATTTTGTCAGACACTTTCTCTTCACTACCATGCTCATGAAAATAATCTTTCTGTTAACTTTCTGCGGTTTCCTAAGTTACAGCGTTCATGCGTGTGAATCACAATGCGTTTCGCTTCTTTTGAAGAACTTGACTGATAAAGCACAATCTTCAATTGTAGTAACTCTTATAAAGAACGCTCAGATTCTTTTGGACGATGCAAACAAGTATGCAACTATGCAAACGAAATGCCCTGTTGCTGAATCAATATGCGAAAAACCGATGGACTGCTCTGAAATAATGGAGAAAAATCCCATGGCTAGTAGCGGCATATACAAGGTGTGGCCTCGCAGTCGGGTGATGACTGGTTCGATTGAAGTGTACTGCGACATGGAGGACGCTGGAGGAGCTTGGATAGTACTTCAAAGGCGAGGAGATTTTGGCAGCGGACAAGATTATTTTTATAGAGATTGGCAGTCTTATAAACATGGTTTCGGAGATCTGAAGAAGGATTTTTGGCTAGGTAATGAGAACATCTTCGCGCTGGCCAATCAAAGGGCGAATGTTGTGAAATTCACCCTGAGTGACTGGGAAGGAAATACAACTTATGCTACGTTCGACGATTTCTGGATTGATGACGAATCTGAGAAATACAAACTGTATGCGACGGGATACAGCGGAACAGCTGGTGATTCTTTGTCTCGTCATAATGGAAAGGCATTTTCTACTAAAGATCAAGACAATGACAGTAGCAAGGACCGGAACTGTGCTGCTGCTTATAAAGGGGGGTGGTGGTACGCAGATTGTCATGATTCCAATCTTAATGGATTGTATTTTAAAGACTCTCGCTCGTCTAAATTCGACGGAATCGAATGGAAAGGATATAAAGGCTGGAATTATTCTCTGAAAGATACTGTGATA

PhdFibTec44	P85031	5,43E-21	46,988	PF00147	18282.27<br>**15865.24**	MLMKIIFLLTFCGFLS<br>YSVHA**CESQCVSLL**<br>**LKNLTDKAQSSIVV**<br>**TLIKNAQILLDDAN**<br>**KYATMQTKCPVAE**<br>**SICEKPMDCSEIME**<br>**KNPMASSGIYKVW**<br>**PRSRVMTGSIEVYC**<br>**DMEDAGGAWIVL**<br>**QRRGDFGSGQDYF**<br>**YRDWQSYKHGFGD**<br>**LKKDFWLGKLK***	GGAAGTTATAACCGAAGATTTTGTCAGACACTTTCTCTTCACT<br>ACCATGCTCATGAAAATAATCTTTCTGTTAACTTTCTGCGGTT<br>TCCTAAGTTACAGCGTTCATGCGTGTGAATCACAATGCGTTTC<br>GCTTCTTTTGAAGAACTTGACTGATAAAGCACAATCTTCAATT<br>GTAGTAACTCTTATAAAGAACGCTCAGATTCTTTTGGACGAT<br>GCAAACAAGTATGCAACTATGCAAACGAAATGCCCTGTTGC<br>TGAATCAATATGCGAAAAACCGATGGACTGCTCTGAAATAA<br>TGGAGAAAAATCCCATGGCTAGTAGCGGCATATACAAGGTG<br>TGGCCTCGCAGTCGGGTGATGACTGGTTCGATTGAAGTGTAC<br>TGCGACATGGAGGACGCTGGAGGAGCTTGGATAGTACTTCA<br>AAGGCGAGGAGATTTTGGCAGCGGACAAGATTATTTTTATAG<br>AGATTGGCAGTCTTATAAACATGGTTTCGGAGATCTGAAGAA<br>GGATTTTTGGCTAGGTAAATTAAAATAAAATTAGGCATATTT<br>GATGACTTTACAATTGTGAAATCGCAAAGCTCTTTCTCACGA<br>GAACAGAACTTAAAAATTCTAATGTGTTCTGCCAGCATCGCA<br>CGCACATCAAAGGTTCCTCATTATCACCGATCGCAGAGCTGA<br>TCTTCCCAGGAATTACAGGAACTTAAAACTTCGCTGATGTTG<br>AACACATGTTTGGAAAAGGTGTATCGGCGGTTTCTCACGATT<br>TTTCAAAAGGCCATGCTTGGCGGCCCGAAATCGACGCAGAA<br>TAGCACATGGCGAGTTTTCCCAGAGGGAGAATGTATCGGTCG<br>TTCCCCCCCCCCCTCTCCTGCGAACACAGGAGGACGTGGGCC<br>GAACGCTGTGAGAAAAAGGTTATTTTCCGAGTTCCGTCCGAA<br>ATTACTGTAATGAGCGTGGGACCCCACGTTAATGAGGGATGC<br>GAGGTGATGTCGCATCTTGGCGAACAAACGTTCGTCAGTCAT<br>TAACGAGATTTGCTAGGAGTTAGTTGACTCGAGATGGAGAGC<br>TGAATGATTTCTTGGCCGATATTTCGCCGAGACCTTCTGTATA<br>ACACTCGGGCTGATAGTGAAATCAGCGGGACTCGCCTTGCA<br>GTCCGATTTTGCCGCCCCGGCAGTAAAGTTTTTCTTCGTTCGA

							CACTGAATTGTGCCGACGTTCAGAGCAGAATTTCCCTCTGTAAAGCTCCAGCTGAACTAATTTCAATAGTGGGAGTTATACTCCGTGAACTTAGCCGAAGCACTAGAGATTGCCGTGCTCGAGACCTGCAGCGAAACTGTGTGACGACGCCTAAATCCCTGAGGAGCGAGATATCCCGATGACCTATCTAAAGAATCCTCGAATCCATGACTGAACTCTCGATGACTGAATTATACGGAAGATGAAACGGACAATAACCCTGAAATGAATTGGAATCTTGTAAATTAAATATATTAATCAAGCGAACTATGCAAACTAGTTCAAATTAATAAAATCTATTTCTCTGATCGAATAGCTGGTAATGTTCACTACGTCACAGGTGCTTACCTCACAGCCTGATTACTGCACCTCTAATGAACGCTGGATTATTGATTCTGGTTGCGACAGACATATGACATCATGCTCTGAAATGTCTGCAACTTACACTGAATTACCACCGTGTACTGTAAAAACAGGAGGTACAAAGCTTCATAGTGTTGGCGAAGGGTCTATAATTTTTAACATTGAACGGTAAAATAAAATTGTTGAGACCACT

PhdFibTec45	P85031	3,40E-31	53,763	PF00147	21297.17<br><br>**18871.28**	MFSNVLIYLIFWSCVSYSISA**GETECASLLLKNITDQTQNSIVVTLLNNAKNLLEDAKKYVTTKIRCSVTESKCEKPMDCAEIMEKIPFASSGVYTIWPRSRVITGPLEVYCDMKDDGGAWTVLQRRGDFGNPNDNFYKGWQSYKEGFGDKTKDFWLGNDNIFALTNQRPNVVKFSLSDWEKNETYA**	CGTGACCGACGTTTTATCTCTGTCTCCTGCCATGTTTTCGAACGTTTTAATATATTTAATTTTCTGGAGTTGTGTGAGTTACAGCATTTCTGCTGGTGAAACAGAATGTGCTTCGCTGTTGTTAAAGAACATAACCGACCAGACTCAAAATTCTATTGTAGTGACTCTCTTAAATAATGCTAAAAATCTTCTCGAGGATGCAAAGAAATATGTGACCACAAAAATCAGATGTTCTGTTACTGAATCAAAGTGCGAAAAACCAATGGACTGCGCTGAAATAATGGAGAAAATCCCCTTTGCCAGTAGTGGTGTATACACAATCTGGCCTCGCAGCCGGGTGATTACTGGTCCATTAGAAGTTTACTGTGACATGAAGGACGACGGAGGTGCTTGGACAGTACTGCAGAGGAGAGGAGATTTCGGCAACCCGAACGATAATTTTTATAAAGGCTGGCAATCCTATAAGGAAGGATTCGGAGATAAAACGAAAGATTTTTGGCTAGGTAATGACAACATCTTTGCTCTCACGAACCAACGGCCGAATGTCGTAAAATTTTCTTTGAGTGATTGGGAAAAAAATGAAACGTATGCAA
PhdFibTec46	P85031	9,50E-35	55,914	PF00147	19329.87<br><br>**16903.98**	MFSNVLIYLIFWSCVSYSISA**GETECASLLLKNITDQTKSSVVLTLLNSAENLLEDAKRYVAIKSRCSVTESICEKPMDCAEIMEKNPFANSGVYKIWPRSRVITNPIDVYCDMEDDGGAWTVLQRRGDFDSGKDYFYKDWKSYKEGFGDIERDFWLGNDNIFSVTNQK**	CGTGACCGACGTTTTATCTCTGTCTCCTGCCATGTTTTCGAACGTTTTAATATATTTAATTTTCTGGAGTTGTGTGAGTTACAGCATTTCTGCTGGTGAAACAGAATGTGCTTCGCTGTTGTTAAAGAACATAACCGACCAAACGAAATCTTCTGTTGTATTGACTCTCTTAAATAGTGCTGAAAACCTTCTGGAGGATGCAAAGAGATATGTAGCCATAAAATCCAGATGCTCTGTTACTGAATCAATATGTGAAAAACCAATGGACTGTGCTGAAATAATGGAGAAAAATCCCTTTGCAAACAGTGGTGTGTACAAAATCTGGCCTCGCAGTCGTGTGATTACTAATCCGATCGATGTCTACTGTGACATGGAGGATGACGGAGGAGCTTGGACGGTACTGCAGAGACGAGGAGATTTCGATAGTGGGAAAGATTATTTTTATAAAGACTGGAAAAGCTACAAAGAAGGATTTGGAGACATAGAAAGAGATTTTTGGTTGGGAAATGATAATATATTTTCTGTGACCAACCAGAAA

PhdFibTec4<br>7	P85031	2,03E-32	69,118	PF00147	9727,75	NPFANSGVYKIWPR<br>SRVVPCPIDVYCDM<br>EDDGGAWTVLQRR<br>GDFDSGKDYFYKDW<br>KSYKEGFGDIERDFW<br>LGNDNIFSVTNQK	AATCCCTTTGCAAACAGTGGTGTATACAAAATCTGGCCTCGC<br>AGTCGGGTGGTTCCTTGTCCTATCGATGTCTATTGTGACATGG<br>AGGATGACGGAGGAGCTTGGACGGTATTGCAGAGACGAGGA<br>GATTTCGATAGTGGGAAAGATTATTTTATAAAGACTGGAAA<br>AGCTACAAAGAAGGATTTGGAGACATAGAAAGAGATTTTTG<br>GTTGGGAAATGATAATATATTTCTGTGACCAACCAGAAA
PhdFibTec4<br>8	P85031	1,70E-64	49,541	PF00147	35398.62<br><br>**32972.74**	MFSNVLIYLIFWSCV<br>SYSISA**GETECASLLL**<br>**KNITDQTKSSVVLT**<br>**LLNSAENLLEDAKR**<br>**YVAIKSRCSVTESIC**<br>**EKPMDCAEIMEKN**<br>**PFANSGVYKIWPRS**<br>**RVITNPIDVYCDME**<br>**DDGGAWTVLQRR**<br>**GDFDSGKDYFYKD**<br>**WKSYKEGFGDKKK**<br>**DFWLGNDNIFAVT**<br>**NQRSNVVKIFLRD**<br>**WEKNETYATFDEF**<br>**WIEDETRKYTMHV**<br>**SGYRGTAGDSFSVT**<br>**NGLPFTTKDQDND**<br>**KHDKNCAVLFKGA**<br>**WWYEDCHSSNLNG**<br>**MYLKGVHETHADG**<br>**VEWHSFRGHYYSL**	CGTGACCGACGTTTTATCTCTGTCTCCTGCCATGTTTTCGAAC<br>GTTTTAATATATTTAATTTTCTGGAGTTGTGTGAGTTACAGCA<br>TTTCTGCTGGTGAAACAGAATGTGCTTCGCTGTTGTTAAAGA<br>ACATAACCGACCAAACGAAATCTTCTGTTGTATTGACTCTCTT<br>AAATAGTGCTGAAAACCTTCTGGAGGATGCAAAGAGATATG<br>TAGCCATAAAATCCAGATGCTCTGTTACTGAATCAATATGTG<br>AAAAACCAATGGACTGTGCTGAAATAATGGAGAAAAATCCC<br>TTTGCAAACAGTGGTGTGTACAAAATCTGGCCTCGCAGTCGT<br>GTGATTACTAATCCGATCGATGTCTACTGTGACATGGAGGAT<br>GACGGAGGAGCTTGGACGGTACTGCAGAGACGAGGAGATTT<br>CGATAGTGGGAAAGATTATTTTATAAAGACTGGAAATCTTA<br>TAAAGAAGGATTCGGCGATAAAAAGAAAGATTTTTGGCTGG<br>GTAATGACAACATTTTTGCTGTTACGAACCAACGTTCGAATG<br>TCGTAAAAATTTTTTTGAGAGACTGGGAAAAAAATGAAACTT<br>ATGCAACTTTTGACGAATTCTGGATTGAAGACGAAACTCGTA<br>AATACACCATGCATGTGAGTGGATACAGAGGAACAGCTGGT<br>GATTCTTTTTCTGTTACTAATGGATTACCATTTACCACCAAGG<br>ATCAAGATAATGACAAGCATGATAAGAACTGTGCTGTTCTGT<br>TTAAAGGGGCTTGGTGGTATGAAGATTGCCATTCTTCGAATC<br>TTAATGGAATGTACTTGAAGGGCGTGCATGAAACTCATGCCG<br>ATGGTGTTGAATGGCATTCCTTCAGAGGTCACTACTATTCTCT<br>GAAAGATACTGTGATAAAAATTCGCCCTAGAGATTTCCAGA

						**KDTVIKIRPRDFQK NSDDITPH***	AGAATTCAGATGATATTACACCACATTAACCACTTTCCATTC GTTGTATCTTGATTGATAACCGACATTTATTTCCAGAGATTGA TCAGAACAATGGTTTCAGTCATTTTAAGAAATTAACAATGAT TTCCAAGTTTACACATGGATTCCTAACTGCAATTAGCAATTTC CATCAAACTAAATAATTATAGTATTGTAATAATTATAAGAGT TGAATAATGAATTCTATATATATAATGTTTGTGTTTTTATATGT ATATATTTTATGTGTG
PhdFibTec4 9	P85031	9,49E-27	53,488	PF00147	18208.66 **15775.70**	MFTKVFILLTFCGFLS YSVYA**NESQCASLL LNNLTDHTQGSVV VALIKNAQTLLDGA SKYAKIQARCTAIES ACDKPIDCAEVLEK NPLATSGVYKVWL RSRMITGHTNVYC DMEDDGGAWTVI QRRGDFDSGQEYF YKDWNAYKHGFG DLMKDFWLGNDNI**	GGAATTTCTATCTGAAGATTCAGTCTCTTTCTCTCTTTCAACTA CTACCATGTTCACGAAAGTGTTTATTCTTTTAACATTCTGCGG TTTCCTAAGTTACAGTGTTTATGCAAATGAATCACAATGCGCT TCGCTTCTGTTGAATAACTTGACTGATCACACCCAAGGTTCTG TTGTAGTTGCTCTCATAAAAAACGCCCAAACTCTTCTGGATG GCGCAAGCAAATATGCAAAGATTCAAGCGAGATGTACTGCA ATTGAATCAGCATGTGATAAACCGATAGACTGCGCTGAAGT ATTGGAGAAGAACCCCTTAGCAACTAGTGGAGTGTACAAGG TCTGGCTTCGCAGTCGGATGATAACTGGTCATACCAATGTTT ACTGCGATATGGAAGACGACGGAGGAGCTTGGACAGTAATT CAGAGGCGAGGAGATTTTGACAGTGGACAAGAATATTTTTAT

							AAAGATTGGAACGCTTATAAACATGGTTTCGGAGATCTAATG AAGGATTTCTGGCTAGGTAATGACAATATA
PhdFibTec50	P85031	2,19E-26	52,326	PF00147	18471.05<br>**16064.12**	MFTKVFILLTFCGFLSYSVHA**NESQCASLLLNNLTDQTQGSVVVALIKNAQILLDGANKYAKIQTSFRCAVADSVCDKPMDCSEVMAKNPLATSGVYKVWLRSRMITGHTNVYCDMEDDGGAWTVIQRRGDFDSGQEYFYKDWKSYKHGFGDLMKDFWLGNDNI**	TCTTTCAACTACTACCATGTTCACGAAAGTGTTTATTCTTTTAACATTCTGCGGTTTCCTAAGTTACAGTGTTCATGCAAATGAATCGCAATGCGCTTCGCTTCTGTTGAATAACTTGACTGACCAGACGCAAGGTTCTGTTGTAGTTGCTCTCATAAAAAATGCCCAAATCCTTCTGGATGGCGCAAACAAATATGCAAAGATTCAAACGAGTTTCAGATGTGCCGTGGCTGACTCAGTATGTGATAAACCAATGGATTGCTCTGAAGTAATGGCGAAGAACCCCTTAGCAACTAGTGGTGTGTACAAAGTCTGGCTTCGCAGTCGGATGATAACTGGTCACACCAATGTCTACTGCGACATGGAGGACGACGGAGGAGCTTGGACAGTAATTCAGAGGCGAGGAGATTTTGACAGTGGACAAGAATATTTTTATAAAGATTGGAAGTCTTATAAACATGGTTTCGGAGATCTAATGAAGGATTTCTGGCTAGGTAATGACAATATA
PhdFibTec51	P85031	6,94E-18	49,315	PF00147	16696.13<br>**14263.16**	MFTKVFILLTFCGFLSYSVYA**NESQCASILLNNLTDQAQGSVVVALIKNAQILLDGANKYAKIQTRCPVADSVCDKPMDCSEVMAKNPLATSGVYKVWLRSRMITGHTKVYC**	GGAATTTCTATCTGAAGATTCAGTCTCTTTCTCTCTTTCAACTACTACCATGTTCACGAAAGTGTTTATTCTTTTAACATTCTGCGGTTTCCTAAGTTACAGTGTTTATGCAAATGAATCACAATGCGCTTCGATTCTGTTGAATAACTTGACTGACCAAGCGCAAGGTTCTGTTGTAGTTGCTCTCATAAAAAATGCCCAAATCCTTCTGGATGGCGCAAACAAATATGCAAAGATTCAAACGAGATGCCCCGTGGCTGACTCAGTATGTGATAAACCGATGGACTGCTCTGAAGTTATGGCGAAGAACCCCTTAGCAACTAGTGGTGTGTACAAAGT

						**DMDDDGGAWTVIQRRGDFDSGQEYFYKDWKSYKHGFG**	CTGGCTTCGCAGTCGGATGATAACTGGTCATACCAAAGTCTACTGCGACATGGACGACGACGGGGGAGCTTGGACAGTAATTCAGAGGCGAGGAGATTTTGACAGTGGACAAGAATATTTTTATAAAGATTGGAAATCTTATAAACATGGTTTCGGAGA
PhdFibTec52	P85031	6,72E-27	48,958	PF00147	21564.61<br>**19157.68**	MFTKVFILLTFCGFLSYSVHA**NESQCASLLLNNLTDQTQGSVVVALIKNAQILLDGANKYAKIQTSFRCPVADSVCDKPMDCSEVMAKNPLATSGVYKVWLRSRMITGYTNVYCDMEDDGGAWTVIQRRGDFDSGHEYFHKDWKDYKHGFGDQMKDFWLGNDKIFALTNQRASVVKFSMVDWEWTRIFS***	TCTTTCAACTACTACCATGTTCACGAAAGTGTTTATTCTTTTAACATTCTGCGGTTTCCTAAGTTACAGTGTTCATGCAAATGAATCGCAATGCGCTTCGCTTCTGTTGAATAACTTGACTGACCAGACGCAAGGTTCTGTTGTAGTTGCTCTCATAAAAAATGCCCAAATCCTTCTGGATGGCGCAAACAAATATGCAAAGATTCAAACGAGTTTCAGATGTCCCGTGGCTGACTCAGTATGTGATAAACCAATGGATTGCTCTGAAGTAATGGCGAAGAACCCCTTAGCAACTAGTGGTGTGTACAAAGTCTGGCTTCGCAGTCGGATGATAACTGGTTATACCAATGTCTACTGCGACATGGAGGACGACGGAGGAGCTTGGACAGTAATTCAGAGGCGAGGAGATTTTGACAGTGGACACGAATATTTTCATAAAGATTGGAAGGATTATAAACATGGATTTGGAGATCAAATGAAGGATTTTTGGCTAGGTAATGATAAAATATTTGCTCTAACAAACCAAAGGGCGTCCGTTGTGAAATTTTCGATGGTTGACTGGGAGTGGACACGAATATTTTCATAAAGATTGGAAGGATTATAAACATGGATTTGGAGATCAAATGAAGGATTTTTGGCTAGGTA

PhdFibTec53	P85031	2,29E-26	52,326	PF00147	18413.05<br>**16005.06**	MFIKVFILLTFFGFLSNAVFA**NESQCASLLLNNLTDQTQGSVVVALIKNAQILLDGANKYAKIQASFRCSVADSVCDKPMDCSEILAKNPLATSGVYKVWLRSRVISGQTKVYCDMEDDGGAWTVIQRRGDFDSGQEYFYKDWKSYKHGFGDLMKDFWLGNDNI**	GGAATTTCTATCTGAAGATTCAGTCTCTTTCTCTCTTTCAACTACTACCATGTTCATTAAAGTGTTTATTCTTTTAACGTTTTTCGGTTTTCTAAGTAACGCTGTTTTTGCAAATGAATCGCAATGCGCTTCGCTTCTGTTGAATAACTTGACCGACCAAACGCAAGGTTCTGTTGTAGTTGCTCTCATAAAAAATGCTCAAATTCTTCTGGATGGCGCAAACAAATATGCAAAGATTCAAGCGAGTTTCAGATGTTCCGTGGCTGACTCAGTATGTGATAAACCGATGGACTGCTCTGAAATATTGGCGAAGAACCCCTTAGCAACTAGTGGTGTGTACAAAGTCTGGCTTCGCAGTCGAGTGATAAGTGGTCAAACCAAAGTCTACTGCGACATGGAGGACGACGGAGGAGCTTGGACAGTAATTCAGAGGCGAGGAGATTTTGACAGTGGACAAGAATATTTTTATAAAGATTGGAAGTCTTATAAACATGGTTTCGGAGATCTAATGAAGGATTTCTGGCTAGGTAATGACAATATA

PhdFibTec54	P85031	1,83E-52	43,578	PF00147	34979.15<br>**32815.47**	MFGKPLILTFCGILSCGFYA**SQSQCALPLELNLTDQIQKSLAIVLIKTAHILLDDANKYDTLQAGCPIAESIYEKPMDCADIMEKNPSATNGVYKIWPRNAMRNCPIDVYCDMEDDGGAWTVLQRRGAFGNRHDYFYRGWQSYKHGFGDLKKDFWLGNDNIFLLTNQRANVAKFTLTDWEGNTTYAIFDDFWIEDEKQKYRLHVTGYSGTAGDSVFRNDGQKFSSKDQENDSDSTRNCAVERKGAWWYADCSDSNLNGLHFKEAVTSDYNGIFWDTYRGAKYSLKDTVIKLRPYDIQKHGDSVQPY***	TGCTCATTCACCATTATAAGACTCAGATGTATCCCTAAAATTTCATTTCACTTATTCATTATTTGAAATATAATAATTCATTGTTTTAAATATCTGAATAATTTTTCTCAATATTTTTCTCAATGACAGACGATTCCTTATAAGGGACTTGTTTCCATTCTATATCAGTAACCATTATTGAGAAATTTCTCCGTGTGTATAGGCTGTTTATATAACATATTGTCTTATAACGGATACTTTTTATAATGCCTCTTTTTAAGATATAGATAAAACTAGAACTCTCTAAGTTATAATGCTAATAGTAAATAATTAATATATGTGTTATCCTTTCTTGAGTAAAGCACATATCCTCGTACCTCAGCATAATATTGATTGTGAGAATCAGTTAATATGGTTGAACACTGTCCCCGTGCTTCTGGATGTCATAAGGGCGAAGTTTTATCACAGTATCTTTGAGAGAATATTTCGCTCCTCTGTAAGTATCCCAAAAGATTCCATTGTAATCAGATGTAACAGCTTCTTTAAAATGCAATCCATTGAGATTTGAGTCACTGCAGTCAGCATACCACCAAGCTCCTTTACGTTCAACAGCACAGTTCCTAGTACTGTCGCTATCATTTTCTTGATCTTTACTACTAAACTTTTGTCCATCGTTGCGAAAGACAGAATCACCTGCCGTTCCACTGTACCCCGTCACATGCAATCTGTATTTCTGCTTTTCGTCTTCTATCCAGAAGTCATCGAAGATAGCATAAGTCGTATTTCCTTCCCAGTCAGTCAAAGTAAATTTCGCAACATTCGCTCTTTGATTTGTCAGCAGGAAAATGTTGTCATTACCTAGCCAAAAGTCCTTCTTCAGATCTCCGAAACCATGTTTATAAGATTGCCAACCTCTATAAAAATAATCATGTCGGTTTCCAAAAGCACCTCGCCTCTGAAGTACTGTCCAAGCTCCTCCATCGTCTTCCATGTCGCAGTAGACATCGATCGGACAATTTCTCATCGCATTGCGAGGCCAGATTTTGTACACGCCATTAGTTGCTGAAGGATTTTTCTCCATTATATCAGCGCAGTCCATAGGTTTCTCATATATTGATTCGGCAATGGGGCACCCTGCTTGCAATGTATCATACTTGTTTGCATCATCCAGGAGGATATGTGCGGTCTTTATGAGAACTATTGCAAGAGATTTTTGG

							ATTTGATCAGTCAAGTTCAATTCAAGGGGCAAAGCGCATTGT<br>GATTGAGACGCATAAAAACCGCAACTCAGAATGCCGCAGAA<br>AGTTAGAATCAGCGGTTTCCCGAACATGATGTTAGTGAGAGA<br>CACAGAATTTTAGGCTGCAGTTTCCTCGCAGCGCACTAGATC<br>TCACAGTCTTTCAATCTCTTTTTATAGAGTAAAACAAAAATAT<br>ACTCAGTAACGCGATAATGAAGATGTCGTTTGTCTGTAGACA<br>TTCCAAGAAATACAAGCGAGGATTGCCAAAGCATAAACGAC<br>TTCTCTTCTTGTTTCTTTTTCCTGTGATGGCAGTGAAAAAAGA<br>ATACGACTTCTGGAAGTTCGTGTGACCATGACATCAAAATTT<br>GTAGTTCCGCCAATAGATTAA
PhdFibTec5<br>5	P85031	5,92E-33	47,101	PF00147	17787,5	LGNDNIFSLSNQKV<br>YSVRFDLRDVSNTFR<br>YAQYDSFWIEDESRQ<br>YTLHIDDYSGDAGD<br>AMAYHNNMKFSTR<br>DRNNNNNKDVDSA<br>NAFKGAWWYNAG<br>HNANLNGLYIRGKH	TTGGGAAATGATAATATATTTTCCTTGAGCAACCAGAAAGTT<br>TATTCCGTGAGATTCGATTTGAGAGACGTTTCGAACACCTTC<br>AGATATGCCCAATACGACTCTTTCTGGATTGAAGATGAAAGT<br>CGCCAGTACACTCTGCACATTGATGATTATAGCGGAGATGCA<br>GGCGATGCTATGGCATATCACAATAATATGAAATTTTCTACA<br>AGAGACCGAAACAACAATAATAATAAAGATGTAGATTCTGC<br>CAACGCATTTAAAGGTGCTTGGTGGTATAATGCTGGTCACAA<br>CGCCAATCTGAATGGACTATACATCAGGGGAAAGCATGAAA

						ESFADGINWYQWK<br>ALNESMESTEIKIRPK<br>KFKLPHLTLNTPE*	GTTTTGCTGACGGAATAAACTGGTATCAATGGAAAGCCCTCA<br>ACGAGTCCATGGAATCAACTGAAATAAAGATACGACCAAAA<br>AAATTCAAGCTTCCTCATCTAACCTTAAACACTCCCGAGTGA<br>ATTTTAATAACATATTAGCATCAATATATCTCCGGAG
PhdFibTec5<br>6	P85031	1,48E-72	46,970	PF00147	34327.39<br><br>**32200.69**	MTKYMALLVALAL<br>CFAVSKA**NSSCEQK**<br>**IAESYLDTAVYMIS**<br>**KAKYNIPACIKSGN**<br>**NLTRGALDCSDLFL**<br>**MGYNKSGVYRIHP**<br>**VSRVTEDKPLDVYC**<br>**DMETTGGGWTLIQ**<br>**RRGKFERSSYRDYF**<br>**YKDWKSYKEGFGD**<br>**IERDFWLGNDNIFS**<br>**LSNQKVYSVRFDLR**<br>**DEENNFRYAQYDSF**<br>**WIDDEDHQYTLHIS**<br>**DYSGDAGDSMELH**<br>**KNQKFSTKDRDND**<br>**NNKEQQCATLYKG**<br>**GWWYNSCHHANL**<br>**NGIYIRGKHESFAD**<br>**GLNWYKWKGFNES**	CAGTCACTCATTTATGACGTTATTTAGAGTAAGATTTTAGAGT<br>GGCAAGTGTAATAAGTTAAATTTCCCCAACTGCTCTGTTCCTG<br>TGAGAATAATCTGGGAATTTCTTAAGAGAAAAATGACCAAA<br>TACATGGCATTACTTGTTGCTCTCGCTCTGTGCTTCGCAGTTA<br>GCAAAGCGAATTCAAGTTGTGAGCAGAAAATTGCCGAGTCT<br>TATTTAGATACAGCTGTTTATATGATTTCCAAAGCGAAATAT<br>AACATACCGGCATGTATTAAGAGTGGAAACAATCTGACAAG<br>AGGAGCTCTAGATTGCAGCGACTTATTCCTGATGGGTTACAA<br>CAAAAGTGGTGTGTACAGGATACATCCTGTGAGTAGAGTGA<br>CGGAAGATAAACCACTCGATGTATATTGTGATATGGAAACC<br>ACCGGAGGTGGATGGACTTTAATACAGAGAAGAGGAAAGTT<br>TGAAAGATCATCTTACAGAGATTATTTTTATAAAGACTGGAA<br>AAGTTACAAAGAAGGATTTGGAGACATCGAGAGAGATTTTT<br>GGTTGGGAAATGATAATATATTTTCCTTGAGCAACCAGAAAG<br>TTTATTCCGTGAGGTTCGATTTGAGAGACGAGGAGAACAACT<br>TCAGATATGCCCAGTACGATTCTTTCTGGATTGATGACGAAG<br>ATCACCAGTATACTCTGCACATTTCGGATTATAGTGGAGACG<br>CAGGCGATTCTATGGAACTCCACAAGAATCAGAAATTTTCTA<br>CAAAAGACCGAGACAATGATAATAATAAAGAACAGCAATG<br>CGCCACCTTATATAAAGGTGGATGGTGGTATAATTCTTGTCA<br>CCATGCCAACCTTAATGGAATATACATACGGGGAAAACATG

						**MESTEIKIRPKKFKLPHLTLNTPE***	AAAGTTTTGCTGACGGATTAAACTGGTATAAATGGAAAGGCTTCAACGAATCCATGGAATCAACTGAAATAAAGATACGACCAAAAAAATTCAAGCTTCCTCATCTAACCTTAAACACTCCCGAGTGAATTTTAATAACATATTAGCATCAATATATCTCCGGAG
PhdFibTec57	P85031	4,93E-68	55,789	PF00147	32438.16<br>**30344.57**	MTKYIVQLVALALCCAVSNA**NSSCEPKIAVSYLDTAVDMISKAKYNLTEFISSRNNLTRGALDCSDLLLMGYNKSGVYTVHPVSRVTDGKPVDVYCDMETTRGGWTLIQRRGKFERSSYRDYFYKDWKSYKEGFGDIERDFWLGNDNIFSLSNQKVYSVRFDLRDVENNTRYAQYDSFWIDDEDHQYTLHIAGYSGDAGDSM**	CGAACGATACGGAAGTCTTGCTCAAAATTAGTTATGACACGATAACTGAGCTATTAATAATTTTGTCTCGAGCGCTATATAGTCACTCATTTAGGGTGCAGTTTAGAGGGCCATTTCCGAATCGAAAGTGTGATAGCTTAAATTTTCCGTCAAGTGTACCGTTCTTGTGAATAATCCGTAAATTTCGTTTGTGCAAAATGACCAAATACATAGTACAACTTGTTGCTCTCGCTCTGTGCTGCGCAGTTAGCAATGCGAATTCAAGTTGTGAGCCGAAAATTGCCGTGTCTTATTTAGATACAGCAGTTGATATGATCTCCAAAGCGAAATATAACCTAACGGAATTTATTAGCAGTAGGAACAATCTAACAAGAGGTGCTCTAGATTGCAGTGACTTACTCCTGATGGGTTACAACAAAAGCGGTGTGTACACAGTACATCCTGTGAGTAGAGTGACGGATGGTAAACCAGTCGATGTATACTGTGATATGGAAACGACCAGAGGAGGATGGACTTTAATACAGAGAAGAGGAAAGTTTGAAAGATCATCTTACAGAGATTATTTTATAAAGACTGGAAAAGTTACAAAGAAGGATTTGGAGACATCGAGAGAGATTTTTGGTTGGG

						**IPNHNNQKFTTKDRDNDKHKDVNCAEHHKGAWWYVACQLVNLNGLYHRGKHASLSDGINWYTWKGHNESMESTEIKIRP**	AAATGATAACATATTTTCCCTGAGCAATCAGAAGGTTTATTCCGTCAGATTCGATTTGAGAGATGTGGAAAACAACACCAGATATGCCCAGTACGATTCTTTCTGGATTGATGACGAAGATCACCAGTACACTCTGCACATTGCTGGTTATAGCGGAGATGCAGGTGATTCTATGATACCAAATCATAACAATCAGAAGTTTACCACGAAAGACCGTGATAATGACAAACATAAGGATGTAAATTGTGCCGAACACCATAAAGGAGCATGGTGGTATGTTGCATGTCAATTGGTCAATCTCAACGGTCTCTACCACAGGGGAAAACATGCAAGTCTTTCTGATGGAATAAACTGGTATACCTGGAAAGGCCATAACGAGTCCATGGAATCCACTGAAATAAAGATTCGACCAAA
PhdFibTec58	P85031	3,18E-70	45,211	PF00147	32506.34<br>**30379.63**	MTKYMALLVALALCFAVSKA**NSSCEQKIAESYLDTAVYMISKAKYNIPACIKSGNNLTRGALDCSDLFLMGYNKSGVYRIHPVSRVTEDKPLDVYCDMETTGGGWTLIQRRGKFERSSYRDYFYKDWKSYKEGFGDIERDFWLGNDNIFSLSNQKVYSVRFDLRDVENNTRYAQYDSFWIDDEDHQYTLHI**	CAGTCACTCATTTATGACGTTATTTAGAGTAAGATTTTAGAGTGGCAAGTGTAATAAGTTAAATTTCCCCAACTGCTCTGTTCCTGTGAGAATAATCTGGGAATTTCTTAAGAGAAAAATGACCAAATACATGGCATTACTTGTTGCTCTCGCTCTGTGCTTCGCAGTTAGCAAAGCGAATTCAAGTTGTGAGCAGAAAATTGCCGAGTCTTATTTAGATACAGCTGTTTATATGATTTCCAAAGCGAAATATAACATACCGGCATGTATTAAGAGTGGAAACAATCTGACAAGAGGAGCTCTAGATTGCAGCGACTTATTCCTGATGGGTTACAACAAAAGTGGTGTGTACAGGATACATCCTGTGAGTAGAGTGACGGAAGATAAACCACTCGATGTATATTGTGATATGGAAACCACCGGAGGTGGATGGACTTTAATACAGAGAAGAGGAAAGTTTGAAAGATCATCTTACAGAGATTATTTTTATAAAGACTGGAAAAGTTACAAAGAAGGATTTGGAGACATCGAGAGAGATTTTTGGTTGGGAAATGATAACATATTTTCCCTGAGCAATCAGAAGG

						**AGYSGDAGDSMIP**<br>**NHNNQKFTTKDRD**<br>**NDKHKDVNCAEH**<br>**HKGAWWYVACQL**<br>**VNLNGLYHRGKHA**<br>**SLSDGINWYTWKG**<br>**HNESMESTEIKIRP**	TTTATTCCGTCAGATTCGATTTGAGAGATGTGGAAAACAACA<br>CCAGATATGCCCAGTACGATTCTTTCTGGATTGATGACGAAG<br>ATCACCAGTACACTCTGCACATTGCTGGTTATAGCGGAGATG<br>CAGGTGATTCTATGATACCAAATCATAACAATCAGAAGTTTA<br>CCACGAAAGACCGTGATAATGACAAACATAAGGATGTAAAT<br>TGTGCCGAACACCATAAAGGAGCATGGTGGTATGTTGCATGT<br>CAATTGGTCAATCTCAACGGTCTCTACCACAGGGGAAAACAT<br>GCAAGTCTTTCTGATGGAATAAACTGGTATACCTGGAAAGGC<br>CATAACGAGTCCATGGAATCCACTGAAATAAAGATTCGACC<br>AAA
PhdFibTec5<br>9	P85031	6,08E-65	45,374	PF00147	28441.61<br><br>**26000.66**	MIRYLTIFMFLAIYYE<br>VSNA**TLSCEQKVSIS**<br>**YLDTAVDMISKAK**<br>**YGIPACNNCASDST**<br>**RGALDCSDLLQRG**<br>**YNKSGVYTIWPASR**<br>**IMEEKSLDVYCDM**<br>**QTNGGGWTLIQRR**<br>**GNFQRGKDYFFKD**<br>**WKSYKDGFGDIEK**<br>**DFWLGNDNIFALS**<br>**NQKLYSVRFDLTDV**<br>**ENASKYALYDTFWT**<br>**DDENKQYTLHIAD**<br>**YSGDAGDSVMAYH**	TTTAGAGCGGCGAGTGTGATAAGTTAAATTTTCCCAACTGTA<br>CTGTTCCTGCGAGAATAATCTGTGAATTTCTTGAGCGTAAAA<br>TGATCAGGTATTTGACCATATTTATGTTCCTTGCTATCTATTAC<br>GAAGTTAGCAATGCGACTCTCAGTTGTGAGCAGAAAGTTTCT<br>ATATCCTATTTAGACACAGCAGTGGATATGATTTCCAAGGCG<br>AAATACGGTATTCCAGCGTGTAATAACTGTGCAAGTGATTCG<br>ACTCGAGGTGCCCTAGATTGCAGTGACTTACTTCAAAGGGGT<br>TACAATAAAAGTGGTGTGTACACTATATGGCCCGCAAGTAG<br>AATAATGGAGGAAAAATCATTAGATGTGTATTGTGACATGCA<br>AACGAATGGAGGAGGATGGACTTTAATACAGAGAAGAGGA<br>AACTTTCAGAGAGGAAAGGACTATTTTTTCAAAGACTGGAA<br>AAGTTACAAAGACGGTTTCGGGGACATTGAAAAGGACTTTTG<br>GTTAGGAAACGATAACATATTCGCCTTGAGCAATCAGAAGCT<br>TTATTCCGTCAGATTCGATTTGACAGACGTGGAGAATGCTTCT<br>AAATATGCACTGTATGACACCTTCTGGACTGACGATGAAAAT

						**NHQNFTTKDRDNDNHKDANCAEFHKGGWWFGACRLSNLNGLYH**	AAACAGTACACTCTACACATTGCTGATTACAGCGGAGATGCAGGTGATTCTGTGATGGCATATCATAACCATCAGAATTTTACTACGAAAGACCGTGATAATGACAACCATAAGGACGCAAATTGTGCCGAATTTCATAAAGGAGGATGGTGGTTTGGGGCATGTCGATTGTCAAATCTGAACGGGCTCTACCATAC
PhdFibTec60	P85031	1,46E-71	58,730	PF00147	28258,32	MISKAKFNIPARIKSGDSLTRGALDCSDLLLMGYNKSGVYTIHPVSRVTDDKQFDVYCDMETTGGGWTLIQRRGKFERSSYRGYFYKDWKSYKEGFGDIEKDFWLGNDNIFSLSNQKVYSVRFDLRDVENNTRYAQYDSFWIDDEDHQYTLHIAGYSGDAGDSMETHNNQKFSTKDRDNDSRKDQDCANLYKGGWWYNTCHLANLNGLYILGKHESFANGMN	ATGATTTCAAAAGCGAAATTTAATATTCCCGCACGTATTAAGAGTGGAGACAGTCTCACAAGAGGAGCTCTTGATTGCAGTGACTTACTCCTGATGGGTTACAACAAAAGCGGTGTGTACACAATACATCCTGTGAGTAGAGTTACTGATGATAAACAATTCGATGTATATTGTGATATGGAAACCACTGGAGGAGGTTGGACTTTAATACAGAGAAGAGGAAAGTTTGAGAGATCATCTTACAGAGGTTATTTTTATAAAGATTGGAAAAGTTACAAAGAGGGATTTGGAGACATCGAAAAAGATTTTTGGTTGGGAAATGATAACATATTTTCCCTGAGCAATCAGAAGGTTTATTCCGTCAGATTCGATTTGAGAGACGTGGAAAACAACACCAGATATGCCCAGTACGATTCTTTCTGGATTGATGACGAAGATCACCAGTACACTCTGCACATTGCTGGTTATAGCGGAGATGCAGGTGATTCCATGGAAACCCACAATAATCAGAAATTTTCTACGAAAGACCGAGACAATGATAGTAGGAAAGATCAAGATTGTGCCAACTTATATAAAGGTGGATGGTGGTACAATACGTGTCACCTAGCCAATCTGAACGGACTGTACATCCTGGGAAAACATGAAAGTTTTGCTAATGGCATGAAC

						WKTWKHYNESMKS<br>TEIKIRP	TGGAAAACATGGAAACACTACAATGAGTCCATGAAATCAAC<br>TGAAATAAAGATTCGACCAAA
PhdFibTec6<br>1	P85031	1,56E-60	42,562	PF00147	29984.32<br><br>**27890.73**	MTKYIVQLVALALC<br>CAVSNA**NSSCEPKI**<br>**AVSYLDTAVDMIS**<br>**KAKYNIPACIKSGN**<br>**NLTRGPLDCSDLLL**<br>**MGYNKSGVYRINP**<br>**VSRVTDDKPLDVY**<br>**CDMETTGGGWTLI**<br>**QRRGKFERSGYKD**<br>**YFHKDWKSYKEGF**<br>**GDIERDFWLGNDN**<br>**IFSLSNQNVYSIRID**<br>**LRDAENNTAYAQY**<br>**DSFWIDDEDHQYT**<br>**LHLADFHGNAGDS**<br>**LLAHHNHQKFTTK**<br>**DRDNDNHKDANC**<br>**AEFHKGGWWFGA**	CGAACGATACGGAAGTCTTGCTCAAAATTAGTTATGACACGA<br>TAACTGAGCTATTAATAATTTTGTCTCGAGCGCTATATAGTCA<br>CTCATTTAGGGTGCAGTTTAGAGGGCCATTTCCGAATCGAAA<br>GTGTGATAGCTTAAATTTTCCGTCAAGTGTACCGTTCTTGTGA<br>ATAATCCGTAAATTTCGTTTGTGCAAAATGACCAAATACATA<br>GTACAACTTGTTGCTCTCGCTCTGTGCTGCGCAGTTAGCAATG<br>CGAATTCAAGTTGTGAGCCGAAAATTGCCGTGTCTTATTTAG<br>ATACAGCAGTGGATATGATTTCTAAAGCGAAATATAATATTC<br>CGGCATGTATTAAGAGTGGAAACAATCTGACAAGAGGACCG<br>CTAGATTGCAGCGACTTATTGCTGATGGGTTACAACAAAAGT<br>GGTGTGTACAGAATAAATCCTGTGAGTAGAGTGACGGATGA<br>TAAACCACTCGATGTATATTGTGATATGGAAACCACTGGAGG<br>AGGTTGGACTTTAATACAGAGAAGAGGAAAGTTTGAGAGAT<br>CAGGTTACAAAGATTATTTTCATAAAGATTGGAAAAGTTACA<br>AGGAAGGATTTGGAGACATCGAAAGAGATTTTTGGTTGGGA<br>AATGATAACATATTTTCCTTGAGCAACCAGAATGTATATTCC<br>ATCAGAATCGATTTGCGAGACGCCGAGAACAACACGGCATA<br>TGCTCAATACGATTCTTTTTGGATTGATGACGAAGATCACCA

						**CRLSNLNGLYHTG**<br>**KHESLDGINWYT**	GTACACTCTGCACCTTGCTGATTTCCACGGGAATGCAGGTGA<br>TTCTCTGTTGGCACATCATAATCATCAGAAGTTTACTACGAA<br>AGACCGTGATAATGACAACCATAAGGACGCAAATTGTGCCG<br>AATTTCATAAAGGAGGATGGTGGTTTGGGGCATGTCGATTGT<br>CAAATCTGAACGGGCTCTACCATACGGGAAAACATGAAAGT<br>CTTGATGGAATAAACTGGTATACCT
PhdFibTec6<br>2	P85031	3,21E-24	56,250	PF00147	9802,72	TLIQRRGNFQRGKD<br>YFFKDWKSYKDGFG<br>DIEKDFWLGNDNIY<br>SLSNQKLYSVRFDLT<br>DVENNTKYALYDTF<br>WIDDENQQY	ACTTTAATACAGAGAAGAGGAAACTTTCAGAGAGGAAAGGA<br>CTATTTTTTCAAAGACTGGAAAAGTTACAAAGACGGCTTCGG<br>GGACATCGAAAAAGATTTTTGGTTAGGAAACGATAATATAT<br>ATTCCTTGAGCAACCAGAAACTTTATTCCGTCAGATTTGATTT<br>GACAGACGTGGAGAACAACACTAAATATGCCCTATATGATA<br>CTTTCTGGATTGACGATGAAAATCAACAGTACA
PhdFibTec6<br>3	P85031	1,39E-36	42,169	PF00147	21534.24<br><br>**19093.30**	MIRYLTIFMFLAIYYE<br>VSNA**TLSCEQKVSIS**<br>**YLDTAVDMISKAK**<br>**YGIPACNNCASDST**<br>**RGALDCSDLLQRG**<br>**YNKSGVYTIWPVSR**<br>**MMEEKSLEVYCDM**<br>**QTNGGGWTLIQRR**<br>**GNFQRGKDYFLKD**	TTTAGAGCGGCGAGTGTGATAAGTTAAATTTTCCCAACTGTA<br>CTGTTCCTGCGAGAATAATCTGTGAATTTCTTGAGCGTAAAA<br>TGATCAGGTATTTGACCATATTTATGTTCCTTGCTATCTATTAC<br>GAAGTTAGCAATGCGACTCTCAGTTGTGAGCAGAAAGTTTCT<br>ATATCCTATTTAGACACAGCAGTGGATATGATTTCCAAGGCG<br>AAATACGGTATTCCAGCGTGTAATAACTGTGCAAGTGATTCG<br>ACTCGAGGTGCCCTAGATTGCAGTGACTTACTTCAAAGGGGT<br>TACAATAAAAGTGGTGTGTACACTATATGGCCCGTGAGTAGA<br>ATGATGGAAGAAAAATCATTAGAAGTATATTGTGACATGCA

						**WKSYKDGFGDMEKDFWLGNDNIFALSNQKLYSVRFDLTDVENNTKYALYDTFWIDDENQQY**	AACAAATGGAGGAGGATGGACTTTAATACAGAGGAGAGGAAACTTTCAAAGGGGAAAGGACTATTTCCTGAAAGACTGGAAAAGTTACAAAGATGGTTTCGGGGACATGGAAAAGGATTTTTGGTTAGGAAACGATAACATATTCGCCTTGAGCAATCAGAAGCTTTATTCCGTCAGATTTGATTTGACAGACGTGGAGAACAACACTAAATATGCCCTATATGATACTTTCTGGATTGACGATGAAAATCAACAGTACA
PhdFibTec64	P85031	2,12E-32	40,361	PF00147	21385.12<br>**19258.41**	MTKYMALLVALALCFAVSKA**NSSCEQKIAESYLDTAVYMISKAKYNIPACIKSGNNLTRGALDCSDLFLMGYNKSGVYRIHPVSRVTEDKPLDVYCDMETTGGGWTLIQRRGKFERSGYKDYFHKDWKSYKEGFGDIERDFWLGNDNIFSVTNQKVYSVRFDLRDVENNVRYAQYDSFWIDDEDH**	CAGTCACTCATTTATGACGTTATTTAGAGTAAGATTTTAGAGTGGCAAGTGTAATAAGTTAAATTTCCCCAACTGCTCTGTTCCTGTGAGAATAATCTGGGAATTTCTTAAGAGAAAAATGACCAAATACATGGCATTACTTGTTGCTCTCGCTCTGTGCTTCGCAGTTAGCAAAGCGAATTCAAGTTGTGAGCAGAAAATTGCCGAGTCTTATTTAGATACAGCTGTTTATATGATTTCCAAAGCGAAATATAACATACCGGCATGTATTAAGAGTGGAAACAATCTGACAAGAGGAGCTCTAGATTGCAGCGACTTATTCCTGATGGGTTACAACAAAAGTGGTGTGTACAGGATACATCCTGTGAGTAGAGTGACGGAAGATAAACCACTCGATGTATATTGTGATATGGAAACCACTGGAGGAGGTTGGACTTTAATACAGAGAAGAGGAAAGTTTGAGAGATCAGGTTACAAAGATTATTTCATAAAGATTGGAAAAGTTACAAGGAAGGATTTGGAGACATCGAAAGAGATTTTTGGTTGGGAAATGATAATATATTTTCTGTGACCAACCAGAAAGTTTATTCCGTGAGATTCGATTTGAGAGACGTGGAGAACAACGTGAGATATGCTCAGTACGATTCTTTTTGGATTGATGACGAAGATCACCA

PhdFibTec65	P85031	3,21E-70	46,591	PF00147	34259.21<br><br>**32165.63**	MTKYIVQLVALALCCAVSNA**NSSCEPKIAVSYLDTAVDMISKAKYNLTEFISSRNNLTRGALDCSDLLLMGYNKSGVYTVHPVSRVTDGKPVDVYCDMETTRGGWTLIQRRGKFERSSYRDYFYKDWKSYKEGFGDIERDFWLGNDNIFSLSNQKVYSVRFDLRDEENNFRYAQYDSFWIDDEDHQYTLHISDYSGDAGDSMELHKNQKFSTKDRDNDNNKEQQCATLYKGGWWYNSCHHANLNGIYIRGKHESFADGLNWYKWKGFNESMESTEIKIRPKKFKLPHLTLNTPE***	CGAACGATACGGAAGTCTTGCTCAAAATTAGTTATGACACGATAACTGAGCTATTAATAATTTTGTCTCGAGCGCTATATAGTCACTCATTTAGGGTGCAGTTTAGAGGGCCATTTCCGAATCGAAAGTGTGATAGCTTAAATTTTCCGTCAAGTGTACCGTTCTTGTGAATAATCCGTAAATTTCGTTTGTGCAAAATGACCAAATACATAGTACAACTTGTTGCTCTCGCTCTGTGCTGCGCAGTTAGCAATGCGAATTCAAGTTGTGAGCCGAAAATTGCCGTGTCTTATTTAGATACAGCAGTTGATATGATCTCCAAAGCGAAATATAACCTAACGGAATTTATTAGCAGTAGGAACAATCTAACAAGAGGTGCTCTAGATTGCAGTGACTTACTCCTGATGGGTTACAACAAAAGCGGTGTGTACACAGTACATCCTGTGAGTAGAGTGACGGATGGTAAACCAGTCGATGTATACTGTGATATGGAAACGACCAGAGGAGGATGGACTTTAATACAGAGAAGAGGAAAGTTTGAAAGATCATCTTACAGAGATTATTTTTATAAAGACTGGAAAAGTTACAAAGAAGGATTTGGAGACATCGAGAGAGATTTTTGGTTGGGAAATGATAATATATTTTCCTTGAGCAACCAGAAAGTTTATTCCGTGAGGTTCGATTTGAGAGACGAGGAGAACAACTTCAGATATGCCCAGTACGATTCTTTCTGGATTGATGACGAAGATCACCAGTATACTCTGCACATTTCGGATTATAGTGGAGACGCAGGCGATTCTATGGAACTCCACAAGAATCAGAAATTTTCTACAAAAGACCGAGACAATGATAATAATAAAGAACAGCAATGCGCCACCTTATATAAAGGTGGATGGTGGTATAATTCTTGTCACCATGCCAACCTTAATGGAATATACATACGGGGAAAACATGAAAGTTTTGCTGACGGATTAAACTGGTATAAATGGAAAGGCTTCAACGAATCCATGGAATCAACTGAAATAAAGATACGACCAAAAAAATTCAAGCTTCCTCATCTAACCTTAAACACTCCCGAGTGAATTTTAATAACATATTAGCATCAATATATCTCCGGAG

PhdFibTec66	P85031	8,16E-51	60,630	PF00147	17867,67	LLLMGYNRSGVYTI<br>WPLSRILERKSLEVY<br>CDMETNGGGWTVI<br>QRRGNFQRPKDYFF<br>KDWKSYKTGFGDIE<br>KDFWLGNDNIFALS<br>NQKVYSARFDLTDV<br>ENNTKYALYDTFWI<br>DDENQQYTLHIADY<br>SGDAGDSMTERHN<br>NEKFSTKDRDND	CTTACTTTTAATGGGTTACAATAGGAGTGGTGTGTACACTATA<br>TGGCCCCTGAGTAGAATATTGGAGAGAAAATCATTAGAAGT<br>ATATTGTGACATGGAAACGAATGGAGGAGGATGGACTGTAA<br>TACAAAGAAGAGGAAATTTTCAGAGACCAAAGGACTATTTT<br>TTCAAAGACTGGAAAAGTTACAAGACTGGCTTCGGCGACATT<br>GAAAAAGATTTTTGGCTAGGAAACGATAATATATTTGCTTTG<br>AGCAACCAGAAAGTTTATTCCGCCAGATTTGATTTGACAGAC<br>GTGGAGAACAACACTAAATATGCCCTATATGATACTTTCTGG<br>ATTGACGATGAAAATCAACAGTACACCCTGCACATTGCTGAT<br>TACAGCGGGGATGCAGGTGATTCTATGACGGAGAGACATAA<br>CAATGAGAAGTTTAGTACGAAAGACCGTGACAATGACA
PhdFibTec67	P85031	2,43E-73	47,876	PF00147	33832.82<br>**31391.87**	MIRYLTIFMFLAIYYE<br>VSNA**TLSCEQKVSIS**<br>**YLDTAVDMISKAK**<br>**YGIPACNNCASDST**<br>**RGALDCSDLLQRG**<br>**YNKSGVYTIWPASR**<br>**IMEEKSLDVYCDM**<br>**QTNGGGWTLIQRR**<br>**GNFQRGKDYFFKD**<br>**WKSYKDGFGDIEK**<br>**DFWLGNDNIFALS**<br>**NQKLYSVRFDLTDV**<br>**ENASKYALYDTFWT**<br>**DDENKQYTLHIAD**<br>**YSGDAGDSMMER**<br>**HNNQKFSTKDSDH**<br>**DSHKDLNCAKQFK**	TTTAGAGCGGCGAGTGTGATAAGTTAAATTTTCCCAACTGTA<br>CTGTTCCTGCGAGAATAATCTGTGAATTTCTTGAGCGTAAAA<br>TGATCAGGTATTTGACCATATTTATGTTCCTTGCTATCTATTAC<br>GAAGTTAGCAATGCGACTCTCAGTTGTGAGCAGAAAGTTTCT<br>ATATCCTATTTAGACACAGCAGTGGATATGATTTCCAAGGCG<br>AAATACGGTATTCCAGCGTGTAATAACTGTGCAAGTGATTCG<br>ACTCGAGGTGCCCTAGATTGCAGTGACTTACTTCAAAGGGGT<br>TACAATAAAAGTGGTGTGTACACTATATGGCCCGCAAGTAG<br>AATAATGGAGGAAAAATCATTAGATGTGTATTGTGACATGCA<br>AACGAATGGAGGAGGATGGACTTTAATACAGAGAAGAGGA<br>AACTTTCAGAGAGGAAAGGACTATTTTTTCAAAGACTGGAA<br>AAGTTACAAAGACGGTTTCGGGGACATTGAAAAGGACTTTTG<br>GTTAGGAAACGATAACATATTCGCCTTGAGCAATCAGAAGCT<br>TTATTCCGTCAGATTCGATTTGACAGACGTGGAGAATGCTTCT<br>AAATATGCACTGTATGACACCTTCTGGACTGACGATGAAAAT<br>AAACAGTACACTCTACACATTGCTGATTACAGCGGAGATGC<br>AGGTGATTCCATGATGGAAAGACATAACAACCAGAAGTTTA

						**GGWWYGACHFANLNGLYHRGKHESEADGVNWFTWKGHKESLESTEIKIRPKNFKLPHVTMNTPL***	GTACGAAAGACAGTGATCATGACAGCCATAAGGATTTAAATTGTGCCAAACAATTTAAAGGAGGATGGTGGTATGGTGCCTGTCACTTCGCCAATCTGAACGGACTCTACCATAGGGGAAAACATGAAAGTGAAGCAGATGGAGTAAACTGGTTTACCTGGAAAGGCCATAAGGAATCTTTGGAATCAACTGAAATAAAGATTCGACCAAAAAATTTCAAGTTGCCTCATGTAACCATGAATACTCCTTTGTGAAATTTTATAACTGAGTAGCGTTAATAACGCCTGAATACTTATATCGCTT
PhdFibTec68	P85031	3,96E-72	46,743	PF00147	32433.15<br>**30339.56**	MTKYIVQLVALALCCAVSNA**NSSCEPKIAVSYLDTAVDMISKAKYNLTEFISSRNNLTRGALDCSDLLLMGYNKSGVYTVHPVSRVTDGKPVDVYCDMETTRGGWTLIQRRGKFERSSYRDYFYKDWKSYKEGFGDIERDFWLGNDNIFSLSNQKVYSVRFDLRDVENNTRYAQYDSFWIDDEDHQYTLHIAGYSGDAGDSM**	CGAACGATACGGAAGTCTTGCTCAAAATTAGTTATGACACGATAACTGAGCTATTAATAATTTTGTCTCGAGCGCTATATAGTCACTCATTTAGGGTGCAGTTTAGAGGGCCATTTCCGAATCGAAAGTGTGATAGCTTAAATTTTCCGTCAAGTGTACCGTTCTTGTGAATAATCCGTAAATTTCGTTTGTGCAAAATGACCAAATACATAGTACAACTTGTTGCTCTCGCTCTGTGCTGCGCAGTTAGCAATGCGAATTCAAGTTGTGAGCCGAAAATTGCCGTGTCTTATTTAGATACAGCAGTTGATATGATCTCCAAAGCGAAATATAACCTAACGGAATTTATTAGCAGTAGGAACAATCTAACAAGAGGTGCTCTAGATTGCAGTGACTTACTCCTGATGGGTTACAACAAAAGCGGTGTGTACACAGTACATCCTGTGAGTAGAGTGACGGATGGTAAACCAGTCGATGTATACTGTGATATGGAAACGACCAGAGGAGGATGGACTTTAATACAGAGAAGAGGAAAGTTTGAAAGATCATCTTACAGAGATTATTTTTATAAAGACTGGAAAAGTTACAAAGAAGGATTTGGAGACATCGAGAGAGATTTTTGGTTGGG

						**ETHNNQKFSTKDR DNDSRKDQDCANL YKGGWWYNTCHL ANLNGLYILGKHES FANGMNWKTWKH YNESMKSTEIKIRP**	AAATGATAACATATTTTCCCTGAGCAATCAGAAGGTTTATTC CGTCAGATTCGATTTGAGAGATGTGGAAAACAACACCAGAT ATGCCCAGTACGATTCTTTCTGGATTGATGACGAAGATCACC AGTACACTCTGCACATTGCTGGTTATAGCGGAGATGCAGGTG ATTCCATGGAAACCCACAATAATCAGAAATTTTCTACGAAAG ACCGAGACAATGATAGTAGGAAAGATCAAGATTGTGCCAAC TTATATAAAGGTGGATGGTGGTACAATACGTGTCACCTAGCC AATCTGAACGGACTGTACATCCTGGGAAAACATGAAAGTTTT GCTAATGGCATGAACTGGAAAACATGGAAACACTACAATGA GTCCATGAAATCAACTGAAATAAAGATTCGACCAAA
PhdFibTec69	P85031	3,34E-56	53,047	PF00147	26012,62	MISKAKFNIPARIKS GDSLTRGALDCSDL LLMGYNKSGVYTIH PVSRVTDDKQFDVY CDMETTGGGWTLIQ RRGKFERSSYRGYFY KDWKSYKEGFGDIE KDFWLGNDNIFSLS NQNVYSIRIDLRDAE NNTAYAQYDSFWID DEDHQYTLHLADFH GNAGDSLLAHHNH QKFTTKDRDNDNH KDANCAEFHKGGW	ATGATTTCAAAAGCGAAATTTAATATTCCCGCACGTATTAAG AGTGGAGACAGTCTCACAAGAGGAGCTCTTGATTGCAGTGA CTTACTCCTGATGGGTTACAACAAAAGCGGTGTGTACACAAT ACATCCTGTGAGTAGAGTTACTGATGATAAACAATTCGATGT ATATTGTGATATGGAAACCACTGGAGGAGGTTGGACTTTAAT ACAGAGAAGAGGAAAGTTTGAGAGATCATCTTACAGAGGTT ATTTTTATAAAGATTGGAAAAGTTACAAAGAGGGATTTGGAG ACATCGAAAAAGATTTTTGGTTGGGAAATGATAACATATTTT CCTTGAGCAACCAGAATGTATATTCCATCAGAATCGATTTGC GAGACGCCGAGAACAACACGGCATATGCTCAATACGATTCT TTTTGGATTGATGACGAAGATCACCAGTACACTCTGCACCTT GCTGATTTCCACGGGAATGCAGGTGATTCTCTGTTGGCACAT CATAATCATCAGAAGTTTACTACGAAAGACCGTGATAATGA CAACCATAAGGACGCAAATTGTGCCGAATTTCATAAAGGAG

						WFGACRLSNLNGLYHTGKHESLDGINWYT	GATGGTGGTTTGGGGCATGTCGATTGTCAAATCTGAACGGGCTCTACCATACGGGAAAACATGAAAGTCTTGATGGAATAAACTGGTATACCT
PhdFibTec70	P85031	2,12E-74	47,126	PF00147	32501.33<br>**30374.62**	MTKYMALLVALALCFAVSKA**NSSCEQKIAESYLDTAVYMISKAKYNIPACIKSGNNLTRGALDCSDLFLMGYNKSGVYRIHPVSRVTEDKPLDVYCDMETTGGGWTLIQRRGKFERSSYRDYFYKDWKSYKEGFGDIERDFWLGNDNIFSLSNQKVYSVRFDLRDVENNTRYAQYDSFWIDDEDHQYTLHIAGYSGDAGDSMETHNNQKFSTKDRDNDSRKDQDCANLYKGGWWYNTCHLANLNGLYILGKHESFA**	CAGTCACTCATTTATGACGTTATTTAGAGTAAGATTTTAGAGTGGCAAGTGTAATAAGTTAAATTTCCCCAACTGCTCTGTTCCTGTGAGAATAATCTGGGAATTTCTTAAGAGAAAAATGACCAAATACATGGCATTACTTGTTGCTCTCGCTCTGTGCTTCGCAGTTAGCAAAGCGAATTCAAGTTGTGAGCAGAAAATTGCCGAGTCTTATTTAGATACAGCTGTTTATATGATTTCCAAAGCGAAATATAACATACCGGCATGTATTAAGAGTGGAAACAATCTGACAAGAGGAGCTCTAGATTGCAGCGACTTATTCCTGATGGGTTACAACAAAAGTGGTGTGTACAGGATACATCCTGTGAGTAGAGTGACGGAAGATAAACCACTCGATGTATATTGTGATATGGAAACCACCGGAGGTGGATGGACTTTAATACAGAGAAGAGGAAAGTTTGAAAGATCATCTTACAGAGATTATTTTTATAAAGACTGGAAAAGTTACAAAGAAGGATTTGGAGACATCGAGAGAGATTTTTGGTTGGGAAATGATAACATATTTTCCCTGAGCAATCAGAAGGTTTATTCCGTCAGATTCGATTTGAGAGATGTGGAAAACAACACCAGATATGCCCAGTACGATTCTTTCTGGATTGATGACGAAGATCACCAGTACACTCTGCACATTGCTGGTTATAGCGGAGATGCAGGTGATTCCATGGAAACCCACAATAATCAGAAATTTTCTACGAAAGACCGAGACAATGATAGTAGGAAAGATCAAGATTGT

						<u>**NGMNWKTWKHYNESMKSTEIKIRP**</u>	GCCAACTTATATAAAGGTGGATGGTGGTACAATACGTGTCACCTAGCCAATCTGAACGGACTGTACATCCTGGGAAAACATGAAAGTTTTGCTAATGGCATGAACTGGAAAACATGGAAACACTACAATGAGTCCATGAAATCAACTGAAATAAAGATTCGACCAAA

PhdFibTec71	P85031	5,04E-60	44,534	PF00147	35132.00<br>**32780.26**	MFSNVLIYVTLWSFLGYSASA**DDPECSSLLLKNITVRTQSSIVLTLLSNARNLIDDAKRYVTVKTRCCDAGSVCEKPMDCAEITEKYLSERSGVYKIWPRSRAITGPIDVYCDMEDDGGAWTVLQRRGDFDSEKDYFFKDWESYKNGFGDKTKDFWIGNDNIFALTNQRSNVVKFSLTDWQKNETYATFDEFWIEDQSRNYTMHVSGYRGTAGDSFSSTNERQFTTKDKDNDSHDNGNCAVQYKGAWWYTNCHNSNLNGLYLKGAHENKDGVIWGTFRGHRISLKDTTIKIRPRDFKKNSDATTPH***	TTCGATTTTTTTCTCATATGCTTTGGTATTTAAACAGGGAAAGTTCCTTGAGATTTAGCGTCTCAAAAAGATCGTGACCGACGTTTTAACTCTATCTCCTGCCATGTTTTCGAACGTTTTAATTTATGTAACTCTCTGGAGTTTTTTGGGTTACAGTGCGTCCGCAGATGATCCGGAATGCTCTTCGCTGCTGTTAAAGAATATAACCGTCCGAACACAATCTTCCATTGTATTGACTCTCTTAAGTAATGCTAGAAATCTTATAGACGATGCAAAGAGATATGTAACCGTAAAAACCAGATGCTGTGATGCTGGATCAGTGTGTGAGAAACCAATGGACTGCGCTGAGATAACAGAAAAATATCTCTCTGAACGCAGTGGTGTATATAAAATCTGGCCTCGAAGCAGAGCGATCACTGGTCCTATCGATGTCTATTGCGATATGGAGGACGATGGAGGAGCTTGGACGGTACTTCAGAGACGAGGAGATTTCGATAGCGAGAAAGATTATTTTTTTAAAGACTGGGAATCTTATAAAAACGGTTTTGGCGATAAAACGAAAGATTTCTGGATAGGTAATGACAACATCTTTGCTCTCACGAATCAAAGGTCGAATGTCGTAAAATTTTCTTTGACTGACTGGCAAAAAAATGAAACGTACGCAACCTTCGACGAATTCTGGATTGAAGACCAATCTCGTAACTACACCATGCATGTGAGTGGGTATAGAGGAACAGCTGGCGATTCATTTTCGAGTACTAATGAAAGACAGTTTACTACTAAAGATAAAGACAATGATAGTCATGATAATGGGAATTGTGCTGTCCAGTATAAAGGTGCTTGGTGGTATACCAACTGCCATAATTCAAACCTTAACGGACTGTACTTGAAAGGAGCTCATGAAAATAAGGATGGAGTAATATGGGGCACCTTTAGAGGACACCGCATATCTCTGAAGGATACTACCATAAAGATTCGCCCTAGAGATTTCAAGAAGAATTCAGATGCTACTACACCGCATTAACTGCTTTCCATCCCTAGTAGCTCGACTGAAAACAACATTTTCTTTCAAAACTCGATTGCAACAATGATTTCCATTAGTGAAAAATCAACAACTACCGTTAAATTTCAGCATTCAGCAAATTCTATCAAATGGAACAGAATGGTATCATCA

							TAAAAATTCTTATTTTGCTCTGATATATATATATATATATGAC<br>ACATTTATATATATGTATGTGTGTGTGTGTGTGTGTGAAAGAG<br>AGAGAGAGAGAGAGAGAGTAATAAAATATTAAAAAGACTT<br>AGACTTTAGTTTAAATTAATATTCTTATTTCATATCTGGTTTAC<br>ACATCACAATTACAGACTATGAACAAAATTTTCTAGACTTAC<br>ATCCATACCTTAATCGCTTAATCGATATCGAGCAACTGACTG<br>ATTTTACAGCAGTTACAATCCTTTCTGAAAGACTTTAAGACA<br>CCTTATCGGATAAAGACATCTTAAACACCACGCCGGAGAAG<br>ATGCATGAGAATGGACATTATTGGACTTTTGAAACGGAGGTT<br>TTAAGTGAAACTTACTTTTTTGGGAAGCAACAGATACATCAT<br>CCATAACAGCAACTGTTACTTTCATCAATTTTTGGAACTAGTG<br>GGTGATTTCACGCATTCCATTTTAGTTGTTCAGCTTTTGACCGT<br>AAAATAAATCTGTGGTGTTGTTGCTATCAGTGACTTTGCTAGC<br>ATATAAATTTTTGGATGAATAATTATATATGAAAATTTATTTC<br>TCTCCCTGACCACTCTGAGGTAAATTTAAAAGTAAGAGCATT<br>GAAGCGCGTATAAATACTACGTGGTATTGATAAAATGACAT<br>GTCAATTTTGTGAGCAAGTTATCTTAGTATATTAGAAAGAATT<br>CACGCTATTAAGATCGAAACGAAAAAAAACTAATGGCATTT<br>AGCTTGCGCCCCATGCT

PhdFibTec72	P85031	1,67E-58	44,534	PF00147	35132.00<br>**32780.26**	MFSNVLIYVTLWSFLGYSASA**DDPECSSLLLKNITVRTQSSIVLTLLSNARNLIDDAKRYVTVKTRCCDAGSVCEKPMDCAEITEKYLSERSGVYKIWPRSRAITGPIDVYCDMEDDGGAWTVLQRRGDFDSEKDYFFKDWESYKNGFGDKTKDFWIGNDNIFALTNQRSNVVKFSLTDWQKNETYATFDEFWIEDQSRNYTMHVSGYRGTAGDSFSSTNERQFTTKDKDNDSHDNGNCAVQYKGAWWYTNCHNSNLNGLYLKGAHENKDGVIWGTFRGHRISLKDTTIKIRPRDFKKNSDATTPH***	TTCGATTTTTTTCTCATATGCTTTGGTATTTAAACAGGGAAAGTTCCTTGAGATTTAGCGTCTCAAAAAGATCGTGACCGACGTTTTAACTCTATCTCCTGCCATGTTTTCGAACGTTTTAATTTATGTAACTCTCTGGAGTTTTTTGGGTTACAGTGCGTCCGCAGATGATCCGGAATGCTCTTCGCTGCTGTTAAAGAATATAACCGTCCGAACACAATCTTCCATTGTATTGACTCTCTTAAGTAATGCTAGAAATCTTATAGACGATGCAAAGAGATATGTAACCGTAAAAACCAGATGCTGTGATGCTGGATCAGTGTGTGAGAAACCAATGGACTGCGCTGAGATAACAGAAAAATATCTCTCTGAACGCAGTGGTGTATATAAAATCTGGCCTCGAAGCAGAGCGATCACTGGTCCTATCGATGTCTATTGCGATATGGAGGACGATGGAGGAGCTTGGACGGTACTTCAGAGACGAGGAGATTTCGATAGCGAGAAAGATTATTTTTTTAAAGACTGGGAATCTTATAAAAACGGTTTTGGCGATAAAACGAAAGATTTCTGGATAGGTAATGACAACATCTTTGCTCTCACGAATCAAAGGTCGAATGTCGTAAAATTTTCTTTGACTGACTGGCAAAAAAAATGAAACGTACGCAACCTTCGACGAATTCTGGATTGAAGACCAATCTCGTAACTACACCATGCATGTGAGTGGGTATAGAGGAACAGCTGGCGATTCATTTTCGAGTACTAATGAAAGACAGTTTACTACTAAAGATAAAGACAATGATAGTCATGATAATGGGAATTGTGCTGTCCAGTATAAAGGTGCTTGGTGGTATACCAACTGCCATAATTCAAACCTTAACGGACTGTACTTGAAAGGAGCTCATGAAAATAAGGATGGAGTAATATGGGGCACCTTTAGAGGACACCGCATATCTCTGAAGGATACTACCATAAAGATTCGCCCTAGAGATTTCAAGAAGAATTCAGATGCTACTACACCGCATTAACTGCTTTCCATCCCTAGTAGCTCGACTGAAAACAACATTTTCTTTCAAAACTCGATTGCAACAATGATTTCCATTAGTGAAAAATCAACAACTACCGTTAAATTTCAGCATTCAGCAAATTCTATCAAATGGAACAGAATGGTATCATCA

							TAAAAATTCTTATTTTGCTCTGATATATATATATATATATGAC<br>ACATTTATATATATGTATGTGTGTGTGTGTGTGTGAAAGAG<br>AGAGAGAGAGAGAGAGAGTAATAAAATATTAAAAAGACTT<br>AGACTTTAGTTTAAATTAATATTCTTATTTCATATCTGGTTTAC<br>ACATCACAATTACAGACTATGAACAAAATTTTCTAGACTTAC<br>ATCCATACCTTAATCGCTTAATCGATATCGAGCAACTGACTG<br>ATTTTACAGCAGTTACAATCCTTTCTGAAAGACTTTAAGACA<br>CCTTATCGGATAAAGACATCTTAAACACCACGCCGGAGAAG<br>ATGCATGAGAATGGACATTATTGGACTTTTGAAACGGAGGTT<br>TTAAGTGAAACTTACTTTTTTGGGAAGCAACAGATACATCAT<br>CCATAACAGCAACTGTTACTTTCATCAATTTTTGGAACTAGTG<br>GGTGATTTCACGCATTCCATTTTAGTTGTTCAGCTTTTGACCGT<br>AAAATAAATCTGTGGTGTTGTTGCTATCAGTGACTTTGCTAGC<br>ATATAAATTTTTGGATGAATAATTATATATGAAAAATTTTTTC<br>TGACCACTCTTAGGTTAATTTGAAAGCAAAAGCATGGGACAC<br>ATACACATACTACAAGGTACTGATAAAATGCCGTATCAATTG<br>GATGAACAAGGTATTGTCTTAGTATATTAGAAAGAATTCATA<br>CTGTTAAGAACAAAACTAATATTAATATCCAAACTACAGGTA<br>GAGCTTATCGGTAAATTTAAAAGTAAAAGCACGTTTACTTTT<br>ACCCTGTCAACAGGAATTTTAGCCTGTAAAACTAGACAAATG<br>CTCTCTGATTATGAATTGATATGTTCCACTATACTTTGAGAAA<br>AGCATTTATTCATAGTTTACAATTGTAATGCATATATGAATTA<br>ATAAATGAAATTTTCATTTAAAGTAATTCTGTTGCACTTTATA<br>CATCACATATACGTTGTAAAAATTTAGAAAGTACTTCACTAA<br>TTCTCCTTAATGTGCATTATACTTTTTAATTTGAACTCATTCCC<br>TTTTTGACATAACAATGATCTGTAGGTTTTCACTCAGAGATAA<br>ACTGGTGGGTGCTTGCATGCTTTCAGTGCTCATCTGGTACTAA<br>GGGGTGTGGAATCTATAAATGCTACACAAAACAATGGAGCC

							TTCATTTCTGTGAATAGGGTCTGAACTCGCCATCTATACACTTTACTCTTTACAGCAAAAGATTCCAGTTGTGTTTTATTTGAGGATGATACACGCCTTTTAGTTATCCTGTACATTATGCTATCTATCTCCAATAAATCATGTTAGGTTCATGTAACTGCAAAGTTTCTTTGCTTAAAATACTTTTATATTTCCTGAGGACGTGATACTGTTTAGACTCTTAATGGACTATATTGCCTTGCTGTTCATCTTTACAAATCTAAAATTTTCCCAAGCCCTTTTTGTAGCCTCCACAGACTTTCCAAATTGATGGACAGATGCCACCTTCTAATTTGCTCTGCCCAGAATGGGACAACTATATCAGTTTGTTACTGGGAAGCTCAACATCTGCTTATGGGTATATCTAATGGCTAATGCAAATTTCTTTCTGCTCGCATATACCCTTTTGAGCCATATTGGAAATATATACACACACATACATAAAATCCTCTATATTTTTACTCAGAAATATATATATTCCTGCATAAAAATAAAATTATTCAATTTAAAAAAATTAAGTCACTTACTATCCGAAACAATTTATGAATACATTCATATACATACATGCCTGTGCACAATGTACAATCAGAAATTAGAACTACATAAGCATATTCCAAGCATATCATACCATATATTTTCATAACTGCAACCTAAGACAATATAAATAAAAAAAAAAATTCACATTTTTATACAAACTTCCACAAATTCTTTTTAAAAATGTATTGATAAAAATCTATATTGAA

PhdFibTec73	P85031	9,11E-11	36,145	PF00147	13446.23 **11318.64**	MARSSVLQVLLCLCYACSNA**TSNCEQEISTSYLDAAVDMITKAKSNIPTCVNQRNTFSHGALDCSDLFQRGSNKSGVYKIQPVNRIFDDKSLEVYCDMETNGGGWTLIQRRGKFERTPYR**	CGATTTCTGATATAAGTAATTAAGTTCTTTCTAAACACCAACATACTTTTGAATAATCTGTGAATATCGTCAGTGTAAAATGGCCAGATCCTCTGTACTGCAGGTTCTGCTCTGCCTTTGCTACGCATGTAGCAATGCGACTTCCAATTGTGAGCAGGAAATTTCCACGTCTTACTTAGATGCAGCGGTTGATATGATTACTAAAGCCAAAAGTAATATTCCAACTTGTGTTAACCAAAGAAACACTTTCTCACATGGAGCCTTAGATTGCAGTGACTTATTTCAAAGGGGTTCTAACAAAAGTGGTGTTTACAAAATACAGCCTGTCAATAGAATATTTGATGATAAATCACTGGAAGTATACTGTGATATGGAAACAAACGGAGGAGGATGGACTTTGATACAAAGAAGGGGAAAGTTTGAGAGAACGCCTTACAGAG
PhdFibTec74	P85031	5,50E-27	51,485	PF00147	13253,61	WIDDENHLYTLHIADYSGTAGDSLTYHNNMNFSTRDQDNDRDKDGSCAEAYKGAWWYKNCVSSNLNGMYKKGENITHGVSWFRWKNYYESLESTEIKIRPKKLNFPHVPLNKP	AGCGGTTTGTTTAACGGAACATGTGGGAAATTAAGCTTTTTGGGACGAATCTTTATTTCAGTTGATTCGAGAGATTCGTAGTAGTTTTTCCACCTAAACCAGCTTACTCCATGAGTAATATTTTCCCCTTTTTTGTACATTCCATTTAAATTGGACGATACACAATTTTTGTACCACCATGCTCCTTTATAAGCTTCGGCGCAGCTTCCATCTTTATCCCTGTCATTATCTTGGTCTCTGGTCGAAAAATTCATATTGTTATGATACGTAAGAGAGTCACCTGCAGTTCCGCTATAATCAGCAATGTGCAGAGTGTACAAGTGATTTTCATCGTCAATCCAG
PhdFibTec75	P85031	5,82E-10	36,364	PF00147	9984,23	EISSCEQSVTLSYLNLAVEIITKAKDNIPTCNKCGYNLTRGALDCSDLFQMGYNKSGVYTLWPTSRTTEDKSIEVYCDMQTNGGGWTVIQ	GAAATTTCTAGTTGTGAGCAGAGTGTAACTCTGTCCTATTTAAATTTGGCTGTGGAAATTATCACAAAAGCGAAAGATAATATTCCAACCTGTAATAAATGTGGATACAATCTGACTCGGGGAGCCCTAGATTGTAGCGACTTGTTCCAGATGGGTTATAACAAAAGTGGCGTGTACACACTATGGCCTACGAGTAGAACAACAGAGGATAAATCAATAGAAGTGTACTGTGATATGCAAACGAATGGAGGAGGATGGACTGTTATCCAAA

PhdFibTec7<br>6	P85031	7,52E-30	56,989	PF00147	12192,29	TLHIADYSGDAGDS<br>LQPHNNQKFTTRDQ<br>DNDNHKDVNCAQ<br>NHKGGWWYGACH<br>TSNLNGLYLSGKHES<br>YADGINWLGWKGY<br>HESMETTEIKIRPKN<br>FRMPNTSKDTPL*	ACACCCTCCACATTGCTGATTATAGCGGAGATGCAGGTGACT<br>CATTGCAGCCGCATAACAATCAGAAATTTACCACAAGAGAC<br>CAGGATAATGACAACCATAAAGATGTAAATTGTGCGCAAAA<br>TCATAAAGGAGGATGGTGGTACGGCGCTTGCCACACGTCCA<br>ATCTGAATGGTCTCTACCTCAGTGGGAAACATGAAAGTTATG<br>CTGACGGAATAAACTGGCTTGGATGGAAAGGCTACCACGAA<br>TCAATGGAAACCACAGAAATAAAGATTCGGCCAAAAAATTT<br>CAGGATGCCAAATACTAGCAAGGACACACCTTTGTGAATGT<br>GATTCTATGATAAGAGTTCATGACAATCAGAAGTTTAGTACG<br>AAAGACCGTGATAATGACATCAGTAATGATAAAAATTGTGC<br>CGAACAATATAAAGGAG
PhdFibTec7<br>7	P85031	1,49E-29	52,000	PF00147	429,19	MEDAGGAWTVIQR<br>RGDFSSGEDYFYRG<br>WQSYKHGFGDLNK<br>DFWLGNENIFALTN<br>QRANAVKFTLSDWE<br>GNTTYATFDDFWID<br>DESENYK	TTTGTAATTCTCAGATTCGTCATCAATCCAGAAATCGTCGAAT<br>GTAGCATAAGTTGTATTTCCTTCCCAGTCACTCAAGGTAAATT<br>TCACAGCATTCGCCCTTTGATTGGTCAGCGCGAAGATGTTCTC<br>ATTGCCTAGCCAGAAATCCTTATTCAGATCTCCGAAGCCATG<br>TTTATAAGACTGCCAACCTCTATAAAAATAATCTTCTCCGCTG<br>CTAAAATCTCCTCGTCTTTGAATTACTGTCCAAGCTCCTCCAG<br>CGTCCTCCATATCGCACTACACTTCGATCGAAGCAGTCATCA<br>CCCGACTGCG
PhdFibTec7<br>8	P85031	1,84E-33	46,825	PF00147	17504.86<br><br>**16236.24**	MILLAILTAVASA**VS**<br>**LDTPEFSAPDCNTK**<br>**GKVSLELDLAIQLIN**<br>**KAKKSIPNWQNTD**<br>**LPMDCGDIHRSGN**<br>**NVSGVYEVWPRNR**<br>**IITGPISVWCDMET**<br>**DGGGWTLIQRRGN**<br>**NLRPKDYFQRNWE**	GTCGAAAAGGGCTCAAATAATTTGGAGACCAGAAGAAAGAT<br>GATTCTCCTCGCAATTTTGACTGCAGTTGCATCTGCTGTCTCTT<br>TGGACACCCCTGAGTTTTCGGCTCCGGACTGTAACACCAAAG<br>GAAAGGTGAGTTTGGAACTGGATTTGGCTATACAGCTAATTA<br>ACAAAGCAAAGAAAAGTATTCCAAATTGGCAGAACACAGAT<br>CTTCCAATGGATTGCGGAGATATCCATCGGAGTGGAAATAAC<br>GTCAGTGGTGTGTATGAAGTTTGGCCCAGAAACAGAATAATA<br>ACTGGACCTATTTCCGTTTGGTGTGATATGGAAACTGATGGA<br>GGAGGATGGACGTTAATTCAACGTCGTGGTAACAATCTTCGT

						**<u>PYKKGFGDVKEDF WLGNDNIYALSNQ KPYSVR</u>**	CCAAAAGATTATTTCCAAAGAAATTGGGAACCATATAAAAAAGGATTTGGCGACGTAAAAGAAGATTTCTGGCTAGGAAACGACAATATATATGCCTTGAGTAACCAAAAGCCGTATTCCGTTCGATT

<u>*GROWTH FACTORS*</u>

Cysteine rich growth factor

Transcript ID	Uniprot ID reference protein	e-value	Identity (%)	PFAM domain	MW (Da)	Amino acid sequence	Nucleotide sequence
PhdGrFCys 01	A6MFK8	4,68E-10	40,278	PF00008<br>PF00594<br>PF00089	9166,12	PGFVGSLCQANVDDCLTKPCSNGGTCHDLINDYRCDCMPGFAGKDCSVNIDECESNPCFNGGSCEDLVNEFVCHCADGFQGKQCQN	TATTCTGGCACTGTTTGCCTTGAAATCCATCAGCACAGTGACACACAAATTCGTTAACGAGGTCTTCACACGAACCACCGTTGAAGCACGGGTTGGATTCGCACTCATCAATGTTTACGGAGCAGTCTTTTCCCGCAAAGCCAGGCATGCAGTCGCAGCGATAGTCATTGATGAGGTCGTGGCATGTACCACCATTGGAACACGGTTTGGTGAGGCAGTCATCCACATTTGCTTGACACAGAGAGCCAACGAAGCCCGG
PhdGrFCys 02	A6MFK8	2,36E-06	50,000	PF00008<br>PF00594<br>PF00089	9674,94	YSCSCRPGFTGVKCEKNIDDCRPNPCRNGGTCHDLVNNFVCSCPHGTQGILCEINIDECFEGACHHGGTCVDRVGSYECLCPPGFVGPRC	ATATTCTTGCTCGTGTCGCCCTGGATTCACAGGTGTGAAATGCGAGAAGAACATCGACGACTGCAGACCCAACCCCTGCAGAAATGGGGGGACGTGCCACGACTTGGTCAACAATTTCGTCTGCTCCTGCCCCCACGGGACGCAGGGTATATTGTGCGAGATAAACATCGATGAGTGTTTCGAAGGGGCCTGTCACCACGGCGGGACCTGCGTCGACAGAGTTGGAAGTTATGAATGCCTCTGCCCTCCCGGATTTGTGGGTCCTAGGTGCG

PhdGrFCys 03	Q58L93	3,30E-07	38,000	PF00008 PF00594 PF00089	10713,99	DCKPNPCFNGGHCIDGINWFMCECTPGFTGPDCRINVNECASSPCAEGSTCKDDINDFQCICPPDKTGKLCDTDIKKTCEFQGIKQLEGSQWEYECN	GACTGCAAGCCTAATCCTTGTTTTAATGGCGGCCATTGCATTGATGGAATAAACTGGTTTATGTGTGAATGCACACCAGGTTTTACTGGACCAGACTGCAGGATAAATGTTAACGAATGTGCATCTTCACCTTGTGCAGAAGGCTCAACGTGCAAAGATGATATTAATGATTTCCAGTGCATTTGTCCTCCTGACAAAACAGGAAAACTTTGTGATACTGATATTAAAAAGACATGTGAATTTCAAGGTATCAAACAACTTGAAGGAAGCCAGTGGGAATATGAATGTAAT
PhdGrFCys 04	A6MFK8	2,31E-09	38,356	PF00008 PF00594 PF00089	9563,65	GIHCDIEFNECLSMPCKNDAVCNDLVNNYSCTCLPGFAGRNCETDINECASNPCLNGATCYDQINKYTCSCVEGFAGRNCEINIDECA	TGCACATTCATCTATGTTTATTTCACAATTTCTTCCGGCAAATCCTTCAACACAGGAACATGTATACTTATTAATTTGGTCATAACATGTAGCTCCATTAAGGCATGGATTTGAAGCACATTCATTGATATCAGTTTCACAGTTACGGCCAGCAAATCCTGGTAAACATGTGCAACTATAGTTATTAACTAAATCATTACAGACAGCATCATTTTTGCAAGGCATTGAAAGGCACTCATTAAATTCAATATCACAGTGAATTCCTG

PhdGrFCys 05	Q1L658	2,42E-05	36,364	PF00008 PF00594 PF00089	154288,5 2	LRQGQCFLDVTAEY NGRGICSTELGERLS KVTCCCSLGRGWGP TCEVCPRVNTTEYN QLCPGGTGFRPNQIT VVLEDIDECREFNN VCKGGRCSNTFGSFT CICPDGYTLDSTLRN CVDVNECHIRPGIC GPGTCLNSEGGYSCI CPPGFVQISGGCVD MRKSLCFRQAYNTT TRFPRLVCEHPMMS NETKLTCCCSVGRA WGDPCEPCPSANSE EYMKLCTPTPGTYV NPMTGSVDDVDEC KTGVCENGYCTNTI GSFVCECYNGFRFNS FINKCEDINECIESPG VCQGAASCINNIGSF ECRCPDGYKLMPNE RECLDVNECAKTGM CDNGVCKNLDGSFI CTCNNGFYLSPSGE HCLDVDECVRSPGIC SDGICTNVFGSYRCD CNPGYQLSPNGDCF	AATTGCACTGAGCTTTCAAATAAAATCTAATCTGTATTACAT GGTTATATGCACATTTCATTTTTTTTTTTCAGTGTAATATTTGC AAAATTCCATTTTAGTCTAAGGAAATAAATTCTTTTCTCCAAT TCATATTTCTCAGAAGGCAGTGTTACAGAACTCTATGTTTGTT GAAATCTCACTTAGTAATTATTAGGTGCACTGTTAAATAAAA TGGTTTAGTGTCAAAAGTACTAAGTTTAGGTGTCACATTTTCA ACTGTTGCTCTAATTTTCAGAATGTATGTTCCTGGTTGTCGTA CTCTATCTCTTGTGTGTAATACAGCAATACCTTGCCTGTTCGA TATTTCAAAGGTTCTATCTTTGTTTCCTTCAATTATGTGGAATT TTTCATTATTTCTTAAATAAGTCACAGATGGTTGTATCTTGAG CAAATCAACAGCAGGATGCATTTTGTGCAGTGGTACAAATAC TGTAACATGTAATTCTCCTTTTCCTGTATCTGTAATATTAGAT GTTTTCATCTGAGAATGTAAATGACGATGATGTCTTGATTGCC CATATGCCAGCCTGTTGGATTGTTTCTTTTCTGTTCTTCTTATG GCATGATGTGTTGACTTGCTTGCTTTTTGTTGCCTTCTAGTAAT AATGTTCATCCTTGTAACATTAGATATATCTCGCCTTTGACGG TTATTACTGTTTAATTTACAAGAGTAACATCCCTCAGTGGAA ATTATTTTCTCTCCAGAGACAGAGTAACTTGTTTCACTCGGAA TATGCAAATTACCAAGAGGTAATAAGCTGTTGTCTAAGTTTC CTCCACTGGGTACCAAATTTCCTAGACTTGAACCCAAGTTTC CTAAATTAGTTCCAAGTATCTGTCCATTTCCATAGGATGTTGA AGAAATGGTAGACAAACAATGCCCTTGACCAAGTCTCTGGT ATCCATCAGGGCAACCACAAGAATAGCCAGGTGGGCCTGAA GGGGTGCAACCATAACTGCAGGGTGAATTCTGACATGATGC ACTTGCACCTAATTCTATGCAGATCATATGATTGCCATCAAA TACATATCCTGGTGGACAGCTGCATGAATAGCTTCCAACCGT ATTTTCACAGCTTGCAGAACCACAGACAGAGCCTGAAGCAC ATTCATTCTCATCAATACACTGATTCCAATAGTAATGTCTTCT

						DIDECRTQYGICRNG<br>RCRNLDGGFTCQCQ<br>NGYVLSEDGRNCIDI<br>NECLEDDICIDGVCR<br>NSEGSFQCTCNDGY<br>RLSADRRRCEDVDE<br>CRTIPSICDGGSCTN<br>QKGSYVCSCPKGFRL<br>SFDGKECIDIRQNFC<br>FQEYKNGICRSPKSS<br>NMTRKECCCMKGS<br>VAWGSEPCERCPDQ<br>RSEQFRKLCPDGFGY<br>VITNGVIEDINECM<br>MDPTLCENGICINTD<br>GNFRCECQDGFKID<br>STGTKCIDMDECRE<br>MGPVCGNGTCSNV<br>MGGFECDCRDGFTA<br>GPSQTCEDINECDE<br>MGSLCAFRCQNTPG<br>SFKCTCPYGYTLAA<br>DGRHCEDLDECSTP<br>ANDCRFDCKNLIGS<br>FMCICPDGYEQVGM<br>VDDCQDINECRTDP<br>GICGNGRCINTRGSY<br>RCECFDGFEPSRTQK	AAAACCTTCAGGACATGCACAACGATAACCACCAATCATAT<br>TTTCACAGCCATGCTGACAACGTGCATCATCCAAGCATTCAT<br>TAGTATCCATGCAGAATCTTCCAGAAGGATCCAAGCTGAAA<br>CCTCTCTGACAAAGACACTTGAAACTTCCAGGCATATTCATA<br>CAAGTACCCATAGGAGTGCACAGATGAGGCTCTTCGGTGCAT<br>TCATTTACATCTAAGCACTGCCCTCCAATCTGATTAAATCCTT<br>TCTGACAAGAACACTGATAACTTCCTTGAGTATTTACACATT<br>GATTTTGACAGTCATGTTGTCCTGTTTTACACTCATCAATATC<br>TCTGCATGTAATTCCATCAGGTGCTAAAATATACCCCTTAGG<br>GCAAGAGCATCTAAAACTACCTTCTGTATTTTCACAAGAAAA<br>CTTACAGGGTGCAGGTGTAAGTTCACATTCATTAACATCAAA<br>ACATCTAGTTCCAGATTCATCTGGTTTATATCCTTTGTTACAA<br>ATGCATCTGTAAGAGCCCATTGTATTGATACAGCGTCCATTTT<br>GACAAAGATTATTCATTAAAATACATTCATCCACATCTCTTC<br>CATCTTTATCAAATCCAACTCCATGTGGACAAAGATCATTAT<br>ATTCTTTACTGCCTTTCCGAGGACATAATTCACACCGGGAGC<br>CCCATGCCACACCCATACCACAGCAACAGTCAATTTTTGTAA<br>TTGCAGTAATGTCTCGTGACTGTAGAGAACATGATGGAACAC<br>TGAAGCAAAACCCAGTGCGGATATCTATGCACTCTTTCTGTG<br>TACGTGATGGTTCAAAGCCATCAAAACATTCACAGCGATATG<br>AACCTCTCGTGTTAATGCATCTCCCATTACCACAAATTCCAG<br>GATCAGTTCGGCATTCATTAATATCTTGGCAATCATCTACCAT<br>GCCAACTTGTTCATAACCATCAGGACAAATGCACATAAAAG<br>ATCCAATAAGGTTTTTGCAGTCAAATCGACAATCATTAGCTG<br>GTGTACTACATTCATCTAAATCTTCACAATGTCTTCCATCTGC<br>TGCTAATGTGTAACCATATGGACATGTACATTTAAAACTCCC<br>CGGCGTATTTTGACATCGAAATGCACATAAAGAACCCATTTC<br>ATCACATTCATTGATATCTTCACATGTTTGACTTGGTCCAGCT

						ECIDIRTGFCFSVPSC	GTGAAACCATCTCTGCAATCACATTCAAATCCACCCATGACA
						SLQSRDITAITKIDCC	TTAGAACAAGTTCCATTGCCACAAACAGGACCCATTTCCCTG
						CGMGVAWGSRCEL	CATTCATCCATGTCAATGCATTTAGTACCAGTAGAATCAATTT
						CPRKGSKEYNDLCP	TAAAACCATCTTGACATTCACAGCGGAAGTTACCATCTGTGT
						HGVGFDKDGRDVD	TTATACATATACCATTCTCACAAAGTGTTGGATCCATCATAC
						ECILMNNLCQNGRC	ACTCATTGATATCTTCGATAACTCCATTTGTAATGACGTATCC
						INTMGSYRCICNKG	AAAACCATCTGGACACAATTTTCTAAATTGTTCTGAACGTTG
						YKPDESGTRCFDVN	ATCTGGGCATCGTTCACATGGTTCAGATCCCCAGGCTACCGA
						ECELTPAPCKFSCEN	ACCTTTCATACAACAACATTCCTTCCGTGTCATGTTAGAAGA
						TEGSFRCSCPKGYIL	CTTAGGACTTCTACAAATGCCATTTTTATATTCTTGAAAACAA
						APDGITCRDIDECKT	AAGTTTTGTCTAATATCTATACATTCTTTGCCATCAAATGATA
						GQHDCQNQCVNTQ	GTCTAAATCCCTTAGGACATGAGCACACATAGCTTCCTTTTTG
						GSYQCSCQKGFNQI	ATTGGTGCAAGAACCGCCATCACATATTGAAGGAATAGTTCG
						GGQCLDVNECTEEP	ACATTCATCCACATCTTCACAACGCCTTCTATCTGCAGATAAT
						HLCTPMGTCMNMP	CGATATCCATCATTGCAAGTACATTGGAATGAGCCTTCAGAA
						GSFKCLCQRGFSLDP	TTTCTGCATACTCCATCAATACAAATATCATCTTCTAGGCATT
						SGRFCMDTNECLDD	CATTTATATCAATACAATTACGTCCATCTTCACTGAGAACAT
						ARCQHGCENMIGG	AGCCATTTTGGCACTGGCAAGTGAAACCTCCATCTAAATTTC
						YRCACPEGFRRHYY	TACAGCGTCCATTCCTACATATGCCATATTGTGTTCTACATTC
						WNQCIDENECASGS	ATCAATATCAAAACAATCTCCATTTGGTGATAACTGATAACC
						VCGSASCENTVGSYS	TGGATTACAATCGCAGCGGTAGGATCCAAAGACATTTGTGCA
						CSCPPGYVFDGNHM	AATACCATCAGAACAAATACCAGGGCTACGAACACATTCAT
						ICIELGASASCQNSP	CAACATCAAGACAATGTTCACCAGAGGGAGACAAATAAAAT
						CSYGCTPSGPPGYSC	CCATTATTGCAAGTACATATAAAGCTCCCATCCAGGTTTTTAC
						GCPDGYQRLGQGH	AGACACCATTATCACACATTCCAGTTTTTGCACATTCATTTAC
						CLSTISSTSYGNGQIL	ATCTAAACATTCTCTTTCATTAGGCATAAGCTTATAACCATCT
						GTNLGNLGSSLGNL	GGGCAGCGGCATTCAAAGCTCCCAATGTTATTGATACAACTA
						VPSGGNLDNSLLPL	GCAGCACCTTGACAAACCCCGGGAGATTCAATGCATTCATTT

						GNLHIPSETSYSVSGE<br>KIISTEGCYSCKLNSN<br>NRQRRDISNVTRMN<br>IITRRQQKASKSTHH<br>AIRRTEKKQSNRLAY<br>GQSRHHRHLHSQM<br>KTSNITDTGKGELHV<br>TVFVPLHKMHPAVD<br>LLKIQPSVTYLRNNE<br>KFHIIEGNKDRTFEIS<br>NRQGIAVLHTRDRV<br>RQPGTYILKIRATVE<br>NVTPKLSTFDTKPFY<br>LTVHLIITK*	ATATCTTCACATTTATTAATAAAGGAATTAAATCTAAATCCA<br>TTATAACATTCACAAACAAAACTTCCAATAGTGTTGGTACAA<br>TAGCCATTTTCACAAACTCCAGTTTTGCATTCATCCACATCAT<br>CAACAGAACCTGTCATAGGATTAACATACGTTCCTGGTGTGG<br>GAGTACATAATTTCATATATTCCTCAGAGTTAGCAGAAGGAC<br>ATGGTTCACAAGGATCACCCCACGCACGACCAACAGAGCAA<br>CAGCACGTAAGTTTTGTTTCATTGGACATCATAGGATGCTCA<br>CACACAAGGCGAGGAAATCTTGTTGTTGTGTTGTAAGCCTGC<br>CTGAAGCAAAGAGATTTTCTCATATCGACACAACCGCCTGAA<br>ATTTGTACAAAACCAGGTGGGCAGATACAGGAATAGCCACC<br>CTCAGAATTTAGACATGTACCTGGTCCACAAATACCTGGTCT<br>AATATGGCATTCATTCACATCAACGCAGTTCCTAAGTGTAGA<br>ATCTAAAGTATAACCATCAGGGCAGATACATGTAAAACTAC<br>CAAATGTGTTAGAACAACGTCCTCCTTTACACACATTGTTGA<br>ATTCTCTGCATTCATCAATGTCTTCCAAAACAACAGTTATCTG<br>ATTGGGTCTGAAACCTGTACCACCAGGGCATAGTTGATTATA<br>TTCAGTTGTGTTTACACGTGGACAAACCTCACATGTAGGACC<br>CCAGCCTCTTCCAAGAGAGCAACAGCAAGTAACTTTTGAAA<br>GTCTTTCGCCTAATTCTGTACTACATATGCCTCTACCATTATA<br>TTCAGCAGTTACATCAAGAAAACATTGACCTTGTCTAAG

PhdGrFCys 06	Q56VR3	2,22E-05	30,769	PF00008<br><br>PF00594<br><br>PF00089	132752,2 1	PELLDRATRDIRNHF STAGSFQPRSLFIVT WDDVGYYDRATDK VNTFQVVLASDGED TYAYFLYPSGGVQWI QGKGKNPNLPDAR AQAGFMSGDGRLYT LRGSGTEQARNFDR MSNARKAGVWMFH IGRPFSAEGNVLPAD LESSGGSEDRQDGQS GSTCADTLLPCPPHS TCVDHADRRGFCCL CKDGYFGNGRNCV EKNSPQRLNGKVSG VINEVQLSDADMHA YIVTEDGRTYTAVSR VPPAVGSDLQVLTPL GGVVGWLFAVSRSG APNGFMITGGAFNR TVEVDFPQSGHHVYI EEAFLGPDVFNYMR VQVKVRGSVPSVPL GSKIEIPDYEEEYTRIS PGLIRSHASRFYRLE GNNLEVPFKIDQTIM YTECEVPMSSTRLKV ARNFIVYDAQEQIVR	TCCAGAACTGCTGGATCGAGCTACTAGAGATATCAGGAATC ACTTTTCCACCGCTGGATCATTTCAACCTCGTAGCCTCTTCAT AGTGACATGGGACGATGTTGGATATTATGATAGAGCCACAG ACAAGGTTAACACTTTTCAAGTTGTGCTTGCTTCAGACGGGG AAGACACTTATGCTTATTTTCTATACCCATCTGGAGGCGTTCA GTGGATTCAAGGTAAAGGTAAGAACCCCAACTTGCCAGATG CTAGAGCTCAAGCTGGTTTTATGTCCGGCGATGGAAGACTGT ACACACTCAGAGGATCAGGAACAGAACAAGCAAGAAATTTT GATAGGATGTCAAACGCTAGGAAAGCTGGAGTATGGATGTT CCATATTGGACGTCCTTTCAGTGCTGAGGGTAATGTCTTGCCA GCAGATTTGGAGTCATCTGGTGGATCGGAGGATCGCCAAGA CGGACAATCAGGGTCTACATGTGCAGATACATTATTGCCATG CCCTCCACATTCAACGTGCGTTGACCATGCCGACAGAAGAG GATTTTGCTGCCTATGCAAGGATGGATACTTTGGAAATGGAC GAAACTGCGTGGAGAAAAACTCTCCTCAGCGATTGAATGGC AAAGTGAGTGGTGTCATCAATGAAGTTCAACTGTCGGATGCT GATATGCATGCATACATTGTAACCGAAGACGGAAGAACATA TACAGCTGTGAGTCGTGTACCACCAGCAGTTGGCTCAGATTT ACAAGTTTTAACACCACTTGGTGGAGTCGTTGGGTGGTTGTTT GCCGTTTCGAGAAGTGGAGCACCTAACGGTTTCATGATAACA GGTGGCGCATTTAACCGTACTGTAGAAGTAGATTTTCCTCAA TCGGGACACCATGTGTACATCGAAGAAGCATTCCTTGGTCCA GATGTCTTTAATTACATGAGAGTGCAAGTAAAAGTTCGAGGC AGCGTGCCATCCGTACCTCTTGGATCCAAGATCGAAATACCA GATTATGAAGAGGAATACACAAGGATATCCCCAGGTCTAAT CCGATCCCATGCTTCCCGTTTCTATCGTCTGGAAGGGAATAA CCTGGAAGTACCTTTCAAAATAGACCAGACCATAATGTATAC TGAGTGTGAAGTACCAATGTCCAGCACTCGACTGAAAGTGG

						YAMTNKISPLTVEDP<br>CKEGQHQCGPHSN<br>CVVEGDSFRCACAQ<br>GYQDIYDENSAHER<br>GVNCIDMNECTIGR<br>HNCHVYAECLNLP<br>GSFTCRCLAGYIGDG<br>VNCERKRTCDDVNC<br>DPNADCVERNPQR<br>GPECQCRPGFVGNG<br>ATCEPGTEDDCSLIN<br>YCDTRAECVFNDQH<br>QRHECRCRSGYTGD<br>GVLCVVDSCDVAN<br>NCSPYGTCLFDTEGE<br>VYYCACNPGFSGDG<br>YHCVPEGATCDVID<br>HCHPNAQCLFDPQ<br>AQRYQCRCSVGYRG<br>DGRTCVRTEITCDQ<br>ANICSPQAACVRDE<br>LAGRYVCRCYEGFE<br>GDGETCTPVDECNT<br>AENCDENAQCLYDS<br>TSQRYRCQCLPGFR<br>GTGGRGGCVPDPDA<br>SCNIRNNCHQLATC<br>AYDPTDLVYRCQCN	CTCGTAATTTCATTGTGTATGATGCACAGGAGCAGATCGTTC<br>GATATGCCATGACGAACAAGATATCTCCACTAACCGTGGAA<br>GATCCGTGCAAGGAAGGCCAACACCAGTGTGGACCTCACAG<br>CAACTGTGTGGTAGAAGGGGATTCTTTCCGCTGTGCCTGCGC<br>CCAGGGATACCAGGATATCTATGATGAAAATTCAGCCCACG<br>AACGTGGCGTCAACTGCATTGATATGAACGAATGTACTATCG<br>GTCGTCACAACTGCCATGTTTATGCAGAATGTCTTAATCTTCC<br>TGGATCCTTCACATGCCGTTGTCTGGCTGGATATATCGGAGA<br>TGGAGTCAACTGTGAACGCAAAAGGACATGCGATGACGTCA<br>ACTGCGACCCAAATGCTGACTGCGTTGAAAGAAACCCTCAG<br>CGAGGACCCGAGTGCCAGTGTAGACCTGGTTTCGTGGGTAAC<br>GGCGCCACCTGTGAACCCGGTACAGAGGATGACTGCAGTCT<br>CATCAACTACTGTGATACGAGAGCGGAGTGTGTGTTCAACGA<br>CCAGCATCAAAGACACGAGTGCCGCTGTAGATCTGGCTACA<br>CCGGTGATGGTGTTTTGTGTGTAGTCGATTCCTGCGACGTAGC<br>CAACAATTGCAGCCCGTACGGCACCTGTCTCTTTGATACCGA<br>AGGAGAAGTTTACTATTGTGCATGTAACCCAGGATTTTCTGG<br>CGATGGATATCACTGCGTCCCGGAAGGAGCTACTTGTGATGT<br>TATCGACCACTGCCATCCCAATGCTCAATGTCTCTTCGATCCA<br>CAAGCTCAGAGGTACCAGTGTCGATGTAGTGTTGGATACCGA<br>GGCGACGGCAGGACGTGTGTAAGAACCGAAATAACGTGCGA<br>TCAAGCCAACATATGCTCTCCTCAAGCTGCGTGTGTAAGGGA<br>TGAACTTGCTGGAAGATATGTCTGTCGTTGCTATGAGGGATT<br>CGAAGGTGATGGTGAAACTTGTACGCCTGTAGACGAATGCA<br>ATACGGCAGAAAACTGCGACGAGAACGCTCAGTGCCTGTAT<br>GACTCCACGAGTCAGAGATACAGATGTCAGTGCTTGCCAGG<br>ATTCCGAGGTACAGGTGGAAGAGGTGGATGCGTACCAGATC<br>CAGATGCTTCTTGCAATATTCGCAATAATTGCCACCAGCTGG

						PGYQGDGYNCEKTA<br>IPCNVINTCHVRAEC<br>IYDANSREYVCRCIS<br>GYEGDGNECRPLRS<br>CREDFYQCDRNADC<br>LPDLASGDYACQCK<br>QGYLGDGITCIVAPQ<br>HSGGYLVFAHGMSL<br>LRVPTTPTKNNPGQ<br>LLLMEPNQTPVGLA<br>TDCQQGHLYWADA<br>SLKVIRRANYNGSD<br>VTMIISSDMLSPEGIA<br>VDWLGRTIFWTDSG<br>KDTIEVASLSKHYRK<br>VLISEGLDNPRGIAV<br>HPGLGKMYWTDWS<br>RNAPKIEMANMDG<br>TGRKELVSDNLALP<br>NMVVIDFNRNNLC<br>WTDSGLGRIECIGLN<br>GQGRRIVYTPAAYPF<br>GITIHESYIYWTDWEI<br>KFLHRVDVNGGEAE<br>PLEIPAGGSGRMYGL<br>VSLPSFCPSVGSPCA<br>VENGGCRYLCLPDG	CCACTTGCGCGTATGATCCTACAGATCTGGTTTACAGATGTC<br>AGTGCAATCCAGGATACCAAGGTGACGGTTATAACTGCGAA<br>AAGACTGCAATTCCTTGCAATGTTATCAACACCTGTCATGTC<br>AGAGCTGAATGTATCTACGATGCTAACTCCAGGGAATATGTG<br>TGCCGGTGCATATCTGGTTACGAGGGAGATGGCAACGAATGT<br>CGCCCTCTGAGGTCATGCCGAGAAGATTTTTACCAGTGTGAC<br>AGAAATGCTGATTGCCTTCCGGATCTCGCAAGCGGAGATTAT<br>GCTTGTCAGTGTAAGCAAGGATATCTAGGCGATGGTATTACC<br>TGCATTGTTGCCCCTCAGCATAGTGGTGGATATCTAGTCTTTG<br>CCCATGGAATGTCTCTCCTTCGTGTACCTACTACACCCACAA<br>AGAATAATCCAGGTCAATTACTTTTAATGGAACCTAACCAGA<br>CCCCTGTTGGACTAGCCACAGACTGTCAGCAAGGTCATCTAT<br>ACTGGGCTGACGCTTCGCTAAAAGTAATTCGAAGAGCCAAC<br>TACAATGGCTCCGATGTCACCATGATTATTTCTAGTGATATGC<br>TTTCACCTGAAGGAATTGCTGTCGATTGGTTGGGCAGAACAA<br>TATTCTGGACTGATTCAGGAAAGGATACGATAGAGGTTGCTA<br>GTCTTTCCAAGCATTACAGAAAAGTCCTAATTTCCGAAGGTC<br>TAGACAATCCTAGAGGCATTGCTGTACATCCAGGATTGGGAA<br>AGATGTACTGGACAGATTGGAGTCGAAATGCACCCAAGATT<br>GAAATGGCTAACATGGATGGAACAGGCAGAAAAGAACTGG<br>TTTCAGATAATCTCGCATTACCCAATATGGTAGTCATAGACTT<br>CAACAGGAATAATTTGTGCTGGACAGATTCAGGTCTTGGAAG<br>AATTGAATGTATTGGCCTCAACGGACAAGGTAGAAGAATTG<br>TTTATACGCCTGCGGCATATCCTTTTGGAATCACAATACATG<br>AAAGCTACATATATTGGACTGACTGGGAAATAAAATTTTTGC<br>ATCGAGTGGACGTAAACGGTGGTGAAGCTGAGCCTTTGGAA<br>ATACCAGCCGGTGGCAGTGGAAGAATGTATGGATTGGTGTC<br>GTTGCCATCCTTCTGTCCATCAGTGGGAAGCCCCTGCGCTGTC

						RGSRSCICPESGEDDGEAIECNELVV*	GAGAATGGCGGATGTCGCTATCTGTGCCTTCCAGATGGCAGAGGAAGTCGTTCGTGTATATGTCCAGAATCCGGCGAAGACGATGGTGAAGCAATCGAGTGTAATGAGCTCGTGGTATAAAGTGTTTTTCCAAGCAATTGAAGGAACATCACATATCCTCTCTCTTTTACCTTTACAGATAGATGTTTGTGAACTGTAAAATATCATACGAAGTCATCCGTTTTTATATTCACCCTTTATGTATGTTCATCACTCTAATTGCAATCGAACAAATAATATTTTTTATGTATTGTACTTGAG

PhdGrFCys 07	Q1L658	3,57E-07	32,110	PF00008<br>PF00594<br>PF00089	192543,3	KTPVSFDCLTSGIPAP<br>TFTWFKNGIPLIPGY<br>FQNFHINKDGNELR<br>LPSPELKDSGKYICL<br>AMNPAGNDSRNFV<br>LNVLEPPTVEKTPSM<br>VKAHQGKEITLDCV<br>VKGVPKPEISWTKD<br>GSLIPHLSSKLLQKSS<br>LFLDNLKLQDSGIYT<br>CIASNEVGFDTKDVE<br>LQVGAPPIIKDPEMY<br>HKVLSGDSVSLSCD<br>ASGFPMPHKIWLKD<br>SVLIEDSLEHIITPDG<br>TLHLFNVSTFDSGIY<br>ACLAENEVGSQEAV<br>RHLNVLTPPTIAENS<br>VDHYETTQAHPVVL<br>HCSSEGTPQPAIYWE<br>HNDKPVDLYNPRFK<br>VFPSGDLHISLAQSS<br>DMGTYTCIAKNEAG<br>SATRSVDLFVFVPPV<br>VKINAPRVIIVTKGR<br>PINITCMATGFPVPK<br>LVWQKNGNFLPPSA<br>STDISGQLKFENVQL	AAAACTCCTGTCAGTTTTGATTGCTTAACTTCTGGAATTCCTG<br>CTCCAACATTCACATGGTTCAAAAATGGAATACCTCTAATTC<br>CTGGGTATTTTCAGAATTTTCACATTAATAAGGATGGGAATG<br>AACTCAGACTACCAAGTCCTGAATTGAAAGACTCTGGAAAA<br>TATATTTGCTTAGCTATGAATCCAGCTGGAAATGATTCTCGA<br>AATTTTGTATTGAATGTTTTAGAGCCTCCCACTGTTGAAAAAA<br>CTCCATCAATGGTAAAAGCTCATCAAGGGAAAGAAATCACT<br>CTAGACTGTGTTGTGAAAGGAGTTCCAAAGCCTGAAATTTCA<br>TGGACTAAGGATGGCTCTTTGATACCTCATTTAAGCTCCAAG<br>TTACTGCAAAAGTCTTCCCTCTTTTTAGATAATTTGAAATTAC<br>AAGATTCTGGGATTTACACTTGCATAGCTTCCAATGAAGTGG<br>GCTTTGATACAAAAGATGTGGAATTGCAAGTTGGGGCACCA<br>CCTATCATCAAAGATCCAGAAATGTATCATAAAGTGTTGTCT<br>GGAGATTCTGTATCTTTATCCTGTGATGCTTCTGGTTTCCCGAT<br>GCCTCATAAAATATGGCTGAAAGACTCAGTTTTAATAGAAGA<br>CAGTTTAGAACATATCATTACACCTGATGGCACACTACACCT<br>GTTCAATGTAAGCACATTTGATAGTGGTATCTATGCATGTTTG<br>GCAGAAAATGAGGTTGGTTCACAGGAAGCTGTTCGGCACTTG<br>AATGTTTTAACACCTCCTACTATTGCTGAAAATTCAGTGGATC<br>ACTATGAAACCACACAAGCTCATCCAGTTGTTCTTCATTGTTC<br>TTCAGAGGGAACACCCCAGCCTGCAATTTATTGGGAACATA<br>ATGACAAACCTGTTGATTTGTATAATCCCCGTTTCAAAGTGTT<br>CCCATCTGGTGATTTGCACATATCCTTAGCGCAATCCTCAGAT<br>ATGGGAACTTACACTTGTATTGCCAAAAATGAAGCTGGTTCA<br>GCTACAAGAAGCGTGGATCTCTTTGTCTTTGTTCCACCAGTAG<br>TTAAAATAAATGCTCCTCGTGTGATTATTGTGACTAAAGGCA<br>GACCAATAAATATCACTTGTATGGCTACTGGCTTCCCAGTGC<br>CTAAACTTGTGTGGCAGAAAAATGGTAATTTTCTTCCTCCTTC

						EDRGNYACTASNSA<br>GRDQQSITLEVHVPP<br>VITTLPKSQDILLGDS<br>FTLVCEATGNPTPSI<br>TWLLNNTQVVGTT<br>HSGLGRSKLTFENV<br>GKDDQGTYICLAQN<br>AAGERKAAAAVRV<br>RAPPVILESSDVESV<br>HLKETVILNCLVKG<br>DPLPNIIWIKNGQQI<br>ELNHRIHLGQNGSL<br>LIYNSSDHDSGEYKC<br>VASNDFGSSERVAEL<br>IIQSRPQFVIEPHNTK<br>VEVGADVYLHCRAK<br>GEPKPVIYWTRGFES<br>LQNSERIIILPNNTLK<br>IVVVQPSDEDVYTC<br>NANNSLGYVYAQA<br>QLIVQVNGKWSQW<br>QEWGSCSTSCGDGV<br>QIRKRFCNDPAPRN<br>GGKACIGLSNEAKY<br>CSQKPCAVNGEWG<br>NWMEWEACSQTCG<br>VGQQQRYRKCDNP<br>QPQFGGDDCIGTSV	AGCAAGTACAGATATTTCTGGACAGTTGAAATTTGAAAATGT<br>ACAGCTTGAAGACAGAGGTAACTATGCTTGCACTGCTTCGAA<br>TTCTGCTGGTCGAGATCAACAAAGTATAACATTAGAAGTTCA<br>TGTCCCTCCTGTCATTACTACTCTGCCAAAAAGTCAAGATATT<br>CTACTAGGAGATTCTTTTACATTGGTCTGTGAAGCAACTGGT<br>AATCCTACTCCATCAATAACTTGGCTGTTAAATAACACTCAA<br>GTAGTTGGTACTACACATTCTGGTCTTGGCCGTAGTAAATTA<br>ACTTTTGAAAATGTTGGAAAAGACGATCAGGGTACTTACATT<br>TGTCTTGCCCAGAATGCTGCTGGAGAGAGGAAAGCAGCTGC<br>TGCTGTAAGAGTTCGAGCTCCCCCAGTAATATTGGAATCTTCT<br>GATGTTGAAAGTGTTCATTTAAAAGAAACTGTCATTCTGAAT<br>TGTTTAGTCAAAGGTGATCCATTGCCTAATATTATTTGGATCA<br>AAAATGGACAGCAGATTGAACTCAATCATCGTATTCATTTGG<br>GTCAGAATGGTTCACTCCTGATTTATAATTCTTCTGATCATGA<br>TTCTGGTGAATACAAATGTGTAGCGAGCAATGATTTTGGATC<br>TTCCGAAAGAGTTGCAGAACTCATAATACAAAGTAGGCCCC<br>AATTCGTAATTGAACCCCATAATACAAAAGTTGAAGTAGGA<br>GCTGATGTCTACTTGCACTGCAGAGCCAAAGGAGAGCCCAA<br>ACCTGTTATTTACTGGACTAGAGGTTTTGAAAGTCTGCAGAA<br>CAGTGAAAGGATTATAATACTTCCAAATAATACTTTAAAAAT<br>AGTTGTTGTGCAGCCATCAGATGAGGATGTGTATACATGTAA<br>TGCTAACAACTCCCTTGGATATGTGTACGCTCAAGCGCAATT<br>AATTGTTCAAGTGAATGGTAAGTGGTCACAGTGGCAAGAAT<br>GGGGAAGTTGTTCGACTTCATGTGGAGATGGTGTTCAGATTC<br>GTAAGCGATTTTGTAATGATCCAGCTCCTAGGAATGGTGGTA<br>AAGCATGCATCGGACTTTCAAATGAGGCAAAGTATTGTAGTC<br>AGAAACCTTGTGCAGTTAATGGTGAATGGGGTAATTGGATGG<br>AATGGGAAGCTTGCAGTCAGACTTGCGGTGTTGGTCAACAAC

						ETASCIHAKCSVDG	AGAGGTACAGAAAATGTGATAATCCCCAACCTCAATTTGGG
						QWSRWSEWHACSV	GGAGATGACTGCATTGGCACCAGTGTTGAAACAGCTTCTTGT
						SCGKGFRYRSRECN	ATTCATGCCAAATGTTCAGTTGATGGTCAGTGGAGTCGATGG
						DPEPLHGGQHCFGE	TCTGAATGGCATGCTTGCAGTGTTTCTTGTGGTAAAGGCTTCC
						SKETDECELEECAIH	GGTATAGATCTCGGGAGTGCAATGATCCAGAACCATTACATG
						GEWGEWNEWSLCSS	GAGGTCAGCACTGCTTTGGTGAAAGTAAAGAAACAGATGAA
						SCGGGTRERSRKCDS	TGTGAGCTGGAAGAGTGTGCCATTCATGGTGAGTGGGGAGA
						PAPAFGGHYCSGIH	ATGGAATGAATGGTCCTTGTGTTCTTCATCCTGTGGAGGAGG
						VQIDYCNNAQCPV	AACTAGAGAAAGAAGTCGGAAATGTGATTCTCCAGCTCCTG
						NGNWGEWSPWGEC	CCTTTGGAGGTCATTATTGCTCTGGAATACATGTTCAAATAG
						SASCNKGQRKRFRA	ATTACTGCAATAATGCTCAGTGCCCAGTGAATGGCAATTGGG
						CDNPAPVGGGRDCT	GAGAGTGGTCTCCATGGGGTGAATGTTCTGCCTCTTGCAACA
						GSSQQIEPCSMHMC	AAGGTCAAAGGAAACGATTCCGTGCCTGTGATAATCCAGCT
						PTDGKWSNWSSWS	CCTGTTGGAGGGGGTCGAGACTGCACTGGATCATCTCAGCAA
						ACSVSCGVGIRSRIRS	ATAGAACCTTGTAGTATGCACATGTGTCCAACTGATGGAAAA
						CDSPEPIHGGKECSG	TGGAGTAATTGGAGTTCTTGGTCTGCATGTTCAGTCTCTTGTG
						DNEELEECGDEACD	GTGTTGGAATTCGATCAAGAATACGATCTTGTGATAGTCCTG
						AIPVQAVGHLIGVIN	AACCCATACATGGTGGAAAAGAATGTTCTGGGGACAATGAG
						GIDFGLSTLIVNISQT	GAGTTGGAAGAATGTGGAGATGAAGCTTGTGATGCAATTCC
						GMEQMVSARIENVT	AGTTCAGGCTGTAGGACACTTGATTGGTGTTATAAATGGAAT
						EDIGNWLKLLVPLLS	TGATTTTGGATTATCAACACTGATTGTAAATATCTCTCAGACT
						PIYWTTAYEIGPAAN	GGAATGGAGCAAATGGTATCTGCAAGAATCGAGAATGTGAC
						GYTTTKGFFRRETQI	TGAAGATATCGGTAACTGGCTGAAACTACTTGTACCCCTCTT
						NFVTGQILFMTHVA	AAGCCCTATTTACTGGACTACTGCATATGAGATTGGACCAGC
						RGLDKRGVLLVDIVI	TGCTAATGGGTACACTACTACTAAAGGTTTCTTCAGAAGAGA
						SGEVPDIPILSQIVLH	AACTCAAATAAACTTCGTTACTGGTCAAATTCTATTCATGACT
						PYTEDYVQIGPGAIY	CATGTTGCTCGAGGACTTGATAAACGCGGTGTGCTATTGGTT
						AFSTRTYEIEGHVLP	GATATCGTTATAAGTGGGGAAGTTCCAGATATACCTATTTTG

						YSWNHTIYYDETLD<br>GMPFPTQRLYANSIS<br>SCYDNDAEEMNYNI<br>SAAIGKGYLNSECAL<br>GFFLSSDGLYCLDID<br>ECEADNECDHICSN<br>FPGGFSCQCFNGFVL<br>DLDGKSCNDVDECE<br>LNLAACSPNEECVN<br>TFGSFKCQIICRNGY<br>ERNEDELFCKDVNE<br>CDLNLHNCTQICM<br>NTIGSYSCSCYEGYH<br>LISRYECLDIDECNRL<br>TTPCNHFCTNTPGSF<br>TCSCQPGFKLHESRN<br>CIDIDECQDKTHTCL<br>FSEECKNTIGGYECH<br>TRCAAGLVSASNGS<br>CIDIDECETAESECH<br>FTQVCINTYGSYHCS<br>CFRGYYSDGPGKPC<br>QDINECDDKGSCQH<br>MCENTQGSYRCICP<br>TGYQLDLYGKKCLD<br>IDECQESNIDCGPDR<br>MCFNTRGSYECIETP<br>CPPKYDRDPVTGYCI	AGTCAAATAGTGCTTCATCCTTATACAGAGGATTATGTTCAG<br>ATTGGACCTGGAGCAATCTATGCCTTTTCCACCCGCACATAT<br>GAAATAGAAGGTCATGTTCTACCATATTCTTGGAATCATACA<br>ATATATTATGATGAAACATTGGATGGCATGCCATTCCCTACC<br>CAGAGATTATATGCAAATAGCATCAGCAGCTGTTATGATAAT<br>GATGCTGAGGAAATGAACTACAATATATCTGCTGCAATTGGA<br>AAAGGTTATTTGAATTCAGAATGTGCTCTTGGTTTCTTCTTAA<br>GTTCAGATGGCTTATATTGTCTTGATATTGATGAATGTGAAGC<br>AGATAATGAATGTGACCACATATGTAGTAATTTTCCTGGAGG<br>ATTTTCATGCCAGTGTTTTAATGGGTTTGTCCTAGATTTAGAT<br>GGGAAAAGTTGTAATGATGTGGATGAATGTGAATTAAATTTA<br>GCTGCATGTTCCCCTAATGAAGAATGTGTCAATACATTTGGA<br>TCATTTAAGTGTCAGATTATATGCAGAAATGGTTATGAGAGA<br>AATGAAGATGAGTTGTTTTGTAAAGATGTCAATGAATGTGAT<br>TTAAATTTACATAACTGCACCCAAATTTGCATGAACACTATC<br>GGAAGTTATAGTTGCAGTTGTTATGAAGGTTATCATCTTATTA<br>GTAGATATGAATGTTTAGACATTGATGAATGCAACCGATTAA<br>CTACACCATGCAACCACTTTTGTACAAATACCCCTGGTAGCT<br>TTACATGTTCATGCCAACCAGGTTTTAAATTGCATGAAAGCA<br>GAAATTGTATAGATATTGATGAATGCCAGGATAAAACACAT<br>ACTTGTCTGTTTTCTGAAGAATGCAAAAATACAATTGGTGGA<br>TACGAATGTCATACCAGGTGTGCAGCAGGTCTTGTAAGTGCA<br>TCCAATGGCTCTTGCATTGATATTGATGAATGTGAAACAGCA<br>GAATCAGAATGTCACTTTACTCAAGTGTGTATTAATACATAT<br>GGGAGCTACCATTGTTCATGTTTCAGAGGATATTATTCTGATG<br>GTCCTGGTAAACCATGCCAAGATATTAATGAATGTGATGACA<br>AAGGCAGCTGTCAACATATGTGTGAAAATACCCAAGGTAGT<br>TATCGATGCATATGTCCAACAGGTTATCAACTAGACCTTTAT

						LECEKANSKCPPNIKYSEILAFKTIALPSGIQAYQDLIRLVAYDENGIQLPNTVFSIIENDTGIPFRIRLENGKGVLYTQKSMESNKEYKIKVQAISYDVNNHTIHYKTKFLVFVSVSQYPY*	GGAAAGAAATGCCTAGACATTGACGAATGCCAGGAATCGAATATTGACTGTGGTCCTGATCGAATGTGTTTCAACACAAGAGGTTCCTATGAATGTATTGAAACTCCTTGTCCCCCTAAATATGACAGAGATCCTGTAACAGGTTATTGTATTTTGGAATGTGAAAAAGCGAACTCCAAGTGCCCTCCTAACATCAAGTATTCAGAAATTTTGGCATTTAAAACAATAGCATTACCTAGTGGAATTCAAGCTTATCAGGATCTAATACGACTCGTTGCTTATGATGAGAATGGCATTCAGCTACCGAATACAGTATTTTCCATTATAGAGAATGACACAGGAATTCCTTTCAGGATAAGACTGGAAAATGGAAAGGGTGTTCTTTATACACAAAAAAGCATGGAATCCAATAAGGAGTACAAGATAAAAGTTCAAGCTATATCATATGATGTAAATAACCATACCATTCACTATAAAACAAAGTTTTTAGTGTTTGTTTCAGTTTCACAGTACCCATATTAAAAAGTTTTTTTTTTGCTTAGTGTATTTACATTTAAAAATCTTGCTTTATAATGTATTTAATTTTCTGATGTTTAAAAAAAAAATTGTATTATATTTTATATTTATCATGCATGTCTGTAAATATAATGTTTCTAGAAAGATTTTCATAACTACATAATAGTATAAAAGTAAGTATATTTTTGTTACATATATACTGAACTGCTGTCAGTATATAGAAAAATATTGAAGTAATTAATTATGAAAAAACTCAGGTCTTGTAATAAGAGCACTAGATTGATACATAGTTATCACAATCTTCAAAGACTCTCACATTTTCAGTATCTGTGCTTTGTAAAGATTTGATTTATCATCATCATTTAAAAGAAAATAAAATTTTTATTTTAGAATTTTCATTATAAAAAAAA

PhdGrFCys 08	Q1L658	1,33E-05	38,824	PF00008<br>PF00594<br>PF00089	79397,76	FEVCPEGIYGTNCLK<br>ICLCGMRECDPVTG<br>TCLCDPGKMGALCT<br>QSCPKGKFGKSCLKT<br>CTCENGGTCNPKNG<br>KCSCPTGFIGKSCEK<br>KCPPDKYGSDCQNQ<br>CYCKNGATCDPING<br>RCHCSAGWTGIKCD<br>QPCSPGSYGPMCKH<br>HCECLNGGECDRYT<br>GECICAPGWTGGEC<br>EKPCSAGKYGENCE<br>QDCPCENGATCDHI<br>SGACICGPGWSGTY<br>CEKPCPRGYHGIECK<br>NTCDCGHGISCHPE<br>TGLCHCPPGKHGDK<br>CLKTCPAGFYGNHC<br>REHCHCQNGAVCE<br>PNDGTCHCLPGWY<br>GDTCHLACPLGTYG<br>YDCEGVCLCQNDG<br>NCDPKDGRCKCKP<br>GYRGKHCEMVCKH<br>GFFGLHCAQMCSCK<br>KDEPCHHITGECDC<br>PAGTKGKGCELTCP	TTTTTTTTATAACCCATTTTTAATGTAGGACATTTTTATATTAC<br>AAGAAAATCATTCAAAACTAATTATCTAATAAAATCATCGGT<br>ATAAATTAAATACCTCGTGAAGCATAAAATTTTACTGTGCAG<br>AGGAAATTCATTTCAAAAGTTGTCTCACTCAGTCAATCTTTCA<br>GATTTGTCAACACTGAATTTTTTTAACCTGTTTTTAAATTCTCT<br>TAATAATTTTTCATTATAATTTCATTTTGAACAGCAAATATTA<br>AATTAGAAATTTCTCAGACTGAATATTTGACATGTATAGCAC<br>AAAATCAGATTAGATATATGCACACATTATATATATCTTACA<br>GAAATTTGTATAAATGTTATACTATAAATTAACAAAATAATT<br>TTTTTAGGGTTTTTTTTTCCTAAAACTGAAATATATTTCAACA<br>AAAGTAAAAAAATAGATATTGATTGTATTTCATCTTTTACACA<br>ATTTTCTTGCTCCACATTATTCTTTTTATTTCCTGAGAAAAGTG<br>TTCTGTGTTGAAATCTTGGCTCTCCTAAGCCGAAAGCACAAA<br>CATTATTACAAATGCTTTGATGACTTGAAGGTGTTTTCTCAAA<br>ATGTTTCATAAGCTCATTTTATTTAGCTGATACCATTGGAAGC<br>TTCATTATCTGCAGATTCATAAATTGATCCATCTTTAGCATCA<br>CATGTGGATCCTATCCATCCTGGAGGGCAAGTGCATTTACCA<br>GATACTGTATCACACGTGCCGCCATGCTGGCAATGGCAACGC<br>TGTAGACATTTTGGACCATATTTTCCATCATGGCATAATTCAT<br>TGCAATGTGGTCCAATAAATCCTGGAGGACAATGACATTCTC<br>CAGTAACGGGATCACAAGGATGTGTTCCACACTTGCATTCTT<br>CTTGGCATCCATGACCATAGAAGCCCAAGACGCATTCTTTCT<br>CACATTTGGTACCATGGTGTCCAGCTGTACAGCTGCAAGTAC<br>CTGTCACCGGATGACATGGTCGATCCCCGACGCATTCACAGC<br>TTTCTTGACAAGATTTACCAAACCATCCTGATGGACACTCAT<br>ATTCACAGTGTCCTCCAATATAACCATCTGGGCAGTCACAAC<br>AGCCAGTTATAGGATCACAGGAAGCATTATGTTTACAATGAC<br>ATGCTAGCAAGCAATTTGGTCCAAAGAAGCCATGAGGGCAA

						EGRFGPDCKEKCKC<br>PSNQQCDPVNGKCF<br>CRSGFRGPNCSKICP<br>NGTFGLNCSQLCAC<br>ENEADCDPLTGECIC<br>KPGFTGPTCGQECPE<br>GYHGDMCLKRCHC<br>QNGAKCDPETGKC<br>NCPLGFRGEKCEEP<br>CPAGTYGEDCKDDC<br>QCSSNKCDPKTGQC<br>ICPPYTSGNECKEGC<br>PHGFFGPNCLLACH<br>CKHNASCDPITGCC<br>DCPDGYIGGHCEYE<br>CPSGWFGKSCQESC<br>ECVGDRPCHPVTGT<br>CSCTAGHHGTKCEK<br>ECVLGFYGHGCQEE<br>CKCGTHPCDPVTGE<br>CHCPPGFIGPHCNEL<br>CHDGKYGPKCLQRC<br>HCQHGGTCDTVSG<br>KCTCPPGWIGSTCD<br>AKDGSIYESADNEAS<br>NGIS*	CCTTCTTTACACTCATTCCCACTCGTATACGGTGGACAAATGC<br>ATTGCCCAGTTTTGGGATCACATTTATTACTAGAACACTGGC<br>AATCATCTTTACAGTCTTCTCCATATGTGCCTGCAGGACAAG<br>GTTCTTCGCATTTCTCTCCTCTGAAACCAAGAGGACAATTAC<br>ATTTGCCAGTCTCTGGGTCACATTTCGCCCCGTTCTGACAGTG<br>ACACCTTTTCAGACACATATCTCCGTGATATCCCTCTGGGCAT<br>TCTTGTCCACAAGTAGGTCCAGTGAATCCTGGTTTACAAATG<br>CATTCTCCAGTCAAGGGATCACAATCAGCTTCATTCTCACAT<br>GCGCATAACTGCGAACAATTTAAACCAAACGTTCCATTTGGG<br>CATATTTTACTACAGTTTGGACCTCGAAATCCAGACCTACAA<br>AAGCATTTACCGTTTACTGGATCGCACTGCTGATTCGATGGA<br>CATTTACACTTTTCTTTGCAATCAGGACCAAAGCGTCCTTCCG<br>GGCAAGTTAGTTCGCATCCTTTGCCCTTGGTTCCTGCAGGACA<br>ATCGCATTCGCCTGTAATGTGATGACATGGTTCATCTTTTTTA<br>CACGAACACATTTGTGCGCAATGGAGGCCGAAAAATCCATG<br>TTTACAAACCATTTCACAATGCTTTCCTCTATATCCCGGCTTA<br>CACTTGCAACGTCCGTCTTTCGGATCGCAATTACCATCATTCT<br>GACACAGACATACGCCTTCACAATCGTATCCGTAAGTTCCAA<br>GTGGACAAGCCAAGTGGCACGTATCGCCATACCAGCCTGGT<br>AGACAATGACACGTGCCGTCGTTTGGTTCACAAACTGCACCG<br>TTCTGGCAATGGCAATGTTCTCTGCAGTGATTTCCGTAAAAA<br>CCAGCAGGGCAAGTTTTCAGACACTTATCACCATGCTTTCCA<br>GGTGGACAGTGGCACAGTCCAGTTTCCGGATGACAAGATAT<br>GCCATGCCCACAGTCACAAGTATTTTTGCATTCTATACCGTG<br>GTATCCGCGAGGACAAGGTTTTTCACAGTACGTGCCGCTCCA<br>TCCAGGCCCACAAATACAGGCACCGCTAATGTGATCACAAG<br>TCGCTCCATTCTCACAAGGACAGTCTTGTTCGCAATTCTCTCC<br>ATACTTGCCAGCACTGCATGGCTTTTCGCATTCACCTCCTGTC

							CACCCAGGAGCACATATGCATTCCCCTGTGTATCGGTCGCAT<br>TCTCCTCCGTTCAAACACTCACAATGATGCTTGCACATTGGTC<br>CGTAGCTTCCTGGACTGCATGGTTGGTCGCATTTGATCCCTGT<br>CCAACCTGCAGAACAATGGCATCTACCATTGATTGGGTCACA<br>AGTCGCCCCATTCTTACAATAACACTGATTTTGACAGTCAGA<br>TCCGTACTTATCAGGAGGACATTTTTTTTCACACGATTTTCCG<br>ATGAATCCAGTTGGACAAGAGCATTTGCCATTTTTAGGATTG<br>CATGTTCCTCCATTTTCGCAAGTGCATGTTTTCAAACAGCTTT<br>TTCCAAATTTTCCTTTGGGACAACTTTGAGTACACAGAGCAC<br>CCATTTTTCCTGGATCACACAAACATGTTCCTGTCACTGGGTC<br>ACATTCTCTCATGCCACATAAGCAAATTTTTAGACAATTTGTG<br>CCATATATTCCTTCAGGACACACTTCGAATTACATTTCTGTGT<br>ATGTTCCTTGAAGCATTTGCACTGGTTTCGGCAGTTTGGTCCA<br>AAGGTATGTGGGGGACAAGGGATGTTACATAAAGGTCCAAA<br>GAAACCAGGTTCACATTCGCAGTAACCAAATAACTTATTACA<br>GCGCCCAGTAGGACAACGTTGAGGGCATTTTTTCTCGCACTT<br>TTCACCGAAGCTTCCTGGGGGACATCCATCTTCACAGCTCTT<br>GCCTTGAATTCCTGGTGGACAGAGGCAAGCGCCTGTCACTGG<br>GTCGCATGAACCGCCATTTTGACACTTGCATTTTTTCGTGCAG<br>CCATCGCCATAAGTCCCCTTGGCACACGTTTCATTGCAAAGA<br>AGTCCTGTGAATCCTGGTTGGCAAGTACAGCTAAGAGCTCGT<br>ATATCACATTTACCTCCATTCTGGCAATTGTCACAATTGAACA<br>TGCAGCCAGGTCCGAATGTTCCCCGACCGCAAGATTGTCTGA<br>GGTCTGGTCCTTCGAGAGCTAAAAAGTGACGGAATTCTTGGT<br>CAAGGAGTGGAACAACAGTTGGCGGTGAAACTTGGAAACGA<br>ATCCTTGGCGAATTTTGGTGTGGAGGAGTGTCCGATCGGCTG<br>TGCGACGTCTCGATTAACACTGCCTGATGCGGTGGCACAACA<br>TCACAACTTTTTTTATCTGAGAGAAGTTTATACCCTTCCAAGC

							AAAGACAACGATATGATCCAAGGGTGTTTTCACACGTTTGGTCACATCCTCCATTGTTGACAAGGCATTCGTTGTTGTCATTGCAAGTGATCCCATCTCTGTCTAGAGAGTATCCAAGTCGACATGCGCAGGAATAACCACCAGGGCTATTGTTGCAGTCTTGCTTGCATTGGGATAAACCACGTCGACACTCATCAATATCATTGCAAGTTTTGCCATCCGCGTCGAGCTCGTAACCGGAGTAGCAGCTGCAGATTGGTCCACCCGGTCCGTGGCGACATTTTTGATCACATCCGCCGTTTTTATCTGCACATGTGTTCACCACTTCCATTTCAATTCGGAAACAGGATCTACCGTCATTTCCAAGCTGAAAGGATAGATGACACATGCAAGAATACGATCCAGGAGTATTGTGACACACATGAGAACAGGGGTTATTAGTACTGCACTCGTCAATATTTCAATTTCAGAGTTCTTTCGTTTCTCAAAATTTCATTTTATTTATTTCAGTCTATCATAAGACTTATTGTTCGTAAACAGTACACAGAAATCCCAAACAACTTCAGATTGTGTCGCAACGTGCAGGCCCTCTATCGCCCGTTACGCAATGCCAAGTTCTTGGAACGCGCCTCGTGC

PhdGrFCys 09	Q1L658	1,39E-05	38,824	PF00008<br>PF00594<br>PF00089	127231,6 3	NIDECSTNNPCSHV<br>CHNTPGSYSCMCHL<br>SFQLGNDGRSCFRIE<br>MEVVNTCADKNGG<br>CDQKCRHGPGGPIC<br>SCYSGYELDADGKT<br>CNDIDECRRGLSQC<br>KQDCNNSPGGYSCA<br>CRLGYSLDRDGITCN<br>DNNECLVNNGGCD<br>QTCENTLGSYRCLCL<br>EGYKLLSDKKSCDV<br>VPPHQAVLIETSHSR<br>SDTPPHQNSPRIRFQ<br>VSPPTVVPLLDQEFR<br>HFLALEGPDLRQSC<br>GRGTFGPGCMFNCD<br>NCQNGGKCDIRALS<br>CTCQPGFTGLLCNE<br>TCAKGTYGDGCTKK<br>CKCQNGGSCDPVTG<br>ACLCPPGIQGKSCED<br>GCPPGSFGEKCEKK<br>CPQRCPTGRCNKLF<br>GYCECEPGFFGPLCN<br>IPCPPHTFGPNCRNQ<br>CKCFKEHTQKCNSK<br>TGKCECKVGFTGAK	TTTTTTTTATAACCCATTTTTAATGTAGGACATTTTTATATTAC<br>AAGAAAATCATTCAAAACTAATTATCTAATAAAATCATCGGT<br>ATAAATTAAATACCTCGTGAAGCATAAAATTTTACTGTGCAG<br>AGGAAATTCATTTCAAAAGTTGTCTCACTCAGTCAATCTTTCA<br>GATTTGTCAACACTGAATTTTTTTAACCTGTTTTTAAATTCTCT<br>TAATAATTTTTCATTATAATTTCATTTTGAACAGCAAATATTA<br>AATTAGAAATTTCTCAGACTGAATATTTGACATGTATAGCAC<br>AAAATCAGATTAGATATATGCACACATTATATATATCTTACA<br>GAAATTTGTATAAATGTTATACTATAAATTAACAAAATAATT<br>TTTTTAGGGTTTTTTTTTCCTAAAACTGAAATATATTTCAACA<br>AAAGTAAAAAAATAGATATTGATTGTATTTCATCTTTTACACA<br>ATTTTCTTGCTCCACATTATTCTTTTTATTTCCTGAGAAAAGTG<br>TTCTGTGTTGAAATCTTGGCTCTCCTAAGCCGAAAGCACAAA<br>CATTATTACAAATGCTTTGATGACTTGAAGGTGTTTTCTCAAA<br>ATGTTTCATAAGCTCATTTTATTTAGCTGATACCATTGGAAGC<br>TTCATTATCTGCAGATTCATAAATTGATCCATCTTTAGCATCA<br>CATGTGGATCCTATCCATCCTGGAGGGCAAGTGCATTTACCA<br>GATACTGTATCACACGTGCCGCCATGCTGGCAATGGCAACGC<br>TGTAGACATTTTGGACCATATTTTCCATCATGGCATAATTCAT<br>TGCAATGTGGTCCAATAAATCCTGGAGGACAATGACATTCTC<br>CAGTAACGGGATCACAAGGATGTGTTCCACACTTGCATTCTT<br>CTTGGCATCCATGACCATAGAAGCCCAAGACGCATTCTTTCT<br>CACATTTGGTACCATGGTGTCCAGCTGTACAGCTGCAAGTAC<br>CTGTCACCGGATGACATGGTCGATCCCCGACGCATTCACAGC<br>TTTCTTGACAAGATTTACCAAACCATCCTGATGGACACTCAT<br>ATTCACAGTGTCCTCCAATATAACCATCTGGGCAGTCACAAC<br>AGCCAGTTATAGGATCACAGGAAGCATTATGTTTACAATGAC<br>ATGCTAGCAAGCAATTTGGTCCAAAGAAGCCATGAGGGCAA

						CETECPPNRWGETC	CCTTCTTTACACTCATTCCCACTCGTATACGGTGGACAAATGC
						QNPCNCPSGTICNP	ATTGCCCAGTTTTGGGATCACATTTATTACTAGAACACTGGC
						GNGECMKECPPGLN	AATCATCTTTACAGTCTTCTCCATATGTGCCTGCAGGACAAG
						GTNCDEVCPEGIYG	GTTCTTCGCATTTCTCTCCTCTGAAACCAAGAGGACAATTAC
						TNCLKICLCGMREC	ATTTGCCAGTCTCTGGGTCACATTTCGCCCCGTTCTGACAGTG
						DPVTGTCLCDPGKM	ACACCTTTTCAGACACATATCTCCGTGATATCCCTCTGGGCAT
						GALCTQSCPKGKFG	TCTTGTCCACAAGTAGGTCCAGTGAATCCTGGTTTACAAATG
						KSCLKTCTCENGGT	CATTCTCCAGTCAAGGGATCACAATCAGCTTCATTCTCACAT
						CNPKNGKCSCPTGFI	GCGCATAACTGCGAACAATTTAAACCAAACGTTCCATTTGGG
						GKSCEKKCPPDKYG	CATATTTACTACAGTTTGGACCTCGAAATCCAGACCTACAA
						SDCQNQCYCKNGA	AAGCATTTACCGTTTACTGGATCGCACTGCTGATTCGATGGA
						TCDPINGRCHCSAG	CATTTACACTTTTCTTTGCAATCAGGACCAAAGCGTCCTTCCG
						WTGIKCDQPCSPGS	GGCAAGTTAGTTCGCATCCTTTGCCCTTGGTTCCTGCAGGACA
						YGPMCKHHCECLN	ATCGCATTCGCCTGTAATGTGATGACATGGTTCATCTTTTTA
						GGECDRYTGECICAP	CACGAACACATTTGTGCGCAATGGAGGCCGAAAAATCCATG
						GWTGGECEKPCSAG	TTTACAAACCATTTCACAATGCTTTCCTCTATATCCCGGCTTA
						KYGENCEQDCPCEN	CACTTGCAACGTCCGTCTTTCGGATCGCAATTACCATCATTCT
						GATCDHISGACICGP	GACACAGACATACGCCTTCACAATCGTATCCGTAAGTTCCAA
						GWSGTYCEKPCPRG	GTGGACAAGCCAAGTGGCACGTATCGCCATACCAGCCTGGT
						YHGIECKNTCDCGH	AGACAATGACACGTGCCGTCGTTTGGTTCACAAACTGCACCG
						GISCHPETGLCHCPP	TTCTGGCAATGGCAATGTTCTCTGCAGTGATTTCCGTAAAAA
						GKHGDKCLKTCPA	CCAGCAGGGCAAGTTTTCAGACACTTATCACCATGCTTTCCA
						GFYGNHCREHCHC	GGTGGACAGTGGCACAGTCCAGTTTCCGGATGACAAGATAT
						QNGAVCEPNDGTC	GCCATGCCCACAGTCACAAGTATTTTTGCATTCTATACCGTG
						HCLPGWYGDTCHL	GTATCCGCGAGGACAAGGTTTTTCACAGTACGTGCCGCTCCA
						ACPLGTYGYDCEGV	TCCAGGCCCACAAATACAGGCACCGCTAATGTGATCACAAG
						CLCQNDGNCDPKD	TCGCTCCATTCTCACAAGGACAGTCTTGTTCGCAATTCTCTCC
						GRCKCKPGYRGKHC	ATACTTGCCAGCACTGCATGGCTTTTCGCATTCACCTCCTGTC

						EMVCKHGFFGLHC<br>AQMCSCKKDEPCH<br>HITGECDCPAGTKG<br>KGCELTCPEGRFGPD<br>CKEKCKCPSNQQCD<br>PVNGKCFCRSGFRG<br>PNCSKICPNGTFGLN<br>CSQLCACENEADCD<br>PLTGECICKPGFTGP<br>TCGQECPEGYHGD<br>MCLKRCHCQNGAK<br>CDPETGKCNCPLGF<br>RGEKCEEPCPAGTY<br>GEDCKDDCQCSSNK<br>CDPKTGQCICPPYTS<br>GNECKEGCPHGFFG<br>PNCLLACHCKHNA<br>SCDPITGCCDCPDGY<br>IGGHCEYECPSGWF<br>GKSCQESCECVGDR<br>PCHPVTGTCSCTAG<br>HHGTKCEKECVLGF<br>YGHGCQEECKCGT<br>HPCDPVTGECHCPP<br>GFIGPHCNELCHDG<br>KYGPKCLQRCHCQ<br>HGGTCDTVSGKCTC	CACCCAGGAGCACATATGCATTCCCCTGTGTATCGGTCGCAT<br>TCTCCTCCGTTCAAACACTCACAATGATGCTTGCACATTGGTC<br>CGTAGCTTCCTGGACTGCATGGTTGGTCGCATTTGATCCCTGT<br>CCAACCTGCAGAACAATGGCATCTACCATTGATTGGGTCACA<br>AGTCGCCCCATTCTTACAATAACACTGATTTTGACAGTCAGA<br>TCCGTACTTATCAGGAGGACATTTTTTTCACACGATTTTCCG<br>ATGAATCCAGTTGGACAAGAGCATTTGCCATTTTTAGGATTG<br>CATGTTCCTCCATTTTCGCAAGTGCATGTTTTCAAACAGCTTT<br>TTCCAAATTTTCCTTTGGGACAACTTTGAGTACACAGAGCAC<br>CCATTTTTCCTGGATCACACAAACATGTTCCTGTCACTGGGTC<br>ACATTCTCTCATGCCACATAAGCAAATTTTTAGACAATTTGTG<br>CCATATATTCCTTCAGGACACACTTCATCACAGTTAGTTCCAT<br>TTAATCCTGGTGGGCACTCTTTCATGCACTCGCCGTTTCCGGG<br>GTTACAGATGGTACCAGAGGGGCAGTTGCAAGGATTCTGGC<br>AAGTCTCTCCCCACCGGTTTGGCGGACATTCAGTCTCACATTT<br>AGCGCCGGTGAAACCCACTTTACATTCACATTTCCCTGTCTTC<br>GAATTACATTTCTGTGTATGTTCCTTGAAGCATTTGCACTGGT<br>TTCGGCAGTTTGGTCCAAAGGTATGTGGGGGACAAGGGATGT<br>TACATAAAGGTCCAAAGAAACCAGGTTCACATTCGCAGTAA<br>CCAAATAACTTATTACAGCGCCCAGTAGGACAACGTTGAGG<br>GCATTTTTTCTCGCACTTTTCACCGAAGCTTCCTGGGGGACAT<br>CCATCTTCACAGCTCTTGCCTTGAATTCCTGGTGGACAGAGG<br>CAAGCGCCTGTCACTGGGTCGCATGAACCGCCATTTTGACAC<br>TTGCATTTTTTCGTGCAGCCATCGCCATAAGTCCCCTTGGCAC<br>ACGTTTCATTGCAAAGAAGTCCTGTGAATCCTGGTTGGCAAG<br>TACAGCTAAGAGCTCGTATATCACATTTACCTCCATTCTGGC<br>AATTGTCACAATTGAACATGCAGCCAGGTCCGAATGTTCCCC<br>GACCGCAAGATTGTCTGAGGTCTGGTCCTTCGAGAGCTAAAA

						PPGWIGSTCDAKDGSIYESADNEASNGIS*	AGTGACGGAATTCTTGGTCAAGGAGTGGAACAACAGTTGGCGGTGAAACTTGGAAACGAATCCTTGGCGAATTTTGGTGTGGAGGAGTGTCCGATCGGCTGTGCGACGTCTCGATTAACACTGCCTGATGCGGTGGCACAACATCACAACTTTTTTTATCTGAGAGAAGTTTATACCCTTCCAAGCAAAGACAACGATATGATCCAAGGGTGTTTTCACACGTTTGGTCACATCCTCCATTGTTGACAAGGCATTCGTTGTTGTCATTGCAAGTGATCCCATCTCTGTCTAGAGAGTATCCAAGTCGACATGCGCAGGAATAACCACCAGGGCTATTGTTGCAGTCTTGCTTGCATTGGGATAAACCACGTCGACACTCATCAATATCATTGCAAGTTTTGCCATCCGCGTCGAGCTCGTAACCGGAGTAGCAGCTGCAGATTGGTCCACCCGGTCCGTGGCGACATTTTTGATCACATCCGCCGTTTTTATCTGCACATGTGTTCACCACTTCCATTTCAATTCGGAAACAGGATCTACCGTCATTTCCAAGCTGAAAGGATAGATGACACATGCAAGAATACGATCCAGGAGTATTGTGACACACATGAGAACAGGGGTTATTAGTACTGCACTCGTCAATATTTCAATTTCAGAGTTCTTTCGTTTCTCAAAATTTCATTTTATTTATTTCAGTCTATCATAAGACTTATTGTTCGTAAACAGTACACAGAAATCCCAAACAACTTCAGATTGTGTCGCAACGTGCAGGCCCTCTATCGCCCGTTACGCAATGCCAAGTTCTTGGAACGCGCCTCGTGC

PhdGrFCys 10	P81428	6,64E-06	32,292	PF00008<br>PF00594<br>PF00089	155738.43<br>**153646.83**	MFGLSPLGVTLFSLV<br>LIAQC**NDGMCYNS**<br>**GRCLGRGRNGFNF**<br>**RCLCQSGFQGHRC**<br>**QYDVNECLNNNGG**<br>**CHHHCCNTLGSYY**<br>**CRCNPGYRLNADG**<br>**RTCSDIDECLHHNG**<br>**GCQNKCINYDGGY**<br>**SCECSPGQRLHTDG**<br>**RTCIDMGNQCSIN**<br>**NGGCQHICEDSHG**<br>**RSRCRCRQGYRLNP**<br>**DGRTCEALDACSR**<br>**DNGGCSQRCDNV**<br>**GGRAICNCLPGYQL**<br>**SRDGKTCTEIDACA**<br>**RDKGGCSHICESVS**<br>**GRPVCSCLPGFQLT**<br>**RDGRTCEDIDECST**<br>**NNPCSHVCHNTPG**<br>**SYSCMCHLSFQLGN**<br>**DGRSCFRIEMEVVN**<br>**TCADKNGGCDQK**<br>**CRHGPGGPICSCYS**<br>**GYELDADGKTCND**<br>**IDECRRGLSQCKQD**<br>**CNNSPGGYSCACR**	TTTTTTTTATAACCCATTTTTAATGTAGGACATTTTTATATTAC<br>AAGAAAATCATTCAAAACTAATTATCTAATAAAATCATCGGT<br>ATAAATTAAATACCTCGTGAAGCATAAAATTTTACTGTGCAG<br>AGGAAATTCATTTCAAAAGTTGTCTCACTCAGTCAATCTTTCA<br>GATTTGTCAACACTGAATTTTTTTAACCTGTTTTTAAATTCTCT<br>TAATAATTTTTCATTATAATTTCATTTTGAACAGCAAATATTA<br>AATTAGAAATTTCTCAGACTGAATATTTGACATGTATAGCAC<br>AAAATCAGATTAGATATATGCACACATTATATATATCTTACA<br>GAAATTTGTATAAATGTTATACTATAAATTAACAAAATAATT<br>TTTTTAGGGTTTTTTTTTCCTAAAACTGAAATATATTTCAACA<br>AAAGTAAAAAAATAGATATTGATTGTATTTCATCTTTTACACA<br>ATTTTCTTGCTCCACATTATTCTTTTTATTTCCTGAGAAAAGTG<br>TTCTGTGTTGAAATCTTGGCTCTCCTAAGCCGAAAGCACAAA<br>CATTATTACAAATGCTTTGATGACTTGAAGGTGTTTTCTCAAA<br>ATGTTTCATAAGCTCATTTTATTTAGCTGATACCATTGGAAGC<br>TTCATTATCTGCAGATTCATAAATTGATCCATCTTTAGCATCA<br>CATGTGGATCCTATCCATCCTGGAGGGCAAGTGCATTTACCA<br>GATACTGTATCACACGTGCCGCCATGCTGGCAATGGCAACGC<br>TGTAGACATTTTGGACCATATTTTCCATCATGGCATAATTCAT<br>TGCAATGTGGTCCAATAAATCCTGGAGGACAATGACATTCTC<br>CAGTAACGGGATCACAAGGATGTGTTCCACACTTGCATTCTT<br>CTTGGCATCCATGACCATAGAAGCCCAAGACGCATTCTTTCT<br>CACATTTGGTACCATGGTGTCCAGCTGTACAGCTGCAAGTAC<br>CTGTCACCGGATGACATGGTCGATCCCCGACGCATTCACAGC<br>TTTCTTGACAAGATTTACCAAACCATCCTGATGGACACTCAT<br>ATTCACAGTGTCCTCCAATATAACCATCTGGGCAGTCACAAC<br>AGCCAGTTATAGGATCACAGGAAGCATTATGTTTACAATGAC<br>ATGCTAGCAAGCAATTTGGTCCAAAGAAGCCATGAGGGCAA

						**LGYSLDRDGITCND**	CCTTCTTTACACTCATTCCCACTCGTATACGGTGGACAAATGC
						**NNECLVNNGGCD**	ATTGCCCAGTTTTGGGATCACATTTATTACTAGAACACTGGC
						**QTCENTLGSYRCLC**	AATCATCTTTACAGTCTTCTCCATATGTGCCTGCAGGACAAG
						**LEGYKLLSDKKSCD**	GTTCTTCGCATTTCTCTCCTCTGAAACCAAGAGGACAATTAC
						**VVPPHQAVLIETSH**	ATTTGCCAGTCTCTGGGTCACATTTCGCCCCGTTCTGACAGTG
						**SRSDTPPHQNSPRI**	ACACCTTTTCAGACACATATCTCCGTGATATCCCTCTGGGCAT
						**RFQVSPPTVVPLLD**	TCTTGTCCACAAGTAGGTCCAGTGAATCCTGGTTTACAAATG
						**QEFRHFLALEGPDL**	CATTCTCCAGTCAAGGGATCACAATCAGCTTCATTCTCACAT
						**RQSCGRGTFGPGC**	GCGCATAACTGCGAACAATTTAAACCAAACGTTCCATTTGGG
						**MFNCDNCQNGGK**	CATATTTTACTACAGTTTGGACCTCGAAATCCAGACCTACAA
						**CDIRALSCTCQPGF**	AAGCATTTACCGTTTACTGGATCGCACTGCTGATTCGATGGA
						**TGLLCNETCAKGTY**	CATTTACACTTTTCTTTGCAATCAGGACCAAAGCGTCCTTCCG
						**GDGCTKKCKCQN**	GGCAAGTTAGTTCGCATCCTTTGCCCTTGGTTCCTGCAGGACA
						**GGSCDPVTGACLC**	ATCGCATTCGCCTGTAATGTGATGACATGGTTCATCTTTTTA
						**PPGIQGKSCEDGCP**	CACGAACACATTTGTGCGCAATGGAGGCCGAAAAATCCATG
						**PGSFGEKCEKKCPQ**	TTTACAAACCATTTCACAATGCTTTCCTCTATATCCCGGCTTA
						**RCPTGRCNKLFGYC**	CACTTGCAACGTCCGTCTTTCGGATCGCAATTACCATCATTCT
						**ECEPGFFGPLCNIPC**	GACACAGACATACGCCTTCACAATCGTATCCGTAAGTTCCAA
						**PPHTFGPNCRNQC**	GTGGACAAGCCAAGTGGCACGTATCGCCATACCAGCCTGGT
						**KCFKEHTQKCNSK**	AGACAATGACACGTGCCGTCGTTTGGTTCACAAACTGCACCG
						**TGKCECKVGFTGA**	TTCTGGCAATGGCAATGTTCTCTGCAGTGATTTCCGTAAAAA
						**KCETECPPNRWGET**	CCAGCAGGGCAAGTTTTCAGACACTTATCACCATGCTTTCCA
						**CQNPCNCPSGTICN**	GGTGGACAGTGGCACAGTCCAGTTTCCGGATGACAAGATAT
						**PGNGECMKECPPG**	GCCATGCCCACAGTCACAAGTATTTTTGCATTCTATACCGTG
						**LNGTNCDEVCPEGI**	GTATCCGCGAGGACAAGGTTTTTCACAGTACGTGCCGCTCCA
						**YGTNCLKICLCGMR**	TCCAGGCCCACAAATACAGGCACCGCTAATGTGATCACAAG
						**ECDPVTGTCLCDPG**	TCGCTCCATTCTCACAAGGACAGTCTTGTTCGCAATTCTCTCC
						**KMGALCTQSCPKG**	ATACTTGCCAGCACTGCATGGCTTTTCGCATTCACCTCCTGTC

						**KFGKSCLKTCTCEN**	CACCCAGGAGCACATATGCATTCCCCTGTGTATCGGTCGCAT
						**GGTCNPKNGKCSC**	TCTCCTCCGTTCAAACACTCACAATGATGCTTGCACATTGGTC
						**PTGFIGKSCEKKCPP**	CGTAGCTTCCTGGACTGCATGGTTGGTCGCATTTGATCCCTGT
						**DKYGSDCQNQCYC**	CCAACCTGCAGAACAATGGCATCTACCATTGATTGGGTCACA
						**KNGATCDPINGRC**	AGTCGCCCCATTCTTACAATAACACTGATTTTGACAGTCAGA
						**HCSAGWTGIKCDQ**	TCCGTACTTATCAGGAGGACATTTTTTTCACACGATTTTCCG
						**PCSPGSYGPMCKH**	ATGAATCCAGTTGGACAAGAGCATTTGCCATTTTTAGGATTG
						**HCECLNGGECDRY**	CATGTTCCTCCATTTTCGCAAGTGCATGTTTTCAAACAGCTTT
						**TGECICAPGWTGG**	TTCCAAATTTTCCTTTGGGACAACTTTGAGTACACAGAGCAC
						**ECEKPCSAGKYGEN**	CCATTTTTCCTGGATCACACAAACATGTTCCTGTCACTGGGTC
						**CEQDCPCENGATC**	ACATTCTCTCATGCCACATAAGCAAATTTTTAGACAATTTGTG
						**DHISGACICGPGWS**	CCATATATTCCTTCAGGACACACTTCATCACAGTTAGTTCCAT
						**GTYCEKPCPRGYH**	TTAATCCTGGTGGGCACTCTTTCATGCACTCGCCGTTTCCGGG
						**GIECKNTCDCGHGI**	GTTACAGATGGTACCAGAGGGGCAGTTGCAAGGATTCTGGC
						**SCHPETGLCHCPPG**	AAGTCTCTCCCCACCGGTTTGGCGGACATTCAGTCTCACATTT
						**KHGDKCLKTCPAG**	AGCGCCGGTGAAACCCACTTTACATTCACATTTCCCTGTCTTC
						**FYGNHCREHCHCQ**	GAATTACATTTCTGTGTATGTTCCTTGAAGCATTTGCACTGGT
						**NGAVCEPNDGTCH**	TTCGGCAGTTTGGTCCAAAGGTATGTGGGGGACAAGGGATGT
						**CLPGWYGDTCHLA**	TACATAAAGGTCCAAAGAAACCAGGTTCACATTCGCAGTAA
						**CPLGTYGYDCEGV**	CCAAATAACTTATTACAGCGCCCAGTAGGACAACGTTGAGG
						**CLCQNDGNCDPKD**	GCATTTTTTCTCGCACTTTTCACCGAAGCTTCCTGGGGGACAT
						**GRCKCKPGYRGKH**	CCATCTTCACAGCTCTTGCCTTGAATTCCTGGTGGACAGAGG
						**CEMVCKHGFFGLH**	CAAGCGCCTGTCACTGGGTCGCATGAACCGCCATTTTGACAC
						**CAQMCSCKKDEPC**	TTGCATTTTTTCGTGCAGCCATCGCCATAAGTCCCCTTGGCAC
						**HHITGECDCPAGT**	ACGTTTCATTGCAAAGAAGTCCTGTGAATCCTGGTTGGCAAG
						**KGKGCELTCPEGRF**	TACAGCTAAGAGCTCGTATATCACATTTACCTCCATTCTGGC
						**GPDCKEKCKCPSN**	AATTGTCACAATTGAACATGCAGCCAGGTCCGAATGTTCCCC
						**QQCDPVNGKCFCR**	GACCGCAAGATTGTCTGAGGTCTGGTCCTTCGAGAGCTAAAA

						**SGFRGPNCSKICPN<br>GTFGLNCSQLCACE<br>NEADCDPLTGECIC<br>KPGFTGPTCGQECP<br>EGYHGDMCLKRCH<br>CQNGAKCDPETGK<br>CNCPLGFRGEKCEE<br>PCPAGTYGEDCKD<br>DCQCSSNKCDPKT<br>GQCICPPYTSGNEC<br>KEGCPHGFFGPNCL<br>LACHCKHNASCDPI<br>TGCCDCPDGYIGG<br>HCEYECPSGWFGKS<br>CQESCECVGDRPC<br>HPVTGTCSCTAGH<br>HGTKCEKECVLGFY<br>GHGCQEECKCGTH<br>PCDPVTGECHCPPG<br>FIGPHCNELCHDGK<br>YGPKCLQRCHCQH<br>GGTCDTVSGKCTC<br>PPGWIGSTCDAKD<br>GSIYESADNEASNG<br>IS***	AGTGACGGAATTCTTGGTCAAGGAGTGGAACAACAGTTGGC<br>GGTGAAACTTGGAAACGAATCCTTGGCGAATTTTGGTGTGGA<br>GGAGTGTCCGATCGGCTGTGCGACGTCTCGATTAACACTGCC<br>TGATGCGGTGGCACAACATCACAACTTTTTTATCTGAGAGA<br>AGTTTATACCCTTCCAAGCAAAGACAACGATATGATCCAAG<br>GGTGTTTTCACACGTTTGGTCACATCCTCCATTGTTGACAAGG<br>CATTCGTTGTTGTCATTGCAAGTGATCCCATCTCTGTCTAGAG<br>AGTATCCAAGTCGACATGCGCAGGAATAACCACCAGGGCTA<br>TTGTTGCAGTCTTGCTTGCATTGGGATAAACCACGTCGACACT<br>CATCAATATCATTGCAAGTTTTGCCATCCGCGTCGAGCTCGT<br>AACCGGAGTAGCAGCTGCAGATTGGTCCACCCGGTCCGTGG<br>CGACATTTTTGATCACATCCGCCGTTTTTATCTGCACATGTGT<br>TCACCACTTCCATTTCAATTCGGAAACAGGATCTACCGTCATT<br>TCCAAGCTGAAAGGATAGATGACACATGCAAGAATACGATC<br>CAGGAGTATTGTGACACACATGAGAACAGGGGTTATTAGTA<br>CTGCACTCGTCAATATCTTCACAAGTTCTACCATCTCGAGTAA<br>GCTGAAACCCGGGAAGACAGCTGCAGACAGGACGACCGCTT<br>ACACTTTCACATATGTGCGAGCAACCTCCTTTATCACGTGCA<br>CACGCATCAATTTCAGTGCATGTTTTACCATCCCTTGACAACT<br>GGTATCCAGGTAAACAGTTGCAAATTGCTCTACCACCAACAT<br>TATCACATCGCTGAGAACAACCACCATTATCTCGACTACATG<br>CATCTAAAGCTTCGCAGGTTCTTCCATCAGGATTCAGTCTGTA<br>CCCTTGACGGCAGCGGCAACGACTTCTTCCGTGACTGTCTTC<br>GCAAATGTGCTGGCAACCTCCATTATTAATGCTACATTGGTTT<br>CCCATGTCAATGCAAGTCCTTCCATCTGTATGAAGTCGTTGA<br>CCAGGTGAACATTCACAGGAGTATCCGCCATCGTAGTTGATG<br>CATTTATTTTGGCAGCCACCGTTGTGATGAAGGCATTCATCA<br>ATATCAGAGCATGTTCGACCGTCAGCGTTTAGCCTATAACCA

							GGATTGCAACGACAATAGTAGCTTCCTAAGGTATTACAACA<br>ATGATGGTGGCATCCTCCGTTGTTATTGAGGCATTCATTCACA<br>TCATATTGGCATCGATGCCCTTGAAAACCGCTTTGGCAGAGA<br>CATCTGAAGTTGAAGCCATTCCTTCCTCGTCCCAAACATCTTC<br>CGGAGTTATAGCACATCCCATCGTTACATTGGGCTATCAGCA<br>CAAGAGAGAATAATGTCACTCCAAGAGGGCTTAAGCCAAAC<br>ATTTTTGAAATTTAATTCAAAATATAATACGATGAGCAAGTG<br>GGAATCTATATGCTATACGATTATGGCAATAAACAAATGACA<br>TGGGAGTAAAAGTCTGGCAGTCTTTCAAATTTTAAAATAAAA<br>TGAAAATGTCAACAATTCCCTAAAAATTCCGCAGA

PhdGrFCys 11	P81428	6,19E-06	32,292	PF00008<br>PF00594<br>PF00089	71416.29<br>**69324.69**	MFGLSPLGVTLFSLV<br>LIAQC**NDGMCYNS**<br>**GRCLGRGRNGFNF**<br>**RCLCQSGFQGHRC**<br>**QYDVNECLNNNGG**<br>**CHHHCCNTLGSYY**<br>**CRCNPGYRLNADG**<br>**RTCSDIDECLHHNG**<br>**GCQNKCINYDGGY**<br>**SCECSPGQRLHTDG**<br>**RTCIDMGNQCSIN**<br>**NGGCQHICEDSHG**<br>**RSRCRCRQGYRLNP**<br>**DGRTCEALDACSR**<br>**DNGGCSQRCDNV**<br>**GGRAICNCLPGYQL**<br>**SRDGKTCTEIDACA**<br>**RDKGGCSHICESVS**<br>**GRPVCSCLPGFQLT**<br>**RDGRTCEDIDECST**<br>**NNPCSHVCHNTPG**<br>**SYSCMCHLSFQLGN**<br>**DGRSCFRIEMEVVN**<br>**TCADKNGGCDQK**<br>**CRHGPGGPICSCYS**<br>**GYELDADGKTCND**<br>**IDECRRGLSQCKQD**<br>**CNNSPGGYSCACR**	TTTTTTTTATAACCCATTTTTAATGTAGGACATTTTTATATTAC<br>AAGAAAATCATTCAAAACTAATTATCTAATAAAATCATCGGT<br>ATAAATTAAATACCTCGTGAAGCATAAAATTTTACTGTGCAG<br>AGGAAATTCATTTCAAAAGTTGTCTCACTCAGTCAATCTTTCA<br>GATTTGTCAACACTGAATTTTTTTAACCTGTTTTTAAATTCTCT<br>TAATAATTTTTCATTATAATTTCATTTTGAACAGCAAATATTA<br>AATTAGAAATTTCTCAGACTGAATATTTGACATGTATAGCAC<br>AAAATCAGATTAGATATATGCACACATTATATATATCTTACA<br>GAAATTTGTATAAATGTTATACTATAAATTAACAAAATAATT<br>TTTTTAGGGTTTTTTTTTCCTAAAACTGAAATATATTTCAACA<br>AAAGTAAAAAATAGATATTGATTGTATTTCATCTTTTACACA<br>ATTTTCTTGCTCCACATTATTCTTTTTATTTCCTGAGAAAAGTG<br>TTCTGTGTTGAAATCTTGGCTCTCCTAAGCCGAAAGCACAAA<br>CATTATTACAAATGCTTTGATGACTTGAAGGTGTTTTCTCAAA<br>ATGTTTCATAAGCTCATTTTATTTAGCTGATACCATTGGAAGC<br>TTCATTATCTGCAGATTCATAAATTGATCCATCTTTAGCATCA<br>CATGTGGATCCTATCCATCCTGGAGGGCAAGTGCATTTACCA<br>GATACTGTATCACACGTGCCGCCATGCTGGCAATGGCAACGC<br>TGTAGACATTTTGGACCATATTTTCCATCATGGCATAATTCAT<br>TGCAATGTGGTCCAATAAATCCTGGAGGACAATGACATTCTC<br>CAGTAACGGGATCACAAGGATGTGTTCCACACTTGCATTCTT<br>CTTGGCATCCATGACCATAGAAGCCCAAGACGCATTCTTTCT<br>CACATTTGGTACCATGGTGTCCAGCTGTACAGCTGCAAGTAC<br>CTGTCACCGGATGACATGGTCGATCCCCGACGCATTCACAGC<br>TTTCTTGACAAGATTTACCAAACCATCCTGATGGACACTCAT<br>ATTCACAGTGTCCTCCAATATAACCATCTGGGCAGTCACAAC<br>AGCCAGTTATAGGATCACAGGAAGCATTATGTTTACAATGAC<br>ATGCTAGCAAGCAATTTGGTCCAAAGAAGCCATGAGGGCAA

						**LGYSLDRDGITCND**<br>**NNECLVNNGGCD**<br>**QTCENTLGSYRCLC**<br>**LEGYKLLSDKKSCD**<br>**VVPPHQAVLIETSH**<br>**SRSDTPPHQNSPRI**<br>**RFQVSPPTVVPLLD**<br>**QEFRHFLALEGPDL**<br>**RQSCGRGTFGPGC**<br>**MFNCDNCQNGGK**<br>**CDIRALSCTCQPGF**<br>**TGLLCNETCAKGTY**<br>**GDGCTKKCKCQN**<br>**GGSCDPVTGACLC**<br>**PPGIQGKSCEDGCP**<br>**PGSFGEKCEKKCPQ**<br>**RCPTGRCNKLFGYC**<br>**ECEPGFFGPLCNIPC**<br>**PPHTFGPNCRNQC**<br>**KCFKEHTQKCNSK**<br>**CVLKEYMAQIV***	CCTTCTTTACACTCATTCCCACTCGTATACGGTGGACAAATGC<br>ATTGCCCAGTTTTGGGATCACATTTATTACTAGAACACTGGC<br>AATCATCTTTACAGTCTTCTCCATATGTGCCTGCAGGACAAG<br>GTTCTTCGCATTTCTCTCCTCTGAAACCAAGAGGACAATTAC<br>ATTTGCCAGTCTCTGGGTCACATTTCGCCCCGTTCTGACAGTG<br>ACACCTTTTCAGACACATATCTCCGTGATATCCCTCTGGGCAT<br>TCTTGTCCACAAGTAGGTCCAGTGAATCCTGGTTTACAAATG<br>CATTCTCCAGTCAAGGGATCACAATCAGCTTCATTCTCACAT<br>GCGCATAACTGCGAACAATTTAAACCAAACGTTCCATTTGGG<br>CATATTTTACTACAGTTTGGACCTCGAAATCCAGACCTACAA<br>AAGCATTTACCGTTTACTGGATCGCACTGCTGATTCGATGGA<br>CATTTACACTTTTCTTTGCAATCAGGACCAAAGCGTCCTTCCG<br>GGCAAGTTAGTTCGCATCCTTTGCCCTTGGTTCCTGCAGGACA<br>ATCGCATTCGCCTGTAATGTGATGACATGGTTCATCTTTTTA<br>CACGAACACATTTGTGCGCAATGGAGGCCGAAAAATCCATG<br>TTTACAAACCATTTCACAATGCTTTCCTCTATATCCCGGCTTA<br>CACTTGCAACGTCCGTCTTTCGGATCGCAATTACCATCATTCT<br>GACACAGACATACGCCTTCACAATCGTATCCGTAAGTTCCAA<br>GTGGACAAGTTTTCAGACACTTATCACCATGCTTTCCAGGTG<br>GACAGTGGCACAGTCCAGTTTCCGGATGACAAGATATGCCAT<br>GCCCACAGTCACAAGTATTTTTGCATTCTATACCGTGGTATCC<br>GCGAGGACAAGGTTTTTCACAGTACGTGCCGCTCCATCCAGG<br>CCCACAAATACAGGCACCGCTAATGTGATCACAAGTCGCTC<br>CATTCTCACAAGGACAGTCTTGTTCGCAATTCTCTCCATACTT<br>GCCAGCACTGCATGGCTTTTCGCATTCACCTCCTGTCCACCCA<br>GGAGCACATATGCATTCCCCTGTGTATCGGTCGCATTCTCCTC<br>CGTTCAAACACTCACAATGATGCTTGCACATTGGTCCGTAGC<br>TTCCTGGACTGCATGGTTGGTCGCATTTGATCCCTGTCCAACC

							TGCAGAACAATGGCATCTACCATTGATTGGGTCACAAGTCGC<br>CCCATTCTTACAATAACACTGATTTTGACAGTCAGATCCGTA<br>CTTATCAGGAGGACATTTTTTTTCACACGATTTTCCGATGAAT<br>CCAGTTGGACAAGAGCATTTGCCATTTTTAGGATTGCATGTTC<br>CTCCATTTTCGCAAGTGCATGTTTTCAAACAGCTTTTTCCAAA<br>TTTTCCTTTGGGACAACTTTGAGTACACAGAGCACCCATTTTT<br>CCTGGATCACACAAACATGTTCCTGTCACTGGGTCACATTCT<br>CTCATGCCACATAAGCAAATTTTTAGACAATTTGTGCCATAT<br>ATTCCTTCAGGACACACTTCGAATTACATTTCTGTGTATGTTC<br>CTTGAAGCATTTGCACTGGTTTCGGCAGTTTGGTCCAAAGGT<br>ATGTGGGGGACAAGGGATGTTACATAAAGGTCCAAAGAAAC<br>CAGGTTCACATTCGCAGTAACCAAATAACTTATTACAGCGCC<br>CAGTAGGACAACGTTGAGGGCATTTTTTCTCGCACTTTTCACC<br>GAAGCTTCCTGGGGGACATCCATCTTCACAGCTCTTGCCTTG<br>AATTCCTGGTGGACAGAGGCAAGCGCCTGTCACTGGGTCGC<br>ATGAACCGCCATTTTGACACTTGCATTTTTTCGTGCAGCCATC<br>GCCATAAGTCCCCTTGGCACACGTTTCATTGCAAAGAAGTCC<br>TGTGAATCCTGGTTGGCAAGTACAGCTAAGAGCTCGTATATC<br>ACATTTACCTCCATTCTGGCAATTGTCACAATTGAACATGCA<br>GCCAGGTCCGAATGTTCCCCGACCGCAAGATTGTCTGAGGTC<br>TGGTCCTTCGAGAGCTAAAAAGTGACGGAATTCTTGGTCAAG<br>GAGTGGAACAACAGTTGGCGGTGAAACTTGGAAACGAATCC<br>TTGGCGAATTTTGGTGTGGAGGAGTGTCCGATCGGCTGTGCG<br>ACGTCTCGATTAACACTGCCTGATGCGGTGGCACAACATCAC<br>AACTTTTTTTATCTGAGAGAAGTTTATACCCTTCCAAGCAAAG<br>ACAACGATATGATCCAAGGGTGTTTTCACACGTTTGGTCACA<br>TCCTCCATTGTTGACAAGGCATTCGTTGTTGTCATTGCAAGTG<br>ATCCCATCTCTGTCTAGAGAGTATCCAAGTCGACATGCGCAG

							GAATAACCACCAGGGCTATTGTTGCAGTCTTGCTTGCATTGG<br>GATAAACCACGTCGACACTCATCAATATCATTGCAAGTTTTG<br>CCATCCGCGTCGAGCTCGTAACCGGAGTAGCAGCTGCAGATT<br>GGTCCACCCGGTCCGTGGCGACATTTTTGATCACATCCGCCG<br>TTTTTATCTGCACATGTGTTCACCACTTCCATTTCAATTCGGA<br>AACAGGATCTACCGTCATTTCCAAGCTGAAAGGATAGATGA<br>CACATGCAAGAATACGATCCAGGAGTATTGTGACACACATG<br>AGAACAGGGGTTATTAGTACTGCACTCGTCAATATCTTCACA<br>AGTTCTACCATCTCGAGTAAGCTGAAACCCGGGAAGACAGC<br>TGCAGACAGGACGACCGCTTACACTTTCACATATGTGCGAGC<br>AACCTCCTTTATCACGTGCACACGCATCAATTTCAGTGCATGT<br>TTTACCATCCCTTGACAACTGGTATCCAGGTAAACAGTTGCA<br>AATTGCTCTACCACCAACATTATCACATCGCTGAGAACAACC<br>ACCATTATCTCGACTACATGCATCTAAAGCTTCGCAGGTTCTT<br>CCATCAGGATTCAGTCTGTACCCTTGACGGCAGCGGCAACGA<br>CTTCTTCCGTGACTGTCTTCGCAAATGTGCTGGCAACCTCCAT<br>TATTAATGCTACATTGGTTTCCCATGTCAATGCAAGTCCTTCC<br>ATCTGTATGAAGTCGTTGACCAGGTGAACATTCACAGGAGTA<br>TCCGCCATCGTAGTTGATGCATTTATTTTGGCAGCCACCGTTG<br>TGATGAAGGCATTCATCAATATCAGAGCATGTTCGACCGTCA<br>GCGTTTAGCCTATAACCAGGATTGCAACGACAATAGTAGCTT<br>CCTAAGGTATTACAACAATGATGGTGGCATCCTCCGTTGTTA<br>TTGAGGCATTCATTCACATCATATTGGCATCGATGCCCTTGAA<br>AACCGCTTTGGCAGAGACATCTGAAGTTGAAGCCATTCCTTC<br>CTCGTCCCAAACATCTTCCGGAGTTATAGCACATCCCATCGT<br>TACATTGGGCTATCAGCACAAGAGAGAATAATGTCACTCCA<br>AGAGGGCTTAAGCCAAACATTTTTGAAATTTAATTCAAAATA<br>TAATACGATGAGCAAGTGGGAATCTATATGCTATACGATTAT

							GGCAATAAACAAATGACATGGGAGTAAAAGTCTGGCAGTCTTTCAAATTTTAAAATAAAATGAAAATGTCAACAATTCCCTAAAAATTCCGCAGA

PhdGrFCys 12	Q1L658	1,35E-05	38,824	PF00008<br>PF00594<br>PF00089	122571,4 7	NIDECSTNNPCSHV<br>CHNTPGSYSCMCHL<br>SFQLGNDGRSCFRIE<br>MEVVNTCADKNGG<br>CDQKCRHGPGGPIC<br>SCYSGYELDADGKT<br>CNDIDECRRGLSQC<br>KQDCNNSPGGYSCA<br>CRLGYSLDRDGITCN<br>DNNECLVNNGGCD<br>QTCENTLGSYRCLCL<br>EGYKLLSDKKSCDV<br>VPPHQAVLIETSHSR<br>SDTPPHQNSPRIRFQ<br>VSPPTVVPLLDQEFR<br>HFLALEGPDLRQSC<br>GRGTFGPGCMFNCD<br>NCQNGGKCDIRALS<br>CTCQPGFTGLLCNE<br>TCAKGTYGDGCTKK<br>CKCQNGGSCDPVTG<br>ACLCPPGIQGKSCED<br>GCPPGSFGEKCEKK<br>CPQRCPTGRCNKLF<br>GYCECEPGFFGPLCN<br>IPCPPHTFGPNCRNQ<br>CKCFKEHTQKCNSK<br>TGKCECKVGFTGAK	TTTTTTTTATAACCCATTTTTAATGTAGGACATTTTTATATTAC<br>AAGAAAATCATTCAAAACTAATTATCTAATAAAATCATCGGT<br>ATAAATTAAATACCTCGTGAAGCATAAAATTTTACTGTGCAG<br>AGGAAATTCATTTCAAAAGTTGTCTCACTCAGTCAATCTTTCA<br>GATTTGTCAACACTGAATTTTTTTAACCTGTTTTTAAATTCTCT<br>TAATAATTTTTCATTATAATTTCATTTTGAACAGCAAATATTA<br>AATTAGAAATTTCTCAGACTGAATATTTGACATGTATAGCAC<br>AAAATCAGATTAGATATATGCACACATTATATATATCTTACA<br>GAAATTTGTATAAATGTTATACTATAAATTAACAAAATAATT<br>TTTTTAGGGTTTTTTTTTCCTAAAACTGAAATATATTTCAACA<br>AAAGTAAAAAAATAGATATTGATTGTATTTCATCTTTTACACA<br>ATTTTCTTGCTCCACATTATTCTTTTTATTTCCTGAGAAAAGTG<br>TTCTGTGTTGAAATCTTGGCTCTCCTAAGCCGAAAGCACAAA<br>CATTATTACAAATGCTTTGATGACTTGAAGGTGTTTTCTCAAA<br>ATGTTTCATAAGCTCATTTTATTTAGCTGATACCATTGGAAGC<br>TTCATTATCTGCAGATTCATAAATTGATCCATCTTTAGCATCA<br>CATGTGGATCCTATCCATCCTGGAGGGCAAGTGCATTTACCA<br>GATACTGTATCACACGTGCCGCCATGCTGGCAATGGCAACGC<br>TGTAGACATTTTGGACCATATTTTCCATCATGGCATAATTCAT<br>TGCAATGTGGTCCAATAAATCCTGGAGGACAATGACATTCTC<br>CAGTAACGGGATCACAAGGATGTGTTCCACACTTGCATTCTT<br>CTTGGCATCCATGACCATAGAAGCCCAAGACGCATTCTTTCT<br>CACATTTGGTACCATGGTGTCCAGCTGTACAGCTGCAAGTAC<br>CTGTCACCGGATGACATGGTCGATCCCCGACGCATTCACAGC<br>TTTCTTGACAAGATTTACCAAACCATCCTGATGGACACTCAT<br>ATTCACAGTGTCCTCCAATATAACCATCTGGGCAGTCACAAC<br>AGCCAGTTATAGGATCACAGGAAGCATTATGTTTACAATGAC<br>ATGCTAGCAAGCAATTTGGTCCAAAGAAGCCATGAGGGCAA

						CETECPPNRWGETC	CCTTCTTTACACTCATTCCCACTCGTATACGGTGGACAAATGC
						QNPCNCPSGTICNP	ATTGCCCAGTTTTGGGATCACATTTATTACTAGAACACTGGC
						GNGECMKECPPGLN	AATCATCTTTACAGTCTTCTCCATATGTGCCTGCAGGACAAG
						GTNCDEVCPEGIYG	GTTCTTCGCATTTCTCTCCTCTGAAACCAAGAGGACAATTAC
						TNCLKICLCGMREC	ATTTGCCAGTCTCTGGGTCACATTTCGCCCCGTTCTGACAGTG
						DPVTGTCLCDPGKM	ACACCTTTTCAGACACATATCTCCGTGATATCCCTCTGGGCAT
						GALCTQSCPKGKFG	TCTTGTCCACAAGTAGGTCCAGTGAATCCTGGTTTACAAATG
						KSCLKTCTCENGGT	CATTCTCCAGTCAAGGGATCACAATCAGCTTCATTCTCACAT
						CNPKNGKCSCPTGFI	GCGCATAACTGCGAACAATTTAAACCAAACGTTCCATTTGGG
						GKSCEKKCPPDKYG	CATATTTTACTACAGTTTGGACCTCGAAATCCAGACCTACAA
						SDCQNQCYCKNGA	AAGCATTTACCGTTTACTGGATCGCACTGCTGATTCGATGGA
						TCDPINGRCHCSAG	CATTTACACTTTTCTTTGCAATCAGGACCAAAGCGTCCTTCCG
						WTGIKCDQPCSPGS	GGCAAGTTAGTTCGCATCCTTTGCCCTTGGTTCCTGCAGGACA
						YGPMCKHHCECLN	ATCGCATTCGCCTGTAATGTGATGACATGGTTCATCTTTTTA
						GGECDRYTGECICAP	CACGAACACATTTGTGCGCAATGGAGGCCGAAAAATCCATG
						GWTGGECEKPCSAG	TTTACAAACCATTTCACAATGCTTTCCTCTATATCCCGGCTTA
						KYGENCEQDCPCEN	CACTTGCAACGTCCGTCTTTCGGATCGCAATTACCATCATTCT
						GATCDHISGACICGP	GACACAGACATACGCCTTCACAATCGTATCCGTAAGTTCCAA
						GWSGTYCEKPCPRG	GTGGACAAGTTTTCAGACACTTATCACCATGCTTTCCAGGTG
						YHGIECKNTCDCGH	GACAGTGGCACAGTCCAGTTTCCGGATGACAAGATATGCCAT
						GISCHPETGLCHCPP	GCCCACAGTCACAAGTATTTTTGCATTCTATACCGTGGTATCC
						GKHGDKCLKTCPLG	GCGAGGACAAGGTTTTTCACAGTACGTGCCGCTCCATCCAGG
						TYGYDCEGVCLCQN	CCCACAAATACAGGCACCGCTAATGTGATCACAAGTCGCTC
						DGNCDPKDGRCKC	CATTCTCACAAGGACAGTCTTGTTCGCAATTCTCTCCATACTT
						KPGYRGKHCEMVC	GCCAGCACTGCATGGCTTTTCGCATTCACCTCCTGTCCACCCA
						KHGFFGLHCAQMC	GGAGCACATATGCATTCCCCTGTGTATCGGTCGCATTCTCCTC
						SCKKDEPCHHITGEC	CGTTCAAACACTCACAATGATGCTTGCACATTGGTCCGTAGC
						DCPAGTKGKGCELT	TTCCTGGACTGCATGGTTGGTCGCATTTGATCCCTGTCCAACC

						CPEGRFGPDCKEKC	TGCAGAACAATGGCATCTACCATTGATTGGGTCACAAGTCGC
						KCPSNQQCDPVNG	CCCATTCTTACAATAACACTGATTTTGACAGTCAGATCCGTA
						KCFCRSGFRGPNCSK	CTTATCAGGAGGACATTTTTTTTCACACGATTTTCCGATGAAT
						ICPNGTFGLNCSQLC	CCAGTTGGACAAGAGCATTTGCCATTTTTAGGATTGCATGTTC
						ACENEADCDPLTGE	CTCCATTTTCGCAAGTGCATGTTTTCAAACAGCTTTTTCCAAA
						CICKPGFTGPTCGQE	TTTTCCTTTGGGACAACTTTGAGTACACAGAGCACCCATTTTT
						CPEGYHGDMCLKR	CCTGGATCACACAAACATGTTCCTGTCACTGGGTCACATTCT
						CHCQNGAKCDPET	CTCATGCCACATAAGCAAATTTTTAGACAATTTGTGCCATAT
						GKCNCPLGFRGEKC	ATTCCTTCAGGACACACTTCATCACAGTTAGTTCCATTTAATC
						EEPCPAGTYGEDCK	CTGGTGGGCACTCTTTCATGCACTCGCCGTTTCCGGGGTTACA
						DDCQCSSNKCDPKT	GATGGTACCAGAGGGGCAGTTGCAAGGATTCTGGCAAGTCT
						GQCICPPYTSGNECK	CTCCCCACCGGTTTGGCGGACATTCAGTCTCACATTTAGCGC
						EGCPHGFFGPNCLL	CGGTGAAACCCACTTTACATTCACATTTCCCTGTCTTCGAATT
						ACHCKHNASCDPIT	ACATTTCTGTGTATGTTCCTTGAAGCATTTGCACTGGTTTCGG
						GCCDCPDGYIGGHC	CAGTTTGGTCCAAAGGTATGTGGGGGACAAGGGATGTTACAT
						EYECPSGWFGKSCQ	AAAGGTCCAAAGAAACCAGGTTCACATTCGCAGTAACCAAA
						ESCECVGDRPCHPV	TAACTTATTACAGCGCCCAGTAGGACAACGTTGAGGGCATTT
						TGTCSCTAGHHGTK	TTTCTCGCACTTTTCACCGAAGCTTCCTGGGGGACATCCATCT
						CEKECVLGFYGHGC	TCACAGCTCTTGCCTTGAATTCCTGGTGGACAGAGGCAAGCG
						QEECKCGTHPCDPV	CCTGTCACTGGGTCGCATGAACCGCCATTTTGACACTTGCATT
						TGECHCPPGFIGPHC	TTTTCGTGCAGCCATCGCCATAAGTCCCCTTGGCACACGTTTC
						NELCHDGKYGPKCL	ATTGCAAAGAAGTCCTGTGAATCCTGGTTGGCAAGTACAGCT
						QRCHCQHGGTCDT	AAGAGCTCGTATATCACATTTACCTCCATTCTGGCAATTGTCA
						VSGKCTCPPGWIGST	CAATTGAACATGCAGCCAGGTCCGAATGTTCCCCGACCGCA
						CDAKDGSIYESADN	AGATTGTCTGAGGTCTGGTCCTTCGAGAGCTAAAAAGTGACG
						EASNGIS*	GAATTCTTGGTCAAGGAGTGGAACAACAGTTGGCGGTGAAA
							CTTGGAAACGAATCCTTGGCGAATTTTGGTGTGGAGGAGTGT
							CCGATCGGCTGTGCGACGTCTCGATTAACACTGCCTGATGCG

							GTGGCACAACATCACAACTTTTTTTATCTGAGAGAAGTTTAT<br>ACCCTTCCAAGCAAAGACAACGATATGATCCAAGGGTGTTTT<br>CACACGTTTGGTCACATCCTCCATTGTTGACAAGGCATTCGTT<br>GTTGTCATTGCAAGTGATCCCATCTCTGTCTAGAGAGTATCCA<br>AGTCGACATGCGCAGGAATAACCACCAGGGCTATTGTTGCA<br>GTCTTGCTTGCATTGGGATAAACCACGTCGACACTCATCAAT<br>ATCATTGCAAGTTTTGCCATCCGCGTCGAGCTCGTAACCGGA<br>GTAGCAGCTGCAGATTGGTCCACCCGGTCCGTGGCGACATTT<br>TTGATCACATCCGCCGTTTTTATCTGCACATGTGTTCACCACT<br>TCCATTTCAATTCGGAAACAGGATCTACCGTCATTTCCAAGC<br>TGAAAGGATAGATGACACATGCAAGAATACGATCCAGGAGT<br>ATTGTGACACACATGAGAACAGGGGTTATTAGTACTGCACTC<br>GTCAATATTTCAATTTCAGAGTTCTTTCGTTTCTCAAAATTTCA<br>TTTTATTTATTTCAGTCTATCATAAGACTTATTGTTCGTAAACA<br>GTACACAGAAATCCCAAACAACTTCAGATTGTGTCGCAACGT<br>GCAGGCCCTCTATCGCCCGTTACGCAATGCCAAGTTCTTGGA<br>ACGCGCCTCGTGC

PhdGrFCys 13	P81428	6,45E-06	32,292	PF00008<br>PF00594<br>PF00089	151078.27<br>**148986.68**	MFGLSPLGVTLFSLV<br>LIAQC**NDGMCYNS**<br>**GRCLGRGRNGFNF**<br>**RCLCQSGFQGHRC**<br>**QYDVNECLNNNGG**<br>**CHHHCCNTLGSYY**<br>**CRCNPGYRLNADG**<br>**RTCSDIDECLHHNG**<br>**GCQNKCINYDGGY**<br>**SCECSPGQRLHTDG**<br>**RTCIDMGNQCSIN**<br>**NGGCQHICEDSHG**<br>**RSRCRCRQGYRLNP**<br>**DGRTCEALDACSR**<br>**DNGGCSQRCDNV**<br>**GGRAICNCLPGYQL**<br>**SRDGKTCTEIDACA**<br>**RDKGGCSHICESVS**<br>**GRPVCSCLPGFQLT**<br>**RDGRTCEDIDECST**<br>**NNPCSHVCHNTPG**<br>**SYSCMCHLSFQLGN**<br>**DGRSCFRIEMEVVN**<br>**TCADKNGGCDQK**<br>**CRHGPGGPICSCYS**<br>**GYELDADGKTCND**<br>**IDECRRGLSQCKQD**<br>**CNNSPGGYSCACR**	TTTTTTTTATAACCCATTTTTAATGTAGGACATTTTTATATTAC<br>AAGAAAATCATTCAAAACTAATTATCTAATAAAATCATCGGT<br>ATAAATTAAATACCTCGTGAAGCATAAAATTTTACTGTGCAG<br>AGGAAATTCATTTCAAAAGTTGTCTCACTCAGTCAATCTTTCA<br>GATTTGTCAACACTGAATTTTTTTAACCTGTTTTTAAATTCTCT<br>TAATAATTTTTCATTATAATTTCATTTTGAACAGCAAATATTA<br>AATTAGAAATTTCTCAGACTGAATATTTGACATGTATAGCAC<br>AAAATCAGATTAGATATATGCACACATTATATATATCTTACA<br>GAAATTTGTATAAATGTTATACTATAAATTAACAAAATAATT<br>TTTTTAGGGTTTTTTTTTCCTAAAACTGAAATATATTTCAACA<br>AAAGTAAAAAATAGATATTGATTGTATTTCATCTTTTACACA<br>ATTTTCTTGCTCCACATTATTCTTTTTATTTCCTGAGAAAAGTG<br>TTCTGTGTTGAAATCTTGGCTCTCCTAAGCCGAAAGCACAAA<br>CATTATTACAAATGCTTTGATGACTTGAAGGTGTTTTCTCAAA<br>ATGTTTCATAAGCTCATTTTATTTAGCTGATACCATTGGAAGC<br>TTCATTATCTGCAGATTCATAAATTGATCCATCTTTAGCATCA<br>CATGTGGATCCTATCCATCCTGGAGGGCAAGTGCATTTACCA<br>GATACTGTATCACACGTGCCGCCATGCTGGCAATGGCAACGC<br>TGTAGACATTTTGGACCATATTTTCCATCATGGCATAATTCAT<br>TGCAATGTGGTCCAATAAATCCTGGAGGACAATGACATTCTC<br>CAGTAACGGGATCACAAGGATGTGTTCCACACTTGCATTCTT<br>CTTGGCATCCATGACCATAGAAGCCCAAGACGCATTCTTTCT<br>CACATTTGGTACCATGGTGTCCAGCTGTACAGCTGCAAGTAC<br>CTGTCACCGGATGACATGGTCGATCCCCGACGCATTCACAGC<br>TTTCTTGACAAGATTTACCAAACCATCCTGATGGACACTCAT<br>ATTCACAGTGTCCTCCAATATAACCATCTGGGCAGTCACAAC<br>AGCCAGTTATAGGATCACAGGAAGCATTATGTTTACAATGAC<br>ATGCTAGCAAGCAATTTGGTCCAAAGAAGCCATGAGGGCAA

						**LGYSLDRDGITCND**	CCTTCTTTACACTCATTCCCACTCGTATACGGTGGACAAATGC
						**NNECLVNNGGCD**	ATTGCCCAGTTTTGGGATCACATTTATTACTAGAACACTGGC
						**QTCENTLGSYRCLC**	AATCATCTTTACAGTCTTCTCCATATGTGCCTGCAGGACAAG
						**LEGYKLLSDKKSCD**	GTTCTTCGCATTTCTCTCCTCTGAAACCAAGAGGACAATTAC
						**VVPPHQAVLIETSH**	ATTTGCCAGTCTCTGGGTCACATTTCGCCCCGTTCTGACAGTG
						**SRSDTPPHQNSPRI**	ACACCTTTTCAGACACATATCTCCGTGATATCCCTCTGGGCAT
						**RFQVSPPTVVPLLD**	TCTTGTCCACAAGTAGGTCCAGTGAATCCTGGTTTACAAATG
						**QEFRHFLALEGPDL**	CATTCTCCAGTCAAGGGATCACAATCAGCTTCATTCTCACAT
						**RQSCGRGTFGPGC**	GCGCATAACTGCGAACAATTTAAACCAAACGTTCCATTTGGG
						**MFNCDNCQNGGK**	CATATTTTACTACAGTTTGGACCTCGAAATCCAGACCTACAA
						**CDIRALSCTCQPGF**	AAGCATTTACCGTTTACTGGATCGCACTGCTGATTCGATGGA
						**TGLLCNETCAKGTY**	CATTTACACTTTTCTTTGCAATCAGGACCAAAGCGTCCTTCCG
						**GDGCTKKCKCQN**	GGCAAGTTAGTTCGCATCCTTTGCCCTTGGTTCCTGCAGGACA
						**GGSCDPVTGACLC**	ATCGCATTCGCCTGTAATGTGATGACATGGTTCATCTTTTTA
						**PPGIQGKSCEDGCP**	CACGAACACATTTGTGCGCAATGGAGGCCGAAAAATCCATG
						**PGSFGEKCEKKCPQ**	TTTACAAACCATTTCACAATGCTTTCCTCTATATCCCGGCTTA
						**RCPTGRCNKLFGYC**	CACTTGCAACGTCCGTCTTTCGGATCGCAATTACCATCATTCT
						**ECEPGFFGPLCNIPC**	GACACAGACATACGCCTTCACAATCGTATCCGTAAGTTCCAA
						**PPHTFGPNCRNQC**	GTGGACAAGTTTTCAGACACTTATCACCATGCTTTCCAGGTG
						**KCFKEHTQKCNSK**	GACAGTGGCACAGTCCAGTTTCCGGATGACAAGATATGCCAT
						**TGKCECKVGFTGA**	GCCCACAGTCACAAGTATTTTTGCATTCTATACCGTGGTATCC
						**KCETECPPNRWGET**	GCGAGGACAAGGTTTTTCACAGTACGTGCCGCTCCATCCAGG
						**CQNPCNCPSGTICN**	CCCACAAATACAGGCACCGCTAATGTGATCACAAGTCGCTC
						**PGNGECMKECPPG**	CATTCTCACAAGGACAGTCTTGTTCGCAATTCTCTCCATACTT
						**LNGTNCDEVCPEGI**	GCCAGCACTGCATGGCTTTTCGCATTCACCTCCTGTCCACCCA
						**YGTNCLKICLCGMR**	GGAGCACATATGCATTCCCCTGTGTATCGGTCGCATTCTCCTC
						**ECDPVTGTCLCDPG**	CGTTCAAACACTCACAATGATGCTTGCACATTGGTCCGTAGC
						**KMGALCTQSCPKG**	TTCCTGGACTGCATGGTTGGTCGCATTTGATCCCTGTCCAACC

						**KFGKSCLKTCTCEN**	TGCAGAACAATGGCATCTACCATTGATTGGGTCACAAGTCGC
						**GGTCNPKNGKCSC**	CCCATTCTTACAATAACACTGATTTTGACAGTCAGATCCGTA
						**PTGFIGKSCEKKCPP**	CTTATCAGGAGGACATTTTTTTTCACACGATTTTCCGATGAAT
						**DKYGSDCQNQCYC**	CCAGTTGGACAAGAGCATTTGCCATTTTTAGGATTGCATGTTC
						**KNGATCDPINGRC**	CTCCATTTTCGCAAGTGCATGTTTTCAAACAGCTTTTTCCAAA
						**HCSAGWTGIKCDQ**	TTTTCCTTTGGGACAACTTTGAGTACACAGAGCACCCATTTTT
						**PCSPGSYGPMCKH**	CCTGGATCACACAAACATGTTCCTGTCACTGGGTCACATTCT
						**HCECLNGGECDRY**	CTCATGCCACATAAGCAAATTTTTAGACAATTTGTGCCATAT
						**TGECICAPGWTGG**	ATTCCTTCAGGACACACTTCATCACAGTTAGTTCCATTTAATC
						**ECEKPCSAGKYGEN**	CTGGTGGGCACTCTTTCATGCACTCGCCGTTTCCGGGGTTACA
						**CEQDCPCENGATC**	GATGGTACCAGAGGGGCAGTTGCAAGGATTCTGGCAAGTCT
						**DHISGACICGPGWS**	CTCCCCACCGGTTTGGCGGACATTCAGTCTCACATTTAGCGC
						**GTYCEKPCPRGYH**	CGGTGAAACCCACTTTACATTCACATTTCCCTGTCTTCGAATT
						**GIECKNTCDCGHGI**	ACATTTCTGTGTATGTTCCTTGAAGCATTTGCACTGGTTTCGG
						**SCHPETGLCHCPPG**	CAGTTTGGTCCAAAGGTATGTGGGGGACAAGGGATGTTACAT
						**KHGDKCLKTCPLG**	AAAGGTCCAAAGAAACCAGGTTCACATTCGCAGTAACCAAA
						**TYGYDCEGVCLCQ**	TAACTTATTACAGCGCCCAGTAGGACAACGTTGAGGGCATTT
						**NDGNCDPKDGRC**	TTTCTCGCACTTTTCACCGAAGCTTCCTGGGGGACATCCATCT
						**KCKPGYRGKHCEM**	TCACAGCTCTTGCCTTGAATTCCTGGTGGACAGAGGCAAGCG
						**VCKHGFFGLHCAQ**	CCTGTCACTGGGTCGCATGAACCGCCATTTTGACACTTGCATT
						**MCSCKKDEPCHHI**	TTTTCGTGCAGCCATCGCCATAAGTCCCCTTGGCACACGTTTC
						**TGECDCPAGTKGK**	ATTGCAAAGAAGTCCTGTGAATCCTGGTTGGCAAGTACAGCT
						**GCELTCPEGRFGPD**	AAGAGCTCGTATATCACATTTACCTCCATTCTGGCAATTGTCA
						**CKEKCKCPSNQQC**	CAATTGAACATGCAGCCAGGTCCGAATGTTCCCCGACCGCA
						**DPVNGKCFCRSGF**	AGATTGTCTGAGGTCTGGTCCTTCGAGAGCTAAAAAGTGACG
						**RGPNCSKICPNGTF**	GAATTCTTGGTCAAGGAGTGGAACAACAGTTGGCGGTGAAA
						**GLNCSQLCACENE**	CTTGGAAACGAATCCTTGGCGAATTTTGGTGTGGAGGAGTGT
						**ADCDPLTGECICKP**	CCGATCGGCTGTGCGACGTCTCGATTAACACTGCCTGATGCG

						**GFTGPTCGQECPEG**<br>**YHGDMCLKRCHC**<br>**QNGAKCDPETGKC**<br>**NCPLGFRGEKCEEP**<br>**CPAGTYGEDCKDD**<br>**CQCSSNKCDPKTG**<br>**QCICPPYTSGNECK**<br>**EGCPHGFFGPNCLL**<br>**ACHCKHNASCDPI**<br>**TGCCDCPDGYIGG**<br>**HCEYECPSGWFGKS**<br>**CQESCECVGDRPC**<br>**HPVTGTCSCTAGH**<br>**HGTKCEKECVLGFY**<br>**GHGCQEECKCGTH**<br>**PCDPVTGECHCPPG**<br>**FIGPHCNELCHDGK**<br>**YGPKCLQRCHCQH**<br>**GGTCDTVSGKCTC**<br>**PPGWIGSTCDAKD**<br>**GSIYESADNEASNG**<br>**IS***	GTGGCACAACATCACAACTTTTTTTATCTGAGAGAAGTTTAT<br>ACCCTTCCAAGCAAAGACAACGATATGATCCAAGGGTGTTTT<br>CACACGTTTGGTCACATCCTCCATTGTTGACAAGGCATTCGTT<br>GTTGTCATTGCAAGTGATCCCATCTCTGTCTAGAGAGTATCCA<br>AGTCGACATGCGCAGGAATAACCACCAGGGCTATTGTTGCA<br>GTCTTGCTTGCATTGGGATAAACCACGTCGACACTCATCAAT<br>ATCATTGCAAGTTTTGCCATCCGCGTCGAGCTCGTAACCGGA<br>GTAGCAGCTGCAGATTGGTCCACCCGGTCCGTGGCGACATTT<br>TTGATCACATCCGCCGTTTTTATCTGCACATGTGTTCACCACT<br>TCCATTTCAATTCGGAAACAGGATCTACCGTCATTTCCAAGC<br>TGAAAGGATAGATGACACATGCAAGAATACGATCCAGGAGT<br>ATTGTGACACACATGAGAACAGGGGTTATTAGTACTGCACTC<br>GTCAATATCTTCACAAGTTCTACCATCTCGAGTAAGCTGAAA<br>CCCGGGAAGACAGCTGCAGACAGGACGACCGCTTACACTTT<br>CACATATGTGCGAGCAACCTCCTTTATCACGTGCACACGCAT<br>CAATTTCAGTGCATGTTTTACCATCCCTTGACAACTGGTATCC<br>AGGTAAACAGTTGCAAATTGCTCTACCACCAACATTATCACA<br>TCGCTGAGAACAACCACCATTATCTCGACTACATGCATCTAA<br>AGCTTCGCAGGTTCTTCCATCAGGATTCAGTCTGTACCCTTGA<br>CGGCAGCGGCAACGACTTCTTCCGTGACTGTCTTCGCAAATG<br>TGCTGGCAACCTCCATTATTAATGCTACATTGGTTTCCCATGT<br>CAATGCAAGTCCTTCCATCTGTATGAAGTCGTTGACCAGGTG<br>AACATTCACAGGAGTATCCGCCATCGTAGTTGATGCATTTAT<br>TTTGGCAGCCACCGTTGTGATGAAGGCATTCATCAATATCAG<br>AGCATGTTCGACCGTCAGCGTTTAGCCTATAACCAGGATTGC<br>AACGACAATAGTAGCTTCCTAAGGTATTACAACAATGATGGT<br>GGCATCCTCCGTTGTTATTGAGGCATTCATTCACATCATATTG<br>GCATCGATGCCCTTGAAAACCGCTTTGGCAGAGACATCTGAA

							GTTGAAGCCATTCCTTCCTCGTCCCAAACATCTTCCGGAGTTATAGCACATCCCATCGTTACATTGGGCTATCAGCACAAGAGAGAATAATGTCACTCCAAGAGGGCTTAAGCCAAACATTTTTGAAATTTAATTCAAAATATAATACGATGAGCAAGTGGGAATCTATATGCTATACGATTATGGCAATAAACAAATGACATGGGAGTAAAAGTCTGGCAGTCTTTCAAATTTTAAAATAAAATGAAAATGTCAACAATTCCCTAAAAATTCCGCAGA

PhdGrFCys 14	P81428	6,37E-06	32,292	PF00008<br>PF00594<br>PF00089	71416.29<br>**69324.69**	MFGLSPLGVTLFSLV<br>LIAQC**NDGMCYNS**<br>**GRCLGRGRNGFNF**<br>**RCLCQSGFQGHRC**<br>**QYDVNECLNNNGG**<br>**CHHHCCNTLGSYY**<br>**CRCNPGYRLNADG**<br>**RTCSDIDECLHHNG**<br>**GCQNKCINYDGGY**<br>**SCECSPGQRLHTDG**<br>**RTCIDMGNQCSIN**<br>**NGGCQHICEDSHG**<br>**RSRCRCRQGYRLNP**<br>**DGRTCEALDACSR**<br>**DNGGCSQRCDNV**<br>**GGRAICNCLPGYQL**<br>**SRDGKTCTEIDACA**<br>**RDKGGCSHICESVS**<br>**GRPVCSCLPGFQLT**<br>**RDGRTCEDIDECST**<br>**NNPCSHVCHNTPG**<br>**SYSCMCHLSFQLGN**<br>**DGRSCFRIEMEVVN**<br>**TCADKNGGCDQK**<br>**CRHGPGGPICSCYS**<br>**GYELDADGKTCND**<br>**IDECRRGLSQCKQD**<br>**CNNSPGGYSCACR**	TTTTTTTTATAACCCATTTTTAATGTAGGACATTTTTATATTAC<br>AAGAAAATCATTCAAAACTAATTATCTAATAAAATCATCGGT<br>ATAAATTAAATACCTCGTGAAGCATAAAATTTTACTGTGCAG<br>AGGAAATTCATTTCAAAAGTTGTCTCACTCAGTCAATCTTTCA<br>GATTTGTCAACACTGAATTTTTTTAACCTGTTTTTAAATTCTCT<br>TAATAATTTTTCATTATAATTTCATTTTGAACAGCAAATATTA<br>AATTAGAAATTTCTCAGACTGAATATTTGACATGTATAGCAC<br>AAAATCAGATTAGATATATGCACACATTATATATATCTTACA<br>GAAATTTGTATAAATGTTATACTATAAATTAACAAAATAATT<br>TTTTTAGGGTTTTTTTTTCCTAAAACTGAAATATATTTCAACA<br>AAAGTAAAAAATAGATATTGATTGTATTTCATCTTTTACACA<br>ATTTTCTTGCTCCACATTATTCTTTTTATTTCCTGAGAAAAGTG<br>TTCTGTGTTGAAATCTTGGCTCTCCTAAGCCGAAAGCACAAA<br>CATTATTACAAATGCTTTGATGACTTGAAGGTGTTTTCTCAAA<br>ATGTTTCATAAGCTCATTTTATTTAGCTGATACCATTGGAAGC<br>TTCATTATCTGCAGATTCATAAATTGATCCATCTTTAGCATCA<br>CATGTGGATCCTATCCATCCTGGAGGGCAAGTGCATTTACCA<br>GATACTGTATCACACGTGCCGCCATGCTGGCAATGGCAACGC<br>TGTAGACATTTTGGACCATATTTTCCATCATGGCATAATTCAT<br>TGCAATGTGGTCCAATAAATCCTGGAGGACAATGACATTCTC<br>CAGTAACGGGATCACAAGGATGTGTTCCACACTTGCATTCTT<br>CTTGGCATCCATGACCATAGAAGCCCAAGACGCATTCTTTCT<br>CACATTTGGTACCATGGTGTCCAGCTGTACAGCTGCAAGTAC<br>CTGTCACCGGATGACATGGTCGATCCCCGACGCATTCACAGC<br>TTTCTTGACAAGATTTACCAAACCATCCTGATGGACACTCAT<br>ATTCACAGTGTCCTCCAATATAACCATCTGGGCAGTCACAAC<br>AGCCAGTTATAGGATCACAGGAAGCATTATGTTTACAATGAC<br>ATGCTAGCAAGCAATTTGGTCCAAAGAAGCCATGAGGGCAA

						**LGYSLDRDGITCND<br>NNECLVNNGGCD<br>QTCENTLGSYRCLC<br>LEGYKLLSDKKSCD<br>VVPPHQAVLIETSH<br>SRSDTPPHQNSPRI<br>RFQVSPPTVVPLLD<br>QEFRHFLALEGPDL<br>RQSCGRGTFGPGC<br>MFNCDNCQNGGK<br>CDIRALSCTCQPGF<br>TGLLCNETCAKGTY<br>GDGCTKKCKCQN<br>GGSCDPVTGACLC<br>PPGIQGKSCEDGCP<br>PGSFGEKCEKKCPQ<br>RCPTGRCNKLFGYC<br>ECEPGFFGPLCNIPC<br>PPHTFGPNCRNQC<br>KCFKEHTQKCNSK<br>CVLKEYMAQIV***	CCTTCTTTACACTCATTCCCACTCGTATACGGTGGACAAATGC<br>ATTGCCCAGTTTTGGGATCACATTTATTACTAGAACACTGGC<br>AATCATCTTTACAGTCTTCTCCATATGTGCCTGCAGGACAAG<br>GTTCTTCGCATTTCTCTCCTCTGAAACCAAGAGGACAATTAC<br>ATTTGCCAGTCTCTGGGTCACATTTCGCCCCGTTCTGACAGTG<br>ACACCTTTTCAGACACATATCTCCGTGATATCCCTCTGGGCAT<br>TCTTGTCCACAAGTAGGTCCAGTGAATCCTGGTTTACAAATG<br>CATTCTCCAGTCAAGGGATCACAATCAGCTTCATTCTCACAT<br>GCGCATAACTGCGAACAATTTAAACCAAACGTTCCATTTGGG<br>CATATTTTACTACAGTTTGGACCTCGAAATCCAGACCTACAA<br>AAGCATTTACCGTTTACTGGATCGCACTGCTGATTCGATGGA<br>CATTTACACTTTTCTTTGCAATCAGGACCAAAGCGTCCTTCCG<br>GGCAAGTTAGTTCGCATCCTTTGCCCTTGGTTCCTGCAGGACA<br>ATCGCATTCGCCTGTAATGTGATGACATGGTTCATCTTTTTA<br>CACGAACACATTTGTGCGCAATGGAGGCCGAAAAATCCATG<br>TTTACAAACCATTTCACAATGCTTTCCTCTATATCCCGGCTTA<br>CACTTGCAACGTCCGTCTTTCGGATCGCAATTACCATCATTCT<br>GACACAGACATACGCCTTCACAATCGTATCCGTAAGTTCCAA<br>GTGGACAAGCCAAGTGGCACGTATCGCCATACCAGCCTGGT<br>AGACAATGACACGTGCCGTCGTTTGGTTCACAAACTGCACCG<br>TTCTGGCAATGGCAATGTTCTCTGCAGTGATTTCCGTAAAAA<br>CCAGCAGGGCAAGTTTTCAGACACTTATCACCATGCTTTCCA<br>GGTGGACAGTGGCACAGTCCAGTTTCCGGATGACAAGATAT<br>GCCATGCCCACAGTCACAAGTATTTTTGCATTCTATACCGTG<br>GTATCCGCGAGGACAAGGTTTTTCACAGTACGTGCCGCTCCA<br>TCCAGGCCCACAAATACAGGCACCGCTAATGTGATCACAAG<br>TCGCTCCATTCTCACAAGGACAGTCTTGTTCGCAATTCTCTCC<br>ATACTTGCCAGCACTGCATGGCTTTTCGCATTCACCTCCTGTC

							CACCCAGGAGCACATATGCATTCCCCTGTGTATCGGTCGCAT<br>TCTCCTCCGTTCAAACACTCACAATGATGCTTGCACATTGGTC<br>CGTAGCTTCCTGGACTGCATGGTTGGTCGCATTTGATCCCTGT<br>CCAACCTGCAGAACAATGGCATCTACCATTGATTGGGTCACA<br>AGTCGCCCCATTCTTACAATAACACTGATTTTGACAGTCAGA<br>TCCGTACTTATCAGGAGGACATTTTTTTTCACACGATTTTCCG<br>ATGAATCCAGTTGGACAAGAGCATTTGCCATTTTTAGGATTG<br>CATGTTCCTCCATTTTCGCAAGTGCATGTTTTCAAACAGCTTT<br>TTCCAAATTTTCCTTTGGGACAACTTTGAGTACACAGAGCAC<br>CCATTTTTCCTGGATCACACAAACATGTTCCTGTCACTGGGTC<br>ACATTCTCTCATGCCACATAAGCAAATTTTTAGACAATTTGTG<br>CCATATATTCCTTCAGGACACACTTCGAATTACATTTCTGTGT<br>ATGTTCCTTGAAGCATTTGCACTGGTTTCGGCAGTTTGGTCCA<br>AAGGTATGTGGGGGACAAGGGATGTTACATAAAGGTCCAAA<br>GAAACCAGGTTCACATTCGCAGTAACCAAATAACTTATTACA<br>GCGCCCAGTAGGACAACGTTGAGGGCATTTTTTCTCGCACTT<br>TTCACCGAAGCTTCCTGGGGGACATCCATCTTCACAGCTCTT<br>GCCTTGAATTCCTGGTGGACAGAGGCAAGCGCCTGTCACTGG<br>GTCGCATGAACCGCCATTTTGACACTTGCATTTTTTCGTGCAG<br>CCATCGCCATAAGTCCCCTTGGCACACGTTTCATTGCAAAGA<br>AGTCCTGTGAATCCTGGTTGGCAAGTACAGCTAAGAGCTCGT<br>ATATCACATTTACCTCCATTCTGGCAATTGTCACAATTGAACA<br>TGCAGCCAGGTCCGAATGTTCCCCGACCGCAAGATTGTCTGA<br>GGTCTGGTCCTTCGAGAGCTAAAAAGTGACGGAATTCTTGGT<br>CAAGGAGTGGAACAACAGTTGGCGGTGAAACTTGGAAACGA<br>ATCCTTGGCGAATTTTGGTGTGGAGGAGTGTCCGATCGGCTG<br>TGCGACGTCTCGATTAACACTGCCTGATGCGGTGGCACAACA<br>TCACAACTTTTTTTATCTGAGAGAAGTTTATACCCTTCCAAGC

							AAAGACAACGATATGATCCAAGGGTGTTTTCACACGTTTGGT<br>CACATCCTCCATTGTTGACAAGGCATTCGTTGTTGTCATTGCA<br>AGTGATCCCATCTCTGTCTAGAGAGTATCCAAGTCGACATGC<br>GCAGGAATAACCACCAGGGCTATTGTTGCAGTCTTGCTTGCA<br>TTGGGATAAACCACGTCGACACTCATCAATATCATTGCAAGT<br>TTTGCCATCCGCGTCGAGCTCGTAACCGGAGTAGCAGCTGCA<br>GATTGGTCCACCCGGTCCGTGGCGACATTTTTGATCACATCC<br>GCCGTTTTTATCTGCACATGTGTTCACCACTTCCATTTCAATTC<br>GGAAACAGGATCTACCGTCATTTCCAAGCTGAAAGGATAGA<br>TGACACATGCAAGAATACGATCCAGGAGTATTGTGACACAC<br>ATGAGAACAGGGGTTATTAGTACTGCACTCGTCAATATCTTC<br>ACAAGTTCTACCATCTCGAGTAAGCTGAAACCCGGGAAGAC<br>AGCTGCAGACAGGACGACCGCTTACACTTTCACATATGTGCG<br>AGCAACCTCCTTTATCACGTGCACACGCATCAATTTCAGTGC<br>ATGTTTTACCATCCCTTGACAACTGGTATCCAGGTAAACAGT<br>TGCAAATTGCTCTACCACCAACATTATCACATCGCTGAGAAC<br>AACCACCATTATCTCGACTACATGCATCTAAAGCTTCGCAGG<br>TTCTTCCATCAGGATTCAGTCTGTACCCTTGACGGCAGCGGC<br>AACGACTTCTTCCGTGACTGTCTTCGCAAATGTGCTGGCAAC<br>CTCCATTATTAATGCTACATTGGTTTCCCATGTCAATGCAAGT<br>CCTTCCATCTGTATGAAGTCGTTGACCAGGTGAACATTCACA<br>GGAGTATCCGCCATCGTAGTTGATGCATTTATTTTGGCAGCC<br>ACCGTTGTGATGAAGGCATTCATCAATATCAGAGCATGTTCG<br>ACCGTCAGCGTTTAGCCTATAACCAGGATTGCAACGACAATA<br>GTAGCTTCCTAAGGTATTACAACAATGATGGTGGCATCCTCC<br>GTTGTTATTGAGGCATTCATTCACATCATATTGGCATCGATGC<br>CCTTGAAAACCGCTTTGGCAGAGACATCTGAAGTTGAAGCC<br>ATTCCTTCCTCGTCCCAAACATCTTCCGGAGTTATAGCACATC

							CCATCGTTACATTGGGCTATCAGCACAAGAGAGAATAATGTCACTCCAAGAGGGCTTAAGCCAAACATTTTTGAAATTTAATTCAAAATATAATACGATGAGCAAGTGGGAATCTATATGCTATACGATTATGGCAATAAACAAATGACATGGGAGTAAAAGTCTGGCAGTCTTTCAAATTTTAAAATAAAATGAAAATGTCAACAATTCCCTAAAAATTCCGCAGA

PhdGrFCys 15	Q58L93	4,30E-05	39,583	PF00008 PF00594 PF00089	10791,4	ATLNRFKQIELHCGVLLDLGICSSQPCLNGGTCVPTNSDFMCHCPPRYQGNFCEIDLDPCISNPCLNGGKCHNVDHKFICSCSTGVTGPRCEYGRHCKP	GCAACATTGAACAGATTCAAGCAAATAGAACTGCACTGTGGAGTTTTACTTGACTTAGGAATATGCAGCAGTCAGCCGTGTTTAAATGGTGGTACTTGTGTTCCTACAAATAGTGATTTTATGTGCCATTGTCCACCTCGATATCAAGGAAATTTCTGTGAAATTGACTTGGATCCCTGTATATCAAATCCTTGCTTGAATGGAGGAAAATGCCACAACGTTGATCATAAATTTATTTGTTCTTGCAGTACTGGGGTAACAGGACCTCGATGTGAATATGGTCGGCACTGTAAACCTAG
PhdGrFCys 16	Q58L93	3,50E-06	54,054	PF00008 PF00594 PF00089	31841,63	YCQNDINECSSQPCQNGATCNDLVGEYSCDCPPGFEGVNCELNIDDCASNPCRHGGTCHDLVNKFVCSCPHGTLGMLCEMNVDECFEGSCHHDGYCIDKIGGYECQCRPGYVGPRCEGDVNECLSDPCNRPGTQDCVQLVNDYRCDCKPGYMGRHCEIKLNYCATNPCMNGGVCTTNPNGTPFCICPEGFWGDTCAMTNTTCASYPCKNGGQCKPQHNGYTCVCPQGTGGRHCEKDLVDDCVSAPCLHGGECIDLLGKFECNCPPEWNGYRCDIYDPT	GTTATTGCCAGAATGATATAAATGAATGTAGTTCTCAACCATGTCAGAATGGCGCTACTTGCAATGATTTAGTGGGTGAATATTCATGTGACTGTCCTCCTGGCTTTGAAGGTGTGAATTGTGAATTAAATATTGATGATTGCGCTTCCAATCCTTGTCGCCATGGAGGTACTTGCCATGACTTAGTCAACAAATTTGTATGCTCATGCCCCCATGGAACTCTTGGCATGTTATGTGAGATGAATGTGGATGAATGTTTTGAAGGTTCTTGCCATCATGATGGTTATTGTATAGATAAAATTGGAGGTTATGAATGCCAGTGTCGACCAGGTTATGTGGGACCAAGATGTGAAGGGGATGTCAATGAATGCCTTTCTGATCCCTGCAATCGTCCTGGGACTCAAGATTGCGTACAGCTAGTCAATGACTATCGATGTGACTGCAAACCAGGTTATATGGGCAGGCACTGTGAAATAAAGCTAAATTATTGTGCTACTAATCCCTGCATGAATGGAGGAGTATGCACAACTAATCCAAATGGAACTCCCTTCTGTATTTGTCCTGAGGGTTTCTGGGGAGATACTTGTGCTATGACAAATACTACCTGTGCTAGTTATCCCTGCAAAAATGGAGGCCAGTGCAAACCTCAGCATAATGGCTACACCTGTGTTTGTCCCCAAGGTACAGGTGGACGTCATTGTGAAAAAGACCTTGTAGATGACTGTGTATCTGCACCTTGTCTCCATGGGGGTGAATGTATAGATCTGTTAGGTAAATTTGAATGCAATTGTCCTCCTGAAT

						FGGGIINVMPPQEPP Q	GGAATGGTTATCGTTGTGATATCTATGATCCAACTTTCGGTGG TGGAATCATTAATGTCATGCCTCCTCAAGAACCTCCTCAGAA
PhdGrFCys 17	A6MFK7	2,99E-09	53,659	PF00008 PF00594 PF00089	11791,13	SQPCLNNGVCVDLI NQFHCNCSNTGFM GLFCEINIDECASNP CQNGAWCEDKIKD YHCHCYAGYSGKN CEIDIEECASSPCLNS ALCLENSNQTLYDM NYLGFFQS	AATGATTGAAAGAATCCCAGATAGTTCATATCATATAATGTC TGATTTGAATTTTCTAAACAAAGAGCACTATTTAAACAAGGT GATGAGGCACATTCTTCAATATCTATCTCACAGTTCTTTCCAC TATAACCAGCATAGCAATGACAGTGATAATCCTTAATCTTAT CTTCACACCATGCTCCATTTTGGCAAGGGTTTGAGGCACACT CATCAATATTTATTTCACAGAATAAACCCATGAATCCTGTGTT GGAGCAGTTGCAATGAAATTGATTGATTAAATCTACACAGAC TCCATTGTTGAGGCAAGGTTGGGA
PhdGrFCys 18	Q58L95	3,55E-06	47,368	PF00008 PF00594 PF00089	13638,21	GYTGSNCQFHINEC DSQPCQSGGTCIDH VGYYTCHCPFGYTG LHCDTFVDWCSTSP CMNGATCMQLNNT YGCTCQPGWTGML CDVSMVSCADAAA QKGIDIHDLCKNGGI	TGGGTATACAGGTTCTAATTGCCAGTTCCATATAAATGAATG TGATTCACAACCATGCCAGAGTGGAGGAACTTGCATTGATCA TGTTGGATATTACACTTGCCACTGCCCATTTGGTTACACAGGG TTGCATTGTGATACATTTGTGGATTGGTGCAGTACAAGTCCCT GTATGAATGGTGCCACCTGTATGCAATTGAATAATACTTATG GCTGTACCTGTCAACCTGGATGGACTGGAATGCTCTGTGATG TGTCTATGGTCTCTTGTGCTGATGCTGCAGCTCAGAAAGGTAT TGACATCCATGACTTATGCAAAAATGGAGGAATCTGTGAAA

						CENIGNSHRCICASGYEG	ATATTGGAAACAGTCATCGATGTATTTGTGCTAGTGGGTATGAAGGAA
PhdGrFCys 19	Q58L95	1,37E-05	47,368	PF00008 PF00594 PF00089	27404,07	YRGHNCEENINECEENNPCYTGSQCFDRYGGYICNCPPGFGGKNCDQEVNECDSNPCQNMGTCIDNVNGYLCDCLIGFAGINCEENIDDCSGVTCPANSHCVDGINSFSCMCNPGYAGAVPHCQEIDECAESPCLNGGTCQDQLNGFSCVCADGFVGRHCEINIDECETQPCQNGATCKPKPLRDYECICAPGCHGTNCEYVIDACYGNPCENGGTCKVLEAGRFSCHCPAGYEGDRCETNIDDCVDNKCENNAT	ATGTCGCATTATTTTCGCATTTGTTGTCAACACAGTCATCGATGTTAGTTTCGCAGCGATCACCCTCGTAGCCAGCAGGGCAATGGCAGCTGAAACGTCCAGCCTCAAGAACTTTGCAGGTACCCCCATTTTCACAAGGGTTTCCATAACAAGCATCAATCACATATTCACAATTTGTTCCGTGGCAGCCAGGAGCACAGATGCATTCATAATCTCGTAAAGGCTTAGGTTTACACGTAGCTCCATTTTGACATGGCTGTGTCTCACATTCATCAATATTAATTTCACAGTGTCTACCAACAAATCCATCAGCACAGACACAAGAAAATCCATTTAGTTGGTCTTGGCAAGTGCCACCATTTAAACAAGGACTTTCTGCACATTCATCAATTTCTTGACAATGAGGGACAGCACCTGCATATCCAGGATTGCACATACAAGAAAATGAATTAATTCCATCAACACAATGGCTGTTAGCTGGACATGTGACTCCACTACAATCATCTATATTTTCTTCACAATTTATACCAGCAAATCCAATTAAACAATCACATAAATATCCATTAACATTATCAATACATGTGCCCATATTTTGGCATGGGTTTGAATCGCATTCATTTACTTCTTGATCACAATTTTTCCCTCCAAACCCAGGAGGACAGTTGCATATATAGCCCCCATATCGATCAAAGCACTGACTACCAGTATAACATGGGTTGTTTTCTTCACACTCATTGATATTTTCTTCACAGTTGTGTCCTCTATA

PhdGrFCys 20	A6MFK7	1,99E-08	56,098	PF00008<br>PF00594<br>PF00089	17557,34	LDFACSCELGFTGHL<br>CDEDIDECIVSSPCR<br>NGATCVNTNGSYTC<br>TCAKGYEGKDCLVN<br>TDDCRPFPCQNGGT<br>CLDGIGEYTCMCVD<br>GFGGKHCESDINEC<br>ESSPCLNGATCNDY<br>VNSYTCTCPLGFSGT<br>NCQINDEDCTASSC<br>MNGGTCIDGINIYIC<br>QCTPGYTGS	TTAGAACCTGTATACCCAGGTGTGCACTGACATATATAGATA<br>TTTATTCCATCAATGCATGTTCCACCATTCATACAAGAGCTGG<br>CAGTACAATCTTCATCATTAATCTGACAGTTGGTTCCACTGA<br>ATCCAAGAGGGCAAGTACAGGTATAACTGTTGACATAATCA<br>TTACATGTAGCTCCATTCAAACAGGGAGAGCTCTCACATTCA<br>TTTATGTCACTTTCACAATGCTTCCCTCCAAATCCATCAACAC<br>ACATGCATGTGTATTCTCCAATTCCATCTAAGCATGTACCTCC<br>ATTCTGACAAGGAAAGGGACGACAGTCATCTGTATTGACCA<br>GGCAATCTTTTCCTTCATAGCCTTTTGCACAAGTACAAGTATA<br>GGATCCATTAGTATTCACACAAGTAGCACCATTACGACAAG<br>GTGATGATACAATACACTCATCAATATCTTCATCACATAAAT<br>GACCTGTAAAACCAAGTTCACAAGAGCAGGCAAAATCCAG
PhdGrFCys 21	Q58L93	1,92E-10	57,895	PF00008<br>PF00594<br>PF00089	15038,39	SDVNECASNPCLNG<br>GSCEDEVNKFTCHC<br>LDGYGGHRCEVEID<br>ECQSNPCQHGGSCQ<br>DRLASYECSCLPGYS<br>GQNCETNIDDCASS<br>PCLNGGSCIDLVDSY<br>QCVCEVPFTGPRCD<br>TELDPCSPNKCKNN<br>ARCTPSSNYLDF	GCAAAATCCAGATAATTAGATGATGGTGTACATCTTGCATTA<br>TTCTTACATTTATTAGGAGAGCAAGGGTCTAATTCAGTATCA<br>CATCTGGGACCAGTGAAAGGCACTTCACAAACACATTGGTA<br>GGAGTCGACAAGATCAATACATGATCCACCATTTAAGCAAG<br>GGCTACTGGCACAGTCATCAATATTTGTTTCACAGTTCTGTCC<br>ACTATAGCCTGGTAAGCAGGAGCATTCATAAGATGCAAGAC<br>GGTCTTGACAGCTGCCACCATGCTGGCAAGGATTAGATTGGC<br>ACTCATCAATTTCAACCTCACATCGATGACCTCCATACCCAT<br>CTAAGCAATGGCAAGTAAATTTGTTGACTTCATCTTCACAAC<br>TGCCACCATTAAGACAAGGATTACTAGCACATTCATTTACAT<br>CAGA
PhdGrFCys 22	Q56VR3	2,22E-05	30,769	PF00008<br>PF00594<br>PF00089	7797,66	YNINECSSNPCRNG<br>AVCIDGINSYTCQCV<br>PGYTGLHCETNINE<br>CASNPCANGGICTD	ATATAACATCAATGAATGTTCCAGTAATCCATGTCGAAATGG<br>AGCAGTTTGTATAGATGGCATTAATTCATATACTTGTCAATGT<br>GTTCCAGGATATACAGGTTTGCACTGTGAAACAAACATCAAT<br>GAATGTGCTAGTAACCCTTGTGCAAATGGAGGAATTTGTACA

						MINGFKCDCPRGYFDA	GATATGATTAATGGCTTTAAATGTGATTGTCCTAGAGGTTACTTTGATGCTCG
PhdGrFCys 23	A6MFK8	4,65E-06	33,871	PF00008 PF00594 PF00089	8137,26	GYAGPFCEHKTCFANPCEYGSTCLPNMDYGYLCICPYGKHGKNCELDLPVTIPSFYGNLMGYSSYVQYQLLAD	TGGATATGCAGGCCCATTTTGTGAACACAAAACATGCTTTGCTAATCCCTGTGAATATGGAAGCACTTGTCTGCCGAATATGGATTATGGCTATCTTTGCATTTGCCCATATGGAAAACATGGAAAAAATTGTGAACTTGATCTACCGGTTACTATTCCTTCCTTTTATGGGAATTTAATGGGATATTCTTCTTATGTTCAGTATCAGTTGTTGGCTGATTA
PhdGrFCys 24	Q1L658	2,45E-05	38,095	PF00008 PF00594 PF00089	10624,27	SYACHRDFGYSVKDHEQGCTDDNECETGLSECDEHADCVNTLGSYECTCLDGFRGDGYECRDINECLRDNGGCDPDAACINTMGSFQCSCDEGFTGNG	TCCATTTCCAGTGAATCCCTCATCACAAGAACACTGAAAAGAGCCCATAGTGTTAATGCAGGCAGCATCAGGATCACATCCACCATTATCTCTAAGGCACTCATTAATATCTCTACATTCATATCCATCTCCTCTAAAACCATCTAAACATGTGCATTCATAAGATCCTAGAGTATTGACACAATCGGCATGTTCATCACATTCACTCAGCCCTGTTTCACATTCATTATCATCTGTGCATCCTTGTTCATGATCTTTTACAGAATATCCAAAATCACGATGACATGCATATGAG
PhdGrFCys 25	Q58L96	1,73E-05	29,670	PF00008 PF00594 PF00089	12752,12	NGGTCVDGLNSFQCFCQEGWEGRYCQINKNDCDPNPCHNNGSCIDAIADYMCNCKNEWKGKTCNLRHGHCDLSTCQNGGTCLDLGETFVCHCKSGWEGHTCQIAKNPSCHSNPCHN	AATGGAGGAACATGTGTTGATGGTCTGAATTCCTTCCAGTGCTTTTGCCAAGAAGGTTGGGAAGGAAGATATTGCCAAATTAATAAAAATGACTGTGATCCAAATCCATGCCACAATAATGGAAGCTGCATTGATGCAATTGCTGATTATATGTGCAACTGTAAAAATGAATGGAAGGGAAAAACTTGCAACTTACGTCATGGCCACTGTGATTTGAGTACATGCCAAAATGGAGGTACATGCCTTGATTTAGGAGAAACTTTTGTATGTCATTGTAAATCAGGATGGGAAGGACATACTTGTCAAATAGCTAAAAATCCAAGTTGTCATTCTAACCCATGCCATAACGG

PhdGrFCys 26	A6MFK8	1,05E-06	50,000	PF00008<br>PF00594<br>PF00089	17315,17	FHCNCTKGFTGPRCEININECDSNPCKNEGTCLDERGGFRCVCMPGYSGSHCEEDINDCSPNPCLNGGICSDRINGFKCLCPIGFSGKKCEVNEDDCVNHPCQNGGNCSDGIASFTCHCNPGFTGTHCETNINDCQSSPCHHGQCIDGTNSFICVCDPGYKG	AGTCCTTTATAACCAGGGTCACACACACAAATGAAAGAATTTGTACCATCAATGCATTGTCCATGATGACATGGAGAAGATTGACAATCATTAATGTTAGTTTCACAATGTGTTCCGGTAAAACCTGGATTGCAGTGGCAAGTAAAGCTAGCAATTCCATCAGAACAATTTCCACCATTCTGGCATGGATGATTAACACAATCATCTTCATTAACTTCACACTTCTTTCCCGAAAACCCAATGGGACACAGACATTTAAAACCATTGATTCTATCACTACATATACCACCATTAAGGCAAGGATTTGGAGAGCAGTCGTTGATGTCTTCTTCACAGTGAGAACCACTGTAGCCTGGCATGCACACACAACGGAATCCACCTCTCTCATCAAGACATGTCCCTTCGTTTTTGCACGGATTAGAATCACATTCGTTAATATTTATTTCACATCGTGGCCCTGTAAATCCTTTTGTGCAGTTACAGTGAAA
PhdGrFCys 27	A6MFK7	4,80E-07	40,278	PF00008<br>PF00594<br>PF00089	9927,93	TFSCSCPVGYSASLCEIAVPNSCDSNPCQNGGTCNLVTLDNYTCSCTTGYRGHHCEDVDHCASRPCRNGATCVSRRDSYKCTCAEGFTGSTCTT	ACGTTTTCGTGTTCGTGTCCTGTGGGCTATTCTGCATCGCTATGCGAGATAGCCGTCCCAAACAGCTGTGACAGCAATCCATGCCAGAACGGTGGAACTTGTAATCTAGTCACATTAGACAATTACACCTGTTCCTGTACGACTGGCTACAGAGGACATCATTGTGAAGATGTGGACCATTGTGCATCCAGACCATGTCGTAATGGAGCTACTTGCGTATCACGAAGAGATAGCTACAAATGTACCTGCGCCGAAGGTTTTACAGGGAGCACATGTACGACAG
PhdGrFCys 28	Q58L93	3,83E-08	36,986	PF00008<br>PF00594<br>PF00089	13080,45	NRITNRGMRRYFAKVKMLEDKVNHLTELLSTNECSSNPCRNGGTCVDSYNGYVCNCPSNWEGPTCENDVNECALYSGTAFGCQHGATCTNLPGSYRCDCAANWFGIHCTEQLDDCNAAS	GGGAGGCCGCGTTGCAGTCATCCAGCTGTTCCGTGCAGTGGATGCCAAACCAGTTCGCGGCACAGTCACACCTGTAACTTCCAGGGAGATTTGTGCACGTGGCTCCGTGCTGACAACCAAACGCTGTTCCAGAATACAAAGCACATTCATTGACGTCGTTTTCGCACGTTGGACCCTCCCAGTTGGACGGACAGTTGCAGACGTATCCGTTGTAGGAGTCCACGCAGGTTCCTCCGTTCCTACAGGGGTTGCTGGAACACTCGTTCGTAGAAAGCAACTCCGTCAAGTGATTGACTTTGTCTTCCAGCATTTTGACCTTAGCAAAGTATCTTCTCATGCCCCTGTTGGTGATGCGATT

PhdGrFCys 29	A6MFK8	9,90E-05	33,333	PF00008<br>PF00594<br>PF00089	11128,39	PNPCRNGAPCTDM VNDFFCDCLPGWEG KTCVDNINECQSQP CMHGQCVDFLNNF TCNCDPGFSGILCDI NINECDSSPCFNGGT CTDLINDFSCQCPEN FMG	ACCCAATCCATGCAGAAATGGTGCCCCATGTACTGACATGGT AAATGACTTCTTTTGTGACTGCCTCCCAGGCTGGGAGGGCAA GACTTGTGTTGACAATATCAATGAATGTCAGTCCCAACCCTG CATGCATGGTCAATGTGTTGATTTCCTGAACAATTTTACTTGT AATTGTGATCCTGGATTCTCTGGTATTCTCTGTGATATTAATA TAAATGAATGTGATTCAAGTCCCTGTTTTAATGGCGGTACTTG CACTGATTTAATAAATGACTTTTCCTGCCAGTGTCCAGAAAA TTTTATGGGG
PhdGrFCys 30	P86468	4,65E-05	50,000	PF00008	7736.45	GTHCEIMVDNTFCD FMPCRNGGTCNINH SRDSYVCTCPQGFA GTDCELNIDECQSAP CQNNGSCTDEINAY	GTATATGCATTTATTTCATCAGTACAAGAGCCATTATTCTGAC AAGGTGCAGACTGGCATTCATCAATATTCAATTCACAATCAG TCCCAGCAAATCCTTGTGGACAAGTGCATACATAACTGTCTC TGGAGTGGTTGATATTACATGTTCCTCCATTACGACATGGCAT AAAATCACAGAATGTATTATCTACCATGATCTCACAGTGTGT GCCAG
PhdGrFCys 31	P86468	2,54E-05	60,000	PF00008	13246.58	QNCESQYIPCEPSPC ENGGTCHPLDSLSY QCACPEGFTGTNCE VNIDDCPGHICQNG AVCIDGINTYTCQCP PTYTGTYCSKDVDE CSVRPSVCKNGATCI NTVGSYSCICVNGW SGPDCSENID	GACAAAACTGTGAATCGCAGTACATTCCCTGTGAACCAAGC CCTTGTGAAAATGGTGGGACTTGTCATCCTTTAGATTCTTTAA GTTACCAGTGCGCTTGCCCAGAAGGTTTTACTGGTACAAATT GTGAAGTAAACATAGATGATTGCCCTGGGCATATTTGTCAGA ATGGAGCTGTTTGCATCGATGGCATCAACACTTACACATGTC AGTGCCCACCAACATATACTGGTACTTACTGCAGTAAAGATG TAGATGAGTGTTCCGTACGACCTAGTGTGTGCAAAAATGGTG CTACATGTATCAACACTGTAGGAAGTTACTCATGCATCTGTG TCAATGGCTGGTCAGGTCCAGATTGCAGTGAGAACATCGATG
Insulin-like growth factor binding protein							
**Transcript ID**	**Uniprot ID reference protein**	**e-value**	**Identity (%)**	**PFAM domain**	**MW (Da)**	**Amino acid sequence**	**Nucleotide sequence**

PhdGrFIns01	P0CJ14	3,17E-13	38,372	PF00219	12054.63<br>**10193.26**	MKVLLLLSIGIFALT<br>GSC**HMLTCLCNSED**<br>**CSDPDDCKYGKTL**<br>**DPCECCQECLKGPG**<br>**EKCGGPWNMDGV**<br>**CAENYTCIRESDYN**<br>**THEDYEGGHKTTP**<br>**APLDEFESGICRKIS***	AAAATAGGCAGATTCACTATATTACCATTATTGGAGAATTCGTTTAAAGATAAGAACATTTTGCTCTCTACTGATAATACCTGTCAAAAATGAAATATGGCTCGCACTTGACTTAATTTCGTCACACCACTTCCTCGTTCTAAATCTGTTTGTGAGGTTCGAAGAAAAAGCCTGTTGTTGCAAAAGTTTTTCCCGGTATTTTAGTTATTGCAGATCAAACTGAAATCCGTGGCTCGAGATATTTTCATTCTCCTGATAGTTATCGGTCTGTTTTTGACAGAAAAATGAAGGTTCTCCTGTTGCTTTCTATCGGAATTTTTGCACTCACAGGGAGCTGCCATATGCTGACCTGTCTTTGTAATTCCGAAGATTGTTCTGATCCTGATGACTGTAAATACGGTAAAACATTAGACCCTTGCGAATGCTGTCAAGAATGTCTCAAGGGTCCTGGTGAGAAATGTGGTGGACCATGGAACATGGATGGTGTTTGCGCTGAAAATTACACCTGTATTCGAGAATCAGACTACAACACGCATGAAGATTATGAAGGTGGTCATAAGACGACTCCTGCACCTCTGGACGAATTTGAAAGTGGAATCTGCCGAAAAATATCATAGATTTCGCGCAGACGATAACTTCACGCAAATGTATTTTTTATGAAATAAAATGGACGTACATAAACGTTTGTTTTACTGAACAATTGACATGTTGCATATTCGTTTATTAGTAACTTTAGCGGGTGACCAATATTATCTTTATCATAGGTAGTCGTAACAACATGAAAGGATGGCATTGCAATCTTGTAGAAAATGATAAGTTAAGGAATTAAACTATTTCATTAAATACATGCCCTAATGCAGTAATTTAACGAGGCAAATAAAATCAAGCATTTTTACTTGTTATTCGATTAAAAAGATCTGAAAGCTCTTTTTCTGCACATGCTGTCTTTTACGTGAGACTTTAAAAGGGGATTAATACGAAACTCTTGCGCAGTAAAGTAAAAGCTTTATGACATGGAATGACTTTCGACAATTATCTATTTTGCTAGACTGTCTTAGACGTCGACAGAATACTTGTTTCAAAATTCGCGTAGAGTTGCCAAAGTCATCTAACGAGAGGGTTTGAAATTGCTTGAGG

PhdGrFIns02	P0CJ14	2,93E-11	44,304	PF00219	10341.02<br><br>**8376.51**	MKTFLLLAVGTALIGSCLM**LSCIPCDKDNCTDPGPCKYGTTLDPCQCCLECRKGPGERCGGPWNLFGVCAQHLICAREGDPSTPVTQGEEGICRQRS***	TTTTTTTTCAATGAAAGAAAGTTTATTATCAGTATATCAATATTATTTCAACAACAGCCTTTTTTCTGGAAATAGACGTCAACAACTCTTTTATGACCTTTGCCGACAAATTCCCTCTTCACCCTGAGTCACGGGCGTAGAAGGATCTCCTTCTCGGGCGCAAATGAGATGCTGAGCACAGACACCGAACAAATTCCACGGTCCTCCACATCTCTCGCCTGGACCCTTGCGACATTCTAGACAGCACTGGCAAGGATCTAATGTTGTCCCATACTTGCAAGGACCAGGGTCAGTACAGTTATCTTTATCACACGGGATACAGCTCAACATCAGGCAACTCCCTATAAGTGCAGTTCCAACAGCGAGCAGCAGGAAGGTCTTCATTTTTGTGCTATAGAAGAGATAAATGTCTTATACAAATTTGAAGTAACTATTTCATCCGCAATGGCTTAAATACCG
PhdGrFIns03	P0CJ14	1,81E-10	37,500	PF00219	10225.08<br><br>**8260.56**	MKTFLLLAVGTALIGSCLM**LSCIPCDKDNCTDPGPCKYGRTLDACQCCQVCRKGPGERCGGPSNLFGVCAKHLICAEGGNPAKPVTLDTEGICQKKW***	AGTATATCAATTTTATTTCAAAAACAACCATTTTGCTGGATATTGACGCCGAGTATTTTACCACTTTTTCTGACAAATTCCCTCTGTATCCAGAGTCACAGGCTTAGCAGGATTTCCTCCTTCGGCACAAATGAGATGCTTAGCACAGACACCGAACAAATTAGACGGTCCTCCACATCTCTCGCCTGGACCCTTGCGACATACTTGACAGCACTGGCAAGCATCTAATGTTCTACCATACTTGCAAGGACCAGGGTCAGTACAGTTATCTTTATCACACGGGATACAGCTCAACATCAGGCAACTCCCTATAAGTGCAGTTCCAACAGCGAGCAGCAGGAAGGTCTTCATTTTTGTGCTATAGAAGAGATAAATGTCTTATACAAATTTGAAGTAACTATTTCATCCGCAATGGCTTAAATACCG

PhdGrFIns04	P0CJ14	8,07E-06	45,652	PF00219	11318.82<br>**9536.57**	MKTLLSFAIGMMLVASS**FEHHSDDHFECAECKSELCEDPRPCEYGVTKDACDCCLTCFQGPDDYCGGPKNLFGVCAEGFQCAPLVADEELQDDPAVVGRCIEIP***	TGCTTTATTAATTTCCACTTTTTAGAACAGATTATTTATTAATT<br>TCAACTTTCTAAGAATATTAACTGCGTTGCTACATAATTCAG<br>ATGATAAAATATATTTTGCTGCTGTTTCACGCACTGTTACTAT<br>AGAGGTTTAGGTATTTCGGCACACGTTCCAACCATCTCGGGA<br>TCCTCTTGAGCTTCTTTATCTGCAACTAATGGGACACAGACA<br>GATCCTCCAGCGCAAGTACCAAACATACCCTTCGGTCCACCG<br>CAGTAATCTCCAGGTCCCTGAAGAGATGAGAATGACAGTAA<br>GTTAAAAGGCGTAAAGTGTATTTAAGCTTCACGTGATACCTG<br>CTCTGCACTACATGCAGCAAATTATCTTACTCCGTATAATCTA<br>TATCCTTCTTAAATAGAGCATCGTGTTTCGAAACATGGATAG<br>CCGTATTCAGCTACCATCTGATATGCAGCATTAAATCCTGTA<br>CGTAATACACATATTCGTAGATGCAATAATATATTATTGATG<br>ATCACAGAAAGGGTTAGAAATTACATTGATTTTAACGACAGC<br>AAGAAATTACACAAACGTTATAAAAAAAAAAATTGAAATGAT<br>TTCTTGAGTGATTTGCAACGTGAGCGTTCATTCATATCATTTA<br>CAACATTCACAGTTAACAGTGTTGACTGTCCTGGGCTCTTGCT<br>TGTTCTTCATACCTTCAATTTAATGAAATTTTTTACTTATACTC<br>AAAATGGTTAACCTGTCGTTAACAGGCAACCAAGGAAAGTT<br>CATCTGAAACTCTCATTGTGCATGCTTGTCAGGGTTATATCTG<br>AGGTAAGATTTCACAATCAATAGATGTTCATTCTAGGAAAAA<br>GAATAGGATCACACAGCACAGTGCTAATGAATGCCTAAGGT<br>TTAAAGAATTAGTCTCAGTCACGCGAACTATGATCTGTTTTCC<br>TTATTTAAAGTAATGCCATTAAGATTTCATCTTCGACTAAATG<br>GGGCACAGTGTATAATGAAATTTTGTACTTATACTCAAAATT<br>GTTAACCCGTTGTTAACAGACAACCAAAGTTCATCTGAAACT<br>CTCACTGTGCATACTTGTAAAATTTGAATGGGATTTCAAGTTC<br>GAATTAGTTGCACAAGTTATTAGAACCGCGCTGTTTTTAGAA<br>AAGCATTAATATTGCAAAGATTTAAAATTGCAAAGCAATAA

							GTTGATGAAGAAATATTAGAAGGATATTTCAGACAACAACA<br>AAACATGTCTTTATGGTATGATTTATTAATATCAAACAAAAG<br>AATATTAACTCCGTTCACACAAAATATTTATATTGAAATATCT<br>TTTTCTGTTGTCTCATCCACTGTTGCTGTGGAAATTATGGTAA<br>CATCTAAGGTATTTCGATGCATCTTCCAACTACTGCCGGATC<br>ATCTTGAAGCTCCTCATCAGCAACCAACGGAGCACATTGAA<br>AACCTTCAGCACAGACACCAAACAAATTTTTTGGCCCTCCAC<br>AGTAATCATCAGGTCCCTGGAAGCAAGTCAAGCAACAATCA<br>CAAGCATCCTTCGTGACGCCGTATTCACAAGGCCTGGGATCT<br>TCACATAGTTCACTTTTGCACTCTGCACATTCAAAGTGATCAT<br>CTGAATGATGTTCGAAGGAGCTTGCAACAAGCATCATTCCAA<br>TGGCAAAGGAAAGTAGAGTCTTCATTTTATCAGTAAAATCAG<br>AGTAGGATTCGGTGCGGAAATAACAGAGATTCAATTCTGAA<br>TATCACAGTCAAAGCAACTACAGCGGTATATGGACGTCGGA<br>AATTCCTGACTGTCAGGAAGGAGGTTGAATCAGGTCGGATC<br>ACTTCCCTCTAATTTTCCAAGTATCTTCATTTGCG

PhdGrFIns05	P0CJ14	1,17E-07	44,898	PF00219	11393.02<br><br>**9610.77**	MKTLLSFAIGMMLVASS**FEHHSDDHFECAECKSELCEDPRPCEYGVTKDACDCCLTCFQGPGDYCGGPKGMFGTCAGGSVCVPLVADKEAQEDPEMVGTCAEIPKPL***	TGCTTTATTAATTTCCACTTTTTAGAACAGATTATTTATTAATTTCAACTTTCTAAGAATATTAACTGCGTTGCTACATAATTCAGATGATAAAATATATTTTGCTGCTGTTTCACGCACTGTTACTATAGAGGTTTAGGTATTTCGGCACACGTTCCAACCATCTCGGGATCCTCTTGAGCTTCTTTATCTGCAACTAATGGGACACAGACAGATCCTCCAGCGCAAGTACCAAACATACCCTTCGGTCCACCGCAGTAATCTCCAGGTCCCTGGAAGCAAGTCAAGCAACAATCACAAGCATCCTTCGTGACGCCGTATTCACAAGGCCTGGGATCTTCACATAGTTCACTTTTGCACTCTGCACATTCAAAGTGATCATCTGAATGATGTTCGAAGGAGCTTGCAACAAGCATCATTCCAATGGCAAAGGAAAGTAGAGTCTTCATTTTATCAGTAAAATCAGAGTAGGATTCGGTGCGGAAATAACAGAGATTCAATTCTGAATATCACAGTCAAAGCAACTACAGCGGTATATGGACGTCGGAAATTCCTGACTGTCAGGAAGGAGGTTGAATCAGGTCGGATCACTTCCCTCTAATTTTCCAAGTATCTTCATTTGCG
PhdGrFIns06	P0CJ14	5,56E-15	41,053	PF00219	9468.05<br><br>**7503.53**	MKTLLLFAVGTALIGSCLM**LKCVCDPDSCTDPGPCTYGTTLNACGCCQVCRKGPGEKCGGPWNAGGVCAQHLTCARVGDPATPVTGNERGICQ***	TTTTTTTTTTCGATGAAGCAAAGTTTATTATGAATATATCAGTTTTATTTTAACGACAGCTTTTTTGCTGGAAATTGACTCTAACAAGTATTTTATGACCTTTATTGACAAATTCCACGTTCATTTCCAGTCACAGGCGTAGCAGGATCTCCTACTCGGGCGCAAGTGAGATGCTGAGCACAGACACCGCCGGCATTCCACGGTCCTCCACATTTCTCGCCTGGACCCTTGCGACATACTTGACAGCATCCGCAAGCATTTAATGTTGTGCCATACGTGCAAGGACCAGGGTCAGTACAACTGTCTGGATCACAAACACATTTCAGCATCAGGCAACTCCCTATGAGTGCAGTTCCAACAGCGAACAGCAAAAGAGTCTTCATTTTTGTGCTATTAGAAGAGATAAATGTCTCGCACAACTTTGAAATAACTATTTACAATGGTTTAAATACTGATAATAGTGTAAATGAAACAGACTTTTCCTCGAACGGATTTCATACGAGAAAGAAACGGAAAGGAA

PhdGrFIns07	P0CJ14	7.08e-09	35,802	PF00219	14938.19	QQCKHENDSCTRQVNLSRRFFLIGIVAHLQIMAHLQSSILLVFLCCISSLMAIQDDREMLCSEISCDDYECPITDDCVYGEVPDVCRCCYTCAKGPGEQCGGMFDLAGICAEGYYCEPHPKYPSMPGVCDKESK*	TGTTAACAGCAATGTAAGCATGAAAACGACTCTTGTACCAGACAGGTGAATCTCAGCCGAAGGTTCTTTCTTATAGGTATCGTTGCACATTTACAGATCATGGCACATTTACAGTCTTCCATCCTGCTCGTCTTCCTTTGCTGTATATCTTCATTAATGGCGATACAAGATGACCGTGAGATGTTATGCAGTGAAATCTCGTGTGATGACTATGAGTGTCCTATCACCGACGATTGCGTGTACGGCGAAGTACCCGATGTATGTCGTTGTTGCTACACATGTGCAAAGGGCCCTGGCGAACAGTGTGGAGGTATGTTCGACCTCGCAGGCATATGCGCGGAGGGTTACTACTGCGAGCCTCATCCCAAGTATCCATCCATGCCGGGCGTGTGCGATAAGGAATCAAAATAATGCCAGGACACACATTTATGCTGGACCTTCTTTCTTAAAGGACGCGTGTGCGATAAGGAATCAAAA
PhdGrFIns08	P0CJ14	5.52e-12	46,154	PF00219	10245.27<br><br>**8194.62**	MKTLLLFIIGTTLIGSCLM**LSCTQCYKNKCPAPGPCKYGSTLDACHCCQVCRKVPGDKCGGPSNLGGVCAMHLFCARHGYPAKPVARGEYGICRGKA***	AATTTTATTTCAATAACAGCCGTTTTGCTGGAAATTGACGCCAACAAGCATTTTATGCCTTTCCCCGACAAATTCCATATTCACCACGCGCCACAGGCTTTGCAGGATATCCATGTCGGGCGCAAAAGAGATGCATAGCACAGACACCGCCCAAATTCGATGGTCCTCCACATTTGTCCCCTGGAACCTTGCGACATACTTGACAGCAATGGCAAGCATCTAATGTTGAACCATACTTGCAAGGACCAGGAGCAGGGCATTTATTTTTATAACACTGGGTACAGCTCAACATGAGGCAACTCCCGATGAGAGTAGTTCCAATAATGAACAGCAGAAGAGTCTTCATTTTTGCGCTATAAGAGAGATAAAT
PhdGrFIns09	P0CJ14	1.57e-06	37,097	PF00219	6565.60<br><br>**4532.96**	MKAVLLIFVGIALIDSCLM**TCPPCEAQRCSAPGDCEYGTTLDGCGCCPECFKGPGENCGGNDSV**	TCGAAATATTTTTATTCTGCTGATAACTATCGAAAAAAAATGAAGGCTGTGCTCTTGATTTTTGTTGGAATCGCACTCATAGACAGCTGCTTGATGACTTGTCCTCCGTGTGAAGCCCAAAGGTGTTCTGCTCCTGGTGATTGTGAATATGGTACAACATTAGACGGTTGTGGCTGTTGTCCAGAATGCTTCAAAGGTCCTGGTGAAAATTGCGGTGGGAATGACAGTGTGGA

PhdGrFIns10	P0CJ14	4.99e-10	44,828	PF00219	12151.17<br><br>**10204.67**	MKTLLLISVGIALTG SCLM**LKCIKCDPEK CIDPIDCKFGKTLD VCFCCKACRKGPG EECGGAWNMRGIC AKHLICARKSEDNK HENYEGGHATTWE PISEHEDGICRERS***	TCCATCAAAAATATATTTGCTTCAAATTATTGTCTATACAAGAAAACTATGATCTTTCACGGCAGATTCCATCTTCATGCTCGCTGATCGGTTCCCAAGTTGTCGCATGACCACCTTCGTAATTTTCATGTTTGTTGTCTTCTGATTTTCGAGCACATATGAGATGTTTAGCGCAGATACCTCGCATATTCCATGCTCCACCACATTCCTCACCAGGACCCTTGCGACATGCTTTACAGCAGAAACAGACGTCTAAGGTTTTACCAAATTTGCAATCAATAGGATCGATGCACTTTTCGGGATCACACTTGATACACTTCAACATCAGGCAGCTCCCTGTGAGCGCAATTCCAACAGAAATCAATAGGAGAGTCTTCATATCTTTCGATAGCTATCAGGAGAATAAAAATACCTCACCCTACGTTGAAATGGATATCTGCTTCACCAATAGCTATCAGGAGAATAAAAATATCTCCTGGCACGGATATCAG
Platelet derived growth factor							
**Transcript ID**	**Uniprot ID reference protein**	**e-value**	**Identity (%)**	**PFAM domain**	**MW (Da)**	**Amino acid sequence**	**Nucleotide sequence**

PhdGrFPD G01	C0K3N5	4,68E-11	23,832	PF00341<br><br>PF14554	32761.41	MFRLDYMYCFKIILIS FISVTRSQDRIIVSNL NLDEIHSNSSLENDT IPEKLLIQLKEVRNVS DLLQRFVAFNDDDA SLLGPSRRNGTDSAR RGLSAVAASSIAQPA NCQTEQHVIELDKP ENGATLFWPSCIRVR RCGGCCTSKMLACK PLVISILNVTVLQMY YNHQNPGSFESQGT KLFSLEQHDRCACT CKESPEDCNLNVHD YRAEECRCACKNSE QSTSCTGPRKIWDSR ECLCKCRNIRLCSSG RYFNILTCRCEILRRS LAIDNLNTQHLNTS LALQVSAASP*	ATTATATGTTTACAGTGCCTTATTGACTTTAAGAAAATAATTA AATTTTTCTCATTTGTTCAAATTTTTCGTTTCCTTCGTTCCACG GTACAATGAACTTATCTTCATTATTCAAGGAGAGGCTGCTGA TACTTGTAATGCCAAAGAAGTATTAAGATGCTGTGTGTTCAA ATTATCAATAGCTAAAGATCGCCTTAATATCTCACACCTACA TGTTAGTATATTGAAGTAGCGTCCAGAGGAACACAAGCGAA TATTCCTACATTTGCATAAACATTCTCTACTATCCCATATTTTC CGCGGTCCAGTGCAGGATGTTGACTGTTCTGAGTTTTTACAA GCACACCTACATTCCTCAGCTCTGTAATCGTGTACATTTAGAT TGCAGTCCTCGGGACTTTCCTTGCAAGTACAGGCACAACGGT CATGCTGTTCTAAAGAAAATAGTTTTGTTCCTTGGCTTTCGAA ACTACCCGGGTTTTGGTGATTATAGTACATTTGAAGAACCGT CACATTCAAAATTGAAATGACAAGAGGCTTGCACGCAAGCA TTTTGCTAGTACAACATCCACCGCAACGTCGAACACGGATAC ACGAGGGCCAGAAAAGAGTGGCTCCATTTTCAGGTTTGTCCA ATTCTATCACATGCTGCTCTGTTTGGCAATTAGCTGGTTGAGC AATACTGGAAGCAGCAACAGCAGACAATCCTCTTCTAGCAC TGTCAGTTCCATTTCTTCTAGAAGGTCCAAGCAGGGATGCAT CATCATCATTAAAGGCAACAAAACGTTGGAGAAGGTCGGAT ACATTTCGTACCTCTTTCAGTTGAATTAGCAACTTTTCAGGTA TTGTGTCGTTTTCCAGTGACGAATTACTATGAATTTCATCTAA ATTCAAATTTGATACTATTATCCGATCTTGACTTCGTGTCACA GAAATAAAAGAAATCAAAATTATTTTGAAACAATACATATA ATCCAACCTAAACATTTCAAGTTCCTTGCTATAACGGAGCCA GCGGTTTAGAGGTTATTACTATTCGAAGGATGTCGAGCATGT GAGGAAATATATTCTTAGAGAATTCCATCAACGCTTACTTCC TTCTTTA

PhdGrFPD G02	C0K3N5	1,05E-07	32,558	PF00341<br><br>PF14554	44230.05<br><br>**41424.60**	MEWCRMYTLRVTM<br>CVVLLCSGLQS**VVP**<br>**DTMPDEQLMSSRD**<br>**QKLQELLRSLDAYS**<br>**TETEPKFSSRTTATT**<br>**TTPSSSPLHSPSLESF**<br>**LHHHDTRARIGTEV**<br>**SRDNRLPDPSEIRS**<br>**NQDFNKVFFGSQE**<br>**GVNRFKKQFGPTG**<br>**LQKARAMYPDVNK**<br>**DIMNMKMANNHI**<br>**LRIRSSAKCQKPVP**<br>**QVVRVKDYYPDPS**<br>**REYYPRCTILHRCG**<br>**DDTGCCEHDAFRC**<br>**VPSAVQEVTLYFYS**<br>**LTVGKVGFVDNRV**<br>**EKLLFENHTACQCQ**<br>**QINDLPRMSDGRES**<br>**PDEEEPTPKPKCGE**<br>**CPVPFTSRIYKDGR**<br>**CGCDCFDRQKPCIR**<br>**IKRGREPLPEIERRC**<br>**VEANHCHIPDCEYG**<br>**LYDPKNGYCPRRH**<br>**DEDQRIPHRKQYH**<br>**GSASQRWAFIERD***	TGGTGATCTCTTGAAGGATCCGCACTACTCAGTCTCTGTGTGC<br>TCTCCCCATCCACTCAGAACCGGTAGTCCGATGCTCCACCGG<br>ACGTACCTGCCGGACTCTCAGAAACCCACTCCGACTTTATGG<br>CCGAACACTCATTGCTAACGACACTTGCTGTATGGAATGGTG<br>CCGGATGTATACTCTGAGAGTGACAATGTGTGTAGTGCTCCT<br>GTGCAGTGGACTCCAAAGTGTGGTGCCGGACACGATGCCGG<br>ACGAACAGTTAATGTCCAGCAGGGACCAGAAATTACAGGAG<br>TTACTAAGGTCCTTGGATGCTTACAGTACAGAAACGGAGCCC<br>AAATTTTCATCCAGAACGACAGCAACAACAACAACACCTTC<br>TTCCAGTCCACTACACTCTCCAAGCCTAGAATCCTTTTTGCAT<br>CATCATGATACCAGAGCCAGAATAGGGACTGAAGTGTCCCG<br>TGACAATCGCCTTCCAGATCCATCAGAGATCAGATCAAATCA<br>AGACTTCAACAAAGTATTTTTCGGATCACAAGAAGGTGTCAA<br>TCGTTTCAAAAAGCAGTTCGGTCCAACTGGATTACAGAAAGC<br>CAGGGCTATGTACCCAGATGTCAACAAAGATATCATGAACA<br>TGAAGATGGCCAACAACCACATTCTCCGAATCCGCAGTAGT<br>GCAAAGTGCCAAAAACCAGTGCCGCAAGTCGTACGCGTAAA<br>AGACTATTATCCCGATCCATCGAGGGAGTACTATCCTCGTTG<br>CACAATCCTTCACAGATGCGGCGACGACACAGGATGTTGCG<br>AGCATGATGCATTTCGTTGCGTTCCGAGCGCGGTCCAAGAAG<br>TCACATTGTATTTTTACTCCCTAACTGTAGGCAAAGTCGGCTT<br>CGTGGACAATCGCGTTGAGAAGCTCTTATTTGAAAACCATAC<br>TGCTTGCCAATGCCAGCAGATAAACGATCTTCCCAGGATGAG<br>CGACGGACGTGAATCACCTGACGAAGAGGAGCCAACACCCA<br>AACCAAAATGCGGAGAATGTCCTGTACCATTTACCAGTCGTA<br>TTTATAAAGATGGCCGATGCGGCTGCGATTGTTTCGACAGGC<br>AGAAACCTTGCATCAGGATAAAGCGAGGAAGGGAGCCCCTG<br>CCTGAGATTGAGAGAAGATGTGTGGAAGCTAACCATTGCCA

							CATTCCGGATTGCGAGTATGGTCTGTATGATCCTAAAAATGG CTATTGCCCTAGAAGACACGATGAAGATCAAAGAATACCAC ATCGCAAGCAATATCATGGGTCTGCAAGTCAACGGTGGGCA TTTATAGAAAGAGACTGACTTCACTAAAGATGTCCCATAAAT AGAACAATCCTTGGACTTTTAATATGGACCACTGTACGAATG AACAGTGTAAAAATGCAATCTTGACGAATTTTCTCAGAAGTA TACTCGTACTACAAAACTCAAAATTTACTTTTAATCAAAATT ATTTAAAATTTTTTTTTATAAAATTGGTATATTTTAATTGCATA CAGCATCCTTTAAATGAAGCACAAAATAATTACGGAATAAA AAAAAATGGGCTAGAAAATAAAAAAATTGTATTGTTCCTCTT GAAAATGTTCTTAGAGATTAAAACACAGATGCGCGTTGAATC GAATTTACATGAAATTATTTTTCTACTGAGAATATTTATTAAT GTAATAGCTCTTTATAATTAACTCACATGCAACCTCCCTTAAT GGACAAATGTCTTGGTTTCTTGAGTATACGCTATCGGGAGGT CCAGCTGCAATTAATTTATGTATCTTGCATACTGTTGGACTTG ATTAAGGAGCTAAATATAAATAAAGTGTGTTTGATGAAGTTT ATTTTTTGACTAATTAGACAAGACGATATAAAATTTTCATAG AAATGAAATCAATGGTTCATGCTTAAATGCGTTTCCTGGAGA ATCAAATTTTGACAAAATTGTACCAAATTTTTCTGTTACGCTC GATTGAACTTTGACAGATAATCTTCCATACCATTGGACTTAA GATAATTGTTTGCCTCTCGATGCTCTTATTTACTCCTTCAATAA TCAGCGAAAACTGCGCGTTAATATTGTGGGGCAGTTAAAATT AATATTTTTATACTGTTGAACACAAAATGTTTCTTAGATAATA GTCTATTCTGAAGACAATGGTCGTATTTACATGAATACTGAA AAAGGATCAAATTTAATTATTTTTATATATTG

PhdGrFPD G03	C0K3N5	1,06E-07	32,558	PF00341<br>PF14554	44230.05<br>**41424.60**	MEWCRMYTLRVTM<br>CVVLLCSGLQS**VVP**<br>**DTMPDEQLMSSRD**<br>**QKLQELLRSLDAYS**<br>**TETEPKFSSRTTATT**<br>**TTPSSSPLHSPSLESF**<br>**LHHHDTRARIGTEV**<br>**SRDNRLPDPSEIRS**<br>**NQDFNKVFFGSQE**<br>**GVNRFKKQFGPTG**<br>**LQKARAMYPDVNK**<br>**DIMNMKMANNHI**<br>**LRIRSSAKCQKPVP**<br>**QVVRVKDYYPDPS**<br>**REYYPRCTILHRCG**<br>**DDTGCCEHDAFRC**<br>**VPSAVQEVTLYFYS**<br>**LTVGKVGFVDNRV**<br>**EKLLFENHTACQCQ**<br>**QINDLPRMSDGRES**<br>**PDEEEPTPKPKCGE**<br>**CPVPFTSRIYKDGR**<br>**CGCDCFDRQKPCIR**<br>**IKRGREPLPEIERRC**<br>**VEANHCHIPDCEYG**<br>**LYDPKNGYCPRRH**<br>**DEDQRIPHRKQYH**<br>**GSASQRWAFIERD***	TGGTGATCTCTTGAAGGATCCGCACTACTCAGTCTCTGTGTGC<br>TCTCCCCATCCACTCAGAACCGGTAGTCCGATGCTCCACCGG<br>ACGTACCTGCCGGACTCTCAGAAACCCACTCCGACTTTATGG<br>CCGAACACTCATTGCTAACGACACTTGCTGTATGGAATGGTG<br>CCGGATGTATACTCTGAGAGTGACAATGTGTGTAGTGCTCCT<br>GTGCAGTGGACTCCAAAGTGTGGTGCCGGACACGATGCCGG<br>ACGAACAGTTAATGTCCAGCAGGGACCAGAAATTACAGGAG<br>TTACTAAGGTCCTTGGATGCTTACAGTACAGAAACGGAGCCC<br>AAATTTTCATCCAGAACGACAGCAACAACAACAACACCTTC<br>TTCCAGTCCACTACACTCTCCAAGCCTAGAATCCTTTTTGCAT<br>CATCATGATACCAGAGCCAGAATAGGGACTGAAGTGTCCCG<br>TGACAATCGCCTTCCAGATCCATCAGAGATCAGATCAAATCA<br>AGACTTCAACAAAGTATTTTTCGGATCACAAGAAGGTGTCAA<br>TCGTTTCAAAAAGCAGTTCGGTCCAACTGGATTACAGAAAGC<br>CAGGGCTATGTACCCAGATGTCAACAAAGATATCATGAACA<br>TGAAGATGGCCAACAACCACATTCTCCGAATCCGCAGTAGT<br>GCAAAGTGCCAAAAACCAGTGCCGCAAGTCGTACGCGTAAA<br>AGACTATTATCCCGATCCATCGAGGGAGTACTATCCTCGTTG<br>CACAATCCTTCACAGATGCGGCGACGACACAGGATGTTGCG<br>AGCATGATGCATTTCGTTGCGTTCCGAGCGCGGTCCAAGAAG<br>TCACATTGTATTTTTACTCCCTAACTGTAGGCAAAGTCGGCTT<br>CGTGGACAATCGCGTTGAGAAGCTCTTATTTGAAAACCATAC<br>TGCTTGCCAATGCCAGCAGATAAACGATCTTCCCAGGATGAG<br>CGACGGACGTGAATCACCTGACGAAGAGGAGCCAACACCCA<br>AACCAAAATGCGGAGAATGTCCTGTACCATTTACCAGTCGTA<br>TTTATAAAGATGGCCGATGCGGCTGCGATTGTTTCGACAGGC<br>AGAAACCTTGCATCAGGATAAAGCGAGGAAGGGAGCCCCTG<br>CCTGAGATTGAGAGAAGATGTGTGGAAGCTAACCATTGCCA

							CATTCCGGATTGCGAGTATGGTCTGTATGATCCTAAAAATGG<br>CTATTGCCCTAGAAGACACGATGAAGATCAAAGAATACCAC<br>ATCGCAAGCAATATCATGGGTCTGCAAGTCAACGGTGGGCA<br>TTTATAGAAAGAGACTGACTTCACTAAAGATGTCCCATAAAT<br>AGAACAATCCTTGGACTTTTAATATGGACCACTGTACGAATG<br>AACAGTGTAAAAATGCAATCTTGACGAATTTTCTCAGAAGTA<br>TACTCGTACTACAAAACTCAAAATTTACTTTTAATCAAAATT<br>ATTTAAAATTTTTTTTTATAAAATTGGTATATTTTAATTGCATA<br>CAGCATCCTTTAAATGAAGCACAAAATAATTACGGAATAAA<br>AAAAAATGGGCTAGAAAATAAAAAAATTGTATTGTTCCTCTT<br>GAAAATGTTCTTAGAGATTAAAACACAGATGCGCGTTGAATC<br>GAATTTACATGAAATTATTTTTCTACTGAGAATATTTATTAAT<br>GTAATAGCTCTTTATAATTAACTCACATGCAACCTCCCTTAAT<br>GGACAAATGTCTTGGTTTCTTGAGTATACGCTATCGGGAGGT<br>CCAGCTGCAATTAATTTATGTATCTTGCATACTGTTGGACTTG<br>ATTAAGGAGCTAAATATAAATAAAGTGTGTTTGATGAAGTTT<br>ATTTTTTGACTAATTAGACAAGACGATATAAAATTTTCATAG<br>AAATGAAATCAATGGTTCATGCTTAAATGCGTTTCCTGGAGA<br>ATCAAATTTTGACAAAATTGTACCATGAATTGTACCGTCTCA<br>TCCAAATTTTTCTGTTACGCTCGATTGAACTTTGACAGATAAT<br>CTTCCATACCATTGGACTTAAGATAATTGTTTGCCTCTCGATG<br>CTCTTATTTACTCCTTCAATAATCAGCGAAAACTGCGCGTTAA<br>TATTGTGGGGCAGTTAAAATTAATATTTTTATACTGTTGAACA<br>CAAAATGTTTCTTAGATAATAGTCTATTCTGAAGACAATGGT<br>CGTATTTACATGAATACTGAAAAAGGATCAAATTTAATTATT<br>TTTATATATTG

PhdGrFPD G04	C0K3N3	3,30E-06	38,806	PF00341	8471.92	KYYLPHCTLLHRCG KHAGCCAVESLKCV AAEKEKVTLYFYSVK FDGGDLTERKVEKL VFTNHTRCACAPVD HIPK*	AAATATTACTTGCCACACTGCACTCTTCTTCATAGATGTGGG AAACATGCCGGGTGCTGTGCCGTTGAAAGCCTCAAATGTGTC GCAGCTGAAAAGGAAAAGGTGACACTGTACTTCTATAGTGT AAAATTTGACGGTGGTGATCTTACGGAAAGGAAAGTAGAAA AGCTGGTTTTTACCAATCACACTCGATGCGCCTGCGCGCCAG TGGATCACATTCCCAAGTGAATAAAATCGAGCTTGATGAGG AATAAAGGGTGAGAACAGAAGAATTAAGGATCTGGGTTATA AATATGAGTGAGATTTCATGAAAATGTTATTAGGCATGGGTT TTTTATATCAGGACCTGATATGTTTGTTTTATTATAACTGTAAT AATATACCTTACCAAAATAAGTATTAGTTACTACTTATGTGCT TTATACTGAAAAATTTCACGACCCATCCACAGATACAGATTT AGCAAAATGAGAGAATGGTATGATTTTTTCATACTACTGAAT TCCGACGCTTCAACCAAAATATATCATCCGTTATTCCGATGT GCCGTAACCTCATGCAGTGTGCAGTAACATCATTTATTTGAG CTTATAAGTAAAGTTGGTATTGTAATACTGATTTACTATTTTT AAATTTCATCCAAACAAATGCTTATGGATCTCTCAGAGTGAT TGCAGTTTCCAAGTTCAGCAAGTTCATAATATTTTCTCATCGA GTAATGAACTTTCATGACGCGAGAAAACACGAGAGAGTTCC TCCAGTTTTAAATTATTTACATGTTATTTAAATTTCTAGTTTTA ATCGTATGTGTTTCATGAACACCAACTATCTTAAAGCTGTATT GTGAGAATTGTATCTTTTGTTATTGTACATTTATTGTATTTGTA GTTATTCCATTTCACGATGTAACGTTGTCAAGAAATTTTCGAA ACATTTAAATTACATTTAATAGTGTTCCCCAAAATATGATTCT TGAGGTGCTAAGTTGTACTTTGCAAAAGTTTTAGGTGGTATTT TTCAGCTGTGGGACATGAATAATTTAAATGGTCAATCTTTTTG TATAAAGAAGTTTGCAATTGTCACATACTAACATATAAATTC ATTCAAATGAAGTTAAATGAAGCATTTAGAATTATTTTAAGT TTTGTAAGAAGATAGTTGGATTGGTTTCCTTAAAAAAATAGAC

							GTCTTTTAGTTACTACAATTTTGTAGGGTGCATTTACTCTGGAGCAGATGATAGTTGAAGCAGTGAGCATCTTTTTATAGCTGCAAATAAAGATTTCCGGAAATTGAATTGTGCTAAACGAGGAAATTTCCAAAGACGAGAAATCATAACAAATATTGTGACATTAAGATATTAGAATTTAGATTAATTTTATTTTAAGATTTAAAATCCCAATATTTCTCATATGATTCAGAATAGGTAATTAAAAGTAATAGGAAG

PhdGrFPD G05	P83942	5,31E-08	35,000	PF00341	25002.33	RWPRAQVVCVKDL YPEKEFLPRCTLLHR CTETAGCCEDEGLQ CGPKAMQEVALHF YVLGGDHLSSDVEK LLFVNHTECECKPKP SPKPAPAPSTTARSST TEHYAQRDQQNPK CRSCPTPFFVREHED GRCSCDCFDRHKPCI RIKRGRDAIADKERR CVQAGECNLPECEY GPYDPDRGRCPKRP EHLGPREGDVMRRP VPNHKNPNHRWLY FERD	GCCGTTGGCCACGAGCTCAGGTGGTGTGCGTTAAGGACCTAT ATCCGGAGAAGGAGTTTTTGCCGAGGTGTACCCTCCTGCATC GGTGCACCGAGACCGCGGGATGCTGCGAGGACGAAGGACTT CAATGCGGACCCAAGGCCATGCAAGAGGTGGCTCTGCATTT CTACGTTTTGGGCGGCGACCACCTGTCCTCGGACGTCGAGAA GCTGCTGTTCGTGAACCACACGGAGTGCGAGTGCAAACCGA AACCGTCGCCGAAGCCCGCCCCTGCCCCGTCCACGACCGCTC GGAGTTCGACGACAGAACACTACGCACAGAGGGACCAGCA GAATCCGAAGTGTCGTTCTTGCCCCACGCCGTTCTTCGTACGC GAACATGAAGATGGAAGATGCAGCTGCGACTGTTTCGATCG ACATAAGCCATGCATTCGCATCAAGAGAGGAAGGGATGCAA TCGCCGATAAGGAAAGAAGGTGCGTCCAAGCGGGTGAGTGC AATCTGCCGGAATGCGAGTACGGCCCGTATGATCCAGACAG AGGTCGGTGTCCCAAAAGGCCCGAACATCTCGGCCCACGTG AGGGAGATGTGATGAGAAGACCCGTGCCCAATCACAAGAAT CCAAACCATCGATGGTTATATTTTGAAAGAGATTGATGCCCA AGGTTATATATCTACGAGCGTCCTTTTGAAGTTTTGCCACTTA GGGAGAATTTTTGTAATGATCGAACCGTGTCTGGCTCGAAGA AGTGTGTAGTTTTTCCGAAGAAATCGCATTTCGCATCCTCTGT TCTTCGAAAACGATCCGTGTAAATAGACGATAGTCTACCAGA AAACTTTTATTTTGAAATGTGCCACTCGGACAATATTTTCCAA GTTTTGAACTCAAGTGTTTTCATCGCATTTTTTTCTGCGGCAG AGTTGAAAAACCCTGTAATTAGGAGACTGTCCAATTGCAAAT GTCATCTCATCGACCTGATTTAAACAAGAACGTTTAGGGAGA GTCCAAGGTGATACTGCTTTCGTATTTTGTAGATAGTGTCGAG ACTTAAAATATATCGCGTAGGTTTTAAGTCGATAAGCGTTTC AGTGCGCGTGGATTTGTGGATTTTGTTCCTTTGCAAATTGCCT TTTTTTATACATATTCTTAAAAGGGAACAAAAGTTAACGAAA

							CCTTCTCTTACATTCTTATACGTCTTCGCTACGTATGTTATTTC<br>TTGCCTCCAAACTACTTTACTTATGATCTCATATTTTTCAGAT<br>ATGCGTGAAATAAAACACATTATCTCGGTGGAAAAGTGTTTC<br>AATACGCAAGAATATGGAAATTAGAAATTTTAAGTCCTTTTC<br>TTTACTCTTTAATTTCCTTTTTCTTATATCTGTAGTTTCTCTTGG<br>CGAGGAACTATCATGGCGGAAGCAGCACTGATCTAACTTCC<br>GCCAGACATAGAGCTTTCTTTGAAATATAAGTACATAACTAT<br>GCCATATTTGTTTAGTTTTTTTTTTTCCCTCCAAGAACAAGAC<br>GCAGTACATGATGAGTGACAATTTTCTTTCTGTGTATCGTCTA<br>AGTGCATCAATTTTGTTTTCTGACGTTATCAGTGCCTGTTGCA<br>AAATGTAGTATTTTTAAGGAAACTTCAAATTTTCTCATAAATC<br>GTTGTTTCAGTTGCTCTGCCTTGCCACTGCACATTTAAGTTAC<br>TTTCAATCATTACGTGTTTAAGGTTTAATCACTCTCACGACAA<br>AGATTTTGAAGCAATAATTACATATGTTGAACGGAAGAATGC<br>TAGGGAATTTTCAATTCATCTCGGACTGTCTTTCTGAAGCAGA<br>AAAACAGGACACACGATTATCTATCTATCTATCTCACCAGAC<br>AGTCCGAGATTAAGTTAGTCGCGTACTTGTGGATTTTAAGAA<br>GCTTTTTATCAATTACTACCAGCATATGGAAATGTCGAAATT<br>ATGTAATATTGTATGTGTAATATTCTCGCTGTCTAAATTTTCAT<br>TACTTAAAATGCA
Nerve related growth factor							

Transcript ID	Uniprot ID reference protein	e-value	Identity (%)	PFAM domain	MW (Da)	Amino acid sequence	Nucleotide sequence
PhdGrFNeR 01	Q3HXY3	1,61E-05	28,030	PF00243	18565,32	KLISMASITRGRFLSQ<br>TAAFGFSLVVVCVST<br>VSSNSLDKEFSATTH<br>IQKKSLEDVELIEVCS<br>SVQSFEQLETAVNV<br>DLQTVEIHPEAWVF<br>ATRCRVPGKACRGF<br>SDDIQSICRPKKSWV<br>QMYGRTSGDTWRP<br>HWVAVDTACVCSIR<br>RKRSSNLSTGRMPLT<br>GKTFLP*	GTTTAAGTACGTTTTATTTCACAGCGCAACACACTATACGCA<br>GGAAGTCCCACGGCCGTCCCGTTACATACCCAAACAAACAC<br>ACAATCTGCGTGCCACAAGTAATCTACATCGAAACACATGAT<br>TGTCTAGTGTGAATGATTATAAACATGTGCAAAAAAAAAAA<br>AACAAAACGGACGAGAAACGAAAGGATCCTGCAAGGCAGT<br>CCGTGGACAAGAGACGAACGACTCCGATGGACGCAGACATG<br>TCACTCGACACTTCTGGATATGGGAGGGAGTGGATAGAAGT<br>GTGTCTTCAGGGTAGGAACGTCTTCCCCGTCAGCGGCATCCT<br>GCCGGTGGACAGATTACTGGACCTCTTTCTGCGGATGGAACA<br>AACGCAGGCTGTGTCTACGGCGACCCAGTGGGGCCTCCAGG<br>TGTCCCCGGAGGTGCGGCCGTACATTTGTACCCAGGATTTCTT<br>CGGTCGACATATGGATTGAATGTCGTCTGAGAATCCCCGGCA<br>GGCTTTTCCCGGCACCCTACATCGGGTGGCGAATACCCAGGC<br>TTCCGGATGTATTTCTACGGTCTGCAGATCGACGTTAACCGCC<br>GTTTCGAGCTGTTCGAAACTCTGCACAGAACTGCACACCTCT<br>ATCAGTTCGACGTCTTCCAGTGACTTCTTTTGTATATGTGTGG<br>TTGCACTGAATTCTTTATCTAGAGAATTTGAACTCACTGTAGA<br>AACACAGACGACGACTAAAGAGAATCCGAAAGCGGCTGTCT<br>GACTGAGGAAACGACCTCGTGTGATACTGGCCATCGAGATA<br>AGCTTTCAATCTGTTGACAAGTGTCCTCCTGCCGTTACTCAGT<br>CGTGATAGAAGTGGTGGCCACTTTTAAGGGTGGTGGGGGTTT<br>TATGAAGGGCGCTAAGTGCGTGCGCCTATGGGTGCGCCAGA

PhdGrFNeR 02	Q3HXY3	8,03E-05	27,273	PF00243	18437,14	KLISMASITRGRFLSQ<br>TAAFGFSLVVVCVST<br>VSSNSLDKEFSATTH<br>IQKSLEDVELIEVCSS<br>VQSFEQLETAVNVD<br>LQTVEIHPEAWVFA<br>TRCRVPGKACRGFS<br>DDIQSICRPKKSWVQ<br>MYGRTSGDTWRPH<br>WVAVDTACVCSIRR<br>KRSSNLSTGRMPLTG<br>KTFLP*	GTTTAAGTACGTTTTATTTCACAGCGCAACACACTATACGCA<br>GGAAGTCCCACGGCCGTCCCGTTACATACCCAAACAAACAC<br>ACAATCTGCGTGCCACAAGTAATCTACATCGAAACACATGAT<br>TGTCTAGTGTGAATGATTATAAACATGTGCAAAAAAAAAAA<br>AACAAAACGGACGAGAAACGAAAGGATCCTGCAAGGCAGT<br>CCGTGGACAAGAGACGAACGACTCCGATGGACGCAGACATG<br>TCACTCGACACTTCTGGATATGGGAGGGAGTGGATAGAAGT<br>GTGTCTTCAGGGTAGGAACGTCTTCCCCGTCAGCGGCATCCT<br>GCCGGTGGACAGATTACTGGACCTCTTTCTGCGGATGGAACA<br>AACGCAGGCTGTGTCTACGGCGACCCAGTGGGGCCTCCAGG<br>TGTCCCCGGAGGTGCGGCCGTACATTTGTACCCAGGATTTCTT<br>CGGTCGACATATGGATTGAATGTCGTCTGAGAATCCCCGGCA<br>GGCTTTTCCCGGCACCCTACATCGGGTGGCGAATACCCAGGC<br>TTCCGGATGTATTTCTACGGTCTGCAGATCGACGTTAACCGCC<br>GTTTCGAGCTGTTCGAAACTCTGCACAGAACTGCACACCTCT<br>ATCAGTTCGACGTCTTCCAGTGACTTTTGTATATGTGTGGTTG<br>CACTGAATTCTTTATCTAGAGAATTTGAACTCACTGTAGAAA<br>CACAGACGACGACTAAAGAGAATCCGAAAGCGGCTGTCTGA<br>CTGAGGAAACGACCTCGTGTGATACTGGCCATCGAGATAAG<br>CTTTCAATCTGTTGACAAGTGTCCTCCTGCCGTTACTCAGTCG<br>TGATAGAAGTGGTGGCCACTTTTAAGGGTGGTGGGGGTTTTA<br>TGAAGGGCGCTAAGTGCGTGCGCCTATGGGTGCGCCAGA
CRISP							
Cysteine rich secretory protein							
**Transcript ID**	**Uniprot ID reference protein**	**e-value**	**Identity (%)**	**PFAM domain**	**MW (Da)**	**Amino acid sequence**	**Nucleotide sequence**

PhdCRI01	Q7YT83	1,69E-24	30,698	PF00188	51004,48	MTPVSVMQVLTYVI<br>AGFYSIEWQGTCSDI<br>PKQNDSHDDSFIFVT<br>SENNEEASYLETTEIP<br>VHERLNRGSQESFA<br>ADFLKAEPIISDPLFE<br>DVPWNKSSVKQLSM<br>KRDVSQGAWTHMN<br>KMQILQLHNVYRG<br>NVTPPAGNMAYME<br>WDEELEKLAQIWAD<br>RCVFEHGMPKNDYP<br>ETIGQNLYIGTDPTG<br>YLGLYMWYEEYHN<br>YELRNEHCLPNKQC<br>GHYVQMAWSESTK<br>LGCAMTKCSMRYYV<br>VCHYSPQALKGVQ<br>MYLVAKPCSQCSAK<br>NGVQCLKNLCMTR<br>EQCKNNPFDCQDA<br>ECNLECHHCGRLDK<br>EKCTCECMDGWDSP<br>DCSHLCKNKHKRC<br>GMSPGWPTKASCSL<br>NGYAVAKTYCRKM<br>CEMCHPLLDTSNVT<br>CCEGKLCDEGFVLD	ACGAGGCAAGCAGTCAAACCTCAAATTAAATATCTTGAGAC<br>TTTTTAAAGAGAAGGTTATCATTTTCTGCAAAGGAATATGAC<br>ACCGGTCTCAGTTATGCAAGTTTTGACGTACGTCATCGCGGG<br>CTTTTATTCTATAGAATGGCAAGGAACTTGCTCCGATATTCCC<br>AAACAGAACGATAGTCACGACGATTCATTCATTTTTGTTACTT<br>CAGAAAATAACGAGGAAGCGTCTTATTTAGAGACAACTGAG<br>ATACCTGTTCACGAAAGACTGAATCGTGGTTCACAAGAGTCC<br>TTTGCGGCTGATTTCTTGAAAGCAGAACCTATAATTTCGGATC<br>CCCTCTTCGAGGACGTGCCATGGAATAAATCCAGCGTCAAGC<br>AGTTGAGCATGAAAAGGGATGTATCACAGGGAGCATGGACT<br>CATATGAACAAGATGCAAATTCTCCAGTTGCATAATGTATAC<br>AGAGGTAATGTGACACCACCAGCGGGTAATATGGCATACAT<br>GGAATGGGACGAAGAACTTGAGAAACTTGCCCAGATATGGG<br>CAGATCGCTGTGTATTTGAACATGGTATGCCTAAAAATGATT<br>ACCCAGAAACTATAGGACAAAATCTGTATATAGGAACAGAT<br>CCCACGGGATATCTTGGACTATATATGTGGTATGAAGAATAT<br>CATAACTACGAACTGAGAAATGAACATTGTCTACCCAACAA<br>GCAATGTGGACATTATGTGCAAATGGCATGGTCCGAAAGCA<br>CCAAACTGGGATGTGCGATGACTAAATGCAGTATGCGCTACT<br>ACGTCGTTTGTCACTATAGTCCACAAGCTTTGAAAGGAGTGC<br>AAATGTATCTTGTAGCTAAACCGTGCTCGCAGTGTTCGGCTA<br>AAAATGGCGTGCAGTGCTTAAAAAACTTATGCATGACTCGA<br>GAACAATGCAAAAATAATCCTTTCGACTGTCAAGACGCTGA<br>ATGCAACTTAGAGTGTCATCACTGCGGTCGTTTGGATAAAGA<br>AAAATGCACGTGCGAATGTATGGACGGTTGGGATTCTCCGGA<br>TTGCTCACATTTATGCAAGAATAAGCACAAGAGGTGTGGCAT<br>GAGTCCTGGCTGGCCTACGAAAGCTTCCTGTTCCTTGAATGG<br>CTATGCAGTCGCTAAAACATATTGTCGTAAAATGTGTGAGAT

						PHSKSCQCILLCPGPLCDGKEAAVTQAALEGSAVCIRIIPHFVLCMIILRLFESVLWKYL*	GTGCCATCCTTTGCTGGACACATCGAATGTTACTTGCTGTGAGGGGAAGCTATGTGACGAAGGATTTGTTCTTGATCCGCACAGCAAATCATGCCAGTGCATTCTTTTATGCCCAGGTCCCCTTTGTGATGGCAAAGAAGCAGCCGTGACCCAAGCTGCCCTAGAAGGTAGTGCTGTTTGCATAAGAATCATCCCGCATTTTGTTCTCTGCATGATAATTCTCAGACTGTTTGAGAGTGTCCTATGGAAATACCTTTAAAACAATTTTTCATCTACAGTGGATGCGTGTGAATATTCGCATTATCAGAGATACATTAAAAAACTAACGCAAATTACTGAAAGTTAAATTTTCGGCAAATGTGTCAAAAACTCTGGTCTTGGTTGTATATAAATGATAACAGTGTGAGATCTTATTTTTCAATTACTTGCAATTTGTATTTCACAAGCGCCTGATCTCTAATACTGCATAAATATATATTAATGTTTTCTGAAGAATGAGGCACAGTTTTTAATGTTGAATGTTGTGTATTTAAGCAAAAAAAAATTTTATATAATTGTTTTTTAATAAAATTCTTGTCTCTTTCAAAAA

PhdCRI02	Q7YT83	1,69E-24	30,698	PF00188	50990,46	MTPVSVMQVLTYVI<br>AGFYSIEWQGTCSDI<br>PKQNDSHDDSFIFVT<br>SENNEEASYLETTEIP<br>VHERLNRGSQESFA<br>ADFLKAEPIISDPLFE<br>DVPWNKSSVKQLSM<br>KRDVSQGAWTHMN<br>KMQILQLHNVYRG<br>NVTPPAGNMAYME<br>WDEELEKLAQIWAD<br>RCVFEHGMPKNDYP<br>ETIGQNLYIGTDPTG<br>YLGLYMWYEEYHN<br>YELRNEHCLPNKQC<br>GHYVQMAWSESTK<br>LGCAMTKCSMRYYV<br>VCHYSPQALKGVQ<br>MYLVAKPCSQCSAK<br>NGVQCLKNLCMTR<br>EQCKNNPFDCQDA<br>ECNLECHHCGRLDK<br>EKCTCECMDGWDSP<br>DCSHLCKNKHKRC<br>GMSPGWPTKASCSL<br>NGYAVAKTYCRKM<br>CEMCHPLLDTSNVT<br>CCEGKLCDEGFVLD	ACGAGGCAAGCAGTCAAACCTCAAATTAAATATCTTGAGAC<br>TTTTTAAAGAGAAGGTTATCATTTTCTGCAAAGGAATATGAC<br>ACCGGTCTCAGTTATGCAAGTTTTGACGTACGTCATCGCGGG<br>CTTTTATTCTATAGAATGGCAAGGAACTTGCTCCGATATTCCC<br>AAACAGAACGATAGTCACGACGATTCATTCATTTTTGTTACTT<br>CAGAAAATAACGAGGAAGCGTCTTATTTAGAGACAACTGAG<br>ATACCTGTTCACGAAAGACTGAATCGTGGTTCACAAGAGTCC<br>TTTGCGGCTGATTTCTTGAAAGCAGAACCTATAATTTCGGATC<br>CCCTCTTCGAGGACGTGCCATGGAATAAATCCAGCGTCAAGC<br>AGTTGAGCATGAAAAGGGATGTATCACAGGGAGCATGGACT<br>CATATGAACAAGATGCAAATTCTCCAGTTGCATAATGTATAC<br>AGAGGTAATGTGACACCACCAGCGGGTAATATGGCATACAT<br>GGAATGGGACGAAGAACTTGAGAAACTTGCCCAGATATGGG<br>CAGATCGCTGTGTATTTGAACATGGTATGCCTAAAAATGATT<br>ACCCAGAAACTATAGGACAAAATCTGTATATAGGAACAGAT<br>CCCACGGGATATCTTGGACTATATATGTGGTATGAAGAATAT<br>CATAACTACGAACTGAGAAATGAACATTGTCTACCCAACAA<br>GCAATGTGGACATTATGTGCAAATGGCATGGTCCGAAAGCA<br>CCAAACTGGGATGTGCGATGACTAAATGCAGTATGCGCTACT<br>ACGTCGTTTGTCACTATAGTCCACAAGCTTTGAAAGGAGTGC<br>AAATGTATCTTGTAGCTAAACCGTGCTCGCAGTGTTCGGCTA<br>AAAATGGCGTGCAGTGCTTAAAAAACTTATGCATGACTCGA<br>GAACAATGCAAAAATAATCCTTTCGACTGTCAAGACGCTGA<br>ATGCAACTTAGAGTGTCATCACTGCGGTCGTTTGGATAAAGA<br>AAAATGCACGTGCGAATGTATGGACGGTTGGGATTCTCCGGA<br>TTGCTCACATTTATGCAAGAATAAGCACAAGAGGTGTGGCAT<br>GAGTCCTGGCTGGCCTACGAAAGCTTCCTGTTCCTTGAATGG<br>CTATGCAGTCGCTAAAACATATTGTCGTAAAATGTGTGAGAT

						PHSKSCQCILLCPGPLCDGKEAAVTQAALEGSAVCIRIIPHFVLCMIILRLFESVLWKYV*	GTGCCATCCTTTGCTGGACACATCGAATGTTACTTGCTGTGAGGGGAAGCTATGTGACGAAGGATTTGTTCTTGATCCGCACAGCAAATCATGCCAGTGCATTCTTTTATGCCCAGGTCCCCTTTGTGATGGCAAAGAAGCAGCCGTGACCCAAGCTGCCCTAGAAGGTAGTGCTGTTTGCATAAGAATCATCCCGCATTTTGTTCTCTGCATGATAATTCTCAGACTGTTTGAGAGTGTCCTATGGAAATACGTTTAAGACAATTTTTCATCTACAGTGGATGCGTGTGAATATTCGCATTATCAGAGATACATTAAAAAACTAACGCAAATTACTGAAAGTTAAATTTTCGGCAAATGTGTCAAAAACTCTGGTCTTGGTTGTATATAAATGATAACAGTGTGAGATCTTATTTTTCAATTACTTGCAATTTGTATTTCACAAGCGCCTGATCTCTAATACTGCATAAATATATATTAATGTTTTCTGAAGAATGAGGCACAGTTTTTAATGTTGAATGTTGTGTATTTAAGCAAAAAAAAATTTTATATAATTGTTTTTTAATAAAATTCTTGTCTCTTTCAAAAA

PhdCRI03	W4VS53	3,76E-72	35,207	PF00188	38845,87	MESPMTWDDELAEL<br>AQKHAESCIYGHDC<br>VGCRVLEQFTVGQN<br>VGMKWTVSSLVEVE<br>KDWSWFITTLYDEVS<br>RFPKNLVQSYVAGP<br>SDPYWQYGHFTQIV<br>WGKTWRIGCGWVL<br>YQEGYYNSSFYVCN<br>YGPGGNIYHKKMYT<br>EGDPCSACPQNSCC<br>GETCEKFNVTSKHK<br>GLCKIIDEQLPPDGF<br>GPHGVDEFFFCNFH<br>NETDCEMTTRGKDD<br>WKLVETIGGNWITL<br>VLNGGESTVMTMN<br>TSFKSPCHIFCFKILIR<br>SGAEVDYQPYGFKG<br>VVTFLADDGKSLDF<br>KIWGNTVRQLFSTY<br>RLDVNLPTDAPITVS<br>ITLSVPHGVPPQFFDI<br>KEMSAKENHCQWN<br>S*	GGGAAACTTCTTTTCGGACTCTCTCTCGTATTTTAAACCACGA<br>TACTTCTACCAAATAACGAAGAACTTTTCTGTTTTTTACTTTA<br>AAAATTCCATTTTATTAAACGTATTTATATGGCTTTACATGAT<br>TCATACATTTTGTCATTCATGTCTTCATTCACAAGTCTGATAC<br>AGATGGCCTGTCTGTCATTTAGCTATTCCACTGACAATGGTTC<br>TCTTTCGCCGACATTTCTTTGATATCGAAAAACTGGGGTGGC<br>ACTCCATGAGGCACGGAAAGTGTTATAGAGACCGTTATAGG<br>GGCATCGGTGGGTAGGTTGACATCTAGTCTATAGGTAGAGAA<br>CAGTTGTCTGACTGTATTTCCCCAGATCTTGAAATCTAAAGA<br>CTTGCCATCGTCGGCAAGAAAAGTAACAACACCCTTGAAAC<br>CGTAGGGTTGATAATCGACTTCTGCACCAGAGCGAATAAGA<br>ATCTTGAAGCAGAAGATGTGGCAAGGGCTTTTAAAACTCGTG<br>TTCATTGTCATGACTGTAGATTCCCCTCCATTTAACACGAGGG<br>TGATCCAGTTGCCGCCGATTGTTTCGACAAGTTTCCAGTCATC<br>CTTTCCTCGGGTCGTCATCTCGCAGTCGGTTTCATTGTGGAAG<br>TTGCAGAAGAAAAATTCGTCGACACCGTGAGGACCAAATCC<br>ATCTGGGGGCAGCTGTTCGTCGATTATTTTGCATAGACCTTTG<br>TGCTTGCTTGTTACGTTAAATTTTTCACATGTCTCTCCACAGC<br>AAGAGTTTTGGGGACAAGCCGAACAAGGATCGCCTTCTGTGT<br>ACATTTTTTTGTGATAAATATTACCGCCTGGTCCATAATTGCA<br>AACGTAGAAACTGCTGTTATAGTATCCTTCTTGGTACAGGAC<br>CCATCCACAACCTATTCTCCAGGTTTTTCCCCACACTATCTGG<br>GTGAAATGTCCGTACTGCCAATAGGGATCACTGGGTCCTGCC<br>ACGTACGACTGCACCAAGTTTTTAGGAAAGCGAGAAACTTC<br>GTCGTAAAGCGTTGTGATGAACCAACTCCAGTCTTTTTCCACT<br>TCCACCAGCGAGGAGACTGTCCACTTCATTCCTACATTCTGC<br>CCAACCGTAAACTGCTCTAGCACTCGACACCCCACACAATC<br>GTGTCCGTAGATGCAGCTTTCTGCATGCTTCTGAGCAAGCTC

							GGCCAGCTCATCGTCCCAGGTCATGGGACTTTCCATGGTGCCTACGGAAAGATAGAGCTGTTTCCCTTAATTCACGGATCTCTGCTCCTATGAGCTTGGTAACACCTAGTGAAATAAACAACAACAACATAAAGATTAGTTCAGGGGGGGGAAATCTGGATGTATAAGGTGAAAAGGCATGTAAAAATGTGGCACTTTGTCAAATTCACGATTTAAAATAAAAATCAAAAGATATCAAGAAATCGATCTCGAATCATT

PhdCRI04	P10736	8,61E-10	27,072	PF00188	19628,11	MKFDPAMEMRDFK<br>RDTLNAHNDYRTL<br>HGCPPLAMSEELTN<br>TAQAWADKLADRG<br>ILQYSENPSLGENICL<br>VDLDHSPSRKGELIV<br>KEWYKEIDNYNYSD<br>PGWKKSSYRVSQLL<br>WKSTSEIGVGVSKVP<br>GQNRAYVVVNYRP<br>AGNNNMPGEFERN<br>VLPLQKRRDDPPWK<br>RRP*	GGGATGGGGGAATTTCTCTTTCTGGATTCTGCTGGAAAGGAT<br>TTCATTTCTGCATTTTACAACTAATTAGGAAGCTCTGAAGTTG<br>CGACAAAATCCTGAAGCAATTTTATGGAGTTAGGTTCTTAAG<br>GTCCGAAGCCATCCAAACACACTCTTTTGACCTGCGTCTCTG<br>CTCTGCGTCAGTCGGTGAAGCTGTGAATCGCAAACAAACTGC<br>GACAGAAACATGAAATTTGATCCAGCAATGGAGATGCGCGA<br>CTTCAAGCGAGACACGTTGAACGCTCACAACGACTACAGGA<br>CTCTCCATGGATGTCCGCCCCTGGCCATGTCCGAAGAGCTCA<br>CCAACACAGCGCAAGCTTGGGCCGACAAGCTAGCCGACAGA<br>GGAATACTTCAGTATAGCGAGAATCCAAGTCTCGGAGAAAA<br>CATTTGCCTTGTGGATCTTGATCATTCGCCATCTCGCAAAGGT<br>GAGCTTATTGTAAAAGAGTGGTATAAGGAGATTGACAACTA<br>CAACTATAGCGACCCAGGATGGAAAAAGAGTTCCTACCGCG<br>TCTCCCAGCTTCTATGGAAAAGCACAAGTGAGATCGGCGTTG<br>GTGTCTCGAAGGTGCCGGGACAGAATCGAGCGTATGTGGTG<br>GTCAATTACAGACCGGCGGGCAATAACAATATGCCCGGCGA<br>ATTCGAGCGAAATGTACTACCCCTCCAGAAGAGGAGGGATG<br>ACCCTCCCTGGAAAAGGCGTCCTTGATTATCTTTCCATAGAT<br>ACATGGATGTTCTAAGCACCGTATTTGTCATGACATGTCATC<br>GAAAATATTATGTCATGAGACTGTCATTTTGTAACAGAACTA<br>TATACCAAAACCATAAGAAATGTTCATATTTTTACAGAGATG<br>GACAGTATATTTTTAAGTAAATCTAATATTTATTTGTGTAGTA<br>TATTTTTAGGGAGTTCATAATTGTAATATTCCAAATACAATTT<br>ATAGATACACATAAATAGAAGCAGGAAACGATAGTAGTTTC<br>CAATTTAATTTTATTTGAATATAGTCATAATAAAGGTCTCTTT<br>ATACGCAGAGTAATAAAAACTAAATTGCTGCATATTTTGATG<br>AGTATGATTAATTTAGCAAACGGGAAAGTACTTAAGGAAAC<br>CGTTATATCTACATAACTGTGGTAAATTAAAAGTAGTATATT

							GCATATTGAATATACATATTCGGCAGTAACTATATTGTATATT<br>TTGGGGTAAAACGAGAATGCATATGTATATTACATATAATAT<br>ACTTCTGGAAAGCGTATTTTCCATCTCTGTATCATACATCTGT<br>TCAGAAGAGTGTCCTTCAGTTAATCACTTAGAATTTTATTTAT<br>TTATTTGTTTATTTATTATTACTGTAAAATCCAGTGCTGTAATG<br>AGAAGGAATTCTGACGATTATTTGAAAACGTACTTTTAAATT<br>ACTTTTTTTCAATAACAGAAATATAGATTATCACAAACAAAC<br>CGCGTTTGTCAAGCAAGACTGTTTGATTCCTATTAAAGTACTT<br>CATTTCATTCACCGTGTAAAAAAAAAGGTCTATGTACACAAT<br>GCTCGCTATGTCTAAAAAACATGTTCTTCTCCCACATCTTTAC<br>CTCTTCTAGTCAATTACTTCGTCCTTTCCAAGTCATCACTGTG<br>CCAAATTCATCTCCGAATGACTATTGACCTCAACTTGCCTCA<br>AACAGTCAGAAATTAATTTTACGCAGAATATATGGGAACTA<br>ATTGTTTCTTTATTAGATGCATGAAAGAAGATCAGTTAGGGA<br>AGCAACACATATAGTACCAATCAATATACAGTTCAAAAAGG<br>ATCTTCGTGCAATCTATTGATTTTAATTGTTAACAATTTATCA<br>ACGAAGATAATGGCAGTTTTCCATGAAATGAAAAGTTGTATA<br>ATATGATTATCAGCTAAATTTTTTTATTCATAGATGATTAATA<br>TTTTATGTAAAATTGAAAATAAAATTTACAACACAC

PhdCRI05	P10736	3,65E-09	27,778	PF00188	21128,55	YSSSRGEGTHTKTER<br>QTDPAMEMRDFKR<br>DTLNAHNDYRTLH<br>GCPPLAMSEELTNT<br>AQAWADKLADRGI<br>LQYSENPSLGENICL<br>VDLDHSPSRKGELIV<br>KEWYKEIDNYNYSD<br>PGWKKSSYRVSQLL<br>WKSTSEIGVGVSKVP<br>GQNRAYVVVNYRP<br>AGNNNMPGEFERN<br>VLPLQKRRDDPPWK<br>RRP*	CGCCAACCGCCAAGACAGAATCCGCCAAAGCGTTTTTCTTGG<br>GCACCGGGTCTCTGGGACAAACACTCTTCATCTCTCGGAGTC<br>GGTGGTGGGAAGTTTGCCAGTGAAAAGTGATATTCGAGCAG<br>CCGTGGAGAAGGAACTCACACGAAGACAGAAAGACAGACA<br>GATCCAGCAATGGAGATGCGCGACTTCAAGCGAGACACGTT<br>GAACGCTCACAACGACTACAGGACTCTCCATGGATGTCCGC<br>CCCTGGCCATGTCCGAAGAGCTCACCAACACAGCGCAAGCT<br>TGGGCCGACAAGCTAGCCGACAGAGGAATACTTCAGTATAG<br>CGAGAATCCAAGTCTCGGAGAAAACATTTGCCTTGTGGATCT<br>TGATCATTCGCCATCTCGCAAAGGTGAGCTTATTGTAAAAGA<br>GTGGTATAAGGAGATTGACAACTACAACTATAGCGACCCAG<br>GATGGAAAAAGAGTTCCTACCGCGTCTCCCAGCTTCTATGGA<br>AAAGCACAAGTGAGATCGGCGTTGGTGTCTCGAAGGTGCCG<br>GGACAGAATCGAGCGTATGTGGTGGTCAATTACAGACCGGC<br>GGGCAATAACAATATGCCCGGCGAATTCGAGCGAAATGTAC<br>TACCCCTCCAGAAGAGGAGGGATGACCCTCCCTGGAAAAGG<br>CGTCCTTGATTATCTTTCCATAGATACATGGATGTTCTAAGCA<br>CCGTATTTGTCATGACATGTCATCGAAAATATTATGTCATGA<br>GACTGTCATTTTGTAACAGAACTATATACCAAAACCATAAGA<br>AATGTTCATATTTTTACAGAGATGGACAGTATATTTTTAAGTA<br>AATCTAATATTTATTTGTGTAGTATATTTTTAGGGAGTTCATA<br>ATTGTAATATTCCAAATACAATTTATAGATACACATAAATAG<br>AAGCAGGAAACGATAGTAGTTTCCAATTTAATTTTATTTGAA<br>TATAGTCATAATAAAGGTCTCTTTATACGCAGAGTAATAAAA<br>ACTAAATTGCTGCATATTTTGATGAGTATGATTAATTTAGCAA<br>ACGGGAAAGTACTTAAGGAAACCGTTATATCTACATAACTGT<br>GGTAAATTAAAAGTAGTATATTGCATATTGAATATACATATT<br>CGGCAGTAACTATATTGTATATTTTGGGGTAAAACGAGAATG

							CATATGTATATTACATATAATATACTTCTGGAAAGCGTATTTT<br>CCATCTCTGTATCATACATCTGTTCAGAAGAGTGTCCTTCAGT<br>TAATCACTTAGAATTTTATTTATTTATTTGTTTATTTATTATTAC<br>TGTAAAATCCAGTGCTGTAATGAGAAGGAATTCTGACGATTA<br>TTTGAAAACGTACTTTTAAATTACTTTTTTTCAATAACAGAAA<br>TATAGATTATCACAAACAAACCGCGTTTGTCAAGCAAGACTG<br>TTTGATTCCTATTAAAGTACTTCATTTCATTCACCGTGTAAAA<br>AAAAAGGTCTATGTACACAATGCTCGCTATGTCTAAAAAAC<br>ATGTTCTTCTCCCACATCTTTACCTCTTCTAGTCAATTACTTCG<br>TCCTTTCCAAGTCATCACTGTGCCAAATTCATCTCCGAATGAC<br>TATTGACCTCAACTTGCCTCAAACAGTCAGAAATTAATTTTA<br>CGCAGAATATATGGGAACTAATTGTTTCTTTATTAGATGCATG<br>AAAGAAGATCAGTTAGGGAAGCAACACATATAGTACCAATC<br>AATATACAGTTCAAAAAGGATCTTCGTGCAATCTATTGATTTT<br>AATTGTTAACAATTTATCAACGAAGATAATGGCAGTTTTCCA<br>TGAAATGAAAAGTTGTATAATATGATTATCAGCTAAATTTTTT<br>TATTCATAGATGATTAATATTTTATGTAAAATTGAAAATAAA<br>ATTTACAACACAC

PhdCRI06	P0DPU1	6,47E-25	32,227	PF00188	26001,9	LLPFSIPLHRLDHSF<br>NRRLSTLAAQIVHL<br>KANSLCNKSVFALG<br>MKSFVAFGQGYICP<br>GSKLSPQQRGEFLAK<br>HNHLRAVLANGKS<br>VDKDGVRLPPARN<br>MVPVGWSCEAEQV<br>AQRWAEHCVFEHS<br>DRNFRKGLGENLYM<br>SYPPTDEGVKIGAER<br>ASQSWWNELPELYH<br>FSMNRDLVLTPQVF<br>GTGIGHWTQMAWA<br>NTTHIGCAIKKCEQ<br>MVLVVCEYAPAGN<br>VINRKIYEPR*	TTTTTTTTGAACAGTTAATTTTTTATCAGTGCAAATCAAAATC<br>ACGAGTGTATCACTCAAAAAAGAGTTATCACATTATTGCTTA<br>TCGTGGTTCGTAGATTTTTCGGTTGATTACATTGCCGGCAGGG<br>GCATATTCACAGACGACCAAGACCATTTGTTCGCACTTCTTG<br>ATAGCGCATCCAATATGAGTGGTGTTCGCCCAAGCCATCTGT<br>GTCCAGTGTCCAATGCCTGTACCAAACACTTGAGGTGTCAAG<br>ACGAGATCTCTGTTCATTGAGAAGTGGTAAAGTTCTGGAAGC<br>TCGTTCCACCACGACTGACTGGCTCTCTCAGCACCAATCTTC<br>ACACCTTCGTCTGTTGGCGGATATGACATGTAGAGATTTTCA<br>CCAAGTCCTTTCCTGAAGTTCCGGTCAGAATGCTCGAAGACG<br>CAATGTTCTGCCCATCTCTGCGCTACCTGTTCTGCTTCGCAGC<br>TCCAGCCCACTGGCACCATATTCCTAGCAGGTGGCAGTCGCA<br>CACCATCCTTGTCTACTGACTTCCCGTTTGCAAGTACTGCACG<br>CAGATGATTATGCTTCGCGAGGAATTCTCCACGTTGCTGTGG<br>TGACAGTTTGCTGCCTGGGCATATATAGCCCTGTCCAAATGC<br>TACAAAGCTTTTCATTCCCAAAGCAAACACAGATTTGTTACA<br>CAAGGAATTTGCTTTAAGATGCACGATTTGTGCAGCAAGTGT<br>GGAAAGGCGACGGTTAAAAGAGTGATCGAGGCGGTGGAGG<br>GGAATTGAGAAGGGGAGGAG
PhdCRI07	C0ITL3	1,63E-27	35,028	PF00188	22423.14<br>**20199.51**	MVYCGSLALIFVFSA<br>VYDVSG**QTCQGSQ**<br>**LTPQQRQDFLDTH**<br>**NRLRQQLAAGQAK**<br>**DKNGRNMPAAAK**<br>**MPSLTWSCEAEEIA**<br>**QKWAGNCQFQHSS**<br>**RSFRKNFGENIYMS**<br>**WPANTEAVNSAGQ**	CGCGTAAAAATTTATTATTTCTAATCACAATTGTTACAATGAT<br>TATTCAATGAAAATGAGCACAATGATCATCTTTCCTCGTAAA<br>TTTTGCTGTTCAGCATGTTTCCCGCCGGATCATATTCGCAAAC<br>TACGAGAACCTTATCACCGCAATTTTTAACACCGCAGCCAAG<br>CTTTGTAGTTTTTTGCCAAGCCATCTGTGTCCAGTGACCAACA<br>CCCCAACTGAAAACTTGATTGGACAGGACGTTATTTGGGTTT<br>TTGTCATAACTGTAGAGTTCTGGAAGCTCGTCCCACCATGAC<br>TGACTAGCCTTTTGACCGGCAGAGTTCACGGCTTCAGTGTTTG<br>CCGGCCATGACATGTAGATATTTTCTCCAAAATTCTTTCGGA

						**KASQSWWDELPEL<br>YSYDKNPNNVLSN<br>QVFSWGVGHWTQ<br>MAWQKTTKLGCG<br>VKNCGDKVLVVCE<br>YDPAGNMLNSKIYE<br>ER***	AGCTTCTACTGGAATGTTGAAATTGACAGTTACCTGCCCATTT<br>TTGGGCAATTTCTTCCGCTTCGCAGCTCCAAGTCAGCGAAGG<br>CATTTTTGCTGCTGCAGGCATATTTCTTCCATTTTTGTCTTTTG<br>CTTGTCCAGCAGCAAGTTGTTGGCGTAAACGATTGTGAGTAT<br>CAAGAAAATCTTGGCGCTGTTGCGGCGTTAATTGACTGCCTT<br>GGCAAGTTTGACCGGAGACGTCGTAAACAGCAGAAAACACA<br>AAAATAAGGGCTAGCGAGCCACAATACACCATCGCGCTGAA<br>TTCAGAAAAATGGCTGAG

PhdCRI08	W4VS53	4,88E-100	39,192	PF00188	47087.13<br><br>**44785.28**	MNYIIFLAACIFSLFL<br>HSLC**QECPSIYLRYS**<br>**REHSFCKPKSATCP**<br>**VYHSGVTTAERRQI**<br>**LDIHNKYRSLVATG**<br>**REKKAGGLSQAAD**<br>**MLQMVWDTELAA**<br>**VAQRRAEQCIYEHD**<br>**CSNCRRVENFSVG**<br>**QNIAYREKQCKGT**<br>**GKCPLNSTPDWPD**<br>**VIKMFYDEVAVFD**<br>**KKYVKSVLFVPGKE**<br>**YGHFTQMVWSKT**<br>**WRIGCGFVAFSKG**<br>**NGYRQLYVCNYGP**<br>**SGNVVKTPMYKIG**<br>**PKCSSCPMNSCCG**<br>**RSCKRANYGGLCR**<br>**MNRNNEVPVYRPE**<br>**QDHIFYCDFRNAEG**<br>**DCSAAVVGPNKW**<br>**KYVKALGGSFAEV**<br>**VLKGGETSTLKLKK**<br>**AMKPEQNFCLVVK**<br>**YRSGPALDGQKSS**<br>**NKATVTFEMPNAR**<br>**TNTMVLPKGRDTQ**	TTTTTTTTTTTTTTAAAATCGAAGACTTTTATTTTCTATGACATT<br>GCAAAAAGTGACGATCCACAGGAAAAAGTTTAAGAATTTGA<br>CTCCGGAATGTTTAAATGTTACTATAGAGAGTCGTTTGTGCG<br>ATCATATACGTGAAAATATGCCTGACGAATTTTCAACCAAAG<br>ATACAAATTCATTATCATAGCAGGGATTGCTGTTTATCATTAA<br>CATAAGTAATAGTTTTTTACATAATACTGAATAAACGTATTA<br>CAGCATCAAATAATAAAATATCTCATAACACGGGTATTTGGA<br>AACTATTGTCAAATTCCATACAAAGCAGTAGAACTGCATAGT<br>TAAAAATTTCTAAATCAAAGATTTACTTCTATGAATTTAGTTA<br>TCTCCATGGAGATCATTATGTACATCTAAAGCGAACTCTGAT<br>CTCGAGAAGAGTTCCAAAATTTTGACATGAGTCCTAAAAAC<br>AATTAAAACTCCTTCAGCACACTTAAAATGTCCTCAAATTAA<br>TTGATGCTGAGTCACAACGAGCAGTTTTGATTTAAAAAAAAC<br>TACATAAATTACTATTTAAGTCATTAAATTATTCATAAATATT<br>TAATGTATTAGAAGAAAATTGTAATAAGTTAACATGAGAAA<br>TATGAATGCAAGAAATAATGGAGTTAACAGAATCAAATTCTT<br>TTGCATTAACATAGTAAGGCATCAGTCGGAATAAGAAATGA<br>GGTATGCCAAAGTGAATATTTTTTAAAACATCTCGCGGAATT<br>TTCATATTCTTCCATGCAGATATTTCTTCTGTTGCTTGTGAAAA<br>TGTAGAGGCAGCGGTGGCTCGTGCACATTCTTAGTGGTAAGG<br>CCCGAAATGGATTTCTTAGCTGATAGAAACATGGATGTTGTG<br>CTTAAATATACTAGGTAAGTTTGTTTTTTAAACAGAAATGTGT<br>AGCACGGATGTTTAAGAGTTTGATTTTAAAGTGACATATTAG<br>CATATTCTTTTGCCATTGATCACTCTCTAAAGAAGGTATCCGT<br>AATAGATTGTTCACTGAATTTATTTTTTAAGTTTTAAAATACT<br>GATTCCAATAGGTTAAAGTGACGATGGCAAGGCGACGACAA<br>CAAAAATGGGATAAAGATGACTGTAGCTTATAATACTGCACT<br>GCAAGTAAAATTAGTACCTCGTGGCTATTCACTTTCTGAACA

						<u>**EFFQYSISLGWRYDTLVSFTFSVARGDPTQFFNIQLVTAKNGRC***</u>	ATAATCAGTAATAGCTGGTTTAACATATTTAAAGCACGTTATACTGCCTTCCACAACGCTAGGGCCCAGTGAAACTGGTCTCAAGTTTTGCCAAGTCCAGACAGAAAATCGGAAGAAATGTCGAACGAACAGTGCGTAGTCTCAATGATGTCCAAAGCCAGGGAAAAGGGCACCATTTTTTGTGCCCAGCAAGAGGGGGCGAACATTGCATTCTGTTTATCTTTTAACTAGTAAAGTAGGGGGAACAAAAAAAAATTATAGATAAATTTTTAAAGCTACGTGATTTTTTCAGAAGGTGTACTTTTAGCAGTCTAAGTTTGATTTTAGTAACAATCATGGTTGCAGCGACAGCTCGGCTATCACGTCAAAATTCCAAAGTTCCCACGTAAGCCCAATAGTAATAACATAATTTTGCCTCGGTTATAACGGCGCAAAATTACCAGTAAACTGATACGTCAAAATAAATCCCCACGCATAAATTTCTTTAGTTCAGTTTTAGTTTTTACGCTGTCATATTTCGACATTTGGCACATTGTATTTAATCACTGGCGCCCTTTTTGGGTGAAGTGGGTAAAGTGCCTCTTCACCTAATTTTTTCAGTATAGTACACAAATCTCAGAATTGTTTAATTCTAAGAATATCGTATACATAAATTGCAAAGTATCAGCGAATGGAGATATTTCAAAACAAATGCATAACATTACTTCTTTGCCCTGCCGTTTGCCGTTTGCATCTGAAAATGCTACAAAATATCTCTTAATAATAATTGAGGGGAAGTAACACTGAAAAATCTTGAGTCACACGTTGCTATGGCAATCGCGAGGACATCTCTCTCGCACTTTGGACCATGAGCCCGCGAAATGAAAAGATTTCCGTGGATGTGGGCTCTCACTTTGCAGTGAGAGCGAGATAGAAGCCCCTCTCAACAACATGCAAACAGGAACATTTTTACCGAAGTTTTTATAAATTGTTTTAATTTATTTCGTCTATGAGTGTGTTTCTTGTCAGAAGCTTACAAAAACATGCTAGAGAAATGCTATGATAGAAGACAGACCCCTCTTCGTCAAATATTTTCATTCAAGCGAGAACTTTTAATTGACATTCTAAAGAAACACCCATGTTATATGTTGATATTCGAAAGCTGACAAACTAAAACGAACGGGAGAATATACTAGA

							AGTACGAGGTGAATCAATGTTTAAAAAATTTTAATTACATAA<br>ATTGGGCAAAATACCACTGCCATCAAGATGGTCAAAATTAA<br>TGCAAATTATGTTCTTCCCGTTCCAATAGATATTTTGATTCAG<br>TTCGTGATGGATTATACAAGCTGACTGAAAAAATTGAAGCAT<br>ATAACTGTCATTGTAAAATACATCTAAAAGTAGTTTCCGACA<br>ATTTGATGCCTATTTAATAACTTTTTTCCTATGAGTTCATTATG<br>TGACTCTTGTGTTTTCAGCAGCTAGAAGAGCTACAGTGGCAT<br>TAGTTTATTTTTTCCCATTACATTATATTAAAATTGTGTGCACT<br>AATAATGCCTTATACCAAATTTAATAATATTTAGGAACATTTT<br>AAAATTAATTATCAATTATGGAGTTAAGATTTCAGCGGTTCC<br>TTGATTGCCGTTATAATCGAGATCAGGTCTGTAAGTAGAAAG<br>AGTGAGAAAATGGATATATAGAGTTAATTTATTTGTAAAAAA<br>AATTCAGTATATTTTATTTTTATTTATTTCGGTAATTTAAAGAG<br>CAGTTTATTCTTTGCCTTCAAATGAACATTAAATTAGAGGAC<br>ACCAATGATCTGTTTCCAGATTTCTTTTAAAAAGTCTGGTCGC<br>CTTATGCATATATCAACAATCATCCAAGTGTAAACTGATATT<br>GACATATATTTATCAACTGATATCGGATATCAAAGTAAAACT<br>TCTCAAAATTGCACAATGATTCGAAGTTTTAAATCAATGATA<br>CCGAGTCGTCATATTCCGATAAACAATCCATATATAGAAACA<br>GTACTGAGAAAAGTTCAGCGATAACTTTAGGCCTTAAAAATA<br>TGCTTATGAGAATTTCCGCAGCCGATGCAAAATTATCTGGGC<br>TTGTTGTCTTAAAAGGTACGAATTCTGACCTAGTTAAAAATG<br>CGCGTAAACTCAAAAGTCCTTAAATTTGTGAAAGAATATCAT<br>GCTGACCGCTTTAGACTCTCTAAAGTATTCTACAGGCTTTGAC<br>TTTCAAAACTGAAAATTACATGCTTTGTGATGTTGGCTGTTTC<br>CTTTCTATCATCTAGCATCTTCCATTCTTCGCAGTCACAAGCT<br>GAATGTTGAAAAACTGCGTTGGATCCCCTCGTGCAACGGAG<br>AAGGTAAAGGACACCAGAGTGTCATATCTCCAACCCAGACT

							AATACTGTATTGGAAGAACTCTTGGGTATCTCTACCTTTCGGA<br>AGAACCATTGTGTTTGTACGAGCATTAGGCATTTCGAAAGTT<br>ACAGTTGCTTTATTTGAAGACTTCTGACCGTCTAAAGCAGGA<br>CCACTTCTATACTTTACCACAAGACAAAAATTTTGCTCTGGTT<br>TCATCGCCTTTTTCAGTTTTAACGTGCTGGTCTCGCCTCCTTTC<br>AGAACAACTTCAGCAAAGGAACCTCCGAGAGCTTTCACATA<br>TTTCCATTTATTGGGTCCAACAACTGCAGCGCTACAGTCTCCT<br>TCCGCGTTCCGGAAATCGCAGTAGAAAATGTGGTCTTGCTCT<br>GGTCGGTAAACAGGAACTTCGTTGTTTCGATTCATCCGACAA<br>AGCCCACCATAATTTGCTCGTTTACAAGATCTTCCGCAGCAA<br>GAATTCATTGGACACGAGCTACACTTTGGTCCGATTTTATAC<br>ATAGGAGTTTTTACAACATTCCCACTTGGTCCATAATTGCAG<br>ACATAGAGCTGCCTGTAACCGTTTCCCTTACTGAAAGCAACA<br>AATCCACAGCCTATTCTCCATGTCTTCGACCAAACCATCTGG<br>GTAAAATGTCCATACTCTTTTCCAGGAACAAACAATACTGAT<br>TTCACATATTTTTTATCGAATACGGCAACTTCATCGTAAAACA<br>TTTTGATGACGTCAGGCCAATCAGGCGTGGAATTTAAAGGGC<br>ATTTTCCGGTTCCCTTGCACTGCTTTTCCCGATATGCTATATTC<br>TGGCCCACAGAGAAGTTTTCTACTCTTCTACAGTTGCTACAGT<br>CGTGTTCGTAAATGCACTGTTCAGCTCTTCTTTGGGCAACAGC<br>AGCCAGTTCTGTGTCCCAAACCATCTGCAACATGTCTGCTGC<br>TTGGGATAAACCTCCTGCCTTCTTTTCTCTTCCTGTTGCAACTA<br>GGCTTCTGTACTTATTGTGAATATCTAAGATCTGTCGCCTTTC<br>AGCGGTCGTCACTCCTGAATGGTATACTGGGCAAGTGGCGCT<br>TTTAGGTTTGCAGAAGGAATGTTCTCTGCTGTAGCGCAAGTA<br>GATGGAAGGACACTCCTGACATAACGAATGCAGGAAAAGCG<br>AGAAGATGCATGCAGCGAGAAATATAATGTAATTCATTTGG<br>AAACAGTCTTCTTTATTACGTTCAATAAATTTTGGATGCTTCG

							CTTGCACAAGCTAACGGCGAAAAGGAAGGTGATTTTTTTTTA AGTTCACAGCGTCTACCTAGTACTAATGATTTCAACCTTGGA ACAATTAGCAGCTGTCACTAAAATGTTTCAAGCACAAAATTA ACCCGTCAAAGATGCTCAGAGTCCGTTTCATAAGAAG

PhdCRI09	W4VS53	3,24E-77	39,577	PF00188	39647,68	KKITFLFAVSLCKRSI<br>QNLLNVIKVWDTEL<br>AAVAQRRAEQCIYE<br>HDCSNCRRVENFSV<br>GQNIAYREKQCKGT<br>GKCPLNSTPDWPDV<br>IKMFYDEVAVFDKK<br>YVKSVLFVPGKEYG<br>HFTQMVWSKTWRI<br>GCGFVAFSKGNGYR<br>QLYVCNYGPSGNVV<br>KTPMYKIGPKCSSCP<br>MNSCCGRSCKRANY<br>GGLCRMNRNNEVP<br>VYRPEQDHIFYCDFR<br>NAEGDCSAAVVGP<br>NKWKYVKALGGSF<br>AEVVLKGGETSTLKL<br>KKAMKPEQNFCLVV<br>KYRSGPALDGQKSS<br>NKATVTFEMPNART<br>NTMVLPKGRDTQEF<br>FQYSISLGWRYDTLV<br>SFTFSVARGDPTQFF<br>NIQLVTAKNGRC*	TTTTTTTTTTTTTTTAAAATCGAAGACTTTTATTTTCTATGACATT<br>GCAAAAAGTGACGATCCACAGGAAAAAGTTTAAGAATTTGA<br>CTCCGGAATGTTTAAATGTTACTATAGAGAGTCGTTTGTGCG<br>ATCATATACGTGAAAATATGCCTGACGAATTTTCAACCAAAG<br>ATACAAATTCATTATCATAGCAGGGATTGCTGTTTATCATTAA<br>CATAAGTAATAGTTTTTTACATAATACTGAATAAACGTATTA<br>CAGCATCAAATAATAAAATATCTCATAACACGGGTATTTGGA<br>AACTATTGTCAAATTCCATACAAAGCAGTAGAACTGCATAGT<br>TAAAAATTTCTAAATCAAAGATTTACTTCTATGAATTTAGTTA<br>TCTCCATGGAGATCATTATGTACATCTAAAGCGAACTCTGAT<br>CTCGAGAAGAGTTCCAAAATTTTGACATGAGTCCTAAAAAC<br>AATTAAAACTCCTTCAGCACACTTAAAATGTCCTCAAATTAA<br>TTGATGCTGAGTCACAACGAGCAGTTTTGATTTAAAAAAAAC<br>TACATAAATTACTATTTAAGTCATTAAATTATTCATAAATATT<br>TAATGTATTAGAAGAAAATTGTAATAAGTTAACATGAGAAA<br>TATGAATGCAAGAAATAATGGAGTTAACAGAATCAAATTCTT<br>TTGCATTAACATAGTAAGGCATCAGTCGGAATAAGAAATGA<br>GGTATGCCAAAGTGAATATTTTTTAAAACATCTCGCGGAATT<br>TTCATATTCTTCCATGCAGATATTTCTTCTGTTGCTTGTGAAAA<br>TGTAGAGGCAGCGGTGGCTCGTGCACATTCTTAGTGGTAAGG<br>CCCGAAATGGATTTCTTAGCTGATAGAAACATGGATGTTGTG<br>CTTAAATATACTAGGTAAGTTTGTTTTTTAAACAGAAATGTGT<br>AGCACGGATGTTTAAGAGTTTGATTTTAAAGTGACATATTAG<br>CATATTCTTTTGCCATTGATCACTCTCTAAAGAAGGTATCCGT<br>AATAGATTGTTCACTGAATTTATTTTTTAAGTTTTAAAATACT<br>GATTCCAATAGGTTAAAGTGACGATGGCAAGGCGACGACAA<br>CAAAAATGGGATAAAGATGACTGTAGCTTATAATACTGCACT<br>GCAAGTAAAATTAGTACCTCGTGGCTATTCACTTTCTGAACA

							ATAATCAGTAATAGCTGGTTTAACATATTTAAAGCACGTTAT<br>ACTGCCTTCCACAACGCTAGGGCCCAGTGAAACTGGTCTCAA<br>GTTTTGCCAAGTCCAGACAGAAAATCGGAAGAAATGTCGAA<br>CGAACAGTGCGTAGTCTCAATGATGTCCAAAGCCAGGGAAA<br>AGGGCACCATTTTTTGTGCCCAGCAAGAGGGGGCGAACATT<br>GCATTCTGTTTATCTTTTAACTAGTAAAGTAGGGGGAACAAA<br>AAAAAATTATAGATAAATTTTTAAAGCTACGTGATTTTTTCA<br>GAAGGTGTACTTTTAGCAGTCTAAGTTTGATTTTAGTAACAAT<br>CATGGTTGCAGCGACAGCTCGGCTATCACGTCAAAATTCCAA<br>AGTTCCCACGTAAGCCCAATAGTAATAACATAATTTTGCCTC<br>GGTTATAACGGCGCAAAATTACCAGTAAACTGATACGTCAA<br>AATAAATCCCCACGCATAAATTTCTTTAGTTCAGTTTTAGTTT<br>TTACGCTGTCATATTTCGACATTTGGCACATTGTATTTAATCA<br>CTGGCGCCCTTTTTGGGTGAAGTGGGTAAAGTGCCTCTTCAC<br>CTAATTTTTTCAGTATAGTACACAAATCTCAGAATTGTTTAAT<br>TCTAAGAATATCGTATACATAAATTGCAAAGTATCAGCGAAT<br>GGAGATATTTCAAAACAAATGCATAACATTACTTCTTTGCCC<br>TGCCGTTTGCCGTTTGCATCTGAAAATGCTACAAAATATCTCT<br>TAATAATAATTGAGGGGAAGTAACACTGAAAAATCTTGAGT<br>CACACGTTGCTATGGCAATCGCGAGGACATCTCTCTCGCACT<br>TTGGACCATGAGCCCGCGAAATGAAAAGATTTCCGTGGATGT<br>GGGCTCTCACTTTGCAGTGAGAGCGAGATAGAAGCCCCTCTC<br>AACAACATGCAAACAGGAACATTTTTACCGAAGTTTTTATAA<br>ATTGTTTTAATTTATTTCGTCTATGAGTGTGTTTCTTGTCAGAA<br>GCTTACAAAAACATGCTAGAGAAATGCTATGATAGAAGACA<br>GACCCCTCTTCGTCAAATATTTTCATTCAAGCGAGAACTTTTA<br>ATTGACATTCTAAAGAAACACCCATGTTATATGTTGATATTC<br>GAAAGCTGACAAACTAAAACGAACGGGAGAATATACTAGA

							AGTACGAGGTGAATCAATGTTTAAAAAATTTTAATTACATAA<br>ATTGGGCAAAATACCACTGCCATCAAGATGGTCAAAATTAA<br>TGCAAATTATGTTCTTCCCGTTCCAATAGATATTTTGATTCAG<br>TTCGTGATGGATTATACAAGCTGACTGAAAAAATTGAAGCAT<br>ATAACTGTCATTGTAAAATACATCTAAAAGTAGTTTCCGACA<br>ATTTGATGCCTATTTAATAACTTTTTTCCTATGAGTTCATTATG<br>TGACTCTTGTGTTTTCAGCAGCTAGAAGAGCTACAGTGGCAT<br>TAGTTTATTTTTTCCCATTACATTATATTAAAATTGTGTGCACT<br>AATAATGCCTTATACCAAATTTAATAATATTTAGGAACATTTT<br>AAAATTAATTATCAATTATGGAGTTAAGATTTCAGCGGTTCC<br>TTGATTGCCGTTATAATCGAGATCAGGTCTGTAAGTAGAAAG<br>AGTGAGAAAATGGATATATAGAGTTAATTTATTTGTAAAAAA<br>AATTCAGTATATTTTATTTTTATTTATTTCGGTAATTTAAAGAG<br>CAGTTTATTCTTTGCCTTCAAATGAACATTAAATTAGAGGAC<br>ACCAATGATCTGTTTCCAGATTTCTTTTAAAAAGTCTGGTCGC<br>CTTATGCATATATCAACAATCATCCAAGTGTAAACTGATATT<br>GACATATATTTATCAACTGATATCGGATATCAAAGTAAAACT<br>TCTCAAAATTGCACAATGATTCGAAGTTTTAAATCAATGATA<br>CCGAGTCGTCATATTCCGATAAACAATCCATATATAGAAACA<br>GTACTGAGAAAAGTTCAGCGATAACTTTAGGCCTTAAAAATA<br>TGCTTATGAGAATTTCCGCAGCCGATGCAAAATTATCTGGGC<br>TTGTTGTCTTAAAAGGTACGAATTCTGACCTAGTTAAAAATG<br>CGCGTAAACTCAAAAGTCCTTAAATTTGTGAAAGAATATCAT<br>GCTGACCGCTTTAGACTCTCTAAAGTATTCTACAGGCTTTGAC<br>TTTCAAAACTGAAAATTACATGCTTTGTGATGTTGGCTGTTTC<br>CTTTCTATCATCTAGCATCTTCCATTCTTCGCAGTCACAAGCT<br>GAATGTTGAAAAACTGCGTTGGATCCCCTCGTGCAACGGAG<br>AAGGTAAAGGACACCAGAGTGTCATATCTCCAACCCAGACT

							AATACTGTATTGGAAGAACTCTTGGGTATCTCTACCTTTCGGA<br>AGAACCATTGTGTTTGTACGAGCATTAGGCATTTCGAAAGTT<br>ACAGTTGCTTTATTTGAAGACTTCTGACCGTCTAAAGCAGGA<br>CCACTTCTATACTTTACCACAAGACAAAAATTTTGCTCTGGTT<br>TCATCGCCTTTTTCAGTTTTAACGTGCTGGTCTCGCCTCCTTTC<br>AGAACAACTTCAGCAAAGGAACCTCCGAGAGCTTTCACATA<br>TTTCCATTTATTGGGTCCAACAACTGCAGCGCTACAGTCTCCT<br>TCCGCGTTCCGGAAATCGCAGTAGAAAATGTGGTCTTGCTCT<br>GGTCGGTAAACAGGAACTTCGTTGTTTCGATTCATCCGACAA<br>AGCCCACCATAATTTGCTCGTTTACAAGATCTTCCGCAGCAA<br>GAATTCATTGGACACGAGCTACACTTTGGTCCGATTTTATAC<br>ATAGGAGTTTTTACAACATTCCCACTTGGTCCATAATTGCAG<br>ACATAGAGCTGCCTGTAACCGTTTCCCTTACTGAAAGCAACA<br>AATCCACAGCCTATTCTCCATGTCTTCGACCAAACCATCTGG<br>GTAAAATGTCCATACTCTTTTCCAGGAACAAACAATACTGAT<br>TTCACATATTTTTTATCGAATACGGCAACTTCATCGTAAAACA<br>TTTTGATGACGTCAGGCCAATCAGGCGTGGAATTTAAAGGGC<br>ATTTTCCGGTTCCCTTGCACTGCTTTTCCCGATATGCTATATTC<br>TGGCCCACAGAGAAGTTTTCTACTCTTCTACAGTTGCTACAGT<br>CGTGTTCGTAAATGCACTGTTCAGCTCTTCTTTGGGCAACAGC<br>AGCCAGTTCTGTGTCCCAAACCTTTATTACGTTCAATAAATTT<br>TGGATGCTTCGCTTGCACAAGCTAACGGCGAAAAGGAAGGT<br>GATTTTTTTTTAAGTTCACAGCGTCTACCTAGTACTAATGATT<br>TCAACCTTGGAACAATTAGCAGCTGTCACTAAAATGTTTCAA<br>GCACAAAATTAACCCGTCAAAGATGCTCAGAGTCCGTTTCAT<br>AAGAAG

PhdCRI10	W4VS53	3,72E-09	31,707	PF00188	15589,47	NWTPSNDTVSQFLN<br>HFSPIGNWLSVVLQ<br>GGETTKLIFDRSFPTT<br>GAFCMRLVIRTGPN<br>VDGETESGNGVSYL<br>DYGRSKTKRALDFS<br>GIPKSFRQDFMPFRY<br>GFYARGATTKYTFEY<br>TVPAGSPPQFAEIKE<br>VSAKDGSCR*	ACAGTGGAGAAGACAGCAGGAGGTGAGAAAATTGTAATGA<br>ATATAATGCAGTTAAGTATCAAAATTTCCTATTTCACATTTTA<br>AGGAAGCTCAGGTGGGAATACAGATTCAAATTTCAAAGTAC<br>GTTAAAGGAATTTTCCATTAGGTAGCGCAATGGTGAAGTGAA<br>AAGATCCCAGCTATCACAATTAGGTGCCCCCTCCGTCTCTCT<br>AGTATTCACAAATGAATAGTAAAGTCTTCAGTCTCATGCAGG<br>TTTGTTTCTTATAGGATGCAATCATATTAGTGGAGATTGCCTG<br>TGTATGTCCATCGGCGCGGGTACTTTTTTGACAGCGAGCTATT<br>CGAGGCCTAAACATCAAAATGCTGGACCCAGGGTACATAAA<br>AAATTTTGGGTTCCAATAGCAGATCGCATTCTTTTTACAAGA<br>AGGGGGAGCAAAGCAACAATTACACATGAAATTCCTTTCGC<br>ATGTATACAAGAATTGCGCAAATGTGACGTTCAACAGCACTC<br>GATACACCGGTTAAGTGAAAACAAGAAACTGGAAGATTTTG<br>ATGCCTACGTGTAATATAATAATCGAATGGTGGATTTTAGAA<br>GATGGGGTAGATAGTAAAAAGTAAATATAGATATTCTCTTTG<br>AGGTAAATAATCTTCTAAATTACTTTCTGGAGGGCAGAAATG<br>AAATCCAGTCAGCAATTTGATTGAAATTGATTAGCGATGTAT<br>CTCTTCTAACTGAAAATTAACATCGTTATCCATCTGTTTCCAA<br>ACATGGAAAGAAATGCAATACATAGTACATAGTATAACAGC<br>TTTATCTAGTTTTTTCAAGGGTCAATAGAAAATGAAACGGGG<br>CATTGAGTCCTAGAGATGGGTTTCTTATTTATGTAAGTCATGA<br>TAAAGATACAGATAATAAGAAATGCGCAATTATTTATACTTG<br>TCAAGGGCCCAATATTTCGGCTGAAAAAGTTTGGCGAAAAG<br>ACTTAATGACAACTTTTTAAAGAAAAATAGCGTTAAACATTT<br>CCATGGAACATAAGTTCCTGTAGCCTGTATATTTTAGACCGT<br>ATCTCGGGAAATAATTGGAAAAATTTCGTGGAATTTATATTA<br>AATTTTTCAGGTAAATTCATATTTTAGTATCTTTAATAAAAAT<br>CGAAATATTTCAAGGTTTAAAGGTCGTATCTTCCAATTCACTT

							TTTGAATGTTGGTGATCTCCAGCAGAAATATTTTACGGATTTA<br>TATCTTATAGTGCTTTTTCATGCAAGTTTTACGCAGTTTCCTAA<br>ATGCGCATATTAATTAGAGATAAACTAATCATGTAAGTATGT<br>AAATAGTAATTTGTAAGTAAAATCCGATGTTGTATTACAGGG<br>AAAAAATTTTCTGACATGAAGTACTTAATTTGTAGTATCTTAA<br>GATATTCTCGCGAAGTCCAATTTTCAGTTGAACTCCAGCCTG<br>CTTAGAACGCGGTCTTGCAACGTTGTGGATCGGAGTTCGACT<br>CCCCGATAAGGCTTGGATGGTTCGGTGGTTGTCTCATCATAG<br>GGACTGAACGCCAGATGCAGGCAGCCTACTCCGGTTCAGCC<br>TGAGTGTGAGGAGACAAAATGCCTGTCCTGAATTGATAAAA<br>TCCACCTTCATTCATTGTAGTCACAGACGATCAGACTTATGTA<br>AGCAGAGTGTACAGATCTGCCAATGTCCTGGCCGGACCAAC<br>ACCTCTCATCGGGGGAGCATCTCCCCATCATAGGGGCTAGCG<br>TTGCCAGTCCATGTGCTGCCATTATAATACATTGGACAACAG<br>TTTCGGCAGAATATACGGGCACACTGTTTGTCGCAAAGGGAG<br>AGCTGATGTATTTAATTAAATTTGAGTCGAACGAGAAGCATG<br>AATGTGAATTGCTCCACGTTCTTAGCGTAGAAGTTCCTACGG<br>AATACTTAATAATAATAATAATAATACCCTGAGAATCACCAC<br>TGAAATTAGTAAAATATGCAATATCCTTTATTTTAGTTAGTAA<br>AGAGTGATAGTTTTTACATATGAAAAAAATGAACAATTCGA<br>GGAAAAATGAAAAAAATTATGAAGAATCGAAAAAGGTTAA<br>AATCATACCTTATTTTGGGCGTTTAAGTGCCGAATATCCTCTT<br>AATATTTAAGGCTATCCGCTATCATTAAATCTAAACCTCTTTT<br>GAATAGATGAAGCTGACATTCGCAGAGTATTTGATAGATTGG<br>AAAGTACCAGAAAAGAGTGTCTAGAAGGATAATTATACTTA<br>TATTGTACCCATTCATTAACATCAAGCGCATCTCTTCACTCGA<br>AAGTGAGCTACAATCTATTTTAAAGCATGAATCTTGGTTTTGC<br>ACAAAGTAATCGGGGTGTAGGGCGCCATTTGCCAGTAACTAT

							AGGTACTGAGTGAAATACACGTTGAAGACACAGTTTTTGAG<br>AAATAATTCATCCTATTATATTGCATAAATCTGGTAAATGGTT<br>TTTCAAATTTGAACGTGGGACAGCATATTTATTTATCACTCTA<br>AAACACAGTTCTAACTGCGTATTTGTTTTCCGATGATCCATAC<br>GTTTGGAAATAAAACGTGTCACTTTAATAATTTTGTGCAAAA<br>ATATATTAATTTTATAGGCACCAAATTTGCTGAAAATTCTTCA<br>TTAAGGTTTACTTTAATGTAGCATTAATTTCGCAAAAATAAA<br>ATGTAATATTAATTTCGCAATATTAATATAATATTGATGTCGC<br>AAATTTTGCTTTAATATAATATGAAAACTTCAAACTTATATAT<br>CATATATTAGCTGTGTTTCCACGTACATGCGAATCAGATAGA<br>TCCTCCCAAGAACAGCTTTACATATTTTGTACTTTAAATATTT<br>TTTTTTACTTTCAAACCTTAAATATTTTGTAAATATCTTAAATA<br>TCTTCGCTTTAGGCTCCGCTGAGTAAATCCTGCCTGAGTGCCC<br>CTTTCAGAGAAGAAAACGTTCACAGACAGAATGTGTTAGCG<br>AACGTCATGAAGTAAAATATTTAAAATTGGACTCCATCAAAT<br>GACACTGTTTCACAATTCCTAAATCATTTTTCTCCCATAGGAA<br>ACTGGCTGAGCGTCGTTCTTCAAGGGGGAGAAACAACTAAA<br>CTGATATTTGACAGGAGCTTCCCGACAACAGGTGCATTTTGC<br>ATGAGGTTGGTGATCAGGACAGGTCCGAACGTTGATGGCGA<br>AACTGAAAGTGGCAATGGTGTCAGCTATCTGGATTACGGAC<br>GTTCTAAAACAAAACGTGCCTTGGATTTCTCAGGCATTCCAA<br>AGTCATTCAGGCAGGATTTTATGCCATTTCGTTATGGATTTTA<br>CGCAAGAGGCGCAACGACAAAGTACACTTTTGAGTACACCG<br>TACCTGCCGGCTCTCCACCGCAGTTCGCCGAGATAAAAGAA<br>GTATCAGCAAAAGATGGCTCTTGTCGATGAAGAAAGTTGTA<br>ACTTTATCGACTGTATATCATCTTTATAATTGCAAAT

PhdCRI11	W4VS53	3,71E-09	31,707	PF00188	15589.47	NWTPSNDTVSQFLN HFSPIGNWLSVVLQ GGETTKLIFDRSFPTT GAFCMRLVIRTGPN VDGETESGNGVSYL DYGRSKTKRALDFS GIPKSFRQDFMPFRY GFYARGATTKYTFEY TVPAGSPPQFAEIKE VSAKDGSCR*	ACAGTGGAGAAGACAGCAGGAGGTGAGAAAATTGTAATGA ATATAATGCAGTTAAGTATCAAAATTTCCTATTTCACATTTTA AGGAAGCTCAGGTGGGAATACAGATTCAAATTTCAAAGTAC GTTAAAGGAATTTTCCATTAGGTAGCGCAATGGTGAAGTGAA AAGATCCCAGCTATCACAATTAGGTGCCCCCTCCGTCTCTCT AGTATTCACAAATGAATAGTAAAGTCTTCAGTCTCATGCAGG TTTGTTTCTTATAGGATGCAATCATATTAGTGGAGATTGCCTG TGTATGTCCATCGGCGCGGGTACTTTTTTGACAGCGAGCTATT CGAGGCCTAAACATCAAAATGCTGGACCCAGGGTACATAAA AAATTTTGGGTTCCAATAGCAGATCGCATTCTTTTTACAAGA AGGGGGAGCAAAGCAACAATTACACATGAAATTCCTTTCGC ATGTATACAAGAATTGCGCAAATGTGACGTTCAACAGCACTC GATACACCGGTTAAGTGAAAACAAGAAACTGGAAGATTTTG ATGCCTACGTGTAATATAATAATCGAATGGTGGATTTTAGAA GATGGGGTAGATAGTAAAAAGTAAATATAGATATTCTCTTTG AGGTAAATAATCTTCTAAATTACTTTCTGGAGGGCAGAAATG AAATCCAGTCAGCAATTTGATTGAAATTGATTAGCGATGTAT CTCTTCTAACTGAAAATTAACATCGTTATCCATCTGTTTCCAA ACATGGAAAGAAATGCAATACATAGTACATAGTATAACAGC TTTATCTAGTTTTTTCAAGGGTCAATAGAAAATGAAACGGGG CATTGAGTCCTAGAGATGGGTTTCTTATTTATGTAAGTCATGA TAAAGATACAGATAATAAGAAATGCGCAATTATTTATACTTG TCAAGGGCCCAATATTTCGGCTGAAAAAGTTTGGCGAAAAG ACTTAATGACAACTTTTTAAAGAAAAATAGCGTTAAACATTT CCATGGAACATAAGTTCCTGTAGCCTGTATATTTTAAACCGG AAATAATTGGAAAAATTTCGTGGAATTTATATTAAATTTTTCA GGTAAATTCATATTTTAGTATCTTTAATAAAAATCGAAATATT TCAAGGTTTAAAGGTCGTATCTTCCAATTCACTTTTTGAATGT

							TGGTGATCTCCAGCAGAAATATTTTACGGATTTATATCTTATA<br>GTGCTTTTTCATGCAAGTTTTACGCAGTTTCTAAATGTGCATA<br>TTAATTAGAGATAAATTAACCATATAAGTATATAAATTGTAA<br>TTTGTAAGTAAAATCCGATGTTGTATTACAGGGAAAAAATTT<br>TCTGACATGAAGTACTTAATTTGTAGTATCTTAAGATATTCTC<br>GCGAAGTCCAATTTTCAGTTGAACTCCAGCCTGCTTAGAACG<br>CGGTCTTGCAACGTTGTGGATCGGAGTTCGACTCCCCGATAA<br>GGCTTGGATGGTTCGGTGGTTGTCTCATCATAGGGACTGAAC<br>GCCAGATGCAGGCAGCCTACTCCGGTTCAGCCTGAGTGTGAG<br>GAGACAAAATGCCTGTCCTGAATTGATAAAATCCACCTTCAT<br>TCATTGTAGTCACAGACGATCAGACTTATGTAAGCAGAGTGT<br>ACAGATCTGCCAATGTCCTGGCCGGACCAACACCTCTCATCG<br>GGGGAGCATCTCCCCATCATAGGGGCTAGCGTTGCCAGTCCA<br>TGTGCTGCCATTATAATACATTGGACAACAGTTTCGGCAGAA<br>TATACGGGCACACTGTTTGTCGCAAAGGGAGAGCTGATGTAT<br>TTAATTAAATTTGAGTCGAACGAGAAGCATGAATGTGAATTG<br>CTCCACGTTCTTAGCGTAGAAGTTCCTACGGAATACTTAATA<br>ATAATAATAATAATACCCTGAGAATCACCACTGAAATTAGTA<br>AAATATGCAATATCCTTTATTTTAGTTAGTAAAGAGTGATAGT<br>TTTTACATATGAAAAAAATGAACAATTCGAGGAAAAATGAA<br>AAAAATTATGAAGAATCGAAAAAGGTTAAAATCATACCTTA<br>TTTTGGGCGTTTAAGTGCCGAATATCCTCTTAATATTTAAGGC<br>TATCCGCTATCATTAAATCTAAACCTCTTTTGAATAGATGAA<br>GCTGACATTCGCAGAGTATTTGATAGATTGGAAAGTACCAGA<br>AAAGAGTGTCTAGAAGGATAATTATACTTATATTGTACCCAT<br>TCATTAACATCAAGCGCATCTCTTCACTCGAAAGTGAGCTAC<br>AATCTATTTTAAAGCATGAATCTTGGTTTTGCACAAAGTAAC<br>CGGGGTGTAGGGCGCCATTTGTCAGTAACTATAGGTACTGAG

							TGAAATACACGTTGAAGACACAGTTTTTGAGAAATAATTCAT<br>CCTATTATATTGCATAAATCTGGTAAATGGTTTTCAAATTTGT<br>ACGTGGGACAGCATATTTATTTTTCACTCTAAAACACAGTTCT<br>AACTGCGTATTTGTTTTCCGATGATCCATACGTTTGGAAATAA<br>AACGTGTCACTTTAATAATTTTGTGCAAAAATATATTAATTTT<br>ATAGGCACCAAATTTGCTGAAAATTCTTCATTAAGGTTTACTT<br>TAATGTAGCATTAATTTCGCAAAAATAAAATGTAATATTAAT<br>TTCGCAATATTAATATAATATTGATGTCGCAAATTTTGCTTTA<br>ATATAATATGAAAACTTCAAACTTATATATCATATATTAGCT<br>GTGTTTCCACGTACATGCGAATCAGATAGATCCTCCCAAGAA<br>CAGCTTTACATATTTTGTACTTTAAATATTTTTTTTTACTTTCA<br>AACCTTAAATATTTTGTAAATATCTTAAATATCTTCGCTTTAG<br>GCTCCGCTGAGTAAATCCTGCCTGAGTGCCCCTTTCAGAGAA<br>GAAAACGTTCACAGACAGAATGTGTTAGCGAACGTCATGAA<br>GTAAAATATTTAAAATTGGACTCCATCAAATGACACTGTTTC<br>ACAATTCCTAAATCATTTTTCTCCCATAGGAAACTGGCTGAG<br>CGTCGTTCTTCAAGGGGGAGAAACAACTAAACTGATATTTGA<br>CAGGAGCTTCCCGACAACAGGTGCATTTTGCATGAGGTTGGT<br>GATCAGGACAGGTCCGAACGTTGATGGCGAAACTGAAAGTG<br>GCAATGGTGTCAGCTATCTGGATTACGGACGTTCTAAAACAA<br>AACGTGCCTTGGATTTCTCAGGCATTCCAAAGTCATTCAGGC<br>AGGATTTTATGCCATTTCGTTATGGATTTTACGCAAGAGGCGC<br>AACGACAAAGTACACTTTTGAGTACACCGTACCTGCCGGCTC<br>TCCACCGCAGTTCGCCGAGATAAAAGAAGTATCAGCAAAAG<br>ATGGCTCTTGTCGATGAAGAAAGTTGTAACTTTATCGACTGT<br>ATATCATCTTTATAATTGCAAAT

PhdCRI12	W4VS53	3,71E-09	31,707	PF00188	15589.47	NWTPSNDTVSQFLNHFSPIGNWLSVVLQGGETTKLIFDRSFPTTGAFCMRLVIRTGPNVDGETESGNGVSYLDYGRSKTKRALDFSGIPKSFRQDFMPFRYGFYARGATTKYTFEYTVPAGSPPQFAEIKEVSAKDGSCR*	ACAGTGGAGAAGACAGCAGGAGGTGAGAAAATTGTAATGAATATAATGCAGTTAAGTATCAAAATTTCCTATTTCACATTTTAAGGAAGCTCAGGTGGGAATACAGATTCAAATTTCAAAGTACGTTAAAGGAATTTTCCATTAGGTAGCGCAATGGTGAAGTGAAAAGATCCCAGCTATCACAATTAGGTGCCCCCTCCGTCTCTCTAGTATTCACAAATGAATAGTAAAGTCTTCAGTCTCATGCAGGTTTGTTTCTTATAGGATGCAATCATATTAGTGGAGATTGCCTGTGTATGTCCATCGGCGCGGGTACTTTTTTGACAGCGAGCTATTCGAGGCCTAAACATCAAAATGCTGGACCCAGGGTACATAAAAAATTTTGGGTTCCAATAGCAGATCGCATTCTTTTTACAAGAAGGGGGAGCAAAGCAACAATTACACATGAAATTCCTTTCGCATGTATACAAGAATTGCGCAAATGTGACGTTCAACAGCACTCGATACACCGGTTAAGTGAAAACAAGAAACTGGAAGATTTTGATGCCTACGTGTAATATAATAATCGAATGGTGGATTTTAGAAGATGGGGTAGATAGTAAAAAGTAAATATAGATATTCTCTTTGAGGTAAATAATCTTCTAAATTACTTTCTGGAGGGCAGAAATGAAATCCAGTCAGCAATTTGATTGAAATTGATTAGCGATGTATCTCTTCTAACTGAAAATTAACATCGTTATCCATCTGTTTCCAAACATGGAAAGAAATGCAATACATAGTGTAACAGCTTTATCTAGCTTTTTCAAGGGTCAATAGAAAATGAAACGGGGCATTGAGTCCTAGAGATGGGTTTCTTATTTATGTAAGTCATGATAAAGATACAGATAATAAGAAATGCGCAATTATTTATACTTGTCAAGGGCCCAATATTTCGGCTGAAAAAGTTTGGCGAAAAGACTTAATGACAACTTTTTAAAGAAAAATAGCGTTAAACATTTCCATGGAACATAAGTTCCTGTAGCCTGTATATTTTAGACCGTATCTCGGGAAATAATTGGAAAAATTTCGTGGAATTTATATTAAATTTTTCAGGTAAATTCATATTTTAGTATCTTTAATAAAAATCGAAATATTTCAAGGTTTAAAGGTCGTATCTTCCAATTCACTTTTTGAATGTT

							GGTGATCTCCAGCAGAAATATTTTACGGATTTATATCTTATAG<br>TGCTTTTTCATGCAAGTTTTACGCAGTTTCTAAATGTGCATATT<br>AATTAGAGATAAATTAACCATATAAGTATATAAATTGTAATT<br>TGTAAGTAAAATCCGATGTTGTATTACAGGGAAAAAATTTTC<br>TGACATGAAGTACTTAATTTGTAGTATCTTAAGATATTCTCGC<br>GAAGTCCAATTTTCAGTTGAACTCCAGCCTGCTTAGAACGCG<br>GTCTTGCAACGTTGTGGATCGGAGTTCGACTCCCCGATAAGG<br>CTTGGATGGTTCGGTGGTTGTCTCATCATAGGGACTGAACGC<br>CAGATGCAGGCAGCCTACTCCGGTTCAGCCTGAGTGTGAGG<br>AGACAAAATGCCTGTCCTGAATTGATAAAATCCACCTTCATT<br>CATTGTAGTCACAGACGATCAGACTTATGTAAGCAGAGTGTA<br>CAGATCTGCCAATGTCCTGGCCGGACCAACACCTCTCATCGG<br>GGGAGCATCTCCCCATCATAGGGGCTAGCGTTGCCAGTCCAT<br>GTGCTGCCATTATAATACATTGGACAACAGTTTCGGCAGAAT<br>ATACGGGCACACTGTTTGTCGCAAAGGGAGAGCTGATGTATT<br>TAATTAAATTTGAGTCGAACGAGAAGCATGAATGTGAATTGC<br>TCCACGTTCTTAGCGTAGAAGTTCCTACGGAATACTTAATAA<br>TAATAATAATAATACCCTGAGAATCACCACTGAAATTAGTAA<br>AATATGCAATATCCTTTATTTTAGTTAGTAAAGAGTGATAGTT<br>TTTACATATGAAAAAAATGAACAATTCGAGGAAAAATGAAA<br>AAAATTATGAAGAATCGAAAAAGGTTAAAATCATACCTTATT<br>TTGGGCGTTTAAGTGCCGAATATCCTCTTAATATTTAAGGCTA<br>TCCGCTATCATTAAATCTAAACCTCTTTTGAATAGATGAAGCT<br>GACATTCGCAGAGTATTTGATAGATTGGAAAGTACCAGAAA<br>AGAGTGTCTAGAAGGATAATTATACTTATATTGTACCCATTC<br>ATTAACATCAAGCGCATCTCTTCACTCGAAAGTGAGCTACAA<br>TCTATTTTAAAGCATGAATCTTGGTTTTGCACAAAGTAATCGG<br>GGTGTAGGGCGCCATTTGCCAGTAACTATAGGTACTGAGTGA

							AATACACGTTGAAGACACAGTTTTTGAGAAATAATTCATCCT<br>ATTATATTGCATAAATCTGGTAAATGGTTTTTCAAATTTGAAC<br>GTGGGACAGCATATTTATTTATCACTCTAAAACACAGTTCTA<br>ACTGCGTATTTGTTTTCCGATGATCCATACGTTTGGAAATAAA<br>ACGTGTCACTTTAATAATTTTGTGCAAAAATATATTAATTTTA<br>TAGGCACCAAATTTGCTGAAAATTCTTCATTAAGGTTTACTTT<br>AATGTAGCATTAATTTCGCAAAAATAAAATGTAATATTAATT<br>TCGCAATATTAATATAATATTGATGTCGCAAATTTTGCTTTAA<br>TATAATATGAAAACTTCAAACTTATATATCATATATTAGCTGT<br>GTTTCCACGTACATGCGAATCAGATAGATCCTCCCAAGAACA<br>GCTTTACATATTTTGTACTTTAAATATTTTTTTTTACTTTCAAA<br>CCTTAAATATTTTGTAAATATCTTAAATATCTTCGCTTTAGGC<br>TCCGCTGAGTAAATCCTGCCTGAGTGCCCCTTTCAGAGAAGA<br>AAACGTTCACAGACAGAATGTGTTAGCGAACGTCATGAAGT<br>AAAATATTTAAAATTGGACTCCATCAAATGACACTGTTTCAC<br>AATTCCTAAATCATTTTTCTCCCATAGGAAACTGGCTGAGCG<br>TCGTTCTTCAAGGGGGAGAAACAACTAAACTGATATTTGACA<br>GGAGCTTCCCGACAACAGGTGCATTTTGCATGAGGTTGGTGA<br>TCAGGACAGGTCCGAACGTTGATGGCGAAACTGAAAGTGGC<br>AATGGTGTCAGCTATCTGGATTACGGACGTTCTAAAACAAAA<br>CGTGCCTTGGATTTCTCAGGCATTCCAAAGTCATTCAGGCAG<br>GATTTTATGCCATTTCGTTATGGATTTTACGCAAGAGGCGCA<br>ACGACAAAGTACACTTTTGAGTACACCGTACCTGCCGGCTCT<br>CCACCGCAGTTCGCCGAGATAAAAGAAGTATCAGCAAAAGA<br>TGGCTCTTGTCGATGAAGAAAGTTGTAACTTTATCGACTGTAT<br>ATCATCTTTATAATTGCAAAT

PhdCRI13	W4VS53	3,35E-09	31,707	PF00188	15589.47	NWTPSNDTVSQFLN<br>HFSPIGNWLSVVLQ<br>GGETTKLIFDRSFPTT<br>GAFCMRLVIRTGPN<br>VDGETESGNGVSYL<br>DYGRSKTKRALDFS<br>GIPKSFRQDFMPFRY<br>GFYARGATTKYTFEY<br>TVPAGSPPQFAEIKE<br>VSAKDGSCR*	ACAGTGGAGAAGACAGCAGGAGGTGAGAAAATTGTAATGA<br>ATATAATGCAGTTAAGTATCAAAATTTCCTATTTCACATTTTA<br>AGGAAGCTCAGGTGGGAATACAGATTCAAATTTCAAAGTAC<br>GTTAAAGGAATTTTCCATTAGGTAGCGCAATGGTGAAGTGAA<br>AAGATCCCAGCTATCACAATTAGGTGCCCCCTCCGTCTCTCT<br>AGTATTCACAAATGAATAGTAAAGTCTTCAGTCTCATGCAGG<br>TTTGTTTCTTATAGGATGCAATCATATTAGTGGAGATTGCCTG<br>TGTATGTCCATCGGCGCGGGTACTTTTTTGACAGCGAGCTATT<br>CGAGGCCTAAACATCAAAATGCTGGACCCAGGGTACATAAA<br>AAATTTTGGGTTCCAATAGCAGATCGCATTCTTTTTACAAGA<br>AGGGGGAGCAAAGCAACAATTACACATGAAATTCCTTTCGC<br>ATGTATACAAGAATTGCGCAAATGTGACGTTCAACAGCACTC<br>GATACACCGGTTAAGTGAAAACAAGAAACTGGAAGATTTTG<br>ATGCCTACGTGTAATATAATAATCGAATGGTGGATTTTAGAA<br>GATGGGGTAGATAGTAAAAAGTAAATATAGATATTCTCTTTG<br>AGGTAAATAATCTTCTAAATTACTTTCTGGAGGGCAGAAATG<br>AAATCCAGTCAGCAATTTGATTGAAATTGATTAGCGATGTAT<br>CTCTTCTAACTGAAAATTAACATCGTTATCCATCTGTTTCCAA<br>ACATGGAAAGAAATGCAATACATAGTACATAGTATAACAGC<br>TTTATCTAGTTTTTTCAAGGGTCAATAGAAAATGAAACGGGG<br>CATTGAGTCCTAGAGATGGGTTTCTTATTTATGTAAGTCATGA<br>TAAAGATACAGATAATAAGAAATGCGCAATTATTTATACTTG<br>TCAAGGGCCCAATATTTCGGCTGAAAAAGTTTGGCGAAAAG<br>ACTTAATGACAACTTTTTAAAGAAAAATAGCGTTAAACATTT<br>CCATGGAACATAAGTTCCTGTAGCCTGTATATTTTAAACCGG<br>AAATAATTGGAAAAATTTCGTGGAATTTATATTAAATTTTTCA<br>GGTAAATTCATATTTTAGTATCTTTAATAAAAATCGAAATATT<br>TCAAGGTTTAAAGGTCGTATCTTCCAATTCACTTTTTGAATGT

							TGGTGATCTCCAGCAGAAATATTTTACGGATTTATATCTTATA GTGCTTTTTCATGCAAGTTTTACGCAGTTTCTAAATGTGCATA TTAATTAGAGATAAATTAACCATATAAGTATATAAATTGTAA TTTGTAAGTAAAATCCGATGTTGTATTACAGGGAAAAAATTT TCTGACATGAAGTACTTAATTTGTAGTATCTTAAGATATTCTC GCGAAGTCCAATTTTCAGTTGAACTCCAGCCTGCTTAGAACG CGGTCTTGCAACGTTGTGGATCGGAGTTCGACTCCCCGATAA GGCTTGGATGGTTCGGTGGTTGTCTCATCATAGGGACTGAAC GCCAGATGCAGGCAGCCTACTCCGGTTCAGCCTGAGTGTGAG GAGACAAAATGCCTGTCCTGAATTGATAAAATCCACCTTCAT TCATTGTAGTCACAGACGATCAGACTTATGTAAGCAGAGTGT ACAGATCTGCCAATGTCCTGGCCGGACCAACACCTCTCATCG GGGGAGCATCTCCCCATCATAGGGGCTAGCGTTGCCAGTCCA TGTGCTGCCATTATAATACATTGGACAACAGTTTCGGCAGAA TATACGGGCACACTGTTTGTCGCAAAGGGAGAGCTGATGTAT TTAATTAAATTTGAGTCGAACGAGAAGCATGAATGTGAATTG CTCCACGTTCTTAGCGTAGAAGTTCCTACGGAATACTTAATA ATAATAATAATAATACCCTGAGAATCACCACTGAAATTAGTA AAATATGCAATATCCTTTATTTTAGTTAGTAAAGAGTGATAGT TTTTACATATGAAAAAAATGAACAATTCGAGGAAAAATGAA AAAAATTATGAAGAATCGAAAAAGGTTAAAATCATACCTTA TTTTGGGCGTTTAAGTGCCGAATATCCTCTTAATATTTAAGGC TATCCGCTATCATTAAATCTAAACCTCTTTTGAATAGATGAA GCTGACATTCGCAGAGTATTTGATAGATTGGAAAGTACCAGA AAAGAGTGTCTAGAAGGATAATTATACTTATATTGTACCCAT TCATTAACATCAAGCGCATCTCTTCACTCGAAAGTGAGCTAC AATCTATTTTAAAGCATGAATCTTGGTTTTGCACAAAGTAATC GGGGTGTAGGGCGCCATTTGCCAGTAACTATAGGTACTGAGT

							GAAATACACGTTGAAGACACAGTTTTTGAGAAATAATTCATC<br>CTATTATATTGCATAAATCTGGTAAATGGTTTTTCAAATTTGA<br>ACGTGGGACAGCATATTTATTTATCACTCTAAAACACAGTTC<br>TAACTGCGTATTTGTTTTCCGATGATCCATACGTTTGGAAATA<br>AAACGTGTCACTTTAATAATTTTGTGCAAAAATATATTAATTT<br>TATAGGCACCAAATTTGCTGAAAATTCTTCATTAAGGTTTACT<br>TTAATGTAGCATTAATTTCGCAAAAATAAAATGTAATATTAA<br>TTTCGCAATATTAATATAATATTGATGTCGCAAATTTTGCTTT<br>AATATAATATGAAAACTTCAAACTTATATATCATATATTAGC<br>TGTGTTTCCACGTACATGCGAATCAGATAGATCCTCCCAAGA<br>ACAGCTTTACATATTTTGTACTTTAAATATTTTTTTTTACTTTC<br>AAACCTTAAATATTTTGTAAATATCTTAAATATCTTCGCTTTA<br>GGCTCCGCTGAGTAAATCCTGCCTGAGTGCCCCTTTCAGAGA<br>AGAAAACGTTCACAGACAGAATGTGTTAGCGAACGTCATGA<br>AGTAAAATATTTAAAATTGGACTCCATCAAATGACACTGTTT<br>CACAATTCCTAAATCATTTTTCTCCCATAGGAAACTGGCTGA<br>GCGTCGTTCTTCAAGGGGGAGAAACAACTAAACTGATATTTG<br>ACAGGAGCTTCCCGACAACAGGTGCATTTTGCATGAGGTTGG<br>TGATCAGGACAGGTCCGAACGTTGATGGCGAAACTGAAAGT<br>GGCAATGGTGTCAGCTATCTGGATTACGGACGTTCTAAAACA<br>AAACGTGCCTTGGATTTCTCAGGCATTCCAAAGTCATTCAGG<br>CAGGATTTTATGCCATTTCGTTATGGATTTTACGCAAGAGGCG<br>CAACGACAAAGTACACTTTTGAGTACACCGTACCTGCCGGCT<br>CTCCACCGCAGTTCGCCGAGATAAAAGAAGTATCAGCAAAA<br>GATGGCTCTTGTCGATGAAGAAAGTTGTAACTTTATCGACTG<br>TATATCATCTTTATAATTGCAAATAAATT

PhdCRI14	W4VS53	2,53E-99	42,222	PF00188	46845.17<br>**44572.26**	MLFRIVSLPLLLLAV	CAAAATCTCGTGACTTGCAGAGCTGAAGAGAACAAGAGGAA
						TLVAHA**QQCTSLYK**	ACAACATGTTGTTCCGAATTGTTTCTCTACCACTGCTGCTGTT
						**RYSSEHTSCKPPNK**	GGCCGTGACGTTGGTTGCACATGCTCAACAGTGCACAAGCCT
						**SCNILKRGVSDEDK**	CTACAAGCGGTACAGTTCTGAACATACATCCTGCAAGCCACC
						**KLILKLHNDYRSRI**	CAACAAATCCTGTAACATTTTGAAACGAGGTGTCTCCGACGA
						**ATGQETNAGGMP**	AGATAAAAAACTTATCTTGAAGCTGCACAATGATTACAGGA
						**QAADMLQMIWDD**	GCAGGATAGCCACCGGCCAGGAGACCAATGCAGGCGGCAT
						**ELAAVAQKLAETCK**	GCCACAAGCAGCGGACATGCTCCAAATGATTTGGGACGATG
						**YGHDCNNCRKVKA**	AACTCGCTGCAGTGGCGCAAAAGCTTGCAGAAACTTGCAAA
						**FSVGQNIGRSYGFP**	TACGGTCATGACTGTAACAACTGTCGGAAAGTCAAGGCTTTC
						**KAPDAANWRGFIK**	TCAGTCGGACAAAATATCGGCCGATCATATGGATTTCCCAAA
						**SLYDEVSLFDKSFIK**	GCACCAGACGCTGCTAACTGGAGGGGGTTTATTAAAAGTCTG
						**PYVPSPTSHDKAYG**	TACGACGAAGTTTCGCTCTTTGACAAGAGTTTCATCAAACCA
						**HFTQVTWASSWKV**	TATGTTCCTTCACCAACCAGTCACGATAAAGCGTATGGACAT
						**GCGWTVYKQGNTL**	TTTACTCAAGTAACGTGGGCATCCTCTTGGAAAGTTGGTTGC
						**FSYTVCNYGPAGN**	GGTTGGACTGTTTACAAGCAAGGCAATACACTGTTTAGTTAT
						**MREGEMYKAGPTC**	ACTGTGTGCAACTATGGACCAGCTGGAAATATGCGGGAAGG
						**SACPKNSCCGNSC**	AGAGATGTACAAAGCTGGTCCGACTTGCTCCGCGTGTCCGAA
						**KKHGLTADYPGLC**	GAATTCTTGTTGCGGAAATTCGTGTAAGAAGCATGGTCTGAC
						**KVIDTNTPPEGPGP**	TGCCGATTATCCGGGACTGTGCAAGGTTATAGATACTAACAC
						**HGNDYIFYCDFNN**	TCCACCCGAAGGACCTGGACCCCATGGAAATGATTACATCTT
						**QPDCAVTYEGKTW**	TTATTGTGACTTCAACAATCAGCCGGATTGTGCCGTGACGTA
						**TVEKTAGGNWLSV**	TGAGGGTAAAACTTGGACAGTGGAGAAGACAGCAGGAGGA
						**VLKGGERTKVTFD**	AACTGGCTAAGCGTCGTTCTGAAAGGTGGAGAAAGAACTAA
						**RSFPTTGAFCMRVV**	AGTGACATTTGACAGGAGCTTCCCAACAACAGGTGCATTCTG
						**LRKSPNVDGDTES**	CATGAGGGTGGTGCTCAGGAAAAGCCCGAACGTCGATGGCG
						**GNGVSFLDYGRSK**	ATACTGAAAGTGGCAATGGTGTCAGCTTCCTGGACTATGGCC
						**AKFGVNFSGFPKSL**	GTTCTAAAGCAAAATTTGGCGTGAATTTCTCAGGTTTTCCAA

						**RQNFTPFRYGFNARGATIKYIFEYTVPAGSPPQFVEIKEVSARDGSCR***	AGTCACTCAGGCAGAATTTTACGCCATTTCGTTATGGATTTAACGCAAGAGGCGCAACGATAAAGTACATTTTTGAGTACACCGTACCTGCCGGCTCTCCACCGCAGTTCGTCGAGATAAAAGAAGTATCAGCAAGAGATGGCTCTTGTCGGTGAAGAAAATTGTAATTTTATCGACTGGATATCATCTTTATAATTGCAAATAAATTTAATTGTATTTATATCTAGCACTTAATATTATAATCAGCATTGAGCACAAATGGCTATCACCATTCTAGCTCTTTTT

PhdCRI15	W4VS53	3,51E-103	42,118	PF00188	47069.06<br>**44796.15**	MLFRIVSLPLLLLAV<br>TLVAHA**QQCPDLY**<br>**RRYTSEHTYCKPPN**<br>**QSCNILKRGVSDED**<br>**KELILKLHNDYRNR**<br>**VATGQETNAGGMP**<br>**QSADMLQMVWDD**<br>**DIAAVAQKHAEDC**<br>**NFRHDCDNCRKVK**<br>**AFSVGQNIGQSSVP**<br>**PKAPNSANWRGFI**<br>**KNLYSEVTRFDKNF**<br>**IKPYVPGPPSHDKA**<br>**YGHFTQITWASSW**<br>**KIGCGWTVYKQGS**<br>**KLKSYTVCNYGPPG**<br>**NMRNGEMYKAGP**<br>**TCSACPKNSCCGDS**<br>**CKDHGLTADYPGL**<br>**CKVIDTNTPPEGPG**<br>**PHGNDYIFYCDFNN**<br>**QPDCAVTYEGKTW**<br>**TVEKTAGGNWLSV**<br>**VLKGGERTKVTFD**<br>**RSFPTTGAFCMRVV**<br>**LRKSPNVDGDTES**<br>**GNGVSFLDYGRSK**<br>**AKFGVNFSGFPKSL**	CAAAATCTCGTGACTTGCAGAGCTGAAGAGAACAAGAGGAA<br>ACAACATGTTGTTCCGAATTGTTTCTCTACCACTGCTGCTGTT<br>GGCCGTGACGTTGGTTGCTCATGCTCAACAGTGCCCAGATCT<br>CTACAGGCGGTATACTTCTGAACATACATACTGCAAGCCACC<br>TAACCAATCCTGTAACATTTTGAAACGAGGTGTCTCCGACGA<br>AGATAAAGAACTTATCTTGAAGCTACACAATGATTATAGAA<br>ACAGGGTAGCCACCGGCCAGGAGACCAACGCAGGCGGCAT<br>GCCACAATCAGCGGACATGCTCCAAATGGTTTGGGACGATG<br>ATATCGCTGCCGTGGCGCAGAAGCATGCAGAGGATTGCAAT<br>TTCCGTCATGACTGTGACAACTGTCGGAAAGTCAAGGCCTTC<br>TCAGTCGGACAAAACATCGGCCAATCTTCTGTACCTCCCAAA<br>GCACCAAACTCTGCTAACTGGAGGGGGTTCATTAAAAACCT<br>GTACAGCGAAGTTACACGCTTTGACAAGAATTTCATCAAACC<br>ATATGTTCCCGGACCACCCAGTCACGATAAAGCGTATGGAC<br>ATTTTACTCAAATAACGTGGGCATCCTCTTGGAAAATTGGTT<br>GCGGTTGGACTGTTTACAAGCAAGGCAGTAAACTGAAAAGT<br>TATACCGTGTGCAACTATGGACCACCTGGAAATATGAGGAA<br>CGGAGAGATGTACAAAGCTGGTCCGACTTGTTCTGCGTGTCC<br>GAAGAATTCTTGCTGCGGAGATTCGTGTAAGGATCATGGCCT<br>GACTGCCGATTATCCGGGGCTGTGCAAGGTTATAGATACTAA<br>CACTCCACCCGAAGGACCTGGACCTCATGGAAATGATTACAT<br>CTTTTATTGTGACTTCAACAATCAGCCGGATTGTGCCGTGACG<br>TATGAGGGTAAAACTTGGACAGTGGAAAATACAGCAGGAGG<br>AAACTGGCTGAGCGTCGTTCTTCAAGGGGGAGAAACAACTA<br>AACTGATATTTGACAGGAGCTTCCCGACAACAGGTGCATTTT<br>GCATGAGGTTGGTGATCAGGACAGGTCCGAACGTTGATGGC<br>GAAACTGAAAGTGGCAATGGTGTCAGCTATCTGGATTACGG<br>ACGTTCTAAAACAAAACGTGCCTTGGATTTCTCAGGCATTCC

						**RQNFTPFRYGFNARGATIKYIFEYTVPAGSPPQFVEIKEVSARDGSCR***	AAAGTCATTCAGGCAGGATTTTATGCCATTTCGTTATGGATTTTACGCAAGAGGCGCAACGACAAAGTACACTTTTGAGTACACCGTACCTGCCGGCTCTCCACCGCAGTTCGCCGAGATAAAAGAAGTATCAGCAAAAGATGGCTCTTGTCGATGAAGAAAGTTGTAACTTTATCGACTGTATATCATCTTTATAATTGCAAATAAATTTAATTGTATTTATATCTAGCACTTAATATTATAATCAGCATTGAGCACAAATGGCTATCACCATTTTAGCTTTTTTTTCTTTTCAATATAAATAATGAGCTATATTACTTAATTGCATTTATTTTGAAAAGAAAAA

PhdCRI16	W4VS53	1,78E-102	41,769	PF00188	47057.17<br><br>**44784.26**	MLFRIVSLPLLLLAV<br>TLVAHA**QQCPDLY**<br>**RRYTSEHTYCKPPN**<br>**QSCNILKRGVSDED**<br>**KELILKLHNDYRNR**<br>**VATGQETNAGGMP**<br>**QSADMLQMVWDD**<br>**DIAAVAQKHAEDC**<br>**NFRHDCDNCRKVK**<br>**AFSVGQNIGQSSVP**<br>**PKAPNSANWRGFI**<br>**KNLYSEVTRFDKNF**<br>**IKPYVPGPPSHDKA**<br>**YGHFTQITWASSW**<br>**KIGCGWTVYKQGS**<br>**KLKSYTVCNYGPPG**<br>**NMRNGEMYKAGP**<br>**TCSACPKNSCCGDS**<br>**CKDHGLTADYPGL**<br>**CKVIDTNTPPEGPG**<br>**PHGNDYIFYCDFNN**<br>**QPDCAVTYEGKTW**<br>**TVEKTAGGNWLSV**<br>**VLKGGERTKVTFD**<br>**RSFPTTGAFCMRVV**<br>**LRKSPNVDGDTES**<br>**GNGVSFLDYGRSK**<br>**AKFGVNFSGFPKSL**	CAAAATCTCGTGACTTGCAGAGCTGAAGAGAACAAGAGGAA<br>ACAACATGTTGTTCCGAATTGTTTCTCTACCACTGCTGCTGTT<br>GGCCGTGACGTTGGTTGCTCATGCTCAACAGTGCCCAGATCT<br>CTACAGGCGGTATACTTCTGAACATACATACTGCAAGCCACC<br>TAACCAATCCTGTAACATTTTGAAACGAGGTGTCTCCGACGA<br>AGATAAAGAACTTATCTTGAAGCTACACAATGATTATAGAA<br>ACAGGGTAGCCACCGGCCAGGAGACCAACGCAGGCGGCAT<br>GCCACAATCAGCGGACATGCTCCAAATGGTTTGGGACGATG<br>ATATCGCTGCCGTGGCGCAGAAGCATGCAGAGGATTGCAAT<br>TTCCGTCATGACTGTGACAACTGTCGGAAAGTCAAGGCCTTC<br>TCAGTCGGACAAAACATCGGCCAATCTTCTGTACCTCCCAAA<br>GCACCAAACTCTGCTAACTGGAGGGGGTTCATTAAAAACCT<br>GTACAGCGAAGTTACACGCTTTGACAAGAATTTCATCAAACC<br>ATATGTTCCCGGACCACCCAGTCACGATAAAGCGTATGGAC<br>ATTTTACTCAAATAACGTGGGCATCCTCTTGGAAAATTGGTT<br>GCGGTTGGACTGTTTACAAGCAAGGCAGTAAACTGAAAAGT<br>TATACCGTGTGCAACTATGGACCACCTGGAAATATGAGGAA<br>CGGAGAGATGTACAAAGCTGGTCCGACTTGTTCTGCGTGTCC<br>GAAGAATTCTTGCTGCGGAGATTCGTGTAAGGATCATGGCCT<br>GACTGCCGATTATCCGGGGCTGTGCAAGGTTATAGATACTAA<br>CACTCCACCCGAAGGACCTGGACCTCATGGAAATGATTACAT<br>CTTTTATTGTGACTTCAACAATCAGCCGGATTGTGCCGTGACG<br>TATGAGGGAAAAACTTGGACAGTGGAGAAGACAGCAGGAG<br>GAAACTGGCTAAGCGTCGTTCTGAAAGGTGGAGAAAGAACT<br>AAAGTGACATTTGACAGGAGCTTCCCAACAACAGGTGCATT<br>CTGCATGAGGGTGGTGCTCAGGAAAAGCCCGAACGTCGATG<br>GCGATACTGAAAGTGGCAATGGTGTCAGCTTCCTGGACTATG<br>GCCGTTCTAAAGCAAAATTTGGCGTGAATTTCTCAGGTTTTCC

						**RQNFTPFRYGFNARGATIKYIFEYTVPAGSPPQFVEIKEVSARDGSCR***	AAAGTCACTCAGGCAGAATTTTACGCCATTTCGTTATGGATTTAACGCAAGAGGCGCAACGATAAAGTACATTTTTGAGTACACCGTACCTGCCGGCTCTCCACCGCAGTTCGTCGAGATAAAAGAAGTATCAGCAAGAGATGGCTCTTGTCGGTGAAGAAAATTGTAATTTTATCGACTGGATATCATCTTTATAATTGCAAATAAATTTAATTGTATTTATATCTAGCACTTAATATTATAATCAGCATTGAGCACAAATGGCTATCACCATTCTAGCTCTTTTT

PhdCRI17	W4VS53	2,88E-99	41,975	PF00188	46910.01<br>**44637.11**	MLFRIVSLPLLLLAV<br>TLVAHA**QQCTSLYK**<br>**RYSSEHTSCKPPNK**<br>**SCDILKRGVSDEDK**<br>**ELILKLHNDYRSRIA**<br>**TGQETNAGGMPQ**<br>**AANMLQMVWDDE**<br>**LAAVAQKLAETCK**<br>**YGHDCNNCRKVKA**<br>**FSVGQNIGQSYGFP**<br>**NAPDAADWSGFIT**<br>**DLYDEVSRFNKKFI**<br>**NPYVPGPTSHDKA**<br>**YGHFTQVTWASSW**<br>**KVGCGWTVYKQG**<br>**NTLFSYTVCNYGEA**<br>**GNMRDGEMYKAG**<br>**QTCSACPKNSCCG**<br>**DSCKKHGLTADYP**<br>**GLCKVIDAKTSPEG**<br>**PGPHGNDYIFYCDF**<br>**NNQPDCALTYEGK**<br>**SWTVEKTAGGNWL**<br>**SIVLQGGETTKVTF**<br>**DKRFPTKGDFCLRL**<br>**VIRKSPNVDGDTES**<br>**GNGVSYLDYGRSK**<br>**VQFALKFAGFPKSL**	CAAAATCTCGTGACTTGCAGAGCTGAAGAGAACAAGAGGAA<br>ACAACATGTTGTTCCGAATTGTTTCTCTACCACTGCTGCTGTT<br>GGCCGTGACGTTGGTTGCACATGCTCAACAGTGCACAAGCCT<br>CTACAAGCGGTACAGTTCTGAACATACATCCTGCAAGCCACC<br>CAACAAATCCTGTGACATTTTGAAACGAGGTGTCTCCGACGA<br>AGATAAAGAACTTATCTTGAAACTGCACAATGATTACAGGA<br>GCAGGATAGCCACCGGCCAGGAGACCAATGCAGGCGGCAT<br>GCCACAAGCAGCGAACATGCTCCAAATGGTTTGGGACGATG<br>AACTTGCTGCCGTGGCGCAGAAGCTTGCAGAAACTTGCAAAT<br>ACGGTCATGACTGTAACAACTGTCGGAAAGTCAAGGCTTTCT<br>CAGTCGGACAAAATATCGGCCAATCTTATGGATTTCCCAACG<br>CACCAGACGCTGCTGACTGGAGCGGGTTTATTACAGATCTGT<br>ACGACGAAGTTTCTCGCTTTAACAAGAAATTCATCAACCCAT<br>ATGTTCCCGGACCAACCAGTCACGATAAAGCGTATGGACATT<br>TTACTCAAGTAACGTGGGCATCCTCTTGGAAAGTTGGTTGCG<br>GTTGGACTGTTTACAAGCAAGGCAATACACTGTTTAGTTATA<br>CTGTGTGCAACTATGGAGAAGCTGGAAATATGAGGGATGGA<br>GAGATGTACAAAGCTGGTCAGACATGTTCCGCGTGTCCCAAG<br>AATTCTTGCTGCGGAGATTCGTGTAAGAAGCATGGCCTGACT<br>GCCGATTATCCGGGACTGTGCAAGGTTATAGACGCTAAAACT<br>TCGCCCGAAGGACCTGGACCCCATGGAAATGATTACATCTTT<br>TATTGTGACTTCAACAATCAGCCGGATTGTGCCTTGACCTATG<br>AGGGGAAATCTTGGACAGTGGAAAAGACAGCAGGAGGAAA<br>TTGGCTGAGCATCGTTCTGCAGGGTGGAGAAACAACTAAAG<br>TGACATTTGACAAGAGATTCCCGACAAAAGGTGACTTCTGCC<br>TGAGGTTGGTAATCAGGAAAAGTCCGAACGTCGACGGCGAT<br>ACTGAAAGTGGCAATGGTGTCAGCTACCTGGACTATGGTCGT<br>TCTAAAGTACAATTTGCCTTGAAATTCGCAGGCTTTCCAAAG

						**RQNFTPFRYGFNARGAAIKYIFEYTVPAGSPPQFVEMKEVSAKDGNCVG***	TCACTCAGGCAGAATTTTACGCCATTTCGTTATGGATTTAACGCAAGAGGCGCAGCAATAAAGTACATTTTTGAGTACACCGTACCTGCCGGCTCCCCACCGCAGTTCGTCGAGATGAAAGAAGTATCAGCAAAAGACGGCAACTGTGTTGGGTGAAGATAACTATGCTTTATCGACAGGTTTCATCTTTATAATTGCAAATAAATCGAATTGTATTTATGTCTGGCACTTGTTACTATAATCAACATAGATCACAAATGGCTGTTGCCAGTCCAGTTTTTTTTCCTTTTCAAAATAAATGCAAGTAATATAGCTCATTATATATATTAAATGAATTCACTTTATACGTTAATAAATTAATTATAAAACT

PhdCRI18	W4VS53	4,16E-36	57,000	PF00188	14607.61<br>**12334.70**	MLFRIVSLPLLLLAVTLVAHA**QQCPDLYRRYTSEHTYCKPPNQSCNILKRGVSDEDKELILKLHNDYRNRVATGQETNAGGMPQSADMLQMVWDDDIAAVAQKHAEDCNFRHDCDNCRKVSKYRRNN***	CAAAATCTCGTGACTTGCAGAGCTGAAGAGAACAAGAGGAAACAACATGTTGTTCCGAATTGTTTCTCTACCACTGCTGCTGTTGGCCGTGACGTTGGTTGCTCATGCTCAACAGTGCCCAGATCTCTACAGGCGGTATACTTCTGAACATACATACTGCAAGCCACCTAACCAATCCTGTAACATTTTGAAACGAGGTGTCTCCGACGAAGATAAAGAACTTATCTTGAAGCTACACAATGATTATAGAAACAGGGTAGCCACCGGCCAGGAGACCAACGCAGGCGGCATGCCACAATCAGCGGACATGCTCCAAATGGTTTGGGACGATGATATCGCTGCCGTGGCGCAGAAGCATGCAGAGGATTGCAATTTCCGTCATGACTGTGACAACTGTCGGAAAGTCAGTAAGTATCGAAGAAATAATTGAACATCTTTTTTGGGGGGCCTTTGACTTTGAGAAATAAGAATTAATGCAGTAGGAAAAAAAGTAAAATATCTTTGAACATATGATATTTGTATACAGATTCAAATATCACATGTTCAAAGATATTACTGTAGAATAATAACTGTAATAATAATTTTGAAGTGTTATGTTATTATGGATATGTGTTATATATCAACACACA

PhdCRI19	W4VS53	3,70E-09	31,707	PF00188	15589,47	NWTPSNDTVSQFLN<br>HFSPIGNWLSVVLQ<br>GGETTKLIFDRSFPTT<br>GAFCMRLVIRTGPN<br>VDGETESGNGVSYL<br>DYGRSKTKRALDFS<br>GIPKSFRQDFMPFRY<br>GFYARGATTKYTFEY<br>TVPAGSPPQFAEIKE<br>VSAKDGSCR*	ACAGTGGAGAAGACAGCAGGAGGTGAGAAAATTGTAATGA<br>ATATAATGCAGTTAAGTATCAAAATTTCCTATTTCACATTTTA<br>AGGAAGCTCAGGTGGGAATACAGATTCAAATTTCAAAGTAC<br>GTTAAAGGAATTTTCCATTAGGTAGCGCAATGGTGAAGTGAA<br>AAGATCCCAGCTATCACAATTAGGTGCCCCCTCCGTCTCTCT<br>AGTATTCACAAATGAATAGTAAAGTCTTCAGTCTCATGCAGG<br>TTTGTTTCTTATAGGATGCAATCATATTAGTGGAGATTGCCTG<br>TGTATGTCCATCGGCGCGGGTACTTTTTTGACAGCGAGCTATT<br>CGAGGCCTAAACATCAAAATGCTGGACCCAGGGTACATAAA<br>AAATTTTGGGTTCCAATAGCAGATCGCATTCTTTTTACAAGA<br>AGGGGGAGCAAAGCAACAATTACACATGAAATTCCTTTCGC<br>ATGTATACAAGAATTGCGCAAATGTGACGTTCAACAGCACTC<br>GATACACCGGTTAAGTGAAAACAAGAAACTGGAAGATTTTG<br>ATGCCTACGTGTAATATAATAATCGAATGGTGGATTTTAGAA<br>GATGGGGTAGATAGTAAAAAGTAAATATAGATATTCTCTTTG<br>AGGTAAATAATCTTCTAAATTACTTTCTGGAGGGCAGAAATG<br>AAATCCAGTCAGCAATTTGATTGAAATTGATTAGCGATGTAT<br>CTCTTCTAACTGAAAATTAACATCGTTATCCATCTGTTTCCAA<br>ACATGGAAAGAAATGCAATACATAGTGTAACAGCTTTATCTA<br>GCTTTTTCAAGGGTCAATAGAAAATGAAACGGGGCATTGAGT<br>CCTAGAGATGGGTTTCTTATTTATGTAAGTCATGATAAAGAT<br>ACAGATAATAAGAAATGCGCAATTATTTATACTTGTCAAGGG<br>CCCAATATTTCGGCTGAAAAAGTTTGGCGAAAAGACTTAATG<br>ACAACTTTTTAAAGAAAAATAGCGTTAAACATTTCCATGGAA<br>CATAAGTTCCTGTAGCCTGTATATTTTAAACCGGAAATAATT<br>GGAAAAATTTCGTGGAATTTATATTAAATTTTTCAGGTAAATT<br>CATATTTTAGTATCTTTAATAAAAATCGAAATATTTCAAGGTT<br>TAAAGGTCGTATCTTCCAATTCACTTTTTGAATGTTGGTGATC

							TCCAGCAGAAATATTTTACGGATTTATATCTTATAGTGCTTTT<br>TCATGCAAGTTTTACGCAGTTTCCTAAATGCGCATATTAATTA<br>GAGATAAACTAATCATGTAAGTATGTAAATAGTAATTTGTAA<br>GTAAAATCCGATGTTGTATTACAGGGAAAAAATTTTCTGACA<br>TGAAGTACTTAATTTGTAGTATCTTAAGATATTCTCGCGAAGT<br>CCAATTTTCAGTTGAACTCCAGCCTGCTTAGAACGCGGTCTT<br>GCAACGTTGTGGATCGGAGTTCGACTCCCCGATAAGGCTTGG<br>ATGGTTCGGTGGTTGTCTCATCATAGGGACTGAACGCCAGAT<br>GCAGGCAGCCTACTCCGGTTCAGCCTGAGTGTGAGGAGACA<br>AAATGCCTGTCCTGAATTGATAAAATCCACCTTCATTCATTGT<br>AGTCACAGACGATCAGACTTATGTAAGCAGAGTGTACAGAT<br>CTGCCAATGTCCTGGCCGGACCAACACCTCTCATCGGGGGAG<br>CATCTCCCCATCATAGGGGCTAGCGTTGCCAGTCCATGTGCT<br>GCCATTATAATACATTGGACAACAGTTTCGGCAGAATATACG<br>GGCACACTGTTTGTCGCAAAGGGAGAGCTGATGTATTTAATT<br>AAATTTGAGTCGAACGAGAAGCATGAATGTGAATTGCTCCA<br>CGTTCTTAGCGTAGAAGTTCCTACGGAATACTTAATAATAAT<br>AATAATAATACCCTGAGAATCACCACTGAAATTAGTAAAAT<br>ATGCAATATCCTTTATTTTAGTTAGTAAAGAGTGATAGTTTTT<br>ACATATGAAAAAAATGAACAATTCGAGGAAAAATGAAAAA<br>AATTATGAAGAATCGAAAAAGGTTAAAATCATACCTTATTTT<br>GGGCGTTTAAGTGCCGAATATCCTCTTAATATTTAAGGCTATC<br>CGCTATCATTAAATCTAAACCTCTTTTGAATAGATGAAGCTG<br>ACATTCGCAGAGTATTTGATAGATTGGAAAGTACCAGAAAA<br>GAGTGTCTAGAAGGATAATTATACTTATATTGTACCCATTCAT<br>TAACATCAAGCGCATCTCTTCACTCGAAAGTGAGCTACAATC<br>TATTTTAAAGCATGAATCTTGGTTTTGCACAAAGTAATCGGG<br>GTGTAGGGCGCCATTTGCCAGTAACTATAGGTACTGAGTGAA

							ATACACGTTGAAGACACAGTTTTTGAGAAATAATTCATCCTATTATATTGCATAAATCTGGTAAATGGTTTTTCAAATTTGAACGTGGGACAGCATATTTATTTATCACTCTAAAACACAGTTCTAACTGCGTATTTGTTTTCCGATGATCCATACGTTTGGAAATAAAACGTGTCACTTTAATAATTTTGTGCAAAAATATATTAATTTTATAGGCACCAAATTTGCTGAAAATTCTTCATTAAGGTTTACTTTAATGTAGCATTAATTTCGCAAAAATAAAATGTAATATTAATTTCGCAATATTAATATAATATTGATGTCGCAAATTTTGCTTTAATATAATATGAAAACTTCAAACTTATATATCATATATTAGCTGTGTTTCCACGTACATGCGAATCAGATAGATCCTCCCAAGAACAGCTTTACATATTTTGTACTTTAAATATTTTTTTTTACTTTCAAACCTTAAATATTTTGTAAATATCTTAAATATCTTCGCTTTAGGCTCCGCTGAGTAAATCCTGCCTGAGTGCCCCTTTCAGAGAAGAAAACGTTCACAGACAGAATGTGTTAGCGAACGTCATGAAGTAAAATATTTAAAATTGGACTCCATCAAATGACACTGTTTCACAATTCCTAAATCATTTTTCTCCCATAGGAAACTGGCTGAGCGTCGTTCTTCAAGGGGGAGAAACAACTAAACTGATATTTGACAGGAGCTTCCCGACAACAGGTGCATTTTGCATGAGGTTGGTGATCAGGACAGGTCCGAACGTTGATGGCGAAACTGAAAGTGGCAATGGTGTCAGCTATCTGGATTACGGACGTTCTAAAACAAAACGTGCCTTGGATTTCTCAGGCATTCCAAAGTCATTCAGGCAGGATTTTATGCCATTTCGTTATGGATTTTACGCAAGAGGCGCAACGACAAAGTACACTTTTGAGTACACCGTACCTGCCGGCTCTCCACCGCAGTTCGCCGAGATAAAAGAAGTATCAGCAAAAGATGGCTCTTGTCGATGAAGAAAGTTGTAACTTTATCGACTGTATATCATCTTTATAATTGCAAAT

PhdCRI20	A9QQ26	4,68E-142	63,787	PF00188	49515.77<br><br>**47085.76**	MEIRNILLMLTAFVS<br>AIATIRA**GLCPDKY**<br>**KRYSLQHSFCLPTC**<br>**CSCAFYKKDVSDW**<br>**LKNYILYQHNNLRS**<br>**KVAGGKSYGVDH**<br>**VPRATNMLEMVW**<br>**DDELAAIAQKWVY**<br>**SCKRSHDCDLCRM**<br>**VDRFAVGQSIVEFE**<br>**GSIMEAQDYEAEFF**<br>**THFMKVKYLTKNV**<br>**VANYTESSGKSTIS**<br>**QLLWAKTWRIGCG**<br>**YLYFRHPNGRKYTN**<br>**LICNYGPRGNVEGE**<br>**EIYKGGDICSACPA**<br>**NSCCGDGCKKYGI**<br>**KAKYHGLCKVIDE**<br>**NLPPEGRVPHKKTG**<br>**KEIFYCGFNGEPDC**<br>**SYTVDGVDRWISN**<br>**VTTGGTWISTYLG**<br>**MGGYTTLKFGEPIK**<br>**SKSGKMCLTVVTR**<br>**SGPMVADQAYNYQ**<br>**LEGELSREGRYQSYI**<br>**GFPSPDEKVKHFLH**	TGAAGCCTCTTCACAGCAGAGTCCAGCCGATAACGTCGCAG<br>GAGATGTGGCAATTCCAAAAGTGGAATCTCTGTAATATTTCA<br>CCGGCGACAGATTTGTAAAATTTGTACTGCATTCTGTGGGAC<br>ATACAATGTCTCCAAAGTTGACCGAATTTAACAACGGAACAT<br>CCGTAAGCGGTAAAATTTTACAGAAATACCGCTTTCGTAATT<br>GCCACATCTCCGACGTAATTATCGGCTAGACTCTGCCGAAGA<br>GGCCCCAATAAGGCAGAAACCAGATGAGTTGCTGTTAACTA<br>AGCTGGAAATACATGTATATTTGCATTCCTAGTCTTCTCCCCT<br>TAGAGGCGTTTCTTATATATTTATCATCATCTCCCTCCTAGGA<br>GATGATGAAAAATTTAAGATGATTATAATCAAAGTCTTGGAT<br>CAATGCGGGGAATCTATTCTCAGTTATTAAAATAATAAATTA<br>TAAGCATATTTTGAATGTTCCAAGCGAATTTCCTTCCTGTATA<br>GTGCATAAGCTTTTATTCATAAGGTACATTCTGTATTATAGTA<br>TCAAACTTGGCATGTATCGTGAAGTTTCATTTTACGTATAAGG<br>AGAACCAAAAAAAAATTCATGAAAAAATAGCCCTTTTCTCC<br>ATTTCAGCTATCTTTATACTACCAGCGCCATATCAAATTTTGT<br>GCAAACTATTAAATATTTACTACATGTATTGTTGCTAAGTGTT<br>ATTCAGTATTTTAAATTATGATTGTGAGAGTTTCGGTTTAATA<br>CAAATGCTCGTTGAGCATTTCGACTTATAAAAATCGTAAAAT<br>TTATGGAGTTGAGAAGATTCAATTTAGAACGTATAAATCAGT<br>TTGAATAATTGATTTCATTCCTAAGATATAACGTACACATGA<br>ACACTGGTAACTATGCATATATACGTACAATATAAGTAATTT<br>TTACTTAGTTAACTTACGTATTTAACATACTAGATGTGCCGAG<br>AAGATTAACTCTCTTGAATGTCCACTGTACCATCAAATATAA<br>TTAAATATGTTTAAAATACCTTGTATTCAATGCAAAAGAAGG<br>CTTATACAGTAATTTAAGCTCCAATATAACTTCGCAAAATGC<br>TTTTATTGGGAACATTTTGTATAACAAATATAGAAAGTTTAA<br>AAAATATAGAAACTTTGGAATTCTGTTCCACTTTGACGTTCA

						**KDYFQPSEFPKDKDVKLSLKFSVPYQAAKQYIEIKKIVVEEKDCPKVTAVEGYSFIVD***	GCAACTATTAATCTACAATAAAAGAATATCCTTCAACAGCTGTTACTTTCGGACAATCTTTCTCCTCGACTACAATCTTTTTGATTTCGATGTACTGCTTGGCTGCTTGGTATGGAACAGAAAATTTCAAAGAAAGCTTGACATCCTTATCTTTAGGAAATTCACTTGGCTGGAAGTAGTCCTTGTGAAGAAAGTGCTTAACTTTCTCATCGGGACTGGGGAAGCCTATGTATGATTGGTATCTACCCTCTCTGCTGAGTTCTCCTTCCAGCTGGTAATTGTAGGCTTGATCAGCAACCATGGGTCCACTGCGAGTGACGACAGTGAGACACATCTTACCAGACTTTGATTTGATAGGTTCTCCAAACTTCAGAGTGGTGTAACCTCCCATTCCAAGATAGGTGCTGATCCAAGTGCCTCCAGTAGTAACATTGCTAATCCATCGGTCCACACCATCGACAGTGTAACTGCAATCCGGTTCACCATTGAAACCACAATAAAAGATCTCTTTGCCAGTTTTCTTGTGGGGTACTCTTCCTTCGGGTGGCAAGTTTTCGTCAATTACCTTGCACAATCCATGGTATTTCGCTTTTATGCCATATTTTTTGCAACCATCTCCACAACAGCTGTTTGCTGGACAAGCTGAGCAAATATCTCCACCTTTATAGATTTCTTCACCCTCTACATTTCCACGCGGTCCATAGTTGCATATCAGGTTAGTGTATTTTCGACCATTCGGATGACGGAAATAGAGGTAACCACAACCAATTCGCCACGTTTTTGCCCATAGCAGTTGAGAAATAGTGCTTTTTCCTGAACTTTCAGTGTAGTTCGCAACGACGTTTTTCGTCAAGTATTTAACCTTCATGAAATGCGTAAAGAATTCTGCTTCATAGTCCTGAGCCTCCATTATCGACCCCTCAAATTCGACTATGGATTGTCCCACTGCGAATCTGTCAACCATCCTACAAAGGTCGCAGTCGTGACTTCTTTTGCAAGAATATACCCATTTTTGCGCAATGGCTGCAAGTTCATCGTCCCAAACCATTTCCAGCATGTTTGTAGCTCGTGGGACATGATCCACTCCATACGATTTCCCTCCAGCGACTTTGCTTCTTAGGTTGTTGTGCTGATACAAGATGTAGTTCTTAAGCCAGTCCGAAACATCTTTTTTATAAAAAGCACAG

							GAGCAACAGGTAGGAAGACAGAAAGAATGCTGAAGGCTGT<br>ATCGCTTGTACTTATCGGGGCACAATCCGGCGCGAATCGTTG<br>CGATTGCGCTGACGAAGGCGGTGAGCATTAACAAAATATTTC<br>GGATTTCCATTGTTACAGTCCAGCCGTTGAATAAAATTTCAA<br>ATATAAAGTGAATTTACTGAAAGGTATTTTCACCTTTTATACT<br>CGGAAACTTTCTTAAGAAAAAGATATGAAACCTTATTTTCAG<br>CTGCGGCGCTAAGAGTGACGTTTGTGGTGCTATTCAACCCCT<br>TGACAAGTGCATCTGTTTGGTATTCAGATATCAAACGAAA

PhdCRI21	A9QQ26	5,84E-26	61,111	PF00188	8155,3	CLPTCCSGAFYKKDVTDWLKNYSLYQHNNLRSKVAGGKSHGVDHVPRDTNMLEMVCDDELAAIAEKWVYSCK	TTTGCAAGAATATACCCATTTTTCCGCAATGGCTGCAAGTTCATCGTCACAAACCATTTCCAGCATGTTTGTATCTCGTGGGACATGATCCACTCCATGCGATTTCCCTCCAGCGACTTTGCTCCTTAGGTTGTTGTGCTGATACAAGCTGTAGTTCTTCAGCCAGTCCGTAACATCTTTTTTATAAAAAGCACCGGAGCAACAGGTAGGAAGACAGA
PhdCRI22	A9QQ26	3,84E-07	45,161	PF00188	7511,58	EGISRWVHNTPTRGTWIITFLGSRGYTILKLGEPITSRSGKLCLKVVTRSGPTAADQEYQYELTDKL	GAAGGAATAAGCCGATGGGTTCATAACACACCAACACGCGGTACATGGATCATAACCTTTCTCGGTAGTAGAGGTTACACAATTTTGAAACTTGGAGAGCCCATCACATCAAGATCTGGAAAGCTGTGTTTGAAAGTAGTGACGCGAAGTGGGCCTACTGCGGCTGACCAAGAATATCAGTACGAACTGACTGACAAACTCG

PhdCRI23	A9QQ26	2,67E-68	56,682	PF00188	32106,22	VKTPDIDRFAVGQTI<br>VQFIGSEMTHHDYE<br>MEFSTFFSALENLKK<br>DQVARYTSSAGNSGI<br>TQMLWAKTWRIGC<br>GYLYFHDSSQETKYS<br>LLTCNYGPGGNVEG<br>EEVYKAGSACSACP<br>ANSCCGDGCKKHGI<br>KAKYNGLCKVIDEN<br>LAPEGNAPHTKSGK<br>EVFYCGFNGESDCN<br>YNIEGIDRWVHNTS<br>TGGTWIRTYLGSRGY<br>TILKFEEPITSKSGKL<br>CLKVVTRSGPTAAD<br>QEYKYELTAKLEEEG<br>KYMTIVPFPRATNK<br>VKHMFHTDYLNPYS<br>FPKNRGVKVRITFY*	CTTTATTTCAAGGTATTGTCTGGGGGTGTTGGCCGGAACAGA<br>AAACGCGAGAGTCAGCTGAAAAAGAAAAACACGTTATTCTT<br>TTCAGTCTAGATAACTGGCGTAATGTGTTTAAATTTGTCCGAA<br>AATTGCGATTAAAATTTTGCAGTCGCAATTTGTGGAGTAATA<br>AATGAAATGCTTTGTTACGGTTCTGATTATCGAGAATAACCG<br>ATTACCGGCATAGCTTTTTAGCGAAAGGTCTGACGTCATGGA<br>GGAAAGCAAACATTTATAACTTGATCTGTATGACAACGAAA<br>GAACGAAAAATGAACTTAGTGATAAATGAAATAGTCTGTGA<br>GAGGAAGTAAAATCAGCTGCACTCTCCGTAAAACATTCCAA<br>GCGTCCAATGTCTTATTTAGAGGACGGAGATTATTTCAACAC<br>GCGTAAGCGACAATTCTTTGCGACTTCAGCAATACGTTATAT<br>ATACTTAAAGCATGTAATAGTTTTATATTTATCTATACACTCT<br>AAAACTGAAAGAGTGATTTCTCACTCTTAAAAGTCCACGATA<br>GCACCACGCCGACAAGAGCAGACTCCATTCCTTGAAAACAG<br>TCGAAACTCTCAATCTTTAAGAGTCATAATCTGGAAAATATC<br>ATTCAAAACATGTCAACAAACAACTTCAATCTTTCATGGGGG<br>GAAAGAAATTGCTTTTTTCATTTACAGTGTTAATAACGGGTG<br>AAACACTCATTATAAACCAATTTTTTTCACTTTTTGAAAAAGT<br>GAAGTGTATTTGACACGTTTCTAGTAATATTTTCCCGATGATG<br>ACTCTTAAAGTTGGAAAATTCCCGCTCTTTTCAAAGAATGGA<br>GTGTACTTTTATCAGCATGGTGCTATCATGACCTGTTAAATGT<br>GGACAATCTTTCTTTCGCTTTTAGAATGTGCTAATATTTAAGC<br>AAAGAAAGCTTGTTTTCTTGTAATTGATAATCTGTGAAACTA<br>CCCAACCCATTTGAACATTCTTTCACCGTTAGAGAACGTATTT<br>CTTCCCTCTTAACATAACCTTTATATTAAACAGAACAAGACG<br>AGGAACCACTGTTAATGTTTATTTGAGTACCATATGGGCAGA<br>GAACATTGGCTAACTGGAGTCGCACATCTGTAATAAACTGGA<br>CAACGAAGCAAGGTTGTATGTGGACGAATATCCGACGATAC

							GAGCATGACGCATTGAAAAGTAAGATCATTCTTACTGGCTAT<br>TCTTCAGCAACTTTGTTATTCTTTTATAGAGCTAGTAGAGGTC<br>GACCAAACAAGCAGCTTGATAATAGTCTTTGTACCTACCTAT<br>CTTATAAAAAACCTGAAAGGAATGCTGGTCGAGAAATACTT<br>CTGTCTCAGCGCTATGTTATAAACCATGCAGACTTAAAAATC<br>GATTCCGAAATGTCAATGACGGGCGTTACAACTTTTTATAAT<br>ATTTTCTATTTCGGTTTTCAGCGATTTTAATTAAATAAAATAT<br>ACCCACAATCCAGTAAATTCACCATTCATATATTTTCTTTCGT<br>ATTCACCCACTAGACATAATACTCTGACACCACAAAATCCCC<br>TTCAACGACGGACGCAGACATTGGTAATGTTAAAAGCCTATA<br>TTATATGACATAATTTTGTTAACATTCGAAGTCTGATACAGG<br>GTCTACTGAAAGAATGGCACTGTTTCAAATGTTAATAAAGCA<br>TTCATTTCCAACCTTACACGGAAACAACCTGCACTGTCAGCA<br>GAGCCGGCTGTCCAAGTTCACCCCGCGCTACCAAAATTTCGC<br>TTCTCACTATTGCAGAAGGACCGCAGGAGCATTTTCTAAAAT<br>GGTGTCGCAGCGGGAGAATCCTTCTCCGTGTCCTGCCTTCTG<br>AGGTGTCTAAATCTGCGTTTACAGTACAGCGTAAGTTTCGTTC<br>TCAGTTTGGAAAAGATTCACCGCACAGGAATAACATGAGCA<br>AGTGGTAACGATAGTTTGTAGAAACTGGGTGTTTGTGCAAAG<br>GCAGCGTTTACGTATCACCATTGCCCCGCTCCATACAGGAAC<br>TTCGCGATCGGATAACGCGCGCAATGTGGTCATTACAGTAGA<br>CACGCTACACCGAGTCTTCGATAAATTTGATTATCGTGTAGA<br>TGCTTGTCGTTGTGTCCAAAAGTGCACGCACATAAGGACTGT<br>AATTAAAGTACGAGAAACTTAGGCAGTTGGCGCGGTGCTGA<br>CAATGTAGGTTGCTTCCGTGTAAGGAAGGAAGTAAACTTCTT<br>ATTAACATTTGAAAACGCACCTGTCATTGAGGAAGGCCTGCA<br>TAATTTGATCACTACACGGTTGATATTTATCCTATCAAGTTAT<br>TCAATAAAACGTTATCCTTACCTTTACGCCCCTATTCTTTGGA

							AATGAATACGGATTTAAATAGTCAGTGTGAAACATGTGTTTA<br>ACTTTGTTAGTCGCCCTGGGGAAAGGTACAATTGTCATATAT<br>TTGCCTTCTTCTTCGAGTTTGGCAGTCAGTTCGTACTTATATTC<br>TTGGTCAGCCGCAGTAGGACCACTTCGCGTCACGACTTTCAA<br>ACACAGCTTTCCAGATTTTGATGTGATGGGCTCTTCAAATTTC<br>AAAATTGTGTAACCTCTACTTCCGAGATAGGTTCTGATCCAT<br>GTACCTCCTGTAGATGTGTTATGAACCCATCGGTCTATTCCTT<br>CGATGTTATAGTTGCAATCCGATTCACCATTGAAACCACAAT<br>AGAAGACTTCTTTACCACTTTTTGTGTGAGGGGCGTTTCCTTC<br>CGGTGCTAAGTTTTCGTCTATGACTTTGCATAATCCGTTATAT<br>TTTGCCTTTATTCCGTGCTTTTTGCATCCGTCACCGCAGCAGG<br>AGTTTGCAGGGCAGGCCGAGCAAGCAGATCCAGCTTTGTAC<br>ACTTCCTCACCCTCTACATTTCCGCCGGGTCCGTAGTTGCATG<br>TCAAAAGGCTATATTTTGTTTCTTGGCTGGAATCGTGGAAGTA<br>GAGATAACCGCATCCAATTCGCCATGTCTTCGCCCACAGCAT<br>TTGGGTAATTCCGCTGTTTCCAGCACTGCTTGTATAACGTGCA<br>ACCTGATCCTTCTTTAAATTTTCAAGTGCGCTGAAAAATGTTG<br>AAAACTCCATTTCGTAGTCATGATGGGTCATTTCAGATCCGA<br>TGAACTGGACAATGGTTTGTCCTACTGCGAATCTATCAATGT<br>CTGGGGTCTTTACTCAACGTAAACAGGAGCCGAGCGTTGAA<br>GAACGATTTACAGTCGATAACGGTGGACGTTCAGATGTTAAA<br>TTCCACGTTATCACACTACGTCTGAGAAGCGGATATTCCTAA<br>GGGCGAAAACCATAAAAATTCAATCAATCAACTTACCAACC<br>TAACGGCAGAAATTCCGTCTCCAGTAAAGTTTTATCGAAATT<br>TCGCTCTTAGAATTGCATTTTCTCTGGTGTGGTTATTGGCTGG<br>ACTCCATTGAAGAGGCCTGAACAAGGCCGAAACCTTACTCC<br>AGAGTTACTATTAACTGGAGATATACTTTCTTTTCATTCTCAG<br>ACTATGCTCCTTGGAGTCGTTTCTCGTGCATTTATCTTCAGTTA

							TTTCTGTTTAGAGCAAATTCTGGATAAAATATCAGACTCATTGCCCATCTAACATCAAAATTGGATGATGGGGTGAATAGTTTCAGACAGTAGGATTCCTTTGTAAAGGAAAAACCGGAGAACGGGAACCTGCGTTAGACGAAAGGGGAGAATGCGTAAGAGAATCCGTTGCACGCAGTCCGCAAAAATCTCTGCGCTGTGTCAGTTCTTGTTGGGTGCCATGTGTTCTTTCAGTTACCCCTTCATCCCCAAAATATTATCGTTATGCTGTACATCATCTATGCTCCTGGGCGCACACTTGGGGGGGACACTTACAGTTTGCTATAATTTCAAAGTCATTCATCAAATGCAACTTATTAATACTTCTCGACTTCTTATTTCTGTTTGTTAAGTTTTCAAGTTTGCCCCTCCTGAAAAATATTGTATGGGCGCCCATGATTATGCGAAAATAGAAATTCTGGCAAACTTTAAATTAAATAGGCATTGCATGAAACAACACTACTGTTACACAGTTTGAATGAAGTTCTTTCTGTAAAG

PhdCRI24	A9QQ26	1,82E-114	59,667	PF00188	45865.08<br>**42871.44**	MSFEVRFQNILLLLT<br>VVLYAVTTIRA**GWC**<br>**PDKYERYSLEHSYC**<br>**LRRCCSCPYYKRGI**<br>**TQGLKDLILGQHNS**<br>**YRSKVAGGHSYGV**<br>**GHLSRATNMLEMV**<br>**WDDELAEIAQKRV**<br>**DSCQRNPDCDLCR**<br>**MVDRFAVGQTIVQ**<br>**FIGSEMTHHDYEME**<br>**FSTFFSALENLKKD**<br>**QVARYTSSAGNSGI**<br>**TQMLWAKTWRIGC**<br>**GYLYFHDSSQETKY**<br>**SLLTCNYGPGGNVE**<br>**GEEVYKAGSACSA**<br>**CPANSCCGDGCKK**<br>**HGIKAKYNGLCKVI**<br>**DENLAPEGNAPHT**<br>**KSGKEVFYCGFNGE**<br>**SDCNYNIEGIDRWV**<br>**HNTSTGGTWIRTYL**<br>**GSRGYTILKFEEPIT**<br>**SKSGKLCLKVVTRS**<br>**GPTAADQEYKYELT**<br>**AKLEEEGKYMTIVP**<br>**FPRATNKVKHMFH**	CTTTATTTCAAGGTATTGTCTGGGGGTGTTGGCCGGAACAGA<br>AAACGCGAGAGTCAGCTGAAAAAGAAAAACACGTTATTCTT<br>TTCAGTCTAGATAACTGGCGTAATGTGTTTAAATTTGTCCGAA<br>AATTGCGATTAAAATTTTGCAGTCGCAATTTGTGGAGTAATA<br>AATGAAATGCTTTGTTACGGTTCTGATTATCGAGAATAACCG<br>ATTACCGGCATAGCTTTTTAGCGAAAGGTCTGACGTCATGGA<br>GGAAAGCAAACATTTATAACTTGATCTGTATGACAACGAAA<br>GAACGAAAAATGAACTTAGTGATAAATGAAATAGTCTGTGA<br>GAGGAAGTAAAATCAGCTGCACTCTCCGTAAAACATTCCAA<br>GCGTCCAATGTCTTATTTAGAGGACGGAGATTATTTCAACAC<br>GCGTAAGCGACAATTCTTTGCGACTTCAGCAATACGTTATAT<br>ATACTTAAAGCATGTAATAGTTTTATATTTATCTATACACTCT<br>AAAACTGAAAGAGTGATTTCTCACTCTTAAAAGTCCACGATA<br>GCACCACGCCGACAAGAGCAGACTCCATTCCTTGAAAACAG<br>TCGAAACTCTCAATCTTTAAGAGTCATAATCTGGAAAATATC<br>ATTCAAAACATGTCAACAAACAACTTCAATCTTTCATGGGGG<br>GAAAGAAATTGCTTTTTTCATTTACATTGTTAATAACTGGTGA<br>AACACTCATTATAAACCAATTTTTTTTCACTTTTTGAAAGAGT<br>GAAGTGTATTTGACACGTTTCTAGTAATATTTTCCCGATGATG<br>ACTCTTAAAGTTGGAAAATTCCCGCTCTTTTCAAAGAATGGA<br>GCGTACTCTTATCAGCATGGTGCTATCATGACCTGTTAAATGT<br>GGACAATCTTTCTTTCGCTTTTAGAATGTGCTAATATTTAAGC<br>AAAGAAAGCTTGTTTTCTTGTAATTGATAATCTGTGAAACTA<br>CCCAACCCATTTGAACATTCTTTCACCGTTAGAGAACGTATTT<br>CTTCCCTCTTAACATAACCTTTATATTAAACAGAACAAGACG<br>AGGAACCACTGTTAATGTTTATTTGAGTACCATATGGGCAGA<br>GAACATTGGCTAACTGGAGTCGCACATCTGTAATAAACTGGA<br>CAACGAAGCAAGGTTGTATGTGGACGAATATCCGACGATAC

						**TDYLNPYSFPKNRG VKVRITFY***	GAGCATGACGCATTGAAAAGTAAGATCATTCTTACTGGCTAT TCTTCAGCAACTTTGTTATTCTTTTATAGAGCTAGTAGAGGTC GACCAAACAAGCAGCTTGATAATAGTCTTTGTACCTACCTAT CTTATAAAAAACCTGAAAGGAATGCTGGTCGAGAAATACTT CTGTCTCAGCGCTATGTTATAAACCATGCAGACTTAAAAATC GATTCCGAAATGTCAATGACGGGCGTTACAACTTTTTATAAT ATTTTCTATTTCGGTTTTCAGCGATTTTAATTAAATAAAATAT ACCCACAATCCAGTAAATTCACCATTCATATATTTTCTTTCGT ATTCACCCACTAGACATAATACTCTGACACCACAAAATCCCC TTCAACGACGGACGCAGACATTGGTAATGTTAAAAGCCTATA TTATATGACATAATTTTGTTAACATTCGAAGTCTGATACAGG GTCTACTGAAAGAATGGCACTGTTTCAAATGTTAATAAAGCA TTCATTTCCAACCTTACACGGAAACAACCTGCACTGTCAGCA GAGCCGGCTGTCCAAGTTCACCCCGCGCTACCAAAATTTCGC TTCTCACTATTGCAGAAGGACCGCAGGACCAGTTTCTAAAAT GGTGTCGCAGCGGGAGAATCCTTCTCCGTGTCCTTACTTCTGA GGTGTCTAAATCTGCGTTTACAGTACAGCGTAAGTTTCGTTCT CAGTTTGGAAAAGATTCACCGCACAGGAATAACATGAGCAA GTGGTAACGATAGTTTGTAGAAACTGGGTGTTTGTGCAAAGG CAGCGTTTACGTATCACCATTTCCCCTCTCCATACAGGAACTT CGCGATAGGATAACGCGCGCAATGTGGTCATTACAGTAGAC ACGCTACACCGAGTCATCGATAAATTTGATTTTCGTGTGGAT GCTTGTCGTTGTGTCCAAAGGTGCACGCACATAAGGACTGTA ATTAAAGTACGAGAAACTTAAGCAGTTGGCGCGGTGCTGAC AATATAGGTTGTTTCCGTGTAAGGAAGGAAGTAAACTTCTTA TTAACATTTGAAAACGCACCTGTCATTGAGGAAGGCCTGCAT AATTTGATCACTACACGGTTGATATTTATCCTATCAAGTTATT CAATAAAACGTTATCCTTACCTTTACGCCCCTATTCTTTGGAA

							ATGAATACGGATTTAAATAGTCAGTGTGAAACATGTGTTTAA CTTTGTTAGTCGCCCTGGGGAAAGGTACAATTGTCATATATTT GCCTTCTTCTTCGAGTTTGGCAGTCAGTTCGTACTTATATTCTT GGTCAGCCGCAGTAGGACCACTTCGCGTCACGACTTTCAAAC ACAGCTTTCCAGATTTTGATGTGATGGGCTCTTCAAATTTCAA AATTGTGTAACCTCTACTTCCGAGATAGGTTCTGATCCATGTA CCTCCTGTAGATGTGTTATGAACCCATCGGTCTATTCCTTCGA TGTTATAGTTGCAATCCGATTCACCATTGAAACCACAATAGA AGACTTCTTTACCACTTTTTGTGTGAGGGGCGTTTCCTTCCGG TGCTAAGTTTTCGTCTATGACTTTGCATAATCCGTTATATTTTG CCTTTATTCCGTGCTTTTTGCATCCGTCACCGCAGCAGGAGTT TGCAGGGCAGGCCGAGCAAGCAGATCCAGCTTTGTACACTT CCTCACCCTCTACATTTCCGCCGGGTCCGTAGTTGCATGTCAA AAGGCTATATTTTGTTTCTTGGCTGGAATCGTGGAAGTAGAG ATAACCGCATCCAATTCGCCATGTCTTCGCCCACAGCATTTG GGTAATTCCGCTGTTTCCAGCACTGCTTGTATAACGTGCAAC CTGATCCTTCTTTAAATTTTCAAGTGCGCTGAAAAATGTTGAA AACTCCATTTCGTAGTCATGATGGGTCATTTCAGATCCGATG AACTGGACAATGGTTTGTCCTACTGCGAATCTATCGACCATT CTGCATAGATCACAGTCGGGATTTCTTTGACAGGAATCGACC CTTTTTTGAGCAATTTCTGCAAGTTCGTCATCCCAGACCATTT CCAGCATGTTAGTGGCCCTGGAAAGATGACCCACCCCATAG GAATGCCCGCCGGCGACTTTGCTCCTGTAAGAATTGTGTTGT CCCAATATCAGGTCCTTCAGTCCTTGTGTGATGCCTCGTTTAT AGTAAGGGCAAGAACAACAGCGGCGCAAACAGTAGGAATG CTCAAGACTGTATCTCTCGTACTTGTCGGGACACCATCCTGC ACGGATGGTTGTCACTGCGTAGAGGACTACAGTCAGCAGGA

							GCAAAATATTCTGGAACCTCACCTCAAAGGACATAGCTGGG AGTAATTTTAAATGTCGAAAGCATCTTGGTCTCTTAG

PhdCRI25	W4VS53	7,87E-07	26,214	PF00188	11592	QATTLHFTRETDFSK<br>VMCFKLEYRKGPHI<br>AADRSSNMLSAVFQ<br>STVSNGYYYLNFTSD<br>SKDWTTVEMPVSW<br>WGGPSFMSLTYAVP<br>DGASAQYLDIRQIVV<br>NE	TTCGTTCACTACGATTTGCCGAATATCCAAATACTGGGCTGAT<br>GCTCCATCAGGTACAGCATAGGTCAAAGACATAAAGCTTGG<br>TCCGCCCCACCAAGACACCGGCATCTCCACAGTAGTCCAGTC<br>TTTAGAATCCGAAGTGAAGTTCAAATAGTAGTAACCATTAGA<br>CACCGTGCTTTGAAACACAGCACTTAACATATTACTAGATCT<br>ATCTGCAGCTATATGAGGTCCTTTTCTGTATTCCAGCTTGAAG<br>CACATAACTTTAGAGAAGTCAGTTTCTCTTGTAAAGTGAAGA<br>GTTGTAGCTTG
PhdCRI26	W4VS53	5,63E-36	39,766	PF00188	19827,06	NTDYSGLCKITDDR<br>VPMYSPEGTYIFYCD<br>FNNQSDCNSFITGQ<br>NKWLLYNTLSGNYL<br>GIILNGGEESTMIFN<br>NRIKPSTSSFCFVIRY<br>RKGPNVAGQEENH<br>GATETFTITGNNFEV<br>KQNLPTFSGDAKQQ<br>FSEFSMSLGWNMET<br>EFKVTFSVPAGKPEQ<br>FLEIQRLYVTEGPCE<br>Q*	AATACTGATTATTCGGGGCTCTGCAAGATAACAGATGACAG<br>AGTTCCAATGTACAGTCCCGAAGGAACCTACATCTTTTACTG<br>CGATTTCAATAATCAATCCGATTGCAACAGCTTTATTACGGG<br>ACAAAATAAATGGTTGCTCTACAATACATTATCCGGAAATTA<br>CTTAGGTATAATTCTGAATGGAGGAGAAGAAAGCACTATGA<br>TTTTCAACAATCGAATCAAACCTTCCACATCCTCCTTCTGCTT<br>TGTAATCAGGTATCGCAAAGGACCTAATGTAGCTGGACAGG<br>AGGAAAACCATGGGGCTACAGAAACATTTACGATTACTGGC<br>AACAATTTTGAAGTTAAACAAAATTTACCTACCTTCAGCGGT<br>GATGCCAAACAGCAGTTCAGCGAGTTCAGTATGTCTCTAGGT<br>TGGAATATGGAAACAGAGTTCAAGGTAACATTTTCAGTTCCT<br>GCAGGGAAACCAGAACAGTTCCTCGAAATTCAAAGACTCTA<br>TGTTACAGAAGGACCATGTGAGCAATAAAGCCGGACTGAAT<br>CAGATTTTAAAGGGTGATGGTGTCTATTCATTCAGGGTTTTAT<br>TTTCAAGGTATATGCTACAGAAGTTGCGTGTTCTGTGACCAG<br>ACGAAAAGGGAGAGAAATCATTCTTGTCTTGTATATTTTTCT<br>ATTTGCCATAATAAAAT

PhdCRI27	Q2XXR2	5,90E-10	24,852	PF00188	23927,08	MDQQEDWEDDSIFLPSKKAISLDVDSFRKDMIEAHNHYRTLHDSPPLEHLQILEETAQDWADTIAAKGHLEYCEKLKNVGESLCMVDLESCSVPSAKDMVRKWYKEIKAYTFAYPRWRKGAIHFTQMVWKSSKYIGIGVAPVPKQPRLVIIVRYSPGGNSNYPGEFQRNVQPRISLKSTDSFFMDDSSFVRTYSGRFRSISSEDAYI*	TCTCTCTAAAAGGCAACAGCTTTATCAAATTGTCTCGTGATTCTTCCTGCAAATAAAACAATGACATCAATTTTTTGCGCGCAAGATCGAGAAAGGAAACAGTATGTGTAACACCGGACACAGCATAAATAGAAAAACGGAAACCCCAAGAAACGTCCGATACTCAATTTGAAAACATGGATCAGCAGGAAGACTGGGAAGACGATTCCATCTTCCTTCCTTCGAAGAAAGCCATCTCGCTGGACGTAGACTCCTTCAGAAAGGACATGATCGAAGCGCACAACCACTATCGCACCCTTCACGATAGTCCTCCTCTGGAACATCTGCAAATTCTCGAGGAGACAGCTCAGGACTGGGCGGATACTATTGCAGCGAAGGGTCACCTGGAATACTGTGAAAAATTGAAAAACGTTGGAGAAAGTCTATGCATGGTTGACCTCGAATCATGCTCTGTACCAAGTGCCAAAGACATGGTTCGCAAGTGGTACAAAGAAATTAAAGCCTACACGTTCGCTTATCCAAGATGGAGGAAAGGTGCCATCCACTTCACTCAGATGGTATGGAAAAGCTCAAAGTACATTGGTATAGGCGTAGCTCCAGTTCCCAAACAACCACGTCTTGTCATAATTGTTCGATACTCTCCTGGGGGAAACTCCAACTACCCAGGAGAGTTCCAGAGGAATGTTCAACCACGAATCTCCCTCAAATCAACCGATTCTTTCTTCATGGACGATTCATCCTTCGTTAGGACCTATTCTGGCAGATTCAGAAGCATTTCGTCAGAAGATGCTTACATATGAACGAGAGGAGAAAGTTCACGGAAACAAGTTTACGCTGTAAAATCGACTAAAATGTGTATTTAGTCTATTTTATTCTGTAATCCCTCAATTGCCAAAATGATACGCATTTTGCTTTTAAATTTTATTTTTGATATGAAAACTGTAGTTAATGTTCTTGTTGATGCGAAGTATAGGTAAAATTGTTTATAACCTATCACACGCCTACGTTTTCCTTCCTTTTTAATTCTTTGCGTCTGTCTTGAAAACTTCACTCT

PhdCRI28	Q2XXR2	5,90E-10	24,852	PF00188	23927,08	MDQQEDWEDDSIFL PSKKAISLDVDSFRK DMIEAHNHYRTLH DSPPLEHLQILEETA QDWADTIAAKGHL EYCEKLKNVGESLC MVDLESCSVPSAKD MVRKWYKEIKAYTF AYPRWRKGAIHFTQ MVWKSSKYIGIGVA PVPKQPRLVIIVRYSP GGNSNYPGEFQRNV QPRISLKSTDSFFMD DSSFVRTYSGRFRSIS SEDAYI*	TCTCTCTAAAAGGCAACAGCTTTATCAAATTGTCTCGTGATTC TTCCTGCAAATAAAACAATGACATCAATTTTTTGCGCGCAAG ATCGAGAAAGGAAACAGTATGTGTAACACCGGACACAGCAT AAATAGAAAAACGGAAACCCCAAGAAACGTCCGATACTCA ATTTGAAAACATGGATCAGCAGGAAGACTGGGAAGACGATT CCATCTTCCTTCCTTCGAAGAAAGCCATCTCGCTGGACGTAG ACTCCTTCAGAAAGGACATGATCGAAGCGCACAACCACTAT CGCACCCTTCACGATAGTCCTCCTCTGGAACATCTGCAAATT CTCGAGGAGACAGCTCAGGACTGGGCGGATACTATTGCAGC GAAGGGTCACCTGGAATACTGTGAAAAATTGAAAAACGTTG GAGAAAGTCTATGCATGGTTGACCTCGAATCATGCTCTGTAC CAAGTGCCAAAGACATGGTTCGCAAGTGGTACAAAGAAATT AAAGCCTACACGTTCGCTTATCCAAGATGGAGGAAAGGTGC CATCCACTTCACTCAGATGGTATGGAAAAGCTCAAAGTACAT TGGTATAGGCGTAGCTCCAGTTCCCAAACAACCACGTCTTGT CATAATTGTTCGATACTCTCCTGGGGGAAACTCCAACTACCC AGGAGAGTTCCAGAGGAATGTTCAACCACGAATCTCCCTCA AATCAACCGATTCTTTCTTCATGGACGATTCATCCTTCGTTAG GACCTATTCTGGCAGATTCAGAAGCATTTCGTCAGAAGATGC TTACATATGAACGAGAGGAGAAAGTTCACGGAAACAAGTTT ACGCTGTAAAATCGACTAAAATGTGTATTTAGTCTATTTTATT CTGTAATCCCTCAATTGCCAAAATGATACGCATTTTGCTTTTA AATTTTATTTTTGATATGAAAACTGTAGTTAATGTTCTTGTTG ATGCGAAGTATAGGTAAAATTGTTTATAACCTATCACACGCC TACGTTTTCCTTCCTTTTTTTTTCTCAGAGTGAAGTTTTCAAGA CAGACGC

PhdCRI29	W4VS53	1,49E-76	53,061	PF00188	22415,94	LRSSVNANDITTILRE<br>HNILRSRIATGREQD<br>LPTAANMLQMVWD<br>DELAAVSQKHADQ<br>CLFEHDCSNCRRVQ<br>NYGVGQNLAIEQVS<br>NSPNIPDPNWPSAIK<br>SWYDEVEFFSPNYID<br>PFIAPPPEPTYGHFSQ<br>LVWAESWRIGCGYV<br>LYTDRNMYQKLYVC<br>NYGPAGNTIARSMY<br>QQGQPCSACPENSC<br>CDRTCGANTDY	GAATAATCAGTATTTGCTCCACATGTTCTATCACAACAAGAA<br>TTCTCTGGGCATGCCGAACAAGGTTGACCTTGTTGGTACATA<br>CTTCGAGCTATGGTATTGCCAGCTGGTCCATAATTGCAGACG<br>TAAAGTTTCTGATACATGTTACGATCTGTATATAAGACATATC<br>CACATCCAATGCGCCATGATTCAGCCCAAACCAGCTGGCTG<br>AAATGGCCATAAGTAGGCTCTGGGGGAGGAGCAATAAATGG<br>GTCAATATAGTTTGGGCTGAAAAATTCTACTTCATCATACCA<br>GCTTTTGATGGCACTTGGCCAGTTGGGGTCTGGAATATTAGG<br>ACTGTTTGAAACTTGTTCAATAGCAAGATTTTGGCCCACTCC<br>ATAGTTCTGAACTCTTCGACAGTTTGAACAATCATGTTCAAAT<br>AGACATTGGTCAGCATGCTTTTGAGAAACAGCAGCTAGTTCA<br>TCGTCCCATACCATTTGGAGCATATTTGCAGCTGTTGGCAAGT<br>CCTGTTCCCTACCTGTCGCAATCCTGCTTCTGAGAATATTGTG<br>TTCTCTAAGAATAGTAGTGATGTCATTTGCATTTACAGACGA<br>CCTTAG
PhdCRI30	W4VS53	1,78E-48	47,727	PF00188	22272,93	AQKHADQCIFAHD<br>CPDCRSVEAFSVGQ<br>NIATRAKTGNSLPTP<br>DWESTIQSLYDEVKD<br>FYPQWISPFRAPKYP<br>MTGHYTQALWATS<br>WKVGCGYTIHSYSN<br>DPRFQFIELYTCNYG<br>PTGNLYMQDMYRIG<br>PACSQCPENTCCGK<br>QCELMGVRSDYDGL<br>CKTVSSFGPQPEFPR	GCTCAGAAACATGCCGATCAATGCATTTTTGCCCATGACTGT<br>CCTGATTGTCGATCAGTTGAAGCCTTCTCGGTGGGACAAAAT<br>ATTGCAACAAGGGCTAAAACCGGAAATTCTTTGCCAACTCCT<br>GATTGGGAATCGACCATCCAGAGCCTTTATGATGAAGTGAA<br>AGATTTTTATCCTCAATGGATCAGCCCATTCAGAGCTCCAAA<br>ATATCCTATGACGGGACATTACACTCAGGCACTATGGGCAAC<br>TTCATGGAAAGTTGGCTGCGGATATACCATTCACAGTTATTCT<br>AACGATCCTCGATTTCAATTTATAGAGCTATACACCTGCAAT<br>TATGGGCCCACGGGTAATCTTTACATGCAAGATATGTACAGA<br>ATCGGACCAGCTTGCAGTCAATGTCCTGAAAACACCTGCTGT<br>GGGAAACAGTGTGAATTAATGGGAGTCAGATCGGATTATGA<br>TGGCCTATGCAAAACAGTTAGCTCCTTTGGACCTCAGCCGGA

						GNVYLWCNFREGHPNTDWCEFVIE	ATTCCCTCGGGGAAACGTTTACCTGTGGTGCAATTTTAGAGAAGGCCACCCCAATACAGACTGGTGTGAGTTTGTCATTGAA
PhdCRI31	P0DPU2	5,16E-12	30,833	PF00188	15212,11	VLDEHNRLRASLANGKEINAHRYYMPKASNMRELQWNCTLQKAAQEWMSRCEYQHSPDLSKKSENIGYFAHSAMDKKRIENGGVEITDNFWSELNESYPYNPSNTLTNSVLSAGVVTHFVAVRLKSRIYSIL	GTTCTGGATGAGCACAACAGGCTGCGGGCGAGCTTGGCCAATGGAAAAGAAATTAATGCTCACAGATATTACATGCCAAAAGCATCGAACATGCGTGAACTGCAATGGAACTGTACGCTTCAAAAGGCCGCTCAGGAATGGATGAGTCGCTGCGAATACCAACACTCGCCTGACCTGTCAAAGAAGAGCGAAAATATCGGATATTTCGCGCACTCCGCAATGGACAAAAAGAGGATTGAAAATGGCGGAGTCGAAATAACGGATAATTTCTGGTCTGAACTAAACGAGAGCTACCCATACAATCCATCGAACACACTGACAAACAGCGTTCTTAGCGCTGGAGTCGTCACCCATTTTGTTGCGGTACGTCTGAAATCTCGCATTTACTCTATCTTA
PhdCRI32	W4VS53	2,10E-10	62,857	PF00188	6145.22<br>**3860.35**	MLFRLVSLPLLLLAVTFVADA**QQCPDLYKRYSSEHTYCKPPNKSCDILKRGVSD**	GTCGGAGACACCTCGTTTCAAAATGTCACAGGATTTGTTGGGTGGCTTGCAGTATGTATGTTCAGAACTATACCGCTTGTAGAGGTCTGGGCACTGTTGAGCATCAGCAACGAACGTCACGGCCAACAGCAGCAATGGTAGAGAAACAAGTCGGAACAACATGTTGCGTCCTCTTTTTCTTTGCTCAGAAGAAGTGAAACTCGTGAATTAACCTTCGGGAACGCGATTTTATACGCAGAGATTTGCTTGACTACCTGAAACATTGACAGCAATAT

PhdCRI33	Q8JI38	1,53E-24	33,846	PF00188<br>PF08562	22222.47<br>**20352.19**	MHFGLLVLLALIADS<br>AFG**QGYICPGSKLS**<br>**PQQRGEFLAKHNH**<br>**LRAVLANGKSVDK**<br>**DGVRLPPARNMVP**<br>**VGWSCEAEQVAQR**<br>**WAEHCVFEHSDRN**<br>**FRKGLGENLYMSYP**<br>**PTDEGVKIGAERAS**<br>**QSWWNELPELYHFS**<br>**MNRDLVLTPQVFG**<br>**TGIGHWTQMAWA**<br>**NTTHIGCAIKKCEQ**<br>**MVLVVCEYAPAGN**<br>**VINRKIYEPR***	TTTTTTTTGAACAGTTAATTTTTTATCAGTGCAAATCAAAATC<br>ACGAGTGTATCACTCAAAAAAGAGTTATCACATTATTGCTTA<br>TCGTGGTTCGTAGATTTTTCGGTTGATTACATTGCCGGCAGGG<br>GCATATTCACAGACGACCAAGACCATTTGTTCGCACTTCTTG<br>ATAGCGCATCCAATATGAGTGGTGTTCGCCCAAGCCATCTGT<br>GTCCAGTGTCCAATGCCTGTACCAAACACTTGAGGTGTCAAG<br>ACGAGATCTCTGTTCATTGAGAAGTGGTAAAGTTCTGGAAGC<br>TCGTTCCACCACGACTGACTGGCTCTCTCAGCACCAATCTTC<br>ACACCTTCGTCTGTTGGCGGATATGACATGTAGAGATTTTCA<br>CCAAGTCCTTTCCTGAAGTTCCGGTCAGAATGCTCGAAGACG<br>CAATGTTCTGCCCATCTCTGCGCTACCTGTTCTGCTTCGCAGC<br>TCCAGCCCACTGGCACCATATTCCTAGCAGGTGGCAGTCGCA<br>CACCATCCTTGTCTACTGACTTCCCGTTTGCAAGTACTGCACG<br>CAGATGATTATGCTTCGCGAGGAATTCTCCACGTTGCTGTGG<br>TGACAGTTTGCTGCCTGGGCATATATAGCCCTGTCCAAATGC<br>GCTGTCAGCGATAAGCGCAAGAAGAACCAGGAGACCGAAA<br>TGCATCGCTATTTTGCAGCTATTAGAGTAAGCAAAT

PhdCRI34	Q2XXQ5	1,74E-37	35,433	PF00188<br><br>PF08562	31690.42	RRLSILMTSKSTVLSH<br>LQTILSFLFLSWLLTV<br>EAKLPKPKYHGRAIP<br>QRELDTRYTNTKKKI<br>VLIHNFYRAKVEPPA<br>SNMLEMTWHKGAQ<br>RAAQGWADACQVL<br>THDDPLGRWVEDY<br>GSCGQNIFVSSIQVD<br>WMYVAKVWYMEH<br>KNFTYGSSKNNLSDI<br>GHYTQMVWYNSHR<br>VGCGFHYCGKEVAK<br>KPFYNYVCNYCPIG<br>NSPRSLGKPYEAGR<br>ACGKCTKYCKYKKL<br>CTNSCPFSDLWMNC<br>ADLNTTWNSWLCG<br>NEGNERFKSCQATC<br>RCPHAIH*	GGAGATCTCGCGCTAAAGAAGAAGGAAGAGCGACGCTTGAC<br>GCGATGTGGACGATTTCTTTTGGTGCCGCAGGAAGCCCAGGG<br>TCGCCCAAGGTTGGCTCATCCTAATTAAAGCATAAATGGGTC<br>AGCGCAAATTTCCGATCTCCGTAATCTCCCAAATCGGATACT<br>CATCATCTACCCGAGCTGCCGTGCAGTTGAAATCTCTCTCAG<br>ATACATAGAGGAGACTCAGCATTTTGATGACCTCCAAAAGC<br>ACCGTTCTCTCACATCTCCAAACGATACTTTCATTTCTGTTTTT<br>ATCATGGCTGTTAACAGTAGAAGCAAAATTACCTAAACCAA<br>AATACCACGGCAGAGCCATACCTCAGCGGGAACTAGATACC<br>AGATATACCAACACAAAAAAGAAAATCGTCCTTATTCACAA<br>CTTTTACAGGGCAAAGGTGGAACCCCCAGCTAGCAATATGCT<br>AGAAATGACGTGGCACAAAGGGGCTCAACGTGCAGCTCAGG<br>GATGGGCCGACGCTTGCCAAGTACTCACTCACGACGATCCTT<br>TGGGAAGATGGGTGGAAGATTACGGTTCCTGTGGCCAAAAC<br>ATCTTCGTTTCCAGCATTCAAGTGGATTGGATGTACGTTGCCA<br>AAGTATGGTACATGGAGCACAAGAACTTTACGTATGGAAGT<br>TCCAAAAATAATTTATCTGACATTGGACACTATACGCAGATG<br>GTTTGGTACAACAGCCATCGAGTGGGGTGTGGATTTCACTAC<br>TGCGGAAAAGAAGTTGCAAAAAAGCCTTTTTACAACTACGT<br>CTGCAACTACTGTCCAATAGGCAATAGTCCTAGAAGCCTCGG<br>CAAACCTTACGAAGCTGGAAGAGCTTGCGGAAAGTGCACAA<br>AGTATTGTAAATATAAGAAATTGTGCACTAACAGTTGCCCTT<br>TCTCCGATCTCTGGATGAACTGCGCAGACCTGAACACGACCT<br>GGAACTCTTGGCTTTGCGGAAACGAAGGAAACGAGAGATTC<br>AAGTCTTGCCAAGCCACTTGTAGATGCCCGCACGCCATACAC<br>TGATGTGTGACATTCGCTCGTACACCGACCGACCGCCCTGCT<br>TGCTGGGTTGAACATTTCTTGTAAAAGTTAAAAGAGCTACCA<br>TTCTCGTCAAGCAGAATCGGTAGCTTGCGATACTTGAGATAC

								TCGAAAATCGCTTGATACCTTTCGGAACGCAGTCGTCGTGCG TCAACAAACCTGGCTCGTTTTTCTTTCGTATTTTTTTTCCTTCCT TTTTTGACGGCCTTGCTTTGCTCATTCACGATTAGGTTCTCAG CATTTCTTTTGCTTCTTTCTGCCGAGTTCAAGAATTCTAGAAG GTATCAAGAGAAGCGTACAATGTACAATGTTGTACGTGTAA ATAACGTCCTTTTCGACCGGACCATGGGGGCGAAGAGATTTT CTTTCCTTTTAATGATCAGAATAAGTGGGCAGAATTCAAAAA TATCGAGTTCGATTCTCAAGATGAAGGAAGGGGGTATTCGGC TTAAATTGGCCCAATTCTTTGCACTGGAGATTGAAGGAAAAA GAAATTGCTTTTTTTTTTTTTTTTTTCCAACGATAAGGAATAA

PhdCRI35	Q3SB05	1,59E-15	33,784	PF00188<br>PF08562	16971.28	GKFEDDCLKAHND<br>YRVKHGVPEIVLSKE<br>MCAYSKEWADHLA<br>ATDSFKHRTQDKKY<br>GENIFMKWSSDPNH<br>MITGKEPVDSWYSEI<br>KDHVFGREPTSLKSG<br>HFTQVIWKECKSLG<br>VAWARSKSGKILVV<br>ANYDPAGNFIGKFA<br>QNVPPPKK*	GGAAAATTCGAGGACGATTGCCTGAAAGCTCACAACGACTA<br>TCGGGTCAAGCACGGTGTGCCAGAAATAGTCCTGTCTAAAG<br>AGATGTGTGCGTATTCCAAAGAATGGGCTGACCACCTAGCA<br>GCCACAGATTCTTTCAAGCATCGTACGCAGGACAAAAAGTA<br>CGGCGAAAACATATTTATGAAGTGGAGTTCGGACCCTAACC<br>ACATGATTACAGGGAAGGAGCCAGTGGACAGCTGGTACAGT<br>GAAATTAAAGATCACGTTTTTGGAAGGGAGCCAACGAGCCT<br>GAAATCTGGTCATTTTACTCAAGTAATATGGAAGGAATGCAA<br>ATCATTGGGAGTTGCCTGGGCCAGAAGCAAAAGTGGAAAGA<br>TCCTTGTCGTTGCAAACTACGATCCGGCTGGTAACTTCATCGG<br>AAAGTTCGCGCAAAATGTACCGCCGCCAAAGAAATAAATGG<br>TGAAAAGTGTTGAATCACGTCGGCAACTTTATTGTATGTACC<br>TAAAGTAGAATATTTCTAGTGATGTCTTATTACAACTATTACG<br>AGAATTCTTAATTCTTTCTAAAGAAAGGTATACACACTTTAA<br>AAATGTAATCGTTGATCTCAAGATCAGACATTTGAAGAGAGT<br>ACAAATATTTAAAATTTTTCCGTAGATATGACATGAAGTAGT<br>TGTATAGAAATGAG

PhdCRI36	Q2XXQ5	6,09E-45	39,910	PF00188<br><br>PF08562	29896.5<br><br>**28105.27**	MKTLIFGAVAAVIA<br>VSFA**FPKLSRPKLYG**<br>**TAIPRRDLDPSYNS**<br>**TRRKIVLFHNYFRT**<br>**RVKPTAADMLEMS**<br>**WHDGAAQDAQRW**<br>**AESCQLLLHDNVT**<br>**GRWTDDYGTCGQ**<br>**NIFVANVQVPWFFA**<br>**AKVWFWERLNFTY**<br>**GEDTNDPNVVGHY**<br>**TQMVWFSTHRVGC**<br>**GFSYCDASNTTGQP**<br>**YYSYVCNYCPIGNH**<br>**PERFDRPYTNGTAC**<br>**SGCEGHCRFNALCT**<br>**NGCPYADYWTNCL**<br>**ELNSTWPNWLCDD**<br>**HSTDRYQACRATC**<br>**NCGGSIR***	GTTACAATAGATTTATTTGTACTCTAGGCTTCATATTATTTAT<br>AAAGTGTAAAAATATGCATTTAAATAAGAATCTTTACAAATA<br>TAAGTAACATGCTACAATACAAACATGTTTATAATAACATTG<br>ATTTTATGTATAGAAAACTTTGTTAGTATTAACACTGTACGAA<br>TTCAACTGCGAACATTTTCTCCAGACTTTCACCGTATGGATCC<br>TCCGCAGTTACATGTAGCACGGCATGCTTGGTAGCGGTCTGT<br>AGAGTGGTCATCGCATAGCCAGTTAGGCCACGTAGAATTGA<br>GCTCAAGGCAGTTCGTCCAGTAATCAGCATAAGGACACCCG<br>TTGGTGCAAAGAGCGTTGAATCTACAGTGCCCTTCACAACCC<br>GAGCAAGCTGTTCCATTTGTATACGGGCGATCAAACCTTTCA<br>GGATGATTACCGATGGGGCAATAATTGCAAACATAACTGTA<br>GTATGGCTGTCCTGTTGTATTGCTTGCATCACAATAGCTGAAA<br>CCACATCCTACTCGGTGAGTACTGAACCACACCATCTGTGTA<br>TAATGCCCTACTACATTAGGATCATTAGTGTCTTCACCATACG<br>TAAAATTCAATCTCTCCCAAAACCATACTTTTGCTGCGAAGA<br>ACCAAGGCACTTGGACATTGGCTACGAAGATATTCTGACCGC<br>AAGTTCCGTAGTCATCTGTCCATCTTCCTGTCACATTGTCGTG<br>CAGAAGTAATTGACAAGATTCGGCCCACCTTTGTGCATCTTG<br>CGCGGCACCATCGTGCCAAGACATTTCCAACATATCGGCTGC<br>TGTGGGCTTCACACGGGTCCTGAAGTAGTTGTGAAAAAGGAC<br>GATCTTCCTTCTCGTGGAATTATAAGATGGGTCGAGATCCCG<br>TCGTGGAATAGCTGTTCCATACAGTTTAGGGCGAGATAATTT<br>GGGAAAAGCAAAACTCACTGCTATAACAGCAGCAACTGCTC<br>CAAAAATTAAAGTCTTCATATTGAATAGCTCTGATGTTTTACG<br>CTAAGTAACTGAGACGTGTATGAATATTCTTACGGATACAGA<br>TGTTCTTCATAAGTCGGTCATCTGGATGTGTAGATGGTTGAGT<br>AACGCGATCATCCTGTGCGAAGGGTGTTTTTTCCAGAGGCGC

							GTTGACCATATTTTAGTTCAGGTACAGAAAGGAGTTAGGTTC CACGTAGACTTCCG
TCTP							
Translation-controlled tumor proteins							
**Transcript ID**	**Uniprot ID reference protein**	**e-value**	**Identity (%)**	**PFAM domain**	**MW (Da)**	**Amino acid sequence**	**Nucleotide sequence**

PhdTCT01	M5B4R	1,27E-110	83,815	PF00838	19719,23	MRIFKDLITGDEMFT<br>DSSKYKVIEDCLYEV<br>ECRHVQRRLGDVQL<br>DGANPSQEEADEGT<br>DECVESGLDLVLNQ<br>RLVETGFNKNDYKA<br>YLKLYTKALQDKWK<br>EMGKSDSEIAEAKSK<br>FTEAVKKVLPKIGDL<br>QFFMGESSNPDGLIA<br>LLEYRENASGDETPI<br>MIFFKHGLEEEKV*	TTTTTTTTTTAAGTCAGTAATCAAATTTTAATAATGAAATAAA<br>AAATAATTACAAACTTTTCATATATTGAAAATTTATCGATAA<br>ATTATGGACAGGAAAATTGCGCTATACTTTTTCTTCTTCGAGT<br>CCATGCTTAAAAAAAATCATGATGGGAGTTTCATCTCCAGAG<br>GCGTTTTCACGGTACTCGAGTAACGCGATTAATCCATCAGGA<br>TTTGAGCTCTCTCCCATGAAAAATTGTAAATCGCCGATTTTTG<br>GTAGAACTTTCTTGACGGCCTCGGTGAACTTGGACTTGGCTTC<br>AGCGATTTCTGAATCGCTTTTTCCCATTTCTTTCCATTTATCTT<br>GCAAAGCCTTGGTATACAGCTTCAAATAGGCCTTATAGTCGT<br>TTTTGTTAAATCCAGTCTCCACCAGTCGTTGATTCAAAACTAA<br>ATCTAATCCAGATTCGACACATTCATCAGTACCTTCATCAGC<br>TTCTTCTTGAGAAGGATTAGCGCCATCCAGTTGAACGTCACC<br>TAGTCTTCTTTGAACATGTCTACATTCTACTTCATACAGGCAA<br>TCTTCGATTACCTTGTATTTGGAGGAATCTGTAAACATTTCAT<br>CGCCTGTAATTAAATCTTTGAAAATACGCATCTTTAGGCGTA<br>CACACTAAAATGAAGGACAAGTTACTAAGAAACACGCGGTG<br>CAACAAGGCTTAGCTACAAGCGCTGAATTACTCCACCTCACT<br>CGAGAAGGCCTAAAGTCAAAAGACCGTTGACACGGCAATGA<br>CG

PhdTCT02	M5B4R	1,62E-78	76,190	PF00838	18836,27	MIIFKDLITGDEMFT<br>DSSKYKLIDGCLYEV<br>ECRHVQRRQGDIQL<br>EGANPSQEEMDEGT<br>EECVESGLDLVLNQ<br>RLVETGFTKNDYKN<br>YLKTYTKSLQDKWK<br>EMGKSDNDIAEAKS<br>KTTEAVKKVLPKLG<br>DMQFFMDFPTSHER<br>LLFMLSRAFHGLKG<br>GSNSRENN*	ATTAGCTCTCTACGTACAAATTCTTTCTATCTGTTCCTTTCCTT<br>TTCTGCCTCTGCGCTTTGTGGTGTAAATTTGCTTGGTGTCGTGG<br>TTAAAATTTTCTTTTTTTTCCTACCGTAATGATTATCTTCAAAG<br>ATCTTATTACAGGTGATGAGATGTTCACCGATTCGTCTAAAT<br>ATAAGTTAATTGATGGGTGTTTATATGAAGTCGAATGTCGGC<br>ATGTTCAACGAAGACAAGGAGACATCCAGTTAGAAGGAGCA<br>AACCCTTCTCAAGAAGAAATGGATGAAGGAACTGAAGAATG<br>TGTTGAATCTGGTTTAGATTTAGTTTTAAATCAAAGACTCGTA<br>GAAACTGGTTTTACAAAAAATGACTACAAGAATTATCTAAA<br>AACTTACACAAAATCTTTACAAGACAAGTGGAAAGAAATGG<br>GTAAGAGTGATAATGACATTGCTGAAGCAAAGTCCAAAACT<br>ACAGAAGCTGTTAAAAAGGTTCTTCCAAAGCTTGGAGATATG<br>CAGTTTTTTATGGACTTTCCCACCAGCCATGAGAGGTTGCTGT<br>TTATGCTTTCACGTGCTTTCCACGGTTTGAAGGGAGGGAGTA<br>ACTCTAGAGAAAATAATTAAAGGCACAAGAAACTGCTCTGG<br>CACAGGCACTAGAAGAAATGAGGACAACGATTTGGCACAA<br>AAAAGGTGAAAGCTCAAATCCAGATGGTCTTGTTGCTCTTCT<br>AGAATATCGTGAAAATTCAAGTGGCGATGAAACCCCAATAA<br>TGATATTCTTTAAACATGGTCTAGAAGAAGAAAAAGTTGGA<br>AATTGCTTTCTTCGATTCTTCCTGTCATCCTCAGGATTTCCTTG<br>TCGTCCAGGAACGTTGAAAGGGAGAGACACGGTGAAAGGA<br>AGTATCATAACTACAAGGATCTCTTAGTCAAAGCCTTGTCAA<br>ACTGGCGTTGCAGCACCACCTTATAATAGATTTATCTGCCTA<br>ACAAGAGCTCCATACCCTGCATTTTTGTGACAGAATATGTAT<br>ATAATGTTTTGACAGCTGTGCCTTTTTAATTTATGCTATTTGTT<br>GCCACAACATCATTTCTTGAAATGCAGTGACTTTGACTAAAA<br>AAATTAATCAGCATTGCTATAATTACCTGTCAAATCTTGCATT

							TCTAATGTAAAATGATTGAGGTAATTTCTGCAATAAAATTAT AGATAATTTTGAAAAAAAAAA

PhdTCT03	M5B4R	6,08E-15	57,377	PF00838	9055,41	MGVYMKSNVGLQD<br>KWKEMGKSDNDIA<br>EAKSKTTEAVKKVL<br>PKLGDMQFFMDFPT<br>SHERLLFMLSRAFH<br>GLKGGSNSRENN*	ATTAGCTCTCTACGTACAAATTCTTTCTATCTGTTCCTTTCCTT<br>TTCTGCCTCTGCGCTTTGTGGTGTAAATTTGCTTGGTGTCGTGG<br>TTAAAATTTTCTTTTTTTTCCTACCGTAATGATTATCTTCAAAG<br>ATCTTATTACAGGTGATGAGATGTTCACCGATTCGTCTAAAT<br>ATAAGTTAATTGATGGGTGTTTATATGAAGTCGAATGTCGGTT<br>TACAAGACAAGTGGAAAGAAATGGGTAAGAGTGATAATGA<br>CATTGCTGAAGCAAAGTCCAAAACTACAGAAGCTGTTAAAA<br>AGGTTCTTCCAAAGCTTGGAGATATGCAGTTTTTTATGGACTT<br>TCCCACCAGCCATGAGAGGTTGCTGTTTATGCTTTCACGTGCT<br>TTCCACGGTTTGAAGGGAGGGAGTAACTCTAGAGAAAATAA<br>TTAAAGGCACAAGAAACTGCTCTGGCACAGGCACTAGAAGA<br>AATGAGGACAACGATTTGGCACAAAAAAGTTTTATTTGCAGC<br>AATTTTAAACCGGCGAGAAATATCAACTGCCTCGAGTCAAC<br>ATAAATATTAGTTGGACTTGCCAGCATCAACGTGAGTTCTAG<br>ATAGTTGACGTGATCTTTCTCTTTACTTATGCAGGTGGATACA<br>GAGTAATGGCCATCGAATTTAAAAGCGAAGTTTTTTCGAATC<br>TTCAATAAAGGATGTCACTAGTATCTGCCAGTTGTAATACAT<br>CGCAGCCATAGAAGGTTCTAGTTAGACTTGAGGAGAGCTGT<br>AGCCTGTCCAGAGATGCCAGATGAGGAGCCGGATGGGTTTC<br>TACTTTGACGGATTTTAGTAGCTGAGAAGTCGTCCATGTTTAC<br>CGAAGGATGAGTTTAGCGAGGCCTAGGGAAGTCAAGTGGAT<br>GATGTCAATTAAAGTTTCTACTGGTGTTTAAGGCAAATTGAA<br>GTTCTGACTGGATTTGGATTGACGGTATCGCTACAGAATTTG<br>AAATCAAGTTGCCTATATTAGAAGATTTTCCGGAAAGAAAAC<br>TTTTAGTCCAGTTCCCGTTTTTGATGTTTAATTTGGGCTTTCAC<br>GTTTTTTAAAGTTTTGTCAAAATTTCGGTTAATAGTGGCACTT<br>ATGTTACCGGTTCTGAATTCAACTATCTGGATTAATAATGTAA<br>CGGGTATTTTTATATTTATTTATTACTGTTACTGAAGTCTTTAC

							TGTGTTTATAAATTGTTGCAATGTATGTTATTTAAAATGTTGAACTAAAAGCGTTAAATGCTTCATGTGTGTTTCTGCTGAATTTGTAAGTCATGCTAAATGTTGAAAACTCATTTTAAGAGTTTTTAAAGTGACTTTAAAATTTTAATCACTTTGCACCTTTGAATTTTGAAACTTAATTATTTGCATTAGTTTTATTAGATAGCAGCTCTGTTTGGTTAGCGATTATTTTACTCTATCCATTTAATAAGGTTATGGGCTTTTATTAAATGTGCATGGGTCTGTTTACCATAACAATACCAAAGACTTACTTTGATTTATTATCAGTTAAAGCTTTACTTTTACTTGCACATTCCTTTTATGTAACTATATGTATGCATCGGGATTGCGTAAAATTTTCTCTGGTCAAAAGGGGTTGCGAATGGTAAAAGTTTAACTATGTACAGCAGGGCTTCTTAAAGAGAGATTACTATGGTCGTTACCAGTCGATTTTTATCTAAAGTTTTACAGAGAATGAGAAAATTGGAAAGTGGATTTTCCACATATATGCACCTTTTGTCTGTTGGGTTATAAATTGTACAAAAATGAATATATTTTCAACTGTCTTATACATTTAAAAATTTCAATCCGGTTGATCTGAGTTTCAAAGCACGAAATGTATTATTACAAATAATATTTTGAAATGATCGGTGAAAATCCTTTTTCCGGTAAAATTTTCAAAAAATGTTTTAAAATTAAAATCTTATTTCGCGAAAGTCACATATTAATAACATCATGGATA

PhdTCT04	M5B4R	3,70E-82	81,295	PF00838	16139,25	MIIFKDLITGDEMFTDSSKYKLIDGCLYEVECRHVQRRQGDIQLEGANPSQEEMDEGTEECVESGLDLVLNQRLVETGFTKNDYKNYLKTYTKSLQDKWKEMGKSDNDIAEAKSKTTEAVKKVLPKLGDMQFFMGCQSNTL*	ATTAGCTCTCTACGTACAAATTCTTTCTATCTGTTCCTTTCCTTTTCTGCCTCTGCGCTTTGTGGTGTAAATTTGCTTGGTGTCGTGGTTAAAATTTTCTTTTTTTTCCTACCGTAATGATTATCTTCAAAGATCTTATTACAGGTGATGAGATGTTCACCGATTCGTCTAAATATAAGTTAATTGATGGGTGTTTATATGAAGTCGAATGTCGGCATGTTCAACGAAGACAAGGAGACATCCAGTTAGAAGGAGCAAACCCTTCTCAAGAAGAAATGGATGAAGGAACTGAAGAATGTGTTGAATCTGGTTTAGATTTAGTTTTAAATCAAAGACTCGTAGAAACTGGTTTTACAAAAAAATGACTACAAGAATTATCTAAAAACTTACACAAAATCTTTACAAGACAAGTGGAAAGAAATGGGTAAGAGTGATAATGACATTGCTGAAGCAAAGTCCAAAACTACAGAAGCTGTTAAAAAGGTTCTTCCAAAGCTTGGAGATATGCAGTTTTTTATGGGGTGCCAGTCCAATACTCTGTAGAAAGAGTTGGAGTTTCTATTCTTGGAATGGAATAATTGTTGAACCCAAGTGGGGGTCAGTCCAATTTGAAGTATAATTAGTGTGAAAGCTCAAATCCAGATGGTCTTGTTGCTCTTCTAGAATATCGTGAAAATTCAAGTGGCGATGAAACCCCAATAATGATATTCTTTAAACATGGTCTAGAAGAAGAAAAAGTT

PhdTCT05	M5B4R	3,73E-16	67,925	PF00838	6358,4	MGVYMKSNVGLQD<br>KWKEMGKSDNDIA<br>EAKSKTTEAVKKVL<br>PKLGDMQFFMGCQS<br>NTL*	ATTAGCTCTCTACGTACAAATTCTTTCTATCTGTTCCTTTCCTT<br>TTCTGCCTCTGCGCTTTGTGGTGTAAATTTGCTTGGTGTCGTGG<br>TTAAAATTTTCTTTTTTTTCCTACCGTAATGATTATCTTCAAAG<br>ATCTTATTACAGGTGATGAGATGTTCACCGATTCGTCTAAAT<br>ATAAGTTAATTGATGGGTGTTTATATGAAGTCGAATGTCGGTT<br>TACAAGACAAGTGGAAAGAAATGGGTAAGAGTGATAATGA<br>CATTGCTGAAGCAAAGTCCAAAACTACAGAAGCTGTTAAAA<br>AGGTTCTTCCAAAGCTTGGAGATATGCAGTTTTTTATGGGGTG<br>CCAGTCCAATACTCTGTAGAAAGAGTTGGAGTTTCTATTCTTG<br>GAATGGAATAATTGTTGAACCCAAGTGGGGGTCAGTCCAATT<br>TGAAGTATAATTAGTACTTTCCCACCAGCCATGAGAGGTTGC<br>TGTTTATGCTTTCACGTGCTTTCCACGGTTTGAAGGGAGGGAG<br>TAACTCTAGAGAAAATAATTAAAGGCACAAGAAACTGCTCT<br>GGCACAGGCACTAGAAGAAATGAGGACAACGATTTGGCACA<br>AAAAAGTTTTATTTGCAGCAATTTTAAACCGGCGAGAAATAT<br>CAACTGCCTCGAGTCAACATAAATATTAGTTGGACTTGCCAG<br>CATCAACGTGAGTTCTAGATAGTTGACGTGATCTTTCTCTTTA<br>CTTATGCAGGTGGATACAGAGTAATGGCCATCGAATTTAAAA<br>GCGAAGTTTTTTCGAATCTTCAATAAAGGATGTCACTAGTAT<br>CTGCCAGTTGTAATACATCGCAGCCATAGAAGGTTCTAGTTA<br>GACTTGAGGAGAGCTGTAGCCTGTCCAGAGATGCCAGATGA<br>GGAGCCGGATGGGTTTCTACTTTGACGGATTTTAGTAGCTGA<br>GAAGTCGTCCATGTTTACCGAAGGATGAGTTTAGCGAGGCCT<br>AGGGAAGTCAAGTGGATGATGTCAATTAAAGTTTCTACTGGT<br>GTTTAAGGCAAATTGAAGTTCTGACTGGATTTGGATTGACGG<br>TATCGCTACAGAATTTGAAATCAAGTTGCCTATATTAGAAGA<br>TTTTCCGGAAAGAAAACTTTTAGTCCAGTTCCCGTTTTTGATG<br>TTTAATTTGGGCTTTCACGTTTTTTAAAGTTTTGTCAAAATTTC

							GGTTAATAGTGGCACTTATGTTACCGGTTCTGAATTCAACTAT<br>CTGGATTAATAATGTAACGGGTATTTTTATATTTATTTATTACT<br>GTTACTGAAGTCTTTACTGTGTTTATAAATTGTTGCAATGTAT<br>GTTATTTAAAATGTTGAACTAAAAGCGTTAAATGCTTCATGT<br>GTGTTTCTGCTGAATTTGTAAGTCATGCTAAATGTTGAAAACT<br>CATTTTAAGAGTTTTTAAAGTGACTTTAAAATTTTAATCACTT<br>TGCACCTTTGAATTTTGAAACTTAATTATTTGCATTAGTTTTAT<br>TAGATAGCAGCTCTGTTTGGTTAGCGATTATTTTACTCTATCC<br>ATTTAATAAGGTTATGGGCTTTTATTAAATGTGCATGGGTCTG<br>TTTACCATAACAATACCAAAGACTTACTTTGATTTATTATCAG<br>TTAAAGCTTTACTTTTACTTGCACATTCCTTTTATGTAACTATA<br>TGTATGCATCGGGATTGCGTAAAATTTTCTCTGGTCAAAAGG<br>GGTTGCGAATGGTAAAAGTTTAACTATGTACAGCAGGGCTTC<br>TTAAAGAGAGATTACTATGGTCGTTACCAGTCGATTTTTATCT<br>AAAGTTTTACAGAGAATGAGAAAATTGGAAAGTGGATTTTCC<br>ACATATATGCACCTTTTGTCTGTTGGGTTATAAATTGTACAAA<br>AATGAATATATTTTCAACTGTCTTATACATTTAAAAATTTCAA<br>TCCGGTTGATCTGAGTTTCAAAGCACGAAATGTATTATTACA<br>AATAATATTTTGAAATGATCGGTGAAAATCCTTTTTCCGGTA<br>AAATTTTCAAAAAATGTTTTAAAATTAAAATCTTATTTCGCGA<br>AAGTCACATATTAATAACATCATGGATA

PhdTCT06	M5B4R	3,62E-75	76,190	PF00838	18836,27	MIIFKDLITGDEMFT<br>DSSKYKLIDGCLYEV<br>ECRHVQRRQGDIQL<br>EGANPSQEEMDEGT<br>EECVESGLDLVLNQ<br>RLVETGFTKNDYKN<br>YLKTYTKSLQDKWK<br>EMGKSDNDIAEAKS<br>KTTEAVKKVLPKLG<br>DMQFFMDFPTSHER<br>LLFMLSRAFHGLKG<br>GSNSRENN*	ATTAGCTCTCTACGTACAAATTCTTTCTATCTGTTCCTTTCCTT<br>TTCTGCCTCTGCGCTTTGTGGTGTAAATTTGCTTGGTGTCGTGG<br>TTAAAATTTTCTTTTTTTTCCTACCGTAATGATTATCTTCAAAG<br>ATCTTATTACAGGTGATGAGATGTTCACCGATTCGTCTAAAT<br>ATAAGTTAATTGATGGGTGTTTATATGAAGTCGAATGTCGGC<br>ATGTTCAACGAAGACAAGGAGACATCCAGTTAGAAGGAGCA<br>AACCCTTCTCAAGAAGAAATGGATGAAGGAACTGAAGAATG<br>TGTTGAATCTGGTTTAGATTTAGTTTTAAATCAAAGACTCGTA<br>GAAACTGGTTTTACAAAAAATGACTACAAGAATTATCTAAA<br>AACTTACACAAAATCTTTACAAGACAAGTGGAAAGAAATGG<br>GTAAGAGTGATAATGACATTGCTGAAGCAAAGTCCAAAACT<br>ACAGAAGCTGTTAAAAAGGTTCTTCCAAAGCTTGGAGATATG<br>CAGTTTTTTATGGACTTTCCCACCAGCCATGAGAGGTTGCTGT<br>TTATGCTTTCACGTGCTTTCCACGGTTTGAAGGGAGGGAGTA<br>ACTCTAGAGAAAATAATTAAAGGCACAAGAAACTGCTCTGG<br>CACAGGCACTAGAAGAAATGAGGACAACGATTTGGCACAA<br>AAAAGTTTTATTTGCAGCAATTTTAAACCGGCGAGAAATATC<br>AACTGCCTCGAGTCAACATAAATATTAGTTGGACTTGCCAGC<br>ATCAACGTGAGTTCTAGATAGTTGACGTGATCTTTCTCTTTAC<br>TTATGCAGGTGGATACAGAGTAATGGCCATCGAATTTAAAA<br>GCGAAGTTTTTTCGAATCTTCAATAAAGGATGTCACTAGTAT<br>CTGCCAGTTGTAATACATCGCAGCCATAGAAGGTTCTAGTTA<br>GACTTGAGGAGAGCTGTAGCCTGTCCAGAGATGCCAGATGA<br>GGAGCCGGATGGGTTTCTACTTTGACGGATTTTAGTAGCTGA<br>GAAGTCGTCCATGTTTACCGAAGGATGAGTTTAGCGAGGCCT<br>AGGGAAGTCAAGTGGATGATGTCAATTAAAGTTTCTACTGGT<br>GTTTAAGGCAAATTGAAGTTCTGACTGGATTTGGATTGACGG<br>TATCGCTACAGAATTTGAAATCAAGTTGCCTATATTAGAAGA

							TTTTCCGGAAAGAAAACTTTTAGTCCAGTTCCCGTTTTTGATG<br>TTTAATTTGGGCTTTCACGTTTTTTAAAGTTTTGTCAAAATTTC<br>GGTTAATAGTGGCACTTATGTTACCGGTTCTGAATTCAACTAT<br>CTGGATTAATAATGTAACGGGTATTTTTATATTTATTTATTACT<br>GTTACTGAAGTCTTTACTGTGTTTATAAATTGTTGCAATGTAT<br>GTTATTTAAAATGTTGAACTAAAAGCGTTAAATGCTTCATGT<br>GTGTTTCTGCTGAATTTGTAAGTCATGCTAAATGTTGAAAACT<br>CATTTTAAGAGTTTTTAAAGTGACTTTAAAATTTTAATCACTT<br>TGCACCTTTGAATTTTGAAACTTAATTATTTGCATTAGTTTTAT<br>TAGATAGCAGCTCTGTTTGGTTAGCGATTATTTTACTCTATCC<br>ATTTAATAAGGTTATGGGCTTTTATTAAATGTGCATGGGTCTG<br>TTTACCATAACAATACCAAAGACTTACTTTGATTTATTATCAG<br>TTAAAGCTTTACTTTTACTTGCACATTCCTTTTATGTAACTATA<br>TGTATGCATCGGGATTGCGTAAAATTTTCTCTGGTCAAAAGG<br>GGTTGCGAATGGTAAAAGTTTAACTATGTACAGCAGGGCTTC<br>TTAAAGAGAGATTACTATGGTCGTTACCAGTCGATTTTTATCT<br>AAAGTTTTACAGAGAATGAGAAAATTGGAAAGTGGATTTTCC<br>ACATATATGCACCTTTTGTCTGTTGGGTTATAAATTGTACAAA<br>AATGAATATATTTTCAACTGTCTTATACATTTAAAAATTTCAA<br>TCCGGTTGATCTGAGTTTCAAAGCACGAAATGTATTATTACA<br>AATAATATTTTGAAATGATCGGTGAAAATCCTTTTTCCGGTA<br>AAATTTTCAAAAAATGTTTTAAAATTAAAATCTTATTTCGCGA<br>AAGTCACATATTAATAACATCATGGATA

PhdTCT07	M5B4R	4,03E-107	83,815	PF00838	23758,91	MIIFKDLITGDEMFTDSSKYKLIDGCLYEVECRHVQRRQGDIQLEGANPSQEEMDEGTEECVESGLDLVLNQRLVETGFTKNDYKNYLKTYTKSLQDKWKEMGKSDNDIAEAKSKTTEAVKKVLPKLGDMQFFMGESSNPDGLVALLEYRENSSGDETPIMIFFKHGLEEEKVGNCFLRFFLSSSGFPCRPGTLKGRDTVKGSIITTRIS*	ATTAGCTCTCTACGTACAAATTCTTTCTATCTGTTCCTTTCCTTTTCTGCCTCTGCGCTTTGTGGTGTAAATTTGCTTGGTGTCGTGGTTAAAATTTTCTTTTTTTTCCTACCGTAATGATTATCTTCAAAGATCTTATTACAGGTGATGAGATGTTCACCGATTCGTCTAAATATAAGTTAATTGATGGGTGTTTATATGAAGTCGAATGTCGGCATGTTCAACGAAGACAAGGAGACATCCAGTTAGAAGGAGCAAACCCTTCTCAAGAAGAAATGGATGAAGGAACTGAAGAATGTGTTGAATCTGGTTTAGATTTAGTTTTAAATCAAAGACTCGTAGAAACTGGTTTTACAAAAAATGACTACAAGAATTATCTAAAAACTTACACAAAATCTTTACAAGACAAGTGGAAAGAAATGGGTAAGAGTGATAATGACATTGCTGAAGCAAAGTCCAAAACTACAGAAGCTGTTAAAAAGGTTCTTCCAAAGCTTGGAGATATGCAGTTTTTTATGGGTGAAAGCTCAAATCCAGATGGTCTTGTTGCTCTTCTAGAATATCGTGAAAATTCAAGTGGCGATGAAACCCCAATAATGATATTCTTTAAACATGGTCTAGAAGAAGAAAAAGTTGGAAATTGCTTTCTTCGATTCTTCCTGTCATCCTCAGGATTTCCTTGTCGTCCAGGAACGTTGAAAGGGAGAGACACGGTGAAAGGAAGTATCATAACTACAAGGATCTCTTAGTCAAAGCCTTGTCAAACTGGCGTTGCAGCACCACCTTATAATAGATTTATCTGCCTAACAAGAGCTCCATACCCTGCATTTTTGTGACAGAATATGTATATAATGTTTTGACAGCTGTGCCTTTTTAATTTATGCTATTTGTTGCCACAACATCATTTCTTGAAATGCAGTGACTTTGACTAAAAAAATTAATCAGCATTGCTATAATTACCTGTCAAATCTTGCATTTCTAATGTAAAATGATTGAGGTAATTTCTGCAATAAAATTATAGATAATTTTGAAAAAAAAAA

PhdTCT08	M5B4R	1,56E-75	76,190	PF00838	18836,27	MIIFKDLITGDEMFT DSSKYKLIDGCLYEV ECRHVQRRQGDIQL EGANPSQEEMDEGT EECVESGLDLVLNQ RLVETGFTKNDYKN YLKTYTKSLQDKWK EMGKSDNDIAEAKS KTTEAVKKVLPKLG DMQFFMDFPTSHER LLFMLSRAFHGLKG GSNSRENN*	ATTAGCTCTCTACGTACAAATTCTTTCTATCTGTTCCTTTCCTT TTCTGCCTCTGCGCTTTGTGGTGTAAATTTGCTTGGTGTCGTGG TTAAAATTTTCTTTTTTTTCCTACCGTAATGATTATCTTCAAAG ATCTTATTACAGGTGATGAGATGTTCACCGATTCGTCTAAAT ATAAGTTAATTGATGGGTGTTTATATGAAGTCGAATGTCGGC ATGTTCAACGAAGACAAGGAGACATCCAGTTAGAAGGAGCA AACCCTTCTCAAGAAGAAATGGATGAAGGAACTGAAGAATG TGTTGAATCTGGTTTAGATTTAGTTTTAAATCAAAGACTCGTA GAAACTGGTTTTACAAAAAATGACTACAAGAATTATCTAAA AACTTACACAAAATCTTTACAAGACAAGTGGAAAGAAATGG GTAAGAGTGATAATGACATTGCTGAAGCAAAGTCCAAAACT ACAGAAGCTGTTAAAAAGGTTCTTCCAAAGCTTGGAGATATG CAGTTTTTTATGGACTTTCCCACCAGCCATGAGAGGTTGCTGT TTATGCTTTCACGTGCTTTCCACGGTTTGAAGGGAGGGAGTA ACTCTAGAGAAAATAATTAAAGGCACAAGAAACTGCTCTGG CACAGGCACTAGAAGAAATGAGGACAACGATTTGGCACAA AAAAGGTGGATACAGAGTAATGGCCATCGAATTTAAAAGCG AAGTTTTTTCGAATCTTCAATAAAGGATGTCACTAGTATCTGC CAGTTGTAATACATCGCAGCCATAGAAGGTTCTAGTTAGACT TGAGGAGAGCTGTAGCCTGTCCAGAGATGCCAGATGAGGAG CCGGATGGGTTTCTACTTTGACGGATTTTAGTAGCTGAGAAG TCGTCCATGTTTACCGAAGGATGAGTTTAGCGAGGCCTAGGG AAGTCAAGTGGATGATGTCAATTAAAGTTTCTACTGGTGTTT AAGGCAAATTGAAGTTCTGACTGGATTTGGATTGACGGTATC GCTACAGAATTTGAAATCAAGTTGCCTATATTAGAAGATTTT CCGGAAAGAAAACTTTTAGTCCAGTTCCCGTTTTTGATGTTTA ATTTGGGCTTTCACGTTTTTTAAAGTTTTGTCAAAATTTCGGTT AATAGTGGCACTTATGTTACCGGTTCTGAATTCAACTATCTGG

							ATTAATAATGTAACGGGTATTTTTATATTTATTTATTACTGTTA<br>CTGAAGTCTTTACTGTGTTTATAAATTGTTGCAATGTATGTTA<br>TTTAAAATGTTGAACTAAAAGCGTTAAATGCTTCATGTGTGTT<br>TCTGCTGAATTTGTAAGTCATGCTAAATGTTGAAAACTCATTT<br>TAAGAGTTTTTAAAGTGACTTTAAAATTTTAATCACTTTGCAC<br>CTTTGAATTTTGAAACTTAATTATTTGCATTAGTTTTATTAGAT<br>AGCAGCTCTGTTTGGTTAGCGATTATTTTACTCTATCCATTTA<br>ATAAGGTTATGGGCTTTTATTAAATGTGCATGGGTCTGTTTAC<br>CATAACAATACCAAAGACTTACTTTGATTTATTATCAGTTAA<br>AGCTTTACTTTTACTTGCACATTCCTTTTATGTAACTATATGTA<br>TGCATCGGGATTGCGTAAAATTTTCTCTGGTCAAAAGGGGTT<br>GCGAATGGTAAAAGTTTAACTATGTACAGCAGGGCTTCTTAA<br>AGAGAGATTACTATGGTCGTTACCAGTCGATTTTTATCTAAA<br>GTTTTACAGAGAATGAGAAAATTGGAAAGTGGATTTTCCACA<br>TATATGCACCTTTTGTCTGTTGGGTTATAAATTGTACAAAAAT<br>GAATATATTTTCAACTGTCTTATACATTTAAAAATTTCAATCC<br>GGTTGATCTGAGTTTCAAAGCACGAAATGTATTATTACAAAT<br>AATATTTTGAAATGATCGGTGAAAATCCTTTTTCCGGTAAAA<br>TTTTCAAAAAATGTTTTAAAATTAAAATCTTATTTCGCGAAAG<br>TCACATATTAATAACATCATGGATA

PhdTCT09	M5B4R	2,86E-76	81,295	PF00838	16139,25	MIIFKDLITGDEMFTDSSKYKLIDGCLYEVECRHVQRRQGDIQLEGANPSQEEMDEGTEECVESGLDLVLNQRLVETGFTKNDYKNYLKTYTKSLQDKWKEMGKSDNDIAEAKSKTTEAVKKVLPKLGDMQFFMGCQSNTL*	ATTAGCTCTCTACGTACAAATTCTTTCTATCTGTTCCTTTCCTTTTCTGCCTCTGCGCTTTGTGGTGTAAATTTGCTTGGTGTCGTGGTTAAAATTTTCTTTTTTTTCCTACCGTAATGATTATCTTCAAAGATCTTATTACAGGTGATGAGATGTTCACCGATTCGTCTAAATATAAGTTAATTGATGGGTGTTTATATGAAGTCGAATGTCGGCATGTTCAACGAAGACAAGGAGACATCCAGTTAGAAGGAGCAAACCCTTCTCAAGAAGAAATGGATGAAGGAACTGAAGAATGTGTTGAATCTGGTTTAGATTTAGTTTTAAATCAAAGACTCGTAGAAACTGGTTTTACAAAAAATGACTACAAGAATTATCTAAAAACTTACACAAAATCTTTACAAGACAAGTGGAAAGAAATGGGTAAGAGTGATAATGACATTGCTGAAGCAAAGTCCAAAACTACAGAAGCTGTTAAAAAGGTTCTTCCAAAGCTTGGAGATATGCAGTTTTTTATGGGGTGCCAGTCCAATACTCTGTAGAAAGAGTTGGAGTTTCTATTCTTGGAATGGAATAATTGTTGAACCCAAGTGGGGGTCAGTCCAATTTGAAGTATAATTAGTACTTTCCCACCAGCCATGAGAGGTTGCTGTTTATGCTTTCACGTGCTTTCCACGGTTTGAAGGGAGGGAGTAACTCTAGAGAAAATAATTAAAGGCACAAGAAACTGCTCTGGCACAGGCACTAGAAGAAATGAGGACAACGATTTGGCACAAAAAAGTTTTATTTGCAGCAATTTTAAACCGGCGAGAAATATCAACTGCCTCGAGTCAACATAAATATTAGTTGGACTTGCCAGCATCAACGTGAGTTCTAGATAGTTGACGTGATCTTTCTCTTTACTTATGCAGGTGGATACAGAGTAATGGCCATCGAATTTAAAAGCGAAGTTTTTTCGAATCTTCAATAAAGGATGTCACTAGTATCTGCCAGTTGTAATACATCGCAGCCATAGAAGGTTCTAGTTAGACTTGAGGAGAGCTGTAGCCTGTCCAGAGATGCCAGATGAGGAGCCGGATGGGTTTCTACTTTGACGGATTTTAGTAGCTGAGAAGTCGTCCATGTTTACCGAAGGATGAGTTTAGCGAGGCCTAGGGAAGTCAAGTGGATGATGTC

							AATTAAAGTTTCTACTGGTGTTTAAGGCAAATTGAAGTTCTG<br>ACTGGATTTGGATTGACGGTATCGCTACAGAATTTGAAATCA<br>AGTTGCCTATATTAGAAGATTTTCCGGAAAGAAAACTTTTAG<br>TCCAGTTCCCGTTTTTGATGTTTAATTTGGGCTTTCACGTTTTT<br>TAAAGTTTTGTCAAAATTTCGGTTAATAGTGGCACTTATGTTA<br>CCGGTTCTGAATTCAACTATCTGGATTAATAATGTAACGGGT<br>ATTTTTATATTTATTTATTACTGTTACTGAAGTCTTTACTGTGT<br>TTATAAATTGTTGCAATGTATGTTATTTAAAATGTTGAACTAA<br>AAGCGTTAAATGCTTCATGTGTGTTTCTGCTGAATTTGTAAGT<br>CATGCTAAATGTTGAAAACTCATTTTAAGAGTTTTTAAAGTG<br>ACTTTAAAATTTTAATCACTTTGCACCTTTGAATTTTGAAACT<br>TAATTATTTGCATTAGTTTTATTAGATAGCAGCTCTGTTTGGTT<br>AGCGATTATTTTACTCTATCCATTTAATAAGGTTATGGGCTTT<br>TATTAAATGTGCATGGGTCTGTTTACCATAACAATACCAAAG<br>ACTTACTTTGATTTATTATCAGTTAAAGCTTTACTTTTACTTGC<br>ACATTCCTTTTATGTAACTATATGTATGCATCGGGATTGCGTA<br>AAATTTTCTCTGGTCAAAAGGGGTTGCGAATGGTAAAAGTTT<br>AACTATGTACAGCAGGGCTTCTTAAAGAGAGATTACTATGGT<br>CGTTACCAGTCGATTTTTATCTAAAGTTTTACAGAGAATGAG<br>AAAATTGGAAAGTGGATTTTCCACATATATGCACCTTTTGTCT<br>GTTGGGTTATAAATTGTACAAAAATGAATATATTTTCAACTGT<br>CTTATACATTTAAAAATTTCAATCCGGTTGATCTGAGTTTCAA<br>AGCACGAAATGTATTATTACAAATAATATTTTGAAATGATCG<br>GTGAAAATCCTTTTTCCGGTAAAATTTTCAAAAAATGTTTTAA<br>AATTAAAATCTTATTTCGCGAAAGTCACATATTAATAACATC<br>ATGGATA

PhdTCT10	M5B4R	2,62E-108	83,815	PF00838	19756,24	MIIFKDLITGDEMFT<br>DSSKYKLIDGCLYEV<br>ECRHVQRRQGDIQL<br>EGANPSQEEMDEGT<br>EECVESGLDLVLNQ<br>RLVETGFTKNDYKN<br>YLKTYTKSLQDKWK<br>EMGKSDNDIAEAKS<br>KTTEAVKKVLPKLG<br>DMQFFMGESSNPDG<br>LVALLEYRENSSGDE<br>TPIMIFFKHGLEEEK<br>V*	ATTAGCTCTCTACGTACAAATTCTTTCTATCTGTTCCTTTCCTT<br>TTCTGCCTCTGCGCTTTGTGGTGTAAATTTGCTTGGTGTCGTGG<br>TTAAAATTTTCTTTTTTTTCCTACCGTAATGATTATCTTCAAAG<br>ATCTTATTACAGGTGATGAGATGTTCACCGATTCGTCTAAAT<br>ATAAGTTAATTGATGGGTGTTTATATGAAGTCGAATGTCGGC<br>ATGTTCAACGAAGACAAGGAGACATCCAGTTAGAAGGAGCA<br>AACCCTTCTCAAGAAGAAATGGATGAAGGAACTGAAGAATG<br>TGTTGAATCTGGTTTAGATTTAGTTTTAAATCAAAGACTCGTA<br>GAAACTGGTTTTACAAAAAATGACTACAAGAATTATCTAAA<br>AACTTACACAAAATCTTTACAAGACAAGTGGAAAGAAATGG<br>GTAAGAGTGATAATGACATTGCTGAAGCAAAGTCCAAAACT<br>ACAGAAGCTGTTAAAAAGGTTCTTCCAAAGCTTGGAGATATG<br>CAGTTTTTTATGGGTGAAAGCTCAAATCCAGATGGTCTTGTTG<br>CTCTTCTAGAATATCGTGAAAATTCAAGTGGCGATGAAACCC<br>CAATAATGATATTCTTTAAACATGGTCTAGAAGAAGAAAAA<br>GTTTAATAGATTTATCTGCCTAACAAGAGCTCCATACCCTGC<br>ATTTTTGTGACAGAATATGTATATAATGTTTTGACAGCTGTGC<br>CTTTTTAATTTATGCTATTTGTTGCCACAACATCATTTCTTGAA<br>ATGCAGTGACTTTGACTAAAAAAATTAATCAGCATTGCTATA<br>ATTACCTGTCAAATCTTGCATTTCTAATGTAAAATGATTGAGG<br>TAATTTCTGCAATAAAATTATAGATAATTTTGAAAAAAAAAA

PhdTCT11	M5B4R	9,67E-44	78,161	PF00838	9975,38	MGVYMKSNVGLQDKWKEMGKSDNDIAEAKSKTTEAVKKVLPKLGDMQFFMGESSNPDGLVALLEYRENSSGDETPIMIFFKHGLEEEKV*	ATTAGCTCTCTACGTACAAATTCTTTCTATCTGTTCCTTTCCTTTTCTGCCTCTGCGCTTTGTGGTGTAAATTTGCTTGGTGTCGTGGTTAAAATTTTCTTTTTTTTCCTACCGTAATGATTATCTTCAAAGATCTTATTACAGGTGATGAGATGTTCACCGATTCGTCTAAATATAAGTTAATTGATGGGTGTTTATATGAAGTCGAATGTCGGTTTACAAGACAAGTGGAAAGAAATGGGTAAGAGTGATAATGACATTGCTGAAGCAAAGTCCAAAACTACAGAAGCTGTTAAAAAGGTTCTTCCAAAGCTTGGAGATATGCAGTTTTTTATGGGTGAAAGCTCAAATCCAGATGGTCTTGTTGCTCTTCTAGAATATCGTGAAAATTCAAGTGGCGATGAAACCCCAATAATGATATTCTTTAAACATGGTCTAGAAGAAGAAAAAGTTTAATAGATTTATCTGCCTAACAAGAGCTCCATACCCTGCATTTTTGTGACAGAATATGTATATAATGTTTTGACAGCTGTGCCTTTTTAATTTATGCTATTTGTTGCCACAACATCATTTCTTGAAATGCAGTGACTTTGACTAAAAAAATTAATCAGCATTGCTATAATTACCTGTCAAATCTTGCATTTCTAATGTAAAATGATTGAGGTAATTTCTGCAATAAAATTATAGATAATTTTGAAAAAAAAAA

PhdTCT12	M5B4R	3,38E-79	76,190	PF00838	18836,27	MIIFKDLITGDEMFT<br>DSSKYKLIDGCLYEV<br>ECRHVQRRQGDIQL<br>EGANPSQEEMDEGT<br>EECVESGLDLVLNQ<br>RLVETGFTKNDYKN<br>YLKTYTKSLQDKWK<br>EMGKSDNDIAEAKS<br>KTTEAVKKVLPKLG<br>DMQFFMDFPTSHER<br>LLFMLSRAFHGLKG<br>GSNSRENN*	ATTAGCTCTCTACGTACAAATTCTTTCTATCTGTTCCTTTCCTT<br>TTCTGCCTCTGCGCTTTGTGGTGTAAATTTGCTTGGTGTCGTGG<br>TTAAAATTTTCTTTTTTTTCCTACCGTAATGATTATCTTCAAAG<br>ATCTTATTACAGGTGATGAGATGTTCACCGATTCGTCTAAAT<br>ATAAGTTAATTGATGGGTGTTTATATGAAGTCGAATGTCGGC<br>ATGTTCAACGAAGACAAGGAGACATCCAGTTAGAAGGAGCA<br>AACCCTTCTCAAGAAGAAATGGATGAAGGAACTGAAGAATG<br>TGTTGAATCTGGTTTAGATTTAGTTTTAAATCAAAGACTCGTA<br>GAAACTGGTTTTACAAAAAATGACTACAAGAATTATCTAAA<br>AACTTACACAAAATCTTTACAAGACAAGTGGAAAGAAATGG<br>GTAAGAGTGATAATGACATTGCTGAAGCAAAGTCCAAAACT<br>ACAGAAGCTGTTAAAAAGGTTCTTCCAAAGCTTGGAGATATG<br>CAGTTTTTTATGGACTTTCCCACCAGCCATGAGAGGTTGCTGT<br>TTATGCTTTCACGTGCTTTCCACGGTTTGAAGGGAGGGAGTA<br>ACTCTAGAGAAAATAATTAAAGGCACAAGAAACTGCTCTGG<br>CACAGGCACTAGAAGAAATGAGGACAACGATTTGGCACAA<br>AAAAGGTGAAAGCTCAAATCCAGATGGTCTTGTTGCTCTTCT<br>AGAATATCGTGAAAATTCAAGTGGCGATGAAACCCCAATAA<br>TGATATTCTTTAAACATGGTCTAGAAGAAGAAAAAGTTTAAT<br>AGATTTATCTGCCTAACAAGAGCTCCATACCCTGCATTTTTGT<br>GACAGAATATGTATATAATGTTTTGACAGCTGTGCCTTTTTAA<br>TTTATGCTATTTGTTGCCACAACATCATTTCTTGAAATGCAGT<br>GACTTTGACTAAAAAAATTAATCAGCATTGCTATAATTACCT<br>GTCAAATCTTGCATTTCTAATGTAAAATGATTGAGGTAATTTC<br>TGCAATAAAATTATAGATAATTTTGAAAAAAAAAA

PhdTCT13	M5B4R	2,76E-82	81,295	PF00838	16139,25	MIIFKDLITGDEMFT<br>DSSKYKLIDGCLYEV<br>ECRHVQRRQGDIQL<br>EGANPSQEEMDEGT<br>EECVESGLDLVLNQ<br>RLVETGFTKNDYKN<br>YLKTYTKSLQDKWK<br>EMGKSDNDIAEAKS<br>KTTEAVKKVLPKLG<br>DMQFFMGCQSNTL*	ATTAGCTCTCTACGTACAAATTCTTTCTATCTGTTCCTTTCCTT<br>TTCTGCCTCTGCGCTTTGTGGTGTAAATTTGCTTGGTGTCGTGG<br>TTAAAATTTTCTTTTTTTTCCTACCGTAATGATTATCTTCAAAG<br>ATCTTATTACAGGTGATGAGATGTTCACCGATTCGTCTAAAT<br>ATAAGTTAATTGATGGGTGTTTATATGAAGTCGAATGTCGGC<br>ATGTTCAACGAAGACAAGGAGACATCCAGTTAGAAGGAGCA<br>AACCCTTCTCAAGAAGAAATGGATGAAGGAACTGAAGAATG<br>TGTTGAATCTGGTTTAGATTTAGTTTTAAATCAAAGACTCGTA<br>GAAACTGGTTTTACAAAAAAATGACTACAAGAATTATCTAAA<br>AACTTACACAAAATCTTTACAAGACAAGTGGAAAGAAATGG<br>GTAAGAGTGATAATGACATTGCTGAAGCAAAGTCCAAAACT<br>ACAGAAGCTGTTAAAAAGGTTCTTCCAAAGCTTGGAGATATG<br>CAGTTTTTTATGGGGTGCCAGTCCAATACTCTGTAGAAAGAG<br>TTGGAGTTTCTATTCTTGGAATGGAATAATTGTTGAACCCAAG<br>TGGGGGTCAGTCCAATTTGAAGTATAATTAGTGTAAGTATTA<br>TTTAGAGGAAGATGTGTTGGGGTAAAATATTCAACTTCTTTA<br>AACAAATAAAACATATTTATATCATTTTCTTTAAAAAAA

PhdTCT14	M5B4R	5,59E-15	57,377	PF00838	9055,41	MGVYMKSNVGLQD<br>KWKEMGKSDNDIA<br>EAKSKTTEAVKKVL<br>PKLGDMQFFMDFPT<br>SHERLLFMLSRAFH<br>GLKGGSNSRENN*	ATTAGCTCTCTACGTACAAATTCTTTCTATCTGTTCCTTTCCTT<br>TTCTGCCTCTGCGCTTTGTGGTGTAAATTTGCTTGGTGTCGTGG<br>TTAAAATTTTCTTTTTTTTCCTACCGTAATGATTATCTTCAAAG<br>ATCTTATTACAGGTGATGAGATGTTCACCGATTCGTCTAAAT<br>ATAAGTTAATTGATGGGTGTTTATATGAAGTCGAATGTCGGTT<br>TACAAGACAAGTGGAAAGAAATGGGTAAGAGTGATAATGA<br>CATTGCTGAAGCAAAGTCCAAAACTACAGAAGCTGTTAAAA<br>AGGTTCTTCCAAAGCTTGGAGATATGCAGTTTTTTATGGACTT<br>TCCCACCAGCCATGAGAGGTTGCTGTTTATGCTTTCACGTGCT<br>TTCCACGGTTTGAAGGGAGGGAGTAACTCTAGAGAAAATAA<br>TTAAAGGCACAAGAAACTGCTCTGGCACAGGCACTAGAAGA<br>AATGAGGACAACGATTTGGCACAAAAAAGGTGGATACAGA<br>GTAATGGCCATCGAATTTAAAAGCGAAGTTTTTTCGAATCTT<br>CAATAAAGGATGTCACTAGTATCTGCCAGTTGTAATACATCG<br>CAGCCATAGAAGGTTCTAGTTAGACTTGAGGAGAGCTGTAG<br>CCTGTCCAGAGATGCCAGATGAGGAGCCGGATGGGTTTCTAC<br>TTTGACGGATTTTAGTAGCTGAGAAGTCGTCCATGTTTACCGA<br>AGGATGAGTTTAGCGAGGCCTAGGGAAGTCAAGTGGATGAT<br>GTCAATTAAAGTTTCTACTGGTGTTTAAGGCAAATTGAAGTTC<br>TGACTGGATTTGGATTGACGGTATCGCTACAGAATTTGAAAT<br>CAAGTTGCCTATATTAGAAGATTTTCCGGAAAGAAAACTTTT<br>AGTCCAGTTCCCGTTTTTGATGTTTAATTTGGGCTTTCACGTTT<br>TTTAAAGTTTTGTCAAAATTTCGGTTAATAGTGGCACTTATGT<br>TACCGGTTCTGAATTCAACTATCTGGATTAATAATGTAACGG<br>GTATTTTTATATTTATTTATTACTGTTACTGAAGTCTTTACTGT<br>GTTTATAAATTGTTGCAATGTATGTTATTTAAAATGTTGAACT<br>AAAAGCGTTAAATGCTTCATGTGTGTTTCTGCTGAATTTGTAA<br>GTCATGCTAAATGTTGAAAACTCATTTTAAGAGTTTTTAAAGT

							GACTTTAAAATTTTAATCACTTTGCACCTTTGAATTTTGAAACTTAATTATTTGCATTAGTTTTATTAGATAGCAGCTCTGTTTGGTTAGCGATTATTTTACTCTATCCATTTAATAAGGTTATGGGCTTTTATTAAATGTGCATGGGTCTGTTTACCATAACAATACCAAAGACTTACTTTGATTTATTATCAGTTAAAGCTTTACTTTTACTTGCACATTCCTTTTATGTAACTATATGTATGCATCGGGATTGCGTAAAATTTTCTCTGGTCAAAAGGGGTTGCGAATGGTAAAAGTTTAACTATGTACAGCAGGGCTTCTTAAAGAGAGATTACTATGGTCGTTACCAGTCGATTTTTATCTAAAGTTTTACAGAGAATGAGAAAATTGGAAAGTGGATTTTCCACATATATGCACCTTTTGTCTGTTGGGTTATAAATTGTACAAAAATGAATATATTTTCAACTGTCTTATACATTTAAAAATTTCAATCCGGTTGATCTGAGTTTCAAAGCACGAAATGTATTATTACAAATAATATTTTGAAATGATCGGTGAAAATCCTTTTTCCGGTAAAATTTTCAAAAAAATGTTTTAAAATTAAAATCTTATTTCGCGAAAGTCACATATTAATAACATCATGGATA

PhdTCT15	M5B4R	3,83E-14	58,065	PF00838	6046,03	MGKSDNDIAEAKSK TTEAVKKVLPKLGD MQFFMVLFAAILNR REISTASSQHKY*	TTTACAAGACAAGTGGAAAGAAATGGGTAAGAGTGATAATG ACATTGCTGAAGCAAAGTCCAAAACTACAGAAGCTGTTAAA AAGGTTCTTCCAAAGCTTGGAGATATGCAGTTTTTTATGGTTT TATTTGCAGCAATTTTAAACCGGCGAGAAATATCAACTGCCT CGAGTCAACATAAATATTAGTTGGACTTGCCAGCATCAACGT GAGTTCTAGATAGTTGACGTGATCTTTCTCTTTACTTATGCAG GTGGATACAGAGTAATGGCCATCGAATTTAAAAGCGAAGTT TTTTCGAATCTTCAATAAAGGATGTCACTAGTATCTGCCAGTT GTAATACATCGCAGCCATAGAAGGTTCTAGTTAGACTTGAGG AGAGCTGTAGCCTGTCCAGAGATGCCAGATGAGGAGCCGGA TGGGTTTCTACTTTGACGGATTTTAGTAGCTGAGAAGTCGTCC ATGTTTACCGAAGGATGAGTTTAGCGAGGCCTAGGGAAGTC AAGTGGATGATGTCAATTAAAGTTTCTACTGGTGTTTAAGGC AAATTGAAGTTCTGACTGGATTTGGATTGACGGTATCGCTAC AGAATTTGAAATCAAGTTGCCTATATTAGAAGATTTTCCGGA AAGAAAACTTTTAGTCCAGTTCCCGTTTTTGATGTTTAATTTG GGCTTTCACGTTTTTTAAAGTTTTGTCAAAATTTCGGTTAATA GTGGCACTTATGTTACCGGTTCTGAATTCAACTATCTGGATTA ATAATGTAACGGGTATTTTTATATTTATTTATTACTGTTACTGA AGTCTTTACTGTGTTTATAAATTGTTGCAATGTATGTTATTTAA AATGTTGAACTAAAAGCGTTAAATGCTTCATGTGTGTTTCTGC TGAATTTGTAAGTCATGCTAAATGTTGAAAACTCATTTTAAG AGTTTTTAAAGTGACTTTAAAATTTTAATCACTTTGCACCTTT GAATTTTGAAACTTAATTATTTGCATTAGTTTTATTAGATAGC AGCTCTGTTTGGTTAGCGATTATTTTACTCTATCCATTTAATA AGGTTATGGGCTTTTATTAAATGTGCATGGGTCTGTTTACCAT AACAATACCAAAGACTTACTTTGATTTATTATCAGTTAAAGC TTTACTTTTACTTGCACATTCCTTTTATGTAACTATATGTATGC

							ATCGGGATTGCGTAAAATTTTCTCTGGTCAAAAGGGGTTGCGAATGGTAAAAGTTTAACTATGTACAGCAGGGCTTCTTAAAGAGAGATTACTATGGTCGTTACCAGTCGATTTTTATCTAAAGTTTTACAGAGAATGAGAAAATTGGAAAGTGGATTTTCCACATATATGCACCTTTTGTCTGTTGGGTTATAAATTGTACAAAAATGAATATATTTTCAACTGTCTTATACATTTAAAAATTTCAATCCGGTTGATCTGAGTTTCAAAGCACGAAATGTATTATTACAAATAATATTTTGAAATGATCGGTGAAAATCCTTTTTCCGGTAAAATTTTCAAAAAATGTTTTAAAATTAAAATCTTATTTCGCGAAAGTCACATATTAATAACATCATGGATA

PhdTCT16	T1DKS4	3,93E-115	88,953	PF00838	22935,29	PPERRSGRTALALSSGLLLSQRRCRLPPVAIMIIYRDLISHDEMFSDIYKIREVADGLCLEVEGKMVSRTEGNIDDSLIGGNASAEGPEGEGTESTVITGVDIVMNHHLQETSFTKEAYKKYIKDYMKSIKGKLEEQRPERVKPFMTGAAEQIKHILANFKNYQFFIGENMNPDGMVALLDYREDGVTPYMIFFKDGLEMEKC*	CTCCGCTCCAAATAAATCAACGTTCAAAACAAATGAAGAGCTCGAGATGACATCAGTCCCATCTGTCTAAGTCCTGGTGTTGTGTGGATGAAAAGCAGTAGCCAGCCAGTTATGACGACAGGTGATTGATCCAAAGTAACTGCCAACTTTGTTAACATTTTTCCATCTCTAAACCATCCTTAAAGAAAATCATATATGGGGTTACACCATCCTCACGGTAGTCCAGCAGAGCAACCATGCCATCTGGATTCATGTTTTCACCAATAAAGAACTGATAGTTTTTGAAATTAGCAAGGATGTGCTTGATTTGTTCTGCAGCCCCTGTCATAAAAGGTTTTACTCTTTCTGGTCTCTGTTCTTCAAGTTTCCCTTTGATTGACTTCATGTAATCTTTGATGTACTTCTTGTAGGCTTCTTTTGTGAAGCTGGTTTCCTGCAAGTGATGGTTCATGACAATATCGACACCAGTGATTACTGTGCTTTCGGTACCTTCGCCCTCGGGGCCTTCAGCGGAGGCATTTCCACCAATGAGCGAGTCATCGATGTTACCCTCTGTCCTACTGACCATCTTCCCCTCCACCTCCAGACACAGCCCGTCCGCGACCTCCCGGATCTTGTAGATGTCGGAGAACATCTCGTCATGGCTAATGAGGTCCCGGTAGATGATCATGATGGCGACTGGAGGGAGGCGACAGCGGCGCTGGCTTAGCAGGAGCCCGGAGCTCAGGGCGAGCGCAGTGCGGCCAGAGCGGCGCTCGGGGGGAG
PhdTCT17	G3LU44	4,43E-18	41,935	PF00838	14592,63	MRIYKDAFTDDELASDSFPMKVVDGIVLEFKGRRVVRKEGEIILPGANPSAEGEDEGEGSEELIERGIDFVLNHRLQEMNCYENLATFKSYCKTFVKRVVDLMQKNGKSEDEINEFKKKIQPWVLSL	CAAGTTTGAGGTAATTAATAGGCAGCTTGCCAAGCATTGCACAATGAGGATTTATAAAGACGCCTTCACAGATGATGAACTTGCTTCCGACTCATTCCCAATGAAGGTAGTGGATGGAATAGTTTTGGAATTCAAAGGACGTCGAGTTGTGAGGAAGGAAGGCGAAATTATCCTTCCTGGTGCAAATCCGTCCGCGGAGGGTGAAGATGAGGGCGAAGGCAGTGAAGAACTTATTGAACGAGGAATCGATTTTGTGCTTAATCATCGATTACAAGAGATGAATTGTTATGAAAATTTGGCAACATTTAAATCCTACTGTAAAACATTTGTGAAAAGAGTAGTCGACTTGATGCAGAAGAACGGCAAGTCGGAGGA

							TGAAATTAATGAATTCAAGAAAAAAATTCAGCCTTGGGTGTTATCACTTTG

HOST DEFENSE PEPTIDES

Defensins

Transcript ID	Uniprot ID reference protein	e-value	Identity (%)	PFAM domain	MW (Da)	Amino acid sequence	Nucleotide sequence
PhdHDPDef 01	P0CF77	4,97E-05	47,222	PF01097	6764.92 **4213.74**	MKAVYVLLVISLLVCTFAASTVRA**DRGCPDDPFKCRDYCIKNGFSKGGYCGSANYATCHCFI***	ATTTGAGATTATTATTTAAGAAACAAGCAATCAATAAACGAAGAAAAAAATGTTCGTATGATGCAGAAACTTGCATTCTATATGAAGCAATGGCATGTGGCATAATTAGCACTTCCACAGTAACCTCCTTTGCTAAATCCATTCTTTATACAATAGTCACGGCACTTAAACGGGTCATCAGGACATCCCCTATCGGCTCTTACGGTTGATGCAGCAAAAGTGCAAACAAGTAAAGAAATAACGAGCAGAACGTAAACAGCTTTCATGTTTTAGTTAATGATGTTATGCGTGTTCTTCCGAGAGAAA
PhdHDPDef 02	D9U2B2	0,0000523	34,375		6992.87 **4428.78**	MKTAYVIMFALVLISAFGAYSVEG**SHHNGCPSNTAQCVQYCKNQGYTNGACQGHKNEYCYCTNT***	CGAAAAGAAAAGACTTTCGGCAAGATGAAGACCGCATACGTGATAATGTTTGCTCTTGTGCTGATATCAGCATTTGGTGCATACAGTGTAGAGGGCAGCCATCACAACGGATGTCCTAGTAACACAGCCCAGTGTGTCCAATATTGCAAAAACCAAGGTTACACAAATGGAGCATGTCAGGGACATAAAAACGAATACTGCTACTGTACTAATACCTAACATGTATTGGAAGAAACCCATCATGTGTTTTCCGCACTTCTCGAGAATTGACGAAAATGTTTTCATTTTTCTC

							TTGTTTAAACAAGGTTATAGTAATTGTGTCATTCAATAAAATAT
Antimicrobial peptide							
**Transcript ID**	**Uniprot ID reference protein**	**e-value**	**Identity (%)**	**PFAM domain**	**MW (Da)**	**Amino acid sequence**	**Nucleotide sequence**

PhdOthAM B01	B6DD43	1,22E-05	45,455		9062.57<br><br>**6959.90**	MKRAAVFLTLLIALV<br>CVTSA**QKRGIACPD**<br>**SIYVKCAVRNNRCC**<br>**SDEDCSTGNVCCE**<br>**GISCGNKCIPGDRP**<br>**WMKYHDDCSISRP***	CGTCACTACGTAGCAACTGGAAAGGATGTGATTAAGCAGAA<br>CACTCCATTGTTCTGCCGTTGAAGGAAGATTGCGGTTATATTT<br>CAGCGAAAGCAAACATTATCGTCGACCAAGTGATCAGGAAT<br>AGTTGCTAGCAATTCCATTGTAAAATTGCTTATCTTCTAGTTT<br>TCAATTTCAATTATAATATAGCTGAATTAGACGTTATATTCGC<br>ACGCTCAGAAAAAGCAATTTCAGTGCCTCTAAACTCAGGCTA<br>AATAATTCTTAAACGATGAAGAGAGCTGCTGTTTTTCTGACC<br>CTTCTTATTGCTCTGGTATGCGTGACATCTGCCCAGAAAAGA<br>GGGATAGCCTGTCCTGATTCAATATATGTGAAATGCGCCGTG<br>AGGAACAACCGATGCTGTTCAGATGAAGACTGCTCGACCGG<br>GAATGTATGTTGTGAAGGTATATCTTGCGGTAACAAGTGCAT<br>ACCCGGCGATAGACCTTGGATGAAATATCATGATGACTGCA<br>GCATAAGCCGTCCTTAATCACGATCTGTCGATTCTTGTTAATT<br>ATAACTGATATTCTTTTAATAAAAGCTGTAGACTACTAAAAA<br>AAGGTTCTTTATCTTTATATCAAAATGTCAGTGACTTCTAAAC<br>ACATTTCCCTTAGAATAGGGGCAGAAGGAGGGGAATTATGC<br>ACATATAGTACAGTAGACAGTGGCCGATTTTGAAATTTTGTG<br>CCTTCGAGCATACCATCGGCTATGCCATGCTGATGAAACGTA<br>ATGAGGGCGTTATCATGATACATGACTATTGGGTTTGAGGTA<br>GGTGCCTCAATTTTCATTTCATTGACCTTCCTCCAACGAGGGT<br>GTATTACATGAAATTGTTGCGGCGGATCGTATTTGTTCTCATT<br>TTATTGAAATTCCTGAAAAGAAAATTATTTTTCCTGAAATATG<br>ATTATCTGTCTTAAAATTTCTAATATTTCCCATTTTCAGTGCGT<br>CTCAGTAGTGTCATGGCGTTTCATTATGCTGTTAAGATCCTTC<br>TGGATCTTTCGGGATGAGAATCAAAATTGAAGATAACATGCT<br>ACCTTTTTTTTTTTTTCGAGTTCTATAAACTGAAACCAGAATG<br>AAAATTATGGAAACACTGCAGCATCATTGATACATAGCTGTA<br>ACAGCGAAGGTACAAGGCTGAATAGCGAATAGTATAGGCTG

							AAACGAATGAGATATCGTTCAAACCAAATAATAAAACATTT TAATTAAAATCCATATTTTTTCTCGAACGTTTACTTACACGAA ATAACTGCACGAACTAATGAACTTAATGTAAATATAAACCTT TTGTCCTCGTTTTCCGATAGCGAAATATTGCCTGTTAGTCACA TTAAGCATGTCGGTACAAGGAAATTGTGCACCTGCGCGTGCG GTCACTGCATAGATGCAACGAGACTGTAGTTATCGGTCAGTG GAGAACGTAGTGGTTCGTTTTCAATAATTATGTATAATAAGA AATTGTAAAAATAAAGCTACGTTTATTATAAAAACGATTTTT ATAAAAGCTTTTTATAAGACGATTTATGTCTACAATTTTATTA AAACGCTATTTGCATGCCTTTATATTGTTTGGGCCGCAAAAAT ATGCTCTCTGGGCTGCAAGCAACCAGCAATTTTGACATGCTT GGTGTAGGCGGCTGTATTTGAACTTTGAAAACAGGCTACCTT GGGCCTTACGCGTTTCTTTTTAACGTATTAAACGCAATTCTAA ATAAATGCCAATTTTATGTTTACACGCG
<u>OTHER VENOM COMPONENTS</u>							
von Willebrand factor type C							
**Transcript ID**	**Uniprot ID**	**e-value**	**Identity (%)**	**PFAM domain**	**MW (Da)**	**Amino acid sequence**	**Nucleotide sequence**

	**reference protein**						
PhdOthvW C01	D2CFI7	5,50E-10	31,683	PF15430	11399.95<br>**8958.89**	MVYRSILPLIFAIILVGNKVQA**ATFMGRVDTSDGYCDIPGYGRIKVGEEGYDDDTCQVVYCGKDLFSGFGCGKIGYDEKHCREVPGVGHYPDCCPSVECDDIKD***	TCTACCTTTCAAAGTCTGTACGATGTTTATAAACAAAGATATTTTGAATTCTTGAAGTTCGCTTATTCTATAGCTGAGTCTGCCCGTCAAGTAAAGCAACTGGAAAACATTCATCCTTTAACTTACAAAAAATGGTTTATAGAAGTATTTTACCCCTTATTTTTGCTATTATCTTGGTCGGAAATAAAGTACAAGCTGCTACTTTTATGGGCAGAGTAGACACATCTGACGGTTACTGTGATATACCTGGCTACGGACGAATAAAAGTGGGTGAGGAAGGTTACGACGATGACACTTGCCAAGTGGTGTACTGTGGAAAAGACTTATTTTCTGGATTTGGATGCGGCAAAATCGGCTACGATGAAAAACATTGCCGAGAGGTCCCTGGTGTAGGTCATTATCCAGACTGTTGCCCTTCCGTCGAATGTGATGACATAAAAGACTGAAAACCGCATTTCAACAACGTACGGTCTAATTGATAGAAATTAATGTATTACATATAGTTCTGTATGTCAATTACTTTATGTTTCTCTATCAATAAATTCTTTGATCTCAGGATCTACTTTACATTTG
PhdOthvW C02	P0DQD0	4,90E-11	33,333	PF15430	11495.17<br>**9018.07**	MFHTLKISLLFGLTFLLICING**YTYMEDLNTESGFCERPDYGRIAVGEVGYNDESCEKILCHDGRREVMGCGKAIPPDDSQCELVREVGRYPDCCPRYRCP***	GTGACTTACAAGCAGTCGAATTTAGATAGCCGGCAGCGTATAAGAAGGACTGAAGGATGTTTCACACACTGAAGATTTCGCTGCTCTTTGGACTCACGTTCTTACTTATATGTATTAATGGATATACGTACATGGAAGATCTAAATACCGAAAGTGGTTTTTGCGAGAGGCCGGACTACGGTAGAATAGCTGTGGGCGAAGTAGGATATAACGATGAATCGTGCGAAAAAATCCTATGCCATGATGGAAGGAGAGAGGTGATGGGTTGCGGGAAAGCAATTCCACCAGATGATTCTCAGTGTGAGCTGGTACGAGAAGTAGGTCGTTATCCAGACTGTTGCCCTCGATACAGATGTCCGTGAACATAAAATCACTCACAACAAATCTGTTTGCCACCATGCGGAACTGCTAGATATGTAGCATTGTTGAAGTAGTCATATTTAACAGCTCCTGAACTGAA

							AGCTGTTATAGTGTTTCGAGATTGTTGTCTCTTCACCATATTTT<br>ATGTATTTGTAAAATCTTGAATATATGATTGATCTTAAAAATT<br>TTTAAATTTTAAATATGTTCCACCAAAATAGTTTACGTGCTTT<br>ATATATCTGTATAATTGTAGTTGATATTTTCTGCTGTATACAA<br>CTTTTAAATTAAGTCTAACACATGGAAAAATAGTTTAATTGG<br>CAGCTATTGAATATTTTTGACATTCAATTTCTACATCTTACTTA<br>ATCCGCAAACATCAGTTCCTTAATATTTCAGTCTCGAGTCTGA<br>AATATTAAGTTATTCCGTTAAAATTTCCTGCATTATATATCTA<br>TATTGATGGCGAATATATA
PhdOthvW C03	L0GCW8	3,17E-08	34,831	PF15430	12012.95<br><br>**9676.07**	MILRFFSAVAVLLSM<br>LVSRSGG**YSYLKPIM**<br>**FGEAECFDDDWISH**<br>**PIGSIWYDNNNCEK**<br>**LECVFHKDILYAKG**<br>**YGCATIGVPSGCRL**<br>**VPGKGVQYPYCCP**<br>**QVQCQDGII*****	CGGGATCAGAAGTTAGTAAAATAAACACGCAGCAAAGAAA<br>AACTAGCATTCGAACGTTATCGAGTTTCCTTGTATTCATCCAC<br>ACCTTCTAAGTGGAGGAAGCAGAAAAATCACATTTCCGCTTG<br>ACCCGGAAAAGCGTAATCTATTTTTCTCACTTTCTTGTGGTTT<br>TCAGATGAGTTTCGTGGGCAGTTAGCTTAAATCCCGGTACTTT<br>ACGTAAATGAACTTTCTATTCAGTTTTGAACTGGAAATTACCC<br>ACTATGGTTATGCTTTCGTCATAGCTGTTAACCTTCTATAATC<br>ATTGTCCATAACTTGGTATTGTGCCCTCTGAGGCGGATCAGG<br>ATGATTCTAAGGTTTTTCTCTGCAGTGGCTGTTTTGCTCTCAAT<br>GCTAGTTTCAAGAAGTGGAGGATATTCATATTTAAAACCAAT<br>AATGTTTGGAGAGGCGGAATGTTTTGATGACGACTGGATAAG<br>CCATCCCATAGGCTCCATTTGGTACGATAATAACAACTGTGA<br>GAAGCTAGAATGCGTATTTCATAAGGATATTTTGTATGCCAA<br>AGGTTATGGATGTGCTACCATTGGTGTACCATCAGGTTGCCG<br>ACTAGTTCCTGGGAAAGGAGTTCAGTATCCTTATTGTTGTCCC<br>CAAGTGCAATGTCAGGATGGAATCATTTGAAAGAAAATAAA<br>TGCTGTGGTCA

PhdOthvW<br>C04	L0GCW8	0,00E+00	36,634	PF15430	12227.22<br><br>**9890.34**	MILRFFSAVAVLLSM<br>LVSRSGG**YSYLKPIM**<br>**FGEAECFDDDWISH**<br>**PIGSIWYDNNNCEK**<br>**LECVFHKDILYAKG**<br>**YGCATIGVPSGCRL**<br>**VPGKGVQYPYCCP**<br>**QVQCRDGIIW***	CGGGATCAGAAGTTAGTAAAATAAACACGCAGCAAAGAAA<br>AACTAGCATTCGAACGTTATCGAGTTTCCTTGTATTCATCCAC<br>ACCTTCTAAGTGGAGGAAGCAGAAAAATCACATTTCCGCTTG<br>ACCCGGAAAAGCGTAATCTATTTTTCTCACTTTCTTGTGGTTT<br>TCAGATGAGTTTCGTGGGCAGTTAGCTTAAATCCCGGTACTTT<br>ACGTAAATGAACTTTCTATTCAGTTTTGAACTGGAAATTACCC<br>ACTATGGTTATGCTTTCGTCATAGCTGTTAACCTTCTATAATC<br>ATTGTCCATAACTTGGTATTGTGCCCTCTGAGGCGGATCAGG<br>ATGATTCTAAGGTTTTTCTCTGCAGTGGCTGTTTTGCTCTCAAT<br>GCTAGTTTCAAGAAGTGGAGGATATTCATATTTAAAACCAAT<br>AATGTTTGGAGAGGCGGAATGTTTTGATGACGACTGGATAAG<br>CCATCCCATAGGCTCCATTTGGTACGATAATAACAACTGTGA<br>GAAGCTAGAATGCGTATTTCATAAGGATATTTTGTATGCCAA<br>AGGTTATGGATGTGCTACCATTGGTGTACCATCAGGTTGCCG<br>ACTAGTTCCTGGGAAAGGAGTTCAGTATCCTTATTGTTGTCCC<br>CAAGTGCAATGTCGGGATGGAATCATTTGGTAAATGGTCGTC<br>CTTTTTGTTCGTTTATAGACAAAAAAAACAACGAGATTTGCA<br>TGCGATATCCCTGAAGAAAGAAGTTAATATTACCGCCTTGTA<br>TTGTGAATTAAGTATTACGGTTTTTAAAAAGTTCCAGTGAGA<br>CCAAAATGTCCAAATATTGGTTGTTTATAAACTTAATTATTTT<br>CCACAATTCATCCATGTTATAATAAAGATGGTTGCATTAGCA<br>TTCATCATGCATTGAACATTTCTGGTTCACGTATAATTTCATT<br>GAATTACATTATTAAATAAAAGTATTACATCTAA

PhdOthvW C05	L0GCW8	5,38E-12	36,634	PF15430	12227.22<br><br>**9890.34**	MILRFFSAVAVLLSM LVSRSGG**YSYLKPIM FGEAECFDDDWISH PIGSIWYDNNNCEK LECVFHKDILYAKG YGCATIGVPSGCRL VPGKGVQYPYCCP QVQCRDGIIW***	CGGGATCAGAAGTTAGTAAAATAAACACGCAGCAAAGAAA AACTAGCATTCGAACGTTATCGAGTTTCCTTGTATTCATCCAC ACCTTCTAAGTGGAGGAAGCAGAAAAATCACATTTCCGCTTG ACCCGGAAAAGCGTAATCTATTTTTCTCACTTTCTTGTGGTTT TCAGATGAGTTTCGTGGGCAGTTAGCTTAAATCCCGGTACTTT ACGTAAATGAACTTTCTATTCAGTTTTGAACTGGAAATTACCC ACTATGGTTATGCTTTCGTCATAGCTGTTAACCTTCTATAATC ATTGTCCATAACTTGGTATTGTGCCCTCTGAGGCGGATCAGG ATGATTCTAAGGTTTTTCTCTGCAGTGGCTGTTTTGCTCTCAAT GCTAGTTTCAAGAAGTGGAGGATATTCATATTTAAAACCAAT AATGTTTGGAGAGGCGGAATGTTTTGATGACGACTGGATAAG CCATCCCATAGGCTCCATTTGGTACGATAATAACAACTGTGA GAAGCTAGAATGCGTATTTCATAAGGATATTTTGTATGCCAA AGGTTATGGATGTGCTACCATTGGTGTACCATCAGGTTGCCG ACTAGTTCCTGGGAAAGGAGTTCAGTATCCTTATTGTTGTCCC CAAGTGCAATGTCGGGATGGAATCATTTGGTAAATGGTCGTC CTTTTTGTTCGTTTATAGACAAAAAAAAACAACGAGATTTGC ATGCGATATCCCTGAAGAAAGAAGTTAATATTACCGCCTTGT ATTGTGAATTAAGTATTACGGTTTTTTAAAAAGTTCCAGTGAG ACCAAAATGTCCAAATATTGGTTGTTTATAAACTTAATTATTT TCCACAATTCATCCATGTTATAATAAAGATGGTTGCATTAGC ATTCATCATGCATTGAACATTTCTGGTTCACGTATAATTTCAT TGAATTACATTATTAAATAAAAGTATTACATCTAA

PhdOthvWC06	L0GCW8	5,43E-08	34,831	PF15430	12199.17<br><br>**9862.29**	MILRFFSAVAVLLSMLVSRSGG**YSYLKPIMFGEAECFDDDWISHPIGSIWYDNNNCEKLECVFHKDILYAKGYGCATIGVPSGCRLVPGKGVQYPYCCPQVQCQDGIIW***	CGGGATCAGAAGTTAGTAAAATAAACACGCAGCAAAGAAAAACTAGCATTCGAACGTTATCGAGTTTCCTTGTATTCATCCACACCTTCTAAGTGGAGGAAGCAGAAAAATCACATTTCCGCTTGACCCGGAAAAGCGTAATCTATTTTTCTCACTTTCTTGTGGTTTTCAGATGAGTTTCGTGGGCAGTTAGCTTAAATCCCGGTACTTTACGTAAATGAACTTTCTATTCAGTTTTGAACTGGAAATTACCCACTATGGTTATGCTTTCGTCATAGCTGTTAACCTTCTATAATCATTGTCCATAACTTGGTATTGTGCCCTCTGAGGCGGATCAGGATGATTCTAAGGTTTTTCTCTGCAGTGGCTGTTTTGCTCTCAATGCTAGTTTCAAGAAGTGGAGGATATTCATATTTAAAACCAATAATGTTTGGAGAGGCGGAATGTTTTGATGACGACTGGATAAGCCATCCCATAGGCTCCATTTGGTACGATAATAACAACTGTGAGAAGCTAGAATGCGTATTTCATAAGGATATTTTGTATGCCAAAGGTTATGGATGTGCTACCATTGGTGTACCATCAGGTTGCCGACTAGTTCCTGGGAAAGGAGTTCAGTATCCTTATTGTTGTCCCCAAGTGCAATGTCAGGATGGAATCATTTGGTAAATGGTCGTCCTTTTTGTTCGTTTATAGACAAAAAAAAACAACGAGATTTGCATGCGATATCCCTGAAGAAAGAAGTTAATATTACCGCCTTGTATTGTGAATTAAGTATTACGGTTTTTTAAAAAGTTCCAGTGAGACCAAAATGTCCAAATATTGGTTGTTTATAAACTTAATTATTTTCCACAATTCATCCATGTTATAATAAAGATGGTTGCATTAGCATTCATCATGCATTGAACATTTCTGGTTCACGTATAATTTCATTGAATTACATTATTAAATAAAAGTATTACATCTAA
Diuretic hormone							
**Transcript ID**	**Uniprot ID reference protein**	**e-value**	**Identity (%)**	**PFAM domain**	**MW (Da)**	**Amino acid sequence**	**Nucleotide sequence**

PhdOthHDH01	P0DQF3	7,62E-11	71,429		13022,9	VIVESALQNLPLTMSGSFCVAALFLCVIIATFDSARTYPAANQRAKRSVDVENPEDVFRVLMKLAQDVVDSDTVIRSEKRGIDLGLSRGFSGQQAAKHLMGLAAANFANGPGRRRRDVEN*	TTTTTTTTGTGGAAGAAAAACTGCTTTAATATGCTGAGAATATACAATAATAAAGGAAAACAATATTTTGAAATATAATCAATCTTTGGAAACAAAATGATTGGAGATAAAGTCTTTCCAGCGGTACAAAAACATACGTTGTCAAATCTAGGACCTCTTTCACATCTCCGAGGTTATTTACAAGTGTTAGGAGATAGGCAGTGTCTATTTCATCCTTAATTCTCGACGTCCCTCCTCCTCCGGCCGGGTCCGTTGGCAAAATTAGCGGCAGCTAGTCCCATAAGGTGTTTGGCAGCCTGTTGACCGGAGAAACCACGAGACAAGCCGAGATCAATGCCTCGTTTTTCACTTCTGATAACTGTATCTGAGTCGACCACATCTTGGGCAAGTTTCATTAGAACTCTAAAGACATCTTCAGGGTTTTCAACATCTACGGATCTTTTAGCTCTTTGGTTAGCGGCTGGGTAAGTCCGAGCTGAATCGAAAGTTGCGATGATGACACAAAGGAAAAGTGCGGCAACACAAAAAGAACCAGACATAGTGAGAGGTAGGTTTTGTAGAGCGGATTCTACTATCACTCAAGCACACAGAGTAGTGCCTTCACCTGTTATTTCAAAAGCGCTTGTTCGGAAAACTTGCTCTCTAAGCTCCCGTCTCCGACAGG
Undefined							
**Transcript ID**	**Uniprot ID reference protein**	**e-value**	**Identity (%)**	**PFAM domain**	**MW (Da)**	**Amino acid sequence**	**Nucleotide sequence**

PhdOthUnd 01	Q66S13	1,91E-05	40,299	PF11901	16779.68	MPHAIVYEWVQAE NGEVPPNAVKIGYD AGNAVYVARAKHM GDNIPGKLLPVYGL CYVSWGGREHEYDE YEVLTDPHGSTFEW AYSNGSDIPAGTVQ GGNTANNEPLFIGR VSHNGAMCSGKVR PSDGILYVPYGGIEH QYEIFEYLVRRETL*	CGAAATGTAACAGGAAAGTGTCTTGAATTATATGAGAATTTA TTTCACAAAGTATAGTAAACATTTAAAAACAAACATTTCGAA TTCACATTTAGAAACATAGTGAGATTTTCCAGAATATATATA TGTATACATATATATACACACACACATAGCAGTATTTGATAA AGAAACGAAACAGAGTGCAACAGTATATTTTCATCAATCTAT AGGAAATCTATCTGCGAAGCTCATGTTGATAAAGAGATTTCA AGTTTTAGATTGCTATTTATAAAGTTTCTCTACGGACAAGATA TTCGAAGATTTCATACTGGTGTTCAATCCCGCCGTAGGGCAC ATAAAGAATACCATCGCTGGGACGGACTTTCCCGCTACACAT CGCTCCATTGTGTGAAACACGGCCGATAAATAGAGGTTCGTT ATTGGCAGTGTTTCCACCTTGCACTGTTCCAGCCGGAATGTCA GACCCATTGGAGTAGGCCCATTCAAAAGTGCTACCATGCGG ATCGGTTAAAACTTCGTATTCGTCATATTCATGTTCTCTTCCG CCCCATGAGACGTAGCACAAACCATAGACAGGTAATAATTT TCCGGGAATATTGTCACCCATGTGTTTAGCTCGTGCAACGTA GACGGCATTGCCTGCGTCATAACCTATTTTGACAGCGTTCGG AGGAACCTCGCCGTTCTCAGCTTGGACCCACTCGTATACAAT AGCGTGGGGCATAAGGGCTTTCACAAGAAGATTCTGGCTAG CGATTCGTCTTCCTTTCTTTTGTGCGCAGTCGCCGCGGCTTGA AACAGGAAAATTCGAAAGTGTCCTATATGGCTGCCTCCGATC GTTGACCAGTCGTAGTTATACTGTCAGCAGTAGCTACTCCCA ATCTCTGATTTGCGGATATTGCCACTGTTGTTGTTGATAATAG GAAGACCCTTACTCTTTCTCACTTTTTAGAAGCTAAAATATTA GCTTAGAATGGATCGTATTGCAGTATAAGAAGTAAATCTTGA TCTATGACAGTGCAAAACTTAATAATTATGCAAAGTTGATAC TTAACCCGCTCTTAGTTATTAACTAGGATGACAGACTATGCG CAGTAGATAAGATATCGCAATATAAGG

PhdOthUnd 02	Q66S13	6,00E-07	29,371	PF11901	17005.86	MAYCGPTHYRPVAE<br>WVPSSGGNFPPNAIE<br>VGYDGGDIIYVARA<br>HHQGDNIPGKLVPA<br>HGSCYVAWGGEEH<br>GYHQYEVLVAPYGS<br>NLEWRTCYGSDLPP<br>GAVQGGSTADGEPL<br>FIGRVSHEGAMTCG<br>KVHPSHGVLYIPYG<br>GGEHPHSSYEVLVV<br>RSVNF*	TTCCAGAAAAATCAAAAAGCTTTTAGCATATCGATCACATTT<br>TACTTTGAATTTCTTTAGGAATTTTAGAAGTTCACACTTCTAA<br>CAACCAGAACCTCGTAGCTGCTGTGTGGATGCTCGCCTCCAC<br>CATACGGAATGTAGAGAACACCGTGACTAGGATGAACTTTG<br>CCACAAGTCATGGCACCTTCGTGGGAGACACGGCCTATGAA<br>TAAAGGTTCACCATCAGCTGTGCTTCCACCCTGAACTGCGCC<br>TGGAGGGAGGTCAGATCCGTAGCATGTCCTCCACTCCAAATT<br>CGAGCCATAAGGAGCAACAAGTACTTCGTACTGGTGATATC<br>CATGTTCTTCGCCTCCCCATGCAACATAGCAAGATCCGTGAG<br>CGGGGACCAACTTACCAGGAATGTTGTCGCCCTGGTGGTGGG<br>CACGTGCCACGTAGATTATATCACCGCCATCATAACCAACTT<br>CAATAGCATTGGGCGGAAAATTGCCTCCTGAAGATGGTACCC<br>ATTCGGCAACTGGTCGGTAGTGAGTTGGTCCGCAATAGGCCA<br>TTTTGTCTAGTCAGCCACAGATGTAACCTGAGGATCTAAGGT<br>AGATAAATGCTCAATGCTTTTCGTAGCGAGGAGAGGGCCAC<br>GTAGATTATATCACCG
PhdOthUnd 03	A9XDG1	2,87E-12	48,214		10846.47<br><br>**8911.98**	MKLSIYLVLLLISLVF<br>**SEEEYLEIEDDLEERS<br>PEVKEESARKCTKW<br>RDSCMDNKGGCC<br>WPWSCDCWTQGT<br>YNRHKVRTGSVRK<br>CQCRFVLW***	GAAAAATATTTCTGGAGTGTGAATATGAAGCTTTCTATTTATC<br>TCGTCTTACTGCTGATATCATTAGTATTCTCGGAAGAAGAAT<br>ATCTGGAAATTGAAGATGATTTAGAAGAAAGATCCCCAGAA<br>GTCAAGGAGGAAAGTGCTAGAAAATGCACCAAGTGGCGTGA<br>TTCCTGTATGGACAACAAAGGTGGTTGCTGTTGGCCTTGGTC<br>ATGTGACTGTTGGACGCAAGGCACATATAATAGACATAAAG<br>TCAGGACTGGTTCTGTAAGGAAATGCCAGTGCAGGTTTGTGT<br>TGTGGTAATCAAGAAAATATGAAAATTATAAATCTATTTCTT<br>GCTCATTACTGTAAGCACATTAAATAAATATATAATGCATTT<br>CAAAAAAA

PhdOthUnd 04	B6DD14	2,37E-05	33,333		10846.47<br>**8911.98**	MKLSIYLVLLLISLVF<br>**SEEEYLEIEDDLEERS**<br>**PEVKEESARKCTKW**<br>**RDSCMDNKGGCC**<br>**WPWSCDCWTQGT**<br>**YNRHKVRTGSVRK**<br>**CQCRFVLW*****	GAAAAATATTTCTGGAGTGTGAATATGAAGCTTTCTATTTATC<br>TCGTCTTACTGCTGATATCATTAGTATTCTCGGAAGAAGAAT<br>ATCTGGAAATTGAAGATGATTTAGAAGAAAGATCCCCAGAA<br>GTCAAGGAGGAAAGTGCTAGAAAATGCACCAAGTGGCGTGA<br>TTCCTGTATGGACAACAAAGGTGGTTGCTGTTGGCCTTGGTC<br>ATGTGACTGTTGGACGCAAGGCACATATAATAGACATAAAG<br>TCAGGACTGGTTCTGTAAGGAAATGCCAGTGCAGGTTTGTGT<br>TGTGGTAATCAAGAAAATATGAAAATTATAAATCTATTTCTT<br>GCTCATTACTGTAAGCACATTAAATAAATATATAATGCATTT<br>CAAAAAAA
PhdOthUnd 05	53,9	7,14E-09	35,556		12510.35<br>**9595.70**	MVCSHGVAPCSSAL<br>LCVLLLVLIVTMTTG<br>**QINPNEQPCLAAG**<br>**GVCAHESACSEDR**<br>**RANGTRLCPTQED**<br>**QGAVCCHGVPDDE**<br>**TSCRKNGGVCWSV**<br>**ERCGNSKILGQFDC**<br>**KEGTSCCLLIY***	ATAACTTTAATATTTCATTTTTTATTTCCCTTTATACAAAATAC<br>AAGCCTGTTAATGACATATATAGAGGTTCAGTACTGAATATG<br>CTTCACTAGTAGATTAAAAGGCAACAACTGGTTCCTTCTTTG<br>CAATCGAATTGCCCTAATATCTTAGAATTGCCACATCTCTCTA<br>CTGACCAACACACTCCTCCATTTTTTCTGCAAGATGTTTCATC<br>ATCCGGAACACCATGGCAACAGACAGCTCCTTGGTCTTCTTG<br>CGTTGGACAAAGTCTGGTCCCATTCGCCCTTCTGTCTTCCGAA<br>CAAGCACTTTCGTGTGCGCACACACCGCCTGCTGCTAAACAC<br>GGCTGTTCGTTCGGATTGATTTGTCCTGTTGTCATGGTGACGA<br>TGAGGACGAGAAGCAAAACACAGAGCAACGCAGAGCTGCA<br>GGGAGCCACACCATGCGAGCACACCATAGCTGCCAGCACCA<br>AGCTTCCCGATGAGAGCGTACAAATATGTCCAGGTCAGAAC<br>GTTTCTAGCGGGCCGTCTGTTCTCTTCTGTATCAGAGAGCGAA<br>GTGCTGCTTCCACTTTTACGATGAATTCCTCAATAATGCTGTT<br>TACAAGGCCTCCAATCTCGAGGCCACTGTTTCAAAACTGCAT<br>CATTGTATTGTTAAGCTAGGCAAGGAGACCTTTATAGTCTCTT<br>GCCACGGATGAG

PhdOthUnd 06	P0DPZ6	3,89E-05	25,926		23138.42<br><br>**21461.33**	MFVYILMIFAFGATA<br>**EENTPYIVYGNGAT**<br>**SERNLPLCAGPREA**<br>**CNVVRQRFWLPQI**<br>**VYRMCKCPDRTNC**<br>**PTDFTDLSDKRTIA**<br>**FNARGQLKFCSRV**<br>**GELDYCGLNTVAA**<br>**AIGSDGTIHIHCSC**<br>**GPNHFYKRLYRNST**<br>**GQYFKCVSYGSCQ**<br>**RGDFCGHIAADSYE**<br>**TYHICTCPARHICV**<br>**LQDRNKVYADEFL**<br>**HKGPAYRGICISRN**<br>*	TGTGTTTGTGGAAAGGATTAATTACGAATTTCTTAATTATTGA<br>AAATAATTTATGTAAATTGCATACAGTATGAAAATTCACTTC<br>TTATAAATAAGTCTACTTTTTTGTGATTTTAGTTAATTTACATT<br>CTTTATTAATCAGCCATTATTTAATATTTTCTATATAAGTTCTA<br>TATTAATGAGTCTTTATGATAGTCCATTTATCTTCTGGAAAAT<br>GATATGAATTATTGGTTTTAATTTCTTGAGATGCAGATTCCTC<br>TGTAAGCAGGTCCTTTGTGCAAAAATTCGTCTGCATACACCTT<br>ATTTCTGTCTTGTAAAACGCAGATATGTCTTGCTGGACATGTG<br>CATATGTGATATGTTTCATAAGAGTCTGCTGCTATGTGACCAC<br>AAAAGTCTCCTCTTTGACATGATCCATATGACACACATTTGA<br>AATATTGGCCTGTTGAATTTCTGTAAAGTCTCTTATAAAAATG<br>ATTGGGGCCACAAGAGCAGTGTATATGAATGGTTCCATCTGA<br>TCCAATAGCAGCTGCCACTGTATTAAGACCACAATAATCAAG<br>TTCGCCTACTCGGCTGCAAAACTTTAACTGCCCTCTAGCATTG<br>AAAGCAATAGTTCTTTTGTCTGATAAGTCTGTAAAGTCAGTA<br>GGACAATTTGTTCGATCTGGACACTTGCACATTCTGTAAACA<br>ATCTGTGGCAACCAGAACCTCTGACGAACCACATTGCATGCT<br>TCTCTTGGTCCAGCACATAATGGCAGATTTCTTTCTGATGTAG<br>CCCCGTTTCCGTAAACGATGTATGGCGTATTTTCTTCTGCGGT<br>TGCTCCGAACGCAAATATCATCAGTATATAGACAAACATTGC<br>TACCCCAGTGCCCTTTCACTCATTCCACGTTGTTTTTTTCATT<br>TGCAGGTGATGGAGGCGACGAAGAGCATGGTTCGCTGTCAA<br>CTCTGGGACAAGCTGAAATTTCACATGGAACGTGAGTGGCG<br>CCTTCTGCCTTCACTCTGGCTTGATGCCCAGTGTCAAAACAAT<br>ATTAAGCGAGCCCTGCAAACTGGGTGGCTCATCGATTAATAT<br>TATCATGTAATCATTTTTGCACAA

PhdOthUnd 07	B6DCY1	0	38,667		9726.15 **7589.41**	MNTKLCVLLVVAICLTLVHA**GQHCPEPKRKGTCHMRFRYNDCCKQSDCPDGATCCWLPCGNACQPDPGQASENGKPVDDNAPCEEMEEV***	GCAACCCTTTTACGTGCAGCCACTTTTTCAAAAACAAAACAGGAGATCGTATAGCATAAATTCTGGTACCTGCAGTAACCGCTACACACATTTTCGTAGAACATCTTGTTCCGTGTAGACATTTCAGACCTTAGTTTCAGGCATCAAAATGAATACCAAACTTTGTGTTTTACTCGTAGTTGCCATATGCTTGACTCTGGTGCACGCTGGACAACACTGCCCTGAGCCCAAACGAAAAGGCACATGCCATATGAGATTCCGCTACAACGACTGCTGTAAACAGTCAGATTGTCCAGACGGTGCCACTTGCTGCTGGCTGCCTTGCGGAAACGCTTGTCAACCCGATCCTGGACAGGCCAGCGAGAATGGAAAGCCAGTGGACGATAATGCACCTTGCGAGGAAATGGAAGAGGTGTGAAAGTAAATAGCGATCACTCTGACATATAAGCTCTGACAAAATTGAAGCAAATAATTTCCTTGAGAGGAAATTAATATTGTATGTTAAAGAAACACGAAATTAATAAAATTTAACTCCTGTGAAAAAAAA
PhdOthUnd 08	B6DCY1	0,00000000011	39,706		9726.15 **7589.41**	MNTKLCVLLVVAICLTLVHA**GQHCPEPKRKGTCHMRFRYNDCCKQSDCPDGATCCWLPCGNACQPDPGQASENGKPVDDNAPCEEMEEV***	GCAACCCTTTTACGTGCAGCCACTTTTTCAAAAACAAAACAGGAGATCGTATAGCATAAATTCTGGTACCTGCAGTAACCGCTACACACATTTTCGTAGAACATCTTGTTCCGTGTAGACATTTCAGACCTTAGATAGAGATTATGTTTACATTCCTGCTATGTCAATACGAATACCCAAGACTGAGTCACTGTCTCCCATTTAAATTTCAGGCATCAAAATGAATACCAAACTTTGTGTTTTACTCGTAGTTGCCATATGCTTGACTCTGGTGCACGCTGGACAACACTGCCCTGAGCCCAAACGAAAAGGCACATGCCATATGAGATTCCGCTACAACGACTGCTGTAAACAGTCAGATTGTCCAGACGGTGCCACTTGCTGCTGGCTGCCTTGCGGAAACGCTTGTCAACCCGATCCTGGACAGGCCAGCGAGAATGGAAAGCCAGTGGACGATAATGCACCTTGCGAGGAAATGGAAGAGGTGTGAAAGTAAATAGCGATCACTCTGACATATAAGCTCTGACAAAATTGAAGCAAATAAT

							TTCCTTGAGAGGAAATTAATATTGTATGTTAAAGAAACACGA AATTAATAAAATTTAACTCCTGTGAAAAAAAA
PhdOthUnd 09	B6DCY1	0,000000 000265	37,662		9726.15 **7589.41**	MNTKLCVLLVVAIC LTLVHA**GQHCPEPK RKGTCHMRFRYND CCKQSDCPDGATC CWLPCGNACQPDP GQASENGKPVDDN APCEEMEEV***	TGCACATTCATCCATAGAAATGCATATAATTATGGAGCATTT CTTAAAAGCTATCTGCAAATTCCCTGCACAAATCGCTTTCGTC ACTTATGGCTTGATTCATAGTAGCTTCTTCCGAAATTAATCGC CATATCAGGTTTTTCATAGATTCATCAAACAAACCTTAGAGT TTATATTAGTAAATTCTGATCTAGAAAATAGTCAGTTATAAA TTTAGAACGAAATTCAGACATGGAAAGACATTTAAAATGAT CGCTGATTTATATATAACTTACAATTCTTGTAGCCCCACCCCT CCCTAGAAAAAAAGTAAATAGCTAAAGACTATATACAAATT TTCCACATCTAAAATAAAATATGGTTAAATTTAGCATGAACT GGCAACATTAATTCAGGTTGTAAACTTATCTTTAGAAGGAAA ACTTTTCCAAGGTAAAATATATTTTTAGAAAGATCCAGTATA TGCACTTAGTTGTATCAGCGAGAAATTAACCTCTCAACTTCA GCAACCAAATTCGAGTTGCAAATGAATGCTCATTAATCGATT TCATATTGGATTTTACAAGTGTGTAATAAATAAAACGTATGT GCCATTTACAAAAATACTTTATAGTAACTGAATCTTTAAATA TCAACGTGAATCAACCATGTTCGAAACAATGTTACCCTTTAC TGGATTATCTCGAGAGAAAAATATTTTTATTTATTGTGATTTG AGAATTTCACTGTTTGTATACCATTGTAGATCTAAACGGAGA AATATGAGTTAAAATATTTCTAAAATTTGTATAATATGTAAT

							ACCTCTTTCCAACAGTTTCAGGCATCAAAATGAATACCAAACTTTGTGTTTTACTCGTAGTTGCCATATGCTTGACTCTGGTGCACGCTGGACAACACTGCCCTGAGCCCAAACGAAAAGGCACATGCCATATGAGATTCCGCTACAACGACTGCTGTAAACAGTCAGATTGTCCAGACGGTGCCACTTGCTGCTGGCTGCCTTGCGGAAACGCTTGTCAACCCGATCCTGGACAGGCCAGCGAGAATGGAAAGCCAGTGGACGATAATGCACCTTGCGAGGAAATGGAAGAGGTGTGAAAGTAAATAGCGATCACTCTGACATATAAGCTCTGACAAAATTGAAGCAAATAATTTCCTTGAGAGGAAATTAATATTGTATGTTAAAGAAACACGAAATTAATAAAATTTAACTCCTGTGAAAAAAAA
PhdOthUnd10	B6DD43	0,000000000291	42,188		7204.45<br>**5114.80**	MNTKHCVLLVVAICLTLVHS**GQHCPEPRRRGTCHMRIRYNDCCKPSDCPDGATCCWLSCGNACQP**	CGGGTTGACAAGCGTTTCCGCAAGACAGCCAGCAGCATGTGGCACCGTCTGGACAATCTGACGGTTTACAGCAGTCGTTGTAGCGGATTCTCATATGGCATGTGCCTCTTCGTCTGGGCTCAGGGCAGTGTTGTCCAGAGTGCACCAGAGTCAAGCAGATGGCAACTACGAGTAAAACACAATGTTTGGTATTCATTTTGATGCCTG
PhdOthUnd11	P0C2S9	0,0000143	39,344		8468.04<br>**6496.48**	MKVSIFFVLLLITLVRP**EEANMNFLVEAEDIFPEINEERMPCLEIRKRCENDKDCCKGTTCRPAKFRRLCLS***	TTTCATGTTCGTAATGCATATTTTCAGCCGTTAATAACTGAGAATAAGAAATAGTTTTGTAACATGCAATCGAATTTTTTTATTGTGCTGATGACAATCAGCGGAAAATACAATTATTTAGTAGGGTAGTCACGAGCAGAAAAAAATAATTTCAAAATTTACGTGTCATGAGTATTTCTTTACGACAGGCATAGCCTTCTGAATTTCGCAGGCCTGCACGTCGTACCTTTGCAGCAATCCTTGTCGTTTTCACACCGCTTACGGATTTCAAGGCAAGGCATCCTTTCTTCATTGATT

							TCTGGAAATATGTCTTCTGCTTCAACTAGGAAATTCATATTAG CTTCTTCTGGGCGCACGAGCGTTATCAAAAGTAGAACGAAA AAAATTGAAACCTTCATTGTTCAACTTTTGGAATTAGCTTCAC AAAAAGTCTTAAGAGATTATCAGAA

PhdOthUnd 12	B3EWT5	0	73,494		11409.42<br>**9450.89**	MKVALVYLILCGIIGVSLA**VTSCTQQTDCAEDECCLNNLFAKTPYCEKRYGAGKKCTPSVYKRETDLFYVTCPCVQMYECLGRGSLDENGNTVMKNPKCIMPL***	GTATTAATTCTCATAGTAAGCTTTGAGAAAGTTTCTTATTGAC<br>AGATTAGTTAATGCATTTTTTTGAATCCTGTCTTTTTTGCATCG<br>GTTGGTTGGTTGGTTGGTTAGTTGGTTGATTAGGGTTTATTGG<br>CGCAGAGTTATCTGAGAACATGCTGCTCTAAACACATGGTAC<br>ATGGAAACTGCAATGAATACTACATTAAAAGAGAGCATTGT<br>AGACTTCACAAAATCAGCTGAATTTTGCGAGAAAAAACCAG<br>ATAAAGGAATAACACCGTGCGGTAAAAGATGGATTCCGAAA<br>GGCTACAGAAAAAAATAAAAACTTCAGCCAAATAAAATATG<br>TTGCGTTAGAAGATAAATCATCATGACAAGATTTAAAATAAG<br>AAATAAAAACACGGTGTAGAGAAGCAAGAAAAGCAACTCA<br>AAACATGCGAACATGCAAAATGTTAAAGTATGCTAGAAAAG<br>TTAAAACATGCATGATAAAAGAATACTTTAAAAAATTCGTAT<br>GTAAAACAATGGCAATAAATAGATAAAACTCAAGCGCCAAA<br>TGGTACGCATGAAACAAAAGGAAAAAAAGAAAACCCACGA<br>AAAAAAACATTAAGCAGGCCCTAAGGCGTTACCACACCCAG<br>GATCGCCTGCAGAACAAGAAGGCGACGACAACGTCGCCTCC<br>ATTTCATGTCCATCACGTCTCATCATCAGAAACATACAATTC<br>GAGAGGTGGAGCAGTTTCATAATCTTTTCTATAAAGGTCATA<br>ATATGCTTCTTCTCGCGTTTAGTCAAATTTCGGTAGTAGTACC<br>GAATTAGTAGTAGTACTAATTCGGTACCGAATTAGTAGTAGT<br>ACCGAATATTCGGTAGTAGTACCAGATTCCGGTGTGTCTACA<br>TACGTTTGACTTTTCTTTTTTGCGTTCTGATGTTTGGTAGGTTTC<br>GGGGATGAATCTTTAAGTGGGGAATTAAGCGGTTTTTGTTGG<br>AACACACTTTAGGATGAGGGCAAGGATTAGGTAGTGAGGGT<br>TTTTCAGCGGCAGGAGATTGTGGAGATTTGGTAGGAGAAGG<br>ACTGACAGTTTAATTTGAGGTGACTATTTTTTCGCATTTTGAA<br>TTGTGGGTTTGGACTGCTGGATAATTGACTTTTATGAACTTTT<br>GGGAATATACGGGACACTCAAGTCGACCTTAGGTACAATATT

							AAGATTCTTTTGTATGCTGGAAAGATCTGTGTGAGTGCTGATAGCGCTCTAACGTTTGAGTGTTACAGCATACAACGCCCAGACCTTGGAGTTCGGGTTTCAAAACATTTTCTGGTTTCTGGATAGAAAATACCCTCTATAACTTCAATAACTTGTACTTCTGTCTCAAGAAGCCAGGTGGGACATGTTCTGGAAAACGCTGTATGCTCACCTTTCTAATTAGTGCACTTCACGTTAGCCTGACAACCTTTACCGTCGTGACCCACTTCTACGCACCGAAGACAAATAAAGGTTCCACGAAAAGATGTTTTTGATTGTCCAAACCGTTGGAACTTAATATAGCGTAAAGGATTGGAGAAGAAAGGTCGCACAGGATGCGCAAATAACCAGCTTAAGTATATAACGGCAAACATAGCGAATCAAGTGTCAGGATGACGTTGCTTCGTTGGAAGTATCTTCACATCCCGACGAATTGTAATTCGTCATAACCACAAACATGATGCTCCTTGCTTCCCGATCAAATTACTTCCTCAGAGAGATGGTAAAGCTAGCACTCCGATATGACTATTCGGATGGAGATACCCCTACAACTCAGCAATGCATTATTATATTGGCTTGTACAGTATTTGCAACTTCTCCAAGTAGGTAGCCGGCCAGACTTGGTTGGTTGACTCATAATAGTTTTCATCTTTCAAGTAGGTCAGCGGATCTTTATTTTTTTCACGGTTTTAACCTCACCTATAGTTTTCGTTAGCCATTTGTCAATAAGGAAGGGAGAGACCGTTAACAAGGTCAAGTCCTTCCTTTTGACACGAAAGAAAACCATACCTGTTCGATATCAACACTTGTTACGGTTTGTCGCCTCTTTTGGCCCAAGGGGAGGTGAGGTTGTTGTTTCTAGAAGAAACCATATTTCATTTGTGGGTGTTCAGACCCCAGCCGCATCACCCAACAAGGAGTCTAACGAGGGGATGCTAGATCGCTCCCAATTAAGAGGCATTAAAAGGTGAACCAGTGCTAACCGGGGTCTATACGCGCACAGTGACCTCTTAAGCAGCCTCGACCCAATAAGGTGCCCAGTGGGTTACCGATTGCTTGGCCCAAGCCAGTGACATGTATCGTCCGACCATACGATCGATGCACATGGAACCAGTGTACACCGCCCATTTACCTGAAGCA

							CAGGCCAAAGTAATGTGCTAGGGGTTTCCCTGAATTAACAGGATCCCCTATCCAGGAACGCATCTCCACTCAATGCAAACACGTGGTATCTTACACATCGCGAAAAGCTATGTGGTGAATTATACATAACTTCTCGATAGGTGCCCTCACCTCCGAAAAGGATACCTGAAGGAGGGAGAACCAAAGCAAAAAAGTAGAAGAAAAGAGAAGGGCGAAAGTAAAGTAGGAAGAGATTGCTGGGGTACATCGGGATTACGACACCCGTTATCCACCTGAAGAAGGTGAACCCCGGAGGGGCTTTTTTGCATCGAAAAGTTGACATTTAAATTTAAAACGTGAAGTTTTTCAAGTTCTGAGATTTTTGGAAGTTAATTTCTTATAGAATCTTATGCACATGATGACTCACGCAATAGAATTAAAATAATTTTTAAAAAATTGTAATACTTTTCAAGTTTGCTTAAAAAACTCGAGCATTATTTAATGATCCATTTTAAGGTGAACAAAAAAAACACCAGAACGAATAAATAAGCATTTGTTTTTGTGCAATTAGAAATTTAAATATGAATACATGGATTATTATTGGGAATTTGTCTACAAATTTCCAGTGGAAATCATCCGTAATTTACGGGGTATAATTAATTATTTTAAGCTATCAGTTATTGTTTAATTAAATTGTGAGTATTTTGAAATCATTATTTTCTAAGATTTAAAAGTGGCTTTGCGATTTGGAACCGTTTTCGACTAAAGCATCCAGCTTCCGTTGAGCAGTACATGCCTCTGACACAAAATCGCTGTTTACGTTCAAATACAGAATTTACTTCACCATTAATTCTCATAATGGAGAGAGAAATTCTGGCCTAACGGTCACTGTGCTGGCGCTCTGCTGCGGAGAGGGATGATAATGGAGAGTTTAAAAAACATGAAGAAAAGAGATGTAGGAATAATAACGAAAATAAAACGAGAGAAATGGGACAATAATTAACCTTGGAAAAGGACGGTGAATTCTGTGAAGAAGGCTGTCAAAAATAAATGGGGACGATAGCCACTGGGGAAATAAATACGAGATTGCACATTTGCATTTAAAAATTTTTACAAACTTCTTTTTGCATAACATACGTAATGAGATTTGAAGCTTTAAATTTAAAAAAAGATCACCATTTCGAATTTCAAGAT

							AATTACAATTTTAAGAAAAAACTAAAAACTACAAAAGGGCC<br>TATACTGTTAGATTGGCACGTATCTTATCCAAATAGAAGCTT<br>GTAAAATATTTAAAAGCAAATGTACAATCTCGAATTTACTCT<br>CCGATTTGACTTAACATTGGTATTGAAACCTCAGAGTCTGAG<br>TGAAGTCAGTACGGCGATGATCTCGAGATTCGTACAGTCCTG<br>GTTCGATCCGTGTCATTTTCGAGCTTTGGTCTCGACATTATTA<br>AATCCCATTCTTTTCACTTATTACGGTTATTATGTTCATGAAA<br>AATGTTTAAAACAATTAACTTTCATTAAACATAAAATTTAGG<br>CTATATCGCTATAACACGATATGGTAATAAAGCATTTTTCCC<br>ATGCAAATTGATGACTATATACATTGAATTTGATACGAAATG<br>ATAAATATCGTATCCAGACACAGATCTGTGATCTTAAGGACA<br>GCTGTTTATTCTCCAGTGAAGAGCGGGAATGTATTTCAAGAG<br>GGTGAAAAATAGAGAAAAATTTTATTTAAGAGAGCTGAAAA<br>CTTCTCTTTTCGTCTGGTGCTGAAGTCTTCAGTAGATTCATCAT<br>GAAGGTCGCGCTCGTCTATTTGATTCTCTGTGGAATTATAGGG<br>GTTTCTTTGGCCGTAACCAGTTGCACCCAGCAGACTGACTGT<br>GCAGAGGACGAATGCTGTTTAAATAATTTGTTCGCCAAAACG<br>CCTTACTGCGAGAAGAGGTACGGAGCAGGAAAGAAATGTAC<br>ACCTTCTGTATACAAGCGTGAGACGGACCTCTTCTACGTGAC<br>TTGCCCTTGCGTTCAGATGTATGAATGTCTCGGACGGGGATCT<br>CTCGATGAAAACGGAAATACCGTCATGAAAAACCCCAAATG<br>CATCATGCCATTGTAAATTAGATAATAAAATATTTTGCGTGA

PhdOthUnd 13	P0DQE7	0,0000000907	31,325		13803.03<br>**11241.89**	MNSFKTTIGILSWFIAMVIVVHA**LDKDYKSKGLLSDLVQKPIFYAGEMRNTDRPIGDPCQFSSDCSSGCCLKAKSGKRKCSRKMKKDERCSVSQVKLDLYMEHCPCEKGNKYCSDDPESRCAA***	AAAAAAAATACAGATAAAAATATCTGAAAAATTCGTACATAAAAGTGTACAAATGAATATTAAAATGAAGGTGAAACGACGCAATCTAAGTCGAAAATATATTCATAAATTAAGTTTGGAGGCAGAATGAATGGGAAAGATATCACACACACAAAATTCAGCTTCAAGCTGCGCATCTCGATTCCGGATCATCCGAACAATATTTATTTCCTTTTTCGCAAGGGCAGTGTTCCATGTACAGATCCAGTTTTACTTGGCTGACTGAACACCTTTCATCCTTCTTCATTTTCCTTGAGCATTTTCTCTTGCCACTCTTCGCTTTTAAGCAGCAACCACTGCTGCAGTCAGAGCTGAATTGGCAAGGATCACCAATAGGTCGATCAGTATTTCTCATTTCACCGGCGTAGAAAATGGGCTTCTGGACGAGATCACTCAGGAGGCCTTTGGATTTGTAATCTTTATCCAAGGCGTGAACAACGATCACCATCGCAATGAACCAGCTGAGAATACCGATTGTTGTCTTGAAGGAATTCATCTTTAGCGCTACTGGATAGTGGAGAGCGTGAGATAGATATGACTGTCTCGTGCG
PhdOthUnd 14	B5AJT3	0,000000000329	46,154		7007,94	GGRCSRGTAGFAYVGGACVVNKRLEKVNSVAIIEDSGGFSGIIVAAHEVGHLLGCVHDGSPPPTYLGGPGA	GCGGAGGAAGGTGTAGTAGAGGAACAGCTGGTTTTGCCTACGTGGGCGGAGCGTGTGTGGTGAACAAGAGGTTGGAGAAGGTAAACAGTGTGGCTATTATTGAAGACAGCGGCGGATTCAGTGGAATTATAGTCGCAGCACATGAAGTGGGTCATCTGCTGGGCTGTGTGCACGATGGATCTCCTCCACCAACCTACTTGGGTGGACCTGGTGCTAG
PhdOthUnd 15	B3EWT5	0	87,654		11208<br>**9263.49**	MKITLAFLVLCGILGVSLA**WTSCTKQSDCEEDECCLDNLFFKRPYCEKRYGAGQRCSAAAIYKEDKDLYYFTCPCVKLHECLGPG**	ATACATTTCGGGTTTTTCATGATAGTGTTTCCGTTATCATCGAGCGATCCCGGTCCGAGACATTCATGGAGTTTAACGCAAGGACAGGTGAAATAGTAAAGGTCCTTATCTTCTTTATATATAGCAGCGGCAGAGCATCTTTGTCCTGCTCCGTATCTCTTTTCGCAGTAAGGTCTCTTGAAGAACAGATTATCCAGGCAGCACTCGTCCTCTTCACAGTCGGACTGTTTGGTGCAACTGGTCCAAGCTAGGG

						**SLDDNGNTIMKNPKC**	AGACCCCTAGAATTCCACAAAGAACCAGAAATGCGAGGGTGATTTTCATGGTGAAAAGGTTGTTA
